# Combining membrane proteomics and computational three-way pathway analysis revealed signalling pathways preferentially regulated in human iPSCs and human ESCs

**DOI:** 10.1038/s41598-017-15347-z

**Published:** 2017-11-08

**Authors:** Wei-Sheng Tien, Pei-Mien Chen, Ching-Yu Chuang, Shook-Mun Lui, Hung-Chih Kuo, Yu-Ju Chen, Kun-Pin Wu

**Affiliations:** 1 Institute of Biomedical Informatics, National Yang Ming University, Taipei, 112 Taiwan; 2 Bioinformatics Program, Taiwan International Graduate Program, Academia Sinica, Taipei, 115 Taiwan; 3 Institute of Cellular and Organismic Biology, Academia Sinica, Taipei, 115 Taiwan; 4 Institute of Chemistry, Academia Sinica, Taipei, 115 Taiwan; 5 Genomics Research Center, Academia Sinica, Taipei, 115 Taiwan

## Abstract

Owing to the clinical potential of human induced pluripotent stem cells (hiPSCs) in regenerative medicine, a thorough examination of the similarities and differences between hiPSCs and human embryonic stem cells (hESCs) has become indispensable. Moreover, as the important roles of membrane proteins in biological signalling, functional analyses of membrane proteome are therefore promising. In this study, a pathway analysis by the bioinformatics tool GSEA was first performed to identify significant pathways associated with the three comparative membrane proteomics experiments: hiPSCs versus precursor human foreskin fibroblasts (HFF), hESCs versus precursor HFF, and hiPSCs versus hESCs. A following three-way pathway comparison was conducted to identify the differentially regulated pathways that may contribute to the differences between hiPSCs and hESCs. Our results revealed that pathways related to oxidative phosphorylation and focal adhesion may undergo incomplete regulations during the reprogramming process. This hypothesis was supported by another public proteomics dataset to a certain degree. The identified pathways and their core enriched proteins could serve as the starting point to explore the possible ways to make hiPSCs closer to hESCs.

## Introduction

The breakthroughs in induced pluripotent stem cell (iPSC) technology shed light on the regenerative medicine; by introducing a panel of transcription factors, somatic cells can be converted into iPSCs that exhibit pluripotency and self-renewal capacity similar to embryonic stem cells (ESCs)^1^. The use of human embryo in academic research or clinical practice is quite limited due to the ethical controversies, iPSCs obtained by reprogramming from somatic cells therefore display superior potential for therapeutic applications^2^. How close the iPSCs to their target ESCs, however, has not been thoroughly elucidated. Although many iPSCs were reported remarkably similar to ESCs, iPSCs and ESCs with different gene expression profiles were constantly found in different studies^3,4^. Thus, prior to the clinical application of iPSCs, identifying the similarities and differences between the two types of pluripotent cells and the biological events related to these subtle differences will be valuable.

Comparative proteomics analyses based on mass spectrometry (MS) have been applied to gain insights into the similarities and differences between iPSCs and ESCs^5,6^. Meanwhile, the behaviour of pluripotent cells has been shown to be tightly controlled by extrinsic and intrinsic factors^7^, the plasma membrane-associated proteins that serve as interfaces between the cell and the surrounding environment are of importance in cellular signalling process and therefore are regarded as biomarker candidates^8,9^. Thus, the comparison of membrane proteome may help us identify pathways that translate external factors into internal signals for self-renewal and differentiation induction^10^. Although membrane proteins are known to serve as surface specific markers and of clinical potential^11^, the analyses of membrane proteins from whole cell lysates are technically difficult due to the relatively low abundance and hydrophobic properties of membrane proteins^12^. The studies of iPSCs and ESCs exclusively on membrane proteome therefore became promising but challenging. Nevertheless, currently, no pairwise comparisons were specifically performed on human membrane proteome of ESCs, iPSCs, and their precursor somatic cells to the best of our knowledge. In this context, we aimed at conducting a comparative proteomics analysis of the membrane protein profiles of human ESCs (hESCs), human iPSCs (hiPSCs), and human precursor foreskin fibroblast cell lines (HFF).

There are usually functional analyses following comparative proteomics to gain insights into the underlying biological mechanism behind the proteomic data. Over the past decade, several pathway analysis methods have been developed and applied to numerous comparative omics studies to perform functional analyses^13^. The biomolecules involved in the underlying mechanism behind the long lists of omics profiles are expected to be identified by these pathway analysis tools or databases such as KEGG^14,15^. Pathway analysis approaches have also been used to identify pathways significantly regulated between hiPSCs and their precursor somatic cells, or to assess the differences between hiPSCs and hESCs at the pathway level^5,16,17^. Each of the above two applications of pathway analysis, however, can only identify the differences between hiPSCs and precursor somatic cells, or the differences between hiPSCs and hESCs; we did not know the relations between the two sorts of differences. In other words, we did not know how the reprogramming of hiPSCs affects the differences between hiPSCs and hESCs. Given that hiPSCs are reprogrammed from somatic cells and designated to function as hESCs in an undifferentiated state, a three-way pathway comparison of pathways regulated between hiPSCs and hESCs, hiPSCs and precursor somatic cells, and hESCs and precursor somatic cells might identify pathways that contribute to the subtle differences between hiPSCs and hESCs. Nevertheless, none of the current research performed such a three-way comparison of the pathways regulated among hiPSCs, hESCs, and somatic precursor cells.

In this study, a computational three-way pathway analysis was conducted using the bioinformatics tool Gene Set Enrichment Analysis (GSEA)^18,19^. Three membrane protein profiles of expression ratio of hiPSCs to HFF, hESCs to HFF, and hiPSCs to hESCs were subjected to GSEA to find significantly regulated pathways. On the basis of these identified pathways, our hiPSCs and hESCs samples were found very similar at protein and pathway levels. Moreover, by matching the significant pathways of hiPSCs to hESCs against those of hiPSCs to HFF and hESCs to HFF, certain pathways were hypothesized to undergo an incomplete regulation during the reprogramming process of hiPSCs, which may contribute to the subtle differences between hiPSCs and hESCs. Our results showed that during the reprogramming process of hiPSCs, pathway *focal adhesion* may undergo an incomplete repression and *oxidative phosphorylation* and *TCA cycle and respiratory electron transport* may undergo an incomplete activation.

## Results and Discussion

**Figure 1:**
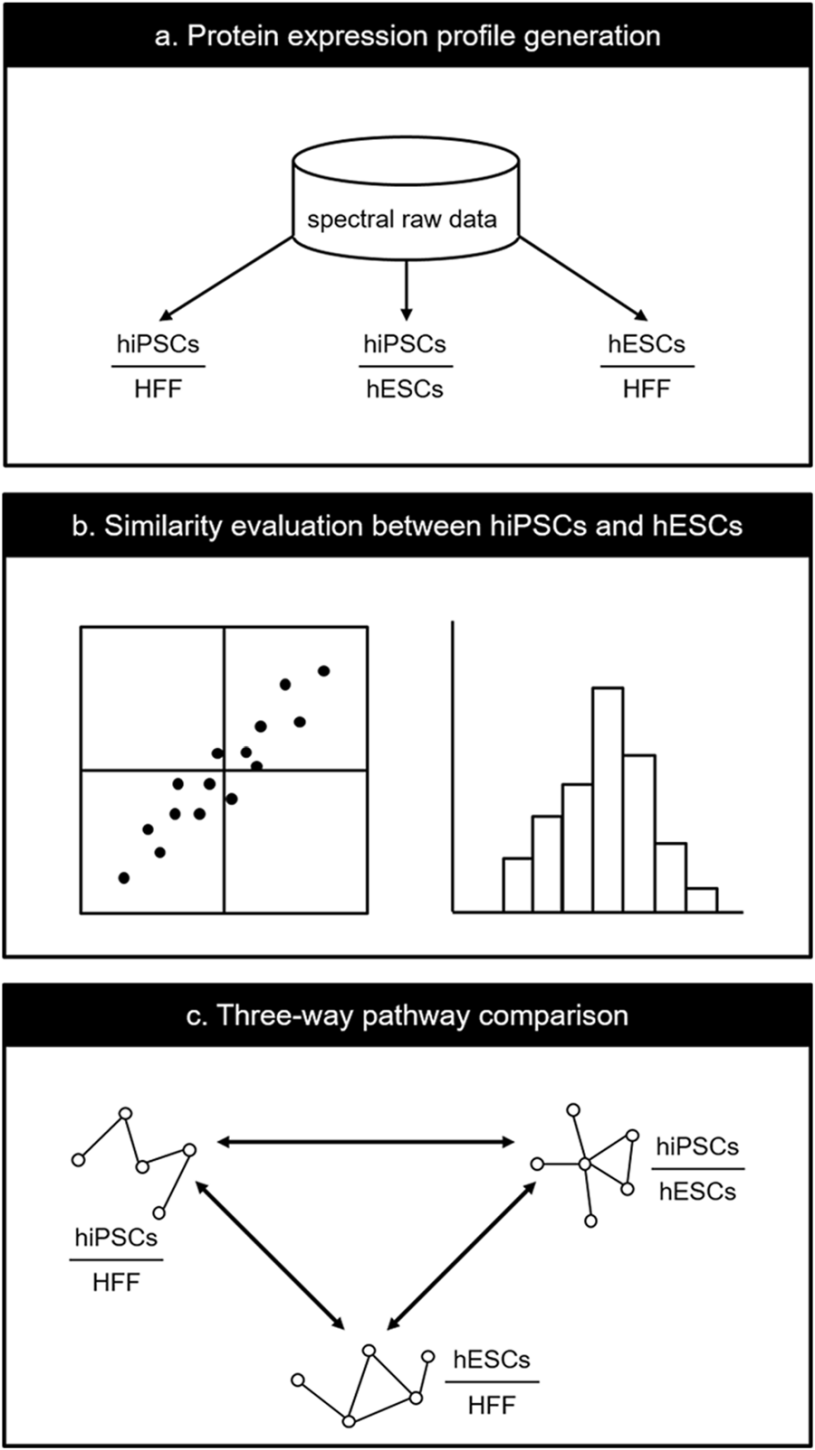
The workflow of our three-way pathway comparison on the comparative membrane proteomics of hiPSCs, hESCs, and HFF. (**a**) On the basis of spectral raw data, three profiles of protein expression log_2_ ratio, hiPSCs/HFF, hESCs/HFF, and hiPSCs/hESCs, were generated for hiPSCs versus HFF, hESCs versus HFF, and hiPSCs versus hESCs. (**b**) The similarity between hiPSCs and hESCs at protein expression level were evaluated by the Spearman correlation coefficient between hiPSCs/HFF and hESCs/HFF and a histogram describing the distribution of protein log_2_ ratios of hiPSCs/hESCs. (**c**) A three-way pathway comparison was performed on the significant pathways (obtained from GSEA) of hiPSCs/HFF, hESCs/HFF, and hiPSCs/hESCs to identify important pathways that may contribute to the differences between hiPSCs and hESCs during the reprogramming process of hiPSCs.

The proteomics analysis in this study was composed of three steps as depicted in Fig. 1. Since our goal aimed at capturing differences that were consistently revealed between hiPSCs and hESCs, the first step of the analysis was to compile our mass spectrometry-based membrane proteomic data into three comparative protein expression profiles: one for hiPSCs versus somatic precursor HFF (hiPSCs/HFF), one for hESCs versus somatic precursor HFF (hESCs/HFF), and one for hiPSCs versus hESCs (hiPSCs/hESCs) (Fig. 1a).

The second step of our analysis was to estimate how close our hiPSC cell lines to hESC cell lines at protein expression level (Fig. 1b); the hiPSC cell lines were expected to be very similar to the hESC cell lines. Since the output of IDEAL-Q, the software we used to quantify proteins, is not absolute expression level but relative expression ratio, we can only compare protein profiles using ratios with respect to the same base (i.e., same denominator). So the similarity between the hiPSC and hESC cell lines was assessed by the following two measures: 1) the Spearman correlation coefficient between the hESCs/HFF and hiPSCs/HFF profiles, and 2) a histogram to show the distribution of the protein expression log_2_ ratios of the hiPSCs/hESCs profile. If most proteins in hiPSCs and hESCs had similar expression levels, there would be a high accumulation of proteins with zero log_2_ ratio in the histogram.

The final step of our analysis was to estimate how close our hiPSC cell lines approach to hESC cell lines at functional level by identifying significant pathways related to the differences among hiPSCs, hESCs, and somatic HFF (Fig. 1c). On the basis of pathways reported by the GSEA, a three-way pathway comparison was performed to answer the following three questions:

1. The similarity between the hiPSCs and hESCs.
2. The pathways related to the differences between the hiPSCs and hESCs.
3. The biological processes during the reprogramming of hiPSCs which may contribute to the differences between hiPSCs and hESCs.

### Expression profiles of hiPSCs/HFF, hESCs/HFF, and hiPSCs/hESCs

The protein identification was achieved by shotgun-based mass spectrometry analysis. To ensure statistical confidence of protein identification, we performed a decoy database search, obtained by searching against a Mascot-created randomized protein sequence database with identical validation criteria and search parameters, to evaluate the false discovery rate (FDR) of protein identification. It is noted that all the protein identification results were obtained at false discovery rate <1% at protein level with at least one unique peptide. An hiPSCs/HFF profile of 792 proteins was obtained by taking the intersection of the two protein identification results for the hiPSC CFB46 cell line versus somatic precursor HFF (1100 proteins) and the hiPSC CFB50 cell line versus somatic precursor HFF (1111 proteins). Similarly, an hESCs/HFF profile of 752 proteins was obtained by taking the intersection of the two profiles for the hESC NTU1 cell line versus somatic precursor HFF (1110 proteins) and the hESC H9 cell line versus somatic precursor HFF (1105 proteins). To generate the hiPSCs/hESCs profile, we first generated two profiles: one of 864 proteins for hiPSCs versus hESC NTU1 by taking the intersection of the two profiles for hiPSC CFB46 versus hESC NTU1 (1125 proteins) and hiPSC CFB50 versus hESC NTU1 (1128 proteins), and the other of 870 proteins for hiPSCs versus hESC H9 by taking the intersection of the two profiles for hiPSC CFB46 versus hESC H9 (1115 proteins) and hiPSC CFB50 versus hESC H9 (1122 proteins). The hiPSCs/hESCs profile of 810 proteins was thereafter obtained by taking the intersection of the two generated profiles. The raw protein profiles from mass spectrometry and the above three integrated protein profiles were listed in Supplementary Tables S1 and S2, respectively.

### Similarity measure of hiPSCs and hESCs at protein expression level

**Figure 2:**
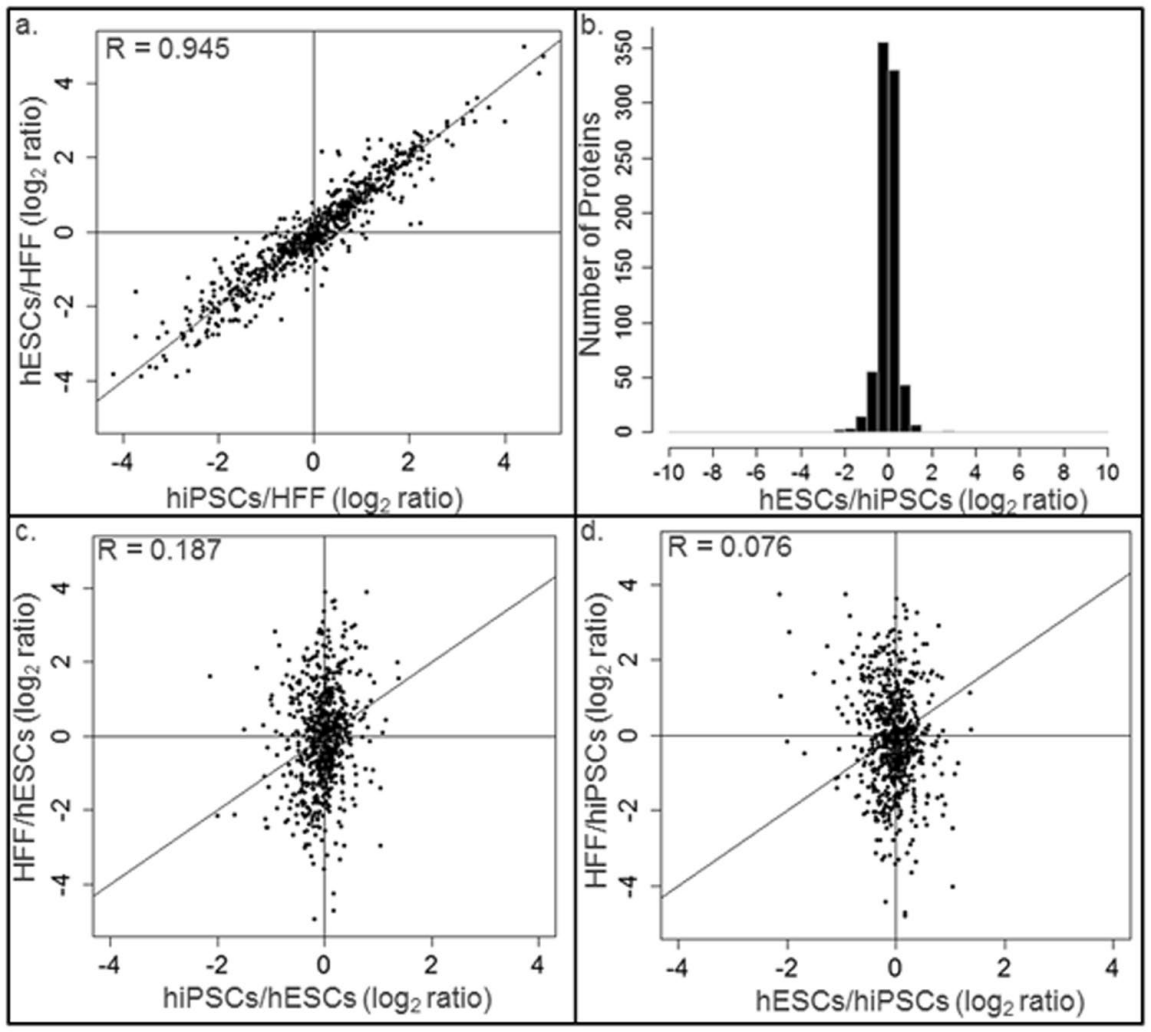
Similarity evaluation of the membrane proteomes among hiPSCs, hESCs, and HFF. (**a**) The protein log_2_ ratios of the profile hiPSCs/HFF are plotted against those of hESCs/HFF. A Spearman correlation coefficient of 0.945 was obtained. (**b**) The histogram depicted the distribution of protein log_2_ ratios of the profile hiPSCs/hESCs. (**c**) The protein log_2_ ratios of the profile hiPSCs/hESCs are plotted against those of HFF/hESCs. A Spearman correlation coefficient of 0.187 was obtained. (**d**) The protein log_2_ ratios of the profile hESCs/hiPSCs are plotted against those of HFF/hiPSCs. A Spearman correlation coefficient of 0.076 was obtained.

The protein log_2_ ratios of the hESCs/HFF profile against that of the hiPSCs/HFF profile were depicted in Fig. 2a. A Spearman correlation coefficient of 0.945 was received to show the high similarity between the proteomic profiles of hESCs and hiPSCs. Moreover, a histogram showing the distribution of the log_2_ ratios of the hiPSCs/hESCs profile was depicted in Fig. 2b. The narrow and high spike near the zero of the x-axis also revealed the high similarity between our hiPSC and hESC samples.

The protein log_2_ ratios of the HFF/hESCs against that of the hiPSCs/hESCs profile were depicted in Fig. 2c. A Spearman correlation coefficient of 0.187 revealed that our hiPSC samples were very different from HFF. The protein log_2_ ratios of the HFF/hiPSCs profile against that of the hECSs/hiPSCs profile were depicted in Fig. 2d. A Spearman correlation coefficient of 0.076 reveal that our hESC samples were also very different from HFF.

### Three-way pathway comparison of hiPSCs/HFF, hESCs/HFF, and hiPSCs/hESCs

#### Pathway comparison between hiPSCs/HFF and hESCs/HFF

To evaluate the results of the reprogramming of hiPSCs, we assessed the similarity between hiPSCs and hESCs at the functional level; we identified significant pathways associated with the profiles hiPSCs/HFF and hESCs/HFF. As shown in Table 1, the GSEA reported 13 significant pathways (4 up-regulated and 9 down-regulated) common to both hESCs/HFF and hiPSCs/HFF while 6 significant pathways (5 up-regulated and 1 down-regulated) exclusive to hiPSCs/HFF. The differentially expressed proteins that contributed to the significance of these pathways were summarized in Supplementary Table S3. Our results revealed that the two pluripotent cells did share some regulation patterns when compared to the somatic HFF cell. The four common down-regulated pathways *platelet activation signaling and aggregation*, *hemostasis*, *adaptive immune system*, and *immune system* are related to hemostasis and immune system. To the best of our knowledge, currently, no evidence was reported to show the regulation of immune-related signalling during reprogramming processes. The GSEA results in this study, however, showed that numerous down-regulated proteins were found enriched in the immune-related pathway between the pluripotent cells and their differentiated counterpart (Supplementary Table S3), which suggests the possible correlation between the reprogramming processes and immune signalling. For example, our results showed that TAP-1 (Antigen peptide transporter 1) and HLA-C (Major Histocompatibility Complex, Class I, C) were down-regulated in hESCs and hiPSCs; the two proteins were reported involved in an immune evasion mechanism of pluripotent cells^20^.

**Table 1:** Significant pathways of hiPSCs/HFF and hESCs/HFF.

Pathway	hiPSCs/HFF (p-val/FDR)	hESCs/HFF (p-val/FDR)
REACTOME: platelet activation signaling and aggregation	DOWN 0.000/0.000	DOWN 0.000/0.000
REACTOME: hemostasis	DOWN 0.000/0.002	DOWN 0.000/0.007
REACTOME: adaptive immune system	DOWN 0.002/0.003	DOWN 0.002/0.002
REACTOME: immune system	DOWN 0.000/0.003	DOWN 0.002/0.006
KEGG: regulation of actin cytoskeleton	DOWN 0.000/0.000	DOWN 0.000/0.000
KEGG: focal adhesion	DOWN 0.000/0.000	DOWN 0.000/0.001
KEGG: leukocyte transendothelial migration	DOWN 0.101/0.132	DOWN 0.060/0.088
REACTOME: axon guidance	DOWN 0.000/0.004	DOWN 0.000/0.010
REACTOME: developmental biology	DOWN 0.002/0.003	DOWN 0.002/0.009
REACTOME: TCA cycle and respiratory electron transport	UP 0.000/0.004	UP 0.000/0.001
KEGG: oxidative phosphorylation	UP 0.000/0.001	UP 0.000/0.000
KEGG: Alzheimer’s disease	UP 0.159/0.196	UP 0.084/0.074
KEGG: Huntington’s disease	UP 0.000/0.001	UP 0.000/0.000
KEGG: tight junction	DOWN 0.222/0.214	N/A—
REACTOME: respiratory electron transport ATP synthesis by chemiosmotic coupling and heat production by uncoupling proteins	UP 0.000/0.003	N/A—
KEGG: Parkinson’s disease	UP 0.000/0.001	N/A—
REACTOME: post-translational protein modification	UP 0.004/0.020	N/A 0.224/0.601
REACTOME: metabolism of proteins	UP 0.002/0.039	N/A 0.088/0.694
REACTOME: asparagine N-linked glycosylation	UP 0.105/0.204	N/A 0.534/0.626

UP: up-regulation; DOWN: down-regulation; N/A: not applicable; hiPSCs: human induced pluripotent stem cells; hESCs: human embryonic stem cells; HFF: human foreskin fibroblast; p-val: p-value; FDR: false discovery rate; —: not in GSEA.

Another three common down-regulated pathways *regulation of actin cytoskeleton*, *focal adhesion*, and *leukocyte transendothelial migration* are related to cell motility and cell adhesion. Previous studies have revealed that pluripotent stem cells adopt tight colony morphology with low adhesive strength, in contrast to the parental somatic fibroblast cells. The differentiation of hiPSCs significantly increases the adhesion strength to the ECM and the differences in adhesive strength were found correlated to more focal adhesions in parental cells than in hiPSCs^21^. Protein signatures related to focal adhesion such as vinculin, talin, actin stress fibre, and α_5_β_1_-integrin were found enriched in human fibroblasts when compared to hiPSCs, and both hESCs and hiPSCs possess lower adhesion strength than fibroblasts^21^. Moreover, to support stem cell self-renewal and prevent differentiation, the pluripotent stem cells were reported to promote the inactivation of focal adhesion kinase (FAK) signalling^22^. The FAK signalling was mentioned related to the focal adhesion pathway^23,24^. Based on our results along with the previous findings, the proteins related to focal adhesion pathway might be modulated to be down-regulated in stem cells to maintain the undifferentiated states.

**Figure 3:**
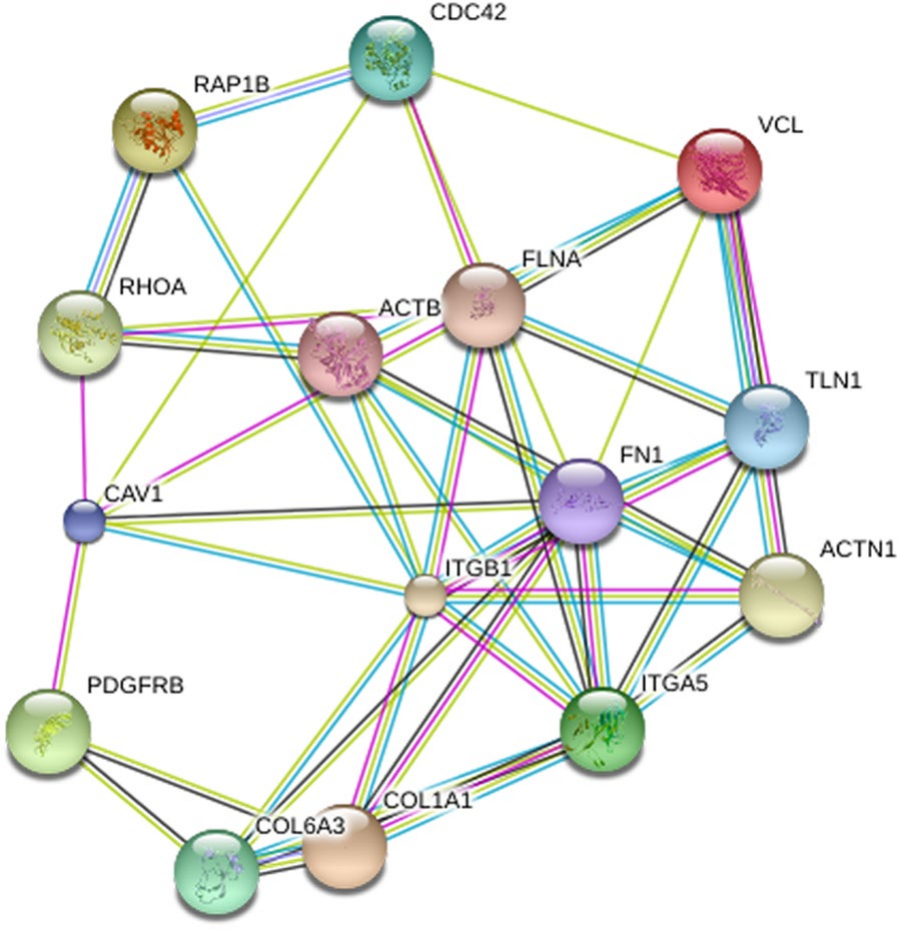
The protein interaction module in the *focal adhesion* pathway regulated between pluripotent cells and somatic fibroblast cells. This module was composed of 15 proteins that were enriched in the *focal adhesion* pathway. The interactions between these proteins were suggested by the STRING database.

In our proteomic data, the enriched protein PDGFRβ (Platelet-derived growth factor receptor-beta) was down-regulated in the *focal adhesion* pathway of both hESCs/HFF and hiPSCs/HFF. The PDGFRβ was reported up-regulated in human fibroblasts when compared to pluripotent cells and known to be involved in cell migration that is suggested related to focal adhesion^25,26^. Our results were basically in agreement with previous findings regarding the shift of adhesive properties during reprogramming^21^. All the enriched proteins in our study were further subjected to the STRING database for protein-protein interaction analysis. A tightly connected protein interaction module was formed by 15 enriched proteins (in both hESCs/HFF and hiPSCs/HFF) including small GTPase proteins RHOA (ras homolog gene family, member A), RAP1B (RAP1B, a member of RAS oncogene family), and CDC42 (cell division cycle 42). Remarkably, all the members of this module were found within the *focal adhesion* pathway, which implies that focal adhesion may function between pluripotent cells and differentiated fibroblast cells (Fig. 3 and Table 2).

**Table 2:** Fifteen enriched proteins within a tightly connected protein interaction module regulated between pluripotent cells and differentiated fibroblast cells in *focal adhesion* pathway.

Gene Symbol	Protein Description
ACTB	Actin, cytoplasmic 1
ACTN1	Alpha-actinin-1
CAV1	Isoform Alpha of Caveolin-1
CDC42	Isoform 2 of Cell division control protein 42 homolog
COL1A1	Collagen alpha-1(I) chain
COL6A3	alpha 3 type VI collagen isoform 4 precursor
FLNA	Isoform 2 of Filamin-A
FN1	Isoform 1 of Fibronectin
ITGA5	Integrin alpha-5
ITGB1	Isoform Beta-1A of Integrin beta-1
PDGFRB	Beta-type platelet-derived growth factor receptor
RAP1B	Ras-related protein Rap-1b
RHOA	Transforming protein RhoA
TLN1	Talin-1
VCL	Isoform 1 of Vinculin

The protein interaction modules were revealed based on the STRING database.

As to the remaining two common down-regulated pathways *axon guidance* and *development biology*, more than half of enriched proteins also appeared in the cell motility and cell adhesion pathways. Thus the down-regulation of these proteins in cell motility and cell adhesion may also contribute to the statistical significance of the two pathways.

On the other hand, the two pathways *TCA cycle and respiratory electron transport* and *oxidative phosphorylation* were found up-regulated in both pluripotent cells when compared to somatic fibroblast cells. The two pathways are mitochondrial-mediated metabolic pathways. Previous studies concluded that pluripotent cells favour glycolysis as a source of energy, whereas the differentiated somatic cells rely on mitochondrial oxidative phosphorylation for energy production^27^. Nevertheless, proper mitochondrial network integrity and functions were reported to be important for the maintenance of pluripotency^28^. Our results were in agreement with the finding of a recent membrane proteomic study on murine iPSCs; the up-regulated proteins in either murine ESCs or iPSCs were found enriched in oxidative phosphorylation and electron transport chain (relative to fibroblast cells)^29^. Another recent proteomic study on human iPSCs also obtained similar results that proteins involved in oxidative phosphorylation were up-regulated^30^. In addition, a study focusing on the energy metabolism of hiPSCs revealed that despite the lower mitochondrial activity, the protein expression level of mitochondrial complexes II, III, and V was found up-regulated in pluripotent cells when compared to differentiated cells possibly due to a higher level of c-myc^31^. All the aforementioned studies support our results (Table 1 and Supplementary Table S3) to the up-regulation of certain mitochondrial complex components in pluripotent cells at protein expression level. The elevated expression of numerous proteins in the electron transport was suggested to be involved in the stoichiometric change of the electron transport chain, which may affect the composition of respiratory supercomplexes and lead to a decrease in efficiency of oxidative phosphorylation^32,33^.

The remaining two common up-regulated pathways *Alzheimer’s disease* and *Huntington’s disease* are related to the biological signalling in diseases. As depicted in the KEGG database, oxidative phosphorylation and mitochondrial-related functions are involved in these disease pathways, the up-regulation of the mitochondrial-related proteins may also contribute to the statistical significance of the two disease pathways. During the pathogenesis of the two diseases, mitochondrial abnormalities and impaired mitochondrial functions have been revealed^34,35^. There is also evidence that the mitochondrial and related respiratory functions are modulated during the reprogramming process in favour of pluripotent glycolytic metabolism^27^. Alzheimer’s and Huntington’s diseases are known to be caused by the accumulation of disordered protein β-amyloid precursor protein (APP) and huntingtin (HTT), respectively^34,36^. Although the proteins APP was not found enriched in our membrane proteomic data, the level of APP was reported to be highly expressed in undifferentiated human transformed pluripotent stem cells^37^. Huntingtin was absent from our data because it is not a membrane protein. However, HTT is required for normal hematopoiesis and has been reported to be expressed in embryonic cells^38^. Whether or not the proteins APP and HTT are involved in the regulation of mitochondrial function during the reprogramming process may require further investigation.

We also investigated those pathways that were differentially regulated between hiPSCs/HFF and hESCs/HFF. As shown in Table 1, the three pathways *tight junction*, *respiratory electron transport ATP synthesis by chemiosmotic coupling and heat production by uncoupling proteins*, and *Parkinson’s disease* were found significant only in hiPSCs/HFF. As to the hESCs/HFF profile, the three pathways were not identified as significant by the GSEA due to the parameter setting of the GSEA; only those pathways containing no less than 20 mapped proteins can be served as the candidates of significant pathways. Both *tight junction* and *Parkinson’s disease* contained 19 proteins in the hESCs/HFF profile, and *respiratory electron transport ATP synthesis by chemiosmotic coupling and heat production by uncoupling proteins* contained 18 proteins in the hESCs/HFF. Since the number of mapped proteins of the three pathways was close to the threshold 20, the three pathways were suggested to be significant to both hiPSCs/HFF and hESCs/HFF.

While further dug into the *tight junction* pathway, we found that many down-regulated enriched proteins in both hiPSCs/HFF and hESCs/HFF were also enriched in cell motility and cell adhesion-related pathways. Since *tight junction* is known to be involved in cell adhesion signalling^39^, the *tight junction* pathway seems to possess similar down-regulation pattern as well as other cell motility and cell adhesion-related pathways. Likewise, many enriched proteins in *respiratory electron transport ATP synthesis by chemiosmotic coupling and heat production by uncoupling proteins* and *Parkinson’s disease* pathways were mostly the mitochondrial-related proteins that were up-regulated in *oxidative phosphorylation* and *TCA cycle and respiratory electron transport* pathways. As the aforementioned pathways *Alzheimer’s disease* and *Huntington’s disease*, the statistical significance of *respiratory electron transport ATP synthesis by chemiosmotic coupling and heat production by uncoupling proteins* and *Parkinson’s disease* may also come from the up-regulation of the mitochondrial-related pathways.

The three pathways *post-translational protein modification*, *metabolism of protein*, and *asparagine N-linked glycosylation* were also reported significant only in hiPSCs/HFF; the three pathways are related to protein metabolism and post-translational modification. We found that proteins UGGT1 (UDP-glucose glycoprotein glucosyltransferase 1), SLC25A4 (solute carrier family 25, member 4), PIGK (phosphatidylinositol glycan anchor biosynthesis, class K), and WBSCR17 (Williams-Beuren syndrome chromosome region 17) were up-regulated and enriched in hiPSCs/HFF but missed in hESCs/HFF. In addition, proteins DOLPP1 (dolichyl pyrophosphate phosphatase 1), FAU (ubiquitin-like protein FUBI), GALNT2 (polypeptide N-acetylgalactosaminyltransferase 2), GALNT7 (polypeptide N-acetylgalactosaminyltransferase 7), RPS19 (ribosomal protein S19), RPS20 (ribosomal protein S20), and PIGT (phosphatidylinositol glycan anchor biosynthesis) were up-regulated at a higher expression level in hiPSCs/HFF than in hESCs/HFF. Pathways related to protein metabolism and post-translational modification may possess different expression patterns among the two pluripotent cells while compared to somatic HFF.

#### Functional analysis of the differences between hiPSCs and hESCs

To reveal the difference in biological functions between hiPSCs and hESCs, we examined the significant pathways of the hiPSCs/hESCs profile. There were 11 pathways (4 up-regulated and 7 down-regulated) differentially regulated between the two pluripotent cells (Table 3). The 11 pathways can be characterized into three functional categories after a cross-reference to the significant pathways of hiPSCs/HFF and hESCs/HFF. As listed in Table 3, the three protein modification-related pathways *N-glycan biosynthesis*, *post-translational modification*, and *asparagine N-linked glycosylation* were up-regulated in hiPSCs/hESCs. The *post-translational modification* and *asparagine N-linked glycosylation* were also up-regulated in hiPSCs/HFF. None of the three was up-regulated in hESCs/HFF. The result indicates that the pathways related to protein modification might be specifically regulated in hiPSCs when compared to either pluripotent hESCs or somatic HFF.

**Table 3:** Three-way pathway comparison of hiPSCs/hESCs, hiPSCs/HFF, and hESCs/HFF.

Pathway	hiPSCs/hESCs (p-val/FDR)	hiPSCs/HFF (p-val/FDR)	hESCs/HFF (p-val/FDR)
KEGG: N-glycan biosynthesis	UP 0.006/0.013	N/A—	N/A—
REACTOME: post translational protein modification	UP 0.000/0.006	UP 0.004/0.020	N/A 0.224/0.601
REACTOME: asparagine N-linked glycosylation	UP 0.008/0.037	UP 0.105/0. 204	N/A 0.534/0.626
KEGG: focal adhesion	UP 0.062/0.111	DOWN 0.000/0.000	DOWN 0.000/0.001
KEGG: oxidative phosphorylation	DOWN 0.033/0.232	UP 0.000/0.001	UP 0.000/0.000
REACTOME: TCA cycle and respiratory electron transport	DOWN 0.056/0.187	UP 0.000/0.004	UP 0.000/0.001
KEGG: Alzheimer’s disease	DOWN 0.066/0.088	UP 0.159/0.196	UP 0.084/0.074
KEGG: Huntington’s disease	DOWN 0.057/0.113	UP 0.000/0.001	UP 0.000/0.000
KEGG: Parkinson’s disease	DOWN 0.063/0.150	UP 0.000/0.001	N/A—
REACTOME: respiratory electron transport ATP synthesis by chemiosmotic coupling and heat production by uncoupling proteins	DOWN 0.032/0.223	UP 0.000/0.003	N/A —
REACTOME: mitochondrial protein import	DOWN 0.061/0.144	N/A —	N/A —

UP: up-regulation; DOWN: down-regulation; N/A: not applicable; hiPSCs: human induced pluripotent stem cells; hESCs: human embryonic stem cells; HFF: human foreskin fibroblast; p-val: p-value; FDR: false discovery rate; —: not in GSEA.

**Figure 4:**
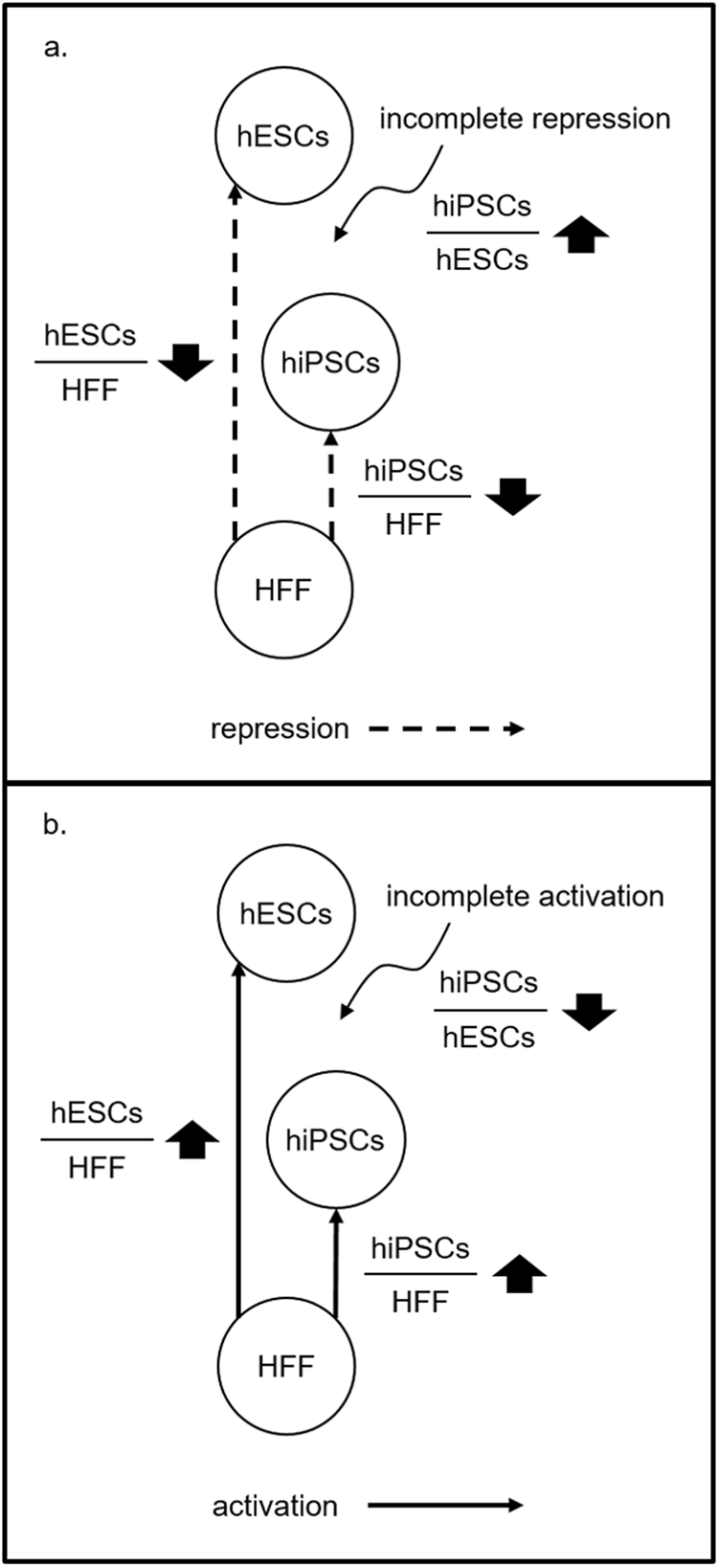
The hypothetical incomplete regulation of pathways during the reprogramming of hiPSCs. (**a**) Down-regulation in hiPSCs/HFF and hESCs/HFF and up-regulation in hiPSCs/hESCs suggest an incomplete repression of pathways. (**b**) Up-regulation in hiPSCs/HFF and hESCs/HFF and down-regulation in hiPSCs/hESCs suggest an incomplete activation of pathways.

The pathway *focal adhesion* was found to be significant in all the three profiles hiPSCs/hESCs, hiPSCs/HFF, and hESCs/HFF. The pathway was up-regulated in hiPSCs/hESCs and down-regulated in both hiPSCs/HFF and hESCs/HFF. The down-regulation of *focal adhesion* in both hiPSCs/HFF and hESCs/HFF suggested that the pathway may be less activated in both pluripotent cells than in somatic HFF cells. The up-regulation of *focal adhesion* in hiPSCs/hESCs further revealed that the pathway may be more activated in hiPSCs than in hESCs. On the basis of the regulation direction of *focal adhesion* in the three profiles, we hypothesized that *focal adhesion* may undergo an incomplete repression during the reprogramming process of hiPSCs (Fig. 4a).

There were 7 down-regulated significant pathways *TCA cycle and respiratory electron transport*, *respiratory electron transport ATP synthesis by chemiosmotic coupling and heat production by uncoupling proteins*, *mitochondrial protein import*, *oxidative phosphorylation*, *Alzheimer’s disease*, *Huntington’s disease*, and *Parkinson’s disease* in hiPSCs/hESCs. When cross-referencing to the significant pathways of hiPSCs/HFF and hESCs/HFF, pathways related to mitochondria functions (*oxidative phosphorylation* and *TCA cycle and respiratory electron transport*) and diseases (*Alzheimer’s disease* and *Huntington’s disease*) were up-regulated in both hiPSCs/HFF and hESCs/HFF. The 4 pathways are either mitochondrial-mediated metabolic pathways or selected as significant pathways because most of their members are involved in mitochondrial function. We therefore paid our attention only to those mitochondrial-related pathways. According to the regulation direction of the mitochondrial-related pathways in the three profiles, *oxidative phosphorylation* and *TCA cycle and respiratory electron transport* were hypothesized to undergo an incomplete activation during the reprogramming process of hiPSCs (Fig. 4b).

The reprogramming of hiPSCs has been shown to be a multi-stage process that involves large-scale changes in the transcriptional and epigenetic states of somatic cells. Several expressed genes and pathways in somatic cells were suggested to act as antagonizing barriers during the reprogramming^40^. The barriers may jeopardize the changes in the adhesive signature in reprogramming and lead to the incomplete regulation of the *focal adhesion* pathways. Besides, metabolic change may serve as a vital progress during the reprogramming of hiPSCs^41^. The genes involved in glycolysis and oxidative phosphorylation undergo epigenetic and gene expression changes for progressive resetting of metabolite levels^42^. The reprogrammed iPSCs, however, may still retain specific chromatin modifications related to cellular respiration and metabolism. Similar to the epigenetic memory, a notion of “metabolic memory” retained in iPSCs was proposed to detain the metabolic reprogramming process and provide a metabolic barrier for reprogramming^43^. The reprogramming barriers and metabolic memory may lead to the incomplete regulation of hiPSCs.

There were several membrane proteins that were enriched in the aforementioned significant pathways and whose regulation directions concurred with the regulation directions of these pathways. These proteins may serve as the starting points of the research to make hiPSCs closer to hESCs since membrane proteins usually play an important role in biological signalling. For the hypothetical incomplete repressed pathway *focal adhesion*, several proteins such as ACTN1 (actinin, alpha 1), VCL, and Rap1B were down-regulated in both hiPSCs/HFF and hESCs/HFF but up-regulated in hiPSCs/hESCs (Table 4A). For the hypothetical incomplete activated pathways *oxidative phosphorylation* and *TCA cycle and respiratory electron transport*, several proteins such as UQCRQ (ubiquinol-cytochrome c reductase, complex III subunit VII), CYC1 (cytochrome c 1), COX4I1 (cytochrome c oxidase subunit IV isoform 1), and ATP5H (ATP synthase, H+ transporting, mitochondrial F0 complex, subunit d) were found up-regulated in hiPSCs/HFF and hESCs/HFF and down-regulated in hiPSC/hESCs (Table 4B). These membrane proteins might all be used for the further study of dissimilarity between hiPSCs and hESCs.

**Table 4:** The expression log_2_ ratio of the discriminated proteins in the hypothetical incomplete regulated pathways. (**A**) Pathway *focal adhesion* (**B**) Pathways *oxidative phosphorylation* and *TCA cycle and respiratory electron transport*.

Gene Symbol	hiPSCs/hESCs	hiPSCs/HFF	hESCs/HFF
**A**.			
ACTN1	0.355	−2.251	−2.696
CAV1	0.195	−3.113	−3.461
FN1	0.350	−1.622	−1.992
RAP1B	0.394	−1.632	−2.044
RHOA	0.233	−1.010	−1.265
VCL	0.647	−1.647	−2.371
**B**.			
ATP5H	−0.361	2.195	2.603
ATP5J2	−0.283	1.804	2.089
ATP5L	−0.329	1.498	1.917
COX4I1	−0.429	1.299	1.794
CYC1	−0.578	0.813	1.576
NDUFA9	−0.427	0.918	1.216
NDUFB10	−0.227	1.319	1.403
NDUFB6	−0.221	2.024	2.223
UQCRFS1	−0.501	1.793	2.349
UQCRQ	−0.577	2.115	2.672

Even though a comprehensive three-way pathway comparison has been performed on the three profiles hiPSCs/hESCs, hiPSCs/HFF, and hESCs/HFF, we are not sure if the obtained hypothetical incomplete repressed and activated pathways are actually involved in the biological events behind the reprogramming process of hiPSCs. To address this issue, we surveyed related proteomic studies and found a proteomics research that supports our results to certain degree^5^. There were two human fibroblast (HF) cell lines IMR90 fetal fibroblasts and 4Skin fetal fibroblasts used in the study, and each of them had two biological replicas. The study therefore obtained 12 profiles of protein relative expression ratio, in which 4 profiles recorded log_2_ ratios of hiPSCs to somatic cells, 4 recorded log_2_ ratios of hESCs to somatic cells, and 4 recorded log_2_ ratios of hiPSCs to hESCs. To be compared to our results, the 12 obtained profiles were subjected to our data integration procedure to produce three integrated profiles, one for hiPSCs versus somatic HFs (hiPSCs/HFs), one for hESCs versus somatic HFs (hESCs/HFs), and one for hiPSCs versus hESCs (hiPSCs/hESCs). The three integrated profiles are shown in Table S4. The three profiles were subjected to GSEA to perform pathway analyses; the identified significant pathways were summarized in Supplementary Table S5 and the differentially expressed proteins that contributed to the significance of these pathways were summarized in Supplementary Table S6. We adopted the same strategy of our pathway analysis. The identified significant pathways of hiPSCs/hESCs were cross-referenced to those of hiPSCs/HFs and hESCs/HFs, and the results are listed in Table 5. The results showed that the pathway *focal adhesion* was up-regulated in hiPSCs/hESCs and down-regulated in both hiPSCs/HFs and hESCs/HFs, which is in agreement with our result: *focal adhesion* may undergo an incomplete repression process during the reprogramming of hiPSCs. On the other hand, the pathway *TCA cycle and respiratory electron transport* was found down-regulated in hiPSCs/hESCs but missed in both hESCs/HFs and hiPSCs/HFs. Although the mitochondrial-related pathway did not totally possess the same regulation patterns as ours, hiPSCs in the study also showed the same trend towards up-regulation when compared to hESCs as revealed by our study. The conclusion drawn from this study is similar to ours, which greatly increase the confidence of our results because there are usually significant differences among pluripotent cells obtained from different laboratories. The *focal adhesion* and mitochondrial-related pathways are therefore suggested to be incomplete events during the reprogramming process of hiPSCs.

**Table 5:** Three-way pathway comparison of hiPSCs/hESCs, hiPSCs/HFs, and hESCs/HFs on a public dataset.

Pathways	hiPSCs/hESCs (p-val /FDR)	hiPSCs/HFs (p-val/FDR)	hESCs/HFs (p-val/FDR)
KEGG: DNA replication	UP 0.096/0.245	UP 0.000/0.000	UP 0.000/0.000
KEGG: ECM-receptor interaction	UP 0.003/0.025	DOWN 0.000/0.000	DOWN 0.000/0.000
KEGG: arrhythmogenic right ventricular cardiomyopathy (ARVC)	UP 0.011/0.145	DOWN 0.051/0.060	DOWN 0.011/0.013
KEGG: focal adhesion*	UP 0.003/0.136	DOWN 0.000/0.000	DOWN 0.000/0.000
KEGG: regulation of actin cytoskeleton	UP 0.002/0.107	DOWN 0.000/0.000	DOWN 0.000/0.000
KEGG: epithelial cell signaling in helicobacter pylori infection	UP 0.083/0.247	DOWN 0.076/0.118	DOWN 0.017/0.021
REACTOME: cell surface interactions at the vascular wall	UP 0.002/0.185	DOWN 0.031/0.031	DOWN 0.004/0.006
REACTOME: integrin cell surface interactions	UP 0.003/0.123	DOWN 0.005/0.007	DOWN 0.004/0.005
REACTOME: glucose transport	DOWN 0.000/0.000	UP 0.000/0.000	UP 0.000/0.000
REACTOME: regulation of glucokinase by glucokinase regulatory protein	DOWN 0.000/0.000	UP 0.000/0.000	UP 0.000/0.000
REACTOME: transport of ribonucleoproteins into the host nucleus	DOWN 0.000/0.000	UP 0.000/0.000	UP 0.000/0.000
REACTOME: processing of capped intron-containing pre-mRNA	DOWN 0.000/0.000	UP 0.000/0.000	UP 0.000/0.000
REACTOME: NEP/NS2 interacts with the cellular export machinery	DOWN 0.027/0.030	UP 0.000/0.000	UP 0.000/0.000
KEGG: arginine and proline metabolism	DOWN 0.055/0.058	N/A 0.615 /0.684	UP 0.053/0.105
KEGG: fatty acid metabolism	DOWN 0.101/0.198	N/A 0.946/1.000	UP 0.025/0.078
KEGG: peroxisome	DOWN 0.000/0.001	N/A 0.589/0.623	N/A 0.388/0.553
REACTOME: TCA cycle and respiratory electron transport*	DOWN 0.000/0.042	N/A 0.999/1.000	N/A 0.327/0.442
REACTOME: respiratory electron transport ATP synthesis by chemiosmotic coupling and heat production by uncoupling proteins*	DOWN 0.086/0.224	N/A 0.728/0.778	N/A 0.558/0.641
REACTOME: pyruvate metabolism and citric acid (TCA) cycle	DOWN 0.000/0.005	N/A 0.998/1.000	N/A 0.539/0.639

^*^Significant pathways found in both public dataset and our dataset; UP: up-regulation; DOWN: down-regulation; N/A: not applicable; hiPSCs: human induced pluripotent stem cells; hESCs: human embryonic stem cells; HF: human fibroblast; p-val: p-value; FDR: false discovery rate.

## Methods

### Culture of hiPSCs and hESCs

Two hESC cell lines NTU1 and H9^44,45^, two hiPSC cell lines CFB46 and CFB50 (retroviral reprogrammed and passaged from our previous studies)^46,47^, and their somatic precursor HFF cells (human foreskin fibroblast cells) were prepared for this study. Both hiPSC cell lines and the two hESC cell lines were maintained on MEF (mouse embryonic fibroblast feeder) with the serum-free medium as previously described^47,48^, and the somatic precursor HFF cells were obtained and cultured in DMEM with 20% FBS (fetal bovine serum) as described in a previous study as well^47^.

### Membrane fractionation and gel-assisted digestion of membrane proteins

The isolation of membrane proteins and the following gel-assisted digestion were performed using the protocol we conducted in our membrane proteomic study^49^. In brief, after washed with PBS (phosphate buffered saline), the cells were collected and homogenized in hypotonic buffer (1.5 mM MgCl_2_, 10 mM HEPES (4-(2-hydroxyethyl)-1-piperazineethanesulfonic acid), pH 7.5, 10 mM KCl, and 1 × protease inhibitor mixture (Calbiochem)). We first removed nuclei by a centrifugation at 3,000 × *g* for 10 min at 4 °C. The post-nuclear supernatant was then mixed with 1.8 M sucrose and subjected to a second centrifugation at 13,000 × *g* for 1 h at 4 °C to obtain membrane pellet. After washed with ice-cold 0.1 M Na_2_CO_3_ (pH 11.5), the membrane pellet was dissolved in 90% (v/v) formic acid and vacuum-dried.

To prepare samples for following MS analyses, the membrane pellet was subjected to gel-assisted digestion^49^. The membrane protein pellet was first resuspended and added with 5 mM TCEP (Tris(2-carboxyethyl) phosphine hydrochloride) and 2 mM MMTS (methyl methanethiosulfonate) for protein reduction and protein alkylation, respectively. Next, the protein sample was applied with 10% (w/v) APS (Ammonium persulfate), Acrylamide/bisacrylamide (40%, 29:1, v/v), and 100% TEMED (N,N,N’,N’-tetramethylethylenediamine) to polymerize as a gel. After the gel was cut into small pieces and washed, the tryptic digestion (protein: trypsin 10:1, w/w) was performed in 25 mM TEABC (triethylammonium bicarbonate) at 37 °C overnight. A sequential extraction with 25 mM TEABC, 0.1% (v/v) TFA (trifluoroacetic acid) in water, 0.1% (v/v) TFA in ACN (acetonitrile), and 100% ACN was performed to extract peptides from the gel. The peptides were then concentrated in a SpeedVac and subjected to MS analysis.

### Mass spectrometry analysis for protein identification and quantification

In this study, the liquid chromatography-tandem mass spectrometry (LC-MS/MS) analyses were performed for protein identification and quantitation using Waters Q-TOF Premier mass spectrometer (Waters Corp., Milford, MA). Follow our previous study^50^, the samples were first injected into a trap column (20-mm × 180-μm), separated by a capillary column (200-mm × 75 mm), and eluted with a linear gradient of 0–80% of 0.1% (v/v) FA in ACN at 300 nl/min for 120 min. Data acquisition was operated under the duty cycle of full MS scan (m/z 400–1600, 0.6 s) followed by three MS/MS scans (m/z 100–1990, 1.2 s for each scan) on the three highest peaks present in the full MS scan.

The MS/MS data were processed and searched against International Protein Index (IPI) human database^51^ (v3.29, 68161 sequences) using Mascot v2.2 (Matrix science, London, United Kingdom, http://www.matrixscience.com/). The search parameters were set as follows: 0.3-Da and 0.1-Da mass tolerances for MS and MS/MS fragment ions, respectively; oxidation (Met) and methylation (Cys) as variable modifications; up to 2 missed cleavages. Only unique peptides with scores higher than 35 (p < 0.05) were confidently assigned. A decoy database search was also performed to evaluate the false discovery rate of protein identification by searching against a Mascot-created randomize protein sequence database with identical validation criteria and search parameters. The Mascot search results were exported in extensive markup language (XML) data format.

In the current study, protein quantitation was performed by a label-free approach using the IDEAL-Q software^52^. The spectral raw data were first converted into mzXML format using a computational tool, massWolf v4.0 (http://sourceforge.net/projects/sashimi/files/massWolf (MassLynx converter)). The obtained mzXML data coupled with Mascot search results in XML format were then submitted to IDEAL-Q for quantitation analysis. The peptide abundance was determined by extracted ion chromatography (XIC) and further normalized to internal peptide standard. The protein ratio was calculated by the weighted ratio of normalized peptide abundance in different samples.

### Data processing and integration

For a comparative proteomics analysis, a protein profile of expression ratio is basically expected for the following pathway analyses. The scale of spectral proteomic data, however, is usually much smaller than that of genomic data. To quantify as many peptides as possible, for each sample we produced two spectral datasets by repeated MS experiments. The two datasets were subjected to the IDEAL-Q to perform protein identification and generate two protein profiles of expression ratio for the sample; the two profiles were further integrated into a single one for pathway analyses. The integration of the two profiles was done as follows. First, all the protein expression ratios were transformed into log_2_ ratios. Next, for each protein that had a different log_2_ ratio in the two profiles, we either removed the protein from our profiles or determined a unique log_2_ ratio for the protein based on the following two criteria. 1) If the expression of the redundant protein was in opposite up- and down-regulation directions in the two profiles and the inconsistency in expression is significant (numerical difference between the two fold changes >5), the protein was removed. 2) Otherwise, the log_2_ ratio of the protein was set to be the average of the two log_2_ ratios of the protein. Finally, all the left proteins were pooled together to form a new profile. We totally obtained eight integrated membrane protein profiles of expression ratio for the following eight experimental samples: hiPSC CFB46 versus somatic precursor HFF, hiPSC CFB50 versus somatic precursor HFF, hESC NTU1 versus somatic precursor HFF, hESC H9 versus somatic precursor HFF, hiPSC CFB46 versus hESC NTU1, hiPSC CFB46 versus hESC H9, hiPSC CFB50 versus hESC NTU1, and hiPSC CFB50 versus hESC H9. The eight profiles respectively contained 1100, 1111, 1110, 1105, 1125, 1115, 1128, and 1122 protein expression ratios.

The eight profiles were further integrated into three profiles: one for hiPSCs versus somatic precursor HFF (hiPSCs/HFF), one for hESCs versus somatic precursor HFF (hESCs/HFF), and one for hiPSCs versus hESCs (hiPSCs/hESCs). We generated the hiPSCs/HFF profile by merging two hiPSC biological replicas, hiPSC CFB46 and hiPSC CFB50. Similarly, the hESCs/HFF profile was obtained by merging two hESC cell lines, hESC NTU1 and hESC H9. To generate the hiPSCs/hESCs profile, we first generated a profile for hiPSCs versus hESC NTU1 and a profile for hiPSCs versus hESC H9 by merging hiPSC biological replicas, and further integrated the two profiles into the hiPSCs/hESCs profile by merging the two hESC cell lines. All the aforementioned merges of two profiles were done by selecting those proteins quantified in both profiles, and the log_2_ ratio of each selected protein was set to be the average of the log_2_ ratios of the protein in the two source profiles.

### Statistical correlation measure

Since the distributions of the protein expression ratios in our datasets were not characterized by any parameter, the Spearman correlation coefficients were therefore used to evaluate the similarity between our profiles. Given two ranked protein expression profiles **x** and **y**, the Spearman correlation coefficient *r*_*s*_ of the two profiles is defined as follows:

$${r}_{s}=1-\frac{6\times {\sum }_{i=1}^{n}{({x}_{i}-{y}_{i})}^{2}}{n({n}^{2}-1)},$$

where *n* is the number of protein expression ratios in a profile, and *x*_*i*_ and *y*_*i*_ are protein expression ratios belong to **x** and **y**, respectively. *r*_*s*_ receives a value of +1 or −1 if **x** and **y** are perfectly monotonic positive or negative correlated, respectively; it receives a value of zero if **x** and **y** are not correlated with each other. In this study, the calculation of the Spearman correlation coefficients was carried out by the R language (https://www.r-project.org/).

### Enrichment of significant pathway

The bioinformatics tool Gene Set Enrichment Analysis (GSEA)^18,19^ was used in this study to enrich the target pathways with statistically significant difference between two cell types (e.g., hiPSC versus hESC). Given a protein profile sorted by the expression ratio between the two cell types, the target pathway is regarded as significant if most of its protein members are enriched in the top (up-regulated) or bottom (down-regulated) region of the profile. On the basis of the Kolmogorov-Smirnov test, the GSEA first calculates an ES score (enrichment score) for the target pathway to measure the degree to which the pathway is enriched in the top-ranked or bottom-ranked region of the profile. Next, a normalized ES (NES) is calculated from the ES to take into account the pathway size. Finally, a false discovery rate (FDR) is determined based on the NES to represent the significance of the target pathway. The GSEA takes into consideration all the proteins from the experimental results without any filtration procedure. Thus, all the proteins are able to contribute to the enrichment analysis even if some of them have a very small fold change in expression^13^. In this study, the parameters of the GSEA were empirically set as follows:

1. The “difference of classes” metric was selected to rank proteins according to the log_2_ ratio between two phenotypic classes.
2. The canonical pathways from KEGG database (MSigDB, v5.0)^14,15^ and Reactome (MSigDB, v5.0)^53,54^ databases were selected as our target pathways.
3. The size of a target pathway ranged from 20 to 200 proteins.
4. The permutation test of type “gene_set” was performed 1000 times for the target pathway.
5. The pathways with an FDR <25% were reported as significant pathways.

### Protein-protein interaction database

To identify protein functions behind our experimental data, the protein-protein interaction database STRING (Search Tool for the Retrieval of Interacting Genes)^55^ was used to capture the functional modularity and interconnectivity among input proteins. Protein interaction relationship is encoded into networks in the STRING database. Proteins are represented by nodes in the networks and known or predicted interactions are represented by edges. Each edge in the networks is labelled a score in the range between 0 and 1 to represent the confidence level of the associated interaction. In this study, Homo sapiens was used as our model organism; those enriched proteins revealed by the GSEA pathway analyses were subjected to STRING v10 to identify interactions among these proteins with a confidence score higher than 0.4.

## Electronic supplementary material

Supplementary Tables S1-S6

Supplementary Tables S1

Supplementary Tables S2

Supplementary Tables S3

Supplementary Tables S4

Supplementary Tables S5

Supplementary Tables S6

## References

[1] Takahashi K, Yamanaka S (2006). Induction of pluripotent stem cells from mouse embryonic and adult fibroblast cultures by defined factors. Cell.

[2] Reiland S, Salekdeh GH, Krijgsveld J (2011). Defining pluripotent stem cells through quantitative proteomic analysis. Expert Rev. Proteomics.

[3] Chin MH (2009). Induced pluripotent stem cells and embryonic stem cells are distinguished by gene expression signatures. Cell Stem Cell.

[4] Liu Y, Cheng D, Li Z, Gao X, Wang H (2012). The gene expression profiles of induced pluripotent stem cells (iPSCs) generated by a non-integrating method are more similar to embryonic stem cells than those of iPSCs generated by an integrating method. Genet. Mol. Biol..

[5] Munoz J (2011). The quantitative proteomes of human-induced pluripotent stem cells and embryonic stem cells. Mol. Syst. Biol..

[6] Kim SY (2012). Comparative proteomic analysis of human somatic cells, induced pluripotent stem cells, and embryonic stem cells. Stem Cells Dev..

[7] Gudjonsson T, Magnusson MK (2005). Stem cell biology and the cellular pathways of carcinogenesis. APMIS.

[8] Volonté C, D’Ambrosi N (2009). Membrane compartments and purinergic signalling: the purinome, a complex interplay among ligands, degrading enzymes, receptors and transporters. FEBS J..

[9] Ludwig JA, Weinstein JN (2005). Biomarkers in cancer staging, prognosis and treatment selection. Nat. Rev. Cancer.

[10] Harkness L (2008). Identification of a membrane proteomic signature for human embryonic stem cells independent of culture conditions. Stem Cell Research.

[11] Bonardi F (2013). *A Proteomics and Transcriptomics A*pproach to Identify Leukemic Stem Cell (LSC) Markers. Molecular & Cellular Proteomics.

[12] Helbig AO, Heck AJR, Slijper M (2010). Exploring the membrane proteome–challenges and analytical strategies. J. Proteomics.

[13] Huang DW, Sherman BT, Lempicki RA (2009). Bioinformatics enrichment tools: paths toward the comprehensive functional analysis of large gene lists. Nucleic Acids Res..

[14] Kanehisa M, Goto S (2000). KEGG: kyoto encyclopedia of genes and genomes. Nucleic Acids Res..

[15] Kanehisa M, Goto S, Sato Y, Furumichi M, Tanabe M (2012). KEGG for integration and interpretation of large-scale molecular data sets. Nucleic Acids Res..

[16] Phanstiel DH (2011). Proteomic and phosphoproteomic comparison of human ES and iPS cells. Nat. Methods.

[17] Yamana R (2013). Rapid and deep profiling of human induced pluripotent stem cell proteome by one-shot NanoLC-MS/MS analysis with meter-scale monolithic silica columns. J. Proteome Res..

[18] Subramanian A (2005). Gene set enrichment analysis: a knowledge-based approach for interpreting genome-wide expression profiles. Proc. Natl. Acad. Sci. USA.

[19] Mootha VK (2003). PGC-1alpha-responsive genes involved in oxidative phosphorylation are coordinately downregulated in human diabetes. Nat. Genet..

[20] Suárez-Alvarez B (2010). Epigenetic mechanisms regulate MHC and antigen processing molecules in human embryonic and induced pluripotent stem cells. PLoS One.

[21] Singh A (2013). Adhesion strength-based, label-free isolation of human pluripotent stem cells. Nat. Methods.

[22] Villa-Diaz LG, Kim JK, Laperle A, Palecek SP, Krebsbach PH (2016). Inhibition of Focal Adhesion Kinase Signaling by Integrin α6β1 Supports Human Pluripotent Stem Cell Self-Renewal. STEM CELLS.

[23] Parsons JT, Slack-Davis J, Tilghman R, Roberts WG (2008). Focal Adhesion Kinase: Targeting Adhesion Signaling Pathways for Therapeutic Intervention. Clinical Cancer Research.

[24] Wozniak MA, Modzelewska K, Kwong L, Keely PJ (2004). Focal adhesion regulation of cell behavior. Biochimica et Biophysica Acta (BBA) - Molecular Cell Research.

[25] Hewitt KJ (2012). PDGFRβ expression and function in fibroblasts derived from pluripotent cells is linked to DNA demethylation. J. Cell Sci..

[26] Gupton SL, Eisenmann K, Alberts AS, Waterman-Storer CM (2007). mDia2 regulates actin and focal adhesion dynamics and organization in the lamella for efficient epithelial cell migration. J. Cell Sci..

[27] Folmes CDL (2011). Somatic oxidative bioenergetics transitions into pluripotency-dependent glycolysis to facilitate nuclear reprogramming. Cell Metab..

[28] Xu X (2013). Mitochondrial regulation in pluripotent stem cells. Cell Metab..

[29] Hao J (2013). Reprogramming- and pluripotency-associated membrane proteins in mouse stem cells revealed by label-free quantitative proteomics. J. Proteomics.

[30] Pripuzova NS (2015). Development of a protein marker panel for characterization of human induced pluripotent stem cells (hiPSCs) using global quantitative proteome analysis. Stem Cell Res..

[31] Varum S (2011). Energy metabolism in human pluripotent stem cells and their differentiated counterparts. PLoS One.

[32] Qin, H. *et al*. Systematic Identification of Barriers to Human iPSC Generation. *Cell* **158**, 449–461, 10.1016/j.cell.2014.05.040. 10.1016/j.cell.2014.05.040 PMC4130998 25036638

[33] Hansson, J. *et al*. Highly Coordinated Proteome Dynamics during Reprogramming of Somatic Cells to Pluripotency. *Cell Reports* **2**, 1579–1592, 10.1016/j.celrep.2012.10.014. 10.1016/j.celrep.2012.10.014 PMC4438680 23260666

[34] Yao J, Brinton RD (2012). Estrogen Regulation of Mitochondrial Bioenergetics. Advances in Pharmacology.

[35] Spuch, C., Ortolano, S. & Navarro, C. New Insights in the Amyloid-Beta Interaction with Mitochondria. *Journal of Aging Research* **2012**, 10.1155/2012/324968 (2012). 10.1155/2012/324968 PMC3317193 22523685

[36] Jin YN, Johnson GVW (2010). The interrelationship between mitochondrial dysfunction and transcriptional dysregulation in Huntington disease. Journal of Bioenergetics and Biomembranes.

[37] Venkataramani V (2012). Amyloid Precursor Protein Is a Biomarker for Transformed Human Pluripotent Stem Cells. The American Journal of Pathology.

[38] Metzler M (2000). Huntingtin is required for normal hematopoiesis. Human Molecular Genetics.

[39] Matter K, Balda MS (2003). Signalling to and from tight junctions. Nat. Rev. Mol. Cell Biol..

[40] Boekema EJ, Braun H-P (2007). Supramolecular Structure of the Mitochondrial Oxidative Phosphorylation System. Journal of Biological Chemistry.

[41] Panopoulos, A. D. *et al*. The metabolome of induced pluripotent stem cells reveals metabolic changes occurring in somatic cell reprogramming. *Cell Res* **22**, 168–177, http://www.nature.com/cr/journal/v22/n1/suppinfo/cr2011177s1.html (2012). 10.1038/cr.2011.177 PMC3252494 22064701

[42] Zhang J (2012). Michael A. Metabolic Regulation in Pluripotent Stem Cells during Reprogramming and Self-Renewal. Cell Stem Cell.

[43] Kim, K. *et al*. Epigenetic memory in induced pluripotent stem cells. *Nature* **467**, 285–290, http://www.nature.com/nature/journal/v467/n7313/abs/nature09342.html#supplementary-information (2010). 10.1038/nature09342 PMC3150836 20644535

[44] Thomson JA (1998). Embryonic stem cell lines derived from human blastocysts. Science.

[45] Chen HF (2007). Derivation, characterization and differentiation of human embryonic stem cells: comparing serum-containing versus serum-free media and evidence of germ cell differentiation. Hum. Reprod..

[46] Huang H-P (2010). Factors from human embryonic stem cell-derived fibroblast-like cells promote topology-dependent hepatic differentiation in primate embryonic and induced pluripotent stem cells. J. Biol. Chem..

[47] Huang H-P (2011). Epithelial cell adhesion molecule (EpCAM) complex proteins promote transcription factor-mediated pluripotency reprogramming. J. Biol. Chem..

[48] Chuang C-Y (2012). Meiotic competent human germ cell-like cells derived from human embryonic stem cells induced by BMP4/WNT3A signaling and OCT4/EpCAM (epithelial cell adhesion molecule) selection. J. Biol. Chem..

[49] Han C-L (2008). A multiplexed quantitative strategy for membrane proteomics: opportunities for mining therapeutic targets for autosomal dominant polycystic kidney disease. Mol. Cell. Proteomics.

[50] Han, C.-L. *et al*. An informatics-assisted label-free approach for personalized tissue membrane proteomics: case study on colorectal cancer. *Mol. Cell. Proteomics* **10**, M110.003087, 10.1074/mcp.M110.003087 (2011). 10.1074/mcp.M110.003087 PMC3069341 21209152

[51] Kersey PJ (2004). The International Protein Index: an integrated database for proteomics experiments. Proteomics.

[52] Tsou C-C (2010). IDEAL-Q, an automated tool for label-free quantitation analysis using an efficient peptide alignment approach and spectral data validation. Mol. Cell. Proteomics.

[53] Milacic M (2012). Annotating cancer variants and anti-cancer therapeutics in reactome. Cancers.

[54] Croft D (2013). Building models using Reactome pathways as templates. Methods Mol. Biol..

[55] Snel B, Lehmann G, Bork P, Huynen MA (2000). STRING: a web-server to retrieve and display the repeatedly occurring neighbourhood of a gene. Nucleic Acids Res..

