# Supplementary material for: Combining membrane proteomics and computational three-way pathway analysis revealed signalling pathways preferentially regulated in human iPSCs and human ESCs
Source: Sci Rep. 2017 Nov 8;7:15055. doi: 10.1038/s41598-017-15347-z (PMC5678157; doi:10.1038/s41598-017-15347-z)
Supplement: Supplementary file 1 — Supplementary Tables S1-S6 [file 41598_2017_15347_MOESM1_ESM.pdf]

Combining membrane proteomics and computational three-way pathway analysis revealed signalling pathways preferentially regulated in human iPSCs and human ESCs

Wei-Sheng Tien[1,2], Pei-Mien Chen[4], Ching-Yu Chuang[5], Shook-Mun Lui[1], Hung-Chih Kuo[3], Yu-Ju Chen[4] and Kun-Pin Wu[1*]

[1] Institute of Biomedical Informatics, National Yang Ming University, Taipei 112, Taiwan

[2] Bioinformatics Program, Taiwan International Graduate Program, Academia Sinica, Taipei 115, Taiwan

[3] Institute of Cellular and Organismic Biology, Academia Sinica, Taipei 115, Taiwan

[4] Institute of Chemistry, Academia Sinica, Taipei 115, Taiwan

[5] Genomics Research Center, Academia Sinica, Taipei 115, Taiwan

* kpwu@ym.edu.tw

Supplementary Table S1

Identified proteins in the human membrane proteome of hiPSCs, hESCs and somatic foreskin fibroblast HFF.

Table S1 Identified proteins in the human membrane proteome of hiPSCs, hESCs and somatic foreskin fibroblast HFF.
Profiles of identified proteins in MS Experiment 1

Data1. AccessionNo (IPI)	Gene Symbol	Description	Score	Mass	# of identified (spectrum)	# of samples identified	Total peptides	# of peptides used	iPS_CFB46 /HFF
IPI00003004	SLC25A22	SLC25A22 Mitochondrial glutamate carrier 1	38	34448	1	1	1	1	-9999
IPI00005668	AKR1C2	AKR1C2 Aldo-keto reductase family 1 member C2	52	36712	1	1	1	1	-9999
IPI00005705	PPP1CC	PPP1CC Isoform Gamma-1 of Serine/threonine-prot	48	36960	1	1	1	1	-9999
IPI00007068	ACTR3B	ACTR3B Isoform 1 of Actin-related protein 3B	152	47577	3	1	1	1	-9999
IPI00007118	SERPINE1	SERPINE1 Plasminogen activator inhibitor 1	194	45031	4	1	2	1	-9999
IPI00007327	TAPBP	TAPBP Isoform 1 of Tapasin	38	47596	1	1	1	1	-9999
IPI00007960	POSTN	POSTN Isoform 1 of Periostin	92	93255	1	1	1	1	-9999
IPI00008494	ICAM1	ICAM1 Intercellular adhesion molecule 1	117	57789	2	1	1	1	-9999
IPI00008711	WFS1	WFS1 Wolframin	72	100241	1	1	1	1	-9999
IPI00009276	PROCR	PROCR Endothelial protein C receptor precursor	245	30696	5	2	1	1	-9999
IPI00009634	SQRDL	SQRDL Sulfide:quinone oxidoreductase, mitochondri	122	49929	2	1	1	1	-9999
IPI00009885	FKBP11	FKBP11 FK506-binding protein 11	47	22166	2	1	1	1	-9999
IPI00011253	RPS3	RPS3 40S ribosomal protein S3	58	26671	2	2	1	1	-9999
IPI00011302	CD59	CD59 CD59 glycoprotein	84	14168	5	2	1	1	-9999
IPI00012493	RPS20	RPS20 40S ribosomal protein S20	128	13364	3	1	2	1	-9999
IPI00013236	VAMP7	VAMP7 Isoform 2 of Vesicle-associated membrane p	55	30197	1	1	1	1	-9999
IPI00013872	KDELR3	KDELR3 Isoform 1 of ER lumen protein retaining rec	104	25010	4	2	1	1	-9999
IPI00014898	PLEC1	PLEC1 Isoform 1 of Plectin-1	114	531466	2	1	1	1	-9999
IPI00016207	TIMM17A	TIMM17A Mitochondrial import inner membrane tran	124	18012	2	2	1	1	-9999
IPI00018274	EGFR	EGFR Isoform 1 of Epidermal growth factor receptor	324	134190	10	2	4	4	-9999
IPI00020007	LMBRD1	LMBRD1 Isoform 1 of Probable lysosomal cobalamir	61	61349	1	1	1	1	-9999
IPI00021888	SLC39A7	SLC39A7 Zinc transporter SLC39A7	420	50087	5	4	1	1	-9999
IPI00021978	PEX11B	PEX11B Peroxisomal membrane protein 11B	53	28413	1	1	1	1	-9999
IPI00022314	SOD2	SOD2 Superoxide dismutase [Mn], mitochondrial	307	24707	6	2	1	1	-9999
IPI00022465	CIT	CIT Isoform 1 of Citron Rho-interacting kinase	40	231286	1	1	1	1	-9999
IPI00023500	DPAGT1	DPAGT1 Isoform 1 of UDP-N-acetylglucosamine--dc	45	46060	1	1	1	1	-9999
IPI00024466	UGGT1	UGGT1 Isoform 1 of UDP-glucose:glycoprotein gluc	39	177078	1	1	1	1	-9999
IPI00024766	PLXNC1	PLXNC1 Plexin-C1	63	175630	1	1	1	1	-9999
IPI00025084	CAPNS1	CAPNS1 Calpain small subunit 1	143	28298	2	1	1	1	-9999
IPI00025512	HSPB1	HSPB1 Heat shock protein beta-1	393	22768	7	1	3	3	-9999
IPI00026314	GSN	GSN Isoform 1 of Gelsolin	54	85644	1	1	1	1	-9999
IPI00026850	TSPO	TSPO Translocator protein	142	18767	6	2	1	1	-9999
IPI00027681	NNMT	NNMT Nicotinamide N-methyltransferase	72	29NaN	2	1	1	1	-9999
IPI00028082	RECK	RECK Reversion-inducing cysteine-rich protein with I	239	106386	6	1	2	2	-9999
IPI00028338	SELI	SELI Ethanolaminephosphotransferase 1	500	45152	10	4	2	1	-9999
IPI00029099	FAM69A	FAM69A Isoform 1 of Protein FAM69A	60	48992	1	1	1	1	-9999
IPI00030280	LRRC32	LRRC32 Leucine-rich repeat-containing protein 32	52	71934	1	1	1	1	-9999
IPI00030530	TMEM55B	TMEM55B Isoform 1 of Transmembrane protein 55B	60	29450	2	1	1	1	-9999
IPI00101037	RCN3	RCN3 Reticulocalbin-3	173	37470	3	1	1	1	-9999
IPI00151462	MAP1LC3B2	MAP1LC3B2 Microtubule-associated proteins 1A/1B	158	14619	6	1	2	2	-9999
IPI00153050	CALHM2	CALHM2 Isoform 1 of Calcium homeostasis modulat	113	36152	2	1	1	1	-9999
IPI00171438	MUTED;TXNDC5	MUTED;TXNDC5 Thioredoxin domain-containing pro	62	47599	1	1	1	1	-9999
IPI00171444	GPR177	GPR177 Isoform 1 of Integral membrane protein GP	40	62212	1	1	1	1	-9999
IPI00217471	HBE1	HBE1 Hemoglobin subunit epsilon	125	16192	3	1	1	1	-9999
IPI00218086	RDH10	RDH10 Retinol dehydrogenase 10	100	38062	3	1	2	2	-9999

Data1. AccessionNo (IPI)	iPS_CFB50/ HFF	iPS_CFB46/ H9ES	iPS_CFB50/ H9ES	iPS_CFB46/N TU1ES	iPS_CFB50/N TU1ES	H9ES/ HFF	NTU1ES/ HFF
IPI00003004	-9999	NaN	NaN	NaN	NaN	-9999	-9999
IPI00005668	-9999	NaN	NaN	NaN	NaN	-9999	-9999
IPI00005705	-9999	NaN	NaN	NaN	NaN	-9999	-9999
IPI00007068	-9999	-9999	-9999	NaN	NaN	0.235	-9999
IPI00007118	-9999	-9999	-9999	NaN	NaN	0.471	-9999
IPI00007327	-9999	NaN	NaN	NaN	NaN	-9999	-9999
IPI00007960	-9999	NaN	NaN	NaN	NaN	-9999	-9999
IPI00008494	-9999	NaN	NaN	NaN	NaN	-9999	-9999
IPI00008711	-9999	-9999	-9999	NaN	NaN	0.509	-9999
IPI00009276	-9999	NaN	NaN	-9999	-9999	-9999	0.24
IPI00009634	-9999	NaN	NaN	NaN	NaN	-9999	-9999
IPI00009885	-9999	NaN	NaN	NaN	NaN	-9999	-9999
IPI00011253	-9999	-9999	-9999	NaN	NaN	0.499	-9999
IPI00011302	-9999	NaN	NaN	NaN	NaN	-9999	-9999
IPI00012493	-9999	NaN	NaN	NaN	NaN	-9999	-9999
IPI00013236	-9999	NaN	NaN	NaN	NaN	-9999	-9999
IPI00013872	-9999	NaN	NaN	NaN	NaN	-9999	-9999
IPI00014898	-9999	NaN	NaN	NaN	NaN	-9999	-9999
IPI00016207	-9999	NaN	NaN	NaN	NaN	-9999	-9999
IPI00018274	-9999	NaN	NaN	NaN	NaN	-9999	-9999
IPI00020007	-9999	NaN	NaN	NaN	NaN	-9999	-9999
IPI00021888	0.795	-9999	0.829	NaN	9999	0.972	-9999
IPI00021978	-9999	NaN	NaN	NaN	NaN	-9999	-9999
IPI00022314	-9999	NaN	NaN	NaN	NaN	-9999	-9999
IPI00022465	-9999	NaN	NaN	NaN	NaN	-9999	-9999
IPI00023500	1.237	-9999	0.984	-9999	0.928	1.274	1.305
IPI00024466	-9999	-9999	-9999	NaN	NaN	0.265	-9999
IPI00024766	-9999	NaN	NaN	-9999	-9999	-9999	0.151
IPI00025084	-9999	NaN	NaN	-9999	-9999	-9999	0.187
IPI00025512	-9999	-9999	-9999	-9999	-9999	0.158	0.686
IPI00026314	-9999	NaN	NaN	NaN	NaN	-9999	-9999
IPI00026850	-9999	NaN	NaN	NaN	NaN	-9999	-9999
IPI00027681	-9999	NaN	NaN	-9999	-9999	-9999	0.149
IPI00028082	-9999	NaN	NaN	NaN	NaN	-9999	-9999
IPI00028338	-9999	NaN	NaN	-9999	-9999	-9999	3.806
IPI00029099	-9999	NaN	NaN	NaN	NaN	-9999	-9999
IPI00030280	0.475	NaN	9999	-9999	0.821	-9999	0.567
IPI00030530	-9999	NaN	NaN	NaN	NaN	-9999	-9999
IPI00101037	-9999	NaN	NaN	NaN	NaN	-9999	-9999
IPI00151462	-9999	NaN	NaN	NaN	NaN	-9999	-9999
IPI00153050	0.135	-9999	1.156	NaN	9999	0.118	-9999
IPI00171438	0.05	NaN	9999	-9999	0.238	-9999	0.207
IPI00171444	-9999	NaN	NaN	NaN	NaN	-9999	-9999
IPI00217471	-9999	NaN	NaN	NaN	NaN	-9999	-9999
IPI00218086	-9999	NaN	NaN	NaN	NaN	-9999	-9999

Table S1 Identified proteins in the human membrane proteome of hiPSCs, hESCs and somatic foreskin fibroblast HFF.
Profiles of identified proteins in MS Experiment 1

Data1. AccessionNo (IPI)	Gene Symbol	Description	Score	Mass	# of identified (spectrum)	# of samples identified	Total peptides	# of peptides used	iPS_CFB46 /HFF
IPI00218658	ITPR1	ITPR1 Isoform 2 of Inositol 1,4,5-trisphosphate recep	53	311981	1	1	1	1	-9999
IPI00219425	PVR	PVR Isoform Beta of Poliovirus receptor	101	40100	6	2	2	2	-9999
IPI00220007	APOL2	APOL2 cDNA FLJ55764, highly similar to Apolipopro	74	49735	1	1	1	1	-9999
IPI00291136	COL6A1	COL6A1 Collagen alpha-1(VI) chain	524	108462	11	2	4	3	-9999
IPI00293073	MFN1	MFN1 Mitochondrial transmembrane GTPase FZO-2	72	86939	2	2	1	1	-9999
IPI00296441	ADA	ADA Adenosine deaminase	146	40739	3	1	1	1	-9999
IPI00297261	PTPN1	PTPN1 Tyrosine-protein phosphatase non-receptor t	39	49935	1	1	1	1	-9999
IPI00299086	SDCBP	SDCBP Syntenin-1	476	32424	9	2	4	4	-9999
IPI00303882	PLIN3	PLIN3 Isoform B of Mannose-6-phosphate receptor-l	220	47018	3	1	1	1	-9999
IPI00328350	FAM129A	FAM129A Protein Niban	83	103070	2	1	1	1	-9999
IPI00329719	MYO1D	MYO1D Isoform 1 of Myosin-Id	219	116129	3	1	1	1	-9999
IPI00387077	SLC12A9	SLC12A9 Isoform 1 of Solute carrier family 12 meml	133	96049	4	3	1	1	-9999
IPI00414467	COLEC12	COLEC12 Isoform 1 of Collectin-12	72	81475	1	1	1	1	-9999
IPI00439446	MAN1A1	MAN1A1 Mannosidase, alpha, class 1A, member 1,	64	40012	1	1	1	1	-9999
IPI00441344	GLB1	GLB1 Isoform 1 of Beta-galactosidase	67	76027	1	1	1	1	-9999
IPI00465028	RCTPI1;TPI1	RCTPI1;TPI1 triosephosphate isomerase 1 isoform 2	82	30772	2	1	2	2	-9999
IPI00479058	RPS15	RPS15 40S ribosomal protein S15	86	17029	2	2	1	1	-9999
IPI00479217	HNRNPU	HNRNPU Isoform Short of Heterogeneous nuclear ri	276	88924	5	3	1	1	-9999
IPI00513773	PTRF	PTRF Isoform 2 of Polymerase I and transcript relea	189	33342	3	1	1	1	-9999
IPI00743359	HLA-A	HLA-A HLA class I histocompatibility antigen, A-26 a	4239	41036	4	1	2	1	-9999
IPI00746105	HLA-B	HLA-B HLA class I histocompatibility antigen, B-40 a	2945	40480	4	1	1	1	-9999
IPI00783726	KTN1	KTN1 kinectin 1 isoform b	169	150260	4	1	2	2	-9999
IPI00018953	DPP4	DPP4 Dipeptidyl peptidase 4	1044	88222	17	2	5	5	0.034
IPI00329598	HSD17B11	HSD17B11 Estradiol 17-beta-dehydrogenase 11	354	32943	7	3	1	1	0.07
IPI00009890	SERPINE2	SERPINE2 Glia-derived nexin	403	43974	6	2	1	1	0.082
IPI00103530	ATL1	ATL1 Atlastin-1	83	63503	3	1	1	1	0.084
IPI00295461	FAP	FAP Isoform 1 of Seprase	733	87683	18	2	6	6	0.084
IPI00000190	CD81	CD81 CD81 antigen	430	25792	6	1	2	2	0.09
IPI00001592	GPNMB	GPNMB Isoform 2 of Transmembrane glycoprotein N	230	62603	3	1	1	1	0.09
IPI00296147	TMEM119	TMEM119 Transmembrane protein 119	295	29185	6	1	2	2	0.092
IPI00247063	MME	MME Neprilysin	2776	85460	60	1	22	19	0.095
IPI00465113	EXD2	EXD2 cDNA FLJ58573, highly similar to Exonucleas	299	70308	7	3	2	2	0.104
IPI00012503	PSAP	PSAP Isoform Sap-mu-0 of Proactivator polypeptide	243	58074	7	2	1	1	0.106
IPI00009030	LAMP2	LAMP2 Isoform LAMP-2A of Lysosome-associated r	1830	44932	46	8	2	2	0.115
IPI00009111	TPBG	TPBG Trophoblast glycoprotein	952	46003	16	5	2	2	0.125
IPI00221224	ANPEP	ANPEP Aminopeptidase N	6287	109471	114	2	39	29	0.125
IPI00100980	EHD2	EHD2 EH domain-containing protein 2	978	61123	18	1	8	7	0.126
IPI00005707	MRC2	MRC2 C-type mannose receptor 2	1348	166549	32	2	5	5	0.139
IPI00412592	FAM108B1	FAM108B1 Isoform 1 of Abhydrolase domain-contair	108	32194	2	1	1	1	0.139
IPI00025346	PEX14	PEX14 Isoform 1 of Peroxisomal membrane protein	626	41212	16	7	3	2	0.14
IPI00031583	USO1	USO1 Putative uncharacterized protein DKFZp451D	43	109119	1	1	1	1	0.14
IPI00006250	DHRS3	DHRS3 Isoform 1 of Short-chain dehydrogenase/red	112	33527	4	1	2	2	0.141
IPI00012011	CFL1	CFL1 Cofilin-1	133	18491	3	1	2	2	0.141
IPI00219219	LGALS1	LGALS1 Galectin-1	181	14706	2	2	1	1	0.141
IPI00294834	ASPH	ASPH Aspartyl/asparaginyl beta-hydroxylase	1304	85809	23	2	6	6	0.145

Data1. AccessionNo (IPI)	iPS_CFB50/ HFF	iPS_CFB46/ H9ES	iPS_CFB50/ H9ES	iPS_CFB46/N TU1ES	iPS_CFB50/N TU1ES	H9ES/ HFF	NTU1ES/ HFF
IPI00218658	-9999	NaN	NaN	NaN	NaN	-9999	-9999
IPI00219425	-9999	NaN	NaN	NaN	NaN	-9999	-9999
IPI00220007	-9999	NaN	NaN	NaN	NaN	-9999	-9999
IPI00291136	0.026	-9999	0.504	NaN	9999	0.051	-9999
IPI00293073	-9999	NaN	NaN	NaN	NaN	-9999	-9999
IPI00296441	-9999	NaN	NaN	NaN	NaN	-9999	-9999
IPI00297261	-9999	NaN	NaN	NaN	NaN	-9999	-9999
IPI00299086	-9999	-9999	-9999	NaN	NaN	0.045	-9999
IPI00303882	-9999	NaN	NaN	NaN	NaN	-9999	-9999
IPI00328350	0.967	NaN	9999	NaN	9999	-9999	-9999
IPI00329719	-9999	NaN	NaN	NaN	NaN	-9999	-9999
IPI00387077	-9999	-9999	-9999	NaN	NaN	0.154	-9999
IPI00414467	-9999	NaN	NaN	-9999	-9999	-9999	0.443
IPI00439446	-9999	NaN	NaN	NaN	NaN	-9999	-9999
IPI00441344	-9999	NaN	NaN	NaN	NaN	-9999	-9999
IPI00465028	-9999	NaN	NaN	-9999	-9999	-9999	0.37
IPI00479058	1.224	-9999	2.935	-9999	1.19	0.423	1.007
IPI00479217	-9999	-9999	-9999	-9999	-9999	5.12	11.109
IPI00513773	-9999	NaN	NaN	-9999	-9999	-9999	0.062
IPI00743359	-9999	NaN	NaN	NaN	NaN	-9999	-9999
IPI00746105	-9999	NaN	NaN	NaN	NaN	-9999	-9999
IPI00783726	1.026	NaN	9999	-9999	0.752	-9999	1.335
IPI00018953	-9999	NaN	-9999	0.874	-9999	0.136	0.037
IPI00329598	0.122	0.58	1.035	1.213	2.144	0.119	0.056
IPI00009890	0.104	0.569	0.738	9999	9999	0.143	-9999
IPI00103530	0.067	0.161	0.131	0.393	0.318	0.515	0.206
IPI00295461	-9999	NaN	-9999	NaN	-9999	0.42	0.836
IPI00000190	0.11	0.546	0.68	0.427	0.526	0.163	0.204
IPI00001592	-9999	9999	NaN	9999	NaN	-9999	-9999
IPI00296147	-9999	NaN	-9999	0.713	-9999	1.302	0.125
IPI00247063	0.07	1.317	1.032	1.016	0.763	0.061	0.074
IPI00465113	1.275	1.431	16.501	1.508	22.56	0.089	0.067
IPI00012503	-9999	1.169	-9999	3.123	-9999	0.09	0.033
IPI00009030	0.111	1.036	1.157	0.736	0.815	0.083	0.104
IPI00009111	0.287	0.586	1.099	0.754	1.344	0.265	0.207
IPI00221224	0.163	1.328	1.424	0.922	1.034	0.088	0.109
IPI00100980	0.299	0.224	1.152	0.133	0.906	0.223	0.351
IPI00005707	0.147	0.116	0.122	0.054	0.06	0.727	1.589
IPI00412592	0.192	0.51	0.722	1.139	1.596	0.269	0.117
IPI00025346	-9999	0.184	-9999	NaN	-9999	0.663	0.292
IPI00031583	0.179	0.664	0.875	0.739	0.965	0.208	0.182
IPI00006250	0.172	0.752	0.945	0.426	0.53	0.185	0.318
IPI00012011	-9999	9999	NaN	9999	NaN	-9999	-9999
IPI00219219	0.16	1.063	1.235	0.909	1.045	0.131	0.15
IPI00294834	0.116	0.784	1.15	0.724	1.384	0.111	0.18

Table S1 Identified proteins in the human membrane proteome of hiPSCs, hESCs and somatic foreskin fibroblast HFF.
Profiles of identified proteins in MS Experiment 1

Data1. AccessionNo (IPI)	Gene Symbol	Description	Score	Mass	# of identified (spectrum)	# of samples identified	Total peptides	# of peptides used	iPS_CFB46 /HFF
IPI00003411	PTGIS	PTGIS Prostacyclin synthase	5719	57068	118	7	22	16	0.146
IPI00020042	PSMC4	PSMC4 Isoform 1 of 26S protease regulatory subuni	260	47337	6	4	1	1	0.151
IPI00221232	GNG12	GNG12 Guanine nucleotide-binding protein G(I)/G(S	590	8001	14	3	1	1	0.155
IPI00470468	EFR3A	EFR3A Isoform 3 of Protein EFR3 homolog A	601	87524	15	4	2	2	0.156
IPI00216127	PLSCR3	PLSCR3 Phospholipid scramblase 3	178	31642	1	1	1	1	0.157
IPI00217169	ATP2B4	ATP2B4 Isoform XB of Plasma membrane calcium-t	2472	133846	54	2	16	11	0.158
IPI00009236	CAV1	CAV1 Isoform Alpha of Caveolin-1	1736	20458	50	8	8	6	0.16
IPI00007426	ARL6IP5	ARL6IP5 PRA1 family protein 3	3104	21600	35	7	3	2	0.17
IPI00012511	RHOQ	RHOQ Rho-related GTP-binding protein RhoQ	196	22645	3	2	1	1	0.17
IPI00013895	S100A11	S100A11 Protein S100-A11	272	11733	3	1	1	1	0.172
IPI00178352	FLNC	FLNC Isoform 1 of Filamin-C	301	290841	8	3	2	2	0.172
IPI00298994	TLN1	TLN1 Talin-1	809	269599	14	2	6	5	0.172
IPI00028513	ADCY3	ADCY3 ADCY3 protein	102	128974	1	1	1	1	0.174
IPI00151710	ANO6	ANO6 Anoctamin-6	76	106096	1	1	1	1	0.174
IPI00397645	MXRA7	MXRA7 Isoform 2 of Matrix-remodeling-associated p	130	17482	1	1	1	1	0.181
IPI00438289	ERBB2IP	ERBB2IP Isoform 4 of Protein LAP2	109	151159	2	1	2	2	0.182
IPI00944575	HLA-C	HLA-C MHC class I antigen (Fragment)	3726	35390	4	1	2	2	0.191
IPI00072917	COL6A3	COL6A3 alpha 3 type VI collagen isoform 4 precursc	1710	321802	30	1	16	14	0.197
IPI00026994	PRAF2;WDR45	PRAF2;WDR45 PRA1 family protein 2	504	19246	16	4	2	2	0.198
IPI00220993	CNP	CNP Isoform CNPI of 2',3'-cyclic-nucleotide 3'-phosp	41	45070	1	1	1	1	0.203
IPI00294398	HADH	HADH Isoform 1 of Hydroxyacyl-coenzyme A dehydr	120	34256	2	1	1	1	0.203
IPI00004672	HLA-H	HLA-H Putative HLA class I histocompatibility antige	3141	40824	6	2	1	1	0.207
IPI00217766	SCARB2	SCARB2 Lysosome membrane protein 2	3372	54255	68	5	12	6	0.208
IPI00030431	ANTXR1	ANTXR1 Isoform 1 of Anthrax toxin receptor 1	125	62749	2	2	1	1	0.209
IPI00217490	FNDC3B	FNDC3B Isoform 1 of Fibronectin type III domain-coi	864	132803	17	7	2	2	0.209
IPI00221240	LNPEP	LNPEP Isoform 2 of Leucyl-cystinyl aminopeptidase	371	115562	5	2	1	1	0.209
IPI00029468	ACTR1A	ACTR1A Alpha-centractin	183	42587	3	1	1	1	0.21
IPI00478003	A2M	A2M Alpha-2-macroglobulin	571	163175	18	1	3	3	0.211
IPI00011229	CTSD	CTSD Cathepsin D	308	44524	7	2	3	3	0.214
IPI00030702	IDH3A	IDH3A Isoform 1 of Isocitrate dehydrogenase [NAD]	177	39566	3	1	1	1	0.216
IPI00031461	GDI2	GDI2 cDNA FLJ60299, highly similar to Rab GDP di:	102	51121	1	1	1	1	0.221
IPI00013508	ACTN1	ACTN1 Alpha-actinin-1	408	102993	9	2	2	2	0.222
IPI00760554	HLA-A	HLA-A HLA class I histocompatibility antigen, A-69 a	5054	40951	6	2	1	1	0.222
IPI00022892	THY1	THY1 Thy-1 membrane glycoprotein	4222	17923	79	8	5	3	0.224
IPI00335168	MYL6B;MYL6	MYL6B;MYL6 Isoform Non-muscle of Myosin light pc	139	16919	6	2	2	2	0.227
IPI00456750	FAM129B	FAM129B Niban-like protein 1	100	82631	1	1	1	1	0.227
IPI00297160	CD44	CD44 Isoform 12 of CD44 antigen	2257	39391	38	2	6	4	0.23
IPI00550523	ATL3	ATL3 Isoform 1 of Atlastin-3	2552	60503	42	5	7	7	0.231
IPI00465431	LGALS3	LGALS3 Galectin-3	1030	26136	26	2	7	5	0.233
IPI00384280	PCYOX1	PCYOX1 Prenylcysteine oxidase 1	836	56604	22	6	8	8	0.234
IPI00018146	YWHAQ	YWHAQ 14-3-3 protein theta	252	27747	1	1	1	1	0.238
IPI00215998	CD63	CD63 CD63 antigen	1244	25619	39	8	5	2	0.239
IPI00465439	ALDOA	ALDOA Fructose-bisphosphate aldolase A	67	39395	1	1	1	1	0.242
IPI00216592	HNRNPC	HNRNPC Isoform C1 of Heterogeneous nuclear ribo	213	32318	7	2	2	2	0.245
IPI00300744	TMEM14B	TMEM14B Transmembrane protein 14B	99	12070	2	2	1	1	0.245

Data1. AccessionNo (IPI)	iPS_CFB50/ HFF	iPS_CFB46/ H9ES	iPS_CFB50/ H9ES	iPS_CFB46/N TU1ES	iPS_CFB50/N TU1ES	H9ES/ HFF	NTU1ES/ HFF
IPI00003411	0.188	1.011	1.181	0.951	1.034	0.183	0.175
IPI00020042	0.188	0.801	1.022	1.092	1.379	0.187	0.134
IPI00221232	0.086	1.196	0.683	2.047	1.157	0.128	0.073
IPI00470468	0.2	0.777	1.022	9999	9999	0.199	-9999
IPI00216127	0.237	0.72	1.118	0.695	1.068	0.215	0.217
IPI00217169	0.146	1.331	1.09	0.818	0.646	0.126	0.167
IPI00009236	0.118	1.19	0.904	1.285	1.097	0.148	0.081
IPI00007426	0.386	0.928	0.95	0.945	0.439	0.393	0.772
IPI00012511	0.194	9999	9999	0.993	1.154	-9999	0.165
IPI00013895	0.283	0.385	0.651	0.699	1.169	0.441	0.237
IPI00178352	0.15	0.874	0.756	9999	9999	0.198	-9999
IPI00298994	0.257	1.252	1.107	0.653	1.149	0.065	0.391
IPI00028513	0.143	1.391	1.169	1.447	1.205	0.124	0.116
IPI00151710	0.279	0.719	1.185	0.561	0.916	0.239	0.298
IPI00397645	0.295	9999	9999	9999	9999	-9999	-9999
IPI00438289	0.128	0.506	0.27	9999	9999	0.479	-9999
IPI00944575	0.176	1.322	1.251	1.387	1.301	0.143	0.133
IPI00072917	0.199	0.448	0.479	0.814	0.824	0.516	0.203
IPI00026994	0.264	0.753	1.029	0.995	1.347	0.26	0.192
IPI00220993	0.652	0.479	1.577	0.844	2.754	0.419	0.232
IPI00294398	-9999	9999	NaN	9999	NaN	-9999	-9999
IPI00004672	0.153	0.996	0.751	9999	9999	0.206	-9999
IPI00217766	0.202	1.102	1.059	1.105	1.066	0.19	0.183
IPI00030431	-9999	0.676	-9999	1.217	-9999	0.306	0.166
IPI00217490	0.349	0.889	1.541	0.398	0.71	0.231	0.419
IPI00221240	0.25	0.78	0.96	1.187	1.446	0.264	0.169
IPI00029468	0.18	0.755	0.665	9999	9999	0.275	-9999
IPI00478003	0.166	2.011	1.67	1.365	1.077	0.104	0.151
IPI00011229	0.135	1.679	1.546	1.277	1.164	0.126	0.162
IPI00030702	-9999	0.503	-9999	9999	NaN	0.425	-9999
IPI00031461	0.22	0.724	0.742	1.609	1.631	0.301	0.132
IPI00013508	0.222	1.089	1.134	1.528	1.578	0.202	0.139
IPI00760554	0.504	0.812	1.895	1.554	3.595	0.27	0.137
IPI00022892	0.295	0.821	1.114	0.858	1.153	0.271	0.253
IPI00335168	0.094	0.991	0.42	2.237	0.938	0.226	0.098
IPI00456750	-9999	9999	NaN	9999	NaN	-9999	-9999
IPI00297160	0.082	0.576	0.329	0.293	0.14	0.163	1.263
IPI00550523	0.281	1.406	1.35	0.891	0.814	0.235	0.272
IPI00465431	0.191	0.676	0.569	0.856	0.714	0.34	0.262
IPI00384280	0.381	0.772	1.247	0.577	0.924	0.293	0.381
IPI00018146	0.15	1.679	1.08	1.882	1.2	0.14	0.122
IPI00215998	0.257	1.093	1.146	0.861	0.888	0.211	0.271
IPI00465439	0.267	0.873	0.989	0.89	0.998	0.274	0.262
IPI00216592	0.34	0.86	1.224	0.652	0.919	0.281	0.362
IPI00300744	0.649	0.54	1.467	0.547	1.47	0.448	0.432

Table S1 Identified proteins in the human membrane proteome of hiPSCs, hESCs and somatic foreskin fibroblast HFF.
Profiles of identified proteins in MS Experiment 1

Data1. AccessionNo (IPI)	Gene Symbol	Description	Score	Mass	# of identified (spectrum)	# of samples identified	Total peptides	# of peptides used	iPS_CFB46 /HFF
IPI00065510	TMEM68	TMEM68 Isoform 2 of Transmembrane protein 68	55	29522	2	1	1	1	0.246
IPI00006205	SLC33A1	SLC33A1 Acetyl-coenzyme A transporter 1	511	60870	13	7	1	1	0.25
IPI00385495	LMF2	LMF2 Isoform 1 of Lipase maturation factor 2	1002	79647	17	5	4	4	0.253
IPI00328715	MTDH	MTDH Protein LYRIC	119	63799	4	1	2	2	0.254
IPI00021048	MYOF	MYOF Isoform 1 of Myoferlin	4261	234561	108	3	35	31	0.255
IPI00032140	SERPINH1	SERPINH1 Serpin H1	7924	46411	155	8	20	16	0.255
IPI00297646	COL1A1	COL1A1 Collagen alpha-1(I) chain	949	138827	30	2	6	6	0.255
IPI00020124	PI4K2A	PI4K2A Phosphatidylinositol 4-kinase type 2-alpha	101	53989	3	1	2	2	0.256
IPI00030363	ACAT1	ACAT1 Acetyl-CoA acetyltransferase, mitochondrial	215	45171	2	1	1	1	0.256
IPI00009456	NT5E	NT5E 5'-nucleotidase	4224	63327	69	2	16	12	0.258
IPI00010491	RAB27B	RAB27B Ras-related protein Rab-27B	119	24592	5	1	2	2	0.258
IPI00095891	GNAS	GNAS Isoform XLas-1 of Guanine nucleotide-binding	1238	110956	10	3	3	2	0.26
IPI00246058	PDCD6IP	PDCD6IP Programmed cell death 6-interacting prote	260	95963	4	1	3	3	0.266
IPI00011454	GANAB	GANAB Isoform 2 of Neutral alpha-glucosidase AB	531	109369	14	4	7	6	0.267
IPI00022418	FN1	FN1 Isoform 1 of Fibronectin	790	262442	14	1	6	6	0.269
IPI00018248	KDELR2	KDELR2 Isoform 1 of ER lumen protein retaining rec	1352	24406	29	6	5	4	0.27
IPI00156689	VAT1	VAT1 Synaptic vesicle membrane protein VAT-1 hor	1013	41893	14	2	4	4	0.27
IPI00749429	TTYH3	TTYH3 Isoform 1 of Protein tweety homolog 3	795	57508	12	6	1	1	0.277
IPI00045921	ATAD3B	ATAD3B TOB3	2945	65075	15	6	1	1	0.28
IPI00027497	GPI	GPI Glucose-6-phosphate isomerase	497	63107	10	3	2	2	0.285
IPI00305551	GNA11	GNA11 Guanine nucleotide-binding protein subunit a	1826	42097	34	8	5	4	0.29
IPI00654777	EIF3F	EIF3F HCG1784554, isoform CRA_a	207	39122	5	4	1	1	0.291
IPI00793199	ANXA4	ANXA4 annexin IV	275	36062	5	2	2	2	0.293
IPI00018871	ARL8B	ARL8B cDNA FLJ56285, highly similar to ADP-ribosy	2425	27235	51	6	8	7	0.294
IPI00217143	SDHA	SDHA 57 kDa protein	101	56716	1	1	1	1	0.298
IPI00180675	TUBA1A	TUBA1A Tubulin alpha-1A chain	13126	50104	22	8	1	1	0.302
IPI00219018	GAPDH	GAPDH Glyceraldehyde-3-phosphate dehydrogenas	5242	36030	98	8	12	7	0.308
IPI00843975	EZR	EZR Ezrin	131	69370	5	2	2	2	0.309
IPI00219682	STOM	STOM Erythrocyte band 7 integral membrane proteir	5295	31711	117	8	15	11	0.31
IPI00329600	SCCPDH	SCCPDH Probable saccharopine dehydrogenase	1272	47121	22	8	2	1	0.313
IPI00217683	AKAP12	AKAP12 A kinase (PRKA) anchor protein 12 isoform	196	181580	3	1	2	2	0.315
IPI00011284	COMT	COMT Isoform Membrane-bound of Catechol O-metl	439	30018	7	2	2	2	0.317
IPI00876963	HLA-A	HLA-A HLA class I histocompatibility antigen, A-2 alp	5270	40896	130	8	15	9	0.317
IPI00002478	ECE1	ECE1 Isoform B of Endothelin-converting enzyme 1	1191	87108	21	2	8	5	0.321
IPI00374076	FITM2	FITM2 Fat storage-inducing transmembrane protein	48	29835	1	1	1	1	0.322
IPI00025252	PDIA3	PDIA3 Protein disulfide-isomerase A3	1258	56747	35	6	8	7	0.323
IPI00022018	DPM1	DPM1 Dolichol-phosphate mannosyltransferase	176	29616	5	4	2	2	0.327
IPI00015972	COX6C	COX6C Cytochrome c oxidase subunit 6C	503	8776	22	5	4	3	0.328
IPI00446235	CYB5R3	CYB5R3 Isoform 2 of NADH-cytochrome b5 reducta:	6077	31608	129	8	13	9	0.329
IPI00002307	NLGN3	NLGN3 Isoform 1 of Neuroligin-3	70	93836	1	1	1	1	0.333
IPI00017344	RAB5B	RAB5B Ras-related protein Rab-5B	1140	23692	5	2	1	1	0.333
IPI00793874	SFXN3	SFXN3 sideroflexin 3	736	35956	6	2	3	3	0.337
IPI00001639	KPNB1	KPNB1 Importin subunit beta-1	458	97108	10	3	3	3	0.34
IPI00295851	COPB1	COPB1 Coatomer subunit beta	162	107074	3	2	1	1	0.34
IPI00010418	MYO1C	MYO1C Isoform 2 of Myosin-Ic	1326	117876	31	3	12	12	0.341

Data1. AccessionNo (IPI)	iPS_CFB50/ HFF	iPS_CFB46/ H9ES	iPS_CFB50/ H9ES	iPS_CFB46/N TU1ES	iPS_CFB50/N TU1ES	H9ES/ HFF	NTU1ES/ HFF
IPI00065510	0.319	0.452	0.602	0.732	0.965	0.538	0.324
IPI00006205	0.568	0.263	0.613	0.338	0.779	0.938	0.714
IPI00385495	0.311	1.11	1.314	0.952	1.194	0.234	0.25
IPI00328715	0.285	1.529	1.758	0.491	0.559	0.164	0.499
IPI00021048	0.542	1.22	1.33	0.963	0.985	0.247	0.734
IPI00032140	0.271	1.009	1.145	0.815	0.917	0.256	0.301
IPI00297646	0.439	1.65	2.915	0.769	1.346	0.165	0.369
IPI00020124	0.295	0.87	1.256	0.557	0.804	0.292	0.359
IPI00030363	0.257	0.572	0.588	9999	9999	0.442	-9999
IPI00009456	0.508	0.713	0.98	0.619	0.707	0.528	0.446
IPI00010491	0.377	0.709	1.064	0.416	0.618	0.359	0.597
IPI00095891	0.147	0.636	0.561	1.163	1.689	0.423	0.084
IPI00246058	1.066	1.108	0.908	9999	9999	0.237	-9999
IPI00011454	0.57	0.832	1.406	0.711	1.213	0.386	0.376
IPI00022418	0.505	0.722	1.734	NaN	3.74	0.368	0.113
IPI00018248	0.351	0.639	0.799	1.349	1.499	0.461	0.213
IPI00156689	0.251	1.013	0.968	0.923	0.873	0.259	0.28
IPI00749429	0.153	1.101	0.623	1.127	0.631	0.248	0.237
IPI00045921	0.238	0.347	0.302	0.827	0.714	0.799	0.327
IPI00027497	0.25	3.071	0.593	1.24	0.218	0.236	0.189
IPI00305551	0.335	0.48	0.562	0.864	1.019	0.599	0.326
IPI00654777	0.441	0.251	0.39	0.776	1.194	1.147	0.362
IPI00793199	0.153	1.421	0.816	2.008	1.142	0.19	0.131
IPI00018871	0.204	1.03	0.757	1.311	0.96	0.292	0.21
IPI00217143	0.313	0.894	0.962	1.276	1.359	0.329	0.225
IPI00180675	0.353	0.859	1.029	0.581	0.69	0.348	0.501
IPI00219018	0.197	1.346	0.906	0.852	0.477	0.236	0.337
IPI00843975	0.221	0.279	0.22	1.208	0.896	0.894	0.248
IPI00219682	0.281	1.388	1.429	0.666	0.683	0.187	0.428
IPI00329600	0.406	0.637	0.849	0.457	0.602	0.484	0.631
IPI00217683	1.02	1.005	3.341	0.319	1.049	0.309	0.952
IPI00011284	0.146	1.924	0.758	4.298	1.676	0.216	0.094
IPI00876963	0.316	1.107	1.082	1.334	1.325	0.285	0.23
IPI00002478	0.283	1.075	1.089	1.286	1.21	0.283	0.251
IPI00374076	0.321	0.106	0.108	1.173	1.187	3.014	0.265
IPI00025252	0.343	0.9	1.007	1.087	1.069	0.354	0.195
IPI00022018	0.765	0.392	0.975	0.529	1.26	0.796	0.6
IPI00015972	0.417	1.724	2.209	0.956	1.413	0.134	0.224
IPI00446235	0.37	1.089	1.239	0.743	0.827	0.308	0.422
IPI00002307	0.358	0.727	0.803	0.697	0.762	0.452	0.46
IPI00017344	0.204	1.128	0.711	1.691	1.054	0.291	0.19
IPI00793874	0.181	9999	9999	2.068	1.13	-9999	0.157
IPI00001639	0.217	0.93	0.665	1.383	0.986	0.366	0.225
IPI00295851	0.254	0.986	0.755	1.107	0.84	0.34	0.296
IPI00010418	0.332	0.723	0.581	0.929	0.742	0.502	0.462

Table S1 Identified proteins in the human membrane proteome of hiPSCs, hESCs and somatic foreskin fibroblast HFF.
Profiles of identified proteins in MS Experiment 1

Data1. AccessionNo (IPI)	Gene Symbol	Description	Score	Mass	# of identified (spectrum)	# of samples identified	Total peptides	# of peptides used	iPS_CFB46 /HFF
IPI00064193	TMX3	TMX3 Isoform 1 of Protein disulfide-isomerase TMX3	617	51839	9	2	3	2	0.341
IPI00002459	ANXA6	ANXA6 annexin VI isoform 2	1823	75229	41	6	9	8	0.342
IPI00410488	CD276	CD276 Isoform 1 of CD276 antigen	290	57199	5	2	3	3	0.344
IPI00418471	VIM	VIM Vimentin	5034	53619	123	3	25	21	0.344
IPI00329801	ANXA5	ANXA5 Annexin A5	6104	35914	123	8	8	8	0.346
IPI00941296	CTSB	CTSB cDNA FLJ40065 fis, clone TESOP2000400, h	289	30153	4	2	2	2	0.35
IPI00306604	ITGA5	ITGA5 Integrin alpha-5	831	114465	20	2	8	8	0.351
IPI00024067	CLTC	CLTC Isoform 1 of Clathrin heavy chain 1	974	191493	25	8	3	3	0.357
IPI00021453	PPAP2B	PPAP2B Lipid phosphate phosphohydrolase 3	145	35093	5	1	3	3	0.362
IPI00304962	COL1A2	COL1A2 Collagen alpha-2(I) chain	333	129209	7	1	3	3	0.375
IPI00015148	RAP1B	RAP1B Ras-related protein Rap-1b	1582	20812	42	8	4	4	0.381
IPI00301280	TMEM43	TMEM43 Transmembrane protein 43	2225	44847	53	7	9	8	0.382
IPI00021263	YWHAZ	YWHAZ 14-3-3 protein zeta/delta	343	27728	18	7	2	2	0.386
IPI00100160	CAND1	CAND1 Isoform 1 of Cullin-associated NEDD8-disso	231	136289	4	4	1	1	0.388
IPI00479186	PKM2	PKM2 Isoform M2 of Pyruvate kinase isozymes M1/M	2388	57900	51	6	12	10	0.393
IPI00027626	CCT6A	CCT6A T-complex protein 1 subunit zeta	854	57988	16	8	1	1	0.395
IPI00215893	HMOX1	HMOX1 Heme oxygenase 1	587	32798	11	2	5	5	0.396
IPI00395488	VASN	VASN Vasorin	45	71668	1	1	1	1	0.396
IPI00171903	HNRNPM	HNRNPM Isoform 1 of Heterogeneous nuclear ribonu	41	77464	1	1	1	1	0.397
IPI00419585	PPIA	PPIA Peptidyl-prolyl cis-trans isomerase A	128	18001	5	2	2	2	0.397
IPI00018364	RAP2B	RAP2B Ras-related protein Rap-2b	717	20491	18	7	1	1	0.399
IPI00021057	SLC12A4	SLC12A4 Isoform 1 of Solute carrier family 12 memb	38	120572	1	1	1	1	0.399
IPI00006865	SEC22B	SEC22B Vesicle-trafficking protein SEC22b	3527	24725	65	8	8	7	0.4
IPI00215997	CD9	CD9 CD9 antigen	723	25399	21	8	1	1	0.402
IPI00291175	VCL	VCL Isoform 1 of Vinculin	520	116649	11	5	2	2	0.403
IPI00418169	ANXA2	ANXA2 Isoform 2 of Annexin A2	5170	40386	121	8	12	11	0.404
IPI00168813	PTK7	PTK7 Isoform 3 of Tyrosine-protein kinase-like 7	263	103515	5	3	2	2	0.407
IPI00296099	THBS1	THBS1 Thrombospondin-1	230	129300	6	2	3	3	0.408
IPI00299571	PDIA6	PDIA6 Isoform 2 of Protein disulfide-isomerase A6	1571	53867	28	8	6	5	0.418
IPI00783097	GARS	GARS Glycyl-tRNA synthetase	118	83087	3	2	1	1	0.419
IPI00853612	BCL2L13	BCL2L13 cDNA FLJ31686 fis, clone NT2RI2005382,	88	34579	3	1	1	1	0.422
IPI00470674	CYB5R1	CYB5R1 NADH-cytochrome b5 reductase 1	340	34073	7	2	2	2	0.425
IPI00797136	IKIP	IKIP Isoform 1 of Inhibitor of nuclear factor kappa-B I	1363	39285	25	2	7	7	0.425
IPI00011578	NPTN	NPTN Isoform 1 of Neuroplastin	420	31272	11	6	1	1	0.426
IPI00102685	MYADM	MYADM Myeloid-associated differentiation marker	981	35250	14	4	3	3	0.426
IPI00036552	ANTXR2	ANTXR2 Isoform 2 of Anthrax toxin receptor 2	64	42892	1	1	1	1	0.43
IPI00217966	LDHA	LDHA L-lactate dehydrogenase	1189	39812	23	2	6	6	0.431
IPI00022048	PTGFRN	PTGFRN Prostaglandin F2 receptor negative regulat	207	98495	4	1	2	2	0.432
IPI00020850	PPP2R2B	PPP2R2B Isoform 1 of Serine/threonine-protein phos	85	51678	1	1	1	1	0.438
IPI00031697	TMEM109	TMEM109 Transmembrane protein 109	747	26194	23	8	1	1	0.441
IPI00296157	RETSAT	RETSAT Isoform 1 of All-trans-retinol 13,14-reductas	326	66777	10	3	2	2	0.444
IPI00289876	STX7	STX7 Isoform 1 of Syntaxin-7	264	29797	4	2	2	2	0.446
IPI00219111	TRAM1	TRAM1 Translocating chain-associated membrane p	569	43044	10	5	2	2	0.448
IPI00005024	MYBBP1A	MYBBP1A Isoform 1 of Myb-binding protein 1A	187	148762	3	2	1	1	0.453
IPI00141318	CKAP4	CKAP4 Isoform 1 of Cytoskeleton-associated protein	25449	65983	477	8	32	24	0.453

Data1. AccessionNo (IPI)	iPS_CFB50/ HFF	iPS_CFB46/ H9ES	iPS_CFB50/ H9ES	iPS_CFB46/N TU1ES	iPS_CFB50/N TU1ES	H9ES/ HFF	NTU1ES/ HFF
IPI00064193	0.356	0.829	0.983	0.742	0.888	0.354	0.443
IPI00002459	0.384	0.644	0.765	1.004	1.255	0.543	0.291
IPI00410488	0.365	1.797	1.925	0.938	1.003	0.271	0.375
IPI00418471	0.406	1.289	1.265	0.723	0.757	0.256	0.345
IPI00329801	0.396	0.642	0.775	0.812	1.002	0.55	0.42
IPI00941296	0.614	2.818	5.056	1.315	2.336	0.174	0.365
IPI00306604	0.191	0.585	0.372	1.499	1.092	0.445	0.178
IPI00024067	0.366	0.856	0.909	1.249	1.316	0.417	0.264
IPI00021453	0.344	1.074	1.047	0.406	0.392	0.333	0.86
IPI00304962	0.319	1.177	1.026	0.905	0.781	0.315	0.375
IPI00015148	0.316	1.318	1.138	1.172	1.002	0.285	0.314
IPI00301280	0.606	1.089	1.505	0.764	1.224	0.402	0.478
IPI00021263	0.243	0.983	0.876	0.996	0.878	0.281	0.271
IPI00100160	0.425	0.664	0.745	1.097	1.221	0.577	0.341
IPI00479186	0.362	0.832	0.763	1.011	0.893	0.27	0.343
IPI00027626	0.513	0.351	0.468	0.782	1.031	1.111	0.487
IPI00215893	0.523	1.396	1.181	1.717	1.372	0.281	0.195
IPI00395488	0.268	0.528	0.366	1.166	0.8	0.741	0.327
IPI00171903	0.345	1.283	1.142	1.312	1.156	0.306	0.292
IPI00419585	0.375	1.236	1.213	1.26	0.987	0.316	0.286
IPI00018364	0.374	0.951	0.914	0.772	0.735	0.414	0.498
IPI00021057	-9999	9999	NaN	9999	NaN	-9999	-9999
IPI00006865	0.32	1.103	0.944	1.247	1.118	0.386	0.388
IPI00215997	0.619	0.605	0.955	0.21	0.329	0.657	1.845
IPI00291175	0.202	1.053	0.542	3.492	1.78	0.378	0.111
IPI00418169	0.271	1.035	0.79	1.121	0.861	0.357	0.297
IPI00168813	1.035	1.078	1.869	0.643	0.98	0.56	1.031
IPI00296099	0.315	1.403	1.286	1.198	1.174	0.544	0.337
IPI00299571	0.486	0.853	0.961	1.03	1.167	0.409	0.313
IPI00783097	0.485	1.052	1.25	1.11	1.306	0.394	0.364
IPI00853612	-9999	1.827	-9999	9999	NaN	0.228	-9999
IPI00470674	0.225	1.156	0.629	1.274	0.686	0.363	0.322
IPI00797136	0.32	0.61	0.808	1.436	1.884	0.233	0.097
IPI00011578	0.571	9999	9999	0.848	1.153	-9999	0.484
IPI00102685	0.356	0.602	0.547	1.026	0.919	0.701	0.376
IPI00036552	1.203	0.503	1.443	0.603	1.711	0.845	0.688
IPI00217966	0.33	0.869	0.717	0.914	0.745	0.499	0.397
IPI00022048	0.558	1.117	1.48	1.01	1.325	0.382	0.412
IPI00020850	0.288	9999	9999	9999	9999	-9999	-9999
IPI00031697	0.632	0.958	1.408	0.59	0.858	0.455	0.722
IPI00296157	0.38	1.636	1.428	1.224	1.056	0.269	0.35
IPI00289876	0.117	1.382	0.344	2.636	1.692	0.321	0.12
IPI00219111	0.583	1.051	1.406	0.763	1.01	0.421	0.566
IPI00005024	0.762	0.437	0.754	0.378	0.646	1.025	1.156
IPI00141318	0.518	1.096	1.313	0.917	1.076	0.406	0.481

Table S1 Identified proteins in the human membrane proteome of hiPSCs, hESCs and somatic foreskin fibroblast HFF.
Profiles of identified proteins in MS Experiment 1

Data1. AccessionNo (IPI)	Gene Symbol	Description	Score	Mass	# of identified (spectrum)	# of samples identified	Total peptides	# of peptides used	iPS_CFB46 /HFF
IPI00455165	PANX1	PANX1 Isoform 1 of Pannexin-1	183	48028	4	2	1	1	0.456
IPI00013623	SLC27A3	SLC27A3 Isoform 1 of Long-chain fatty acid transpor	760	87269	13	6	1	1	0.458
IPI00025416	ACTG2	ACTG2 Actin, gamma-enteric smooth muscle	3021	41850	3	1	1	1	0.46
IPI00031522	HADHA	HADHA Trifunctional enzyme subunit alpha, mitocho	383	82947	7	3	2	2	0.466
IPI00288947	GNAQ	GNAQ Guanine nucleotide-binding protein G(q) subu	1546	42115	4	2	1	1	0.466
IPI00006957	DHRS7	DHRS7 Isoform 1 of Dehydrogenase/reductase SDR	189	38274	4	1	3	3	0.468
IPI00013897	ADAM10	ADAM10 Disintegrin and metalloproteinase domain-c	99	84088	2	1	1	1	0.468
IPI00329332	STX12	STX12 Syntaxin-12	687	31622	15	2	3	3	0.47
IPI00299719	TCIRG1	TCIRG1 Isoform Long of V-type proton ATPase 116	1245	92908	16	2	5	4	0.473
IPI00019502	MYH9	MYH9 Isoform 1 of Myosin-9	3382	226392	64	2	15	15	0.475
IPI00000643	BAG2	BAG2 BAG family molecular chaperone regulator 2	332	23757	7	2	2	2	0.476
IPI00333016	DNAJC11	DNAJC11 Isoform 3 of DnaJ homolog subfamily C m	1073	57161	25	6	4	4	0.477
IPI00302927	CCT4;ILK-2	CCT4;ILK-2 T-complex protein 1 subunit delta	94	57888	1	1	1	1	0.478
IPI00549343	VAMP3	VAMP3 Vesicle-associated membrane protein 3	1395	11302	22	6	2	2	0.479
IPI00215914	ARF1	ARF1 ADP-ribosylation factor 1	358	20684	9	1	3	3	0.48
IPI00016670	C11orf59	C11orf59 UPF0404 protein C11orf59	1170	17734	25	4	6	6	0.487
IPI00030634	GGT7	GGT7 Isoform 1 of Gamma-glutamyltransferase 7	55	70423	1	1	1	1	0.491
IPI00020599	CALR	CALR Calreticulin	453	48112	13	1	6	6	0.493
IPI00646625	TAP1	TAP1 Antigen peptide transporter 1	576	87163	17	7	1	1	0.498
IPI00217563	ITGB1	ITGB1 Isoform Beta-1A of Integrin beta-1	5898	88357	145	8	15	12	0.502
IPI00218398	MMP14	MMP14 Matrix metalloproteinase-14	1297	65842	33	4	8	8	0.507
IPI00185146	IPO9	IPO9 Importin-9	495	115889	5	2	1	1	0.508
IPI00386199	C19orf6	C19orf6 Isoform 1 of Membralin	258	67845	5	4	1	1	0.509
IPI00023958	MPV17	MPV17 Protein Mpv17	590	19720	5	4	1	1	0.51
IPI00020418	RRAS	RRAS Ras-related protein R-Ras	1269	23466	26	3	4	4	0.517
IPI00107357	CLPTM1	CLPTM1 Isoform 2 of Cleft lip and palate transmemb	177	77909	4	2	1	1	0.522
IPI00010438	SNAP23	SNAP23 Isoform SNAP-23a of Synaptosomal-assoc	1129	23340	17	6	2	2	0.526
IPI00020557	LRP1	LRP1 Prolow-density lipoprotein receptor-related pro	2316	504245	54	7	15	14	0.526
IPI00794223	COPG	COPG 13 kDa protein	40	13443	1	1	1	1	0.528
IPI00016786	CDC42	CDC42 Isoform 2 of Cell division control protein 42 h	814	21245	24	7	3	2	0.53
IPI00165651	ERGIC2	ERGIC2 Cd002 protein	216	43467	3	1	1	1	0.53
IPI00007165	DOLK	DOLK Dolichol kinase	206	59229	7	4	3	3	0.531
IPI00021428	ACTA1	ACTA1 Actin, alpha skeletal muscle	3709	42024	28	8	3	2	0.532
IPI00003635	ERGIC1	ERGIC1 cDNA FLJ56280, highly similar to Endoplas	448	26242	9	1	3	3	0.534
IPI00029744	SSBP1	SSBP1 Single-stranded DNA-binding protein, mitoch	44	17249	1	1	1	1	0.536
IPI00014232	ARL6IP1	ARL6IP1 ADP-ribosylation factor-like protein 6-intera	1072	23347	15	7	2	1	0.537
IPI00386427	SMPDL3B	SMPDL3B Isoform 2 of Acid sphingomyelinase-like p	1287	41663	36	5	6	6	0.538
IPI00478231	RHOA	RHOA Transforming protein RhoA	176	21754	2	1	1	1	0.538
IPI00011200	PHGDH	PHGDH D-3-phosphoglycerate dehydrogenase	444	56614	11	4	2	1	0.544
IPI00027438	FLOT1	FLOT1 Flotillin-1	1143	47326	31	4	8	8	0.544
IPI00930070	HLA-C	HLA-C MHC class I antigen (Fragment)	4060	31659	48	8	4	3	0.548
IPI00031691	RPL9	RPL9 60S ribosomal protein L9	172	21850	2	2	1	1	0.551
IPI00014377	RAB32	RAB32 Ras-related protein Rab-32	456	24982	18	2	5	5	0.556
IPI00017567	ENG	ENG Isoform Long of Endoglin	623	70533	7	2	2	2	0.556
IPI00295542	NUCB1	NUCB1 Nucleobindin-1	410	53846	9	3	2	2	0.556

Data1. AccessionNo (IPI)	iPS_CFB50/ HFF	iPS_CFB46/ H9ES	iPS_CFB50/ H9ES	iPS_CFB46/N TU1ES	iPS_CFB50/N TU1ES	H9ES/ HFF	NTU1ES/ HFF
IPI00455165	0.298	0.857	0.575	2.479	1.646	0.525	0.177
IPI00013623	0.54	1.231	1.488	1.082	1.296	0.367	0.408
IPI00025416	0.775	0.619	1.069	9999	9999	0.735	-9999
IPI00031522	0.318	1.007	0.701	1.531	1.06	0.456	0.294
IPI00288947	0.433	0.901	0.858	0.931	0.878	0.511	0.483
IPI00006957	0.554	0.653	0.799	1.546	1.07	0.705	0.509
IPI00013897	0.67	0.72	1.056	0.686	0.997	0.642	0.658
IPI00329332	0.28	1.076	0.844	2.655	1.653	0.345	0.213
IPI00299719	0.821	1.166	1.247	1.013	1.072	0.668	0.75
IPI00019502	0.451	1.29	1.126	1.051	0.833	0.476	0.392
IPI00000643	0.244	9999	9999	1.221	1.135	-9999	0.21
IPI00333016	0.621	0.758	0.864	0.583	0.679	0.966	0.951
IPI00302927	0.775	0.441	0.734	9999	9999	1.071	-9999
IPI00549343	0.345	1.169	0.865	2.734	1.984	0.415	0.179
IPI00215914	0.3	1.506	1.007	1.717	0.894	0.298	0.162
IPI00016670	0.265	1.774	1.02	1.163	0.924	0.316	0.646
IPI00030634	1.112	0.369	0.858	0.896	2.061	1.313	0.528
IPI00020599	0.657	0.886	1.174	0.744	0.981	0.538	0.604
IPI00646625	0.458	0.866	0.816	1.032	0.964	0.569	0.465
IPI00217563	0.611	1.022	1.346	0.843	1.02	0.469	0.532
IPI00218398	0.507	0.45	0.713	0.37	0.523	1.128	0.769
IPI00185146	0.455	0.508	0.467	0.991	0.901	0.987	0.494
IPI00386199	0.611	0.527	0.65	1.407	1.718	0.952	0.348
IPI00023958	0.436	0.979	0.859	1.086	0.944	0.514	0.452
IPI00020418	0.336	1.699	1.133	1.561	1.031	0.3	0.319
IPI00107357	1.191	0.141	0.331	0.546	1.265	3.647	0.922
IPI00010438	0.662	1.437	0.803	1.608	0.707	0.362	0.922
IPI00020557	0.607	1.296	1.317	1.357	1.022	0.482	0.493
IPI00794223	0.NaN	0.803	0.865	1.75	1.866	0.65	0.291
IPI00016786	0.373	1.405	1.015	1.249	0.892	0.367	0.397
IPI00165651	0.549	0.958	1.018	0.636	0.669	0.546	0.803
IPI00007165	1.02	0.722	1.419	0.86	1.683	0.733	0.603
IPI00021428	0.485	0.998	0.943	0.718	0.672	0.544	0.708
IPI00003635	0.771	0.944	1.4	0.846	1.242	0.558	0.606
IPI00029744	0.406	0.844	0.656	1.758	1.354	0.627	0.294
IPI00014232	1.023	0.105	0.206	0.76	1.475	5.036	0.675
IPI00386427	0.489	1.099	0.882	0.7	0.582	0.625	0.705
IPI00478231	0.314	0.918	0.55	2.445	1.45	0.579	0.212
IPI00011200	0.385	1.229	0.892	1.01	0.725	0.437	0.518
IPI00027438	0.661	0.917	1.08	0.806	0.999	0.553	0.605
IPI00930070	0.744	1.051	1.29	0.708	0.831	0.598	2.705
IPI00031691	0.388	1.093	0.79	0.888	0.636	0.498	0.598
IPI00014377	0.392	1.24	1.136	1.066	0.709	0.341	0.488
IPI00017567	-9999	9999	NaN	9999	NaN	-9999	-9999
IPI00295542	0.283	0.961	0.499	0.175	0.082	0.568	2.712

Table S1 Identified proteins in the human membrane proteome of hiPSCs, hESCs and somatic foreskin fibroblast HFF.
Profiles of identified proteins in MS Experiment 1

Data1. AccessionNo (IPI)	Gene Symbol	Description	Score	Mass	# of identified (spectrum)	# of samples identified	Total peptides	# of peptides used	iPS_CFB46 /HFF
IPI00002230	NCEH1	NCEH1 arylacetamide deacetylase-like 1 isoform b	713	49031	16	1	7	7	0.559
IPI00884105	LAMP1	LAMP1 Lysosome-associated membrane glycoprote	3407	44854	70	8	4	4	0.568
IPI00007940	ERLIN1	ERLIN1 ER lipid raft associated 1	3564	39146	33	8	2	2	0.572
IPI00409635	ESYT2	ESYT2 Isoform 2 of Extended synaptotagmin-2	322	98840	7	5	1	1	0.572
IPI00021439	ACTB	ACTB Actin, cytoplasmic 1	9133	41710	234	8	19	12	0.577
IPI00011416	ECH1	ECH1 Delta(3,5)-Delta(2,4)-dienoyl-CoA isomerase,	40	35793	1	1	1	1	0.578
IPI00465248	ENO1	ENO1 Isoform alpha-enolase of Alpha-enolase	541	47139	17	4	5	5	0.58
IPI00300562	RAB3B	RAB3B Ras-related protein Rab-3B	3541	24742	18	6	2	2	0.581
IPI00021267	EPHA2	EPHA2 Ephrin type-A receptor 2	128	108197	5	1	2	2	0.587
IPI00043598	IKIP	IKIP Isoform 4 of Inhibitor of nuclear factor kappa-B I	914	43057	15	2	5	5	0.587
IPI00657706	MBOAT7	MBOAT7 Isoform 1 of Lysophospholipid acyltransfera	2243	52730	44	8	7	6	0.587
IPI00008274	CAP1	CAP1 Isoform 1 of Adenylyl cyclase-associated prote	294	51823	5	2	2	2	0.589
IPI00647457	HLA-A	HLA-A Major histocompatibility complex, class I, A	4244	41342	41	6	5	2	0.59
IPI00015102	ALCAM	ALCAM Isoform 1 of CD166 antigen	156	65061	6	2	3	3	0.591
IPI00005737	SURF4	SURF4 Isoform 1 of Surfeit locus protein 4	7932	30374	109	8	6	4	0.593
IPI00410079	FAM82A2	FAM82A2 Isoform 1 of Regulator of microtubule dyna	772	52086	12	5	1	1	0.594
IPI00012486	SPNS1	SPNS1 61 kDa protein	282	61067	5	2	3	3	0.596
IPI00023768	HAS1	HAS1 HyaluroNaN synthase 1	107	64790	2	1	1	1	0.598
IPI00027230	HSP90B1	HSP90B1 Endoplasmin	2178	92411	26	4	8	7	0.603
IPI00170934	STK11IP	STK11IP Isoform 1 of Serine/threonine kinase 11-inte	54	121385	1	1	1	1	0.604
IPI00002149	SAR1B	SAR1B GTP-binding protein SAR1b	48	22396	2	1	1	1	0.606
IPI00030820	MRPL47	MRPL47 Isoform 1 of 39S ribosomal protein L47, mit	46	29432	2	1	1	1	0.608
IPI00003348	GNB2	GNB2 Guanine nucleotide-binding protein G(I)/G(S)/(	558	37307	14	7	1	1	0.609
IPI00010218	CYP20A1	CYP20A1 Cytochrome P450 monooxygenase	43	49502	1	1	1	1	0.609
IPI00022774	VCP	VCP Transitional endoplasmic reticulum ATPase	1002	89266	18	8	1	1	0.609
IPI00186290	EEF2	EEF2 Elongation factor 2	722	95277	20	6	4	4	0.609
IPI00922108	ITGAV	ITGAV integrin alpha-V isoform 2	2218	111075	58	5	16	15	0.615
IPI00166891	TMEM135	TMEM135 TMEM135 protein	376	17683	10	5	1	1	0.619
IPI00005181	PLSCR1	PLSCR1 Phospholipid scramblase 1	321	35026	4	3	1	1	0.621
IPI00215948	CTNNA1	CTNNA1 Isoform 1 of Catenin alpha-1	659	100009	8	4	3	2	0.626
IPI00215995	ITGA3	ITGA3 Isoform Alpha-3A of Integrin alpha-3	325	116538	8	1	3	2	0.626
IPI00303476	ATP5B	ATP5B ATP synthase subunit beta, mitochondrial	3816	56525	72	8	5	5	0.639
IPI00012728	ACSL1	ACSL1 Isoform 1 of Long-chain-fatty-acid--CoA ligas	38	77893	1	1	1	1	0.643
IPI00646304	PPIB	PPIB Peptidyl-prolyl cis-trans isomerase B	240	23728	6	3	2	2	0.646
IPI00294065	GOLGA5	GOLGA5 Isoform 1 of Golgin subfamily A member 5	157	82940	2	1	1	1	0.648
IPI00005107	NPC1	NPC1 Niemann-Pick C1 protein	868	142074	29	8	4	4	0.65
IPI00006379	NOP58	NOP58 Nucleolar protein 58	98	59541	2	2	1	1	0.65
IPI00029741	ITGB5	ITGB5 Integrin beta-5	471	87996	13	6	2	2	0.651
IPI00100247	TMX4	TMX4 Thioredoxin-related transmembrane protein 4	101	38928	2	2	1	1	0.652
IPI00291928	RAB14	RAB14 Ras-related protein Rab-14	3290	23882	27	6	4	4	0.652
IPI00008732	TRABD	TRABD Isoform 1 of TraB domain-containing protein	337	42294	7	4	2	2	0.656
IPI00016342	RAB7A	RAB7A Ras-related protein Rab-7a	5053	23475	130	8	13	12	0.656
IPI00009253	NAPA	NAPA Alpha-soluble NSF attachment protein	84	33211	1	1	1	1	0.657
IPI00008338	ABCC1	ABCC1 Isoform 2 of Multidrug resistance-associated	270	164835	13	6	2	2	0.658
IPI00015902	PDGFRB	PDGFRB Beta-type platelet-derived growth factor rec	718	123889	13	1	6	6	0.66

Data1. AccessionNo (IPI)	iPS_CFB50/ HFF	iPS_CFB46/ H9ES	iPS_CFB50/ H9ES	iPS_CFB46/N TU1ES	iPS_CFB50/N TU1ES	H9ES/ HFF	NTU1ES/ HFF
IPI00002230	0.59	1.314	0.803	1.247	1.363	0.471	0.434
IPI00884105	0.426	1.377	1.064	0.93	0.713	0.413	0.591
IPI00007940	0.662	1.337	1.331	0.793	0.782	0.67	0.652
IPI00409635	0.382	1.251	0.857	1.151	0.781	0.452	0.479
IPI00021439	0.511	0.965	0.878	0.865	0.783	0.587	0.642
IPI00011416	0.679	0.242	0.292	2.136	2.547	2.356	0.261
IPI00465248	0.489	1.362	1.243	1.308	1.182	0.423	0.414
IPI00300562	0.169	0.845	0.225	6.254	1.913	0.659	0.091
IPI00021267	0.917	0.729	1.158	0.908	1.45	0.795	0.623
IPI00043598	0.611	9999	9999	1.756	1.859	-9999	0.301
IPI00657706	0.879	1.194	1.813	0.903	1.439	0.476	0.642
IPI00008274	0.557	1.381	1.34	0.618	0.593	0.421	0.919
IPI00647457	0.885	0.737	1.093	0.76	1.119	0.805	0.773
IPI00015102	0.863	1.014	1.518	0.525	1.351	0.546	0.582
IPI00005737	0.716	0.712	0.899	0.962	1.164	0.803	0.596
IPI00410079	0.214	1.632	0.602	5.907	2.156	0.36	0.097
IPI00012486	0.397	1.258	0.86	1.076	0.728	0.471	0.523
IPI00023768	-9999	0.605	-9999	9999	NaN	0.975	-9999
IPI00027230	0.685	0.861	0.982	0.88	1.014	0.71	0.65
IPI00170934	-9999	1.122	-9999	2.133	-9999	0.532	0.273
IPI00002149	0.623	9999	9999	9999	9999	-9999	-9999
IPI00030820	0.542	1.229	1.122	1.467	1.327	0.489	0.4
IPI00003348	0.673	0.887	1.005	1.169	1.311	0.678	0.502
IPI00010218	1.113	0.374	0.701	1.363	2.528	1.609	0.431
IPI00022774	0.653	0.872	0.96	1.521	1.656	0.69	0.386
IPI00186290	0.286	1.159	0.57	1.702	0.963	0.554	0.144
IPI00922108	0.808	1.08	1.439	0.486	0.645	0.558	1.469
IPI00166891	1.242	0.404	0.832	0.626	1.275	1.513	0.953
IPI00005181	0.342	1.311	0.74	1.714	0.958	0.468	0.349
IPI00215948	0.545	0.927	0.813	3.112	2.013	0.673	0.248
IPI00215995	-9999	9999	NaN	9999	NaN	-9999	-9999
IPI00303476	0.781	1.125	1.437	1.124	0.889	0.562	0.6
IPI00012728	0.907	9999	9999	9999	9999	-9999	-9999
IPI00646304	0.347	0.897	0.747	1.252	1.033	0.47	0.329
IPI00294065	-9999	9999	NaN	9999	NaN	-9999	-9999
IPI00005107	0.813	0.903	1.112	0.331	0.396	0.701	2.034
IPI00006379	0.746	1.353	1.591	0.696	0.81	0.475	0.901
IPI00029741	1.688	0.28	0.746	0.59	1.553	2.293	1.064
IPI00100247	1.305	0.792	1.626	0.481	0.978	0.813	1.306
IPI00291928	0.367	1.412	0.834	0.234	0.134	0.45	2.022
IPI00008732	0.36	0.062	0.067	1.097	0.626	NaN	0.578
IPI00016342	0.398	1.284	0.76	1.577	0.982	0.444	0.179
IPI00009253	0.68	9999	9999	0.998	1.049	-9999	0.634
IPI00008338	1.701	0.308	0.812	0.643	1.655	2.083	1.084
IPI00015902	0.535	1.238	0.88	0.975	0.913	0.787	0.495

Table S1 Identified proteins in the human membrane proteome of hiPSCs, hESCs and somatic foreskin fibroblast HFF.
Profiles of identified proteins in MS Experiment 1

Data1. AccessionNo (IPI)	Gene Symbol	Description	Score	Mass	# of identified (spectrum)	# of samples identified	Total peptides	# of peptides used	iPS_CFB46 /HFF
IPI00008964	RAB1B	RAB1B Ras-related protein Rab-1B	8579	22157	204	8	8	7	0.663
IPI00787853	IMPAD1	IMPAD1 Inositol monophosphatase 3	432	38657	3	1	1	1	0.664
IPI00794169	ANO10	ANO10 Putative uncharacterized protein ANO10	117	76408	4	3	2	2	0.676
IPI00218924	CHP	CHP Calcium-binding protein p22	167	22442	4	2	1	1	0.677
IPI00293946	UBXN4	UBXN4 UBX domain-containing protein 4	125	56743	4	2	1	1	0.69
IPI00297037	PPAP2A	PPAP2A Isoform 1 of Lipid phosphate phosphohydrc	382	32135	8	6	1	1	0.694
IPI00329389	RPL6	RPL6 60S ribosomal protein L6	1567	32708	27	8	2	2	0.694
IPI00022585	AKAP1	AKAP1 cDNA FLJ56047, highly similar to A kinase a	133	101413	2	1	1	1	0.695
IPI00008708	RSL1D1	RSL1D1 Ribosomal L1 domain-containing protein 1	191	54939	2	1	1	1	0.7
IPI00470649	NCLN	NCLN Isoform 1 of Nicalin	4551	62935	130	8	13	13	0.708
IPI00032038	CPT1A	CPT1A Isoform 1 of Carnitine O-palmitoyltransferase	741	88311	15	7	3	2	0.712
IPI00852623	PLXNB2	PLXNB2 Plexin B2	465	142609	13	2	5	5	0.714
IPI00010697	ITGA6	ITGA6 Isoform Alpha-6X1X2B of Integrin alpha-6	754	126553	29	6	5	5	0.715
IPI00221089	RPS13	RPS13 40S ribosomal protein S13	1264	17212	38	8	4	3	0.715
IPI00024282	RAB8B	RAB8B Ras-related protein Rab-8B	3033	23569	1	1	1	1	0.717
IPI00290085	CDH2	CDH2 Cadherin-2	61	99747	1	1	1	1	0.718
IPI00002790	SEL1L	SEL1L Isoform 1 of Protein sel-1 homolog 1	345	88698	10	3	3	3	0.721
IPI00645518	CDIPT	CDIPT Isoform 1 of CDP-diacylglycerol--inositol 3-ph	708	23523	19	8	3	3	0.722
IPI00303726	IFITM3	IFITM3 Interferon-induced transmembrane protein 3	1046	14622	17	6	3	2	0.723
IPI00789008	FLOT2	FLOT2 Flotillin-2	730	47035	35	7	5	5	0.728
IPI00291695	GPX8	GPX8 Probable glutathione peroxidase 8	1453	23894	67	8	6	5	0.73
IPI00013847	UQCRC1	UQCRC1 Cytochrome b-c1 complex subunit 1, mitoc	3779	52612	101	8	10	9	0.734
IPI00026781	FASN	FASN Fatty acid synthase	1033	273227	23	7	4	4	0.734
IPI00394699	RPL17	RPL17 Putative uncharacterized protein ENSP0000C	39	15278	1	1	1	1	0.74
IPI00294755	CLDN7	CLDN7 Isoform 1 of Claudin-7	130	22375	3	3	1	1	0.742
IPI00026272	HIST1H2AE;HIST	HIST1H2AE;HIST1H2AB;HIST1H2AG;HIST1H2AK;I	1526	14127	35	7	2	2	0.745
IPI00748145	GNAI2	GNAI2 Isoform 1 of Guanine nucleotide-binding prote	4467	40425	53	8	5	5	0.745
IPI00012512	RRAS2	RRAS2 cDNA FLJ61162, highly similar to Ras-relate	846	24180	6	3	2	1	0.747
IPI00550165	DHRS7B	DHRS7B Dehydrogenase/reductase SDR family mer	263	35097	5	3	1	1	0.747
IPI00101651	ABHD8	ABHD8 Abhydrolase domain-containing protein 8	39	47301	1	1	1	1	0.752
IPI00221093	RPS17	RPS17 40S ribosomal protein S17	1451	15540	21	7	3	2	0.752
IPI00028116	KDELR1	KDELR1 ER lumen protein retaining receptor 1	1614	24526	35	8	3	3	0.754
IPI00007061	GOLT1B	GOLT1B Vesicle transport protein GOT1B	1961	15415	25	8	1	1	0.756
IPI00014812	MRPS21	MRPS21 28S ribosomal protein S21, mitochondrial	44	10734	1	1	1	1	0.764
IPI00219217	LDHB	LDHB L-lactate dehydrogenase B chain	875	36615	13	5	1	1	0.766
IPI00008982	ALDH18A1	ALDH18A1 Isoform Long of Delta-1-pyrroline-5-carbo	622	87248	12	6	1	1	0.768
IPI00218918	ANXA1	ANXA1 Annexin A1	1608	38690	32	7	5	5	0.784
IPI00182933	CYB5A	CYB5A Isoform 2 of Cytochrome b5	848	11261	26	6	4	3	0.785
IPI00743576	ATP6V0A1	ATP6V0A1 Isoform 2 of V-type proton ATPase 116 k	335	95694	6	1	2	2	0.785
IPI00215767	B4GALT1	B4GALT1 Isoform Long of Beta-1,4-galactosyltransfe	367	43892	3	1	1	1	0.794
IPI00304331	B3GAT3	B3GAT3 Galactosylgalactosylxylosylprotein 3-beta-g	90	37099	2	1	2	2	0.795
IPI00100030	PIGT	PIGT Isoform 1 of GPI transamidase component PIG	1055	65658	21	8	1	1	0.802
IPI00016339	RAB5C	RAB5C Ras-related protein Rab-5C	3759	23468	95	8	7	5	0.803
IPI00332106	PBXIP1	PBXIP1 Isoform 1 of Pre-B-cell leukemia transcriptio	918	80594	18	3	5	5	0.804
IPI00328112	TAP2	TAP2 Antigen peptide transporter 2	103	75616	5	3	2	2	0.806

Data1. AccessionNo (IPI)	iPS_CFB50/ HFF	iPS_CFB46/ H9ES	iPS_CFB50/ H9ES	iPS_CFB46/N TU1ES	iPS_CFB50/N TU1ES	H9ES/ HFF	NTU1ES/ HFF
IPI00008964	0.488	1.119	0.96	1.309	1.107	0.623	0.416
IPI00787853	0.628	1.112	1.079	2.588	2.486	0.59	0.247
IPI00794169	0.76	1.049	1.212	0.97	1.109	0.635	0.669
IPI00218924	0.597	0.95	0.859	1.307	1.17	0.704	0.5
IPI00293946	0.51	1.761	1.336	5.744	4.313	0.387	0.116
IPI00297037	0.734	0.821	0.891	0.944	1.014	0.835	0.709
IPI00329389	0.658	0.875	0.851	1.284	1.236	0.783	0.521
IPI00022585	1.84	0.036	0.099	0.644	1.731	18.92	1.041
IPI00008708	1.132	0.663	1.098	0.414	0.679	1.044	1.631
IPI00470649	0.823	1.207	1.384	0.814	0.916	0.396	0.86
IPI00032038	1.172	0.826	0.809	1	0.968	1.477	1.185
IPI00852623	0.45	1.016	0.649	1.511	0.949	0.704	7.577
IPI00010697	0.564	1.332	1.083	0.977	0.784	0.561	0.777
IPI00221089	0.875	1.302	1.676	0.869	1.098	0.542	0.78
IPI00024282	0.537	0.828	0.636	1.259	0.957	0.856	0.549
IPI00290085	0.366	1.094	0.571	1.521	0.786	0.649	0.455
IPI00002790	0.976	1.153	1.382	1.292	1.642	0.618	0.495
IPI00645518	0.675	0.96	0.919	1.248	1.184	0.741	0.557
IPI00303726	0.662	1.434	1.365	0.672	0.678	0.499	1.088
IPI00789008	0.791	0.955	1.069	1.01	1.197	0.753	0.591
IPI00291695	0.713	1	1.032	0.644	0.644	0.692	1.106
IPI00013847	0.863	0.919	0.95	1.002	1.032	0.776	0.777
IPI00026781	0.587	0.908	0.753	1.963	1.589	0.791	0.354
IPI00394699	0.806	9999	9999	1.047	1.159	-9999	0.681
IPI00294755	0.76	1.498	1.573	1.455	1.512	0.489	0.492
IPI00026272	0.815	1.265	1.499	0.838	0.983	0.501	0.768
IPI00748145	0.691	1.06	1.01	0.857	0.807	0.695	0.835
IPI00012512	0.348	1.281	0.612	1.716	0.812	0.576	0.42
IPI00550165	0.784	0.778	0.838	0.998	1.064	0.947	0.721
IPI00101651	2.077	0.314	0.89	0.722	2.023	2.364	1.005
IPI00221093	1.247	1.155	1.989	0.285	0.534	0.634	2.469
IPI00028116	0.885	0.613	0.732	1.03	1.239	1.218	0.703
IPI00007061	0.855	1.208	1.402	1.397	1.605	0.618	0.522
IPI00014812	0.565	1.21	0.918	1.777	1.335	0.624	0.414
IPI00219217	0.382	1.467	0.75	1.617	0.819	0.516	0.457
IPI00008982	0.582	0.844	0.656	9999	9999	0.9	-9999
IPI00218918	0.809	1.081	1.14	0.96	1.02	0.715	0.473
IPI00182933	0.988	1.273	1.633	0.919	1.166	0.614	0.828
IPI00743576	-9999	0.616	-9999	1.031	-9999	0.691	0.403
IPI00215767	1.04	1.688	2.268	3.031	4.034	0.465	0.252
IPI00304331	0.825	0.857	0.914	1.074	1.133	0.915	0.713
IPI00100030	0.919	0.898	1.055	1.061	1.234	0.883	0.729
IPI00016339	0.721	0.438	0.39	1.156	1.076	2.526	0.628
IPI00332106	0.843	1.189	1.279	1.414	1.507	0.668	0.848
IPI00328112	0.848	0.805	0.868	0.979	1.044	1.001	0.798

Table S1 Identified proteins in the human membrane proteome of hiPSCs, hESCs and somatic foreskin fibroblast HFF.
Profiles of identified proteins in MS Experiment 1

Data1. AccessionNo (IPI)	Gene Symbol	Description	Score	Mass	# of identified (spectrum)	# of samples identified	Total peptides	# of peptides used	iPS_CFB46 /HFF
IPI00031169	RAB2A	RAB2A Ras-related protein Rab-2A	4833	23531	109	8	8	7	0.81
IPI00018855	ATP6V0C	ATP6V0C V-type proton ATPase 16 kDa proteolipid	773	15725	26	7	4	2	0.815
IPI00043564	SFXN2	SFXN2 Sideroflexin-2	92	36208	2	1	1	1	0.818
IPI00031755	RCE1	RCE1 CAAX prenyl protease 2	37	35809	1	1	1	1	0.821
IPI00641924	MRPS9	MRPS9 28S ribosomal protein S9, mitochondrial	41	45806	1	1	1	1	0.823
IPI00016372	RAB9A	RAB9A Ras-related protein Rab-9A	619	22823	19	5	3	3	0.825
IPI00440493	ATP5A1	ATP5A1 ATP synthase subunit alpha, mitochondrial	4026	59714	72	8	9	9	0.827
IPI00009896	EPHX1	EPHX1 Epoxide hydrolase 1	2287	52915	47	8	7	7	0.828
IPI00290799	C18orf19	C18orf19 Uncharacterized protein C18orf19	390	30785	10	7	1	1	0.834
IPI00013415	RPS7	RPS7 40S ribosomal protein S7	1110	22113	28	8	2	2	0.836
IPI00013271	DERL1	DERL1 Derlin-1	712	28782	23	8	3	3	0.837
IPI00016513	RAB10	RAB10 Ras-related protein Rab-10	3713	22527	25	8	2	2	0.839
IPI00028911	DAG1	DAG1 Dystroglycan	180	97381	3	2	1	1	0.842
IPI00001159	GCN1L1	GCN1L1 Translational activator GCN1	208	292558	6	3	3	3	0.843
IPI00385267	SRPR	SRPR Signal recognition particle receptor subunit al	387	69767	15	6	2	2	0.843
IPI00299468	SCD	SCD Acyl-CoA desaturase	726	41496	20	7	2	2	0.846
IPI00555610	AHNAK	AHNAK Putative uncharacterized protein AHNAK	196	312222	4	2	1	1	0.85
IPI00220740	NPM1	NPM1 Isoform 2 of Nucleophosmin	1493	29446	18	6	2	2	0.852
IPI00025491	EIF4A1;SNORA6	EIF4A1;SNORA67 Eukaryotic initiation factor 4A-I	435	46125	10	5	2	2	0.853
IPI00420108	DLSTP;DLST	DLSTP;DLST Dihydrolipoyllysine-residue succinyltra	1817	48698	40	8	4	4	0.854
IPI00023135	GOSR2	GOSR2 Isoform A of Golgi SNAP receptor complex r	839	24760	12	4	2	2	0.856
IPI00002506	ALG5	ALG5 Dolichyl-phosphate beta-glucosyltransferase	129	36922	3	1	1	1	0.861
IPI00011694	PRSS1	PRSS1 Trypsin-1	378	26541	17	7	1	1	0.861
IPI00328180	RAB34	RAB34 Isoform 1 of Ras-related protein Rab-34	299	29026	4	2	2	2	0.862
IPI00025366	CS	CS Citrate synthase, mitochondrial	112	51680	2	2	1	1	0.87
IPI00002372	ABCD3	ABCD3 Isoform 1 of ATP-binding cassette sub-family	1093	75428	38	8	3	3	0.871
IPI00157790	KIAA0368	KIAA0368 KIAA0368 protein	50	223552	1	1	1	1	0.872
IPI00396485	EEF1A1	EEF1A1 Elongation factor 1-alpha 1	2577	50109	114	8	10	9	0.872
IPI00021983	NCSTN	NCSTN Isoform 1 of Nicastrin	3025	78362	69	8	5	4	0.873
IPI00294242	MRPS31	MRPS31 28S ribosomal protein S31, mitochondrial	107	45290	1	1	1	1	0.874
IPI00005202	PGRMC2	PGRMC2 Membrane-associated progesterone recep	3383	26154	47	8	3	3	0.885
IPI00031397	ACSL3	ACSL3 Long-chain-fatty-acid--CoA ligase 3	1171	80368	21	7	2	2	0.886
IPI00010796	P4HB	P4HB Protein disulfide-isomerase	951	57081	18	2	7	6	0.889
IPI00069693	=-	#NAME?	974	23386	19	7	1	1	0.89
IPI00554648	KRT8	KRT8 Keratin, type II cytoskeletal 8	1079	53671	17	1	6	6	0.891
IPI00290945	PON2	PON2 paraoxonase 2 isoform 1	298	39356	7	3	4	4	0.897
IPI00009950	LMAN2	LMAN2 Vesicular integral-membrane protein VIP36	1174	40203	30	6	6	6	0.9
IPI00294472	TMED5	TMED5 Transmembrane emp24 domain-containing p	577	25988	21	7	2	2	0.9
IPI00453473	HIST1H4K;HIST1	HIST1H4K;HIST1H4E;HIST2H4B;HIST1H4H;HIST1I	2619	11360	78	7	4	4	0.9
IPI00472810	UBE3C	UBE3C Isoform 2 of Ubiquitin-protein ligase E3C	37	74748	1	1	1	1	0.901
IPI00943562	LOC100133770	LOC100133770 similar to vacuolar protein sorting 35	48	41478	1	1	1	1	0.903
IPI00218343	TUBA1C	TUBA1C Tubulin alpha-1C chain	14212	49863	253	8	15	12	0.922
IPI00298289	RTN4	RTN4 Isoform 2 of Reticulon-4	6261	40293	117	8	6	5	0.923
IPI00471978	C2CD2	C2CD2 C2 domain-containing protein 2	163	75486	3	2	1	1	0.924
IPI00397526	MYH10	MYH10 Isoform 1 of Myosin-10	298	228858	3	3	1	1	0.939

Data1. AccessionNo (IPI)	iPS_CFB50/ HFF	iPS_CFB46/ H9ES	iPS_CFB50/ H9ES	iPS_CFB46/N TU1ES	iPS_CFB50/N TU1ES	H9ES/ HFF	NTU1ES/ HFF
IPI00031169	0.705	1.322	1.159	1.162	1.042	0.64	0.659
IPI00018855	0.665	1.004	0.844	0.847	0.704	0.801	0.927
IPI00043564	0.751	0.381	0.359	0.82	0.765	2.121	0.962
IPI00031755	-9999	0.943	-9999	9999	NaN	0.859	-9999
IPI00641924	1.158	0.851	1.229	0.714	1.021	0.955	1.11
IPI00016372	0.712	1.299	1.245	1.559	1.425	0.548	0.513
IPI00440493	0.784	0.874	0.897	1.095	1.097	0.798	0.716
IPI00009896	0.814	1.19	1.209	0.931	0.937	0.691	0.856
IPI00290799	1.992	0.251	0.616	0.39	0.947	3.274	2.06
IPI00013415	0.545	1.234	0.824	1.383	0.916	0.668	0.582
IPI00013271	1.215	1	1.12	0.921	1.02	1.099	1.165
IPI00016513	0.647	1.153	0.912	1.097	0.859	0.719	0.738
IPI00028911	0.627	1.17	0.894	1.343	1.017	0.711	0.604
IPI00001159	0.862	1.079	1.146	1.188	1.233	0.772	0.685
IPI00385267	0.945	1.315	1.534	0.842	0.962	0.637	0.966
IPI00299468	1.082	1.27	1.667	1.086	1.41	0.658	0.751
IPI00555610	0.86	1.128	1.17	1.142	1.173	0.744	0.717
IPI00220740	0.535	1.155	0.754	2.008	1.3	0.726	0.394
IPI00025491	0.528	1.213	0.772	1.23	0.604	0.695	0.719
IPI00420108	0.448	1.265	0.757	2.087	1.179	0.665	0.356
IPI00023135	0.547	1.038	0.687	1.567	1.09	0.815	0.588
IPI00002506	0.736	1.076	0.944	1.581	1.374	0.79	0.525
IPI00011694	1.091	1.034	1.344	0.967	1.245	0.823	0.858
IPI00328180	0.352	1.645	0.687	3.049	1.559	0.512	0.194
IPI00025366	0.465	0.576	0.316	9999	9999	1.492	-9999
IPI00002372	0.539	1.27	0.809	2.001	1.256	0.677	0.4
IPI00157790	0.729	0.998	0.855	0.873	0.741	0.863	0.963
IPI00396485	0.503	1.414	0.906	1.577	1.127	0.575	0.457
IPI00021983	0.751	0.97	0.863	0.996	0.826	0.929	0.873
IPI00294242	0.414	1.063	0.516	9999	9999	0.812	-9999
IPI00005202	1.175	1.048	1.433	0.711	0.957	0.835	1.202
IPI00031397	0.843	0.621	0.606	0.76	0.688	1.411	1.073
IPI00010796	0.646	1.791	1.941	0.72	0.341	0.649	0.1
IPI00069693	0.908	1.243	1.301	1.769	1.833	0.707	0.485
IPI00554648	0.919	1.063	2.524	NaN	1.566	0.709	0.574
IPI00290945	1.132	1.014	1.315	0.966	1.239	0.866	0.893
IPI00009950	0.848	1.704	1.703	1.451	1.262	0.473	0.559
IPI00294472	0.919	1.19	1.246	0.995	1.032	0.747	0.871
IPI00453473	0.775	1.599	1.391	1.187	1.039	0.59	0.728
IPI00472810	1.2	1.781	2.435	0.835	1.13	0.499	1.039
IPI00943562	1.633	1.006	1.865	1.495	2.747	0.887	0.582
IPI00218343	1.106	1.084	1.339	0.683	0.833	0.834	1.328
IPI00298289	0.747	1.266	0.981	1.609	1.188	0.721	0.559
IPI00471978	1.19	0.879	1.16	1.856	2.426	1.039	0.48
IPI00397526	1.008	1.091	1.201	0.955	1.041	0.85	0.948

Table S1 Identified proteins in the human membrane proteome of hiPSCs, hESCs and somatic foreskin fibroblast HFF.
Profiles of identified proteins in MS Experiment 1

Data1. AccessionNo (IPI)	Gene Symbol	Description	Score	Mass	# of identified (spectrum)	# of samples identified	Total peptides	# of peptides used	iPS_CFB46 /HFF
IPI00028481	RAB8A	RAB8A Ras-related protein Rab-8A	4419	23653	24	8	2	2	0.948
IPI00025729	CAMLG	CAMLG Calcium signal-modulating cyclophilin liganc	397	32932	9	6	2	1	0.955
IPI00013485	RPS2	RPS2 40S ribosomal protein S2	561	31305	14	6	1	1	0.957
IPI00008495	MT-ND4	MT-ND4 NADH-ubiquinone oxidoreductase chain 4	957	51677	30	8	5	4	0.96
IPI00024933	RPL12	RPL12 Isoform 1 of 60S ribosomal protein L12	850	17808	28	8	2	2	0.962
IPI00031131	C20orf3	C20orf3 Isoform 1 of Adipocyte plasma membrane-a	6336	46451	168	8	17	11	0.962
IPI00456429	UBA52	UBA52 ubiquitin and ribosomal protein L40 precurso	2005	14719	46	8	3	3	0.964
IPI00301021	SSR1	SSR1 Isoform 1 of Translocon-associated protein su	1556	32215	34	8	2	2	0.965
IPI00788884	LOC643790;HMG	LOC643790;HMGN1 cDNA FLJ31471 fis, clone NT2	39	20231	1	1	1	1	0.969
IPI00059368	RFT1	RFT1 Protein RFT1 homolog	96	60296	2	1	1	1	0.973
IPI00793443	IPO5	IPO5 Isoform 1 of Importin-5	130	123550	2	2	1	1	0.974
IPI00001578	C17orf62	C17orf62 Uncharacterized protein C17orf62	90	20761	2	1	1	1	0.975
IPI00301554	MFSD5	MFSD5 Isoform 1 of Major facilitator superfamily dor	212	49732	3	1	1	1	0.977
IPI00019385	SSR4	SSR4 Translocon-associated protein subunit delta pi	4697	20200	105	8	7	4	0.98
IPI00026302	RPL31	RPL31 60S ribosomal protein L31	224	14454	6	3	2	2	0.983
IPI00000948	TBL2	TBL2 Transducin beta-like protein 2	157	49766	3	1	1	1	0.986
IPI00009346	TMEM14C	TMEM14C Transmembrane protein 14C	3695	11557	58	8	4	2	0.992
IPI00009342	IQGAP1	IQGAP1 Ras GTPase-activating-like protein IQGAP1	155	189134	3	1	3	3	0.993
IPI00021058	SLC4A7	SLC4A7 Solute carrier family 4 sodium bicarbonate c	396	127278	7	3	2	2	0.999
IPI00012750	RPS25	RPS25 40S ribosomal protein S25	445	13734	16	7	2	2	1.001
IPI00182469	CTNND1	CTNND1 Isoform 1AB of Catenin delta-1	2149	107283	42	8	6	5	1.002
IPI00003362	HSPA5	HSPA5 HSPA5 protein	12157	72377	284	8	25	22	1.004
IPI00215637	DDX3X	DDX3X ATP-dependent RNA helicase DDX3X	441	73198	14	4	3	3	1.007
IPI00217081	FUNDC1	FUNDC1 FUN14 domain-containing protein 1	67	17167	1	1	1	1	1.007
IPI00010153	RPL23	RPL23 60S ribosomal protein L23	1461	14856	22	8	1	1	1.008
IPI00022143	ESYT1	ESYT1 Isoform 1 of Extended synaptotagmin-1	10463	122780	228	8	21	20	1.008
IPI00172656	FAF2	FAF2 FAS-associated factor 2	1779	52591	36	8	7	6	1.008
IPI00013296	RPS18;RPS18P9	RPS18;RPS18P9 40S ribosomal protein S18	1938	17708	77	8	8	7	1.011
IPI00026530	LMAN1	LMAN1 Protein ERGIC-53	3514	57513	90	8	11	8	1.014
IPI00296215	EPCAM	EPCAM Epithelial cell adhesion molecule	1290	34910	16	6	2	2	1.016
IPI00019018	TM7SF2	TM7SF2 Isoform 1 of Delta(14)-sterol reductase	81	46375	1	1	1	1	1.018
IPI00465308	PIGS	PIGS Isoform 1 of GPI transamidase component PIG	2341	61617	28	8	4	3	1.019
IPI00554541	ILVBL	ILVBL Isoform 1 of Acetolactate synthase-like protei	5541	67825	75	8	7	6	1.019
IPI00171701	ORMDL2	ORMDL2 ORM1-like protein 2	573	17352	13	7	2	1	1.02
IPI00180386	GYG1	GYG1 Isoform GN-1L of Glycogenin-1	65	39359	2	1	1	1	1.022
IPI00005728	RER1	RER1 RER1 protein	446	24801	11	6	2	2	1.026
IPI00470924	TMTC3	TMTC3 Isoform 2 of Transmembrane and TPR repea	212	103814	7	3	3	3	1.031
IPI00220402	PPP1R2	PPP1R2 Protein phosphatase inhibitor 2	55	23001	3	1	1	1	1.04
IPI00020944	FDFT1	FDFT1 Squalene synthetase	NaN0	48084	107	6	12	10	1.041
IPI00152240	TMEM167A	TMEM167A Transmembrane protein 167A	813	8054	23	8	1	1	1.048
IPI00221092	RPS16	RPS16 40S ribosomal protein S16	1006	16435	26	8	2	2	1.048
IPI00396321	LRRC59	LRRC59 Leucine-rich repeat-containing protein 59	4112	34909	101	8	7	6	1.053
IPI00029447	GOSR1	GOSR1 Golgi SNAP receptor complex member 1	158	28595	3	1	1	1	1.056
IPI00215918	ARF4	ARF4 ADP-ribosylation factor 4	330	20498	4	3	3	3	1.057
IPI00028635	RPN2	RPN2 Dolichyl-diphosphooligosaccharide--protein gly	35883	69241	509	8	30	20	1.058

Data1. AccessionNo (IPI)	iPS_CFB50/ HFF	iPS_CFB46/ H9ES	iPS_CFB50/ H9ES	iPS_CFB46/N TU1ES	iPS_CFB50/N TU1ES	H9ES/ HFF	NTU1ES/ HFF
IPI00028481	0.631	1.21	0.837	1.628	1.073	0.773	0.449
IPI00025729	1.434	0.699	1.104	1.602	2.537	1.314	0.554
IPI00013485	0.757	0.773	0.627	1.365	1.096	1.223	0.676
IPI00008495	0.686	1.31	1.08	1.108	0.906	0.683	0.755
IPI00024933	0.841	1.425	1.278	1.18	1.098	0.665	0.746
IPI00031131	1.199	1.094	1.331	0.802	0.961	0.863	1.238
IPI00456429	0.674	1.573	1.117	1.592	1.131	0.608	0.586
IPI00301021	1.008	1.005	1.025	0.979	0.928	0.996	1.064
IPI00788884	0.947	9999	9999	0.685	0.679	-9999	1.364
IPI00059368	1.474	1.212	1.882	1.339	2.06	0.794	0.701
IPI00793443	0.568	1.282	0.768	1.426	0.846	0.75	0.658
IPI00001578	1.25	1.313	1.726	1.046	1.361	0.734	0.899
IPI00301554	0.985	0.869	0.899	1.545	1.583	1.11	0.609
IPI00019385	1.181	1.121	1.387	0.929	1.134	0.853	1.016
IPI00026302	0.769	1.207	0.974	1.048	0.919	0.803	0.685
IPI00000948	0.886	1.181	1.089	1.022	0.933	0.825	0.93
IPI00009346	1.483	0.723	1.105	0.957	1.447	1.363	0.999
IPI00009342	0.603	1.019	0.687	2.166	1.449	1.003	0.4
IPI00021058	0.578	0.993	0.59	2.237	1.319	0.994	0.425
IPI00012750	1.247	1.119	1.434	1.048	1.331	0.839	0.881
IPI00182469	1.008	0.771	0.835	0.893	0.981	1.285	1.069
IPI00003362	1.176	1.047	1.253	1.58	1.755	1.009	0.629
IPI00215637	0.983	0.785	0.782	0.831	0.802	1.311	1.172
IPI00217081	0.866	0.916	0.808	2.029	1.772	1.086	0.479
IPI00010153	0.999	1.435	1.46	1.025	1.033	0.693	0.947
IPI00022143	0.887	0.984	0.856	1.6	1.406	1.012	0.639
IPI00172656	0.994	0.992	1.041	1.041	1.084	0.991	0.932
IPI00013296	0.885	1.234	1.116	1.045	0.921	0.789	0.875
IPI00026530	1.203	1.079	1.32	1.013	1.184	0.988	0.885
IPI00296215	1.103	0.699	0.778	0.478	0.527	1.436	2.05
IPI00019018	0.721	1.112	0.807	0.924	0.664	0.904	1.062
IPI00465308	1.271	0.644	0.825	0.77	0.973	1.601	1.274
IPI00554541	0.929	1.455	1.434	0.958	0.934	0.668	0.975
IPI00171701	2.058	0.43	0.89	0.832	1.704	2.343	1.182
IPI00180386	-9999	1.29	-9999	9999	NaN	0.783	-9999
IPI00005728	0.979	1.042	1.02	1.397	1.355	0.973	0.708
IPI00470924	0.559	1.432	1.17	1.133	0.918	0.544	1.031
IPI00220402	0.371	9999	9999	9999	9999	-9999	-9999
IPI00020944	0.946	1.298	1.269	1.206	1.181	0.757	0.811
IPI00152240	1.236	1.094	1.323	1.051	1.258	0.946	0.961
IPI00221092	1.151	0.915	1.031	1.059	1.181	1.136	0.95
IPI00396321	0.977	1.1	1.04	1.085	1.011	0.928	0.936
IPI00029447	-9999	0.326	-9999	9999	NaN	3.2	-9999
IPI00215918	0.801	0.852	0.652	0.541	0.623	2.995	0.312
IPI00028635	1.332	1.244	1.598	0.882	1.134	0.844	1.15

Table S1 Identified proteins in the human membrane proteome of hiPSCs, hESCs and somatic foreskin fibroblast HFF.
Profiles of identified proteins in MS Experiment 1

Data1. AccessionNo (IPI)	Gene Symbol	Description	Score	Mass	# of identified (spectrum)	# of samples identified	Total peptides	# of peptides used	iPS_CFB46 /HFF
IPI00916241	TMEM87B	TMEM87B Protein	102	21419	1	1	1	1	1.063
IPI00025874	RPN1	RPN1 Dolichyl-diphosphooligosaccharide--protein gly	16026	72733	383	8	26	21	1.068
IPI00073779	MRPS35	MRPS35 Isoform 1 of 28S ribosomal protein S35, mi	49	36821	1	1	1	1	1.069
IPI00220578	GNAI3	GNAI3 Guanine nucleotide-binding protein G(k) subu	4473	40506	85	8	6	6	1.071
IPI00221091	RPS15A	RPS15A 40S ribosomal protein S15a	266	14830	7	5	2	2	1.071
IPI00020472	TMEM111	TMEM111 Isoform 1 of Transmembrane protein 111	150	29932	2	2	1	1	1.078
IPI00297492	STT3A	STT3A Dolichyl-diphosphooligosaccharide--protein g	5085	80477	163	8	11	10	1.082
IPI00028055	TMED10	TMED10 Transmembrane emp24 domain-containing	7883	24960	157	8	8	5	1.084
IPI00163644	OSBPL8	OSBPL8 Oxysterol-binding protein	520	101234	10	5	2	2	1.088
IPI00007752	TUBB2C	TUBB2C Tubulin beta-2C chain	16343	49799	55	8	6	4	1.091
IPI00011654	TUBB	TUBB Tubulin beta chain	20185	49639	464	8	25	14	1.092
IPI00104128	SEC11A	SEC11A Signal peptidase complex catalytic subunit	1682	20612	67	8	7	6	1.094
IPI00019407	NSDHL	NSDHL Sterol-4-alpha-carboxylate 3-dehydrogenase	1934	41874	43	8	5	3	1.099
IPI00295098	SRPRB	SRPRB Signal recognition particle receptor subunit b	2058	29684	47	8	10	8	1.099
IPI00217519	RALA	RALA Ras-related protein Ral-A	2171	23552	66	8	5	4	1.102
IPI00021304	KRT2	KRT2 Keratin, type II cytoskeletal 2 epidermal	3133	65825	53	8	6	6	1.104
IPI00470467	POR	POR NADPH--cytochrome P450 reductase	4174	76641	78	8	10	8	1.105
IPI00016608	TMED2	TMED2 Transmembrane emp24 domain-containing p	7158	22746	127	8	9	4	1.108
IPI00783698	TMEM87A	TMEM87A Isoform 1 of Transmembrane protein 87A	465	63389	18	7	2	2	1.11
IPI00028369	KIAA1715	KIAA1715 Isoform 1 of Protein lunapark	66	47710	1	1	1	1	1.111
IPI00216514	CD47	CD47 Isoform OA3-293 of Leukocyte surface antiger	45	31722	3	1	2	2	1.114
IPI00306748	ABCB7	ABCB7 Isoform 1 of ATP-binding cassette sub-family	1604	82589	46	6	6	6	1.122
IPI00009407	DAD1	DAD1 Dolichyl-diphosphooligosaccharide--protein gly	1355	12489	50	8	3	3	1.128
IPI00297084	DDOST	DDOST Dolichyl-diphosphooligosaccharide--protein g	9265	50769	234	8	16	11	1.131
IPI00216620	PPAP2C	PPAP2C Isoform 1 of Lipid phosphate phosphohydro	511	32553	10	5	1	1	1.134
IPI00013683	TUBB3	TUBB3 Tubulin beta-3 chain	13096	50400	9	4	3	3	1.139
IPI00646779	TUBB6	TUBB6 TUBB6 protein	9956	50058	23	7	5	3	1.139
IPI00022744	CSE1L	CSE1L Isoform 1 of Exportin-2	167	110346	4	3	1	1	1.144
IPI00027728	SLC7A1	SLC7A1 High affinity cationic amino acid transporter	966	67594	25	6	3	3	1.146
IPI00005068	SLC44A1	SLC44A1 Isoform 2 of Choline transporter-like protei	79	73247	1	1	1	1	1.147
IPI00022434	ALB	ALB Putative uncharacterized protein ALB	729	71658	12	3	2	1	1.15
IPI00029264	CYC1	CYC1 Cytochrome c1, heme protein, mitochondrial	1993	35367	50	6	6	4	1.16
IPI00303283	ITGB3	ITGB3 Isoform Beta-3A of Integrin beta-3	85	87000	2	1	1	1	1.163
IPI00021840	RPS6	RPS6 40S ribosomal protein S6	930	28663	18	6	2	2	1.164
IPI00218466	SEC61A1	SEC61A1 cDNA FLJ59739, highly similar to Protein	5032	52915	107	8	15	8	1.165
IPI00410034	SLC38A2	SLC38A2 Isoform 1 of Sodium-coupled neutral amin	919	55990	20	8	2	2	1.174
IPI00006072	SEC61G	SEC61G Protein transport protein Sec61 subunit gar	98	7736	8	6	1	1	1.179
IPI00010271	RAC1	RAC1 Isoform A of Ras-related C3 botulinum toxin su	426	21436	22	8	3	3	1.18
IPI00017292	CTNNB1	CTNNB1 Isoform 1 of Catenin beta-1	242	85442	6	3	3	3	1.181
IPI00328170	MOGS	MOGS Mannosyl-oligosaccharide glucosidase	4289	91861	99	8	10	9	1.182
IPI00220835	SEC61B	SEC61B Protein transport protein Sec61 subunit beta	974	9968	35	8	2	2	1.183
IPI00298947	MEST	MEST Isoform 1 of Mesoderm-specific transcript hor	1370	38806	21	6	4	4	1.183
IPI00000513	CDH1	CDH1 Cadherin 1, type 1, E-cadherin (Epithelial), isc	1065	90886	28	6	4	4	1.187
IPI00030179	RPL7P32;RPL7	RPL7P32;RPL7 60S ribosomal protein L7	1790	29207	51	8	7	7	1.188
IPI00184311	ENPP1	ENPP1 Ectonucleotide pyrophosphatase/phosphodie	628	104857	15	7	2	2	1.19

Data1. AccessionNo (IPI)	iPS_CFB50/ HFF	iPS_CFB46/ H9ES	iPS_CFB50/ H9ES	iPS_CFB46/N TU1ES	iPS_CFB50/N TU1ES	H9ES/ HFF	NTU1ES/ HFF
IPI00916241	1.329	0.79	1.013	9999	9999	1.329	-9999
IPI00025874	1.468	1.176	1.628	0.847	1.129	0.87	1.239
IPI00073779	-9999	1.159	-9999	9999	NaN	0.911	-9999
IPI00220578	1.297	0.817	1.033	0.783	0.964	1.306	1.322
IPI00221091	1.476	1.07	1.099	1.111	1.129	0.988	0.929
IPI00020472	1.202	0.994	1.136	0.917	1.038	1.072	1.133
IPI00297492	1.463	0.919	1.322	0.851	1.12	1.132	1.279
IPI00028055	1.237	1.025	1.202	0.892	1.038	1.051	1.165
IPI00163644	0.871	1.211	0.994	9999	9999	0.887	-9999
IPI00007752	1.121	1.024	1.037	1.003	0.858	0.741	1.295
IPI00011654	1.315	1.135	1.31	0.682	0.848	0.92	1.488
IPI00104128	1.538	1.165	1.551	0.818	1.061	0.924	1.414
IPI00019407	0.952	1.239	1.107	1.246	1.109	0.875	0.845
IPI00295098	1.135	1.129	1.209	1.007	1.062	0.954	1.047
IPI00217519	0.873	1.193	0.951	0.867	0.786	0.909	0.992
IPI00021304	0.783	1.283	0.997	0.844	0.611	0.77	1.258
IPI00470467	1.362	1	0.965	1.444	1.916	1.552	0.691
IPI00016608	1.105	0.966	0.959	0.947	0.952	1.139	1.153
IPI00783698	1.236	0.877	1.002	1.39	1.609	1.253	0.753
IPI00028369	-9999	9999	NaN	9999	NaN	-9999	-9999
IPI00216514	0.86	1.464	1.174	0.927	0.738	0.75	1.154
IPI00306748	0.976	1.156	1.118	1.134	1.144	1.071	0.676
IPI00009407	1.714	1.183	1.844	0.727	1.106	0.942	1.495
IPI00297084	1.557	1.102	1.557	0.798	1.11	1.018	1.366
IPI00216620	1.482	0.284	0.381	1.112	1.476	3.938	0.983
IPI00013683	1.421	1.354	1.733	0.904	1.146	0.717	1.213
IPI00646779	1.421	1.354	1.733	0.904	1.146	1.294	0.597
IPI00022744	1.534	0.759	1.043	0.735	1.001	1.489	1.5
IPI00027728	0.702	1.746	1.113	1.431	0.923	0.622	0.743
IPI00005068	1.216	1.733	1.884	1.095	1.179	0.654	1.01
IPI00022434	4.738	0.285	1.262	0.968	6.08	3.991	1.145
IPI00029264	1.548	0.897	0.792	0.638	0.557	2.919	2.877
IPI00303283	0.797	1.924	1.353	1.485	1.034	0.597	0.755
IPI00021840	1.151	NaN	NaN	0.913	0.916	0.817	1.651
IPI00218466	1.539	0.696	0.904	0.949	1.14	1.849	1.141
IPI00410034	0.428	1.62	0.644	2.316	0.907	0.675	0.432
IPI00006072	1.344	1.315	1.537	1.188	1.376	0.886	0.956
IPI00010271	0.929	1.393	1.398	1.046	0.837	0.674	1.087
IPI00017292	0.927	1.027	0.828	1.726	1.72	1.128	0.334
IPI00328170	1.513	1.071	1.192	0.716	0.944	1.275	1.655
IPI00220835	1.26	1.44	1.453	NaN	0.905	0.811	1.457
IPI00298947	2.406	0.77	1.39	0.57	1.145	1.4	1.773
IPI00000513	1.087	1.355	1.318	1.168	1.126	0.81	0.913
IPI00030179	1.161	1.033	1.039	0.816	0.813	1.089	1.381
IPI00184311	1.014	1.195	1.044	1.205	1.042	0.982	0.945

Table S1 Identified proteins in the human membrane proteome of hiPSCs, hESCs and somatic foreskin fibroblast HFF.
Profiles of identified proteins in MS Experiment 1

Data1. AccessionNo (IPI)	Gene Symbol	Description	Score	Mass	# of identified (spectrum)	# of samples identified	Total peptides	# of peptides used	iPS_CFB46 /HFF
IPI00337494	SLC25A24	SLC25A24 Isoform 1 of Calcium-binding mitochondri	3140	53320	53	7	6	6	1.191
IPI00101952	SLC35E1	SLC35E1 Isoform 1 of Solute carrier family 35 memb	131	44744	3	1	1	1	1.197
IPI00170692	VAPA	VAPA Vesicle-associated membrane protein-associa	1231	27875	38	7	5	5	1.197
IPI00003935	HIST2H2BE	HIST2H2BE Histone H2B type 2-E	1565	13912	19	7	2	1	1.199
IPI00023780	DNAJC5	DNAJC5 Isoform 2 of DnaJ homolog subfamily C me	292	18789	4	1	2	1	1.199
IPI00027107	TUFM	TUFM Tu translation elongation factor, mitochondrial	1562	49843	42	6	4	4	1.202
IPI00219155	RPL27	RPL27 60S ribosomal protein L27	321	15788	15	6	1	1	1.203
IPI00306332	RPL24	RPL24 60S ribosomal protein L24	1655	17768	46	8	2	2	1.206
IPI00009145	MAN1A2	MAN1A2 Mannosyl-oligosaccharide 1,2-alpha-mann	61	72957	1	1	1	1	1.207
IPI00007166	IER3IP1	IER3IP1 Immediate early response 3-interacting prot	1561	8963	17	7	1	1	1.21
IPI00006666	SLC16A3	SLC16A3 Monocarboxylate transporter 4	251	49437	7	2	2	2	1.212
IPI00176824	-	- 10 kDa protein	1702	10097	30	8	3	2	1.22
IPI00301494	CHST4	CHST4 Carbohydrate sulfotransferase 4	114	45104	3	3	1	1	1.22
IPI00003918	RPL4	RPL4 60S ribosomal protein L4	1084	47667	36	6	4	4	1.23
IPI00022462	TFRC	TFRC Transferrin receptor protein 1	5231	84818	145	8	14	14	1.23
IPI00027180	ZMPSTE24	ZMPSTE24 CAAX prenyl protease 1 homolog	3000	54778	51	8	6	5	1.233
IPI00008511	MT-ND5	MT-ND5 NADH-ubiquinone oxidoreductase chain 5	304	67355	6	3	2	1	1.234
IPI00177817	ATP2A2	ATP2A2 Isoform SERCA2A of Sarcoplasmic/endopla	17113	109620	339	8	28	23	1.245
IPI00025818	GALNT1	GALNT1 Isoform 1 of Polypeptide N-acetylgalactosa	489	64177	12	4	3	3	1.25
IPI00550021	RPL3	RPL3 60S ribosomal protein L3	1402	46080	32	8	3	3	1.254
IPI00221088	RPS9	RPS9 40S ribosomal protein S9	120	22578	5	3	1	1	1.257
IPI00027194	STX18	STX18 Syntaxin-18	187	38650	6	3	2	2	1.261
IPI00329352	NOMO3;NOMO1	NOMO3;NOMO1 Nodal modulator 1	4141	134267	105	8	13	13	1.265
IPI00034277	ATP13A1	ATP13A1 Isoform A of Probable cation-transporting /	260	132870	12	6	4	4	1.267
IPI00218914	ALDH1A1	ALDH1A1 Retinal dehydrogenase 1	291	54827	6	1	3	3	1.27
IPI00022543	PIGK	PIGK GPI-anchor transamidase	590	45223	18	8	2	2	1.271
IPI00299573	RPL7A	RPL7A 60S ribosomal protein L7a	4088	29977	104	8	7	7	1.274
IPI00305304	LASS2	LASS2 LAG1 longevity assurance homolog 2	1035	44847	24	6	5	4	1.277
IPI00215719	RPL18	RPL18 60S ribosomal protein L18	1883	21621	33	8	4	3	1.283
IPI00217600	PNPLA6	PNPLA6 Isoform 2 of Neuropathy target esterase	544	146123	14	5	2	2	1.297
IPI00023542	TMED9	TMED9 Transmembrane emp24 domain-containing p	2248	27260	101	8	6	5	1.298
IPI00009235	SSR3	SSR3 cDNA FLJ52061, highly similar to Translocon-	1460	22596	23	8	2	2	1.299
IPI00219518	ARL1	ARL1 ADP-ribosylation factor-like protein 1	1042	20404	21	8	2	2	1.303
IPI00220473	ATP2C1	ATP2C1 Isoform 2 of Calcium-transporting ATPase t	544	96897	8	2	3	2	1.305
IPI00152441	HM13	HM13 Isoform 1 of Minor histocompatibility antigen H	1175	41462	37	8	2	2	1.314
IPI00455473	MIA3	MIA3 Isoform 1 of Melanoma inhibitory activity protei	48	213570	1	1	1	1	1.316
IPI00008207	MAN1B1	MAN1B1 Endoplasmic reticulum mannosyl-oligosacc	280	79530	6	1	3	3	1.32
IPI00783271	LRPPRC	LRPPRC Leucine-rich PPR motif-containing protein,	2225	157805	43	7	14	13	1.322
IPI00006211	VAPB	VAPB Isoform 1 of Vesicle-associated membrane pr	1010	27211	22	6	3	3	1.324
IPI00019472	SLC1A5	SLC1A5 Neutral amino acid transporter B(0)	4969	56562	114	8	5	5	1.325
IPI00216237	RPL36	RPL36 60S ribosomal protein L36	658	12246	37	8	3	3	1.329
IPI00025292	MPDU1	MPDU1 Mannose-P-dolichol utilization defect 1 prote	1739	26620	48	8	2	2	1.33
IPI00029750	RPS24	RPS24 Isoform 1 of 40S ribosomal protein S24	1042	15413	29	8	3	3	1.335
IPI00021805	MGST1	MGST1 Microsomal glutathione S-transferase 1	3768	17587	70	8	10	4	1.339
IPI00166079	VKORC1L1	VKORC1L1 Vitamin K epoxide reductase complex su	693	19823	20	8	1	1	1.341

Data1. AccessionNo (IPI)	iPS_CFB50/ HFF	iPS_CFB46/ H9ES	iPS_CFB50/ H9ES	iPS_CFB46/N TU1ES	iPS_CFB50/N TU1ES	H9ES/ HFF	NTU1ES/ HFF
IPI00337494	0.954	0.924	0.764	0.649	0.53	1.276	1.775
IPI00101952	0.898	0.595	0.458	1.87	1.425	1.987	0.617
IPI00170692	0.58	1.398	1.151	1.855	0.782	0.872	0.455
IPI00003935	1.061	1.353	1.293	0.969	0.917	0.815	1.091
IPI00023780	0.939	1.063	0.855	1.351	1.084	1.113	0.85
IPI00027107	1.261	1.008	1.084	0.612	0.652	1.178	1.893
IPI00219155	1.136	1.232	1.194	0.851	0.816	0.964	1.363
IPI00306332	0.952	1.052	0.852	1.205	0.967	1.133	0.964
IPI00009145	1.056	1.158	1.039	1.808	1.606	1.03	0.644
IPI00007166	1.049	1.006	0.895	0.876	0.771	1.188	1.332
IPI00006666	0.692	1.967	2.007	1.382	1.068	0.34	0.64
IPI00176824	1.701	0.165	0.297	0.641	0.891	9.46	1.836
IPI00301494	1.714	0.617	0.888	0.861	1.228	1.955	1.367
IPI00003918	1.509	0.879	1.062	0.878	1.099	1.333	1.359
IPI00022462	0.957	0.894	0.693	1.026	0.816	1.334	1.147
IPI00027180	1.68	0.849	1.066	0.861	1.044	1.581	1.572
IPI00008511	1.449	0.622	0.75	0.793	0.946	1.957	1.501
IPI00177817	1.502	1.082	1.36	0.872	1.074	1.116	1.368
IPI00025818	1.113	1.014	1.102	1.658	1.333	0.635	3.23
IPI00550021	1.151	1.093	1.03	0.962	0.897	1.134	1.256
IPI00221088	1.216	0.988	0.98	9999	9999	1.257	-9999
IPI00027194	-9999	9999	NaN	9999	NaN	-9999	-9999
IPI00329352	1.328	1.015	1.185	0.91	1.088	1.022	1.484
IPI00034277	1.219	1.282	1.269	1.085	1.061	0.978	1.125
IPI00218914	1.122	1.233	1.071	1	1.058	1.011	1.035
IPI00022543	1.737	1.041	1.419	0.81	1.094	1.24	1.NaN
IPI00299573	1.053	1.199	1.02	1.091	0.919	1.048	1.122
IPI00305304	1.543	1.087	1.21	0.718	0.918	1.263	1.702
IPI00215719	1.18	1.181	1.115	0.883	0.826	1.072	1.401
IPI00217600	1.596	1.389	1.478	1.253	1.566	1.094	0.998
IPI00023542	1.537	1.067	1.3	0.86	1.039	1.206	1.455
IPI00009235	1.536	0.994	1.2	0.815	0.974	1.291	1.543
IPI00219518	0.8	0.995	0.629	1.214	0.757	1.291	1.022
IPI00220473	1.328	0.526	0.622	2.268	2.344	2.098	0.NaN
IPI00152441	1.401	1.229	1.343	0.773	0.838	1.059	1.639
IPI00455473	1.082	0.818	0.689	1.233	1.029	1.589	1.029
IPI00008207	0.92	1.021	0.782	2.022	1.545	1.23	0.614
IPI00783271	0.908	0.812	0.573	1.128	0.808	1.637	1.15
IPI00006211	2.189	1.788	1.957	1.649	0.941	0.716	0.689
IPI00019472	0.995	1.097	0.84	0.871	0.666	1.193	1.465
IPI00216237	0.916	1.305	0.952	1.278	0.921	0.998	1.108
IPI00025292	1.749	1.073	1.448	0.594	0.792	1.229	2.165
IPI00029750	0.793	1.088	0.66	1.533	0.926	1.211	0.836
IPI00021805	1.769	1.186	1.626	0.667	0.925	1.133	1.88
IPI00166079	1.59	0.927	1.127	0.611	0.736	1.43	2.115

Table S1 Identified proteins in the human membrane proteome of hiPSCs, hESCs and somatic foreskin fibroblast HFF.
Profiles of identified proteins in MS Experiment 1

Data1. AccessionNo (IPI)	Gene Symbol	Description	Score	Mass	# of identified (spectrum)	# of samples identified	Total peptides	# of peptides used	iPS_CFB46 /HFF
IPI00784154	HSPD1	HSPD1 60 kDa heat shock protein, mitochondrial	10219	61016	176	8	19	13	1.353
IPI00414676	HSP90AB1	HSP90AB1 Heat shock protein HSP 90-beta	3061	83212	67	8	10	8	1.36
IPI00395769	ATP5C1	ATP5C1 Isoform Heart of ATP synthase subunit gam	2219	32860	59	7	6	6	1.363
IPI00401819	RPS26	RPS26 Similar to 40S ribosomal protein S26	642	12977	17	8	2	2	1.367
IPI00218922	SEC63	SEC63 Translocation protein SEC63 homolog	1293	87942	55	8	6	6	1.369
IPI00257903	ERMP1	ERMP1 Endoplasmic reticulum metallopeptidase 1	81	100167	2	1	2	2	1.37
IPI00004962	GOLIM4	GOLIM4 Golgi integral membrane protein 4	402	81831	6	4	1	1	1.383
IPI00028387	DDRGK1	DDRGK1 Isoform 1 of DDRGK domain-containing pr	628	35589	10	3	3	3	1.383
IPI00247583	RPL21P19;RPL2	RPL21P19;RPL21 60S ribosomal protein L21	1255	18553	38	8	5	4	1.383
IPI00009364	ORMDL1	ORMDL1 ORM1-like protein 1	443	17360	12	6	2	1	1.384
IPI00012048	NME1	NME1 Isoform 1 of Nucleoside diphosphate kinase A	88	17138	2	1	1	1	1.386
IPI00014577	RAB18	RAB18 Ras-related protein Rab-18	248	22963	10	3	3	3	1.392
IPI00333619	ALDH3A2	ALDH3A2 Isoform 1 of Fatty aldehyde dehydrogenas	2791	54813	91	8	8	7	1.394
IPI00554723	RPL10	RPL10 60S ribosomal protein L10	1060	24588	32	8	2	2	1.403
IPI00025049	M6PR	M6PR Cation-dependent mannose-6-phosphate rece	466	30973	6	4	1	1	1.405
IPI00026942	ERLIN2	ERLIN2 Isoform 1 of Erlin-2	6617	37815	123	8	9	9	1.413
IPI00013930	STX6	STX6 Syntaxin-6	89	29158	1	1	1	1	1.415
IPI00103940	MFSD10	MFSD10 Major facilitator superfamily domain-contair	83	48308	3	2	1	1	1.423
IPI00026111	TMCO1	TMCO1 Isoform 1 of Transmembrane and coiled-coil	1785	21161	33	8	2	1	1.426
IPI00301841	TMEM161A	TMEM161A Transmembrane protein 161A	1041	53567	20	7	1	1	1.437
IPI00002236	MFGE8	MFGE8 Lactadherin	4294	43095	105	8	11	8	1.439
IPI00007676	HSD17B12	HSD17B12 Estradiol 17-beta-dehydrogenase 12	5980	34302	172	8	12	9	1.443
IPI00002520	SHMT2	SHMT2 Serine hydroxymethyltransferase, mitochonc	1162	55958	20	4	5	5	1.445
IPI00013421	GPM6B	GPM6B Isoform 1 of Neuronal membrane glycoprote	149	28969	5	3	1	1	1.446
IPI00151358	CLPTM1L	CLPTM1L Isoform 1 of Cleft lip and palate transmem	975	62189	20	7	2	1	1.45
IPI00296259	TMED4	TMED4 Isoform 1 of Transmembrane emp24 domair	708	25926	53	8	2	2	1.468
IPI00010746	PTDSS1	PTDSS1 Phosphatidylserine synthase 1	659	55491	20	8	3	3	1.472
IPI00045946	YME1L1	YME1L1 Isoform 1 of ATP-dependent metalloproteas	1903	86401	19	6	2	2	1.474
IPI00006657	FAM20B	FAM20B Protein FAM20B	321	46403	6	1	2	2	1.482
IPI00017767	MGST2	MGST2 Microsomal glutathione S-transferase 2	120	16610	2	1	1	1	1.482
IPI00024919	PRDX3	PRDX3 Thioredoxin-dependent peroxide reductase, r	44	27675	1	1	1	1	1.486
IPI00218200	BCAP31	BCAP31 B-cell receptor-associated protein 31	1081	27974	41	8	5	2	1.491
IPI00220327	KRT1	KRT1 Keratin, type II cytoskeletal 1	11090	65999	300	8	22	18	1.492
IPI00152377	STT3B	STT3B Dolichyl-diphosphooligosaccharide--protein g	2598	93614	71	8	5	5	1.496
IPI00003968	NDUFA9	NDUFA9 NADH dehydrogenase [ubiquinone] 1 alpha	3002	42483	93	8	7	7	1.507
IPI00289983	ACPP	ACPP Isoform 2 of Prostatic acid phosphatase	47	48305	3	3	1	1	1.507
IPI00023064	NDUFAF4	NDUFAF4 NADH dehydrogenase [ubiquinone] 1 alph	210	20254	4	2	2	2	1.508
IPI00941747	CANX	CANX Calnexin	21043	67526	479	8	25	17	1.521
IPI00032903	PTRH2	PTRH2 Peptidyl-tRNA hydrolase 2, mitochondrial	675	19181	21	8	1	1	1.523
IPI00009922	C14orf156	C14orf156 SRA stem-loop-interacting RNA-binding p	366	12341	15	5	4	3	1.529
IPI00024266	MGST3	MGST3 Microsomal glutathione S-transferase 3	2746	16506	49	8	3	3	1.547
IPI00300299	SPCS3	SPCS3 Signal peptidase complex subunit 3	1479	20301	44	8	3	2	1.547
IPI00020436	RAB11B	RAB11B Ras-related protein Rab-11B	2613	24473	87	8	7	7	1.561
IPI00018246	HK1	HK1 Isoform 1 of Hexokinase-1	5900	102420	154	8	17	17	1.57
IPI00006433	DNAJC16	DNAJC16 Isoform 1 of DnaJ homolog subfamily C m	429	90534	11	5	1	1	1.575

Data1. AccessionNo (IPI)	iPS_CFB50/ HFF	iPS_CFB46/ H9ES	iPS_CFB50/ H9ES	iPS_CFB46/N TU1ES	iPS_CFB50/N TU1ES	H9ES/ HFF	NTU1ES/ HFF
IPI00784154	1.075	0.623	0.454	1.418	1.034	2.35	0.869
IPI00414676	1.268	1.115	1.067	0.835	0.774	1.215	1.872
IPI00395769	1.117	0.879	0.79	0.954	0.807	1.359	1.628
IPI00401819	-9999	1.272	-9999	9999	NaN	1.061	-9999
IPI00218922	1.718	0.989	1.317	0.772	1.004	1.216	1.726
IPI00257903	1.987	1.006	1.497	0.755	1.112	1.344	1.749
IPI00004962	0.914	1.263	0.856	3.881	2.605	1.082	0.343
IPI00028387	1.035	1.412	0.625	12.619	NaN	0.955	0.069
IPI00247583	1.41	1.006	1.03	1.025	1.04	1.281	1.227
IPI00009364	2.25	0.601	1.003	0.725	1.196	2.273	1.841
IPI00012048	0.499	1.033	0.382	9999	9999	1.325	-9999
IPI00014577	1.196	0.893	0.68	1.078	0.814	1.293	1.044
IPI00333619	1.611	0.779	1.038	1.082	1.146	1.733	1.502
IPI00554723	1.283	1.24	1.194	1.58	1.467	1.479	0.865
IPI00025049	1.522	0.922	1.025	0.928	1.021	1.505	1.459
IPI00026942	1.634	1.187	1.412	0.88	1.033	1.175	1.547
IPI00013930	1.366	1.302	1.289	1.447	1.419	1.073	0.942
IPI00103940	1.848	1.168	1.NaN	0.977	1.289	1.204	1.404
IPI00026111	1.687	0.926	1.125	0.839	1.01	1.52	1.639
IPI00301841	1.666	0.98	1.166	0.752	0.886	1.448	1.841
IPI00002236	0.999	1.39	1.017	1.661	1.156	1.037	0.791
IPI00007676	1.688	1.167	1.406	0.942	1.129	1.225	1.485
IPI00002520	1.158	1.352	0.978	1.923	1.322	1.053	0.866
IPI00013421	1.284	0.886	0.807	0.525	0.473	1.612	2.656
IPI00151358	1.91	0.941	1.271	0.922	1.235	1.525	1.518
IPI00296259	0.835	0.96	0.558	0.875	0.501	1.52	1.621
IPI000010746	1.61	1.013	1.137	0.828	0.919	1.437	1.716
IPI00045946	0.864	1.147	0.69	1.07	0.637	1.269	1.328
IPI00006657	1.219	1.019	0.86	2.048	1.711	1.436	0.698
IPI00017767	2.182	1.512	2.283	1.266	1.893	0.968	1.129
IPI00024919	2.218	1.521	2.329	0.545	0.827	0.965	2.627
IPI00218200	1.5	1.396	1.321	0.982	0.935	1.451	1.569
IPI00220327	1.302	1.221	1.102	0.724	0.645	1.137	1.802
IPI00152377	2.011	1.131	1.554	0.769	1.047	1.307	1.882
IPI00003968	1.618	0.848	0.957	0.866	0.919	1.784	1.671
IPI00289983	1.851	1.135	1.43	0.63	0.786	1.312	2.306
IPI00023064	1.849	0.833	1.047	0.541	0.674	1.789	2.687
IPI00941747	1.829	1.126	1.57	0.854	1.158	1.337	1.717
IPI00032903	2.014	0.85	1.153	0.616	0.827	1.77	2.382
IPI00009922	1.423	1.278	0.875	1.37	0.927	1.308	1.249
IPI00024266	1.617	1.586	1.709	1.316	1.404	0.952	1.123
IPI00300299	3.046	0.926	1.726	0.747	1.303	1.779	1.999
IPI00020436	0.721	1.506	0.986	1.578	1.124	0.922	0.951
IPI00018246	0.809	1.039	0.697	1.58	1.03	1.307	0.671
IPI00006433	1.956	1.034	1.317	1.384	1.745	1.505	1.097

Table S1 Identified proteins in the human membrane proteome of hiPSCs, hESCs and somatic foreskin fibroblast HFF.
Profiles of identified proteins in MS Experiment 1

Data1. AccessionNo (IPI)	Gene Symbol	Description	Score	Mass	# of identified (spectrum)	# of samples identified	Total peptides	# of peptides used	iPS_CFB46 /HFF
IPI00030106	TPST1	TPST1 Protein-tyrosine sulfotransferase 1	52	42161	2	1	1	1	1.575
IPI00413784	SEPN1	SEPN1 Isoform 1 of Selenoprotein N	968	65907	17	7	1	1	1.582
IPI00306516	TIMM44	TIMM44 Mitochondrial import inner membrane transl	177	51323	4	2	2	2	1.584
IPI00217030	RPS4X	RPS4X 40S ribosomal protein S4, X isoform	2266	29579	49	8	4	4	1.585
IPI00032825	TMED7;TMED7-T	TMED7;TMED7-TICAM2;TICAM2 Transmembrane e	782	25156	27	8	3	3	1.588
IPI00216049	HNRNPK	HNRNPK Isoform 1 of Heterogeneous nuclear ribonu	155	50944	4	3	2	2	1.589
IPI00289819	IGF2R	IGF2R Cation-independent mannose-6-phosphate re	450	274100	15	5	4	3	1.596
IPI00003925	PDHB	PDHB Isoform 1 of Pyruvate dehydrogenase E1 com	124	39208	1	1	1	1	1.597
IPI00002732	EXTL2	EXTL2 Exostosin-like 2	78	37441	2	2	1	1	1.618
IPI00024143	AAAS	AAAS Aladin	866	59536	21	4	4	4	1.625
IPI00003926	CLN8	CLN8 Protein CLN8	220	32766	5	3	1	1	1.63
IPI00334282	FAM3C	FAM3C Protein FAM3C	3079	24665	63	7	4	3	1.636
IPI00027434	RHOC	RHOC Rho-related GTP-binding protein RhoC	183	21992	5	1	2	2	1.638
IPI00008167	ATP1B3	ATP1B3 Sodium/potassium-transporting ATPase sul	1999	31492	51	8	5	4	1.642
IPI00009865	KRT10	KRT10 Keratin, type I cytoskeletal 10	5339	58818	142	8	15	14	1.645
IPI00019359	KRT9	KRT9 Keratin, type I cytoskeletal 9	2608	62027	56	8	9	8	1.656
IPI00301202	MAGT1	MAGT1 magnesium transporter 1	179	41504	7	3	3	3	1.656
IPI00302850	SNRPD1	SNRPD1 Small nuclear ribonucleoprotein Sm D1	339	13273	6	4	1	1	1.66
IPI00009225	STX8	STX8 Syntaxin-8	709	26890	13	7	2	1	1.665
IPI00166865	CISD2	CISD2 CDGSH iron sulfur domain-containing protein	827	15268	22	6	2	2	1.689
IPI00395894	SYS1	SYS1 Protein SYS1 homolog	209	17603	2	1	1	1	1.697
IPI00007961	MT-ND1	MT-ND1 NADH-ubiquinone oxidoreductase chain 1	265	35637	7	5	2	2	1.701
IPI00032230	EPB41L3	EPB41L3 Isoform A of Band 4.1-like protein 3	112	120603	3	1	1	1	1.702
IPI00217007	KIAA2013	KIAA2013 Isoform 2 of Uncharacterized protein KIA/	205	72636	4	2	2	2	1.702
IPI00465361	RPL13	RPL13 60S ribosomal protein L13	2639	24247	79	8	5	4	1.71
IPI00033349	PREB	PREB Prolactin regulatory element-binding protein	40	45440	1	1	1	1	1.717
IPI00031458	ICMT	ICMT Protein-S-isoprenylcysteine O-methyltransfera	1068	31917	19	7	4	3	1.719
IPI00018415	TM9SF2	TM9SF2 Transmembrane 9 superfamily member 2	1211	75725	32	7	4	4	1.726
IPI00743716	HLA-C	HLA-C HLA class I histocompatibility antigen, Cw-12	3592	40860	25	6	3	2	1.753
IPI00291006	MDH2	MDH2 Malate dehydrogenase, mitochondrial	115	35481	3	1	2	2	1.755
IPI00328161	FKBP8	FKBP8 cDNA FLJ56370, highly similar to Homo sapi	1399	47142	24	5	4	4	1.76
IPI00003865	HSPA8	HSPA8 Isoform 1 of Heat shock cognate 71 kDa pro	11809	70854	233	8	19	16	1.773
IPI00465034	SLC25A26	SLC25A26 Isoform 1 of S-adenosylmethionine mitoc	289	29362	7	4	2	2	1.776
IPI00026240	BST1	BST1 ADP-ribosyl cyclase 2	304	35701	6	1	2	2	1.786
IPI00024642	CCDC47	CCDC47 Isoform 1 of Coiled-coil domain-containing	2973	55838	99	8	11	10	1.789
IPI00025329	RPL19	RPL19 60S ribosomal protein L19	235	23451	4	3	2	2	1.811
IPI00456758	RPL27A	RPL27A 60S ribosomal protein L27a	1533	16551	46	8	2	2	1.812
IPI00549761	ALG1	ALG1 Chitobiosyldiphosphodolichol beta-mannosyltr	3044	52484	39	8	2	2	1.812
IPI00063544	YIF1B	YIF1B Isoform 3 of Protein YIF1B	602	33943	13	5	1	1	1.814
IPI00019141	AGPAT1	AGPAT1 1-acyl-sn-glycerol-3-phosphate acyltransfer	1626	31696	21	7	3	1	1.818
IPI00000115	CNIH4	CNIH4 Isoform 1 of Protein cornichon homolog 4	1257	16082	22	8	1	1	1.839
IPI00029046	MLEC	MLEC Malectin	1234	32214	40	8	4	4	1.84
IPI00844578	DHX9	DHX9 ATP-dependent RNA helicase A	204	140869	7	4	1	1	1.84
IPI00334579	MRPL43	MRPL43 cDNA FLJ61386, highly similar to Homo sa	86	28658	2	2	2	2	1.842
IPI00452747	LOC653566	LOC653566 Similar to Signal peptidase complex sub	2468	24960	67	8	6	6	1.842

Data1. AccessionNo (IPI)	iPS_CFB50/ HFF	iPS_CFB46/ H9ES	iPS_CFB50/ H9ES	iPS_CFB46/N TU1ES	iPS_CFB50/N TU1ES	H9ES/ HFF	NTU1ES/ HFF
IPI00030106	1.886	1.016	1.247	9999	9999	1.532	-9999
IPI00413784	3.051	0.186	0.368	0.409	0.802	8.411	3.726
IPI00306516	0.991	1.209	0.775	0.866	0.69	1.295	1.406
IPI00217030	1.384	1.311	1.175	0.98	0.87	1.19	1.NaN
IPI00032825	1.999	0.825	1.051	0.717	0.814	1.937	2.3
IPI00216049	1.22	0.721	0.639	0.84	0.601	2.607	1.64
IPI00289819	1.235	1.123	0.991	1.183	1.034	0.831	0.803
IPI00003925	-9999	0.952	-9999	1.315	-9999	1.658	1.17
IPI00002732	1.637	0.828	0.859	1.607	1.651	1.931	0.971
IPI00024143	2.089	0.885	1.557	0.436	0.969	1.454	2.567
IPI00003926	1.946	0.714	0.874	0.865	1.048	2.256	1.817
IPI00334282	1.511	1.121	1.056	1.161	1.082	1.423	1.45
IPI00027434	1.516	1.04	0.988	0.827	0.777	1.308	1.909
IPI00008167	1.751	1.278	1.391	0.607	0.779	1.273	2.194
IPI00009865	1.318	1.283	1.028	0.837	0.653	1.277	2.031
IPI00019359	1.426	1.11	0.977	0.828	0.7	1.536	1.904
IPI00301202	2.225	0.9	1.303	0.575	0.825	1.772	2.703
IPI00302850	2.427	0.568	0.852	0.46	0.683	2.886	3.48
IPI00009225	1.257	1.701	1.317	1.067	0.818	0.967	1.504
IPI00166865	2.115	1.073	1.376	0.77	0.977	1.NaN	2.112
IPI00395894	1.017	1.052	0.647	1.716	1.044	1.593	0.953
IPI00007961	1.345	1.164	0.944	0.749	0.602	1.457	2.204
IPI00032230	0.893	0.861	0.464	2.191	1.167	1.952	0.749
IPI00217007	1.344	0.906	0.798	1.013	0.87	1.633	1.591
IPI00465361	1.459	1.462	1.28	1.134	0.988	1.16	1.447
IPI00033349	1.882	1.033	1.162	1.08	1.203	1.641	1.532
IPI00031458	2.264	0.914	1.239	0.841	0.996	1.842	2.289
IPI00018415	1.424	1.358	1.153	1.587	1.335	1.249	1.031
IPI00743716	2.523	1.7	2.502	1.324	1.904	0.79	1.327
IPI00291006	2.392	0.689	1.52	1.052	2.605	1.667	1.609
IPI00328161	0.493	1.856	0.565	2.46	0.764	0.921	0.623
IPI00003865	1.192	1.38	0.956	1.774	1.305	1.231	0.871
IPI00465034	1.643	0.923	0.986	1.012	1.07	1.831	1.393
IPI00026240	1.142	1.042	0.684	9999	9999	1.693	-9999
IPI00024642	1.893	1.4	1.451	1.337	1.448	1.324	1.289
IPI00025329	1.431	1.318	1.068	1.148	0.921	1.357	1.521
IPI00456758	1.733	1.144	1.172	0.833	0.845	1.578	1.896
IPI00549761	2.105	1.346	1.591	1.272	1.492	1.35	1.37
IPI00063544	1.57	1.557	1.382	1.159	1.018	1.151	1.509
IPI00019141	2.339	1.121	1.377	1.21	1.517	1.711	1.451
IPI00000115	1.855	0.968	1.001	0.707	0.724	1.877	2.507
IPI00029046	2.107	1.199	1.527	0.859	1.083	1.43	1.997
IPI00844578	2.167	0.67	0.81	0.671	0.803	2.711	2.643
IPI00334579	-9999	1.236	-9999	9999	NaN	1.352	-9999
IPI00452747	2.15	1.139	1.46	0.784	0.995	1.534	2.145

Table S1 Identified proteins in the human membrane proteome of hiPSCs, hESCs and somatic foreskin fibroblast HFF.
Profiles of identified proteins in MS Experiment 1

Data1. AccessionNo (IPI)	Gene Symbol	Description	Score	Mass	# of identified (spectrum)	# of samples identified	Total peptides	# of peptides used	iPS_CFB46 /HFF
IPI00847300	-	- Similar to Voltage-dependent anion-selective chanr	6978	27715	5	3	2	1	1.846
IPI00020005	ARSE	ARSE cDNA FLJ50239, highly similar to Arylsulfatas	912	68113	16	6	1	1	1.847
IPI00005751	SPTLC2	SPTLC2 Serine palmitoyltransferase 2	392	62884	9	5	2	2	1.852
IPI00004669	GALNT2	GALNT2 Polypeptide N-acetylgalactosaminyltransfer	495	64691	15	1	7	7	1.856
IPI00306048	ATAD3B	ATAD3B Isoform 1 of ATPase family AAA domain-cc	2894	72527	1	1	1	1	1.869
IPI00062469	TMEM49	TMEM49 Transmembrane protein 49	872	46208	10	5	1	1	1.875
IPI00022275	SACM1L	SACM1L Phosphatidylinositide phosphatase SAC1	2612	66924	66	7	11	11	1.891
IPI00032150	CDS2	CDS2 Isoform 1 of Phosphatidate cytidylyltransfera	583	51384	12	6	2	2	1.894
IPI00171626	LPCAT1	LPCAT1 Lysophosphatidylcholine acyltransferase 1	2316	59113	70	6	7	7	1.905
IPI00026824	HMOX2	HMOX2 Heme oxygenase 2	1033	41643	21	6	5	5	1.912
IPI00009507	SYPL1	SYPL1 Isoform 1 of Synaptophysin-like protein 1	897	28547	21	8	1	1	1.913
IPI00016405	OCIAD1	OCIAD1 Isoform 1 of OCIA domain-containing protei	2107	27609	44	6	5	4	1.921
IPI00455457	=-	#NAME?	47	15421	3	3	2	2	1.927
IPI00074330	TMEM48	TMEM48 Isoform 3 of Nucleoporin NDC1	340	63531	9	4	2	2	1.931
IPI00478838	FAR1	FAR1 Fatty acyl-CoA reductase 1	645	59319	16	5	4	4	1.932
IPI00152938	TOMM40L	TOMM40L Mitochondrial import receptor subunit TOI	98	33895	2	2	1	1	1.934
IPI00642244	KIAA0090	KIAA0090 Isoform 4 of Uncharacterized protein KIA/	3536	109352	77	8	14	13	1.94
IPI00847342	KRT7	KRT7 keratin 7	325	51354	7	1	3	3	1.951
IPI00021985	TM9SF4	TM9SF4 Isoform 1 of Transmembrane 9 superfamily	853	74470	27	8	4	4	1.961
IPI00306419	LPCAT3	LPCAT3 Lysophospholipid acyltransferase 5	79	55998	2	2	2	2	1.961
IPI00794229	SPCS1	SPCS1 9 kDa protein	408	8565	10	5	3	2	1.963
IPI00006482	ATP1A1	ATP1A1 Isoform Long of Sodium/potassium-transpo	20954	112824	441	8	27	22	1.984
IPI00739274	LOC652614	LOC652614 similar to MHC class I antigen	614	41190	4	3	1	1	1.991
IPI00060569	ABHD12	ABHD12 Isoform 2 of Monoacylglycerol lipase ABHD	1817	45529	38	7	4	4	1.993
IPI00299084	TMEM33	TMEM33 Transmembrane protein 33	1346	27960	40	8	4	4	1.996
IPI00019899	SC4MOL	SC4MOL C-4 methylsterol oxidase	712	35193	15	5	2	2	2.01
IPI00291755	NUP210	NUP210 Isoform 1 of Nuclear pore membrane glyco	719	204983	17	2	6	6	2.019
IPI00418497	TIMM50	TIMM50 Isoform 2 of Mitochondrial import inner mem	1381	50433	21	6	4	4	2.022
IPI00295772	CYP51A1;LOC40	CYP51A1;LOC401387 cytochrome P450, family 51,	2875	57242	60	7	6	6	2.036
IPI00007765	HSPA9	HSPA9 Stress-70 protein, mitochondrial	4108	73635	88	8	12	11	2.045
IPI00004671	GOLGB1	GOLGB1 Golgin subfamily B member 1	280	375790	10	1	5	4	2.062
IPI00294159	SLC25A1	SLC25A1 Tricarboxylate transport protein, mitochonc	7665	33991	140	8	9	7	2.062
IPI00030847	TM9SF3	TM9SF3 Transmembrane 9 superfamily member 3	1966	67843	37	7	3	2	2.086
IPI00023526	RAB6A	RAB6A Isoform 1 of Ras-related protein Rab-6A	3473	23578	60	8	5	5	2.093
IPI00000138	MGAT1	MGAT1 Alpha-1,3-mannosyl-glycoprotein 2-beta-N-a	771	50830	16	6	3	3	2.095
IPI00146447	VMA21	VMA21 Vacuolar ATPase assembly integral membra	79	11347	2	2	1	1	2.111
IPI00026202	RPL18A	RPL18A 60S ribosomal protein L18a	261	20749	17	7	2	2	2.112
IPI00386258	MTCH1	MTCH1 Isoform 1 of Mitochondrial carrier homolog 1	2937	41517	75	8	7	6	2.14
IPI00028491	AGPAT5	AGPAT5 1-acyl-sn-glycerol-3-phosphate acyltransfer	381	42045	5	4	1	1	2.141
IPI00010255	RHBDD2	RHBDD2 Rhomboid domain-containing protein 2	44	39177	1	1	1	1	2.145
IPI00005745	SPTLC1	SPTLC1 Serine palmitoyltransferase 1	118	52710	3	3	3	2	2.149
IPI00042580	APOO	APOO Isoform 1 of Apolipoprotein O	1294	22271	37	6	4	4	2.158
IPI00002948	LIN28	LIN28 Lin-28 homolog A	62	22728	9	6	1	1	2.174
IPI00328391	GALNT7	GALNT7 N-acetylgalactosaminyltransferase 7	1442	75341	47	7	5	5	2.176
IPI00220739	PGRMC1	PGRMC1 Membrane-associated progesterone recep	5838	21658	88	8	7	7	2.177

Data1. AccessionNo (IPI)	iPS_CFB50/ HFF	iPS_CFB46/ H9ES	iPS_CFB50/ H9ES	iPS_CFB46/N TU1ES	iPS_CFB50/N TU1ES	H9ES/ HFF	NTU1ES/ HFF
IPI00847300	1.516	2.282	1.924	2.542	2.124	0.799	0.7
IPI00020005	2.684	0.577	0.86	1.412	2.085	3.163	1.26
IPI00005751	1.82	1.544	1.632	1.06	1.114	0.991	1.747
IPI00004669	1.3	1.379	0.98	2.116	1.489	1.339	0.857
IPI00306048	1.825	0.982	0.984	9999	9999	1.88	-9999
IPI00062469	1.793	1.02	1.001	0.965	0.938	1.816	1.873
IPI00022275	2.506	1.002	1.247	0.866	1.083	3.411	2.159
IPI00032150	2.131	1.126	1.199	0.999	1.054	1.678	1.744
IPI00171626	2.126	1.182	1.321	0.891	1.018	1.847	2.176
IPI00026824	1.722	0.862	0.788	1.151	1.008	2.177	1.774
IPI00009507	-9999	1.292	-9999	9999	NaN	1.462	-9999
IPI00016405	1.668	1.223	1.026	1.003	0.833	2.365	2.892
IPI00455457	1.424	0.178	0.298	1.545	2.564	2.081	0.234
IPI00074330	5.855	1.098	1.554	0.672	0.961	1.739	5.966
IPI00478838	1.435	1.313	1.003	1.823	1.419	1.404	1.614
IPI00152938	2.365	0.419	0.525	0.567	0.704	4.561	3.286
IPI00642244	2.007	1.223	1.269	0.91	0.987	1.611	2.186
IPI00847342	2.596	0.96	1.365	0.54	0.76	2.016	3.498
IPI00021985	2.179	0.991	0.998	0.879	0.877	1.907	2.264
IPI00306419	1.921	1.496	1.504	9999	9999	1.295	-9999
IPI00794229	2.067	1.54	1.673	0.743	0.798	1.256	2.541
IPI00006482	2.039	1.078	1.154	0.766	0.807	1.828	2.501
IPI00739274	2.297	1.131	1.338	0.709	0.83	1.739	2.708
IPI00060569	2.678	1.182	1.618	1.009	1.367	1.645	1.889
IPI00299084	2.307	1.049	1.036	0.887	0.867	1.879	2.17
IPI00019899	2.195	1.264	1.449	0.863	0.977	1.939	2.234
IPI00291755	4.497	1.078	2.81	0.359	0.929	0.905	4.457
IPI00418497	2.085	0.844	0.896	0.853	0.9	2.382	2.273
IPI00295772	2.096	1.05	1.1	1.382	1.434	1.745	1.412
IPI00007765	1.095	1.126	0.635	2.964	1.285	1.72	0.846
IPI00004671	1.644	1.278	1.064	0.907	0.767	1.595	2.114
IPI00294159	2.08	0.922	0.983	0.753	0.775	2.209	2.626
IPI00030847	1.484	0.927	0.633	1.501	1.091	2.231	1.332
IPI00023526	1.475	1.895	1.399	1.136	0.817	1.213	1.904
IPI00000138	1.58	1.155	0.903	1.256	0.974	1.776	1.58
IPI00146447	1.307	1.043	0.663	9999	9999	1.999	-9999
IPI00026202	1.674	1.249	1.015	1.054	0.847	1.673	1.939
IPI00386258	1.679	1.062	0.913	0.945	0.813	2.164	2.051
IPI00028491	2.329	0.221	0.246	0.887	0.98	9.585	2.326
IPI00010255	4.759	0.499	1.135	2.56	5.767	4.248	0.808
IPI00005745	1.949	1.353	1.335	1.031	1.007	1.534	1.87
IPI00042580	NaN	0.674	0.676	0.605	0.571	3.872	2.602
IPI00002948	1.72	1.155	0.937	1.27	1.02	1.86	1.65
IPI00328391	1.501	1.109	0.839	1.816	1.333	1.89	0.912
IPI00220739	2.221	1.365	1.485	0.694	0.754	1.548	2.707

Table S1 Identified proteins in the human membrane proteome of hiPSCs, hESCs and somatic foreskin fibroblast HFF.
Profiles of identified proteins in MS Experiment 1

Data1. AccessionNo (IPI)	Gene Symbol	Description	Score	Mass	# of identified (spectrum)	# of samples identified	Total peptides	# of peptides used	iPS_CFB46 /HFF
IPI00339384	RDH11	RDH11 Isoform 1 of Retinol dehydrogenase 11	2054	35363	49	7	5	5	2.179
IPI00329301	NDUFA11	NDUFA11 Isoform 1 of NADH dehydrogenase [ubiqu	393	14843	13	6	1	1	2.198
IPI00003802	MAN2A1	MAN2A1 Alpha-mannosidase 2	5611	131057	113	7	13	13	2.201
IPI00017510	MT-CO2	MT-CO2 Cytochrome c oxidase subunit 2	6223	25548	105	8	7	4	2.202
IPI00479145	KRT19	KRT19 Keratin, type I cytoskeletal 19	485	44065	9	2	4	4	2.215
IPI00298237	TPP1	TPP1 cDNA FLJ56402, highly similar to Tripeptidyl-p	495	62173	6	2	1	1	2.216
IPI00219685	NDUFA13;YJEFN	NDUFA13;YJEFN3 NADH dehydrogenase (ubiquino	2236	25804	66	8	6	6	2.243
IPI00027448	ATP5L	ATP5L ATP synthase subunit g, mitochondrial	1876	11421	63	7	6	5	2.251
IPI00063130	TMEM205	TMEM205 Transmembrane protein 205	835	21184	31	7	3	3	2.277
IPI00232571	GPC4	GPC4 Glypican-4	1982	62372	43	6	7	6	2.285
IPI00019353	AGK	AGK Isoform 1 of Acylglycerol kinase, mitochondrial	1862	47107	20	8	1	1	2.294
IPI00328243	PLD3	PLD3 Phospholipase D3	687	54671	24	5	4	4	2.314
IPI00008998	PTPLAD1	PTPLAD1 Protein tyrosine phosphatase-like protein I	4948	43132	99	8	9	8	2.316
IPI00025086	COX5A	COX5A Cytochrome c oxidase subunit 5A, mitochon	1708	16752	48	6	5	5	2.329
IPI00642329	MFN2	MFN2 Isoform 1 of Mitofusin-2	86	86347	1	1	1	1	2.33
IPI00291417	DCAKD	DCAKD Isoform 1 of Dephospho-CoA kinase domair	339	26533	17	6	2	2	2.332
IPI00303954	CYB5B	CYB5B cytochrome b5 outer mitochondrial membrar	1731	16684	47	8	5	4	2.344
IPI00292135	LBR	LBR Lamin-B receptor	1445	70658	28	6	3	3	2.347
IPI00015920	SLC25A10	SLC25A10 Isoform 1 of Mitochondrial dicarboxylate (	560	31262	7	2	2	2	2.372
IPI00377145	KCNN2	KCNN2 small conductance calcium-activated potass	161	26325	3	3	1	1	2.376
IPI00007309	LOC100287932;T	LOC100287932;TIMM23 Mitochondrial import inner r	1561	21929	40	6	6	5	2.384
IPI00074489	NDUFB10	NDUFB10 NDUFB10 protein	449	20130	19	5	5	4	2.456
IPI00012772	RPL8	RPL8 60S ribosomal protein L8	1274	28007	56	8	5	4	2.478
IPI00334190	STOML2	STOML2 Stomatin-like protein 2	6576	38510	101	8	9	8	2.503
IPI00021338	DLAT	DLAT Dihydrolipoyllysine-residue acetyltransferase c	1558	68953	34	7	5	5	2.52
IPI00171445	ATAD1	ATAD1 Isoform 1 of ATPase family AAA domain-cor	970	40718	18	7	1	1	2.523
IPI00216308	VDAC1	VDAC1 Voltage-dependent anion-selective channel p	11731	30754	234	8	13	10	2.524
IPI00099463	SGPL1	SGPL1 Sphingosine-1-phosphate lyase 1	1834	63483	39	7	5	5	2.526
IPI00000425	ATP6V0A2	ATP6V0A2 V-type proton ATPase 116 kDa subunit a	87	98018	1	1	1	1	2.568
IPI00219729	SLC25A11	SLC25A11 Mitochondrial 2-oxoglutarate/malate carri	2640	34040	81	8	9	8	2.597
IPI00003833	MTCH2	MTCH2 Mitochondrial carrier homolog 2	2492	33309	56	8	5	4	2.598
IPI00291467	SLC25A6	SLC25A6 ADP/ATP translocase 3	9417	32845	367	8	16	12	2.63
IPI00646963	REEP6	REEP6 Receptor accessory protein 6, isoform CRA_	664	23403	18	8	1	1	2.648
IPI00009368	SFXN1	SFXN1 Sideroflexin-1	NaN4	35596	123	8	11	10	2.665
IPI00031821	ITM2B	ITM2B Integral membrane protein 2B	57	30318	1	1	1	1	2.665
IPI00031804	VDAC3	VDAC3 Isoform 1 of Voltage-dependent anion-select	4449	30639	48	7	5	3	2.676
IPI00294250	EPHA1	EPHA1 Ephrin type-A receptor 1	63	107998	1	1	1	1	2.692
IPI00166785	MMGT1	MMGT1 Isoform 2 of Membrane magnesium transpo	79	21867	1	1	1	1	2.7
IPI00016968	SDHC	SDHC Succinate dehydrogenase cytochrome b560 s	248	18598	6	5	1	1	2.712
IPI00374975	PGAM4	PGAM4 Probable phosphoglycerate mutase 4	253	28759	8	3	2	2	2.716
IPI00307572	TMEM165	TMEM165 Transmembrane protein 165	1504	34883	30	7	4	4	2.721
IPI00031064	TMEM126A	TMEM126A Transmembrane protein 126A	778	21513	15	6	2	2	2.724
IPI00028946	RTN3	RTN3 Isoform 3 of Reticulon-3	779	25593	33	7	2	1	2.754
IPI00001754	F11R	F11R Junctional adhesion molecule A	1454	32562	30	6	2	2	2.773
IPI00414717	GLG1	GLG1 Isoform 2 of Golgi apparatus protein 1	6109	137132	109	8	9	8	2.793

Data1. AccessionNo (IPI)	iPS_CFB50/ HFF	iPS_CFB46/ H9ES	iPS_CFB50/ H9ES	iPS_CFB46/N TU1ES	iPS_CFB50/N TU1ES	H9ES/ HFF	NTU1ES/ HFF
IPI00339384	2.974	1.127	1.628	0.801	1.12	1.693	2.58
IPI00329301	1.897	1.168	1.034	9999	9999	1.859	-9999
IPI00003802	1.488	1.124	0.864	1.93	1.433	2.093	1.352
IPI00017510	2.107	1.318	1.187	0.892	0.841	1.662	2.676
IPI00479145	-9999	4.628	-9999	1.278	-9999	0.473	1.67
IPI00298237	2.992	0.614	0.85	1.892	2.594	3.564	1.129
IPI00219685	2.107	1.068	1.012	0.806	0.756	2.136	2.737
IPI00027448	2.281	1.037	1.03	0.682	0.696	2.162	5.188
IPI00063130	3.089	1.155	1.601	0.677	0.927	1.936	3.286
IPI00232571	1.943	0.524	0.469	0.882	0.793	7.801	1.97
IPI00019353	2.117	0.992	0.939	0.937	0.878	2.285	2.359
IPI00328243	1.681	1.393	1.121	1.45	1.155	1.223	1.001
IPI00008998	3.269	1.059	1.498	0.759	1.059	2.131	3.093
IPI00025086	2.008	0.861	0.696	0.717	0.603	2.655	3.454
IPI00642329	2.032	0.979	0.876	0.872	0.772	2.35	2.576
IPI00291417	2.843	0.9	1.197	0.55	0.728	2.797	2.297
IPI00303954	2.516	0.939	0.966	0.905	0.921	2.413	2.729
IPI00292135	4.137	0.837	1.51	0.438	0.779	2.76	5.169
IPI00015920	2.108	1.613	1.248	0.954	0.732	1.978	2.813
IPI00377145	1.096	1.411	0.667	1.234	0.578	1.663	1.855
IPI00007309	1.838	0.97	0.78	0.98	0.862	5.392	2.218
IPI00074489	2.535	1.172	1.066	0.907	0.815	2.677	2.612
IPI00012772	1.725	1.51	1.103	1.371	0.932	1.579	1.815
IPI00334190	1.989	1.382	1.129	1.091	0.884	1.857	2.326
IPI00021338	0.951	1.312	0.513	1.934	0.688	1.898	1.327
IPI00171445	3.079	0.61	0.763	0.795	0.985	4.087	3.06
IPI00216308	2.379	1.218	1.172	0.852	0.811	2.064	2.896
IPI00099463	2.03	1.213	1.029	1.204	1.162	2.012	1.423
IPI00000425	1.849	1.102	0.814	1.085	0.794	2.302	2.281
IPI00219729	2.597	0.739	0.766	0.929	0.938	3.622	2.667
IPI00003833	2.225	1.091	0.993	0.898	0.807	2.248	2.755
IPI00291467	2.755	1.086	1.182	0.859	0.919	2.565	3.068
IPI00646963	3.687	1.078	1.54	0.967	1.368	2.425	2.638
IPI00009368	2.444	1.111	0.986	0.991	0.894	2.638	2.606
IPI00031821	1.909	2.016	1.481	2.375	1.727	1.306	1.082
IPI00031804	2.649	1.275	1.238	0.704	0.751	2.112	3.577
IPI00294250	1.894	1.507	1.088	0.931	0.665	1.764	2.787
IPI00166785	3.546	0.37	0.498	0.741	0.988	7.212	3.514
IPI00016968	2.64	0.753	0.753	0.781	0.772	3.NaN	3.349
IPI00374975	6.184	1.299	1.853	0.906	1.216	0.644	2.017
IPI00307572	2.061	1.25	0.996	1.952	1.527	2.091	1.321
IPI00031064	1.564	1.055	0.621	1.025	0.597	2.551	2.563
IPI00028946	2.275	1.23	1.041	1.135	0.951	2.212	2.342
IPI00001754	3.005	1.03	1.11	0.605	0.646	2.791	4.197
IPI00414717	2.674	1.089	1.067	1.489	1.359	2.875	1.85

Table S1 Identified proteins in the human membrane proteome of hiPSCs, hESCs and somatic foreskin fibroblast HFF.
Profiles of identified proteins in MS Experiment 1

Data1. AccessionNo (IPI)	Gene Symbol	Description	Score	Mass	# of identified (spectrum)	# of samples identified	Total peptides	# of peptides used	iPS_CFB46 /HFF
IPI00009680	MRPL44	MRPL44 39S ribosomal protein L44, mitochondrial	465	37512	14	5	1	1	2.8
IPI00017895	GPD2	GPD2 Isoform 1 of Glycerol-3-phosphate dehydroge	4837	80802	96	7	12	10	2.827
IPI00013744	ITGA2	ITGA2 Integrin alpha-2	419	129214	8	1	3	3	2.88
IPI00032370	ALG8	ALG8 Probable dolichyl pyrophosphate Glc1Man9Gl	269	60048	10	6	1	1	2.917
IPI00009960	IMMT	IMMT Isoform 1 of Mitochondrial inner membrane pr	7613	83626	184	7	21	18	2.973
IPI00329331	UGP2	UGP2 Isoform 1 of UTP--glucose-1-phosphate uridyl	467	56905	14	6	3	3	2.998
IPI00029133	ATP5F1	ATP5F1 ATP synthase subunit b, mitochondrial	7423	28890	202	8	15	12	3.002
IPI00549970	GHITM	GHITM Growth hormone inducible transmembrane p	236	35259	8	5	2	2	3.005
IPI00171573	CCDC109A	CCDC109A Isoform 1 of Coiled-coil domain-containi	2092	39842	29	8	3	3	3.025
IPI00305383	UQCRC2	UQCRC2 Cytochrome b-c1 complex subunit 2, mitoc	7332	48413	139	8	16	10	3.035
IPI00464968	MT-CO1	MT-CO1 Cytochrome c oxidase subunit 1	90	57346	2	2	1	1	3.054
IPI00329410	DOLPP1	DOLPP1 Dolichyldiphosphatase 1	917	27013	20	7	1	1	3.076
IPI00554788	KRT18	KRT18 Keratin, type I cytoskeletal 18	664	48029	17	1	6	6	3.086
IPI00219291	ATP5J2	ATP5J2 Isoform 2 of ATP synthase subunit f, mitoch	2843	11357	80	8	3	2	3.098
IPI00007979	MT-ND2	MT-ND2 NADH-ubiquinone oxidoreductase chain 2	187	38935	9	6	2	2	3.131
IPI00020510	CISD1	CISD1 CDGSH iron sulfur domain-containing protein	871	12191	13	6	1	1	3.147
IPI00019906	BSG	BSG Isoform 2 of Basigin	11069	29203	198	8	13	7	3.167
IPI00183786	FADS2	FADS2 Isoform 1 of Fatty acid desaturase 2	921	52226	27	5	5	4	3.189
IPI00788624	SLC25A3	SLC25A3 31 kDa protein	7941	30503	200	8	14	9	3.207
IPI00171459	HSDL1	HSDL1 Hydroxysteroid dehydrogenase-like protein 1	385	36994	9	5	2	2	3.208
IPI00017592	LETM1	LETM1 Isoform 1 of LETM1 and EF-hand domain-co	1320	83302	41	6	7	7	3.241
IPI00024145	VDAC2	VDAC2 Isoform 2 of Voltage-dependent anion-select	6805	30393	154	8	9	8	3.241
IPI00026964	UQCRFS1	UQCRFS1 Cytochrome b-c1 complex subunit Riesk	4422	29649	91	8	7	6	3.304
IPI00218848	ATP5I	ATP5I ATP synthase, H+ transporting, mitochondrial	512	8302	26	6	3	3	3.32
IPI00220194	SLC2A1	SLC2A1 Solute carrier family 2, facilitated glucose tr	3514	54049	98	8	8	6	3.346
IPI00248911	GNAT3	GNAT3 Guanine nucleotide-binding protein G(t) sub	683	40331	3	3	1	1	3.452
IPI00295992	ATAD3A	ATAD3A Isoform 2 of ATPase family AAA domain-co	4304	66177	126	8	13	13	3.454
IPI00029558	NDUFC2	NDUFC2 NADH dehydrogenase [ubiquinone] 1 subu	273	14178	16	5	2	2	3.487
IPI00294501	DHCR7	DHCR7 7-dehydrocholesterol reductase	2003	54454	29	7	2	2	3.518
IPI00216587	RPS8	RPS8 40S ribosomal protein S8	770	24190	28	8	3	3	3.531
IPI00220281	GNAO1	GNAO1 Isoform Alpha-1 of Guanine nucleotide-bindi	1600	40025	1	1	1	1	3.578
IPI00554701	UCRC	UCRC Cytochrome b-c1 complex subunit 9	789	7304	26	6	3	2	3.588
IPI00419916	ALPL	ALPL Alkaline phosphatase, tissue-nonspecific isozy	3474	57269	82	6	9	9	3.591
IPI00395887	TMX1	TMX1 Thioredoxin-related transmembrane protein 1	1775	31771	56	6	5	4	3.592
IPI00300971	ALG6	ALG6 Asparagine-linked glycosylation 6 homolog	296	58309	9	4	2	2	3.606
IPI00000980	TOMM7	TOMM7 Mitochondrial import receptor subunit TOM7	39	6244	1	1	1	1	3.611
IPI00554711	JUP	JUP Junction plakoglobin	163	81693	5	3	2	2	3.616
IPI00419869	COX15	COX15 Isoform 1 of Cytochrome c oxidase assembl	432	46000	12	6	1	1	3.662
IPI00062151	=-	#NAME?	38	23755	1	1	1	1	3.667
IPI00100656	TECR	TECR Isoform 1 of Synaptic glycoprotein SC2	2615	36011	95	8	7	6	3.685
IPI00027493	LOC442497;SLC	LOC442497;SLC3A2 Isoform 2 of 4F2 cell-surface a	17080	57909	380	8	28	22	3.791
IPI00550382	SLC29A1	SLC29A1 Solute carrier family 29 (Nucleoside trans	253	58925	11	5	2	2	3.86
IPI00168921	WBSCR17	WBSCR17 Putative polypeptide N-acetylgalactosam	101	67708	2	1	1	1	3.911
IPI00473047	PRKAG1	PRKAG1 cDNA FLJ40287 fis, clone TESTI2027909,	62	38508	1	1	1	1	3.916
IPI00171542	NUP85	NUP85 Nucleoporin 85	120	74955	3	2	1	1	3.992

Data1. AccessionNo (IPI)	iPS_CFB50/ HFF	iPS_CFB46/ H9ES	iPS_CFB50/ H9ES	iPS_CFB46/N TU1ES	iPS_CFB50/N TU1ES	H9ES/ HFF	NTU1ES/ HFF
IPI00009680	-9999	1.179	-9999	9999	NaN	2.346	-9999
IPI00017895	2.214	1.048	0.834	1.274	0.971	2.795	1.827
IPI00013744	3.499	1.183	1.474	0.967	1.193	2.405	2.87
IPI00032370	2.953	0.577	0.599	0.85	0.875	4.995	3.306
IPI00009960	2.293	1.101	0.851	1.042	0.83	2.648	2.737
IPI00329331	1.192	1.748	0.697	3.697	1.156	1.661	0.981
IPI00029133	2.752	1.053	0.997	0.905	0.849	2.816	3.16
IPI00549970	2.66	0.8	0.714	0.801	0.706	3.701	3.566
IPI00171573	2.662	1.116	0.993	0.983	0.864	2.683	3.089
IPI00305383	2.869	1.259	1.189	0.928	0.87	2.322	3.217
IPI00464968	3.136	1.606	1.692	0.965	1.007	1.878	3.049
IPI00329410	3.428	1.341	1.533	0.769	0.871	2.266	3.853
IPI00554788	2.732	1.109	1.138	1.286	1.113	NaN	2.404
IPI00219291	2.669	1.159	1.021	0.777	0.68	2.695	3.847
IPI00007979	2.667	1.242	1.111	0.998	0.868	2.391	3.009
IPI00020510	3.881	0.953	1.205	0.883	1.105	3.264	3.437
IPI00019906	3.667	1.015	1.21	0.651	0.768	3.074	4.652
IPI00183786	3.106	1.149	1.165	1.155	1.188	3.292	2.448
IPI00788624	3.104	1.195	1.152	0.98	0.893	2.681	3.401
IPI00171459	2.953	0.628	0.593	0.659	0.616	5.049	4.691
IPI00017592	4.166	0.978	1.329	0.777	0.982	2.99	4.04
IPI00024145	3.368	1.165	1.241	0.847	0.891	2.856	3.801
IPI00026964	3.634	0.565	0.583	1.212	1.199	8.807	2.949
IPI00218848	2.751	1.114	0.989	1.003	0.886	2.782	3.248
IPI00220194	5.639	1.01	2.051	0.425	0.898	3.446	6.591
IPI00248911	2.528	1.43	1.075	1.488	1.107	2.384	2.235
IPI00295992	2.903	1.135	0.973	0.837	0.678	3.353	3.634
IPI00029558	3.146	1.313	1.215	0.702	0.644	2.623	4.785
IPI00294501	3.735	1.195	1.371	0.962	1.093	2.617	3.627
IPI00216587	3.045	1.207	1.047	1.059	0.91	2.793	3.33
IPI00220281	2.568	1.326	0.977	0.826	0.602	2.665	4.175
IPI00554701	3.557	0.999	0.992	0.636	0.626	3.459	6.124
IPI00419916	2.214	1.337	1.172	0.855	0.784	1.949	2.27
IPI00395887	3.046	1.35	1.284	0.925	0.803	2.325	3.697
IPI00300971	2.436	1.074	1.786	0.647	1.06	2.801	3.488
IPI00000980	2.231	1.024	0.649	1.187	0.745	3.483	2.932
IPI00554711	1.356	1.078	0.611	1.536	0.585	2.413	2.269
IPI00419869	2.713	0.884	0.672	1.621	1.22	4.093	2.178
IPI00062151	3.788	9999	9999	0.761	0.799	-9999	4.643
IPI00100656	4.082	1.509	1.886	0.975	1.225	1.914	4
IPI00027493	2.698	1.162	0.842	0.933	0.675	3.236	4.133
IPI00550382	4.291	0.941	1.068	0.696	0.782	4.034	5.314
IPI00168921	1.205	1.421	0.449	9999	9999	2.718	-9999
IPI00473047	3.307	1.201	1.04	3.425	2.938	3.221	1.102
IPI00171542	1.76	0.578	0.261	0.859	0.384	6.823	4.481

Table S1 Identified proteins in the human membrane proteome of hiPSCs, hESCs and somatic foreskin fibroblast HFF.
Profiles of identified proteins in MS Experiment 1

Data1. AccessionNo (IPI)	Gene Symbol	Description	Score	Mass	# of identified (spectrum)	# of samples identified	Total peptides	# of peptides used	iPS_CFB46 /HFF
IPI00014053	TOMM40	TOMM40 Isoform 1 of Mitochondrial import receptor	7884	37869	154	8	13	8	4.025
IPI00008599	EBP	EBP 3-beta-hydroxysteroid-Delta(8),Delta(7)-isomera	497	26336	20	7	2	1	4.064
IPI00007928	PRPF8	PRPF8 Pre-mRNA-processing-splicing factor 8	52	273427	6	4	2	2	4.076
IPI00220487	ATP5H	ATP5H Isoform 1 of ATP synthase subunit d, mitoch	4125	18480	101	8	9	7	4.077
IPI00006579	COX4I1	COX4I1 Cytochrome c oxidase subunit 4 isoform 1, r	3051	19564	108	7	9	7	4.093
IPI00007183	ATL2	ATL2 Isoform 2 of Atlastin-2	206	66045	8	3	1	1	4.141
IPI00396661	CYP2S1	CYP2S1 Isoform 1 of Cytochrome P450 2S1	7394	55781	204	6	19	14	4.15
IPI00007188	SLC25A5	SLC25A5 ADP/ATP translocase 2	8350	32874	73	8	3	3	4.187
IPI00016703	DHCR24	DHCR24 24-dehydrocholesterol reductase	2613	60062	65	7	8	7	4.207
IPI00914855	MTX1	MTX1 Isoform 3 of Metaxin-1	1379	35754	24	6	2	2	4.22
IPI00030911	VAMP8	VAMP8 Vesicle-associated membrane protein 8	302	11431	9	4	1	1	4.228
IPI00024976	TOMM22	TOMM22 Mitochondrial import receptor subunit TOM	3374	15512	70	8	6	5	4.388
IPI00027252	PHB2	PHB2 Prohibitin-2	14142	33276	337	8	15	13	4.423
IPI00017334	PHB	PHB Prohibitin	15907	29786	307	8	17	13	4.522
IPI00015833	CHCHD3	CHCHD3 Coiled-coil-helix-coiled-coil-helix domain-cc	1215	26136	39	6	4	4	4.619
IPI00013475	TUBB2A	TUBB2A Tubulin beta-2A chain	15436	49875	12	6	2	2	4.642
IPI00011770	NDUFA4	NDUFA4 NADH dehydrogenase [ubiquinone] 1 alpha	211	9364	11	6	1	1	4.664
IPI00293476	MRPL23	MRPL23 39S ribosomal protein L23, mitochondrial	51	17770	1	1	1	1	4.694
IPI00024742	UQCRQ	UQCRQ Cytochrome b-c1 complex subunit 8	101	9900	2	1	1	1	4.717
IPI00033217	AASS	AASS Alpha-aminoadipic semialdehyde synthase, m	4411	102066	96	6	19	17	5.014
IPI00171411	GOLM1	GOLM1 Golgi membrane protein 1	529	46245	20	6	2	2	5.019
IPI00024650	SLC16A1	SLC16A1 Monocarboxylate transporter 1	5175	53923	97	7	8	6	5.073
IPI00219385	NDUFB6	NDUFB6 NADH dehydrogenase [ubiquinone] 1 beta	888	15479	23	6	2	2	5.132
IPI00465059	RHOT2	RHOT2 Isoform 1 of Mitochondrial Rho GTPase 2	939	68075	30	6	6	6	5.274
IPI00016014	ITM2C	ITM2C Isoform 1 of Integral membrane protein 2C	1114	30204	22	6	3	3	5.449
IPI00015602	TOMM70A	TOMM70A Mitochondrial import receptor subunit TO	2412	67412	72	7	6	5	5.494
IPI00063903	USMG5	USMG5 Up-regulated during skeletal muscle growth	1450	6453	36	6	2	2	5.9
IPI00007084	SLC25A13	SLC25A13 Calcium-binding mitochondrial carrier pro	12435	74129	276	6	22	20	6.133
IPI00023406	HCCS	HCCS Cytochrome c-type heme lyase	278	30582	12	5	1	1	6.219
IPI00470360	KIRREL	KIRREL Isoform 1 of Kin of IRRE-like protein 1	92	83484	1	1	1	1	6.345
IPI00218487	GJA1	GJA1 Gap junction alpha-1 protein	2931	42981	51	6	6	5	6.729
IPI00021842	APOE	APOE Apolipoprotein E	3510	36132	90	6	8	8	7.212
IPI00003909	SLC2A3	SLC2A3 Solute carrier family 2, facilitated glucose tr	3450	53889	84	6	7	6	7.341
IPI00218565	GPR143	GPR143 G protein-coupled receptor 143	452	46020	8	4	1	1	7.496
IPI00295698	SLC7A3	SLC7A3 Cationic amino acid transporter 3	1409	67125	34	6	4	4	7.966
IPI00218463	Magmas	Magmas Mitochondrial import inner membrane trans	1771	13816	13	6	1	1	8.769
IPI00017533	MT-ATP6;MT-CC	MT-ATP6;MT-CO3 Cytochrome c oxidase subunit 3	40	30094	1	1	1	1	9.122
IPI00023001	FAM162A	FAM162A UPF0389 protein FAM162A	896	17331	25	8	3	3	9.192
IPI00022891	SLC25A4	SLC25A4 ADP/ATP translocase 1	7616	33043	25	7	3	3	9.586
IPI00299116	PODXL	PODXL Podocalyxin-like protein 1 precursor	3027	NaN36	67	6	11	8	9.647
IPI00296190	C10orf58	C10orf58 Uncharacterized protein C10orf58	889	25747	26	6	2	2	10.576
IPI00555902	OCIAD2	OCIAD2 Isoform 1 of OCIA domain-containing protei	2001	16943	43	7	3	3	10.995
IPI00008986	SLC7A5	SLC7A5 Large neutral amino acids transporter small	878	54974	12	3	3	3	14.317
IPI00006721	OPA1	OPA1 Isoform 1 of Dynamin-like 120 kDa protein, mi	1302	111561	24	6	5	5	25.79
IPI00023334	MRPL4	MRPL4 Isoform 1 of 39S ribosomal protein L4, mitoc	185	34897	4	2	1	1	31.509

Data1. AccessionNo (IPI)	iPS_CFB50/ HFF	iPS_CFB46/ H9ES	iPS_CFB50/ H9ES	iPS_CFB46/N TU1ES	iPS_CFB50/N TU1ES	H9ES/ HFF	NTU1ES/ HFF
IPI00014053	3.102	1.142	0.967	1.041	0.838	3.43	4.828
IPI00008599	3.854	1.105	1.18	0.752	0.794	3.537	4.804
IPI00007928	7.712	1.082	2.093	0.446	0.853	3.677	8.844
IPI00220487	3.174	1.095	0.928	0.906	0.755	3.4	4.299
IPI00006579	3.75	1.113	0.996	0.715	0.635	3.742	5.735
IPI00007183	2.572	1.12	0.714	1.215	0.766	3.651	3.286
IPI00396661	4.361	1.378	1.604	0.721	0.83	3.073	5.603
IPI00007188	4.256	0.878	0.949	0.982	1.051	5.114	3.968
IPI00016703	4.922	1.261	1.266	1.691	1.676	3.676	2.837
IPI00914855	3.312	1.122	0.904	0.855	0.723	3.714	4.841
IPI00030911	2.82	1.245	0.852	1.162	0.787	3.355	3.506
IPI00024976	3.327	1.025	0.878	1.034	0.78	3.161	3.944
IPI00027252	4.083	1.11	1.067	0.803	0.794	3.906	5.226
IPI00017334	4.117	0.81	0.78	0.795	0.758	5.047	4.676
IPI00015833	3.72	1.113	0.868	0.955	0.75	3.863	4.753
IPI00013475	3.986	0.981	0.864	0.964	0.841	4.675	4.639
IPI00011770	4.054	1.034	0.921	0.919	0.811	4.457	4.892
IPI00293476	3.509	1.192	0.914	1.25	0.949	3.89	3.621
IPI00024742	3.979	0.77	0.667	0.677	0.58	6.048	6.712
IPI00033217	2.048	1.519	0.679	1.875	1.084	3.231	2.385
IPI00171411	4.598	1.132	1.141	1.327	1.326	3.831	3.967
IPI00024650	4.914	1.186	1.156	0.72	0.678	4.141	6.885
IPI00219385	3.225	1.129	0.727	1.016	0.649	4.474	4.873
IPI00465059	4.164	1.073	0.938	0.917	0.761	3.593	5.625
IPI00016014	3	1.284	0.728	1.661	0.932	4.232	3.133
IPI00015602	4.263	1.149	0.841	0.998	0.725	4.424	4.754
IPI00063903	5.873	1.256	1.186	0.849	0.793	5.004	6.462
IPI00007084	5.025	1.093	1.006	0.862	0.762	5.534	6.985
IPI00023406	4.469	0.994	0.732	1.208	0.881	6.183	4.964
IPI00470360	3.46	8.117	4.541	3.384	1.874	0.772	1.807
IPI00218487	5.037	2.647	1.73	1.508	0.98	2.981	4.982
IPI00021842	5.906	1.373	1.197	1.153	0.995	5.3	5.58
IPI00003909	10.242	1.153	1.455	0.72	0.9	6.638	10.844
IPI00218565	6.479	1.514	1.342	0.601	0.528	4.892	12.022
IPI00295698	5.446	1.519	1.079	0.891	0.633	6.332	9.127
IPI00218463	5.535	1.41	0.912	1.024	0.656	6.145	8.257
IPI00017533	8.58	1.741	1.68	0.808	0.771	5.174	10.888
IPI00023001	9.49	1.184	1.329	0.566	0.628	7.659	15.579
IPI00022891	8.864	9999	9999	0.791	0.793	-9999	8.588
IPI00299116	9.595	1.206	1.036	0.688	0.626	8.48	15.799
IPI00296190	13.692	1.101	1.343	0.824	0.996	10.306	12.92
IPI00555902	8.919	0.645	0.644	1.02	0.922	9.955	9.155
IPI00008986	8.156	1.828	0.824	1.099	0.499	9.637	15.187
IPI00006721	27.38	1.18	1.137	1.11	1.093	16.411	22.021
IPI00023334	23.977	1.354	1.057	1.259	0.973	22.993	24.13

Table S1 Identified proteins in the human membrane proteome of hiPSCs, hESCs and somatic foreskin fibroblast HFF.
Profiles of identified proteins in MS Experiment 1

Data1. AccessionNo (IPI)	Gene Symbol	Description	Score	Mass	# of identified (spectrum)	# of samples identified	Total peptides	# of peptides used	iPS_CFB46 /HFF
IPI00019146	CXADR	CXADR Isoform 1 of Coxsackievirus and adenovirus	1265	40004	19	6	3	3	31.79
IPI00000230	TPM1	TPM1 tropomyosin 1 alpha chain isoform 2	44	32658	1	1	1	1	9999
IPI00000494	RPL5	RPL5 60S ribosomal protein L5	42	34341	1	1	1	1	9999
IPI00002070	LRRC8A	LRRC8A Leucine-rich repeat-containing protein 8A	82	94139	1	1	1	1	9999
IPI00002225	LASS4	LASS4 LAG1 longevity assurance homolog 4	581	46369	18	6	3	2	9999
IPI00002406	BCAM	BCAM Basal cell adhesion molecule	83	67363	2	2	1	1	9999
IPI00006608	APP	APP Isoform APP770 of Amyloid beta A4 protein (Fr	178	86888	9	5	1	1	9999
IPI00007034	UBAC2	UBAC2 Isoform 2 of Ubiquitin-associated domain-co	388	34853	6	5	1	1	9999
IPI00007730	C14orf1	C14orf1 Probable ergosterol biosynthetic protein 28	883	15854	19	6	2	2	9999
IPI00008351	SELT	SELT Selenoprotein T	94	22262	2	2	2	1	9999
IPI00010440	HAX1	HAX1 cDNA FLJ53021, highly similar to HS1-associa	112	32440	2	1	1	1	9999
IPI00011084	CLDN6	CLDN6 Claudin-6	2175	23276	56	6	3	3	9999
IPI00012540	PROM1	PROM1 Prominin-1	255	97140	3	2	2	2	9999
IPI00013449	TSPAN6	TSPAN6 Tetraspanin-6	111	27545	2	2	1	1	9999
IPI00013459	NDUFB5	NDUFB5 NADH dehydrogenase [ubiquinone] 1 beta	166	21737	5	2	1	1	9999
IPI00014376	RAB31	RAB31 RAB31, member RAS oncogene family	207	21686	3	1	1	1	9999
IPI00015140	MT-CYB	MT-CYB Cytochrome b	77	42775	3	2	1	1	9999
IPI00016597	CLN6	CLN6 Ceroid-lipofuscinosis neuronal protein 6	144	35896	3	2	1	1	9999
IPI00016676	TOMM20	TOMM20 Mitochondrial import receptor subunit TOM	44	16288	1	1	1	1	9999
IPI00017297	MATR3	MATR3 Matrin-3	44	94565	1	1	1	1	9999
IPI00019004	SEC62	SEC62 Translocation protein SEC62	459	45833	20	8	1	1	9999
IPI00020883	HIGD2A	HIGD2A HIG1 domain family member 2A	375	11521	9	4	3	1	9999
IPI00021766	RTN4	RTN4 Isoform 1 of Reticulon-4	4732	129851	1	1	1	1	9999
IPI00021770	HMGCR	HMGCR Isoform 1 of 3-hydroxy-3-methylglutaryl-coe	60	97413	1	1	1	1	9999
IPI00021793	COX6A1;COX6A	COX6A1;COX6A1P2 Cytochrome c oxidase subunit	244	12147	10	5	1	1	9999
IPI00022277	CCDC56	CCDC56 Coiled-coil domain-containing protein 56	103	11724	8	4	1	1	9999
IPI00022661	PVRL2	PVRL2 Isoform Delta of Poliovirus receptor-related p	64	57706	1	1	1	1	9999
IPI00023030	SLC38A1	SLC38A1 Sodium-coupled neutral amino acid transp	106	54012	2	1	1	1	9999
IPI00024627	MFF	MFF Isoform 4 of Mitochondrial fission factor	173	25051	7	4	2	2	9999
IPI00027233	SCO1	SCO1 Protein SCO1 homolog, mitochondrial	289	33793	7	3	1	1	9999
IPI00028931	DSG2	DSG2 Desmoglein-2	109	122218	2	2	2	2	9999
IPI00031822	SLC5A6	SLC5A6 Sodium-dependent multivitamin transporter	66	68596	1	1	1	1	9999
IPI00032003	EMD	EMD Emerin	116	28976	4	2	1	1	9999
IPI00032491	LEMD3	LEMD3 Inner nuclear membrane protein Man1	112	99935	2	1	1	1	9999
IPI00056414	MAL2	MAL2 Protein MAL2	181	19113	5	3	1	1	9999
IPI00062206	DDX39	DDX39 DEAD (Asp-Glu-Ala-Asp) box polypeptide 39	55	35072	1	1	1	1	9999
IPI00102509	SLC38A5	SLC38A5 Isoform 1 of Sodium-coupled neutral amin	96	51423	2	2	1	1	9999
IPI00165665	C3orf21	C3orf21 Isoform 2 of Uncharacterized protein C3orf2	476	22290	11	7	1	1	9999
IPI00166483	C17orf61	C17orf61 UPF0451 protein C17orf61	127	11734	2	1	1	1	9999
IPI00166704	TOMM5	TOMM5 Mitochondrial import receptor subunit TOM5	136	6031	10	5	1	1	9999
IPI00166860	DHRSX	DHRSX Dehydrogenase/reductase SDR family mem	71	36420	2	1	1	1	9999
IPI00168848	B3GNT7	B3GNT7 UDP-GlcNAc:betaGal beta-1,3-N-acetylgluc	599	45958	11	5	1	1	9999
IPI00178700	ALG3	ALG3 Dolichyl-P-Man:Man(5)GlcNAc(2)-PP-dolichyl	118	50094	2	2	1	1	9999
IPI00179713	IGF2BP2	IGF2BP2 Isoform 1 of Insulin-like growth factor 2 mR	60	66081	1	1	1	1	9999
IPI00182313	SEC61A2	SEC61A2 Sec61 alpha form 2 isoform b	2091	49561	9	4	2	2	9999

Data1. AccessionNo (IPI)	iPS_CFB50/ HFF	iPS_CFB46/ H9ES	iPS_CFB50/ H9ES	iPS_CFB46/N TU1ES	iPS_CFB50/N TU1ES	H9ES/ HFF	NTU1ES/ HFF
IPI00019146	25.113	1.139	0.928	1.239	1	27.737	24.635
IPI00000230	9999	0.952	1.042	9999	9999	9999	NaN
IPI00000494	NaN	9999	NaN	9999	NaN	NaN	NaN
IPI00002070	9999	1.144	1.648	1.805	2.574	9999	9999
IPI00002225	9999	0.411	2.235	0.195	0.973	9999	9999
IPI00002406	9999	1.305	1.284	0.788	0.768	9999	9999
IPI00006608	NaN	9999	NaN	0.975	-9999	NaN	9999
IPI00007034	9999	1.075	1.105	1.278	1.301	9999	9999
IPI00007730	9999	0.585	1.073	0.489	0.889	9999	9999
IPI00008351	9999	0.468	0.346	9999	9999	9999	NaN
IPI00010440	9999	0.395	0.397	0.428	0.426	9999	9999
IPI00011084	9999	1.274	1.452	0.731	0.825	9999	9999
IPI00012540	9999	0.96	0.828	1.484	1.27	9999	9999
IPI00013449	NaN	9999	NaN	9999	NaN	NaN	NaN
IPI00013459	9999	1.233	1.221	1.599	1.568	9999	9999
IPI00014376	NaN	9999	NaN	9999	NaN	NaN	NaN
IPI00015140	9999	1.136	0.531	9999	9999	9999	NaN
IPI00016597	9999	0.944	1.363	1.087	1.NaN	9999	9999
IPI00016676	9999	0.057	0.211	1.223	4.478	9999	9999
IPI00017297	9999	1.218	0.643	1.528	0.798	9999	9999
IPI00019004	9999	9999	9999	9999	9999	NaN	NaN
IPI00020883	9999	0.094	0.757	9999	9999	9999	NaN
IPI00021766	NaN	9999	NaN	1.09	-9999	NaN	9999
IPI00021770	NaN	9999	NaN	9999	NaN	NaN	NaN
IPI00021793	9999	0.873	0.805	0.891	0.813	9999	9999
IPI00022277	9999	0.773	0.95	0.663	0.807	9999	9999
IPI00022661	9999	0.982	0.852	9999	9999	9999	NaN
IPI00023030	9999	9999	9999	1.603	0.708	NaN	9999
IPI00024627	9999	0.956	1.11	1.459	1.677	9999	9999
IPI00027233	9999	0.561	0.516	0.851	0.775	9999	9999
IPI00028931	9999	0.784	0.398	1.434	0.503	9999	9999
IPI00031822	9999	1.196	1.037	0.849	0.729	9999	9999
IPI00032003	NaN	9999	NaN	9999	NaN	NaN	NaN
IPI00032491	9999	0.852	1.146	0.512	0.682	9999	9999
IPI00056414	9999	1.1	1.048	0.985	0.93	9999	9999
IPI00062206	9999	1.106	1.102	1.297	1.279	9999	9999
IPI00102509	NaN	9999	NaN	9999	NaN	NaN	NaN
IPI00165665	9999	0.582	1.074	1.232	2.252	9999	9999
IPI00166483	9999	0.604	0.67	0.497	0.545	9999	9999
IPI00166704	9999	0.908	0.932	0.63	0.641	9999	9999
IPI00166860	9999	0.665	0.855	9999	9999	9999	NaN
IPI00168848	9999	1.004	1.261	2.807	3.489	9999	9999
IPI00178700	9999	1.044	1.412	0.721	0.965	9999	9999
IPI00179713	9999	1.173	0.837	9999	9999	9999	NaN
IPI00182313	9999	0.134	0.533	0.554	1.828	9999	9999

Table S1 Identified proteins in the human membrane proteome of hiPSCs, hESCs and somatic foreskin fibroblast HFF.
Profiles of identified proteins in MS Experiment 1

Data1. AccessionNo (IPI)	Gene Symbol	Description	Score	Mass	# of identified (spectrum)	# of samples identified	Total peptides	# of peptides used	iPS_CFB46 /HFF
IPI00184708	SLC25A29	SLC25A29 Mitochondrial carnitine/acylcarnitine carri	131	32042	3	2	2	2	9999
IPI00217536	RHOT1	RHOT1 Isoform 3 of Mitochondrial Rho GTPase 1	527	79496	14	6	2	2	9999
IPI00217557	NRM	NRM Isoform 1 of Nurim	139	29360	3	1	1	1	9999
IPI00217975	LMNB1	LMNB1 Lamin-B1	316	66368	11	3	4	4	9999
IPI00219383	NDUFB3	NDUFB3 NADH dehydrogenase [ubiquinone] 1 beta	316	11395	16	6	1	1	9999
IPI00220059	NDUFB4	NDUFB4 NADH dehydrogenase [ubiquinone] 1 beta	132	15199	4	3	2	2	9999
IPI00220416	UQCRB	UQCRB Cytochrome b-c1 complex subunit 7	58	13522	3	2	1	1	9999
IPI00290544	GDAP1	GDAP1 Ganglioside-induced differentiation-associat	613	41226	14	6	2	2	9999
IPI00291262	CLU	CLU Isoform 1 of Clusterin	47	52461	1	1	1	1	9999
IPI00294455	UGT8	UGT8 2-hydroxyacylsphingosine 1-beta-galactosyltra	130	61416	3	2	1	1	9999
IPI00295598	CBARA1	CBARA1 Isoform 3 of Calcium-binding atopy-related	70	54547	1	1	1	1	9999
IPI00297325	SDHD	SDHD Succinate dehydrogenase [ubiquinone] cytocl	40	17032	1	1	1	1	9999
IPI00298417	GYLTL1B	GYLTL1B Isoform 1 of Glycosyltransferase-like prote	46	81735	1	1	1	1	9999
IPI00300096	RAB35	RAB35 Ras-related protein Rab-35	2999	23011	1	1	1	1	9999
IPI00300384	ERBB2	ERBB2 Receptor tyrosine-protein kinase erbB-2	38	137821	1	1	1	1	9999
IPI00304612	RPL13A	RPL13A 60S ribosomal protein L13a	732	23562	24	8	1	1	9999
IPI00305258	MOSC1	MOSC1 Isoform 1 of MOSC domain-containing prote	432	37476	6	3	2	2	9999
IPI00376686	AMFR	AMFR 42 kDa protein	268	42402	7	5	1	1	9999
IPI00377017	PKMYT1	PKMYT1 protein kinase Myt1 isoform 2	53	52113	1	1	1	1	9999
IPI00382470	HSP90AA1	HSP90AA1 Isoform 2 of Heat shock protein HSP 90-	1519	98099	2	2	1	1	9999
IPI00383798	PRODH	PRODH Isoform 4 of Proline dehydrogenase, mitoch	58	67959	2	1	2	2	9999
IPI00384867	SLC35B2	SLC35B2 Isoform 2 of Adenosine 3'-phospho 5'-pho:	41	42939	1	1	1	1	9999
IPI00386271	SLC25A12	SLC25A12 Calcium-binding mitochondrial carrier pro	2479	74715	3	2	2	2	9999
IPI00410714	HBA2;HBA1	HBA2;HBA1 Hemoglobin subunit alpha	89	15248	3	1	1	1	9999
IPI00412713	SAMM50	SAMM50 Sorting and assembly machinery compone	1229	51943	36	6	5	5	9999
IPI00414320	ANXA11	ANXA11 cDNA FLJ55482, highly similar to Annexin /	88	6NaN5	3	2	1	1	9999
IPI00425902	SSR2	SSR2 cDNA FLJ56303, highly similar to Translocon-	57	22007	1	1	1	1	9999
IPI00479357	BSCL2	BSCL2 Seipin	1308	51255	18	5	3	1	9999
IPI00555703	TMEM41B	TMEM41B Isoform 1 of Transmembrane protein 41B	496	32492	14	6	2	2	9999
IPI00642370	MPV17L2	MPV17L2 Isoform 1 of Mpv17-like protein 2	146	23165	3	2	1	1	9999
IPI00644766	TOR1AIP1	TOR1AIP1 Torsin A interacting protein 1	242	47549	6	2	1	1	9999
IPI00645767	GCNT2	GCNT2 glucosaminyl (N-acetyl) transferase 2, I-bran	172	46501	4	3	1	1	9999
IPI00646426	MRS2	MRS2 Isoform 2 of Magnesium transporter MRS2 ho	873	46461	31	6	4	4	9999
IPI00654820	MT-ATP6	MT-ATP6 ATP synthase subunit a	1063	26032	27	8	1	1	9999
IPI00747849	ATP1B1	ATP1B1 Isoform 1 of Sodium/potassium-transportinç	82	35039	1	1	1	1	9999
IPI00784376	MTP18	MTP18 Mitochondrial 18 kDa protein	91	17999	2	1	2	2	9999
IPI00902680	SAMM50	SAMM50 cDNA FLJ39696 fis, clone SMINT2011033	860	30193	8	3	1	1	9999
IPI00935944	HLA-A	HLA-A MHC class I antigen (Fragment)	4345	31427	1	1	1	1	9999
IPI00329596	TMX2	TMX2 Putative uncharacterized protein TMX2	1743	42444	37	8	3	3	1.NaN
IPI00024757	UQCR	UQCR Cytochrome b-c1 complex subunit 10	485	6565	17	6	1	1	2.NaN
IPI00000874	PRDX1	PRDX1 Peroxiredoxin-1	149	22096	5	4	1	1	NaN
IPI00001091	AFG3L2	AFG3L2 AFG3-like protein 2	2634	88528	72	6	8	8	NaN
IPI00003807	ACP2	ACP2 Lysosomal acid phosphatase	109	48313	4	2	1	1	NaN
IPI00003881	HNRNPF	HNRNPF Heterogeneous nuclear ribonucleoprotein I	745	45643	13	6	1	1	NaN
IPI00004267	SIGMAR1	SIGMAR1 Isoform 3 of Sigma 1-type opioid receptor	47	21469	1	1	1	1	NaN

Data1. AccessionNo (IPI)	iPS_CFB50/ HFF	iPS_CFB46/ H9ES	iPS_CFB50/ H9ES	iPS_CFB46/N TU1ES	iPS_CFB50/N TU1ES	H9ES/ HFF	NTU1ES/ HFF
IPI00184708	9999	0.335	0.244	1.432	1.033	9999	9999
IPI00217536	9999	1.036	0.9	1.112	0.956	9999	9999
IPI00217557	9999	0.835	1.794	0.542	1.154	9999	9999
IPI00217975	9999	0.438	1.36	0.769	1.628	9999	9999
IPI00219383	9999	1.253	1.042	0.824	0.679	9999	9999
IPI00220059	9999	1.086	0.837	0.877	0.669	9999	9999
IPI00220416	9999	1.158	1.718	9999	9999	9999	NaN
IPI00290544	9999	1.483	0.802	1.286	1.014	9999	9999
IPI00291262	NaN	1.397	-9999	9999	NaN	9999	NaN
IPI00294455	9999	1.444	1.549	0.867	0.922	9999	9999
IPI00295598	9999	0.424	1.015	0.801	1.898	9999	9999
IPI00297325	9999	1.227	1.387	9999	9999	9999	NaN
IPI00298417	NaN	9999	NaN	9999	NaN	NaN	NaN
IPI00300096	9999	1.487	1.487	9999	9999	9999	NaN
IPI00300384	9999	1.449	0.588	1.625	0.653	9999	9999
IPI00304612	9999	9999	9999	9999	9999	NaN	NaN
IPI00305258	9999	1.023	0.926	0.781	0.699	9999	9999
IPI00376686	9999	0.571	0.868	1.057	1.592	9999	9999
IPI00377017	9999	1.298	1.561	9999	9999	9999	NaN
IPI00382470	9999	1.423	1.041	9999	9999	9999	NaN
IPI00383798	9999	0.921	1.38	0.7	1.036	9999	9999
IPI00384867	9999	1.267	1.242	1.294	1.256	9999	9999
IPI00386271	9999	1.665	1.226	1.186	0.865	9999	9999
IPI00410714	NaN	9999	NaN	9999	NaN	NaN	NaN
IPI00412713	9999	1.114	0.891	1.047	0.82	9999	9999
IPI00414320	9999	1.34	1.501	1.37	1.519	9999	9999
IPI00425902	NaN	9999	NaN	9999	NaN	NaN	NaN
IPI00479357	9999	0.687	0.826	1.522	1.813	9999	9999
IPI00555703	9999	0.929	0.842	0.923	0.829	9999	9999
IPI00642370	9999	1.335	0.798	1.189	0.704	9999	9999
IPI00644766	9999	0.986	1.383	0.708	0.982	9999	9999
IPI00645767	9999	0.959	0.923	2.246	2.14	9999	9999
IPI00646426	9999	0.687	0.484	1.204	0.816	9999	9999
IPI00654820	9999	1.22	1.059	0.884	0.76	9999	9999
IPI00747849	9999	0.845	1.274	0.734	1.096	9999	9999
IPI00784376	9999	0.89	1.014	1.006	1.134	9999	9999
IPI00902680	9999	1.163	1.03	0.488	0.428	9999	9999
IPI00935944	9999	9999	9999	9999	9999	NaN	NaN
IPI00329596	1.31	1.074	1.016	1.031	0.969	1.814	1.296
IPI00024757	1.917	1.402	1.079	1.431	1.091	1.8	1.72
IPI00000874	NaN	NaN	NaN	NaN	NaN	NaN	NaN
IPI00001091	NaN	0.952	0.839	0.756	0.658	0.94	1.404
IPI00003807	NaN	NaN	NaN	NaN	NaN	NaN	NaN
IPI00003881	9999	-9999	1.259	-9999	0.987	9999	9999
IPI00004267	NaN	NaN	NaN	-9999	-9999	NaN	9999

Table S1 Identified proteins in the human membrane proteome of hiPSCs, hESCs and somatic foreskin fibroblast HFF.
Profiles of identified proteins in MS Experiment 1

Data1. AccessionNo (IPI)	Gene Symbol	Description	Score	Mass	# of identified (spectrum)	# of samples identified	Total peptides	# of peptides used	iPS_CFB46 /HFF
IPI00004450	RBMXL2	RBMXL2 RNA-binding motif protein, X-linked-like-2	62	42788	1	1	1	1	NaN
IPI00004454	DPM3	DPM3 Isoform 1 of Dolichol-phosphate mannosyltrar	72	10087	3	1	1	1	NaN
IPI00007611	ATP5O	ATP5O ATP synthase subunit O, mitochondrial	219	23263	3	2	1	1	NaN
IPI00008433	RPS5	RPS5 40S ribosomal protein S5	89	22862	1	1	1	1	NaN
IPI00009607	RAP2C	RAP2C Ras-related protein Rap-2c	440	20731	3	3	1	1	NaN
IPI00009662	NT5DC2	NT5DC2 Isoform 1 of 5'-nucleotidase domain-contair	61	60680	2	1	2	2	NaN
IPI00012429	C9orf5	C9orf5 Protein of unknown function UPF0118 family	208	98324	11	6	1	1	NaN
IPI00016334	MCAM	MCAM Isoform 1 of Cell surface glycoprotein MUC18	119	71563	3	1	1	1	NaN
IPI00016373	RAB13	RAB13 Ras-related protein Rab-13	1501	22760	11	4	2	2	NaN
IPI00017617	DDX5	DDX5 Probable ATP-dependent RNA helicase DDX5	209	69105	2	1	1	1	NaN
IPI00017842	TP53I11	TP53I11 Putative uncharacterized protein (Fragment	103	32353	2	1	2	2	NaN
IPI00020004	TMEM97	TMEM97 Transmembrane protein 97	97	20834	3	1	1	1	NaN
IPI00021147	DEGS1	DEGS1 Sphingolipid delta(4)-desaturase DES1	288	37841	10	5	2	2	NaN
IPI00021785	COX5B	COX5B Cytochrome c oxidase subunit 5B, mitochon	542	13687	21	6	2	2	NaN
IPI00022043	TSC1	TSC1 Hamartin	40	129685	1	1	1	1	NaN
IPI00022334	OAT	OAT Ornithine aminotransferase, mitochondrial	45	48504	1	1	1	1	NaN
IPI00022624	GPRC5A	GPRC5A Retinoic acid-induced protein 3	265	40225	4	1	1	1	NaN
IPI00022748	TMBIM6	TMBIM6 Bax inhibitor 1	120	26520	4	2	1	1	NaN
IPI00024670	REEP5	REEP5 Receptor expression-enhancing protein 5	83	21479	2	1	1	1	NaN
IPI00026241	BST2	BST2 Bone marrow stromal antigen 2	151	19756	3	3	1	1	NaN
IPI00026570	COX7A2	COX7A2 Cytochrome c oxidase polypeptide 7A2, mi	425	12836	16	6	1	1	NaN
IPI00031052	G6PC3	G6PC3 Glucose-6-phosphatase 3	103	38709	4	4	1	1	NaN
IPI00031820	FARSA	FARSA Phenylalanyl-tRNA synthetase alpha chain	40	57528	1	1	1	1	NaN
IPI00056334	PRKCDBP	PRKCDBP Protein kinase C delta-binding protein	70	27609	1	1	1	1	NaN
IPI00063242	PGAM5	PGAM5 Isoform 2 of Phosphoglycerate mutase famil	86	27988	4	3	1	1	NaN
IPI00100199	CYBRD1	CYBRD1 Cytochrome b reductase 1	84	31621	4	2	2	1	NaN
IPI00101374	TM9SF1	TM9SF1 cDNA FLJ61658, highly similar to Transme	94	92427	3	1	1	1	NaN
IPI00103057	FAM36A	FAM36A Isoform 2 of Protein FAM36A	87	14659	2	1	1	1	NaN
IPI00152959	MFSD1	MFSD1 Putative uncharacterized protein MFSD1	42	51433	1	1	1	1	NaN
IPI00153023	CCDC51	CCDC51 Isoform 1 of Coiled-coil domain-containing	40	45783	1	1	1	1	NaN
IPI00168340	SLC38A9	SLC38A9 Isoform 1 of Putative sodium-coupled neut	76	63748	2	2	1	1	NaN
IPI00168565	CNNM3	CNNM3 Isoform 1 of Metal transporter CNNM3	179	76072	4	3	1	1	NaN
IPI00183666	TRPV2	TRPV2 Transient receptor potential cation channel s	67	85926	2	1	1	1	NaN
IPI00216138	TAGLN	TAGLN Transgelin	130	22596	3	1	1	1	NaN
IPI00216691	PFN1	PFN1 Profilin-1	219	15045	7	2	2	2	NaN
IPI00218606	RPS23	RPS23 40S ribosomal protein S23	833	15798	24	8	1	1	NaN
IPI00295394	COX11	COX11 Cytochrome c oxidase assembly protein CO)	67	31410	1	1	1	1	NaN
IPI00297858	PTGES	PTGES Prostaglandin E synthase	46	17091	1	1	1	1	NaN
IPI00298851	CD151	CD151 CD151 antigen	215	28276	7	3	1	1	NaN
IPI00299010	SPG7	SPG7 Isoform 1 of Paraplegin	99	88179	2	1	1	1	NaN
IPI00302605	SLC30A7	SLC30A7 Zinc transporter 7	42	41599	1	1	1	1	NaN
IPI00304925	HSPA1A;HSPA1I	HSPA1A;HSPA1B Heat shock 70 kDa protein 1	2129	70009	1	1	1	1	NaN
IPI00306382	SCAMP3	SCAMP3 Isoform 1 of Secretory carrier-associated n	692	38262	13	6	1	1	NaN
IPI00329025	JAGN1	JAGN1 Protein jagunal homolog 1	42	21111	1	1	1	1	NaN
IPI00329338	PCYT1A	PCYT1A Choline-phosphate cytidylyltransferase A	88	41705	2	2	1	1	NaN

Data1. AccessionNo (IPI)	iPS_CFB50/ HFF	iPS_CFB46/ H9ES	iPS_CFB50/ H9ES	iPS_CFB46/N TU1ES	iPS_CFB50/N TU1ES	H9ES/ HFF	NTU1ES/ HFF
IPI00004450	NaN	NaN	NaN	NaN	NaN	NaN	NaN
IPI00004454	9999	NaN	9999	NaN	9999	NaN	NaN
IPI00007611	NaN	NaN	NaN	NaN	NaN	NaN	NaN
IPI00008433	NaN	-9999	-9999	NaN	NaN	9999	NaN
IPI00009607	NaN	NaN	NaN	-9999	-9999	NaN	9999
IPI00009662	NaN	-9999	-9999	NaN	NaN	9999	NaN
IPI00012429	9999	NaN	9999	NaN	9999	NaN	NaN
IPI00016334	9999	NaN	9999	NaN	9999	NaN	NaN
IPI00016373	0.744	9999	9999	9999	9999	-9999	-9999
IPI00017617	NaN	NaN	NaN	NaN	NaN	NaN	NaN
IPI00017842	NaN	NaN	NaN	NaN	NaN	NaN	NaN
IPI00020004	NaN	-9999	-9999	NaN	NaN	9999	NaN
IPI00021147	1.108	1.548	1.167	1.085	0.773	1.894	2.861
IPI00021785	NaN	NaN	NaN	NaN	NaN	NaN	NaN
IPI00022043	NaN	NaN	NaN	NaN	NaN	NaN	NaN
IPI00022334	NaN	NaN	NaN	NaN	NaN	NaN	NaN
IPI00022624	NaN	NaN	NaN	NaN	NaN	NaN	NaN
IPI00022748	9999	NaN	9999	NaN	9999	NaN	NaN
IPI00024670	NaN	NaN	NaN	NaN	NaN	NaN	NaN
IPI00026241	NaN	NaN	NaN	NaN	NaN	NaN	NaN
IPI00026570	NaN	-9999	-9999	NaN	NaN	9999	NaN
IPI00031052	9999	NaN	9999	-9999	1.814	NaN	9999
IPI00031820	NaN	-9999	-9999	NaN	NaN	9999	NaN
IPI00056334	NaN	NaN	NaN	-9999	-9999	NaN	9999
IPI00063242	NaN	NaN	NaN	NaN	NaN	NaN	NaN
IPI00100199	NaN	NaN	NaN	NaN	NaN	NaN	NaN
IPI00101374	NaN	NaN	NaN	NaN	NaN	NaN	NaN
IPI00103057	9999	-9999	1.13	-9999	1.155	9999	9999
IPI00152959	NaN	NaN	NaN	NaN	NaN	NaN	NaN
IPI00153023	NaN	NaN	NaN	NaN	NaN	NaN	NaN
IPI00168340	NaN	NaN	NaN	NaN	NaN	NaN	NaN
IPI00168565	9999	-9999	0.879	-9999	2.001	9999	9999
IPI00183666	NaN	NaN	NaN	NaN	NaN	NaN	NaN
IPI00216138	NaN	NaN	NaN	NaN	NaN	NaN	NaN
IPI00216691	NaN	-9999	-9999	-9999	-9999	9999	9999
IPI00218606	NaN	NaN	NaN	NaN	NaN	NaN	NaN
IPI00295394	NaN	-9999	-9999	NaN	NaN	9999	NaN
IPI00297858	NaN	NaN	NaN	NaN	NaN	NaN	NaN
IPI00298851	NaN	NaN	NaN	NaN	NaN	NaN	NaN
IPI00299010	NaN	-9999	-9999	-9999	-9999	9999	9999
IPI00302605	NaN	NaN	NaN	NaN	NaN	NaN	NaN
IPI00304925	NaN	NaN	NaN	NaN	NaN	NaN	NaN
IPI00306382	NaN	-9999	-9999	NaN	NaN	9999	NaN
IPI00329025	NaN	NaN	NaN	-9999	-9999	NaN	9999
IPI00329338	NaN	NaN	NaN	-9999	-9999	NaN	9999

Table S1 Identified proteins in the human membrane proteome of hiPSCs, hESCs and somatic foreskin fibroblast HFF.
Profiles of identified proteins in MS Experiment 1

Data1. AccessionNo (IPI)	Gene Symbol	Description	Score	Mass	# of identified (spectrum)	# of samples identified	Total peptides	# of peptides used	iPS_CFB46 /HFF
IPI00337541	NNT	NNT NAD(P) transhydrogenase, mitochondrial	175	113823	7	3	2	2	NaN
IPI00337800	DAAM1	DAAM1 Isoform 1 of Disheveled-associated activato	45	123396	1	1	1	1	NaN
IPI00376394	QSOX2	QSOX2 Sulfhydryl oxidase 2	599	77480	18	6	2	2	NaN
IPI00410211	DPY19L3	DPY19L3 Isoform 1 of Protein dpy-19 homolog 3	38	83142	1	1	1	1	NaN
IPI00457110	MCTP2	MCTP2 Isoform 1 of Multiple C2 and transmembrane	39	99542	2	2	1	1	NaN
IPI00470941	BET1	BET1 Putative uncharacterized protein DKFZp781C(	42	14037	1	1	1	1	NaN
IPI00552671	PLXNA1	PLXNA1 Plexin-A1	53	210953	1	1	1	1	NaN
IPI00607764	DHODH	DHODH Putative uncharacterized protein DHODH	53	22006	1	1	1	1	NaN
IPI00642041	COMTD1	COMTD1 Catechol-O-methyltransferase domain-con	73	28790	2	1	1	1	NaN
IPI00747078	GANAB	GANAB GANAB protein (Fragment)	339	34558	2	1	1	1	NaN
IPI00748932	-	- Similar to Chloride channel protein 3	37	11780	2	1	1	1	NaN
IPI00749454	RFTN1	RFTN1 cDNA FLJ56561	87	86651	3	2	1	1	NaN
IPI00760639	TDRD12	TDRD12 Isoform 2 of Tudor domain-containing prote	50	40674	3	1	1	1	NaN
IPI00784119	ATP6AP1	ATP6AP1 V-type proton ATPase subunit S1	100	51993	4	1	2	2	NaN
IPI00790232	FOXP1	FOXP1 18 kDa protein	45	18173	1	1	1	1	NaN
IPI00847689	HTATIP2	HTATIP2 HIV-1 Tat interactive protein 2, 30kDa isof	61	30112	1	1	1	1	NaN

Data1. AccessionNo (IPI)	iPS_CFB50/ HFF	iPS_CFB46/ H9ES	iPS_CFB50/ H9ES	iPS_CFB46/N TU1ES	iPS_CFB50/N TU1ES	H9ES/ HFF	NTU1ES/ HFF
IPI00337541	NaN	NaN	NaN	NaN	NaN	NaN	NaN
IPI00337800	NaN	NaN	NaN	NaN	NaN	NaN	NaN
IPI00376394	NaN	1.006	0.597	2.937	1.725	1.032	0.417
IPI00410211	9999	-9999	2.254	NaN	9999	9999	NaN
IPI00457110	NaN	NaN	NaN	NaN	NaN	NaN	NaN
IPI00470941	NaN	NaN	NaN	NaN	NaN	NaN	NaN
IPI00552671	NaN	NaN	NaN	NaN	NaN	NaN	NaN
IPI00607764	NaN	NaN	NaN	NaN	NaN	NaN	NaN
IPI00642041	NaN	NaN	NaN	NaN	NaN	NaN	NaN
IPI00747078	NaN	NaN	NaN	NaN	NaN	NaN	NaN
IPI00748932	9999	NaN	9999	NaN	9999	NaN	NaN
IPI00749454	NaN	NaN	NaN	NaN	NaN	NaN	NaN
IPI00760639	NaN	NaN	NaN	NaN	NaN	NaN	NaN
IPI00784119	NaN	NaN	NaN	NaN	NaN	NaN	NaN
IPI00790232	9999	NaN	9999	NaN	9999	NaN	NaN
IPI00847689	NaN	NaN	NaN	NaN	NaN	NaN	NaN

Table S1 Identified proteins in the human membrane proteome of hiPSCs, hESCs and somatic foreskin fibroblast HFF.
Profiles of identified proteins in MS Experiment 2

Data2. AccessionNo (IPI)	Gene Symbol	Description	Score	Mass	# of identified (spectrum)	# of samples identified	Total peptides	# of peptides used	iPS_CFB46/ HFF
IPI00000230	TPM1	TPM1 tropomyosin 1 alpha chain isoform 2	55	32658	2	2	1	1	0.361
IPI00000816	YWHAE	YWHAE 14-3-3 protein epsilon	73	29155	4	2	2	2	0.344
IPI00002506	ALG5	ALG5 Dolichyl-phosphate beta-glucosyltra	74	36922	1	1	1	1	1.124
IPI00003807	ACP2	ACP2 Lysosomal acid phosphatase	108	48313	2	1	2	2	-9999
IPI00006558	SH3GLB1	SH3GLB1 Isoform 1 of Endophilin-B1	40	40771	1	1	1	1	-9999
IPI00007067	GLIPR2	GLIPR2 Golgi-associated plant pathogene	125	17208	2	1	1	1	-9999
IPI00007068	ACTR3B	ACTR3B Isoform 1 of Actin-related protein	108	47577	3	2	1	1	0.419
IPI00007755	RAB21	RAB21 Ras-related protein Rab-21	40	24332	1	1	1	1	-9999
IPI00008274	CAP1	CAP1 Isoform 1 of Adenylyl cyclase-assoc	45	51823	1	1	1	1	0.253
IPI00009253	NAPA	NAPA Alpha-soluble NSF attachment prote	146	33211	3	1	1	1	0.871
IPI00011229	CTSD	CTSD Cathepsin D	56	44524	1	1	1	1	0.376
IPI00011302	CD59	CD59 CD59 glycoprotein	53	14168	2	1	1	1	-9999
IPI00013872	KDELR3	KDELR3 Isoform 1 of ER lumen protein re	79	25010	3	1	1	1	-9999
IPI00013895	S100A11	S100A11 Protein S100-A11	151	11733	3	1	1	1	0.323
IPI00015475	SLC1A1	SLC1A1 Excitatory amino acid transporter	136	57063	2	1	1	1	0.586
IPI00016249	FXR1	FXR1 Isoform 1 of Fragile X mental retard	162	69678	3	2	1	1	5.383
IPI00017375	SEC23A	SEC23A Protein transport protein Sec23A	37	86105	1	1	1	1	-9999
IPI00018953	DPP4	DPP4 Dipeptidyl peptidase 4	407	88222	10	1	6	6	0.069
IPI00021812	AHNAK	AHNAK Neuroblast differentiation-associat	218	628699	6	1	4	4	0.106
IPI00022300	METTL7A	METTL7A Methyltransferase-like protein 7	127	28301	3	1	1	1	0.87
IPI00022608	SORL1	SORL1 Sortilin-related receptor	49	248282	1	1	1	1	2.327
IPI00022624	GPRC5A	GPRC5A Retinoic acid-induced protein 3	217	40225	3	1	1	1	-9999
IPI00024466	UGGT1	UGGT1 Isoform 1 of UDP-glucose:glycopr	68	177078	1	1	1	1	2.643
IPI00026125	DNASE1L1	DNASE1L1 Deoxyribonuclease-1-like 1	37	33871	1	1	1	1	-9999
IPI00026154	PRKCSH	PRKCSH cDNA FLJ59211, highly similar t	269	60096	8	2	3	3	1.261
IPI00027497	GPI	GPI Glucose-6-phosphate isomerase	90	63107	2	1	1	1	0.471
IPI00028082	RECK	RECK Reversion-inducing cysteine-rich pr	40	106386	1	1	1	1	-9999
IPI00056478	IGSF8	IGSF8 Isoform 1 of Immunoglobulin super	53	64994	1	1	1	1	-9999
IPI00100160	CAND1	CAND1 Isoform 1 of Cullin-associated NE	141	136289	3	2	1	1	0.682
IPI00100199	CYBRD1	CYBRD1 Cytochrome b reductase 1	41	31621	1	1	1	1	-9999
IPI00100980	EHD2	EHD2 EH domain-containing protein 2	97	61123	2	1	1	1	-9999
IPI00171438	MUTED;TXN	MUTED;TXNDC5 Thioredoxin domain-cor	193	47599	6	2	2	2	1.134
IPI00171611	HIST2H3A;HI	HIST2H3A;HIST2H3C;HIST2H3D Histone	98	15379	2	2	1	1	-9999
IPI00176221	NEGR1	NEGR1 Neuronal growth regulator 1	51	38694	1	1	1	1	-9999
IPI00178352	FLNC	FLNC Isoform 1 of Filamin-C	259	290841	2	1	1	1	-9999

Data2. AccessionNo (IPI)	iPS_CFB50/ HFF	iPS_CFB46/ H9ES	iPS_CFB50/ H9ES	iPS_CFB46/ NTU1ES	iPS_CFB50/ NTU1ES	H9ES/ HFF	NTU1ES/ HFF
IPI00000230	-9999	0.972	-9999	9999	NaN	0.359	-9999
IPI00000816	0.242	0.445	0.316	9999	9999	0.748	-9999
IPI00002506	1.269	9999	9999	9999	9999	-9999	-9999
IPI00003807	0.91	NaN	9999	NaN	9999	-9999	-9999
IPI00006558	-9999	-9999	-9999	NaN	NaN	0.892	-9999
IPI00007067	-9999	NaN	NaN	NaN	NaN	-9999	-9999
IPI00007068	0.3	9999	9999	9999	9999	-9999	-9999
IPI00007755	-9999	NaN	NaN	NaN	NaN	-9999	-9999
IPI00008274	0.15	9999	9999	9999	9999	-9999	-9999
IPI00009253	0.562	0.546	0.355	9999	9999	1.543	-9999
IPI00011229	0.463	0.933	1.159	9999	9999	0.39	-9999
IPI00011302	-9999	NaN	NaN	NaN	NaN	-9999	-9999
IPI00013872	-9999	-9999	-9999	NaN	NaN	0.651	-9999
IPI00013895	0.191	0.636	0.379	9999	9999	0.492	-9999
IPI00015475	-9999	1.666	-9999	9999	NaN	0.34	-9999
IPI00016249	3.558	1.773	1.181	9999	9999	2.938	-9999
IPI00017375	-9999	-9999	-9999	NaN	NaN	0.463	-9999
IPI00018953	0.061	NaN	NaN	9999	9999	NaN	-9999
IPI00021812	0.99	0.999	1.263	9999	9999	0.815	-9999
IPI00022300	0.655	0.628	0.476	9999	9999	1.341	-9999
IPI00022608	1.714	9999	9999	9999	9999	-9999	-9999
IPI00022624	-9999	-9999	-9999	NaN	NaN	1.878	-9999
IPI00024466	1.989	4.456	3.381	9999	9999	0.574	-9999
IPI00026125	-9999	NaN	NaN	NaN	NaN	-9999	-9999
IPI00026154	1.057	0.36	0.205	9999	9999	3.461	-9999
IPI00027497	0.607	0.656	0.851	9999	9999	0.696	-9999
IPI00028082	-9999	NaN	NaN	NaN	NaN	-9999	-9999
IPI00056478	-9999	NaN	NaN	NaN	NaN	-9999	-9999
IPI00100160	0.558	9999	9999	9999	9999	-9999	-9999
IPI00100199	-9999	NaN	NaN	NaN	NaN	-9999	-9999
IPI00100980	-9999	NaN	NaN	NaN	NaN	-9999	-9999
IPI00171438	-9999	0.654	-9999	9999	NaN	1.679	-9999
IPI00171611	-9999	NaN	NaN	NaN	NaN	-9999	-9999
IPI00176221	-9999	NaN	NaN	NaN	NaN	-9999	-9999
IPI00178352	-9999	NaN	NaN	NaN	NaN	-9999	-9999

Table S1 Identified proteins in the human membrane proteome of hiPSCs, hESCs and somatic foreskin fibroblast HFF.
Profiles of identified proteins in MS Experiment 2

Data2. AccessionNo (IPI)	Gene Symbol	Description	Score	Mass	# of identified (spectrum)	# of samples identified	Total peptides	# of peptides used	iPS_CFB46/ HFF
IPI00215914	ARF1	ARF1 ADP-ribosylation factor 1	101	20684	1	1	1	1	-9999
IPI00215995	ITGA3	ITGA3 Isoform Alpha-3A of Integrin alpha-	79	116538	3	1	2	2	-9999
IPI00216127	PLSCR3	PLSCR3 Phospholipid scramblase 3	174	31642	3	1	1	1	0.113
IPI00216514	CD47	CD47 Isoform OA3-293 of Leukocyte surfa	38	31722	1	1	1	1	1.016
IPI00216691	PFN1	PFN1 Profilin-1	104	15045	3	1	1	1	-9999
IPI00217471	HBE1	HBE1 Hemoglobin subunit epsilon	45	16192	1	1	1	1	-9999
IPI00218086	RDH10	RDH10 Retinol dehydrogenase 10	45	38062	1	1	1	1	-9999
IPI00218850	SCAMP2	SCAMP2 Secretory carrier-associated mei	158	36625	4	3	1	1	-9999
IPI00219219	LGALS1	LGALS1 Galectin-1	41	14706	1	1	1	1	-9999
IPI00220007	APOL2	APOL2 cDNA FLJ55764, highly similar to /	89	49735	2	1	1	1	-9999
IPI00253050	L1TD1	L1TD1 LINE-1 type transposase domain-c	120	98789	5	1	2	2	NaN
IPI00254338	FAM134C	FAM134C Protein FAM134C	129	51364	4	2	1	1	-9999
IPI00290452	TMBIM1	TMBIM1 Transmembrane BAX inhibitor m	57	34585	1	1	1	1	0.091
IPI00294834	ASPH	ASPH Aspartyl/asparaginyl beta-hydroxyla	152	85809	3	1	2	2	0.373
IPI00295741	CTSB	CTSB Cathepsin B	47	37797	1	1	1	1	-9999
IPI00297261	PTPN1	PTPN1 Tyrosine-protein phosphatase non-	63	49935	2	1	1	1	-9999
IPI00303318	FAM49B	FAM49B Protein FAM49B	52	36725	1	1	1	1	-9999
IPI00375676	FTL	FTL Ferritin	47	21283	1	1	1	1	0.703
IPI00465059	RHOT2	RHOT2 Isoform 1 of Mitochondrial Rho GT	148	68075	7	3	2	2	-9999
IPI00465439	ALDOA	ALDOA Fructose-bisphosphate aldolase A	83	39395	2	1	1	1	-9999
IPI00470535	CACNA2D1	CACNA2D1 Dihydropyridine receptor alpha	96	125229	2	1	2	2	-9999
IPI00479145	KRT19	KRT19 Keratin, type I cytoskeletal 19	529	44065	10	4	2	2	1.94
IPI00554648	KRT8	KRT8 Keratin, type II cytoskeletal 8	878	53671	14	3	3	3	1.034
IPI00746105	HLA-B	HLA-B HLA class I histocompatibility antige	868	40480	3	1	1	1	-9999
IPI00793199	ANXA4	ANXA4 annexin IV	180	36062	3	1	1	1	-9999
IPI00909570	EEF2	EEF2 cDNA FLJ56548, highly similar to El	202	62810	8	3	2	2	-9999
IPI00944575	HLA-C	HLA-C MHC class I antigen (Fragment)	1086	35390	3	1	1	1	0.052
IPI00298994	TLN1	TLN1 Talin-1	286	269599	5	1	2	2	0.13
IPI00297160	CD44	CD44 Isoform 12 of CD44 antigen	1009	39391	18	1	6	3	0.059
IPI00514023	PTRF	PTRF cDNA FLJ53495, highly similar to Pt	228	40459	4	1	3	3	-9999
IPI00013508	ACTN1	ACTN1 Alpha-actinin-1	285	102993	11	2	2	2	0.276
IPI00012503	PSAP	PSAP Isoform Sap-mu-0 of Proactivator po	42	58074	1	1	1	1	0.168
IPI00025512	HSPB1	HSPB1 Heat shock protein beta-1	95	22768	3	1	1	1	-9999
IPI00554788	KRT18	KRT18 Keratin, type I cytoskeletal 18	866	48029	27	3	6	6	0.112
IPI00010418	MYO1C	MYO1C Isoform 2 of Myosin-Ic	166	117876	6	3	2	1	0.108

Data2. AccessionNo (IPI)	iPS_CFB50/ HFF	iPS_CFB46/ H9ES	iPS_CFB50/ H9ES	iPS_CFB46/ NTU1ES	iPS_CFB50/ NTU1ES	H9ES/ HFF	NTU1ES/ HFF
IPI00215914	-9999	-9999	-9999	NaN	NaN	0.837	-9999
IPI00215995	NaN	NaN	9999	NaN	9999	-9999	-9999
IPI00216127	0.18	0.404	0.646	9999	9999	0.271	-9999
IPI00216514	1.349	9999	9999	9999	9999	-9999	-9999
IPI00216691	-9999	NaN	NaN	NaN	NaN	-9999	-9999
IPI00217471	-9999	NaN	NaN	NaN	NaN	-9999	-9999
IPI00218086	0.408	NaN	9999	NaN	9999	-9999	-9999
IPI00218850	-9999	NaN	NaN	NaN	NaN	-9999	-9999
IPI00219219	-9999	NaN	NaN	NaN	NaN	-9999	-9999
IPI00220007	-9999	NaN	NaN	NaN	NaN	-9999	-9999
IPI00253050	0.428	0.694	1.111	9999	9999	NaN	-9999
IPI00254338	4.474	NaN	9999	NaN	9999	-9999	-9999
IPI00290452	-9999	9999	NaN	9999	NaN	-9999	-9999
IPI00294834	0.336	9999	9999	9999	9999	-9999	-9999
IPI00295741	-9999	-9999	-9999	NaN	NaN	1.336	-9999
IPI00297261	-9999	NaN	NaN	NaN	NaN	-9999	-9999
IPI00303318	-9999	NaN	NaN	NaN	NaN	-9999	-9999
IPI00375676	0.645	0.621	0.573	9999	9999	1.097	-9999
IPI00465059	-9999	-9999	-9999	NaN	NaN	5.034	-9999
IPI00465439	-9999	NaN	NaN	NaN	NaN	-9999	-9999
IPI00470535	-9999	NaN	NaN	NaN	NaN	-9999	-9999
IPI00479145	2.22	9999	9999	9999	9999	-9999	-9999
IPI00554648	-9999	9999	NaN	9999	NaN	-9999	-9999
IPI00746105	-9999	NaN	NaN	NaN	NaN	-9999	-9999
IPI00793199	-9999	NaN	NaN	NaN	NaN	-9999	-9999
IPI00909570	-9999	NaN	NaN	NaN	NaN	-9999	-9999
IPI00944575	0.069	9999	9999	9999	9999	-9999	-9999
IPI00298994	0.116	9999	9999	6.923	6.232	-9999	0.019
IPI00297160	0.045	0.669	0.28	1.631	0.75	0.123	0.035
IPI00514023	0.029	-9999	1.01	-9999	0.409	0.028	0.052
IPI00013508	0.143	0.732	0.384	4.143	2.068	0.367	0.055
IPI00012503	0.136	1.484	1.217	2.513	2.054	0.109	0.066
IPI00025512	0.453	NaN	9999	-9999	6.594	-9999	0.069
IPI00554788	0.163	5.425	2.945	2.575	2.417	NaN	0.075
IPI00010418	0.132	0.865	0.715	1.212	1.546	0.138	0.086

Table S1 Identified proteins in the human membrane proteome of hiPSCs, hESCs and somatic foreskin fibroblast HFF.
Profiles of identified proteins in MS Experiment 2

Data2. AccessionNo (IPI)	Gene Symbol	Description	Score	Mass	# of identified (spectrum)	# of samples identified	Total peptides	# of peptides used	iPS_CFB46/ HFF
IPI00003411	PTGIS	PTGIS Prostacyclin synthase	1097	57068	39	4	11	10	0.072
IPI00009236	CAV1	CAV1 Isoform Alpha of Caveolin-1	365	20458	16	4	4	3	0.11
IPI00221232	GNG12	GNG12 Guanine nucleotide-binding protei	154	8001	4	1	2	2	0.09
IPI00418471	VIM	VIM Vimentin	1817	53619	68	3	16	14	0.102
IPI00095891	GNAS	GNAS Isoform XLas-1 of Guanine nucleoti	337	110956	3	1	1	1	0.394
IPI00221224	ANPEP	ANPEP Aminopeptidase N	2192	109471	49	1	21	18	0.101
IPI00302592	FLNA	FLNA Isoform 2 of Filamin-A	392	279843	11	3	3	3	0.29
IPI00291175	VCL	VCL Isoform 1 of Vinculin	79	116649	2	1	2	1	0.567
IPI00479186	PKM2	PKM2 Isoform M2 of Pyruvate kinase isoz	599	57900	14	2	6	5	0.18
IPI00012490	ATP2B4	ATP2B4 Isoform XD of Plasma membrane	774	137833	20	1	11	8	0.205
IPI00171705	NLGN4Y	NLGN4Y Isoform 1 of Neuroligin-4, Y-linke	125	91962	4	1	2	2	0.157
IPI00032370	ALG8	ALG8 Probable dolichyl pyrophosphate Gl	44	60048	1	1	1	1	0.12
IPI00000190	CD81	CD81 CD81 antigen	293	25792	5	1	2	2	0.065
IPI00156689	VAT1	VAT1 Synaptic vesicle membrane protein	178	41893	2	1	1	1	0.408
IPI00218918	ANXA1	ANXA1 Annexin A1	1492	38690	31	3	9	7	0.218
IPI00306604	ITGA5	ITGA5 Integrin alpha-5	395	114465	7	1	3	3	0.103
IPI00221240	LNPEP	LNPEP Isoform 2 of Leucyl-cystinyl amino	316	115562	7	2	2	2	0.291
IPI00246058	PDCD6IP	PDCD6IP Programmed cell death 6-intera	67	95963	1	1	1	1	0.507
IPI00019502	MYH9	MYH9 Isoform 1 of Myosin-9	1986	226392	48	2	14	14	0.533
IPI00418169	ANXA2	ANXA2 Isoform 2 of Annexin A2	3732	40386	98	4	18	15	0.278
IPI00300562	RAB3B	RAB3B Ras-related protein Rab-3B	1402	24742	10	4	1	1	0.26
IPI00022892	THY1	THY1 Thy-1 membrane glycoprotein	1751	17923	41	4	6	4	0.164
IPI00219018	GAPDH	GAPDH Glyceraldehyde-3-phosphate deh	3118	36030	70	4	10	6	0.246
IPI00219365	MSN	MSN Moesin	164	67778	6	2	2	2	0.336
IPI00386139	FAR1	FAR1 cDNA: FLJ22728 fis, clone HSI1561	101	38354	3	2	1	1	0.25
IPI00743716	HLA-C	HLA-C HLA class I histocompatibility antig	1304	40860	13	4	2	2	0.147
IPI00025818	GALNT1	GALNT1 Isoform 1 of Polypeptide N-acety	143	64177	5	2	3	3	NaN
IPI00215998	CD63	CD63 CD63 antigen	781	25619	24	4	5	2	0.155
IPI00009456	NT5E	NT5E 5'-nucleotidase	2350	63327	54	1	21	12	0.317
IPI00329332	STX12	STX12 Syntaxin-12	256	31622	6	1	3	3	0.273
IPI00017344	RAB5B	RAB5B Ras-related protein Rab-5B	296	23692	1	1	1	1	0.248
IPI00029266	SNRPE	SNRPE Small nuclear ribonucleoprotein E	107	10797	3	2	1	1	0.403
IPI00297646	COL1A1	COL1A1 Collagen alpha-1(I) chain	158	138827	5	1	3	3	0.209
IPI00005707	MRC2	MRC2 C-type mannose receptor 2	298	166549	6	1	2	2	0.15
IPI00549343	VAMP3	VAMP3 Vesicle-associated membrane pro	1356	11302	21	4	3	2	0.255

Data2. AccessionNo (IPI)	iPS_CFB50/ HFF	iPS_CFB46/ H9ES	iPS_CFB50/ H9ES	iPS_CFB46/ NTU1ES	iPS_CFB50/ NTU1ES	H9ES/ HFF	NTU1ES/ HFF
IPI00003411	0.095	0.145	0.192	0.784	1.06	0.2	0.089
IPI00009236	0.086	1.348	1.253	1.208	0.951	0.063	0.09
IPI00221232	-9999	1.976	-9999	0.938	-9999	0.044	0.096
IPI00418471	0.153	0.752	0.869	1.115	1.316	0.127	0.1
IPI00095891	0.183	3.292	1.541	3.58	1.671	0.116	0.11
IPI00221224	0.079	0.8	0.756	0.941	0.911	0.083	0.115
IPI00302592	0.21	1.03	0.822	1.808	0.853	0.26	0.115
IPI00291175	0.225	1.643	0.764	4.181	1.939	0.287	0.116
IPI00479186	0.118	0.552	0.356	1.324	0.945	0.306	0.121
IPI00012490	0.155	0.965	1.093	0.898	1	0.109	0.131
IPI00171705	0.078	0.581	0.301	1.032	0.527	0.254	0.131
IPI00032370	0.189	3.166	5.019	0.88	1.391	0.037	0.136
IPI00000190	0.05	0.728	0.571	0.471	0.369	0.086	0.137
IPI00156689	0.325	3.05	2.446	2.957	2.364	0.129	0.137
IPI00218918	0.151	0.676	0.585	1.483	1.488	0.305	0.14
IPI00306604	0.487	1.655	2.084	1.078	1.354	0.09	0.141
IPI00221240	0.249	4.364	3.757	1.823	1.565	0.065	0.159
IPI00246058	-9999	9999	NaN	3.15	-9999	-9999	0.16
IPI00019502	0.178	1.267	0.529	2.745	1.13	0.4	0.184
IPI00418169	0.183	0.92	0.614	1.45	0.976	0.3	0.189
IPI00300562	0.297	2.236	2.578	1.344	1.546	0.113	0.193
IPI00022892	0.15	0.591	0.562	0.845	0.8	0.271	0.195
IPI00219018	0.198	0.439	0.342	1.606	0.903	0.551	0.196
IPI00219365	0.205	0.508	0.314	1.691	1.044	0.64	0.196
IPI00386139	0.282	1.238	1.411	1.262	1.434	0.195	0.197
IPI00743716	0.214	0.449	0.665	0.68	1.005	0.303	0.197
IPI00025818	NaN	0.803	0.882	0.794	0.869	NaN	0.203
IPI00215998	0.213	0.534	0.814	0.679	1.015	0.254	0.212
IPI00009456	0.183	0.904	0.993	0.739	0.812	0.201	0.213
IPI00329332	0.309	1.408	1.311	1.542	1.446	0.191	0.213
IPI00017344	0.225	1.015	0.931	1.125	1.028	0.236	0.219
IPI00029266	0.337	0.295	0.248	1.801	1.51	1.322	0.223
IPI00297646	0.265	0.366	0.523	0.846	1.176	0.487	0.224
IPI00005707	0.174	0.842	0.984	0.657	0.766	0.172	0.227
IPI00549343	0.29	1.042	1.016	1.091	1.093	0.226	0.228

Table S1 Identified proteins in the human membrane proteome of hiPSCs, hESCs and somatic foreskin fibroblast HFF.
Profiles of identified proteins in MS Experiment 2

Data2. AccessionNo (IPI)	Gene Symbol	Description	Score	Mass	# of identified (spectrum)	# of samples identified	Total peptides	# of peptides used	iPS_CFB46/ HFF
IPI00007426	ARL6IP5	ARL6IP5 PRA1 family protein 3	712	21600	15	3	4	2	0.451
IPI00009030	LAMP2	LAMP2 Isoform LAMP-2A of Lysosome-as	993	44932	26	4	4	3	0.201
IPI00554521	FTH1	FTH1 Ferritin heavy chain	99	21212	3	1	2	2	-9999
IPI00644020	SOAT1	SOAT1 Sterol O-acyltransferase (Acyl-Coe	72	29874	2	2	1	1	0.384
IPI00219682	STOM	STOM Erythrocyte band 7 integral membra	1064	31711	32	3	8	7	0.174
IPI00305551	GNA11	GNA11 Guanine nucleotide-binding protei	278	42097	10	3	3	2	0.338
IPI00012011	CFL1	CFL1 Cofilin-1	85	18491	1	1	1	1	0.274
IPI00419585	PPIA	PPIA Peptidyl-prolyl cis-trans isomerase A	88	18001	3	1	1	1	0.307
IPI00217766	SCARB2	SCARB2 Lysosome membrane protein 2	1235	54255	38	4	11	7	0.24
IPI00440493	ATP5A1	ATP5A1 ATP synthase subunit alpha, mitc	2352	59714	51	4	12	9	0.886
IPI00021439	ACTB	ACTB Actin, cytoplasmic 1	5646	41710	143	4	17	10	0.506
IPI00006865	SEC22B	SEC22B Vesicle-trafficking protein SEC22	2311	24725	48	4	9	6	0.209
IPI00000643	BAG2	BAG2 BAG family molecular chaperone re	95	23757	3	1	1	1	0.255
IPI00021048	MYOF	MYOF Isoform 1 of Myoferlin	1277	234561	44	1	21	20	0.127
IPI00242630	HEATR2	HEATR2 Isoform 1 of HEAT repeat-contai	39	93474	3	3	1	1	0.209
IPI00012512	RRAS2	RRAS2 cDNA FLJ61162, highly similar to	544	24180	4	2	1	1	0.41
IPI00020418	RRAS	RRAS Ras-related protein R-Ras	600	23466	13	2	5	4	0.231
IPI00419916	ALPL	ALPL Alkaline phosphatase, tissue-nonspe	1055	57269	29	3	6	5	0.248
IPI00554742	API5	API5 Isoform 2 of Apoptosis inhibitor 5	62	56735	1	1	1	1	0.291
IPI00002459	ANXA6	ANXA6 annexin VI isoform 2	1709	75229	48	4	16	12	0.363
IPI00021428	ACTA1	ACTA1 Actin, alpha skeletal muscle	1939	42024	17	4	4	3	0.632
IPI00015148	RAP1B	RAP1B Ras-related protein Rap-1b	821	20812	24	4	2	2	0.331
IPI00328257	AP1B1	AP1B1 Isoform A of AP-1 complex subunit	51	104540	1	1	1	1	0.354
IPI00003935	HIST2H2BE	HIST2H2BE Histone H2B type 2-E	858	13912	16	3	3	2	0.957
IPI00168184	PPP2R1A	PPP2R1A cDNA FLJ34068 fis, clone FCBI	99	56812	2	2	1	1	0.5
IPI00217468	HIST1H1B	HIST1H1B Histone H1.5	37	22566	1	1	1	1	0.559
IPI00922108	ITGAV	ITGAV integrin alpha-V isoform 2	992	111075	33	4	9	9	0.353
IPI00289876	STX7	STX7 Isoform 1 of Syntaxin-7	219	29797	4	2	2	2	0.407
IPI00045946	YME1L1	YME1L1 Isoform 1 of ATP-dependent met	218	86401	5	2	1	1	0.661
IPI00797136	IKIP	IKIP Isoform 1 of Inhibitor of nuclear factor	426	39285	9	1	3	3	0.433
IPI00002230	NCEH1	NCEH1 arylacetamide deacetylase-like 1 i	654	49031	16	1	6	6	0.397
IPI00009111	TPBG	TPBG Trophoblast glycoprotein	670	46003	13	3	3	2	0.265
IPI00000105	MVP	MVP Major vault protein	62	99266	2	1	2	2	0.518
IPI00396485	EEF1A1	EEF1A1 Elongation factor 1-alpha 1	1734	50109	61	4	9	7	0.49
IPI00026569	HLA-A	HLA-A HLA class I histocompatibility antig	1040	40820	7	3	2	1	0.334

Data2. AccessionNo (IPI)	iPS_CFB50/ HFF	iPS_CFB46/ H9ES	iPS_CFB50/ H9ES	iPS_CFB46/ NTU1ES	iPS_CFB50/ NTU1ES	H9ES/ HFF	NTU1ES/ HFF
IPI00007426	0.475	2.783	2.838	2.131	2.182	0.177	0.23
IPI00009030	0.192	1.129	1.095	0.864	0.826	0.168	0.232
IPI00554521	-9999	NaN	NaN	-9999	-9999	-9999	0.233
IPI00644020	0.306	0.358	0.288	1.565	1.252	1.037	0.244
IPI00219682	0.172	0.983	1.033	0.689	0.678	0.127	0.253
IPI00305551	0.233	1.413	1.042	1.331	0.98	0.234	0.253
IPI00012011	0.282	0.474	0.492	1.062	1.099	0.56	0.257
IPI00419585	0.256	0.422	0.354	1.185	0.992	0.705	0.258
IPI00217766	0.254	0.769	0.698	0.827	0.864	0.506	0.265
IPI00440493	0.333	0.335	0.182	2.507	1.266	2.814	0.265
IPI00021439	0.243	0.809	0.388	1.877	0.91	0.605	0.268
IPI00006865	0.314	0.737	1.038	0.76	1.127	0.3	0.27
IPI00000643	-9999	9999	NaN	0.915	-9999	-9999	0.277
IPI00021048	0.255	1.062	0.788	1.11	1.046	0.289	0.279
IPI00242630	0.178	1.328	1.144	0.737	0.634	0.152	0.282
IPI00012512	0.362	0.974	0.867	1.439	1.277	0.407	0.284
IPI00020418	0.223	0.93	0.907	0.776	0.754	0.243	0.285
IPI00419916	0.202	1.342	1.272	0.769	0.721	0.176	0.29
IPI00554742	0.174	0.573	0.345	0.991	0.596	0.493	0.293
IPI00002459	0.269	0.574	0.426	1.286	0.919	0.622	0.294
IPI00021428	0.383	0.838	0.487	1.99	1.233	0.727	0.294
IPI00015148	0.272	2.433	2.017	1.116	0.923	0.131	0.295
IPI00328257	0.274	0.618	0.481	1.189	0.923	0.NaN	0.297
IPI00003935	0.437	1.117	0.518	3.173	1.418	0.828	0.299
IPI00168184	0.39	0.795	0.625	1.663	1.303	0.608	0.299
IPI00217468	0.27	1.146	0.559	1.84	0.894	0.472	0.302
IPI00922108	0.389	0.874	0.976	1.134	1.263	0.401	0.306
IPI00289876	0.482	1.065	1.263	1.326	1.567	0.371	0.308
IPI00045946	0.336	1.019	0.521	2.094	1.068	0.628	0.314
IPI00797136	0.426	NaN	NaN	1.354	1.339	0.176	0.318
IPI00002230	0.492	0.663	0.714	1.357	1.456	0.746	0.319
IPI00009111	0.391	1.613	2.638	0.833	1.203	0.138	0.324
IPI00000105	0.5	9999	9999	1.578	1.529	-9999	0.327
IPI00396485	0.442	0.762	0.53	1.493	1.324	0.703	0.327
IPI00026569	0.362	3.218	2.408	1.188	1.137	0.315	0.331

Table S1 Identified proteins in the human membrane proteome of hiPSCs, hESCs and somatic foreskin fibroblast HFF.
Profiles of identified proteins in MS Experiment 2

Data2. AccessionNo (IPI)	Gene Symbol	Description	Score	Mass	# of identified (spectrum)	# of samples identified	Total peptides	# of peptides used	iPS_CFB46/ HFF
IPI00329801	ANXA5	ANXA5 Annexin A5	1503	35914	38	3	10	10	0.38
IPI00479058	RPS15	RPS15 40S ribosomal protein S15	96	17029	1	1	1	1	1.526
IPI00011578	NPTN	NPTN Isoform 1 of Neuroplastin	390	31272	9	4	1	1	0.301
IPI00102685	MYADM	MYADM Myeloid-associated differentiation	816	35250	13	3	3	2	0.311
IPI00064193	TMX3	TMX3 Isoform 1 of Protein disulfide-isome	146	51839	5	3	2	2	0.248
IPI00298851	CD151	CD151 CD151 antigen	192	28276	7	2	2	1	0.4
IPI00295461	FAP	FAP Isoform 1 of Seprase	599	87683	15	1	6	5	0.303
IPI00022418	FN1	FN1 Isoform 1 of Fibronectin	948	262442	20	1	10	10	0.249
IPI00218398	MMP14	MMP14 Matrix metalloproteinase-14	342	65842	11	3	4	4	0.33
IPI00465248	ENO1	ENO1 Isoform alpha-enolase of Alpha-enc	199	47139	8	3	4	3	0.471
IPI00005719	RAB1A	RAB1A Isoform 1 of Ras-related protein R	2033	22663	7	3	1	1	0.452
IPI00022434	ALB	ALB Putative uncharacterized protein ALB	472	71658	13	4	2	1	1.598
IPI00025086	COX5A	COX5A Cytochrome c oxidase subunit 5A,	189	16752	6	4	2	2	NaN
IPI00446235	CYB5R3	CYB5R3 Isoform 2 of NADH-cytochrome b	2954	31608	71	4	12	7	0.27
IPI00930070	HLA-C	HLA-C MHC class I antigen (Fragment)	1311	31659	37	4	8	7	0.342
IPI00291928	RAB14	RAB14 Ras-related protein Rab-14	1432	23882	14	4	4	4	0.316
IPI00217563	ITGB1	ITGB1 Isoform Beta-1A of Integrin beta-1	2862	88357	76	4	14	10	0.385
IPI00024067	CLTC	CLTC Isoform 1 of Clathrin heavy chain 1	1595	191493	55	4	15	14	0.652
IPI00550987	CEP135	CEP135 Isoform 1 of Centrosomal protein	38	133422	1	1	1	1	-9999
IPI00005107	NPC1	NPC1 Niemann-Pick C1 protein	367	142074	10	4	3	3	0.39
IPI00021266	RPL23A	RPL23A 60S ribosomal protein L23a	557	17684	13	4	1	1	0.579
IPI00009276	PROCR	PROCR Endothelial protein C receptor pre	251	30696	6	2	2	2	0.271
IPI00018248	KDELR2	KDELR2 Isoform 1 of ER lumen protein re	258	24406	10	3	4	3	0.612
IPI00030431	ANTXR1	ANTXR1 Isoform 1 of Anthrax toxin recept	58	62749	1	1	1	1	2.127
IPI00023860	NAP1L1	NAP1L1 Nucleosome assembly protein 1-l	91	45346	3	1	1	1	1.154
IPI00010796	P4HB	P4HB Protein disulfide-isomerase	325	57081	10	3	2	2	0.767
IPI00646304	PPIB	PPIB Peptidyl-prolyl cis-trans isomerase B	279	23728	8	3	2	2	0.842
IPI00028387	DDRGK1	DDRGK1 Isoform 1 of DDRGK domain-co	333	35589	7	2	3	3	0.835
IPI00020557	LRP1	LRP1 Prolow-density lipoprotein receptor-r	860	504245	27	4	8	8	0.473
IPI00456969	DYNC1H1	DYNC1H1 Cytoplasmic dynein 1 heavy ch	38	532072	1	1	1	1	0.543
IPI00025252	PDIA3	PDIA3 Protein disulfide-isomerase A3	1479	56747	44	4	11	11	1.349
IPI00031804	VDAC3	VDAC3 Isoform 1 of Voltage-dependent ar	1518	30639	16	4	2	2	0.317
IPI00643041	RANP1;RAN	RANP1;RAN GTP-binding nuclear protein	43	24408	1	1	1	1	1.01
IPI00017617	DDX5	DDX5 Probable ATP-dependent RNA helic	389	69105	16	3	3	3	0.615
IPI00002478	ECE1	ECE1 Isoform B of Endothelin-converting e	91	87108	2	2	2	2	0.479

Data2. AccessionNo (IPI)	iPS_CFB50/ HFF	iPS_CFB46/ H9ES	iPS_CFB50/ H9ES	iPS_CFB46/ NTU1ES	iPS_CFB50/ NTU1ES	H9ES/ HFF	NTU1ES/ HFF
IPI00329801	0.281	0.467	0.367	1.112	0.789	0.802	0.336
IPI00479058	0.701	9999	9999	4.424	2.044	-9999	0.343
IPI00011578	0.343	0.851	0.978	0.872	0.999	0.342	0.344
IPI00102685	0.273	0.983	0.873	0.871	0.77	0.299	0.345
IPI00064193	0.332	0.928	1.413	0.696	0.94	0.254	0.353
IPI00298851	0.382	1.276	1.23	0.668	1.063	0.303	0.354
IPI00295461	0.16	NaN	NaN	0.845	0.447	0.254	0.358
IPI00022418	0.329	1.143	1.241	1.007	1.036	0.264	0.364
IPI00218398	NaN	NaN	NaN	0.85	1.605	0.628	0.371
IPI00465248	0.349	0.559	0.414	1.187	0.952	0.831	0.374
IPI00005719	0.248	0.763	0.422	1.202	0.663	0.573	0.375
IPI00022434	0.371	2.531	0.724	2.784	0.758	0.571	0.376
IPI00025086	NaN	2.418	1.152	1.017	0.483	NaN	0.377
IPI00446235	0.357	1.007	1.375	0.7	0.944	0.257	0.386
IPI00930070	0.296	0.892	0.856	0.851	0.825	0.376	0.395
IPI00291928	0.443	0.747	0.98	0.938	1.116	0.414	0.397
IPI00217563	0.377	0.945	0.962	0.95	0.989	0.389	0.398
IPI00024067	0.485	0.709	0.536	1.669	1.18	0.838	0.401
IPI00550987	-9999	NaN	NaN	-9999	-9999	-9999	0.402
IPI00005107	0.545	0.544	NaN	0.87	0.709	0.504	0.405
IPI00021266	-9999	9999	NaN	1.417	-9999	-9999	0.407
IPI00009276	0.317	0.542	0.639	0.723	0.692	0.484	0.408
IPI00018248	0.302	1.02	0.518	1.314	0.701	0.568	0.411
IPI00030431	1.378	9999	9999	5.109	3.327	-9999	0.415
IPI00023860	0.693	1.286	0.779	2.755	1.663	0.868	0.417
IPI00010796	0.383	1.066	0.537	NaN	NaN	1.346	0.42
IPI00646304	0.648	0.451	0.351	1.637	1.069	1.819	0.423
IPI00028387	1.319	1.18	1.881	1.494	2.137	0.668	0.424
IPI00020557	0.391	1.217	0.929	1.091	0.84	0.373	0.435
IPI00456969	0.43	2.602	2.076	1.235	0.982	0.202	0.438
IPI00025252	0.966	0.485	0.371	2.988	2.263	2.795	0.442
IPI00031804	0.361	0.713	0.807	0.735	0.83	0.446	0.442
IPI00643041	0.737	0.976	0.718	2.246	1.648	1.001	0.448
IPI00017617	0.653	0.553	0.585	1.21	1.276	1.262	0.449
IPI00002478	0.453	1.664	1.585	1.061	1.008	0.279	0.45

Table S1 Identified proteins in the human membrane proteome of hiPSCs, hESCs and somatic foreskin fibroblast HFF.
Profiles of identified proteins in MS Experiment 2

Data2. AccessionNo (IPI)	Gene Symbol	Description	Score	Mass	# of identified (spectrum)	# of samples identified	Total peptides	# of peptides used	iPS_CFB46/ HFF
IPI00852623	PLXNB2	PLXNB2 Plexin B2	141	142609	4	1	3	3	0.266
IPI00003348	GNB2	GNB2 Guanine nucleotide-binding protein	146	37307	4	2	1	1	0.604
IPI00215948	CTNNA1	CTNNA1 Isoform 1 of Catenin alpha-1	1520	100009	27	4	7	5	1.924
IPI00013744	ITGA2	ITGA2 Integrin alpha-2	837	129214	25	1	11	10	0.352
IPI00012048	NME1	NME1 Isoform 1 of Nucleoside diphosphat	50	17138	1	1	1	1	0.43
IPI00176692	-	- 32 kDa protein	309	32142	14	3	3	2	0.295
IPI00016670	C11orf59	C11orf59 UPF0404 protein C11orf59	419	17734	10	3	3	3	0.509
IPI00166444	SFT2D3	SFT2D3 Vesicle transport protein SFT2C	81	21776	2	1	1	1	1.408
IPI00018871	ARL8B	ARL8B cDNA FLJ56285, highly similar to /	894	27235	32	4	6	5	0.479
IPI00017567	ENG	ENG Isoform Long of Endoglin	427	70533	9	1	3	3	-9999
IPI00024143	AAAS	AAAS Aladin	429	59536	14	3	3	3	0.554
IPI00030906	PHF7	PHF7 PHD finger protein 7	38	43738	1	1	1	1	0.585
IPI00215893	HMOX1	HMOX1 Heme oxygenase 1	104	32798	2	1	1	1	0.306
IPI00298289	RTN4	RTN4 Isoform 2 of Reticulon-4	2610	40293	55	4	6	5	0.497
IPI00470649	NCLN	NCLN Isoform 1 of Nicalin	1603	62935	34	4	8	8	2.15
IPI00295851	COPB1	COPB1 Coatomer subunit beta	117	107074	3	3	1	1	0.696
IPI00301280	TMEM43	TMEM43 Transmembrane protein 43	1162	44847	25	4	4	4	0.365
IPI00007752	TUBB2C	TUBB2C Tubulin beta-2C chain	7638	49799	4	2	2	2	0.429
IPI00022774	VCP	VCP Transitional endoplasmic reticulum A	331	89266	10	3	2	2	1.099
IPI00478231	RHOA	RHOA Transforming protein RhoA	50	21754	1	1	1	1	0.534
IPI00884105	LAMP1	LAMP1 Lysosome-associated membrane (	639	44854	21	4	4	4	0.451
IPI00014377	RAB32	RAB32 Ras-related protein Rab-32	163	24982	5	1	3	3	0.474
IPI00000875	TUT1;EEF1G	TUT1;EEF1G cDNA FLJ56389, highly sim	57	56114	1	1	1	1	0.754
IPI00021338	DLAT	DLAT Dihydrolipoyllysine-residue acetyltra	162	68953	3	1	1	1	0.616
IPI00019912	HSD17B4	HSD17B4 Peroxisomal multifunctional enz	645	79636	16	2	5	5	1.329
IPI00220740	NPM1	NPM1 Isoform 2 of Nucleophosmin	511	29446	9	2	2	2	1.253
IPI00221092	RPS16	RPS16 40S ribosomal protein S16	770	16435	19	4	2	2	0.841
IPI00008530	RPLP0	RPLP0 60S acidic ribosomal protein P0	43	34252	1	1	1	1	0.781
IPI00444262	NCL	NCL cDNA FLJ45706 fis, clone FEBRA20	183	65922	6	2	3	3	0.778
IPI00215918	ARF4	ARF4 ADP-ribosylation factor 4	177	20498	9	3	5	5	0.488
IPI00037619	RPL39P5	RPL39P5 Putative 60S ribosomal protein L	57	6319	4	2	1	1	1.231
IPI00032140	SERPINH1	SERPINH1 Serpin H1	5038	46411	112	4	16	12	0.9
IPI00033349	PREB	PREB Prolactin regulatory element-binding	37	45440	1	1	1	1	0.392
IPI00217519	RALA	RALA Ras-related protein Ral-A	1114	23552	43	4	7	4	0.484
IPI00749454	RFTN1	RFTN1 cDNA FLJ56561	208	86651	4	1	2	2	0.282

Data2. AccessionNo (IPI)	iPS_CFB50/ HFF	iPS_CFB46/ H9ES	iPS_CFB50/ H9ES	iPS_CFB46/ NTU1ES	iPS_CFB50/ NTU1ES	H9ES/ HFF	NTU1ES/ HFF
IPI00852623	NaN	9999	9999	0.6	0.815	-9999	0.452
IPI00003348	0.417	0.964	0.671	1.328	0.922	0.607	0.453
IPI00215948	1.126	1.403	1.772	4.344	2.512	0.942	0.459
IPI00013744	0.537	0.518	0.825	0.75	1.114	0.665	0.463
IPI00012048	-9999	0.794	-9999	0.922	-9999	0.523	0.464
IPI00176692	0.488	0.506	0.778	0.669	1.038	0.63	0.466
IPI00016670	0.522	0.747	0.769	1.092	1.124	0.58	0.467
IPI00166444	1.625	0.888	1.034	3.002	3.484	1.534	0.467
IPI00018871	0.416	1.432	1.25	1.007	0.881	0.323	0.469
IPI00017567	0.326	-9999	NaN	-9999	0.69	0.091	0.473
IPI00024143	NaN	1.173	1.076	1.072	0.866	0.471	0.473
IPI00030906	0.691	9999	9999	1.226	1.456	-9999	0.475
IPI00215893	0.358	1.39	1.638	0.641	0.753	0.213	0.476
IPI00298289	0.617	0.874	1.081	1.022	1.259	0.455	0.476
IPI00470649	0.525	1.299	1.501	0.888	1.067	0.285	0.476
IPI00295851	0.703	0.826	0.839	1.452	1.472	0.816	0.478
IPI00301280	0.472	1.122	1.46	0.733	0.977	0.318	0.485
IPI00007752	0.535	0.772	0.972	0.867	1.086	0.536	0.493
IPI00022774	0.854	0.497	0.389	2.209	1.727	2.141	0.495
IPI00478231	0.674	1.046	1.333	1.072	1.362	0.493	0.496
IPI00884105	0.419	0.913	0.857	0.893	0.837	0.478	0.503
IPI00014377	1.013	1.278	NaN	1.246	1.182	0.322	0.506
IPI00000875	0.483	0.999	0.645	1.474	0.949	0.73	0.509
IPI00021338	0.702	1.031	1.186	1.183	1.356	0.578	0.518
IPI00019912	0.871	0.306	0.256	2.112	1.865	4.458	0.52
IPI00220740	0.821	0.693	0.46	2.79	1.853	1.756	0.534
IPI00221092	0.867	0.776	0.807	1.557	1.614	1.048	0.538
IPI00008530	0.733	0.644	0.609	1.441	1.359	1.174	0.54
IPI00444262	0.624	0.584	0.473	1.431	1.154	1.288	0.541
IPI00215918	0.476	0.866	0.75	0.774	0.87	0.436	0.543
IPI00037619	1.529	9999	9999	2.25	2.809	-9999	0.545
IPI00032140	0.667	0.541	0.405	1.621	1.211	1.635	0.554
IPI00033349	0.517	0.67	0.89	0.702	0.93	0.566	0.556
IPI00217519	0.49	0.936	0.978	0.836	0.863	0.492	0.558
IPI00749454	-9999	9999	NaN	0.503	-9999	-9999	0.559

Table S1 Identified proteins in the human membrane proteome of hiPSCs, hESCs and somatic foreskin fibroblast HFF.
Profiles of identified proteins in MS Experiment 2

Data2. AccessionNo (IPI)	Gene Symbol	Description	Score	Mass	# of identified (spectrum)	# of samples identified	Total peptides	# of peptides used	iPS_CFB46/ HFF
IPI00026272	HIST1H2AE;H	HIST1H2AE;HIST1H2AB;HIST1H2AG;HIS	552	14127	15	3	2	2	0.734
IPI00328161	FKBP8	FKBP8 cDNA FLJ56370, highly similar to H	420	47142	10	3	3	3	0.752
IPI00001639	KPNB1	KPNB1 Importin subunit beta-1	92	97108	3	1	1	1	0.869
IPI00221089	RPS13	RPS13 40S ribosomal protein S13	657	17212	29	4	4	3	0.567
IPI00006666	SLC16A3	SLC16A3 Monocarboxylate transporter 4	284	49437	10	1	5	5	0.416
IPI00009328	EIF4A3	EIF4A3 Eukaryotic initiation factor 4A-III	97	46841	4	2	1	1	-9999
IPI00219518	ARL1	ARL1 ADP-ribosylation factor-like protein 1	254	20404	6	4	2	2	0.821
IPI00026302	RPL31	RPL31 60S ribosomal protein L31	195	14454	8	2	2	2	0.655
IPI00018364	RAP2B	RAP2B Ras-related protein Rap-2b	483	20491	12	4	1	1	0.417
IPI00394699	RPL17	RPL17 Putative uncharacterized protein EI	89	15278	4	3	1	1	0.849
IPI00003831	ATP2B3	ATP2B3 Isoform XB of Plasma membrane	295	134112	1	1	1	1	0.477
IPI00018274	EGFR	EGFR Isoform 1 of Epidermal growth facto	474	134190	14	1	6	6	0.459
IPI00217465	HIST1H1C	HIST1H1C Histone H1.2	117	21352	4	1	2	2	0.701
IPI00026240	BST1	BST1 ADP-ribosyl cyclase 2	157	35701	6	1	3	3	0.426
IPI00031697	TMEM109	TMEM109 Transmembrane protein 109	490	26194	12	4	1	1	0.459
IPI00217966	LDHA	LDHA L-lactate dehydrogenase	169	39812	5	1	2	2	-9999
IPI00419880	RPS3A	RPS3A 40S ribosomal protein S3a	126	29926	4	2	2	2	1.073
IPI00221093	RPS17	RPS17 40S ribosomal protein S17	506	15540	11	4	2	1	0.512
IPI00011284	COMT	COMT Isoform Membrane-bound of Catec	70	30018	2	1	2	1	0.431
IPI00008438	RPS10	RPS10 40S ribosomal protein S10	111	18886	5	2	1	1	1.285
IPI00021842	APOE	APOE Apolipoprotein E	1440	36132	41	3	8	8	0.787
IPI00219217	LDHB	LDHB L-lactate dehydrogenase B chain	344	36615	7	4	1	1	0.786
IPI00020470	GLT8D1	GLT8D1 Isoform 1 of Glycosyltransferase	38	41909	1	1	1	1	0.459
IPI00012750	RPS25	RPS25 40S ribosomal protein S25	431	13734	20	4	4	4	0.836
IPI00453473	HIST1H4K;HI	HIST1H4K;HIST1H4E;HIST2H4B;HIST1H	1404	11360	42	3	5	5	0.613
IPI00001755	GPC6	GPC6 Glypican-6	294	62695	11	4	3	2	0.979
IPI00337415	GNAI1	GNAI1 Guanine nucleotide-binding protein	614	40335	1	1	1	1	0.365
IPI00465431	LGALS3	LGALS3 Galectin-3	155	26136	7	1	5	4	0.519
IPI00149849	COG4	COG4 component of oligomeric golgi com	40	89444	1	1	1	1	0.737
IPI00383730	CACNA2D1	CACNA2D1 Neuronal voltage-dependent c	80	11298	1	1	1	1	0.871
IPI00030911	VAMP8	VAMP8 Vesicle-associated membrane pro	254	11431	10	3	3	3	NaN
IPI00026781	FASN	FASN Fatty acid synthase	660	273227	14	3	3	3	2.385
IPI00166860	DHRSX	DHRSX Dehydrogenase/reductase SDR fa	62	36420	1	1	1	1	1.111
IPI00329600	SCCPDH	SCCPDH Probable saccharopine dehydro	142	47121	5	3	2	2	1.409
IPI00302927	CCT4;ILK-2	CCT4;ILK-2 T-complex protein 1 subunit d	332	57888	11	3	4	4	1.058

Data2. AccessionNo (IPI)	iPS_CFB50/ HFF	iPS_CFB46/ H9ES	iPS_CFB50/ H9ES	iPS_CFB46/ NTU1ES	iPS_CFB50/ NTU1ES	H9ES/ HFF	NTU1ES/ HFF
IPI00026272	0.557	0.952	0.685	1.396	0.994	0.795	0.563
IPI00328161	1.072	0.853	1.197	1.27	1.775	0.847	0.563
IPI00001639	0.463	1.395	0.75	1.526	0.817	0.602	0.567
IPI00221089	0.729	0.864	1.12	0.994	1.285	0.634	0.567
IPI00006666	0.317	0.701	0.539	0.675	0.517	0.361	0.571
IPI00009328	-9999	-9999	-9999	-9999	-9999	0.584	0.571
IPI00219518	0.634	5.359	3.95	2.39	1.086	0.148	0.582
IPI00026302	0.603	0.707	0.655	1.116	1.031	0.897	0.585
IPI00018364	0.442	1.014	1.083	0.704	0.75	0.398	0.589
IPI00394699	-9999	0.683	-9999	1.432	-9999	1.203	0.591
IPI00003831	0.429	0.692	0.628	0.798	0.722	0.668	0.595
IPI00018274	0.58	NaN	NaN	0.765	0.971	0.866	0.597
IPI00217465	0.802	0.54	0.622	1.17	1.345	1.257	0.597
IPI00026240	0.716	0.869	0.651	0.788	0.681	NaN	0.599
IPI00031697	0.613	1.313	1.768	0.76	1.021	0.338	0.601
IPI00217966	0.325	-9999	0.412	-9999	0.536	0.769	0.606
IPI00419880	0.986	0.833	0.765	1.538	1.151	1.262	0.606
IPI00221093	0.72	0.804	1.138	0.83	1.179	0.61	0.613
IPI00011284	0.75	0.937	1.097	1.049	1.224	0.667	0.614
IPI00008438	1.062	0.651	0.543	2.072	1.721	1.909	0.618
IPI00021842	0.682	1.061	0.879	1.057	1.014	0.763	0.618
IPI00219217	0.578	0.472	0.35	1.267	0.937	1.611	0.618
IPI00020470	0.664	0.805	1.174	0.738	1.073	0.552	0.62
IPI00012750	0.852	0.715	0.752	1.339	1.37	1.117	0.622
IPI00453473	0.553	0.844	0.738	0.951	0.885	0.728	0.622
IPI00001755	0.481	0.778	0.485	0.522	0.339	0.502	0.624
IPI00337415	0.311	1.956	1.684	0.577	0.496	0.18	0.629
IPI00465431	0.316	9999	9999	0.822	0.859	-9999	0.629
IPI00149849	0.603	0.788	0.65	1.161	0.955	0.904	0.632
IPI00383730	0.793	1.888	1.732	1.372	1.254	0.446	0.632
IPI00030911	0.681	0.769	0.55	0.726	0.809	NaN	0.633
IPI00026781	1.783	7.642	4.569	3.675	2.748	0.38	0.634
IPI00166860	1.527	9999	9999	1.739	2.402	-9999	0.636
IPI00329600	1.504	9999	9999	2.206	2.367	-9999	0.636
IPI00302927	0.944	0.798	0.826	1.159	1.201	0.919	0.637

Table S1 Identified proteins in the human membrane proteome of hiPSCs, hESCs and somatic foreskin fibroblast HFF.
Profiles of identified proteins in MS Experiment 2

Data2. AccessionNo (IPI)	Gene Symbol	Description	Score	Mass	# of identified (spectrum)	# of samples identified	Total peptides	# of peptides used	iPS_CFB46/ HFF
IPI00396378	HNRNPA2B1	HNRNPA2B1 Isoform B1 of Heterogeneou	502	37407	22	3	4	3	0.711
IPI00017292	CTNNB1	CTNNB1 Isoform 1 of Catenin beta-1	641	85442	17	3	6	5	1.606
IPI00288947	GNAQ	GNAQ Guanine nucleotide-binding protein	229	42115	7	4	1	1	0.502
IPI00217490	FNDC3B	FNDC3B Isoform 1 of Fibronectin type III c	394	132803	9	3	1	1	1.706
IPI00291417	DCAKD	DCAKD Isoform 1 of Dephospho-CoA kina	315	26533	14	3	3	3	0.411
IPI00004962	GOLIM4	GOLIM4 Golgi integral membrane protein	471	81831	10	4	1	1	0.757
IPI00409635	ESYT2	ESYT2 Isoform 2 of Extended synaptotagr	70	98840	2	1	2	2	0.547
IPI00027438	FLOT1	FLOT1 Flotillin-1	1241	47326	34	4	10	9	0.676
IPI00016372	RAB9A	RAB9A Ras-related protein Rab-9A	112	22823	4	2	3	3	0.106
IPI00296099	THBS1	THBS1 Thrombospondin-1	466	129300	11	1	4	4	0.828
IPI00550523	ATL3	ATL3 Isoform 1 of Atlastin-3	228	60503	6	1	4	4	0.721
IPI00384016	DLSTP;DLST	DLSTP;DLST Full-length cDNA 5-PRIME	233	29606	6	2	2	2	0.663
IPI00011454	GANAB	GANAB Isoform 2 of Neutral alpha-glucosi	1696	109369	46	3	13	11	1.873
IPI00290928	GNA13	GNA13 Guanine nucleotide-binding protein	333	44022	4	3	2	2	0.623
IPI00219111	TRAM1	TRAM1 Translocating chain-associated m	248	43044	3	1	1	1	0.484
IPI00385495	LMF2	LMF2 Isoform 1 of Lipase maturation facto	125	79647	3	1	1	1	0.517
IPI00001891	AUP1	AUP1 Isoform Long of Ancient ubiquitous	89	52995	4	2	1	1	0.741
IPI00395903	TMEM106B	TMEM106B Transmembrane protein 106E	88	31108	2	1	1	1	0.717
IPI00012772	RPL8	RPL8 60S ribosomal protein L8	652	28007	24	4	5	4	0.503
IPI00021840	RPS6	RPS6 40S ribosomal protein S6	689	28663	16	4	3	3	0.784
IPI00025491	EIF4A1;SNOF	EIF4A1;SNORA67 Eukaryotic initiation fac	174	46125	6	3	1	1	0.897
IPI00215780	RPS19	RPS19 40S ribosomal protein S19	235	16051	10	3	3	3	1.478
IPI00008318	EPHA4	EPHA4 Ephrin type-A receptor 4	65	109790	2	1	1	1	0.686
IPI00217143	SDHA	SDHA 57 kDa protein	119	56716	3	1	1	1	1.44
IPI00456429	UBA52	UBA52 ubiquitin and ribosomal protein L4(	1089	14719	26	4	2	2	0.427
IPI00299571	PDIA6	PDIA6 Isoform 2 of Protein disulfide-isome	2696	53867	50	4	9	8	2.103
IPI00789008	FLOT2	FLOT2 Flotillin-2	1383	47035	38	4	11	10	0.707
IPI00748145	GNAI2	GNAI2 Isoform 1 of Guanine nucleotide-bi	2776	40425	69	4	10	7	0.512
IPI00021695	ATP2B1	ATP2B1 Isoform D of Plasma membrane	558	138668	6	1	3	3	0.669
IPI00027230	HSP90B1	HSP90B1 Endoplasmin	3065	92411	70	4	16	15	1.725
IPI00005202	PGRMC2	PGRMC2 Membrane-associated progeste	1337	26154	23	4	4	4	0.433
IPI00328243	PLD3	PLD3 Phospholipase D3	836	54671	31	4	3	3	0.708
IPI00221088	RPS9	RPS9 40S ribosomal protein S9	378	22578	31	4	5	5	0.798
IPI00008964	RAB1B	RAB1B Ras-related protein Rab-1B	2391	22157	59	4	6	5	0.402
IPI00410488	CD276	CD276 Isoform 1 of CD276 antigen	115	57199	2	1	2	2	0.434

Data2. AccessionNo (IPI)	iPS_CFB50/ HFF	iPS_CFB46/ H9ES	iPS_CFB50/ H9ES	iPS_CFB46/ NTU1ES	iPS_CFB50/ NTU1ES	H9ES/ HFF	NTU1ES/ HFF
IPI00396378	0.798	0.341	0.451	0.86	1.132	1.818	0.637
IPI00017292	0.901	0.978	0.575	2.284	1.273	1.748	0.638
IPI00288947	0.492	1.398	1.381	0.782	0.77	0.347	0.639
IPI00217490	2.271	6.179	8.293	2.638	3.53	0.267	0.644
IPI00291417	0.593	0.376	0.57	0.67	0.95	1.52	0.645
IPI00004962	0.98	9999	9999	1.165	1.517	-9999	0.647
IPI00409635	1.202	0.726	1.608	0.841	1.856	0.729	0.648
IPI00027438	0.64	0.945	0.955	1.016	0.991	0.656	0.654
IPI00016372	0.145	9999	9999	0.085	0.573	-9999	0.66
IPI00296099	2.225	0.514	1.392	0.885	2.391	1.56	0.671
IPI00550523	1.035	0.578	0.628	1.011	1.233	1.708	0.675
IPI00384016	0.637	0.958	0.93	0.974	0.946	0.667	0.677
IPI00011454	1.576	0.659	0.589	2.677	2.45	2.746	0.679
IPI00290928	0.356	1.066	0.615	0.913	0.525	0.566	0.68
IPI00219111	0.42	1.267	1.109	0.706	0.616	0.37	0.683
IPI00385495	0.845	9999	9999	0.753	1.238	-9999	0.683
IPI00001891	0.683	9999	9999	1.075	0.997	-9999	0.686
IPI00395903	0.787	1.533	1.697	1.035	1.142	0.452	0.69
IPI00012772	0.474	0.816	0.787	0.658	0.651	0.607	0.693
IPI00021840	0.715	0.95	0.881	1.128	1.029	0.806	0.693
IPI00025491	0.802	0.827	0.746	1.271	1.143	1.049	0.702
IPI00215780	1.752	0.721	0.96	2.091	2.49	1.781	0.704
IPI00008318	0.681	9999	9999	0.968	0.967	-9999	0.705
IPI00217143	-9999	0.328	-9999	2.027	-9999	4.246	0.707
IPI00456429	0.579	0.639	0.874	0.6	0.816	0.646	0.71
IPI00299571	1.868	0.535	0.449	2.922	2.701	3.788	0.714
IPI00789008	0.491	0.985	0.678	1	0.693	0.706	0.714
IPI00748145	0.571	0.998	1.048	0.703	0.779	0.524	0.721
IPI00021695	0.609	1.312	1.255	0.835	0.791	0.383	0.722
IPI00027230	1.36	0.513	0.394	2.319	1.699	3.293	0.722
IPI00005202	0.727	0.758	1.176	0.766	1.161	0.553	0.727
IPI00328243	0.878	0.99	1.179	0.939	1.117	0.666	0.731
IPI00221088	0.891	0.769	0.777	1.113	1.214	0.993	0.736
IPI00008964	0.723	0.624	1.094	0.546	0.988	0.646	0.737
IPI00410488	0.633	0.735	1.078	0.58	0.857	0.567	0.737

Table S1 Identified proteins in the human membrane proteome of hiPSCs, hESCs and somatic foreskin fibroblast HFF.
Profiles of identified proteins in MS Experiment 2

Data2. AccessionNo (IPI)	Gene Symbol	Description	Score	Mass	# of identified (spectrum)	# of samples identified	Total peptides	# of peptides used	iPS_CFB46/ HFF
IPI00002790	SEL1L	SEL1L Isoform 1 of Protein sel-1 homolog	164	88698	5	3	1	1	0.589
IPI00220835	SEC61B	SEC61B Protein transport protein Sec61 s	258	9968	11	3	2	2	0.622
IPI00008495	MT-ND4	MT-ND4 NADH-ubiquinone oxidoreductas	42	51677	1	1	1	1	0.516
IPI00029954	VEZT	VEZT Isoform 1 of Vezatin	47	88609	1	1	1	1	0.431
IPI00043598	IKIP	IKIP Isoform 4 of Inhibitor of nuclear factor	353	43057	7	1	3	3	0.686
IPI00013683	TUBB3	TUBB3 Tubulin beta-3 chain	6177	50400	1	1	1	1	-9999
IPI00011253	RPS3	RPS3 40S ribosomal protein S3	559	26671	29	4	6	6	0.949
IPI00794169	ANO10	ANO10 Putative uncharacterized protein A	204	76408	9	4	2	2	0.499
IPI00141318	CKAP4	CKAP4 Isoform 1 of Cytoskeleton-associa	9888	65983	212	4	31	23	0.627
IPI00003865	HSPA8	HSPA8 Isoform 1 of Heat shock cognate 7	4862	70854	103	4	15	11	0.827
IPI00412607	RPL35	RPL35 60S ribosomal protein L35	172	14543	7	4	1	1	0.722
IPI00008557	IGF2BP1	IGF2BP1 Insulin-like growth factor 2 mRN	162	63417	3	2	2	2	1.234
IPI00010153	RPL23	RPL23 60S ribosomal protein L23	544	14856	12	4	1	1	1.039
IPI00022143	ESYT1	ESYT1 Isoform 1 of Extended synaptotagr	4924	122780	119	4	18	16	0.729
IPI00019770	FAU	FAU ubiquitin-like protein fubi and ribosom	130	14381	3	1	1	1	1.592
IPI00012493	RPS20	RPS20 40S ribosomal protein S20	445	13364	20	4	3	2	1.254
IPI00902496	SEC63	SEC63 cDNA FLJ14468 fis, clone MAMM	741	47900	33	4	6	6	1.341
IPI00376798	RPL11	RPL11 Isoform 1 of 60S ribosomal protein	437	20240	8	3	2	2	1.044
IPI00031169	RAB2A	RAB2A Ras-related protein Rab-2A	1882	23531	41	4	7	6	0.708
IPI00218924	CHP	CHP Calcium-binding protein p22	207	22442	7	3	1	1	0.834
IPI00410034	SLC38A2	SLC38A2 Isoform 1 of Sodium-coupled ne	234	55990	7	3	2	2	0.641
IPI00023780	DNAJC5	DNAJC5 Isoform 2 of DnaJ homolog subfa	221	18789	4	1	2	1	0.905
IPI00303726	IFITM3	IFITM3 Interferon-induced transmembrane	847	14622	18	4	4	2	0.369
IPI00010438	SNAP23	SNAP23 Isoform SNAP-23a of Synaptosoı	774	23340	23	4	4	4	0.672
IPI00328170	MOGS	MOGS Mannosyl-oligosaccharide glucosid	2221	91861	50	4	8	7	0.549
IPI00174794	PTK7	PTK7 Isoform 5 of Tyrosine-protein kinase	341	89707	11	3	2	2	0.617
IPI00296157	RETSAT	RETSAT Isoform 1 of All-trans-retinol 13,1	95	66777	3	2	2	2	0.702
IPI00005737	SURF4	SURF4 Isoform 1 of Surfeit locus protein 4	3061	30374	45	4	6	4	0.783
IPI00396321	LRRC59	LRRC59 Leucine-rich repeat-containing pr	2227	34909	66	4	8	5	1.11
IPI00411639	RPSA;RPSAF	RPSA;RPSAP15 Laminin receptor-like pro	169	32975	4	1	2	2	1.366
IPI00329389	RPL6	RPL6 60S ribosomal protein L6	1546	32708	37	4	5	5	1.515
IPI00005181	PLSCR1	PLSCR1 Phospholipid scramblase 1	70	35026	2	2	1	1	0.733
IPI00007730	C14orf1	C14orf1 Probable ergosterol biosynthetic p	285	15854	10	3	2	2	0.528
IPI00013485	RPS2	RPS2 40S ribosomal protein S2	569	31305	17	4	2	2	1.19
IPI00009896	EPHX1	EPHX1 Epoxide hydrolase 1	1642	52915	40	4	6	5	0.931

Data2. AccessionNo (IPI)	iPS_CFB50/ HFF	iPS_CFB46/ H9ES	iPS_CFB50/ H9ES	iPS_CFB46/ NTU1ES	iPS_CFB50/ NTU1ES	H9ES/ HFF	NTU1ES/ HFF
IPI00002790	0.927	0.618	0.981	0.792	1.253	0.922	0.741
IPI00220835	0.762	0.922	0.897	0.838	1.029	0.858	0.741
IPI00008495	0.488	9999	9999	0.688	0.654	-9999	0.746
IPI00029954	0.545	0.95	1.21	0.576	0.732	0.439	0.746
IPI00043598	0.68	0.701	0.7	0.914	0.868	0.947	0.747
IPI00013683	0.741	NaN	9999	-9999	0.988	-9999	0.751
IPI00011253	0.972	0.787	0.771	1.292	1.263	1.36	0.753
IPI00794169	0.678	0.894	1.226	0.658	0.898	0.541	0.755
IPI00141318	0.726	1.189	1.405	0.843	0.997	0.533	0.763
IPI00003865	0.971	0.81	0.947	1.083	1.268	0.998	0.765
IPI00412607	0.587	9999	9999	0.94	0.768	-9999	0.765
IPI00008557	1.035	2.128	1.798	1.601	1.349	0.561	0.767
IPI00010153	1.219	0.7	0.828	1.349	1.591	1.436	0.767
IPI00022143	0.936	0.901	1.173	0.934	1.221	0.716	0.767
IPI00019770	1.835	1.139	1.325	2.049	2.375	1.352	0.773
IPI00012493	1.673	0.83	1.121	1.361	2.002	1.459	0.776
IPI00902496	1.746	0.827	1.072	0.732	0.947	0.849	0.785
IPI00376798	0.841	0.69	0.56	1.313	1.088	1.466	0.787
IPI00031169	0.762	1.19	1.29	0.891	0.968	0.576	0.788
IPI00218924	0.784	9999	9999	1.044	0.988	-9999	0.795
IPI00410034	0.907	1.621	1.827	1.204	1.131	0.383	0.8
IPI00023780	0.429	1.363	0.402	1.104	0.513	1.024	0.801
IPI00303726	0.499	0.657	0.742	0.422	0.612	0.607	0.805
IPI00010438	0.679	1.016	1.038	0.829	0.844	0.64	0.806
IPI00328170	0.848	1.145	1.621	0.703	0.998	1.541	0.806
IPI00174794	0.761	0.939	1.168	0.759	0.942	0.636	0.808
IPI00296157	0.807	1.441	1.672	0.862	0.997	0.47	0.81
IPI00005737	0.725	1.609	1.275	0.962	0.928	0.541	0.811
IPI00396321	1.482	1.065	1.397	0.888	1.163	1.026	0.813
IPI00411639	1.016	0.453	0.327	1.668	1.248	3.033	0.815
IPI00329389	1.037	1.068	0.756	1.415	1.058	1.217	0.821
IPI00005181	0.49	1.179	0.794	0.884	0.594	0.602	0.826
IPI00007730	0.678	0.842	0.987	0.716	0.858	0.731	0.828
IPI00013485	0.898	0.842	0.641	1.425	1.081	1.367	0.831
IPI00009896	0.9	1.522	1.475	1.113	1.081	0.595	0.832

Table S1 Identified proteins in the human membrane proteome of hiPSCs, hESCs and somatic foreskin fibroblast HFF.
Profiles of identified proteins in MS Experiment 2

Data2. AccessionNo (IPI)	Gene Symbol	Description	Score	Mass	# of identified (spectrum)	# of samples identified	Total peptides	# of peptides used	iPS_CFB46/ HFF
IPI00219155	RPL27	RPL27 60S ribosomal protein L27	313	15788	14	4	3	2	0.897
IPI00036552	ANTXR2	ANTXR2 Isoform 2 of Anthrax toxin recept	63	42892	2	1	2	2	-9999
IPI00387077	SLC12A9	SLC12A9 Isoform 1 of Solute carrier family	91	96049	3	1	2	2	0.576
IPI00291695	GPX8	GPX8 Probable glutathione peroxidase 8	221	23894	14	3	3	3	0.709
IPI00217600	PNPLA6	PNPLA6 Isoform 2 of Neuropathy target es	231	146123	7	3	2	2	0.859
IPI00012486	SPNS1	SPNS1 61 kDa protein	64	61067	1	1	1	1	-9999
IPI00218200	BCAP31	BCAP31 B-cell receptor-associated protein	411	27974	18	4	3	3	0.784
IPI00183666	TRPV2	TRPV2 Transient receptor potential cation	155	85926	5	1	3	3	1.143
IPI00032825	TMED7;TMED	TMED7;TMED7-TICAM2;TICAM2 Transm	250	25156	11	4	1	1	0.713
IPI00063544	YIF1B	YIF1B Isoform 3 of Protein YIF1B	363	33943	10	4	1	1	0.718
IPI00180675	TUBA1A	TUBA1A Tubulin alpha-1A chain	6379	50104	10	4	1	1	0.32
IPI00295098	SRPRB	SRPRB Signal recognition particle recepto	1486	29684	39	4	7	7	0.69
IPI00306332	RPL24	RPL24 60S ribosomal protein L24	1095	17768	39	4	4	3	0.896
IPI00215997	CD9	CD9 CD9 antigen	127	25399	5	2	1	1	0.621
IPI00014577	RAB18	RAB18 Ras-related protein Rab-18	381	22963	15	4	3	3	0.863
IPI00218466	SEC61A1	SEC61A1 cDNA FLJ59739, highly similar t	2026	52915	55	4	10	6	1.044
IPI00303476	ATP5B	ATP5B ATP synthase subunit beta, mitoch	1246	56525	31	3	10	10	2.64
IPI00019353	AGK	AGK Isoform 1 of Acylglycerol kinase, mitc	339	47107	9	4	1	1	0.663
IPI00013296	RPS18;RPS1	RPS18;RPS18P9 40S ribosomal protein S	1091	17708	43	4	10	8	0.686
IPI00007765	HSPA9	HSPA9 Stress-70 protein, mitochondrial	838	73635	27	4	7	7	4.096
IPI00003362	HSPA5	HSPA5 HSPA5 protein	6178	72377	160	4	23	20	1.454
IPI00554723	RPL10	RPL10 60S ribosomal protein L10	450	24588	16	3	3	3	1.041
IPI00152441	HM13	HM13 Isoform 1 of Minor histocompatibility	463	41462	12	4	1	1	0.604
IPI00019359	KRT9	KRT9 Keratin, type I cytoskeletal 9	5102	62027	106	4	19	16	1.224
IPI00020472	TMEM111	TMEM111 Isoform 1 of Transmembrane p	299	29932	10	3	3	3	0.771
IPI00304962	COL1A2	COL1A2 Collagen alpha-2(I) chain	73	129209	1	1	1	1	1.769
IPI00000948	TBL2	TBL2 Transducin beta-like protein 2	102	49766	2	1	1	1	0.767
IPI00017964	SNRPD3	SNRPD3 Small nuclear ribonucleoprotein S	127	13907	4	3	2	1	1.055
IPI00247583	RPL21P19;RF	RPL21P19;RPL21 60S ribosomal protein L	670	18553	23	4	4	3	0.934
IPI00784376	MTP18	MTP18 Mitochondrial 18 kDa protein	46	17999	1	1	1	1	0.825
IPI00007940	ERLIN1	ERLIN1 ER lipid raft associated 1	904	39146	16	4	2	2	1.11
IPI00002236	MFGE8	MFGE8 Lactadherin	797	43095	24	4	4	3	1.416
IPI00029133	ATP5F1	ATP5F1 ATP synthase subunit b, mitochoi	860	28890	27	4	9	8	0.863
IPI00028481	RAB8A	RAB8A Ras-related protein Rab-8A	1842	23653	10	4	2	2	0.713
IPI00787853	IMPAD1	IMPAD1 Inositol monophosphatase 3	53	38657	1	1	1	1	2.006

Data2. AccessionNo (IPI)	iPS_CFB50/ HFF	iPS_CFB46/ H9ES	iPS_CFB50/ H9ES	iPS_CFB46/ NTU1ES	iPS_CFB50/ NTU1ES	H9ES/ HFF	NTU1ES/ HFF
IPI00219155	1.118	0.886	1.114	1.069	1.339	0.98	0.836
IPI00036552	-9999	NaN	NaN	-9999	-9999	-9999	0.838
IPI00387077	0.644	1	1.109	0.705	0.78	0.577	0.842
IPI00291695	0.823	0.728	0.851	0.837	0.974	0.952	0.844
IPI00217600	1.168	1.044	1.478	1.006	1.369	0.83	0.848
IPI00012486	-9999	NaN	NaN	-9999	-9999	-9999	0.856
IPI00218200	0.921	0.676	0.829	0.903	1.066	1.153	0.865
IPI00183666	0.846	0.737	0.569	1.314	0.916	1.501	0.866
IPI00032825	0.721	0.967	0.986	0.809	0.822	0.714	0.878
IPI00063544	0.998	0.758	1.062	0.814	1.137	0.917	0.878
IPI00180675	0.657	1.056	2.186	0.361	0.745	0.293	0.882
IPI00295098	0.86	1.31	1.718	0.776	0.976	0.716	0.884
IPI00306332	0.939	1.025	1.064	0.736	1.01	0.862	0.884
IPI00215997	0.697	0.692	0.783	0.699	0.788	0.869	0.885
IPI00014577	0.959	1.209	1.354	0.964	1.075	0.689	0.89
IPI00218466	0.879	1.043	0.935	1.017	0.854	1.162	0.89
IPI00303476	1.437	0.176	0.099	2.709	1.534	14.659	0.891
IPI00019353	0.564	0.749	0.643	0.733	0.627	0.857	0.9
IPI00013296	0.864	0.89	1.132	0.749	0.909	0.744	0.902
IPI00007765	1.526	0.487	0.406	2.631	1.743	3.754	0.905
IPI00003362	1.285	0.756	0.681	1.525	1.394	1.885	0.908
IPI00554723	1.059	0.826	0.844	1.14	1.162	1.219	0.909
IPI00152441	0.728	0.697	0.847	0.656	0.795	0.839	0.917
IPI00019359	1.005	1.399	1.146	1.395	1.134	0.866	0.924
IPI00020472	0.907	1.231	1.456	0.805	0.944	0.598	0.937
IPI00304962	1.751	1.493	1.49	1.861	1.852	1.146	0.946
IPI00000948	1.088	1.313	1.878	0.798	1.138	0.565	0.957
IPI00017964	-9999	0.401	-9999	1.093	-9999	2.548	0.961
IPI00247583	1.223	1.041	1.402	0.967	1.271	0.879	0.962
IPI00784376	-9999	0.734	-9999	0.853	-9999	1.088	0.963
IPI00007940	1.599	0.989	1.492	0.817	1.221	0.541	0.977
IPI00002236	1.493	1.2	1.369	1.327	1.507	0.856	0.979
IPI00029133	0.85	0.768	0.741	0.793	0.748	0.876	0.982
IPI00028481	0.74	0.697	0.725	0.721	0.749	0.989	0.984
IPI00787853	1.342	2.368	1.598	2.01	1.352	0.819	0.994

Table S1 Identified proteins in the human membrane proteome of hiPSCs, hESCs and somatic foreskin fibroblast HFF.
Profiles of identified proteins in MS Experiment 2

Data2. AccessionNo (IPI)	Gene Symbol	Description	Score	Mass	# of identified (spectrum)	# of samples identified	Total peptides	# of peptides used	iPS_CFB46/ HFF
IPI00009890	SERPINE2	SERPINE2 Glia-derived nexin	224	43974	5	1	2	1	0.545
IPI00024933	RPL12	RPL12 Isoform 1 of 60S ribosomal protein	482	17808	19	4	2	2	1.056
IPI00784119	ATP6AP1	ATP6AP1 V-type proton ATPase subunit S	184	51993	8	2	3	3	2.047
IPI00003635	ERGIC1	ERGIC1 cDNA FLJ56280, highly similar to	291	26242	6	1	3	3	0.711
IPI00016513	RAB10	RAB10 Ras-related protein Rab-10	1791	22527	12	4	1	1	0.71
IPI00029750	RPS24	RPS24 Isoform 1 of 40S ribosomal protein	596	15413	18	4	3	2	0.917
IPI00790135	OSBPL8	OSBPL8 79 kDa protein	314	79160	11	4	2	2	0.532
IPI00016786	CDC42	CDC42 Isoform 2 of Cell division control pı	133	21245	4	1	2	1	0.891
IPI00216237	RPL36	RPL36 60S ribosomal protein L36	679	12246	29	4	4	3	0.887
IPI00645518	CDIPT	CDIPT Isoform 1 of CDP-diacylglycerol--in	483	23523	16	4	3	3	0.738
IPI00020124	PI4K2A	PI4K2A Phosphatidylinositol 4-kinase type	221	53989	8	2	4	4	0.86
IPI00007166	IER3IP1	IER3IP1 Immediate early response 3-intera	832	8963	13	4	2	2	1.417
IPI00328715	MTDH	MTDH Protein LYRIC	230	63799	9	4	3	2	0.927
IPI00009976	TMED1	TMED1 Transmembrane emp24 domain-c	49	25190	1	1	1	1	1.506
IPI00009950	LMAN2	LMAN2 Vesicular integral-membrane prote	469	40203	14	3	3	3	1.296
IPI00013930	STX6	STX6 Syntaxin-6	128	29158	2	2	1	1	1.138
IPI00217030	RPS4X	RPS4X 40S ribosomal protein S4, X isofor	701	29579	23	4	5	5	0.99
IPI00025329	RPL19	RPL19 60S ribosomal protein L19	576	23451	13	4	3	3	1.264
IPI00013415	RPS7	RPS7 40S ribosomal protein S7	1196	22113	27	4	5	4	1.256
IPI00216587	RPS8	RPS8 40S ribosomal protein S8	1146	24190	26	4	4	4	0.909
IPI00221354	FUS	FUS Isoform Short of RNA-binding protein	158	53323	4	1	2	2	0.243
IPI00001091	AFG3L2	AFG3L2 AFG3-like protein 2	247	88528	8	3	3	3	0.448
IPI00005068	SLC44A1	SLC44A1 Isoform 2 of Choline transporter-	131	73247	7	4	1	1	-9999
IPI00220327	KRT1	KRT1 Keratin, type II cytoskeletal 1	7071	65999	195	4	22	20	0.968
IPI00290085	CDH2	CDH2 Cadherin-2	426	99747	12	2	4	4	1.257
IPI00026530	LMAN1	LMAN1 Protein ERGIC-53	1396	57513	44	4	8	6	0.535
IPI00549761	ALG1	ALG1 Chitobiosyldiphosphodolichol beta-n	1089	52484	14	4	2	2	0.716
IPI00410079	FAM82A2	FAM82A2 Isoform 1 of Regulator of microt	55	52086	1	1	1	1	1.372
IPI00017767	MGST2	MGST2 Microsomal glutathione S-transfer	212	16610	5	3	1	1	2.784
IPI00011200	PHGDH	PHGDH D-3-phosphoglycerate dehydroge	504	56614	16	3	6	6	1.28
IPI00550021	RPL3	RPL3 60S ribosomal protein L3	682	46080	23	4	7	5	1.088
IPI00031131	C20orf3	C20orf3 Isoform 1 of Adipocyte plasma me	1980	46451	81	4	12	8	0.818
IPI00013475	TUBB2A	TUBB2A Tubulin beta-2A chain	8046	49875	20	3	3	2	1.299
IPI00009346	TMEM14C	TMEM14C Transmembrane protein 14C	419	11557	13	4	3	2	1.364
IPI00291006	MDH2	MDH2 Malate dehydrogenase, mitochondr	49	35481	2	1	1	1	1.541

Data2. AccessionNo (IPI)	iPS_CFB50/ HFF	iPS_CFB46/ H9ES	iPS_CFB50/ H9ES	iPS_CFB46/ NTU1ES	iPS_CFB50/ NTU1ES	H9ES/ HFF	NTU1ES/ HFF
IPI00009890	0.46	0.742	0.637	0.97	0.83	1.264	0.995
IPI00024933	1.069	0.952	0.973	1.055	1.075	1.072	0.996
IPI00784119	1.NaN	0.939	0.719	2.031	1.551	2.11	1.004
IPI00003635	0.732	1.026	0.927	0.704	0.708	0.767	1.008
IPI00016513	0.866	1.411	1.735	0.699	0.857	0.487	1.012
IPI00029750	0.867	1.392	1.221	1	0.856	0.637	1.015
IPI00790135	0.823	0.65	1.01	0.83	1.638	0.802	1.019
IPI00016786	0.646	1.116	1.547	0.87	0.634	0.58	1.02
IPI00216237	1.247	0.961	1.262	1.003	1.304	0.872	1.021
IPI00645518	0.925	0.725	0.947	0.755	0.907	0.905	1.022
IPI00020124	0.894	1.384	1.418	0.797	0.845	0.65	1.025
IPI00007166	1.225	1.146	1.211	1.417	0.908	1.188	1.026
IPI00328715	1.222	1.038	1.06	0.909	0.96	0.865	1.032
IPI00009976	1.845	1.102	1.361	1.447	1.783	1.323	1.036
IPI00009950	1.45	0.717	0.918	0.913	0.953	0.92	1.04
IPI00013930	0.932	0.779	0.643	1.088	0.896	1.413	1.041
IPI00217030	0.89	0.82	0.768	0.951	0.877	1.125	1.041
IPI00025329	1.324	0.805	0.849	1.196	1.257	1.516	1.055
IPI00013415	1.222	0.995	0.972	1.176	1.148	1.23	1.061
IPI00216587	0.68	0.919	0.821	0.869	0.774	0.972	1.064
IPI00221354	1.03	0.303	1.272	0.227	0.968	0.79	1.065
IPI00001091	0.519	0.97	0.94	0.693	0.675	0.44	1.066
IPI00005068	-9999	NaN	NaN	-9999	-9999	-9999	1.066
IPI00220327	0.816	0.916	0.781	0.89	0.755	1.023	1.069
IPI00290085	0.795	1.172	1.319	1.116	1.002	1.097	1.081
IPI00026530	0.95	0.852	1.409	0.7	1.044	0.852	1.087
IPI00549761	0.784	1.082	0.982	0.655	0.721	0.741	1.088
IPI00410079	2.202	1.216	1.967	1.242	2.004	1.092	1.1
IPI00017767	1.926	3.981	2.777	2.518	1.751	0.677	1.101
IPI00011200	1.181	0.49	0.455	1.154	1.094	2.589	1.103
IPI00550021	1.042	0.76	0.733	0.983	0.946	1.385	1.104
IPI00031131	1.191	0.985	1.453	0.753	1.063	0.751	1.112
IPI00013475	0.962	1.29	0.954	1.165	0.865	0.989	1.114
IPI00009346	1.839	1.506	2.155	1.214	1.647	0.961	1.117
IPI00291006	1.507	0.224	0.221	1.37	1.347	6.648	1.12

Table S1 Identified proteins in the human membrane proteome of hiPSCs, hESCs and somatic foreskin fibroblast HFF.
Profiles of identified proteins in MS Experiment 2

Data2. AccessionNo (IPI)	Gene Symbol	Description	Score	Mass	# of identified (spectrum)	# of samples identified	Total peptides	# of peptides used	iPS_CFB46/ HFF
IPI00294472	TMED5	TMED5 Transmembrane emp24 domain-c	101	25988	6	3	1	1	0.811
IPI00171903	HNRNPM	HNRNPM Isoform 1 of Heterogeneous nuc	134	77464	5	2	4	3	1.383
IPI00015102	ALCAM	ALCAM Isoform 1 of CD166 antigen	129	65061	6	1	3	3	1.02
IPI00646625	TAP1	TAP1 Antigen peptide transporter 1	68	87163	2	1	1	1	0.678
IPI00301202	MAGT1	MAGT1 magnesium transporter 1	81	41504	6	3	2	2	0.881
IPI00008338	ABCC1	ABCC1 Isoform 2 of Multidrug resistance-a	80	164835	3	2	2	2	1.055
IPI00002070	LRRC8A	LRRC8A Leucine-rich repeat-containing pr	252	94139	4	3	1	1	3.293
IPI00397526	MYH10	MYH10 Isoform 1 of Myosin-10	614	228858	6	1	2	2	1.858
IPI00793443	IPO5	IPO5 Isoform 1 of Importin-5	53	123550	1	1	1	1	2.54
IPI00031030	APLP2	APLP2 Isoform 1 of Amyloid-like protein 2	91	86900	1	1	1	1	3.499
IPI00016608	TMED2	TMED2 Transmembrane emp24 domain-c	2397	22746	61	4	10	5	1.265
IPI00023135	GOSR2	GOSR2 Isoform A of Golgi SNAP receptor	394	24760	10	4	1	1	1.61
IPI00006433	DNAJC16	DNAJC16 Isoform 1 of DnaJ homolog sub	289	90534	7	3	1	1	2.222
IPI00022018	DPM1	DPM1 Dolichol-phosphate mannosyltransf	192	29616	6	2	2	2	1.24
IPI00030179	RPL7P32;RPI	RPL7P32;RPL7 60S ribosomal protein L7	485	29207	22	3	6	5	0.947
IPI00028116	KDELR1	KDELR1 ER lumen protein retaining recep	347	24526	9	3	3	3	0.996
IPI00013271	DERL1	DERL1 Derlin-1	128	28782	5	3	2	2	0.899
IPI00010491	RAB27B	RAB27B Ras-related protein Rab-27B	49	24592	1	1	1	1	1.084
IPI00177817	ATP2A2	ATP2A2 Isoform SERCA2A of Sarcoplasm	8173	109620	193	4	30	20	0.965
IPI00218343	TUBA1C	TUBA1C Tubulin alpha-1C chain	6976	49863	139	4	15	11	0.763
IPI00550165	DHRS7B	DHRS7B Dehydrogenase/reductase SDR	162	35097	4	3	2	2	2.174
IPI00554711	JUP	JUP Junction plakoglobin	311	81693	7	3	2	2	1.326
IPI00645667	MBOAT7	MBOAT7 Isoform 3 of Lysophospholipid ac	1216	38445	25	4	3	3	0.83
IPI00299573	RPL7A	RPL7A 60S ribosomal protein L7a	1435	29977	42	4	6	6	0.871
IPI00414676	HSP90AB1	HSP90AB1 Heat shock protein HSP 90-be	2508	83212	52	4	11	9	1.717
IPI00031397	ACSL3	ACSL3 Long-chain-fatty-acid--CoA ligase 3	323	80368	7	2	3	3	0.69
IPI00555744	RPL14	RPL14 Ribosomal protein L14 variant	725	23772	15	4	2	2	1.066
IPI00100656	TECR	TECR Isoform 1 of Synaptic glycoprotein S	1059	36011	33	4	4	4	0.94
IPI00289819	IGF2R	IGF2R Cation-independent mannose-6-ph	492	274100	16	4	2	2	0.998
IPI00021983	NCSTN	NCSTN Isoform 1 of Nicastrin	1021	78362	29	4	5	5	1.178
IPI00456758	RPL27A	RPL27A 60S ribosomal protein L27a	835	16551	19	4	2	2	1.017
IPI00022744	CSE1L	CSE1L Isoform 1 of Exportin-2	214	110346	7	2	2	2	2.008
IPI00020599	CALR	CALR Calreticulin	425	48112	11	2	3	3	1.093
IPI00470528	RPL15	RPL15 60S ribosomal protein L15	251	24131	11	2	3	3	1.712
IPI00465361	RPL13	RPL13 60S ribosomal protein L13	1389	24247	53	4	6	5	1.132

Data2. AccessionNo (IPI)	iPS_CFB50/ HFF	iPS_CFB46/ H9ES	iPS_CFB50/ H9ES	iPS_CFB46/ NTU1ES	iPS_CFB50/ NTU1ES	H9ES/ HFF	NTU1ES/ HFF
IPI00294472	0.957	9999	9999	0.72	0.855	-9999	1.121
IPI00171903	1.659	1.262	1.302	1.318	1.347	0.714	1.14
IPI00015102	1.165	0.847	0.976	0.935	1.074	1.165	1.155
IPI00646625	0.87	0.723	0.935	0.583	0.752	0.907	1.158
IPI00301202	0.756	1.035	0.896	0.755	0.651	0.823	1.162
IPI00008338	0.671	1.386	0.896	0.899	0.581	0.729	1.163
IPI00002070	2.817	9999	9999	2.784	2.394	-9999	1.178
IPI00397526	1.178	1.004	0.745	1.363	1.008	1.NaN	1.179
IPI00793443	1.081	1.757	0.754	2.127	0.91	1.399	1.189
IPI00031030	4.378	9999	9999	2.917	3.668	-9999	1.194
IPI00016608	1.148	1.188	1.074	0.995	0.816	1.054	1.196
IPI00023135	1.859	1.059	1.233	1.333	1.548	1.471	1.202
IPI00006433	2.504	2.745	3.119	1.832	2.076	0.783	1.207
IPI00022018	1.163	1.271	1.167	1.005	0.956	1.015	1.21
IPI00030179	0.944	0.916	0.921	0.779	0.779	0.999	1.211
IPI00028116	0.813	2.167	1.784	0.875	0.584	0.444	1.219
IPI00013271	1.277	0.989	1.416	0.73	1.043	0.88	1.225
IPI00010491	1.072	0.951	0.948	0.878	0.873	1.103	1.23
IPI00177817	1.139	0.975	1.255	0.734	0.906	0.889	1.234
IPI00218343	1.157	0.705	1.08	0.618	0.936	1.044	1.235
IPI00550165	2.07	3.881	4.709	1.733	1.691	0.449	1.238
IPI00554711	1.204	2.724	2.493	1.065	0.972	0.471	1.24
IPI00645667	1.079	1.565	1.987	0.663	0.867	0.463	1.243
IPI00299573	1.125	0.781	1.029	0.696	0.904	1.068	1.247
IPI00414676	1.337	0.704	0.547	1.38	1.083	2.383	1.25
IPI00031397	0.834	0.511	0.655	0.551	0.668	1.305	1.257
IPI00555744	1.393	0.959	1.264	0.787	1.034	1.076	1.258
IPI00100656	1.102	0.914	1.114	0.737	0.892	1.082	1.266
IPI00289819	1.266	0.794	1.016	0.784	0.999	1.216	1.268
IPI00021983	1.168	1.059	1.076	0.926	0.922	1.165	1.271
IPI00456758	1.246	0.9	1.241	0.718	0.987	1.059	1.281
IPI00022744	1.7	3.154	2.802	1.647	1.449	0.798	1.282
IPI00020599	1.143	0.295	0.31	3.809	0.902	3.146	1.285
IPI00470528	1.731	0.826	0.722	1.319	1.341	1.981	1.292
IPI00465361	1.542	0.966	1.348	0.874	1.212	1.142	1.297

Table S1 Identified proteins in the human membrane proteome of hiPSCs, hESCs and somatic foreskin fibroblast HFF.
Profiles of identified proteins in MS Experiment 2

Data2. AccessionNo (IPI)	Gene Symbol	Description	Score	Mass	# of identified (spectrum)	# of samples identified	Total peptides	# of peptides used	iPS_CFB46/ HFF
IPI00182469	CTNND1	CTNND1 Isoform 1AB of Catenin delta-1	1361	107283	40	4	7	4	1.12
IPI00026466	NIPBL	NIPBL Isoform 2 of Nipped-B-like protein	41	304154	1	1	1	1	0.947
IPI00018855	ATP6V0C	ATP6V0C V-type proton ATPase 16 kDa p	64	15725	2	1	2	1	0.615
IPI00007061	GOLT1B	GOLT1B Vesicle transport protein GOT1B	780	15415	12	4	1	1	0.805
IPI00749429	TTYH3	TTYH3 Isoform 1 of Protein tweety homolo	587	57508	13	4	2	1	1.508
IPI00010697	ITGA6	ITGA6 Isoform Alpha-6X1X2B of Integrin a	638	126553	24	3	7	7	1.257
IPI00296259	TMED4	TMED4 Isoform 1 of Transmembrane emp	190	25926	21	4	2	2	1.133
IPI00009225	STX8	STX8 Syntaxin-8	327	26890	5	2	1	1	1.552
IPI00172656	FAF2	FAF2 FAS-associated factor 2	808	52591	22	4	5	5	1.441
IPI00641829	BAT1	BAT1 Isoform 2 of Spliceosome RNA helic	394	50647	11	3	2	2	1.577
IPI00017895	GPD2	GPD2 Isoform 1 of Glycerol-3-phosphate c	637	80802	14	3	3	3	1.173
IPI00027078	CPD	CPD Carboxypeptidase D	418	152835	14	3	5	5	0.9
IPI00028055	TMED10	TMED10 Transmembrane emp24 domain-	3381	24960	90	4	9	5	1.028
IPI00027626	CCT6A	CCT6A T-complex protein 1 subunit zeta	398	57988	6	4	1	1	2.692
IPI00006211	VAPB	VAPB Isoform 1 of Vesicle-associated mer	387	27211	15	4	3	3	0.99
IPI00021304	KRT2	KRT2 Keratin, type II cytoskeletal 2 epider	4356	65825	96	4	17	15	0.825
IPI00023542	TMED9	TMED9 Transmembrane emp24 domain-c	1277	27260	48	4	5	4	1.141
IPI00329331	UGP2	UGP2 Isoform 1 of UTP--glucose-1-phosp	79	56905	2	1	1	1	1.501
IPI00023030	SLC38A1	SLC38A1 Sodium-coupled neutral amino a	99	54012	3	1	1	1	1.004
IPI00395998	RPL32;SNOR	RPL32;SNORA7A 60S ribosomal protein L	49	15850	1	1	1	1	1.12
IPI00941747	CANX	CANX Calnexin	7629	67526	177	4	22	14	1.162
IPI00401819	RPS26	RPS26 Similar to 40S ribosomal protein S2	198	12977	12	4	2	2	-9999
IPI00165651	ERGIC2	ERGIC2 Cd002 protein	48	43467	1	1	1	1	1.08
IPI00011654	TUBB	TUBB Tubulin beta chain	10541	49639	279	4	26	15	0.766
IPI00029741	ITGB5	ITGB5 Integrin beta-5	333	87996	8	3	3	3	3.294
IPI00412107	ANKRD26P1	ANKRD26P1 Ankyrin repeat domain-conta	0	102367	3	2	1	1	0.728
IPI00010740	SFPQ	SFPQ Isoform Long of Splicing factor, prol	284	76102	6	3	2	2	1.741
IPI00472810	UBE3C	UBE3C Isoform 2 of Ubiquitin-protein ligas	49	74748	3	2	1	1	1.599
IPI00009865	KRT10	KRT10 Keratin, type I cytoskeletal 10	9733	58818	198	4	31	22	0.895
IPI00024975	KIF15	KIF15 Isoform 1 of Kinesin-like protein KIF	37	160061	1	1	1	1	0.815
IPI00003431	MAPK6	MAPK6 Mitogen-activated protein kinase 6	39	82629	4	2	1	1	1.406
IPI00026202	RPL18A	RPL18A 60S ribosomal protein L18a	65	20749	2	1	1	1	1.129
IPI00031691	RPL9	RPL9 60S ribosomal protein L9	600	21850	16	4	3	3	1.472
IPI00022462	TFRC	TFRC Transferrin receptor protein 1	3122	84818	110	4	17	16	1.003
IPI00002372	ABCD3	ABCD3 Isoform 1 of ATP-binding cassette	184	75428	9	4	2	2	1.381

Data2. AccessionNo (IPI)	iPS_CFB50/ HFF	iPS_CFB46/ H9ES	iPS_CFB50/ H9ES	iPS_CFB46/ NTU1ES	iPS_CFB50/ NTU1ES	H9ES/ HFF	NTU1ES/ HFF
IPI00182469	0.997	0.882	0.789	0.879	0.783	1.221	1.298
IPI00026466	1.376	1.02	1.496	0.724	1.059	0.898	1.301
IPI00018855	1.07	0.483	0.562	0.486	0.83	0.759	1.312
IPI00007061	1.253	1.225	1.923	0.61	0.956	0.636	1.313
IPI00749429	1.784	1.408	1.67	1.121	1.325	0.996	1.317
IPI00010697	0.927	1.213	1.069	0.746	0.778	0.847	1.32
IPI00296259	0.942	1.049	0.879	0.854	0.726	1.045	1.321
IPI00009225	-9999	0.733	-9999	1.166	-9999	2.047	1.325
IPI00172656	1.618	1.451	1.682	1.024	1.263	0.941	1.329
IPI00641829	1.048	0.73	0.491	1.147	0.769	2.044	1.333
IPI00017895	0.716	0.87	0.548	0.837	0.519	1.311	1.334
IPI00027078	1.119	0.986	1.213	0.674	0.813	0.9	1.342
IPI00028055	1.171	1.123	1.291	0.77	0.87	0.901	1.342
IPI00027626	3.938	5.901	8.704	1.996	2.935	0.441	1.343
IPI00006211	1.23	1.247	1.16	0.764	0.895	1.218	1.354
IPI00021304	0.642	0.644	0.47	0.63	0.527	1.232	1.354
IPI00023542	1.267	1.013	1.107	0.832	0.931	1.087	1.367
IPI00329331	1.304	0.642	0.562	1.086	0.948	2.264	1.377
IPI00023030	0.996	0.691	0.691	0.724	0.721	1.406	1.382
IPI00395998	1.494	0.861	1.159	0.799	1.073	1.258	1.394
IPI00941747	1.59	0.996	1.353	0.824	1.085	1.122	1.399
IPI00401819	1.981	-9999	1.501	-9999	1.416	1.287	1.4
IPI00165651	1.069	0.848	0.847	0.765	0.761	1.231	1.405
IPI00011654	1.354	0.689	1.211	0.537	0.948	1.077	1.408
IPI00029741	1.733	2.611	2.256	2.284	1.222	0.666	1.419
IPI00412107	0.513	0.634	0.45	0.511	0.362	1.111	1.419
IPI00010740	1.587	0.503	0.432	1.291	1.112	3.24	1.424
IPI00472810	1.93	1.398	1.701	1.111	1.348	1.107	1.434
IPI00009865	0.696	0.7	0.56	0.63	0.5	1.257	1.436
IPI00024975	-9999	0.675	-9999	0.562	-9999	1.168	1.445
IPI00003431	1.362	1.053	1.028	0.968	0.943	1.293	1.446
IPI00026202	1.151	0.79	0.812	0.777	0.797	1.383	1.446
IPI00031691	1.393	0.845	0.823	0.995	0.964	1.673	1.446
IPI00022462	0.971	0.749	0.724	0.692	0.672	1.338	1.453
IPI00002372	1.917	1.435	1.909	0.934	1.297	0.826	1.459

Table S1 Identified proteins in the human membrane proteome of hiPSCs, hESCs and somatic foreskin fibroblast HFF.
Profiles of identified proteins in MS Experiment 2

Data2. AccessionNo (IPI)	Gene Symbol	Description	Score	Mass	# of identified (spectrum)	# of samples identified	Total peptides	# of peptides used	iPS_CFB46/ HFF
IPI00220739	PGRMC1	PGRMC1 Membrane-associated progeste	2361	21658	40	4	8	7	0.903
IPI00003985	BCS1L	BCS1L Mitochondrial chaperone BCS1	297	47504	8	4	1	1	1.532
IPI00337494	SLC25A24	SLC25A24 Isoform 1 of Calcium-binding m	382	53320	11	4	3	3	3.326
IPI00025346	PEX14	PEX14 Isoform 1 of Peroxisomal membrar	157	41212	5	3	2	1	3.783
IPI00065486	ABCB6	ABCB6 Isoform 2 of ATP-binding cassette	107	88543	2	1	1	1	1.363
IPI00465113	EXD2	EXD2 cDNA FLJ58573, highly similar to E:	109	70308	4	3	1	1	-9999
IPI00743576	ATP6V0A1	ATP6V0A1 Isoform 2 of V-type proton ATF	190	95694	6	2	3	3	1.176
IPI00014236	SLC39A14	SLC39A14 Isoform 1 of Zinc transporter ZI	641	54161	14	4	2	2	1.232
IPI00006957	DHRS7	DHRS7 Isoform 1 of Dehydrogenase/redu	67	38274	2	1	2	2	0.771
IPI00304612	RPL13A	RPL13A 60S ribosomal protein L13a	342	23562	11	4	1	1	1.088
IPI00021267	EPHA2	EPHA2 Ephrin type-A receptor 2	390	108197	13	1	5	4	1.254
IPI00215719	RPL18	RPL18 60S ribosomal protein L18	1169	21621	27	4	4	4	1.466
IPI00020436	RAB11B	RAB11B Ras-related protein Rab-11B	1196	24473	40	4	7	7	1.63
IPI00025049	M6PR	M6PR Cation-dependent mannose-6-phos	312	30973	5	2	1	1	1.737
IPI00152240	TMEM167A	TMEM167A Transmembrane protein 167A	241	8054	10	4	2	2	1.711
IPI00784154	HSPD1	HSPD1 60 kDa heat shock protein, mitoch	3635	61016	82	4	22	16	5.654
IPI00395894	SYS1	SYS1 Protein SYS1 homolog	54	17603	1	1	1	1	1.18
IPI00031522	HADHA	HADHA Trifunctional enzyme subunit alph	100	82947	2	1	2	2	1.309
IPI00016339	RAB5C	RAB5C Ras-related protein Rab-5C	1051	23468	32	4	6	5	0.422
IPI00006482	ATP1A1	ATP1A1 Isoform Long of Sodium/potassiu	9279	112824	218	4	30	24	1.41
IPI00220578	GNAI3	GNAI3 Guanine nucleotide-binding protein	1591	40506	22	4	3	3	1.155
IPI00642244	KIAA0090	KIAA0090 Isoform 4 of Uncharacterized pr	2131	109352	51	4	10	10	1.943
IPI00019385	SSR4	SSR4 Translocon-associated protein subu	1892	20200	51	4	7	4	1.578
IPI00221091	RPS15A	RPS15A 40S ribosomal protein S15a	613	14830	27	4	4	3	1.12
IPI00333619	ALDH3A2	ALDH3A2 Isoform 1 of Fatty aldehyde deh	1903	54813	67	3	9	8	1.347
IPI00107357	CLPTM1	CLPTM1 Isoform 2 of Cleft lip and palate t	81	77909	2	1	1	1	2.592
IPI00009407	DAD1	DAD1 Dolichyl-diphosphooligosaccharide--	658	12489	22	4	2	2	1.325
IPI00024551	C15orf24	C15orf24 UPF0480 protein C15orf24	40	26454	1	1	1	1	1.959
IPI00184311	ENPP1	ENPP1 Ectonucleotide pyrophosphatase/p	108	104857	2	1	1	1	1.982
IPI00146447	VMA21	VMA21 Vacuolar ATPase assembly integr	102	11347	4	2	1	1	1.297
IPI00179964	PTBP1	PTBP1 Isoform 1 of Polypyrimidine tract-bi	422	57186	10	3	3	3	2.257
IPI00001159	GCN1L1	GCN1L1 Translational activator GCN1	79	292558	3	1	2	2	1.658
IPI00000115	CNIH4	CNIH4 Isoform 1 of Protein cornichon hor	227	16082	8	2	2	1	0.857
IPI00306748	ABCB7	ABCB7 Isoform 1 of ATP-binding cassette	104	82589	3	1	1	1	1.498
IPI00452747	LOC653566	LOC653566 Similar to Signal peptidase co	432	24960	16	4	4	4	1.435

Data2. AccessionNo (IPI)	iPS_CFB50/ HFF	iPS_CFB46/ H9ES	iPS_CFB50/ H9ES	iPS_CFB46/ NTU1ES	iPS_CFB50/ NTU1ES	H9ES/ HFF	NTU1ES/ HFF
IPI00220739	1.179	0.94	1.238	0.649	0.809	0.934	1.461
IPI00003985	0.759	2.855	1.426	1.04	0.518	0.519	1.466
IPI00337494	0.828	1.126	0.762	0.714	0.491	0.889	1.478
IPI00025346	2.454	9999	9999	3.072	1.655	-9999	1.48
IPI00065486	1.406	1.169	1.215	0.912	0.945	1.128	1.489
IPI00465113	1.171	-9999	0.858	-9999	0.785	1.331	1.494
IPI00743576	1.335	1.19	1.351	0.78	0.882	0.956	1.517
IPI00014236	1.268	0.785	0.813	0.803	0.829	1.525	1.527
IPI00006957	0.725	0.565	0.536	0.502	0.475	1.319	1.528
IPI00304612	1.499	0.847	1.177	0.703	0.973	1.243	1.542
IPI00021267	0.952	0.869	2.627	0.748	0.714	2.769	1.545
IPI00215719	1.267	0.733	0.649	1.256	1.053	1.908	1.56
IPI00020436	1.8	1.135	1.266	1.01	1.183	1.364	1.565
IPI00025049	1.211	1.448	1.018	1.088	0.763	1.161	1.59
IPI00152240	2.206	1.969	2.559	1.069	1.385	0.841	1.594
IPI00784154	1.912	0.484	0.205	2.527	1.077	12.386	1.6
IPI00395894	1.232	0.719	0.756	0.726	0.762	1.589	1.618
IPI00031522	2.077	0.448	0.716	0.675	1.501	2.781	1.621
IPI00016339	0.762	0.513	0.734	0.399	0.611	0.989	1.623
IPI00006482	1.411	1.076	1.067	0.774	0.767	1.144	1.628
IPI00220578	1.254	0.898	0.988	0.697	0.77	1.239	1.629
IPI00642244	1.748	0.829	0.691	1.207	1.016	2.222	1.635
IPI00019385	1.875	1.131	1.378	0.957	1.145	1.322	1.643
IPI00221091	1.237	1.009	1.086	0.777	0.909	1.179	1.68
IPI00333619	1.918	1.361	1.776	0.837	1.08	0.923	1.703
IPI00107357	1.566	9999	9999	1.51	0.917	-9999	1.709
IPI00009407	1.631	0.938	1.165	0.766	0.948	1.366	1.722
IPI00024551	2.742	1.009	1.424	1.123	1.58	1.878	1.737
IPI00184311	2.335	2.267	2.692	1.127	1.335	0.846	1.751
IPI00146447	1.983	1.099	1.694	0.735	1.13	1.141	1.757
IPI00179964	1.599	1.32	0.877	1.198	0.889	1.858	1.758
IPI00001159	2.233	1.NaN	2.094	0.962	1.306	1.079	1.771
IPI00000115	1.339	0.604	0.946	0.464	0.724	2.196	1.774
IPI00306748	2.399	1.188	1.919	0.84	1.352	1.22	1.776
IPI00452747	1.217	0.562	0.477	0.809	0.807	1.736	1.779

Table S1 Identified proteins in the human membrane proteome of hiPSCs, hESCs and somatic foreskin fibroblast HFF.
Profiles of identified proteins in MS Experiment 2

Data2. AccessionNo (IPI)	Gene Symbol	Description	Score	Mass	# of identified (spectrum)	# of samples identified	Total peptides	# of peptides used	iPS_CFB46/ HFF
IPI00294159	SLC25A1	SLC25A1 Tricarboxylate transport protein,	1920	33991	43	4	6	5	1.358
IPI00029046	MLEC	MLEC Malectin	878	32214	25	4	3	3	1.251
IPI00465308	PIGS	PIGS Isoform 1 of GPI transamidase comp	1032	61617	22	4	4	3	1.73
IPI00003802	MAN2A1	MAN2A1 Alpha-mannosidase 2	1748	131057	43	4	8	8	1.099
IPI00303954	CYB5B	CYB5B cytochrome b5 outer mitochondria	879	16684	21	4	4	4	1.378
IPI00395887	TMX1	TMX1 Thioredoxin-related transmembrane	933	31771	25	4	4	4	1.167
IPI00339384	RDH11	RDH11 Isoform 1 of Retinol dehydrogenas	791	35363	21	4	4	4	1.841
IPI00471978	C2CD2	C2CD2 C2 domain-containing protein 2	99	75486	3	1	1	1	4.316
IPI00024266	MGST3	MGST3 Microsomal glutathione S-transfer	883	16506	17	4	2	2	1.835
IPI00000877	HYOU1	HYOU1 Hypoxia up-regulated protein 1	357	111266	11	2	6	6	2.83
IPI00006072	SEC61G	SEC61G Protein transport protein Sec61 s	153	7736	10	4	1	1	1.496
IPI00008167	ATP1B3	ATP1B3 Sodium/potassium-transporting A	1915	31492	46	4	5	4	1.626
IPI00301021	SSR1	SSR1 Isoform 1 of Translocon-associated	881	32215	26	4	2	2	2.195
IPI00007309	LOC1002879	LOC100287932;TIMM23 Mitochondrial im	250	21929	8	3	3	3	1.38
IPI00010746	PTDSS1	PTDSS1 Phosphatidylserine synthase 1	350	55491	12	4	2	2	1.585
IPI00013623	SLC27A3	SLC27A3 Isoform 1 of Long-chain fatty aci	357	87269	8	3	1	1	1.81
IPI00216049	HNRNPK	HNRNPK Isoform 1 of Heterogeneous nuc	441	50944	12	3	2	2	2.749
IPI00215637	DDX3X	DDX3X ATP-dependent RNA helicase DD	234	73198	3	3	2	2	2.197
IPI00300299	SPCS3	SPCS3 Signal peptidase complex subunit	635	20301	22	3	3	3	1.418
IPI00027180	ZMPSTE24	ZMPSTE24 CAAX prenyl protease 1 homo	1235	54778	20	4	5	4	1.946
IPI00003918	RPL4	RPL4 60S ribosomal protein L4	912	47667	34	3	6	6	2.574
IPI00025874	RPN1	RPN1 Dolichyl-diphosphooligosaccharide-	6264	72733	167	4	23	18	1.631
IPI00002520	SHMT2	SHMT2 Serine hydroxymethyltransferase,	163	55958	5	2	2	2	2.987
IPI00297492	STT3A	STT3A Dolichyl-diphosphooligosaccharide	1164	80477	46	4	8	8	1.511
IPI00019472	SLC1A5	SLC1A5 Neutral amino acid transporter B(	1909	56562	60	4	7	6	1.33
IPI00005745	SPTLC1	SPTLC1 Serine palmitoyltransferase 1	920	52710	20	4	4	3	0.809
IPI00019407	NSDHL	NSDHL Sterol-4-alpha-carboxylate 3-dehy	1171	41874	32	4	8	5	1.982
IPI00027232	IGF1R	IGF1R Insulin-like growth factor 1 receptor	175	154693	3	2	1	1	1.859
IPI00024642	CCDC47	CCDC47 Isoform 1 of Coiled-coil domain-c	1132	55838	39	3	8	8	1.875
IPI00009235	SSR3	SSR3 cDNA FLJ52061, highly similar to Tr	920	22596	15	4	1	1	2.183
IPI00018415	TM9SF2	TM9SF2 Transmembrane 9 superfamily m	520	75725	16	4	3	2	1.35
IPI00024757	UQCR	UQCR Cytochrome b-c1 complex subunit	37	6565	1	1	1	1	2.294
IPI00021147	DEGS1	DEGS1 Sphingolipid delta(4)-desaturase D	41	37841	1	1	1	1	1.327
IPI00297084	DDOST	DDOST Dolichyl-diphosphooligosaccharid	3807	50769	87	4	12	11	1.773
IPI00470467	POR	POR NADPH--cytochrome P450 reductase	3344	76641	69	4	12	10	1.635

Data2. AccessionNo (IPI)	iPS_CFB50/ HFF	iPS_CFB46/ H9ES	iPS_CFB50/ H9ES	iPS_CFB46/ NTU1ES	iPS_CFB50/ NTU1ES	H9ES/ HFF	NTU1ES/ HFF
IPI00294159	1.417	0.972	1.032	0.755	0.796	1.325	1.789
IPI00029046	1.859	0.989	1.18	0.698	1.014	1.224	1.79
IPI00465308	2.106	1.006	1.257	0.96	1.186	1.682	1.796
IPI00003802	1.447	1.254	1.638	0.633	0.818	0.709	1.797
IPI00303954	1.434	1.472	1.82	0.713	0.865	0.926	1.808
IPI00395887	1.518	0.947	1.291	0.667	0.885	1.121	1.831
IPI00339384	2.108	0.883	1	0.97	1.097	1.993	1.866
IPI00471978	4.038	3.743	3.53	2.256	2.122	1.116	1.905
IPI00024266	2.192	0.995	1.196	0.952	1.141	1.786	1.919
IPI00000877	1.983	0.459	0.371	1.271	0.841	5.988	1.924
IPI00006072	2.087	0.989	1.391	0.768	1.077	1.464	1.939
IPI00008167	1.495	1.321	1.2	0.827	0.748	1.2	1.943
IPI00301021	2.202	1.663	1.704	1.12	1.134	1.274	1.943
IPI00007309	1.496	1.03	0.772	1.785	1.367	1.598	1.958
IPI00010746	2.007	0.996	1.587	0.802	1.012	1.695	1.967
IPI00013623	5.404	2.597	7.816	0.916	2.75	0.674	1.967
IPI00216049	2.432	0.666	0.59	1.401	1.244	4.138	1.967
IPI00215637	1.996	0.79	0.721	1.096	1.001	2.675	1.973
IPI00300299	1.865	0.975	1.212	0.867	0.947	1.499	1.974
IPI00027180	1.28	1.545	1.064	0.966	1.195	1.162	1.994
IPI00003918	2.267	0.803	0.704	1.293	1.132	3.176	2.003
IPI00025874	2.072	1.045	1.352	0.805	1.022	1.442	2.007
IPI00002520	1.674	0.503	0.273	1.474	0.831	5.982	2.017
IPI00297492	2.075	1.048	1.425	0.745	1.023	1.408	2.037
IPI00019472	1.266	1.075	1.018	0.693	0.624	1.21	2.059
IPI00005745	1.018	1.115	1.619	0.816	1.01	1.476	2.075
IPI00019407	2.408	1.276	1.579	0.974	1.157	1.501	2.08
IPI00027232	1.62	1.087	0.955	0.887	0.777	1.654	2.086
IPI00024642	2.408	0.551	0.745	0.824	1.103	3.877	2.088
IPI00009235	2.12	1.198	1.172	1.028	1.003	1.764	2.114
IPI00018415	1.761	0.931	1.262	0.607	0.871	1.404	2.114
IPI00024757	2.026	0.873	0.777	1.08	0.958	2.544	2.116
IPI00021147	1.953	9999	9999	0.622	0.92	-9999	2.125
IPI00297084	2.325	1.068	1.38	0.787	1.028	1.507	2.129
IPI00470467	2.376	1.085	1.559	0.735	1.05	1.478	2.148

Table S1 Identified proteins in the human membrane proteome of hiPSCs, hESCs and somatic foreskin fibroblast HFF.
Profiles of identified proteins in MS Experiment 2

Data2. AccessionNo (IPI)	Gene Symbol	Description	Score	Mass	# of identified (spectrum)	# of samples identified	Total peptides	# of peptides used	iPS_CFB46/ HFF
IPI00063130	TMEM205	TMEM205 Transmembrane protein 205	291	21184	10	3	2	2	2.08
IPI00305383	UQCRC2	UQCRC2 Cytochrome b-c1 complex subu	362	48413	11	3	3	3	2.207
IPI00028635	RPN2	RPN2 Dolichyl-diphosphooligosaccharide-	12468	69241	218	4	25	20	1.691
IPI00007676	HSD17B12	HSD17B12 Estradiol 17-beta-dehydrogena	1719	34302	52	4	10	7	1.413
IPI00295772	CYP51A1;LO	CYP51A1;LOC401387 cytochrome P450,	820	57242	30	4	5	5	2.072
IPI00183786	FADS2	FADS2 Isoform 1 of Fatty acid desaturase	1187	52226	37	3	6	4	2.169
IPI00000138	MGAT1	MGAT1 Alpha-1,3-mannosyl-glycoprotein	394	50830	12	4	2	2	1.709
IPI00554541	ILVBL	ILVBL Isoform 1 of Acetolactate synthase-	1954	67825	42	4	9	4	1.62
IPI00021058	SLC4A7	SLC4A7 Solute carrier family 4 sodium bic	963	127278	23	4	4	4	1.18
IPI00003968	NDUFA9	NDUFA9 NADH dehydrogenase [ubiquino	146	42483	5	3	3	3	2.486
IPI00164018	CYP2S1	CYP2S1 Isoform 2 of Cytochrome P450 2	3123	62192	82	3	18	11	1.48
IPI00793874	SFXN3	SFXN3 sideroflexin 3	302	35956	7	1	3	3	1.745
IPI00329352	NOMO3;NOM	NOMO3;NOMO1 Nodal modulator 1	942	134267	25	4	5	5	1.849
IPI00027194	STX18	STX18 Syntaxin-18	104	38650	3	2	2	2	2.739
IPI00104128	SEC11A	SEC11A Signal peptidase complex catalyti	1434	20612	39	4	4	3	1.845
IPI00151358	CLPTM1L	CLPTM1L Isoform 1 of Cleft lip and palate	453	62189	10	4	1	1	1.501
IPI00017510	MT-CO2	MT-CO2 Cytochrome c oxidase subunit 2	1949	25548	49	4	8	4	1.361
IPI00022275	SACM1L	SACM1L Phosphatidylinositide phosphata	1041	66924	28	4	5	5	2.005
IPI00032038	CPT1A	CPT1A Isoform 1 of Carnitine O-palmitoylt	269	88311	8	3	2	1	1.327
IPI00100030	PIGT	PIGT Isoform 1 of GPI transamidase com	386	65658	10	4	1	1	2.326
IPI00005751	SPTLC2	SPTLC2 Serine palmitoyltransferase 2	134	62884	5	4	1	1	1.614
IPI00028946	RTN3	RTN3 Isoform 3 of Reticulon-3	433	25593	17	4	3	2	1.991
IPI00641435	LASS2	LASS2 LAG1 homolog, ceramide synthase	369	15423	12	3	2	2	1.717
IPI00018246	HK1	HK1 Isoform 1 of Hexokinase-1	856	102420	25	4	7	6	0.279
IPI00021805	MGST1	MGST1 Microsomal glutathione S-transfer	1279	17587	30	4	7	3	1.355
IPI00019141	AGPAT1	AGPAT1 1-acyl-sn-glycerol-3-phosphate a	557	31696	11	3	2	1	4.473
IPI00027107	TUFM	TUFM Tu translation elongation factor, mit	743	49843	23	3	5	5	3.966
IPI00013881	HNRNPH1	HNRNPH1 Heterogeneous nuclear ribonu	753	49198	14	3	2	2	2.537
IPI00299084	TMEM33	TMEM33 Transmembrane protein 33	622	27960	18	4	2	2	1.998
IPI00171626	LPCAT1	LPCAT1 Lysophosphatidylcholine acyltran	717	59113	25	3	5	5	1.509
IPI00334282	FAM3C	FAM3C Protein FAM3C	1278	24665	25	4	4	3	1.855
IPI00103940	MFSD10	MFSD10 Major facilitator superfamily dom	158	48308	9	3	1	1	2.103
IPI00152377	STT3B	STT3B Dolichyl-diphosphooligosaccharide	1390	93614	37	4	6	5	1.661
IPI00029264	CYC1	CYC1 Cytochrome c1, heme protein, mito	702	35367	14	4	2	2	1.906
IPI00216308	VDAC1	VDAC1 Voltage-dependent anion-selective	2266	30754	62	4	5	5	1.796

Data2. AccessionNo (IPI)	iPS_CFB50/ HFF	iPS_CFB46/ H9ES	iPS_CFB50/ H9ES	iPS_CFB46/ NTU1ES	iPS_CFB50/ NTU1ES	H9ES/ HFF	NTU1ES/ HFF
IPI00063130	2.203	0.856	0.981	0.779	0.89	2.439	2.149
IPI00305383	2.327	0.512	0.548	1.099	1.174	5.261	2.151
IPI00028635	2.125	1.143	1.448	0.789	0.994	1.421	2.162
IPI00007676	1.63	0.975	1.127	0.65	0.752	1.402	2.167
IPI00295772	2.368	1.18	1.363	0.944	1.093	1.698	2.173
IPI00183786	2.812	1.01	1.344	0.975	1.294	1.892	2.174
IPI00000138	2.348	0.962	1.332	0.775	1.07	1.719	2.189
IPI00554541	2.197	0.969	1.307	0.726	0.998	1.619	2.208
IPI00021058	0.96	0.789	0.647	0.531	0.428	1.445	2.22
IPI00003968	2.105	0.461	0.415	0.917	0.825	4.392	2.224
IPI00164018	2.046	1.147	1.773	0.67	0.966	2.002	2.228
IPI00793874	0.776	0.78	0.38	0.694	0.339	1.967	2.253
IPI00329352	2.815	0.96	1.487	0.799	1.228	1.883	2.266
IPI00027194	2.584	NaN	3.068	1.203	1.376	0.627	2.267
IPI00104128	2.491	1.156	1.582	0.787	1.079	1.513	2.284
IPI00151358	2.52	1.91	3.233	0.653	1.102	0.76	2.288
IPI00017510	1.649	0.569	0.648	0.64	0.749	3.515	2.309
IPI00022275	2.12	1.086	1.158	0.884	0.927	1.784	2.357
IPI00032038	1.539	1.207	1.28	0.633	0.668	0.968	2.358
IPI00100030	3.155	2.612	3.571	0.976	1.33	0.862	2.374
IPI00005751	2.08	0.69	0.897	0.671	0.87	2.262	2.394
IPI00028946	2.989	0.986	1.476	0.822	1.227	1.958	2.41
IPI00641435	2.661	1.104	1.705	0.711	1.094	1.5	2.414
IPI00018246	1.23	0.255	0.316	0.605	0.648	1.483	2.417
IPI00021805	1.819	0.926	1.241	0.553	0.933	1.427	2.436
IPI00019141	4.127	1.458	1.706	1.767	2.069	2.862	2.444
IPI00027107	2.011	0.651	0.316	1.57	0.817	6.377	2.454
IPI00013881	3.029	0.463	0.NaN	1.029	1.233	5.301	2.457
IPI00299084	2.764	0.921	1.284	0.749	1.004	2.099	2.478
IPI00171626	2.257	0.836	1.153	0.622	0.865	4.004	2.484
IPI00334282	1.63	1.407	1.181	0.674	0.654	1.281	2.486
IPI00103940	3.382	1.038	1.683	0.84	1.359	1.96	2.491
IPI00152377	2.511	1.267	1.612	0.81	1.018	1.393	2.517
IPI00029264	2.787	0.546	0.729	0.726	NaN	3.727	2.525
IPI00216308	1.931	0.754	0.823	0.701	0.763	2.288	2.546

Table S1 Identified proteins in the human membrane proteome of hiPSCs, hESCs and somatic foreskin fibroblast HFF.
Profiles of identified proteins in MS Experiment 2

Data2. AccessionNo (IPI)	Gene Symbol	Description	Score	Mass	# of identified (spectrum)	# of samples identified	Total peptides	# of peptides used	iPS_CFB46/ HFF
IPI00000425	ATP6V0A2	ATP6V0A2 V-type proton ATPase 116 kDa	151	98018	3	2	2	2	2.14
IPI00026044	PIGU	PIGU Isoform 1 of Phosphatidylinositol gly	74	50019	1	1	1	1	1.858
IPI00062469	TMEM49	TMEM49 Transmembrane protein 49	122	46208	2	2	1	1	2.672
IPI00022202	SLC25A3	SLC25A3 Isoform A of Phosphate carrier p	2222	40069	56	4	8	5	2.126
IPI00794229	SPCS1	SPCS1 9 kDa protein	38	8565	1	1	1	1	1.294
IPI00006579	COX4I1	COX4I1 Cytochrome c oxidase subunit 4 is	575	19564	19	3	5	3	1.384
IPI00170692	VAPA	VAPA Vesicle-associated membrane prote	346	27875	2	2	2	2	1.219
IPI00334190	STOML2	STOML2 Stomatin-like protein 2	541	38510	13	4	3	3	2.597
IPI00023526	RAB6A	RAB6A Isoform 1 of Ras-related protein Ra	1697	23578	26	4	3	3	1.918
IPI00305698	GGCX	GGCX Vitamin K-dependent gamma-carbo	49	87505	1	1	1	1	1.885
IPI00654820	MT-ATP6	MT-ATP6 ATP synthase subunit a	457	26032	12	4	1	1	1.984
IPI00032903	PTRH2	PTRH2 Peptidyl-tRNA hydrolase 2, mitoch	271	19181	10	4	2	2	0.626
IPI00220556	PBXIP1	PBXIP1 Isoform 3 of Pre-B-cell leukemia tr	196	72876	7	2	3	3	0.783
IPI00291467	SLC25A6	SLC25A6 ADP/ATP translocase 3	2250	32845	101	4	13	11	1.888
IPI00329596	TMX2	TMX2 Putative uncharacterized protein TM	554	42444	14	4	2	2	4.437
IPI00300127	NAT10	NAT10 N-acetyltransferase 10	83	115631	2	1	1	1	5.475
IPI00023334	MRPL4	MRPL4 Isoform 1 of 39S ribosomal protein	44	34897	1	1	1	1	4.111
IPI00296215	EPCAM	EPCAM Epithelial cell adhesion molecule	427	34910	6	2	1	1	1.394
IPI00013847	UQCRC1	UQCRC1 Cytochrome b-c1 complex subun	319	52612	9	3	3	3	3.35
IPI00219729	SLC25A11	SLC25A11 Mitochondrial 2-oxoglutarate/m	1015	34040	25	4	4	4	1.617
IPI00418497	TIMM50	TIMM50 Isoform 2 of Mitochondrial import	89	50433	4	3	1	1	3.666
IPI00217007	KIAA2013	KIAA2013 Isoform 2 of Uncharacterized pr	338	72636	7	3	1	1	2.505
IPI00166079	VKORC1L1	VKORC1L1 Vitamin K epoxide reductase c	359	19823	12	4	2	2	2.045
IPI00395769	ATP5C1	ATP5C1 Isoform Heart of ATP synthase su	636	32860	19	4	2	2	1.217
IPI00025292	MPDU1	MPDU1 Mannose-P-dolichol utilization def	405	26620	11	4	1	1	1.885
IPI00005198	ILF2	ILF2 Interleukin enhancer-binding factor 2	643	43035	25	3	5	5	1.756
IPI00411937	NOP56	NOP56 Nucleolar protein 56	65	66009	1	1	1	1	0.9
IPI00024145	VDAC2	VDAC2 Isoform 2 of Voltage-dependent an	2053	30393	53	4	4	4	2.496
IPI00033217	AASS	AASS Alpha-aminoadipic semialdehyde sy	328	102066	8	2	4	4	5.227
IPI00008998	PTPLAD1	PTPLAD1 Protein tyrosine phosphatase-lik	2398	43132	64	4	8	6	2.576
IPI00020944	FDFT1	FDFT1 Squalene synthetase	4021	48084	84	3	14	9	2.948
IPI00012434	STARD3NL	STARD3NL Isoform 1 of MLN64 N-termina	115	26638	3	1	1	1	-9999
IPI00019906	BSG	BSG Isoform 2 of Basigin	5143	29203	108	4	12	7	2.706
IPI00216592	HNRNPC	HNRNPC Isoform C1 of Heterogeneous nu	1405	32318	29	3	4	3	3.113
IPI00031821	ITM2B	ITM2B Integral membrane protein 2B	78	30318	3	3	2	2	2.037

Data2. AccessionNo (IPI)	iPS_CFB50/ HFF	iPS_CFB46/ H9ES	iPS_CFB50/ H9ES	iPS_CFB46/ NTU1ES	iPS_CFB50/ NTU1ES	H9ES/ HFF	NTU1ES/ HFF
IPI00000425	2.641	1.09	1.356	0.86	0.714	1.9	2.612
IPI00026044	2.432	2.234	2.948	0.7	0.922	0.805	2.641
IPI00062469	3.419	1.025	1.323	1.005	1.293	2.521	2.647
IPI00022202	2.089	0.998	1.004	0.842	0.843	2.393	2.654
IPI00794229	1.807	0.874	1.231	0.482	0.677	1.432	2.672
IPI00006579	1.725	0.665	0.735	0.551	0.685	2.519	2.677
IPI00170692	0.616	1.459	0.638	0.469	0.165	0.803	2.7
IPI00334190	2.093	0.894	0.723	0.96	0.777	2.869	2.708
IPI00023526	2.426	0.839	0.948	0.726	0.904	1.767	2.725
IPI00305698	3.295	1.13	1.991	0.686	1.206	1.614	2.735
IPI00654820	2.315	0.719	0.846	0.719	0.844	2.67	2.747
IPI00032903	3.306	0.103	0.853	0.652	0.714	3.778	2.762
IPI00220556	1.232	0.804	1.155	0.28	0.601	1.043	2.783
IPI00291467	1.99	0.78	0.846	0.672	0.733	2.36	2.786
IPI00329596	6.184	3.111	4.371	1.107	1.511	1.38	2.807
IPI00300127	3.516	2.694	1.744	1.921	1.24	1.966	2.838
IPI00023334	3.114	0.836	0.638	1.361	1.037	4.76	3.007
IPI00296215	1.529	0.639	0.706	0.459	0.506	2.113	3.024
IPI00013847	3.056	0.531	0.488	1.079	0.99	6.108	3.09
IPI00219729	2.474	0.605	1.007	0.487	0.808	2.371	3.094
IPI00418497	2.883	0.473	0.375	1.164	0.92	7.507	3.135
IPI00217007	2.381	2.394	2.294	0.795	0.759	1.013	3.139
IPI00166079	2.054	1.487	1.505	0.685	0.808	1.331	3.161
IPI00395769	2.456	0.395	0.45	0.715	0.773	3.063	3.178
IPI00025292	2.601	1.147	1.595	0.589	0.817	1.591	3.187
IPI00005198	1.691	0.818	0.937	0.568	0.648	2.578	3.238
IPI00411937	2.22	0.144	0.358	0.277	0.686	6.049	3.239
IPI00024145	2.738	0.817	0.897	0.763	0.835	2.959	3.271
IPI00033217	3.858	0.513	0.354	1.591	0.917	13.738	3.298
IPI00008998	3.64	1.151	1.603	0.784	1.087	2.247	3.31
IPI00020944	2.729	1.187	1.375	0.864	1.013	1.689	3.439
IPI00012434	2.923	NaN	9999	-9999	0.847	-9999	3.454
IPI00019906	2.854	0.997	1.071	0.757	0.812	2.6	3.486
IPI00216592	2.859	0.588	0.588	1.059	0.992	5.385	3.495
IPI00031821	2.81	2.35	3.267	0.556	0.805	0.839	3.496

Table S1 Identified proteins in the human membrane proteome of hiPSCs, hESCs and somatic foreskin fibroblast HFF.
Profiles of identified proteins in MS Experiment 2

Data2. AccessionNo (IPI)	Gene Symbol	Description	Score	Mass	# of identified (spectrum)	# of samples identified	Total peptides	# of peptides used	iPS_CFB46/ HFF
IPI00016703	DHCR24	DHCR24 24-dehydrocholesterol reductase	1250	60062	36	4	6	5	4.539
IPI00307572	TMEM165	TMEM165 Transmembrane protein 165	711	34883	16	4	4	4	2.35
IPI00021985	TM9SF4	TM9SF4 Isoform 1 of Transmembrane 9 s	530	74470	18	4	4	3	1.808
IPI00644766	TOR1AIP1	TOR1AIP1 Torsin A interacting protein 1	187	47549	5	3	1	1	3.053
IPI00470924	TMTC3	TMTC3 Isoform 2 of Transmembrane and	200	103814	9	4	1	1	3.248
IPI00099463	SGPL1	SGPL1 Sphingosine-1-phosphate lyase 1	1097	63483	27	3	5	5	2.896
IPI00032150	CDS2	CDS2 Isoform 1 of Phosphatidate cytidylyl	451	51384	6	2	2	2	2.968
IPI00414717	GLG1	GLG1 Isoform 2 of Golgi apparatus protei	3318	137132	72	4	9	6	2.86
IPI00027252	PHB2	PHB2 Prohibitin-2	6186	33276	152	4	15	12	2.805
IPI00009368	SFXN1	SFXN1 Sideroflexin-1	1525	35596	33	4	5	5	3.465
IPI00747849	ATP1B1	ATP1B1 Isoform 1 of Sodium/potassium-tr	148	35039	5	3	1	1	3.605
IPI00026111	TMCO1	TMCO1 Isoform 1 of Transmembrane and	728	21161	17	4	2	1	3.22
IPI00017334	PHB	PHB Prohibitin	4966	29786	120	4	16	12	2.322
IPI00027493	LOC442497;S	LOC442497;SLC3A2 Isoform 2 of 4F2 cell	8334	57909	212	4	24	20	4.49
IPI00910210	ATAD1	ATAD1 cDNA FLJ54240, highly similar to /	260	30615	7	4	1	1	5.396
IPI00030847	TM9SF3	TM9SF3 Transmembrane 9 superfamily m	1274	67843	29	4	5	3	2.754
IPI00171573	CCDC109A	CCDC109A Isoform 1 of Coiled-coil domai	255	39842	6	2	2	2	2.275
IPI00016405	OCIAD1	OCIAD1 Isoform 1 of OCIA domain-contai	196	27609	7	2	3	3	1.603
IPI00007084	SLC25A13	SLC25A13 Calcium-binding mitochondrial	3311	74129	80	4	15	14	4.669
IPI00295992	ATAD3A	ATAD3A Isoform 2 of ATPase family AAA	1171	66177	34	4	5	5	5.901
IPI00386427	SMPDL3B	SMPDL3B Isoform 2 of Acid sphingomyeli	391	41663	12	3	3	3	2.403
IPI00003833	MTCH2	MTCH2 Mitochondrial carrier homolog 2	224	33309	7	4	1	1	7.426
IPI00300384	ERBB2	ERBB2 Receptor tyrosine-protein kinase e	56	137821	1	1	1	1	3.946
IPI00218487	GJA1	GJA1 Gap junction alpha-1 protein	861	42981	25	3	6	4	4.417
IPI00295542	NUCB1	NUCB1 Nucleobindin-1	127	53846	3	1	2	2	5.513
IPI00063903	USMG5	USMG5 Up-regulated during skeletal musc	623	6453	12	4	1	1	4.056
IPI00008708	RSL1D1	RSL1D1 Ribosomal L1 domain-containing	568	54939	10	3	3	3	4.639
IPI00024650	SLC16A1	SLC16A1 Monocarboxylate transporter 1	2574	53923	58	4	10	6	3.903
IPI00027448	ATP5L	ATP5L ATP synthase subunit g, mitochonc	726	11421	21	4	4	3	2.806
IPI00298947	MEST	MEST Isoform 1 of Mesoderm-specific trai	856	38806	19	4	2	2	2.922
IPI00292135	LBR	LBR Lamin-B receptor	615	70658	12	3	2	2	3.146
IPI00017297	MATR3	MATR3 Matrin-3	49	94565	1	1	1	1	8.968
IPI00219291	ATP5J2	ATP5J2 Isoform 2 of ATP synthase subun	1209	11357	37	4	3	2	4.343
IPI00014053	TOMM40	TOMM40 Isoform 1 of Mitochondrial impor	1251	37869	36	4	7	6	6.571
IPI00844578	DHX9	DHX9 ATP-dependent RNA helicase A	832	140869	31	3	6	5	7.603

Data2. AccessionNo (IPI)	iPS_CFB50/ HFF	iPS_CFB46/ H9ES	iPS_CFB50/ H9ES	iPS_CFB46/ NTU1ES	iPS_CFB50/ NTU1ES	H9ES/ HFF	NTU1ES/ HFF
IPI00016703	5.145	1.363	1.514	1.342	1.511	3.062	3.507
IPI00307572	3.044	0.98	1.385	0.61	0.859	2.194	3.566
IPI00021985	2.946	1.061	1.407	0.62	0.827	2.166	3.57
IPI00644766	3.334	1.289	1.419	0.826	0.907	2.292	3.68
IPI00470924	4.459	1.29	1.786	0.874	1.206	2.436	3.7
IPI00099463	3.545	1.678	2.042	0.848	1.279	1.952	3.714
IPI00032150	4.575	1.043	1.621	0.787	1.139	2.753	3.833
IPI00414717	3.569	0.872	1.222	0.694	0.966	2.94	3.876
IPI00027252	2.539	0.752	0.758	0.748	0.727	3.441	3.919
IPI00009368	4.058	0.925	1.047	0.895	1.029	3.505	4.051
IPI00747849	3.779	1.08	1.141	0.878	0.925	3.231	4.088
IPI00026111	4.218	1.445	1.913	0.778	1.025	2.163	4.128
IPI00017334	2.993	0.82	0.899	0.76	0.823	3.715	4.258
IPI00027493	4.337	1.511	1.444	1.017	0.952	2.898	4.426
IPI00910210	5.783	3.349	3.618	1.179	1.27	1.559	4.557
IPI00030847	3.815	1.088	1.506	0.586	0.814	2.074	4.582
IPI00171573	2.569	0.46	0.523	0.494	0.56	4.789	4.589
IPI00016405	1.268	1.262	0.938	1.156	0.796	1.413	4.668
IPI00007084	3.731	0.979	0.961	0.864	0.814	4.662	4.811
IPI00295992	4.824	0.793	0.698	0.665	0.59	1.144	4.825
IPI00386427	2.456	0.973	1.009	0.571	0.395	2.482	4.998
IPI00003833	4.531	3.128	1.924	1.479	0.907	2.297	5.001
IPI00300384	4.701	0.815	0.979	0.78	0.935	4.682	5.035
IPI00218487	5.201	1.799	2.074	0.949	1.057	2.412	5.043
IPI00295542	3.373	0.597	0.29	1.231	0.656	3.301	5.102
IPI00063903	4.078	0.739	0.749	0.764	0.772	5.314	5.29
IPI00008708	5.199	0.634	0.591	0.829	0.762	9.177	5.299
IPI00024650	3.957	0.992	0.94	0.693	0.672	3.786	5.354
IPI00027448	4.415	0.887	0.816	0.558	0.79	3.383	5.363
IPI00298947	5.82	1.506	3.024	0.5	1.315	1.877	5.424
IPI00292135	3.116	1.167	1.165	0.562	0.559	2.608	5.576
IPI00017297	6.316	1.942	1.379	1.601	1.133	4.468	5.577
IPI00219291	4.136	0.802	0.793	0.729	0.719	5.597	5.646
IPI00014053	5.442	1.295	1.18	0.969	0.927	2.357	5.82
IPI00844578	7.429	1.213	1.274	1.239	1.27	3.688	6.013

Table S1 Identified proteins in the human membrane proteome of hiPSCs, hESCs and somatic foreskin fibroblast HFF.
Profiles of identified proteins in MS Experiment 2

Data2. AccessionNo (IPI)	Gene Symbol	Description	Score	Mass	# of identified (spectrum)	# of samples identified	Total peptides	# of peptides used	iPS_CFB46/ HFF
IPI00220194	SLC2A1	SLC2A1 Solute carrier family 2, facilitated	1901	54049	56	4	8	7	4.038
IPI00296190	C10orf58	C10orf58 Uncharacterized protein C10orf5	698	25747	23	3	4	4	8.587
IPI00001754	F11R	F11R Junctional adhesion molecule A	271	32562	9	3	2	2	4.784
IPI00295698	SLC7A3	SLC7A3 Cationic amino acid transporter 3	447	67125	13	3	3	3	4.432
IPI00329410	DOLPP1	DOLPP1 Dolichyldiphosphatase 1	181	27013	6	3	1	1	7.372
IPI00220487	ATP5H	ATP5H Isoform 1 of ATP synthase subunit	737	18480	25	4	4	4	7.206
IPI00063784	VTI1B	VTI1B Isoform Long of Vesicle transport th	189	26672	3	1	2	1	-9999
IPI00166865	CISD2	CISD2 CDGSH iron sulfur domain-containi	211	15268	9	3	1	1	4.925
IPI00219685	NDUFA13;YJI	NDUFA13;YJEFN3 NADH dehydrogenase	365	25804	18	4	3	3	6.215
IPI00016014	ITM2C	ITM2C Isoform 1 of Integral membrane pro	178	30204	5	3	1	1	10.378
IPI00026942	ERLIN2	ERLIN2 Isoform 1 of Erlin-2	2215	37815	50	4	7	6	1.042
IPI00554701	UCRC	UCRC Cytochrome b-c1 complex subunit !	348	7304	7	3	2	1	4.48
IPI00298237	TPP1	TPP1 cDNA FLJ56402, highly similar to Tr	131	62173	2	1	1	1	5.829
IPI00293946	UBXN4	UBXN4 UBX domain-containing protein 4	85	56743	1	1	1	1	9.359
IPI00302850	SNRPD1	SNRPD1 Small nuclear ribonucleoprotein :	299	13273	6	3	1	1	10.909
IPI00479217	HNRNPU	HNRNPU Isoform Short of Heterogeneous	1110	88924	28	3	6	6	9.75
IPI00294501	DHCR7	DHCR7 7-dehydrocholesterol reductase	931	54454	20	3	3	3	11.76
IPI00299116	PODXL	PODXL Podocalyxin-like protein 1 precurs	2392	NaN36	46	3	9	7	3.993
IPI00003909	SLC2A3	SLC2A3 Solute carrier family 2, facilitated	2630	53889	66	3	11	6	22.664
IPI00060523	TLCD1	TLCD1 TLC domain-containing protein 1	138	28529	3	2	1	1	13.708
IPI00011084	CLDN6	CLDN6 Claudin-6	955	23276	23	3	3	3	22.114
IPI00000024	PCDH1	PCDH1 Isoform 1 of Protocadherin-1	42	114673	1	1	1	1	9999
IPI00001587	KIR2DS5	KIR2DS5 Killer cell immunoglobulin-like re	43	33623	1	1	1	1	9999
IPI00002225	LASS4	LASS4 LAG1 longevity assurance homolo(	169	46369	5	3	3	1	9999
IPI00003926	CLN8	CLN8 Protein CLN8	40	32766	1	1	1	1	9999
IPI00004450	RBMXL2	RBMXL2 RNA-binding motif protein, X-link	248	42788	9	3	1	1	NaN
IPI00004454	DPM3	DPM3 Isoform 1 of Dolichol-phosphate ma	119	10087	6	3	1	1	9999
IPI00004669	GALNT2	GALNT2 Polypeptide N-acetylgalactosami	78	64691	2	1	2	2	NaN
IPI00005024	MYBBP1A	MYBBP1A Isoform 1 of Myb-binding protei	246	148762	7	3	3	3	9999
IPI00006702	PELP1	PELP1 cDNA FLJ56414, highly similar to I	118	124789	3	2	1	1	9999
IPI00007144	RPL26L1	RPL26L1 60S ribosomal protein L26-like 1	124	17246	7	3	3	3	9999
IPI00007188	SLC25A5	SLC25A5 ADP/ATP translocase 2	2070	32874	18	4	3	3	9999
IPI00008403	CHST7	CHST7 Carbohydrate sulfotransferase 7	70	54232	1	1	1	1	9999
IPI00008433	RPS5	RPS5 40S ribosomal protein S5	101	22862	5	3	1	1	NaN
IPI00008986	SLC7A5	SLC7A5 Large neutral amino acids transp(	1200	54974	18	4	2	2	9999

Data2. AccessionNo (IPI)	iPS_CFB50/ HFF	iPS_CFB46/ H9ES	iPS_CFB50/ H9ES	iPS_CFB46/ NTU1ES	iPS_CFB50/ NTU1ES	H9ES/ HFF	NTU1ES/ HFF
IPI00220194	4.186	0.748	0.786	0.662	0.707	5.268	6.116
IPI00296190	9.521	1.854	2.333	1.098	1.427	4.217	6.519
IPI00001754	4.232	1.478	1.407	0.657	0.621	4.25	6.728
IPI00295698	5.649	1.772	1.656	0.808	0.684	0.775	6.909
IPI00329410	10.505	1.603	2.303	1.036	1.484	4.45	7.086
IPI00220487	4.714	0.669	0.536	0.821	0.659	12.804	7.275
IPI00063784	-9999	NaN	NaN	-9999	-9999	-9999	7.436
IPI00166865	7.285	0.955	1.424	0.626	0.93	4.99	7.84
IPI00219685	5.763	0.73	0.742	0.705	0.715	7.247	8.801
IPI00016014	8.214	0.95	0.758	1.14	0.907	10.57	9.067
IPI00026942	1.334	1.128	1.468	0.131	0.185	0.894	9.109
IPI00554701	5.033	0.716	0.811	0.471	0.532	1.881	9.467
IPI00298237	5.565	1.516	1.459	0.539	0.517	3.721	10.765
IPI00293946	11.342	0.82	1.002	0.857	1.044	11.042	10.877
IPI00302850	12.07	0.971	1.083	0.975	1.084	10.87	11.142
IPI00479217	7.816	0.726	0.833	0.814	0.728	6.659	11.659
IPI00294501	14.957	1.51	1.936	0.947	1.173	7.536	12.667
IPI00299116	6.297	0.796	0.841	0.744	0.806	1.977	13.361
IPI00003909	15.451	2.05	1.544	1.438	1.079	9.774	14.383
IPI00060523	19.188	5.599	7.902	0.54	0.759	2.369	25.289
IPI00011084	20.844	0.914	0.94	0.593	0.621	24.822	39.09
IPI00000024	9999	9999	9999	0.891	0.689	NaN	9999
IPI00001587	9999	9999	9999	0.83	0.948	NaN	9999
IPI00002225	9999	9999	9999	0.99	0.913	NaN	9999
IPI00003926	9999	3.902	4.529	1.417	1.64	9999	9999
IPI00004450	9999	-9999	0.343	-9999	1.255	9999	9999
IPI00004454	9999	9999	9999	0.593	0.671	NaN	9999
IPI00004669	9999	NaN	9999	-9999	0.999	NaN	9999
IPI00005024	9999	1.395	1.371	1.256	0.858	9999	9999
IPI00006702	9999	1.296	0.861	0.832	0.551	9999	9999
IPI00007144	9999	1.016	1.092	1.132	1.214	9999	9999
IPI00007188	9999	0.763	0.762	0.782	0.813	9999	9999
IPI00008403	9999	9999	9999	1.082	1.897	NaN	9999
IPI00008433	NaN	-9999	-9999	-9999	-9999	9999	9999
IPI00008986	9999	1.369	1.128	0.952	0.781	9999	9999

Table S1 Identified proteins in the human membrane proteome of hiPSCs, hESCs and somatic foreskin fibroblast HFF.
Profiles of identified proteins in MS Experiment 2

Data2. AccessionNo (IPI)	Gene Symbol	Description	Score	Mass	# of identified (spectrum)	# of samples identified	Total peptides	# of peptides used	iPS_CFB46/ HFF
IPI00009904	PDIA4	PDIA4 Protein disulfide-isomerase A4	41	72887	1	1	1	1	NaN
IPI00009960	IMMT	IMMT Isoform 1 of Mitochondrial inner mei	401	83626	14	4	3	3	9999
IPI00010427	TMEM93	TMEM93 Transmembrane protein 93	67	12010	2	1	1	1	9999
IPI00010951	EPPK1	EPPK1 epiplakin 1	192	NaN279	4	2	1	1	9999
IPI00011913	HNRNPA0	HNRNPA0 Heterogeneous nuclear ribonuc	43	30822	1	1	1	1	9999
IPI00011970	HSD17B7	HSD17B7 Isoform 1 of 3-keto-steroid redu	174	38182	4	2	1	1	9999
IPI00013293	TMEM147	TMEM147 Transmembrane protein 147	39	25244	1	1	1	1	NaN
IPI00015473	SLC1A3	SLC1A3 Excitatory amino acid transporter	202	59534	3	1	1	1	9999
IPI00015602	TOMM70A	TOMM70A Mitochondrial import receptor s	61	67412	1	1	1	1	9999
IPI00015833	CHCHD3	CHCHD3 Coiled-coil-helix-coiled-coil-helix	238	26136	10	4	2	2	9999
IPI00015920	SLC25A10	SLC25A10 Isoform 1 of Mitochondrial dica	94	31262	1	1	1	1	9999
IPI00016373	RAB13	RAB13 Ras-related protein Rab-13	557	22760	1	1	1	1	9999
IPI00016597	CLN6	CLN6 Ceroid-lipofuscinosis neuronal prote	39	35896	1	1	1	1	NaN
IPI00017592	LETM1	LETM1 Isoform 1 of LETM1 and EF-hand	137	83302	5	1	2	2	9999
IPI00018034	SURF1	SURF1 Isoform 1 of Surfeit locus protein 1	51	33310	1	1	1	1	9999
IPI00018140	SYNCRIP	SYNCRIP Isoform 1 of Heterogeneous nuc	136	69560	4	2	2	2	9999
IPI00019004	SEC62	SEC62 Translocation protein SEC62	192	45833	8	3	1	1	NaN
IPI00019018	TM7SF2	TM7SF2 Isoform 1 of Delta(14)-sterol redu	38	46375	1	1	1	1	9999
IPI00019146	CXADR	CXADR Isoform 1 of Coxsackievirus and a	344	40004	9	3	2	2	9999
IPI00019899	SC4MOL	SC4MOL C-4 methylsterol oxidase	329	35193	7	3	1	1	9999
IPI00020004	TMEM97	TMEM97 Transmembrane protein 97	87	20834	4	2	1	1	9999
IPI00020005	ARSE	ARSE cDNA FLJ50239, highly similar to A	321	68113	8	3	1	1	9999
IPI00022543	PIGK	PIGK GPI-anchor transamidase	323	45223	10	4	1	1	9999
IPI00022891	SLC25A4	SLC25A4 ADP/ATP translocase 1	1887	33043	11	4	1	1	9999
IPI00024279	HEATR1	HEATR1 HEAT repeat-containing protein 1	45	242215	1	1	1	1	9999
IPI00024976	TOMM22	TOMM22 Mitochondrial import receptor su	131	15512	4	2	2	2	9999
IPI00025366	CS	CS Citrate synthase, mitochondrial	52	51680	1	1	1	1	9999
IPI00026241	BST2	BST2 Bone marrow stromal antigen 2	207	19756	5	2	1	1	NaN
IPI00026570	COX7A2	COX7A2 Cytochrome c oxidase polypeptic	54	12836	2	1	1	1	NaN
IPI00026964	UQCRFS1	UQCRFS1 Cytochrome b-c1 complex subu	84	29649	2	2	1	1	9999
IPI00027728	SLC7A1	SLC7A1 High affinity cationic amino acid tr	346	67594	11	3	3	3	9999
IPI00028077	PSEN1	PSEN1 Isoform 1 of Presenilin-1	40	52634	1	1	1	1	9999
IPI00028338	SELI	SELI Ethanolaminephosphotransferase 1	85	45152	2	2	1	1	9999
IPI00028931	DSG2	DSG2 Desmoglein-2	107	122218	3	2	1	1	9999
IPI00029731	RPL35A	RPL35A 60S ribosomal protein L35a	51	12530	1	1	1	1	9999

Data2. AccessionNo (IPI)	iPS_CFB50/ HFF	iPS_CFB46/ H9ES	iPS_CFB50/ H9ES	iPS_CFB46/ NTU1ES	iPS_CFB50/ NTU1ES	H9ES/ HFF	NTU1ES/ HFF
IPI00009904	NaN	-9999	-9999	-9999	-9999	9999	9999
IPI00009960	9999	0.559	0.373	1.026	0.577	9999	9999
IPI00010427	9999	9999	9999	0.591	1.129	NaN	9999
IPI00010951	9999	4.004	11.33	3.118	8.797	9999	9999
IPI00011913	9999	0.549	0.84	1.077	1.644	9999	9999
IPI00011970	9999	1.001	1.097	0.891	0.974	9999	9999
IPI00013293	NaN	NaN	NaN	-9999	-9999	NaN	9999
IPI00015473	9999	1.01	1.001	0.673	0.666	9999	9999
IPI00015602	9999	0.731	0.549	0.811	0.608	9999	9999
IPI00015833	9999	0.596	0.506	0.801	0.679	9999	9999
IPI00015920	9999	1.447	1.07	0.68	0.502	9999	9999
IPI00016373	9999	0.806	0.836	1.563	1.617	9999	9999
IPI00016597	9999	NaN	9999	-9999	1.171	NaN	9999
IPI00017592	9999	0.704	0.831	1.28	1.42	9999	9999
IPI00018034	9999	9999	9999	1.008	0.923	NaN	9999
IPI00018140	9999	0.876	0.251	0.91	0.21	9999	9999
IPI00019004	9999	NaN	9999	-9999	0.704	NaN	9999
IPI00019018	9999	9999	9999	6.109	4.657	NaN	9999
IPI00019146	9999	1.339	1.654	0.878	1.075	9999	9999
IPI00019899	9999	1.225	1.765	0.733	1.053	9999	9999
IPI00020004	9999	1.131	1.694	0.707	1.055	9999	9999
IPI00020005	9999	3.294	3.34	1.138	1.15	9999	9999
IPI00022543	9999	1.359	1.542	0.845	0.956	9999	9999
IPI00022891	9999	1.025	1.035	0.901	0.907	9999	9999
IPI00024279	9999	0.61	0.401	0.54	0.354	9999	9999
IPI00024976	9999	0.698	0.598	1.115	0.96	9999	9999
IPI00025366	NaN	1.542	-9999	1.419	-9999	9999	9999
IPI00026241	NaN	NaN	NaN	-9999	-9999	NaN	9999
IPI00026570	9999	NaN	9999	-9999	0.693	NaN	9999
IPI00026964	9999	0.566	0.324	1.115	0.636	9999	9999
IPI00027728	9999	1.432	1.209	1.463	1.23	9999	9999
IPI00028077	9999	9999	9999	1.168	2.163	NaN	9999
IPI00028338	9999	2.135	3.028	1.219	1.724	9999	9999
IPI00028931	9999	0.903	1.005	0.725	0.804	9999	9999
IPI00029731	9999	9999	9999	1.038	1.326	NaN	9999

Table S1 Identified proteins in the human membrane proteome of hiPSCs, hESCs and somatic foreskin fibroblast HFF.
Profiles of identified proteins in MS Experiment 2

Data2. AccessionNo (IPI)	Gene Symbol	Description	Score	Mass	# of identified (spectrum)	# of samples identified	Total peptides	# of peptides used	iPS_CFB46/ HFF
IPI00031458	ICMT	ICMT Protein-S-isoprenylcysteine O-methy	205	31917	4	2	1	1	9999
IPI00034277	ATP13A1	ATP13A1 Isoform A of Probable cation-tra	48	132870	1	1	1	1	NaN
IPI00042580	APOO	APOO Isoform 1 of Apolipoprotein O	44	22271	1	1	1	1	9999
IPI00056414	MAL2	MAL2 Protein MAL2	92	19113	3	1	1	1	9999
IPI00060569	ABHD12	ABHD12 Isoform 2 of Monoacylglycerol lip	227	45529	6	3	1	1	9999
IPI00074330	TMEM48	TMEM48 Isoform 3 of Nucleoporin NDC1	87	63531	2	1	2	1	9999
IPI00106966	TMEM70	TMEM70 Isoform 1 of Transmembrane pro	45	28951	1	1	1	1	9999
IPI00165665	C3orf21	C3orf21 Isoform 2 of Uncharacterized prot	349	22290	7	3	1	1	NaN
IPI00166483	C17orf61	C17orf61 UPF0451 protein C17orf61	38	11734	1	1	1	1	NaN
IPI00167232	CASC4	CASC4 cDNA FLJ40946 fis, clone UTERU	61	15502	1	1	1	1	9999
IPI00168336	LEMD2	LEMD2 LEM domain-containing protein 2	87	56940	3	1	1	1	NaN
IPI00168848	B3GNT7	B3GNT7 UDP-GlcNAc:betaGal beta-1,3-N	159	45958	3	1	1	1	9999
IPI00176824	-	- 10 kDa protein	67	10097	1	1	1	1	9999
IPI00178667	TOP2A	TOP2A 183 kDa protein	43	183009	1	1	1	1	9999
IPI00182313	SEC61A2	SEC61A2 Sec61 alpha form 2 isoform b	1144	49561	3	2	2	2	9999
IPI00217536	RHOT1	RHOT1 Isoform 3 of Mitochondrial Rho GT	106	79496	1	1	1	1	9999
IPI00217975	LMNB1	LMNB1 Lamin-B1	180	66368	4	2	2	2	9999
IPI00218565	GPR143	GPR143 G protein-coupled receptor 143	214	46020	3	1	1	1	9999
IPI00219330	ILF3	ILF3 Isoform 5 of Interleukin enhancer-bin	133	74560	3	3	1	1	9999
IPI00219383	NDUFB3	NDUFB3 NADH dehydrogenase [ubiquinor	38	11395	1	1	1	1	9999
IPI00220834	XRCC5	XRCC5 ATP-dependent DNA helicase 2 su	45	82652	1	1	1	1	9999
IPI00232571	GPC4	GPC4 Glypican-4	267	62372	2	1	1	1	9999
IPI00237446	ATP11C	ATP11C Isoform 2 of Probable phospholip	69	126629	2	1	1	1	NaN
IPI00291755	NUP210	NUP210 Isoform 1 of Nuclear pore membr	131	204983	3	1	2	2	9999
IPI00294455	UGT8	UGT8 2-hydroxyacylsphingosine 1-beta-ga	185	61416	5	2	1	1	9999
IPI00294755	CLDN7	CLDN7 Isoform 1 of Claudin-7	48	22375	1	1	1	1	9999
IPI00299468	SCD	SCD Acyl-CoA desaturase	270	41496	9	3	1	1	9999
IPI00301323	DDX18	DDX18 ATP-dependent RNA helicase DD)	90	75359	3	2	1	1	9999
IPI00301841	TMEM161A	TMEM161A Transmembrane protein 161A	382	53567	10	4	1	1	9999
IPI00304967	CYP26A1	CYP26A1 Cytochrome P450 26A1	165	56162	6	2	2	2	9999
IPI00306382	SCAMP3	SCAMP3 Isoform 1 of Secretory carrier-as	387	38262	7	3	1	1	NaN
IPI00328391	GALNT7	GALNT7 N-acetylgalactosaminyltransferas	206	75341	6	3	3	2	9999
IPI00328840	THOC4	THOC4 THO complex 4	38	27541	1	1	1	1	NaN
IPI00376686	AMFR	AMFR 42 kDa protein	62	42402	1	1	1	1	9999
IPI00377017	PKMYT1	PKMYT1 protein kinase Myt1 isoform 2	55	52113	2	2	1	1	9999

Data2. AccessionNo (IPI)	iPS_CFB50/ HFF	iPS_CFB46/ H9ES	iPS_CFB50/ H9ES	iPS_CFB46/ NTU1ES	iPS_CFB50/ NTU1ES	H9ES/ HFF	NTU1ES/ HFF
IPI00031458	9999	9999	9999	0.763	0.789	NaN	9999
IPI00034277	9999	NaN	9999	-9999	1.199	NaN	9999
IPI00042580	9999	0.532	0.528	0.522	0.516	9999	9999
IPI00056414	9999	0.786	0.645	0.606	0.496	9999	9999
IPI00060569	9999	1.125	1.499	0.813	1.08	9999	9999
IPI00074330	9999	9999	9999	1.537	2.397	NaN	9999
IPI00106966	9999	0.443	0.455	0.45	0.461	9999	9999
IPI00165665	9999	NaN	9999	-9999	1.769	NaN	9999
IPI00166483	9999	-9999	0.603	-9999	0.579	9999	9999
IPI00167232	9999	1.448	0.772	1.403	0.746	9999	9999
IPI00168336	9999	NaN	9999	-9999	0.895	NaN	9999
IPI00168848	9999	1.677	4.856	0.607	1.753	9999	9999
IPI00176824	9999	2.528	1.652	1.361	0.887	9999	9999
IPI00178667	9999	0.663	0.7	1.081	1.139	9999	9999
IPI00182313	9999	3.96	1.423	6.393	2.291	9999	9999
IPI00217536	9999	2.033	2.907	1.402	2	9999	9999
IPI00217975	9999	1.269	1.417	1.622	1.562	9999	9999
IPI00218565	9999	1.406	1.65	0.295	0.345	9999	9999
IPI00219330	9999	0.685	0.602	0.722	0.632	9999	9999
IPI00219383	9999	1.92	2.502	2.397	3.114	9999	9999
IPI00220834	9999	9999	9999	0.753	0.912	NaN	9999
IPI00232571	NaN	0.75	-9999	0.591	-9999	9999	9999
IPI00237446	NaN	NaN	NaN	-9999	-9999	NaN	9999
IPI00291755	9999	0.711	1.018	0.505	0.722	9999	9999
IPI00294455	9999	2.598	5.224	0.462	0.926	9999	9999
IPI00294755	9999	9999	9999	0.468	0.419	NaN	9999
IPI00299468	9999	9999	9999	0.807	1.061	NaN	9999
IPI00301323	9999	3.115	1.74	1.459	0.812	9999	9999
IPI00301841	9999	1.523	2.376	0.628	0.976	9999	9999
IPI00304967	9999	9999	9999	1.963	2.561	NaN	9999
IPI00306382	9999	-9999	1.177	-9999	1.049	9999	9999
IPI00328391	9999	0.999	1.321	1.182	1.558	9999	9999
IPI00328840	NaN	-9999	-9999	-9999	-9999	9999	9999
IPI00376686	9999	9999	9999	2.487	1.994	NaN	9999
IPI00377017	9999	0.906	0.952	0.792	0.83	9999	9999

Table S1 Identified proteins in the human membrane proteome of hiPSCs, hESCs and somatic foreskin fibroblast HFF.
Profiles of identified proteins in MS Experiment 2

Data2. AccessionNo (IPI)	Gene Symbol	Description	Score	Mass	# of identified (spectrum)	# of samples identified	Total peptides	# of peptides used	iPS_CFB46/ HFF
IPI00386258	MTCH1	MTCH1 Isoform 1 of Mitochondrial carrier	487	41517	14	4	2	1	NaN
IPI00396435	DHX15	DHX15 Putative pre-mRNA-splicing factor	80	90875	2	1	1	1	9999
IPI00412713	SAMM50	SAMM50 Sorting and assembly machinery	65	51943	2	1	1	1	9999
IPI00413784	SEPN1	SEPN1 Isoform 1 of Selenoprotein N	274	65907	6	3	1	1	9999
IPI00414320	ANXA11	ANXA11 cDNA FLJ55482, highly similar tc	160	6NaN5	5	1	3	3	9999
IPI00420014	SNRNP200	SNRNP200 Isoform 1 of U5 small nuclear	316	244353	5	2	2	2	9999
IPI00445690	PHLDB2	PHLDB2 cDNA FLJ43567 fis, clone PUAE	39	43398	1	1	1	1	NaN
IPI00455473	MIA3	MIA3 Isoform 1 of Melanoma inhibitory act	64	213570	3	2	3	3	9999
IPI00470360	KIRREL	KIRREL Isoform 1 of Kin of IRRE-like prot	86	83484	2	1	1	1	9999
IPI00479357	BSCL2	BSCL2 Seipin	178	51255	2	1	1	1	9999
IPI00555902	OCIAD2	OCIAD2 Isoform 1 of OCIA domain-contai	568	16943	14	4	3	3	9999
IPI00645966	NONO	NONO 24 kDa protein	473	23658	8	2	2	2	9999
IPI00646426	MRS2	MRS2 Isoform 2 of Magnesium transporte	151	46461	6	3	2	2	9999
IPI00646963	REEP6	REEP6 Receptor accessory protein 6, isof	279	23403	7	3	1	1	9999
IPI00783698	TMEM87A	TMEM87A Isoform 1 of Transmembrane p	197	63389	7	4	2	1	9999
IPI00788072	STX5	STX5 Protein	75	17601	2	1	2	1	NaN
IPI00790232	FOXP1	FOXP1 18 kDa protein	70	18173	3	2	1	1	9999
IPI00848226	GNB2L1	GNB2L1 Guanine nucleotide-binding prote	65	35055	3	2	1	1	9999
IPI00902680	SAMM50	SAMM50 cDNA FLJ39696 fis, clone SMIN	65	30193	2	2	1	1	NaN
IPI00936742	LOC1002934	LOC100293491 similar to golgi membrane	55	14512	2	2	1	1	9999
IPI00016342	RAB7A	RAB7A Ras-related protein Rab-7a	3033	23475	78	4	10	9	0.525
IPI00007928	PRPF8	PRPF8 Pre-mRNA-processing-splicing fac	412	273427	11	3	3	3	5.202
IPI00000015	SFRS4	SFRS4 Splicing factor, arginine/serine-rich	45	56645	2	1	1	1	NaN
IPI00000513	CDH1	CDH1 Cadherin 1, type 1, E-cadherin (Epi	486	90886	14	3	3	3	NaN
IPI00000874	PRDX1	PRDX1 Peroxiredoxin-1	79	22096	3	1	1	1	NaN
IPI00001895	PCDH8	PCDH8 Isoform 1 of Protocadherin-8	38	112949	1	1	1	1	9999
IPI00003377	SFRS7	SFRS7 Isoform 1 of Splicing factor, argini	92	27350	2	1	1	1	9999
IPI00007611	ATP5O	ATP5O ATP synthase subunit O, mitochor	94	23263	2	2	1	1	9999
IPI00007866	RAB17	RAB17 Ras-related protein Rab-17	59	23476	2	1	1	1	9999
IPI00008239	GPRC5B	GPRC5B cDNA FLJ55176, highly similar t	163	47664	3	1	1	1	9999
IPI00008982	ALDH18A1	ALDH18A1 Isoform Long of Delta-1-pyrroli	124	87248	3	2	1	1	9999
IPI00009634	SQRDL	SQRDL Sulfide:quinone oxidoreductase, n	146	49929	3	1	1	1	9999
IPI00009885	FKBP11	FKBP11 FK506-binding protein 11	0	22166	1	1	1	1	NaN
IPI00010270	RAC2	RAC2 Ras-related C3 botulinum toxin sub	151	21415	8	4	2	2	NaN
IPI00010896	CLIC1	CLIC1 Chloride intracellular channel protei	43	26906	1	1	1	1	NaN

Data2. AccessionNo (IPI)	iPS_CFB50/ HFF	iPS_CFB46/ H9ES	iPS_CFB50/ H9ES	iPS_CFB46/ NTU1ES	iPS_CFB50/ NTU1ES	H9ES/ HFF	NTU1ES/ HFF
IPI00386258	NaN	-9999	-9999	-9999	-9999	9999	9999
IPI00396435	9999	0.407	0.262	1.36	0.874	9999	9999
IPI00412713	9999	0.815	0.646	0.946	0.748	9999	9999
IPI00413784	9999	9999	9999	1.367	1.271	NaN	9999
IPI00414320	9999	0.973	0.751	0.839	0.646	9999	9999
IPI00420014	9999	0.486	0.374	0.982	0.711	9999	9999
IPI00445690	9999	NaN	9999	-9999	0.762	NaN	9999
IPI00455473	9999	9999	9999	0.937	NaN	NaN	9999
IPI00470360	9999	9999	9999	0.885	0.177	NaN	9999
IPI00479357	9999	2.687	3.183	1.404	1.659	9999	9999
IPI00555902	9999	0.994	0.77	1.054	0.815	9999	9999
IPI00645966	9999	2.304	1.219	4.08	2.153	9999	9999
IPI00646426	9999	0.909	0.868	0.575	0.599	9999	9999
IPI00646963	9999	2.387	4.335	0.827	1.498	9999	9999
IPI00783698	9999	2.534	1.768	0.797	0.554	9999	9999
IPI00788072	9999	NaN	9999	-9999	1.364	NaN	9999
IPI00790232	NaN	9999	NaN	2.178	-9999	NaN	9999
IPI00848226	9999	0.638	0.621	1.272	1.234	9999	9999
IPI00902680	NaN	NaN	NaN	-9999	-9999	NaN	9999
IPI00936742	9999	1.4	1.148	0.753	0.616	9999	9999
IPI00016342	0.565	0.989	1.087	0.981	1.08	0.486	0.NaN
IPI00007928	5.752	0.875	0.933	0.757	0.791	5.629	7.NaN
IPI00000015	NaN	NaN	NaN	NaN	NaN	NaN	NaN
IPI00000513	0.631	0.442	0.409	0.724	0.69	1.594	NaN
IPI00000874	NaN	NaN	NaN	NaN	NaN	NaN	NaN
IPI00001895	NaN	0.572	-9999	9999	NaN	9999	NaN
IPI00003377	NaN	0.369	-9999	9999	NaN	9999	NaN
IPI00007611	9999	0.203	0.128	9999	9999	9999	NaN
IPI00007866	9999	9999	9999	9999	9999	NaN	NaN
IPI00008239	9999	1.795	0.655	9999	9999	9999	NaN
IPI00008982	9999	2.221	1.686	9999	9999	9999	NaN
IPI00009634	NaN	9999	NaN	9999	NaN	NaN	NaN
IPI00009885	NaN	NaN	NaN	NaN	NaN	NaN	NaN
IPI00010270	NaN	9999	9999	1.032	1.363	-9999	NaN
IPI00010896	NaN	NaN	NaN	NaN	NaN	NaN	NaN

Table S1 Identified proteins in the human membrane proteome of hiPSCs, hESCs and somatic foreskin fibroblast HFF.
Profiles of identified proteins in MS Experiment 2

Data2. AccessionNo (IPI)	Gene Symbol	Description	Score	Mass	# of identified (spectrum)	# of samples identified	Total peptides	# of peptides used	iPS_CFB46/ HFF
IPI00013676	SQSTM1	SQSTM1 Phosphotyrosine independent lig	91	45693	2	1	1	1	NaN
IPI00014232	ARL6IP1	ARL6IP1 ADP-ribosylation factor-like prote	435	23347	8	4	3	1	2.359
IPI00014376	RAB31	RAB31 RAB31, member RAS oncogene fa	166	21686	5	1	2	2	NaN
IPI00016334	MCAM	MCAM Isoform 1 of Cell surface glycoprote	171	71563	4	1	2	2	NaN
IPI00024046	CDH13	CDH13 cDNA FLJ52398, highly similar to (	70	83356	2	1	1	1	NaN
IPI00024670	REEP5	REEP5 Receptor expression-enhancing pr	53	21479	1	1	1	1	NaN
IPI00026089	SF3B1	SF3B1 Splicing factor 3B subunit 1	38	145723	1	1	1	1	9999
IPI00026271	RPS14	RPS14 40S ribosomal protein S14	403	16263	12	4	1	1	9999
IPI00026994	PRAF2;WDR4	PRAF2;WDR45 PRA1 family protein 2	101	19246	3	1	1	1	NaN
IPI00028588	ProSAPiP1	ProSAPiP1 Uncharacterized protein KIAA(	0	71747	1	1	1	1	NaN
IPI00030275	TRAP1	TRAP1 Heat shock protein 75 kDa, mitoch	750	80060	1	1	1	1	9999
IPI00031822	SLC5A6	SLC5A6 Sodium-dependent multivitamin tr	84	68596	2	1	1	1	NaN
IPI00032003	EMD	EMD Emerin	136	28976	5	3	1	1	NaN
IPI00056310	SCAMP4	SCAMP4 Isoform 1 of Secretory carrier-as	48	25711	2	1	1	1	NaN
IPI00060546	C10orf35	C10orf35 Uncharacterized protein C10orf3	151	13230	3	2	1	1	9999
IPI00060990	UGT3A1	UGT3A1 UDP-glucuronosyltransferase 3A	37	59113	1	1	1	1	NaN
IPI00063334	TMEM41A	TMEM41A Transmembrane protein 41A	40	29646	1	1	1	1	9999
IPI00152959	MFSD1	MFSD1 Putative uncharacterized protein M	158	51433	3	1	1	1	NaN
IPI00167261	C2orf61	C2orf61 Uncharacterized protein C2orf61	43	19910	1	1	1	1	NaN
IPI00179713	IGF2BP2	IGF2BP2 Isoform 1 of Insulin-like growth fa	44	66081	1	1	1	1	NaN
IPI00218606	RPS23	RPS23 40S ribosomal protein S23	479	15798	12	4	1	1	NaN
IPI00220473	ATP2C1	ATP2C1 Isoform 2 of Calcium-transporting	65	96897	2	1	1	1	9999
IPI00290544	GDAP1	GDAP1 Ganglioside-induced differentiatior	73	41226	2	2	1	1	NaN
IPI00295774	SLC23A2	SLC23A2 Solute carrier family 23 member	41	70291	1	1	1	1	NaN
IPI00299719	TCIRG1	TCIRG1 Isoform Long of V-type proton AT	1042	92908	24	1	9	7	2.304
IPI00301263	CAD	CAD CAD protein	86	242829	1	1	1	1	9999
IPI00302605	SLC30A7	SLC30A7 Zinc transporter 7	46	41599	1	1	1	1	NaN
IPI00329025	JAGN1	JAGN1 Protein jagunal homolog 1	57	21111	2	1	1	1	NaN
IPI00384280	PCYOX1	PCYOX1 Prenylcysteine oxidase 1	81	56604	2	2	1	1	NaN
IPI00395808	SLC17A5	SLC17A5 Sialin	40	58683	1	1	1	1	NaN
IPI00399320	TMEM59	TMEM59 Transmembrane protein 59	109	36200	3	1	1	1	9999
IPI00412579	RPL10A	RPL10A 60S ribosomal protein L10a	75	24816	2	2	1	1	NaN
IPI00414231	LRP10	LRP10 Isoform 1 of Low-density lipoprotei	113	76145	3	1	2	2	9999
IPI00470674	CYB5R1	CYB5R1 NADH-cytochrome b5 reductase	73	34073	2	1	1	1	NaN
IPI00555703	TMEM41B	TMEM41B Isoform 1 of Transmembrane p	39	32492	1	1	1	1	NaN

Data2. AccessionNo (IPI)	iPS_CFB50/ HFF	iPS_CFB46/ H9ES	iPS_CFB50/ H9ES	iPS_CFB46/ NTU1ES	iPS_CFB50/ NTU1ES	H9ES/ HFF	NTU1ES/ HFF
IPI00013676	NaN	NaN	NaN	NaN	NaN	NaN	NaN
IPI00014232	2.249	2.898	7.215	1.754	4.993	NaN	NaN
IPI00014376	-9999	NaN	-9999	1.433	-9999	0.608	NaN
IPI00016334	NaN	NaN	NaN	NaN	NaN	NaN	NaN
IPI00024046	NaN	NaN	NaN	NaN	NaN	NaN	NaN
IPI00024670	NaN	NaN	NaN	NaN	NaN	NaN	NaN
IPI00026089	NaN	9999	NaN	9999	NaN	NaN	NaN
IPI00026271	NaN	0.544	-9999	9999	NaN	9999	NaN
IPI00026994	NaN	NaN	NaN	NaN	NaN	NaN	NaN
IPI00028588	NaN	NaN	NaN	NaN	NaN	NaN	NaN
IPI00030275	9999	0.688	0.465	9999	9999	9999	NaN
IPI00031822	NaN	NaN	NaN	NaN	NaN	NaN	NaN
IPI00032003	NaN	NaN	NaN	NaN	NaN	NaN	NaN
IPI00056310	NaN	NaN	NaN	NaN	NaN	NaN	NaN
IPI00060546	9999	9999	9999	9999	9999	NaN	NaN
IPI00060990	NaN	-9999	-9999	NaN	NaN	9999	NaN
IPI00063334	9999	9999	9999	9999	9999	NaN	NaN
IPI00152959	NaN	NaN	NaN	NaN	NaN	NaN	NaN
IPI00167261	NaN	NaN	NaN	NaN	NaN	NaN	NaN
IPI00179713	9999	-9999	1.002	NaN	9999	9999	NaN
IPI00218606	NaN	-9999	-9999	NaN	NaN	9999	NaN
IPI00220473	9999	9999	9999	9999	9999	NaN	NaN
IPI00290544	NaN	NaN	NaN	NaN	NaN	NaN	NaN
IPI00295774	NaN	NaN	NaN	NaN	NaN	NaN	NaN
IPI00299719	2.207	1.002	0.661	1.459	0.662	1.547	NaN
IPI00301263	9999	0.601	1.351	9999	9999	9999	NaN
IPI00302605	NaN	NaN	NaN	NaN	NaN	NaN	NaN
IPI00329025	NaN	NaN	NaN	NaN	NaN	NaN	NaN
IPI00384280	NaN	NaN	NaN	NaN	NaN	NaN	NaN
IPI00395808	NaN	NaN	NaN	NaN	NaN	NaN	NaN
IPI00399320	NaN	9999	NaN	9999	NaN	NaN	NaN
IPI00412579	NaN	-9999	-9999	NaN	NaN	9999	NaN
IPI00414231	NaN	0.723	-9999	9999	NaN	9999	NaN
IPI00470674	NaN	NaN	NaN	NaN	NaN	NaN	NaN
IPI00555703	9999	NaN	9999	NaN	9999	NaN	NaN

Table S1 Identified proteins in the human membrane proteome of hiPSCs, hESCs and somatic foreskin fibroblast HFF.
Profiles of identified proteins in MS Experiment 2

Data2. AccessionNo (IPI)	Gene Symbol	Description	Score	Mass	# of identified (spectrum)	# of samples identified	Total peptides	# of peptides used	iPS_CFB46/ HFF
IPI00747078	GANAB	GANAB GANAB protein (Fragment)	391	34558	3	1	1	1	NaN
IPI00783271	LRPPRC	LRPPRC Leucine-rich PPR motif-containir	636	157805	14	3	5	5	0.632
IPI00892737	-	- Major histocompatibility complex, class I,	1100	38307	1	1	1	1	NaN
IPI00908672	HGSNAT	HGSNAT heparan-alpha-glucosaminide N-	42	70451	1	1	1	1	NaN

Data2. AccessionNo (IPI)	iPS_CFB50/ HFF	iPS_CFB46/ H9ES	iPS_CFB50/ H9ES	iPS_CFB46/ NTU1ES	iPS_CFB50/ NTU1ES	H9ES/ HFF	NTU1ES/ HFF
IPI00747078	NaN	NaN	NaN	NaN	NaN	NaN	NaN
IPI00783271	0.423	0.634	0.312	2.079	0.846	1.332	NaN
IPI00892737	NaN	NaN	NaN	NaN	NaN	NaN	NaN
IPI00908672	NaN	NaN	NaN	NaN	NaN	NaN	NaN

Supplementary Table S2

The integrated datasets for the comparison of hiPSCs/HFF, hESCs/HFF, and hiPSCs/hESCs.

Table S2: The integrated datasets for the comparison of hiPSCs/HFF, hESCs/HFF, and hiPSCs/hESCs.

Integrated dataset hiPSCs/HFF		
IPI	Gene Symbol	log2 ratio
IPI00000105	MVP	-0.974
IPI00000115	CNIH4	0.492
IPI00000138	MGAT1	0.933
IPI00000190	CD81	-3.731
IPI00000425	ATP6V0A2	1.187
IPI00000513	CDH1	-0.012
IPI00000643	BAG2	-1.778
IPI00000816	YWHAE	-1.793
IPI00000875	TUT1;EEF1G	-0.729
IPI00000877	HYOU1	1.244
IPI00000948	TBL2	-0.114
IPI00000980	TOMM7	1.505
IPI00001091	AFG3L2	-1.052
IPI00001159	GCN1L1	0.357
IPI00001578	C17orf62	0.143
IPI00001639	KPNB1	-1.269
IPI00001754	F11R	1.850
IPI00001755	GPC6	-0.543
IPI00001891	AUP1	-0.491
IPI00002070	LRRC8A	1.607
IPI00002149	SAR1B	-0.703
IPI00002230	NCEH1	-0.989
IPI00002236	MFGE8	0.401
IPI00002307	NLGN3	-1.534
IPI00002372	ABCD3	0.078
IPI00002459	ANXA6	-1.571
IPI00002478	ECE1	-1.416
IPI00002506	ALG5	-0.036
IPI00002520	SHMT2	0.766
IPI00002732	EXTL2	0.703
IPI00002790	SEL1L	-0.345
IPI00002948	LIN28	0.951
IPI00003348	GNB2	-0.819
IPI00003362	HSPA5	0.285
IPI00003411	PTGIS	-3.095
IPI00003431	MAPK6	0.469
IPI00003635	ERGIC1	-0.556
IPI00003802	MAN2A1	0.595
IPI00003831	ATP2B3	-1.144
IPI00003833	MTCH2	1.901
IPI00003865	HSPA8	0.191
IPI00003909	SLC2A3	3.671
IPI00003918	RPL4	0.859
IPI00003926	CLN8	0.833
IPI00003935	HIST2H2BE	-0.228
IPI00003968	NDUFA9	0.918

Integrated dataset hESCs/HFF		
IPI	Gene Symbol	log2 ratio
IPI00000115	CNIH4	1.049
IPI00000138	MGAT1	0.850
IPI00000190	CD81	-2.829
IPI00000425	ATP6V0A2	1.176
IPI00000513	CDH1	0.027
IPI00000875	TUT1;EEF1G	-0.714
IPI00000877	HYOU1	1.763
IPI00000948	TBL2	-0.317
IPI00000980	TOMM7	1.676
IPI00001091	AFG3L2	-0.173
IPI00001159	GCN1L1	0.004
IPI00001578	C17orf62	-0.300
IPI00001639	KPNB1	-1.288
IPI00001754	F11R	2.097
IPI00001755	GPC6	-0.837
IPI00002230	NCEH1	-1.090
IPI00002236	MFGE8	-0.135
IPI00002307	NLGN3	-1.133
IPI00002372	ABCD3	-0.404
IPI00002459	ANXA6	-1.278
IPI00002478	ECE1	-1.702
IPI00002506	ALG5	-0.635
IPI00002520	SHMT2	0.865
IPI00002732	EXTL2	0.453
IPI00002790	SEL1L	-0.565
IPI00002948	LIN28	0.809
IPI00003348	GNB2	-0.854
IPI00003362	HSPA5	0.030
IPI00003411	PTGIS	-2.694
IPI00003431	MAPK6	0.451
IPI00003635	ERGIC1	-0.484
IPI00003802	MAN2A1	0.463
IPI00003831	ATP2B3	-0.666
IPI00003833	MTCH2	1.538
IPI00003865	HSPA8	-0.072
IPI00003909	SLC2A3	3.326
IPI00003918	RPL4	0.882
IPI00003925	PDHB	0.478
IPI00003926	CLN8	1.018
IPI00003935	HIST2H2BE	-0.546
IPI00003968	NDUFA9	1.216
IPI00003985	BCS1L	-0.197
IPI00004669	GALNT2	0.099
IPI00004671	GOLGB1	0.877
IPI00004962	GOLIM4	-0.486
IPI00005024	MYBBP1A	0.122

Integrated dataset hiPSCs/hESCs		
IPI	Gene Symbol	log2 ratio
IPI00000115	CNIH4	-0.424
IPI00000138	MGAT1	0.055
IPI00000190	CD81	-0.922
IPI00000425	ATP6V0A2	-0.064
IPI00000513	CDH1	-0.280
IPI00000875	TUT1;EEF1G	-0.037
IPI00000877	HYOU1	-0.614
IPI00000948	TBL2	0.182
IPI00000980	TOMM7	-0.192
IPI00001091	AFG3L2	-0.320
IPI00001159	GCN1L1	0.295
IPI00001578	C17orf62	0.422
IPI00001639	KPNB1	0.017
IPI00001754	F11R	-0.175
IPI00001755	GPC6	-0.976
IPI00002070	LRRC8A	0.848
IPI00002225	LASS4	-0.349
IPI00002230	NCEH1	0.093
IPI00002236	MFGE8	0.395
IPI00002307	NLGN3	-0.422
IPI00002372	ABCD3	0.387
IPI00002406	BCAM	0.005
IPI00002459	ANXA6	-0.310
IPI00002478	ECE1	0.295
IPI00002506	ALG5	0.285
IPI00002520	SHMT2	-0.103
IPI00002732	EXTL2	0.229
IPI00002790	SEL1L	0.128
IPI00002948	LIN28	0.122
IPI00003348	GNB2	0.014
IPI00003362	HSPA5	0.249
IPI00003431	MAPK6	-0.004
IPI00003635	ERGIC1	-0.075
IPI00003802	MAN2A1	0.189
IPI00003831	ATP2B3	-0.499
IPI00003833	MTCH2	0.333
IPI00003865	HSPA8	0.211
IPI00003909	SLC2A3	0.302
IPI00003918	RPL4	-0.053
IPI00003935	HIST2H2BE	0.252
IPI00003968	NDUFA9	-0.427
IPI00003985	BCS1L	0.283
IPI00004669	GALNT2	0.451
IPI00004671	GOLGB1	-0.020
IPI00004962	GOLIM4	0.548
IPI00005024	MYBBP1A	-0.324

Table S2: The integrated datasets for the comparison of hiPSCs/HFF, hESCs/HFF, and hiPSCs/hESCs.

Integrated dataset hiPSCs/HFF		
IPI	Gene Symbol	log2 ratio
IPI00003985	BCS1L	0.109
IPI00004669	GALNT2	0.635
IPI00004671	GOLGB1	0.881
IPI00004672	HLA-H	-2.490
IPI00004962	GOLIM4	-0.023
IPI00005024	MYBBP1A	-0.767
IPI00005068	SLC44A1	0.240
IPI00005107	NPC1	-0.789
IPI00005181	PLSCR1	-0.928
IPI00005198	ILF2	0.785
IPI00005202	PGRMC2	-0.403
IPI00005707	MRC2	-2.718
IPI00005719	RAB1A	-1.579
IPI00005728	RER1	0.003
IPI00005737	SURF4	-0.513
IPI00005745	SPTLC1	0.447
IPI00005751	SPTLC2	0.875
IPI00006072	SEC61G	0.577
IPI00006205	SLC33A1	-1.408
IPI00006211	VAPB	0.455
IPI00006250	DHRS3	-2.683
IPI00006379	NOP58	-0.522
IPI00006433	DNAJC16	1.025
IPI00006482	ATP1A1	0.752
IPI00006579	COX4I1	1.299
IPI00006657	FAM20B	0.427
IPI00006666	SLC16A3	-0.794
IPI00006721	OPA1	4.732
IPI00006865	SEC22B	-1.724
IPI00006957	DHRS7	-0.697
IPI00007061	GOLT1B	-0.154
IPI00007068	ACTR3B	-1.496
IPI00007084	SLC25A13	2.267
IPI00007165	DOLK	-0.442
IPI00007166	IER3IP1	0.285
IPI00007183	ATL2	1.706
IPI00007188	SLC25A5	2.078
IPI00007309	LOC10028793	0.794
IPI00007426	ARL6IP5	-1.538
IPI00007676	HSD17B12	0.622
IPI00007730	C14orf1	-0.741
IPI00007752	TUBB2C	-0.458
IPI00007765	HSPA9	0.952
IPI00007928	PRPF8	2.469
IPI00007940	ERLIN1	-0.143
IPI00007961	MT-ND1	0.597

Integrated dataset hESCs/HFF		
IPI	Gene Symbol	log2 ratio
IPI00005068	SLC44A1	-0.280
IPI00005181	PLSCR1	-0.906
IPI00005198	ILF2	1.531
IPI00005202	PGRMC2	-0.327
IPI00005719	RAB1A	-1.109
IPI00005728	RER1	-0.269
IPI00005737	SURF4	-0.563
IPI00005745	SPTLC1	0.784
IPI00005751	SPTLC2	0.807
IPI00006072	SEC61G	0.316
IPI00006205	SLC33A1	-0.289
IPI00006211	VAPB	-0.074
IPI00006250	DHRS3	-2.044
IPI00006379	NOP58	-0.612
IPI00006433	DNAJC16	0.160
IPI00006482	ATP1A1	0.772
IPI00006579	COX4I1	1.794
IPI00006657	FAM20B	0.002
IPI00006666	SLC16A3	-1.120
IPI00006721	OPA1	4.249
IPI00006865	SEC22B	-1.591
IPI00006957	DHRS7	-0.117
IPI00007061	GOLT1B	-0.473
IPI00007084	SLC25A13	2.440
IPI00007165	DOLK	-0.589
IPI00007166	IER3IP1	0.237
IPI00007183	ATL2	1.792
IPI00007188	SLC25A5	2.171
IPI00007309	LOC100287932	1.306
IPI00007426	ARL6IP5	-1.585
IPI00007676	HSD17B12	0.617
IPI00007730	C14orf1	-0.362
IPI00007752	TUBB2C	-0.495
IPI00007765	HSPA9	0.576
IPI00007928	PRPF8	2.665
IPI00007940	ERLIN1	-0.529
IPI00007961	MT-ND1	0.842
IPI00007979	MT-ND2	1.423
IPI00008167	ATP1B3	0.676
IPI00008207	MAN1B1	-0.203
IPI00008274	CAP1	-0.685
IPI00008338	ABCC1	0.234
IPI00008438	RPS10	0.119
IPI00008495	MT-ND4	-0.482
IPI00008511	MT-ND5	0.777
IPI00008530	RPLP0	-0.329

Integrated dataset hiPSCs/hESCs		
IPI	Gene Symbol	log2 ratio
IPI00005068	SLC44A1	0.519
IPI00005107	NPC1	-0.543
IPI00005181	PLSCR1	-0.044
IPI00005198	ILF2	-0.456
IPI00005202	PGRMC2	-0.038
IPI00005707	MRC2	-1.959
IPI00005719	RAB1A	-0.491
IPI00005728	RER1	0.252
IPI00005737	SURF4	0.049
IPI00005745	SPTLC1	0.185
IPI00005751	SPTLC2	0.013
IPI00006072	SEC61G	0.239
IPI00006205	SLC33A1	-1.140
IPI00006211	VAPB	0.303
IPI00006250	DHRS3	-0.660
IPI00006379	NOP58	0.070
IPI00006433	DNAJC16	0.843
IPI00006482	ATP1A1	-0.117
IPI00006579	COX4I1	-0.429
IPI00006657	FAM20B	0.405
IPI00006666	SLC16A3	-0.048
IPI00006702	PELP1	-0.242
IPI00006721	OPA1	0.176
IPI00006865	SEC22B	-0.009
IPI00006957	DHRS7	-0.501
IPI00007034	UBAC2	0.245
IPI00007061	GOLT1B	0.298
IPI00007084	SLC25A13	-0.133
IPI00007144	RPL26L1	0.152
IPI00007165	DOLK	0.142
IPI00007166	IER3IP1	0.015
IPI00007183	ATL2	-0.107
IPI00007188	SLC25A5	-0.207
IPI00007309	LOC100287932;T	0.039
IPI00007676	HSD17B12	-0.012
IPI00007730	C14orf1	-0.355
IPI00007752	TUBB2C	-0.079
IPI00007765	HSPA9	0.163
IPI00007928	PRPF8	-0.156
IPI00007940	ERLIN1	0.087
IPI00007961	MT-ND1	-0.253
IPI00007979	MT-ND2	0.064
IPI00008167	ATP1B3	-0.035
IPI00008207	MAN1B1	0.330
IPI00008274	CAP1	-0.140
IPI00008438	RPS10	0.084

Table S2: The integrated datasets for the comparison of hiPSCs/HFF, hESCs/HFF, and hiPSCs/hESCs.

Integrated dataset hiPSCs/HFF		
IPI	Gene Symbol	log2 ratio
IPI00007979	MT-ND2	1.531
IPI00008167	ATP1B3	0.701
IPI00008207	MAN1B1	0.140
IPI00008274	CAP1	-1.582
IPI00008318	EPHA4	-0.549
IPI00008338	ABCC1	-0.084
IPI00008438	RPS10	0.224
IPI00008495	MT-ND4	-0.648
IPI00008511	MT-ND5	0.419
IPI00008530	RPLP0	-0.402
IPI00008557	IGF2BP1	0.176
IPI00008599	EBP	1.985
IPI00008732	TRABD	-1.041
IPI00008964	RAB1B	-0.853
IPI00008982	ALDH18A1	-0.581
IPI00008986	SLC7A5	3.434
IPI00008998	PTPLAD1	1.537
IPI00009030	LAMP2	-2.747
IPI00009111	TPBG	-2.018
IPI00009145	MAN1A2	0.175
IPI00009225	STX8	0.507
IPI00009235	SSR3	0.802
IPI00009236	CAV1	-3.113
IPI00009253	NAPA	-0.548
IPI00009276	PROCR	-1.771
IPI00009342	IQGAP1	-0.370
IPI00009346	TMEM14C	0.471
IPI00009364	ORMDL1	0.819
IPI00009368	SFXN1	1.629
IPI00009407	DAD1	0.516
IPI00009456	NT5E	-1.760
IPI00009865	KRT10	0.108
IPI00009890	SERPINE2	-2.217
IPI00009896	EPHX1	-0.206
IPI00009922	C14orf156	0.561
IPI00009950	LMAN2	0.130
IPI00009960	IMMT	1.385
IPI00009976	TMED1	0.737
IPI00010153	RPL23	0.088
IPI00010218	CYP20A1	-0.281
IPI00010255	RHBDD2	1.676
IPI00010271	RAC1	0.066
IPI00010418	MYO1C	-2.319
IPI00010438	SNAP23	-0.663
IPI00010697	ITGA6	-0.272
IPI00010740	SFPQ	0.733

Integrated dataset hESCs/HFF		
IPI	Gene Symbol	log2 ratio
IPI00008557	IGF2BP1	-0.608
IPI00008599	EBP	2.043
IPI00008708	RSL1D1	1.593
IPI00008964	RAB1B	-0.755
IPI00008986	SLC7A5	3.597
IPI00008998	PTPLAD1	1.404
IPI00009030	LAMP2	-2.884
IPI00009111	TPBG	-2.168
IPI00009145	MAN1A2	-0.296
IPI00009225	STX8	0.495
IPI00009235	SSR3	0.723
IPI00009236	CAV1	-3.461
IPI00009253	NAPA	-0.016
IPI00009276	PROCR	-1.362
IPI00009328	EIF4A3	-0.792
IPI00009342	IQGAP1	-0.659
IPI00009346	TMEM14C	0.137
IPI00009364	ORMDL1	1.033
IPI00009368	SFXN1	1.652
IPI00009407	DAD1	0.432
IPI00009456	NT5E	-1.658
IPI00009865	KRT10	0.557
IPI00009896	EPHX1	-0.443
IPI00009922	C14orf156	0.354
IPI00009950	LMAN2	-0.496
IPI00009960	IMMT	1.429
IPI00009976	TMED1	0.227
IPI00010153	RPL23	-0.117
IPI00010218	CYP20A1	-0.264
IPI00010255	RHBDD2	0.890
IPI00010271	RAC1	-0.224
IPI00010418	MYO1C	-2.126
IPI00010438	SNAP23	-0.635
IPI00010491	RAB27B	-0.446
IPI00010697	ITGA6	-0.259
IPI00010740	SFPQ	1.103
IPI00010746	PTDSS1	0.760
IPI00010796	P4HB	-1.192
IPI00011084	CLDN6	4.961
IPI00011229	CTSD	-2.400
IPI00011253	RPS3	-0.344
IPI00011284	COMT	-1.728
IPI00011416	ECH1	-0.351
IPI00011578	NPTN	-1.421
IPI00011654	TUBB	0.263
IPI00011694	PRSS1	-0.251

Integrated dataset hiPSCs/hESCs		
IPI	Gene Symbol	log2 ratio
IPI00008495	MT-ND4	-0.018
IPI00008511	MT-ND5	-0.379
IPI00008530	RPLP0	-0.095
IPI00008557	IGF2BP1	0.762
IPI00008599	EBP	-0.090
IPI00008708	RSL1D1	-0.546
IPI00008732	TRABD	-2.113
IPI00008964	RAB1B	-0.100
IPI00008986	SLC7A5	-0.010
IPI00008998	PTPLAD1	0.125
IPI00009030	LAMP2	-0.082
IPI00009111	TPBG	0.185
IPI00009145	MAN1A2	0.451
IPI00009225	STX8	0.106
IPI00009235	SSR3	0.057
IPI00009236	CAV1	0.195
IPI00009253	NAPA	-0.575
IPI00009276	PROCR	-0.632
IPI00009342	IQGAP1	0.284
IPI00009346	TMEM14C	0.355
IPI00009364	ORMDL1	-0.234
IPI00009368	SFXN1	-0.026
IPI00009407	DAD1	0.060
IPI00009456	NT5E	-0.325
IPI00009865	KRT10	-0.436
IPI00009890	SERPINE2	-0.370
IPI00009896	EPHX1	0.220
IPI00009922	C14orf156	0.127
IPI00009950	LMAN2	0.201
IPI00009960	IMMT	-0.415
IPI00009976	TMED1	0.488
IPI00010153	RPL23	0.183
IPI00010218	CYP20A1	-0.037
IPI00010255	RHBDD2	0.766
IPI00010271	RAC1	0.192
IPI00010418	MYO1C	-0.197
IPI00010438	SNAP23	-0.006
IPI00010440	HAX1	-1.282
IPI00010491	RAB27B	-0.362
IPI00010697	ITGA6	-0.033
IPI00010740	SFPQ	-0.420
IPI00010746	PTDSS1	0.021
IPI00010796	P4HB	-0.382
IPI00010951	EPPK1	2.570
IPI00011084	CLDN6	-0.188
IPI00011200	PHGDH	-0.268

Table S2: The integrated datasets for the comparison of hiPSCs/HFF, hESCs/HFF, and hiPSCs/hESCs.

Integrated dataset hiPSCs/HFF		
IPI	Gene Symbol	log2 ratio
IPI00010746	PTDSS1	0.729
IPI00010796	P4HB	-0.642
IPI00011084	CLDN6	4.424
IPI00011200	PHGDH	-0.415
IPI00011229	CTSD	-1.909
IPI00011253	RPS3	-0.058
IPI00011284	COMT	-1.516
IPI00011416	ECH1	-0.675
IPI00011578	NPTN	-1.329
IPI00011654	TUBB	0.144
IPI00011694	PRSS1	-0.045
IPI00011770	NDUFA4	2.120
IPI00012011	CFL1	-2.087
IPI00012048	NME1	-0.688
IPI00012486	SPNS1	-1.040
IPI00012490	ATP2B4	-2.488
IPI00012493	RPS20	0.534
IPI00012503	PSAP	-2.892
IPI00012511	RHOQ	-2.461
IPI00012512	RRAS2	-1.174
IPI00012728	ACSL1	-0.389
IPI00012750	RPS25	-0.042
IPI00013271	DERL1	0.056
IPI00013296	RPS18;RPS18	-0.229
IPI00013415	RPS7	-0.129
IPI00013421	GPM6B	0.446
IPI00013485	RPS2	-0.092
IPI00013508	ACTN1	-2.251
IPI00013683	TUBB3	0.112
IPI00013881	HNRNPH1	1.471
IPI00013895	S100A11	-2.095
IPI00013897	ADAM10	-0.837
IPI00013930	STX6	0.259
IPI00014053	TOMM40	2.201
IPI00014236	SLC39A14	0.322
IPI00014377	RAB32	-0.814
IPI00014577	RAB18	0.116
IPI00014812	MRPS21	-0.606
IPI00015102	ALCAM	-0.181
IPI00015148	RAP1B	-1.632
IPI00015602	TOMM70A	2.275
IPI00015833	CHCHD3	2.051
IPI00015902	PDGFRB	-0.751
IPI00015920	SLC25A10	1.161
IPI00015972	COX6C	-1.435
IPI00016014	ITM2C	2.611

Integrated dataset hESCs/HFF		
IPI	Gene Symbol	log2 ratio
IPI00011770	NDUFA4	2.223
IPI00012011	CFL1	-1.398
IPI00012048	NME1	-0.686
IPI00012486	SPNS1	-0.833
IPI00012490	ATP2B4	-3.065
IPI00012493	RPS20	0.090
IPI00012503	PSAP	-3.879
IPI00012512	RRAS2	-1.290
IPI00012750	RPS25	-0.240
IPI00012772	RPL8	0.067
IPI00013271	DERL1	0.116
IPI00013296	RPS18;RPS18F	-0.277
IPI00013415	RPS7	-0.245
IPI00013421	GPM6B	1.049
IPI00013485	RPS2	-0.023
IPI00013508	ACTN1	-2.696
IPI00013683	TUBB3	-0.274
IPI00013881	HNRNPH1	1.852
IPI00013895	S100A11	-1.590
IPI00013897	ADAM10	-0.622
IPI00013930	STX6	0.143
IPI00014053	TOMM40	1.957
IPI00014232	ARL6IP1	0.883
IPI00014236	SLC39A14	0.610
IPI00014377	RAB32	-1.301
IPI00014577	RAB18	-0.068
IPI00014812	MRPS21	-0.976
IPI00015102	ALCAM	-0.306
IPI00015148	RAP1B	-2.044
IPI00015602	TOMM70A	2.197
IPI00015833	CHCHD3	2.099
IPI00015902	PDGFRB	-0.680
IPI00015920	SLC25A10	1.238
IPI00015972	COX6C	-2.529
IPI00016014	ITM2C	2.578
IPI00016339	RAB5C	0.337
IPI00016342	RAB7A	-1.794
IPI00016372	RAB9A	-0.824
IPI00016405	OCIAD1	1.374
IPI00016513	RAB10	-0.484
IPI00016608	TMED2	0.182
IPI00016670	C11orf59	-1.044
IPI00016703	DHCR24	1.702
IPI00016786	CDC42	-0.884
IPI00017292	CTNNB1	-0.313
IPI00017297	MATR3	2.320

Integrated dataset hiPSCs/hESCs		
IPI	Gene Symbol	log2 ratio
IPI00011229	CTSD	0.329
IPI00011253	RPS3	-0.004
IPI00011284	COMT	0.474
IPI00011416	ECH1	-0.345
IPI00011454	GANAB	0.170
IPI00011578	NPTN	-0.095
IPI00011654	TUBB	-0.182
IPI00011694	PRSS1	0.186
IPI00011770	NDUFA4	-0.124
IPI00011913	HNRNPA0	-0.073
IPI00011970	HSD17B7	-0.017
IPI00012011	CFL1	-0.469
IPI00012486	SPNS1	-0.060
IPI00012490	ATP2B4	-0.020
IPI00012493	RPS20	0.336
IPI00012503	PSAP	0.801
IPI00012512	RRAS2	0.095
IPI00012540	PROM1	0.146
IPI00012750	RPS25	0.143
IPI00012772	RPL8	-0.097
IPI00013271	DERL1	0.021
IPI00013296	RPS18;RPS18P9	-0.017
IPI00013415	RPS7	0.094
IPI00013421	GPM6B	-0.623
IPI00013459	NDUFB5	0.479
IPI00013475	TUBB2A	-0.029
IPI00013485	RPS2	-0.091
IPI00013508	ACTN1	0.355
IPI00013623	SLC27A3	0.880
IPI00013683	TUBB3	0.294
IPI00013744	ITGA2	-0.060
IPI00013847	UQCRC1	-0.250
IPI00013895	S100A11	-0.579
IPI00013897	ADAM10	-0.236
IPI00013930	STX6	0.094
IPI00014053	TOMM40	0.050
IPI00014236	SLC39A14	-0.309
IPI00014377	RAB32	0.148
IPI00014577	RAB18	-0.018
IPI00014812	MRPS21	0.349
IPI00015102	ALCAM	-0.018
IPI00015148	RAP1B	0.394
IPI00015473	SLC1A3	-0.285
IPI00015602	TOMM70A	-0.357
IPI00015833	CHCHD3	-0.392
IPI00015902	PDGFRB	-0.011

Table S2: The integrated datasets for the comparison of hiPSCs/HFF, hESCs/HFF, and hiPSCs/hESCs.

Integrated dataset hiPSCs/HFF		
IPI	Gene Symbol	log2 ratio
IPI00016249	FXR1	2.130
IPI00016339	RAB5C	-0.606
IPI00016342	RAB7A	-0.923
IPI00016372	RAB9A	-1.698
IPI00016405	OCIAD1	0.676
IPI00016513	RAB10	-0.396
IPI00016608	TMED2	0.208
IPI00016670	C11orf59	-1.217
IPI00016703	DHCR24	2.229
IPI00016786	CDC42	-0.784
IPI00016968	SDHC	1.420
IPI00017292	CTNNB1	0.166
IPI00017297	MATR3	2.912
IPI00017334	PHB	1.754
IPI00017344	RAB5B	-2.011
IPI00017510	MT-CO2	0.845
IPI00017533	MT-ATP6;MT-(	3.145
IPI00017567	ENG	-1.232
IPI00017592	LETM1	1.878
IPI00017617	DDX5	-0.658
IPI00017767	MGST2	1.029
IPI00017895	GPD2	0.599
IPI00018146	YWHAQ	-2.404
IPI00018248	KDELR2	-1.459
IPI00018274	EGFR	-0.955
IPI00018364	RAP2B	-1.296
IPI00018415	TM9SF2	0.637
IPI00018855	ATP6V0C	-0.372
IPI00018871	ARL8B	-1.597
IPI00018953	DPP4	-4.201
IPI00019018	TM7SF2	-0.223
IPI00019141	AGPAT1	1.574
IPI00019146	CXADR	4.820
IPI00019353	AGK	0.215
IPI00019359	KRT9	0.385
IPI00019385	SSR4	0.444
IPI00019407	NSDHL	0.580
IPI00019472	SLC1A5	0.288
IPI00019502	MYH9	-1.405
IPI00019770	FAU	0.773
IPI00019899	SC4MOL	1.071
IPI00019906	BSG	1.622
IPI00019912	HSD17B4	0.106
IPI00020005	ARSE	1.155
IPI00020042	PSMC4	-2.569
IPI00020124	PI4K2A	-1.027

Integrated dataset hESCs/HFF		
IPI	Gene Symbol	log2 ratio
IPI00017334	PHB	2.136
IPI00017344	RAB5B	-2.113
IPI00017510	MT-CO2	1.293
IPI00017533	MT-ATP6;MT-C	2.908
IPI00017567	ENG	-2.269
IPI00017592	LETM1	1.797
IPI00017617	DDX5	-0.410
IPI00017767	MGST2	-0.074
IPI00017895	GPD2	0.790
IPI00017964	SNRPD3	0.646
IPI00018146	YWHAQ	-2.936
IPI00018246	HK1	0.413
IPI00018248	KDELR2	-1.362
IPI00018274	EGFR	-0.476
IPI00018364	RAP2B	-1.093
IPI00018415	TM9SF2	0.484
IPI00018855	ATP6V0C	-0.109
IPI00018871	ARL8B	-1.688
IPI00018953	DPP4	-3.817
IPI00019018	TM7SF2	-0.029
IPI00019141	AGPAT1	1.030
IPI00019146	CXADR	4.708
IPI00019353	AGK	0.514
IPI00019359	KRT9	0.307
IPI00019385	SSR4	0.228
IPI00019407	NSDHL	0.302
IPI00019472	SLC1A5	0.531
IPI00019502	MYH9	-1.547
IPI00019770	FAU	0.032
IPI00019899	SC4MOL	1.057
IPI00019906	BSG	1.755
IPI00019912	HSD17B4	0.606
IPI00020005	ARSE	0.997
IPI00020042	PSMC4	-2.659
IPI00020124	PI4K2A	-0.960
IPI00020418	RRAS	-1.809
IPI00020436	RAB11B	0.226
IPI00020470	GLT8D1	-0.773
IPI00020472	TMEM111	-0.139
IPI00020510	CISD1	1.744
IPI00020557	LRP1	-1.174
IPI00021048	MYOF	-1.524
IPI00021147	DEGS1	1.112
IPI00021263	YWHAZ	-1.857
IPI00021267	EPHA2	0.271
IPI00021304	KRT2	0.173

Integrated dataset hiPSCs/hESCs		
IPI	Gene Symbol	log2 ratio
IPI00015920	SLC25A10	-0.054
IPI00015972	COX6C	0.591
IPI00016014	ITM2C	0.013
IPI00016339	RAB5C	-0.710
IPI00016342	RAB7A	0.098
IPI00016373	RAB13	0.192
IPI00016405	OCIAD1	0.024
IPI00016513	RAB10	0.067
IPI00016597	CLN6	0.178
IPI00016608	TMED2	-0.026
IPI00016670	C11orf59	0.057
IPI00016676	TOMM20	-0.981
IPI00016703	DHCR24	0.530
IPI00016786	CDC42	0.075
IPI00016968	SDHC	-0.387
IPI00017292	CTNNB1	0.256
IPI00017297	MATR3	0.277
IPI00017334	PHB	-0.314
IPI00017344	RAB5B	0.080
IPI00017510	MT-CO2	-0.284
IPI00017533	MT-ATP6;MT-CO	0.216
IPI00017592	LETM1	0.010
IPI00017617	DDX5	-0.250
IPI00017767	MGST2	1.082
IPI00017895	GPD2	-0.270
IPI00018140	SYNCRIP	-1.143
IPI00018146	YWHAQ	0.508
IPI00018248	KDELR2	-0.124
IPI00018364	RAP2B	-0.226
IPI00018415	TM9SF2	0.130
IPI00018855	ATP6V0C	-0.522
IPI00018871	ARL8B	0.080
IPI00019141	AGPAT1	0.586
IPI00019146	CXADR	0.182
IPI00019353	AGK	-0.320
IPI00019359	KRT9	0.084
IPI00019385	SSR4	0.186
IPI00019407	NSDHL	0.263
IPI00019472	SLC1A5	-0.248
IPI00019502	MYH9	0.175
IPI00019770	FAU	0.719
IPI00019899	SC4MOL	0.171
IPI00019906	BSG	-0.164
IPI00019912	HSD17B4	-0.424
IPI00020004	TMEM97	0.129
IPI00020042	PSMC4	0.075

Table S2: The integrated datasets for the comparison of hiPSCs/HFF, hESCs/HFF, and hiPSCs/hESCs.

Integrated dataset hiPSCs/HFF		
IPI	Gene Symbol	log2 ratio
IPI00020418	RRAS	-1.701
IPI00020436	RAB11B	0.431
IPI00020470	GLT8D1	-0.857
IPI00020472	TMEM111	-0.036
IPI00020510	CISD1	1.805
IPI00020557	LRP1	-1.020
IPI00020599	CALR	-0.326
IPI00020850	PPP2R2B	-1.493
IPI00020944	FDFT1	0.746
IPI00021048	MYOF	-1.951
IPI00021058	SLC4A7	-0.153
IPI00021147	DEGS1	0.482
IPI00021263	YWHAZ	-1.707
IPI00021267	EPHA2	-0.160
IPI00021304	KRT2	-0.282
IPI00021428	ACTA1	-1.000
IPI00021439	ACTB	-1.196
IPI00021453	PPAP2B	-1.503
IPI00021695	ATP2B1	-0.648
IPI00021805	MGST1	0.636
IPI00021812	AHNAK	-1.626
IPI00021840	RPS6	-0.103
IPI00021983	NCSTN	-0.037
IPI00021985	TM9SF4	1.127
IPI00022018	DPM1	-0.368
IPI00022048	PTGFRN	-1.026
IPI00022143	ESYT1	-0.178
IPI00022202	SLC25A3	1.075
IPI00022275	SACM1L	1.083
IPI00022300	METTL7A	-0.406
IPI00022418	FN1	-1.622
IPI00022462	TFRC	0.049
IPI00022543	PIGK	0.571
IPI00022585	AKAP1	0.177
IPI00022608	SORL1	0.998
IPI00022744	CSE1L	0.646
IPI00022774	VCP	-0.355
IPI00022891	SLC25A4	3.204
IPI00022892	THY1	-2.316
IPI00023001	FAM162A	3.223
IPI00023030	SLC38A1	0.000
IPI00023064	NDUFAF4	0.740
IPI00023135	GOSR2	0.122
IPI00023334	MRPL4	3.310
IPI00023406	HCCS	2.398
IPI00023526	RAB6A	0.961

Integrated dataset hESCs/HFF		
IPI	Gene Symbol	log2 ratio
IPI00021338	DLAT	-0.102
IPI00021428	ACTA1	-0.901
IPI00021439	ACTB	-1.008
IPI00021453	PPAP2B	-0.902
IPI00021695	ATP2B1	-0.927
IPI00021805	MGST1	0.722
IPI00021840	RPS6	-0.102
IPI00021983	NCSTN	0.066
IPI00021985	TM9SF4	1.265
IPI00022018	DPM1	-0.192
IPI00022048	PTGFRN	-1.334
IPI00022143	ESYT1	-0.373
IPI00022202	SLC25A3	1.333
IPI00022275	SACM1L	1.238
IPI00022418	FN1	-1.992
IPI00022462	TFRC	0.393
IPI00022585	AKAP1	2.150
IPI00022744	CSE1L	0.298
IPI00022774	VCP	-0.456
IPI00022892	THY1	-2.027
IPI00023001	FAM162A	3.449
IPI00023030	SLC38A1	0.479
IPI00023064	NDUFAF4	1.133
IPI00023135	GOSR2	-0.060
IPI00023334	MRPL4	3.239
IPI00023406	HCCS	2.470
IPI00023500	DPAGT1	0.367
IPI00023526	RAB6A	0.869
IPI00023542	TMED9	0.346
IPI00023780	DNAJC5	-0.091
IPI00023860	NAP1L1	-0.733
IPI00023958	MPV17	-1.053
IPI00024067	CLTC	-1.189
IPI00024145	VDAC2	1.679
IPI00024266	MGST3	0.468
IPI00024282	RAB8B	-0.545
IPI00024551	C15orf24	0.853
IPI00024642	CCDC47	0.947
IPI00024650	SLC16A1	2.294
IPI00024742	UQCRQ	2.672
IPI00024757	UQCR	1.015
IPI00024919	PRDX3	0.671
IPI00024933	RPL12	-0.229
IPI00024975	KIF15	0.378
IPI00024976	TOMM22	1.820
IPI00025049	M6PR	0.505

Integrated dataset hiPSCs/hESCs		
IPI	Gene Symbol	log2 ratio
IPI00020124	PI4K2A	-0.079
IPI00020418	RRAS	0.077
IPI00020436	RAB11B	0.272
IPI00020470	GLT8D1	-0.105
IPI00020472	TMEM111	0.069
IPI00020510	CISD1	0.041
IPI00020557	LRP1	0.162
IPI00020944	FDFT1	0.218
IPI00021048	MYOF	0.073
IPI00021147	DEGS1	0.081
IPI00021263	YWHAZ	-0.102
IPI00021267	EPHA2	0.055
IPI00021304	KRT2	-0.489
IPI00021338	DLAT	0.102
IPI00021428	ACTA1	-0.142
IPI00021439	ACTB	-0.213
IPI00021453	PPAP2B	-0.621
IPI00021695	ATP2B1	0.030
IPI00021793	COX6A1;COX6A	-0.244
IPI00021805	MGST1	-0.063
IPI00021840	RPS6	-0.070
IPI00021842	APOE	0.114
IPI00021983	NCSTN	-0.072
IPI00021985	TM9SF4	-0.097
IPI00022018	DPM1	-0.183
IPI00022048	PTGFRN	0.286
IPI00022143	ESYT1	0.149
IPI00022202	SLC25A3	-0.123
IPI00022275	SACM1L	0.034
IPI00022277	CCDC56	-0.337
IPI00022418	FN1	0.350
IPI00022462	TFRC	-0.367
IPI00022543	PIGK	0.143
IPI00022585	AKAP1	-1.994
IPI00022774	VCP	0.080
IPI00022891	SLC25A4	-0.099
IPI00022892	THY1	-0.287
IPI00023001	FAM162A	-0.210
IPI00023030	SLC38A1	-0.361
IPI00023064	NDUFAF4	-0.413
IPI00023135	GOSR2	0.214
IPI00023334	MRPL4	0.050
IPI00023406	HCCS	-0.092
IPI00023526	RAB6A	0.045
IPI00023542	TMED9	0.013
IPI00023780	DNAJC5	-0.159

Table S2: The integrated datasets for the comparison of hiPSCs/HFF, hESCs/HFF, and hiPSCs/hESCs.

Integrated dataset hiPSCs/HFF		
IPI	Gene Symbol	log2 ratio
IPI00023542	TMED9	0.382
IPI00023780	DNAJC5	-0.298
IPI00023860	NAP1L1	-0.161
IPI00023958	MPV17	-1.085
IPI00024067	CLTC	-1.149
IPI00024143	AAAS	0.494
IPI00024145	VDAC2	1.555
IPI00024266	MGST3	0.833
IPI00024282	RAB8B	-0.688
IPI00024466	UGGT1	1.197
IPI00024551	C15orf24	1.213
IPI00024642	CCDC47	0.984
IPI00024650	SLC16A1	2.147
IPI00024742	UQCRQ	2.115
IPI00024757	UQCR	1.088
IPI00024919	PRDX3	0.860
IPI00024933	RPL12	-0.033
IPI00024976	TOMM22	1.934
IPI00025049	M6PR	0.542
IPI00025086	COX5A	1.113
IPI00025292	MPDU1	0.878
IPI00025329	RPL19	0.529
IPI00025366	CS	-0.653
IPI00025416	ACTG2	-0.744
IPI00025491	EIF4A1;SNOR	-0.406
IPI00025729	CAMLG	0.227
IPI00025818	GALNT1	0.238
IPI00025874	RPN1	0.601
IPI00026044	PIGU	1.088
IPI00026111	TMCO1	1.258
IPI00026154	PRKCSH	0.207
IPI00026202	RPL18A	0.550
IPI00026272	HIST1H2AE;H	-0.503
IPI00026302	RPL31	-0.436
IPI00026466	NIPBL	0.191
IPI00026530	LMAN1	-0.172
IPI00026569	HLA-A	-1.524
IPI00026781	FASN	0.218
IPI00026824	HMOX2	0.860
IPI00026942	ERLIN2	0.421
IPI00026964	UQCRFS1	1.793
IPI00026994	PRAF2;WDR4	-2.129
IPI00027078	CPD	0.005
IPI00027107	TUFM	0.899
IPI00027180	ZMPSTE24	0.592
IPI00027194	STX18	1.132

Integrated dataset hESCs/HFF		
IPI	Gene Symbol	log2 ratio
IPI00025292	MPDU1	0.938
IPI00025329	RPL19	0.431
IPI00025491	EIF4A1;SNORA	-0.361
IPI00025512	HSPB1	-2.431
IPI00025729	CAMLG	-0.229
IPI00025874	RPN1	0.410
IPI00026044	PIGU	0.544
IPI00026111	TMCO1	1.119
IPI00026202	RPL18A	0.674
IPI00026240	BST1	0.010
IPI00026272	HIST1H2AE;HIS	-0.634
IPI00026302	RPL31	-0.448
IPI00026466	NIPBL	0.112
IPI00026530	LMAN1	-0.076
IPI00026569	HLA-A	-1.631
IPI00026781	FASN	-0.972
IPI00026824	HMOX2	0.975
IPI00026942	ERLIN2	0.972
IPI00026964	UQCRFS1	2.349
IPI00026994	PRAF2;WDR45	-2.162
IPI00027078	CPD	0.136
IPI00027107	TUFM	1.281
IPI00027180	ZMPSTE24	0.631
IPI00027194	STX18	0.254
IPI00027232	IGF1R	0.893
IPI00027252	PHB2	2.026
IPI00027434	RHOC	0.660
IPI00027438	FLOT1	-0.700
IPI00027448	ATP5L	1.917
IPI00027493	LOC442497;SL(	1.856
IPI00027497	GPI	-1.853
IPI00027626	CCT6A	-0.410
IPI00027728	SLC7A1	-0.557
IPI00028055	TMED10	0.142
IPI00028116	KDELR1	-0.277
IPI00028387	DDRGK1	-1.436
IPI00028481	RAB8A	-0.391
IPI00028491	AGPAT5	2.239
IPI00028513	ADCY3	-3.060
IPI00028635	RPN2	0.394
IPI00028911	DAG1	-0.610
IPI00028946	RTN3	1.153
IPI00029046	MLEC	0.661
IPI00029133	ATP5F1	0.734
IPI00029264	CYC1	1.576
IPI00029266	SNRPE	-0.881

Integrated dataset hiPSCs/hESCs		
IPI	Gene Symbol	log2 ratio
IPI00023860	NAP1L1	0.550
IPI00023958	MPV17	-0.054
IPI00024067	CLTC	-0.008
IPI00024143	AAAS	-0.069
IPI00024145	VDAC2	-0.122
IPI00024266	MGST3	0.337
IPI00024279	HEATR1	-1.105
IPI00024282	RAB8B	-0.164
IPI00024551	C15orf24	0.338
IPI00024627	MFF	0.344
IPI00024642	CCDC47	0.069
IPI00024650	SLC16A1	-0.223
IPI00024742	UQCRQ	-0.577
IPI00024757	UQCR	0.091
IPI00024919	PRDX3	0.169
IPI00024933	RPL12	0.164
IPI00024976	TOMM22	-0.203
IPI00025049	M6PR	0.016
IPI00025086	COX5A	-0.187
IPI00025252	PDIA3	0.045
IPI00025292	MPDU1	-0.080
IPI00025329	RPL19	0.077
IPI00025491	EIF4A1;SNORA6	-0.085
IPI00025729	CAMLG	0.412
IPI00025818	GALNT1	0.034
IPI00025874	RPN1	0.136
IPI00026044	PIGU	0.522
IPI00026111	TMCO1	0.120
IPI00026202	RPL18A	-0.144
IPI00026240	BST1	-0.388
IPI00026272	HIST1H2AE;HIST	0.062
IPI00026302	RPL31	-0.091
IPI00026466	NIPBL	0.057
IPI00026530	LMAN1	0.073
IPI00026569	HLA-A	0.847
IPI00026824	HMOX2	-0.086
IPI00026964	UQCRFS1	-0.501
IPI00026994	PRAF2;WDR45	0.014
IPI00027078	CPD	-0.152
IPI00027107	TUFM	-0.390
IPI00027180	ZMPSTE24	0.078
IPI00027230	HSP90B1	-0.092
IPI00027232	IGF1R	-0.121
IPI00027233	SCO1	-0.597
IPI00027252	PHB2	-0.262
IPI00027434	RHOC	-0.150

Table S2: The integrated datasets for the comparison of hiPSCs/HFF, hESCs/HFF, and hiPSCs/hESCs.

Integrated dataset hiPSCs/HFF		
IPI	Gene Symbol	log2 ratio
IPI00027230	HSP90B1	-0.011
IPI00027232	IGF1R	0.795
IPI00027252	PHB2	1.752
IPI00027434	RHOC	0.656
IPI00027438	FLOT1	-0.671
IPI00027448	ATP5L	1.498
IPI00027493	LOC442497;SI	1.909
IPI00027497	GPI	-1.404
IPI00027728	SLC7A1	-0.157
IPI00028055	TMED10	0.173
IPI00028116	KDELR1	-0.222
IPI00028387	DDRGK1	0.164
IPI00028481	RAB8A	-0.416
IPI00028491	AGPAT5	1.159
IPI00028513	ADCY3	-2.664
IPI00028635	RPN2	0.585
IPI00028911	DAG1	-0.461
IPI00028946	RTN3	1.305
IPI00029046	MLEC	0.793
IPI00029133	ATP5F1	0.650
IPI00029264	CYC1	0.813
IPI00029266	SNRPE	-1.440
IPI00029468	ACTR1A	-2.363
IPI00029558	NDUFC2	1.728
IPI00029744	SSBP1	-1.100
IPI00029750	RPS24	-0.062
IPI00029954	VEZT	-1.045
IPI00030106	TPST1	0.785
IPI00030179	RPL7P32;RPL	0.076
IPI00030363	ACAT1	-1.963
IPI00030634	GGT7	-0.437
IPI00030820	MRPL47	-0.801
IPI00030847	TM9SF3	1.256
IPI00030906	PHF7	-0.653
IPI00031030	APLP2	1.969
IPI00031064	TMEM126A	1.045
IPI00031131	C20orf3	0.042
IPI00031169	RAB2A	-0.425
IPI00031397	ACSL3	-0.305
IPI00031458	ICMT	0.980
IPI00031461	GDI2	-2.181
IPI00031583	USO1	-2.659
IPI00031691	RPL9	-0.297
IPI00031697	TMEM109	-0.918
IPI00031821	ITM2B	1.216
IPI00032038	CPT1A	0.192

Integrated dataset hESCs/HFF		
IPI	Gene Symbol	log2 ratio
IPI00029558	NDUFC2	1.825
IPI00029741	ITGB5	0.301
IPI00029744	SSBP1	-1.220
IPI00029750	RPS24	-0.153
IPI00029954	VEZT	-0.805
IPI00030179	RPL7P32;RPL7	0.216
IPI00030431	ANTXR1	-1.819
IPI00030634	GGT7	-0.264
IPI00030820	MRPL47	-1.177
IPI00030847	TM9SF3	1.205
IPI00031064	TMEM126A	1.354
IPI00031131	C20orf3	-0.041
IPI00031169	RAB2A	-0.596
IPI00031397	ACSL3	0.328
IPI00031458	ICMT	1.038
IPI00031461	GDI2	-2.327
IPI00031583	USO1	-2.362
IPI00031691	RPL9	-0.118
IPI00031697	TMEM109	-0.976
IPI00031821	ITM2B	0.513
IPI00032038	CPT1A	0.500
IPI00032150	CDS2	1.237
IPI00032230	EPB41L3	0.274
IPI00032825	TMED7;TMED7	0.370
IPI00032903	PTRH2	1.365
IPI00033217	AASS	2.112
IPI00033349	PREB	-0.084
IPI00034277	ATP13A1	0.069
IPI00036552	ANTXR2	-0.320
IPI00042580	APOO	1.666
IPI00043564	SFXN2	0.514
IPI00043598	IKIP	-0.578
IPI00045921	ATAD3B	-0.968
IPI00059368	RFT1	-0.423
IPI00060523	TLCD1	2.952
IPI00060569	ABHD12	0.818
IPI00062469	TMEM49	1.126
IPI00063130	TMEM205	1.265
IPI00063544	YIF1B	0.121
IPI00063903	USMG5	2.457
IPI00064193	TMX3	-1.538
IPI00065486	ABCB6	0.374
IPI00065510	TMEM68	-1.260
IPI00069693	RPL14	-0.772
IPI00072917	COL6A3	-1.628
IPI00074330	TMEM48	1.688

Integrated dataset hiPSCs/hESCs		
IPI	Gene Symbol	log2 ratio
IPI00027438	FLOT1	-0.058
IPI00027448	ATP5L	-0.329
IPI00027493	LOC442497;SLC	0.048
IPI00027728	SLC7A1	0.375
IPI00028055	TMED10	0.018
IPI00028116	KDELR1	0.022
IPI00028338	SELI	0.941
IPI00028387	DDRGK1	0.925
IPI00028481	RAB8A	-0.131
IPI00028491	AGPAT5	-1.101
IPI00028513	ADCY3	0.376
IPI00028635	RPN2	0.171
IPI00028911	DAG1	0.129
IPI00028931	DSG2	-0.384
IPI00028946	RTN3	0.128
IPI00029046	MLEC	0.062
IPI00029133	ATP5F1	-0.235
IPI00029264	CYC1	-0.578
IPI00029266	SNRPE	-0.582
IPI00029558	NDUFC2	-0.118
IPI00029744	SSBP1	0.100
IPI00029750	RPS24	0.071
IPI00029954	VEZT	-0.261
IPI00030179	RPL7P32;RPL7	-0.182
IPI00030634	GGT7	-0.194
IPI00030820	MRPL47	0.356
IPI00030847	TM9SF3	-0.052
IPI00030911	VAMP8	-0.257
IPI00031064	TMEM126A	-0.330
IPI00031131	C20orf3	0.045
IPI00031169	RAB2A	0.162
IPI00031397	ACSL3	-0.671
IPI00031458	ICMT	-0.079
IPI00031461	GDI2	0.124
IPI00031522	HADHA	-0.178
IPI00031583	USO1	-0.318
IPI00031691	RPL9	-0.203
IPI00031697	TMEM109	0.037
IPI00031804	VDAC3	-0.221
IPI00031822	SLC5A6	-0.095
IPI00032038	CPT1A	-0.155
IPI00032140	SERPINH1	-0.179
IPI00032150	CDS2	0.138
IPI00032230	EPB41L3	0.008
IPI00032491	LEMD3	-0.388
IPI00032825	TMED7;TMED7-T	-0.205

Table S2: The integrated datasets for the comparison of hiPSCs/HFF, hESCs/HFF, and hiPSCs/hESCs.

Integrated dataset hiPSCs/HFF		
IPI	Gene Symbol	log2 ratio
IPI00032140	SERPINH1	-1.148
IPI00032150	CDS2	1.444
IPI00032230	EPB41L3	0.302
IPI00032825	TMED7;TMED	0.177
IPI00032903	PTRH2	0.667
IPI00033217	AASS	1.924
IPI00034277	ATP13A1	0.314
IPI00036552	ANTXR2	-0.475
IPI00037619	RPL39P5	0.456
IPI00043564	SFXN2	-0.351
IPI00043598	IKIP	-0.645
IPI00045921	ATAD3B	-1.954
IPI00045946	YME1L1	-0.455
IPI00059368	RFT1	0.260
IPI00060523	TLCD1	4.020
IPI00060569	ABHD12	1.208
IPI00062151	=-	1.898
IPI00062469	TMEM49	1.235
IPI00063130	TMEM205	1.253
IPI00063544	YIF1B	0.257
IPI00063903	USMG5	2.291
IPI00064193	TMX3	-1.661
IPI00065486	ABCB6	0.469
IPI00065510	TMEM68	-1.836
IPI00069693	RPL14	-0.154
IPI00072917	COL6A3	-2.336
IPI00074330	TMEM48	1.750
IPI00074489	NDUFB10	1.319
IPI00095891	GNAS	-2.126
IPI00099463	SGPL1	1.430
IPI00100030	PIGT	0.609
IPI00100160	CAND1	-0.999
IPI00100247	TMX4	-0.117
IPI00100656	TECR	0.990
IPI00100980	EHD2	-2.365
IPI00101651	ABHD8	0.322
IPI00101952	SLC35E1	0.052
IPI00102685	MYADM	-1.570
IPI00103530	ATL1	-3.737
IPI00103940	MFSD10	1.056
IPI00104128	SEC11A	0.738
IPI00141318	CKAP4	-0.807
IPI00146447	VMA21	0.707
IPI00149849	COG4	-0.585
IPI00151358	CLPTM1L	0.847
IPI00151710	ANO6	-2.182

Integrated dataset hESCs/HFF		
IPI	Gene Symbol	log2 ratio
IPI00074489	NDUFB10	1.403
IPI00095891	GNAS	-2.777
IPI00099463	SGPL1	1.094
IPI00100030	PIGT	0.099
IPI00100160	CAND1	-1.173
IPI00100247	TMX4	0.043
IPI00100656	TECR	0.848
IPI00100980	EHD2	-1.838
IPI00101651	ABHD8	0.624
IPI00101952	SLC35E1	0.147
IPI00102685	MYADM	-1.300
IPI00103530	ATL1	-1.618
IPI00103940	MFSD10	0.761
IPI00104128	SEC11A	0.544
IPI00107357	CLPTM1	1.097
IPI00141318	CKAP4	-0.914
IPI00146447	VMA21	0.704
IPI00149849	COG4	-0.404
IPI00151358	CLPTM1L	0.502
IPI00151710	ANO6	-1.906
IPI00152240	TMEM167A	0.071
IPI00152377	STT3B	0.777
IPI00152441	HM13	0.104
IPI00152938	TOMM40L	1.953
IPI00156689	VAT1	-2.402
IPI00157790	KIAA0368	-0.133
IPI00164018	CYP2S1	1.079
IPI00165651	ERGIC2	-0.100
IPI00166079	VKORC1L1	0.917
IPI00166444	SFT2D3	-0.241
IPI00166785	MMGT1	2.332
IPI00166865	CISD2	2.172
IPI00166891	TMEM135	0.264
IPI00168184	PPP2R1A	-1.230
IPI00168813	PTK7	-0.396
IPI00170934	STK11IP	-1.392
IPI00171411	GOLM1	1.963
IPI00171438	MUTED;TXNDC	-0.762
IPI00171445	ATAD1	1.822
IPI00171459	HSDL1	2.283
IPI00171542	NUP85	2.467
IPI00171573	CCDC109A	1.877
IPI00171626	LPCAT1	1.330
IPI00171701	ORMDL2	0.735
IPI00171705	NLGN4Y	-2.455
IPI00171903	HNRNPM	-0.945

Integrated dataset hiPSCs/hESCs		
IPI	Gene Symbol	log2 ratio
IPI00032903	PTRH2	-0.702
IPI00033217	AASS	-0.106
IPI00033349	PREB	-0.090
IPI00034277	ATP13A1	0.248
IPI00036552	ANTXR2	-0.104
IPI00042580	APOO	-0.799
IPI00043564	SFXN2	-0.886
IPI00043598	IKIP	-0.085
IPI00045921	ATAD3B	-1.004
IPI00045946	YME1L1	-0.080
IPI00056414	MAL2	-0.329
IPI00059368	RFT1	0.663
IPI00060523	TLCD1	1.045
IPI00060569	ABHD12	0.246
IPI00062206	DDX39	0.254
IPI00062469	TMEM49	0.088
IPI00063130	TMEM205	-0.071
IPI00063544	YIF1B	0.115
IPI00063903	USMG5	-0.201
IPI00064193	TMX3	-0.140
IPI00065486	ABCB6	0.073
IPI00065510	TMEM68	-0.595
IPI00069693	RPL14	0.598
IPI00072917	COL6A3	-0.699
IPI00074330	TMEM48	0.349
IPI00074489	NDUFB10	-0.029
IPI00099463	SGPL1	0.337
IPI00100030	PIGT	0.489
IPI00100160	CAND1	-0.148
IPI00100247	TMX4	-0.181
IPI00100656	TECR	0.148
IPI00100980	EHD2	-1.252
IPI00101651	ABHD8	-0.323
IPI00101952	SLC35E1	-0.115
IPI00102685	MYADM	-0.311
IPI00103530	ATL1	-2.142
IPI00103940	MFSD10	0.288
IPI00104128	SEC11A	0.161
IPI00106966	TMEM70	-1.145
IPI00107357	CLPTM1	-1.113
IPI00141318	CKAP4	0.124
IPI00146447	VMA21	-0.021
IPI00149849	COG4	-0.204
IPI00151358	CLPTM1L	0.325
IPI00151710	ANO6	-0.298
IPI00152240	TMEM167A	0.479

Table S2: The integrated datasets for the comparison of hiPSCs/HFF, hESCs/HFF, and hiPSCs/hESCs.

Integrated dataset hiPSCs/HFF		
IPI	Gene Symbol	log2 ratio
IPI00152240	TMEM167A	0.572
IPI00152377	STT3B	0.912
IPI00152441	HM13	-0.076
IPI00152938	TOMM40L	1.097
IPI00156689	VAT1	-1.700
IPI00157790	KIAA0368	-0.327
IPI00163644	OSBPL8	-0.039
IPI00164018	CYP2S1	0.799
IPI00165651	ERGIC2	-0.393
IPI00166079	VKORC1L1	0.791
IPI00166444	SFT2D3	0.597
IPI00166785	MMGT1	1.630
IPI00166860	DHRSX	0.381
IPI00166865	CISD2	1.750
IPI00166891	TMEM135	-0.190
IPI00168184	PPP2R1A	-1.179
IPI00168813	PTK7	-0.624
IPI00168921	WBSCR17	1.118
IPI00170692	VAPA	-0.235
IPI00171411	GOLM1	2.264
IPI00171438	MUTED;TXND	-2.070
IPI00171445	ATAD1	1.479
IPI00171459	HSDL1	1.622
IPI00171542	NUP85	1.406
IPI00171573	CCDC109A	1.389
IPI00171626	LPCAT1	0.946
IPI00171701	ORMDL2	0.535
IPI00171705	NLGN4Y	-3.176
IPI00172656	FAF2	0.306
IPI00174794	PTK7	-0.545
IPI00176692	-	-1.398
IPI00176824	-	0.527
IPI00177817	ATP2A2	0.260
IPI00178352	FLNC	-2.638
IPI00179964	PTBP1	0.926
IPI00180675	TUBA1A	-1.370
IPI00182469	CTNND1	0.043
IPI00182933	CYB5A	-0.183
IPI00183666	TRPV2	-0.024
IPI00183786	FADS2	1.479
IPI00184311	ENPP1	0.620
IPI00185146	IPO9	-1.057
IPI00186290	EEF2	-1.261
IPI00215637	DDX3X	0.529
IPI00215719	RPL18	0.373
IPI00215767	B4GALT1	-0.138

Integrated dataset hESCs/HFF		
IPI	Gene Symbol	log2 ratio
IPI00172656	FAF2	0.052
IPI00174794	PTK7	-0.480
IPI00176692	-	-0.884
IPI00176824	-	2.059
IPI00177817	ATP2A2	0.186
IPI00179964	PTBP1	0.854
IPI00180675	TUBA1A	-1.118
IPI00182469	CTNND1	0.281
IPI00182933	CYB5A	-0.488
IPI00183666	TRPV2	0.189
IPI00183786	FADS2	1.263
IPI00184311	ENPP1	0.115
IPI00185146	IPO9	-0.518
IPI00186290	EEF2	-1.824
IPI00215637	DDX3X	0.755
IPI00215719	RPL18	0.540
IPI00215767	B4GALT1	-1.547
IPI00215780	RPS19	0.163
IPI00215893	HMOX1	-1.873
IPI00215914	ARF1	-1.814
IPI00215948	CTNNA1	-0.948
IPI00215997	CD9	-0.025
IPI00215998	CD63	-2.086
IPI00216049	HNRNPK	1.280
IPI00216127	PLSCR3	-2.127
IPI00216237	RPL36	-0.006
IPI00216308	VDAC1	1.280
IPI00216514	CD47	-0.104
IPI00216587	RPS8	0.816
IPI00216620	PPAP2C	0.976
IPI00217007	KIAA2013	0.762
IPI00217030	RPS4X	0.134
IPI00217081	FUNDC1	-0.471
IPI00217169	ATP2B4	-2.785
IPI00217465	HIST1H1C	-0.207
IPI00217468	HIST1H1B	-1.405
IPI00217490	FNDC3B	-1.477
IPI00217519	RALA	-0.504
IPI00217563	ITGB1	-1.174
IPI00217600	PNPLA6	-0.095
IPI00217683	AKAP12	-0.883
IPI00217766	SCARB2	-1.936
IPI00217966	LDHA	-0.859
IPI00218200	BCAP31	0.296
IPI00218343	TUBA1C	0.129
IPI00218398	MMP14	-0.577

Integrated dataset hiPSCs/hESCs		
IPI	Gene Symbol	log2 ratio
IPI00152377	STT3B	0.157
IPI00152441	HM13	-0.200
IPI00152938	TOMM40L	-0.877
IPI00156689	VAT1	0.671
IPI00157790	KIAA0368	-0.214
IPI00164018	CYP2S1	0.099
IPI00165651	ERGIC2	-0.316
IPI00165665	C3orf21	0.155
IPI00166079	VKORC1L1	-0.098
IPI00166444	SFT2D3	0.816
IPI00166483	C17orf61	-0.805
IPI00166704	TOMM5	-0.387
IPI00166785	MMGT1	-0.723
IPI00166860	DHRSX	0.312
IPI00166865	CISD2	-0.023
IPI00166891	TMEM135	-0.475
IPI00167232	CASC4	0.057
IPI00168184	PPP2R1A	0.027
IPI00168813	PTK7	0.086
IPI00170692	VAPA	-0.322
IPI00171411	GOLM1	0.296
IPI00171445	ATAD1	-0.364
IPI00171459	HSDL1	-0.681
IPI00171542	NUP85	-1.082
IPI00171573	CCDC109A	-0.500
IPI00171626	LPCAT1	-0.056
IPI00171701	ORMDL2	-0.221
IPI00171705	NLGN4Y	-0.849
IPI00171903	HNRNPM	0.337
IPI00172656	FAF2	0.235
IPI00174794	PTK7	-0.088
IPI00176692	-	-0.468
IPI00177817	ATP2A2	0.021
IPI00178667	TOP2A	-0.202
IPI00178700	ALG3	0.009
IPI00179964	PTBP1	0.076
IPI00180675	TUBA1A	-0.273
IPI00182469	CTNND1	-0.236
IPI00182933	CYB5A	0.289
IPI00183666	TRPV2	-0.247
IPI00183786	FADS2	0.207
IPI00184311	ENPP1	0.481
IPI00184708	SLC25A29	-0.762
IPI00185146	IPO9	-0.560
IPI00186290	EEF2	0.029
IPI00215637	DDX3X	-0.246

Table S2: The integrated datasets for the comparison of hiPSCs/HFF, hESCs/HFF, and hiPSCs/hESCs.

Integrated dataset hiPSCs/HFF		
IPI	Gene Symbol	log2 ratio
IPI00215780	RPS19	0.686
IPI00215893	HMOX1	-1.365
IPI00215914	ARF1	-1.398
IPI00215918	ARF4	-0.587
IPI00215948	CTNNA1	-0.109
IPI00215997	CD9	-0.804
IPI00215998	CD63	-2.236
IPI00216049	HNRNPK	0.924
IPI00216127	PLSCR3	-2.592
IPI00216237	RPL36	0.107
IPI00216308	VDAC1	1.095
IPI00216514	CD47	0.098
IPI00216620	PPAP2C	0.374
IPI00217007	KIAA2013	0.943
IPI00217030	RPS4X	0.238
IPI00217081	FUNDC1	-0.099
IPI00217169	ATP2B4	-2.719
IPI00217465	HIST1H1C	-0.415
IPI00217468	HIST1H1B	-1.364
IPI00217519	RALA	-0.533
IPI00217563	ITGB1	-1.122
IPI00217600	PNPLA6	0.264
IPI00217683	AKAP12	-0.819
IPI00217766	SCARB2	-2.152
IPI00217966	LDHA	-1.412
IPI00218200	BCAP31	0.173
IPI00218343	TUBA1C	-0.038
IPI00218398	MMP14	-1.135
IPI00218463	Magmas	2.800
IPI00218466	SEC61A1	0.180
IPI00218487	GJA1	2.401
IPI00218565	GPR143	2.801
IPI00218848	ATP5I	1.596
IPI00218914	ALDH1A1	0.255
IPI00218918	ANXA1	-1.395
IPI00218922	SEC63	0.617
IPI00218924	CHP	-0.480
IPI00219018	GAPDH	-2.101
IPI00219111	TRAM1	-1.059
IPI00219155	RPL27	0.114
IPI00219217	LDHB	-0.728
IPI00219219	LGALS1	-2.735
IPI00219291	ATP5J2	1.804
IPI00219365	MSN	-1.930
IPI00219385	NDUFB6	2.024
IPI00219518	ARL1	-0.221

Integrated dataset hESCs/HFF		
IPI	Gene Symbol	log2 ratio
IPI00218463	Magmas	2.833
IPI00218466	SEC61A1	0.281
IPI00218487	GJA1	1.874
IPI00218565	GPR143	2.939
IPI00218848	ATP5I	1.588
IPI00218914	ALDH1A1	0.033
IPI00218918	ANXA1	-1.528
IPI00218922	SEC63	0.535
IPI00218924	CHP	-0.586
IPI00219018	GAPDH	-1.716
IPI00219111	TRAM1	-1.013
IPI00219155	RPL27	0.027
IPI00219217	LDHB	-0.523
IPI00219219	LGALS1	-2.835
IPI00219291	ATP5J2	2.089
IPI00219365	MSN	-1.497
IPI00219385	NDUFB6	2.223
IPI00219682	STOM	-2.151
IPI00219685	NDUFA13;YJEF	2.136
IPI00219729	SLC25A11	1.537
IPI00220194	SLC2A1	2.379
IPI00220281	GNAO1	1.738
IPI00220327	KRT1	0.291
IPI00220487	ATP5H	2.603
IPI00220556	PBXIP1	0.769
IPI00220578	GNAI3	0.450
IPI00220739	PGRMC1	0.629
IPI00220740	NPM1	-0.475
IPI00220835	SEC61B	-0.103
IPI00220993	CNP	-1.681
IPI00221088	RPS9	-0.141
IPI00221089	RPS13	-0.680
IPI00221091	RPS15A	0.216
IPI00221092	RPS16	-0.179
IPI00221224	ANPEP	-3.354
IPI00221232	GNG12	-3.657
IPI00221240	LNPEP	-2.771
IPI00221354	FUS	-0.125
IPI00232571	GPC4	1.971
IPI00242630	HEATR2	-2.272
IPI00246058	PDCD6IP	-2.360
IPI00247063	MME	-3.896
IPI00247583	RPL21P19;RPL	0.103
IPI00248911	GNAT3	1.207
IPI00257903	ERMP1	0.617
IPI00288947	GNAQ	-1.048

Integrated dataset hiPSCs/hESCs		
IPI	Gene Symbol	log2 ratio
IPI00215719	RPL18	-0.091
IPI00215767	B4GALT1	1.387
IPI00215780	RPS19	0.462
IPI00215893	HMOX1	0.262
IPI00215914	ARF1	0.305
IPI00215918	ARF4	-0.451
IPI00215948	CTNNA1	0.875
IPI00215997	CD9	-0.799
IPI00215998	CD63	-0.225
IPI00216049	HNRNPK	-0.331
IPI00216127	PLSCR3	-0.389
IPI00216237	RPL36	0.152
IPI00216308	VDAC1	-0.201
IPI00216514	CD47	0.058
IPI00216587	RPS8	-0.087
IPI00216592	HNRNPC	-0.266
IPI00216620	PPAP2C	-0.623
IPI00217007	KIAA2013	0.135
IPI00217030	RPS4X	-0.067
IPI00217081	FUNDC1	0.353
IPI00217143	SDHA	0.047
IPI00217169	ATP2B4	-0.096
IPI00217465	HIST1H1C	-0.230
IPI00217468	HIST1H1B	0.019
IPI00217519	RALA	-0.121
IPI00217536	RHOT1	0.505
IPI00217557	NRM	-0.023
IPI00217563	ITGB1	0.002
IPI00217600	PNPLA6	0.387
IPI00217683	AKAP12	0.042
IPI00217766	SCARB2	-0.115
IPI00217966	LDHA	-0.469
IPI00217975	LMNB1	0.221
IPI00218200	BCAP31	-0.016
IPI00218343	TUBA1C	-0.182
IPI00218398	MMP14	-0.650
IPI00218463	Magmas	-0.053
IPI00218466	SEC61A1	-0.099
IPI00218487	GJA1	0.583
IPI00218565	GPR143	-0.339
IPI00218848	ATP5I	-0.008
IPI00218914	ALDH1A1	0.121
IPI00218918	ANXA1	0.009
IPI00218922	SEC63	0.003
IPI00218924	CHP	0.009
IPI00219018	GAPDH	-0.402

Table S2: The integrated datasets for the comparison of hiPSCs/HFF, hESCs/HFF, and hiPSCs/hESCs.

Integrated dataset hiPSCs/HFF		
IPI	Gene Symbol	log2 ratio
IPI00219682	STOM	-2.146
IPI00219685	NDUFA13;YJE	1.851
IPI00219729	SLC25A11	1.188
IPI00220194	SLC2A1	2.079
IPI00220281	GNAO1	1.600
IPI00220327	KRT1	0.154
IPI00220402	PPP1R2	-0.687
IPI00220473	ATP2C1	0.397
IPI00220487	ATP5H	2.195
IPI00220556	PBXIP1	-0.026
IPI00220578	GNAI3	0.252
IPI00220739	PGRMC1	0.591
IPI00220740	NPM1	-0.273
IPI00220835	SEC61B	-0.125
IPI00220993	CNP	-1.459
IPI00221088	RPS9	0.030
IPI00221089	RPS13	-0.488
IPI00221091	RPS15A	0.283
IPI00221092	RPS16	-0.046
IPI00221093	RPS17	-0.383
IPI00221224	ANPEP	-3.147
IPI00221232	GNG12	-3.311
IPI00221240	LNPEP	-2.011
IPI00221354	FUS	-0.999
IPI00232571	GPC4	1.075
IPI00242630	HEATR2	-2.374
IPI00246058	PDCD6IP	-0.677
IPI00247063	MME	-3.616
IPI00247583	RPL21P19;RP	0.289
IPI00248911	GNAT3	1.563
IPI00257903	ERMP1	0.722
IPI00288947	GNAQ	-1.082
IPI00289819	IGF2R	0.329
IPI00289876	STX7	-1.653
IPI00289983	ACPP	0.740
IPI00290085	CDH2	-0.482
IPI00290799	C18orf19	0.366
IPI00290928	GNA13	-1.086
IPI00290945	PON2	0.011
IPI00291006	MDH2	0.821
IPI00291175	VCL	-1.647
IPI00291467	SLC25A6	1.192
IPI00291695	GPX8	-0.430
IPI00291755	NUP210	1.591
IPI00291928	RAB14	-1.225
IPI00292135	LBR	1.643

Integrated dataset hESCs/HFF		
IPI	Gene Symbol	log2 ratio
IPI00289819	IGF2R	0.010
IPI00289876	STX7	-1.957
IPI00289983	ACPP	0.799
IPI00290085	CDH2	-0.378
IPI00290799	C18orf19	1.377
IPI00290928	GNA13	-0.689
IPI00290945	PON2	-0.185
IPI00291006	MDH2	1.080
IPI00291175	VCL	-2.371
IPI00291417	DCAKD	0.664
IPI00291467	SLC25A6	1.423
IPI00291695	GPX8	-0.175
IPI00291755	NUP210	1.006
IPI00292135	LBR	1.924
IPI00293476	MRPL23	1.908
IPI00294159	SLC25A1	0.945
IPI00294250	EPHA1	1.149
IPI00294472	TMED5	-0.219
IPI00294501	DHCR7	2.456
IPI00294755	CLDN7	-1.028
IPI00294834	ASPH	-2.823
IPI00295098	SRPRB	-0.165
IPI00295461	FAP	-1.242
IPI00295772	CYP51A1;LOC4	0.796
IPI00295851	COPB1	-1.168
IPI00295992	ATAD3A	1.518
IPI00296099	THBS1	-0.595
IPI00296147	TMEM119	-1.310
IPI00296157	RETSAT	-1.201
IPI00296190	C10orf58	2.959
IPI00296215	EPCAM	1.058
IPI00296259	TMED4	0.442
IPI00297037	PPAP2A	-0.378
IPI00297084	DDOST	0.539
IPI00297492	STT3A	0.513
IPI00297646	COL1A1	-1.809
IPI00298237	TPP1	1.833
IPI00298289	RTN4	-0.880
IPI00298851	CD151	-1.610
IPI00298947	MEST	1.165
IPI00298994	TLN1	-3.740
IPI00299084	TMEM33	1.102
IPI00299116	PODXL	2.947
IPI00299468	SCD	-0.508
IPI00299573	RPL7A	0.162
IPI00299719	TCIRG1	-0.196

Integrated dataset hiPSCs/hESCs		
IPI	Gene Symbol	log2 ratio
IPI00219111	TRAM1	-0.065
IPI00219155	RPL27	0.066
IPI00219217	LDHB	-0.226
IPI00219219	LGALS1	0.080
IPI00219291	ATP5J2	-0.283
IPI00219330	ILF3	-0.602
IPI00219365	MSN	-0.457
IPI00219385	NDUFB6	-0.221
IPI00219682	STOM	-0.153
IPI00219685	NDUFA13;YJEFN	-0.309
IPI00219729	SLC25A11	-0.385
IPI00220059	NDUFB4	-0.227
IPI00220194	SLC2A1	-0.275
IPI00220281	GNAO1	-0.159
IPI00220327	KRT1	-0.216
IPI00220473	ATP2C1	0.200
IPI00220487	ATP5H	-0.361
IPI00220556	PBXIP1	-0.669
IPI00220578	GNAI3	-0.215
IPI00220739	PGRMC1	-0.078
IPI00220740	NPM1	0.238
IPI00220835	SEC61B	0.022
IPI00220993	CNP	0.203
IPI00221088	RPS9	0.010
IPI00221089	RPS13	0.170
IPI00221091	RPS15A	0.024
IPI00221092	RPS16	0.112
IPI00221093	RPS17	-0.209
IPI00221224	ANPEP	-0.012
IPI00221232	GNG12	0.188
IPI00221354	FUS	-0.890
IPI00232571	GPC4	-0.643
IPI00242630	HEATR2	-0.124
IPI00247063	MME	0.019
IPI00247583	RPL21P19;RPL2	0.123
IPI00248911	GNAT3	0.335
IPI00257903	ERMP1	0.085
IPI00288947	GNAQ	-0.056
IPI00289819	IGF2R	-0.027
IPI00289876	STX7	0.321
IPI00289983	ACPP	-0.079
IPI00290085	CDH2	0.046
IPI00290544	GDAP1	0.158
IPI00290799	C18orf19	-1.033
IPI00290928	GNA13	-0.418
IPI00290945	PON2	0.169

Table S2: The integrated datasets for the comparison of hiPSCs/HFF, hESCs/HFF, and hiPSCs/hESCs.

Integrated dataset hiPSCs/HFF		
IPI	Gene Symbol	log2 ratio
IPI00293476	MRPL23	2.021
IPI00294159	SLC25A1	0.761
IPI00294242	MRPS31	-0.733
IPI00294250	EPHA1	1.175
IPI00294472	TMED5	-0.160
IPI00294501	DHCR7	2.794
IPI00294755	CLDN7	-0.413
IPI00294834	ASPH	-2.222
IPI00295098	SRPRB	-0.109
IPI00295461	FAP	-2.646
IPI00295698	SLC7A3	2.521
IPI00295772	CYP51A1;LOC	1.097
IPI00295851	COPB1	-1.141
IPI00295992	ATAD3A	2.039
IPI00296157	RETSAT	-0.847
IPI00296190	C10orf58	3.383
IPI00296215	EPCAM	0.314
IPI00296259	TMED4	0.097
IPI00297037	PPAP2A	-0.487
IPI00297084	DDOST	0.715
IPI00297160	CD44	-3.571
IPI00297492	STT3A	0.578
IPI00297646	COL1A1	-1.833
IPI00298237	TPP1	1.937
IPI00298289	RTN4	-0.560
IPI00298851	CD151	-1.355
IPI00298947	MEST	1.399
IPI00298994	TLN1	-2.638
IPI00299084	TMEM33	1.167
IPI00299116	PODXL	2.796
IPI00299468	SCD	-0.064
IPI00299573	RPL7A	0.099
IPI00300127	NAT10	2.133
IPI00300299	SPCS3	0.910
IPI00300384	ERBB2	2.107
IPI00300562	RAB3B	-1.761
IPI00300744	TMEM14B	-1.326
IPI00300971	ALG6	1.567
IPI00301021	SSR1	0.558
IPI00301202	MAGT1	0.324
IPI00301280	TMEM43	-1.162
IPI00301494	CHST4	0.532
IPI00301554	MFSD5	-0.028
IPI00301841	TMEM161A	0.630
IPI00302592	FLNA	-2.019
IPI00302850	SNRPD1	2.263

Integrated dataset hESCs/HFF		
IPI	Gene Symbol	log2 ratio
IPI00300127	NAT10	1.240
IPI00300299	SPCS3	0.849
IPI00300384	ERBB2	2.280
IPI00300562	RAB3B	-2.395
IPI00300744	TMEM14B	-1.185
IPI00300971	ALG6	1.644
IPI00301021	SSR1	0.348
IPI00301202	MAGT1	0.549
IPI00301280	TMEM43	-1.269
IPI00301494	CHST4	0.709
IPI00301554	MFSD5	-0.282
IPI00301841	TMEM161A	0.707
IPI00302592	FLNA	-2.532
IPI00302850	SNRPD1	2.562
IPI00302927	CCT4;ILK-2	-0.331
IPI00303283	ITGB3	-0.575
IPI00303726	IFITM3	-0.479
IPI00303954	CYB5B	0.866
IPI00304331	B3GAT3	-0.308
IPI00304612	RPL13A	0.469
IPI00304962	COL1A2	-0.741
IPI00305304	LASS2	0.552
IPI00305383	UQCRC2	1.600
IPI00305551	GNA11	-1.609
IPI00305698	GGCX	1.071
IPI00306332	RPL24	-0.066
IPI00306516	TIMM44	0.432
IPI00306604	ITGA5	-2.490
IPI00306748	ABCB7	0.162
IPI00307572	TMEM165	1.108
IPI00328112	TAP2	-0.162
IPI00328161	FKBP8	-0.467
IPI00328170	MOGS	0.348
IPI00328180	RAB34	-1.666
IPI00328243	PLD3	-0.187
IPI00328391	GALNT7	0.393
IPI00328715	MTDH	-0.944
IPI00329331	UGP2	0.586
IPI00329332	STX12	-2.096
IPI00329352	NOMO3;NOMO	0.674
IPI00329389	RPL6	-0.324
IPI00329410	DOLPP1	2.026
IPI00329596	TMX2	0.797
IPI00329598	HSD17B11	-3.615
IPI00329600	SCCPDH	-0.853
IPI00329801	ANXA5	-1.001

Integrated dataset hiPSCs/hESCs		
IPI	Gene Symbol	log2 ratio
IPI00291175	VCL	0.647
IPI00291417	DCAKD	-0.511
IPI00291467	SLC25A6	-0.200
IPI00291695	GPX8	-0.276
IPI00291755	NUP210	-0.238
IPI00292135	LBR	-0.305
IPI00293476	MRPL23	0.093
IPI00294159	SLC25A1	-0.206
IPI00294250	EPHA1	0.005
IPI00294455	UGT8	0.422
IPI00294472	TMED5	0.059
IPI00294501	DHCR7	0.310
IPI00294755	CLDN7	0.158
IPI00294834	ASPH	-0.037
IPI00295098	SRPRB	0.164
IPI00295598	CBARA1	-0.153
IPI00295698	SLC7A3	0.073
IPI00295772	CYP51A1;LOC40	0.241
IPI00295851	COPB1	0.005
IPI00295992	ATAD3A	-0.360
IPI00296099	THBS1	0.243
IPI00296157	RETSAT	0.331
IPI00296190	C10orf58	0.380
IPI00296215	EPCAM	-0.765
IPI00296259	TMED4	-0.362
IPI00297037	PPAP2A	-0.129
IPI00297084	DDOST	0.107
IPI00297492	STT3A	0.050
IPI00298289	RTN4	0.191
IPI00298851	CD151	0.039
IPI00298947	MEST	0.133
IPI00299084	TMEM33	-0.054
IPI00299116	PODXL	-0.276
IPI00299468	SCD	0.319
IPI00299571	PDIA6	0.113
IPI00299573	RPL7A	-0.086
IPI00299719	TCIRG1	0.002
IPI00300127	NAT10	0.871
IPI00300299	SPCS3	0.074
IPI00300384	ERBB2	-0.116
IPI00300744	TMEM14B	-0.163
IPI00300971	ALG6	0.099
IPI00301021	SSR1	0.219
IPI00301202	MAGT1	-0.247
IPI00301280	TMEM43	0.106
IPI00301323	DDX18	0.671

Table S2: The integrated datasets for the comparison of hiPSCs/HFF, hESCs/HFF, and hiPSCs/hESCs.

Integrated dataset hiPSCs/HFF		
IPI	Gene Symbol	log2 ratio
IPI00302927	CCT4;ILK-2	-0.359
IPI00303283	ITGB3	-0.055
IPI00303726	IFITM3	-0.876
IPI00303954	CYB5B	0.886
IPI00304331	B3GAT3	-0.304
IPI00304612	RPL13A	0.353
IPI00305304	LASS2	0.489
IPI00305383	UQCRC2	1.371
IPI00305551	GNA11	-1.758
IPI00305698	GGCX	1.317
IPI00306048	ATAD3B	0.885
IPI00306332	RPL24	-0.012
IPI00306419	LPCAT3	0.957
IPI00306516	TIMM44	0.325
IPI00306604	ITGA5	-2.054
IPI00306748	ABCB7	0.494
IPI00307572	TMEM165	1.332
IPI00328112	TAP2	-0.275
IPI00328161	FKBP8	-0.129
IPI00328170	MOGS	-0.066
IPI00328180	RAB34	-0.860
IPI00328243	PLD3	0.318
IPI00328257	AP1B1	-1.683
IPI00328391	GALNT7	0.854
IPI00329301	NDUFA11	1.030
IPI00329331	UGP2	0.702
IPI00329332	STX12	-1.623
IPI00329352	NOMO3;NOM(	0.782
IPI00329389	RPL6	-0.120
IPI00329410	DOLPP1	2.418
IPI00329596	TMX2	1.829
IPI00329598	HSD17B11	-3.436
IPI00329801	ANXA5	-1.524
IPI00332106	PBXIP1	-0.281
IPI00333016	DNAJC11	-0.878
IPI00333619	ALDH3A2	0.634
IPI00334190	STOML2	1.190
IPI00334282	FAM3C	0.725
IPI00335168	MYL6B;MYL6	-2.775
IPI00337415	GNAI1	-1.570
IPI00337494	SLC25A24	0.411
IPI00339384	RDH11	1.163
IPI00374076	FITM2	-1.637
IPI00374975	PGAM4	2.035
IPI00375676	FTL	-0.571
IPI00376798	RPL11	-0.094

Integrated dataset hESCs/HFF		
IPI	Gene Symbol	log2 ratio
IPI00332106	PBXIP1	-0.410
IPI00333016	DNAJC11	-0.061
IPI00333619	ALDH3A2	0.508
IPI00334190	STOML2	1.267
IPI00334282	FAM3C	0.679
IPI00335168	MYL6B;MYL6	-2.748
IPI00337415	GNAI1	-1.571
IPI00337494	SLC25A24	0.393
IPI00339384	RDH11	1.005
IPI00374076	FITM2	-0.162
IPI00374975	PGAM4	0.189
IPI00376394	QSOX2	-0.608
IPI00376798	RPL11	0.103
IPI00377145	KCNN2	0.813
IPI00383730	CACNA2D1	-0.913
IPI00384016	DLSTP;DLST	-0.574
IPI00384280	PCYOX1	-1.582
IPI00385267	SRPR	-0.350
IPI00385495	LMF2	-1.685
IPI00386139	FAR1	-2.351
IPI00386199	C19orf6	-0.797
IPI00386258	MTCH1	1.075
IPI00387077	SLC12A9	-0.997
IPI00394699	RPL17	-0.195
IPI00395488	VASN	-1.023
IPI00395769	ATP5C1	1.107
IPI00395887	TMX1	1.035
IPI00395894	SYS1	0.491
IPI00395903	TMEM106B	-0.840
IPI00395998	RPL32;SNORA	0.405
IPI00396321	LRRC59	-0.116
IPI00396378	HNRNPA2B1	0.106
IPI00396485	EEF1A1	-1.012
IPI00396661	CYP2S1	2.053
IPI00397526	MYH10	-0.077
IPI00401819	RPS26	0.355
IPI00409635	ESYT2	-0.822
IPI00410034	SLC38A2	-0.871
IPI00410488	CD276	-1.139
IPI00411639	RPSA;RPSAP1	0.653
IPI00411937	NOP56	2.146
IPI00412107	ANKRD26P1	0.328
IPI00412592	FAM108B1	-2.495
IPI00413784	SEPN1	2.485
IPI00414676	HSP90AB1	0.690
IPI00414717	GLG1	1.480

Integrated dataset hiPSCs/hESCs		
IPI	Gene Symbol	log2 ratio
IPI00301494	CHST4	-0.197
IPI00301554	MFSD5	0.234
IPI00301841	TMEM161A	0.094
IPI00302592	FLNA	0.096
IPI00302850	SNRPD1	-0.321
IPI00302927	CCT4;ILK-2	-0.159
IPI00303283	ITGB3	0.500
IPI00303726	IFITM3	-0.394
IPI00303954	CYB5B	0.040
IPI00304331	B3GAT3	-0.017
IPI00304612	RPL13A	-0.138
IPI00304962	COL1A2	0.339
IPI00305258	MOSC1	-0.238
IPI00305304	LASS2	-0.052
IPI00305383	UQCRC2	-0.149
IPI00305551	GNA11	-0.142
IPI00305698	GGCX	0.224
IPI00306332	RPL24	-0.030
IPI00306516	TIMM44	-0.209
IPI00306748	ABCB7	0.265
IPI00307572	TMEM165	0.175
IPI00328112	TAP2	-0.121
IPI00328161	FKBP8	0.273
IPI00328170	MOGS	0.021
IPI00328180	RAB34	0.606
IPI00328243	PLD3	0.210
IPI00328257	AP1B1	-0.404
IPI00328391	GALNT7	0.307
IPI00328715	MTDH	-0.062
IPI00329331	UGP2	0.119
IPI00329332	STX12	0.504
IPI00329352	NOMO3;NOMO1	0.092
IPI00329389	RPL6	0.064
IPI00329410	DOLPP1	0.371
IPI00329596	TMX2	0.579
IPI00329598	HSD17B11	0.161
IPI00329801	ANXA5	-0.505
IPI00332106	PBXIP1	0.424
IPI00333016	DNAJC11	-0.487
IPI00333619	ALDH3A2	0.141
IPI00334190	STOML2	-0.058
IPI00334282	FAM3C	0.015
IPI00335168	MYL6B;MYL6	-0.049
IPI00337415	GNAI1	-0.021
IPI00337494	SLC25A24	-0.472
IPI00339384	RDH11	0.079

Table S2: The integrated datasets for the comparison of hiPSCs/HFF, hESCs/HFF, and hiPSCs/hESCs.

Integrated dataset hiPSCs/HFF		
IPI	Gene Symbol	log2 ratio
IPI00377145	KCNN2	0.690
IPI00383730	CACNA2D1	-0.267
IPI00384016	DLSTP;DLST	-0.622
IPI00384280	PCYOX1	-1.744
IPI00385267	SRPR	-0.164
IPI00385495	LMF2	-1.216
IPI00386139	FAR1	-1.913
IPI00386199	C19orf6	-0.843
IPI00386258	MTCH1	0.923
IPI00387077	SLC12A9	-0.715
IPI00394699	RPL17	-0.323
IPI00395488	VASN	-1.618
IPI00395769	ATP5C1	0.547
IPI00395887	TMX1	1.069
IPI00395894	SYS1	0.332
IPI00395903	TMEM106B	-0.413
IPI00395998	RPL32;SNOR/	0.371
IPI00396321	LRRC59	0.190
IPI00396378	HNRNPA2B1	-0.409
IPI00396485	EEF1A1	-0.849
IPI00396661	CYP2S1	2.089
IPI00397526	MYH10	0.263
IPI00397645	MXRA7	-2.114
IPI00401819	RPS26	0.719
IPI00409635	ESYT2	-0.700
IPI00410034	SLC38A2	-0.444
IPI00410488	CD276	-1.214
IPI00411639	RPSA;RPSAP	0.236
IPI00411937	NOP56	0.499
IPI00412107	ANKRD26P1	-0.710
IPI00412592	FAM108B1	-2.614
IPI00412607	RPL35	-0.619
IPI00413784	SEPN1	1.136
IPI00414676	HSP90AB1	0.496
IPI00414717	GLG1	1.563
IPI00418169	ANXA2	-1.872
IPI00418471	VIM	-2.210
IPI00418497	TIMM50	1.369
IPI00419585	PPIA	-1.604
IPI00419869	COX15	1.656
IPI00419880	RPS3A	0.041
IPI00420108	DLSTP;DLST	-0.693
IPI00438289	ERBB2IP	-2.712
IPI00440493	ATP5A1	-0.597
IPI00444262	NCL	-0.521
IPI00446235	CYB5R3	-1.603

Integrated dataset hESCs/HFF		
IPI	Gene Symbol	log2 ratio
IPI00418169	ANXA2	-1.844
IPI00418471	VIM	-2.450
IPI00418497	TIMM50	1.748
IPI00419585	PPIA	-1.482
IPI00419869	COX15	1.578
IPI00419880	RPS3A	-0.193
IPI00420108	DLSTP;DLST	-1.039
IPI00440493	ATP5A1	-0.308
IPI00444262	NCL	-0.261
IPI00446235	CYB5R3	-1.569
IPI00452747	LOC653566	0.836
IPI00453473	HIST1H4K;HIST	-0.591
IPI00455165	PANX1	-1.714
IPI00455457	=-	-0.519
IPI00455473	MIA3	0.355
IPI00456429	UBA52	-0.653
IPI00456758	RPL27A	0.505
IPI00456969	DYNC1H1	-1.749
IPI00464968	MT-CO1	1.259
IPI00465034	SLC25A26	0.675
IPI00465059	RHOT2	2.290
IPI00465248	ENO1	-1.050
IPI00465308	PIGS	0.656
IPI00465361	RPL13	0.328
IPI00465431	LGALS3	-1.429
IPI00465439	ALDOA	-1.900
IPI00470360	KIRREL	0.240
IPI00470467	POR	0.442
IPI00470528	RPL15	0.678
IPI00470649	NCLN	-1.109
IPI00470674	CYB5R1	-1.548
IPI00471978	C2CD2	0.021
IPI00472810	UBE3C	-0.070
IPI00473047	PRKAG1	0.914
IPI00478003	A2M	-2.996
IPI00478231	RHOA	-1.265
IPI00478838	FAR1	0.590
IPI00479058	RPS15	-1.004
IPI00479145	KRT19	-0.170
IPI00479186	PKM2	-2.047
IPI00479217	HNRNPU	3.027
IPI00514023	PTRF	-4.712
IPI00549343	VAMP3	-2.007
IPI00549761	ALG1	0.144
IPI00549970	GHITM	1.861
IPI00550021	RPL3	0.281

Integrated dataset hiPSCs/hESCs		
IPI	Gene Symbol	log2 ratio
IPI00374076	FITM2	-1.493
IPI00374975	PGAM4	0.352
IPI00376394	QSOX2	0.401
IPI00376686	AMFR	0.129
IPI00376798	RPL11	-0.214
IPI00377017	PKMYT1	-0.051
IPI00377145	KCNN2	-0.144
IPI00383730	CACNA2D1	0.623
IPI00383798	PRODH	-0.029
IPI00384016	DLSTP;DLST	-0.071
IPI00384280	PCYOX1	-0.241
IPI00384867	SLC35B2	0.339
IPI00385267	SRPR	0.177
IPI00385495	LMF2	0.147
IPI00386139	FAR1	0.415
IPI00386199	C19orf6	-0.068
IPI00386258	MTCH1	-0.106
IPI00386271	SLC25A12	0.267
IPI00386427	SMPDL3B	-0.439
IPI00387077	SLC12A9	-0.178
IPI00395488	VASN	-0.618
IPI00395769	ATP5C1	-0.531
IPI00395887	TMX1	-0.013
IPI00395894	SYS1	-0.181
IPI00395903	TMEM106B	0.405
IPI00395998	RPL32;SNORA7/	-0.056
IPI00396321	LRRC59	0.118
IPI00396378	HNRNPA2B1	-0.685
IPI00396435	DHX15	-0.745
IPI00396485	EEF1A1	0.108
IPI00396661	CYP2S1	0.101
IPI00397526	MYH10	0.053
IPI00409635	ESYT2	0.102
IPI00410034	SLC38A2	0.393
IPI00410079	FAM82A2	0.777
IPI00410488	CD276	0.045
IPI00411639	RPSA;RPSAP15	-0.424
IPI00411937	NOP56	-1.668
IPI00412107	ANKRD26P1	-1.061
IPI00412592	FAM108B1	-0.145
IPI00412713	SAMM50	-0.207
IPI00413784	SEPN1	-1.069
IPI00414320	ANXA11	0.091
IPI00414676	HSP90AB1	-0.147
IPI00414717	GLG1	0.094
IPI00418169	ANXA2	-0.083

Table S2: The integrated datasets for the comparison of hiPSCs/HFF, hESCs/HFF, and hiPSCs/hESCs.

Integrated dataset hiPSCs/HFF		
IPI	Gene Symbol	log2 ratio
IPI00452747	LOC653566	0.697
IPI00453473	HIST1H4K;HIS	-0.520
IPI00455165	PANX1	-1.440
IPI00455457	=-	0.728
IPI00455473	MIA3	0.255
IPI00456429	UBA52	-0.660
IPI00456758	RPL27A	0.498
IPI00456969	DYNC1H1	-1.049
IPI00464968	MT-CO1	1.630
IPI00465034	SLC25A26	0.772
IPI00465059	RHOT2	2.228
IPI00465113	EXD2	-1.488
IPI00465248	ENO1	-1.106
IPI00465308	PIGS	0.560
IPI00465361	RPL13	0.531
IPI00465431	LGALS3	-1.775
IPI00465439	ALDOA	-1.976
IPI00470360	KIRREL	2.228
IPI00470467	POR	0.637
IPI00470468	EFR3A	-2.501
IPI00470528	RPL15	0.784
IPI00470649	NCLN	-0.151
IPI00470674	CYB5R1	-1.693
IPI00472810	UBE3C	0.435
IPI00473047	PRKAG1	1.847
IPI00478003	A2M	-2.418
IPI00478231	RHOA	-1.010
IPI00478838	FAR1	0.736
IPI00479058	RPS15	0.250
IPI00479145	KRT19	1.101
IPI00479186	PKM2	-2.093
IPI00479217	HNRNPU	3.126
IPI00549343	VAMP3	-1.589
IPI00549761	ALG1	0.275
IPI00549970	GHITM	1.499
IPI00550021	RPL3	0.178
IPI00550165	DHRS7B	0.350
IPI00550382	SLC29A1	2.025
IPI00550523	ATL3	-1.092
IPI00554541	ILVBL	0.438
IPI00554648	KRT8	-0.090
IPI00554701	UCRC	2.042
IPI00554711	JUP	0.742
IPI00554723	RPL10	0.247
IPI00554742	API5	-2.152
IPI00555610	AHNAK	-0.226

Integrated dataset hESCs/HFF		
IPI	Gene Symbol	log2 ratio
IPI00550165	DHRS7B	-0.349
IPI00550382	SLC29A1	2.211
IPI00554541	ILVBL	0.305
IPI00554648	KRT8	-0.649
IPI00554701	UCRC	2.140
IPI00554723	RPL10	0.126
IPI00554742	API5	-1.396
IPI00555610	AHNAK	-0.453
IPI00555744	RPL14	0.218
IPI00555902	OCIAD2	3.255
IPI00641435	LASS2	0.928
IPI00641829	BAT1	0.723
IPI00641924	MRPS9	0.042
IPI00642244	KIAA0090	0.919
IPI00642329	MFN2	1.299
IPI00643041	RANP1;RAN	-0.578
IPI00644020	SOAT1	-0.991
IPI00644766	TOR1AIP1	1.538
IPI00645518	CDIPT	-0.347
IPI00645667	MBOAT7	-0.399
IPI00646304	PPIB	-0.768
IPI00646625	TAP1	-0.462
IPI00646779	TUBB6	-0.186
IPI00646963	REEP6	1.339
IPI00647457	HLA-A	-0.342
IPI00654777	EIF3F	-0.634
IPI00654820	MT-ATP6	1.437
IPI00657706	MBOAT7	-0.855
IPI00739274	LOC652614	1.118
IPI00743576	ATP6V0A1	-0.327
IPI00747849	ATP1B1	1.862
IPI00748145	GNAI2	-0.547
IPI00760554	HLA-A	-2.378
IPI00783097	GARS	-1.401
IPI00783271	LRPPRC	0.382
IPI00783698	TMEM87A	-0.042
IPI00784119	ATP6AP1	0.542
IPI00784154	HSPD1	1.335
IPI00784376	MTP18	0.034
IPI00787853	IMPAD1	-0.769
IPI00788624	SLC25A3	1.594
IPI00789008	FLOT2	-0.539
IPI00790135	OSBPL8	-0.146
IPI00793199	ANXA4	-2.664
IPI00793443	IPO5	-0.071
IPI00794169	ANO10	-0.632

Integrated dataset hiPSCs/hESCs		
IPI	Gene Symbol	log2 ratio
IPI00418471	VIM	-0.028
IPI00418497	TIMM50	-0.398
IPI00419585	PPIA	-0.201
IPI00419869	COX15	0.058
IPI00419880	RPS3A	0.043
IPI00419916	ALPL	-0.001
IPI00420014	SNRNP200	-0.745
IPI00420108	DLSTP;DLST	0.309
IPI00440493	ATP5A1	-0.307
IPI00444262	NCL	-0.283
IPI00446235	CYB5R3	-0.050
IPI00452747	LOC653566	-0.267
IPI00453473	HIST1H4K;HIST1	0.066
IPI00455165	PANX1	0.252
IPI00455457	=-	-0.563
IPI00455473	MIA3	-0.170
IPI00456429	UBA52	-0.026
IPI00456758	RPL27A	-0.053
IPI00456969	DYNC1H1	0.678
IPI00464968	MT-CO1	0.350
IPI00465034	SLC25A26	-0.005
IPI00465059	RHOT2	-0.127
IPI00465248	ENO1	-0.068
IPI00465308	PIGS	-0.100
IPI00465361	RPL13	0.192
IPI00465431	LGALS3	-0.496
IPI00465439	ALDOA	-0.096
IPI00470467	POR	0.225
IPI00470528	RPL15	0.019
IPI00470649	NCLN	0.150
IPI00470674	CYB5R1	-0.164
IPI00470924	TMTC3	0.260
IPI00472810	UBE3C	0.483
IPI00473047	PRKAG1	0.913
IPI00478003	A2M	0.576
IPI00478231	RHOA	0.233
IPI00478838	FAR1	0.442
IPI00479186	PKM2	-0.353
IPI00479217	HNRNPU	-0.370
IPI00479357	BSCL2	0.620
IPI00549343	VAMP3	0.349
IPI00549761	ALG1	0.129
IPI00549970	GHITM	-0.408
IPI00550021	RPL3	-0.124
IPI00550382	SLC29A1	-0.218
IPI00550523	ATL3	-0.085

Table S2: The integrated datasets for the comparison of hiPSCs/HFF, hESCs/HFF, and hiPSCs/hESCs.

Integrated dataset hiPSCs/HFF		
IPI	Gene Symbol	log2 ratio
IPI00555744	RPL14	0.285
IPI00555902	OCIAD2	3.308
IPI00641435	LASS2	1.096
IPI00641829	BAT1	0.362
IPI00641924	MRPS9	-0.035
IPI00642244	KIAA0090	0.931
IPI00642329	MFN2	1.122
IPI00643041	RANP1;RAN	-0.213
IPI00644020	SOAT1	-1.545
IPI00644766	TOR1AIP1	1.674
IPI00645518	CDIPT	-0.397
IPI00645667	MBOAT7	-0.080
IPI00646304	PPIB	-0.758
IPI00646625	TAP1	-0.723
IPI00646779	TUBB6	0.347
IPI00646963	REEP6	1.644
IPI00647457	HLA-A	-0.469
IPI00654777	EIF3F	-1.481
IPI00654820	MT-ATP6	1.100
IPI00657706	MBOAT7	-0.477
IPI00739274	LOC652614	1.097
IPI00743576	ATP6V0A1	0.180
IPI00747849	ATP1B1	1.884
IPI00748145	GNAI2	-0.683
IPI00760554	HLA-A	-1.580
IPI00783097	GARS	-1.149
IPI00783271	LRPPRC	-0.410
IPI00783698	TMEM87A	0.228
IPI00784154	HSPD1	0.994
IPI00787853	IMPAD1	0.042
IPI00788624	SLC25A3	1.658
IPI00788884	LOC643790;HI	-0.062
IPI00789008	FLOT2	-0.581
IPI00790135	OSBPL8	-0.596
IPI00793199	ANXA4	-2.240
IPI00793443	IPO5	0.151
IPI00794169	ANO10	-0.631
IPI00794229	SPCS1	0.812
IPI00797136	IKIP	-1.329
IPI00843975	EZR	-1.936
IPI00844578	DHX9	1.954
IPI00847300	-	0.742
IPI00847342	KRT7	1.170
IPI00852623	PLXNB2	-1.175
IPI00876963	HLA-A	-1.660
IPI00884105	LAMP1	-1.113

Integrated dataset hESCs/HFF		
IPI	Gene Symbol	log2 ratio
IPI00794223	COPG	-1.201
IPI00794229	SPCS1	0.903
IPI00797136	IKIP	-2.407
IPI00843975	EZR	-1.087
IPI00844578	DHX9	1.828
IPI00847300	-	-0.419
IPI00847342	KRT7	1.409
IPI00876963	HLA-A	-1.966
IPI00884105	LAMP1	-1.023
IPI00902496	SEC63	-0.293
IPI00910210	ATAD1	1.414
IPI00914855	MTX1	2.084
IPI00941296	CTSB	-1.988
IPI00941747	CANX	0.462
IPI00943562	LOC100133770	-0.477
IPI00944575	HLA-C	-2.858

Integrated dataset hiPSCs/hESCs		
IPI	Gene Symbol	log2 ratio
IPI00554541	ILVBL	0.097
IPI00554701	UCRC	-0.515
IPI00554723	RPL10	0.208
IPI00554742	API5	-0.775
IPI00554788	KRT18	0.936
IPI00555610	AHNAK	0.206
IPI00555703	TMEM41B	-0.185
IPI00555744	RPL14	-0.005
IPI00555902	OCIAD2	-0.245
IPI00641435	LASS2	0.138
IPI00641829	BAT1	-0.415
IPI00641924	MRPS9	-0.098
IPI00642244	KIAA0090	-0.004
IPI00642329	MFN2	-0.198
IPI00642370	MPV17L2	-0.041
IPI00643041	RANP1;RAN	0.344
IPI00644020	SOAT1	-0.577
IPI00644766	TOR1AIP1	0.047
IPI00645518	CDIPT	-0.088
IPI00645667	MBOAT7	0.209
IPI00645767	GCNT2	0.522
IPI00645966	NONO	1.156
IPI00646304	PPIB	-0.257
IPI00646426	MRS2	-0.437
IPI00646625	TAP1	-0.283
IPI00646779	TUBB6	0.320
IPI00646963	REEP6	0.602
IPI00647457	HLA-A	-0.136
IPI00654777	EIF3F	-0.866
IPI00654820	MT-ATP6	-0.205
IPI00657706	MBOAT7	0.373
IPI00739274	LOC652614	-0.042
IPI00743576	ATP6V0A1	-0.032
IPI00743716	HLA-C	0.141
IPI00747849	ATP1B1	-0.026
IPI00748145	GNAI2	-0.155
IPI00749429	TTYH3	0.096
IPI00760554	HLA-A	0.776
IPI00783097	GARS	0.233
IPI00783271	LRPPRC	-0.345
IPI00783698	TMEM87A	0.245
IPI00784119	ATP6AP1	0.272
IPI00784154	HSPD1	-0.395
IPI00784376	MTP18	-0.054
IPI00787853	IMPAD1	0.789
IPI00788624	SLC25A3	0.067

Table S2: The integrated datasets for the comparison of hiPSCs/HFF, hESCs/HFF, and hiPSCs/hESCs.

Integrated dataset hiPSCs/HFF		
IPI	Gene Symbol	log2 ratio
IPI00902496	SEC63	0.614
IPI00910210	ATAD1	2.482
IPI00914855	MTX1	1.902
IPI00916241	TMEM87B	0.249
IPI00922108	ITGAV	-0.968
IPI00930070	HLA-C	-1.150
IPI00941296	CTSB	-1.109
IPI00941747	CANX	0.590
IPI00943562	LOC10013377	0.280
IPI00944575	HLA-C	-3.254

Integrated dataset hESCs/HFF		
IPI	Gene Symbol	log2 ratio

Integrated dataset hiPSCs/hESCs		
IPI	Gene Symbol	log2 ratio
IPI00789008	FLOT2	-0.101
IPI00790135	OSBPL8	-0.041
IPI00793199	ANXA4	0.353
IPI00793443	IPO5	0.201
IPI00793874	SFXN3	-0.546
IPI00794169	ANO10	-0.022
IPI00794223	COPG	0.295
IPI00794229	SPCS1	-0.112
IPI00797136	IKIP	0.032
IPI00843975	EZR	-0.978
IPI00844578	DHX9	-0.061
IPI00847300	-	1.142
IPI00847342	KRT7	-0.224
IPI00848226	GNB2L1	-0.171
IPI00852623	PLXNB2	-0.214
IPI00876963	HLA-A	0.271
IPI00884105	LAMP1	-0.102
IPI00902496	SEC63	-0.176
IPI00902680	SAMM50	-0.500
IPI00910210	ATAD1	1.045
IPI00914855	MTX1	-0.168
IPI00922108	ITGAV	-0.094
IPI00930070	HLA-C	-0.153
IPI00936742	LOC100293491	-0.106
IPI00941296	CTSB	1.363
IPI00941747	CANX	0.134
IPI00943562	LOC100133770	0.736
IPI00944575	HLA-C	0.394

Supplementary Table S3

The differential proteins contribute to the enrichment of the significant pathways.

Table S3 The differential proteins contribute to the enrichment of the significant pathways.

Core enrichment proteins in down-regulated pathways for hiPSCs/HFF (hiPSCs vs HFF)

Gene Symbol	KEGG FOCAL_ADHESION	KEGG REGULATION_OF_ACTIN_CYTOSKELETON	REACTOME DEVELOPMENTAL_BIOLOGY	REACTOME AXON_GUIDANCE	REACTOME PLATELET_ACTIVATION_SIGNALING_AND_AGGREGATION	REACTOME HEMOSTASIS	KEGG LEUKOCYTE_TRANSENDOTHELIAL_MIGRATION	REACTOME ADAPTIVE_IMMUNE_SYSTEM
ITGB1	1	1	1	1	0	1	1	1
RHOA	1	1	1	1	1	1	1	0
RAP1B	1	0	0	0	1	1	1	1
CDC42	1	1	0	0	1	1	0	1
COL1A1	1	0	1	1	1	1	0	0
ITGA5	1	1	1	1	0	1	0	0
TLN1	1	0	1	1	1	1	0	0
VCL	1	1	0	0	1	1	1	0
ACTN1	1	1	0	0	1	1	1	0
CFL1	0	1	1	1	1	1	0	0
YWHAZ	0	0	0	0	1	1	0	1
FN1	1	1	0	0	1	1	0	0
CAP1	0	0	1	1	1	1	0	0
PPP2R1A	0	0	0	0	0	1	0	1
GNG12	0	1	0	0	1	1	0	0
GNAI1	0	0	0	0	1	1	1	0
GNA13	0	1	0	0	1	1	0	0
PSAP	0	0	0	0	1	1	0	0
A2M	0	0	0	0	1	1	0	0
CAV1	1	0	0	0	0	1	0	0
GNAQ	0	0	0	0	1	1	0	0
LAMP2	0	0	0	0	1	1	0	0
ALDOA	0	0	0	0	1	1	0	0
GNA11	0	0	0	0	1	1	0	0
PPIA	0	0	0	0	1	1	0	0
CD63	0	0	0	0	1	1	0	0
GNAS	0	0	0	0	0	1	0	0
SLC16A3	0	0	0	0	0	1	0	0
EHD2	0	0	0	0	0	1	0	0
CLTC	0	0	1	1	0	0	0	1
MSN	0	1	1	1	0	0	1	0
EZR	0	1	1	1	0	0	1	0
MYH9	0	1	1	1	0	0	0	0
RRAS	0	1	1	1	0	0	0	0
ACTB	1	1	0	0	0	0	1	0
COL6A3	1	0	1	1	0	0	0	0
LNPEP	0	0	0	0	0	0	0	1
CTSB	0	0	0	0	0	0	0	1

Gene Symbol	REACTOME IMMUNE_SYSTEM	KEGG_TIGHT_ JUNCTION
ITGB1	1	0
RHOA	0	1
RAP1B	1	0
CDC42	1	1
COL1A1	0	0
ITGA5	0	0
TLN1	0	0
VCL	0	0
ACTN1	0	1
CFL1	0	0
YWHAZ	1	0
FN1	0	0
CAP1	0	0
PPP2R1A	1	1
GNG12	0	0
GNAI1	0	1
GNA13	0	0
PSAP	0	0
A2M	0	0
CAV1	0	0
GNAQ	0	0
LAMP2	0	0
ALDOA	0	0
GNA11	0	0
PPIA	0	0
CD63	0	0
GNAS	0	0
SLC16A3	0	0
EHD2	0	0
CLTC	1	0
MSN	0	0
EZR	0	0
MYH9	0	1
RRAS	0	1
ACTB	0	1
COL6A3	0	0
LNPEP	1	0
CTSB	1	0

Table S3 The differential proteins contribute to the enrichment of the significant pathways.

Core enrichment proteins in down-regulated pathways for hiPSCs/HFF (hiPSCs vs HFF)

Gene Symbol	KEGG FOCAL_ADHESION	KEGG REGULATION_OF_ACTIN_CYTOSKELETON	REACTOME DEVELOPMENTAL_BIOLOGY	REACTOME AXON_GUIDANCE	REACTOME PLATELET_ACTIVATION_SIGNALING_AND_AGGREGATION	REACTOME HEMOSTASIS	KEGG LEUKOCYTE_TRANSENDOTHELIAL_MIGRATION	REACTOME ADAPTIVE_IMMUNE_SYSTEM
DYNC1H1	0	0	0	0	0	0	0	1
HLA-C	0	0	0	0	0	0	0	1
CTSD	0	0	0	0	0	0	0	1
ARF1	0	0	0	0	0	0	0	1
RAB7A	0	0	0	0	0	0	0	1
PSMC4	0	0	0	0	0	0	0	1
CD81	0	0	0	0	0	0	0	1
PDGFRB	1	1	0	0	0	0	0	0
PANX1	0	0	0	0	0	0	0	0
KPNB1	0	0	0	0	0	0	0	0
LGALS3	0	0	0	0	0	0	0	0
FLNA	1	0	0	0	0	0	0	0
RRAS2	0	1	0	0	0	0	0	0
THY1	0	0	0	0	0	0	1	0
ACTR1A	0	0	0	0	0	0	0	1
SAR1B	0	0	0	0	0	0	0	1
ITGAV	1	1	1	1	0	1	0	1
TAP1	0	0	0	0	0	0	0	1
AP1B1	0	0	0	0	0	0	0	1
MRC2	0	0	0	0	0	0	0	1
CD44	0	0	0	0	0	0	0	0
IFITM3	0	0	0	0	0	0	0	0
UBA52	0	0	0	0	0	0	0	1
EGFR	1	1	1	1	0	0	0	0
FLNC	1	0	0	0	0	0	0	0
GNB2	0	0	0	0	1	1	0	0
ATP2B3	0	0	0	0	0	1	0	0
CD9	0	0	0	0	1	1	0	0
GNAI2	0	0	0	0	0	0	0	0
PPP2R2B	0	0	0	0	0	0	0	0
RAB3B	0	0	0	0	0	0	0	0

Gene Symbol	REACTOME IMMUNE_SYSTEM	KEGG_TIGHT_ JUNCTION
DYNC1H1	1	0
HLA-C	1	0
CTSD	1	0
ARF1	1	0
RAB7A	1	0
PSMC4	1	0
CD81	1	0
PDGFRB	0	0
PANX1	1	0
KPNB1	1	0
LGALS3	1	0
FLNA	0	0
RRAS2	0	0
THY1	0	0
ACTR1A	1	0
SAR1B	1	0
ITGAV	1	0
TAP1	1	0
AP1B1	1	0
MRC2	1	0
CD44	1	0
IFITM3	1	0
UBA52	0	0
EGFR	0	0
FLNC	0	0
GNB2	0	0
ATP2B3	0	0
CD9	0	0
GNAI2	0	1
PPP2R2B	0	1
RAB3B	0	1

Table S3 The differential proteins contribute to the enrichment of the significant pathways.

Core enrichment proteins in up-regulated pathways for hiPSCs/HFF (hiPSCs vs HFF)

Gene Symbol	KEGG OXIDATIVE_ PHOSPHORYLATI ON	KEGG HUNTINGTONS_ DISEASE	KEGG ALZHEIMERS_ DISEASE	KEGG PARKINSONS_ DISEASE	REACTOME TCA_CYCLE_AND_ RESPIRATORY_ ELECTRON_ TRANSPORT	REACTOME RESPIRATORY_ELECTRON _ TRANSPORT_ATP_SYNTHE SIS_BY_CHEMIOSMOTIC_C OUPLING_AND_HEAT_PRO DUCTION_BY_UNCOUPLIN	REACTOME METABOLISM_OF_ PROTEINS	REACTOME POST_TRANSLATI ONAL_PROTEIN_ MODIFICATION
ALG6	0	0	0	0	0	0	1	1
ARSE	0	0	0	0	0	0	1	1
ATP5H	1	1	1	1	1	1	0	0
ATP5I	1	0	0	0	1	1	0	0
ATP5J2	1	0	0	0	1	1	0	0
ATP5L	1	0	0	0	1	1	0	0
ATP6V0A2	1	0	0	0	0	0	0	0
BSG	0	0	0	0	1	0	0	0
CANX	0	0	0	0	0	0	1	1
COX15	1	0	0	0	0	0	0	0
COX4I1	1	1	1	1	1	1	0	0
COX5A	1	1	1	1	1	1	0	0
CYC1	1	0	0	0	1	0	1	0
DAD1	0	0	0	0	0	0	1	1
DDOST	0	0	0	0	0	0	1	1
DOLPP1	0	0	0	0	0	0	1	1
FAU	0	0	0	0	0	0	1	0
GALNT2	0	0	0	0	0	0	1	1
GALNT7	0	0	0	0	0	0	1	1
GGCX	0	0	0	0	0	0	1	1
HSPA9	0	0	0	0	0	0	1	0
HSPD1	0	0	0	0	0	0	1	0
LOC653566	0	0	0	0	0	0	1	0
MAN2A1	0	0	0	0	0	0	1	1
MDH2	0	0	0	0	1	0	0	0
MGAT1	0	0	0	0	0	0	1	1
MLEC	0	0	0	0	0	0	1	1
MTX1	0	0	0	0	0	0	1	0
NDUFA11	1	0	0	0	1	1	0	0
NDUFA4	1	1	1	1	1	1	0	0

Gene Symbol	REACTOME ASPARAGINE_ N_LINKED_ GLYCOSYLATION
ALG6	1
ARSE	0
ATP5H	0
ATP5I	0
ATP5J2	0
ATP5L	0
ATP6V0A2	0
BSG	0
CANX	1
COX15	0
COX4I1	0
COX5A	0
CYC1	0
DAD1	1
DDOST	1
DOLPP1	1
FAU	0
GALNT2	0
GALNT7	0
GGCX	0
HSPA9	0
HSPD1	0
LOC653566	0
MAN2A1	1
MDH2	0
MGAT1	1
MLEC	1
MTX1	0
NDUFA11	0
NDUFA4	0

Table S3 The differential proteins contribute to the enrichment of the significant pathways.

Core enrichment proteins in up-regulated pathways for hiPSCs/HFF (hiPSCs vs HFF)

Gene Symbol	KEGG OXIDATIVE_ PHOSPHORYLATI ON	KEGG HUNTINGTONS_ DISEASE	KEGG ALZHEIMERS_ DISEASE	KEGG PARKINSONS_ DISEASE	REACTOME TCA_CYCLE_AND_ RESPIRATORY_ ELECTRON_ TRANSPORT	REACTOME RESPIRATORY_ELECTRON _ TRANSPORT_ATP_SYNTHE SIS_BY_CHEMIOSMOTIC_C OUPLING_AND_HEAT_PRO DUCTION_BY_UNCOUPLIN	REACTOME METABOLISM_OF_ PROTEINS	REACTOME POST_TRANSLATI ONAL_PROTEIN_ MODIFICATION
NDUFA9	1	0	0	0	1	1	0	0
NDUFB10	1	1	1	1	1	1	0	0
NDUFB6	1	1	1	1	1	1	0	0
NDUFC2	1	1	1	1	1	1	0	0
NOP56	0	0	0	0	0	0	1	0
PIGK	0	0	0	0	0	0	1	1
PIGS	0	0	0	0	0	0	1	1
PIGT	0	0	0	0	0	0	1	1
PIGU	0	0	0	0	0	0	1	1
RPL13	0	0	0	0	0	0	1	0
RPL15	0	0	0	0	0	0	1	0
RPL18A	0	0	0	0	0	0	1	0
RPL19	0	0	0	0	0	0	1	0
RPL27A	0	0	0	0	0	0	1	0
RPL4	0	0	0	0	0	0	1	0
RPN1	0	0	0	0	0	0	1	1
RPN2	0	0	0	0	0	0	1	1
RPS19	0	0	0	0	0	0	1	0
RPS20	0	0	0	0	0	0	1	0
RPS26	0	0	0	0	0	0	1	0
SDHC	1	1	1	1	1	1	0	0
SEC11A	0	0	0	0	0	0	1	0
SEC61G	0	0	0	0	0	0	1	0
SLC16A1	0	0	0	0	1	0	0	0
SLC25A13	0	0	0	0	0	0	1	0
SLC25A4	0	1	0	1	0	0	1	0
SLC25A5	0	1	0	1	0	0	0	0
SLC25A6	0	1	0	1	0	0	1	0
SPCS1	0	0	0	0	0	0	1	0
SPCS3	0	0	0	0	0	0	1	0
SSR1	0	0	0	0	0	0	1	0
SSR3	0	0	0	0	0	0	1	0
STT3A	0	0	0	0	0	0	1	1
TIMM50	0	0	0	0	0	0	1	0
TOMM22	0	0	0	0	0	0	1	0
TOMM40	0	0	0	0	0	0	1	0
TOMM7	0	0	0	0	0	0	1	0

Gene Symbol	REACTOME ASPARAGINE_ N_LINKED_ GLYCOSYLATION
NDUFA9	0
NDUFB10	0
NDUFB6	0
NDUFC2	0
NOP56	0
PIGK	0
PIGS	0
PIGT	0
PIGU	0
RPL13	0
RPL15	0
RPL18A	0
RPL19	0
RPL27A	0
RPL4	0
RPN1	1
RPN2	1
RPS19	0
RPS20	0
RPS26	0
SDHC	0
SEC11A	0
SEC61G	0
SLC16A1	0
SLC25A13	0
SLC25A4	0
SLC25A5	0
SLC25A6	0
SPCS1	0
SPCS3	0
SSR1	0
SSR3	0
STT3A	1
TIMM50	0
TOMM22	0
TOMM40	0
TOMM7	0

Table S3 The differential proteins contribute to the enrichment of the significant pathways.

Core enrichment proteins in up-regulated pathways for hiPSCs/HFF (hiPSCs vs HFF)

Gene Symbol	KEGG OXIDATIVE_ PHOSPHORYLATI ON	KEGG HUNTINGTONS_ DISEASE	KEGG ALZHEIMERS_ DISEASE	KEGG PARKINSONS_ DISEASE	REACTOME TCA_CYCLE_AND_ RESPIRATORY_ ELECTRON_ TRANSPORT	REACTOME RESPIRATORY_ELECTRON _ TRANSPORT_ATP_SYNTHE SIS_BY_CHEMIOSMOTIC_C OUPLING_AND_HEAT_PRO DUCTION_BY_UNCOUPLIN	REACTOME METABOLISM_OF_ PROTEINS	REACTOME POST_TRANSLATI ONAL_PROTEIN_ MODIFICATION
TOMM70A	0	0	0	0	0	0	1	0
UGGT1	0	0	0	0	0	0	1	1
UQCRC2	1	1	1	1	1	1	0	0
UQCRFS1	1	1	1	1	1	1	0	0
UQCRQ	1	1	1	1	1	1	0	0
VDAC1	0	1	0	1	0	0	1	0
VDAC2	0	1	0	1	0	0	0	0
WBSCR17	0	0	0	0	0	0	1	1

Gene Symbol	REACTOME ASPARAGINE_ N_LINKED_ GLYCOSYLATION
TOMM70A	0
UGGT1	1
UQCRC2	0
UQCRFS1	0
UQCRQ	0
VDAC1	0
VDAC2	0
WBSCR17	0

Table S3 The differential proteins contribute to the enrichment of the significant pathways.

Core enrichment proteins in down-regulated pathways of hESCs/HFF (hESCs vs HFF)

Gene Symbol	KEGG FOCAL_ADHESION	KEGG REGULATION_OF_ACTIN_CYTOSKELETON	REACTOME DEVELOPMENTAL_BIOLOGY	REACTOME AXON_GUIDANCE	REACTOME PLATELET_ACTIVATION_SIGNALING_AND_AGGREGATION	REACTOME HEMOSTASIS	KEGG LEUKOCYTE_TRANSENDOTHELIAL_MIGRATION	REACTOME ADAPTIVE_IMMUNE_SYSTEM
A2M	0	0	0	0	1	1	0	0
ACTB	1	1	0	0	0	0	1	0
ACTN1	1	1	0	0	1	1	1	0
ALDOA	0	0	0	0	1	1	0	0
ARF1	0	0	0	0	0	0	0	1
CAP1	0	0	1	1	0	0	0	0
CAV1	1	0	0	0	0	1	0	0
CD63	0	0	0	0	1	1	0	0
CD81	0	0	0	0	0	0	0	1
CDC42	1	1	1	1	0	0	1	0
CFL1	0	1	1	1	1	1	0	0
CLTC	0	0	1	1	0	0	0	1
COL1A1	1	0	1	1	1	1	0	0
COL6A3	1	0	1	1	0	0	0	0
CTSB	0	0	0	0	0	0	0	1
CTSD	0	0	0	0	0	0	0	1
DYNC1H1	0	0	0	0	0	0	0	1
EHD2	0	0	0	0	0	1	0	0
EZR	0	1	1	1	0	0	1	0
FLNA	1	0	0	0	0	0	0	0
FN1	1	1	0	0	1	1	0	0
GNA11	0	0	0	0	1	1	0	0
GNA13	0	1	0	0	0	0	0	0
GNAI1	0	0	0	0	1	1	1	0
GNAQ	0	0	0	0	0	1	0	0
GNAS	0	0	0	0	0	1	0	0
GNG12	0	1	0	0	1	1	0	0
HLA-C	0	0	0	0	0	0	0	1
ITGA5	1	1	1	1	0	1	0	0
ITGB1	1	1	1	1	0	1	1	1
KPNB1	0	0	0	0	0	0	0	0
LAMP2	0	0	0	0	1	1	0	0
LGALS3	0	0	0	0	0	0	0	0
LNPEP	0	0	0	0	0	0	0	1
MSN	0	1	1	1	0	0	1	0
MYH9	0	1	1	1	0	0	0	0
PANX1	0	0	0	0	0	0	0	0
PDGFRB	1	1	0	0	0	0	0	0
PPIA	0	0	0	0	1	1	0	0
PPP2R1A	0	0	0	0	0	1	0	1
PSAP	0	0	0	0	1	1	0	0
PSMC4	0	0	0	0	0	0	0	1

Gene Symbol	REACTOME IMMUNE_SYSTEM
A2M	0
ACTB	0
ACTN1	0
ALDOA	0
ARF1	1
CAP1	0
CAV1	0
CD63	0
CD81	1
CDC42	0
CFL1	0
CLTC	1
COL1A1	0
COL6A3	0
CTSB	1
CTSD	1
DYNC1H1	1
EHD2	0
EZR	0
FLNA	0
FN1	0
GNA11	0
GNA13	0
GNAI1	0
GNAQ	0
GNAS	0
GNG12	0
HLA-C	1
ITGA5	0
ITGB1	1
KPNB1	1
LAMP2	0
LGALS3	1
LNPEP	1
MSN	0
MYH9	0
PANX1	1
PDGFRB	0
PPIA	0
PPP2R1A	1
PSAP	0
PSMC4	1

Table S3 The differential proteins contribute to the enrichment of the significant pathways.

Core enrichment proteins in down-regulated pathways of hESCs/HFF (hESCs vs HFF)

Gene Symbol	KEGG FOCAL_ADHESION	KEGG REGULATION_OF_ACTIN_CYTOSKELETON	REACTOME DEVELOPMENTAL_BIOLOGY	REACTOME AXON_GUIDANCE	REACTOME PLATELET_ACTIVATION_SIGNALING_AND_AGGREGATION	REACTOME HEMOSTASIS	KEGG LEUKOCYTE_TRANSENDOTHELIAL_MIGRATION	REACTOME ADAPTIVE_IMMUNE_SYSTEM
RAB7A	0	0	0	0	0	0	0	1
RAP1B	1	0	0	0	1	1	1	1
RHOA	1	1	1	1	1	1	1	0
RRAS	0	1	1	1	0	0	0	0
RRAS2	0	1	0	0	0	0	0	0
SLC16A3	0	0	0	0	0	1	0	0
THY1	0	0	0	0	0	0	1	0
TLN1	1	0	1	1	1	1	0	0
VCL	1	1	0	0	1	1	1	0
YWHAZ	0	0	0	0	1	1	0	1
CLDN7	0	0	0	0	0	0	1	0
COL1A2	1	0	1	1	0	0	0	0
CTNNA1	0	0	1	0	0	0	1	0
IQGAP1	0	1	0	0	0	0	0	0
ITGB3	1	0	0	0	0	0	0	0
THBS1	1	0	0	0	0	0	0	0

Gene Symbol	REACTOME IMMUNE_SYSTEM
RAB7A	1
RAP1B	1
RHOA	0
RRAS	0
RRAS2	0
SLC16A3	0
THY1	0
TLN1	0
VCL	0
YWHAZ	1
CLDN7	0
COL1A2	0
CTNNA1	0
IQGAP1	0
ITGB3	0
THBS1	0

Table S3 The differential proteins contribute to the enrichment of the significant pathways.

Core enrichment proteins in up-regulated pathways for hESCs/HFF (hESCs vs HFF)

Gene Symbol	KEGG OXIDATIVE_ PHOSPHOR YLATION	KEGG HUNTINGTONS_ DISEASE	KEGG ALZHEIMERS_ DISEASE	REACTOME TCA_CYCLE_AND_RE SPIRATORY_ ELECTRON_ TRANSPORT
ATP5H	1	1	1	1
ATP5I	1	0	0	1
ATP5J2	1	0	0	1
ATP5L	1	0	0	1
ATP6V0A2	1	0	0	0
BSG	0	0	0	1
COX15	1	0	0	0
COX4I1	1	1	1	1
CYC1	1	1	1	1
MDH2	0	0	0	1
NDUFA4	1	1	1	1
NDUFA9	1	1	1	1
NDUFB10	1	1	1	1
NDUFB6	1	1	1	1
NDUFC2	1	1	1	1
SLC16A1	0	0	0	1
SLC25A5	0	1	0	0
SLC25A6	0	1	0	0
UQCRC2	1	1	1	1
UQCRFS1	1	1	1	1
UQCRQ	1	1	1	1
VDAC1	0	1	0	0
VDAC2	0	1	0	0
ATP5C1	1	1	1	1

Table S3 The differential proteins contribute to the enrichment of the significant pathways.

Core enrichment proteins in down-regulated pathways for hiPSCs/hESCs (hiPSCs vs hESCs)

Gene Symbol	KEGG OXIDATIVE_ PHOSPHORY LATION	KEGG HUNTINGTONS_ DISEASE	KEGG ALZHEIMERS_ DISEASE	KEGG PARKINSONS_ DISEASE	REACTOME TCA_CYCLE_AND _RESPIRATORY_ ELECTRON_ TRANSPORT	REACTOME RESPIRATORY_ELECTRON_TRANSPO RT_ATP_SYNTHESIS_BY_CHEMIOSM OTIC_COUPLING_AND_HEAT_PRODU CTION_BY_UNCOUPLING_PROTEINS	REACTOME MITOCHONDRIAL_ PROTEIN_IMPORT
ADAM10	0	0	1	0	0	0	0
ATP5A1	1	1	1	1	1	1	1
ATP5C1	1	1	1	1	1	1	0
ATP5F1	1	1	1	1	1	1	0
ATP5H	1	1	1	1	1	1	0
ATP5J2	1	0	0	0	1	1	0
ATP5L	1	0	0	0	1	1	0
ATP6V0C	1	0	0	0	0	0	0
COX4I1	1	1	1	1	1	1	0
COX5A	0	1	0	1	0	0	0
CYC1	1	1	1	1	1	1	1
GAPDH	0	0	1	0	0	0	0
HSPD1	0	0	0	0	0	0	1
LDHA	0	0	0	0	1	0	0
LDHB	0	0	0	0	1	0	0
MTX1	0	0	0	0	0	0	1
NDUFA4	0	1	0	1	0	0	0
NDUFA9	1	1	1	1	1	1	0
NDUFB4	1	1	1	1	1	1	0
NDUFB6	1	1	1	1	1	1	0
NDUFC2	0	1	0	1	0	0	0
SAMM50	0	0	0	0	0	0	1
SDHC	1	1	1	1	1	1	0
SLC16A1	0	0	0	0	1	0	0
SLC25A5	0	1	0	1	0	0	0
SLC25A6	0	1	0	1	0	0	1
TIMM44	0	0	0	0	0	0	1
TIMM50	0	0	0	0	0	0	1
TOMM20	0	0	0	0	0	0	1
TOMM22	0	0	0	0	0	0	1
TOMM5	0	0	0	0	0	0	1
TOMM7	0	0	0	0	0	0	1
TOMM70A	0	0	0	0	0	0	1
UQCRC1	1	1	1	1	1	1	0
UQCRC2	0	1	0	1	0	0	0
UQCRFS1	1	1	1	1	1	1	0
UQCRQ	1	1	1	1	1	1	0
VDAC1	0	1	0	1	0	0	1
VDAC2	0	1	0	1	0	0	0

Table S3 The differential proteins contribute to the enrichment of the significant pathways.

Core enrichment proteins in down-regulated pathways for hiPSCs/hESCs (hiPSCs vs hESCs)

Gene Symbol	KEGG OXIDATIVE_ PHOSPHORY LATION	KEGG HUNTINGTONS_ DISEASE	KEGG ALZHEIMERS_ DISEASE	KEGG PARKINSONS_ DISEASE	REACTOME TCA_CYCLE_AND _RESPIRATORY_ ELECTRON_ TRANSPORT	REACTOME RESPIRATORY_ELECTRON_TRANSPO RT_ATP_SYNTHESIS_BY_CHEMIOSM OTIC_COUPLING_AND_HEAT_PRODU CTION_BY_UNCOUPLING_PROTEINS	REACTOME MITOCHONDRIAL_ PROTEIN_IMPORT
VDAC3	0	1	0	1	0	0	0

Table S3 The differential proteins contribute to the enrichment of the significant pathways.

Core enrichment proteins in up-regulated pathways for hiPSCs/hESCs (hiPSCs vs hESCs)

Gene Symbol	REACTOME POST_TRANSLATIONAL_PROTEIN_ MODIFICATION	REACTOME ASPARAGINE_ N_LINKED_ GLYCOSYLATION	KEGG N_GLYCAN_ BIOSYNTHESIS	KEGG FOCAL_ADHESION
ACTN1	0	0	0	1
ALG1	1	1	1	0
ALG5	1	1	1	0
ALG6	0	1	1	0
B4GALT1	1	1	1	0
CANX	1	1	0	0
CAV1	0	0	0	1
COL1A2	0	0	0	1
CTNNB1	0	0	0	1
DDOST	0	1	1	0
DOLK	1	1	0	0
DOLPP1	1	1	1	0
FN1	0	0	0	1
GALNT2	1	0	0	0
GALNT7	1	0	0	0
GANAB	1	1	1	0
GGCX	1	0	0	0
ITGB3	0	0	0	1
MAN1A2	1	1	1	0
MAN1B1	1	1	1	0
MAN2A1	1	1	1	0
PIGK	1	0	0	0
PIGT	1	0	0	0
PIGU	1	0	0	0
RAC1	0	0	0	1
RAP1B	0	0	0	1
RFT1	1	1	1	0
RHOA	0	0	0	1
RPN1	1	1	1	0
RPN2	1	1	1	0
STT3B	0	0	1	0
THBS1	0	0	0	1
VCL	0	0	0	1

Supplementary Table S4

The integrated datasets for the comparison of hiPSCs/HFs, hESCs/HFs, and hiPSCs/hESCs from the public datasets.

Table S4 The integrated datasets for the comparison of hiPSCs/HFs, hESCs/HFs, and hiPSCs/hESCs from the public datasets.

AccessionNo	GeneSymbol	hiPSCs/HFs	AccsessionNo	GeneSymbol	hESCs/HFs	AccessionNo	GeneSymbol	hiPSCs/hESCs
IPI00922466.1	-	2.899	CON_IPI0047914ŧ	KRT19	2.5	CON_IPI0047914ŧ	KRT19	0.7295
IPI00909336.1	-	-0.205	CON_IPI0055464ł	KRT8	3.32325	CON_IPI0055464ł	KRT8	0.39025
IPI00909229.1	-	-0.4155	IPI:CON_0069890	ACTB	-1.7805	IPI:CON_0069890	ACTB	0.8035
IPI00922290.1	-	-1.49175	IPI00000005.1	NRAS	-0.20825	IPI00000005.1	NRAS	0.1785
IPI00918020.1	-	-6.833	IPI00000015.2	SRSF4	1.05975	IPI00000015.2	SRSF4	-0.04325
IPI00024143.4	AAAS	1.8945	IPI00000051.4	PFDN1	-1.11025	IPI00000051.4	PFDN1	0.626
IPI00217272.7	AACS	0.39225	IPI00000105.4	MVP	-4.55075	IPI00000105.4	MVP	-1.10875
IPI00916534.1	AAK1	-2.878	IPI00000138.1	MGAT1	-2.33375	IPI00000138.1	MGAT1	0.64975
IPI00924477.1	AAMP	0.5545	IPI00000190.1	CD81	-2.472	IPI00000190.1	CD81	0.757
IPI00027442.4	AARS	0.09775	IPI00000279.3	ZC3H15	0.0095	IPI00000279.3	ZC3H15	0.47075
IPI00394788.4	AARS2	0.7035	IPI00000305.3	MED11	1.318	IPI00000305.3	MED11	0.009
IPI00916940.1	AARSD1	-0.278	IPI00000335.1	HINT2	0.88925	IPI00000335.1	HINT2	-0.371
IPI00250297.3	AASDHPPT	-0.416	IPI00000494.6	SNORD21	-0.45625	IPI00000494.6	SNORD21	0.353
IPI00033217.3	AASS	3.884	IPI00000606.5	TTC4	0.09775	IPI00000606.5	TTC4	-0.1385
IPI00302238.3	AATF	1.443	IPI00000643.1	BAG2	-2.971	IPI00000643.1	BAG2	0.7285
IPI00908369.1	ABCC4	-2.94875	IPI00000686.2	RBM19	1.18325	IPI00000686.2	RBM19	-0.0905
IPI00291373.2	ABCD1	-2.5845	IPI00000733.4	UTP18	2.0985	IPI00000733.4	UTP18	-0.112
IPI00002372.1	ABCD3	-1.1885	IPI00000735.5	TSPAN13	1.99625	IPI00000735.5	TSPAN13	-0.244
IPI00303207.3	ABCE1	-0.2065	IPI00000760.1	DDAH2	-0.97375	IPI00000760.1	DDAH2	0.62625
IPI00013495.1	ABCF1	0.38025	IPI00000769.2	KIF22	2.31225	IPI00000769.2	KIF22	0.15475
IPI00005045.1	ABCF2	-1.29325	IPI00000792.1	CRYZ	0.68875	IPI00000792.1	CRYZ	-0.2765
IPI00910706.1	ABHD14B	-4.15775	IPI00000816.1;P622	YWHAE	-0.13875	IPI00000816.1;P622	YWHAE	0.3295
IPI00442211.1	ABI2	-0.0925	IPI00000821.2	MRPL16	0.079	IPI00000821.2	MRPL16	0.406
IPI00216969.3	ABL1	-0.52375	IPI00000846.1	CHD4	1.79575	IPI00000846.1	CHD4	0.4065
IPI00002938.1	ABT1	2.14175	IPI00000861.1	LASP1	-3.6215	IPI00000861.1	LASP1	0.911
IPI00001539.8	ACAA2	0.97525	IPI00000873.3	VARS	-0.8735	IPI00000873.3	VARS	0.4185
IPI00005040.1	ACADM	0.56225	IPI00000877.1	HYOU1	0.0485	IPI00000877.1	HYOU1	0.33925
IPI00024623.3	ACADSB	0.33775	IPI00001022.5	PNKD	0.27825	IPI00001022.5	PNKD	0.138
IPI00030363.1	ACAT1	-1.36225	IPI00001091.4	AFG3L2	1.7585	IPI00001091.4	AFG3L2	-0.15425

Table S4 The integrated datasets for the comparison of hiPSCs/HFs, hESCs/HFs, and hiPSCs/hESCs from the public datasets.

AccessionNo	GeneSymbol	hiPSCs/HFs
IPI00291419.6	ACAT2	1.54075
IPI00009315.6	ACBD3	-1.75225
IPI00911038.2	ACIN1	1.681
IPI00008485.1	ACO1	-1.7295
IPI00017855.1	ACO2	0.27575
IPI00219452.1	ACOT7	-0.43025
IPI00921986.1	ACOT9	-0.40175
IPI00296907.4	ACOX1	-1.40475
IPI00304071.4	ACSF2	-0.901
IPI00031397.3	ACSL3	0.17475
IPI00219897.1	ACSL4	0.55675
00698900.1;;IPI00	ACTB	-1.2765
IPI00003269.1	ACTBL2	-1.21625
IPI00216622.1	ACTL6A	1.02175
IPI00019884.1	ACTN2	-2.702
IPI00013808.1	ACTN4	-2.618
I00029468.1;P611	ACTR1A	-1.8145
I00005159.3;P611	ACTR2	-1.9815
IPI00028091.3	ACTR3	-2.12075
IPI00747502.1	ACTR6	0.772
IPI00013897.1	ADAM10	0.53875
IPI00019904.2	ADD2	2.6385
IPI00220754.1	ADD3	-1.312
IPI00746777.3	ADH5	0.05275
IPI00651738.1	ADI1	-0.799
IPI00022215.1	ADNP	1.973
IPI00045939.4	ADO	0.09725
IPI00015865.6	ADPRHL2	-0.93925
IPI00470921.4	ADRM1	-0.7155

AccsessionNo	GeneSymbol	hESCs/HFs
IPI00001146.1;P623	LSM6	1.291
IPI00001159.10	GCN1L1	-0.6455
IPI00001287.1	C20orf72	2.37425
IPI00001453.2	INA	-1.3965
IPI00001538.3	FXC1	0.567
IPI00001539.8	ACAA2	1.592
IPI00001541.1	TIMM9	0.48775
IPI00001543.1	TIMM10	0.50375
IPI00001568.1	ATP6V1D	-1.2585
IPI00001580.4	FYCO1	-3.8265
IPI00001589.1	TIMM13	1.68725
IPI00001593.1	PRCP	-0.386
IPI00001639.2	KPNB1	-0.50375
IPI00001730.3	FHOD1	-1.7035
IPI00001738.4	NUP88	0.769
IPI00001754.1	F11R	3.34125
IPI00001757.1;Q9CW	RBM8A	0.9645
IPI00001871.2	PAWR	0.77575
IPI00001883.1	SNX9	-2.40725
IPI00001960.4	CLIC4	-1.5605
IPI00002070.6	LRRC8A	-0.31575
IPI00002135.1	TACC3	2.3075
IPI00002188.2	ARFGEF1	0.6015
IPI00002214.1	KPNA2	1.59475
IPI00002236.3	MFGE8	0.16025
IPI00002240.1	FAM118B	1.59625
IPI00002335.1	HTT	-0.466
IPI00002349.2	NUFIP2	-0.0595
IPI00002372.1	ABCD3	-1.5195

AccessionNo	GeneSymbol	hiPSCs/hESCs
IPI00001146.1;P623	LSM6	0.1855
IPI00001159.10	GCN1L1	0.0425
IPI00001287.1	C20orf72	-0.37525
IPI00001453.2	INA	-0.547
IPI00001538.3	FXC1	0.23525
IPI00001539.8	ACAA2	-0.31325
IPI00001541.1	TIMM9	0.07725
IPI00001543.1	TIMM10	0.10675
IPI00001568.1	ATP6V1D	0.7505
IPI00001580.4	FYCO1	1.10875
IPI00001589.1	TIMM13	0.2255
IPI00001593.1	PRCP	0.6065
IPI00001639.2	KPNB1	0.407
IPI00001730.3	FHOD1	0.3725
IPI00001738.4	NUP88	0.2175
IPI00001754.1	F11R	0.20925
IPI00001757.1;Q9CW	RBM8A	0.284
IPI00001871.2	PAWR	0.12975
IPI00001883.1	SNX9	0.4915
IPI00001960.4	CLIC4	0.44975
IPI00002070.6	LRRC8A	0.2235
IPI00002135.1	TACC3	0.72575
IPI00002188.2	ARFGEF1	0.421
IPI00002214.1	KPNA2	0.92475
IPI00002236.3	MFGE8	0.07075
IPI00002240.1	FAM118B	-0.0705
IPI00002335.1	HTT	0.528
IPI00002349.2	NUFIP2	0.44225
IPI00002372.1	ABCD3	0.60275

Table S4 The integrated datasets for the comparison of hiPSCs/HFs, hESCs/HFs, and hiPSCs/hESCs from the public datasets.

AccessionNo	GeneSymbol	hiPSCs/HFs
IPI00942092.1	ADSL	0.09725
IPI00026833.4	ADSS	-0.016
IPI00398154.2	AFAP1	-3.889
IPI00001091.4	AFG3L2	1.3205
IPI00026259.1	AGA	-1.9735
IPI00514126.2	AGL	0.65275
IPI00028491.4	AGPAT5	2.47375
IPI00010349.1	AGPS	0.50975
IPI00374563.3	AGRN	1.55375
IPI00954159.1	AHCTF1P1	1.39275
IPI00012007.6	AHCY	0.79525
IPI00021812.2	AHNAK	-5.822
IPI00030706.1	AHSA1	-0.30325
IPI00157908.4	AIFM1	1.03775
IPI00294840.6	AIM1	-0.346
IPI00006252.3	AIMP1	-0.77575
IPI00011916.1	AIMP2	-0.4565
IPI00953925.1	AIP	-1.47025
IPI00018342.5	AK1	-2.96075
IPI00465256.4	AK3	-0.6655
IPI00016568.1	AK4	3.8345
IPI00942186.1	AKAP1	2.97925
IPI00024024.4	AKAP17A	1.685
IPI00014474.1	AKAP8	0.647
IPI00297455.5	AKAP8L	1.1105
IPI00220627.1	AKAP9	-0.71925
IPI00220271.3	AKR1A1	0.56125
IPI00413641.7	AKR1B1	-0.614
IPI00946527.1	AKR7A2	-1.73375

AccsessionNo	GeneSymbol	hESCs/HFs
IPI00002412.1	PPT1	2.45925
IPI00002521.1	ATP5J	0.4535
IPI00002525.3	NENF	-1.8215
IPI00002535.2	FKBP2	-0.764
IPI00002557.1	CHD3	0.9
IPI00002564.3	XRCC1	1.6895
IPI00002570.1	EIF4EBP2	0.6
IPI00002580.2	PIK3C2A	1.53275
IPI00002612.2	ZNF219	2.7545
IPI00002745.1	CTSZ	-2.204
IPI00002790.3	SEL1L	-1.71
IPI00002803.6	PKN1	-0.98175
IPI00002824.7	CSRP2	0.10475
IPI00002828.3	MLF1IP	2.3385
IPI00002853.1	PPP2R5E	-0.303
IPI00002879.5	C14orf169	2.66
IPI00002894.2	POLD1	2.15775
IPI00002902.5	NOL9	1.0995
IPI00002938.1	ABT1	2.159
IPI00002948.3	LIN28A	3.87025
IPI00002966.2	HSPA4	0.6015
IPI00003004.1	SLC25A22	0.1165
IPI00003016.4	STRN4	-0.067
IPI00003084.1	DRAP1	-0.54275
IPI00003168.1	PRPSAP2	0.13
IPI00003217.3	PSMB7	-0.6315
IPI00003269.1	ACTBL2	-1.133
IPI00003311.1;P628	POLR2L	0.66475
IPI00003326.4	ARL2	1.20475

AccessionNo	GeneSymbol	hiPSCs/hESCs
IPI00002412.1	PPT1	0.081
IPI00002521.1	ATP5J	-0.07625
IPI00002525.3	NENF	-0.37725
IPI00002535.2	FKBP2	-0.05475
IPI00002557.1	CHD3	0.3225
IPI00002564.3	XRCC1	-0.114
IPI00002570.1	EIF4EBP2	0.88175
IPI00002580.2	PIK3C2A	0.40225
IPI00002612.2	ZNF219	-0.1115
IPI00002745.1	CTSZ	-0.055
IPI00002790.3	SEL1L	0.434
IPI00002803.6	PKN1	0.24775
IPI00002824.7	CSRP2	0.0935
IPI00002828.3	MLF1IP	0.74275
IPI00002853.1	PPP2R5E	0.41175
IPI00002879.5	C14orf169	-0.47525
IPI00002894.2	POLD1	0.3065
IPI00002902.5	NOL9	0.314
IPI00002938.1	ABT1	0.1405
IPI00002948.3	LIN28A	0.175
IPI00002966.2	HSPA4	0.22225
IPI00003004.1	SLC25A22	-0.22025
IPI00003016.4	STRN4	0.076
IPI00003084.1	DRAP1	0.0475
IPI00003168.1	PRPSAP2	0.14575
IPI00003217.3	PSMB7	0.50225
IPI00003269.1	ACTBL2	0.28475
IPI00003311.1;P628	POLR2L	0.3665
IPI00003326.4	ARL2	0.752

Table S4 The integrated datasets for the comparison of hiPSCs/HFs, hESCs/HFs, and hiPSCs/hESCs from the public datasets.

AccessionNo	GeneSymbol	hiPSCs/HFs
IPI00217920.7	ALDH16A1	-0.09025
IPI00103467.4	ALDH1B1	1.09875
IPI00006663.1	ALDH2	0.1805
IPI00217871.4	ALDH4A1	1.221
IPI00024990.6	ALDH6A1	1.6375
IPI00936002.1	ALDH7A1	0.981
IPI00479877.4	ALDH9A1	-0.85175
IPI00465439.5	ALDOA	-1.025
IPI00418262.5	ALDOC	0.6165
IPI00909749.1	ALG5	-1.41175
IPI00419916.4	ALPL	2.52875
IPI00033907.1	ANAPC1	0.4445
IPI00945732.1	ANAPC7	0.234
IPI00829741.2	ANKLE2	-0.9655
IPI00894366.1	ANKMY2	-1.01575
IPI00395663.5	ANKS1A	1.2515
IPI00759824.2	ANP32B	-1.11
IPI00030431.1	ANTXR1	-0.665
IPI00218918.5	ANXA1	-6.833
IPI00909703.2	ANXA11	-3.47325
IPI00455315.4	ANXA2	-5.11775
IPI00024095.3	ANXA3	3.128
IPI00872780.1	ANXA4	-4.33925
IPI00872379.1	ANXA5	-3.414
IPI00953297.1	ANXA7	-2.03225
IPI00032516.5	AP1M1	-2.276
256684.2;IPI00793	AP2A1	-1.63525
IPI00914938.1	AP2A2	-0.87375
IPI00619900.2	AP2M1	-1.41925

AccsessionNo	GeneSymbol	hESCs/HFs
IPI00003327.1	ARL3	-0.426
IPI00003348.3;P628	GNB2	-0.22675
IPI00003362.2;P200	HSPA5	-1.54475
IPI00003406.4	DBN1	-0.7165
IPI00003419.1	C11orf58	-0.57225
IPI00003438.2	DNAJC8	0.26025
IPI00003479.3	MAPK1	-2.21525
IPI00003482.1	DECR1	-1.19675
IPI00003515.1	TRIP11	-1.872
IPI00003527.5	SLC9A3R1	3.868
IPI00003704.4	RBM4	2.0715
IPI00003766.4	ETHE1	-3.3735
IPI00003783.1	MAP2K2	-1.33
IPI00003802.2	MAN2A1	-0.24975
IPI00003815.3	ARHGDIA	-3.1215
IPI00003833.3	MTCH2	0.26575
IPI00003848.1	DNAJB4	-4.47975
IPI00003865.1	HSPA8	-0.699
IPI00003870.1	CLPP	-0.1485
IPI00003881.5	HNRNPF	1.3355
IPI00003886.3	SNORD19B	1.04875
IPI00003909.1	SLC2A3	5.12675
IPI00003918.6	RPL4	-0.315
IPI00003923.1	UMPS	-0.271
IPI00003927.5	PPID	-0.41425
IPI00003944.1	DBT	2.04125
IPI00003964.4	USP9X	0.50975
IPI00003968.1	NDUFA9	-0.384
IPI00003990.1	BPHL	1.70825

AccessionNo	GeneSymbol	hiPSCs/hESCs
IPI00003327.1	ARL3	0.30925
IPI00003348.3;P628	GNB2	-0.24875
IPI00003362.2;P200	HSPA5	0.59375
IPI00003406.4	DBN1	0.55325
IPI00003419.1	C11orf58	0.422
IPI00003438.2	DNAJC8	0.26425
IPI00003479.3	MAPK1	0.531
IPI00003482.1	DECR1	-0.57325
IPI00003515.1	TRIP11	0.5085
IPI00003527.5	SLC9A3R1	0.30925
IPI00003704.4	RBM4	-0.035
IPI00003766.4	ETHE1	-0.20025
IPI00003783.1	MAP2K2	0.0415
IPI00003802.2	MAN2A1	0.856
IPI00003815.3	ARHGDIA	0.80975
IPI00003833.3	MTCH2	-0.35125
IPI00003848.1	DNAJB4	-0.039
IPI00003865.1	HSPA8	0.46925
IPI00003870.1	CLPP	0.0905
IPI00003881.5	HNRNPF	-0.0175
IPI00003886.3	SNORD19B	0.41
IPI00003909.1	SLC2A3	-0.836
IPI00003918.6	RPL4	0.2865
IPI00003923.1	UMPS	0.65575
IPI00003927.5	PPID	1.52375
IPI00003944.1	DBT	-0.58675
IPI00003964.4	USP9X	0.01925
IPI00003968.1	NDUFA9	0.28575
IPI00003990.1	BPHL	0.13625

Table S4 The integrated datasets for the comparison of hiPSCs/HFs, hESCs/HFs, and hiPSCs/hESCs from the public datasets.

AccessionNo	GeneSymbol	hiPSCs/HFs
IPI00021129.5	AP3B1	-1.87075
IPI00014624.2	AP3S1	-1.82625
IPI00337741.4	APEH	0.5225
IPI00215911.3	APEX1	0.3635
IPI00554742.3	API5	1.0305
IPI00021842.1	APOE	2.79075
IPI00877964.1	APOL2	-3.91275
IPI00843886.1	APOO	1.45075
IPI00015836.1	APPL1	-1.3285
IPI00297572.5	AQR	1.4405
IPI00872240.1	ARAF	-0.379
IPI00103380.1	ARAP3	1.029
IPI00514053.1	ARCN1	-1.6925
IPI00215920.8	ARF6	-1.513
IPI00175169.3	ARFGAP1	-1.26775
IPI00915304.1	ARFGAP3	-2.94075
IPI00002188.2	ARFGEF1	0.63525
IPI00020332.1	ARG2	2.90275
IPI00020567.3	ARHGAP1	-2.2435
IPI00718985.1	ARHGAP35	-1.007
IPI00003815.3	ARHGDIA	-2.60575
IPI00157442.1	ARHGEF11	-0.3825
IPI00152007.1	ARHGEF17	-4.0465
IPI00941678.1	ARHGEF2	-1.17275
IPI00018134.4	ARHGEF40	-0.0755
IPI00643722.1	ARID1A	1.84375
IPI00742937.1	ARID1B	1.117
IPI00018500.1	ARID3A	2.37375
IPI00793438.1	ARID3B	2.937

AccsessionNo	GeneSymbol	hESCs/HFs
IPI00004233.2	MKI67	3.31125
IPI00004273.6	RBM25	1.28075
IPI00004290.3	DIEXF	1.20725
IPI00004358.4	PYGB	-2.47025
IPI00004416.1	CHMP2A	-0.3815
IPI00004436.1	LSM1	-0.39725
IPI00004454.3	DPM3	-0.02425
IPI00004459.1	DIMT1	0.56425
IPI00004488.2	ATP6V1F	-1.169
IPI00004523.2	ZFAND6	1.26975
IPI00004533.1	KIF3B	-1.461
IPI00004534.4	PFAS	0.78475
IPI00004584.1	KIAA0391	1.80875
IPI00004655.1	FRG1	0.7095
IPI00004656.3	B2M	-1.469
IPI00004669.1	GALNT2	-2.03975
IPI00004671.2	GOLGB1	-0.654
IPI00004795.1	CLNS1A	1.009
IPI00004839.1	CRKL	0.57125
IPI00004845.4	NIPSNAP3A	-1.8205
IPI00004859.1	BLM	4.286
IPI00004860.2	RARS	-0.7285
IPI00004902.1	ETFB	-0.23575
IPI00004942.1	ZNF330	-1.38375
IPI00004962.2	GOLIM4	-2.91025
IPI00004968.1	PRPF19	1.195
IPI00004970.4	UTP20	1.32225
IPI00005024.3	MYBBP1A	0.9975
IPI00005036.1	RBM5	0.832

AccessionNo	GeneSymbol	hiPSCs/hESCs
IPI00004233.2	MKI67	0.76
IPI00004273.6	RBM25	0.16925
IPI00004290.3	DIEXF	-0.1135
IPI00004358.4	PYGB	0.5635
IPI00004416.1	CHMP2A	0.06875
IPI00004436.1	LSM1	0.42025
IPI00004454.3	DPM3	0.16925
IPI00004459.1	DIMT1	0.65375
IPI00004488.2	ATP6V1F	0.70225
IPI00004523.2	ZFAND6	0.69475
IPI00004533.1	KIF3B	0.93175
IPI00004534.4	PFAS	0.5755
IPI00004584.1	KIAA0391	0.41875
IPI00004655.1	FRG1	-0.045
IPI00004656.3	B2M	-1.54475
IPI00004669.1	GALNT2	0.4385
IPI00004671.2	GOLGB1	0.00575
IPI00004795.1	CLNS1A	-0.0605
IPI00004839.1	CRKL	0.1665
IPI00004845.4	NIPSNAP3A	0.087
IPI00004859.1	BLM	0.2845
IPI00004860.2	RARS	0.257
IPI00004902.1	ETFB	-0.43875
IPI00004942.1	ZNF330	0.46025
IPI00004962.2	GOLIM4	0.65775
IPI00004968.1	PRPF19	0.04725
IPI00004970.4	UTP20	0.598
IPI00005024.3	MYBBP1A	0.39525
IPI00005036.1	RBM5	0.002

Table S4 The integrated datasets for the comparison of hiPSCs/HFs, hESCs/HFs, and hiPSCs/hESCs from the public datasets.

AccessionNo	GeneSymbol	hiPSCs/HFs
IPI00003326.4	ARL2	1.24175
IPI00003327.1	ARL3	-0.4345
IPI00014232.1	ARL6IP1	0.31275
IPI00908696.2	ARL6IP5	-2.6555
IPI00167074.4	ARL6IP6	0.70475
IPI00060031.3	ARL8A	-0.34425
IPI00789069.2	ARL8B	-1.217
IPI00018260.1	ARMC1	-0.02475
IPI00006142.3	ARMCX2	0.36025
IPI00009906.5	ARMCX3	-1.84175
IPI00333068.4	ARPC1A	-1.2165
IPI00737530.1	ARPC1B	-2.66325
IPI00005161.3	ARPC2	-1.97
IPI00554811.2	ARPC4	-1.946
IPI00550234.4	ARPC5	-2.87475
IPI00414554.5	ARPC5L	-1.01525
IPI00336017.5	ARRB1	1.58675
IPI00306576.1	ARSB	-1.76
IPI00879180.1	ARVCF	2.37
IPI00434390.2	ASCC1	-1.113
IPI00430472.2	ASCC3	-0.80575
IPI00249080.2	ASMTL	0.679
IPI00013466.4	ASNA1	-0.192
IPI00294834.6	ASPH	-3.996
IPI00170548.1	ATAD2	1.6465
IPI00295992.4	ATAD3A	-0.34175
IPI00449201.2	ATG3	-1.48875
IPI00289499.3	ATIC	0.33225
IPI00550523.5	ATL3	-3.35325

AccsessionNo	GeneSymbol	hESCs/HFs
IPI00005040.1	ACADM	0.7755
IPI00005045.1	ABCF2	-1.71075
IPI00005050.1	MRPS14	0.63225
IPI00005087.1	TMOD3	-0.3405
IPI00005101.6	SENP1	0.87175
IPI00005102.3	SMS	0.1835
IPI00005104.1	CHUK	-2.778
IPI00005154.1	SSRP1	1.52525
IPI00005159.3;P611	ACTR2	-2.29975
IPI00005161.3	ARPC2	-2.314
IPI00005198.2;Q9CX	ILF2	1.12175
IPI00005202.2	PGRMC2	-1.0825
IPI00005492.2	WDR5	1.51025
IPI00005511.1	PHF5A	1.131
IPI00005537.3	SLC25A10	0.677
IPI00005578.1	EHD4	-2.17075
IPI00005585.5	TAX1BP3	-2.82075
IPI00005613.3	U2AF1	0.66625
IPI00005614.6	SPTBN1	0.38125
IPI00005634.3	TTC37	-1.8765
IPI00005648.1	SAFB2	1.1215
IPI00005657.1	PFDN6	-0.738
IPI00005675.3	NKRF	0.56625
IPI00005707.6	MRC2	-4.6375
IPI00005719.1;P628	RAB1A	-1.0345
IPI00005745.1	SPTLC1	-0.226
IPI00005826.1	HERC2	0.042
IPI00005861.1	PRPF3	0.66775
IPI00005904.3	DDX20	0.8195

AccessionNo	GeneSymbol	hiPSCs/hESCs
IPI00005040.1	ACADM	0.01475
IPI00005045.1	ABCF2	0.682
IPI00005050.1	MRPS14	0.26825
IPI00005087.1	TMOD3	-0.00925
IPI00005101.6	SENP1	0.339
IPI00005102.3	SMS	-0.03825
IPI00005104.1	CHUK	1.8205
IPI00005154.1	SSRP1	0.07175
IPI00005159.3;P611	ACTR2	0.56025
IPI00005161.3	ARPC2	0.61725
IPI00005198.2;Q9CX	ILF2	0.2535
IPI00005202.2	PGRMC2	0.12775
IPI00005492.2	WDR5	-0.032
IPI00005511.1	PHF5A	0.127
IPI00005537.3	SLC25A10	-0.08625
IPI00005578.1	EHD4	0.66275
IPI00005585.5	TAX1BP3	0.772
IPI00005613.3	U2AF1	0.17025
IPI00005614.6	SPTBN1	0.15775
IPI00005634.3	TTC37	0.351
IPI00005648.1	SAFB2	0.36225
IPI00005657.1	PFDN6	0.50025
IPI00005675.3	NKRF	0.6355
IPI00005707.6	MRC2	0.23
IPI00005719.1;P628	RAB1A	0.2355
IPI00005745.1	SPTLC1	0.292
IPI00005826.1	HERC2	0.536
IPI00005861.1	PRPF3	0.461
IPI00005904.3	DDX20	0.25625

Table S4 The integrated datasets for the comparison of hiPSCs/HFs, hESCs/HFs, and hiPSCs/hESCs from the public datasets.

AccessionNo	GeneSymbol	hiPSCs/HFs
IPI00010863.5	ATOX1	-1.50075
IPI00218353.3	ATP13A1	-0.768
IPI00744194.1	ATP1A1	0.9575
IPI00006482.1	ATP1A1	0.64025
IPI00871221.1	ATP1B1	0.94325
IPI00008167.1	ATP1B3	0.7115
IPI00219078.5	ATP2A2	-0.7855
IPI00216529.1	ATP2B1	-0.513
IPI00607568.3	ATP2C1	-0.54725
IPI00440493.2	ATP5A1	0.09025
IPI00303476.1	ATP5B	0.084
IPI00395769.2	ATP5C1	0.15625
IPI00024920.1	ATP5D	-0.07025
IPI00029133.4	ATP5F1	0.07525
IPI00220487.4	ATP5H	0.2385
IPI00218848.5	ATP5I	0.129
IPI00002521.1	ATP5J	0.06075
IPI00027448.3	ATP5L	-0.315
IPI00007611.1	ATP5O	0.07075
IPI00784119.1	ATP6AP1	0.52225
IPI00743576.1	ATP6V0A1	-0.22625
IPI00018855.1	ATP6V0C	-0.2835
IPI00034159.1	ATP6V0D1	-0.91775
IPI00007682.2	ATP6V1A	-0.8005
IPI00007812.1	ATP6V1B2	-1.15275
IPI00001568.1	ATP6V1D	-1.1615
IPI00719806.1	ATP6V1E1	-0.9725
IPI00004488.2	ATP6V1F	-0.76325
IPI00025285.3	ATP6V1G1	-1.12275

AccsessionNo	GeneSymbol	hESCs/HFs
IPI00005914.4	SHPK	1.08025
IPI00005966.6	NDUFA12	-0.75825
IPI00005996.1	HMGA2	-0.1625
IPI00006025.1	SART3	0.91575
IPI00006052.3	PFDN2	-0.65575
IPI00006092.1	PMM2	-1.264
IPI00006099.1	BMS1	1.58175
IPI00006103.1	CD2BP2	-0.17875
IPI00006142.3	ARMCX2	0.7325
IPI00006167.1	PPM1G	0.261
IPI00006181.1	EIF3D	-0.58
IPI00006196.3	NUMA1	2.38075
IPI00006211.4	VAPB	0.10925
IPI00006252.3	AIMP1	-0.53725
IPI00006378.3	CCDC72	0.53025
IPI00006379.1	NOP58	1.2105
IPI00006408.4	NOSIP	-0.97625
IPI00006440.6	MRPS7	0.272
IPI00006442.1	COIL	2.28
IPI00006482.1	ATP1A1	0.46525
IPI00006538.1	PTDSS2	-0.145
IPI00006558.3	SH3GLB1	-2.136
IPI00006579.1	COX4I1	0.55125
IPI00006606.1	MRPS26	1.047
IPI00006663.1	ALDH2	-0.54275
IPI00006702.4	PELP1	1.88575
IPI00006713.1	DNAJC3	-1.29775
IPI00006715.3	RAD21	2.21
IPI00006864.3	MRPL33	-0.15725

AccessionNo	GeneSymbol	hiPSCs/hESCs
IPI00005914.4	SHPK	0.341
IPI00005966.6	NDUFA12	0.51075
IPI00005996.1	HMGA2	0.19675
IPI00006025.1	SART3	-0.04725
IPI00006052.3	PFDN2	0.36275
IPI00006092.1	PMM2	0.486
IPI00006099.1	BMS1	0.12125
IPI00006103.1	CD2BP2	0.34325
IPI00006167.1	PPM1G	0.41775
IPI00006181.1	EIF3D	0.46225
IPI00006196.3	NUMA1	0.328
IPI00006211.4	VAPB	0.13625
IPI00006252.3	AIMP1	0.1055
IPI00006378.3	CCDC72	0.59325
IPI00006379.1	NOP58	0.3645
IPI00006408.4	NOSIP	1.12425
IPI00006440.6	MRPS7	0.4435
IPI00006442.1	COIL	-0.14125
IPI00006482.1	ATP1A1	0.487
IPI00006538.1	PTDSS2	0.42
IPI00006558.3	SH3GLB1	0.262
IPI00006579.1	COX4I1	0.2385
IPI00006606.1	MRPS26	0.0325
IPI00006663.1	ALDH2	0.99475
IPI00006702.4	PELP1	0.00325
IPI00006713.1	DNAJC3	-0.13975
IPI00006715.3	RAD21	-0.09675
IPI00006864.3	MRPL33	0.4505
IPI00006865.3;O085	SEC22B	0.24875

Table S4 The integrated datasets for the comparison of hiPSCs/HFs, hESCs/HFs, and hiPSCs/hESCs from the public datasets.

AccessionNo	GeneSymbol	hiPSCs/HFs	AccsessionNo	GeneSymbol	hESCs/HFs	AccessionNo	GeneSymbol	hiPSCs/hESCs
IPI00553153.1	ATPIF1	-0.0815	IPI00006865.3;O085	SEC22B	-3.2195	IPI00006907.1	C12orf5	0.80725
IPI00456359.1	ATXN2L	-0.1845	IPI00006907.1	C12orf5	-1.1085	IPI00006970.2	MRPS2	0.25125
IPI00102904.1	AUH	2.0085	IPI00006970.2	MRPS2	0.8785	IPI00006980.1	C14orf166	0.0005
IPI00215815.3	AUP1	-0.15225	IPI00006980.1	C14orf166	-0.15125	IPI00006987.1	DDX24	0.38625
IPI00004656.3	B2M	-3.483	IPI00006987.1	DDX24	0.873	IPI00007004.4	RRP15	0.27275
IPI00101987.6	BABAM1	-0.131	IPI00007004.4	RRP15	1.209	IPI00007019.1	PPIL1	0.16575
IPI00024291.2	BAD	-2.81125	IPI00007019.1	PPIL1	1.00975	IPI00007024.1	FAM96B	0.32325
IPI00000643.1	BAG2	-2.524	IPI00007024.1	FAM96B	-0.5175	IPI00007032.1	SALL4	0.40475
IPI00641582.1	BAG3	-3.45125	IPI00007032.1	SALL4	2.25325	IPI00007052.6	FIS1	-0.558
IPI00798092.1	BAG4	-0.05875	IPI00007052.6	FIS1	-0.5335	IPI00007058.1	CORO1B	0.292
IPI00007731.1	BAG5	-0.2005	IPI00007058.1	CORO1B	-1.33775	IPI00007063.5	HDGFRP3	-0.126
IPI00640922.1	BAG6	0.11125	IPI00007063.5	HDGFRP3	1.02025	IPI00007074.5	YARS	0.5625
IPI00179326.7	BAIAP2L1	1.57425	IPI00007074.5	YARS	-0.689	IPI00007084.3	SLC25A13	0.4575
IPI00026087.1	BANF1	0.9115	IPI00007084.3	SLC25A13	2.83475	IPI00007087.4	FBXO2	-0.836
IPI00332572.3	BANP	1.393	IPI00007087.4	FBXO2	2.31275	IPI00007163.1	LSM7	0.028
IPI00299024.9	BASP1	-0.66525	IPI00007163.1	LSM7	1.569	IPI00007188.5	SLC25A5	0.50425
IPI00071059.4	BAX	-0.17525	IPI00007188.5	SLC25A5	0.15525	IPI00007208.4	DDX41	-0.08025
IPI00383565.5	BAZ1A	1.043	IPI00007208.4	DDX41	1.55425	IPI00007306.1	CFDP1	0.12825
IPI00216695.1	BAZ1B	0.0745	IPI00007306.1	CFDP1	1.47375	IPI00007402.3	IPO7	0.43125
IPI00759742.2	BAZ2A	2.3295	IPI00007402.3	IPO7	-0.76175	IPI00007611.1	ATP5O	-0.113
IPI00218200.8	BCAP31	-0.64225	IPI00007611.1	ATP5O	0.4085	IPI00007675.6	DYNC1LI1	0.54825
IPI00025178.3	BCAS2	1.20925	IPI00007675.6	DYNC1LI1	-1.315	IPI00007676.3	HSD17B12	0.1085
IPI00181135.4	BCAT2	-0.07975	IPI00007676.3	HSD17B12	0.9145	IPI00007682.2	ATP6V1A	0.23025
IPI00413672.1	BCLAF1	1.0355	IPI00007682.2	ATP6V1A	-0.6865	IPI00007694.5	PPME1	0.0935
IPI00607799.5	BDH2	-0.084	IPI00007694.5	PPME1	-1.513	IPI00007702.1	HSPA2	-1.26525
IPI00741958.1	BEND3	2.542	IPI00007702.1	HSPA2	-0.07925	IPI00007729.1	NOL7	0.1405
IPI00924776.1	BET1	0.1465	IPI00007729.1	NOL7	1.1375	IPI00007731.1	BAG5	0.23275
IPI00178185.1	BICD2	-2.65925	IPI00007731.1	BAG5	-0.05675	IPI00007755.3	RAB21	1.7225
IPI00943215.1	BID	-1.9575	IPI00007755.3	RAB21	-2.33175	IPI00007765.5	HSPA9	0.207

Table S4 The integrated datasets for the comparison of hiPSCs/HFs, hESCs/HFs, and hiPSCs/hESCs from the public datasets.

AccessionNo	GeneSymbol	hiPSCs/HFs
IPI00939720.1	BIRC6	-0.0605
IPI00004859.1	BLM	4.16825
IPI00294158.1	BLVRA	-1.89
IPI00783862.2	BLVRB	-1.74225
IPI00006099.1	BMS1	1.43375
IPI00791679.2	BNIP3L	-0.862
IPI00797574.3	BOD1L	0.657
IPI00747913.2	BOLA2B	0.19325
IPI00028955.4	BOP1	0.83025
IPI00003990.1	BPHL	1.637
IPI00014414.2	BRD2	1.13425
IPI00440727.1	BRD4	1.81825
IPI00181728.1	BRIX1	0.731
IPI00844524.1	BSDC1	-1.27775
IPI00019906.1	BSG	1.095
IPI00419473.5	BTF3	-0.465
IPI00328987.3	BYSL	0.36775
REV_IPI00006254.	BZRAP1	0.02225
IPI00939991.1	C10ORF58	3.22225
IPI00003419.1	C11orf58	-0.35525
IPI00100775.1	C11orf67	-0.8775
IPI00432065.1	C11ORF83	2.0395
IPI00029444.1	C12orf10	-0.42475
IPI00550986.4	C12orf11	0.8835
IPI00184546.4	C12orf23	-3.95125
IPI00795249.2	C12orf43	0.90775
IPI00006907.1	C12orf5	-0.6895
IPI00796572.1	C12ORF57	-0.8715
IPI00032506.2	C14orf142	-0.80325

AccsessionNo	GeneSymbol	hESCs/HFs
IPI00007765.5	HSPA9	0.92475
IPI00007797.3	FABP5	1.23625
IPI00007811.1	CDK4	-2.70225
IPI00007812.1	ATP6V1B2	-1.24025
IPI00007927.4;IPI00232	SMC2	0.41875
IPI00007928.4	PRPF8	1.14875
IPI00007935.5	PDLIM5	-3.1355
IPI00008034.1	RAB23	-1.1735
IPI00008164.2	PREP	0.0595
IPI00008167.1	ATP1B3	0.36625
IPI00008215.1	ME1	-4.11725
IPI00008219.1	RAD23A	-1.3625
IPI00008240.2	MARS	0.009
IPI00008274.7	CAP1	-3.15725
IPI00008380.1	PPP2CA	-0.0205
IPI00008433.4	RPS5	-0.28675
IPI00008438.1	RPS10	-0.14
IPI00008454.1	DNAJB11	-1.178
IPI00008475.1	HMGCS1	1.429
IPI00008477.1	TPX2	3.36525
IPI00008485.1	ACO1	-1.651
IPI00008524.1	PABPC1	-0.15875
IPI00008527.3	RPLP1	-0.4195
IPI00008529.1	RPLP2	-0.93825
IPI00008530.1	RPLP0	-0.47825
IPI00008531.1	RCOR1	1.2845
IPI00008536.3	YEATS4	1.6625
IPI00008552.6	GLRX3	-0.37225
IPI00008557.5	IGF2BP1	3.159

AccessionNo	GeneSymbol	hiPSCs/hESCs
IPI00007797.3	FABP5	1.03825
IPI00007811.1	CDK4	1.178
IPI00007812.1	ATP6V1B2	0.35025
IPI00007927.4;IPI00232	SMC2	0.52525
IPI00007928.4	PRPF8	0.31475
IPI00007935.5	PDLIM5	0.98025
IPI00008034.1	RAB23	0.396
IPI00008164.2	PREP	0.59
IPI00008167.1	ATP1B3	0.63525
IPI00008215.1	ME1	0.6545
IPI00008219.1	RAD23A	-0.1085
IPI00008240.2	MARS	0.2825
IPI00008274.7	CAP1	0.72425
IPI00008380.1	PPP2CA	0.31625
IPI00008433.4	RPS5	0.175
IPI00008438.1	RPS10	0.31225
IPI00008454.1	DNAJB11	0.339
IPI00008475.1	HMGCS1	2.1145
IPI00008477.1	TPX2	0.151
IPI00008485.1	ACO1	0.29275
IPI00008524.1	PABPC1	0.6455
IPI00008527.3	RPLP1	0.2225
IPI00008529.1	RPLP2	0.57025
IPI00008530.1	RPLP0	0.24175
IPI00008531.1	RCOR1	0.065
IPI00008536.3	YEATS4	0.30625
IPI00008552.6	GLRX3	0.4445
IPI00008557.5	IGF2BP1	0.167
IPI00008616.3	SLC12A7	-0.31125

Table S4 The integrated datasets for the comparison of hiPSCs/HFs, hESCs/HFs, and hiPSCs/hESCs from the public datasets.

AccessionNo	GeneSymbol	hiPSCs/HFs
IPI00006980.1	C14orf166	-0.44825
IPI00002879.5	C14orf169	1.9015
IPI00749237.1	C14orf2	-0.03125
IPI00216999.2	C14orf21	1.143
IPI00395462.4	C15orf63	-0.834
IPI00554560.4	C16orf88	2.78375
IPI00790228.1	C17orf49	2.089
IPI00782974.1	C17orf85	0.3565
IPI00152407.3	C18orf25	0.319
IPI00056357.3	C19orf10	-2.69375
IPI00157215.5	C19orf52	-0.351
IPI00384722.2	C19orf63	-0.63025
IPI00101299.3	C1GALT1C1	0.77275
IPI00016605.3	C1orf123	-0.05875
IPI00940320.1	C1ORF144	-1.408
IPI00844507.2	C1orf174	0.674
IPI00646750.2	C1orf31	0.6455
IPI00871233.1	C1orf55	1.431
IPI00014230.1	C1QBP	1.44
IPI00016634.1	C20orf11	-0.74875
IPI00166013.1	C20orf4	0.07625
IPI00297121.5	C20orf43	-1.03175
IPI00001287.1	C20orf72	1.54175
IPI00550689.3	C22orf28	-0.57825
IPI00014194.3	C2orf29	1.31025
IPI00910109.1	C2orf56	2.161
IPI00945551.1	C3orf26	1.95325
IPI00385928.2	C4orf14	0.99225
IPI00016532.3	C4orf27	1.13125

AccsessionNo	GeneSymbol	hESCs/HFs
IPI00008616.3	SLC12A7	1.29575
IPI00008708.5	RSL1D1	1.0975
IPI00008711.3	WFS1	-1.7025
IPI00008728.1	CLPX	0.276
IPI00008732.1	TRABD	1.075
IPI00008787.3	NAGLU	-0.09225
IPI00008868.3	MAP1B	-2.88775
IPI00008918.1	LIMA1	-2.34775
IPI00008961.1	TERF2IP	-0.07225
IPI00008964.3	RAB1B	-1.482
IPI00008986.1	SLC7A5	0.13825
IPI00009009.4	CWC15	1.206
IPI00009010.3	TRMT112	0.48825
IPI00009032.1	SSB	0.3185
IPI00009104.7	RUVBL2	0.2535
IPI00009111.1	TPBG	-3.12125
IPI00009253.2	NAPA	-1.23925
IPI00009305.1	GNPDA1	-0.05575
IPI00009315.6	ACBD3	-1.4825
IPI00009328.4	EIF4A3	1.4225
IPI00009329.2	UTRN	-0.9925
IPI00009342.1	IQGAP1	-2.58425
IPI00009368.4	SFXN1	0.21075
IPI00009407.8	DAD1	-0.65975
IPI00009464.1	EXOSC10	1.0495
IPI00009471.1	WDR3	1.8525
IPI00009505.1	SNTB2	-2.9745
IPI00009542.1	MAGED2	-0.763
IPI00009598.1	SMN2	1.1445

AccessionNo	GeneSymbol	hiPSCs/hESCs
IPI00008708.5	RSL1D1	0.1985
IPI00008711.3	WFS1	0.15925
IPI00008732.1	TRABD	0.29175
IPI00008787.3	NAGLU	-0.37775
IPI00008868.3	MAP1B	0.99375
IPI00008918.1	LIMA1	0.0505
IPI00008961.1	TERF2IP	-0.32275
IPI00008964.3	RAB1B	0.46525
IPI00008986.1	SLC7A5	1.65975
IPI00009009.4	CWC15	0.04975
IPI00009010.3	TRMT112	0.1915
IPI00009032.1	SSB	0.28775
IPI00009104.7	RUVBL2	0.2235
IPI00009111.1	TPBG	0.682
IPI00009253.2	NAPA	0.39175
IPI00009305.1	GNPDA1	0.4975
IPI00009315.6	ACBD3	-0.0295
IPI00009328.4	EIF4A3	0.079
IPI00009329.2	UTRN	-0.08675
IPI00009342.1	IQGAP1	0.5895
IPI00009368.4	SFXN1	0.1485
IPI00009407.8	DAD1	0.14525
IPI00009464.1	EXOSC10	0.47375
IPI00009471.1	WDR3	0.05525
IPI00009505.1	SNTB2	0.1555
IPI00009542.1	MAGED2	0.85975
IPI00009598.1	SMN2	0.08975
IPI00009659.3	RPRD1B	-0.04825
IPI00009680.3	MRPL44	0.44775

Table S4 The integrated datasets for the comparison of hiPSCs/HFs, hESCs/HFs, and hiPSCs/hESCs from the public datasets.

AccessionNo	GeneSymbol	hiPSCs/HFs
IPI00019962.3	C4orf43	1.568
IPI00855846.1	C6orf115	1.47175
IPI00939281.1	C6orf120	-0.48325
IPI00514293.1	C6orf125	0.44
IPI00152083.3	C7orf11	0.8575
IPI00892533.1	C7orf50	1.15225
IPI00030968.4	C9orf142	-0.41625
IPI00166873.3	C9orf23	-0.156
IPI00607692.2	C9orf5	-0.37575
IPI00303812.7	C9orf82	1.28925
IPI00032561.1	CAB39	-1.27675
IPI00953262.1	CACNA2D1	-1.08325
IPI00395627.3	CACYBP	0.4455
IPI00893035.1	CAD	0.3455
IPI00020599.1	CALR	-1.46725
IPI00941900.1	CALU	-2.7035
IPI00025729.1	CAMLG	-0.31375
IPI00176702.4	CAMSAP3	2.87825
IPI00100160.3	CAND1	-0.32025
941747.1;IPI00020	CANX	-0.56275
IPI00939159.2	CAP1	-2.61625
IPI00008274.7	CAP1	-2.71725
IPI00027341.1	CAPG	-0.50125
IPI00011285.1	CAPN1	-1.562
IPI00289758.6	CAPN2	-5.72675
IPI00025084.3	CAPNS1	-3.54975
IPI00873926.1	CAPRIN1	-0.23175
IPI00873484.1	CAPZA1	-1.64025
IPI00026182.5	CAPZA2	-1.2465

AccsessionNo	GeneSymbol	hESCs/HFs
IPI00009659.3	RPRD1B	0.669
IPI00009680.3	MRPL44	0.19825
IPI00009688.1	PIP4K2A	-0.70375
IPI00009703.1	SOX2	3.57125
IPI00009730.1	MID1IP1	0.334
IPI00009755.3	METTL3	1.01675
IPI00009790.1	PFKP	-1.69175
IPI00009844.1	GMPR2	-0.50475
IPI00009896.1	EPHX1	0.296
IPI00009901.1	NUTF2	-0.6735
IPI00009904.1	PDIA4	0.11375
IPI00009906.5	ARMCX3	-1.88125
IPI00009922.3	SLIRP	1.13825
IPI00009923.1	P4HA1	-3.392
IPI00009946.4	TOMM34	-1.36475
IPI00009950.1	LMAN2	-0.68925
IPI00010080.2	OXSR1	-0.6535
IPI00010090.1	GCLM	-2.56125
IPI00010105.1	EIF6	-0.604
IPI00010141.4	POLE3	-0.26825
IPI00010153.5;P628	RPL23	-0.48125
IPI00010157.1	MAT2A	-0.23475
IPI00010182.4	DBI	-0.72825
IPI00010219.1	SPC25	0.249
IPI00010271.3;P630	RAC1	-1.365
IPI00010278.6	MRPS30	0.44175
IPI00010320.1	CBX1	2.17225
IPI00010346.1	NLN	1.61125
IPI00010349.1	AGPS	0.3575

AccessionNo	GeneSymbol	hiPSCs/hESCs
IPI00009688.1	PIP4K2A	-0.4005
IPI00009703.1	SOX2	-0.31375
IPI00009730.1	MID1IP1	1.2985
IPI00009755.3	METTL3	-0.0285
IPI00009790.1	PFKP	-0.14025
IPI00009844.1	GMPR2	0.1365
IPI00009896.1	EPHX1	0.45875
IPI00009901.1	NUTF2	0.6425
IPI00009904.1	PDIA4	0.207
IPI00009906.5	ARMCX3	0.28275
IPI00009922.3	SLIRP	0.17525
IPI00009923.1	P4HA1	-0.225
IPI00009946.4	TOMM34	0.55475
IPI00009950.1	LMAN2	-0.11325
IPI00010080.2	OXSR1	0.0815
IPI00010090.1	GCLM	0.42675
IPI00010105.1	EIF6	0.2715
IPI00010141.4	POLE3	0.973
IPI00010153.5;P628	RPL23	0.54925
IPI00010157.1	MAT2A	0.39775
IPI00010182.4	DBI	0.6
IPI00010219.1	SPC25	0.602
IPI00010271.3;P630	RAC1	0.45975
IPI00010278.6	MRPS30	0.384
IPI00010320.1	CBX1	0.01375
IPI00010346.1	NLN	0.2535
IPI00010349.1	AGPS	0.42825
IPI00010400.1	PLCB3	0.1735
IPI00010404.1	SF3B5	0.0595

Table S4 The integrated datasets for the comparison of hiPSCs/HFs, hESCs/HFs, and hiPSCs/hESCs from the public datasets.

AccessionNo	GeneSymbol	hiPSCs/HFs
IPI00642256.2	CAPZB	-1.25675
IPI00304409.3	CARHSP1	0.9375
IPI00412880.2	CARM1	-0.40725
IPI00336016.3	CARS2	-0.97525
IPI00292140.4	CASP3	0.44375
IPI00023876.4	CASP6	-0.44975
IPI00761069.1	CAST	-4.68525
IPI00465436.4	CAT	-4.6065
IPI00295386.7	CBR1	-2.94125
IPI00290462.5	CBR3	-4.3605
IPI00219352.4	CBS	2.4925
IPI00010320.1	CBX1	1.8615
IPI00297579.4	CBX3	1.1835
IPI00024662.1	CBX5	1.95325
IPI00759686.1	CC2D1A	-0.48975
IPI00939119.1	CCAR1	1.1855
IPI00553183.1	CCBL2	0.48325
IPI00060627.2	CCDC124	-0.12475
IPI00401962.2	CCDC137	0.013
IPI00792109.1	CCDC43	-0.25575
IPI00383423.3	CCDC50	-3.68475
IPI00795428.3	CCDC51	0.45875
IPI00022277.3	CCDC56	-0.18475
IPI00945719.1	CCDC58	1.42075
IPI00329594.4	CCDC59	2.1995
IPI00006378.3	CCDC72	0.84425
IPI00012199.1	CCDC86	0.73225
IPI00654603.4	CCDC88A	-0.23225
IPI00177642.8	CCDC9	-0.7435

AccsessionNo	GeneSymbol	hESCs/HFs
IPI00010400.1	PLCB3	0.37375
IPI00010404.1	SF3B5	1.0015
IPI00010414.4	PDLIM1	-1.57225
IPI00010463.5	GTPBP1	1.30175
IPI00010700.2	PRRC2A	0.05175
IPI00010706.1	GSS	-1.99275
IPI00010720.1	CCT5	-0.397
IPI00010740.1	SFPQ	1.01075
IPI00010779.4	TPM4	-2.42925
IPI00010796.1	P4HB	-2.1055
IPI00010800.2	NES	-2.0205
IPI00010863.5	ATOX1	-2.0825
IPI00010865.1;P678	CSNK2B	0.30675
IPI00010882.3	DFFA	-0.265
IPI00010896.3	CLIC1	-2.2945
IPI00010951.2	EPPK1	3.15625
IPI00011084.2	CLDN6	2.9795
IPI00011107.2	IDH2	0.29825
IPI00011126.6;P621	PSMC1	-0.7695
IPI00011200.5	PHGDH	1.20175
IPI00011201.1	ME2	1.41575
IPI00011217.3	NDUFS4	0.02
IPI00011229.1	CTSD	0.614
IPI00011250.3	UCHL3	-1.16075
IPI00011253.3	RPS3	-0.412
IPI00011274.3	HNRPDL	0.6535
IPI00011285.1	CAPN1	-1.81275
IPI00011302.1	CD59	-4.07975
IPI00011416.2	ECH1	2.512

AccessionNo	GeneSymbol	hiPSCs/hESCs
IPI00010414.4	PDLIM1	0.409
IPI00010463.5	GTPBP1	-0.063
IPI00010700.2	PRRC2A	0.6535
IPI00010706.1	GSS	0.5105
IPI00010720.1	CCT5	0.29175
IPI00010740.1	SFPQ	0.40375
IPI00010779.4	TPM4	0.4135
IPI00010796.1	P4HB	0.31125
IPI00010800.2	NES	0.33225
IPI00010863.5	ATOX1	0.85
IPI00010865.1;P678	CSNK2B	0.23525
IPI00010882.3	DFFA	0.52825
IPI00010896.3	CLIC1	0.5685
IPI00010951.2	EPPK1	-0.1055
IPI00011084.2	CLDN6	0.23725
IPI00011107.2	IDH2	-0.442
IPI00011126.6;P621	PSMC1	0.30675
IPI00011200.5	PHGDH	0.3385
IPI00011201.1	ME2	-0.419
IPI00011217.3	NDUFS4	0.23775
IPI00011229.1	CTSD	-0.114
IPI00011250.3	UCHL3	0.31975
IPI00011253.3	RPS3	0.32075
IPI00011274.3	HNRPDL	0.39025
IPI00011285.1	CAPN1	0.54325
IPI00011302.1	CD59	-0.27175
IPI00011416.2	ECH1	-0.7695
IPI00011454.1	GANAB	0.9825
IPI00011528.1	CSTF1	0.11075

Table S4 The integrated datasets for the comparison of hiPSCs/HFs, hESCs/HFs, and hiPSCs/hESCs from the public datasets.

AccessionNo	GeneSymbol	hiPSCs/HFs
IPI00306471.1	CCDC94	1.431
IPI00745793.1	CCNB1	3.397
IPI00028266.1	CCNB2	2.90725
IPI00021389.1	CCS	-0.1145
IPI00297779.7	CCT2	-0.39475
IPI00290770.3	CCT3	-0.52575
IPI00302927.6	CCT4	-0.358
IPI00010720.1	CCT5	-0.3835
IPI00027626.3	CCT6A	-0.51025
IPI00018465.1	CCT7	-0.44675
IPI00302925.4	CCT8	-0.417
IPI00298851.4	CD151	-3.194
IPI00412771.1	CD2AP	0.648
IPI00006103.1	CD2BP2	-0.00125
IPI00645816.1	CD3EAP	1.95225
IPI00011302.1	CD59	-4.62525
IPI00000190.1	CD81	-1.964
IPI00642948.1	CD99	-3.16525
IPI00797796.1	CDC27	-0.26775
IPI00013122.1	CDC37	-1.36525
IPI00016786.1	CDC42	-1.5905
IPI00477763.4	CDC42BPB	-1.10025
IPI00015894.1	CDC42EP4	-1.35275
I00300659.4;Q8JZI	CDC73	1.045
IPI00892724.1	CDCA7L	2.74025
IPI00303099.2	CDCA8	1.978
IPI00025861.3	CDH1	1.8885
IPI00658202.1	CDH2	-1.0825
IPI00645614.2	CDH3	1.60425

AccsessionNo	GeneSymbol	hESCs/HFs
IPI00011454.1	GANAB	0.8465
IPI00011528.1	CSTF1	1.19625
IPI00011603.2	PSMD3	-0.4915
IPI00011604.1	GCSH	2.40475
IPI00011619.4	PAPSS1	-0.3585
IPI00011631.6	ZW10	-0.4705
IPI00011662.1	SPINT2	3.00825
IPI00011676.2	WASL	-0.5415
IPI00011726.1	RTCD1	-1.9985
IPI00011770.1	NDUFA4	-0.05975
IPI00011857.1	CHAF1B	2.1965
IPI00011913.1	HNRNPA0	1.81875
IPI00011916.1	AIMP2	-0.37775
IPI00011937.1	PRDX4	0.45325
IPI00012007.6	AHCY	0.533
IPI00012011.6	CFL1	-1.84775
IPI00012048.1	NME1	-0.86925
IPI00012074.3	HNRNPR	0.7875
IPI00012102.1	GNS	-1.6325
IPI00012149.1	MPHOSPH10	1.75875
IPI00012197.1	DCTPP1	1.364
IPI00012199.1	CCDC86	0.1575
IPI00012268.3	PSMD2	-0.74975
IPI00012315.2	NME3	0.9445
IPI00012340.1	SRSF9	1.6965
IPI00012382.3	SNRPA	0.754
IPI00012426.1	SCPEP1	1.30125
IPI00012442.1	G3BP1	-0.291
IPI00012451.3	GNB4	-0.33225

AccessionNo	GeneSymbol	hiPSCs/hESCs
IPI00011603.2	PSMD3	0.264
IPI00011604.1	GCSH	-0.019
IPI00011619.4	PAPSS1	0.457
IPI00011662.1	SPINT2	0.05825
IPI00011676.2	WASL	0.32375
IPI00011726.1	RTCD1	0.58625
IPI00011770.1	NDUFA4	0.47325
IPI00011857.1	CHAF1B	0.56875
IPI00011913.1	HNRNPA0	0.287
IPI00011916.1	AIMP2	0.183
IPI00011937.1	PRDX4	-0.27075
IPI00012007.6	AHCY	0.5485
IPI00012011.6	CFL1	0.90275
IPI00012048.1	NME1	0.48175
IPI00012074.3	HNRNPR	0.4815
IPI00012102.1	GNS	0.151
IPI00012149.1	MPHOSPH10	-0.042
IPI00012197.1	DCTPP1	0.39125
IPI00012199.1	CCDC86	0.77675
IPI00012268.3	PSMD2	0.2555
IPI00012315.2	NME3	-0.00375
IPI00012340.1	SRSF9	0.0125
IPI00012382.3	SNRPA	0.1545
IPI00012426.1	SCPEP1	-0.4705
IPI00012442.1	G3BP1	0.6215
IPI00012451.3	GNB4	0.274
IPI00012493.1;P608	SNORD54	0.4515
IPI00012503.1	PSAP	-0.04725
IPI00012512.2	RRAS2	-0.40275

Table S4 The integrated datasets for the comparison of hiPSCs/HFs, hESCs/HFs, and hiPSCs/hESCs from the public datasets.

AccessionNo	GeneSymbol	hiPSCs/HFs	AccsessionNo	GeneSymbol	hESCs/HFs	AccessionNo	GeneSymbol	hiPSCs/hESCs
IPI00868781.2	CDK12	1.22375	IPI00012493.1;P608	SNORD54	-0.54025	IPI00012535.1	DNAJA1	0.61725
IPI00029162.3	CDK13	0.9215	IPI00012503.1	PSAP	0.7995	IPI00012575.1	PIR	-0.36725
IPI00792446.1	CDK2AP1	1.45425	IPI00012512.2	RRAS2	0.67575	IPI00012578.1	KPNA4	0.2205
IPI00007811.1	CDK4	-1.82175	IPI00012535.1	DNAJA1	-0.055	IPI00012585.1	HEXB	-0.1935
IPI00414442.3	CDK5RAP3	-1.246	IPI00012575.1	PIR	1.35275	IPI00012772.8;P629	RPL8	0.374
IPI00020991.2	CDKN2AIP	0.16225	IPI00012578.1	KPNA4	-0.90125	IPI00012795.3	EIF3I	0.28525
IPI00887796.1	CDKN2AIPNL	1.5285	IPI00012585.1	HEXB	-0.9155	IPI00012837.1	KIF5B	0.404
IPI00306723.3	CEBPZ	2.45875	IPI00012772.8;P629	RPL8	-0.4985	IPI00012887.1	CTSL1	-0.191
IPI00299177.4	CECR5	2.27525	IPI00012795.3	EIF3I	-0.66825	IPI00012912.1	CPT2	-0.4125
IPI00853320.1	CELF2	1.2305	IPI00012837.1	KIF5B	-2.16425	IPI00012966.1	TCF12	0.55075
IPI00855998.1	CENPF	1.65775	IPI00012887.1	CTSL1	-1.59975	IPI00013010.6	WWP2	0.50025
IPI00186194.5	CEP170	-1.9995	IPI00012912.1	CPT2	1.664	IPI00013068.1;P602	EIF3E	0.391
IPI00215928.4	CETN2	1.0115	IPI00012966.1	TCF12	-0.26075	IPI00013122.1	CDC37	0.71675
IPI00007306.1	CFDP1	1.28775	IPI00013010.6	WWP2	0.26325	IPI00013174.2	RBM14	0.1
IPI00012011.6	CFL1	-1.40275	IPI00013068.1;P602	EIF3E	-0.4635	IPI00013216.1	ORC2	0.03225
IPI00413344.3	CFL2	-5.01975	IPI00013122.1	CDC37	-1.8165	IPI00013219.1	ILK	0.6745
IPI00844508.1	CGN	2.82675	IPI00013174.2	RBM14	1.3935	IPI00013296.3;P622	RPS18	0.46275
IPI00307829.7	CGNL1	2.82175	IPI00013216.1	ORC2	2.7635	IPI00013297.1	PDAP1	0.43975
IPI00011857.1	CHAF1B	2.1255	IPI00013219.1	ILK	-3.3775	IPI00013396.3	SNRPC	-0.02175
IPI00060107.3	CHCHD1	0.50875	IPI00013296.3;P622	RPS18	-0.6445	IPI00013415.1;P620	RPS7	0.41
IPI00015833.1	CHCHD3	0.41175	IPI00013297.1	PDAP1	-0.901	IPI00013452.10	EPRS	0.14225
IPI00796105.1	CHCHD6	-0.647	IPI00013396.3	SNRPC	0.86875	IPI00013466.4	ASNA1	0.5465
IPI00954192.1	CHD1	1.801	IPI00013415.1;P620	RPS7	-0.3825	IPI00013475.1;Q7TM	TUBB2A	1.22675
IPI00002557.1	CHD3	0.9605	IPI00013452.10	EPRS	-0.588	IPI00013485.3	RPS2	0.41125
IPI00000846.1	CHD4	1.9275	IPI00013466.4	ASNA1	-0.5345	IPI00013495.1	ABCF1	0.3385
IPI00220289.7	CHD6	2.055	IPI00013475.1;Q7TM	TUBB2A	-0.7495	IPI00013679.1	DUT	0.2105
IPI00794880.1	CHD7	3.325	IPI00013485.3	RPS2	-0.526	IPI00013683.2	TUBB3	0.12775
IPI00045536.2	CHID1	0.73425	IPI00013495.1	ABCF1	0.2725	IPI00013706.5	MRPL20	0.291
IPI00382452.1	CHMP1A	-0.72275	IPI00013679.1	DUT	0.9265	IPI00013721.2	PRPF4B	-0.01425

Table S4 The integrated datasets for the comparison of hiPSCs/HFs, hESCs/HFs, and hiPSCs/hESCs from the public datasets.

AccessionNo	GeneSymbol	hiPSCs/HFs
IPI00156984.3	CHMP1B	0.80925
IPI00004416.1	CHMP2A	-0.71425
IPI00025974.3	CHMP4B	-0.731
IPI00100796.4	CHMP5	-1.06525
IPI00789431.1	CHMP7	-0.20825
IPI00218924.5	CHP	-0.425
IPI00872454.1	CHTF18	1.51675
IPI00644245.1	CHTOP	1.441
IPI00005104.1	CHUK	-1.2385
IPI00025333.4	CIAPIN1	0.5015
IPI00180954.4	CIRBP	-0.41025
IPI00239815.9	CIRH1A	1.56675
IPI00020510.1	CISD1	1.70875
IPI00141318.2	CKAP4	-3.71525
IPI00472887.3	CKAP5	0.20875
IPI00022977.1	CKB	3.21675
IPI00658109.1	CKMT1B	2.42275
IPI00514250.1	CKS1B	2.058
IPI00015105.1	CKS2	2.1995
IPI00413568.2	CLCN3	-0.67325
IPI00011084.2	CLDN6	2.9195
IPI00010896.3	CLIC1	-2.10275
IPI00001960.4	CLIC4	-1.40875
IPI00291930.6	CLINT1	0.13025
IPI00939839.1	CLINT1	-1.48625
IPI00217113.1	CLIP1	-2.97125
IPI00246616.2	CLIP2	-2.175
IPI00004795.1	CLNS1A	0.6875
IPI00216192.3	CLPB	-0.1715

AccsessionNo	GeneSymbol	hESCs/HFs
IPI00013683.2	TUBB3	-2.31525
IPI00013706.5	MRPL20	-0.30625
IPI00013721.2	PRPF4B	0.889
IPI00013723.3	PIN1	-1.4835
IPI00013744.1	ITGA2	-4.373
IPI00013774.1	HDAC1	0.752
IPI00013808.1	ACTN4	-2.69625
IPI00013830.1	SNW1	1.21225
IPI00013847.4	UQCRC1	0.6685
IPI00013860.3	HIBADH	0.5545
IPI00013871.1	RRM1	0.0365
IPI00013881.6	HNRNPH1	0.90225
IPI00013891.1	TRA2A	0.857
IPI00013894.1	STIP1	-0.13925
IPI00013895.1	S100A11	-3.3045
IPI00013897.1	ADAM10	0.32075
IPI00013917.3;IPI00847	RPS12	-0.31425
IPI00013930.1	STX6	0.8435
IPI00013933.2	DSP	2.89825
IPI00013939.3	RPA2	1.47625
IPI00013949.1	SGTA	-0.7555
IPI00013968.1	COX7C	0.17575
IPI00013976.3	LAMB1	-1.653
IPI00013981.4	YES1	0.81275
IPI00014053.3	TOMM40	1.17825
IPI00014149.3	TTC35	-0.3625
IPI00014151.3	PSMD6	-0.59425
IPI00014177.3	2-Sep	-1.73725
IPI00014194.3	C2orf29	1.38625

AccessionNo	GeneSymbol	hiPSCs/hESCs
IPI00013723.3	PIN1	0.623
IPI00013744.1	ITGA2	-0.09575
IPI00013774.1	HDAC1	0.11725
IPI00013808.1	ACTN4	0.353
IPI00013830.1	SNW1	0.069
IPI00013847.4	UQCRC1	0.0685
IPI00013860.3	HIBADH	-0.55275
IPI00013871.1	RRM1	0.771
IPI00013881.6	HNRNPH1	0.31375
IPI00013891.1	TRA2A	0.31975
IPI00013894.1	STIP1	0.37825
IPI00013895.1	S100A11	0.787
IPI00013897.1	ADAM10	0.4115
IPI00013917.3;IPI00847	RPS12	0.194
IPI00013930.1	STX6	-0.08325
IPI00013933.2	DSP	0.191
IPI00013939.3	RPA2	0.14525
IPI00013949.1	SGTA	0.5935
IPI00013968.1	COX7C	0.258
IPI00013976.3	LAMB1	0.97675
IPI00013981.4	YES1	0.2535
IPI00014053.3	TOMM40	-0.00075
IPI00014149.3	TTC35	0.4105
IPI00014151.3	PSMD6	0.328
IPI00014177.3	2-Sep	0.3345
IPI00014194.3	C2orf29	0.30875
IPI00014198.2	EXOSC7	0.40175
IPI00014230.1	C1QBP	-0.108
IPI00014232.1	ARL6IP1	0.122

Table S4 The integrated datasets for the comparison of hiPSCs/HFs, hESCs/HFs, and hiPSCs/hESCs from the public datasets.

AccessionNo	GeneSymbol	hiPSCs/HFs
IPI00003870.1	CLPP	-0.057
IPI00396411.4	CLPTM1	-0.97575
IPI00186429.8	CLPTM1L	-0.7565
IPI00008728.1	CLPX	0.37825
IPI00216393.1	CLTA	-1.01925
IPI00216472.1	CLTB	-3.38425
IPI00455383.4	CLTC	-1.32475
IPI00303158.3	CMAS	0.87575
IPI00177728.3	CNDP2	0.92825
IPI00021264.1	CNN1	-2.66325
IPI00216682.5	CNN3	-0.71625
IPI00395777.6	CNOT7	0.496
IPI00220993.1	CNP	-1.1215
IPI00443909.1	CNPY2	0.03675
IPI00551062.2	CNPY3	0.64
IPI00219249.4	CNTNAP1	-4.123
IPI00184821.1	COASY	-0.80625
IPI00006442.1	COIL	1.877
IPI00414694.3	COL18A1	1.67475
IPI00297646.4	COL1A1	-5.4405
IPI00304962.3	COL1A2	-6.24125
IPI00873684.1	COL4A1	0.071
IPI00844090.1	COL5A1	-3.976
IPI00291136.4	COL6A1	-4.1365
IPI00304840.4	COL6A2	-5.22875
IPI00295857.7	COPA	-1.82475
IPI00295851.4	COPB1	-1.6665
IPI00220219.6	COPB2	-1.62675
IPI00465132.4	COPE	-1.822

AccsessionNo	GeneSymbol	hESCs/HFs
IPI00014198.2	EXOSC7	1.29
IPI00014230.1	C1QBP	1.82375
IPI00014232.1	ARL6IP1	0.49575
IPI00014235.3	RAB3GAP1	-1.62625
IPI00014238.2	KARS	-0.16325
IPI00014253.4	RRS1	0.65175
IPI00014263.1	EIF4H	-1.75175
IPI00014361.1	TSTA3	-1.05575
IPI00014414.2	BRD2	1.5795
IPI00014474.1	AKAP8	0.74475
IPI00014513.1	YY1	1.744
IPI00014572.1	SPARC	-3.766
IPI00014577.1	RAB18	-1.33225
IPI00014624.2	AP3S1	-1.4745
IPI00014808.1	PAFAH1B3	0.55475
IPI00014812.1	MRPS21	0.0215
IPI00014849.3	PDK3	2.1525
IPI00014938.3	SARNP	1.397
IPI00014978.3	PPP2R5A	-0.05175
IPI00015018.1	PPA1	-1.24025
IPI00015105.1	CKS2	2.24525
IPI00015180.1	SHROOM2	3.39925
IPI00015195.1	CSTF3	1.445
IPI00015286.2	DOCK1	-0.79925
IPI00015361.1	PFDN5	-0.62275
IPI00015580.3	FNBP1L	2.6325
IPI00015602.1	TOMM70A	0.29475
IPI00015688.1	GPC1	-3.8485
IPI00015806.3	GTF3C3	2.50125

AccessionNo	GeneSymbol	hiPSCs/hESCs
IPI00014235.3	RAB3GAP1	0.261
IPI00014238.2	KARS	0.32975
IPI00014253.4	RRS1	0.37025
IPI00014263.1	EIF4H	0.75525
IPI00014361.1	TSTA3	0.71625
IPI00014414.2	BRD2	-0.192
IPI00014474.1	AKAP8	0.215
IPI00014513.1	YY1	0.206
IPI00014572.1	SPARC	1.416
IPI00014577.1	RAB18	0.5655
IPI00014624.2	AP3S1	-0.16
IPI00014808.1	PAFAH1B3	0.11175
IPI00014812.1	MRPS21	0.43625
IPI00014849.3	PDK3	-1.06175
IPI00014938.3	SARNP	0.19825
IPI00014978.3	PPP2R5A	0.2395
IPI00015018.1	PPA1	0.62275
IPI00015105.1	CKS2	0.3515
IPI00015180.1	SHROOM2	-0.20725
IPI00015195.1	CSTF3	0.059
IPI00015286.2	DOCK1	0.491
IPI00015361.1	PFDN5	0.55325
IPI00015580.3	FNBP1L	0.207
IPI00015602.1	TOMM70A	-0.08425
IPI00015688.1	GPC1	0.7575
IPI00015806.3	GTF3C3	0.096
IPI00015808.3	GNL2	0.63725
IPI00015833.1	CHCHD3	0.02
IPI00015836.1	APPL1	0.229

Table S4 The integrated datasets for the comparison of hiPSCs/HFs, hESCs/HFs, and hiPSCs/hESCs from the public datasets.

AccessionNo	GeneSymbol	hiPSCs/HFs
IPI00783982.1	COPG	-3.0305
I00743825.1;P612	COPS2	-1.396
IPI00025721.3	COPS3	-1.33125
IPI00171844.3	COPS4	-1.40275
IPI00924636.1	COPS6	-1.34375
IPI00301419.2	COPS7A	-2.93925
IPI00377080.1	COPS8	-1.13825
IPI00032851.1	COPZ1	-1.4225
IPI00456965.5	COQ5	1.4985
IPI00470631.2	COQ9	0.87925
IPI00007058.1	CORO1B	-1.371
IPI00943173.1	CORO1C	-2.5345
IPI00023736.2	CORO2A	2.26825
IPI00940326.1	CORO7	0.62425
IPI00017704.3	COTL1	-2.9815
IPI00455190.2	COX15	0.40875
IPI00167538.3	COX18	0.6545
IPI00006579.1	COX4I1	0.512
IPI00025086.4	COX5A	0.4745
IPI00021785.2	COX5B	0.08825
IPI00216085.3	COX6B1	0.2305
IPI00015972.1	COX6C	0.36125
IPI00872879.1	COX7A2	0.25675
IPI00013968.1	COX7C	0.23275
IPI00027078.3	CPD	-0.24825
IPI00917016.1	CPNE1	-1.1015
IPI00024403.1	CPNE3	-2.16025
IPI00093057.6	CPOX	-0.29
IPI00026219.4	CPSF1	1.61225

AccsessionNo	GeneSymbol	hESCs/HFs
IPI00015808.3	GNL2	0.842
IPI00015833.1	CHCHD3	0.64425
IPI00015836.1	APPL1	-1.05025
IPI00015838.3	LYAR	1.66775
IPI00015842.1	RCN1	-1.80375
IPI00015865.6	ADPRHL2	-0.7685
IPI00015891.1	PFDN4	-0.5055
IPI00015894.1	CDC42EP4	-1.5885
IPI00015902.3	PDGFRB	-2.43
IPI00015911.2	DLD	0.4785
IPI00015924.1	TFIP11	1.262
IPI00015947.5	DNAJB1	0.0835
IPI00015953.3	DDX21	1.52275
IPI00015954.1	SAR1A	-1.7305
IPI00015955.5	EXOSC5	1.613
IPI00015972.1	COX6C	0.423
IPI00015973.1	EPB41L2	-0.61875
IPI00016006.2	GPHN	-1.2115
IPI00016112.6	PXDN	-2.04125
IPI00016179.1	S100A13	-2.27875
IPI00016250.6	FXR2	-1.15825
IPI00016255.4	PLBD1	2.17725
IPI00016334.2	MCAM	-2.80125
IPI00016339.4	RAB5C	-3.01875
IPI00016342.1	RAB7A	-0.4255
IPI00016372.1	RAB9A	0.8525
IPI00016373.3	RAB13	0.646
IPI00016405.1	OCIAD1	0.53
IPI00016513.5;P610	RAB10	-0.45725

AccessionNo	GeneSymbol	hiPSCs/hESCs
IPI00015838.3	LYAR	0.38375
IPI00015842.1	RCN1	0.21725
IPI00015865.6	ADPRHL2	0.14675
IPI00015891.1	PFDN4	0.485
IPI00015894.1	CDC42EP4	0.549
IPI00015902.3	PDGFRB	0.31175
IPI00015911.2	DLD	-0.1865
IPI00015924.1	TFIP11	0.20925
IPI00015947.5	DNAJB1	0.398
IPI00015953.3	DDX21	0.75575
IPI00015954.1	SAR1A	0.44125
IPI00015955.5	EXOSC5	0.258
IPI00015972.1	COX6C	0.2205
IPI00015973.1	EPB41L2	0.6795
IPI00016006.2	GPHN	0.67625
IPI00016112.6	PXDN	1.11975
IPI00016179.1	S100A13	0.36975
IPI00016250.6	FXR2	0.29775
IPI00016255.4	PLBD1	0.27
IPI00016334.2	MCAM	0.8655
IPI00016339.4	RAB5C	0.9265
IPI00016342.1	RAB7A	0.0555
IPI00016372.1	RAB9A	-0.59275
IPI00016373.3	RAB13	-0.137
IPI00016405.1	OCIAD1	0.14075
IPI00016513.5;P610	RAB10	0.21225
IPI00016532.3	C4orf27	-0.02425
IPI00016568.1	AK4	-0.13575
IPI00016605.3	C1orf123	0.47225

Table S4 The integrated datasets for the comparison of hiPSCs/HFs, hESCs/HFs, and hiPSCs/hESCs from the public datasets.

AccessionNo	GeneSymbol	hiPSCs/HFs	AccsessionNo	GeneSymbol	hESCs/HFs	AccessionNo	GeneSymbol	hiPSCs/hESCs
IPI00419531.2	CPSF2	1.525	IPI00016532.3	C4orf27	1.551	IPI00016610.2;P603	PCBP1	0.18475
IPI00946752.1	CPSF3	1.1945	IPI00016568.1	AK4	4.452	IPI00016634.1	C20orf11	0.7255
IPI00306882.4	CPSF3L	1.2085	IPI00016605.3	C1orf123	-0.20475	IPI00016639.7	PRKCI	0.5355
IPI00719106.1	CPSF7	1.2805	IPI00016610.2;P603	PCBP1	0.65575	IPI00016670.3	LAMTOR1	0.01525
IPI00012912.1	CPT2	0.90625	IPI00016634.1	C20orf11	-1.175	IPI00016703.2	DHCR24	0.945
IPI00219930.7	CRABP1	2.0785	IPI00016639.7	PRKCI	-0.3415	IPI00016725.2	GTF3C4	0.19775
IPI00216088.3	CRABP2	1.1125	IPI00016670.3	LAMTOR1	-0.3025	IPI00016736.1	PLCG1	0.9005
IPI00029140.7	CRAT	-2.51	IPI00016703.2	DHCR24	1.41525	IPI00016763.3	GTPBP6	0.15
IPI00021997.1	CREG1	0.8055	IPI00016725.2	GTF3C4	1.94975	IPI00016786.1	CDC42	0.28125
IPI00305469.4	CRK	-1.58675	IPI00016736.1	PLCG1	-0.22225	IPI00016801.1	GLUD1	0.16475
IPI00004839.1	CRKL	0.463	IPI00016763.3	GTPBP6	1.89925	IPI00016802.1	SIRT1	-0.10225
IPI00414123.4	CRMP1	2.9075	IPI00016786.1	CDC42	-1.5405	IPI00016832.1	PSMA1	0.49325
IPI00219317.3	CRNKL1	0.896	IPI00016801.1	GLUD1	-0.6775	IPI00016868.3	TELO2	0.3545
IPI00456492.2	CROCC	1.899	IPI00016802.1	SIRT1	1.94475	IPI00016912.1	TTC1	0.6125
IPI00000792.1	CRYZ	0.1275	IPI00016832.1	PSMA1	-0.849	IPI00017184.2	EHD1	0.94725
IPI00873244.1	CSDE1	-0.21425	IPI00016868.3	TELO2	0.3045	IPI00017283.2	IARS2	-0.171
IPI00219994.2	CSE1L	0.762	IPI00016912.1	TTC1	-0.559	IPI00017297.1	MATR3	0.1565
IPI00183400.8	CSNK1A1	-0.2885	IPI00017184.2	EHD1	-4.802	IPI00017303.1	MSH2	0.05825
IPI00020602.1	CSNK2A2	0.189	IPI00017283.2	IARS2	0.068	IPI00017334.1	PHB	0.06275
I00010865.1;P678	CSNK2B	0.263	IPI00017297.1	MATR3	1.7995	IPI00017341.3	SF3A2	0.05975
IPI00019157.2	CSPG4	-5.84575	IPI00017303.1	MSH2	2.80225	IPI00017342.1	RHOG	0.2625
IPI00442073.5	CSRP1	-4.052	IPI00017334.1	PHB	1.099	IPI00017344.3;P610	RAB5B	0.04725
IPI00002824.7	CSRP2	-0.1185	IPI00017341.3	SF3A2	1.013	IPI00017373.1	RPA3	0.103
IPI00021828.1	CSTB	-1.3765	IPI00017342.1	RHOG	-1.66575	IPI00017375.2	SEC23A	0.3085
IPI00011528.1	CSTF1	1.1135	IPI00017344.3;P610	RAB5B	-1.236	IPI00017376.2	SEC23B	0.419
IPI00909622.1	CSTF2	0.83925	IPI00017373.1	RPA3	1.7595	IPI00017412.1	RFC2	0.03875
IPI00550906.6	CSTF2T	1.82725	IPI00017375.2	SEC23A	-3.30925	IPI00017448.1	RPS21	0.35525
IPI00015195.1	CSTF3	1.2495	IPI00017376.2	SEC23B	0.6145	IPI00017451.1	SF3A1	0.22775
IPI00554626.3	CTBP1	-0.612	IPI00017412.1	RFC2	1.76975	IPI00017454.4	TUBA4B	0.56825

Table S4 The integrated datasets for the comparison of hiPSCs/HFs, hESCs/HFs, and hiPSCs/hESCs from the public datasets.

AccessionNo	GeneSymbol	hiPSCs/HFs
IPI00871437.1	CTDSPL2	2.77425
IPI00215948.4	CTNNA1	0.0805
IPI00844214.1	CTNNBL1	0.932
IPI00219870.1	CTNND1	0.18925
IPI00290142.5	CTPS	-0.41225
IPI00645702.1	CTPS2	1.50575
IPI00477468.1	CTR9	0.64175
IPI00021794.8	CTSA	-2.32875
IPI00909303.2	CTSB	-4.72725
IPI00022810.1	CTSC	1.7185
IPI00011229.1	CTSD	0.18725
IPI00012887.1	CTSL1	-2.0795
IPI00002745.1	CTSZ	-2.52275
IPI00029601.6	CTTN	-2.5385
IPI00514311.1	CTTNBP2NL	-1.15425
IPI00382458.1	CUL3	-0.059
IPI00300408.3	CUTC	2.379
IPI00009009.4	CWC15	0.96025
IPI00177381.8	CWC22	1.31725
IPI00107104.1	CXorf26	-0.03875
IPI00182933.5	CYB5A	0.5965
IPI00470674.5	CYB5R1	-2.75675
IPI00446235.2	CYB5R3	-2.4255
IPI00029264.3	CYC1	0.69825
IPI00917605.1	CYCS	1.26125
IPI00644231.3	CYFIP1	-1.688
IPI00719600.5	CYFIP2	2.5985
IPI00396661.3	CYP2S1	3.15825
IPI00009407.8	DAD1	-0.6625

AccsessionNo	GeneSymbol	hESCs/HFs
IPI00017448.1	RPS21	-0.54375
IPI00017451.1	SF3A1	0.986
IPI00017454.4	TUBA4B	-1.3105
IPI00017510.3	MT-CO2	0.273
IPI00017592.1	LETM1	-0.3395
IPI00017617.1	DDX5	0.84775
IPI00017672.4	PNP	0.60525
IPI00017704.3	COTL1	-3.0725
IPI00017726.1	HSD17B10	0.377
IPI00017855.1	ACO2	0.662
IPI00017895.3	GPD2	0.01925
IPI00017963.1;P623	SNRPD2	1.26875
IPI00018009.2	EDC3	-0.0815
IPI00018134.4	ARHGEF40	-0.005
IPI00018140.3	SYNCRIP	0.031
IPI00018146.1	YWHAQ	-1.16125
IPI00018196.5	NLE1	0.6775
IPI00018206.3	GOT2	1.1175
IPI00018235.3	PEF1	-1.36125
IPI00018236.2	GM2A	1.18425
IPI00018260.1	ARMC1	-0.15475
IPI00018274.1	EGFR	-4.66275
IPI00018288.1	POLR2C	0.78275
IPI00018342.5	AK1	-3.12225
IPI00018349.5	MCM4	1.562
IPI00018352.1	UCHL1	-1.006
IPI00018398.4	PSMC3	-0.50175
IPI00018402.1	TBCE	-0.25725
IPI00018465.1	CCT7	-0.649

AccessionNo	GeneSymbol	hiPSCs/hESCs
IPI00017510.3	MT-CO2	0.188
IPI00017592.1	LETM1	0.036
IPI00017617.1	DDX5	0.514
IPI00017672.4	PNP	0.78275
IPI00017704.3	COTL1	-0.66975
IPI00017726.1	HSD17B10	0.29125
IPI00017855.1	ACO2	-0.073
IPI00017895.3	GPD2	-0.04025
IPI00017963.1;P623	SNRPD2	0.26275
IPI00018009.2	EDC3	0.1005
IPI00018134.4	ARHGEF40	0.2435
IPI00018140.3	SYNCRIP	0.108
IPI00018146.1	YWHAQ	0.5345
IPI00018196.5	NLE1	0.59725
IPI00018206.3	GOT2	-0.1415
IPI00018235.3	PEF1	0.53925
IPI00018236.2	GM2A	0.16175
IPI00018260.1	ARMC1	0.348
IPI00018274.1	EGFR	-0.39125
IPI00018288.1	POLR2C	0.22575
IPI00018342.5	AK1	0.58125
IPI00018349.5	MCM4	0.32575
IPI00018352.1	UCHL1	0.267
IPI00018398.4	PSMC3	0.25225
IPI00018402.1	TBCE	0.53025
IPI00018465.1	CCT7	0.43475
IPI00018500.1	ARID3A	0.46725
IPI00018840.6	TRIP4	0.406
IPI00018855.1	ATP6V0C	0.636

Table S4 The integrated datasets for the comparison of hiPSCs/HFs, hESCs/HFs, and hiPSCs/hESCs from the public datasets.

AccessionNo	GeneSymbol	hiPSCs/HFs
IPI00028911.2	DAG1	-0.50675
IPI00909319.2	DAGLB	0.1625
IPI00551024.4	DAK	0.5555
IPI00910684.1	DAP3	0.33225
IPI00216951.2	DARS	0.01125
IPI00100460.2	DARS2	0.32025
IPI00335930.1	DAZAP1	1.37925
IPI00010182.4	DBI	-0.42575
IPI00003406.4	DBN1	-0.421
IPI00456925.3	DBNL	-2.6515
IPI00003944.1	DBT	1.0905
IPI00306642.3	DCAF13	1.45375
IPI00291417.2	DCAKD	0.99225
IPI00020454.1	DCK	-1.23
IPI00335385.4	DCPS	-0.507
IPI00916757.1	DCTN1	-2.23125
IPI00220503.9	DCTN2	-2.0505
IPI00012197.1	DCTPP1	1.4285
IPI00165361.3	DCUN1D5	0.771
IPI00797249.1	DCXR	-0.05275
IPI00220342.5	DDAH1	-1.60625
IPI00000760.1	DDAH2	-0.741
IPI00293464.5	DDB1	0.1185
IPI00796836.2	DDI2	0.437
IPI00297084.7	DDOST	-0.51275
IPI00028387.3	DDRGK1	-1.16425
IPI00293867.7	DDT	-0.148
IPI00293655.3	DDX1	-0.57925
IPI00297900.4	DDX10	2.02125

AccsessionNo	GeneSymbol	hESCs/HFs
IPI00018500.1	ARID3A	2.157
IPI00018840.6	TRIP4	-1.64625
IPI00018855.1	ATP6V0C	-0.62125
IPI00018873.1	NAMPT	0.21275
IPI00018878.5	DPPA4	4.43275
IPI00018914.2	PTPN14	-1.612
IPI00018924.3	HMG20A	1.105
IPI00018931.6	VPS35	-1.97475
IPI00018953.1	DPP4	-3.31125
IPI00018963.3	PARVA	-5.0125
IPI00019025.2	PARD6B	2.61375
IPI00019148.1	IGBP1	-1.64525
IPI00019157.2	CSPG4	-5.377
IPI00019169.3	SH3GL1	-2.2675
IPI00019195.2	RPP38	0.4415
IPI00019205.1	IRF2BPL	0.40775
IPI00019269.3	WDR61	0.0335
IPI00019329.1;P631	DYNLL1	-0.23175
IPI00019345.1;P628	RAP1A	-1.42875
IPI00019355.1	TSC22D1	-1.1135
IPI00019380.1	NCBP1	0.96275
IPI00019385.3	SSR4	-0.9815
IPI00019407.1	NSDHL	0.517
IPI00019472.4	SLC1A5	-0.806
IPI00019488.1	IMP3	1.63325
IPI00019502.3	MYH9	-3.59125
IPI00019600.3	UBE2V2	-0.5135
IPI00019640.1	VRK1	2.5465
IPI00019733.1	RAE1	0.77425

AccessionNo	GeneSymbol	hiPSCs/hESCs
IPI00018873.1	NAMPT	0.3225
IPI00018878.5	DPPA4	-0.331
IPI00018914.2	PTPN14	0.96775
IPI00018924.3	HMG20A	0.12175
IPI00018931.6	VPS35	0.473
IPI00018953.1	DPP4	-1.254
IPI00018963.3	PARVA	0.941
IPI00019025.2	PARD6B	0.1305
IPI00019148.1	IGBP1	0.4375
IPI00019157.2	CSPG4	0.546
IPI00019169.3	SH3GL1	0.7645
IPI00019195.2	RPP38	0.51
IPI00019205.1	IRF2BPL	-0.38925
IPI00019269.3	WDR61	-0.01
IPI00019329.1;P631	DYNLL1	0.31775
IPI00019345.1;P628	RAP1A	0.07525
IPI00019355.1	TSC22D1	1.053
IPI00019380.1	NCBP1	0.186
IPI00019385.3	SSR4	0.2095
IPI00019407.1	NSDHL	0.562
IPI00019472.4	SLC1A5	0.777
IPI00019488.1	IMP3	0.1125
IPI00019502.3	MYH9	0.49575
IPI00019600.3	UBE2V2	0.48075
IPI00019640.1	VRK1	0.397
IPI00019733.1	RAE1	0.00825
IPI00019755.3	GSTO1	1.25825
IPI00019770.3	FAU	0.46825
IPI00019812.1	PPP5C	0.58325

Table S4 The integrated datasets for the comparison of hiPSCs/HFs, hESCs/HFs, and hiPSCs/hESCs from the public datasets.

AccessionNo	GeneSymbol	hiPSCs/HFs
889541.2;IPI00023	DDX17	1.024
IPI00301323.1	DDX18	1.36025
IPI00019918.5	DDX19A	-0.40275
IPI00005904.3	DDX20	0.7465
IPI00015953.3	DDX21	2.0115
IPI00903057.1	DDX23	0.9755
IPI00006987.1	DDX24	0.964
IPI00293078.1	DDX27	1.121
940237.1;IPI00644	DDX39A	0.9895
IPI00215637.5	DDX3X	-0.447
IPI00007208.4	DDX41	1.16625
IPI00409671.3	DDX42	0.85725
IPI00329791.12	DDX46	0.49275
IPI00023972.5	DDX47	1.7555
IPI00017617.1	DDX5	1.10175
IPI00031554.1	DDX50	1.0055
IPI00879999.1	DDX54	0.57675
IPI00657777.4	DDX55	1.5085
IPI00295503.2	DDX58	-0.82025
IPI00514037.3	DDX59	1.00825
IPI00030320.4	DDX6	0.17
IPI00003482.1	DECR1	-2.1395
IPI00005721.1	DEFA1	-1.90075
IPI00021147.1	DEGS1	-1.2895
IPI00020021.3	DEK	1.52075
IPI00010882.3	DFFA	0.087
IPI00549380.1	DGCR8	1.418
IPI00016703.2	DHCR24	2.10125
IPI00294501.1	DHCR7	1.64675

AccsessionNo	GeneSymbol	hESCs/HFs
IPI00019755.3	GSTO1	-3.723
IPI00019770.3	FAU	-0.10125
IPI00019812.1	PPP5C	-0.49525
IPI00019884.1	ACTN2	-3.2125
IPI00019903.1	TACO1	0.35575
IPI00019904.2	ADD2	2.94375
IPI00019906.1	BSG	1.2905
IPI00019912.3	HSD17B4	1.888
IPI00019918.5	DDX19A	-0.455
IPI00019924.1	TBCC	-1.34025
IPI00019927.2	PSMD7	-0.57
IPI00019962.3	C4orf43	1.7595
IPI00019981.1	GTF2E2	1.38375
IPI00019994.4	TXLNG	2.88875
IPI00019996.3	SLTM	1.47625
IPI00019997.1	LIN7C	1.04975
IPI00020021.3	DEK	1.75725
IPI00020127.1	RPA1	1.70575
IPI00020128.2	UTP6	2.06025
IPI00020194.1	TAF15	0.7045
IPI00020332.1	ARG2	2.43425
IPI00020418.1	RRAS	-2.5855
IPI00020436.4	RAB11B	-0.2675
IPI00020454.1	DCK	-0.71125
IPI00020495.1	MRPS36	-0.3115
IPI00020510.1	CISD1	2.43925
IPI00020557.1	LRP1	-2.60675
IPI00020567.3	ARHGAP1	-2.5975
IPI00020599.1	CALR	-1.66475

AccessionNo	GeneSymbol	hiPSCs/hESCs
IPI00019884.1	ACTN2	0.5935
IPI00019903.1	TACO1	-0.07025
IPI00019904.2	ADD2	0.21775
IPI00019906.1	BSG	0.05675
IPI00019912.3	HSD17B4	-0.135
IPI00019918.5	DDX19A	0.352
IPI00019924.1	TBCC	-0.05975
IPI00019927.2	PSMD7	0.293
IPI00019962.3	C4orf43	0.0625
IPI00019981.1	GTF2E2	0.32125
IPI00019994.4	TXLNG	-0.23575
IPI00019996.3	SLTM	0.45775
IPI00019997.1	LIN7C	0.2225
IPI00020021.3	DEK	0.01275
IPI00020127.1	RPA1	-0.046
IPI00020128.2	UTP6	-0.15725
IPI00020194.1	TAF15	0.22675
IPI00020332.1	ARG2	1.07
IPI00020418.1	RRAS	-0.10575
IPI00020436.4	RAB11B	0.42575
IPI00020454.1	DCK	-0.13525
IPI00020495.1	MRPS36	-0.07475
IPI00020510.1	CISD1	-0.35575
IPI00020557.1	LRP1	0.2305
IPI00020567.3	ARHGAP1	0.60225
IPI00020599.1	CALR	0.4675
IPI00020602.1	CSNK2A2	0.499
IPI00020672.4	DPP3	0.76225
IPI00020719.2	MAVS	-0.0385

Table S4 The integrated datasets for the comparison of hiPSCs/HFs, hESCs/HFs, and hiPSCs/hESCs from the public datasets.

AccessionNo	GeneSymbol	hiPSCs/HFs
IPI00910072.1	DHDPSL	-0.7475
IPI00339238.1	DHRS7	-1.33725
IPI00550165.4	DHRS7B	0.137
IPI00396435.3	DHX15	1.2405
IPI00217413.3	DHX29	-1.1385
IPI00477295.4	DHX30	0.434
IPI00217760.2	DHX36	0.81875
IPI00217630.1	DHX37	1.413
IPI00294211.2	DHX38	-0.06225
IPI00888071.1	DHX40	1.49375
IPI00168885.5	DHX57	-0.094
IPI00031508.1	DHX8	1.003
IPI00844578.1	DHX9	1.37325
IPI00852685.1	DIAPH1	-0.202
IPI00514075.5	DIAPH2	-0.24175
IPI00219036.5	DICER1	0.52275
IPI00619921.3	DIDO1	0.9095
IPI00004290.3	DIEXF	0.755
IPI00004459.1	DIMT1	0.917
IPI00465045.2	DIP2B	-1.20325
IPI00015911.2	DLD	0.00225
IPI00552682.2	DLG1	0.15925
IPI00420108.5	DLST	-0.49475
IPI00294728.1	DMXL1	0.938
IPI00012535.1	DNAJA1	0.2435
IPI00032406.1	DNAJA2	-0.05975
IPI00015947.5	DNAJB1	0.16875
IPI00008454.1	DNAJB11	-1.2195
IPI00003848.1	DNAJB4	-4.86575

AccsessionNo	GeneSymbol	hESCs/HFs
IPI00020602.1	CSNK2A2	-0.0815
IPI00020672.4	DPP3	-0.935
IPI00020719.2	MAVS	-0.931
IPI00020906.1	IMPA1	-0.919
IPI00020928.1	TFAM	2.31725
IPI00020944.1	FDFT1	2.2795
IPI00020956.1	HDGF	-0.423
IPI00020985.4	EP300	1.762
IPI00020991.2	CDKN2AIP	0.057
IPI00021058.2	SLC4A7	1.4985
IPI00021129.5	AP3B1	-1.9335
IPI00021147.1	DEGS1	-1.04025
IPI00021187.4	RUVBL1	0.19175
IPI00021248.1	PLK1	3.18825
IPI00021263.3	YWHAZ	-1.27675
IPI00021264.1	CNN1	-2.65225
IPI00021266.1;P627	RPL23A	-0.39325
IPI00021267.2	EPHA2	-2.56825
IPI00021327.3	GRB2	-0.00225
IPI00021389.1	CCS	-0.45275
IPI00021405.3	LMNA	-4.88725
IPI00021417.3	SART1	1
IPI00021435.3	PSMC2	-0.672
IPI00021475.1	RAB33B	-0.6905
IPI00021700.3	PCNA	0.23475
IPI00021728.3	EIF2S2	-0.81875
IPI00021766.5	RTN4	-2.0495
IPI00021785.2	COX5B	-0.01475
IPI00021794.8	CTSA	-2.56325

AccessionNo	GeneSymbol	hiPSCs/hESCs
IPI00020906.1	IMPA1	0.72725
IPI00020928.1	TFAM	0.11775
IPI00020944.1	FDFT1	1.0805
IPI00020956.1	HDGF	0.328
IPI00020985.4	EP300	0.02075
IPI00020991.2	CDKN2AIP	0.33125
IPI00021058.2	SLC4A7	0.085
IPI00021129.5	AP3B1	0.5255
IPI00021147.1	DEGS1	0.07
IPI00021187.4	RUVBL1	0.26325
IPI00021248.1	PLK1	0.43925
IPI00021263.3	YWHAZ	0.529
IPI00021264.1	CNN1	0.34675
IPI00021266.1;P627	RPL23A	0.41525
IPI00021267.2	EPHA2	1.674
IPI00021327.3	GRB2	0.36475
IPI00021389.1	CCS	0.471
IPI00021405.3	LMNA	0.448
IPI00021417.3	SART1	0.2225
IPI00021435.3	PSMC2	0.279
IPI00021475.1	RAB33B	0.24175
IPI00021700.3	PCNA	0.8315
IPI00021728.3	EIF2S2	0.428
IPI00021766.5	RTN4	0.3235
IPI00021785.2	COX5B	0.2665
IPI00021794.8	CTSA	0.4495
IPI00021812.2	AHNAK	0.25975
IPI00021828.1	CSTB	0.44125
IPI00021831.1	PRKAR1A	0.276

Table S4 The integrated datasets for the comparison of hiPSCs/HFs, hESCs/HFs, and hiPSCs/hESCs from the public datasets.

AccessionNo	GeneSymbol	hiPSCs/HFs
IPI00074870.1	DNAJC1	-0.50975
IPI00307259.11	DNAJC13	-2.04775
IPI00830108.1	DNAJC2	0.148
IPI00006713.1	DNAJC3	-1.7225
IPI00329629.6	DNAJC7	-0.1685
IPI00003438.2	DNAJC8	0.124
IPI00922554.1	DNASE2	0.087
IPI00914615.1	DNM3	-1.6445
IPI00031519.3	DNMT1	0.8395
IPI00218357.1	DNMT3B	2.628
IPI00290410.3	DNTTIP2	1.20825
IPI00015286.2	DOCK1	-0.60125
IPI00940115.1	DOCK6	0.49075
IPI00816106.3	DOCK7	-1.87675
IPI00022018.1	DPM1	-0.47675
IPI00004454.3	DPM3	-0.1555
IPI00020672.4	DPP3	-0.397
IPI00018953.1	DPP4	-5.85
IPI00296141.4	DPP7	-3.72475
IPI00018878.5	DPPA4	3.60775
IPI00028109.1	DPY30	1.365
IPI00257508.4	DPYSL2	-0.866
IPI00029111.3	DPYSL3	-0.75975
IPI00003084.1	DRAP1	-0.823
IPI00031836.3	DRG1	0.258
IPI00916796.1	DSCR3	-2.071
IPI00028931.2	DSG2	1.81825
IPI00013933.2	DSP	2.80575
IPI00642259.2	DST	-2.783

AccsessionNo	GeneSymbol	hESCs/HFs
IPI00021812.2	AHNAK	-5.533
IPI00021828.1	CSTB	-1.4075
IPI00021831.1	PRKAR1A	-3.6195
IPI00021840.1;P627	RPS6	-0.5945
IPI00021842.1	APOE	3.23025
IPI00021924.1	H1FX	2.50775
IPI00021926.4;P62334;IPI0	PSMC6	-0.4235
IPI00021954.1	GBF1	-2.20225
IPI00021978.1	PEX11B	-1.244
IPI00021997.1	CREG1	-0.22275
IPI00022018.1	DPM1	-0.56525
IPI00022019.1	SAP30	3.14875
IPI00022033.6	RAB11FIP5	-3.38075
IPI00022082.7	8-Sep	-2.18375
IPI00022143.3	ESYT1	-1.36075
IPI00022145.6	NUCKS1	-0.49825
IPI00022215.1	ADNP	1.83375
IPI00022228.2	HDLBP	-2.2795
IPI00022239.7	METAP1	1.05025
IPI00022275.6	SACM1L	0.72575
IPI00022277.3	CCDC56	0.09125
IPI00022316.3	MRPS18B	0.38925
IPI00022334.1	OAT	1.478
IPI00022344.1	FLVCR1	3.1195
IPI00022373.2	NOB1	0.32875
IPI00022442.2	NDUFAB1	0.92625
IPI00022462.2	TFRC	-2.5715
IPI00022463.1	TF	1.61675
IPI00022542.1	ROCK1	-1.09725

AccessionNo	GeneSymbol	hiPSCs/hESCs
IPI00021840.1;P627	RPS6	0.537
IPI00021842.1	APOE	0.08175
IPI00021924.1	H1FX	-0.0875
IPI00021926.4;P62334;IPI0	PSMC6	0.18875
IPI00021954.1	GBF1	0.043
IPI00021978.1	PEX11B	-0.56575
IPI00021997.1	CREG1	1.36975
IPI00022018.1	DPM1	0.1685
IPI00022019.1	SAP30	-0.1705
IPI00022033.6	RAB11FIP5	-0.54375
IPI00022082.7	8-Sep	0.48625
IPI00022143.3	ESYT1	0.08525
IPI00022145.6	NUCKS1	0.268
IPI00022215.1	ADNP	0.35325
IPI00022228.2	HDLBP	0.5845
IPI00022239.7	METAP1	-0.21125
IPI00022275.6	SACM1L	-0.30425
IPI00022277.3	CCDC56	-0.12
IPI00022316.3	MRPS18B	0.358
IPI00022334.1	OAT	0.06825
IPI00022344.1	FLVCR1	1.198
IPI00022373.2	NOB1	0.65425
IPI00022442.2	NDUFAB1	-0.002
IPI00022462.2	TFRC	1.441
IPI00022463.1	TF	1.17675
IPI00022542.1	ROCK1	0.4375
IPI00022597.1;P610	UBE2M	0.363
IPI00022608.1	SORL1	0.1075
IPI00022613.4	NOP14	0.165

Table S4 The integrated datasets for the comparison of hiPSCs/HFs, hESCs/HFs, and hiPSCs/hESCs from the public datasets.

AccessionNo	GeneSymbol	hiPSCs/HFs
IPI00845388.1	DSTN	-2.06725
IPI00152692.2	DTD1	-0.43875
IPI00306353.3	DUSP23	0.125
IPI00013679.1	DUT	0.8155
IPI00023103.1	DVL2	-0.74175
IPI00456969.1	DYNC1H1	-1.4165
IPI00007675.6	DYNC1LI1	-1.0545
I00019329.1;P631	DYNLL1	-0.20375
IPI00642156.1	EARS2	0.98725
IPI00745955.4	EBNA1BP2	0.9735
IPI00216760.1	ECE1	-3.05625
IPI00011416.2	ECH1	1.38625
IPI00024993.4	ECHS1	-0.32075
IPI00419263.4	ECI2	-0.402
IPI00924582.1	ECT2	2.629
IPI00018009.2	EDC3	-0.286
IPI00399105.1	EDIL3	-0.52225
IPI00329536.2	EEA1	-2.8065
IPI00171248.1	EED	2.4355
IPI00396485.3	EEF1A1	-1.23625
IPI00023048.4	EEF1D	-1.65725
937615.2;IPI00000	EEF1G	-1.6115
IPI00186290.6	EEF2	-0.797
IPI00640276.1	EFHA1	0.19125
IPI00060181.1	EFHD2	-2.61775
IPI00917777.1	EFTUD2	1.167
IPI00018274.1	EGFR	-5.401
IPI00328822.1	EGLN1	-1.2085
IPI00433194.2	EHBP1	-2.03925

AccsessionNo	GeneSymbol	hESCs/HFs
IPI00022597.1;P610	UBE2M	-0.755
IPI00022608.1	SORL1	2.32375
IPI00022613.4	NOP14	1.23575
IPI00022648.2	EIF5	-1.0675
IPI00022664.3	RABGGTA	-1.87375
IPI00022694.3	PSMD4	-1.05625
IPI00022745.1	MVD	0.19525
IPI00022774.3;Q018	VCP	-1.06225
IPI00022790.2	MFAP1	1.53975
IPI00022793.5	HADHB	-0.5305
IPI00022810.1	CTSC	2.12725
IPI00022891.3	SLC25A4	1.87475
IPI00022977.1	CKB	3.4505
IPI00023048.4	EEF1D	-1.76975
IPI00023064.1	NDUFAF4	1.565
IPI00023086.3	MRPL15	0.3655
IPI00023095.1	MLF2	0.2275
IPI00023103.1	DVL2	-0.9535
IPI00023122.1	PDLIM7	-4.89325
IPI00023126.1	WDR46	1.4255
IPI00023234.3	UBA2	0.1035
IPI00023344.2	SYMPK	1.667
IPI00023406.1	HCCS	0.29625
IPI00023461.2	MLLT4	2.80025
IPI00023504.1	RAB3A	-1.68225
IPI00023542.6	TMED9	-0.8315
IPI00023591.1	PURA	-3.6635
IPI00023598.2;Q9D6	TUBB4	-1.649
IPI00023640.3	PDCD5	-1.1145

AccessionNo	GeneSymbol	hiPSCs/hESCs
IPI00022648.2	EIF5	0.57625
IPI00022664.3	RABGGTA	0.58675
IPI00022694.3	PSMD4	0.51975
IPI00022745.1	MVD	0.6035
IPI00022774.3;Q018	VCP	0.58875
IPI00022790.2	MFAP1	0.2465
IPI00022793.5	HADHB	-0.2785
IPI00022810.1	CTSC	-0.085
IPI00022891.3	SLC25A4	-0.24525
IPI00022977.1	CKB	0.28475
IPI00023048.4	EEF1D	0.2945
IPI00023064.1	NDUFAF4	-0.451
IPI00023086.3	MRPL15	0.26
IPI00023095.1	MLF2	0.21125
IPI00023103.1	DVL2	0.952
IPI00023122.1	PDLIM7	0.6925
IPI00023126.1	WDR46	0.094
IPI00023234.3	UBA2	0.226
IPI00023344.2	SYMPK	0.02575
IPI00023406.1	HCCS	0.349
IPI00023461.2	MLLT4	0.15175
IPI00023504.1	RAB3A	0.4005
IPI00023542.6	TMED9	-0.12725
IPI00023591.1	PURA	0.99225
IPI00023598.2;Q9D6	TUBB4	0.90875
IPI00023640.3	PDCD5	0.59125
IPI00023647.4	UBA6	0.36
IPI00023704.1	LPP	0.8365
IPI00023728.1	GGH	-0.17625

Table S4 The integrated datasets for the comparison of hiPSCs/HFs, hESCs/HFs, and hiPSCs/hESCs from the public datasets.

AccessionNo	GeneSymbol	hiPSCs/HFs
IPI00296421.2	EHBP1L1	-5.10975
IPI00017184.2	EHD1	-4.55075
IPI00100980.9	EHD2	-6.28825
IPI00005578.1	EHD4	-1.70875
IPI00220795.4	EHMT2	2.10425
IPI00298618.1	EIF1AD	0.456
00232533.5;Q8BM	EIF1AX	0.8545
IPI00456685.2	EIF2AK4	-1.19575
IPI00221300.2	EIF2B1	-0.80775
IPI00028083.1	EIF2B2	-0.89925
REV_IPI00925085.	EIF2B5	-1.322
IPI00745433.2	EIF2C2	0.618
IPI00219678.3	EIF2S1	-0.62525
IPI00021728.3	EIF2S2	-0.63475
IPI00297982.7	EIF2S3	-0.64275
IPI00029012.1	EIF3A	-0.5875
IPI00396370.6	EIF3B	-0.4235
IPI00006181.1	EIF3D	-0.44875
I00013068.1;P602	EIF3E	-0.38275
IPI00941255.1	EIF3F	-0.7365
IPI00290460.3	EIF3G	-0.62175
IPI00941359.1	EIF3H	-0.6225
IPI00012795.3	EIF3I	-0.5885
IPI00290461.3	EIF3J	-0.758
IPI00033143.1	EIF3K	-0.27275
IPI00102069.5	EIF3M	-0.302
I00025491.1;P608	EIF4A1	-0.677
IPI00009328.4	EIF4A3	1.2475
IPI00908588.1	EIF4B	-0.452

AccsessionNo	GeneSymbol	hESCs/HFs
IPI00023647.4	UBA6	-1.04525
IPI00023704.1	LPP	-3.19975
IPI00023728.1	GGH	0.62725
IPI00023736.2	CORO2A	2.6295
IPI00023748.3	NACA	-0.43775
IPI00023756.2	JARID2	3.56625
IPI00023832.5	SH3PXD2B	-0.487
IPI00023876.4	CASP6	-0.34625
IPI00023972.5	DDX47	1.71225
IPI00024012.4	FZD7	-1.44275
IPI00024024.4	AKAP17A	1.74775
IPI00024095.3	ANXA3	2.71125
IPI00024143.4	AAAS	2.10575
IPI00024157.1	FKBP3	0.2935
IPI00024163.1	POLR3A	1.025
IPI00024167.1	ESF1	2.115
IPI00024175.3	PSMA7	-0.76375
IPI00024214.1	TERF2	1.328
IPI00024255.2	GPKOW	-0.406
IPI00024282.1	RAB8B	-0.07025
IPI00024284.5	HSPG2	-3.593
IPI00024291.2	BAD	-2.561
IPI00024317.1	GCDH	1.82725
IPI00024320.1	RBM3	-2.16625
IPI00024403.1	CPNE3	-1.9045
IPI00024502.2	UBQLN4	1.236
IPI00024524.4	PNO1	0.84125
IPI00024623.3	ACADSB	-0.08375
IPI00024661.5	SEC24C	-1.664

AccessionNo	GeneSymbol	hiPSCs/hESCs
IPI00023736.2	CORO2A	0.005
IPI00023748.3	NACA	0.7685
IPI00023756.2	JARID2	0.31175
IPI00023832.5	SH3PXD2B	0.77925
IPI00023876.4	CASP6	0.19875
IPI00023972.5	DDX47	0.31
IPI00024012.4	FZD7	1.18775
IPI00024024.4	AKAP17A	0.18675
IPI00024095.3	ANXA3	0.97275
IPI00024143.4	AAAS	0.0205
IPI00024157.1	FKBP3	0.36075
IPI00024163.1	POLR3A	0.6945
IPI00024167.1	ESF1	0.07975
IPI00024175.3	PSMA7	0.62825
IPI00024214.1	TERF2	-0.2085
IPI00024255.2	GPKOW	0.79775
IPI00024282.1	RAB8B	0.10975
IPI00024284.5	HSPG2	0.7595
IPI00024291.2	BAD	0.09025
IPI00024317.1	GCDH	-0.03125
IPI00024320.1	RBM3	0.4475
IPI00024403.1	CPNE3	0.6565
IPI00024502.2	UBQLN4	0.09725
IPI00024524.4	PNO1	0.513
IPI00024623.3	ACADSB	0.30375
IPI00024661.5	SEC24C	0.27975
IPI00024662.1	CBX5	-0.26225
IPI00024821.1	PSMD14	0.161
IPI00024911.1	ERP29	0.46675

Table S4 The integrated datasets for the comparison of hiPSCs/HFs, hESCs/HFs, and hiPSCs/hESCs from the public datasets.

AccessionNo	GeneSymbol	hiPSCs/HFs
IPI00027485.3	EIF4E	0.18425
IPI00002570.1	EIF4EBP2	1.579
IPI00941589.1	EIF4G2	-0.5425
IPI00874215.2	EIF4G3	-0.9715
IPI00014263.1	EIF4H	-0.72125
IPI00022648.2	EIF5	-0.716
IPI00299254.4	EIF5B	-0.45025
IPI00010105.1	EIF6	-0.5795
IPI00396627.1	ELAC2	1.18225
940851.1;IPI00301	ELAVL1	1.08775
IPI00910626.1	ELOVL1	-2.89025
IPI00032003.1	EMD	-0.948
IPI00940934.1	EML4	0.12525
IPI00411623.3	ENAH	1.049
IPI00465248.5	ENO1	-0.39775
IPI00184311.4	ENPP1	-0.02575
IPI00791152.1	ENY2	0.96175
IPI00020985.4	EP300	1.33575
IPI00015973.1	EPB41L2	-0.2255
IPI00021267.2	EPHA2	-1.21175
IPI00009896.1	EPHX1	0.49075
IPI00397365.1	EPN1	-0.7765
IPI00010951.2	EPPK1	2.60275
IPI00013452.10	EPRS	-0.766
IPI00645613.1	EPS15L1	-1.22025
IPI00026512.2	ERAL1	1.14825
IPI00477831.2	ERAP1	-2.0105
IPI00845478.1	ERBB2	2.27675
953445.1;IPI00003	ERGIC1	-2.27025

AccsessionNo	GeneSymbol	hESCs/HFs
IPI00024662.1	CBX5	2.47225
IPI00024821.1	PSMD14	-0.534
IPI00024911.1	ERP29	0.69275
IPI00024920.1	ATP5D	0.4075
IPI00024933.3	RPL12	-0.47925
IPI00024934.4	MUT	0.4335
IPI00024971.1	OSBP	-0.73425
IPI00024976.5	TOMM22	0.54
IPI00024990.6	ALDH6A1	2.131
IPI00024993.4	ECHS1	0.08075
IPI00025019.3	PSMB1	-0.67075
IPI00025039.1	FBL	1.388
IPI00025049.1	M6PR	-0.061
IPI00025084.3	CAPNS1	-3.726
IPI00025086.4	COX5A	0.6075
IPI00025091.3;P622	RPS11	-0.5415
IPI00025156.4	STUB1	-1.5095
IPI00025160.2	VAC14	0.59325
IPI00025176.1	SMNDC1	1.2625
IPI00025178.3	BCAS2	1.409
IPI00025252.1	PDIA3	-0.3395
IPI00025273.1	GART	0.507
IPI00025277.5	PDCD6	-0.31825
IPI00025285.3	ATP6V1G1	-1.3065
IPI00025310.1	ZNF217	2.35875
IPI00025318.1	SH3BGRL	-1.96
IPI00025329.1;P840	RPL19	-0.64975
IPI00025333.4	CIAPIN1	0.22125
IPI00025344.1	NDUFS6	-0.43025

AccessionNo	GeneSymbol	hiPSCs/hESCs
IPI00024920.1	ATP5D	-0.216
IPI00024933.3	RPL12	0.26775
IPI00024934.4	MUT	-0.30975
IPI00024971.1	OSBP	0.141
IPI00024976.5	TOMM22	0.22
IPI00024990.6	ALDH6A1	-0.29125
IPI00024993.4	ECHS1	-0.098
IPI00025019.3	PSMB1	0.5285
IPI00025039.1	FBL	0.3025
IPI00025049.1	M6PR	0.0005
IPI00025084.3	CAPNS1	0.57475
IPI00025086.4	COX5A	0.117
IPI00025091.3;P622	RPS11	0.512
IPI00025156.4	STUB1	0.195
IPI00025160.2	VAC14	-0.06625
IPI00025176.1	SMNDC1	0.28525
IPI00025178.3	BCAS2	0.0845
IPI00025252.1	PDIA3	0.2535
IPI00025273.1	GART	0.45825
IPI00025277.5	PDCD6	0.0015
IPI00025285.3	ATP6V1G1	0.39825
IPI00025310.1	ZNF217	0.27925
IPI00025318.1	SH3BGRL	0.724
IPI00025329.1;P840	RPL19	0.4335
IPI00025333.4	CIAPIN1	0.6175
IPI00025344.1	NDUFS6	0.228
IPI00025491.1;P608	EIF4A1	0.77025
IPI00025512.2	HSPB1	0.49375
IPI00025721.3	COPS3	0.30525

Table S4 The integrated datasets for the comparison of hiPSCs/HFs, hESCs/HFs, and hiPSCs/hESCs from the public datasets.

AccessionNo	GeneSymbol	hiPSCs/HFs
IPI00029631.1	ERH	0.8625
IPI00936931.1	ERLIN1	-0.703
IPI00026942.5	ERLIN2	-0.31525
IPI00946242.1	ERMP1	1.60825
IPI00386755.2	ERO1L	-1.08425
IPI00024911.1	ERP29	0.9195
IPI00401264.5	ERP44	-1.06625
IPI00411706.1	ESD	-1.8975
IPI00024167.1	ESF1	1.932
IPI00022143.3	ESYT1	-1.5725
IPI00429191.3	ETF1	-0.74
IPI00004902.1	ETFB	-1.0305
IPI00003766.4	ETHE1	-4.00425
IPI00215762.7	EXOC1	-1.187
IPI00783559.1	EXOC2	-1.03575
IPI00953403.1	EXOC3	-1.37525
IPI00059279.5	EXOC4	-0.96225
IPI00829808.1	EXOC5	-1.0795
IPI00641168.1	EXOC7	-1.237
IPI00028264.3	EXOC8	-1.245
IPI00009464.1	EXOSC10	1.376
IPI00745613.2	EXOSC4	1.09325
IPI00015955.5	EXOSC5	1.644
IPI00073602.1	EXOSC6	1.31775
IPI00014198.2	EXOSC7	1.447
IPI00654592.3	EXOSC9	1.51025
IPI00843975.1	EZR	-0.03275
IPI00001754.1	F11R	3.20825
IPI00219684.3	FABP3	2.64775

AccsessionNo	GeneSymbol	hESCs/HFs
IPI00025491.1;P608	EIF4A1	-1.1395
IPI00025512.2	HSPB1	-4.967
IPI00025721.3	COPS3	-1.2565
IPI00025729.1	CAMLG	-0.05425
IPI00025796.3	NDUFS3	0.31525
IPI00025815.2	TARDBP	1.0925
IPI00025861.3	CDH1	2.352
IPI00025869.1	GLA	2.3215
IPI00025874.2	RPN1	-0.5645
IPI00025974.3	CHMP4B	-0.72775
IPI00026087.1	BANF1	1.2
IPI00026089.4	SF3B1	1.08025
IPI00026182.5	CAPZA2	-0.93175
IPI00026202.1	RPL18A	-0.5085
IPI00026215.1	FEN1	1.2145
IPI00026216.4	NPEPPS	-0.82275
IPI00026219.4	CPSF1	1.735
IPI00026230.1	HNRNPH2	0.3525
IPI00026259.1	AGA	-0.97725
IPI00026268.3;P628	GNB1	0.221
IPI00026271.5;P622	RPS14	-0.48475
IPI00026328.3	TXNDC12	0.347
IPI00026445.3	POLR1B	1.23625
IPI00026512.2	ERAL1	0.31475
IPI00026513.6	RPIA	0.17575
IPI00026516.1	OXCT1	-3.54225
IPI00026519.1	PPIF	0.248
IPI00026530.4	LMAN1	-1.004
IPI00026546.1	PAFAH1B2	-0.3185

AccessionNo	GeneSymbol	hiPSCs/hESCs
IPI00025729.1	CAMLG	0.01575
IPI00025796.3	NDUFS3	0.014
IPI00025815.2	TARDBP	0.10775
IPI00025861.3	CDH1	-0.29
IPI00025869.1	GLA	-0.371
IPI00025874.2	RPN1	0.166
IPI00025974.3	CHMP4B	0.33725
IPI00026087.1	BANF1	-0.04225
IPI00026089.4	SF3B1	0.1985
IPI00026182.5	CAPZA2	-0.0535
IPI00026202.1	RPL18A	0.317
IPI00026215.1	FEN1	0.4255
IPI00026216.4	NPEPPS	0.464
IPI00026219.4	CPSF1	0.1455
IPI00026230.1	HNRNPH2	-0.1
IPI00026259.1	AGA	-0.5335
IPI00026268.3;P628	GNB1	0.04575
IPI00026271.5;P622	RPS14	0.428
IPI00026328.3	TXNDC12	0.10225
IPI00026445.3	POLR1B	0.37975
IPI00026512.2	ERAL1	1.004
IPI00026513.6	RPIA	0.46825
IPI00026516.1	OXCT1	4.01525
IPI00026519.1	PPIF	-0.212
IPI00026530.4	LMAN1	-0.279
IPI00026546.1	PAFAH1B2	0.3175
IPI00026606.2	SURF6	0.05375
IPI00026627.4	RP2	0.48275
IPI00026781.3	FASN	0.892

Table S4 The integrated datasets for the comparison of hiPSCs/HFs, hESCs/HFs, and hiPSCs/hESCs from the public datasets.

AccessionNo	GeneSymbol	hiPSCs/HFs
IPI00007797.3	FABP5	2.0385
IPI00926423.2	FADS1	2.39975
IPI00183786.1	FADS2	1.918
IPI00070643.6	FAF1	-0.621
IPI00172656.6	FAF2	-0.9715
IPI00552360.2	FAHD1	-1.6855
IPI00101734.5	FAM105B	-0.58475
IPI00031670.6	FAM114A1	-6.028
IPI00002240.1	FAM118B	1.289
IPI00472054.2	FAM120A	-3.175
IPI00456750.2	FAM129B	-4.318
IPI00303722.5	FAM136A	2.29675
IPI00945585.1	FAM162A	2.182
IPI00166051.5	FAM177A1	-0.643
IPI00746221.2	FAM195B	-1.10325
IPI00334282.2	FAM3C	-0.07975
IPI00030098.5	FAM50A	-0.353
IPI00410079.3	FAM82A2	-0.38425
IPI00922140.1	FAM83D	-0.338
IPI00152671.7	FAM91A1	-1.947
IPI00007024.1	FAM96B	-0.5125
IPI00167572.4	FAM98B	0.0795
IPI00031820.3	FARSA	-1.127
IPI00300074.3	FARSB	-0.82625
IPI00026781.3	FASN	0.774
IPI00414973.1	FASTKD5	1.42425
IPI00019770.3	FAU	0.06925
IPI00025039.1	FBL	1.439
IPI00007087.4	FBXO2	1.012

AccsessionNo	GeneSymbol	hESCs/HFs
IPI00026606.2	SURF6	1.1615
IPI00026627.4	RP2	-1.17525
IPI00026781.3	FASN	0.14625
IPI00026824.2	HMOX2	-0.70825
IPI00026833.4	ADSS	-0.041
IPI00026848.3	LRPAP1	-1.41475
IPI00026942.5	ERLIN2	-0.0415
IPI00026958.4	FDXR	0.92375
IPI00026964.2	UQCRFS1	0.8075
IPI00026969.4	SEC23IP	-1.54425
IPI00026970.4	SUPT16H	1.654
IPI00027078.3	CPD	-0.36925
IPI00027107.5	TUFM	0.861
IPI00027180.1	ZMPSTE24	-0.894
IPI00027223.2	IDH1	1.21475
IPI00027228.1	PET112	1.143
IPI00027230.3	HSP90B1	-0.6615
IPI00027252.6;O351	PHB2	1.04025
IPI00027255.1	MYL6B	0.14275
IPI00027270.1;P612	RPL26	-0.43725
IPI00027341.1	CAPG	1.128
IPI00027378.5	UBXN1	-1.1895
IPI00027438.2	FLOT1	-0.35125
IPI00027442.4	AARS	-0.3285
IPI00027444.1	SERPINB1	-1.2485
IPI00027448.3	ATP5L	-0.283
IPI00027485.3	EIF4E	0.171
IPI00027493.1	SLC3A2	0.18925
IPI00027497.5	GPI	-0.23875

AccessionNo	GeneSymbol	hiPSCs/hESCs
IPI00026824.2	HMOX2	-0.00275
IPI00026833.4	ADSS	0.36425
IPI00026848.3	LRPAP1	0.50975
IPI00026942.5	ERLIN2	-0.05475
IPI00026958.4	FDXR	-0.078
IPI00026964.2	UQCRFS1	0.0065
IPI00026969.4	SEC23IP	0.37
IPI00026970.4	SUPT16H	0.028
IPI00027078.3	CPD	0.335
IPI00027107.5	TUFM	-0.27175
IPI00027180.1	ZMPSTE24	0.446
IPI00027223.2	IDH1	0.9405
IPI00027228.1	PET112	-0.01125
IPI00027230.3	HSP90B1	0.2615
IPI00027252.6;O351	PHB2	0.025
IPI00027255.1	MYL6B	0.27875
IPI00027270.1;P612	RPL26	0.47275
IPI00027341.1	CAPG	-1.06975
IPI00027378.5	UBXN1	0.563
IPI00027438.2	FLOT1	0.02275
IPI00027442.4	AARS	0.73825
IPI00027444.1	SERPINB1	0.575
IPI00027448.3	ATP5L	0.0135
IPI00027485.3	EIF4E	0.30725
IPI00027493.1	SLC3A2	1.47925
IPI00027497.5	GPI	0.18225
IPI00027626.3	CCT6A	0.4015
IPI00027699.2	PSMD10	-0.442
IPI00027705.2	PRIM2	0.539

Table S4 The integrated datasets for the comparison of hiPSCs/HFs, hESCs/HFs, and hiPSCs/hESCs from the public datasets.

AccessionNo	GeneSymbol	hiPSCs/HFs
IPI00020944.1	FDFT1	3.003
IPI00914971.1	FDPS	0.74275
IPI00026958.4	FDXR	0.434
IPI00026215.1	FEN1	1.3415
IPI00065435.3	FGD4	0.44275
IPI00930557.1	FGF2	0.90475
IPI00759715.1	FH	-0.63175
IPI00743342.2	FHL2	-4.65375
IPI00001730.3	FHOD1	-1.4405
IPI00219570.1	FIBP	0.28225
IPI00007052.6	FIS1	-1.50725
IPI00303300.3	FKBP10	-3.229
IPI00853400.1	FKBP15	-2.66975
IPI00873810.1	FKBP1A	-1.571
IPI00002535.2	FKBP2	-1.23825
IPI00024157.1	FKBP3	0.36975
IPI00219005.3	FKBP4	1.37125
IPI00218775.2	FKBP5	1.6725
IPI00942796.1	FKBP7	-4.40975
IPI00640341.1	FKBP8	0.76525
IPI00398214.2	FLAD1	0.742
IPI00031023.1	FLII	-1.94975
IPI00302592.2	FLNA	-2.181
IPI00289334.1	FLNB	-0.62075
IPI00027438.2	FLOT1	-0.6065
IPI00789008.1	FLOT2	-0.16075
IPI00022344.1	FLVCR1	3.87375
IPI00060521.1	FLYWCH2	-1.84225
IPI00015580.3	FNBP1L	2.485

AccsessionNo	GeneSymbol	hESCs/HFs
IPI00027626.3	CCT6A	-0.63275
IPI00027699.2	PSMD10	0.76575
IPI00027705.2	PRIM2	2.02525
IPI00027717.1	GEMIN4	0.0355
IPI00027808.1	POLR2B	0.69575
IPI00027834.3	HNRNPL	1.9155
IPI00027838.1	RBM4B	0.33375
IPI00028004.2	PSMB3	-0.80725
IPI00028005.1	NUP107	1.321
IPI00028006.1	PSMB2	-0.798
IPI00028055.4	TMED10	-0.54425
IPI00028083.1	EIF2B2	-0.7015
IPI00028091.3	ACTR3	-2.28225
IPI00028109.1	DPY30	1.58875
IPI00028122.1	PSIP1	4.21825
IPI00028264.3	EXOC8	-1.272
IPI00028266.1	CCNB2	2.7835
IPI00028277.8	FTO	-0.16025
IPI00028357.2	XPO4	0.946
IPI00028376.1	TIMM8A	1.07825
IPI00028387.3	DDRGK1	-0.803
IPI00028390.1	MRPL34	0.57925
IPI00028481.1	RAB8A	-0.97175
IPI00028491.4	AGPAT5	2.49925
IPI00028570.2	GSK3B	-0.79425
IPI00028908.3	NID2	-3.7525
IPI00028911.2	DAG1	-0.957
IPI00028931.2	DSG2	1.7115
IPI00028954.1	MCM3AP	0.78075

AccessionNo	GeneSymbol	hiPSCs/hESCs
IPI00027717.1	GEMIN4	0.313
IPI00027808.1	POLR2B	0.4245
IPI00027834.3	HNRNPL	0.0335
IPI00027838.1	RBM4B	0.6825
IPI00028004.2	PSMB3	0.63125
IPI00028005.1	NUP107	0.09575
IPI00028006.1	PSMB2	0.5805
IPI00028055.4	TMED10	-0.10425
IPI00028083.1	EIF2B2	0.04175
IPI00028091.3	ACTR3	0.48775
IPI00028109.1	DPY30	0.07525
IPI00028122.1	PSIP1	0.029
IPI00028264.3	EXOC8	0.31175
IPI00028266.1	CCNB2	0.39075
IPI00028277.8	FTO	0.3535
IPI00028357.2	XPO4	0.0745
IPI00028376.1	TIMM8A	0.07675
IPI00028387.3	DDRGK1	0.0665
IPI00028390.1	MRPL34	0.699
IPI00028481.1	RAB8A	0.2725
IPI00028491.4	AGPAT5	0.27275
IPI00028570.2	GSK3B	0.57575
IPI00028908.3	NID2	1.0545
IPI00028911.2	DAG1	0.7465
IPI00028931.2	DSG2	0.4655
IPI00028954.1	MCM3AP	0.583
IPI00028955.4	BOP1	0.521
IPI00029012.1	EIF3A	0.35875
IPI00029046.1	MLEC	-0.04175

Table S4 The integrated datasets for the comparison of hiPSCs/HFs, hESCs/HFs, and hiPSCs/hESCs from the public datasets.

AccessionNo	GeneSymbol	hiPSCs/HFs
IPI00170778.4	FNBP4	0.578
IPI00556645.3	FOXK1	-1.7785
IPI00220426.2	FOXK2	0.72975
IPI00943593.1	FRAS1	0.99825
IPI00004655.1	FRG1	0.3465
IPI00163187.10	FSCN1	-0.527
IPI00375676.8	FTL	-0.59175
IPI00028277.8	FTO	-0.19375
IPI00217686.3	FTSJ3	1.017
IPI00166153.3	FTSJD2	0.2645
IPI00853059.2	FUBP1	0.25975
IPI00375441.2	FUBP1	-0.0685
IPI00377261.1	FUBP3	0.34825
IPI00909890.1	FUS	0.7155
IPI00001538.3	FXC1	0.5375
IPI00946824.1	FXN	0.7565
IPI00016250.6	FXR2	-1.18775
IPI00001580.4	FYCO1	-3.395
IPI00024012.4	FZD7	-0.523
IPI00012442.1	G3BP1	0.055
IPI00179890.2	G3BP2	0.26675
IPI00289800.7	G6PD	-3.13825
IPI00293088.6	GAA	0.7375
IPI00299413.1	GABPA	0.20675
IPI00552587.1	GADD45GIP1	1.9555
IPI00298949.1	GAK	-0.71425
IPI00004669.1	GALNT2	-1.8375
IPI00328391.3	GALNT7	1.65475
IPI00011454.1	GANAB	1.38275

AccsessionNo	GeneSymbol	hESCs/HFs
IPI00028955.4	BOP1	0.4405
IPI00029012.1	EIF3A	-0.76225
IPI00029046.1	MLEC	0.533
IPI00029056.2	SEPHS1	2.156
IPI00029079.5	GMPS	0.4735
IPI00029111.3	DPYSL3	-0.438
IPI00029114.1	ICT1	0.1055
IPI00029133.4	ATP5F1	0.4765
IPI00029140.7	CRAT	-0.704
IPI00029162.3	CDK13	0.7275
IPI00029175.5	KIAA0196	-1.972
IPI00029264.3	CYC1	1.4165
IPI00029266.1	SNRPE	1.1765
IPI00029267.1	SNRPB2	1.297
IPI00029422.1	KIF20A	1.6225
IPI00029444.1	C12orf10	-0.52
IPI00029468.1;P611	ACTR1A	-2.27025
IPI00029515.3	PLEKHA5	1.198
IPI00029534.1	PPAT	2.8025
IPI00029561.1	NDUFA10	-0.2645
IPI00029601.6	CTTN	-2.7175
IPI00029623.1	PSMA6	-0.6695
IPI00029628.1	RCN2	0.24425
IPI00029629.3	TRIM25	-1.234
IPI00029631.1	ERH	0.87975
IPI00029730.1	STX4	-0.4635
IPI00029731.8	RPL35A	-0.6275
IPI00029741.1	ITGB5	-0.372
IPI00029764.1	SF3A3	1.10775

AccessionNo	GeneSymbol	hiPSCs/hESCs
IPI00029056.2	SEPHS1	-0.075
IPI00029079.5	GMPS	0.3885
IPI00029111.3	DPYSL3	0.02825
IPI00029114.1	ICT1	0.23175
IPI00029133.4	ATP5F1	-0.13725
IPI00029140.7	CRAT	-1.4385
IPI00029162.3	CDK13	0.456
IPI00029175.5	KIAA0196	0.6185
IPI00029264.3	CYC1	-0.454
IPI00029266.1	SNRPE	0.105
IPI00029267.1	SNRPB2	0.175
IPI00029422.1	KIF20A	0.40425
IPI00029444.1	C12orf10	0.4235
IPI00029468.1;P611	ACTR1A	0.6795
IPI00029515.3	PLEKHA5	0.14725
IPI00029534.1	PPAT	0.14075
IPI00029561.1	NDUFA10	0.26525
IPI00029601.6	CTTN	0.52675
IPI00029623.1	PSMA6	0.542
IPI00029628.1	RCN2	0.35925
IPI00029629.3	TRIM25	-0.31775
IPI00029631.1	ERH	0.19725
IPI00029730.1	STX4	-0.01125
IPI00029731.8	RPL35A	0.55975
IPI00029741.1	ITGB5	1.12125
IPI00029764.1	SF3A3	0.12925
IPI00029778.3	TP53BP1	0.49075
IPI00029997.1	PGLS	0.61975
IPI00030009.4	PAPSS2	0.62125

Table S4 The integrated datasets for the comparison of hiPSCs/HFs, hESCs/HFs, and hiPSCs/hESCs from the public datasets.

AccessionNo	GeneSymbol	hiPSCs/HFs
IPI00383581.4	GANAB	0.431
IPI00219018.7	GAPDH	-1.34375
IPI00946050.1	GAPVD1	-1.58075
IPI00607820.1	GAR1	1.12175
IPI00025273.1	GART	0.64975
IPI00103554.1	GATAD2B	0.937
IPI00021954.1	GBF1	-2.3525
IPI00024317.1	GCDH	1.4585
IPI00215768.3	GCLC	-0.1255
IPI00010090.1	GCLM	-2.27375
IPI00001159.10	GCN1L1	-0.9205
IPI00011604.1	GCSH	2.10325
940148.1;IPI00031	GDI2	-0.493
IPI00027717.1	GEMIN4	0.06975
IPI00291783.3	GEMIN5	0.3255
IPI00154473.5	GFM1	0.42375
IPI00299506.9	GFPT1	-1.0155
IPI00216159.14	GFPT2	2.2735
IPI00023728.1	GGH	0.11325
IPI00549970.1	GHITM	0.7445
IPI00916026.1	GIGYF2	-0.37125
IPI00218487.3	GJA1	2.8525
IPI00025869.1	GLA	1.69975
IPI00927191.1	GLB1	-0.6305
IPI00843789.2	GLDC	2.413
IPI00641153.3	GLG1	0.1205
IPI00220766.5	GLO1	0.35525
IPI00008552.6	GLRX3	-0.318
IPI00333763.7	GLRX5	1.28375

AccsessionNo	GeneSymbol	hESCs/HFs
IPI00029778.3	TP53BP1	-0.4955
IPI00029997.1	PGLS	-2.0405
IPI00030009.4	PAPSS2	-4.895
IPI00030098.5	FAM50A	-0.7905
IPI00030131.3	TMPO	1.3615
IPI00030179.3	RPL7P32	-0.5565
IPI00030243.1;P612	PSME3	-0.6655
IPI00030255.1	PLOD3	-2.74925
IPI00030275.5	TRAP1	2.071
IPI00030320.4	DDX6	0.39625
IPI00030356.4	MCL1	1.80975
IPI00030362.1	PLP2	-4.032
IPI00030363.1	ACAT1	-0.4175
IPI00030364.1	TAF10	0.64575
IPI00030383.1	PPP1R8	0.932
IPI00030431.1	ANTXR1	-1.9635
IPI00030578.2	VAT1L	0.6305
IPI00030706.1	AHSA1	-0.31125
IPI00030781.1	STAT1	-2.84725
IPI00030847.3	TM9SF3	-0.42575
IPI00030877.2	15-Sep	-0.32075
IPI00030919.3	LAMTOR3	-0.1485
IPI00030920.2	GMPPB	-2.036
IPI00030968.4	C9orf142	0.23125
IPI00031023.1	FLII	-1.669
IPI00031106.1	PSMG3	-0.176
IPI00031109.4	NDUFAF2	0.9885
IPI00031169.1	RAB2A	-1.288
IPI00031357.1	PPOX	1.6395

AccessionNo	GeneSymbol	hiPSCs/hESCs
IPI00030098.5	FAM50A	0.63275
IPI00030131.3	TMPO	0.7195
IPI00030179.3	RPL7P32	0.44
IPI00030243.1;P612	PSME3	0.8925
IPI00030255.1	PLOD3	2.73125
IPI00030275.5	TRAP1	-0.27275
IPI00030320.4	DDX6	0.076
IPI00030356.4	MCL1	0.56025
IPI00030362.1	PLP2	-1.88725
IPI00030363.1	ACAT1	-0.55025
IPI00030364.1	TAF10	0.286
IPI00030383.1	PPP1R8	0.03975
IPI00030431.1	ANTXR1	1.54475
IPI00030578.2	VAT1L	1.21825
IPI00030706.1	AHSA1	0.3345
IPI00030781.1	STAT1	0.5335
IPI00030847.3	TM9SF3	0.193
IPI00030877.2	15-Sep	0.78225
IPI00030919.3	LAMTOR3	-0.2205
IPI00030920.2	GMPPB	0.674
IPI00030968.4	C9orf142	-0.3175
IPI00031023.1	FLII	0.05925
IPI00031106.1	PSMG3	0.6355
IPI00031109.4	NDUFAF2	0.10675
IPI00031169.1	RAB2A	0.177
IPI00031357.1	PPOX	-0.30475
IPI00031370.3;Q9CW	TUBB2B	0.9535
IPI00031397.3	ACSL3	0.31575
IPI00031410.1	MTOR	-0.0355

Table S4 The integrated datasets for the comparison of hiPSCs/HFs, hESCs/HFs, and hiPSCs/hESCs from the public datasets.

AccessionNo	GeneSymbol	hiPSCs/HFs
IPI00215687.1	GLS	-2.13025
IPI00168262.2	GLT25D1	-2.00575
IPI00016801.1	GLUD1	-0.767
IPI00018236.2	GM2A	1.00675
IPI00549557.3	GMFB	-0.56625
IPI00101782.3	GMPPA	-2.3055
IPI00030920.2	GMPPB	-1.71325
IPI00009844.1	GMPR2	-0.86075
IPI00029079.5	GMPS	0.55125
IPI00290928.2	GNA13	0.12725
IPI00220578.3	GNAI3	0.906
IPI00288947.3	GNAQ	-0.2085
I00026268.3;P628	GNB1	-0.01125
I00003348.3;P628	GNB2	-0.769
I00848226.1;P680	GNB2L1	-0.182
IPI00012451.3	GNB4	-0.35125
IPI00221232.9	GNG12	-3.5065
IPI00384745.4	GNL1	-0.8345
IPI00015808.3	GNL2	1.20225
IPI00009305.1	GNPDA1	0.0735
IPI00012102.1	GNS	-1.82325
IPI00333637.1	GOLGA5	-0.92475
IPI00004671.2	GOLGB1	-0.99625
IPI00004962.2	GOLIM4	-2.5455
IPI00759659.1	GOLM1	1.34175
IPI00916299.1	GORASP2	-1.19575
IPI00219029.3	GOT1	-0.09925
IPI00018206.3	GOT2	0.712
IPI00829835.2	GPATCH4	1.16475

AccsessionNo	GeneSymbol	hESCs/HFs
IPI00031370.3;Q9CW	TUBB2B	3.8305
IPI00031397.3	ACSL3	0.1355
IPI00031410.1	MTOR	-0.30475
IPI00031420.3	UGDH	-2.14975
IPI00031479.1	PDIA5	-0.3925
IPI00031508.1	DHX8	0.82825
IPI00031517.1	MCM6	2.1355
IPI00031519.3	DNMT1	0.2005
IPI00031522.2	HADHA	-0.4565
IPI00031554.1	DDX50	1.3045
IPI00031570.1	NTPCR	1.1045
IPI00031615.3	LLPH	-0.204
IPI00031627.4	POLR2A	1.42475
IPI00031647.2	PDCD2L	1.2105
IPI00031661.1	NOC4L	1.4295
IPI00031670.6	FAM114A1	-5.518
IPI00031768.1	HOOK3	-4.17675
IPI00031804.1	VDAC3	0.048
IPI00031812.3	YBX1	-1.09225
IPI00031820.3	FARSA	-0.92475
IPI00031836.3	DRG1	0.19675
IPI00031982.1	NCKAP1	-1.29
IPI00032003.1	EMD	-0.50275
IPI00032139.1	SERPINB9	2.27225
IPI00032140.4	SERPINH1	-1.45275
IPI00032206.2	PDLIM4	-4.3525
IPI00032304.2	PLS1	1.78325
IPI00032406.1	DNAJA2	0.27375
IPI00032409.1	LAMTOR2	0.2045

AccessionNo	GeneSymbol	hiPSCs/hESCs
IPI00031420.3	UGDH	0.304
IPI00031479.1	PDIA5	-0.27625
IPI00031508.1	DHX8	0.43
IPI00031517.1	MCM6	0.07325
IPI00031519.3	DNMT1	0.92125
IPI00031522.2	HADHA	-0.192
IPI00031554.1	DDX50	-0.01025
IPI00031570.1	NTPCR	0.34325
IPI00031615.3	LLPH	0.802
IPI00031627.4	POLR2A	0.27425
IPI00031647.2	PDCD2L	0.1565
IPI00031661.1	NOC4L	0.01525
IPI00031670.6	FAM114A1	0.0535
IPI00031768.1	HOOK3	1.078
IPI00031804.1	VDAC3	0.3535
IPI00031812.3	YBX1	0.60825
IPI00031820.3	FARSA	0.187
IPI00031836.3	DRG1	0.3555
IPI00031982.1	NCKAP1	0.62175
IPI00032139.1	SERPINB9	2.09075
IPI00032140.4	SERPINH1	0.329
IPI00032206.2	PDLIM4	0.193
IPI00032304.2	PLS1	0.103
IPI00032406.1	DNAJA2	-0.0265
IPI00032409.1	LAMTOR2	-0.0465
IPI00032439.1	POLR1D	0.47425
IPI00032460.3;O359	LSM2	0.26125
IPI00032491.1	LEMD3	0.72075
IPI00032506.2	C14orf142	0.42775

Table S4 The integrated datasets for the comparison of hiPSCs/HFs, hESCs/HFs, and hiPSCs/hESCs from the public datasets.

AccessionNo	GeneSymbol	hiPSCs/HFs
IPI00015688.1	GPC1	-3.39925
IPI00017895.3	GPD2	-0.2995
IPI00016006.2	GPHN	-0.80675
IPI00027497.5	GPI	-0.44575
IPI00024255.2	GPKOW	0.1225
IPI00921612.1	GPN1	0.15525
IPI00921488.1	GPS1	-1.072
IPI00152432.2	GPT2	2.34125
IPI00927606.1	GPX1	0.76875
IPI00745335.7	GPX4	1.8095
IPI00021327.3	GRB2	0.10825
IPI00037448.3	GRHPR	-1.33875
IPI00644786.3	GRIPAP1	0.32425
IPI00292228.1	GSK3A	-0.36125
IPI00028570.2	GSK3B	-0.532
IPI00909083.1	GSPT1	-0.8555
IPI00010706.1	GSS	-1.86625
IPI00019755.3	GSTO1	-2.554
IPI00219757.13	GSTP1	0.3135
IPI00019981.1	GTF2E2	1.34925
IPI00477686.2	GTF2F2	-0.15625
IPI00426640.1	GTF2H2	0.105
IPI00293242.1	GTF2I	2.2815
IPI00807491.2	GTF3C1	2.46175
IPI00878252.1	GTF3C2	2.6285
IPI00015806.3	GTF3C3	2.31675
IPI00016725.2	GTF3C4	1.97725
IPI00010463.5	GTPBP1	0.84775
IPI00385042.4	GTPBP4	1.07325

AccsessionNo	GeneSymbol	hESCs/HFs
IPI00032439.1	POLR1D	0.4145
IPI00032460.3;O359	LSM2	1.1215
IPI00032491.1	LEMD3	0.12925
IPI00032506.2	C14orf142	-0.954
IPI00032516.5	AP1M1	-2.449
IPI00032533.3	WDR18	2.0215
IPI00032561.1	CAB39	-2.281
IPI00032597.2	RBMX2	1.67325
IPI00032827.1	SF3B14	1.134
IPI00032831.4	SNAP29	-2.87325
IPI00032849.2	NOP16	0.757
IPI00032851.1	COPZ1	-1.2545
IPI00032853.1;Q9CQ	NOP10	1.633
IPI00032872.3	MRPS16	0.86875
IPI00032881.2	MRPS23	0.7325
IPI00032903.3	PTRH2	0.55225
IPI00032905.2	VPS33B	-0.36925
IPI00032957.1	UBE2I	0.56575
IPI00032970.2	OSBPL11	-0.73525
IPI00033036.1	METAP2	-0.27975
IPI00033130.3	SAE1	0.26875
IPI00033143.1	EIF3K	-0.534
IPI00033153.1	NXF1	0.5155
IPI00033217.3	AASS	3.88125
IPI00033907.1	ANAPC1	0.245
IPI00034069.1	TFB2M	-0.0015
IPI00034159.1	ATP6V0D1	-1.22625
IPI00037448.3	GRHPR	-0.828
IPI00037619.4	RPL39P5	-0.39725

AccessionNo	GeneSymbol	hiPSCs/hESCs
IPI00032516.5	AP1M1	0.53325
IPI00032533.3	WDR18	-0.03325
IPI00032561.1	CAB39	1.58775
IPI00032597.2	RBMX2	0.37375
IPI00032827.1	SF3B14	0.13125
IPI00032831.4	SNAP29	0.86
IPI00032849.2	NOP16	0.609
IPI00032851.1	COPZ1	0.21
IPI00032853.1;Q9CQ	NOP10	0.098
IPI00032872.3	MRPS16	0.12725
IPI00032881.2	MRPS23	0.169
IPI00032903.3	PTRH2	0.08225
IPI00032905.2	VPS33B	0.37275
IPI00032957.1	UBE2I	0.3575
IPI00032970.2	OSBPL11	0.65325
IPI00033036.1	METAP2	0.066
IPI00033130.3	SAE1	0.27875
IPI00033143.1	EIF3K	0.53675
IPI00033153.1	NXF1	0.4545
IPI00033217.3	AASS	0.08225
IPI00033907.1	ANAPC1	0.47725
IPI00034069.1	TFB2M	0.29575
IPI00034159.1	ATP6V0D1	0.5665
IPI00037448.3	GRHPR	-0.03975
IPI00037619.4	RPL39P5	0.24275
IPI00043598.2	IKBIP	0.73225
IPI00044761.4	PUS7	0.003
IPI00045051.3	PURB	0.2215
IPI00045207.2	NACC1	0.16625

Table S4 The integrated datasets for the comparison of hiPSCs/HFs, hESCs/HFs, and hiPSCs/hESCs from the public datasets.

AccessionNo	GeneSymbol	hiPSCs/HFs
IPI00016763.3	GTPBP6	1.6225
IPI00871779.2	GTSE1	1.31675
IPI00794444.2	GYG1	-3.642
IPI00157144.2	GYS1	-1.65325
IPI00550239.4	H1F0	1.72275
IPI00021924.1	H1FX	2.17175
IPI00219037.5	H2AFX	0.732
IPI00744148.2	H2AFY	0.1825
IPI00220994.3	H2AFY2	1.8345
IPI00607861.2	H6PD	-3.152
IPI00909416.1	HACL1	2.615
IPI00294398.1	HADH	0.97625
IPI00031522.2	HADHA	-0.96025
IPI00022793.5	HADHB	-1.13725
IPI00745553.1	HAGH	-1.248
IPI00604587.1	HAX1	1.52525
IPI00935729.1	HBXIP	-0.31675
IPI00023406.1	HCCS	0.402
IPI00013774.1	HDAC1	0.60275
IPI00929375.1	HDAC2	1.84
IPI00020956.1	HDGF	-0.35025
IPI00885081.1	HDGFRP2	-0.225
IPI00007063.5	HDGFRP3	0.6765
IPI00022228.2	HDLBP	-2.02275
IPI00654611.2	HEATR2	0.1225
IPI00148063.1	HEBP1	-1.88875
IPI00871372.1	HECTD1	-1.04175
IPI00807597.1	HELLS	3.866
IPI00005826.1	HERC2	0.05125

AccsessionNo	GeneSymbol	hESCs/HFs
IPI00043598.2	IKBIP	-4.1005
IPI00044761.4	PUS7	1.834
IPI00045051.3	PURB	-1.55225
IPI00045207.2	NACC1	2.1115
IPI00045536.2	CHID1	0.845
IPI00045550.4	PPP1R9B	-2.4225
IPI00045914.1	SPEN	1.0915
IPI00045939.4	ADO	0.276
IPI00056357.3	C19orf10	-2.57625
IPI00056880.7	ZNF787	1.6
IPI00058192.1	POFUT1	-1.7555
IPI00059242.3	SYAP1	-1.12575
IPI00059279.5	EXOC4	-0.993
IPI00060031.3	ARL8A	0.122
IPI00060107.3	CHCHD1	0.43725
IPI00060181.1	EFHD2	-1.9
IPI00060521.1	FLYWCH2	-1.329
IPI00060627.2	CCDC124	-0.16825
IPI00061142.3	NUS1	-0.03175
IPI00061178.1	RBMXL1	2.30275
IPI00061245.4	MRPS10	0.46725
IPI00061403.4	YIPF6	0.21125
IPI00061531.4	MRPL53	0.16025
IPI00062040.1	WDR89	2.0125
IPI00062120.1	S100A16	-3.79725
IPI00063130.2	TMEM205	1.0225
IPI00063234.1	PRKAR2A	-1.194
IPI00063762.1	HPDL	2.572
IPI00063903.5	USMG5	0.4005

AccessionNo	GeneSymbol	hiPSCs/hESCs
IPI00045536.2	CHID1	0.191
IPI00045550.4	PPP1R9B	0.04075
IPI00045914.1	SPEN	0.2455
IPI00045939.4	ADO	0.2005
IPI00056334.5	PRKCDBP	-0.2815
IPI00056357.3	C19orf10	0.19475
IPI00056880.7	ZNF787	0.67575
IPI00058192.1	POFUT1	0.04575
IPI00059242.3	SYAP1	0.2515
IPI00059279.5	EXOC4	0.208
IPI00060031.3	ARL8A	-0.14275
IPI00060107.3	CHCHD1	0.2145
IPI00060181.1	EFHD2	-0.20375
IPI00060521.1	FLYWCH2	-0.103
IPI00060627.2	CCDC124	0.37825
IPI00061142.3	NUS1	0.86325
IPI00061178.1	RBMXL1	0.13525
IPI00061245.4	MRPS10	0.43675
IPI00061403.4	YIPF6	-0.233
IPI00061531.4	MRPL53	0.16975
IPI00062040.1	WDR89	-0.166
IPI00062120.1	S100A16	10.5625
IPI00063130.2	TMEM205	-0.151
IPI00063234.1	PRKAR2A	-0.139
IPI00063762.1	HPDL	0.31125
IPI00063903.5	USMG5	0.0505
IPI00064212.2	ZNF828	0.00925
IPI00065435.3	FGD4	0.34
IPI00065671.1	UCK2	1.00625

Table S4 The integrated datasets for the comparison of hiPSCs/HFs, hESCs/HFs, and hiPSCs/hESCs from the public datasets.

AccessionNo	GeneSymbol	hiPSCs/HFs
IPI00941167.1	HEXA	-1.191
IPI00012585.1	HEXB	-1.5485
IPI00939822.1	HGS	-1.70025
IPI00013860.3	HIBADH	-0.37575
IPI00419802.4	HIBCH	0.941
IPI00239077.5	HINT1	-0.87975
IPI00000335.1	HINT2	0.21775
IPI00170924.2	HINT3	-0.50425
IPI00217469.3	HIST1H1A	0.19825
IPI00217468.3	HIST1H1B	1.7995
IPI00217467.3	HIST1H1E	0.8435
IPI00216730.3	HIST2H2AB	1.947
I00453473.6;P628	HIST2H4B	1.53525
00166293.5;Q8CG	HIST3H2BB	1.258
IPI00219877.1	HMBS	0.083
IPI00018924.3	HMG20A	0.96
IPI00177716.7	HMGA1	1.28475
IPI00179700.3	HMGA1	1.66625
IPI00005996.1	HMGA2	-0.236
IPI00419258.4	HMGB1	0.4075
IPI00219097.4	HMGB2	0.816
IPI00008475.1	HMGCS1	3.23125
IPI00217950.5	HMGN2	0.2415
IPI00220484.3	HMGN4	1.13975
IPI00215893.8	HMOX1	-1.86175
IPI00026824.2	HMOX2	-1.0665
IPI00917313.1	HN1	-0.63725
IPI00011913.1	HNRNPA0	1.815
IPI00215965.2	HNRNPA1	1.02275

AccsessionNo	GeneSymbol	hESCs/HFs
IPI00064212.2	ZNF828	2.1495
IPI00065435.3	FGD4	0.40325
IPI00065671.1	UCK2	-0.458
IPI00070643.6	FAF1	-0.91575
IPI00071059.4	BAX	-0.8225
IPI00072377.1	SET	3.071
IPI00072541.4	PCID2	1.00525
IPI00073602.1	EXOSC6	1.41875
IPI00073779.1	MRPS35	0.597
IPI00074870.1	DNAJC1	-0.6985
IPI00090327.1	VPS45	-0.2595
IPI00093057.6	CPOX	-0.1655
IPI00099311.3	TRMT6	-0.4525
IPI00099463.2	SGPL1	0.699
IPI00099529.3	YME1L1	-0.3785
IPI00099996.2	RG9MTD1	1.63825
IPI00100160.3	CAND1	-0.4705
IPI00100460.2	DARS2	0.746
IPI00100656.3	TECR	0.69525
IPI00100775.1	C11orf67	0.0365
IPI00100796.4	CHMP5	-1.29025
IPI00100980.9	EHD2	-5.3965
IPI00101037.3	RCN3	-3.87725
IPI00101299.3	C1GALT1C1	0.70375
IPI00101664.2	NARS2	2.251
IPI00101734.5	FAM105B	-1.19675
IPI00101782.3	GMPPA	-2.991
IPI00101987.6	BABAM1	-0.2795
IPI00102069.5	EIF3M	-0.52525

AccessionNo	GeneSymbol	hiPSCs/hESCs
IPI00070643.6	FAF1	0.57825
IPI00071059.4	BAX	0.97025
IPI00072377.1	SET	-0.045
IPI00072541.4	PCID2	0.5275
IPI00073602.1	EXOSC6	0.14325
IPI00073779.1	MRPS35	0.39075
IPI00074870.1	DNAJC1	0.55025
IPI00090327.1	VPS45	0.05125
IPI00093057.6	CPOX	0.1445
IPI00099311.3	TRMT6	0.446
IPI00099463.2	SGPL1	0.5435
IPI00099529.3	YME1L1	0.62375
IPI00099996.2	RG9MTD1	0.07525
IPI00100160.3	CAND1	0.4665
IPI00100460.2	DARS2	-0.155
IPI00100656.3	TECR	-0.046
IPI00100775.1	C11orf67	-0.52725
IPI00100796.4	CHMP5	0.55875
IPI00100980.9	EHD2	-0.40375
IPI00101037.3	RCN3	-0.541
IPI00101299.3	C1GALT1C1	0.35925
IPI00101664.2	NARS2	-0.233
IPI00101734.5	FAM105B	0.90525
IPI00101782.3	GMPPA	0.75175
IPI00101987.6	BABAM1	0.6025
IPI00102069.5	EIF3M	0.46
IPI00102313.3	SRA1	0.23825
IPI00102815.1	NOC3L	0.57525
IPI00102904.1	AUH	-0.7895

Table S4 The integrated datasets for the comparison of hiPSCs/HFs, hESCs/HFs, and hiPSCs/hESCs from the public datasets.

AccessionNo	GeneSymbol	hiPSCs/HFs
IPI00396378.3	HNRNPA2B1	1.1565
IPI00927677.1	HNRNPA3	1.236
IPI00334713.1	HNRNPAB	1.7285
IPI00909232.1	HNRNPC	1.89325
IPI00220684.1	HNRNPD	1.188
IPI00003881.5	HNRNPF	1.0305
IPI00013881.6	HNRNPH1	0.973
IPI00026230.1	HNRNPH2	-0.0275
IPI00910458.1	HNRNPK	1.09575
IPI00027834.3	HNRNPL	1.71525
IPI00383296.5	HNRNPM	1.34825
IPI00012074.3	HNRNPR	0.95125
883857.2;IPI00644	HNRNPU	1.211
IPI00167147.1	HNRNPUL1	0.38425
IPI00456887.2	HNRNPUL2	0.30625
IPI00011274.3	HNRPDL	0.76075
IPI00745396.1	HNRPLL	0.58
IPI00031768.1	HOOK3	-3.4995
IPI00063762.1	HPDL	1.935
IPI00218493.7	HPRT1	0.087
IPI00300050.3	HSD11B2	3.529
IPI00017726.1	HSD17B10	0.4105
IPI00007676.3	HSD17B12	0.7395
IPI00019912.3	HSD17B4	1.44
IPI00784295.2	HSP90AA1	-0.29725
IPI00414676.6	HSP90AB1	0.5785
IPI00027230.3	HSP90B1	-0.66375
IPI00292499.4	HSPA14	0.56325
IPI00304925.5	HSPA1B	-0.813

AccsessionNo	GeneSymbol	hESCs/HFs
IPI00102313.3	SRA1	-1.37175
IPI00102815.1	NOC3L	0.405
IPI00102904.1	AUH	3.15075
IPI00103059.4	NAT14	0.1805
IPI00103142.1	NUDCD2	1.428
IPI00103341.3	TRUB1	0.4745
IPI00103380.1	ARAP3	1.17175
IPI00103419.4	SUGP1	0.79275
IPI00103467.4	ALDH1B1	2.66775
IPI00103525.1	PSPC1	2.221
IPI00103554.1	GATAD2B	0.9985
IPI00103654.2	PPP4R2	0.8365
IPI00103925.2	IRGQ	-1.6345
IPI00103940.4	MFSD10	0.22875
IPI00104050.3	THRAP3	1.411
IPI00104128.3	SEC11A	-0.28875
IPI00105598.3	PSMD11	-0.605
IPI00106491.3	MRTO4	0.6445
IPI00106495.1	NCAPG	0.4925
IPI00106567.2	WDR33	1.8565
IPI00106642.4	SDF2L1	-1.02575
IPI00106698.1	PELO	-0.70725
IPI00107104.1	CXorf26	-0.261
IPI00107745.3	LUC7L3	0.6855
IPI00140420.4	SND1	-0.786
IPI00141318.2	CKAP4	-3.4895
IPI00145260.3	IBA57	1.4825
IPI00145593.7	NOM1	1.59425
IPI00147874.1	NANS	-2.1545

AccessionNo	GeneSymbol	hiPSCs/hESCs
IPI00103059.4	NAT14	-0.014
IPI00103142.1	NUDCD2	-0.0985
IPI00103341.3	TRUB1	0.34075
IPI00103380.1	ARAP3	0.16525
IPI00103419.4	SUGP1	0.3015
IPI00103467.4	ALDH1B1	-1.22925
IPI00103525.1	PSPC1	0.03775
IPI00103554.1	GATAD2B	0.23825
IPI00103654.2	PPP4R2	0.261
IPI00103925.2	IRGQ	1.21975
IPI00103940.4	MFSD10	0.1895
IPI00104050.3	THRAP3	0.176
IPI00104128.3	SEC11A	0.222
IPI00105598.3	PSMD11	0.25875
IPI00106491.3	MRTO4	0.4975
IPI00106495.1	NCAPG	0.57475
IPI00106567.2	WDR33	0.048
IPI00106642.4	SDF2L1	0.466
IPI00106698.1	PELO	-0.00675
IPI00107104.1	CXorf26	0.475
IPI00107745.3	LUC7L3	0.311
IPI00140420.4	SND1	0.23975
IPI00141318.2	CKAP4	0.0265
IPI00145260.3	IBA57	-0.39675
IPI00145593.7	NOM1	0.47125
IPI00147874.1	NANS	0.4915
IPI00148063.1	HEBP1	0.0705
IPI00149650.3	PPWD1	0.28525
IPI00151462.3	MAP1LC3B2	0.7595

Table S4 The integrated datasets for the comparison of hiPSCs/HFs, hESCs/HFs, and hiPSCs/hESCs from the public datasets.

AccessionNo	GeneSymbol	hiPSCs/HFs
IPI00911039.1	HSPA1B	-0.82025
IPI00007702.1	HSPA2	-1.97825
IPI00002966.2	HSPA4	0.4645
I00003362.2;P200	HSPA5	-1.2085
IPI00003865.1	HSPA8	-0.5245
IPI00007765.5	HSPA9	0.82525
IPI00025512.2	HSPB1	-4.95025
IPI00784154.1	HSPD1	1.7865
IPI00220362.5	HSPE1	1.90425
IPI00024284.5	HSPG2	-3.15425
IPI00218993.1	HSPH1	-0.323
IPI00002335.1	HTT	-0.29825
IPI00445401.3	HUWE1	-0.132
IPI00000877.1	HYOU1	0.1385
IPI00644127.2	IARS	-0.43475
IPI00017283.2	IARS2	-0.35575
IPI00145260.3	IBA57	0.79125
IPI00029114.1	ICT1	0.093
IPI00027223.2	IDH1	1.81325
IPI00011107.2	IDH2	-0.44525
IPI00304417.7	IDH3B	-0.51775
IPI00019148.1	IGBP1	-1.56025
IPI00940809.1	IGF1R	2.08775
IPI00008557.5	IGF2BP1	3.05075
IPI00180983.1	IGF2BP2	0.07175
IPI00658000.3	IGF2BP3	1.52125
IPI00289819.4	IGF2R	-0.5285
IPI00186736.3	IGSF8	0.8005
IPI00043598.2	IKBIP	-3.6575

AccsessionNo	GeneSymbol	hESCs/HFs
IPI00148063.1	HEBP1	-1.701
IPI00149650.3	PPWD1	1.21875
IPI00151462.3	MAP1LC3B2	-2.12975
IPI00151988.4	ZNF532	4.04725
IPI00152007.1	ARHGEF17	-4.77
IPI00152083.3	C7orf11	1.07575
IPI00152377.1	STT3B	-0.48
IPI00152407.3	C18orf25	0.02525
IPI00152432.2	GPT2	2.1755
IPI00152671.7	FAM91A1	-1.60575
IPI00152692.2	DTD1	-0.16075
IPI00152708.3	UTP15	2.02925
IPI00152853.3	KIAA1949	-3.72625
IPI00152946.1	RACGAP1	0.86025
IPI00152998.3	LRRC40	0.66075
IPI00153032.1	LTV1	-0.9495
IPI00154162.5	LEO1	0.90875
IPI00154473.5	GFM1	0.51825
IPI00154528.3	SMC6	2.23
IPI00154590.6	MKI67IP	0.8495
IPI00155601.1	MACROD1	1.9615
IPI00156005.3	NOVA2	3.4085
IPI00156374.6	IPO4	-0.273
IPI00156689.3	VAT1	-0.88575
IPI00156793.5	XPC	-0.1705
IPI00156984.3	CHMP1B	1.081
IPI00157144.2	GYS1	-1.6125
IPI00157215.5	C19orf52	-0.05575
IPI00157442.1	ARHGEF11	-0.10675

AccessionNo	GeneSymbol	hiPSCs/hESCs
IPI00151988.4	ZNF532	0.105
IPI00152007.1	ARHGEF17	1.1845
IPI00152083.3	C7orf11	0.06175
IPI00152377.1	STT3B	0.4755
IPI00152407.3	C18orf25	0.628
IPI00152432.2	GPT2	0.2905
IPI00152671.7	FAM91A1	0.19875
IPI00152692.2	DTD1	0.05925
IPI00152708.3	UTP15	-0.127
IPI00152853.3	KIAA1949	0.45775
IPI00152946.1	RACGAP1	0.56375
IPI00152998.3	LRRC40	0.13175
IPI00153032.1	LTV1	1.1615
IPI00154162.5	LEO1	0.22475
IPI00154473.5	GFM1	0.1635
IPI00154528.3	SMC6	-0.1115
IPI00154590.6	MKI67IP	0.70475
IPI00155601.1	MACROD1	-1.23825
IPI00156005.3	NOVA2	-0.2135
IPI00156374.6	IPO4	0.5285
IPI00156689.3	VAT1	0.3915
IPI00156793.5	XPC	1.1935
IPI00156984.3	CHMP1B	0.06975
IPI00157144.2	GYS1	0.21975
IPI00157215.5	C19orf52	-0.0215
IPI00157442.1	ARHGEF11	0.03675
IPI00157790.7	KIAA0368	0.25825
IPI00157908.4	AIFM1	0.363
IPI00158296.6	KIAA0564	-0.045

Table S4 The integrated datasets for the comparison of hiPSCs/HFs, hESCs/HFs, and hiPSCs/hESCs from the public datasets.

AccessionNo	GeneSymbol	hiPSCs/HFs
IPI00797136.2	IKBIP	-4.355
IPI00293735.2	IKBKAP	-0.04225
00005198.2;Q9CX	ILF2	1.152
IPI00418313.3	ILF3	1.107
IPI00013219.1	ILK	-2.964
IPI00554541.2	ILVBL	-0.354
IPI00019488.1	IMP3	1.43725
IPI00020906.1	IMPA1	-0.35775
IPI00291510.3	IMPDH2	-0.305
IPI00001453.2	INA	-2.4965
IPI00759472.1	INCENP	2.12225
IPI00876931.1	INTS1	0.7555
IPI00477759.1	INTS2	0.76875
IPI00418336.3	INTS3	0.7925
IPI00156374.6	IPO4	-0.0865
IPI00793443.2	IPO5	-0.4905
IPI00007402.3	IPO7	-0.68125
IPI00185146.5	IPO9	0.17975
IPI00009342.1	IQGAP1	-2.283
IPI00645608.2	IRF2BP1	0.45625
IPI00019205.1	IRF2BPL	-0.4075
IPI00103925.2	IRGQ	-0.746
IPI00375631.6	ISG15	-2.112
IPI00884070.1	ISOC1	1.6655
IPI00552736.1	ISY1	0.919
IPI00176010.1	ITCH	-2.22675
IPI00013744.1	ITGA2	-4.7485
IPI00215995.1	ITGA3	-3.5285
IPI00306604.5	ITGA5	-3.15525

AccsessionNo	GeneSymbol	hESCs/HFs
IPI00157790.7	KIAA0368	-0.01875
IPI00157908.4	AIFM1	0.999
IPI00158296.6	KIAA0564	0.342
IPI00160421.3	MRPL18	-0.17975
IPI00163084.3	XAB2	1.05125
IPI00163100.1	UBXN6	-2.20025
IPI00163187.10	FSCN1	-1.2705
IPI00163381.4	LEPRE1	-2.63625
IPI00165092.3	YARS2	1.04575
IPI00165261.6	SCFD1	-0.65475
IPI00165361.3	DCUN1D5	0.7755
IPI00165506.4	POLDIP2	-0.148
IPI00166013.1	C20orf4	-0.30775
IPI00166051.5	FAM177A1	-0.1915
IPI00166137.5	RALYL	0.6115
IPI00166153.3	FTSJD2	0.24825
IPI00166293.5;Q8CG	HIST3H2BB	0.53825
IPI00166749.3	PMPCA	0.09925
IPI00166873.3	C9orf23	-0.59675
IPI00167074.4	ARL6IP6	1.0685
IPI00167147.1	HNRNPUL1	0.63725
IPI00167538.3	COX18	0.544
IPI00167572.4	FAM98B	0.46275
IPI00167941.1	MDN1	3.532
IPI00168262.2	GLT25D1	-2.01625
IPI00168388.1	SRP68	-0.71725
IPI00168407.3	PDPR	1.37225
IPI00168698.1	PDZD8	0.0345
IPI00168878.1	TOR1AIP2	-1.6275

AccessionNo	GeneSymbol	hiPSCs/hESCs
IPI00160421.3	MRPL18	0.07625
IPI00163084.3	XAB2	0.32525
IPI00163100.1	UBXN6	0.55425
IPI00163187.10	FSCN1	1.0755
IPI00163381.4	LEPRE1	0.55925
IPI00165092.3	YARS2	0.3795
IPI00165261.6	SCFD1	0.199
IPI00165361.3	DCUN1D5	0.3195
IPI00165506.4	POLDIP2	0.28675
IPI00166013.1	C20orf4	0.70875
IPI00166051.5	FAM177A1	-0.15725
IPI00166137.5	RALYL	0.48625
IPI00166153.3	FTSJD2	0.4385
IPI00166293.5;Q8CG	HIST3H2BB	1.617
IPI00166749.3	PMPCA	0.49175
IPI00166873.3	C9orf23	0.6225
IPI00167074.4	ARL6IP6	-0.11225
IPI00167147.1	HNRNPUL1	0.0905
IPI00167538.3	COX18	0.36425
IPI00167572.4	FAM98B	-0.02725
IPI00167941.1	MDN1	0.3205
IPI00168262.2	GLT25D1	0.2915
IPI00168388.1	SRP68	0.25325
IPI00168407.3	PDPR	-0.474
IPI00168698.1	PDZD8	-0.05175
IPI00168878.1	TOR1AIP2	0.532
IPI00168885.5	DHX57	0.6345
IPI00169285.6	PLBD2	0.135
IPI00169325.1	WDR36	-0.002

Table S4 The integrated datasets for the comparison of hiPSCs/HFs, hESCs/HFs, and hiPSCs/hESCs from the public datasets.

AccessionNo	GeneSymbol	hiPSCs/HFs
IPI00216221.2	ITGA6	0.25425
IPI00922108.1	ITGAV	-1.0765
217563.4;IPI00645	ITGB1	-2.84975
IPI00029741.1	ITGB5	0.539
IPI00645805.4	IVD	-0.121
IPI00784013.1	JAK1	-2.92775
IPI00023756.2	JARID2	3.51475
IPI00641392.4	JMJD1C	3.0745
IPI00896458.1	JMJD6	-1.7505
IPI00554711.3	JUP	2.71625
IPI00014238.2	KARS	-0.15
IPI00456631.5	KDM1A	2.34
IPI00479786.5	KHSRP	0.46325
IPI00791325.1	KIAA0020	0.9775
IPI00029175.5	KIAA0196	-1.603
IPI00749513.5	KIAA0319L	1.61425
IPI00157790.7	KIAA0368	-0.07575
IPI00004584.1	KIAA0391	1.9305
IPI00158296.6	KIAA0564	0.04125
IPI00939707.1	KIAA0664	-0.15
IPI00298991.9	KIAA1033	-1.52225
IPI00477355.3	KIAA1279	-0.6615
IPI00784901.1	KIAA1715	-3.1835
IPI00748360.2	KIAA1797	-1.14525
IPI00152853.3	KIAA1949	-3.64775
IPI00182757.10	KIAA1967	1.45025
IPI00305289.2	KIF11	2.45475
IPI00604711.3	KIF1A	2.39
IPI00029422.1	KIF20A	1.692

AccsessionNo	GeneSymbol	hESCs/HFs
IPI00168885.5	DHX57	-0.34175
IPI00169285.6	PLBD2	-0.70575
IPI00169325.1	WDR36	1.7245
IPI00169383.3	PGK1	0.909
IPI00170548.1	ATAD2	1.4135
IPI00170581.1	NUP50	1.39825
IPI00170596.1	SIN3A	2.55475
IPI00170692.4	VAPA	-0.742
IPI00170778.4	FNBP4	0.20175
IPI00170796.1	VPS29	-2.078
IPI00170924.2	HINT3	0.5335
IPI00170935.1	LRRC47	0.3465
IPI00171087.7	SRBD1	1.4005
IPI00171127.1	UBAP2	-0.1455
IPI00171199.5	PSMA3	-0.69025
IPI00171248.1	EED	1.8475
IPI00171390.2	PRPF38A	1.69625
IPI00171573.2	MCU	0.422
IPI00171626.3	LPCAT1	2.273
IPI00171665.1	NUP37	1.2965
IPI00171798.1	MTA2	-0.3895
IPI00171844.3	COPS4	-1.407
IPI00172591.5	MRPL17	0.0205
IPI00172656.6	FAF2	-0.70375
IPI00173346.3	PGM2L1	1.54525
IPI00175096.1	TTC9C	-1.32875
IPI00175136.5	RBM15B	1.46725
IPI00175146.5	ZFC3H1	2.56875
IPI00175169.3	ARFGAP1	-1.599

AccessionNo	GeneSymbol	hiPSCs/hESCs
IPI00169383.3	PGK1	-0.3215
IPI00170548.1	ATAD2	0.49975
IPI00170581.1	NUP50	0.33475
IPI00170596.1	SIN3A	0.08425
IPI00170692.4	VAPA	0.02
IPI00170778.4	FNBP4	0.59525
IPI00170796.1	VPS29	0.547
IPI00170924.2	HINT3	-0.62325
IPI00170935.1	LRRC47	-0.0485
IPI00171087.7	SRBD1	0.297
IPI00171127.1	UBAP2	0.922
IPI00171199.5	PSMA3	0.55675
IPI00171248.1	EED	0.81675
IPI00171390.2	PRPF38A	0.1815
IPI00171573.2	MCU	-0.301
IPI00171626.3	LPCAT1	-0.38525
IPI00171665.1	NUP37	0.05175
IPI00171798.1	MTA2	0.63925
IPI00171844.3	COPS4	0.377
IPI00172591.5	MRPL17	0.3475
IPI00172656.6	FAF2	0.05
IPI00173346.3	PGM2L1	0.5485
IPI00175096.1	TTC9C	0.374
IPI00175136.5	RBM15B	-0.146
IPI00175146.5	ZFC3H1	0.24
IPI00175169.3	ARFGAP1	0.632
IPI00176010.1	ITCH	0.384
IPI00176702.4	CAMSAP3	-0.231
IPI00176706.1	RBM17	0.3525

Table S4 The integrated datasets for the comparison of hiPSCs/HFs, hESCs/HFs, and hiPSCs/hESCs from the public datasets.

AccessionNo	GeneSymbol	hiPSCs/HFs
IPI00000769.2	KIF22	2.417
IPI00293884.6	KIF23	1.18525
IPI00867529.1	KIF2A	0.32975
IPI00290435.3	KIF2C	2.793
IPI00004533.1	KIF3B	-0.71375
IPI00178150.5	KIF4A	1.38175
IPI00012837.1	KIF5B	-2.103
IPI00306400.9	KIFC1	2.26125
IPI00921892.1	KIN	1.69125
IPI00002214.1	KPNA2	2.237
IPI00299033.1	KPNA3	-0.3085
IPI00012578.1	KPNA4	-1.10725
IPI00747764.3	KPNA6	-0.4935
IPI00001639.2	KPNB1	-0.386
IPI00423570.3	KRAS	-0.006
IPI00220327.4	KRT1	1.46325
IPI00554788.5	KRT18	3.747
00479145.2;;IPI00	KRT19	2.92075
00554648.3;;IPI00	KRT8	3.39
IPI00646281.1	L1CAM	1.73025
IPI00253050.2	L1TD1	3.1875
IPI00878749.1	LAGE3	0.115
IPI00375294.3	LAMA1	2.0865
IPI00783665.4	LAMA5	2.644
IPI00013976.3	LAMB1	-0.8155
IPI00296922.4	LAMB2	-1.4945
IPI00298281.4	LAMC1	-0.774
IPI00884105.2	LAMP1	-0.899
IPI00016670.3	LAMTOR1	-0.59275

AccsessionNo	GeneSymbol	hESCs/HFs
IPI00176010.1	ITCH	-2.36575
IPI00176702.4	CAMSAP3	3.3725
IPI00176706.1	RBM17	1.1075
IPI00177008.1	PGP	-0.0255
IPI00177381.8	CWC22	0.966
IPI00177642.8	CCDC9	-0.80625
IPI00177716.7	HMGA1	1.3195
IPI00177728.3	CNDP2	0.60775
IPI00177938.2	TLE3	2.14575
IPI00177940.2	RDH14	-0.38625
IPI00177965.5	NT5DC1	1.27175
IPI00178150.5	KIF4A	0.84525
IPI00178185.1	BICD2	-2.5825
IPI00178375.3	PPIP5K2	-0.6685
IPI00178440.3	SNORA41	-1.5205
IPI00178861.1	TRMT1	0.7705
IPI00179326.7	BAIAP2L1	1.92325
IPI00179330.6	RPS27A	-0.83275
IPI00179473.9	SQSTM1	-1.88975
IPI00179700.3	HMGA1	1.23825
IPI00179890.2	G3BP2	0.07875
IPI00179953.2	NASP	3.0185
IPI00179964.5	PTBP1	1.2485
IPI00180240.2	TMSL3	-0.52775
IPI00180454.4	UTP11L	1.702
IPI00180954.4	CIRBP	-0.23
IPI00180983.1	IGF2BP2	0.1325
IPI00181135.4	BCAT2	-0.0405
IPI00181359.5	SCAF11	2.00775

AccessionNo	GeneSymbol	hiPSCs/hESCs
IPI00177008.1	PGP	0.48625
IPI00177381.8	CWC22	0.75
IPI00177642.8	CCDC9	0.3135
IPI00177716.7	HMGA1	0.2515
IPI00177728.3	CNDP2	0.602
IPI00177938.2	TLE3	0.08325
IPI00177940.2	RDH14	0.3995
IPI00177965.5	NT5DC1	-0.0815
IPI00178150.5	KIF4A	0.984
IPI00178185.1	BICD2	0.34725
IPI00178375.3	PPIP5K2	0.0275
IPI00178440.3	SNORA41	0.316
IPI00178861.1	TRMT1	0.459
IPI00179326.7	BAIAP2L1	-0.0425
IPI00179330.6	RPS27A	0.41925
IPI00179473.9	SQSTM1	-0.4765
IPI00179700.3	HMGA1	0.86825
IPI00179890.2	G3BP2	0.37
IPI00179953.2	NASP	0.38475
IPI00179964.5	PTBP1	0.177
IPI00180240.2	TMSL3	0.115
IPI00180454.4	UTP11L	0.24075
IPI00180954.4	CIRBP	0.12575
IPI00180983.1	IGF2BP2	0.238
IPI00181135.4	BCAT2	0.25375
IPI00181359.5	SCAF11	0.62675
IPI00181728.1	BRIX1	0.3185
IPI00182289.6	RPS29	0.1465
IPI00182373.2	P4HA2	-0.28375

Table S4 The integrated datasets for the comparison of hiPSCs/HFs, hESCs/HFs, and hiPSCs/hESCs from the public datasets.

AccessionNo	GeneSymbol	hiPSCs/HFs
IPI00032409.1	LAMTOR2	-0.17525
IPI00030919.3	LAMTOR3	-0.638
IPI00789806.2	LAP3	-0.8495
IPI00185919.3	LARP1	-0.034
IPI00645869.1	LAS1L	1.59625
IPI00000861.1	LASP1	-2.98025
IPI00872246.1	LCMT1	0.35275
IPI00760574.1	LDB1	0.711
217966.9;IPI00217	LDHA	-1.66875
IPI00219217.3	LDHB	0.68175
IPI00032491.1	LEMD3	0.61625
IPI00154162.5	LEO1	0.83525
IPI00163381.4	LEPRE1	-2.31725
IPI00217055.1	LEPREL1	-3.79325
IPI00017592.1	LETM1	-0.538
IPI00219219.3	LGALS1	-6.27425
IPI00465431.8	LGALS3	-6.0735
IPI00384155.1	LGMN	1.24375
IPI00219841.6	LIG1	2.1115
IPI00008918.1	LIMA1	-2.5685
IPI00002948.3	LIN28A	2.02
IPI00019997.1	LIN7C	1.14375
IPI00791938.1	LLGL1	0.845
IPI00031615.3	LLPH	0.25525
IPI00026530.4	LMAN1	-1.5725
IPI00009950.1	LMAN2	-1.034
IPI00852693.1	LMF2	-2.94025
IPI00021405.3	LMNA	-4.76925
IPI00217975.4	LMNB1	1.95675

AccsessionNo	GeneSymbol	hESCs/HFs
IPI00181728.1	BRIX1	0.6895
IPI00182289.6	RPS29	-0.97125
IPI00182373.2	P4HA2	-3.7505
IPI00182533.5	RPL28	-0.58325
IPI00182728.2	VPS4B	-1.03825
IPI00182757.10	KIAA1967	1.60825
IPI00182933.5	CYB5A	1.24125
IPI00183400.8	CSNK1A1	-0.50025
IPI00183695.9	S100A10	-4.80925
IPI00183786.1	FADS2	0.0855
IPI00183938.6	TTC27	-0.43375
IPI00184311.4	ENPP1	0.362
IPI00184330.5	MCM2	2.18375
IPI00184546.4	C12orf23	-4.046
IPI00184821.1	COASY	-0.81425
IPI00185146.5	IPO9	-0.169
IPI00185374.4	PSMD12	-0.49
IPI00185533.7	SEH1L	1.41975
IPI00185859.2	MRPL45	0.0565
IPI00185919.3	LARP1	0.118
IPI00186194.5	CEP170	-2.08225
IPI00186290.6	EEF2	-1.01625
IPI00186429.8	CLPTM1L	-0.85875
IPI00186736.3	IGSF8	1.5315
IPI00187011.4	ZC3H4	0.838
IPI00215630.1	VCAN	0.347
IPI00215637.5	DDX3X	-0.5795
IPI00215687.1	GLS	-2.30775
IPI00215719.6	RPL18	-0.476

AccessionNo	GeneSymbol	hiPSCs/hESCs
IPI00182533.5	RPL28	0.531
IPI00182728.2	VPS4B	0.4155
IPI00182757.10	KIAA1967	0.0795
IPI00182933.5	CYB5A	-0.4175
IPI00183400.8	CSNK1A1	0.5245
IPI00183695.9	S100A10	1.0055
IPI00183786.1	FADS2	2.1495
IPI00183938.6	TTC27	0.5
IPI00184311.4	ENPP1	0.25925
IPI00184330.5	MCM2	0.01975
IPI00184546.4	C12orf23	0.4285
IPI00184821.1	COASY	0.407
IPI00185146.5	IPO9	0.53575
IPI00185374.4	PSMD12	0.24925
IPI00185533.7	SEH1L	0.01725
IPI00185859.2	MRPL45	0.4445
IPI00185919.3	LARP1	0.1425
IPI00186194.5	CEP170	0.28575
IPI00186290.6	EEF2	0.56475
IPI00186429.8	CLPTM1L	0.36575
IPI00186736.3	IGSF8	-0.4065
IPI00187011.4	ZC3H4	0.09225
IPI00215630.1	VCAN	1.86425
IPI00215637.5	DDX3X	0.41625
IPI00215687.1	GLS	0.4515
IPI00215719.6	RPL18	0.3725
IPI00215762.7	EXOC1	0.44
IPI00215768.3	GCLC	0.716
IPI00215777.1	SLC25A3	0.319

Table S4 The integrated datasets for the comparison of hiPSCs/HFs, hESCs/HFs, and hiPSCs/hESCs from the public datasets.

AccessionNo	GeneSymbol	hiPSCs/HFs
IPI00291802.3	LMO7	-5.46975
IPI00221241.3	LNPEP	-3.535
IPI00328306.9	LOC441155	2.265
IPI00745893.2	LOC728825	0.74775
IPI00924782.1	LOC729020	-1.7635
IPI00334291.9	LONP1	0.76475
IPI00294839.6	LOXL2	-3.668
IPI00171626.3	LPCAT1	1.6305
IPI00023704.1	LPP	-2.5795
IPI00945818.1	LRBA	2.1575
IPI00376144.3	LRCH1	-2.06925
IPI00020557.1	LRP1	-2.64175
IPI00026848.3	LRPAP1	-1.16175
IPI00783271.1	LRPPRC	1.07575
IPI00152998.3	LRRC40	0.545
IPI00170935.1	LRRC47	-0.1395
IPI00396321.1	LRRC59	-1.59175
IPI00002070.6	LRRC8A	-0.3365
IPI00300094.6	LSG1	0.44
IPI00004436.1	LSM1	-0.27175
I00032460.3;O359	LSM2	1.07975
IPI00219229.3	LSM3	1.08725
IPI00294955.3	LSM4	1.26225
I00001146.1;P623	LSM6	1.1985
IPI00007163.1	LSM7	1.29675
IPI00219077.4	LTA4H	0.2145
IPI00153032.1	LTV1	-0.046
IPI00893087.1	LUC7L2	0.0775
IPI00107745.3	LUC7L3	0.721

AccsessionNo	GeneSymbol	hESCs/HFs
IPI00215762.7	EXOC1	-1.387
IPI00215768.3	GCLC	-0.53475
IPI00215780.5	RPS19	-0.53875
IPI00215790.6	RPL38	-0.22625
IPI00215801.1	RBM39	1.39125
IPI00215815.3	AUP1	-0.264
IPI00215884.4;Q6PD	SRSF1	1.256
IPI00215888.4	SRP72	-0.53
IPI00215893.8	HMOX1	-2.926
IPI00215911.3	APEX1	0.6075
IPI00215920.8	ARF6	-1.33575
IPI00215928.4	CETN2	1.10225
IPI00215948.4	CTNNA1	-0.042
IPI00215965.2	HNRNPA1	0.973
IPI00215978.4	POLR3D	0.90525
IPI00215995.1	ITGA3	-3.14325
IPI00216048.9	PITPNA	-1.285
IPI00216057.5	SORD	0.12325
IPI00216085.3	COX6B1	0.28075
IPI00216088.3	CRABP2	1.08175
IPI00216138.6	TAGLN	-4.6525
IPI00216139.3	6-Sep	1.06175
IPI00216159.14	GFPT2	2.786
IPI00216192.3	CLPB	-0.07625
IPI00216221.2	ITGA6	0.24375
IPI00216230.3	TMPO	0.82775
IPI00216237.5	RPL36	-0.52475
IPI00216247.2	PSMD4	-1.92
IPI00216293.6	TST	0.64175

AccessionNo	GeneSymbol	hiPSCs/hESCs
IPI00215780.5	RPS19	0.3815
IPI00215790.6	RPL38	0.32
IPI00215801.1	RBM39	0.025
IPI00215815.3	AUP1	0.35175
IPI00215884.4;Q6PD	SRSF1	0.07525
IPI00215888.4	SRP72	0.24475
IPI00215893.8	HMOX1	1.31675
IPI00215911.3	APEX1	0.05675
IPI00215920.8	ARF6	0.19475
IPI00215928.4	CETN2	0.192
IPI00215948.4	CTNNA1	0.35675
IPI00215965.2	HNRNPA1	0.39825
IPI00215978.4	POLR3D	0.94825
IPI00215995.1	ITGA3	0.13375
IPI00216048.9	PITPNA	0.527
IPI00216057.5	SORD	0.6865
IPI00216085.3	COX6B1	0.1805
IPI00216088.3	CRABP2	0.2755
IPI00216138.6	TAGLN	1.51975
IPI00216139.3	6-Sep	0.166
IPI00216159.14	GFPT2	-0.06525
IPI00216192.3	CLPB	0.12625
IPI00216221.2	ITGA6	0.28475
IPI00216230.3	TMPO	0.27925
IPI00216237.5	RPL36	0.24875
IPI00216247.2	PSMD4	1.02275
IPI00216293.6	TST	-0.41375
IPI00216308.5	VDAC1	0.088
IPI00216313.7	VSNL1	-0.1395

Table S4 The integrated datasets for the comparison of hiPSCs/HFs, hESCs/HFs, and hiPSCs/hESCs from the public datasets.

AccessionNo	GeneSymbol	hiPSCs/HFs
IPI00015838.3	LYAR	1.828
IPI00398727.3	LYPLA1	0.8355
IPI00025049.1	M6PR	-0.38725
IPI00155601.1	MACROD1	0.41225
IPI00940535.1	MAD1L1	0.52625
IPI00009542.1	MAGED2	-0.16525
I00219306.1;P613	MAGOH	1.07625
IPI00646719.1	MAGT1	-0.622
IPI00332428.5	MAK16	1.10375
IPI00003802.2	MAN2A1	0.303
IPI00298793.4	MANBA	0.3615
IPI00924819.1	MANF	-0.5725
IPI00887765.1	MAP1A	-6.25575
IPI00008868.3	MAP1B	-2.11025
IPI00415014.3	MAP1LC3A	-3.22575
IPI00151462.3	MAP1LC3B2	-1.7075
IPI00296485.6	MAP1S	-0.04925
IPI00219604.3	MAP2K1	-0.63925
IPI00003783.1	MAP2K2	-1.43125
IPI00218857.1	MAP2K3	-2.891
IPI00003479.3	MAPK1	-2.0515
IPI00910350.1	MAPRE1	-0.279
IPI00219301.7	MARCKS	-0.64075
IPI00641181.5	MARCKSL1	2.68825
IPI00008240.2	MARS	0.0315
IPI00010157.1	MAT2A	-0.1505
IPI00017297.1	MATR3	1.69875
IPI00020719.2	MAVS	-1.16625
IPI00219929.1	MAX	0.62075

AccsessionNo	GeneSymbol	hESCs/HFs
IPI00216308.5	VDAC1	0.02525
IPI00216313.7	VSNL1	1.53225
IPI00216319.3	YWHAH	-0.601
IPI00216361.1	ZC3H7B	-2.00125
IPI00216393.1	CLTA	-0.94475
IPI00216470.1	PIP4K2B	-1.72225
IPI00216472.1	CLTB	-3.416
IPI00216529.1	ATP2B1	-0.95
IPI00216587.9;P622	RPS8	-0.537
IPI00216622.1	ACTL6A	1.09375
IPI00216682.5	CNN3	-1.0425
IPI00216691.5	PFN1	-1.49425
IPI00216695.1	BAZ1B	1.46325
IPI00216730.3	HIST2H2AB	1.90375
IPI00216760.1	ECE1	-3.547
IPI00216816.1	TOP3B	0.41975
IPI00216919.1	YAP1	-1.4445
IPI00216951.2	DARS	0.25775
IPI00216969.3	ABL1	-0.37025
IPI00216999.2	C14orf21	0.35025
IPI00217030.10;P627	RPS4X	-0.4215
IPI00217055.1	LEPREL1	-3.3255
IPI00217113.1	CLIP1	-3.252
IPI00217143.3	SDHA	0.7335
IPI00217223.1	PAICS	1.11375
IPI00217232.2	SUCLA2	-0.14325
IPI00217236.4	TBCA	-0.03475
IPI00217240.1	WDR75	1.99975
IPI00217272.7	AACS	-0.0215

AccessionNo	GeneSymbol	hiPSCs/hESCs
IPI00216319.3	YWHAH	0.45125
IPI00216361.1	ZC3H7B	0.34825
IPI00216393.1	CLTA	0.2115
IPI00216470.1	PIP4K2B	0.448
IPI00216472.1	CLTB	0.33025
IPI00216529.1	ATP2B1	0.8165
IPI00216587.9;P622	RPS8	0.4875
IPI00216622.1	ACTL6A	0.15775
IPI00216682.5	CNN3	0.58725
IPI00216691.5	PFN1	0.61175
IPI00216695.1	BAZ1B	0.8305
IPI00216730.3	HIST2H2AB	0.26525
IPI00216760.1	ECE1	0.61025
IPI00216816.1	TOP3B	0.61575
IPI00216919.1	YAP1	0.52125
IPI00216951.2	DARS	0.03625
IPI00216969.3	ABL1	0.138
IPI00216999.2	C14orf21	1.04175
IPI00217030.10;P627	RPS4X	0.369
IPI00217055.1	LEPREL1	0.05675
IPI00217113.1	CLIP1	0.53075
IPI00217143.3	SDHA	-0.662
IPI00217223.1	PAICS	0.35025
IPI00217232.2	SUCLA2	0.496
IPI00217236.4	TBCA	0.961
IPI00217240.1	WDR75	-0.098
IPI00217272.7	AACS	0.625
IPI00217386.1	POLR1C	0.3305
IPI00217413.3	DHX29	0.22175

Table S4 The integrated datasets for the comparison of hiPSCs/HFs, hESCs/HFs, and hiPSCs/hESCs from the public datasets.

AccessionNo	GeneSymbol	hiPSCs/HFs
IPI00439195.1	MBD3	2.364
IPI00016334.2	MCAM	-2.10725
IPI00789428.1	MCCC2	1.0915
IPI00030356.4	MCL1	2.13125
IPI00184330.5	MCM2	1.93775
940786.1;IPI00013	MCM3	2.07375
IPI00028954.1	MCM3AP	0.7245
IPI00018349.5	MCM4	1.58125
IPI00031517.1	MCM6	1.918
IPI00299904.3	MCM7	1.9085
IPI00552546.1	MCMBP	0.88125
IPI00171573.2	MCU	-0.179
IPI00916111.2	MDH1	-0.007
IPI00291006.2	MDH2	0.2585
IPI00167941.1	MDN1	3.43175
IPI00008215.1	ME1	-3.73575
IPI00011201.1	ME2	0.7165
IPI00829826.1	MED1	0.67625
IPI00000305.3	MED11	1.06375
IPI00297191.2	MED14	0.938
IPI00554734.1	MED15	0.081
IPI00909893.1	MED17	0.8615
IPI00935435.1	MED18	0.32175
IPI00645426.2	MED22	0.75475
IPI00942495.1	MED6	1.212
IPI00399089.4	MESDC2	-0.64175
IPI00022239.7	METAP1	0.55675
IPI00033036.1	METAP2	-0.4955
IPI00783001.1	METTL15	0.8645

AccsessionNo	GeneSymbol	hESCs/HFs
IPI00217386.1	POLR1C	0.55825
IPI00217413.3	DHX29	-0.9675
IPI00217467.3	HIST1H1E	-0.26975
IPI00217468.3	HIST1H1B	1.3165
IPI00217469.3	HIST1H1A	2.7875
IPI00217519.3	RALA	0.4815
IPI00217553.1	MRPL41	-0.061
IPI00217563.4;IPI00645	ITGB1	-3.295
IPI00217630.1	DHX37	0.89675
IPI00217661.4	RAVER1	1.49125
IPI00217686.3	FTSJ3	0.82825
IPI00217709.1	TOP2B	1.63125
IPI00217760.2	DHX36	0.66625
IPI00217766.3	SCARB2	-1.9345
IPI00217816.3	UAP1	-3.99975
IPI00217862.5	RRP9	1.20775
IPI00217871.4	ALDH4A1	1.43375
IPI00217874.2	RCOR2	2.748
IPI00217920.7	ALDH16A1	0.10125
IPI00217949.12	UBE2S	1.13375
IPI00217950.5	HMGN2	0.256
IPI00217966.9;IPI00217	LDHA	-1.08825
IPI00217975.4	LMNB1	1.64225
IPI00218200.8	BCAP31	-0.536
IPI00218207.1	SPTBN2	0.61175
IPI00218236.6;P621	PPP1CB	-0.656
IPI00218342.10	MTHFD1	-0.33825
IPI00218343.4	TUBA1C	-1.4305
IPI00218353.3	ATP13A1	-0.49775

AccessionNo	GeneSymbol	hiPSCs/hESCs
IPI00217467.3	HIST1H1E	1.54125
IPI00217468.3	HIST1H1B	0.9765
IPI00217469.3	HIST1H1A	-1.452
IPI00217519.3	RALA	-0.11
IPI00217553.1	MRPL41	0.3915
IPI00217563.4;IPI00645	ITGB1	0.72075
IPI00217630.1	DHX37	0.6965
IPI00217661.4	RAVER1	0.10225
IPI00217686.3	FTSJ3	0.5035
IPI00217709.1	TOP2B	0.511
IPI00217760.2	DHX36	0.32775
IPI00217766.3	SCARB2	-0.15925
IPI00217816.3	UAP1	-0.35625
IPI00217862.5	RRP9	0.24425
IPI00217871.4	ALDH4A1	0.06125
IPI00217874.2	RCOR2	0.399
IPI00217920.7	ALDH16A1	0.13075
IPI00217949.12	UBE2S	1.082
IPI00217950.5	HMGN2	0.26325
IPI00217966.9;IPI00217	LDHA	-0.0975
IPI00217975.4	LMNB1	0.542
IPI00218200.8	BCAP31	0.21025
IPI00218207.1	SPTBN2	0.66475
IPI00218236.6;P621	PPP1CB	-0.03725
IPI00218342.10	MTHFD1	1.1855
IPI00218343.4	TUBA1C	0.743
IPI00218353.3	ATP13A1	0.00075
IPI00218357.1	DNMT3B	0.57375
IPI00218398.5	MMP14	0.79525

Table S4 The integrated datasets for the comparison of hiPSCs/HFs, hESCs/HFs, and hiPSCs/hESCs from the public datasets.

AccessionNo	GeneSymbol	hiPSCs/HFs
IPI00009755.3	METTL3	0.67275
IPI00022790.2	MFAP1	1.516
IPI00002236.3	MFGE8	-0.165
IPI00642329.2	MFN2	0.14125
IPI00103940.4	MFSD10	0.14525
IPI00000138.1	MGAT1	-2.004
IPI00009730.1	MID1IP1	1.6325
IPI00293276.10	MIF	0.344
IPI00293748.3	MINPP1	-0.62125
IPI00241860.4	MIPEP	0.3675
I00328328.3;P106	MIR1248	-0.01775
IPI00004233.2	MKI67	3.71425
IPI00154590.6	MKI67IP	1.0535
IPI00927374.1	MKRN2	-1.84775
IPI00029046.1	MLEC	0.211
IPI00002828.3	MLF1IP	2.4905
IPI00023095.1	MLF2	0.1875
IPI00218500.6	MLL	1.55725
IPI00023461.2	MLLT4	2.6515
IPI00218398.5	MMP14	-2.2165
IPI00294701.1	MNAT1	0.003
IPI00930346.1	MORF4L1	1.98125
IPI00556107.1	MPG	0.7505
IPI00012149.1	MPHOSPH10	1.44625
IPI00910583.1	MPP2	2.3015
IPI00005707.6	MRC2	-4.417
IPI00940798.1	MRE11A	0.57675
IPI00549381.5	MRPL1	0.25675
IPI00418290.1	MRPL14	0.25275

AccsessionNo	GeneSymbol	hESCs/HFs
IPI00218357.1	DNMT3B	2.9975
IPI00218398.5	MMP14	-2.71225
IPI00218435.1	PRPF4	1.14
IPI00218465.10	PLAA	-0.66925
IPI00218487.3	GJA1	2.787
IPI00218493.7	HPRT1	-0.37075
IPI00218500.6	MLL	1.5
IPI00218606.7;P622	RPS23	-0.5255
IPI00218638.10	MYO1F	-0.6055
IPI00218682.1	P4HA1	-0.1695
IPI00218728.4	PAFAH1B1	-1.228
IPI00218733.6	SOD1	-0.64775
IPI00218775.2	FKBP5	1.5195
IPI00218848.5	ATP5I	0.4395
IPI00218857.1	MAP2K3	-3.315
IPI00218918.5	ANXA1	-4.17325
IPI00218922.5	SEC63	0.0065
IPI00218924.5	CHP	-0.7135
IPI00218971.4	PDP1	0.32475
IPI00218993.1	HSPH1	-0.76925
IPI00219005.3	FKBP4	1.339
IPI00219006.5	RPS19BP1	1.44975
IPI00219018.7	GAPDH	-1.36725
IPI00219029.3	GOT1	-0.57375
IPI00219034.3	NDUFA8	0.49225
IPI00219036.5	DICER1	0.251
IPI00219037.5	H2AFX	0.87775
IPI00219077.4	LTA4H	0.0375
IPI00219078.5	ATP2A2	-0.69

AccessionNo	GeneSymbol	hiPSCs/hESCs
IPI00218435.1	PRPF4	0.039
IPI00218465.10	PLAA	0.32075
IPI00218487.3	GJA1	0.3435
IPI00218493.7	HPRT1	0.80075
IPI00218500.6	MLL	0.217
IPI00218606.7;P622	RPS23	0.493
IPI00218638.10	MYO1F	0.50325
IPI00218682.1	P4HA1	-0.56225
IPI00218728.4	PAFAH1B1	0.37825
IPI00218733.6	SOD1	0.162
IPI00218775.2	FKBP5	0.46575
IPI00218848.5	ATP5I	-0.142
IPI00218857.1	MAP2K3	0.70925
IPI00218918.5	ANXA1	0.22375
IPI00218922.5	SEC63	-0.09725
IPI00218924.5	CHP	0.45975
IPI00218971.4	PDP1	-0.4295
IPI00218993.1	HSPH1	0.71925
IPI00219005.3	FKBP4	0.32175
IPI00219006.5	RPS19BP1	0.20175
IPI00219018.7	GAPDH	0.3665
IPI00219029.3	GOT1	0.73775
IPI00219034.3	NDUFA8	-0.236
IPI00219036.5	DICER1	0.4685
IPI00219037.5	H2AFX	0.17125
IPI00219077.4	LTA4H	0.39425
IPI00219078.5	ATP2A2	0.15075
IPI00219097.4	HMGB2	0.29575
IPI00219155.5;P613	RPL27	0.4435

Table S4 The integrated datasets for the comparison of hiPSCs/HFs, hESCs/HFs, and hiPSCs/hESCs from the public datasets.

AccessionNo	GeneSymbol	hiPSCs/HFs
IPI00023086.3	MRPL15	0.3585
IPI00000821.2	MRPL16	0.16275
IPI00172591.5	MRPL17	0.09325
IPI00160421.3	MRPL18	-0.52425
IPI00013706.5	MRPL20	-0.29025
IPI00293476.3	MRPL23	0.21175
IPI00790114.1	MRPL27	0.43275
IPI00006864.3	MRPL33	0.03575
IPI00028390.1	MRPL34	0.844
IPI00295427.4	MRPL39	0.41825
IPI00217553.1	MRPL41	0.06725
IPI00428288.1	MRPL43	0.496
IPI00009680.3	MRPL44	0.3525
IPI00185859.2	MRPL45	0.24025
IPI00797533.1	MRPL46	-0.1155
IPI00794978.1	MRPL47	0.1915
IPI00939308.2	MRPL48	-0.3165
IPI00329036.2	MRPL50	0.30025
IPI00607627.3	MRPL51	0.144
IPI00061531.4	MRPL53	0.1005
IPI00061245.4	MRPS10	0.617
IPI00005050.1	MRPS14	0.5635
IPI00550037.3	MRPS15	0.3985
IPI00032872.3	MRPS16	0.67325
IPI00022316.3	MRPS18B	0.47875
IPI00006970.2	MRPS2	0.848
IPI00014812.1	MRPS21	0.18025
IPI00791542.2	MRPS22	0.58525
IPI00032881.2	MRPS23	0.56975

AccsessionNo	GeneSymbol	hESCs/HFs
IPI00219097.4	HMGB2	0.85425
IPI00219155.5;P613	RPL27	-0.54075
IPI00219156.7;P628	RPL30	-0.45025
IPI00219160.3	RPL34	-0.47125
IPI00219197.3	UBE3A	-0.04475
IPI00219217.3	LDHB	0.562
IPI00219219.3	LGALS1	-4.80975
IPI00219229.3	LSM3	1.41475
IPI00219249.4	CNTNAP1	-3.2195
IPI00219299.4	TLN2	-1.31275
IPI00219301.7	MARCKS	-1.26175
IPI00219306.1;P613	MAGOH	1.14325
IPI00219317.3	CRNKL1	0.48625
IPI00219352.4	CBS	1.7985
IPI00219381.5	NDUFA2	0.02675
IPI00219385.3	NDUFB6	-0.14675
IPI00219420.3	SMC3	2.1895
IPI00219426.1	PVR	-3.42675
IPI00219446.5	PEBP1	0.317
IPI00219452.1	ACOT7	-1.02925
IPI00219483.1	SNRNP70	0.9275
IPI00219504.1	USP15	-1.96275
IPI00219512.2	UCHL5	-0.63225
IPI00219525.10	PGD	-0.70325
IPI00219526.6	PGM1	0.412
IPI00219538.3	POLB	2.32325
IPI00219570.1	FIBP	0.43925
IPI00219604.3	MAP2K1	-0.4325
IPI00219616.7	PRPS1	0.5785

AccessionNo	GeneSymbol	hiPSCs/hESCs
IPI00219156.7;P628	RPL30	0.36575
IPI00219160.3	RPL34	0.3975
IPI00219197.3	UBE3A	0.11725
IPI00219217.3	LDHB	0.42925
IPI00219219.3	LGALS1	-0.2245
IPI00219229.3	LSM3	0.019
IPI00219249.4	CNTNAP1	-0.64575
IPI00219299.4	TLN2	0.9585
IPI00219301.7	MARCKS	0.88975
IPI00219306.1;P613	MAGOH	0.17325
IPI00219317.3	CRNKL1	0.535
IPI00219352.4	CBS	1.03775
IPI00219381.5	NDUFA2	0.0525
IPI00219385.3	NDUFB6	-0.819
IPI00219420.3	SMC3	-0.16475
IPI00219426.1	PVR	0.532
IPI00219446.5	PEBP1	0.251
IPI00219452.1	ACOT7	0.8525
IPI00219483.1	SNRNP70	0.27225
IPI00219504.1	USP15	0.8025
IPI00219512.2	UCHL5	0.25325
IPI00219525.10	PGD	0.74
IPI00219526.6	PGM1	0.65125
IPI00219538.3	POLB	0.1415
IPI00219570.1	FIBP	0.164
IPI00219604.3	MAP2K1	0.245
IPI00219616.7	PRPS1	-0.376
IPI00219617.5	PRPS2	-0.732
IPI00219622.3	PSMA2	0.5465

Table S4 The integrated datasets for the comparison of hiPSCs/HFs, hESCs/HFs, and hiPSCs/hESCs from the public datasets.

AccessionNo	GeneSymbol	hiPSCs/HFs
IPI00006606.1	MRPS26	0.80975
IPI00942760.1	MRPS27	0.6675
IPI00010278.6	MRPS30	0.50525
IPI00294242.2	MRPS31	0.553
IPI00945880.1	MRPS33	0.257
IPI00073779.1	MRPS35	0.67875
IPI00020495.1	MRPS36	-0.69025
IPI00305668.7	MRPS6	1.0725
IPI00006440.6	MRPS7	0.4745
IPI00641924.2	MRPS9	0.829
IPI00106491.3	MRTO4	0.8365
IPI00017303.1	MSH2	2.58325
IPI00384456.4	MSH6	2.729
IPI00872814.1	MSN	-3.16325
IPI00879166.1	MTA1	2.68475
IPI00171798.1	MTA2	-0.0245
IPI00942294.1	MTAP	-0.453
IPI00003833.3	MTCH2	-0.40225
IPI00017510.3	MT-CO2	0.17325
IPI00328715.4	MTDH	-1.6645
IPI00910377.1	MTG1	0.36975
IPI00218342.10	MTHFD1	0.82075
IPI00031410.1	MTOR	-0.70225
IPI00470416.2	MTPAP	1.33575
IPI00924816.1	MTPN	-2.08925
IPI00221302.1	MTRR	-1.94825
IPI00024934.4	MUT	-0.1005
IPI00022745.1	MVD	0.5895
IPI00000105.4	MVP	-5.94875

AccsessionNo	GeneSymbol	hESCs/HFs
IPI00219617.5	PRPS2	0.8115
IPI00219622.3	PSMA2	-0.7385
IPI00219678.3	EIF2S1	-0.582
IPI00219682.6	STOM	-2.90225
IPI00219684.3	FABP3	3.4105
IPI00219685.5	YJEFN3	0.12425
IPI00219754.1	PPFIA1	-0.91725
IPI00219757.13	GSTP1	-0.00225
IPI00219793.1	PPAN	1.0615
IPI00219841.6	LIG1	2.0885
IPI00219866.2	ZRANB2	-0.401
IPI00219870.1	CTNND1	0.4095
IPI00219877.1	HMBS	0.32225
IPI00219897.1	ACSL4	0.68725
IPI00219929.1	MAX	0.4445
IPI00219930.7	CRABP1	3.227
IPI00219994.2	CSE1L	0.73375
IPI00220063.5	NDUFS5	-0.05125
IPI00220219.6	COPB2	-1.7145
IPI00220271.3	AKR1A1	0.872
IPI00220278.5	MYL9	-5.47775
IPI00220289.7	CHD6	1.9405
IPI00220301.5	PRDX6	-1.24425
IPI00220302.1	WTAP	1.68325
IPI00220317.3	POLA1	2.05725
IPI00220327.4	KRT1	0.82975
IPI00220342.5	DDAH1	-1.536
IPI00220344.9;P83882;IPI0(	RPL36A	-0.23325
IPI00220362.5	HSPE1	2.3335

AccessionNo	GeneSymbol	hiPSCs/hESCs
IPI00219678.3	EIF2S1	0.23475
IPI00219682.6	STOM	-0.153
IPI00219684.3	FABP3	-0.38975
IPI00219685.5	YJEFN3	0.11
IPI00219754.1	PPFIA1	0.655
IPI00219757.13	GSTP1	0.6335
IPI00219793.1	PPAN	0.477
IPI00219841.6	LIG1	0.30975
IPI00219866.2	ZRANB2	-0.08275
IPI00219870.1	CTNND1	0.04475
IPI00219877.1	HMBS	0.133
IPI00219897.1	ACSL4	0.08925
IPI00219929.1	MAX	0.43975
IPI00219930.7	CRABP1	-0.816
IPI00219994.2	CSE1L	0.3345
IPI00220063.5	NDUFS5	0.33325
IPI00220219.6	COPB2	0.41075
IPI00220271.3	AKR1A1	0.04175
IPI00220278.5	MYL9	1.6215
IPI00220289.7	CHD6	0.3625
IPI00220301.5	PRDX6	0.406
IPI00220302.1	WTAP	0.0935
IPI00220317.3	POLA1	0.67625
IPI00220327.4	KRT1	1.1775
IPI00220342.5	DDAH1	0.283
IPI00220344.9;P83882;IPI0(	RPL36A	0.373
IPI00220362.5	HSPE1	-0.133
IPI00220381.2	TCF20	-0.22725
IPI00220416.3	UQCRB	0.16475

Table S4 The integrated datasets for the comparison of hiPSCs/HFs, hESCs/HFs, and hiPSCs/hESCs from the public datasets.

AccessionNo	GeneSymbol	hiPSCs/HFs
IPI00878612.1	MYADM	-1.6275
IPI00005024.3	MYBBP1A	1.136
IPI00397526.3	MYH10	0.834
IPI00019502.3	MYH9	-3.3985
IPI00027255.1	MYL6B	0.20725
IPI00220278.5	MYL9	-4.2235
IPI00828082.1	MYO18A	-1.141
IPI00329672.4	MYO1E	-0.54275
IPI00218638.10	MYO1F	-0.38125
IPI00873959.3	MYO5A	-1.9575
IPI00645867.2	MYOF	-5.2
IPI00440719.2	NAA10	-0.60775
IPI00788069.1	NAA30	-0.5655
IPI00023748.3	NACA	0.0195
IPI00045207.2	NACC1	2.0345
IPI00604652.1	NAE1	-0.07425
IPI00900361.1	NAF1	1.465
IPI00414909.1	NAGA	-0.54625
IPI00008787.3	NAGLU	-0.7865
IPI00018873.1	NAMPT	0.18025
IPI00793309.1	NANOG	4.19025
IPI00147874.1	NANS	-1.974
IPI00789029.1	NAP1L1	0.189
941463.1;IPI00017	NAP1L4	-0.1575
IPI00009253.2	NAPA	-1.14875
IPI00293817.3	NAPG	-1.012
IPI00306960.3	NARS	-0.202
IPI00101664.2	NARS2	1.72975
IPI00179953.2	NASP	3.03025

AccsessionNo	GeneSymbol	hESCs/HFs
IPI00220381.2	TCF20	1.919
IPI00220416.3	UQCRB	0.6785
IPI00220426.2	FOXK2	0.57375
IPI00220484.3	HMGN4	1.0785
IPI00220487.4	ATP5H	0.66725
IPI00220503.9	DCTN2	-2.4725
IPI00220527.5	SNX1	-2.70575
IPI00220528.6;P623	SNRPF	1.46975
IPI00220578.3	GNAI3	1.01525
IPI00220627.1	AKAP9	-0.77525
IPI00220637.5	SARS	-1.105
IPI00220642.7;P619	YWHAG	-0.58575
IPI00220644.8	PKM2	-4.603
IPI00220648.5	PMVK	-0.19725
IPI00220684.1	HNRNPD	1.27
IPI00220716.2	RBM15	1.61525
IPI00220739.3	PGRMC1	1.272
IPI00220754.1	ADD3	-1.64375
IPI00220766.5	GLO1	0.11125
IPI00220795.4	EHMT2	2.18825
IPI00220827.5;Q6ZW	TMSB10	-4.32325
IPI00220833.1	UBTF	1.49825
IPI00220834.8	XRCC5	1.05475
IPI00220835.7	SEC61B	-1.35275
IPI00220844.1	SLC12A2	-0.6515
IPI00220993.1	CNP	-0.774
IPI00220994.3	H2AFY2	0.89725
IPI00221088.5;Q6ZW	RPS9	-0.65075
IPI00221089.5;P623	RPS13	-0.7275

AccessionNo	GeneSymbol	hiPSCs/hESCs
IPI00220426.2	FOXK2	0.4565
IPI00220484.3	HMGN4	0.35625
IPI00220487.4	ATP5H	-0.1295
IPI00220503.9	DCTN2	0.71675
IPI00220527.5	SNX1	0.8555
IPI00220528.6;P623	SNRPF	-0.0065
IPI00220578.3	GNAI3	0.2065
IPI00220627.1	AKAP9	0.25025
IPI00220637.5	SARS	0.576
IPI00220642.7;P619	YWHAG	0.25475
IPI00220644.8	PKM2	0.4595
IPI00220648.5	PMVK	-0.33025
IPI00220684.1	HNRNPD	0.1555
IPI00220716.2	RBM15	0.13725
IPI00220739.3	PGRMC1	0.09825
IPI00220754.1	ADD3	0.663
IPI00220766.5	GLO1	0.59075
IPI00220795.4	EHMT2	0.2535
IPI00220827.5;Q6ZW	TMSB10	1.33975
IPI00220833.1	UBTF	0.133
IPI00220834.8	XRCC5	0.039
IPI00220835.7	SEC61B	0.3955
IPI00220844.1	SLC12A2	0.2635
IPI00220993.1	CNP	-0.02425
IPI00220994.3	H2AFY2	1.215
IPI00221088.5;Q6ZW	RPS9	0.561
IPI00221089.5;P623	RPS13	0.57375
IPI00221091.9	RPS15A	0.3325
IPI00221092.8;P141	RPS16	0.42075

Table S4 The integrated datasets for the comparison of hiPSCs/HFs, hESCs/HFs, and hiPSCs/hESCs from the public datasets.

AccessionNo	GeneSymbol	hiPSCs/HFs
IPI00644361.2	NAT1	-1.3095
IPI00300127.3	NAT10	1.3765
IPI00103059.4	NAT14	-0.11225
IPI00641137.2	NAV1	-0.48225
IPI00299524.2	NCAPD2	0.74825
IPI00747787.2	NCAPD3	1.6025
IPI00106495.1	NCAPG	0.7665
IPI00019380.1	NCBP1	0.92625
IPI00925612.1	NCEH1	-5.7985
IPI00031982.1	NCKAP1	-1.2305
IPI00604620.3	NCL	1.0605
IPI00607732.1	NCLN	-0.44075
IPI00288941.1	NCOA5	0.77375
IPI00923531.1	NCOR2	-0.2105
IPI00029561.1	NDUFA10	-0.2275
IPI00005966.6	NDUFA12	-0.5405
IPI00219381.5	NDUFA2	-0.1145
IPI00011770.1	NDUFA4	0.20875
IPI00945153.1	NDUFA6	0.4185
IPI00452731.6	NDUFA7	-0.16975
IPI00219034.3	NDUFA8	-0.0535
IPI00003968.1	NDUFA9	-0.38675
IPI00022442.2	NDUFAB1	0.769
IPI00031109.4	NDUFAF2	0.865
IPI00023064.1	NDUFAF4	0.641
IPI00479905.5	NDUFB10	-0.14725
IPI00472058.1	NDUFB11	-0.0795
IPI00947492.1	NDUFB2	-0.4115
IPI00556190.2	NDUFB5	-0.4235

AccsessionNo	GeneSymbol	hESCs/HFs
IPI00221091.9	RPS15A	-0.342
IPI00221092.8;P141	RPS16	-0.42725
IPI00221093.7	RPS17	-0.5505
IPI00221106.5	SF3B2	0.84075
IPI00221111.1	PACSIN2	-1.56975
IPI00221222.7	SUB1	0.16875
IPI00221232.9	GNG12	-3.89225
IPI00221241.3	LNPEP	-3.009
IPI00221300.2	EIF2B1	-0.95675
IPI00221302.1	MTRR	-2.7765
IPI00221325.3	RANBP2	1.601
IPI00221345.1	YTHDF1	0.71325
IPI00221360.1	PYCARD	0.02725
IPI00232533.5;Q8BM	EIF1AX	0.1975
IPI00234252.3	SMARCC1	3.032
IPI00239077.5	HINT1	-0.9665
IPI00239405.4	SYNE2	3.21525
IPI00239815.9	CIRH1A	1.93625
IPI00241860.4	MIPEP	0.957
IPI00246058.10	PDCD6IP	-1.97475
IPI00246616.2	CLIP2	-1.52725
IPI00247439.3	SLK	-1.314
IPI00249080.2	ASMTL	0.43575
IPI00250297.3	AASDHPPT	-0.3435
IPI00251559.8	RNF20	0.771
IPI00253050.2	L1TD1	3.57375
IPI00256684.2;IPI00793	AP2A1	-1.632
IPI00257508.4	DPYSL2	-0.6925
IPI00288941.1	NCOA5	0.5025

AccessionNo	GeneSymbol	hiPSCs/hESCs
IPI00221093.7	RPS17	0.43475
IPI00221106.5	SF3B2	0.18875
IPI00221111.1	PACSIN2	0.6195
IPI00221222.7	SUB1	0.34025
IPI00221232.9	GNG12	2.949
IPI00221241.3	LNPEP	-0.3655
IPI00221300.2	EIF2B1	0.41675
IPI00221302.1	MTRR	1.10225
IPI00221325.3	RANBP2	0.1415
IPI00221345.1	YTHDF1	-0.09575
IPI00221360.1	PYCARD	-0.281
IPI00232533.5;Q8BM	EIF1AX	0.4045
IPI00232571.1	GPC4	1.13975
IPI00234252.3	SMARCC1	0.18525
IPI00238688.2	RBPMS2	0.682
IPI00239077.5	HINT1	0.4765
IPI00239405.4	SYNE2	-0.395
IPI00239815.9	CIRH1A	-0.07575
IPI00241860.4	MIPEP	-0.2595
IPI00246058.10	PDCD6IP	0.369
IPI00246616.2	CLIP2	-0.25
IPI00247439.3	SLK	0.076
IPI00249080.2	ASMTL	0.618
IPI00250297.3	AASDHPPT	-0.0755
IPI00251559.8	RNF20	0.3155
IPI00253050.2	L1TD1	0.1935
IPI00256684.2;IPI00793	AP2A1	0.3505
IPI00257508.4	DPYSL2	0.14775
IPI00288941.1	NCOA5	0.47075

Table S4 The integrated datasets for the comparison of hiPSCs/HFs, hESCs/HFs, and hiPSCs/hESCs from the public datasets.

AccessionNo	GeneSymbol	hiPSCs/HFs
IPI00219385.3	NDUFB6	-0.94825
IPI00642807.1	NDUFB8	-0.98475
IPI00940744.1	NDUFS1	-0.108
IPI00946334.1	NDUFS2	0.031
IPI00025796.3	NDUFS3	-0.0205
IPI00011217.3	NDUFS4	-0.16375
IPI00220063.5	NDUFS5	0.0355
IPI00025344.1	NDUFS6	-0.468
IPI00291328.3	NDUFV2	-0.43475
IPI00291016.8	NDUFV3	-1.25975
IPI00873768.1	NEDD8	-0.91225
IPI00301609.8	NEK9	-1.9905
IPI00301618.7	NEMF	-1.29275
IPI00002525.3	NENF	-2.48975
IPI00010800.2	NES	-1.8295
IPI00844287.2	NES	-1.76075
IPI00479248.3	NFRKB	1.2895
IPI00643101.1	NFS1	-0.1385
IPI00442274.3	NFXL1	0.81075
IPI00410360.3	NGDN	1.45275
IPI00939140.1	NHP2L1	1.1465
IPI00384542.4	NID1	1.24775
IPI00028908.3	NID2	-3.207
IPI00304435.3	NIPSNAP1	1.6375
IPI00004845.4	NIPSNAP3A	-2.007
IPI00456663.1	NIT1	-0.19125
IPI00005675.3	NKRF	0.959
IPI00018196.5	NLE1	0.84225
IPI00010346.1	NLN	1.67925

AccsessionNo	GeneSymbol	hESCs/HFs
IPI00288947.3	GNAQ	0.4435
IPI00289334.1	FLNB	0.89525
IPI00289499.3	ATIC	0.32025
IPI00289758.6	CAPN2	-5.41525
IPI00289800.7	G6PD	-3.813
IPI00289819.4	IGF2R	-0.924
IPI00289862.5	SCRN1	-1.804
IPI00290142.5	CTPS	-0.6115
IPI00290192.1	RNASEH2A	1.818
IPI00290272.2	POLA2	0.824
IPI00290410.3	DNTTIP2	1.50825
IPI00290416.3	OLA1	-0.57125
IPI00290435.3	KIF2C	3.1585
IPI00290460.3	EIF3G	-0.8715
IPI00290461.3	EIF3J	-1.42425
IPI00290462.5	CBR3	-3.91925
IPI00290543.5	NPLOC4	-1.4355
IPI00290566.1	TCP1	-0.55725
IPI00290684.3	PNKP	1.9475
IPI00290770.3	CCT3	-0.58525
IPI00290812.2	TTF2	1.63525
IPI00290928.2	GNA13	0.53875
IPI00291006.2	MDH2	0.50975
IPI00291016.8	NDUFV3	-1.16275
IPI00291093.3	POLR2E	1.04075
IPI00291131.6	THOC7	1.8585
IPI00291136.4	COL6A1	-4.37125
IPI00291175.7	VCL	-2.05025
IPI00291200.2	NUP133	1.32525

AccessionNo	GeneSymbol	hiPSCs/hESCs
IPI00288947.3	GNAQ	-0.363
IPI00289334.1	FLNB	-0.97875
IPI00289499.3	ATIC	0.348
IPI00289758.6	CAPN2	0.98525
IPI00289800.7	G6PD	0.95
IPI00289819.4	IGF2R	0.64275
IPI00289862.5	SCRN1	0.6595
IPI00290142.5	CTPS	0.55325
IPI00290192.1	RNASEH2A	0.05325
IPI00290272.2	POLA2	0.6485
IPI00290410.3	DNTTIP2	-0.00275
IPI00290416.3	OLA1	0.465
IPI00290435.3	KIF2C	0.03575
IPI00290460.3	EIF3G	0.5925
IPI00290461.3	EIF3J	0.699
IPI00290462.5	CBR3	0.7425
IPI00290543.5	NPLOC4	0.51975
IPI00290566.1	TCP1	0.3395
IPI00290684.3	PNKP	-0.33575
IPI00290770.3	CCT3	0.35275
IPI00290812.2	TTF2	-0.061
IPI00290928.2	GNA13	-0.1235
IPI00291006.2	MDH2	0.002
IPI00291016.8	NDUFV3	0.18575
IPI00291093.3	POLR2E	0.202
IPI00291131.6	THOC7	-0.05425
IPI00291136.4	COL6A1	0.517
IPI00291175.7	VCL	0.43025
IPI00291200.2	NUP133	0.03525

Table S4 The integrated datasets for the comparison of hiPSCs/HFs, hESCs/HFs, and hiPSCs/hESCs from the public datasets.

AccessionNo	GeneSymbol	hiPSCs/HFs
IPI00790937.3	NMD3	-0.596
IPI00012048.1	NME1	-0.80175
IPI00795292.1	NME1	-0.9985
IPI00012315.2	NME3	0.62475
IPI00329692.3	NMT1	-0.59575
IPI00337541.3	NNT	-1.86425
IPI00022373.2	NOB1	0.6785
IPI00411886.4	NOC2L	1.61475
IPI00102815.1	NOC3L	0.764
IPI00031661.1	NOC4L	1.18
IPI00303813.5	NOL11	1.402
IPI00556218.1	NOL6	1.12625
IPI00007729.1	NOL7	1.0135
IPI00002902.5	NOL9	1.14275
IPI00145593.7	NOM1	1.8805
IPI00941810.1	NOMO3	-0.05875
IPI00304596.3	NONO	1.2715
00032853.1;Q9CC	NOP10	1.4135
IPI00022613.4	NOP14	1.244
IPI00032849.2	NOP16	1.0605
IPI00654555.3	NOP2	1.39325
IPI00006379.1	NOP58	1.321
IPI00006408.4	NOSIP	-0.1375
IPI00297655.4	NOTCH2	-2.82175
IPI00156005.3	NOVA2	2.8285
IPI00940960.1	NPC2	0.6985
IPI00026216.4	NPEPPS	-0.66525
IPI00290543.5	NPLOC4	-1.28775
IPI00549248.4	NPM1	1.2615

AccsessionNo	GeneSymbol	hESCs/HFs
IPI00291328.3	NDUFV2	-0.0675
IPI00291373.2	ABCD1	-3.4285
IPI00291412.1	PPM1F	-2.71325
IPI00291417.2	DCAKD	0.59325
IPI00291419.6	ACAT2	0.78575
IPI00291467.7	SLC25A6	-0.73975
IPI00291510.3	IMPDH2	0.5185
IPI00291525.1	TFB1M	0.238
IPI00291669.3	UBLCP1	-0.359
IPI00291755.6	NUP210	3.32625
IPI00291783.3	GEMIN5	0.4625
IPI00291802.3	LMO7	-5.1015
IPI00291916.5	PHIP	2.31675
IPI00291922.2;Q9Z2	PSMA5	-0.79325
IPI00291928.8;Q91V	RAB14	-1.57175
IPI00291930.6	CLINT1	0.22
IPI00291939.1	SMC1A	2.07775
IPI00292020.3	SRM	-1.9555
IPI00292059.2	NUP153	0.912
IPI00292140.4	CASP3	-0.05475
IPI00292221.3	RPF1	0.56875
IPI00292228.1	GSK3A	-0.47
IPI00292499.4	HSPA14	0.702
IPI00292894.5	TSR1	0.48675
IPI00292975.4	RBM27	1.39425
IPI00293078.1	DDX27	1.03475
IPI00293088.6	GAA	1.251
IPI00293167.4	SDF2	-1.3135
IPI00293242.1	GTF2I	2.2905

AccessionNo	GeneSymbol	hiPSCs/hESCs
IPI00291328.3	NDUFV2	0.00575
IPI00291373.2	ABCD1	1.09625
IPI00291412.1	PPM1F	0.087
IPI00291417.2	DCAKD	0.63925
IPI00291419.6	ACAT2	1.00175
IPI00291467.7	SLC25A6	0.4725
IPI00291510.3	IMPDH2	-0.481
IPI00291525.1	TFB1M	1.05575
IPI00291669.3	UBLCP1	0.47075
IPI00291755.6	NUP210	-0.461
IPI00291783.3	GEMIN5	0.1535
IPI00291802.3	LMO7	0.2
IPI00291916.5	PHIP	-0.16875
IPI00291922.2;Q9Z2	PSMA5	0.4555
IPI00291928.8;Q91V	RAB14	0.36625
IPI00291930.6	CLINT1	0.264
IPI00291939.1	SMC1A	-0.01675
IPI00292020.3	SRM	0.406
IPI00292059.2	NUP153	0.159
IPI00292140.4	CASP3	0.78875
IPI00292221.3	RPF1	0.71475
IPI00292228.1	GSK3A	0.395
IPI00292499.4	HSPA14	0.19425
IPI00292894.5	TSR1	0.5635
IPI00292975.4	RBM27	0.198
IPI00293078.1	DDX27	0.35725
IPI00293088.6	GAA	-0.25175
IPI00293167.4	SDF2	0.20825
IPI00293242.1	GTF2I	0.25125

Table S4 The integrated datasets for the comparison of hiPSCs/HFs, hESCs/HFs, and hiPSCs/hESCs from the public datasets.

AccessionNo	GeneSymbol	hiPSCs/HFs
IPI00657724.1	NPTN	-2.023
IPI00000005.1	NRAS	-0.27875
IPI00604756.2	NRBP1	-1.78975
IPI00478723.3	NRD1	-1.7195
IPI00645046.1	NRM	0.39775
IPI00019407.1	NSDHL	0.811
IPI00936987.1	NSF	-2.324
IPI00306369.3	NSUN2	-0.00075
IPI00647805.1	NT5C3	-0.69975
IPI00177965.5	NT5DC1	0.69375
IPI00783118.2	NT5DC2	1.16225
IPI00031570.1	NTPCR	1.1585
IPI00869040.2	NUBP1	0.23125
IPI00295542.5	NUCB1	-0.903
IPI00022145.6	NUCKS1	-0.4495
IPI00306398.7	NUDCD1	0.31425
IPI00103142.1	NUDCD2	0.92925
IPI00396056.6	NUDT1	0.7495
IPI00646917.1	NUDT21	1.15575
IPI00296913.1	NUDT5	0.2345
IPI00415040.1	NUDT9	0.04
IPI00002349.2	NUFIP2	0.09175
IPI00028005.1	NUP107	1.20225
IPI00291200.2	NUP133	1.116
IPI00292059.2	NUP153	0.8355
IPI00376609.1	NUP155	1.17025
IPI00748807.2	NUP160	1.3835
IPI00783781.1	NUP205	1.2105
IPI00291755.6	NUP210	2.55675

AccsessionNo	GeneSymbol	hESCs/HFs
IPI00293276.10	MIF	0.424
IPI00293307.1	PLIN2	2.3255
IPI00293331.3	POP1	-1.3135
IPI00293350.3	TSNAX	-0.3835
IPI00293434.2	SRP14	0.96375
IPI00293464.5	DDB1	0.56675
IPI00293476.3	MRPL23	0.235
IPI00293533.4	NUP62	1.07575
IPI00293655.3	DDX1	-0.15125
IPI00293735.2	IKBKAP	-0.322
IPI00293748.3	MINPP1	-0.634
IPI00293817.3	NAPG	-1.17125
IPI00293867.7	DDT	0.04125
IPI00293884.6	KIF23	0.85275
IPI00293946.5	UBXN4	-0.704
IPI00294158.1	BLVRA	-1.935
IPI00294159.3	SLC25A1	0.848
IPI00294211.2	DHX38	-0.21975
IPI00294229.6	RCL1	1.88525
IPI00294242.2	MRPS31	0.34625
IPI00294398.1	HADH	1.4405
IPI00294495.5	UFC1	-1.34675
IPI00294501.1	DHCR7	1.37575
IPI00294603.6	ZMYM2	3.755
IPI00294701.1	MNAT1	-0.2835
IPI00294728.1	DMXL1	1.255
IPI00294739.1	SAMHD1	1.0625
IPI00294834.6	ASPH	-3.81925
IPI00294839.6	LOXL2	-3.604

AccessionNo	GeneSymbol	hiPSCs/hESCs
IPI00293276.10	MIF	0.28525
IPI00293307.1	PLIN2	-0.38725
IPI00293331.3	POP1	0.2545
IPI00293350.3	TSNAX	0.29475
IPI00293434.2	SRP14	0.3485
IPI00293464.5	DDB1	-0.1145
IPI00293476.3	MRPL23	0.195
IPI00293533.4	NUP62	0.1025
IPI00293655.3	DDX1	-0.0525
IPI00293735.2	IKBKAP	0.51675
IPI00293748.3	MINPP1	0.2845
IPI00293817.3	NAPG	0.4585
IPI00293867.7	DDT	0.269
IPI00293884.6	KIF23	0.58075
IPI00293946.5	UBXN4	0.1065
IPI00294158.1	BLVRA	0.475
IPI00294159.3	SLC25A1	-0.1365
IPI00294211.2	DHX38	0.41725
IPI00294229.6	RCL1	-0.1175
IPI00294242.2	MRPS31	0.527
IPI00294398.1	HADH	-0.143
IPI00294495.5	UFC1	0.88975
IPI00294501.1	DHCR7	0.56275
IPI00294603.6	ZMYM2	0.10325
IPI00294701.1	MNAT1	0.511
IPI00294728.1	DMXL1	-0.0085
IPI00294739.1	SAMHD1	-1.32025
IPI00294834.6	ASPH	0.11325
IPI00294839.6	LOXL2	0.22075

Table S4 The integrated datasets for the comparison of hiPSCs/HFs, hESCs/HFs, and hiPSCs/hESCs from the public datasets.

AccessionNo	GeneSymbol	hiPSCs/HFs	AccsessionNo	GeneSymbol	hESCs/HFs	AccessionNo	GeneSymbol	hiPSCs/hESCs
IPI00900318.1	NUP214	0.68425	IPI00294840.6	AIM1	1.2535	IPI00294840.6	AIM1	-1.413
IPI00916892.1	NUP35	1.65925	IPI00294879.1	RANGAP1	-0.181	IPI00294879.1	RANGAP1	0.36675
IPI00171665.1	NUP37	1.03925	IPI00294911.1	SDHB	0.5255	IPI00294911.1	SDHB	-0.57375
IPI00742943.1	NUP43	0.792	IPI00294955.3	LSM4	1.062	IPI00294955.3	LSM4	0.29625
IPI00170581.1	NUP50	1.46225	IPI00295081.1	TUBG1	-0.724	IPI00295081.1	TUBG1	0.42725
IPI00940095.1	NUP54	1.0175	IPI00295098.3	SRPRB	-1.711	IPI00295098.3	SRPRB	0.29325
IPI00293533.4	NUP62	0.89475	IPI00295386.7	CBR1	-0.7495	IPI00295386.7	CBR1	-1.09975
IPI00001738.4	NUP88	0.687	IPI00295400.1	WARS	-1.68325	IPI00295400.1	WARS	0.963
IPI00397904.6	NUP93	1.10325	IPI00295427.4	MRPL39	0.23775	IPI00295427.4	MRPL39	0.438
IPI00910174.1	NUPL1	1.0645	IPI00295503.2	DDX58	0.10475	IPI00295503.2	DDX58	-0.332
IPI00061142.3	NUS1	0.649	IPI00295542.5	NUCB1	-0.5005	IPI00295542.5	NUCB1	-0.10925
IPI00009901.1	NUTF2	-0.19125	IPI00295698.6	SLC7A3	1.992	IPI00295698.6	SLC7A3	0.42825
IPI00033153.1	NXF1	0.699	IPI00295851.4	COPB1	-1.8885	IPI00295851.4	COPB1	0.45575
IPI00304267.3	NXN	-0.62475	IPI00295857.7	COPA	-1.8125	IPI00295857.7	COPA	0.306
IPI00022334.1	OAT	1.286	IPI00295889.2	SRP19	-0.761	IPI00295889.2	SRP19	0.39775
IPI00016405.1	OCIAD1	0.41325	IPI00295992.4	ATAD3A	-0.2255	IPI00295992.4	ATAD3A	0.0485
IPI00555902.1	OCIAD2	3.15125	IPI00296099.6	THBS1	-5.99825	IPI00296099.6	THBS1	1.283
IPI00926312.1	OGDH	-0.0145	IPI00296141.4	DPP7	-2.438	IPI00296141.4	DPP7	-1.0325
IPI00290416.3	OLA1	-0.4545	IPI00296337.2	PRKDC	1.168	IPI00296215.2	EPCAM	-0.1495
IPI00941628.2	OPLAH	0.58525	IPI00296380.1	TNFRSF10B	0.852	IPI00296337.2	PRKDC	0.18375
IPI00013216.1	ORC2	2.41575	IPI00296421.2	EHBP1L1	-5.40475	IPI00296380.1	TNFRSF10B	0.03075
IPI00910327.1	ORC3	1.90475	IPI00296485.6	MAP1S	0.34125	IPI00296421.2	EHBP1L1	0.55825
IPI00024971.1	OSBP	-0.93275	IPI00296907.4	ACOX1	0.33825	IPI00296485.6	MAP1S	-0.01725
IPI00032970.2	OSBPL11	-0.32475	IPI00296913.1	NUDT5	0.061	IPI00296907.4	ACOX1	-0.4495
IPI00394737.1	OSBPL8	-0.17325	IPI00296922.4	LAMB2	-0.82775	IPI00296913.1	NUDT5	0.507
IPI00869148.1	OSGEPL1	1.273	IPI00297084.7	DDOST	-0.4435	IPI00296922.4	LAMB2	-0.40025
IPI00414836.6	OSTF1	-2.03175	IPI00297121.5	C20orf43	-0.95775	IPI00297084.7	DDOST	0.182
IPI00939174.1	OTUB1	-0.45725	IPI00297191.2	MED14	0.86575	IPI00297121.5	C20orf43	0.216
IPI00935722.1	OTUD6B	0.044	IPI00297211.1	SMARCA5	2.3645	IPI00297191.2	MED14	0.391

Table S4 The integrated datasets for the comparison of hiPSCs/HFs, hESCs/HFs, and hiPSCs/hESCs from the public datasets.

AccessionNo	GeneSymbol	hiPSCs/HFs
IPI00026516.1	OXCT1	-0.47725
IPI00010080.2	OXSR1	-0.977
IPI00218682.1	P4HA1	-1.234
IPI00009923.1	P4HA1	-3.9695
IPI00182373.2	P4HA2	-4.44175
IPI00010796.1	P4HB	-2.0955
IPI00299000.5	PA2G4	0.037
IPI00008524.1	PABPC1	0.221
IPI00414963.2	PABPN1	1.70025
IPI00221111.1	PACSIN2	-1.20375
IPI00329572.4	PACSIN3	0.73775
IPI00300333.4	PAF1	0.66875
IPI00218728.4	PAFAH1B1	-1.208
IPI00026546.1	PAFAH1B2	-0.3585
IPI00014808.1	PAFAH1B3	0.339
IPI00217223.1	PAICS	1.16225
IPI00656138.1	PAK1	2.25525
IPI00549540.3	PAK1IP1	1.464
IPI00419979.3	PAK2	-1.47725
IPI00910339.1	PANK4	-0.964
IPI00940660.1	PAPD5	1.19775
IPI00384028.5	PAPOLA	0.618
IPI00011619.4	PAPSS1	-0.1695
IPI00030009.4	PAPSS2	-4.72975
IPI00019025.2	PARD6B	2.40725
IPI00298547.3	PARK7	-0.91125
IPI00909122.1	PARN	1.32275
IPI00449049.5	PARP1	2.79625
IPI00018963.3	PARVA	-4.22275

AccsessionNo	GeneSymbol	hESCs/HFs
IPI00297261.3	PTPN1	-1.08675
IPI00297357.1	PINX1	1.88675
IPI00297455.5	AKAP8L	1.4045
IPI00297477.3	SNRPA1	1.1825
IPI00297492.2	STT3A	-0.686
IPI00297572.5	AQR	1.51975
IPI00297579.4	CBX3	1.262
IPI00297626.4	STXBP3	-0.31375
IPI00297646.4	COL1A1	-5.4215
IPI00297655.4	NOTCH2	-3.17175
IPI00297779.7	CCT2	-0.557
IPI00297900.4	DDX10	2.3085
IPI00297982.7	EIF2S3	-0.67675
IPI00298057.2	PPL	0.922
IPI00298281.4	LAMC1	-0.54
IPI00298547.3	PARK7	-1.01175
IPI00298618.1	EIF1AD	-0.2125
IPI00298702.2	SLC39A6	-0.24075
IPI00298731.2	PPP1R10	0.90275
IPI00298738.3	POLRMT	1.851
IPI00298793.4	MANBA	0.00375
IPI00298851.4	CD151	-2.591
IPI00298926.4	TAF5	2.12775
IPI00298949.1	GAK	-0.7855
IPI00298991.9	KIAA1033	-1.74875
IPI00298994.6	TLN1	-4.491
IPI00299000.5	PA2G4	-0.01875
IPI00299003.2	WASF3	0.50425
IPI00299024.9	BASP1	-1.625

AccessionNo	GeneSymbol	hiPSCs/hESCs
IPI00297211.1	SMARCA5	0.313
IPI00297261.3	PTPN1	0.28375
IPI00297357.1	PINX1	0.83825
IPI00297455.5	AKAP8L	-0.0315
IPI00297477.3	SNRPA1	0.26275
IPI00297492.2	STT3A	0.04
IPI00297572.5	AQR	0.187
IPI00297579.4	CBX3	0.1825
IPI00297626.4	STXBP3	0.05325
IPI00297646.4	COL1A1	0.06475
IPI00297655.4	NOTCH2	0.7035
IPI00297779.7	CCT2	0.44775
IPI00297900.4	DDX10	-0.016
IPI00297982.7	EIF2S3	0.30725
IPI00298057.2	PPL	0.22675
IPI00298281.4	LAMC1	0.14475
IPI00298547.3	PARK7	0.41625
IPI00298618.1	EIF1AD	0.96175
IPI00298702.2	SLC39A6	0.07175
IPI00298731.2	PPP1R10	0.1825
IPI00298738.3	POLRMT	0.179
IPI00298793.4	MANBA	0.748
IPI00298851.4	CD151	-0.353
IPI00298926.4	TAF5	0.2645
IPI00298949.1	GAK	0.43925
IPI00298991.9	KIAA1033	0.434
IPI00298994.6	TLN1	0.88975
IPI00299000.5	PA2G4	0.34525
IPI00299003.2	WASF3	0.0505

Table S4 The integrated datasets for the comparison of hiPSCs/HFs, hESCs/HFs, and hiPSCs/hESCs from the public datasets.

AccessionNo	GeneSymbol	hiPSCs/HFs
IPI00001871.2	PAWR	0.64775
IPI00299402.1	PC	-0.229
I00016610.2;P603	PCBP1	0.5355
IPI00072541.4	PCID2	1.21375
IPI00797038.1	PCK2	0.471
IPI00795923.3	PCM1	1.4785
IPI00411680.10	PCMT1	-1.05725
IPI00021700.3	PCNA	0.78775
IPI00384280.5	PCYOX1	-0.87925
IPI00013297.1	PDAP1	-0.77
IPI00400922.5	PDCD11	1.61975
IPI00031647.2	PDCD2L	1.09325
IPI00023640.3	PDCD5	-0.782
IPI00025277.5	PDCD6	-0.5195
IPI00246058.10	PDCD6IP	-1.92975
IPI00886797.1	PDE12	-1.88325
IPI00015902.3	PDGFRB	-2.37525
IPI00549885.4	PDHB	1.0445
IPI00913991.1	PDHX	0.71675
IPI00025252.1	PDIA3	-0.349
IPI00893541.1	PDIA3	-0.38025
IPI00009904.1	PDIA4	0.03
IPI00031479.1	PDIA5	-1.12425
IPI00644989.2	PDIA6	-0.3355
IPI00014849.3	PDK3	0.93875
IPI00010414.4	PDLIM1	-1.54975
IPI00032206.2	PDLIM4	-4.5185
IPI00007935.5	PDLIM5	-2.4685
IPI00023122.1	PDLIM7	-4.6055

AccsessionNo	GeneSymbol	hESCs/HFs
IPI00299033.1	KPNA3	-0.35125
IPI00299063.2	STIM1	-1.76275
IPI00299084.1	TMEM33	0.11375
IPI00299095.2	SNX2	-0.21475
IPI00299177.4	CECR5	3.39475
IPI00299193.1	SYNJ2BP	0.6005
IPI00299254.4	EIF5B	-0.309
IPI00299402.1	PC	-0.93625
IPI00299413.1	GABPA	0.38225
IPI00299506.9	GFPT1	-1.2905
IPI00299524.2	NCAPD2	0.1895
IPI00299573.12	RPL7A	-0.4485
IPI00299719.2	TCIRG1	-3.95675
IPI00299755.2	PIK3C3	-0.64125
IPI00299904.3	MCM7	2.20825
IPI00300050.3	HSD11B2	4.019
IPI00300060.4	WDR70	-0.3765
IPI00300074.3	FARSB	-0.6635
IPI00300078.6	PWP2	2.03525
IPI00300086.3	QPRT	2.4745
IPI00300094.6	LSG1	0.2205
IPI00300096.4;Q6PH	RAB35	-0.8665
IPI00300127.3	NAT10	1.57675
IPI00300299.6	SPCS3	-0.33425
IPI00300333.4	PAF1	0.5355
IPI00300371.5	SF3B3	0.872
IPI00300408.3	CUTC	3.099
IPI00300504.5	UPF2	-0.0865
IPI00300562.2	RAB3B	-3.1795

AccessionNo	GeneSymbol	hiPSCs/hESCs
IPI00299024.9	BASP1	1.30925
IPI00299033.1	KPNA3	0.1355
IPI00299063.2	STIM1	0.035
IPI00299084.1	TMEM33	0.40525
IPI00299095.2	SNX2	0.42325
IPI00299177.4	CECR5	-0.8195
IPI00299193.1	SYNJ2BP	0.08475
IPI00299254.4	EIF5B	0.2715
IPI00299402.1	PC	1.07675
IPI00299413.1	GABPA	0.09475
IPI00299506.9	GFPT1	0.62175
IPI00299524.2	NCAPD2	0.67
IPI00299573.12	RPL7A	0.3755
IPI00299719.2	TCIRG1	-0.17625
IPI00299755.2	PIK3C3	0.15125
IPI00299904.3	MCM7	0.03
IPI00300050.3	HSD11B2	-0.20825
IPI00300060.4	WDR70	0.61875
IPI00300074.3	FARSB	0.173
IPI00300078.6	PWP2	0.14175
IPI00300086.3	QPRT	0.0335
IPI00300094.6	LSG1	0.4375
IPI00300096.4;Q6PH	RAB35	0.30675
IPI00300299.6	SPCS3	0.2685
IPI00300333.4	PAF1	0.23425
IPI00300371.5	SF3B3	0.24025
IPI00300408.3	CUTC	-0.384
IPI00300504.5	UPF2	0.15875
IPI00300562.2	RAB3B	0.97375

Table S4 The integrated datasets for the comparison of hiPSCs/HFs, hESCs/HFs, and hiPSCs/hESCs from the public datasets.

AccessionNo	GeneSymbol	hiPSCs/HFs
IPI00218971.4	PDP1	-0.50925
IPI00168407.3	PDPR	0.6235
IPI00845446.2	PDS5B	1.8105
IPI00645809.1	PDXDC1	-0.70425
IPI00168698.1	PDZD8	-0.2915
IPI00737545.4	PEAK1	-2.68275
IPI00219446.5	PEBP1	0.18425
IPI00018235.3	PEF1	-1.09925
IPI00106698.1	PELO	-1.0475
IPI00006702.4	PELP1	1.59875
IPI00878536.1	PES1	0.87125
IPI00027228.1	PET112	0.78175
IPI00021978.1	PEX11B	-2.1245
IPI00922479.1	PEX14	-0.509
IPI00004534.4	PFAS	1.079
IPI00000051.4	PFDN1	-0.71
IPI00006052.3	PFDN2	-0.565
IPI00015891.1	PFDN4	-0.40425
IPI00015361.1	PFDN5	-0.423
IPI00005657.1	PFDN6	-0.58525
IPI00332371.9	PFKL	-0.91725
IPI00009790.1	PFKP	-2.228
IPI00216691.5	PFN1	-1.1865
IPI00945855.1	PFN2	-1.212
IPI00795892.1	PFN2	-1.674
IPI00788907.2	PGAM5	1.1695
IPI00219525.10	PGD	-0.24175
IPI00169383.3	PGK1	-0.013
IPI00029997.1	PGLS	-1.67875

AccsessionNo	GeneSymbol	hESCs/HFs
IPI00300659.4;Q8JZI	CDC73	1.104
IPI00300952.3	PPIL3	0.3325
IPI00301058.5	VASP	-1.74825
IPI00301163.1	POGLUT1	-0.881
IPI00301280.2	TMEM43	-2.00575
IPI00301323.1	DDX18	1.30775
IPI00301364.3	SKP1	-0.0705
IPI00301419.2	COPS7A	-3.241
IPI00301609.8	NEK9	-2.26825
IPI00301618.7	NEMF	-1.67125
IPI00301719.1	TRNT1	0.28725
IPI00302238.3	AATF	1.698
IPI00302458.10	XPO7	0.662
IPI00302592.2	FLNA	-2.19025
IPI00302850.4;P623	SNRPD1	0.81525
IPI00302925.4	CCT8	-0.445
IPI00302927.6	CCT4	-0.5065
IPI00303099.2	CDCA8	1.168
IPI00303105.3;P631	SUMO1	1.6385
IPI00303158.3	CMAS	1.622
IPI00303207.3	ABCE1	-0.1945
IPI00303300.3	FKBP10	-4.04125
IPI00303343.7	SCAF1	1.0925
IPI00303452.5	SLC39A14	0.1915
IPI00303476.1	ATP5B	0.51425
IPI00303568.3	PTGES2	0.1085
IPI00303722.5	FAM136A	2.501
IPI00303753.6	SELRC1	2.795
IPI00303812.7	C9orf82	1.241

AccessionNo	GeneSymbol	hiPSCs/hESCs
IPI00300659.4;Q8JZI	CDC73	0.235
IPI00300952.3	PPIL3	0.45875
IPI00301058.5	VASP	0.56925
IPI00301163.1	POGLUT1	0.58725
IPI00301280.2	TMEM43	0.1425
IPI00301323.1	DDX18	0.29275
IPI00301364.3	SKP1	0.28625
IPI00301419.2	COPS7A	0.569
IPI00301609.8	NEK9	0.52675
IPI00301618.7	NEMF	0.70275
IPI00301719.1	TRNT1	-0.47875
IPI00302238.3	AATF	-0.034
IPI00302458.10	XPO7	0.03325
IPI00302592.2	FLNA	0.316
IPI00302850.4;P623	SNRPD1	0.244
IPI00302925.4	CCT8	0.3205
IPI00302927.6	CCT4	0.42525
IPI00303099.2	CDCA8	1.0785
IPI00303105.3;P631	SUMO1	0.15575
IPI00303158.3	CMAS	-0.373
IPI00303207.3	ABCE1	0.29325
IPI00303300.3	FKBP10	1.0015
IPI00303343.7	SCAF1	0.27625
IPI00303452.5	SLC39A14	0.7435
IPI00303476.1	ATP5B	-0.14925
IPI00303568.3	PTGES2	-0.20925
IPI00303722.5	FAM136A	0.06475
IPI00303753.6	SELRC1	-0.027
IPI00303812.7	C9orf82	0.34375

Table S4 The integrated datasets for the comparison of hiPSCs/HFs, hESCs/HFs, and hiPSCs/hESCs from the public datasets.

AccessionNo	GeneSymbol	hiPSCs/HFs
IPI00219526.6	PGM1	0.56125
IPI00550364.8	PGM2	-1.09975
IPI00173346.3	PGM2L1	1.65625
IPI00177008.1	PGP	0.11725
IPI00220739.3	PGRMC1	1.14225
IPI00005202.2	PGRMC2	-1.151
IPI00017334.1	PHB	0.89725
I00027252.6;O351	PHB2	0.7825
IPI00555755.3	PHC1	2.46325
IPI00793412.1	PHF10	1.75875
IPI00847550.1	PHF3	1.7595
IPI00005511.1	PHF5A	1.0335
IPI00011200.5	PHGDH	1.19675
IPI00291916.5	PHIP	1.87925
IPI00400849.2	PICALM	-1.6315
IPI00554703.1	PIGS	-1.04725
IPI00402109.4	PIGU	0.1885
IPI00550995.2	PIH1D1	0.14175
IPI00002580.2	PIK3C2A	1.7275
IPI00299755.2	PIK3C3	-0.776
IPI00013723.3	PIN1	-1.13475
IPI00941201.1	PIN4	0.4205
IPI00297357.1	PINX1	2.521
IPI00009688.1	PIP4K2A	-1.37825
IPI00216470.1	PIP4K2B	-1.543
IPI00554660.1	PIPOX	2.50125
IPI00012575.1	PIR	0.3945
IPI00216048.9	PITPNA	-1.09975
IPI00787827.1	PITRM1	-2.5935

AccsessionNo	GeneSymbol	hESCs/HFs
IPI00303813.5	NOL11	1.69475
IPI00303992.3	VRTN	0.211
IPI00304071.4	ACSF2	-0.04825
IPI00304187.8	RBM28	0.72675
IPI00304232.1	WDR12	1.25775
IPI00304267.3	NXN	-0.978
IPI00304409.3	CARHSP1	1.1405
IPI00304417.7	IDH3B	-0.541
IPI00304435.3	NIPSNAP1	2.88625
IPI00304493.5	SRFBP1	1.58575
IPI00304589.4	TNKS1BP1	-1.76375
IPI00304596.3	NONO	1.201
IPI00304612.9	SNORD32A	-0.3745
IPI00304692.1	RBMX	1.4655
IPI00304742.5	STK10	-2.91575
IPI00304840.4	COL6A2	-5.50225
IPI00304925.5	HSPA1B	-0.40875
IPI00304932.2	RRP8	0.4905
IPI00304962.3	COL1A2	-5.7175
IPI00305068.5	PRPF6	1.2375
IPI00305166.2	SDHA	0.496
IPI00305282.2	RAD50	0.8185
IPI00305289.2	KIF11	2.211
IPI00305374.5	THOC1	1.41025
IPI00305383.1	UQCRC2	0.57975
IPI00305469.4	CRK	-1.76375
IPI00305668.7	MRPS6	1.13725
IPI00305692.5	TXNL1	-0.35875
IPI00305833.3;Q3UK	SMU1	1.5885

AccessionNo	GeneSymbol	hiPSCs/hESCs
IPI00303813.5	NOL11	-0.05175
IPI00303992.3	VRTN	0.81
IPI00304071.4	ACSF2	-0.58225
IPI00304187.8	RBM28	0.8275
IPI00304232.1	WDR12	0.035
IPI00304267.3	NXN	0.65925
IPI00304409.3	CARHSP1	0.235
IPI00304417.7	IDH3B	0.21525
IPI00304435.3	NIPSNAP1	-0.8745
IPI00304493.5	SRFBP1	0.06375
IPI00304589.4	TNKS1BP1	0.68325
IPI00304596.3	NONO	0.33075
IPI00304612.9	SNORD32A	0.38825
IPI00304692.1	RBMX	0.40375
IPI00304742.5	STK10	0.168
IPI00304840.4	COL6A2	0.43025
IPI00304925.5	HSPA1B	0.016
IPI00304932.2	RRP8	0.1
IPI00304962.3	COL1A2	-0.094
IPI00305068.5	PRPF6	0.12925
IPI00305166.2	SDHA	-0.5035
IPI00305282.2	RAD50	-0.04625
IPI00305289.2	KIF11	0.46325
IPI00305374.5	THOC1	0.18375
IPI00305383.1	UQCRC2	0.172
IPI00305469.4	CRK	0.476
IPI00305668.7	MRPS6	0.19225
IPI00305692.5	TXNL1	0.30075
IPI00305833.3;Q3UK	SMU1	0.15

Table S4 The integrated datasets for the comparison of hiPSCs/HFs, hESCs/HFs, and hiPSCs/hESCs from the public datasets.

AccessionNo	GeneSymbol	hiPSCs/HFs
479186.7;IPI00479	PKM2	-1.94275
IPI00220644.8	PKM2	-4.62625
IPI00002803.6	PKN1	-1.04175
IPI00413780.3	PKN3	2.6105
IPI00218465.10	PLAA	-0.68875
IPI00016255.4	PLBD1	1.94425
IPI00169285.6	PLBD2	-0.85
IPI00010400.1	PLCB3	0.263
IPI00016736.1	PLCG1	0.4435
IPI00328243.2	PLD3	-0.293
IPI00029515.3	PLEKHA5	1.11
IPI00293307.1	PLIN2	1.2815
IPI00021248.1	PLK1	3.299
IPI00943008.1	PLOD1	-2.571
IPI00947400.1	PLOD2	-2.542
IPI00030255.1	PLOD3	-2.5885
IPI00030362.1	PLP2	-6.62975
IPI00032304.2	PLS1	1.45125
IPI00853369.1	PLXNB2	-2.1185
IPI00006092.1	PMM2	-1.24925
IPI00166749.3	PMPCA	0.30625
IPI00927892.1	PMPCB	0.34625
IPI00220648.5	PMVK	-0.88575
IPI00001022.5	PNKD	0.1595
IPI00290684.3	PNKP	1.24525
IPI00789041.2	PNN	1.52775
IPI00024524.4	PNO1	1.09975
IPI00017672.4	PNP	1.0665
IPI00921822.2	PNPLA6	-0.99625

AccsessionNo	GeneSymbol	hESCs/HFs
IPI00306290.5	XPOT	-0.2815
IPI00306332.4;Q8BP	RPL24	-0.5775
IPI00306353.3	DUSP23	0.53475
IPI00306369.3	NSUN2	-0.136
IPI00306398.7	NUDCD1	0.384
IPI00306400.9	KIFC1	2.3575
IPI00306446.2	ZNF24	1.544
IPI00306471.1	CCDC94	0.8245
IPI00306516.1	TIMM44	0.8355
IPI00306576.1	ARSB	-2.00325
IPI00306604.5	ITGA5	-4.3335
IPI00306642.3	DCAF13	1.7975
IPI00306723.3	CEBPZ	3.301
IPI00306749.8	SLC4A1AP	0.3525
IPI00306882.4	CPSF3L	0.4495
IPI00306960.3	NARS	-0.24425
IPI00307155.8	ROCK2	-2.714
IPI00307259.11	DNAJC13	-2.26425
IPI00307572.1	TMEM165	-1.6325
IPI00307829.7	CGNL1	3.2895
IPI00328243.2	PLD3	-1.12925
IPI00328306.9	LOC441155	2.3895
IPI00328328.3;P106	MIR1248	-0.211
IPI00328361.7	SARS2	2.17925
IPI00328391.3	GALNT7	1.83075
IPI00328715.4	MTDH	-1.49925
IPI00328822.1	EGLN1	-0.686
IPI00328840.9	THOC4	0.5385
IPI00328929.4	ZC3H18	1.0395

AccessionNo	GeneSymbol	hiPSCs/hESCs
IPI00306290.5	XPOT	0.31025
IPI00306332.4;Q8BP	RPL24	0.465
IPI00306353.3	DUSP23	-0.057
IPI00306369.3	NSUN2	0.4595
IPI00306398.7	NUDCD1	0.227
IPI00306400.9	KIFC1	0.216
IPI00306446.2	ZNF24	0.32975
IPI00306471.1	CCDC94	0.84125
IPI00306576.1	ARSB	0.531
IPI00306604.5	ITGA5	1.551
IPI00306642.3	DCAF13	0.04225
IPI00306723.3	CEBPZ	-0.58375
IPI00306749.8	SLC4A1AP	-0.051
IPI00306882.4	CPSF3L	1.07
IPI00306960.3	NARS	0.297
IPI00307155.8	ROCK2	0.59625
IPI00307259.11	DNAJC13	0.34875
IPI00307572.1	TMEM165	0.8295
IPI00307829.7	CGNL1	-0.95225
IPI00328243.2	PLD3	1.14075
IPI00328306.9	LOC441155	0.16325
IPI00328328.3;P106	MIR1248	0.4465
IPI00328361.7	SARS2	-0.1345
IPI00328391.3	GALNT7	0.09875
IPI00328715.4	MTDH	0.06225
IPI00328822.1	EGLN1	-0.1415
IPI00328840.9	THOC4	0.556
IPI00328929.4	ZC3H18	0.18525
IPI00328987.3	BYSL	0.091

Table S4 The integrated datasets for the comparison of hiPSCs/HFs, hESCs/HFs, and hiPSCs/hESCs from the public datasets.

AccessionNo	GeneSymbol	hiPSCs/HFs
IPI00744711.2	PNPT1	0.6315
IPI00926421.1	PODXL	3.2765
IPI00058192.1	POFUT1	-1.96125
IPI00301163.1	POGLUT1	-0.60125
IPI00514078.1	POGZ	2.59075
IPI00290272.2	POLA2	1.153
IPI00219538.3	POLB	2.135
IPI00002894.2	POLD1	2.04025
IPI00394926.1	POLD3	1.93775
IPI00165506.4	POLDIP2	-0.0345
IPI00940039.1	POLDIP3	1.656
IPI00010141.4	POLE3	0.454
IPI00026445.3	POLR1B	1.48
IPI00217386.1	POLR1C	0.60775
IPI00032439.1	POLR1D	0.79275
IPI00550638.2	POLR1E	1.531
IPI00031627.4	POLR2A	1.32975
IPI00027808.1	POLR2B	0.876
IPI00018288.1	POLR2C	0.8165
IPI00291093.3	POLR2E	1.048
IPI00927876.1	POLR2H	1.094
I00003311.1;P628	POLR2L	0.773
IPI00024163.1	POLR3A	1.39725
IPI00215978.4	POLR3D	1.42375
IPI00298738.3	POLRMT	1.77
IPI00293331.3	POP1	-1.06775
IPI00470467.5	POR	0.73825
IPI00015018.1	PPA1	-0.81875
IPI00219793.1	PPAN	1.30175

AccsessionNo	GeneSymbol	hESCs/HFs
IPI00328987.3	BYSL	0.55775
IPI00329036.2	MRPL50	-0.3495
IPI00329331.6	UGP2	1.529
IPI00329332.1	STX12	-2.93875
IPI00329389.8	RPL6P27	-0.38075
IPI00329536.2	EEA1	-2.80575
IPI00329572.4	PACSIN3	0.803
IPI00329594.4	CCDC59	2.42825
IPI00329629.6	DNAJC7	-0.8075
IPI00329633.5	TARS	-0.44175
IPI00329672.4	MYO1E	-1.00425
IPI00329692.3	NMT1	-0.513
IPI00329791.12	DDX46	0.61625
IPI00332371.9	PFKL	-0.4395
IPI00332428.5	MAK16	1.11475
IPI00332511.5;Q6P1	PPP2R2A	-0.425
IPI00332572.3	BANP	1.68625
IPI00333015.7	SPTBN1	2.5415
IPI00333068.4	ARPC1A	-1.704
IPI00333619.4	ALDH3A2	3.67475
IPI00333637.1	GOLGA5	-0.0595
IPI00333699.5	SAP30BP	0.93525
IPI00333763.7	GLRX5	1.47375
IPI00334159.6	VBP1	-0.68075
IPI00334190.4	STOML2	0.51975
IPI00334282.2	FAM3C	-0.86925
IPI00334291.9	LONP1	1.3175
IPI00334657.7	YIPF5	-2.001
IPI00334713.1	HNRNPAB	1.744

AccessionNo	GeneSymbol	hiPSCs/hESCs
IPI00329036.2	MRPL50	0.60325
IPI00329331.6	UGP2	0.3935
IPI00329332.1	STX12	-0.189
IPI00329389.8	RPL6P27	0.34225
IPI00329536.2	EEA1	0.254
IPI00329572.4	PACSIN3	0.18
IPI00329594.4	CCDC59	0.04925
IPI00329629.6	DNAJC7	0.8625
IPI00329633.5	TARS	0.44375
IPI00329672.4	MYO1E	0.64925
IPI00329692.3	NMT1	0.1835
IPI00329791.12	DDX46	0.16375
IPI00332371.9	PFKL	0.0135
IPI00332428.5	MAK16	0.3425
IPI00332511.5;Q6P1	PPP2R2A	0.4475
IPI00332572.3	BANP	-0.03875
IPI00333015.7	SPTBN1	0.192
IPI00333068.4	ARPC1A	0.74575
IPI00333619.4	ALDH3A2	0.791
IPI00333637.1	GOLGA5	-0.46225
IPI00333699.5	SAP30BP	0.3905
IPI00333763.7	GLRX5	0.113
IPI00334159.6	VBP1	0.47075
IPI00334190.4	STOML2	0.082
IPI00334282.2	FAM3C	1.08425
IPI00334291.9	LONP1	-0.262
IPI00334657.7	YIPF5	0.14075
IPI00334713.1	HNRNPAB	0.23975
IPI00334914.2	TRMT1L	-0.1765

Table S4 The integrated datasets for the comparison of hiPSCs/HFs, hESCs/HFs, and hiPSCs/hESCs from the public datasets.

AccessionNo	GeneSymbol	hiPSCs/HFs
IPI00029534.1	PPAT	2.54425
IPI00219754.1	PPFIA1	-0.54225
IPI00917244.1	PPHLN1	0.34725
IPI00419585.9	PPIA	-0.412
IPI00646304.4	PPIB	-1.30725
IPI00003927.5	PPID	0.87375
IPI00409606.1	PPIE	0.608
IPI00026519.1	PPIF	-0.32125
IPI00643915.1	PPIH	1.04875
IPI00007019.1	PPIL1	0.9045
IPI00300952.3	PPIL3	0.496
IPI00642862.1	PPIL4	0.33375
IPI00178375.3	PPIP5K2	-0.95925
IPI00298057.2	PPL	0.777
IPI00291412.1	PPM1F	-2.89375
IPI00006167.1	PPM1G	0.36325
IPI00007694.5	PPME1	-1.854
IPI00031357.1	PPOX	1.08725
IPI00550451.1	PPP1CA	-0.6995
I00218236.6;P621	PPP1CB	-0.9195
IPI00298731.2	PPP1R10	0.8335
IPI00395771.1	PPP1R7	-1.664
IPI00030383.1	PPP1R8	0.73575
IPI00045550.4	PPP1R9B	-2.7055
IPI00008380.1	PPP2CA	0.0725
IPI00429689.3	PPP2CB	-1.42275
I00332511.5;Q6P1	PPP2R2A	-0.44675
IPI00014978.3	PPP2R5A	-0.10725
IPI00002853.1	PPP2R5E	-0.334

AccsessionNo	GeneSymbol	hESCs/HFs
IPI00334914.2	TRMT1L	1.558
IPI00335385.4	DCPS	-0.3215
IPI00335589.5	RNMTL1	1.8055
IPI00335930.1	DAZAP1	1.622
IPI00336016.3	CARS2	-0.72275
IPI00336017.5	ARRB1	1.7145
IPI00337541.3	NNT	-1.85325
IPI00337588.1	TP53BP2	-0.019
IPI00337741.4	APEH	0.51125
IPI00339238.1	DHRS7	-0.2475
IPI00339384.5	RDH11	-0.111
IPI00373877.3	ZNF326	1.502
IPI00374151.1	PRDX3	0.8265
IPI00374563.3	AGRN	1.439
IPI00375145.1	USP5	-0.953
IPI00375294.3	LAMA1	2.051
IPI00375330.6	WAPAL	1.5675
IPI00375359.2	RFC1	1.77125
IPI00375441.2	FUBP1	-0.01
IPI00375462.1	SREK1	0.2765
IPI00375533.5	UBA3	0.055
IPI00375631.6	ISG15	-2.18075
IPI00375676.8	FTL	-0.305
IPI00376144.3	LRCH1	-2.28075
IPI00376222.1	YTHDC1	1.551
IPI00376226.2	SIGMAR1	0.103
IPI00376283.1	RBBP6	1.275
IPI00376394.4	QSOX2	0.332
IPI00376403.2	SPINT1	1.958

AccessionNo	GeneSymbol	hiPSCs/hESCs
IPI00335385.4	DCPS	0.15575
IPI00335589.5	RNMTL1	-0.38475
IPI00335930.1	DAZAP1	0.062
IPI00336016.3	CARS2	0.04425
IPI00336017.5	ARRB1	0.31675
IPI00337541.3	NNT	0.19125
IPI00337588.1	TP53BP2	0.301
IPI00337741.4	APEH	0.38825
IPI00339238.1	DHRS7	-0.6035
IPI00339384.5	RDH11	0.77825
IPI00373877.3	ZNF326	0.194
IPI00374151.1	PRDX3	-0.19625
IPI00374563.3	AGRN	0.39125
IPI00375145.1	USP5	0.16775
IPI00375294.3	LAMA1	0.2665
IPI00375330.6	WAPAL	-0.0215
IPI00375359.2	RFC1	0.6265
IPI00375441.2	FUBP1	0.20325
IPI00375462.1	SREK1	0.49025
IPI00375533.5	UBA3	0.31575
IPI00375631.6	ISG15	0.5725
IPI00375676.8	FTL	0.20975
IPI00376144.3	LRCH1	0.41425
IPI00376222.1	YTHDC1	0.1045
IPI00376226.2	SIGMAR1	-0.1855
IPI00376283.1	RBBP6	0.32925
IPI00376394.4	QSOX2	0.51375
IPI00376403.2	SPINT1	0.58625
IPI00376609.1	NUP155	-0.02875

Table S4 The integrated datasets for the comparison of hiPSCs/HFs, hESCs/HFs, and hiPSCs/hESCs from the public datasets.

AccessionNo	GeneSymbol	hiPSCs/HFs
IPI00103654.2	PPP4R2	0.553
IPI00019812.1	PPP5C	-0.24925
IPI00890827.1	PPP6C	-0.1765
IPI00873586.3	PPP6R1	-1.22175
IPI00002412.1	PPT1	2.2495
IPI00149650.3	PPWD1	1.212
IPI00954553.1	PQBP1	0.8955
IPI00399306.1	PRCC	0.60275
IPI00001593.1	PRCP	0.01475
IPI00374151.1	PRDX3	0.3375
IPI00011937.1	PRDX4	-0.1045
IPI00759663.1	PRDX5	-0.442
IPI00220301.5	PRDX6	-1.1255
IPI00008164.2	PREP	0.31275
IPI00027705.2	PRIM2	2.19575
IPI00792482.1	PRKAA1	-2.96975
IPI00021831.1	PRKAR1A	-3.61775
IPI00063234.1	PRKAR2A	-1.68275
IPI00554752.3	PRKAR2B	0.7085
IPI00385449.4	PRKCA	-3.9405
IPI00792364.1	PRKCD	0.296
IPI00016639.7	PRKCI	-0.0655
IPI00792916.2	PRKCSH	-0.66175
IPI00296337.2	PRKDC	1.11225
IPI00914913.1	PRMT1	0.146
IPI00383798.3	PRODH	0.23425
IPI00004968.1	PRPF19	0.99
IPI00005861.1	PRPF3	0.924
IPI00171390.2	PRPF38A	1.59475

AccsessionNo	GeneSymbol	hESCs/HFs
IPI00376609.1	NUP155	1.488
IPI00377080.1	COPS8	-1.32925
IPI00377261.1	FUBP3	0.585
IPI00382452.1	CHMP1A	-0.69225
IPI00382458.1	CUL3	-0.05525
IPI00383163.5	THYN1	2.1485
IPI00383296.5	HNRNPM	1.2775
IPI00383423.3	CCDC50	-3.8185
IPI00383449.2	RAB15	-1.0855
IPI00383565.5	BAZ1A	0.172
IPI00383581.4	GANAB	0.24075
IPI00383798.3	PRODH	2.91925
IPI00384028.5	PAPOLA	0.868
IPI00384155.1	LGMN	0.841
IPI00384280.5	PCYOX1	-0.37325
IPI00384456.4	MSH6	3.00575
IPI00384542.4	NID1	1.51325
IPI00384722.2	C19orf63	-0.348
IPI00384745.4	GNL1	-1.12875
IPI00385042.4	GTPBP4	0.62025
IPI00385267.4	SRPR	-1.707
IPI00385449.4	PRKCA	-3.29975
IPI00385928.2	C4orf14	0.394
IPI00386120.3	SF1	0.45825
IPI00386206.2	PRRC1	-1.85825
IPI00386294.2	TOMM6	1.40975
IPI00386718.4;IPI00513	SMARCA2	-1.23975
IPI00386755.2	ERO1L	-0.34025
IPI00394737.1	OSBPL8	-0.21825

AccessionNo	GeneSymbol	hiPSCs/hESCs
IPI00377080.1	COPS8	0.43875
IPI00377261.1	FUBP3	0.03375
IPI00382452.1	CHMP1A	0.30575
IPI00382458.1	CUL3	0.292
IPI00383163.5	THYN1	0.0285
IPI00383296.5	HNRNPM	0.30625
IPI00383423.3	CCDC50	0.4835
IPI00383449.2	RAB15	0.1905
IPI00383565.5	BAZ1A	1.332
IPI00383581.4	GANAB	0.55525
IPI00383798.3	PRODH	-2.08
IPI00384028.5	PAPOLA	0.24
IPI00384155.1	LGMN	0.66975
IPI00384280.5	PCYOX1	-0.21025
IPI00384456.4	MSH6	0.07
IPI00384542.4	NID1	0.023
IPI00384722.2	C19orf63	0.033
IPI00384745.4	GNL1	0.61325
IPI00385042.4	GTPBP4	0.6955
IPI00385267.4	SRPR	0.4665
IPI00385449.4	PRKCA	-0.32925
IPI00385928.2	C4orf14	0.8695
IPI00386120.3	SF1	0.36725
IPI00386206.2	PRRC1	0.4675
IPI00386294.2	TOMM6	-0.122
IPI00386718.4;IPI00513	SMARCA2	0.31425
IPI00386755.2	ERO1L	-0.40575
IPI00394737.1	OSBPL8	0.30025
IPI00394788.4	AARS2	-0.46925

Table S4 The integrated datasets for the comparison of hiPSCs/HFs, hESCs/HFs, and hiPSCs/hESCs from the public datasets.

AccessionNo	GeneSymbol	hiPSCs/HFs
IPI00218435.1	PRPF4	0.917
IPI00013721.2	PRPF4B	0.604
IPI00305068.5	PRPF6	1.07825
IPI00007928.4	PRPF8	1.2065
IPI00219616.7	PRPS1	-0.06075
IPI00219617.5	PRPS2	-0.3695
IPI00003168.1	PRPSAP2	0.37125
IPI00855833.1	PRR12	1.64
IPI00386206.2	PRRC1	-1.68475
IPI00010700.2	PRRC2A	0.46875
IPI00741537.5	PRRC2B	-0.0525
IPI00012503.1	PSAP	0.50975
IPI00930609.1	PSAT1	0.9545
IPI00028122.1	PSIP1	3.62875
IPI00016832.1	PSMA1	-0.79775
IPI00219622.3	PSMA2	-0.4785
IPI00171199.5	PSMA3	-0.39625
I00291922.2;Q9Z2	PSMA5	-0.645
IPI00029623.1	PSMA6	-0.42575
IPI00024175.3	PSMA7	-0.3905
IPI00025019.3	PSMB1	-0.4825
IPI00028006.1	PSMB2	-0.511
IPI00028004.2	PSMB3	-0.4385
IPI00555956.2	PSMB4	-0.58725
IPI00479306.1	PSMB5	-0.3445
IPI00003217.3	PSMB7	-0.42825
I00011126.6;P621	PSMC1	-0.79925
IPI00021435.3	PSMC2	-0.72675
IPI00018398.4	PSMC3	-0.58475

AccsessionNo	GeneSymbol	hESCs/HFs
IPI00394788.4	AARS2	1.48875
IPI00394926.1	POLD3	1.49825
IPI00395462.4	C15orf63	-0.53925
IPI00395474.1	SAMD1	2.08325
IPI00395627.3	CACYBP	0.22575
IPI00395646.1	TXNDC5	-1.837
IPI00395663.5	ANKS1A	0.80675
IPI00395674.1	SNRPB	1.02775
IPI00395769.2	ATP5C1	0.477
IPI00395771.1	PPP1R7	-1.97575
IPI00395777.6	CNOT7	0.58575
IPI00395887.4	TMX1	0.648
IPI00395903.1	TMEM106B	-0.14575
IPI00396056.6	NUDT1	0.80375
IPI00396321.1	LRRC59	-1.73675
IPI00396370.6	EIF3B	-0.68475
IPI00396378.3	HNRNPA2B1	1.29575
IPI00396411.4	CLPTM1	-1.53675
IPI00396435.3	DHX15	1.3925
IPI00396485.3	EEF1A1	-1.39025
IPI00396627.1	ELAC2	0.685
IPI00396661.3	CYP2S1	3.916
IPI00397024.1	YTHDF2	0.4785
IPI00397365.1	EPN1	-1.64925
IPI00397526.3	MYH10	0.82325
IPI00397794.4	SCAF4	1.219
IPI00397904.6	NUP93	1.2715
IPI00398154.2	AFAP1	-3.0875
IPI00398214.2	FLAD1	0.70075

AccessionNo	GeneSymbol	hiPSCs/hESCs
IPI00394926.1	POLD3	0.7125
IPI00395462.4	C15orf63	0.0225
IPI00395474.1	SAMD1	0.292
IPI00395627.3	CACYBP	0.517
IPI00395646.1	TXNDC5	0.348
IPI00395663.5	ANKS1A	0.4165
IPI00395674.1	SNRPB	0.37025
IPI00395769.2	ATP5C1	-0.03375
IPI00395771.1	PPP1R7	0.58825
IPI00395777.6	CNOT7	0.21025
IPI00395887.4	TMX1	-0.09075
IPI00395903.1	TMEM106B	-0.226
IPI00396056.6	NUDT1	0.24225
IPI00396321.1	LRRC59	0.36775
IPI00396370.6	EIF3B	0.56875
IPI00396378.3	HNRNPA2B1	0.132
IPI00396411.4	CLPTM1	0.94
IPI00396435.3	DHX15	0.10025
IPI00396485.3	EEF1A1	0.45275
IPI00396627.1	ELAC2	0.6855
IPI00396661.3	CYP2S1	-0.0725
IPI00397024.1	YTHDF2	0.31575
IPI00397365.1	EPN1	0.6705
IPI00397526.3	MYH10	0.27725
IPI00397794.4	SCAF4	-0.00825
IPI00397904.6	NUP93	0.0555
IPI00398154.2	AFAP1	-0.441
IPI00398214.2	FLAD1	0.17
IPI00398727.3	LYPLA1	-0.05375

Table S4 The integrated datasets for the comparison of hiPSCs/HFs, hESCs/HFs, and hiPSCs/hESCs from the public datasets.

AccessionNo	GeneSymbol	hiPSCs/HFs
IPI00745502.2	PSMC5	-0.66575
926.4;P62334;IPI00	PSMC6	-0.548
IPI00027699.2	PSMD10	-0.05
IPI00105598.3	PSMD11	-0.72175
IPI00185374.4	PSMD12	-0.55875
IPI00549672.2	PSMD13	-0.648
IPI00024821.1	PSMD14	-0.70625
IPI00012268.3	PSMD2	-0.80325
IPI00011603.2	PSMD3	-0.578
IPI00022694.3	PSMD4	-0.795
IPI00216247.2	PSMD4	-1.166
IPI00909766.1	PSMD5	-2.161
IPI00014151.3	PSMD6	-0.589
IPI00019927.2	PSMD7	-0.648
IPI00479722.2	PSME1	-1.73475
IPI00746205.1	PSME2	-1.6845
I00030243.1;P612	PSME3	-0.05175
IPI00827831.3	PSMF1	-1.60425
IPI00401072.1	PSMG1	-0.2195
IPI00031106.1	PSMG3	0.01075
IPI00103525.1	PSPC1	1.99525
IPI00925737.1	PSPH	-0.215
IPI00179964.5	PTBP1	1.11925
IPI00514064.1	PTBP2	2.546
IPI00783302.1	PTCD3	0.75325
IPI00006538.1	PTDSS2	0.00575
IPI00303568.3	PTGES2	-0.35525
IPI00550020.3	PTMS	-1.0485
IPI00938044.1	PTPLAD1	0.88975

AccsessionNo	GeneSymbol	hESCs/HFs
IPI00398727.3	LYPLA1	1.27575
IPI00399089.4	MESDC2	-0.71
IPI00399105.1	EDIL3	-0.65675
IPI00399170.1	UPF1	-0.68425
IPI00399300.1	TUBGCP3	-0.62625
IPI00399306.1	PRCC	0.50075
IPI00400849.2	PICALM	-1.697
IPI00400922.5	PDCD11	1.58675
IPI00401072.1	PSMG1	-0.55
IPI00401264.5	ERP44	-0.79025
IPI00401804.3	VAMP7	-1.31325
IPI00401962.2	CCDC137	-0.4865
IPI00402109.4	PIGU	0.18875
IPI00409606.1	PPIE	0.186
IPI00409671.3	DDX42	0.98025
IPI00410034.2	SLC38A2	-2.11725
IPI00410079.3	FAM82A2	-0.207
IPI00410112.2	VKORC1	-0.48475
IPI00410360.3	NGDN	1.83475
IPI00411356.5	VPS4A	-0.975
IPI00411426.3	VPS26A	-2.16675
IPI00411614.1	WDHD1	2.30375
IPI00411623.3	ENAH	0.9775
IPI00411680.10	PCMT1	-1.0805
IPI00411706.1	ESD	-2.43975
IPI00411886.4	NOC2L	2.23025
IPI00411937.4	SNORD110	1.151
IPI00412147.1	SLC27A4	-0.6445
IPI00412404.1	SUPV3L1	0.95775

AccessionNo	GeneSymbol	hiPSCs/hESCs
IPI00399089.4	MESDC2	0.3145
IPI00399105.1	EDIL3	0.43675
IPI00399170.1	UPF1	0.3885
IPI00399300.1	TUBGCP3	0.37375
IPI00399306.1	PRCC	0.4395
IPI00400849.2	PICALM	0.331
IPI00400922.5	PDCD11	0.309
IPI00401072.1	PSMG1	0.6015
IPI00401264.5	ERP44	0.03525
IPI00401804.3	VAMP7	1.20175
IPI00401962.2	CCDC137	0.93225
IPI00402109.4	PIGU	0.23575
IPI00409606.1	PPIE	0.55575
IPI00409671.3	DDX42	0.1835
IPI00410034.2	SLC38A2	2.23325
IPI00410079.3	FAM82A2	0.08625
IPI00410112.2	VKORC1	-0.5895
IPI00410360.3	NGDN	-0.0665
IPI00411356.5	VPS4A	0.4365
IPI00411426.3	VPS26A	0.445
IPI00411614.1	WDHD1	-0.08875
IPI00411623.3	ENAH	0.32325
IPI00411680.10	PCMT1	0.374
IPI00411706.1	ESD	0.92575
IPI00411886.4	NOC2L	-0.30725
IPI00411937.4	SNORD110	0.39925
IPI00412147.1	SLC27A4	0.177
IPI00412404.1	SUPV3L1	-0.15825
IPI00412579.6	RPL10A	0.36675

Table S4 The integrated datasets for the comparison of hiPSCs/HFs, hESCs/HFs, and hiPSCs/hESCs from the public datasets.

AccessionNo	GeneSymbol	hiPSCs/HFs
IPI00297261.3	PTPN1	-1.14175
IPI00018914.2	PTPN14	-0.89675
IPI00852804.1	PTPN9	-0.80075
IPI00465186.3	PTPRF	0.89725
IPI00873341.2	PTPRG	0.75975
IPI00032903.3	PTRH2	0.352
IPI00788826.1	PUF60	0.544
IPI00023591.1	PURA	-3.405
IPI00045051.3	PURB	-1.6225
IPI00044761.4	PUS7	1.364
IPI00219426.1	PVR	-3.14825
IPI00300078.6	PWP2	1.80525
IPI00016112.6	PXDN	-1.22
IPI00221360.1	PYCARD	-0.8225
941557.1;IPI00550	PYCR1	0.3615
IPI00470610.4	PYCR2	0.79
IPI00004358.4	PYGB	-2.1315
IPI00943894.1	PYGL	-0.44875
IPI00759828.1	QKI	0.85575
IPI00300086.3	QPRT	2.04325
IPI00376394.4	QSOX2	0.64125
I00016513.5;P610	RAB10	-0.51025
IPI00020436.4	RAB11B	-0.14825
IPI00022033.6	RAB11FIP5	-4.502
IPI00419932.5	RAB12	-0.99575
IPI00016373.3	RAB13	0.00125
I00291928.8;Q91V	RAB14	-1.44
IPI00383449.2	RAB15	-1.08275
IPI00014577.1	RAB18	-1.069

AccsessionNo	GeneSymbol	hESCs/HFs
IPI00412579.6	RPL10A	-0.48575
IPI00412713.4	SAMM50	0.60325
IPI00412752.3	STAT3	-0.9155
IPI00412771.1	CD2AP	0.324
IPI00412880.2	CARM1	-0.1885
IPI00413324.6	RPL17	-0.611
IPI00413344.3	CFL2	-4.8395
IPI00413568.2	CLCN3	-0.066
IPI00413611.1	TOP1	0.8475
IPI00413641.7	AKR1B1	-0.71325
IPI00413672.1	BCLAF1	1.00225
IPI00413780.3	PKN3	2.614
IPI00414123.4	CRMP1	2.5325
IPI00414442.3	CDK5RAP3	-0.986
IPI00414554.5	ARPC5L	-1.03775
IPI00414629.2	UNC119B	0.489
IPI00414676.6	HSP90AB1	0.4875
IPI00414694.3	COL18A1	1.43175
IPI00414709.3	RBPMS	1.91325
IPI00414827.1	SRGAP1	-3.76425
IPI00414836.6	OSTF1	-2.66175
IPI00414896.1	RNASET2	2.3105
IPI00414909.1	NAGA	-0.259
IPI00414963.2	PABPN1	1.747
IPI00414973.1	FASTKD5	1.5465
IPI00415014.3	MAP1LC3A	-2.8955
IPI00415040.1	NUDT9	0.21625
IPI00418262.5	ALDOC	0.8185
IPI00418290.1	MRPL14	-0.13875

AccessionNo	GeneSymbol	hiPSCs/hESCs
IPI00412713.4	SAMM50	-0.056
IPI00412752.3	STAT3	0.348
IPI00412771.1	CD2AP	0.59025
IPI00412880.2	CARM1	0.1605
IPI00413324.6	RPL17	0.44075
IPI00413344.3	CFL2	0.1725
IPI00413568.2	CLCN3	-0.22875
IPI00413611.1	TOP1	0.669
IPI00413641.7	AKR1B1	0.38525
IPI00413672.1	BCLAF1	0.3385
IPI00413780.3	PKN3	0.25925
IPI00414123.4	CRMP1	0.9035
IPI00414442.3	CDK5RAP3	0.00775
IPI00414554.5	ARPC5L	0.37325
IPI00414629.2	UNC119B	0.02775
IPI00414676.6	HSP90AB1	0.411
IPI00414694.3	COL18A1	0.52675
IPI00414709.3	RBPMS	-0.073
IPI00414827.1	SRGAP1	0.36475
IPI00414836.6	OSTF1	0.92625
IPI00414896.1	RNASET2	-0.869
IPI00414909.1	NAGA	0.06
IPI00414963.2	PABPN1	0.21575
IPI00414973.1	FASTKD5	0.0995
IPI00415014.3	MAP1LC3A	0.021
IPI00415040.1	NUDT9	-0.10525
IPI00418262.5	ALDOC	0.20075
IPI00418290.1	MRPL14	0.65725
IPI00418313.3	ILF3	0.30525

Table S4 The integrated datasets for the comparison of hiPSCs/HFs, hESCs/HFs, and hiPSCs/hESCs from the public datasets.

AccessionNo	GeneSymbol	hiPSCs/HFs
I00005719.1;P628	RAB1A	-1.0835
IPI00008964.3	RAB1B	-1.28775
IPI00007755.3	RAB21	-1.57175
IPI00008034.1	RAB23	-1.0475
IPI00031169.1	RAB2A	-1.3755
IPI00021475.1	RAB33B	-0.71225
00300096.4;Q6PH	RAB35	-0.82675
IPI00023504.1	RAB3A	-1.2025
IPI00300562.2	RAB3B	-2.4965
IPI00014235.3	RAB3GAP1	-1.71625
IPI00554590.1	RAB3GAP2	-1.3865
IPI00939242.1	RAB4A	-1.095
IPI00927674.1	RAB5A	-0.3515
I00017344.3;P610	RAB5B	-1.6075
IPI00016339.4	RAB5C	-2.2295
IPI00016342.1	RAB7A	-0.67225
IPI00028481.1	RAB8A	-1.00175
IPI00024282.1	RAB8B	-0.26625
IPI00016372.1	RAB9A	-0.06975
IPI00022664.3	RABGGTA	-1.639
IPI00514956.1	RABGGTB	-1.00025
I00010271.3;P630	RAC1	-1.18725
IPI00152946.1	RACGAP1	1.077
IPI00006715.3	RAD21	1.82075
IPI00008219.1	RAD23A	-1.8445
IPI00642549.2	RAD23B	-1.349
IPI00305282.2	RAD50	0.48625
IPI00019733.1	RAE1	0.6265
IPI00217519.3	RALA	0.07475

AccsessionNo	GeneSymbol	hESCs/HFs
IPI00418313.3	ILF3	1.07775
IPI00418336.3	INTS3	0.54275
IPI00418471.6	VIM	-5.5855
IPI00419258.4	HMGB1	0.30375
IPI00419263.4	ECI2	0.43825
IPI00419473.5	BTF3	-1.009
IPI00419531.2	CPSF2	1.40175
IPI00419585.9	PPIA	-0.54475
IPI00419802.4	HIBCH	1.02325
IPI00419856.1	TNPO2	0.19125
IPI00419880.6	SNORD73A	-0.58925
IPI00419916.4	ALPL	2.18275
IPI00419919.5	RPL29	-0.4595
IPI00419932.5	RAB12	-0.0735
IPI00419979.3	PAK2	-1.78275
IPI00420014.2	SNRNP200	1.42675
IPI00420108.5	DLST	0.3845
IPI00423570.3	KRAS	-0.44425
IPI00425560.2	SCRIB	0.19975
IPI00426640.1	GTF2H2	0.3575
IPI00427330.3	SBDS	-3.4075
IPI00428288.1	MRPL43	0.18925
IPI00429191.3	ETF1	-1.3585
IPI00429689.3	PPP2CB	-1.8505
IPI00430472.2	ASCC3	-0.851
IPI00430622.1	SPG20	0.15725
IPI00432065.1	C11ORF83	2.7105
IPI00433194.2	EHBP1	-2.0545
IPI00433833.1	THOC3	1.9425

AccessionNo	GeneSymbol	hiPSCs/hESCs
IPI00418336.3	INTS3	0.52025
IPI00418471.6	VIM	1.006
IPI00419258.4	HMGB1	0.4285
IPI00419263.4	ECI2	-0.51875
IPI00419473.5	BTF3	0.83125
IPI00419531.2	CPSF2	0.3435
IPI00419585.9	PPIA	0.451
IPI00419802.4	HIBCH	0.19675
IPI00419856.1	TNPO2	-0.251
IPI00419880.6	SNORD73A	0.47175
IPI00419916.4	ALPL	0.6995
IPI00419932.5	RAB12	-0.468
IPI00419979.3	PAK2	0.56775
IPI00420014.2	SNRNP200	0.15875
IPI00420108.5	DLST	-0.561
IPI00423570.3	KRAS	0.59875
IPI00425560.2	SCRIB	0.02975
IPI00426640.1	GTF2H2	0.0805
IPI00427330.3	SBDS	0.85975
IPI00428288.1	MRPL43	0.55125
IPI00429191.3	ETF1	0.7695
IPI00429689.3	PPP2CB	0.709
IPI00430472.2	ASCC3	0.414
IPI00430622.1	SPG20	0.22425
IPI00432065.1	C11ORF83	-0.40325
IPI00433194.2	EHBP1	0.28525
IPI00433833.1	THOC3	-0.35075
IPI00434390.2	ASCC1	0.222
IPI00438229.2	TRIM28	0.09925

Table S4 The integrated datasets for the comparison of hiPSCs/HFs, hESCs/HFs, and hiPSCs/hESCs from the public datasets.

AccessionNo	GeneSymbol	hiPSCs/HFs
IPI00166137.5	RALYL	0.8165
IPI00878075.1	RANBP1	0.18425
IPI00221325.3	RANBP2	1.44875
IPI00456729.1	RANBP3	-0.44575
IPI00294879.1	RANGAP1	-0.06675
I00643041.3;P628	RANP1	0.453
I00019345.1;P628	RAP1A	-1.64825
IPI00607591.3	RAP1GDS1	-3.0805
IPI00004860.2	RARS	-0.786
IPI00941179.1	RASA1	-2.483
IPI00217661.4	RAVER1	1.2185
IPI00645329.1	RBBP4	1.861
IPI00376283.1	RBBP6	1.365
IPI00879702.2	RBBP7	0.6395
IPI00885127.2	RBFOX2	0.303
IPI00550308.1	RBM12	0.3905
IPI00013174.2	RBM14	1.1795
IPI00220716.2	RBM15	1.48975
IPI00175136.5	RBM15B	1.12425
IPI00829652.1	RBM16	0.69175
IPI00176706.1	RBM17	1.184
IPI00000686.2	RBM19	1.1205
IPI00004273.6	RBM25	1.17475
IPI00292975.4	RBM27	1.27225
IPI00304187.8	RBM28	1.3045
IPI00024320.1	RBM3	-2.06
IPI00658072.2	RBM34	1.24875
IPI00215801.1	RBM39	1.13975
IPI00003704.4	RBM4	1.742

AccsessionNo	GeneSymbol	hESCs/HFs
IPI00434390.2	ASCC1	-1.03425
IPI00438229.2	TRIM28	2.074
IPI00439195.1	MBD3	2.4485
IPI00439936.1	TAPBP	-0.75925
IPI00440493.2	ATP5A1	0.548
IPI00440719.2	NAA10	-0.865
IPI00440727.1	BRD4	1.50475
IPI00442073.5	CSRP1	-3.908
IPI00442211.1	ABI2	-0.17075
IPI00442274.3	NFXL1	1.0605
IPI00443909.1	CNPY2	0.11025
IPI00445401.3	HUWE1	0.19275
IPI00446235.2	CYB5R3	-2.64225
IPI00449049.5	PARP1	3.49425
IPI00449201.2	ATG3	-2.062
IPI00452731.6	NDUFA7	-0.29
IPI00452919.3	SS18	1.03825
IPI00453473.6;P628	HIST2H4B	1.532
IPI00455190.2	COX15	0.6025
IPI00455268.4	TRMT5	0.57825
IPI00455315.4	ANXA2	-5.80875
IPI00455383.4	CLTC	-1.14725
IPI00456359.1	ATXN2L	-0.335
IPI00456492.2	CROCC	1.525
IPI00456631.5	KDM1A	2.426
IPI00456663.1	NIT1	0.02425
IPI00456685.2	EIF2AK4	-0.254
IPI00456729.1	RANBP3	-0.58575
IPI00456750.2	FAM129B	-4.3225

AccessionNo	GeneSymbol	hiPSCs/hESCs
IPI00439195.1	MBD3	-0.13075
IPI00439936.1	TAPBP	-1.8275
IPI00440493.2	ATP5A1	-0.17575
IPI00440719.2	NAA10	0.59625
IPI00440727.1	BRD4	0.745
IPI00442073.5	CSRP1	-0.3045
IPI00442211.1	ABI2	0.46175
IPI00442274.3	NFXL1	0.05475
IPI00443909.1	CNPY2	0.2075
IPI00445401.3	HUWE1	0.037
IPI00446235.2	CYB5R3	0.42125
IPI00449049.5	PARP1	-0.39925
IPI00449201.2	ATG3	0.94575
IPI00452731.6	NDUFA7	0.3745
IPI00452919.3	SS18	0.42625
IPI00453473.6;P628	HIST2H4B	0.28775
IPI00455190.2	COX15	-0.03825
IPI00455268.4	TRMT5	0.1065
IPI00455315.4	ANXA2	1.139
IPI00455383.4	CLTC	0.12025
IPI00456359.1	ATXN2L	0.4715
IPI00456492.2	CROCC	0.54975
IPI00456631.5	KDM1A	0.1605
IPI00456663.1	NIT1	0.12475
IPI00456685.2	EIF2AK4	-0.37775
IPI00456729.1	RANBP3	0.424
IPI00456750.2	FAM129B	0.441
IPI00456758.4	RPL27A	0.3065
IPI00456887.2	HNRNPUL2	-0.099

Table S4 The integrated datasets for the comparison of hiPSCs/HFs, hESCs/HFs, and hiPSCs/hESCs from the public datasets.

AccessionNo	GeneSymbol	hiPSCs/HFs
IPI00027838.1	RBM4B	0.72175
IPI00005036.1	RBM5	0.605
00001757.1;Q9CW	RBM8A	0.9645
IPI00304692.1	RBMX	1.611
IPI00032597.2	RBMX2	1.8655
IPI00061178.1	RBMXL1	2.2035
IPI00940513.1	RBP1	2.09825
IPI00414709.3	RBPMS	1.65575
IPI00747309.1	RCC1	1.44725
IPI00465044.2	RCC2	3.53625
IPI00294229.6	RCL1	1.48525
IPI00015842.1	RCN1	-1.87125
IPI00029628.1	RCN2	0.392
IPI00101037.3	RCN3	-4.87875
IPI00008531.1	RCOR1	1.11825
IPI00217874.2	RCOR2	2.80175
IPI00339384.5	RDH11	0.44525
IPI00177940.2	RDH14	-0.28625
IPI00903145.1	RDX	-1.31525
IPI00872438.1	RECQL	-1.228
IPI00883897.1	RELA	-3.43375
IPI00375359.2	RFC1	2.02975
IPI00017412.1	RFC2	1.527
IPI00792595.2	RFC4	2.12225
IPI00099996.2	RG9MTD1	1.421
IPI00478231.2	RHOA	-0.443
IPI00647268.1	RHOC	-3.72575
IPI00017342.1	RHOG	-1.68025
IPI00759775.1	RHOT1	0.59275

AccsessionNo	GeneSymbol	hESCs/HFs
IPI00456758.4	RPL27A	-0.479
IPI00456887.2	HNRNPUL2	0.67075
IPI00456925.3	DBNL	-3.163
IPI00456940.5	RPL7L1	0.543
IPI00456965.5	COQ5	2.28075
IPI00456969.1	DYNC1H1	-1.4145
IPI00465044.2	RCC2	3.94925
IPI00465132.4	COPE	-2.00875
IPI00465186.3	PTPRF	0.87975
IPI00465248.5	ENO1	-0.27575
IPI00465256.4	AK3	0.23325
IPI00465361.4	RPL13	-0.394
IPI00465431.8	LGALS3	-5.038
IPI00465436.4	CAT	-3.26225
IPI00465439.5	ALDOA	-1.0605
IPI00470416.2	MTPAP	1.43125
IPI00470467.5	POR	1.11175
IPI00470489.1	SMARCE1	1.5565
IPI00470610.4	PYCR2	0.88125
IPI00470631.2	COQ9	1.56825
IPI00470674.5	CYB5R1	-1.68225
IPI00470779.2	TXLNA	-1.75775
IPI00470921.4	ADRM1	-0.78075
IPI00470924.2	TMTC3	-0.39275
IPI00472054.2	FAM120A	-3.2875
IPI00472058.1	NDUFB11	-0.38125
IPI00472242.3	SCAMP4	-1.52125
IPI00472498.3	TMEM189	-1.4225
IPI00472583.2	TARBP2	1.1935

AccessionNo	GeneSymbol	hiPSCs/hESCs
IPI00456925.3	DBNL	0.80125
IPI00456940.5	RPL7L1	0.76525
IPI00456965.5	COQ5	-0.5995
IPI00456969.1	DYNC1H1	0.31025
IPI00465044.2	RCC2	-0.02975
IPI00465132.4	COPE	0.45075
IPI00465186.3	PTPRF	0.1545
IPI00465248.5	ENO1	0.28625
IPI00465256.4	AK3	-0.56575
IPI00465361.4	RPL13	0.41425
IPI00465431.8	LGALS3	-0.5715
IPI00465436.4	CAT	-0.803
IPI00465439.5	ALDOA	0.417
IPI00470416.2	MTPAP	0.14075
IPI00470467.5	POR	-0.07075
IPI00470489.1	SMARCE1	0.1475
IPI00470610.4	PYCR2	0.20725
IPI00470631.2	COQ9	-0.383
IPI00470674.5	CYB5R1	-0.75275
IPI00470779.2	TXLNA	0.41725
IPI00470921.4	ADRM1	0.321
IPI00470924.2	TMTC3	0.22375
IPI00472054.2	FAM120A	0.44175
IPI00472058.1	NDUFB11	0.5515
IPI00472242.3	SCAMP4	0.394
IPI00472498.3	TMEM189	0.7815
IPI00472583.2	TARBP2	0.33375
IPI00472887.3	CKAP5	0.11825
IPI00477001.3	STRN3	0.21475

Table S4 The integrated datasets for the comparison of hiPSCs/HFs, hESCs/HFs, and hiPSCs/hESCs from the public datasets.

AccessionNo	GeneSymbol	hiPSCs/HFs
IPI00749304.1	RIC8A	-1.787
IPI00930723.1	RIOK2	-1.75875
IPI00290192.1	RNASEH2A	1.37475
IPI00414896.1	RNASET2	0.99025
IPI00908327.1	RNF114	-0.288
IPI00917561.1	RNF181	-1.1695
IPI00251559.8	RNF20	0.81675
IPI00828098.2	RNF213	-1.05275
IPI00550069.3	RNH1	-3.6855
IPI00747403.20	RNMT	0.82875
IPI00335589.5	RNMTL1	1.11975
IPI00953520.1	RNPS1	1.572
IPI00022542.1	ROCK1	-0.95525
IPI00307155.8	ROCK2	-2.28975
IPI00640632.1	ROD1	1.531
IPI00026627.4	RP2	-0.9495
IPI00020127.1	RPA1	1.41725
IPI00013939.3	RPA2	1.3665
IPI00017373.1	RPA3	1.53425
IPI00292221.3	RPF1	0.988
IPI00026513.6	RPIA	0.5495
IPI00412579.6	RPL10A	-0.43175
IPI00746438.2	RPL11	-0.30475
IPI00024933.3	RPL12	-0.46475
IPI00465361.4	RPL13	-0.2755
IPI00555744.6	RPL14	-0.2885
IPI00413324.6	RPL17	-0.41425
IPI00215719.6	RPL18	-0.38325
IPI00026202.1	RPL18A	-0.456

AccsessionNo	GeneSymbol	hESCs/HFs
IPI00472887.3	CKAP5	0.4345
IPI00477001.3	STRN3	-0.787
IPI00477139.1	SLC4A2	-0.13025
IPI00477295.4	DHX30	0.21975
IPI00477355.3	KIAA1279	-0.2585
IPI00477468.1	CTR9	0.77175
IPI00477686.2	GTF2F2	-0.31675
IPI00477759.1	INTS2	0.70175
IPI00477763.4	CDC42BPB	-1.453
IPI00477812.3	TRIM22	-0.3185
IPI00477831.2	ERAP1	-1.2865
IPI00477971.4	TBL3	1.8665
IPI00478231.2	RHOA	-0.22675
IPI00478296.3	TAF4	1.9465
IPI00478302.2	TP53I11	0.06175
IPI00478631.6	ZNF512	0.52125
IPI00478689.5	UNC13B	1.22225
IPI00478723.3	NRD1	-2.58175
IPI00479186.7;IPI00479	PKM2	-2.04875
IPI00479248.3	NFRKB	0.9275
IPI00479306.1	PSMB5	-0.7095
IPI00479676.5	SPAG9	-1.95625
IPI00479722.2	PSME1	-1.20825
IPI00479786.5	KHSRP	0.495
IPI00479823.3	RSF1	1.3155
IPI00479877.4	ALDH9A1	-0.90775
IPI00479890.4	SHOC2	-1.4215
IPI00479905.5	NDUFB10	-0.64625
IPI00479997.4	STMN1	-0.5315

AccessionNo	GeneSymbol	hiPSCs/hESCs
IPI00477139.1	SLC4A2	0.676
IPI00477295.4	DHX30	0.497
IPI00477355.3	KIAA1279	0.13925
IPI00477468.1	CTR9	0.1165
IPI00477686.2	GTF2F2	0.44325
IPI00477759.1	INTS2	0.43675
IPI00477763.4	CDC42BPB	0.6125
IPI00477812.3	TRIM22	0.582
IPI00477831.2	ERAP1	-0.62575
IPI00477971.4	TBL3	-0.091
IPI00478231.2	RHOA	0.088
IPI00478296.3	TAF4	-0.00075
IPI00478302.2	TP53I11	0.12875
IPI00478631.6	ZNF512	0.68525
IPI00478689.5	UNC13B	0.41425
IPI00478723.3	NRD1	1.038
IPI00479186.7;IPI00479	PKM2	0.47125
IPI00479248.3	NFRKB	0.59075
IPI00479306.1	PSMB5	0.654
IPI00479676.5	SPAG9	0.55725
IPI00479722.2	PSME1	-0.112
IPI00479786.5	KHSRP	0.222
IPI00479823.3	RSF1	-0.0155
IPI00479877.4	ALDH9A1	0.37275
IPI00479890.4	SHOC2	0.234
IPI00479905.5	NDUFB10	0.56675
IPI00479997.4	STMN1	0.847
IPI00514037.3	DDX59	-0.32775
IPI00514053.1	ARCN1	0.39275

Table S4 The integrated datasets for the comparison of hiPSCs/HFs, hESCs/HFs, and hiPSCs/hESCs from the public datasets.

AccessionNo	GeneSymbol	hiPSCs/HFs	AccsessionNo	GeneSymbol	hESCs/HFs	AccessionNo	GeneSymbol	hiPSCs/hESCs
I00025329.1;P840	RPL19	-0.497	IPI00514037.3	DDX59	1.79475	IPI00514064.1	PTBP2	0.00375
IPI00945264.1	RPL22L1	-1.85625	IPI00514053.1	ARCN1	-1.78225	IPI00514075.5	DIAPH2	0.18725
I00010153.5;P628	RPL23	-0.1945	IPI00514064.1	PTBP2	2.72475	IPI00514078.1	POGZ	0.13975
I00021266.1;P627	RPL23A	-0.2715	IPI00514075.5	DIAPH2	-0.26125	IPI00514126.2	AGL	0.4285
I00306332.4;Q8BP	RPL24	-0.389	IPI00514078.1	POGZ	2.6985	IPI00514250.1	CKS1B	0.42575
I00027270.1;P612	RPL26	-0.31325	IPI00514126.2	AGL	0.62825	IPI00514293.1	C6orf125	-0.2105
I00219155.5;P613	RPL27	-0.3595	IPI00514250.1	CKS1B	2.00975	IPI00514311.1	CTTNBP2NL	0.499
IPI00456758.4	RPL27A	-0.4605	IPI00514293.1	C6orf125	1.01425	IPI00514669.1	SH3BGRL3	0.0095
IPI00182533.5	RPL28	-0.3105	IPI00514311.1	CTTNBP2NL	-1.372	IPI00514856.4	UBAP2L	0.34225
IPI00419919.5	RPL29	-0.32275	IPI00514669.1	SH3BGRL3	-3.7735	IPI00514956.1	RABGGTB	0.92425
I00219156.7;P628	RPL30	-0.36775	IPI00514856.4	UBAP2L	-0.613	IPI00549189.4	THOP1	0.59025
IPI00927458.1	RPL32	-0.34575	IPI00514956.1	RABGGTB	-1.71975	IPI00549248.4	NPM1	0.06375
IPI00219160.3	RPL34	-0.3675	IPI00549189.4	THOP1	-1.228	IPI00549343.3	TNFRSF9	0.5385
IPI00029731.8	RPL35A	-0.30875	IPI00549248.4	NPM1	1.4555	IPI00549380.1	DGCR8	0.648
IPI00216237.5	RPL36	-0.54925	IPI00549343.3	TNFRSF9	-2.69725	IPI00549381.5	MRPL1	0.14025
344.9;P83882;IPI0	RPL36A	-0.12275	IPI00549380.1	DGCR8	1.08325	IPI00549516.3	TOE1	0.354
IPI00215790.6	RPL38	-0.11375	IPI00549381.5	MRPL1	0.482	IPI00549540.3	PAK1IP1	0.46975
IPI00037619.4	RPL39P5	-0.4555	IPI00549516.3	TOE1	1.572	IPI00549557.3	GMFB	0.63725
IPI00003918.6	RPL4	-0.29925	IPI00549540.3	PAK1IP1	1.24075	IPI00549672.2	PSMD13	0.234
IPI00329389.8	RPL6P27	-0.3075	IPI00549557.3	GMFB	-0.90725	IPI00549885.4	PDHB	-0.1315
IPI00299573.12	RPL7A	-0.36225	IPI00549672.2	PSMD13	-0.54825	IPI00549970.1	GHITM	0.3265
IPI00456940.5	RPL7L1	1.027	IPI00549885.4	PDHB	1.4505	IPI00550020.3	PTMS	0.2495
IPI00030179.3	RPL7P32	-0.41875	IPI00549970.1	GHITM	0.741	IPI00550021.4	SNORD43	0.3505
I00012772.8;P629	RPL8	-0.40975	IPI00550020.3	PTMS	-1.03425	IPI00550037.3	MRPS15	1.0035
IPI00008530.1	RPLP0	-0.519	IPI00550021.4	SNORD43	-0.37125	IPI00550069.3	RNH1	0.09175
IPI00008527.3	RPLP1	-0.519	IPI00550037.3	MRPS15	-0.23675	IPI00550165.4	DHRS7B	0.81025
IPI00008529.1	RPLP2	-0.58075	IPI00550069.3	RNH1	-3.69325	IPI00550234.4	ARPC5	0.89275
IPI00025874.2	RPN1	-0.66475	IPI00550165.4	DHRS7B	-0.456	IPI00550239.4	H1F0	0.436
IPI00019195.2	RPP38	0.73275	IPI00550234.4	ARPC5	-3.25325	IPI00550243.2	THUMPD1	0.227

Table S4 The integrated datasets for the comparison of hiPSCs/HFs, hESCs/HFs, and hiPSCs/hESCs from the public datasets.

AccessionNo	GeneSymbol	hiPSCs/HFs
IPI00009659.3	RPRD1B	0.31025
IPI00639879.2	RPRD2	1.03175
IPI00008438.1	RPS10	-0.113
I00025091.3;P622	RPS11	-0.26175
013917.3;IPI00847	RPS12	-0.418
I00221089.5;P623	RPS13	-0.37125
I00026271.5;P622	RPS14	-0.367
IPI00221091.9	RPS15A	-0.2845
I00221092.8;P141	RPS16	-0.2995
IPI00221093.7	RPS17	-0.398
I00013296.3;P622	RPS18	-0.467
IPI00215780.5	RPS19	-0.4655
IPI00219006.5	RPS19BP1	1.39425
IPI00013485.3	RPS2	-0.4605
IPI00017448.1	RPS21	-0.42875
I00218606.7;P622	RPS23	-0.34175
IPI00847986.1	RPS24	-0.263
IPI00179330.6	RPS27A	-0.675
IPI00746004.2	RPS27L	-0.7955
IPI00719622.1	RPS28	-0.428
IPI00182289.6	RPS29	-1.3585
IPI00011253.3	RPS3	-0.404
00217030.10;P627	RPS4X	-0.36
IPI00008433.4	RPS5	-0.43525
I00021840.1;P627	RPS6	-0.34375
I00013415.1;P620	RPS7	-0.25925
I00216587.9;P622	RPS8	-0.3425
00221088.5;Q6ZW	RPS9	-0.384
IPI00020418.1	RRAS	-3.03925

AccsessionNo	GeneSymbol	hESCs/HFs
IPI00550239.4	H1F0	1.532
IPI00550243.2	THUMPD1	0.0965
IPI00550308.1	RBM12	0.3985
IPI00550363.3	TAGLN2	-4.6575
IPI00550364.8	PGM2	-0.98375
IPI00550451.1	PPP1CA	-0.87875
IPI00550523.5	ATL3	-3.3315
IPI00550638.2	POLR1E	1.28875
IPI00550655.4	SPIN1	1.39775
IPI00550689.3	C22orf28	-0.22825
IPI00550766.1	RRP1	0.82325
IPI00550900.1	TPT1	-2.34075
IPI00550906.6	CSTF2T	2.253
IPI00550917.3	TWF2	-1.29775
IPI00550986.4	C12orf11	1.2285
IPI00550995.2	PIH1D1	-0.11025
IPI00551024.4	DAK	0.80475
IPI00551062.2	CNPY3	1.2915
IPI00552360.2	FAHD1	-1.2675
IPI00552546.1	MCMBP	0.6585
IPI00552587.1	GADD45GIP1	2.03225
IPI00552682.2	DLG1	-0.30525
IPI00552736.1	ISY1	0.6755
IPI00552913.4	STX7	-1.0165
IPI00552978.1	STAG2	2.2495
IPI00553153.1	ATPIF1	0.5605
IPI00553183.1	CCBL2	0.51775
IPI00554438.1	SUGP2	1.9055
IPI00554541.2	ILVBL	0.02975

AccessionNo	GeneSymbol	hiPSCs/hESCs
IPI00550308.1	RBM12	0.371
IPI00550363.3	TAGLN2	1.27325
IPI00550364.8	PGM2	0.24725
IPI00550451.1	PPP1CA	0.439
IPI00550523.5	ATL3	0.22675
IPI00550638.2	POLR1E	0.452
IPI00550655.4	SPIN1	0.0665
IPI00550689.3	C22orf28	-0.0185
IPI00550766.1	RRP1	0.40625
IPI00550900.1	TPT1	0.6265
IPI00550906.6	CSTF2T	-0.1585
IPI00550917.3	TWF2	-0.28825
IPI00550986.4	C12orf11	-0.04775
IPI00550995.2	PIH1D1	0.50475
IPI00551024.4	DAK	0.1985
IPI00551062.2	CNPY3	-0.2735
IPI00552360.2	FAHD1	-0.06975
IPI00552546.1	MCMBP	0.6685
IPI00552587.1	GADD45GIP1	0.40825
IPI00552682.2	DLG1	0.64025
IPI00552736.1	ISY1	0.34975
IPI00552913.4	STX7	0.464
IPI00552978.1	STAG2	-0.11525
IPI00553153.1	ATPIF1	-0.3605
IPI00553183.1	CCBL2	0.27875
IPI00554438.1	SUGP2	0.35125
IPI00554541.2	ILVBL	-0.11125
IPI00554560.4	C16orf88	0.28575
IPI00554590.1	RAB3GAP2	0.24475

Table S4 The integrated datasets for the comparison of hiPSCs/HFs, hESCs/HFs, and hiPSCs/hESCs from the public datasets.

AccessionNo	GeneSymbol	hiPSCs/HFs
IPI00012512.2	RRAS2	-0.0335
IPI00013871.1	RRM1	0.4905
IPI00946732.1	RRM2	1.591
IPI00550766.1	RRP1	0.9845
940901.1;IPI00294	RRP12	0.85025
IPI00007004.4	RRP15	1.25075
IPI00787473.1	RRP36	1.9585
IPI00879006.1	RRP7A	1.2325
IPI00304932.2	RRP8	0.3335
IPI00217862.5	RRP9	1.14625
IPI00014253.4	RRS1	0.79
IPI00479823.3	RSF1	1.09475
IPI00008708.5	RSL1D1	1.01375
IPI00872495.2	RSRC2	1.9205
IPI00011726.1	RTCD1	-1.5305
IPI00783965.4	RTF1	0.5235
IPI00021766.5	RTN4	-1.97925
IPI00021187.4	RUVBL1	0.2035
IPI00009104.7	RUVBL2	0.215
IPI00183695.9	S100A10	-4.1205
IPI00013895.1	S100A11	-2.825
IPI00016179.1	S100A13	-2.0635
IPI00062120.1	S100A16	-3.632
IPI00022275.6	SACM1L	0.11225
IPI00784002.2	SACS	-0.5505
IPI00033130.3	SAE1	0.19575
IPI00005648.1	SAFB2	1.1695
IPI00007032.1	SALL4	2.334
IPI00395474.1	SAMD1	2.175

AccsessionNo	GeneSymbol	hESCs/HFs
IPI00554560.4	C16orf88	3.227
IPI00554590.1	RAB3GAP2	-1.349
IPI00554626.3	CTBP1	-0.3655
IPI00554660.1	PIPOX	3.13775
IPI00554703.1	PIGS	-0.66525
IPI00554711.3	JUP	2.5645
IPI00554734.1	MED15	-0.10125
IPI00554742.3	API5	1.123
IPI00554752.3	PRKAR2B	1.4095
IPI00554786.5	TXNRD1	-2.93475
IPI00554788.5	KRT18	4.212
IPI00554811.2	ARPC4	-2.35725
IPI00555577.1	THY1	-1.721
IPI00555744.6	RPL14	-0.70425
IPI00555755.3	PHC1	3.06975
IPI00555902.1	OCIAD2	3.2245
IPI00555956.2	PSMB4	-0.70975
IPI00556107.1	MPG	0.8055
IPI00556190.2	NDUFB5	-0.96875
IPI00556218.1	NOL6	1.5785
IPI00556594.3	ZCCHC8	1.693
IPI00556619.1	TMX2	-0.661
IPI00556645.3	FOXK1	-2.67575
IPI00604527.2	TARS2	1.8645
IPI00604587.1	HAX1	1.498
IPI00604599.2	TMED3	0.28325
IPI00604620.3	NCL	1.264
IPI00604652.1	NAE1	-0.146
IPI00604660.4	VPS13A	1.40025

AccessionNo	GeneSymbol	hiPSCs/hESCs
IPI00554626.3	CTBP1	0.04575
IPI00554660.1	PIPOX	-0.287
IPI00554703.1	PIGS	-0.07975
IPI00554711.3	JUP	0.426
IPI00554734.1	MED15	0.448
IPI00554742.3	API5	0.181
IPI00554752.3	PRKAR2B	-0.325
IPI00554786.5	TXNRD1	0.52075
IPI00554788.5	KRT18	0.25075
IPI00554811.2	ARPC4	0.71975
IPI00555577.1	THY1	-0.7915
IPI00555744.6	RPL14	0.735
IPI00555755.3	PHC1	-0.60725
IPI00555902.1	OCIAD2	0.253
IPI00555956.2	PSMB4	0.4725
IPI00556107.1	MPG	0.21325
IPI00556190.2	NDUFB5	0.79725
IPI00556218.1	NOL6	-0.1525
IPI00556594.3	ZCCHC8	0.124
IPI00556619.1	TMX2	0.7705
IPI00556645.3	FOXK1	1.1985
IPI00604527.2	TARS2	-0.47025
IPI00604587.1	HAX1	0.1795
IPI00604599.2	TMED3	-0.02575
IPI00604620.3	NCL	0.11125
IPI00604652.1	NAE1	0.3825
IPI00604660.4	VPS13A	-0.095
IPI00604711.3	KIF1A	-0.038
IPI00604756.2	NRBP1	0.52

Table S4 The integrated datasets for the comparison of hiPSCs/HFs, hESCs/HFs, and hiPSCs/hESCs from the public datasets.

AccessionNo	GeneSymbol	hiPSCs/HFs
IPI00294739.1	SAMHD1	-0.854
IPI00412713.4	SAMM50	0.26125
IPI00022019.1	SAP30	2.753
IPI00333699.5	SAP30BP	1.08475
IPI00015954.1	SAR1A	-1.5695
IPI00014938.3	SARNP	1.3305
IPI00220637.5	SARS	-0.879
IPI00328361.7	SARS2	1.74775
IPI00021417.3	SART1	0.97725
IPI00006025.1	SART3	0.52375
IPI00427330.3	SBDS	-3.11525
IPI00303343.7	SCAF1	1.12375
IPI00397794.4	SCAF4	0.9475
IPI00472242.3	SCAMP4	-1.38475
IPI00217766.3	SCARB2	-2.4395
IPI00646334.1	SCD	2.553
IPI00165261.6	SCFD1	-0.776
IPI00793065.1	SCO1	0.42775
IPI00012426.1	SCPEP1	0.51625
IPI00425560.2	SCRIB	-0.0135
IPI00289862.5	SCRN1	-1.57675
IPI00293167.4	SDF2	-1.30025
IPI00106642.4	SDF2L1	-0.837
IPI00305166.2	SDHA	-0.31975
IPI00217143.3	SDHA	-0.32875
IPI00294911.1	SDHB	-0.47925
IPI00643286.4	SDR39U1	1.11075
IPI00104128.3	SEC11A	-0.3525
IPI00641384.2	SEC16A	-1.7895

AccsessionNo	GeneSymbol	hESCs/HFs
IPI00604711.3	KIF1A	2.88975
IPI00604756.2	NRBP1	-2.01825
IPI00604778.1	VPS13C	-0.79225
IPI00607568.3	ATP2C1	-0.99675
IPI00607591.3	RAP1GDS1	-3.80025
IPI00607627.3	MRPL51	-0.044
IPI00607692.2	C9orf5	0.0385
IPI00607732.1	NCLN	-0.51175
IPI00607769.4	STAM	-1.36775
IPI00607799.5	BDH2	-1.21075
IPI00607820.1	GAR1	1.14075
IPI00607861.2	H6PD	-4.136
IPI00619900.2	AP2M1	-1.5845
IPI00619903.3	UGGT1	-0.96425
IPI00619921.3	DIDO1	1.15025
IPI00619944.3	STAG1	0.4145
IPI00639879.2	RPRD2	1.11925
IPI00640276.1	EFHA1	-0.02025
IPI00640341.1	FKBP8	0.93875
IPI00640357.1	USP14	-0.67375
IPI00640595.2	TBP	1.6665
IPI00640597.1	VARS2	3.10375
IPI00640632.1	ROD1	1.93
IPI00640703.3	XPO5	2.0515
IPI00640922.1	BAG6	-0.0415
IPI00640980.1	SNX27	-0.62525
IPI00641137.2	NAV1	-0.3595
IPI00641153.3	GLG1	0.41975
IPI00641168.1	EXOC7	-1.31275

AccessionNo	GeneSymbol	hiPSCs/hESCs
IPI00604778.1	VPS13C	-0.0105
IPI00607568.3	ATP2C1	0.689
IPI00607591.3	RAP1GDS1	0.968
IPI00607627.3	MRPL51	0.47025
IPI00607692.2	C9orf5	-0.08425
IPI00607732.1	NCLN	0.341
IPI00607769.4	STAM	0.4245
IPI00607799.5	BDH2	1.43975
IPI00607820.1	GAR1	0.24575
IPI00607861.2	H6PD	0.9705
IPI00619903.3	UGGT1	0.4305
IPI00619921.3	DIDO1	0.037
IPI00619944.3	STAG1	0.306
IPI00639879.2	RPRD2	0.11825
IPI00640276.1	EFHA1	0.58375
IPI00640341.1	FKBP8	0.102
IPI00640357.1	USP14	0.527
IPI00640595.2	TBP	0.16625
IPI00640597.1	VARS2	-0.3115
IPI00640632.1	ROD1	0.03725
IPI00640703.3	XPO5	0.112
IPI00640922.1	BAG6	0.47025
IPI00640980.1	SNX27	0.638
IPI00641137.2	NAV1	0.13225
IPI00641153.3	GLG1	-0.006
IPI00641168.1	EXOC7	0.43275
IPI00641181.5	MARCKSL1	0.63675
IPI00641384.2	SEC16A	0.0385
IPI00641392.4	JMJD1C	0.31175

Table S4 The integrated datasets for the comparison of hiPSCs/HFs, hESCs/HFs, and hiPSCs/hESCs from the public datasets.

AccessionNo	GeneSymbol	hiPSCs/HFs
I00006865.3;O085	SEC22B	-3.169
IPI00017375.2	SEC23A	-3.36
IPI00017376.2	SEC23B	0.729
IPI00026969.4	SEC23IP	-1.42725
IPI00873472.1	SEC24A	-1.69725
IPI00785208.1	SEC24B	-1.5965
IPI00024661.5	SEC24C	-1.71875
IPI00747830.2	SEC24D	-3.07025
I72.1;P61620;IPI0(	SEC61A1	-1.5665
IPI00220835.7	SEC61B	-1.267
IPI00945991.2	SEC62	-1.171
IPI00218922.5	SEC63	-0.294
IPI00185533.7	SEH1L	1.22675
IPI00002790.3	SEL1L	-1.36
IPI00303753.6	SELRC1	2.44675
IPI00930282.1	SELS	-0.0695
IPI00005101.6	SENP1	1.0275
IPI00939968.1	SENP3	1.0995
IPI00030877.2	15-Sep	0.1365
IPI00029056.2	SEPHS1	1.65875
IPI00927054.1	10-Sep	1.7375
IPI00014177.3	2-Sep	-1.64025
IPI00216139.3	6-Sep	0.901
IPI00022082.7	8-Sep	-2.54675
IPI00871679.3	9-Sep	-1.915
IPI00894485.1	SERF2	0.00875
IPI00027444.1	SERPINB1	-0.969
IPI00749398.1	SERPINB6	-3.46075
IPI00032139.1	SERPINB9	3.367

AccsessionNo	GeneSymbol	hESCs/HFs
IPI00641181.5	MARCKSL1	2.37025
IPI00641384.2	SEC16A	-1.47125
IPI00641392.4	JMJD1C	3.02675
IPI00641582.1	BAG3	-3.96575
IPI00641719.1	SURF4	-1.34525
IPI00641924.2	MRPS9	0.66275
IPI00641957.4	SRSF11	0.73275
IPI00642156.1	EARS2	1.19725
IPI00642195.2	ZMYM4	2.013
IPI00642256.2	CAPZB	-1.17075
IPI00642259.2	DST	-2.97
IPI00642329.2	MFN2	-0.02525
IPI00642416.1	TROVE2	-0.586
IPI00642510.3	TEX10	1.9995
IPI00642549.2	RAD23B	-1.52925
IPI00642807.1	NDUFB8	-1.2505
IPI00642816.2	SRP9	1.15625
IPI00642862.1	PPIL4	0.30875
IPI00642948.1	CD99	-3.48725
IPI00643041.3;P628	RANP1	0.574
IPI00643101.1	NFS1	0.28225
IPI00643286.4	SDR39U1	2.15625
IPI00643588.1	HMGN5	3.43325
IPI00643653.1	UBFD1	-1.39275
IPI00643722.1	ARID1A	1.68425
IPI00643915.1	PPIH	1.228
IPI00644127.2	IARS	-0.4295
IPI00644231.3	CYFIP1	-2.09375
IPI00644245.1	CHTOP	1.234

AccessionNo	GeneSymbol	hiPSCs/hESCs
IPI00641582.1	BAG3	0.743
IPI00641719.1	SURF4	0.033
IPI00641924.2	MRPS9	0.4765
IPI00641957.4	SRSF11	0.26025
IPI00642156.1	EARS2	0.039
IPI00642195.2	ZMYM4	0.39
IPI00642256.2	CAPZB	0.213
IPI00642259.2	DST	0.2315
IPI00642329.2	MFN2	0.42275
IPI00642416.1	TROVE2	0.3505
IPI00642510.3	TEX10	0.243
IPI00642549.2	RAD23B	0.4305
IPI00642807.1	NDUFB8	0.46625
IPI00642816.2	SRP9	0.279
IPI00642862.1	PPIL4	0.33675
IPI00642948.1	CD99	0.6435
IPI00643041.3;P628	RANP1	0.16275
IPI00643101.1	NFS1	-0.08625
IPI00643286.4	SDR39U1	-0.659
IPI00643588.1	HMGN5	4.2185
IPI00643653.1	UBFD1	0.847
IPI00643722.1	ARID1A	0.33325
IPI00643915.1	PPIH	0.01225
IPI00644127.2	IARS	0.31925
IPI00644231.3	CYFIP1	0.69925
IPI00644245.1	CHTOP	0.50925
IPI00644361.2	NAT1	0.61975
IPI00644708.1	TIAL1	0.23175
IPI00644712.4	XRCC6	0.092

Table S4 The integrated datasets for the comparison of hiPSCs/HFs, hESCs/HFs, and hiPSCs/hESCs from the public datasets.

AccessionNo	GeneSymbol	hiPSCs/HFs
IPI00032140.4	SERPINH1	-1.4005
REV_IPI00910487.	SERPINH1	-1.5125
IPI00072377.1	SET	2.64575
IPI00386120.3	SF1	0.54325
IPI00017451.1	SF3A1	0.96525
IPI00017341.3	SF3A2	0.79275
IPI00029764.1	SF3A3	0.97475
IPI00026089.4	SF3B1	1.03525
IPI00032827.1	SF3B14	1.077
IPI00221106.5	SF3B2	0.7635
IPI00300371.5	SF3B3	0.834
IPI00010404.1	SF3B5	0.76225
IPI00010740.1	SFPQ	1.14475
IPI00749245.1	SFRP1	1.2905
IPI00009368.4	SFXN1	0.08675
IPI00793874.1	SFXN3	-5.00375
IPI00099463.2	SGPL1	0.9
IPI00013949.1	SGTA	-0.36725
IPI00025318.1	SH3BGRL	-1.5095
IPI00514669.1	SH3BGRL3	-4.17775
IPI00019169.3	SH3GL1	-1.764
IPI00006558.3	SH3GLB1	-2.19125
IPI00023832.5	SH3PXD2B	0.065
IPI00943132.1	SHC1	-1.37875
IPI00789370.3	SHMT2	0.888
IPI00479890.4	SHOC2	-1.47
IPI00005914.4	SHPK	1.2305
IPI00015180.1	SHROOM2	3.09275
IPI00376226.2	SIGMAR1	-0.33075

AccsessionNo	GeneSymbol	hESCs/HFs
IPI00644361.2	NAT1	-1.3935
IPI00644708.1	TIAL1	-0.015
IPI00644712.4	XRCC6	0.99275
IPI00644786.3	GRIPAP1	0.09975
IPI00644795.3	TMEM2	-2.6015
IPI00644989.2	PDIA6	-0.2245
IPI00645046.1	NRM	0.89775
IPI00645078.1	UBA1	-0.49325
IPI00645268.4	SPIRE1	-3.3425
IPI00645329.1	RBBP4	1.65025
IPI00645384.3	SRSF10	1.5275
IPI00645426.2	MED22	0.7045
IPI00645585.2	STK4	-0.2225
IPI00645608.2	IRF2BP1	0.84825
IPI00645613.1	EPS15L1	-1.022
IPI00645614.2	CDH3	1.94025
IPI00645702.1	CTPS2	2.01125
IPI00645805.4	IVD	0.83
IPI00645809.1	PDXDC1	-1.252
IPI00645816.1	CD3EAP	1.59
IPI00645867.2	MYOF	-4.549
IPI00645869.1	LAS1L	1.78825
IPI00646281.1	L1CAM	1.68775
IPI00646304.4	PPIB	-1.552
IPI00646334.1	SCD	1.68425
IPI00646605.3	UBR4	-0.60925
IPI00646625.1	TAP1	-0.76275
IPI00646689.1	TXNDC17	-0.7555
IPI00646719.1	MAGT1	-0.35325

AccessionNo	GeneSymbol	hiPSCs/hESCs
IPI00644786.3	GRIPAP1	0.387
IPI00644795.3	TMEM2	3.2935
IPI00644989.2	PDIA6	0.16225
IPI00645046.1	NRM	-0.2375
IPI00645078.1	UBA1	0.1865
IPI00645268.4	SPIRE1	0.4625
IPI00645329.1	RBBP4	0.4485
IPI00645384.3	SRSF10	0.137
IPI00645426.2	MED22	0.397
IPI00645585.2	STK4	0.6105
IPI00645608.2	IRF2BP1	-0.05225
IPI00645613.1	EPS15L1	0.0345
IPI00645614.2	CDH3	-0.07125
IPI00645702.1	CTPS2	-0.46375
IPI00645805.4	IVD	-0.582
IPI00645809.1	PDXDC1	0.9345
IPI00645816.1	CD3EAP	0.74025
IPI00645867.2	MYOF	-0.03625
IPI00645869.1	LAS1L	0.12525
IPI00646281.1	L1CAM	0.16125
IPI00646304.4	PPIB	0.5235
IPI00646334.1	SCD	1.1545
IPI00646605.3	UBR4	0.32175
IPI00646625.1	TAP1	-1.585
IPI00646689.1	TXNDC17	0.27125
IPI00646719.1	MAGT1	0.01425
IPI00646721.1	USP7	0.36075
IPI00646750.2	C1orf31	-0.0305
IPI00646917.1	NUDT21	0.22925

Table S4 The integrated datasets for the comparison of hiPSCs/HFs, hESCs/HFs, and hiPSCs/hESCs from the public datasets.

AccessionNo	GeneSymbol	hiPSCs/HFs
IPI00170596.1	SIN3A	2.42975
IPI00016802.1	SIRT1	1.70825
IPI00647217.2	SKIV2L2	1.5365
IPI00301364.3	SKP1	-0.08575
IPI00220844.1	SLC12A2	-0.633
IPI00008616.3	SLC12A7	0.6585
IPI00019472.4	SLC1A5	-0.318
IPI00294159.3	SLC25A1	0.39225
IPI00005537.3	SLC25A10	0.2845
IPI00945233.1	SLC25A11	-0.14425
IPI00940497.1	SLC25A12	-0.80475
IPI00007084.3	SLC25A13	2.919
IPI00003004.1	SLC25A22	-0.4
IPI00215777.1	SLC25A3	-0.255
IPI00022891.3	SLC25A4	1.2845
IPI00007188.5	SLC25A5	0.63875
IPI00291467.7	SLC25A6	-0.5515
IPI00412147.1	SLC27A4	-0.7485
IPI00909237.1	SLC2A1	1.46975
IPI00003909.1	SLC2A3	3.99575
IPI00410034.2	SLC38A2	0.312
IPI00303452.5	SLC39A14	0.55425
IPI00298702.2	SLC39A6	-0.52
IPI00027493.1	SLC3A2	1.38825
IPI00306749.8	SLC4A1AP	-0.107
IPI00477139.1	SLC4A2	0.2675
IPI00021058.2	SLC4A7	1.22375
IPI00295698.6	SLC7A3	1.4745
IPI00008986.1	SLC7A5	1.4785

AccsessionNo	GeneSymbol	hESCs/HFs
IPI00646721.1	USP7	0.68075
IPI00646750.2	C1orf31	1.01925
IPI00646917.1	NUDT21	1.20025
IPI00647217.2	SKIV2L2	1.56825
IPI00647268.1	RHOC	-4.441
IPI00647510.5	SMARCA1	0.949
IPI00647664.7	ZNF281	1.49175
IPI00647720.1	SRRM1	1.09125
IPI00647805.1	NT5C3	-1.15425
IPI00651629.3	SPECC1	0.9385
IPI00651738.1	ADI1	-1.402
IPI00654555.3	NOP2	1.04225
IPI00654592.3	EXOSC9	1.49075
IPI00654603.4	CCDC88A	-1.00875
IPI00654611.2	HEATR2	0.22975
IPI00655641.1	SUPT5H	0.67475
IPI00656071.1	TIMM50	0.826
IPI00656138.1	PAK1	1.7755
IPI00657724.1	NPTN	-2.33425
IPI00657777.4	DDX55	1.1635
IPI00657874.1	ZNF259	-1.38675
IPI00658000.3	IGF2BP3	1.8895
IPI00658072.2	RBM34	0.821
IPI00658109.1	CKMT1B	3.56025
IPI00658202.1	CDH2	-4.37425
IPI00658210.1	WDR48	1.04875
IPI00718985.1	ARHGAP35	-1.27825
IPI00719053.1	TRIM71	3.4785
IPI00719106.1	CPSF7	1.1375

AccessionNo	GeneSymbol	hiPSCs/hESCs
IPI00647217.2	SKIV2L2	0.24425
IPI00647268.1	RHOC	0.56975
IPI00647510.5	SMARCA1	0.4995
IPI00647664.7	ZNF281	0.36275
IPI00647720.1	SRRM1	0.72325
IPI00647805.1	NT5C3	0.6715
IPI00651629.3	SPECC1	0.2745
IPI00651738.1	ADI1	0.86475
IPI00654555.3	NOP2	0.60275
IPI00654592.3	EXOSC9	0.27325
IPI00654603.4	CCDC88A	1.02125
IPI00654611.2	HEATR2	0.316
IPI00655641.1	SUPT5H	0.338
IPI00656071.1	TIMM50	0.16525
IPI00656138.1	PAK1	0.7635
IPI00657724.1	NPTN	0.4945
IPI00657777.4	DDX55	0.59625
IPI00657874.1	ZNF259	0.75175
IPI00658000.3	IGF2BP3	-0.087
IPI00658072.2	RBM34	0.673
IPI00658109.1	CKMT1B	-0.656
IPI00658202.1	CDH2	6.0015
IPI00658210.1	WDR48	0.07675
IPI00718985.1	ARHGAP35	0.46325
IPI00719053.1	TRIM71	-0.652
IPI00719106.1	CPSF7	0.40625
IPI00719600.5	CYFIP2	-0.10675
IPI00719622.1	RPS28	0.24825
IPI00719806.1	ATP6V1E1	0.435

Table S4 The integrated datasets for the comparison of hiPSCs/HFs, hESCs/HFs, and hiPSCs/hESCs from the public datasets.

AccessionNo	GeneSymbol	hiPSCs/HFs
IPI00003527.5	SLC9A3R1	3.66625
IPI00009922.3	SLIRP	1.007
IPI00247439.3	SLK	-1.5425
IPI00019996.3	SLTM	1.66775
IPI00903182.1	SMAD5	0.12275
IPI00647510.5	SMARCA1	1.11875
386718.4;IPI00513	SMARCA2	-1.25425
IPI00900338.1	SMARCA4	2.33375
IPI00297211.1	SMARCA5	2.37225
IPI00745019.1	SMARCB1	1.74725
IPI00234252.3	SMARCC1	2.9895
IPI00935702.2	SMARCD2	1.6915
IPI00470489.1	SMARCE1	1.39425
IPI00291939.1	SMC1A	1.8215
007927.4;IPI00232	SMC2	0.70425
IPI00219420.3	SMC3	1.79675
IPI00793575.2	SMC4	0.56975
IPI00154528.3	SMC6	1.878
IPI00746325.2	SMEK2	0.29575
IPI00847523.2	SMG8	-0.16825
IPI00009598.1	SMN2	0.9925
IPI00025176.1	SMNDC1	1.2445
IPI00005102.3	SMS	-0.05225
I00305833.3;Q3UK	SMU1	1.499
IPI00032831.4	SNAP29	-2.2865
IPI00140420.4	SND1	-0.8825
IPI00178440.3	SNORA41	-1.487
IPI00927101.1	SNORA6	-0.23
IPI00411937.4	SNORD110	1.30825

AccsessionNo	GeneSymbol	hESCs/HFs
IPI00719600.5	CYFIP2	3.23375
IPI00719622.1	RPS28	-0.41675
IPI00719806.1	ATP6V1E1	-0.94075
IPI00735181.2	UNC45A	-1.4295
IPI00737530.1	ARPC1B	-2.784
IPI00737545.4	PEAK1	-2.0285
IPI00741537.5	PRRC2B	-0.08475
IPI00741630.4	WIZ	1.49925
IPI00741684.1	TRIOBP	-2.43075
IPI00741958.1	BEND3	2.641
IPI00742124.2	UBXN7	0.7105
IPI00742682.2	TPR	1.088
IPI00742937.1	ARID1B	0.86775
IPI00742943.1	NUP43	1.24775
IPI00743342.2	FHL2	-4.69825
IPI00743576.1	ATP6V0A1	-0.8655
IPI00743825.1;P612	COPS2	-1.36025
IPI00744062.1	TCERG1	1.28
IPI00744148.2	H2AFY	-0.225
IPI00744194.1	ATP1A1	1.03325
IPI00744692.1	TALDO1	-0.87875
IPI00744711.2	PNPT1	0.9345
IPI00745019.1	SMARCB1	1.819
IPI00745335.7	GPX4	1.898
IPI00745396.1	HNRPLL	0.604
IPI00745433.2	EIF2C2	0.39175
IPI00745502.2	PSMC5	-0.6365
IPI00745553.1	HAGH	-0.80275
IPI00745613.2	EXOSC4	1.1115

AccessionNo	GeneSymbol	hiPSCs/hESCs
IPI00735181.2	UNC45A	0.52825
IPI00737530.1	ARPC1B	0.40225
IPI00737545.4	PEAK1	-0.3635
IPI00741537.5	PRRC2B	0.32275
IPI00741630.4	WIZ	0.36375
IPI00741684.1	TRIOBP	-0.107
IPI00741958.1	BEND3	0.33375
IPI00742124.2	UBXN7	0.34775
IPI00742682.2	TPR	0.08825
IPI00742937.1	ARID1B	0.488
IPI00742943.1	NUP43	-0.44075
IPI00743342.2	FHL2	0.37775
IPI00743576.1	ATP6V0A1	0.57775
IPI00743825.1;P612	COPS2	0.31975
IPI00744062.1	TCERG1	-0.063
IPI00744148.2	H2AFY	0.5905
IPI00744194.1	ATP1A1	0.4
IPI00744692.1	TALDO1	0.21975
IPI00744711.2	PNPT1	-0.022
IPI00745019.1	SMARCB1	0.20475
IPI00745335.7	GPX4	0.27825
IPI00745396.1	HNRPLL	0.201
IPI00745433.2	EIF2C2	0.47575
IPI00745502.2	PSMC5	0.225
IPI00745553.1	HAGH	-0.09625
IPI00745613.2	EXOSC4	0.253
IPI00745793.1	CCNB1	0.281
IPI00745893.2	LOC728825	0.34125
IPI00745906.2	SUOX	0.94275

Table S4 The integrated datasets for the comparison of hiPSCs/HFs, hESCs/HFs, and hiPSCs/hESCs from the public datasets.

AccessionNo	GeneSymbol	hiPSCs/HFs
IPI00003886.3	SNORD19B	1.23525
IPI00000494.6	SNORD21	-0.39175
IPI00304612.9	SNORD32A	-0.26
IPI00550021.4	SNORD43	-0.29675
I00012493.1;P608	SNORD54	-0.4325
IPI00419880.6	SNORD73A	-0.422
IPI00420014.2	SNRNP200	1.31075
IPI00940685.1	SNRNP40	1.45125
IPI00219483.1	SNRNP70	0.91825
IPI00012382.3	SNRPA	0.6595
IPI00297477.3	SNRPA1	1.18
IPI00395674.1	SNRPB	1.02725
IPI00029267.1	SNRPB2	1.20725
IPI00013396.3	SNRPC	0.60175
I00302850.4;P623	SNRPD1	0.83525
I00017963.1;P623	SNRPD2	1.22075
IPI00878876.1	SNRPD3	1.0815
IPI00029266.1	SNRPE	0.9885
I00220528.6;P623	SNRPF	1.169
IPI00009505.1	SNTB2	-3.31025
IPI00013830.1	SNW1	0.968
IPI00220527.5	SNX1	-2.08625
IPI00844112.1	SNX12	-1.088
IPI00299095.2	SNX2	-0.1685
IPI00640980.1	SNX27	-0.2855
IPI00815770.2	SNX3	-2.33975
IPI00001883.1	SNX9	-2.19675
IPI00218733.6	SOD1	-0.922
IPI00216057.5	SORD	0.63525

AccsessionNo	GeneSymbol	hESCs/HFs
IPI00745793.1	CCNB1	3.45675
IPI00745893.2	LOC728825	0.719
IPI00745906.2	SUOX	-0.5625
IPI00745955.4	EBNA1BP2	0.86425
IPI00746004.2	RPS27L	-1.054
IPI00746072.3	TLK1	1.461
IPI00746165.2	WDR1	-1.89425
IPI00746205.1	PSME2	-1.4735
IPI00746221.2	FAM195B	-1.44825
IPI00746325.2	SMEK2	0.5245
IPI00746438.2	RPL11	-0.25275
IPI00746642.1	TMEM48	1.8955
IPI00746777.3	ADH5	-0.08475
IPI00747309.1	RCC1	1.6495
IPI00747403.20	RNMT	1.40525
IPI00747502.1	ACTR6	0.57725
IPI00747764.3	KPNA6	-0.52375
IPI00747787.2	NCAPD3	1.27275
IPI00747830.2	SEC24D	-3.93175
IPI00747913.2	BOLA2B	0.25625
IPI00748303.3	ZFR	0.7195
IPI00748360.2	KIAA1797	-1.465
IPI00748807.2	NUP160	1.62675
IPI00749237.1	C14orf2	0.5755
IPI00749245.1	SFRP1	0.471
IPI00749304.1	RIC8A	-2.772
IPI00749398.1	SERPINB6	-3.3135
IPI00749513.5	KIAA0319L	2.276
IPI00759472.1	INCENP	1.71775

AccessionNo	GeneSymbol	hiPSCs/hESCs
IPI00745955.4	EBNA1BP2	0.39825
IPI00746004.2	RPS27L	0.5375
IPI00746072.3	TLK1	-0.06375
IPI00746165.2	WDR1	0.23575
IPI00746205.1	PSME2	0.10375
IPI00746221.2	FAM195B	0.6005
IPI00746325.2	SMEK2	0.0595
IPI00746438.2	RPL11	0.24025
IPI00746642.1	TMEM48	0.174
IPI00746777.3	ADH5	0.82
IPI00747309.1	RCC1	0.01275
IPI00747403.20	RNMT	-0.282
IPI00747502.1	ACTR6	0.46
IPI00747764.3	KPNA6	0.26525
IPI00747787.2	NCAPD3	0.5945
IPI00747830.2	SEC24D	1.1095
IPI00747913.2	BOLA2B	0.231
IPI00748303.3	ZFR	0.188
IPI00748360.2	KIAA1797	0.5905
IPI00748807.2	NUP160	-0.03825
IPI00749237.1	C14orf2	-0.35
IPI00749245.1	SFRP1	2.8265
IPI00749304.1	RIC8A	1.16025
IPI00749398.1	SERPINB6	0.28775
IPI00749513.5	KIAA0319L	-0.345
IPI00759472.1	INCENP	0.65825
IPI00759607.1	TCEAL6	-1.1005
IPI00759659.1	GOLM1	0.2605
IPI00759663.1	PRDX5	-0.10225

Table S4 The integrated datasets for the comparison of hiPSCs/HFs, hESCs/HFs, and hiPSCs/hESCs from the public datasets.

AccessionNo	GeneSymbol	hiPSCs/HFs
IPI00022608.1	SORL1	2.234
IPI00009703.1	SOX2	2.81325
IPI00852778.1	SP3	0.93875
IPI00794341.1	SPAG7	1.52
IPI00479676.5	SPAG9	-1.6615
IPI00014572.1	SPARC	-2.93575
IPI00923524.1	SPAST	0.32925
IPI00010219.1	SPC25	0.5765
IPI00300299.6	SPCS3	-0.3345
IPI00651629.3	SPECC1	1.0075
IPI00045914.1	SPEN	1.124
IPI00430622.1	SPG20	0.09875
IPI00550655.4	SPIN1	1.216
IPI00376403.2	SPINT1	2.26525
IPI00011662.1	SPINT2	2.5305
IPI00645268.4	SPIRE1	-3.392
IPI00333015.7	SPTBN1	2.464
IPI00005614.6	SPTBN1	0.2425
IPI00218207.1	SPTBN2	0.67525
IPI00005745.1	SPTLC1	-0.18925
IPI00179473.9	SQSTM1	-2.77325
IPI00102313.3	SRA1	-1.6235
IPI00171087.7	SRBD1	1.36575
IPI00375462.1	SREK1	0.5175
IPI00304493.5	SRFBP1	1.31375
IPI00414827.1	SRGAP1	-3.8095
IPI00946099.1	SRI	-0.93975
IPI00292020.3	SRM	-1.86075
IPI00293434.2	SRP14	1.00075

AccsessionNo	GeneSymbol	hESCs/HFs
IPI00759607.1	TCEAL6	-1.08175
IPI00759659.1	GOLM1	1.366
IPI00759663.1	PRDX5	-0.013
IPI00759686.1	CC2D1A	-1.67125
IPI00759715.1	FH	-0.19325
IPI00759742.2	BAZ2A	1.51425
IPI00759775.1	RHOT1	0.72125
IPI00759824.2	ANP32B	-1.61925
IPI00759828.1	QKI	0.7375
IPI00759832.1	YWHAB	-1.5795
IPI00760574.1	LDB1	0.47475
IPI00760748.1	TMEM30A	0.18025
IPI00761069.1	CAST	-4.12225
IPI00782966.1	ZFP106	2.757
IPI00782974.1	C17orf85	0.1675
IPI00782992.3	SRRM2	0.86325
IPI00783001.1	METTL15	1.59325
IPI00783118.2	NT5DC2	1.296
IPI00783271.1	LRPPRC	1.235
IPI00783302.1	PTCD3	0.78475
IPI00783378.3	UBE2O	0.7355
IPI00783559.1	EXOC2	-1.07625
IPI00783665.4	LAMA5	2.918
IPI00783781.1	NUP205	1.459
IPI00783862.2	BLVRB	-0.81
IPI00783965.4	RTF1	0.59475
IPI00783982.1	COPG	-3.029
IPI00784002.2	SACS	-0.978
IPI00784013.1	JAK1	-3.008

AccessionNo	GeneSymbol	hiPSCs/hESCs
IPI00759686.1	CC2D1A	1.03475
IPI00759715.1	FH	-0.138
IPI00759742.2	BAZ2A	1.458
IPI00759775.1	RHOT1	0.019
IPI00759824.2	ANP32B	0.75425
IPI00759828.1	QKI	0.418
IPI00759832.1	YWHAB	0.60825
IPI00760574.1	LDB1	0.47725
IPI00760748.1	TMEM30A	0.0815
IPI00761069.1	CAST	-0.1115
IPI00782966.1	ZFP106	0.26
IPI00782974.1	C17orf85	0.38325
IPI00782992.3	SRRM2	0.2755
IPI00783001.1	METTL15	-0.447
IPI00783118.2	NT5DC2	0.1685
IPI00783271.1	LRPPRC	0.146
IPI00783302.1	PTCD3	0.25975
IPI00783378.3	UBE2O	0.17475
IPI00783559.1	EXOC2	0.30675
IPI00783665.4	LAMA5	0.089
IPI00783781.1	NUP205	0.01425
IPI00783862.2	BLVRB	-0.31925
IPI00783965.4	RTF1	0.3185
IPI00783982.1	COPG	0.40025
IPI00784002.2	SACS	0.69025
IPI00784013.1	JAK1	0.38675
IPI00784119.1	ATP6AP1	0.81725
IPI00784154.1	HSPD1	-0.09375
IPI00784161.1	SUPT6H	0.3695

Table S4 The integrated datasets for the comparison of hiPSCs/HFs, hESCs/HFs, and hiPSCs/hESCs from the public datasets.

AccessionNo	GeneSymbol	hiPSCs/HFs
IPI00295889.2	SRP19	-0.696
IPI00168388.1	SRP68	-0.776
IPI00215888.4	SRP72	-0.591
IPI00642816.2	SRP9	1.13975
IPI00385267.4	SRPR	-1.414
IPI00295098.3	SRPRB	-1.68825
IPI00647720.1	SRRM1	1.199
IPI00782992.3	SRRM2	0.86675
00215884.4;Q6PD	SRSF1	1.084
IPI00645384.3	SRSF10	1.37525
IPI00641957.4	SRSF11	0.6825
IPI00843996.1	SRSF3	0.986
IPI00000015.2	SRSF4	0.73525
IPI00856075.1	SRSF8	2.0225
IPI00012340.1	SRSF9	1.48275
IPI00452919.3	SS18	1.284
IPI00009032.1	SSB	0.275
IPI00945960.1	SSR3	-1.42675
IPI00019385.3	SSR4	-1.04575
IPI00005154.1	SSRP1	1.333
IPI00954445.1	ST13	0.04575
IPI00619944.3	STAG1	0.49625
IPI00552978.1	STAG2	1.83775
IPI00607769.4	STAM	-1.2445
IPI00030781.1	STAT1	-2.633
IPI00412752.3	STAT3	-0.90725
IPI00299063.2	STIM1	-2.02575
IPI00013894.1	STIP1	-0.108
IPI00304742.5	STK10	-2.92025

AccsessionNo	GeneSymbol	hESCs/HFs
IPI00784119.1	ATP6AP1	-0.03375
IPI00784154.1	HSPD1	2.18825
IPI00784161.1	SUPT6H	0.839
IPI00784295.2	HSP90AA1	-0.5295
IPI00784901.1	KIAA1715	-2.6775
IPI00785208.1	SEC24B	-1.39375
IPI00787473.1	RRP36	2.04075
IPI00787827.1	PITRM1	-2.28425
IPI00788069.1	NAA30	-0.9345
IPI00788826.1	PUF60	0.422
IPI00788849.1	TFG	-2.36
IPI00788907.2	PGAM5	1.207
IPI00789008.1	FLOT2	0.105
IPI00789029.1	NAP1L1	-0.25375
IPI00789041.2	PNN	1.7725
IPI00789069.2	ARL8B	-0.91
IPI00789370.3	SHMT2	1.1625
IPI00789428.1	MCCC2	1.89175
IPI00789431.1	CHMP7	-0.1775
IPI00789806.2	LAP3	-0.8185
IPI00790114.1	MRPL27	-0.57575
IPI00790228.1	C17orf49	1.966
IPI00790634.2	WIBG	0.4205
IPI00790937.3	NMD3	-0.37775
IPI00791152.1	ENY2	0.6335
IPI00791325.1	KIAA0020	0.69875
IPI00791542.2	MRPS22	0.7345
IPI00791573.1	SUGT1	-0.6205
IPI00791679.2	BNIP3L	-0.29075

AccessionNo	GeneSymbol	hiPSCs/hESCs
IPI00784295.2	HSP90AA1	0.471
IPI00784901.1	KIAA1715	-0.139
IPI00785208.1	SEC24B	0.201
IPI00787473.1	RRP36	0.27325
IPI00787827.1	PITRM1	-0.02675
IPI00788069.1	NAA30	0.5645
IPI00788826.1	PUF60	0.297
IPI00788849.1	TFG	0.67775
IPI00788907.2	PGAM5	0.085
IPI00789008.1	FLOT2	0.02
IPI00789029.1	NAP1L1	0.73275
IPI00789041.2	PNN	0.08575
IPI00789069.2	ARL8B	0.04525
IPI00789370.3	SHMT2	0.02075
IPI00789428.1	MCCC2	-0.459
IPI00789431.1	CHMP7	0.23275
IPI00789806.2	LAP3	0.289
IPI00790114.1	MRPL27	2.287
IPI00790228.1	C17orf49	0.43025
IPI00790634.2	WIBG	0.56275
IPI00790937.3	NMD3	0.09875
IPI00791152.1	ENY2	0.646
IPI00791325.1	KIAA0020	0.50525
IPI00791542.2	MRPS22	0.1595
IPI00791573.1	SUGT1	0.616
IPI00791679.2	BNIP3L	-0.30425
IPI00791938.1	LLGL1	0.08225
IPI00792109.1	CCDC43	0.487
IPI00792364.1	PRKCD	-0.05125

Table S4 The integrated datasets for the comparison of hiPSCs/HFs, hESCs/HFs, and hiPSCs/hESCs from the public datasets.

AccessionNo	GeneSymbol	hiPSCs/HFs
IPI00645585.2	STK4	0.10975
IPI00479997.4	STMN1	0.0475
IPI00219682.6	STOM	-3.372
IPI00334190.4	STOML2	0.338
IPI00941907.1	STRAP	-0.5445
IPI00477001.3	STRN3	-0.927
IPI00003016.4	STRN4	-0.27275
IPI00297492.2	STT3A	-0.91425
IPI00152377.1	STT3B	-0.31825
IPI00025156.4	STUB1	-1.59725
IPI00329332.1	STX12	-3.25025
IPI00029730.1	STX4	-0.743
IPI00013930.1	STX6	0.45225
IPI00552913.4	STX7	-0.76275
IPI00297626.4	STXBP3	-0.59275
IPI00221222.7	SUB1	0.24325
IPI00217232.2	SUCLA2	0.0985
IPI00921173.1	SUCLG1	-0.9985
IPI00103419.4	SUGP1	1.01375
IPI00554438.1	SUGP2	1.993
IPI00791573.1	SUGT1	-0.28875
I00303105.3;P631	SUMO1	1.5015
IPI00873723.2	SUN1	0.2715
IPI00745906.2	SUOX	0.099
IPI00026970.4	SUPT16H	1.36875
IPI00655641.1	SUPT5H	0.70475
IPI00784161.1	SUPT6H	0.933
IPI00412404.1	SUPV3L1	0.46275
IPI00641719.1	SURF4	-1.6015

AccsessionNo	GeneSymbol	hESCs/HFs
IPI00791938.1	LLGL1	1.0635
IPI00792109.1	CCDC43	-0.47525
IPI00792364.1	PRKCD	0.607
IPI00792446.1	CDK2AP1	1.1395
IPI00792482.1	PRKAA1	-2.34425
IPI00792595.2	RFC4	2.37575
IPI00792916.2	PRKCSH	-0.51675
IPI00793065.1	SCO1	0.952
IPI00793309.1	NANOG	4.69075
IPI00793375.1	XPNPEP1	-1.99875
IPI00793412.1	PHF10	1.875
IPI00793438.1	ARID3B	2.9745
IPI00793443.2	IPO5	-0.96875
IPI00793575.2	SMC4	0.2465
IPI00793874.1	SFXN3	-4.12275
IPI00793999.2	TBC1D15	-0.0165
IPI00794341.1	SPAG7	1.06625
IPI00794444.2	GYG1	-3.032
IPI00794663.1	TUBA4A	1.29225
IPI00794880.1	CHD7	2.39675
IPI00794978.1	MRPL47	-0.0115
IPI00795249.2	C12orf43	0.996
IPI00795292.1	NME1	-1.313
IPI00795428.3	CCDC51	1.2835
IPI00795892.1	PFN2	-1.8465
IPI00795923.3	PCM1	1.24175
IPI00796105.1	CHCHD6	-0.5215
IPI00796572.1	C12ORF57	-0.714
IPI00796836.2	DDI2	0.668

AccessionNo	GeneSymbol	hiPSCs/hESCs
IPI00792446.1	CDK2AP1	0.45975
IPI00792482.1	PRKAA1	0.1535
IPI00792595.2	RFC4	0.0435
IPI00792916.2	PRKCSH	0.138
IPI00793065.1	SCO1	-0.2555
IPI00793309.1	NANOG	-0.3845
IPI00793375.1	XPNPEP1	0.438
IPI00793412.1	PHF10	0.168
IPI00793438.1	ARID3B	-0.0195
IPI00793443.2	IPO5	0.73675
IPI00793575.2	SMC4	0.5875
IPI00793874.1	SFXN3	-1.02825
IPI00793999.2	TBC1D15	0.33475
IPI00794341.1	SPAG7	0.66925
IPI00794444.2	GYG1	-0.512
IPI00794663.1	TUBA4A	-0.13275
IPI00794880.1	CHD7	1.12525
IPI00794978.1	MRPL47	0.422
IPI00795249.2	C12orf43	0.13475
IPI00795292.1	NME1	0.5995
IPI00795428.3	CCDC51	-0.44625
IPI00795892.1	PFN2	0.4915
IPI00795923.3	PCM1	0.476
IPI00796105.1	CHCHD6	0.15425
IPI00796572.1	C12ORF57	0.28175
IPI00796836.2	DDI2	0.02075
IPI00797038.1	PCK2	-0.6785
IPI00797136.2	IKBIP	0.2885
IPI00797249.1	DCXR	-0.336

Table S4 The integrated datasets for the comparison of hiPSCs/HFs, hESCs/HFs, and hiPSCs/hESCs from the public datasets.

AccessionNo	GeneSymbol	hiPSCs/HFs
IPI00026606.2	SURF6	0.99075
IPI00059242.3	SYAP1	-1.2485
IPI00023344.2	SYMPK	1.4165
IPI00018140.3	SYNCRIP	-0.04425
IPI00239405.4	SYNE2	2.54025
IPI00299193.1	SYNJ2BP	0.433
IPI00002135.1	TACC3	2.56225
IPI00019903.1	TACO1	-0.02625
IPI00030364.1	TAF10	0.693
IPI00020194.1	TAF15	0.61425
IPI00478296.3	TAF4	1.7085
IPI00298926.4	TAF5	2.19125
IPI00216138.6	TAGLN	-3.906
IPI00550363.3	TAGLN2	-3.5225
IPI00744692.1	TALDO1	-1.0095
IPI00646625.1	TAP1	-2.60325
IPI00439936.1	TAPBP	-2.9995
IPI00472583.2	TARBP2	0.9695
IPI00025815.2	TARDBP	0.92875
IPI00329633.5	TARS	-0.3105
IPI00604527.2	TARS2	1.23025
IPI00005585.5	TAX1BP3	-2.388
IPI00793999.2	TBC1D15	-0.02575
IPI00926241.1	TBC1D4	0.2395
IPI00942761.1	TBC1D8B	-0.82775
IPI00217236.4	TBCA	0.67075
IPI00019924.1	TBCC	-1.467
IPI00018402.1	TBCE	-0.0945
IPI00922336.1	TBL1XR1	0.5585

AccsessionNo	GeneSymbol	hESCs/HFs
IPI00797038.1	PCK2	1.50475
IPI00797136.2	IKBIP	-4.4015
IPI00797249.1	DCXR	0.69975
IPI00797533.1	MRPL46	0.264
IPI00797574.3	BOD1L	0.5865
IPI00797796.1	CDC27	-0.3615
IPI00798092.1	BAG4	-0.42575
IPI00807491.2	GTF3C1	2.4135
IPI00807597.1	HELLS	3.74325
IPI00815770.2	SNX3	-3.297
IPI00816106.3	DOCK7	-2.379
IPI00816829.2	TWF1	-4.12225
IPI00827831.3	PSMF1	-3.11
IPI00828082.1	MYO18A	-0.71075
IPI00828098.2	RNF213	-0.8475
IPI00829652.1	RBM16	0.74475
IPI00829741.2	ANKLE2	-0.99925
IPI00829808.1	EXOC5	-1.35375
IPI00829826.1	MED1	0.54025
IPI00829835.2	GPATCH4	0.8945
IPI00829908.1	U2SURP	1.04775
IPI00829966.1	TMEM126B	1.0595
IPI00830039.1	U2AF2	0.7025
IPI00830108.1	DNAJC2	0.0275
IPI00843789.2	GLDC	1.07325
IPI00843790.1	USP36	2.06225
IPI00843876.1	TNPO1	-0.274
IPI00843886.1	APOO	2.28425
IPI00843975.1	EZR	-0.299

AccessionNo	GeneSymbol	hiPSCs/hESCs
IPI00797533.1	MRPL46	-0.18375
IPI00797574.3	BOD1L	0.358
IPI00797796.1	CDC27	0.3685
IPI00798092.1	BAG4	0.7895
IPI00807491.2	GTF3C1	0.30575
IPI00807597.1	HELLS	0.51
IPI00815770.2	SNX3	1.30525
IPI00816106.3	DOCK7	0.75725
IPI00816829.2	TWF1	0.812
IPI00827831.3	PSMF1	1.6295
IPI00828082.1	MYO18A	-0.2395
IPI00828098.2	RNF213	0.08
IPI00829652.1	RBM16	0.2225
IPI00829741.2	ANKLE2	0.25375
IPI00829808.1	EXOC5	0.486
IPI00829826.1	MED1	0.3995
IPI00829835.2	GPATCH4	0.9375
IPI00829908.1	U2SURP	0.43525
IPI00829966.1	TMEM126B	-0.36475
IPI00830039.1	U2AF2	0.15275
IPI00830108.1	DNAJC2	0.378
IPI00843789.2	GLDC	1.906
IPI00843790.1	USP36	0.14725
IPI00843876.1	TNPO1	0.216
IPI00843886.1	APOO	-0.53325
IPI00843975.1	EZR	0.58925
IPI00843996.1	SRSF3	0.039
IPI00844000.1	UFL1	-0.11975
IPI00844090.1	COL5A1	1.94725

Table S4 The integrated datasets for the comparison of hiPSCs/HFs, hESCs/HFs, and hiPSCs/hESCs from the public datasets.

AccessionNo	GeneSymbol	hiPSCs/HFs
IPI00927538.1	TBL2	-1.87825
IPI00477971.4	TBL3	1.5975
IPI00640595.2	TBP	1.57
IPI00940204.1	TBRG4	1.34225
IPI00935732.1	TCEA1	0.79075
IPI00759607.1	TCEAL6	-2.674
IPI00917694.1	TCEB2	-0.634
IPI00744062.1	TCERG1	0.981
IPI00012966.1	TCF12	-0.067
IPI00220381.2	TCF20	1.5225
IPI00299719.2	TCIRG1	-3.9845
IPI00290566.1	TCP1	-0.49
IPI00100656.3	TECR	0.40075
IPI00024214.1	TERF2	0.82375
IPI00008961.1	TERF2IP	-0.59225
IPI00642510.3	TEX10	1.8995
IPI00022463.1	TF	2.72675
IPI00020928.1	TFAM	2.228
IPI00291525.1	TFB1M	1.0715
IPI00034069.1	TFB2M	-0.03925
IPI00788849.1	TFG	-1.772
IPI00015924.1	TFIP11	1.23125
IPI00022462.2	TFRC	-1.4005
IPI00855725.1	TGOLN2	-1.517
IPI00296099.6	THBS1	-6.02475
IPI00305374.5	THOC1	1.33375
IPI00433833.1	THOC3	1.4145
IPI00328840.9	THOC4	0.85225
IPI00291131.6	THOC7	1.52275

AccsessionNo	GeneSymbol	hESCs/HFs
IPI00843996.1	SRSF3	1.1995
IPI00844000.1	UFL1	-0.85825
IPI00844090.1	COL5A1	-5.54325
IPI00844112.1	SNX12	-1.049
IPI00844214.1	CTNNBL1	1.204
IPI00844287.2	NES	-2.24675
IPI00844507.2	C1orf174	0.31775
IPI00844508.1	CGN	3.18475
IPI00844524.1	BSDC1	-1.38775
IPI00844578.1	DHX9	1.38225
IPI00845388.1	DSTN	-2.144
IPI00845446.2	PDS5B	1.73775
IPI00845478.1	ERBB2	2.21425
IPI00847523.2	SMG8	-0.08925
IPI00847550.1	PHF3	2.27275
IPI00847986.1	RPS24	-0.4415
IPI00848226.1;P680	GNB2L1	-0.21875
IPI00852685.1	DIAPH1	-0.64225
IPI00852693.1	LMF2	-3.23325
IPI00852778.1	SP3	0.96425
IPI00852804.1	PTPN9	-1.08125
IPI00853059.2	FUBP1	0.204
IPI00853320.1	CELF2	1.79325
IPI00853369.1	PLXNB2	-1.99975
IPI00853400.1	FKBP15	-2.75625
IPI00854766.1	TXNDC15	-1.74375
IPI00855725.1	TGOLN2	-2.0575
IPI00855833.1	PRR12	1.61275
IPI00855846.1	C6orf115	1.2145

AccessionNo	GeneSymbol	hiPSCs/hESCs
IPI00844112.1	SNX12	0.289
IPI00844214.1	CTNNBL1	0.02525
IPI00844287.2	NES	0.772
IPI00844507.2	C1orf174	0.68675
IPI00844508.1	CGN	-0.09775
IPI00844524.1	BSDC1	0.42225
IPI00844578.1	DHX9	0.24875
IPI00845388.1	DSTN	0.375
IPI00845446.2	PDS5B	0.30625
IPI00845478.1	ERBB2	0.43475
IPI00847523.2	SMG8	0.268
IPI00847550.1	PHF3	0.0045
IPI00847986.1	RPS24	0.46325
IPI00848226.1;P680	GNB2L1	0.31075
IPI00852685.1	DIAPH1	0.62225
IPI00852693.1	LMF2	0.393
IPI00852778.1	SP3	0.2245
IPI00852804.1	PTPN9	0.52775
IPI00853059.2	FUBP1	0.31775
IPI00853320.1	CELF2	-0.27975
IPI00853369.1	PLXNB2	0.19925
IPI00853400.1	FKBP15	0.44975
IPI00854766.1	TXNDC15	0.9445
IPI00855725.1	TGOLN2	0.785
IPI00855833.1	PRR12	0.4445
IPI00855846.1	C6orf115	0.66775
IPI00855998.1	CENPF	0.413
IPI00856075.1	SRSF8	0.4175
IPI00867529.1	KIF2A	0.1255

Table S4 The integrated datasets for the comparison of hiPSCs/HFs, hESCs/HFs, and hiPSCs/hESCs from the public datasets.

AccessionNo	GeneSymbol	hiPSCs/HFs
IPI00549189.4	THOP1	-0.90625
IPI00104050.3	THRAP3	1.31575
IPI00550243.2	THUMPD1	-0.0035
IPI00555577.1	THY1	-2.08675
IPI00383163.5	THYN1	1.9165
IPI00926358.1	TIA1	1.08575
IPI00644708.1	TIAL1	-0.038
IPI00001543.1	TIMM10	0.36175
IPI00001589.1	TIMM13	1.64625
IPI00306516.1	TIMM44	0.9085
IPI00656071.1	TIMM50	0.71075
IPI00028376.1	TIMM8A	0.90925
IPI00001541.1	TIMM9	0.36475
942979.1;IPI00643	TKT	0.03125
IPI00177938.2	TLE3	1.89
IPI00746072.3	TLK1	1.11275
IPI00298994.6	TLN1	-3.884
IPI00219299.4	TLN2	-0.635
IPI00030847.3	TM9SF3	-0.5125
IPI00028055.4	TMED10	-0.941
IPI00604599.2	TMED3	0.00175
IPI00914908.1	TMED4	-0.3445
IPI00023542.6	TMED9	-1.302
IPI00395903.1	TMEM106B	-0.8495
IPI00871302.1	TMEM120A	-0.8455
IPI00829966.1	TMEM126B	0.34575
IPI00307572.1	TMEM165	-1.0835
IPI00472498.3	TMEM189	-0.93275
IPI00644795.3	TMEM2	-0.9275

AccsessionNo	GeneSymbol	hESCs/HFs
IPI00855998.1	CENPF	1.52125
IPI00856075.1	SRSF8	1.8545
IPI00867529.1	KIF2A	0.5435
IPI00868781.2	CDK12	1.0945
IPI00869040.2	NUBP1	-0.6965
IPI00869148.1	OSGEPL1	1.5245
IPI00871221.1	ATP1B1	0.52
IPI00871233.1	C1orf55	1.5685
IPI00871302.1	TMEM120A	-0.559
IPI00871372.1	HECTD1	-1.23225
IPI00871437.1	CTDSPL2	2.45225
IPI00871679.3	9-Sep	-2.30275
IPI00871779.2	GTSE1	1.806
IPI00872240.1	ARAF	-1.16925
IPI00872246.1	LCMT1	0.382
IPI00872379.1	ANXA5	-3.371
IPI00872438.1	RECQL	-0.963
IPI00872454.1	CHTF18	1.87025
IPI00872495.2	RSRC2	2.2535
IPI00872780.1	ANXA4	-3.95225
IPI00872814.1	MSN	-3.43975
IPI00872879.1	COX7A2	0.3275
IPI00872952.1	UQCRH	1.1915
IPI00873067.1	TNRC6B	0.178
IPI00873244.1	CSDE1	-0.61425
IPI00873341.2	PTPRG	-0.27825
IPI00873472.1	SEC24A	-2.43375
IPI00873484.1	CAPZA1	-1.7
IPI00873586.3	PPP6R1	-1.063

AccessionNo	GeneSymbol	hiPSCs/hESCs
IPI00868781.2	CDK12	0.40925
IPI00869040.2	NUBP1	1.283
IPI00869148.1	OSGEPL1	0.041
IPI00871221.1	ATP1B1	0.6825
IPI00871233.1	C1orf55	0.209
IPI00871302.1	TMEM120A	-0.0115
IPI00871372.1	HECTD1	0.4735
IPI00871437.1	CTDSPL2	0.6145
IPI00871535.1	SPTAN1	-0.22575
IPI00871679.3	9-Sep	0.5355
IPI00871779.2	GTSE1	0.09075
IPI00872240.1	ARAF	1.07375
IPI00872246.1	LCMT1	0.2465
IPI00872379.1	ANXA5	0.3555
IPI00872438.1	RECQL	0.04375
IPI00872454.1	CHTF18	-0.23425
IPI00872495.2	RSRC2	-0.05225
IPI00872780.1	ANXA4	0.171
IPI00872814.1	MSN	0.6105
IPI00872879.1	COX7A2	0.19575
IPI00872952.1	UQCRH	-0.354
IPI00873067.1	TNRC6B	0.157
IPI00873244.1	CSDE1	0.71375
IPI00873341.2	PTPRG	1.3145
IPI00873472.1	SEC24A	1.02425
IPI00873484.1	CAPZA1	0.3575
IPI00873586.3	PPP6R1	0.133
IPI00873684.1	COL4A1	1.3565
IPI00873723.2	SUN1	-0.1245

Table S4 The integrated datasets for the comparison of hiPSCs/HFs, hESCs/HFs, and hiPSCs/hESCs from the public datasets.

AccessionNo	GeneSymbol	hiPSCs/HFs
IPI00063130.2	TMEM205	0.479
IPI00890756.1	TMEM209	0.84725
IPI00760748.1	TMEM30A	-0.02175
IPI00299084.1	TMEM33	0.27175
IPI00301280.2	TMEM43	-2.09675
IPI00746642.1	TMEM48	1.76525
IPI00005087.1	TMOD3	-0.64375
IPI00030131.3	TMPO	1.6405
IPI00216230.3	TMPO	0.8565
00220827.5;Q6ZW	TMSB10	-3.27775
IPI00180240.2	TMSL3	-0.65825
IPI00470924.2	TMTC3	-0.515
IPI00395887.4	TMX1	0.32
IPI00556619.1	TMX2	-0.10125
IPI00296380.1	TNFRSF10B	0.516
IPI00549343.3	TNFRSF9	-2.42525
IPI00304589.4	TNKS1BP1	-1.4245
IPI00843876.1	TNPO1	-0.34275
IPI00419856.1	TNPO2	-0.2145
IPI00873067.1	TNRC6B	0.00075
IPI00549516.3	TOE1	1.672
IPI00879680.2	TOM1	-1.85775
IPI00024976.5	TOMM22	0.619
IPI00009946.4	TOMM34	-1.129
IPI00014053.3	TOMM40	0.88025
IPI00386294.2	TOMM6	0.93775
IPI00015602.1	TOMM70A	-0.11825
IPI00413611.1	TOP1	1.157
IPI00217709.1	TOP2B	1.8355

AccsessionNo	GeneSymbol	hESCs/HFs
IPI00873684.1	COL4A1	-0.9635
IPI00873723.2	SUN1	0.66075
IPI00873768.1	NEDD8	-0.966
IPI00873810.1	FKBP1A	-1.933
IPI00873926.1	CAPRIN1	-0.631
IPI00873959.3	MYO5A	-1.1325
IPI00874051.1	UBE2N	-0.52475
IPI00874215.2	EIF4G3	-1.16825
IPI00876931.1	INTS1	0.53125
IPI00877061.1	TTYH3	-2.1355
IPI00877964.1	APOL2	-2.64825
IPI00878075.1	RANBP1	0.03
IPI00878252.1	GTF3C2	2.45575
IPI00878536.1	PES1	0.4415
IPI00878612.1	MYADM	-1.49525
IPI00878749.1	LAGE3	0.4445
IPI00878876.1	SNRPD3	1.09225
IPI00879002.1	TTLL12	1.2345
IPI00879006.1	RRP7A	1.28225
IPI00879166.1	MTA1	2.51075
IPI00879180.1	ARVCF	2.559
IPI00879680.2	TOM1	-1.833
IPI00879702.2	RBBP7	1.20475
IPI00879999.1	DDX54	0.6965
IPI00883857.2;IPI00644	HNRNPU	1.171
IPI00883897.1	RELA	-3.80975
IPI00884070.1	ISOC1	1.622
IPI00884105.2	LAMP1	-1.0545
IPI00885081.1	HDGFRP2	-0.104

AccessionNo	GeneSymbol	hiPSCs/hESCs
IPI00873768.1	NEDD8	0.3895
IPI00873810.1	FKBP1A	0.66175
IPI00873926.1	CAPRIN1	0.6575
IPI00873959.3	MYO5A	-0.56075
IPI00874051.1	UBE2N	0.48625
IPI00874215.2	EIF4G3	0.45475
IPI00876931.1	INTS1	0.49275
IPI00877061.1	TTYH3	1.456
IPI00877964.1	APOL2	-0.86125
IPI00878075.1	RANBP1	0.4885
IPI00878252.1	GTF3C2	0.487
IPI00878536.1	PES1	0.66775
IPI00878612.1	MYADM	0.254
IPI00878749.1	LAGE3	0.05375
IPI00878876.1	SNRPD3	0.2555
IPI00879002.1	TTLL12	0.1175
IPI00879006.1	RRP7A	0.231
IPI00879166.1	MTA1	0.44075
IPI00879180.1	ARVCF	0.147
IPI00879680.2	TOM1	0.27225
IPI00879702.2	RBBP7	-0.35875
IPI00879999.1	DDX54	0.19575
IPI00883857.2;IPI00644	HNRNPU	0.29825
IPI00883897.1	RELA	0.4995
IPI00884070.1	ISOC1	0.36375
IPI00884105.2	LAMP1	0.43625
IPI00885081.1	HDGFRP2	0.146
IPI00885127.2	RBFOX2	0.7665
IPI00886797.1	PDE12	-0.32925

Table S4 The integrated datasets for the comparison of hiPSCs/HFs, hESCs/HFs, and hiPSCs/hESCs from the public datasets.

AccessionNo	GeneSymbol	hiPSCs/HFs
IPI00216816.1	TOP3B	0.61275
IPI00936738.1	TOR1AIP1	-1.41725
IPI00168878.1	TOR1AIP2	-1.4295
IPI00910152.1	TOX4	0.50875
IPI00029778.3	TP53BP1	-0.26125
IPI00337588.1	TP53BP2	0.0775
IPI00478302.2	TP53I11	-0.10975
IPI00009111.1	TPBG	-2.4435
IPI00896474.2	TPD52	2.159
IPI00010779.4	TPM4	-2.32075
IPI00742682.2	TPR	0.958
IPI00550900.1	TPT1	-1.8815
IPI00008477.1	TPX2	3.24775
IPI00013891.1	TRA2A	0.919
IPI00927500.1	TRA2B	1.30575
IPI00008732.1	TRABD	1.07
IPI00030275.5	TRAP1	1.4805
IPI00477812.3	TRIM22	-0.14175
IPI00029629.3	TRIM25	-1.932
IPI00438229.2	TRIM28	1.87425
IPI00719053.1	TRIM71	3.1795
IPI00741684.1	TRIOBP	-2.7765
IPI00003515.1	TRIP11	-1.648
IPI00018840.6	TRIP4	-1.52225
IPI00178861.1	TRMT1	0.931
IPI00009010.3	TRMT112	0.32375
IPI00334914.2	TRMT1L	1.04775
IPI00455268.4	TRMT5	0.3685
IPI00099311.3	TRMT6	0.05275

AccsessionNo	GeneSymbol	hESCs/HFs
IPI00885127.2	RBFOX2	-0.01175
IPI00886797.1	PDE12	-1.09925
IPI00887765.1	MAP1A	-4.80825
IPI00887796.1	CDKN2AIPNL	1.43275
IPI00888071.1	DHX40	0.79
IPI00889541.2;IPI00023	DDX17	1.21275
IPI00890756.1	TMEM209	1.2175
IPI00890827.1	PPP6C	-0.2235
IPI00892533.1	C7orf50	0.40575
IPI00892724.1	CDCA7L	3.22775
IPI00893013.1	XPO1	0.652
IPI00893035.1	CAD	0.56225
IPI00893087.1	LUC7L2	-0.005
IPI00893541.1	PDIA3	-0.39075
IPI00894366.1	ANKMY2	-2.05725
IPI00894485.1	SERF2	0.2245
IPI00896458.1	JMJD6	-2.0435
IPI00896474.2	TPD52	2.191
IPI00900318.1	NUP214	0.9345
IPI00900338.1	SMARCA4	2.16625
IPI00900361.1	NAF1	1.4765
IPI00903057.1	DDX23	1.2275
IPI00903145.1	RDX	-1.90125
IPI00903182.1	SMAD5	-0.33225
IPI00908327.1	RNF114	-0.523
IPI00908369.1	ABCC4	-1.992
IPI00908469.1	TUBB6	-4.002
IPI00908588.1	EIF4B	-0.77225
IPI00908660.1	WDR77	-0.659

AccessionNo	GeneSymbol	hiPSCs/hESCs
IPI00887765.1	MAP1A	-0.48275
IPI00887796.1	CDKN2AIPNL	0.377
IPI00888071.1	DHX40	1.05475
IPI00889541.2;IPI00023	DDX17	0.1065
IPI00890756.1	TMEM209	-0.1285
IPI00890827.1	PPP6C	0.338
IPI00892533.1	C7orf50	0.9705
IPI00892724.1	CDCA7L	0.096
IPI00893013.1	XPO1	0.20775
IPI00893035.1	CAD	0.106
IPI00893087.1	LUC7L2	0.33325
IPI00893541.1	PDIA3	0.2805
IPI00894366.1	ANKMY2	1.29275
IPI00894485.1	SERF2	0.1195
IPI00896458.1	JMJD6	0.64475
IPI00896474.2	TPD52	0.29775
IPI00900318.1	NUP214	0.06125
IPI00900338.1	SMARCA4	0.35125
IPI00900361.1	NAF1	0.42175
IPI00903057.1	DDX23	0.04275
IPI00903145.1	RDX	0.857
IPI00903182.1	SMAD5	0.69525
IPI00908327.1	RNF114	0.56425
IPI00908369.1	ABCC4	-0.7165
IPI00908469.1	TUBB6	0.30925
IPI00908588.1	EIF4B	0.654
IPI00908660.1	WDR77	0.5175
IPI00908696.2	ARL6IP5	-0.21225
IPI00909083.1	GSPT1	0.3425

Table S4 The integrated datasets for the comparison of hiPSCs/HFs, hESCs/HFs, and hiPSCs/hESCs from the public datasets.

AccessionNo	GeneSymbol	hiPSCs/HFs
IPI00301719.1	TRNT1	-0.47225
IPI00642416.1	TROVE2	-0.57075
IPI00926412.1	TRRAP	1.45675
IPI00103341.3	TRUB1	0.45025
IPI00019355.1	TSC22D1	-0.34025
IPI00917623.1	TSN	-0.346
IPI00293350.3	TSNAX	-0.44575
IPI00000735.5	TSPAN13	1.3835
IPI00292894.5	TSR1	0.782
IPI00216293.6	TST	-0.07025
IPI00014361.1	TSTA3	-0.6725
IPI00016912.1	TTC1	-0.23075
IPI00183938.6	TTC27	-0.34625
IPI00014149.3	TTC35	-0.26725
IPI00005634.3	TTC37	-1.75825
IPI00000606.5	TTC4	-0.52625
IPI00175096.1	TTC9C	-1.134
IPI00290812.2	TTF2	1.45225
IPI00879002.1	TTLL12	1.094
IPI00877061.1	TTYH3	-0.9735
IPI00218343.4	TUBA1C	-0.9785
IPI00794663.1	TUBA4A	0.6675
IPI00017454.4	TUBA4B	-1.03225
00013475.1;Q7TM	TUBB2A	0.1705
IPI00013683.2	TUBB3	-2.50675
I00023598.2;Q9D6	TUBB4	-1.06625
IPI00908469.1	TUBB6	-3.98225
IPI00295081.1	TUBG1	-0.4995
IPI00399300.1	TUBGCP3	-0.496

AccsessionNo	GeneSymbol	hESCs/HFs
IPI00908696.2	ARL6IP5	-2.085
IPI00909083.1	GSPT1	-0.88325
IPI00909122.1	PARN	1.528
IPI00909229.1	-	-0.53325
IPI00909232.1	HNRNPC	1.929
IPI00909237.1	SLC2A1	2.9535
IPI00909303.2	CTSB	-4.314
IPI00909319.2	DAGLB	0.0625
IPI00909336.1	-	-0.0255
IPI00909416.1	HACL1	3.508
IPI00909622.1	CSTF2	0.916
IPI00909703.2	ANXA11	-3.68125
IPI00909749.1	ALG5	-1.27175
IPI00909766.1	PSMD5	-2.30625
IPI00909828.1	BARD1	4.3105
IPI00909890.1	FUS	0.70425
IPI00909893.1	MED17	0.57975
IPI00910072.1	DHDPSL	-0.524
IPI00910109.1	C2orf56	4.078
IPI00910152.1	TOX4	0.35975
IPI00910174.1	NUPL1	1.27475
IPI00910327.1	ORC3	2.4
IPI00910339.1	PANK4	-1.0005
IPI00910350.1	MAPRE1	-0.50175
IPI00910377.1	MTG1	-0.86225
IPI00910458.1	HNRNPK	1.222
IPI00910583.1	MPP2	2.2115
IPI00910626.1	ELOVL1	-3.01025
IPI00910684.1	DAP3	0.212

AccessionNo	GeneSymbol	hiPSCs/hESCs
IPI00909122.1	PARN	0.08075
IPI00909229.1	-	0.51225
IPI00909232.1	HNRNPC	0.22975
IPI00909237.1	SLC2A1	-0.993
IPI00909303.2	CTSB	-0.05125
IPI00909319.2	DAGLB	0.36675
IPI00909336.1	-	0.25075
IPI00909416.1	HACL1	-0.6915
IPI00909622.1	CSTF2	0.17575
IPI00909703.2	ANXA11	0.567
IPI00909749.1	ALG5	0.1335
IPI00909766.1	PSMD5	0.44225
IPI00909828.1	BARD1	0.19275
IPI00909890.1	FUS	0.2675
IPI00909893.1	MED17	0.564
IPI00910072.1	DHDPSL	0.0585
IPI00910109.1	C2orf56	-1.37425
IPI00910152.1	TOX4	0.48025
IPI00910174.1	NUPL1	0.21
IPI00910327.1	ORC3	-0.143
IPI00910339.1	PANK4	0.34375
IPI00910350.1	MAPRE1	0.50775
IPI00910377.1	MTG1	1.47525
IPI00910458.1	HNRNPK	0.1555
IPI00910583.1	MPP2	0.4195
IPI00910626.1	ELOVL1	0.28725
IPI00910684.1	DAP3	0.35175
IPI00910706.1	ABHD14B	-0.40325
IPI00910819.1	WBP11	0.23675

Table S4 The integrated datasets for the comparison of hiPSCs/HFs, hESCs/HFs, and hiPSCs/hESCs from the public datasets.

AccessionNo	GeneSymbol	hiPSCs/HFs
IPI00027107.5	TUFM	0.3405
IPI00816829.2	TWF1	-3.775
IPI00550917.3	TWF2	-1.859
IPI00470779.2	TXLNA	-1.57025
IPI00019994.4	TXLNG	2.4185
IPI00026328.3	TXNDC12	0.173
IPI00854766.1	TXNDC15	-1.14
IPI00646689.1	TXNDC17	-0.8045
IPI00395646.1	TXNDC5	-1.7045
IPI00305692.5	TXNL1	-0.42275
IPI00554786.5	TXNRD1	-2.66575
IPI00005613.3	U2AF1	0.57325
IPI00830039.1	U2AF2	0.6605
IPI00829908.1	U2SURP	1.2375
IPI00217816.3	UAP1	-5.09175
IPI00645078.1	UBA1	-0.60975
IPI00023234.3	UBA2	-0.0435
IPI00375533.5	UBA3	-0.0165
IPI00023647.4	UBA6	-1.1485
IPI00171127.1	UBAP2	0.61075
IPI00514856.4	UBAP2L	-0.5455
IPI00032957.1	UBE2I	0.65825
I00022597.1;P610	UBE2M	-0.68575
IPI00874051.1	UBE2N	-0.312
IPI00783378.3	UBE2O	0.6425
IPI00217949.12	UBE2S	1.976
IPI00019600.3	UBE2V2	-0.2775
IPI00219197.3	UBE3A	-0.21
IPI00643653.1	UBFD1	-0.804

AccsessionNo	GeneSymbol	hESCs/HFs
IPI00910706.1	ABHD14B	-3.3705
IPI00910819.1	WBP11	1.18825
IPI00911038.2	ACIN1	1.84275
IPI00911039.1	HSPA1B	-0.41525
IPI00913991.1	PDHX	1.192
IPI00914615.1	DNM3	-1.0385
IPI00914908.1	TMED4	0.28575
IPI00914913.1	PRMT1	-0.826
IPI00914938.1	AP2A2	-0.99175
IPI00914971.1	FDPS	0.12375
IPI00915304.1	ARFGAP3	-3.29425
IPI00916026.1	GIGYF2	-0.3665
IPI00916111.2	MDH1	-0.3755
IPI00916299.1	GORASP2	-0.9135
IPI00916534.1	AAK1	-3.84475
IPI00916757.1	DCTN1	-2.43175
IPI00916796.1	DSCR3	-1.821
IPI00916892.1	NUP35	1.66375
IPI00916940.1	AARSD1	-0.5255
IPI00917016.1	CPNE1	-0.66975
IPI00917244.1	PPHLN1	0.76675
IPI00917313.1	HN1	-1.27225
IPI00917561.1	RNF181	-1.953
IPI00917605.1	CYCS	1.67575
IPI00917623.1	TSN	-0.49675
IPI00917694.1	TCEB2	-0.619
IPI00917777.1	EFTUD2	1.235
IPI00918020.1	-	-4.6325
IPI00921173.1	SUCLG1	-0.6575

AccessionNo	GeneSymbol	hiPSCs/hESCs
IPI00911038.2	ACIN1	0.08575
IPI00911039.1	HSPA1B	0.004
IPI00913991.1	PDHX	-0.217
IPI00914615.1	DNM3	-0.027
IPI00914908.1	TMED4	-0.258
IPI00914913.1	PRMT1	1.31975
IPI00914938.1	AP2A2	0.39925
IPI00914971.1	FDPS	0.88875
IPI00915304.1	ARFGAP3	0.6685
IPI00916026.1	GIGYF2	0.2865
IPI00916111.2	MDH1	0.69625
IPI00916299.1	GORASP2	-0.14675
IPI00916534.1	AAK1	0.98
IPI00916757.1	DCTN1	0.493
IPI00916796.1	DSCR3	0.10425
IPI00916892.1	NUP35	0.24775
IPI00916940.1	AARSD1	0.402
IPI00917016.1	CPNE1	-0.04525
IPI00917244.1	PPHLN1	-0.133
IPI00917313.1	HN1	0.93675
IPI00917561.1	RNF181	1.23825
IPI00917605.1	CYCS	-0.11025
IPI00917623.1	TSN	0.55025
IPI00917694.1	TCEB2	0.26625
IPI00917777.1	EFTUD2	0.18325
IPI00918020.1	-	-0.101
IPI00921173.1	SUCLG1	-0.018
IPI00921488.1	GPS1	0.44625
IPI00921612.1	GPN1	0.40175

Table S4 The integrated datasets for the comparison of hiPSCs/HFs, hESCs/HFs, and hiPSCs/hESCs from the public datasets.

AccessionNo	GeneSymbol	hiPSCs/HFs
IPI00291669.3	UBLCP1	-0.192
IPI00024502.2	UBQLN4	1.079
IPI00646605.3	UBR4	-0.555
IPI00220833.1	UBTF	1.353
IPI00027378.5	UBXN1	-0.99475
IPI00293946.5	UBXN4	-0.8585
IPI00163100.1	UBXN6	-1.916
IPI00742124.2	UBXN7	0.949
IPI00018352.1	UCHL1	-1.084
IPI00011250.3	UCHL3	-1.1735
IPI00219512.2	UCHL5	-0.68925
IPI00065671.1	UCK2	0.2245
IPI00294495.5	UFC1	-0.77575
IPI00844000.1	UFL1	-1.275
IPI00031420.3	UGDH	-2.114
IPI00619903.3	UGGT1	-0.78575
IPI00329331.6	UGP2	1.63875
IPI00942199.1	UHRF1	1.64125
IPI00003923.1	UMPS	0.184
IPI00414629.2	UNC119B	0.165
IPI00478689.5	UNC13B	1.366
IPI00735181.2	UNC45A	-1.231
IPI00399170.1	UPF1	-0.626
IPI00300504.5	UPF2	-0.1345
IPI00220416.3	UQCRB	0.5815
IPI00013847.4	UQCRC1	0.43
IPI00305383.1	UQCRC2	0.52475
IPI00026964.2	UQCRFS1	0.57325
IPI00872952.1	UQCRH	0.4535

AccsessionNo	GeneSymbol	hESCs/HFs
IPI00921488.1	GPS1	-1.2115
IPI00921612.1	GPN1	0.2285
IPI00921822.2	PNPLA6	-0.55125
IPI00921892.1	KIN	1.3025
IPI00921986.1	ACOT9	-0.48325
IPI00922108.1	ITGAV	-1.73775
IPI00922140.1	FAM83D	1.132
IPI00922290.1	-	-1.34275
IPI00922336.1	TBL1XR1	0.30325
IPI00922466.1	-	2.757
IPI00922479.1	PEX14	-0.075
IPI00922554.1	DNASE2	0.45175
IPI00923524.1	SPAST	0.48725
IPI00923531.1	NCOR2	-0.14725
IPI00924477.1	AAMP	0.18725
IPI00924582.1	ECT2	2.339
IPI00924636.1	COPS6	-1.3835
IPI00924776.1	BET1	0.5665
IPI00924782.1	LOC729020	-1.70525
IPI00924816.1	MTPN	-2.35025
IPI00924819.1	MANF	-0.53325
IPI00925612.1	NCEH1	-5.78
IPI00925737.1	PSPH	-0.634
IPI00926241.1	TBC1D4	1.10725
IPI00926312.1	OGDH	0.0765
IPI00926358.1	TIA1	1.00875
IPI00926412.1	TRRAP	1.9765
IPI00926421.1	PODXL	3.2445
IPI00926423.2	FADS1	1.4495

AccessionNo	GeneSymbol	hiPSCs/hESCs
IPI00921822.2	PNPLA6	-0.14925
IPI00921892.1	KIN	0.68725
IPI00921986.1	ACOT9	0.43075
IPI00922108.1	ITGAV	0.8585
IPI00922140.1	FAM83D	-1.14525
IPI00922290.1	-	-0.02525
IPI00922336.1	TBL1XR1	0.51625
IPI00922466.1	-	0.46175
IPI00922479.1	PEX14	-0.21125
IPI00922554.1	DNASE2	-0.1835
IPI00923524.1	SPAST	0.1335
IPI00923531.1	NCOR2	0.0745
IPI00924477.1	AAMP	0.6995
IPI00924582.1	ECT2	0.5085
IPI00924636.1	COPS6	0.34525
IPI00924776.1	BET1	-0.14575
IPI00924782.1	LOC729020	0.2345
IPI00924816.1	MTPN	0.623
IPI00924819.1	MANF	0.2285
IPI00925612.1	NCEH1	-0.167
IPI00925737.1	PSPH	0.2875
IPI00925950.1	TNS1	0.22
IPI00926241.1	TBC1D4	-0.49675
IPI00926312.1	OGDH	0.137
IPI00926358.1	TIA1	0.36075
IPI00926412.1	TRRAP	-0.98425
IPI00926421.1	PODXL	0.09625
IPI00926423.2	FADS1	1.227
IPI00926625.1	ZYX	0.98925

Table S4 The integrated datasets for the comparison of hiPSCs/HFs, hESCs/HFs, and hiPSCs/hESCs from the public datasets.

AccessionNo	GeneSymbol	hiPSCs/HFs
IPI00063903.5	USMG5	0.1575
IPI00941161.1	USO1	-1.1385
IPI00640357.1	USP14	-0.469
IPI00219504.1	USP15	-1.57275
IPI00843790.1	USP36	1.91425
IPI00375145.1	USP5	-1.12775
IPI00646721.1	USP7	0.731
IPI00003964.4	USP9X	0.2985
IPI00180454.4	UTP11L	1.639
IPI00152708.3	UTP15	1.68475
IPI00000733.4	UTP18	1.6775
IPI00004970.4	UTP20	1.61975
IPI00020128.2	UTP6	1.65175
IPI00009329.2	UTRN	-1.3835
IPI00025160.2	VAC14	0.12525
IPI00401804.3	VAMP7	-0.6285
IPI00170692.4	VAPA	-1.001
IPI00006211.4	VAPB	-0.01125
IPI00000873.3	VARS	-0.76325
IPI00640597.1	VARS2	2.4485
IPI00301058.5	VASP	-1.51825
IPI00156689.3	VAT1	-0.86225
IPI00030578.2	VAT1L	1.4645
IPI00334159.6	VBP1	-0.6125
IPI00215630.1	VCAN	1.84575
IPI00291175.7	VCL	-1.97975
I00022774.3;Q018	VCP	-0.785
IPI00216308.5	VDAC1	-0.1725
IPI00031804.1	VDAC3	0.129

AccsessionNo	GeneSymbol	hESCs/HFs
IPI00926625.1	ZYX	-4.12875
IPI00926728.1	WDR6	-1.543
IPI00927054.1	10-Sep	1.36625
IPI00927101.1	SNORA6	-0.20225
IPI00927191.1	GLB1	-0.18175
IPI00927374.1	MKRN2	-0.65675
IPI00927458.1	RPL32	-0.39225
IPI00927500.1	TRA2B	1.486
IPI00927538.1	TBL2	-1.22375
IPI00927606.1	GPX1	1.065
IPI00927674.1	RAB5A	0.09675
IPI00927677.1	HNRNPA3	1.36225
IPI00927876.1	POLR2H	1.042
IPI00927892.1	PMPCB	0.27575
IPI00929375.1	HDAC2	1.83475
IPI00929723.1	WDR4	-0.6595
IPI00930282.1	SELS	-0.26125
IPI00930346.1	MORF4L1	1.98275
IPI00930557.1	FGF2	0.36875
IPI00930609.1	PSAT1	0.35475
IPI00930723.1	RIOK2	-1.818
IPI00935435.1	MED18	0.22075
IPI00935702.2	SMARCD2	1.3295
IPI00935722.1	OTUD6B	0.2965
IPI00935729.1	HBXIP	0.13675
IPI00935732.1	TCEA1	1.35875
IPI00936002.1	ALDH7A1	1.266
IPI00936738.1	TOR1AIP1	-0.75775
IPI00936931.1	ERLIN1	-0.258

AccessionNo	GeneSymbol	hiPSCs/hESCs
IPI00926728.1	WDR6	1.04375
IPI00927054.1	10-Sep	0.634
IPI00927101.1	SNORA6	0.2745
IPI00927191.1	GLB1	-0.1055
IPI00927374.1	MKRN2	-0.68325
IPI00927458.1	RPL32	0.32575
IPI00927500.1	TRA2B	0.15675
IPI00927538.1	TBL2	-0.378
IPI00927606.1	GPX1	0.0365
IPI00927674.1	RAB5A	-0.04825
IPI00927677.1	HNRNPA3	0.135
IPI00927876.1	POLR2H	0.3295
IPI00927892.1	PMPCB	0.3565
IPI00929375.1	HDAC2	0.24925
IPI00929723.1	WDR4	0.53375
IPI00930282.1	SELS	0.5555
IPI00930346.1	MORF4L1	0.179
IPI00930557.1	FGF2	0.789
IPI00930609.1	PSAT1	0.861
IPI00930723.1	RIOK2	0.367
IPI00935435.1	MED18	0.41475
IPI00935702.2	SMARCD2	0.62625
IPI00935722.1	OTUD6B	0.16975
IPI00935729.1	HBXIP	-0.10175
IPI00935732.1	TCEA1	0.067
IPI00936002.1	ALDH7A1	0.028
IPI00936738.1	TOR1AIP1	-0.24975
IPI00936931.1	ERLIN1	-0.08925
IPI00936987.1	NSF	0.24025

Table S4 The integrated datasets for the comparison of hiPSCs/HFs, hESCs/HFs, and hiPSCs/hESCs from the public datasets.

AccessionNo	GeneSymbol	hiPSCs/HFs
IPI00418471.6	VIM	-6.31725
IPI00410112.2	VKORC1	-1.47675
IPI00604660.4	VPS13A	0.98525
IPI00604778.1	VPS13C	-1.22075
IPI00411426.3	VPS26A	-1.944
IPI00170796.1	VPS29	-2.705
IPI00032905.2	VPS33B	-0.3345
IPI00018931.6	VPS35	-1.81125
IPI00090327.1	VPS45	-0.5095
IPI00411356.5	VPS4A	-1.06425
IPI00182728.2	VPS4B	-0.88325
IPI00019640.1	VRK1	2.40675
IPI00303992.3	VRTN	1.17475
IPI00216313.7	VSNL1	1.106
IPI00375330.6	WAPAL	1.22575
IPI00295400.1	WARS	-1.0195
IPI00299003.2	WASF3	0.2165
IPI00011676.2	WASL	-0.5465
IPI00910819.1	WBP11	1.10975
IPI00411614.1	WDHD1	1.93425
IPI00746165.2	WDR1	-1.9845
IPI00304232.1	WDR12	1.02375
IPI00032533.3	WDR18	1.71625
IPI00009471.1	WDR3	1.5855
IPI00106567.2	WDR33	1.6575
IPI00169325.1	WDR36	1.5255
IPI00929723.1	WDR4	-0.4245
IPI00937477.1	WDR43	1.6105
IPI00023126.1	WDR46	1.256

AccsessionNo	GeneSymbol	hESCs/HFs
IPI00936987.1	NSF	-2.32075
IPI00937477.1	WDR43	1.89025
IPI00937615.2;IPI00000	EEF1G	-1.5045
IPI00938044.1	PTPLAD1	0.8915
IPI00939119.1	CCAR1	1.4775
IPI00939140.1	NHP2L1	1.39
IPI00939159.2	CAP1	-3.2795
IPI00939174.1	OTUB1	-0.57625
IPI00939242.1	RAB4A	-1.59175
IPI00939281.1	C6orf120	-0.3735
IPI00939308.2	MRPL48	0.0715
IPI00939707.1	KIAA0664	-0.132
IPI00939720.1	BIRC6	-0.37875
IPI00939822.1	HGS	-2.075
IPI00939839.1	CLINT1	-1.18475
IPI00939968.1	SENP3	1.01625
IPI00939991.1	C10ORF58	2.60475
IPI00940039.1	POLDIP3	1.64475
IPI00940095.1	NUP54	1.25175
IPI00940115.1	DOCK6	0.95375
IPI00940148.1;IPI00031	GDI2	-0.69525
IPI00940204.1	TBRG4	1.22225
IPI00940237.1;IPI00644	DDX39A	-0.36525
IPI00940320.1	C1ORF144	-1.48675
IPI00940326.1	CORO7	1.37525
IPI00940497.1	SLC25A12	-0.70125
IPI00940513.1	RBP1	1.3475
IPI00940535.1	MAD1L1	0.77425
IPI00940660.1	PAPD5	1.675

AccessionNo	GeneSymbol	hiPSCs/hESCs
IPI00937477.1	WDR43	-0.014
IPI00937615.2;IPI00000	EEF1G	0.26025
IPI00938044.1	PTPLAD1	0.23125
IPI00939119.1	CCAR1	-0.0865
IPI00939140.1	NHP2L1	0.01525
IPI00939159.2	CAP1	0.719
IPI00939174.1	OTUB1	0.42675
IPI00939242.1	RAB4A	0.789
IPI00939281.1	C6orf120	0.20175
IPI00939308.2	MRPL48	-0.06325
IPI00939707.1	KIAA0664	0.27675
IPI00939720.1	BIRC6	0.651
IPI00939822.1	HGS	0.514
IPI00939839.1	CLINT1	-0.03325
IPI00939968.1	SENP3	0.32625
IPI00939991.1	C10ORF58	0.9935
IPI00940039.1	POLDIP3	0.308
IPI00940095.1	NUP54	0.0395
IPI00940115.1	DOCK6	-0.19875
IPI00940148.1;IPI00031	GDI2	0.52575
IPI00940204.1	TBRG4	0.4295
IPI00940237.1;IPI00644	DDX39A	0.2345
IPI00940320.1	C1ORF144	0.35025
IPI00940326.1	CORO7	-0.27175
IPI00940497.1	SLC25A12	0.30375
IPI00940513.1	RBP1	1.06225
IPI00940535.1	MAD1L1	0.02375
IPI00940660.1	PAPD5	-0.18275
IPI00940685.1	SNRNP40	0.2155

Table S4 The integrated datasets for the comparison of hiPSCs/HFs, hESCs/HFs, and hiPSCs/hESCs from the public datasets.

AccessionNo	GeneSymbol	hiPSCs/HFs
IPI00658210.1	WDR48	0.74675
IPI00005492.2	WDR5	1.1945
IPI00926728.1	WDR6	-0.787
IPI00019269.3	WDR61	-0.20775
IPI00300060.4	WDR70	0.24625
IPI00217240.1	WDR75	1.6665
IPI00908660.1	WDR77	-0.3835
IPI00062040.1	WDR89	1.62375
IPI00008711.3	WFS1	-1.84125
IPI00790634.2	WIBG	0.65525
IPI00741630.4	WIZ	1.602
IPI00220302.1	WTAP	1.483
IPI00013010.6	WWP2	0.48425
IPI00163084.3	XAB2	1.14475
IPI00156793.5	XPC	0.77525
IPI00793375.1	XPNPEP1	-1.84375
IPI00893013.1	XPO1	0.58725
IPI00028357.2	XPO4	0.70325
IPI00640703.3	XPO5	1.90875
IPI00302458.10	XPO7	0.3725
IPI00306290.5	XPOT	-0.321
IPI00002564.3	XRCC1	1.36125
IPI00220834.8	XRCC5	0.796
IPI00644712.4	XRCC6	0.79
IPI00216919.1	YAP1	-1.11
IPI00007074.5	YARS	-0.435
IPI00165092.3	YARS2	1.1675
IPI00031812.3	YBX1	-0.78125
IPI00008536.3	YEATS4	1.69125

AccsessionNo	GeneSymbol	hESCs/HFs
IPI00940685.1	SNRNP40	1.3735
IPI00940744.1	NDUFS1	0.05025
IPI00940786.1;IPI00013	MCM3	2.27125
IPI00940798.1	MRE11A	0.7945
IPI00940809.1	IGF1R	1.5055
IPI00940851.1;IPI00301	ELAVL1	1.2605
IPI00940901.1;IPI00294	RRP12	0.89625
IPI00940934.1	EML4	0.3255
IPI00940960.1	NPC2	1.07025
IPI00941161.1	USO1	-0.884
IPI00941167.1	HEXA	-0.59475
IPI00941172.1;P61620;IPI0(	SEC61A1	-1.597
IPI00941179.1	RASA1	-2.96975
IPI00941201.1	PIN4	0.07025
IPI00941255.1	EIF3F	-0.77675
IPI00941359.1	EIF3H	-0.6525
IPI00941463.1;IPI00017	NAP1L4	-0.631
IPI00941557.1;IPI00550	PYCR1	0.72725
IPI00941589.1	EIF4G2	-0.71025
IPI00941628.2	OPLAH	0.42575
IPI00941678.1	ARHGEF2	-0.658
IPI00941747.1;IPI00020	CANX	-0.33725
IPI00941810.1	NOMO3	-0.5225
IPI00941900.1	CALU	-2.76175
IPI00941907.1	STRAP	-0.66125
IPI00942092.1	ADSL	-0.2205
IPI00942186.1	AKAP1	3.93875
IPI00942199.1	UHRF1	-0.97275
IPI00942294.1	MTAP	-0.737

AccessionNo	GeneSymbol	hiPSCs/hESCs
IPI00940744.1	NDUFS1	0.1025
IPI00940786.1;IPI00013	MCM3	0.10825
IPI00940798.1	MRE11A	0.10425
IPI00940809.1	IGF1R	0.799
IPI00940851.1;IPI00301	ELAVL1	0.08375
IPI00940901.1;IPI00294	RRP12	0.2375
IPI00940934.1	EML4	0.12725
IPI00940960.1	NPC2	-0.065
IPI00941161.1	USO1	0.05125
IPI00941167.1	HEXA	-0.3245
IPI00941172.1;P61620;IPI0(	SEC61A1	0.3825
IPI00941179.1	RASA1	0.9685
IPI00941201.1	PIN4	0.571
IPI00941255.1	EIF3F	0.2975
IPI00941359.1	EIF3H	0.31875
IPI00941463.1;IPI00017	NAP1L4	0.703
IPI00941557.1;IPI00550	PYCR1	-0.0895
IPI00941589.1	EIF4G2	0.43625
IPI00941628.2	OPLAH	0.74975
IPI00941678.1	ARHGEF2	-0.2315
IPI00941747.1;IPI00020	CANX	0.0485
IPI00941810.1	NOMO3	0.798
IPI00941900.1	CALU	0.321
IPI00941907.1	STRAP	0.42175
IPI00942092.1	ADSL	0.64975
IPI00942186.1	AKAP1	-0.5095
IPI00942199.1	UHRF1	3.0405
IPI00942294.1	MTAP	0.47375
IPI00942495.1	MED6	0.29975

Table S4 The integrated datasets for the comparison of hiPSCs/HFs, hESCs/HFs, and hiPSCs/hESCs from the public datasets.

AccessionNo	GeneSymbol	hiPSCs/HFs
IPI00013981.4	YES1	0.73875
IPI00334657.7	YIPF5	-2.15125
IPI00061403.4	YIPF6	-0.32775
IPI00219685.5	YJEFN3	-0.07075
IPI00099529.3	YME1L1	-0.01275
IPI00376222.1	YTHDC1	1.37325
IPI00221345.1	YTHDF1	0.13225
IPI00397024.1	YTHDF2	0.474
IPI00759832.1	YWHAB	-1.28825
I00000816.1;P622	YWHAE	-0.105
I00220642.7;P619	YWHAG	-0.67025
IPI00216319.3	YWHAH	-0.408
IPI00018146.1	YWHAQ	-0.899
IPI00021263.3	YWHAZ	-1.068
IPI00014513.1	YY1	1.69075
IPI00000279.3	ZC3H15	0.2255
IPI00328929.4	ZC3H18	1.235
IPI00187011.4	ZC3H4	0.6435
IPI00216361.1	ZC3H7B	-2.01825
IPI00945081.1	ZC3HC1	0.84775
IPI00556594.3	ZCCHC8	1.533
IPI00004523.2	ZFAND6	1.6285
IPI00175146.5	ZFC3H1	2.489
IPI00782966.1	ZFP106	2.74175
IPI00748303.3	ZFR	0.68325
IPI00027180.1	ZMPSTE24	-0.73725
IPI00294603.6	ZMYM2	3.29525
IPI00642195.2	ZMYM4	2.17925
IPI00025310.1	ZNF217	2.40525

AccsessionNo	GeneSymbol	hESCs/HFs
IPI00942495.1	MED6	1.14075
IPI00942760.1	MRPS27	0.89
IPI00942761.1	TBC1D8B	-0.59825
IPI00942796.1	FKBP7	-5.02
IPI00942979.1;IPI00643	TKT	0.54525
IPI00943008.1	PLOD1	-2.46875
IPI00943132.1	SHC1	-1.7875
IPI00943173.1	CORO1C	-2.51775
IPI00943215.1	BID	-1.423
IPI00943593.1	FRAS1	-0.21475
IPI00943894.1	PYGL	0.30775
IPI00945081.1	ZC3HC1	1.0645
IPI00945153.1	NDUFA6	0.81525
IPI00945233.1	SLC25A11	0.49425
IPI00945264.1	RPL22L1	-1.853
IPI00945551.1	C3orf26	1.99975
IPI00945585.1	FAM162A	3.496
IPI00945719.1	CCDC58	2.55625
IPI00945732.1	ANAPC7	0.17325
IPI00945818.1	LRBA	1.98125
IPI00945855.1	PFN2	-1.1125
IPI00945880.1	MRPS33	0.12875
IPI00945960.1	SSR3	-1.476
IPI00945991.2	SEC62	-0.83575
IPI00946050.1	GAPVD1	-1.615
IPI00946099.1	SRI	-0.62075
IPI00946242.1	ERMP1	2.33725
IPI00946334.1	NDUFS2	0.3835
IPI00946527.1	AKR7A2	-1.08875

AccessionNo	GeneSymbol	hiPSCs/hESCs
IPI00942760.1	MRPS27	0.08725
IPI00942761.1	TBC1D8B	0.10125
IPI00942796.1	FKBP7	0.842
IPI00942979.1;IPI00643	TKT	-0.059
IPI00943008.1	PLOD1	0.17075
IPI00943132.1	SHC1	0.6835
IPI00943173.1	CORO1C	0.27125
IPI00943215.1	BID	-0.062
IPI00943593.1	FRAS1	1.852
IPI00943894.1	PYGL	-0.173
IPI00945081.1	ZC3HC1	0.0465
IPI00945153.1	NDUFA6	-0.037
IPI00945233.1	SLC25A11	-0.32675
IPI00945264.1	RPL22L1	0.53575
IPI00945551.1	C3orf26	0.256
IPI00945585.1	FAM162A	-0.841
IPI00945719.1	CCDC58	-0.48825
IPI00945732.1	ANAPC7	0.3175
IPI00945818.1	LRBA	0.4825
IPI00945855.1	PFN2	0.26575
IPI00945880.1	MRPS33	0.602
IPI00945960.1	SSR3	0.26575
IPI00945991.2	SEC62	0.02175
IPI00946050.1	GAPVD1	0.345
IPI00946099.1	SRI	-0.056
IPI00946242.1	ERMP1	-0.42775
IPI00946334.1	NDUFS2	-0.05675
IPI00946527.1	AKR7A2	-0.214
IPI00946732.1	RRM2	2.21575

Table S4 The integrated datasets for the comparison of hiPSCs/HFs, hESCs/HFs, and hiPSCs/hESCs from the public datasets.

AccessionNo	GeneSymbol	hiPSCs/HFs
IPI00002612.2	ZNF219	2.177
IPI00306446.2	ZNF24	1.2955
IPI00657874.1	ZNF259	-0.9
IPI00647664.7	ZNF281	1.60775
IPI00373877.3	ZNF326	1.434
IPI00004942.1	ZNF330	-1.29725
IPI00478631.6	ZNF512	0.89025
IPI00151988.4	ZNF532	3.76775
IPI00953294.1	ZNF598	-0.77075
IPI00056880.7	ZNF787	1.96875
IPI00064212.2	ZNF828	1.81375
IPI00219866.2	ZRANB2	-0.60375
IPI00011631.6	ZW10	-0.4445
IPI00926625.1	ZYX	-3.4585

AccsessionNo	GeneSymbol	hESCs/HFs
IPI00946732.1	RRM2	-0.153
IPI00946752.1	CPSF3	1.40025
IPI00946824.1	FXN	1.06325
IPI00947400.1	PLOD2	-2.70475
IPI00947492.1	NDUFB2	-0.5555
IPI00953262.1	CACNA2D1	-1.3775
IPI00953294.1	ZNF598	-0.85775
IPI00953297.1	ANXA7	-1.9445
IPI00953403.1	EXOC3	-1.298
IPI00953445.1;IPI00003	ERGIC1	-1.8565
IPI00953520.1	RNPS1	1.69275
IPI00953925.1	AIP	-1.35275
IPI00954159.1	AHCTF1P1	1.20575
IPI00954192.1	CHD1	1.9585
IPI00954445.1	ST13	-0.01225
IPI00954553.1	PQBP1	1.13625
REV_IPI00006254.:	BZRAP1	0.61675
REV_IPI00910487.	SERPINH1	-1.758
REV_IPI00925085.	EIF2B5	-1.25075

AccessionNo	GeneSymbol	hiPSCs/hESCs
IPI00946752.1	CPSF3	0.08625
IPI00946824.1	FXN	-0.0335
IPI00947400.1	PLOD2	0.46525
IPI00947492.1	NDUFB2	0.40925
IPI00953262.1	CACNA2D1	0.57425
IPI00953294.1	ZNF598	0.416
IPI00953297.1	ANXA7	0.263
IPI00953403.1	EXOC3	0.219
IPI00953445.1;IPI00003	ERGIC1	-0.1075
IPI00953520.1	RNPS1	0.15675
IPI00953925.1	AIP	0.26575
IPI00954159.1	AHCTF1P1	0.29175
IPI00954192.1	CHD1	0.2405
IPI00954445.1	ST13	0.42275
IPI00954553.1	PQBP1	0.33275
REV_IPI00006254.:	BZRAP1	-0.1185
REV_IPI00910487.	SERPINH1	0.3945
REV_IPI00925085.	EIF2B5	0.24425

Supplementary Table S5

The significant pathways identified by GSEA for the three integrated profiles from the public dataset.

Table S5 The significant pathways identified by GSEA for the three integrated profiles from the public dataset.

Up-regulated significant enriched pathways

hiPSCs/HFs	FDR (q-val)	hESCs/HFs	FDR (q-val)	hiPSCs/hESCs	FDR (q-val)
KEGG_SPLICEOSOME	0	KEGG_SPLICEOSOME	0	KEGG_ECM_RECEPTOR_INTERACTION	0.0249
KEGG_DNA_REPLICATION	0	KEGG_DNA_REPLICATION	0	KEGG_ARRHYTHMOGENIC_RIGHT_VENTRICULAR_CARDIOMYOPATHY_ARVC	0.1454
KEGG_CELL_CYCLE	0	KEGG_CELL_CYCLE	3.15E-04	KEGG_FOCAL_ADHESION	0.1359
KEGG_RNA_DEGRADATION	0.0169	KEGG_RNA_DEGRADATION	0.0123	KEGG_REGULATION_OF_ACTIN_CYTOSKELETON	0.107
KEGG_OOCYTE_MEIOSIS	0.0748	KEGG_FATTY_ACID_METABOLISM	0.0775	KEGG_EPITHELIAL_CELL_SIGNALING_IN_HELICOBACTER_PYLORI_INFECTION	0.2468
KEGG_PYRIMIDINE_METABOLISM	0.0885	KEGG_ARGININE_AND_PROLINE_METABOLISM	0.105	REACTOME_CELL_SURFACE_INTERACTIONS_AT_THE_VASCULAR_WALL	0.185
KEGG_VALINE_LEUCINE_AND_ISOLEUCINE_DEGRADATION	0.1081	KEGG_VALINE_LEUCINE_AND_ISOLEUCINE_DEGRADATION	0.1192	REACTOME_INTEGRIN_CELL_SURFACE_INTERACTIONS	0.123
REACTOME_PROCESSING_OF_CAPPED_INTRON_CONTAINING_PRE_MRNA	0	KEGG_LYSINE_DEGRADATION	0.1061		
REACTOME_MRNA_PROCESSING	0	KEGG_PYRIMIDINE_METABOLISM	0.174		
REACTOME_MRNA_SPLICING	0	KEGG_OOCYTE_MEIOSIS	0.1987		
REACTOME_TRANSCRIPTION	0	REACTOME_MRNA_PROCESSING	0		
REACTOME_HIV_LIFE_CYCLE	0	REACTOME_PROCESSING_OF_CAPPED_INTRON_CONTAINING_PRE_MRNA	0		
REACTOME_TRANSPORT_OF_MATURE_TRANSCRIPT_TO_CYTOPLASM	0	REACTOME_MRNA_SPLICING	0		
REACTOME_RNA_POL_II_TRANSCRIPTION	0	REACTOME_TRANSCRIPTION	0		
REACTOME_MRNA_SPLICING_MINOR_PATHWAY	0	REACTOME_HIV_LIFE_CYCLE	0		
REACTOME_LATE_PHASE_OF_HIV_LIFE_CYCLE	0	REACTOME_RNA_POL_II_TRANSCRIPTION	0		
REACTOME_METABOLISM_OF_NON_CODING_RNA	0	REACTOME_METABOLISM_OF_NON_CODING_RNA	0		
REACTOME_TRANSPORT_OF_MATURE_MRNA_DERIVED_FROM_AN_INTRONLESS_TRANSCRIP	0	REACTOME_TRANSPORT_OF_MATURE_TRANSCRIPT_TO_CYTOPLASM	0		
REACTOME_CLEAVAGE_OF_GROWING_TRANSCRIPT_IN_THE_TERMINATION_REGION	7.73E-05	REACTOME_LATE_PHASE_OF_HIV_LIFE_CYCLE	0		
REACTOME_RNA_POL_I_RNA_POL_III_AND_MITOCHONDRIAL_TRANSCRIPTION	7.13E-05	REACTOME_CHROMOSOME_MAINTENANCE	0		
REACTOME_INTERACTIONS_OF_VPR_WITH_HOST_CELLULAR_PROTEINS	6.62E-05	REACTOME_MRNA_SPLICING_MINOR_PATHWAY	0		
REACTOME_GLUCOSE_TRANSPORT	2.41E-04	REACTOME_INTERACTIONS_OF_VPR_WITH_HOST_CELLULAR_PROTEINS	0		
REACTOME_MRNA_3_END_PROCESSING	2.26E-04	REACTOME_CLEAVAGE_OF_GROWING_TRANSCRIPT_IN_THE_TERMINATION_REGION_	0		
REACTOME_DNA_REPAIR	2.13E-04	REACTOME_GLUCOSE_TRANSPORT	0		
REACTOME_SLC_MEDIATED_TRANSMEMBRANE_TRANSPORT	2.01E-04	REACTOME_TRANSPORT_OF_MATURE_MRNA_DERIVED_FROM_AN_INTRONLESS_TRANSCRIP	0		
REACTOME_NEP_NS2_INTERACTS_WITH_THE_CELLULAR_EXPORT_MACHINERY	2.50E-04	REACTOME_REGULATION_OF_GLUCOKINASE_BY_GLUCOKINASE_REGULATORY_PROTEIN	0		
REACTOME_REGULATION_OF_GLUCOKINASE_BY_GLUCOKINASE_REGULATORY_PROTEIN	2.38E-04	REACTOME_TRANSPORT_OF_RIBONUCLEOPROTEINS_INTO_THE_HOST_NUCLEUS	0		

Table S5 The significant pathways identified by GSEA for the three integrated profiles from the public dataset.

Up-regulated significant enriched pathways

hiPSCs/HFs	FDR (q-val)	hESCs/HFs	FDR (q-val)	hiPSCs/hESCs	FDR (q-val)
REACTOME_NUCLEOTIDE_EXCISION_REPAIR	2.26E-04	REACTOME_TELOMERE_MAINTENANCE	1.24E-04		
REACTOME_TRANSPORT_OF_RIBONUCLEOPROTEINS_INTO_THE_HOST_NUCLEUS	2.68E-04	REACTOME_NEP_NS2_INTERACTS_WITH_THE_CELLULAR_EXPORT_MACHINERY	1.67E-04		
REACTOME_CHROMOSOME_MAINTENANCE	2.56E-04	REACTOME_SLC_MEDIATED_TRANSMEMBRANE_TRANSPORT	2.54E-04		
REACTOME_RNA_POL_II_PRE_TRANSCRIPTION_EVENTS	8.08E-04	REACTOME_MRNA_3_END_PROCESSING	2.95E-04		
REACTOME_DNA_REPLICATION	0.0021	REACTOME_RNA_POL_I_RNA_POL_III_AND_MITOCHONDRIAL_TRANSCRIPTION	9.06E-04		
REACTOME_CELL_CYCLE_MITOTIC	0.0048	REACTOME_DNA_REPAIR	0.0025		
REACTOME_MITOTIC_PROMETAPHASE	0.0052	REACTOME_MITOTIC_PROMETAPHASE	0.0037		
REACTOME_DEADENYLATION_DEPENDENT_MRNA_DECAY	0.0081	REACTOME_DNA_REPLICATION	0.0053		
REACTOME_CELL_CYCLE	0.0101	REACTOME_RNA_POL_II_PRE_TRANSCRIPTION_EVENTS	0.0062		
REACTOME_MITOTIC_M_M_G1_PHASES	0.0163	REACTOME_CELL_CYCLE	0.0075		
REACTOME_SYNTHESIS_OF_DNA	0.0368	REACTOME_NUCLEOTIDE_EXCISION_REPAIR	0.0113		
REACTOME_MITOCHONDRIAL_PROTEIN_IMPORT	0.0375	REACTOME_MITOCHONDRIAL_PROTEIN_IMPORT	0.0138		
REACTOME_S_PHASE	0.0381	REACTOME_CELL_CYCLE_MITOTIC	0.0144		
REACTOME_MEIOSIS	0.038	REACTOME_MITOTIC_M_M_G1_PHASES	0.2253		
REACTOME_CELL_CYCLE_CHECKPOINTS	0.0614	REACTOME_MEIOSIS	0.0335		
REACTOME_G1_S_TRANSITION	0.1109	REACTOME_DEADENYLATION_DEPENDENT_MRNA_DECAY	0.0348		
REACTOME_MITOTIC_G1_G1_S_PHASES	0.1279	REACTOME_S_PHASE	0.0666		
REACTOME_FACTORS_INVOLVED_IN_MEGAKARYOCYTE_DEVELOPMENT_AND_PLATELET_PRODUCTION	0.2272	REACTOME_SYNTHESIS_OF_DNA	0.0671		
		REACTOME_CELL_CYCLE_CHECKPOINTS	0.102		
		REACTOME_G1_S_TRANSITION	0.1534		
		REACTOME_FACTORS_INVOLVED_IN_MEGAKARYOCYTE_DEVELOPMENT_AND_PLATELET_PRODUCTION	0.2267		
		REACTOME_MITOTIC_G1_G1_S_PHASES	0.2295		
		REACTOME_ANTIVIRAL_MECHANISM_BY_IFN_STIMULATED_GENES	0.2339		
		REACTOME_HIV_INFECTION	0.23		

Table S5 The significant pathways identified by GSEA for the three integrated profiles from the public dataset.

Down-regulated significant enriched pathways

hiPSCs/HFs	FDR (q-val)
KEGG_REGULATION_OF_ACTIN_CYTOSKELETON	0
KEGG_FOCAL_ADHESION	0
KEGG_ECM_RECEPTOR_INTERACTION	0
KEGG_ENDOCYTOSIS	0
KEGG_MAPK_SIGNALING_PATHWAY	0
KEGG_RIBOSOME	0
KEGG_PROTEASOME	0.0021
KEGG_NEUROTROPHIN_SIGNALING_PATHWAY	0.0063
KEGG_FC_GAMMA_R_MEDIATED_PHAGOCYTOSIS	0.0071
KEGG_PATHOGENIC_ESCHERICHIA_COLI_INFECTION	0.0103
KEGG_LEUKOCYTE_TRANSENDOTHELIAL_MIGRATION	0.0177
KEGG_INSULIN_SIGNALING_PATHWAY	0.0169
KEGG_AXON_GUIDANCE	0.0157
KEGG_LYSOSOME	0.0242
KEGG_ERBB_SIGNALING_PATHWAY	0.0472
KEGG_ARRHYTHMOGENIC_RIGHT_VENTRICULAR_CARDIOMYOPATHY_ARVC	0.0602
KEGG_CHEMOKINE_SIGNALING_PATHWAY	0.0729
KEGG_ADHERENS_JUNCTION	0.0936
KEGG_EPITHELIAL_CELL_SIGNALING_IN_HELICOBACTER_PYLORI_INFECTION	0.1182
KEGG_TIGHT_JUNCTION	0.1377
KEGG_PATHWAYS_IN_CANCER	0.1443
KEGG_PROSTATE_CANCER	0.1422
KEGG_PYRUVATE_METABOLISM	0.1998
REACTOME_SIGNALING_BY_PDGF	0
REACTOME_PLATELET_ACTIVATION_SIGNALING_AND_AGGREGATION	4.69E-04
REACTOME_AXON_GUIDANCE	3.13E-04

hESCs/HFs	FDR (q-val)
KEGG_FOCAL_ADHESION	0
KEGG_REGULATION_OF_ACTIN_CYTOSKELETON	0
KEGG_ECM_RECEPTOR_INTERACTION	0
KEGG_ENDOCYTOSIS	0
KEGG_MAPK_SIGNALING_PATHWAY	0
KEGG_RIBOSOME	2.98E-04
KEGG_PROTEASOME	5.21E-04
KEGG_FC_GAMMA_R_MEDIATED_PHAGOCYTOSIS	5.91E-04
KEGG_NEUROTROPHIN_SIGNALING_PATHWAY	0.0011
KEGG_AXON_GUIDANCE	0.003
KEGG_LEUKOCYTE_TRANSENDOTHELIAL_MIGRATION	0.003
KEGG_ARRHYTHMOGENIC_RIGHT_VENTRICULAR_CARDIOMYOPATHY_ARVC	0.0133
KEGG_CHEMOKINE_SIGNALING_PATHWAY	0.0206
KEGG_EPITHELIAL_CELL_SIGNALING_IN_HELICOBACTER_PYLORI_INFECTION	0.021
KEGG_INSULIN_SIGNALING_PATHWAY	0.0214
KEGG_LYSOSOME	0.0265
KEGG_ERBB_SIGNALING_PATHWAY	0.0263
KEGG_PATHOGENIC_ESCHERICHIA_COLI_INFECTION	0.027
KEGG_PROSTATE_CANCER	0.0322
KEGG_ADHERENS_JUNCTION	0.0666
KEGG_WNT_SIGNALING_PATHWAY	0.0926
KEGG_PATHWAYS_IN_CANCER	0.0922
KEGG_TIGHT_JUNCTION	0.0953
REACTOME_AXON_GUIDANCE	0
REACTOME_PLATELET_ACTIVATION_SIGNALING_AND_AGGREGATION	0
REACTOME_SIGNALING_BY_PDGF	0

hiPSCs/hESCs	FDR (q-val)
KEGG_PEROXISOME	0.001
KEGG_ARGININE_AND_PROLINE_METABOLISM	0.058
KEGG_FATTY_ACID_METABOLISM	0.198
REACTOME_GLUCOSE_TRANSPORT	0
REACTOME_PYRUVATE_METABOLISM_AND_CITRIC_ACID_TCA_CYCLE	0.005
REACTOME_REGULATION_OF_GLUCOKINASE_BY_GLUCOKINASE_REGULATORY_PROTEIN	0.029
REACTOME_NEP_NS2_INTERACTS_WITH_THE_CELLULAR_EXPORT_MACHINERY	0.03
REACTOME_TCA_CYCLE_AND_RESPIRATORY_ELECTRON_TRANSPORT	0.042
REACTOME_TRANSPORT_OF_RIBONUCLEOPROTEINS_INTO_THE_HOST_NUCLEUS	0.165
REACTOME_PROCESSING_OF_CAPPED_INTRON_CONTAINING_PRE_MRNA	0.24
REACTOME_RESPIRATORY_ELECTRON_TRANSPORT_ATP_SYNTHESIS_BY_CHEMIOSMOTIC_COUPLING_AND_HEAT_PRODUCTION_BY_UNCOUPLING_PROTEINS	0.224

Table S5 The significant pathways identified by GSEA for the three integrated profiles from the public dataset.

Down-regulated significant enriched pathways

hiPSCs/HFs	FDR (q-val)	hESCs/HFs	FDR (q-val)	hiPSCs/hESCs	FDR (q-val)
REACTOME_TRANSLATION	4.82E-04	REACTOME_DEVELOPMENTAL_BIOLOGY	0		
REACTOME_SIGNALING_BY_GPCR	5.77E-04	REACTOME_TRANSLATION	2.21E-04		
REACTOME_ER_PHAGOSOME_PATHWAY	4.81E-04	REACTOME_3_UTR_MEDIATED_TRANSLATIONAL_REGULATION	1.84E-04		
REACTOME_DEVELOPMENTAL_BIOLOGY	4.12E-04	REACTOME_MEMBRANE_TRAFFICKING	1.58E-04		
REACTOME_ANTIGEN_PROCESSING_CROSS_PRESENTATION	6.10E-04	REACTOME_SIGNALING_BY_GPCR	1.38E-04		
REACTOME_SIGNALING_BY_EGFR_IN_CANCER	6.51E-04	REACTOME_SIGNALING_BY_THE_B_CELL_RECEPTOR_BCR	1.23E-04		
REACTOME_MEMBRANE_TRAFFICKING	7.80E-04	REACTOME_ANTIGEN_PROCESSING_CROSS_PRESENTATION	2.15E-04		
REACTOME_SIGNALING_BY_THE_B_CELL_RECEPTOR_BCR	7.99E-04	REACTOME_SIGNALING_BY_EGFR_IN_CANCER	1.96E-04		
REACTOME_NGF_SIGNALLING_VIA_TRKA_FROM_THE_PLASMA_MEMBRANE	9.68E-04	REACTOME_RESPONSE_TO_ELEVATED_PLATELET_CYTOSOLIC_CA2_	1.79E-04		
REACTOME_DOWNSTREAM_SIGNALING_EVENTS_OF_B_CELL_RECEPTOR_BCR	9.63E-04	REACTOME_CLASS_I_MHC_MEDIATED_ANTIGEN_PROCESSING_PRESENTATION	3.09E-04		
REACTOME_CLASS_I_MHC_MEDIATED_ANTIGEN_PROCESSING_PRESENTATION	0.0011	REACTOME_PEPTIDE_CHAIN_ELONGATION	2.87E-04		
REACTOME_PEPTIDE_CHAIN_ELONGATION	0.001	REACTOME_ER_PHAGOSOME_PATHWAY	3.37E-04		
REACTOME_ACTIVATION_OF_NF_KAPPAB_IN_B_CELLS	0.001	REACTOME_ACTIVATION_OF_NF_KAPPAB_IN_B_CELLS	5.10E-04		
REACTOME_P53_DEPENDENT_G1_DNA_DAMAGE_RESPONSE	0.001	REACTOME_NGF_SIGNALLING_VIA_TRKA_FROM_THE_PLASMA_MEMBRANE	4.80E-04		
REACTOME_3_UTR_MEDIATED_TRANSLATIONAL_REGULATION	0.0011	REACTOME_SRP_DEPENDENT_COTRANSLATIONAL_PROTEIN_TARGETING_TO_MEMBRANE	5.04E-04		
REACTOME_CDK_MEDIATED_PHOSPHORYLATION_AND_REMOVAL_OF_CDC6	0.0011	REACTOME_REGULATION_OF_APOPTOSIS	4.77E-04		
REACTOME_CROSS_PRESENTATION_OF_SOLUBLE_EXOGENOUS_ANTIGENS_ENDOSOMES	0.0011	REACTOME_DOWNSTREAM_SIGNALING_EVENTS_OF_B_CELL_RECEPTOR_BCR	5.02E-04		
REACTOME_SIGNALLING_BY_NGF	0.001	REACTOME_SIGNALING_BY_WNT	5.27E-04		
REACTOME_REGULATION_OF_ORNITHINE_DECARBOXYLASE_ODC	0.001	REACTOME_CROSS_PRESENTATION_OF_SOLUBLE_EXOGENOUS_ANTIGENS_ENDOSOMES	5.03E-04		
REACTOME_VIF_MEDIATED_DEGRADATION_OF_APOBEC3G	9.96E-04	REACTOME_VIF_MEDIATED_DEGRADATION_OF_APOBEC3G	4.81E-04		
REACTOME_P53_INDEPENDENT_G1_S_DNA_DAMAGE_CHECKPOINT	9.96E-04	REACTOME_P53_INDEPENDENT_G1_S_DNA_DAMAGE_CHECKPOINT	4.61E-04		
REACTOME_DOWNSTREAM_SIGNAL_TRANSDUCTION	0.001	REACTOME_SIGNALING_BY_ILS	5.22E-04		
REACTOME_SCF_BETA_TRCP_MEDIATED_DEGRADATION_OF_EMI1	0.001	REACTOME_AUTODEGRADATION_OF_THE_E3_UBIQUITIN_LIGASE_COP1	5.02E-04		
REACTOME_REGULATION_OF_APOPTOSIS	9.96E-04	REACTOME_CDK_MEDIATED_PHOSPHORYLATION_AND_REMOVAL_OF_CDC6	5.23E-04		
REACTOME_AUTODEGRADATION_OF_THE_E3_UBIQUITIN_LIGASE_COP1	9.95E-04	REACTOME_P53_DEPENDENT_G1_DNA_DAMAGE_RESPONSE	5.38E-04		
REACTOME_SIGNALING_BY_WNT	0.0011	REACTOME_ACTIVATION_OF_THE_MRNA_UPON_BINDING_OF_THE_CAP_BINDING_COMPLEX_AND EIFS AND SUBSEQUENT BINDING TO 43S	5.54E-04		

Table S5 The significant pathways identified by GSEA for the three integrated profiles from the public dataset.

Down-regulated significant enriched pathways

hiPSCs/HFs	FDR (q-val)	hESCs/HFs	FDR (q-val)	hiPSCs/hESCs	FDR (q-val)
REACTOME_SRP_DEPENDENT_COTRANSLATIONAL_PROTEIN_TARGETING_TO_MEMBRANE	0.0015	REACTOME_SCF_BETA_TRCP_MEDIATED_DEGRADATION_OF_EMI1	6.71E-04		
REACTOME_INNATE_IMMUNE_SYSTEM	0.0016	REACTOME_REGULATION_OF_ORNITHINE_DECARBOXYLASE_ODC	7.15E-04		
REACTOME_RESPONSE_TO_ELEVATED_PLATELET_CYTOSOLIC_CA2	0.0016	REACTOME_DOWNSTREAM_SIGNAL_TRANSDUCTION	7.23E-04		
REACTOME_DESTABILIZATION_OF_MRNA_BY_AUF1_HNRNP_D0	0.0019	REACTOME_FORMATION_OF_THE_TERNARY_COMPLEX_AND_SUBSEQUENTLY_THE_43S_COMPLEX	7.61E-04		
REACTOME_AUTODEGRADATION_OF_CDH1_BY_CDH1_APC_C	0.0018	REACTOME_TRANSMISSION_ACROSS_CHEMICAL_SYNAPSES	7.38E-04		
REACTOME_SIGNALING_BY_ILS	0.0021	REACTOME_ADAPTIVE_IMMUNE_SYSTEM	7.17E-04		
REACTOME_ADAPTIVE_IMMUNE_SYSTEM	0.0024	REACTOME_AUTODEGRADATION_OF_CDH1_BY_CDH1_APC_C	7.49E-04		
REACTOME_SIGNALING_BY_INSULIN_RECEPTOR	0.0028	REACTOME_NEURONAL_SYSTEM	7.55E-04		
REACTOME_SIGNALING_BY_ERBB2	0.0029	REACTOME_SIGNALLING_BY_NGF	7.35E-04		
REACTOME_GPCR_DOWNSTREAM_SIGNALING	0.003	REACTOME_DESTABILIZATION_OF_MRNA_BY_AUF1_HNRNP_D0	7.92E-04		
REACTOME_NEURONAL_SYSTEM	0.003	REACTOME_SIGNALING_BY_INSULIN_RECEPTOR	9.02E-04		
REACTOME_SIGNALING_BY_FGFR	0.004	REACTOME_GPCR_DOWNSTREAM_SIGNALING	0.0012		
REACTOME_REGULATION_OF_INSULIN_SECRETION	0.0052	REACTOME_SIGNALING_BY_FGFR	0.002		
REACTOME_FORMATION_OF_THE_TERNARY_COMPLEX_AND_SUBSEQUENTLY_THE_43S_COM	0.0055	REACTOME_INNATE_IMMUNE_SYSTEM	0.0027		
REACTOME_ACTIVATION_OF_THE_MRNA_UPON_BINDING_OF_THE_CAP_BINDING_COMPLEX_A	0.0055	REACTOME_SCFSKP2_MEDIATED_DEGRADATION_OF_P27_P21	0.0028		
REACTOME_INTEGRIN_CELL_SURFACE_INTERACTIONS	0.0068	REACTOME_HEMOSTASIS	0.0033		
REACTOME_TRANSMISSION_ACROSS_CHEMICAL_SYNAPSES	0.0067	REACTOME_ANTIGEN_PROCESSING_UBIQUITINATION_PROTEASOME_DEGRADATION	0.0035		
REACTOME_SCFSKP2_MEDIATED_DEGRADATION_OF_P27_P21	0.0066	REACTOME_SEMAPHORIN_INTERACTIONS	0.0043		
REACTOME_INTEGRATION_OF_ENERGY_METABOLISM	0.0101	REACTOME_CYCLIN_E_ASSOCIATED_EVENTS_DURING_G1_S_TRANSITION_	0.0043		
REACTOME_IMMUNE_SYSTEM	0.0101	REACTOME_SIGNALING_BY_ERBB2	0.0047		
REACTOME_L1CAM_INTERACTIONS	0.0112	REACTOME_INTEGRIN_CELL_SURFACE_INTERACTIONS	0.0049		
REACTOME_CYCLIN_E_ASSOCIATED_EVENTS_DURING_G1_S_TRANSITION	0.0125	REACTOME_CELL_SURFACE_INTERACTIONS_AT_THE_VASCULAR_WALL	0.0064		
REACTOME_ANTIGEN_PROCESSING_UBIQUITINATION_PROTEASOME_DEGRADATION	0.013	REACTOME_L1CAM_INTERACTIONS	0.0068		
REACTOME_POST_TRANSLATIONAL_PROTEIN_MODIFICATION	0.0158	REACTOME_METABOLISM_OF_PROTEINS	0.0096		
REACTOME_HEMOSTASIS	0.016	REACTOME_TRANS_GOLGI_NETWORK_VESICLE_BUDDING	0.0097		

Table S5 The significant pathways identified by GSEA for the three integrated profiles from the public dataset.

Down-regulated significant enriched pathways

hiPSCs/HFs	FDR (q-val)	hESCs/HFs	FDR (q-val)	hiPSCs/hESCs	FDR (q-val)
REACTOME_SIGNALING_BY_FGFR_IN_DISEASE	0.0164	REACTOME_APC_C_CDH1_MEDIATED_DEGRADATION_OF_CDC20_AND_OTHER_APC_C_CDH1_TARGETED_PROTEINS_IN_LATE_MITOSIS_EARLY_G1	0.0124		
REACTOME_DIABETES_PATHWAYS	0.0165	REACTOME_DIABETES_PATHWAYS	0.0123		
REACTOME_METABOLISM_OF_PROTEINS	0.0171	REACTOME_IMMUNE_SYSTEM	0.0137		
REACTOME_PREFOLDIN_MEDIATED_TRANSFER_OF_SUBSTRATE_TO_CCT_TRIC	0.0178	REACTOME_UNFOLDED_PROTEIN_RESPONSE	0.0136		
REACTOME_ASPARAGINE_N_LINKED_GLYCOSYLATION	0.0183	REACTOME_ASPARAGINE_N_LINKED_GLYCOSYLATION	0.0187		
REACTOME_PROTEIN_FOLDING	0.0241	REACTOME_APC_C_CDC20_MEDIATED_DEGRADATION_OF_MITOTIC_PROTEINS	0.0203		
REACTOME_TRANS_GOLGI_NETWORK_VESICLE_BUDDING	0.0268	REACTOME_INFLUENZA_VIRAL_RNA_TRANSCRIPTION_AND_REPLICATION	0.0254		
REACTOME_CELL_SURFACE_INTERACTIONS_AT_THE_VASCULAR_WALL	0.0308	REACTOME_MHC_CLASS_II_ANTIGEN_PRESENTATION	0.0269		
REACTOME_UNFOLDED_PROTEIN_RESPONSE	0.0335	REACTOME_CDT1_ASSOCIATION_WITH_THE_CDC6_ORC_ORIGIN_COMPLEX	0.0268		
REACTOME_APC_C_CDH1_MEDIATED_DEGRADATION_OF_CDC20_AND_OTHER_APC_C_CDH1_	0.0345	REACTOME_SIGNALING_BY_FGFR_IN_DISEASE	0.0272		
REACTOME_CDT1_ASSOCIATION_WITH_THE_CDC6_ORC_ORIGIN_COMPLEX	0.0353	REACTOME_POST_TRANSLATIONAL_PROTEIN_MODIFICATION	0.029		
REACTOME_APC_C_CDC20_MEDIATED_DEGRADATION_OF_MITOTIC_PROTEINS	0.0382	REACTOME_GOLGI_ASSOCIATED_VESICLE_BIOGENESIS	0.0286		
REACTOME_SEMAPHORIN_INTERACTIONS	0.0437	REACTOME_CELL_CELL_COMMUNICATION	0.0317		
REACTOME_MHC_CLASS_II_ANTIGEN_PRESENTATION	0.0486	REACTOME_NONSENSE_MEDIATED_DECAY_ENHANCED_BY_THE_EXON_JUNCTION_COMPLEX	0.041		
REACTOME_GOLGI_ASSOCIATED_VESICLE_BIOGENESIS	0.0799	REACTOME_INTEGRATION_OF_ENERGY_METABOLISM	0.0493		
REACTOME_INFLUENZA_VIRAL_RNA_TRANSCRIPTION_AND_REPLICATION	0.1292	REACTOME_REGULATION_OF_INSULIN_SECRETION	0.0538		
REACTOME_CELL_CELL_COMMUNICATION	0.1402	REACTOME_REGULATION_OF_MITOTIC_CELL_CYCLE	0.0951		
REACTOME_GLUCOSE_METABOLISM	0.149	REACTOME_METABOLISM_OF_MRNA	0.1103		
REACTOME_REGULATION_OF_MRNA_STABILITY_BY_PROTEINS_THAT_BIND_AU_RICH_ELEMEN	0.1659	REACTOME_REGULATION_OF_MRNA_STABILITY_BY_PROTEINS_THAT_BIND_AU_RICH_ELEMEN	0.116		
REACTOME_APOPTOSIS	0.1686	REACTOME_APOPTOSIS	0.1218		
REACTOME_NONSENSE_MEDIATED_DECAY_ENHANCED_BY_THE_EXON_JUNCTION_COMPLEX	0.1803	REACTOME_PROTEIN_FOLDING	0.1812		
REACTOME_REGULATION_OF_MITOTIC_CELL_CYCLE	0.1905	REACTOME_PREFOLDIN_MEDIATED_TRANSFER_OF_SUBSTRATE_TO_CCT_TRIC	0.2085		

Supplementary Table S6

The differential proteins contribute to the enrichment of the significant pathways for the public dataset.

Table S6 The differential proteins contribute to the enrichment of the significant pathways for the public dataset

Core enrichment proteins in down-regulated pathways of hiPSCs/HFs (hiPSCs vs HFs)

NAME	REACTOME_GLUCOSE_METABOLISM	REACTOME_UNFOLDED_PROTEIN_RESPONSE	REACTOME_DIABETES_PATHWAYS	REACTOME_PROTEIN_FOLDING	REACTOME_PREFOLDIN_MEDIATED_TRANSFER_OF_SUBSTRATE_TO_CCT_TRIC	REACTOME_ASPARAGINE_N_LINKED_GLYCOSYLATION	REACTOME_POST_TRANSLATIONAL_PROTEIN_MODIFICATION	REACTOME_ACTIVATION_OF_THE_MRNA_UPON_BINDING_OF_THE_CAP_BINDING_COMPLEX_AND_EIFS_AND_SUBSEQUENT_BINDING_TO_43S	REACTOME_FORMATION_OF_THE_TERNARY_COMPLEX_AND_SUBSEQUENTLY_THE_43S_COMPLEX	REACTOME_METABOLISM_OF_PROTEINS	REACTOME_SRP_DEPENDENT_COTRANSLATIONAL_PROTEIN_TARGETING_TO_MEMBRANE	REACTOME_INFLUENZA_VIRAL_RNA_TRANSCRIPTION_AND_REPLICATION	REACTOME_NONSENSE_MEDIATED_DECAY_ENHANCED_BY_THE_EXON_JUNCTION_COMPLEX
ACTB	0	0	0	1	1	0	0	0	0	1	0	0	0
ACTN2	0	0	0	0	0	0	0	0	0	0	0	0	0
ACTN4	0	0	0	0	0	0	0	0	0	0	0	0	0
AP1M1	0	0	0	0	0	0	0	0	0	0	0	0	0
AP2A1	0	0	0	0	0	0	0	0	0	0	0	0	0
AP2A2	0	0	0	0	0	0	0	0	0	0	0	0	0
AP2M1	0	0	0	0	0	0	0	0	0	0	0	0	0
AP3B1	0	0	0	0	0	0	0	0	0	0	0	0	0
AP3S1	0	0	0	0	0	0	0	0	0	0	0	0	0
APPL1	0	0	0	0	0	0	0	0	0	0	0	0	0
ARFGAP1	0	1	1	0	0	0	0	0	0	0	0	0	0
ARHGAP35	0	0	0	0	0	0	0	0	0	0	0	0	0
ARHGDIA	0	0	0	0	0	0	0	0	0	0	0	0	0
ARHGEF2	0	0	0	0	0	0	0	0	0	0	0	0	0
ARSB	0	0	0	0	0	0	1	0	0	1	0	0	0
ATP6V0D1	0	0	1	0	0	0	0	0	0	0	0	0	0
ATP6V1A	0	0	0	0	0	0	0	0	0	0	0	0	0
ATP6V1B2	0	0	0	0	0	0	0	0	0	0	0	0	0
ATP6V1D	0	0	0	0	0	0	0	0	0	0	0	0	0
ATP6V1E1	0	0	0	0	0	0	0	0	0	0	0	0	0
ATP6V1F	0	0	0	0	0	0	0	0	0	0	0	0	0
ATP6V1G1	0	0	0	0	0	0	0	0	0	0	0	0	0
BAD	0	0	0	0	0	0	0	0	0	0	0	0	0
BID	0	0	0	0	0	0	0	0	0	0	0	0	0
CDC42	0	0	0	0	0	0	0	0	0	0	0	0	0
CDK4	0	0	0	0	0	0	0	0	0	0	0	0	0

NAME	REACTOME_PEPTIDE_CHAIN_ELONGATION	REACTOME_3_UTR_MEDIATED_TRANSLATIONAL_REGULATION	REACTOME_TRANSLATION	REACTOME_CELL_CELL_COMMUNICATION	REACTOME_GOLGI_ASSOCIATED_VESICLE_BIOGENESIS	REACTOME_TRANS_GOLGI_NETWORK_VESICLE_BUDDING	REACTOME_MEMBRANE_TRAFFICKING	REACTOME_TRANSMISSION_ACROSS_CHEMICAL_SYNAPSES	REACTOME_NEURONAL_SYSTEM	REACTOME_MHC_CLASS_II_ANTIGEN_PRESENTATION	REACTOME_INNATE_IMMUNE_SYSTEM	REACTOME_IMMUNE_SYSTEM	REACTOME_ADAPTIVE_IMMUNE_SYSTEM
ACTB	0	0	0	0	0	0	0	0	0	0	0	0	0
ACTN2	0	0	0	1	0	0	0	1	1	0	0	0	0
ACTN4	0	0	0	1	0	0	0	0	0	0	0	0	0
AP1M1	0	0	0	0	1	1	1	0	0	1	0	1	1
AP2A1	0	0	0	0	0	0	0	1	1	1	0	1	1
AP2A2	0	0	0	0	0	0	0	0	1	0	0	1	1
AP2M1	0	0	0	0	0	0	1	1	1	1	0	1	1
AP3B1	0	0	0	0	1	1	1	0	0	0	0	0	0
AP3S1	0	0	0	0	1	1	1	0	0	0	0	0	0
APPL1	0	0	0	0	0	0	0	0	0	0	0	0	0
ARFGAP1	0	0	0	0	0	0	1	0	0	0	0	0	0
ARHGAP35	0	0	0	0	0	0	0	0	0	0	0	0	0
ARHGDIA	0	0	0	0	0	0	0	0	0	0	0	0	0
ARHGEF2	0	0	0	0	0	0	0	0	0	0	0	0	0
ARSB	0	0	0	0	0	0	0	0	0	0	0	0	0
ATP6V0D1	0	0	0	0	0	0	0	0	0	0	0	0	0
ATP6V1A	0	0	0	0	0	0	0	0	0	0	0	0	0
ATP6V1B2	0	0	0	0	0	0	0	0	0	0	0	0	0
ATP6V1D	0	0	0	0	0	0	0	0	0	0	0	0	0
ATP6V1E1	0	0	0	0	0	0	0	0	0	0	0	0	0
ATP6V1F	0	0	0	0	0	0	0	0	0	0	0	0	0
ATP6V1G1	0	0	0	0	0	0	0	0	0	0	0	0	0
BAD	0	0	0	0	0	0	0	0	0	0	0	1	1
BID	0	0	0	0	0	0	0	0	0	0	0	0	0
CDC42	0	0	0	0	0	0	0	0	0	0	0	1	1
CDK4	0	0	0	0	0	0	0	0	0	0	0	0	0

NAME	REACTOME_CLASS_I_MHC_MEDIATED_ANTIGEN_PROCESSING_PRESENTATION	REACTOME_APOPTOSIS	REACTOME_ANTIGEN_PROCESSING_UBIQUITINATION_PROTEASOME_DEGRADATION	REACTOME_ING_BY_WNT	REACTOME_REGULATION_OF_MRNA_STABILITY_BY_PROTEINS_THAT_BIND_AU_RICH_ELEMENTS	REACTOME_DESTABILIZATION_OF_MRNA_BY_AUF1_HNRNP_D0	REACTOME_REGULATION_OF_APOPTOSIS	REACTOME_REGULATION_OF_ORNITHINE_DECARBOXYLASE_ODC	REACTOME_CROSS_PRESENTATION_OF_SOLUBLE_EXOGENOUS_ANTIGENS_ENDOSOMES	REACTOME_VIF_MEDIATED_DEGRADATION_OF_APOBEC3G	REACTOME_REGULATION_OF_MITOTIC_CELL_CYCLE	REACTOME_APC_C_CDC20_MEDIATED_DEGRADATION_OF_MITOTIC_PROTEINS	REACTOME_CDT1_ASSOCIATION_WITH_THE_CDC6_ORC_ORIGIN_COMPLEX
ACTB	0	0	0	0	0	0	0	0	0	0	0	0	0
ACTN2	0	0	0	0	0	0	0	0	0	0	0	0	0
ACTN4	0	0	0	0	0	0	0	0	0	0	0	0	0
AP1M1	0	0	0	0	0	0	0	0	0	0	0	0	0
AP2A1	0	0	0	0	0	0	0	0	0	0	0	0	0
AP2A2	0	0	0	0	0	0	0	0	0	0	0	0	0
AP2M1	0	0	0	0	0	0	0	0	0	0	0	0	0
AP3B1	0	0	0	0	0	0	0	0	0	0	0	0	0
AP3S1	0	0	0	0	0	0	0	0	0	0	0	0	0
APPL1	0	1	0	0	0	0	1	0	0	0	0	0	0
ARFGAP1	0	0	0	0	0	0	0	0	0	0	0	0	0
ARHGAP35	0	0	0	0	0	0	0	0	0	0	0	0	0
ARHGDIA	0	0	0	0	0	0	0	0	0	0	0	0	0
ARHGEF2	0	0	0	0	0	0	0	0	0	0	0	0	0
ARSB	0	0	0	0	0	0	0	0	0	0	0	0	0
ATP6V0D1	0	0	0	0	0	0	0	0	0	0	0	0	0
ATP6V1A	0	0	0	0	0	0	0	0	0	0	0	0	0
ATP6V1B2	0	0	0	0	0	0	0	0	0	0	0	0	0
ATP6V1D	0	0	0	0	0	0	0	0	0	0	0	0	0
ATP6V1E1	0	0	0	0	0	0	0	0	0	0	0	0	0
ATP6V1F	0	0	0	0	0	0	0	0	0	0	0	0	0
ATP6V1G1	0	0	0	0	0	0	0	0	0	0	0	0	0
BAD	0	1	0	0	0	0	0	0	0	0	0	0	0
BID	0	1	0	0	0	0	0	0	0	0	0	0	0
CDC42	0	0	0	0	0	0	0	0	0	0	0	0	0
CDK4	0	0	0	0	0	0	0	0	0	0	0	0	0

NAME	REACTOME_APC_C_CDH1_MEDIATED_DEGRADATION_OF_CDC20_AND_OTHER_APC_C_CDH1_TARGETED_PROTEINS_IN_LATE_MITOSIS_EARLY_G1	REACTOME_CYCLIN_E_ASSOCIATED_EVENTS_DURING_G1_S_TRANSITION_	REACTOME_SCFSKP2_MEDIATED_DEGRADATION_OF_P27_P21	REACTOME_AUTODEGRADATION_OF_CDH1_BY_CDH1_APC_C	REACTOME_AUTODEGRADATION_OF_THE_E3_UBIQUITIN_LIGASE_COP1	REACTOME_SCF_BETA_TRCP_MEDIATED_DEGRADATION_OF_EMI1	REACTOME_P53_INDEPENDENT_G1_S_DNA_DAMAGE_CHECKPOINT	REACTOME_CDK_MEDIATED_PHOSPHORYLATION_AND_REMOVAL_OF_CDC6	REACTOME_P53_DEPENDENT_G1_DNA_DAMAGE_RESPONSE	REACTOME_ACTIVATION_OF_NF_KAPPAB_IN_B_CELLS	REACTOME_DOWNSTREAM_ING_EVENTS_OF_B_CELL_RECEPTOR_BCR	REACTOME_ING_BY_THE_B_CELL_RECEPTOR_BCR	REACTOME_ANTIGEN_PROCESSING_CROSS_PRESENTATION
ACTB	0	0	0	0	0	0	0	0	0	0	0	0	0
ACTN2	0	0	0	0	0	0	0	0	0	0	0	0	0
ACTN4	0	0	0	0	0	0	0	0	0	0	0	0	0
AP1M1	0	0	0	0	0	0	0	0	0	0	0	0	0
AP2A1	0	0	0	0	0	0	0	0	0	0	0	0	0
AP2A2	0	0	0	0	0	0	0	0	0	0	0	0	0
AP2M1	0	0	0	0	0	0	0	0	0	0	0	0	0
AP3B1	0	0	0	0	0	0	0	0	0	0	0	0	0
AP3S1	0	0	0	0	0	0	0	0	0	0	0	0	0
APPL1	0	0	0	0	0	0	0	0	0	0	0	0	0
ARFGAP1	0	0	0	0	0	0	0	0	0	0	0	0	0
ARHGAP35	0	0	0	0	0	0	0	0	0	0	0	0	0
ARHGDIA	0	0	0	0	0	0	0	0	0	0	0	0	0
ARHGEF2	0	0	0	0	0	0	0	0	0	0	0	0	0
ARSB	0	0	0	0	0	0	0	0	0	0	0	0	0
ATP6V0D1	0	0	0	0	0	0	0	0	0	0	0	0	0
ATP6V1A	0	0	0	0	0	0	0	0	0	0	0	0	0
ATP6V1B2	0	0	0	0	0	0	0	0	0	0	0	0	0
ATP6V1D	0	0	0	0	0	0	0	0	0	0	0	0	0
ATP6V1E1	0	0	0	0	0	0	0	0	0	0	0	0	0
ATP6V1F	0	0	0	0	0	0	0	0	0	0	0	0	0
ATP6V1G1	0	0	0	0	0	0	0	0	0	0	0	0	0
BAD	0	0	0	0	0	0	0	0	0	0	1	1	0
BID	0	0	0	0	0	0	0	0	0	0	0	0	0
CDC42	0	0	0	0	0	0	0	0	0	0	0	0	0
CDK4	0	0	0	0	0	0	0	0	0	0	0	0	0

NAME	REACTOME_ER_PHAGOSOME_PATHWAY	REACTOME_ING_BY_INSULIN_RECEPTOR	REACTOME_CELL_SURFACE_INTERACTIONS_AT_THE_VASCULAR_WALL	REACTOME_INTEGRIN_CELL_SURFACE_INTERACTIONS	REACTOME_RESPONSE_TO_ELEVATED_PLATELET_CYTOSOLIC_CA2_	REACTOME_HEMOSTASIS	REACTOME_PLATELET_ACTIVATION_ING_AND_AGGREGATION	REACTOME_SEMAPHORIN_INTERACTIONS	REACTOME_L1CAM_INTERACTIONS	REACTOME_DEVELOPMENTAL_BIOLOGY	REACTOME_AXON_GUIDANCE	REACTOME_INTEGRATION_OF_ENERGY_METABOLISM	REACTOME_REGULATION_OF_INSULIN_SECRETION
ACTB	0	0	0	0	0	0	0	0	0	0	0	0	0
ACTN2	0	0	0	0	1	1	1	0	0	0	0	0	0
ACTN4	0	0	0	0	1	1	1	0	0	0	0	0	0
AP1M1	0	0	0	0	0	0	0	0	0	0	0	0	0
AP2A1	0	0	0	0	0	0	0	0	1	1	1	0	0
AP2A2	0	0	0	0	0	0	0	0	1	0	0	0	0
AP2M1	0	0	0	0	0	0	0	0	1	1	1	0	0
AP3B1	0	0	0	0	0	0	0	0	0	0	0	0	0
AP3S1	0	0	0	0	0	0	0	0	0	0	0	0	0
APPL1	0	0	0	0	0	0	0	0	0	0	0	0	0
ARFGAP1	0	0	0	0	0	0	0	0	0	0	0	0	0
ARHGAP35	0	0	0	0	0	0	0	1	0	1	0	0	0
ARHGDIA	0	0	0	0	0	0	0	0	0	0	0	0	0
ARHGEF2	0	0	0	0	0	0	0	0	0	0	0	0	0
ARSB	0	0	0	0	0	0	0	0	0	0	0	0	0
ATP6V0D1	0	1	0	0	0	0	0	0	0	0	0	0	0
ATP6V1A	0	1	0	0	0	0	0	0	0	0	0	0	0
ATP6V1B2	0	1	0	0	0	0	0	0	0	0	0	0	0
ATP6V1D	0	1	0	0	0	0	0	0	0	0	0	0	0
ATP6V1E1	0	1	0	0	0	0	0	0	0	0	0	0	0
ATP6V1F	0	1	0	0	0	0	0	0	0	0	0	0	0
ATP6V1G1	0	1	0	0	0	0	0	0	0	0	0	0	0
BAD	0	0	0	0	0	0	0	0	0	0	0	0	0
BID	0	0	0	0	0	0	0	0	0	0	0	0	0
CDC42	0	0	0	0	0	1	1	1	0	1	1	0	0
CDK4	0	0	0	0	0	0	0	0	0	1	0	0	0

NAME	REACTOME_ING_BY_ILS	REACTOME_GPCR_DOWNSTREAM_ING	REACTOME_ING_BY_GPCR	REACTOME_LING_BY_NGF	REACTOME_NGF_LING_VIA_TRKA_FROM_THE_PLASMA_MEMBRANE	REACTOME_ING_BY_EGFR_IN_CANCER	REACTOME_ING_BY_FGFR_IN_DISEASE	REACTOME_ING_BY_FGFR	REACTOME_ING_BY_ERBB2	REACTOME_DOWNSTREAM__TRANSDUCTION	REACTOME_ING_BY_PDGF	KEGG_RIBOSOME	KEGG_PROTEASOME
ACTB	0	0	0	0	0	0	0	0	0	0	0	0	0
ACTN2	0	0	0	0	0	0	0	0	0	0	0	0	0
ACTN4	0	0	0	0	0	0	0	0	0	0	0	0	0
AP1M1	0	0	0	0	0	0	0	0	0	0	0	0	0
AP2A1	0	0	0	1	1	1	0	0	0	0	0	0	0
AP2A2	0	0	0	0	1	0	0	0	0	0	0	0	0
AP2M1	0	0	0	1	1	1	0	0	0	0	0	0	0
AP3B1	0	0	0	0	0	0	0	0	0	0	0	0	0
AP3S1	0	0	0	0	0	0	0	0	0	0	0	0	0
APPL1	0	0	0	0	0	0	0	0	0	0	0	0	0
ARFGAP1	0	0	0	0	0	0	0	0	0	0	0	0	0
ARHGAP35	0	0	0	0	0	0	0	0	0	0	0	0	0
ARHGDIA	0	0	0	1	0	0	0	0	0	0	0	0	0
ARHGEF2	0	1	1	1	0	0	0	0	0	0	0	0	0
ARSB	0	0	0	0	0	0	0	0	0	0	0	0	0
ATP6V0D1	0	0	0	0	0	0	0	0	0	0	0	0	0
ATP6V1A	0	0	0	0	0	0	0	0	0	0	0	0	0
ATP6V1B2	0	0	0	0	0	0	0	0	0	0	0	0	0
ATP6V1D	0	0	0	0	0	0	0	0	0	0	0	0	0
ATP6V1E1	0	0	0	0	0	0	0	0	0	0	0	0	0
ATP6V1F	0	0	0	0	0	0	0	0	0	0	0	0	0
ATP6V1G1	0	0	0	0	0	0	0	0	0	0	0	0	0
BAD	0	0	0	1	1	1	1	1	1	1	1	0	0
BID	0	0	0	0	0	0	0	0	0	0	0	0	0
CDC42	0	1	1	0	0	1	0	0	0	0	0	0	0
CDK4	0	0	0	0	0	0	0	0	0	0	0	0	0

NAME	KEGG_PYRUVATE_METABOLISM	KEGG_LYSOSOME	KEGG_ENDOCYTOSIS	KEGG_EPITHELIAL_CELL_ING_IN_HELICOBACTER_PYLORI_INFECTION	KEGG_AXON_GUIDANCE	KEGG_NEUROTROPHIN_ING_PATHWAY	KEGG_CHEMOKINE_ING_PATHWAY	KEGG_INSULIN_ING_PATHWAY	KEGG_PROSTATE_CANCER	KEGG_PATHWAYS_IN_CANCER	KEGG_ERBB_ING_PATHWAY	KEGG_MAPK_ING_PATHWAY	KEGG_PATHOGENIC_ESCHERICHIA_COLI_INFECTION
ACTB	0	0	0	0	0	0	0	0	0	0	0	0	1
ACTN2	0	0	0	0	0	0	0	0	0	0	0	0	0
ACTN4	0	0	0	0	0	0	0	0	0	0	0	0	0
AP1M1	0	1	0	0	0	0	0	0	0	0	0	0	0
AP2A1	0	0	1	0	0	0	0	0	0	0	0	0	0
AP2A2	0	0	1	0	0	0	0	0	0	0	0	0	0
AP2M1	0	0	1	0	0	0	0	0	0	0	0	0	0
AP3B1	0	1	0	0	0	0	0	0	0	0	0	0	0
AP3S1	0	1	0	0	0	0	0	0	0	0	0	0	0
APPL1	0	0	0	0	0	0	0	0	0	1	0	0	0
ARFGAP1	0	0	1	0	0	0	0	0	0	0	0	0	0
ARHGAP35	0	0	0	0	0	0	0	0	0	0	0	0	0
ARHGDIA	0	0	0	0	0	1	0	0	0	0	0	0	0
ARHGEF2	0	0	0	0	0	0	0	0	0	0	0	0	1
ARSB	0	1	0	0	0	0	0	0	0	0	0	0	0
ATP6V0D1	0	0	0	1	0	0	0	0	0	0	0	0	0
ATP6V1A	0	0	0	1	0	0	0	0	0	0	0	0	0
ATP6V1B2	0	0	0	1	0	0	0	0	0	0	0	0	0
ATP6V1D	0	0	0	1	0	0	0	0	0	0	0	0	0
ATP6V1E1	0	0	0	1	0	0	0	0	0	0	0	0	0
ATP6V1F	0	0	0	1	0	0	0	0	0	0	0	0	0
ATP6V1G1	0	0	0	1	0	0	0	0	0	0	0	0	0
BAD	0	0	0	0	0	1	0	1	1	1	1	0	0
BID	0	0	0	0	0	0	0	0	0	1	0	0	0
CDC42	0	0	1	1	1	1	1	0	0	1	0	1	1
CDK4	0	0	0	0	0	0	0	0	0	1	0	0	0

NAME	KEGG_TIGHT_JUNCTION	KEGG_ADHERENS_JUNCTION	KEGG_LEUKOCYTE_TRANSENDOTHELIAL_MIGRATION	KEGG_ARRHYTHMOGENIC_RIGHT_VENTRICULAR_CARDIOMYOPATHY_ARVC	KEGG_ECM_RECEPTOR_INTERACTION	KEGG_FOCAL_ADHESION	KEGG_FC_GAMMA_R_MEDIATED_PHAGOCYTOSIS	KEGG_REGULATION_OF_ACTIN_CYTOSKELETON
ACTB	1	1	1	0	0	0	0	1
ACTN2	1	1	1	1	0	1	0	1
ACTN4	1	1	1	1	0	1	0	1
AP1M1	0	0	0	0	0	0	0	0
AP2A1	0	0	0	0	0	0	0	0
AP2A2	0	0	0	0	0	0	0	0
AP2M1	0	0	0	0	0	0	0	0
AP3B1	0	0	0	0	0	0	0	0
AP3S1	0	0	0	0	0	0	0	0
APPL1	0	0	0	0	0	0	0	0
ARFGAP1	0	0	0	0	0	0	0	0
ARHGAP35	0	0	0	0	0	0	0	1
ARHGDIA	0	0	0	0	0	0	0	0
ARHGEF2	0	0	0	0	0	0	0	0
ARSB	0	0	0	0	0	0	0	0
ATP6V0D1	0	0	0	0	0	0	0	0
ATP6V1A	0	0	0	0	0	0	0	0
ATP6V1B2	0	0	0	0	0	0	0	0
ATP6V1D	0	0	0	0	0	0	0	0
ATP6V1E1	0	0	0	0	0	0	0	0
ATP6V1F	0	0	0	0	0	0	0	0
ATP6V1G1	0	0	0	0	0	0	0	0
BAD	0	0	0	0	0	1	0	0
BID	0	0	0	0	0	0	0	0
CDC42	1	1	1	0	0	1	1	1
CDK4	1	0	0	0	0	0	0	0

Table S6 The differential proteins contribute to the enrichment of the significant pathways for the public dataset

Core enrichment proteins in down-regulated pathways of hiPSCs/HFs (hiPSCs vs HFs)

NAME	REACTOME_GLUCOSE_METABOLISM	REACTOME_UNFOLDED_PROTEIN_RESPONSE	REACTOME_DIABETES_PATHWAYS	REACTOME_PROTEIN_FOLDING	REACTOME_PREFOLDIN_MEDIATED_TRANSFER_OF_SUBSTRATE_TO_CCT_TRIC	REACTOME_ASPARAGINE_N_LINKED_GLYCOSYLATION	REACTOME_POST_TRANSLATIONAL_PROTEIN_MODIFICATION	REACTOME_ACTIVATION_OF_THE_MRNA_UPON_BINDING_OF_THE_CAP_BINDING_COMPLEX_AND_EIFS_AND_SUBSEQUENT_BINDING_TO_43S	REACTOME_FORMATION_OF_THE_TERNARY_COMPLEX_AND_SUBSEQUENTLY_THE_43S_COMPLEX	REACTOME_METABOLISM_OF_PROTEINS	REACTOME_SRP_DEPENDENT_COTRANSLATIONAL_PROTEIN_TARGETING_TO_MEMBRANE	REACTOME_INFLUENZA_VIRAL_RNA_TRANSCRIPTION_AND_REPLICATION	REACTOME_NONSENSE_MEDIATED_DECAY_ENHANCED_BY_THE_EXON_JUNCTION_COMPLEX
CFL1	0	0	0	0	0	0	0	0	0	0	0	0	0
CHMP2A	0	0	0	0	0	0	0	0	0	0	0	0	0
CHMP4B	0	0	0	0	0	0	0	0	0	0	0	0	0
CHMP5	0	0	0	0	0	0	0	0	0	0	0	0	0
CHUK	0	0	0	0	0	0	0	0	0	0	0	0	0
CLTA	0	0	0	0	0	0	0	0	0	0	0	0	0
CLTB	0	0	0	0	0	0	0	0	0	0	0	0	0
CLTC	0	0	0	0	0	0	0	0	0	0	0	0	0
COL1A1	0	0	0	0	0	0	0	0	0	0	0	0	0
COL1A2	0	0	0	0	0	0	0	0	0	0	0	0	0
COL5A1	0	0	0	0	0	0	0	0	0	0	0	0	0
COL6A1	0	0	0	0	0	0	0	0	0	0	0	0	0
COL6A2	0	0	0	0	0	0	0	0	0	0	0	0	0
CRK	0	0	0	0	0	0	0	0	0	0	0	0	0
CTSA	0	0	0	0	0	0	0	0	0	0	0	0	0
CTSB	0	0	0	0	0	0	0	0	0	0	0	0	0
CTSL1	0	0	0	0	0	0	0	0	0	0	0	0	0
CTSZ	0	0	0	0	0	0	0	0	0	0	0	0	0
EEA1	0	0	0	0	0	0	0	0	0	0	0	0	0
EGFR	0	0	0	0	0	0	0	0	0	0	0	0	0
EHD1	0	0	0	0	0	0	0	0	0	0	0	0	0
EHD2	0	0	0	0	0	0	0	0	0	0	0	0	0
EXOC3	0	0	1	0	0	0	0	0	0	0	0	0	0
GNG12	0	0	0	0	0	0	0	0	0	0	0	0	0
GNS	0	0	0	0	0	0	0	0	0	0	0	0	0

NAME	REACTOME_PEPTIDE_CHAIN_ELONGATION	REACTOME_3_UTR_MEDIATED_TRANSLATIONAL_REGULATION	REACTOME_TRANSLATION	REACTOME_CELL_CELL_COMMUNICATION	REACTOME_GOLGI_ASSOCIATED_VESICLE_BIOGENESIS	REACTOME_TRANS_GOLGI_NETWORK_VESICLE_BUDDING	REACTOME_MEMBRANE_TRAFFICKING	REACTOME_TRANSMISSION_ACROSS_CHEMICAL_SYNAPSES	REACTOME_NEURONAL_SYSTEM	REACTOME_MHC_CLASS_II_ANTIGEN_PRESENTATION	REACTOME_INNATE_IMMUNE_SYSTEM	REACTOME_IMMUNE_SYSTEM	REACTOME_ADAPTIVE_IMMUNE_SYSTEM
CFL1	0	0	0	0	0	0	0	0	0	0	0	0	0
CHMP2A	0	0	0	0	0	0	1	0	0	0	0	0	0
CHMP4B	0	0	0	0	0	0	1	0	0	0	0	0	0
CHMP5	0	0	0	0	0	0	1	0	0	0	0	0	0
CHUK	0	0	0	0	0	0	0	0	0	0	1	1	1
CLTA	0	0	0	0	1	1	1	0	0	0	0	1	1
CLTB	0	0	0	0	0	0	1	0	0	0	0	0	0
CLTC	0	0	0	0	1	1	1	0	0	1	0	1	1
COL1A1	0	0	0	0	0	0	0	0	0	0	0	0	0
COL1A2	0	0	0	0	0	0	0	0	0	0	0	0	0
COL5A1	0	0	0	0	0	0	0	0	0	0	0	0	0
COL6A1	0	0	0	0	0	0	0	0	0	0	0	0	0
COL6A2	0	0	0	0	0	0	0	0	0	0	0	0	0
CRK	0	0	0	0	0	0	0	0	0	0	0	1	0
CTSA	0	0	0	0	0	0	0	0	0	1	0	1	1
CTSB	0	0	0	0	0	0	0	0	0	1	1	1	1
CTSL1	0	0	0	0	0	0	0	0	0	1	1	1	1
CTSZ	0	0	0	0	0	1	1	0	0	0	0	0	0
EEA1	0	0	0	0	0	0	0	0	0	0	1	1	0
EGFR	0	0	0	0	0	0	0	0	0	0	0	0	0
EHD1	0	0	0	0	0	0	0	0	0	0	0	0	0
EHD2	0	0	0	0	0	0	0	0	0	0	0	0	0
EXOC3	0	0	0	0	0	0	0	0	0	0	0	0	0
GNG12	0	0	0	0	0	0	0	1	1	0	0	0	0
GNS	0	0	0	0	0	1	1	0	0	0	0	0	0

NAME	REACTOME_CLASS_I_MHC_MEDIATED_ANTIGEN_PROCESSING_PRESENTATION	REACTOME_APOPTOSIS	REACTOME_ANTIGEN_PROCESSING_UBIQUITINATION_PROTEASOME_DEGRADATION	REACTOME_ING_BY_WNT	REACTOME_REGULATION_OF_MRNA_STABILITY_BY_PROTEINS_THAT_BIND_AU_RICH_ELEMENTS	REACTOME_DESTABILIZATION_OF_MRNA_BY_AUF1_HNRNP_D0	REACTOME_REGULATION_OF_APOPTOSIS	REACTOME_REGULATION_OF_ORNITHINE_DECARBOXYLASE_ODC	REACTOME_CROSS_PRESENTATION_OF_SOLUBLE_EXOGENOUS_ANTIGENS_ENDOSOMES	REACTOME_VIF_MEDIATED_DEGRADATION_OF_APOBEC3G	REACTOME_REGULATION_OF_MITOTIC_CELL_CYCLE	REACTOME_APC_C_CDC20_MEDIATED_DEGRADATION_OF_MITOTIC_PROTEINS	REACTOME_CDT1_ASSOCIATION_WITH_THE_CDC6_ORC_ORIGIN_COMPLEX
CFL1	0	0	0	0	0	0	0	0	0	0	0	0	0
CHMP2A	0	0	0	0	0	0	0	0	0	0	0	0	0
CHMP4B	0	0	0	0	0	0	0	0	0	0	0	0	0
CHMP5	0	0	0	0	0	0	0	0	0	0	0	0	0
CHUK	0	0	0	0	0	0	0	0	0	0	0	0	0
CLTA	0	0	0	0	0	0	0	0	0	0	0	0	0
CLTB	0	0	0	0	0	0	0	0	0	0	0	0	0
CLTC	0	0	0	0	0	0	0	0	0	0	0	0	0
COL1A1	0	0	0	0	0	0	0	0	0	0	0	0	0
COL1A2	0	0	0	0	0	0	0	0	0	0	0	0	0
COL5A1	0	0	0	0	0	0	0	0	0	0	0	0	0
COL6A1	0	0	0	0	0	0	0	0	0	0	0	0	0
COL6A2	0	0	0	0	0	0	0	0	0	0	0	0	0
CRK	0	0	0	0	0	0	0	0	0	0	0	0	0
CTSA	0	0	0	0	0	0	0	0	0	0	0	0	0
CTSB	0	0	0	0	0	0	0	0	0	0	0	0	0
CTSL1	0	0	0	0	0	0	0	0	0	0	0	0	0
CTSZ	0	0	0	0	0	0	0	0	0	0	0	0	0
EEA1	0	0	0	0	0	0	0	0	0	0	0	0	0
EGFR	0	0	0	0	0	0	0	0	0	0	0	0	0
EHD1	0	0	0	0	0	0	0	0	0	0	0	0	0
EHD2	0	0	0	0	0	0	0	0	0	0	0	0	0
EXOC3	0	0	0	0	0	0	0	0	0	0	0	0	0
GNG12	0	0	0	0	0	0	0	0	0	0	0	0	0
GNS	0	0	0	0	0	0	0	0	0	0	0	0	0

NAME	REACTOME_APC_C_CDH1_MEDIATED_DEGRADATION_OF_CDC20_AND_OTHER_APC_C_CDH1_TARGETED_PROTEINS_IN_LATE_MITOSIS_EARLY_G1	REACTOME_CYCLIN_E_ASSOCIATED_EVENTS_DURING_G1_S_TRANSITION_	REACTOME_SCFSKP2_MEDIATED_DEGRADATION_OF_P27_P21	REACTOME_AUTODEGRADATION_OF_CDH1_BY_CDH1_APC_C	REACTOME_AUTODEGRADATION_OF_THE_E3_UBIQUITIN_LIGASE_COP1	REACTOME_SCF_BETA_TRCP_MEDIATED_DEGRADATION_OF_EMI1	REACTOME_P53_INDEPENDENT_G1_S_DNA_DAMAGE_CHECKPOINT	REACTOME_CDK_MEDIATED_PHOSPHORYLATION_AND_REMOVAL_OF_CDC6	REACTOME_P53_DEPENDENT_G1_DNA_DAMAGE_RESPONSE	REACTOME_ACTIVATION_OF_NF_KAPPAB_IN_B_CELLS	REACTOME_DOWNSTREAM_ING_EVENTS_OF_B_CELL_RECEPTOR_BCR	REACTOME_ING_BY_THE_B_CELL_RECEPTOR_BCR	REACTOME_ANTIGEN_PROCESSING_CROSS_PRESENTATION
CFL1	0	0	0	0	0	0	0	0	0	0	0	0	0
CHMP2A	0	0	0	0	0	0	0	0	0	0	0	0	0
CHMP4B	0	0	0	0	0	0	0	0	0	0	0	0	0
CHMP5	0	0	0	0	0	0	0	0	0	0	0	0	0
CHUK	0	0	0	0	0	0	0	0	0	1	1	1	0
CLTA	0	0	0	0	0	0	0	0	0	0	0	0	0
CLTB	0	0	0	0	0	0	0	0	0	0	0	0	0
CLTC	0	0	0	0	0	0	0	0	0	0	0	0	0
COL1A1	0	0	0	0	0	0	0	0	0	0	0	0	0
COL1A2	0	0	0	0	0	0	0	0	0	0	0	0	0
COL5A1	0	0	0	0	0	0	0	0	0	0	0	0	0
COL6A1	0	0	0	0	0	0	0	0	0	0	0	0	0
COL6A2	0	0	0	0	0	0	0	0	0	0	0	0	0
CRK	0	0	0	0	0	0	0	0	0	0	0	0	0
CTSA	0	0	0	0	0	0	0	0	0	0	0	0	0
CTSB	0	0	0	0	0	0	0	0	0	0	0	0	0
CTSL1	0	0	0	0	0	0	0	0	0	0	0	0	0
CTSZ	0	0	0	0	0	0	0	0	0	0	0	0	0
EEA1	0	0	0	0	0	0	0	0	0	0	0	0	0
EGFR	0	0	0	0	0	0	0	0	0	0	0	0	0
EHD1	0	0	0	0	0	0	0	0	0	0	0	0	0
EHD2	0	0	0	0	0	0	0	0	0	0	0	0	0
EXOC3	0	0	0	0	0	0	0	0	0	0	0	0	0
GNG12	0	0	0	0	0	0	0	0	0	0	0	0	0
GNS	0	0	0	0	0	0	0	0	0	0	0	0	0

NAME	REACTOME_ER_PHAGOSOME_PATHWAY	REACTOME_ING_BY_INSULIN_RECEPTOR	REACTOME_CELL_SURFACE_INTERACTIONS_AT_THE_VASCULAR_WALL	REACTOME_INTEGRIN_CELL_SURFACE_INTERACTIONS	REACTOME_RESPONSE_TO_ELEVATED_PLATELET_CYTOSOLIC_CA2_	REACTOME_HEMOSTASIS	REACTOME_PLATELET_ACTIVATION_ING_AND_AGGREGATION	REACTOME_SEMAPHORIN_INTERACTIONS	REACTOME_L1CAM_INTERACTIONS	REACTOME_DEVELOPMENTAL_BIOLOGY	REACTOME_AXON_GUIDANCE	REACTOME_INTEGRATION_OF_ENERGY_METABOLISM	REACTOME_REGULATION_OF_INSULIN_SECRETION
CFL1	0	0	0	0	0	1	1	1	0	1	1	0	0
CHMP2A	0	0	0	0	0	0	0	0	0	0	0	0	0
CHMP4B	0	0	0	0	0	0	0	0	0	0	0	0	0
CHMP5	0	0	0	0	0	0	0	0	0	0	0	0	0
CHUK	0	0	0	0	0	0	0	0	0	0	0	0	0
CLTA	0	0	0	0	0	0	0	0	1	1	0	0	0
CLTB	0	0	0	0	0	0	0	0	0	0	0	0	0
CLTC	0	0	0	0	0	0	0	0	1	1	1	0	0
COL1A1	0	0	1	1	0	1	1	0	0	1	1	0	0
COL1A2	0	0	1	1	0	1	1	0	0	1	1	0	0
COL5A1	0	0	0	0	0	0	0	0	0	1	1	0	0
COL6A1	0	0	0	0	0	0	0	0	0	1	1	0	0
COL6A2	0	0	0	0	0	0	0	0	0	1	1	0	0
CRK	0	1	0	0	0	1	1	0	0	0	0	0	0
CTSA	0	0	0	0	0	0	0	0	0	0	0	0	0
CTSB	0	0	0	0	0	0	0	0	0	0	0	0	0
CTSL1	0	0	0	0	0	0	0	0	0	0	0	0	0
CTSZ	0	0	0	0	0	0	0	0	0	0	0	0	0
EEA1	0	0	0	0	0	0	0	0	0	0	0	0	0
EGFR	0	0	0	0	0	0	0	0	1	1	1	0	0
EHD1	0	0	0	0	0	1	0	0	0	0	0	0	0
EHD2	0	0	0	0	0	1	0	0	0	0	0	0	0
EXOC3	0	0	0	0	0	0	0	0	0	0	0	0	0
GNG12	0	0	0	0	0	1	1	0	0	0	0	1	1
GNS	0	0	0	0	0	0	0	0	0	0	0	0	0

NAME	REACTOME_ING_BY_ILS	REACTOME_GPCR_DOWNSTREAM_ING	REACTOME_ING_BY_GPCR	REACTOME_LING_BY_NGF	REACTOME_NGF_LING_VIA_TRKA_FROM_THE_PLASMA_MEMBRANE	REACTOME_ING_BY_EGFR_IN_CANCER	REACTOME_ING_BY_FGFR_IN_DISEASE	REACTOME_ING_BY_FGFR	REACTOME_ING_BY_ERBB2	REACTOME_DOWNSTREAM__TRANSDUCTION	REACTOME_ING_BY_PDGF	KEGG_RIBOSOME	KEGG_PROTEASOME
CFL1	0	0	0	0	0	0	0	0	0	0	0	0	0
CHMP2A	0	0	0	0	0	0	0	0	0	0	0	0	0
CHMP4B	0	0	0	0	0	0	0	0	0	0	0	0	0
CHMP5	0	0	0	0	0	0	0	0	0	0	0	0	0
CHUK	1	0	0	1	1	1	1	1	1	1	1	0	0
CLTA	0	0	0	0	1	0	0	0	0	0	0	0	0
CLTB	0	0	0	0	0	0	0	0	0	0	0	0	0
CLTC	0	0	0	1	1	1	0	0	0	0	0	0	0
COL1A1	0	0	0	0	0	0	0	0	0	0	1	0	0
COL1A2	0	0	0	0	0	0	0	0	0	0	1	0	0
COL5A1	0	0	0	0	0	0	0	0	0	0	1	0	0
COL6A1	0	0	0	0	0	0	0	0	0	0	1	0	0
COL6A2	0	0	0	0	0	0	0	0	0	0	1	0	0
CRK	1	0	0	1	1	0	0	0	0	1	1	0	0
CTSA	0	0	0	0	0	0	0	0	0	0	0	0	0
CTSB	0	0	0	0	0	0	0	0	0	0	0	0	0
CTSL1	0	0	0	0	0	0	0	0	0	0	0	0	0
CTSZ	0	0	0	0	0	0	0	0	0	0	0	0	0
EEA1	0	0	0	0	0	0	0	0	0	0	0	0	0
EGFR	0	0	1	0	0	1	0	0	1	0	0	0	0
EHD1	0	0	0	0	0	0	0	0	0	0	0	0	0
EHD2	0	0	0	0	0	0	0	0	0	0	0	0	0
EXOC3	0	0	0	0	0	0	0	0	0	0	0	0	0
GNG12	0	1	1	0	0	0	0	0	0	0	0	0	0
GNS	0	0	0	0	0	0	0	0	0	0	0	0	0

NAME	KEGG_PYRUVATE_METABOLISM	KEGG_LYSOSOME	KEGG_ENDOCYTOSIS	KEGG_EPITHELIAL_CELL_ING_IN_HELICOBACTER_PYLORI_INFECTION	KEGG_AXON_GUIDANCE	KEGG_NEUROTROPHIN_ING_PATHWAY	KEGG_CHEMOKINE_ING_PATHWAY	KEGG_INSULIN_ING_PATHWAY	KEGG_PROSTATE_CANCER	KEGG_PATHWAYS_IN_CANCER	KEGG_ERBB_ING_PATHWAY	KEGG_MAPK_ING_PATHWAY	KEGG_PATHOGENIC_ESCHERICHIA_COLI_INFECTION
CFL1	0	0	0	0	1	0	0	0	0	0	0	0	0
CHMP2A	0	0	1	0	0	0	0	0	0	0	0	0	0
CHMP4B	0	0	1	0	0	0	0	0	0	0	0	0	0
CHMP5	0	0	1	0	0	0	0	0	0	0	0	0	0
CHUK	0	0	0	1	0	0	1	0	1	1	0	1	0
CLTA	0	0	1	0	0	0	0	0	0	0	0	0	0
CLTB	0	1	1	0	0	0	0	0	0	0	0	0	0
CLTC	0	1	1	0	0	0	0	0	0	0	0	0	0
COL1A1	0	0	0	0	0	0	0	0	0	0	0	0	0
COL1A2	0	0	0	0	0	0	0	0	0	0	0	0	0
COL5A1	0	0	0	0	0	0	0	0	0	0	0	0	0
COL6A1	0	0	0	0	0	0	0	0	0	0	0	0	0
COL6A2	0	0	0	0	0	0	0	0	0	0	0	0	0
CRK	0	0	0	0	0	1	1	1	0	1	1	1	0
CTSA	0	1	0	0	0	0	0	0	0	0	0	0	0
CTSB	0	1	0	0	0	0	0	0	0	0	0	0	0
CTSL1	0	1	0	0	0	0	0	0	0	0	0	0	0
CTSZ	0	1	0	0	0	0	0	0	0	0	0	0	0
EEA1	0	0	1	0	0	0	0	0	0	0	0	0	0
EGFR	0	0	1	1	0	0	0	0	1	1	1	1	0
EHD1	0	0	1	0	0	0	0	0	0	0	0	0	0
EHD2	0	0	1	0	0	0	0	0	0	0	0	0	0
EXOC3	0	0	0	0	0	0	0	0	0	0	0	0	0
GNG12	0	0	0	0	0	0	1	0	0	0	0	1	0
GNS	0	1	0	0	0	0	0	0	0	0	0	0	0

NAME	KEGG_TIGHT_JUNCTION	KEGG_ADHERENS_JUNCTION	KEGG_LEUKOCYTE_TRANSENDOTHELIAL_MIGRATION	KEGG_ARRHYTHMOGENIC_RIGHT_VENTRICULAR_CARDIOMYOPATHY_ARVC	KEGG_ECM_RECEPTOR_INTERACTION	KEGG_FOCAL_ADHESION	KEGG_FC_GAMMA_R_MEDIATED_PHAGOCYTOSIS	KEGG_REGULATION_OF_ACTIN_CYTOSKELETON
CFL1	0	0	0	0	0	0	1	1
CHMP2A	0	0	0	0	0	0	0	0
CHMP4B	0	0	0	0	0	0	0	0
CHMP5	0	0	0	0	0	0	0	0
CHUK	0	0	0	0	0	0	0	0
CLTA	0	0	0	0	0	0	0	0
CLTB	0	0	0	0	0	0	0	0
CLTC	0	0	0	0	0	0	0	0
COL1A1	0	0	0	0	1	1	0	0
COL1A2	0	0	0	0	1	1	0	0
COL5A1	0	0	0	0	1	1	0	0
COL6A1	0	0	0	0	1	1	0	0
COL6A2	0	0	0	0	1	1	0	0
CRK	0	0	0	0	0	1	1	1
CTSA	0	0	0	0	0	0	0	0
CTSB	0	0	0	0	0	0	0	0
CTSL1	0	0	0	0	0	0	0	0
CTSZ	0	0	0	0	0	0	0	0
EEA1	0	0	0	0	0	0	0	0
EGFR	0	1	0	0	0	1	0	1
EHD1	0	0	0	0	0	0	0	0
EHD2	0	0	0	0	0	0	0	0
EXOC3	1	0	0	0	0	0	0	0
GNG12	0	0	0	0	0	0	0	1
GNS	0	0	0	0	0	0	0	0

Table S6 The differential proteins contribute to the enrichment of the significant pathways for the public dataset

Core enrichment proteins in down-regulated pathways of hiPSCs/HFs (hiPSCs vs HFs)

NAME	REACTOME_GLUCOSE_METABOLISM	REACTOME_UNFOLDED_PROTEIN_RESPONSE	REACTOME_DIABETES_PATHWAYS	REACTOME_PROTEIN_FOLDING	REACTOME_PREFOLDIN_MEDIATED_TRANSFER_OF_SUBSTRATE_TO_CCT_TRIC	REACTOME_ASPARAGINE_N_LINKED_GLYCOSYLATION	REACTOME_POST_TRANSLATIONAL_PROTEIN_MODIFICATION	REACTOME_ACTIVATION_OF_THE_MRNA_UPON_BINDING_OF_THE_CAP_BINDING_COMPLEX_AND_EIFS_AND_SUBSEQUENT_BINDING_TO_43S	REACTOME_FORMATION_OF_THE_TERNARY_COMPLEX_AND_SUBSEQUENTLY_THE_43S_COMPLEX	REACTOME_METABOLISM_OF_PROTEINS	REACTOME_SRP_DEPENDENT_COTRANSLATIONAL_PROTEIN_TARGETING_TO_MEMBRANE	REACTOME_INFLUENZA_VIRAL_RNA_TRANSCRIPTION_AND_REPLICATION	REACTOME_NONSENSE_MEDIATED_DECAY_ENHANCED_BY_THE_EXON_JUNCTION_COMPLEX
GYS1	1	0	0	0	0	0	0	0	0	0	0	0	0
HGS	0	0	0	0	0	0	0	0	0	0	0	0	0
HSPA1B	0	0	0	0	0	0	0	0	0	0	0	0	0
HSPB1	0	0	0	0	0	0	0	0	0	0	0	0	0
ILK	0	0	0	0	0	0	0	0	0	0	0	0	0
IQGAP1	0	0	0	0	0	0	0	0	0	0	0	0	0
ITGA2	0	0	0	0	0	0	0	0	0	0	0	0	0
ITGA3	0	0	0	0	0	0	0	0	0	0	0	0	0
ITGA5	0	0	0	0	0	0	0	0	0	0	0	0	0
ITGAV	0	0	0	0	0	0	0	0	0	0	0	0	0
ITGB1	0	0	0	0	0	0	0	0	0	0	0	0	0
JAK1	0	0	0	0	0	0	0	0	0	0	0	0	0
LMNA	0	1	1	0	0	0	0	0	0	0	0	0	0
MAP2K1	0	0	0	0	0	0	0	0	0	0	0	0	0
MAP2K2	0	0	0	0	0	0	0	0	0	0	0	0	0
MAP2K3	0	0	0	0	0	0	0	0	0	0	0	0	0
MAPK1	0	0	0	0	0	0	0	0	0	0	0	0	0
MSN	0	0	0	0	0	0	0	0	0	0	0	0	0
MYH9	0	0	0	0	0	0	0	0	0	0	0	0	0
MYL9	0	0	0	0	0	0	0	0	0	0	0	0	0
PAK2	0	0	0	0	0	0	0	0	0	0	0	0	0
PARVA	0	0	0	0	0	0	0	0	0	0	0	0	0
PDGFRB	0	0	0	0	0	0	0	0	0	0	0	0	0
PFN1	0	0	0	0	0	0	0	0	0	0	0	0	0
PFN2	0	0	0	0	0	0	0	0	0	0	0	0	0

NAME	REACTOME_PEPTIDE_CHAIN_ELONGATION	REACTOME_3_UTR_MEDIATED_TRANSLATIONAL_REGULATION	REACTOME_TRANSLATION	REACTOME_CELL_CELL_COMMUNICATION	REACTOME_GOLGI_ASSOCIATED_VESICLE_BIOGENESIS	REACTOME_TRANS_GOLGI_NETWORK_VESICLE_BUDDING	REACTOME_MEMBRANE_TRAFFICKING	REACTOME_TRANSMISSION_ACROSS_CHEMICAL_SYNAPSES	REACTOME_NEURONAL_SYSTEM	REACTOME_MHC_CLASS_II_ANTIGEN_PRESENTATION	REACTOME_INNATE_IMMUNE_SYSTEM	REACTOME_IMMUNE_SYSTEM	REACTOME_ADAPTIVE_IMMUNE_SYSTEM
GYS1	0	0	0	0	0	0	0	0	0	0	0	0	0
HGS	0	0	0	0	0	0	1	0	0	0	0	0	0
HSPA1B	0	0	0	0	0	0	0	0	0	0	0	0	0
HSPB1	0	0	0	0	0	0	0	0	0	0	0	0	0
ILK	0	0	0	1	0	0	0	0	0	0	0	0	0
IQGAP1	0	0	0	1	0	0	0	0	0	0	0	0	0
ITGA2	0	0	0	0	0	0	0	0	0	0	0	0	0
ITGA3	0	0	0	0	0	0	0	0	0	0	0	0	0
ITGA5	0	0	0	0	0	0	0	0	0	0	0	0	0
ITGAV	0	0	0	0	0	0	0	0	0	0	0	1	1
ITGB1	0	0	0	1	0	0	0	0	0	0	0	1	1
JAK1	0	0	0	0	0	0	0	0	0	0	0	1	0
LMNA	0	0	0	0	0	0	0	0	0	0	0	0	0
MAP2K1	0	0	0	0	0	0	0	0	0	0	1	1	0
MAP2K2	0	0	0	0	0	0	0	0	0	0	1	1	0
MAP2K3	0	0	0	0	0	0	0	0	0	0	1	1	0
MAPK1	0	0	0	0	0	0	0	1	1	0	1	1	0
MSN	0	0	0	0	0	0	0	0	0	0	0	0	0
MYH9	0	0	0	0	0	0	0	0	0	0	0	0	0
MYL9	0	0	0	0	0	0	0	0	0	0	0	0	0
PAK2	0	0	0	0	0	0	0	0	0	0	0	1	1
PARVA	0	0	0	1	0	0	0	0	0	0	0	0	0
PDGFRB	0	0	0	0	0	0	0	0	0	0	0	0	0
PFN1	0	0	0	0	0	0	0	0	0	0	0	0	0
PFN2	0	0	0	0	0	0	0	0	0	0	0	0	0

NAME	REACTOME_CLASS_I_MHC_MEDIATED_ANTIGEN_PROCESSING_PRESENTATION	REACTOME_APOPTOSIS	REACTOME_ANTIGEN_PROCESSING_UBIQUITINATION_PROTEASOME_DEGRADATION	REACTOME_ING_BY_WNT	REACTOME_REGULATION_OF_MRNA_STABILITY_BY_PROTEINS_THAT_BIND_AU_RICH_ELEMENTS	REACTOME_DESTABILIZATION_OF_MRNA_BY_AUF1_HNRNP_D0	REACTOME_REGULATION_OF_APOPTOSIS	REACTOME_REGULATION_OF_ORNITHINE_DECARBOXYLASE_ODC	REACTOME_CROSS_PRESENTATION_OF_SOLUBLE_EXOGENOUS_ANTIGENS_ENDOSOMES	REACTOME_VIF_MEDIATED_DEGRADATION_OF_APOBEC3G	REACTOME_REGULATION_OF_MITOTIC_CELL_CYCLE	REACTOME_APC_C_CDC20_MEDIATED_DEGRADATION_OF_MITOTIC_PROTEINS	REACTOME_CDT1_ASSOCIATION_WITH_THE_CDC6_ORC_ORIGIN_COMPLEX
GYS1	0	0	0	0	0	0	0	0	0	0	0	0	0
HGS	0	0	0	0	0	0	0	0	0	0	0	0	0
HSPA1B	0	0	0	0	1	1	0	0	0	0	0	0	0
HSPB1	0	0	0	0	1	1	0	0	0	0	0	0	0
ILK	0	0	0	0	0	0	0	0	0	0	0	0	0
IQGAP1	0	0	0	0	0	0	0	0	0	0	0	0	0
ITGA2	0	0	0	0	0	0	0	0	0	0	0	0	0
ITGA3	0	0	0	0	0	0	0	0	0	0	0	0	0
ITGA5	0	0	0	0	0	0	0	0	0	0	0	0	0
ITGAV	1	0	0	0	0	0	0	0	0	0	0	0	0
ITGB1	0	0	0	0	0	0	0	0	0	0	0	0	0
JAK1	0	0	0	0	0	0	0	0	0	0	0	0	0
LMNA	0	1	0	0	0	0	0	0	0	0	0	0	0
MAP2K1	0	0	0	0	0	0	0	0	0	0	0	0	0
MAP2K2	0	0	0	0	0	0	0	0	0	0	0	0	0
MAP2K3	0	0	0	0	0	0	0	0	0	0	0	0	0
MAPK1	0	0	0	0	0	0	0	0	0	0	0	0	0
MSN	0	0	0	0	0	0	0	0	0	0	0	0	0
MYH9	0	0	0	0	0	0	0	0	0	0	0	0	0
MYL9	0	0	0	0	0	0	0	0	0	0	0	0	0
PAK2	0	1	0	0	0	0	1	0	0	0	0	0	0
PARVA	0	0	0	0	0	0	0	0	0	0	0	0	0
PDGFRB	0	0	0	0	0	0	0	0	0	0	0	0	0
PFN1	0	0	0	0	0	0	0	0	0	0	0	0	0
PFN2	0	0	0	0	0	0	0	0	0	0	0	0	0

NAME	REACTOME_APC_C_CDH1_MEDIATED_DEGRADATION_OF_CDC20_AND_OTHER_APC_C_CDH1_TARGETED_PROTEINS_IN_LATE_MITOSIS_EARLY_G1	REACTOME_CYCLIN_E_ASSOCIATED_EVENTS_DURING_G1_S_TRANSITION_	REACTOME_SCFSKP2_MEDIATED_DEGRADATION_OF_P27_P21	REACTOME_AUTODEGRADATION_OF_CDH1_BY_CDH1_APC_C	REACTOME_AUTODEGRADATION_OF_THE_E3_UBIQUITIN_LIGASE_COP1	REACTOME_SCF_BETA_TRCP_MEDIATED_DEGRADATION_OF_EMI1	REACTOME_P53_INDEPENDENT_G1_S_DNA_DAMAGE_CHECKPOINT	REACTOME_CDK_MEDIATED_PHOSPHORYLATION_AND_REMOVAL_OF_CDC6	REACTOME_P53_DEPENDENT_G1_DNA_DAMAGE_RESPONSE	REACTOME_ACTIVATION_OF_NF_KAPPAB_IN_B_CELLS	REACTOME_DOWNSTREAM_ING_EVENTS_OF_B_CELL_RECEPTOR_BCR	REACTOME_ING_BY_THE_B_CELL_RECEPTOR_BCR	REACTOME_ANTIGEN_PROCESSING_CROSS_PRESENTATION
GYS1	0	0	0	0	0	0	0	0	0	0	0	0	0
HGS	0	0	0	0	0	0	0	0	0	0	0	0	0
HSPA1B	0	0	0	0	0	0	0	0	0	0	0	0	0
HSPB1	0	0	0	0	0	0	0	0	0	0	0	0	0
ILK	0	0	0	0	0	0	0	0	0	0	0	0	0
IQGAP1	0	0	0	0	0	0	0	0	0	0	0	0	0
ITGA2	0	0	0	0	0	0	0	0	0	0	0	0	0
ITGA3	0	0	0	0	0	0	0	0	0	0	0	0	0
ITGA5	0	0	0	0	0	0	0	0	0	0	0	0	0
ITGAV	0	0	0	0	0	0	0	0	0	0	0	0	1
ITGB1	0	0	0	0	0	0	0	0	0	0	0	0	0
JAK1	0	0	0	0	0	0	0	0	0	0	0	0	0
LMNA	0	0	0	0	0	0	0	0	0	0	0	0	0
MAP2K1	0	0	0	0	0	0	0	0	0	0	0	0	0
MAP2K2	0	0	0	0	0	0	0	0	0	0	0	0	0
MAP2K3	0	0	0	0	0	0	0	0	0	0	0	0	0
MAPK1	0	0	0	0	0	0	0	0	0	0	0	0	0
MSN	0	0	0	0	0	0	0	0	0	0	0	0	0
MYH9	0	0	0	0	0	0	0	0	0	0	0	0	0
MYL9	0	0	0	0	0	0	0	0	0	0	0	0	0
PAK2	0	0	0	0	0	0	0	0	0	0	0	0	0
PARVA	0	0	0	0	0	0	0	0	0	0	0	0	0
PDGFRB	0	0	0	0	0	0	0	0	0	0	0	0	0
PFN1	0	0	0	0	0	0	0	0	0	0	0	0	0
PFN2	0	0	0	0	0	0	0	0	0	0	0	0	0

NAME	REACTOME_ER_PHAGOSOME_PATHWAY	REACTOME_ING_BY_INSULIN_RECEPTOR	REACTOME_CELL_SURFACE_INTERACTIONS_AT_THE_VASCULAR_WALL	REACTOME_INTEGRIN_CELL_SURFACE_INTERACTIONS	REACTOME_RESPONSE_TO_ELEVATED_PLATELET_CYTOSOLIC_CA2_	REACTOME_HEMOSTASIS	REACTOME_PLATELET_ACTIVATION_ING_AND_AGGREGATION	REACTOME_SEMAPHORIN_INTERACTIONS	REACTOME_L1CAM_INTERACTIONS	REACTOME_DEVELOPMENTAL_BIOLOGY	REACTOME_AXON_GUIDANCE	REACTOME_INTEGRATION_OF_ENERGY_METABOLISM	REACTOME_REGULATION_OF_INSULIN_SECRETION
GYS1	0	0	0	0	0	0	0	0	0	0	0	0	0
HGS	0	0	0	0	0	0	0	0	0	0	0	0	0
HSPA1B	0	0	0	0	0	0	0	0	0	0	0	0	0
HSPB1	0	0	0	0	0	0	0	0	0	0	0	0	0
ILK	0	0	0	0	0	0	0	0	0	0	0	0	0
IQGAP1	0	0	0	0	0	0	0	0	0	0	0	1	1
ITGA2	0	0	0	1	0	1	0	0	1	1	1	0	0
ITGA3	0	0	1	1	0	1	0	0	0	0	0	0	0
ITGA5	0	0	1	1	0	1	0	0	1	1	1	0	0
ITGAV	0	0	0	0	0	0	0	0	1	1	0	0	0
ITGB1	0	0	1	1	0	1	0	1	1	1	1	0	0
JAK1	0	0	0	0	0	0	0	0	0	0	0	0	0
LMNA	0	0	0	0	0	0	0	0	0	0	0	0	0
MAP2K1	0	1	0	0	0	0	0	0	0	0	0	0	0
MAP2K2	0	1	0	0	0	0	0	0	1	1	1	0	0
MAP2K3	0	0	0	0	0	0	0	0	0	0	0	0	0
MAPK1	0	1	0	0	0	1	1	0	1	1	1	0	0
MSN	0	0	0	0	0	0	0	0	1	1	1	0	0
MYH9	0	0	0	0	0	0	0	1	0	1	1	0	0
MYL9	0	0	0	0	0	0	0	1	0	1	1	0	0
PAK2	0	0	0	0	0	0	0	1	0	1	1	0	0
PARVA	0	0	0	0	0	0	0	0	0	0	0	0	0
PDGFRB	0	0	0	0	0	0	0	0	0	0	0	0	0
PFN1	0	0	0	0	0	0	1	0	0	1	0	0	0
PFN2	0	0	0	0	0	0	0	0	0	1	1	0	0

NAME	REACTOME_ING_BY_ILS	REACTOME_GPCR_DOWNSTREAM_ING	REACTOME_ING_BY_GPCR	REACTOME_LING_BY_NGF	REACTOME_NGF_LING_VIA_TRKA_FROM_THE_PLASMA_MEMBRANE	REACTOME_ING_BY_EGFR_IN_CANCER	REACTOME_ING_BY_FGFR_IN_DISEASE	REACTOME_ING_BY_FGFR	REACTOME_ING_BY_ERBB2	REACTOME_DOWNSTREAM__TRANSDUCTION	REACTOME_ING_BY_PDGF	KEGG_RIBOSOME	KEGG_PROTEASOME
GYS1	0	0	0	0	0	0	0	0	0	0	0	0	0
HGS	0	0	0	0	0	1	0	0	0	0	0	0	0
HSPA1B	0	0	0	0	0	0	0	0	0	0	0	0	0
HSPB1	0	0	0	0	0	0	0	0	0	0	0	0	0
ILK	0	0	0	0	0	0	0	0	0	0	0	0	0
IQGAP1	0	0	0	0	0	0	0	0	0	0	0	0	0
ITGA2	0	0	0	0	0	0	0	0	0	0	0	0	0
ITGA3	0	0	0	0	0	0	0	0	0	0	0	0	0
ITGA5	0	0	0	0	0	0	0	0	0	0	0	0	0
ITGAV	0	0	0	0	0	0	0	0	0	0	0	0	0
ITGB1	0	0	0	0	0	0	0	0	0	0	0	0	0
JAK1	1	0	0	0	0	0	0	0	0	0	0	0	0
LMNA	0	0	0	0	0	0	0	0	0	0	0	0	0
MAP2K1	0	0	0	0	0	0	0	0	0	0	0	0	0
MAP2K2	1	0	1	1	1	1	1	1	1	1	1	0	0
MAP2K3	0	0	0	0	0	0	0	0	0	0	0	0	0
MAPK1	1	0	1	1	1	1	1	1	1	1	1	0	0
MSN	0	0	0	0	0	0	0	0	0	0	0	0	0
MYH9	0	0	0	0	0	0	0	0	0	0	0	0	0
MYL9	0	0	0	0	0	0	0	0	0	0	0	0	0
PAK2	0	0	0	0	0	0	0	0	0	0	0	0	0
PARVA	0	0	0	0	0	0	0	0	0	0	0	0	0
PDGFRB	0	0	0	0	0	0	0	0	0	1	1	0	0
PFN1	0	0	0	0	0	0	0	0	0	0	0	0	0
PFN2	0	0	0	0	0	0	0	0	0	0	0	0	0

NAME	KEGG_PYRUVATE_METABOLISM	KEGG_LYSOSOME	KEGG_ENDOCYTOSIS	KEGG_EPITHELIAL_CELL_ING_IN_HELICOBACTER_PYLORI_INFECTION	KEGG_AXON_GUIDANCE	KEGG_NEUROTROPHIN_ING_PATHWAY	KEGG_CHEMOKINE_ING_PATHWAY	KEGG_INSULIN_ING_PATHWAY	KEGG_PROSTATE_CANCER	KEGG_PATHWAYS_IN_CANCER	KEGG_ERBB_ING_PATHWAY	KEGG_MAPK_ING_PATHWAY	KEGG_PATHOGENIC_ESCHERICHIA_COLI_INFECTION
GYS1	0	0	0	0	0	0	0	1	0	0	0	0	0
HGS	0	0	1	0	0	0	0	0	0	0	0	0	0
HSPA1B	0	0	1	0	0	0	0	0	0	0	0	0	0
HSPB1	0	0	0	0	0	0	0	0	0	0	0	1	0
ILK	0	0	0	0	0	0	0	0	0	0	0	0	0
IQGAP1	0	0	0	0	0	0	0	0	0	0	0	0	0
ITGA2	0	0	0	0	0	0	0	0	0	1	0	0	0
ITGA3	0	0	0	0	0	0	0	0	0	1	0	0	0
ITGA5	0	0	0	0	0	0	0	0	0	0	0	0	0
ITGAV	0	0	0	0	0	0	0	0	0	0	0	0	0
ITGB1	0	0	0	0	1	0	0	0	0	1	0	0	1
JAK1	0	0	0	0	0	0	0	0	0	1	0	0	0
LMNA	0	0	0	0	0	0	0	0	0	0	0	0	0
MAP2K1	0	0	0	0	0	1	0	0	0	0	0	0	0
MAP2K2	0	0	0	0	0	1	0	1	1	1	1	1	0
MAP2K3	0	0	0	0	0	0	0	0	0	0	0	1	0
MAPK1	0	0	0	0	1	1	1	1	1	1	1	1	0
MSN	0	0	0	0	0	0	0	0	0	0	0	0	0
MYH9	0	0	0	0	0	0	0	0	0	0	0	0	0
MYL9	0	0	0	0	0	0	0	0	0	0	0	0	0
PAK2	0	0	0	0	1	0	0	0	0	0	1	1	0
PARVA	0	0	0	0	0	0	0	0	0	0	0	0	0
PDGFRB	0	0	0	0	0	0	0	0	1	1	0	1	0
PFN1	0	0	0	0	0	0	0	0	0	0	0	0	0
PFN2	0	0	0	0	0	0	0	0	0	0	0	0	0

NAME	KEGG_TIGHT_JUNCTION	KEGG_ADHERENS_JUNCTION	KEGG_LEUKOCYTE_TRANSENDOTHELIAL_MIGRATION	KEGG_ARRHYTHMOGENIC_RIGHT_VENTRICULAR_CARDIOMYOPATHY_ARVC	KEGG_ECM_RECEPTOR_INTERACTION	KEGG_FOCAL_ADHESION	KEGG_FC_GAMMA_R_MEDIATED_PHAGOCYTOSIS	KEGG_REGULATION_OF_ACTIN_CYTOSKELETON
GYS1	0	0	0	0	0	0	0	0
HGS	0	0	0	0	0	0	0	0
HSPA1B	0	0	0	0	0	0	0	0
HSPB1	0	0	0	0	0	0	0	0
ILK	0	0	0	0	0	1	0	0
IQGAP1	0	1	0	0	0	0	0	1
ITGA2	0	0	0	1	1	1	0	1
ITGA3	0	0	0	1	1	1	0	1
ITGA5	0	0	0	1	1	1	0	1
ITGAV	0	0	0	0	0	0	0	1
ITGB1	0	0	1	1	1	1	0	1
JAK1	0	0	0	0	0	0	0	0
LMNA	0	0	0	1	0	0	0	0
MAP2K1	0	0	0	0	0	0	0	0
MAP2K2	0	0	0	0	0	0	0	1
MAP2K3	0	0	0	0	0	0	0	0
MAPK1	0	1	0	0	0	1	1	1
MSN	0	0	1	0	0	0	0	1
MYH9	1	0	0	0	0	0	0	1
MYL9	1	0	1	0	0	1	0	1
PAK2	0	0	0	0	0	1	0	1
PARVA	0	0	0	0	0	1	0	0
PDGFRB	0	0	0	0	0	1	0	1
PFN1	0	0	0	0	0	0	0	1
PFN2	0	0	0	0	0	0	0	1

Table S6 The differential proteins contribute to the enrichment of the significant pathways for the public dataset

Core enrichment proteins in down-regulated pathways of hiPSCs/HFs (hiPSCs vs HFs)

NAME	REACTOME_GLUCOSE_METABOLISM	REACTOME_UNFOLDED_PROTEIN_RESPONSE	REACTOME_DIABETES_PATHWAYS	REACTOME_PROTEIN_FOLDING	REACTOME_PREFOLDIN_MEDIATED_TRANSFER_OF_SUBSTRATE_TO_CCT_TRIC	REACTOME_ASPARAGINE_N_LINKED_GLYCOSYLATION	REACTOME_POST_TRANSLATIONAL_PROTEIN_MODIFICATION	REACTOME_ACTIVATION_OF_THE_MRNA_UPON_BINDING_OF_THE_CAP_BINDING_COMPLEX_AND_EIFS_AND_SUBSEQUENT_BINDING_TO_43S	REACTOME_FORMATION_OF_THE_TERNARY_COMPLEX_AND_SUBSEQUENTLY_THE_43S_COMPLEX	REACTOME_METABOLISM_OF_PROTEINS	REACTOME_SRP_DEPENDENT_COTRANSLATIONAL_PROTEIN_TARGETING_TO_MEMBRANE	REACTOME_INFLUENZA_VIRAL_RNA_TRANSCRIPTION_AND_REPLICATION	REACTOME_NONSENSE_MEDIATED_DECAY_ENHANCED_BY_THE_EXON_JUNCTION_COMPLEX
PKM2	1	0	0	0	0	0	0	0	0	0	0	0	0
PPP2CB	1	0	0	0	0	0	0	0	0	0	0	0	0
PRKAA1	0	0	0	0	0	0	0	0	0	0	0	0	0
PRKAR1A	0	0	0	0	0	0	0	0	0	0	0	0	0
PRKAR2A	0	0	0	0	0	0	0	0	0	0	0	0	0
PRKCA	0	0	0	0	0	0	0	0	0	0	0	0	0
PSMA1	0	0	0	0	0	0	0	0	0	0	0	0	0
PSMA2	0	0	0	0	0	0	0	0	0	0	0	0	0
PSMA3	0	0	0	0	0	0	0	0	0	0	0	0	0
PSMA5	0	0	0	0	0	0	0	0	0	0	0	0	0
PSMA6	0	0	0	0	0	0	0	0	0	0	0	0	0
PSMA7	0	0	0	0	0	0	0	0	0	0	0	0	0
PSMB1	0	0	0	0	0	0	0	0	0	0	0	0	0
PSMB2	0	0	0	0	0	0	0	0	0	0	0	0	0
PSMB3	0	0	0	0	0	0	0	0	0	0	0	0	0
PSMB4	0	0	0	0	0	0	0	0	0	0	0	0	0
PSMB7	0	0	0	0	0	0	0	0	0	0	0	0	0
PSMC1	0	0	0	0	0	0	0	0	0	0	0	0	0
PSMC2	0	0	0	0	0	0	0	0	0	0	0	0	0
PSMC3	0	0	0	0	0	0	0	0	0	0	0	0	0
PSMC5	0	0	0	0	0	0	0	0	0	0	0	0	0
PSMC6	0	0	0	0	0	0	0	0	0	0	0	0	0
PSMD11	0	0	0	0	0	0	0	0	0	0	0	0	0
PSMD12	0	0	0	0	0	0	0	0	0	0	0	0	0
PSMD13	0	0	0	0	0	0	0	0	0	0	0	0	0

NAME	REACTOME_PEPTIDE_CHAIN_ELONGATION	REACTOME_3_UTR_MEDIATED_TRANSLATIONAL_REGULATION	REACTOME_TRANSLATION	REACTOME_CELL_CELL_COMMUNICATION	REACTOME_GOLGI_ASSOCIATED_VESICLE_BIOGENESIS	REACTOME_TRANS_GOLGI_NETWORK_VESICLE_BUDDING	REACTOME_MEMBRANE_TRAFFICKING	REACTOME_TRANSMISSION_ACROSS_CHEMICAL_SYNAPSES	REACTOME_NEURONAL_SYSTEM	REACTOME_MHC_CLASS_II_ANTIGEN_PRESENTATION	REACTOME_INNATE_IMMUNE_SYSTEM	REACTOME_IMMUNE_SYSTEM	REACTOME_ADAPTIVE_IMMUNE_SYSTEM
PKM2	0	0	0	0	0	0	0	0	0	0	0	0	0
PPP2CB	0	0	0	0	0	0	0	0	0	0	1	1	1
PRKAA1	0	0	0	0	0	0	0	0	0	0	0	0	0
PRKAR1A	0	0	0	0	0	0	0	0	0	0	0	0	0
PRKAR2A	0	0	0	0	0	0	0	0	0	0	0	0	0
PRKCA	0	0	0	0	0	0	0	1	1	0	0	0	0
PSMA1	0	0	0	0	0	0	0	0	0	0	0	1	1
PSMA2	0	0	0	0	0	0	0	0	0	0	0	0	0
PSMA3	0	0	0	0	0	0	0	0	0	0	0	0	0
PSMA5	0	0	0	0	0	0	0	0	0	0	0	1	1
PSMA6	0	0	0	0	0	0	0	0	0	0	0	0	0
PSMA7	0	0	0	0	0	0	0	0	0	0	0	0	0
PSMB1	0	0	0	0	0	0	0	0	0	0	0	0	0
PSMB2	0	0	0	0	0	0	0	0	0	0	0	0	0
PSMB3	0	0	0	0	0	0	0	0	0	0	0	0	0
PSMB4	0	0	0	0	0	0	0	0	0	0	0	1	1
PSMB7	0	0	0	0	0	0	0	0	0	0	0	0	0
PSMC1	0	0	0	0	0	0	0	0	0	0	0	1	1
PSMC2	0	0	0	0	0	0	0	0	0	0	0	1	1
PSMC3	0	0	0	0	0	0	0	0	0	0	0	1	1
PSMC5	0	0	0	0	0	0	0	0	0	0	0	1	1
PSMC6	0	0	0	0	0	0	0	0	0	0	0	1	1
PSMD11	0	0	0	0	0	0	0	0	0	0	0	1	1
PSMD12	0	0	0	0	0	0	0	0	0	0	0	1	1
PSMD13	0	0	0	0	0	0	0	0	0	0	0	1	1

NAME	REACTOME_CLASS_I_MHC_MEDIATED_ANTIGEN_PROCESSING_PRESENTATION	REACTOME_APOPTOSIS	REACTOME_ANTIGEN_PROCESSING_UBIQUITINATION_PROTEASOME_DEGRADATION	REACTOME_ING_BY_WNT	REACTOME_REGULATION_OF_MRNA_STABILITY_BY_PROTEINS_THAT_BIND_AU_RICH_ELEMENTS	REACTOME_DESTABILIZATION_OF_MRNA_BY_AUF1_HNRNP_D0	REACTOME_REGULATION_OF_APOPTOSIS	REACTOME_REGULATION_OF_ORNITHINE_DECARBOXYLASE_ODC	REACTOME_CROSS_PRESENTATION_OF_SOLUBLE_EXOGENOUS_ANTIGENS_ENDOSOMES	REACTOME_VIF_MEDIATED_DEGRADATION_OF_APOBEC3G	REACTOME_REGULATION_OF_MITOTIC_CELL_CYCLE	REACTOME_APC_C_CDC20_MEDIATED_DEGRADATION_OF_MITOTIC_PROTEINS	REACTOME_CDT1_ASSOCIATION_WITH_THE_CDC6_ORC_ORIGIN_COMPLEX
PKM2	0	0	0	0	0	0	0	0	0	0	0	0	0
PPP2CB	0	0	0	1	0	0	0	0	0	0	0	0	0
PRKAA1	0	0	0	0	0	0	0	0	0	0	0	0	0
PRKAR1A	0	0	0	0	0	0	0	0	0	0	0	0	0
PRKAR2A	0	0	0	0	0	0	0	0	0	0	0	0	0
PRKCA	0	0	0	0	1	0	0	0	0	0	0	0	0
PSMA1	1	1	1	1	1	1	1	1	1	1	1	1	1
PSMA2	1	1	1	1	1	1	1	1	1	1	1	1	1
PSMA3	0	1	1	1	1	1	1	1	1	1	1	1	1
PSMA5	1	1	1	1	1	1	1	1	1	1	1	1	1
PSMA6	1	1	1	1	1	1	1	1	1	1	1	1	1
PSMA7	0	1	1	1	1	1	1	1	1	1	1	1	1
PSMB1	1	1	1	1	1	1	1	1	1	1	1	1	1
PSMB2	1	1	1	1	1	1	1	1	1	1	1	1	1
PSMB3	1	1	1	1	1	1	1	1	1	1	1	1	1
PSMB4	1	1	1	1	1	1	1	1	1	1	1	1	1
PSMB7	1	1	1	1	1	1	1	1	1	1	1	1	1
PSMC1	1	1	1	1	1	1	1	1	1	1	1	1	1
PSMC2	1	1	1	1	1	1	1	1	1	1	1	1	1
PSMC3	1	1	1	1	1	1	1	1	1	1	1	1	1
PSMC5	1	1	1	1	1	1	1	1	1	1	1	1	1
PSMC6	1	1	1	1	1	1	1	1	1	1	1	1	1
PSMD11	1	1	1	1	1	1	1	1	1	1	1	1	1
PSMD12	1	1	1	1	1	1	1	1	1	1	1	1	1
PSMD13	1	1	1	1	1	1	1	1	1	1	1	1	1

NAME	REACTOME_APC_C_CDH1_MEDIATED_DEGRADATION_OF_CDC20_AND_OTHER_APC_C_CDH1_TARGETED_PROTEINS_IN_LATE_MITOSIS_EARLY_G1	REACTOME_CYCLIN_E_ASSOCIATED_EVENTS_DURING_G1_S_TRANSITION_	REACTOME_SCFSKP2_MEDIATED_DEGRADATION_OF_P27_P21	REACTOME_AUTODEGRADATION_OF_CDH1_BY_CDH1_APC_C	REACTOME_AUTODEGRADATION_OF_THE_E3_UBIQUITIN_LIGASE_COP1	REACTOME_SCF_BETA_TRCP_MEDIATED_DEGRADATION_OF_EMI1
PKM2	0	0	0	0	0	0
PPP2CB	0	0	0	0	0	0
PRKAA1	0	0	0	0	0	0
PRKAR1A	0	0	0	0	0	0
PRKAR2A	0	0	0	0	0	0
PRKCA	0	0	0	0	0	0
PSMA1	1	1	1	1	1	1
PSMA2	1	1	1	1	1	1
PSMA3	1	1	1	1	1	1
PSMA5	1	1	1	1	1	1
PSMA6	1	1	1	1	1	1
PSMA7	1	1	1	1	1	1
PSMB1	1	1	1	1	1	1
PSMB2	1	1	1	1	1	1
PSMB3	1	1	1	1	1	1
PSMB4	1	1	1	1	1	1
PSMB7	1	1	1	1	1	1
PSMC1	1	1	1	1	1	1
PSMC2	1	1	1	1	1	1
PSMC3	1	1	1	1	1	1
PSMC5	1	1	1	1	1	1
PSMC6	1	1	1	1	1	1
PSMD11	1	1	1	1	1	1
PSMD12	1	1	1	1	1	1
PSMD13	1	1	1	1	1	1

NAME	REACTOME_P53_INDEPENDENT_G1_S_DNA_DAMAGE_CHECKPOINT	REACTOME_CDK_MEDIATED_PHOSPHORYLATION_AND_REMOVAL_OF_CDC6	REACTOME_P53_DEPENDENT_G1_DNA_DAMAGE_RESPONSE	REACTOME_ACTIVATION_OF_NF_KAPPAB_IN_B_CELLS	REACTOME_DOWNSTREAM_ING_EVENTS_OF_B_CELL_RECEPTOR_BCR	REACTOME_ING_BY_THE_B_CELL_RECEPTOR_BCR	REACTOME_ANTIGEN_PROCESSING_CROSS_PRESENTATION
PKM2	0	0	0	0	0	0	0
PPP2CB	0	0	0	0	0	0	0
PRKAA1	0	0	0	0	0	0	0
PRKAR1A	0	0	0	0	0	0	0
PRKAR2A	0	0	0	0	0	0	0
PRKCA	0	0	0	0	0	0	0
PSMA1	1	1	1	1	1	1	1
PSMA2	1	1	1	1	1	1	1
PSMA3	1	1	1	1	1	1	1
PSMA5	1	1	1	1	1	1	1
PSMA6	1	1	1	1	1	1	1
PSMA7	1	1	1	1	1	1	1
PSMB1	1	1	1	1	1	1	1
PSMB2	1	1	1	1	1	1	1
PSMB3	1	1	1	1	1	1	1
PSMB4	1	1	1	1	1	1	1
PSMB7	1	1	1	1	1	1	1
PSMC1	1	1	1	1	1	1	1
PSMC2	1	1	1	1	1	1	1
PSMC3	1	1	1	1	1	1	1
PSMC5	1	1	1	1	1	1	1
PSMC6	1	1	1	1	1	1	1
PSMD11	1	1	1	1	1	1	1
PSMD12	1	1	1	1	1	1	1
PSMD13	1	1	1	1	1	1	1

NAME	REACTOME_ER_PHAGOSOME_PATHWAY	REACTOME_ING_BY_INSULIN_RECEPTOR	REACTOME_CELL_SURFACE_INTERACTIONS_AT_THE_VASCULAR_WALL	REACTOME_INTEGRIN_CELL_SURFACE_INTERACTIONS	REACTOME_RESPONSE_TO_ELEVATED_PLATELET_CYTOSOLIC_CA2_	REACTOME_HEMOSTASIS	REACTOME_PLATELET_ACTIVATION_ING_AND_AGGREGATION	REACTOME_SEMAPHORIN_INTERACTIONS	REACTOME_L1CAM_INTERACTIONS	REACTOME_DEVELOPMENTAL_BIOLOGY	REACTOME_AXON_GUIDANCE	REACTOME_INTEGRATION_OF_ENERGY_METABOLISM	REACTOME_REGULATION_OF_INSULIN_SECRETION
PKM2	0	0	0	0	0	0	0	0	0	0	0	0	0
PPP2CB	0	0	0	0	0	1	0	0	0	0	0	1	0
PRKAA1	0	1	0	0	0	0	0	0	0	0	0	0	0
PRKAR1A	0	0	0	0	0	1	0	0	0	0	0	1	1
PRKAR2A	0	0	0	0	0	1	0	0	0	0	0	1	1
PRKCA	0	0	0	0	1	1	1	0	0	0	0	1	1
PSMA1	1	0	0	0	0	0	0	0	0	0	0	0	0
PSMA2	1	0	0	0	0	0	0	0	0	0	0	0	0
PSMA3	1	0	0	0	0	0	0	0	0	0	0	0	0
PSMA5	1	0	0	0	0	0	0	0	0	0	0	0	0
PSMA6	1	0	0	0	0	0	0	0	0	0	0	0	0
PSMA7	1	0	0	0	0	0	0	0	0	0	0	0	0
PSMB1	1	0	0	0	0	0	0	0	0	0	0	0	0
PSMB2	1	0	0	0	0	0	0	0	0	0	0	0	0
PSMB3	1	0	0	0	0	0	0	0	0	0	0	0	0
PSMB4	1	0	0	0	0	0	0	0	0	0	0	0	0
PSMB7	1	0	0	0	0	0	0	0	0	0	0	0	0
PSMC1	1	0	0	0	0	0	0	0	0	0	0	0	0
PSMC2	1	0	0	0	0	0	0	0	0	0	0	0	0
PSMC3	1	0	0	0	0	0	0	0	0	0	0	0	0
PSMC5	1	0	0	0	0	0	0	0	0	0	0	0	0
PSMC6	1	0	0	0	0	0	0	0	0	0	0	0	0
PSMD11	1	0	0	0	0	0	0	0	0	0	0	0	0
PSMD12	1	0	0	0	0	0	0	0	0	0	0	0	0
PSMD13	1	0	0	0	0	0	0	0	0	0	0	0	0

NAME	REACTOME_ING_BY_ILS	REACTOME_GPCR_DOWNSTREAM_ING	REACTOME_ING_BY_GPCR	REACTOME_LING_BY_NGF	REACTOME_NGF_LING_VIA_TRKA_FROM_THE_PLASMA_MEMBRANE	REACTOME_ING_BY_EGFR_IN_CANCER	REACTOME_ING_BY_FGFR_IN_DISEASE	REACTOME_ING_BY_FGFR	REACTOME_ING_BY_ERBB2	REACTOME_DOWNSTREAM__TRANSDUCTION	REACTOME_ING_BY_PDGF	KEGG_RIBOSOME	KEGG_PROTEASOME
PKM2	0	0	0	0	0	0	0	0	0	0	0	0	0
PPP2CB	0	0	1	1	1	0	1	1	0	0	0	0	0
PRKAA1	0	0	0	0	0	0	0	0	0	0	0	0	0
PRKAR1A	0	0	1	1	1	1	1	1	1	1	1	0	0
PRKAR2A	0	0	1	1	1	1	1	1	1	1	1	0	0
PRKCA	0	1	1	1	1	1	1	1	1	1	1	0	0
PSMA1	0	0	0	0	0	0	0	0	0	0	0	0	1
PSMA2	0	0	0	0	0	0	0	0	0	0	0	0	1
PSMA3	0	0	0	0	0	0	0	0	0	0	0	0	1
PSMA5	0	0	0	0	0	0	0	0	0	0	0	0	1
PSMA6	0	0	0	0	0	0	0	0	0	0	0	0	1
PSMA7	0	0	0	0	0	0	0	0	0	0	0	0	1
PSMB1	0	0	0	0	0	0	0	0	0	0	0	0	1
PSMB2	0	0	0	0	0	0	0	0	0	0	0	0	1
PSMB3	0	0	0	0	0	0	0	0	0	0	0	0	1
PSMB4	0	0	0	0	0	0	0	0	0	0	0	0	1
PSMB7	0	0	0	0	0	0	0	0	0	0	0	0	1
PSMC1	0	0	0	0	0	0	0	0	0	0	0	0	1
PSMC2	0	0	0	0	0	0	0	0	0	0	0	0	1
PSMC3	0	0	0	0	0	0	0	0	0	0	0	0	1
PSMC5	0	0	0	0	0	0	0	0	0	0	0	0	1
PSMC6	0	0	0	0	0	0	0	0	0	0	0	0	1
PSMD11	0	0	0	0	0	0	0	0	0	0	0	0	1
PSMD12	0	0	0	0	0	0	0	0	0	0	0	0	1
PSMD13	0	0	0	0	0	0	0	0	0	0	0	0	1

NAME	KEGG_PYRUVATE_METABOLISM	KEGG_LYSOSOME	KEGG_ENDOCYTOSIS	KEGG_EPITHELIAL_CELL_ING_IN_HELICOBACTER_PYLORI_INFECTION	KEGG_AXON_GUIDANCE	KEGG_NEUROTROPHIN_ING_PATHWAY	KEGG_CHEMOKINE_ING_PATHWAY	KEGG_INSULIN_ING_PATHWAY	KEGG_PROSTATE_CANCER	KEGG_PATHWAYS_IN_CANCER	KEGG_ERBB_ING_PATHWAY	KEGG_MAPK_ING_PATHWAY	KEGG_PATHOGENIC_ESCHERICHIA_COLI_INFECTION
PKM2	1	0	0	0	0	0	0	0	0	0	0	0	0
PPP2CB	0	0	0	0	0	0	0	0	0	0	0	0	0
PRKAA1	0	0	0	0	0	0	0	1	0	0	0	0	0
PRKAR1A	0	0	0	0	0	0	0	1	0	0	0	0	0
PRKAR2A	0	0	0	0	0	0	0	1	0	0	0	0	0
PRKCA	0	0	0	0	0	0	0	0	0	1	1	1	1
PSMA1	0	0	0	0	0	0	0	0	0	0	0	0	0
PSMA2	0	0	0	0	0	0	0	0	0	0	0	0	0
PSMA3	0	0	0	0	0	0	0	0	0	0	0	0	0
PSMA5	0	0	0	0	0	0	0	0	0	0	0	0	0
PSMA6	0	0	0	0	0	0	0	0	0	0	0	0	0
PSMA7	0	0	0	0	0	0	0	0	0	0	0	0	0
PSMB1	0	0	0	0	0	0	0	0	0	0	0	0	0
PSMB2	0	0	0	0	0	0	0	0	0	0	0	0	0
PSMB3	0	0	0	0	0	0	0	0	0	0	0	0	0
PSMB4	0	0	0	0	0	0	0	0	0	0	0	0	0
PSMB7	0	0	0	0	0	0	0	0	0	0	0	0	0
PSMC1	0	0	0	0	0	0	0	0	0	0	0	0	0
PSMC2	0	0	0	0	0	0	0	0	0	0	0	0	0
PSMC3	0	0	0	0	0	0	0	0	0	0	0	0	0
PSMC5	0	0	0	0	0	0	0	0	0	0	0	0	0
PSMC6	0	0	0	0	0	0	0	0	0	0	0	0	0
PSMD11	0	0	0	0	0	0	0	0	0	0	0	0	0
PSMD12	0	0	0	0	0	0	0	0	0	0	0	0	0
PSMD13	0	0	0	0	0	0	0	0	0	0	0	0	0

NAME	KEGG_TIGHT_JUNCTION	KEGG_ADHERENS_JUNCTION	KEGG_LEUKOCYTE_TRANSENDOTHELIAL_MIGRATION	KEGG_ARRHYTHMOGENIC_RIGHT_VENTRICULAR_CARDIOMYOPATHY_ARVC	KEGG_ECM_RECEPTOR_INTERACTION	KEGG_FOCAL_ADHESION	KEGG_FC_GAMMA_R_MEDIATED_PHAGOCYTOSIS	KEGG_REGULATION_OF_ACTIN_CYTOSKELETON
PKM2	0	0	0	0	0	0	0	0
PPP2CB	1	0	0	0	0	0	0	0
PRKAA1	0	0	0	0	0	0	0	0
PRKAR1A	0	0	0	0	0	0	0	0
PRKAR2A	0	0	0	0	0	0	0	0
PRKCA	1	0	1	0	0	1	1	0
PSMA1	0	0	0	0	0	0	0	0
PSMA2	0	0	0	0	0	0	0	0
PSMA3	0	0	0	0	0	0	0	0
PSMA5	0	0	0	0	0	0	0	0
PSMA6	0	0	0	0	0	0	0	0
PSMA7	0	0	0	0	0	0	0	0
PSMB1	0	0	0	0	0	0	0	0
PSMB2	0	0	0	0	0	0	0	0
PSMB3	0	0	0	0	0	0	0	0
PSMB4	0	0	0	0	0	0	0	0
PSMB7	0	0	0	0	0	0	0	0
PSMC1	0	0	0	0	0	0	0	0
PSMC2	0	0	0	0	0	0	0	0
PSMC3	0	0	0	0	0	0	0	0
PSMC5	0	0	0	0	0	0	0	0
PSMC6	0	0	0	0	0	0	0	0
PSMD11	0	0	0	0	0	0	0	0
PSMD12	0	0	0	0	0	0	0	0
PSMD13	0	0	0	0	0	0	0	0

Table S6 The differential proteins contribute to the enrichment of the significant pathways for the public dataset

Core enrichment proteins in down-regulated pathways of hiPSCs/HFs (hiPSCs vs HFs)

NAME	REACTOME_GLUCOSE_METABOLISM	REACTOME_UNFOLDED_PROTEIN_RESPONSE	REACTOME_DIABETES_PATHWAYS	REACTOME_PROTEIN_FOLDING	REACTOME_PREFOLDIN_MEDIATED_TRANSFER_OF_SUBSTRATE_TO_CCT_TRIC	REACTOME_ASPARAGINE_N_LINKED_GLYCOSYLATION	REACTOME_POST_TRANSLATIONAL_PROTEIN_MODIFICATION	REACTOME_ACTIVATION_OF_THE_MRNA_UPON_BINDING_OF_THE_CAP_BINDING_COMPLEX_AND_EIFS_AND_SUBSEQUENT_BINDING_TO_43S	REACTOME_FORMATION_OF_THE_TERNARY_COMPLEX_AND_SUBSEQUENTLY_THE_43S_COMPLEX	REACTOME_METABOLISM_OF_PROTEINS	REACTOME_SRP_DEPENDENT_COTRANSLATIONAL_PROTEIN_TARGETING_TO_MEMBRANE	REACTOME_INFLUENZA_VIRAL_RNA_TRANSCRIPTION_AND_REPLICATION	REACTOME_NONSENSE_MEDIATED_DECAY_ENHANCED_BY_THE_EXON_JUNCTION_COMPLEX
PSMD14	0	0	0	0	0	0	0	0	0	0	0	0	0
PSMD2	0	0	0	0	0	0	0	0	0	0	0	0	0
PSMD3	0	0	0	0	0	0	0	0	0	0	0	0	0
PSMD4	0	0	0	0	0	0	0	0	0	0	0	0	0
PSMD6	0	0	0	0	0	0	0	0	0	0	0	0	0
PSMD7	0	0	0	0	0	0	0	0	0	0	0	0	0
PSME1	0	0	0	0	0	0	0	0	0	0	0	0	0
PSME2	0	0	0	0	0	0	0	0	0	0	0	0	0
PSMF1	0	0	0	0	0	0	0	0	0	0	0	0	0
PTPN1	0	0	0	0	0	0	0	0	0	0	0	0	0
PYGB	1	0	0	0	0	0	0	0	0	0	0	0	0
RAB5C	0	0	0	0	0	0	0	0	0	0	0	0	0
RAC1	0	0	0	0	0	0	0	0	0	0	0	0	0
RAP1A	0	0	0	0	0	0	0	0	0	0	0	0	0
RASA1	0	0	0	0	0	0	0	0	0	0	0	0	0
RDX	0	0	0	0	0	0	0	0	0	0	0	0	0
RELA	0	0	0	0	0	0	0	0	0	0	0	0	0
ROCK1	0	0	0	0	0	0	0	0	0	0	0	0	0
ROCK2	0	0	0	0	0	0	0	0	0	0	0	0	0
RPL10A	0	0	0	0	0	0	0	0	0	1	1	1	1
RPL11	0	0	0	0	0	0	0	0	0	1	1	1	1
RPL12	0	0	0	0	0	0	0	0	0	1	1	1	1
RPL13	0	0	0	0	0	0	0	0	0	1	1	1	1
RPL14	0	0	0	0	0	0	0	0	0	1	1	1	1
RPL17	0	0	0	0	0	0	0	0	0	1	1	1	1

NAME	REACTOME_PEPTIDE_CHAIN_ELONGATION	REACTOME_3_UTR_MEDIATED_TRANSLATIONAL_REGULATION	REACTOME_TRANSLATION	REACTOME_CELL_CELL_COMMUNICATION	REACTOME_GOLGI_ASSOCIATED_VESICLE_BIOGENESIS	REACTOME_TRANS_GOLGI_NETWORK_VESICLE_BUDDING	REACTOME_MEMBRANE_TRAFFICKING	REACTOME_TRANSMISSION_ACROSS_CHEMICAL_SYNAPSES	REACTOME_NEURONAL_SYSTEM	REACTOME_MHC_CLASS_II_ANTIGEN_PRESENTATION	REACTOME_INNATE_IMMUNE_SYSTEM	REACTOME_IMMUNE_SYSTEM	REACTOME_ADAPTIVE_IMMUNE_SYSTEM
PSMD14	0	0	0	0	0	0	0	0	0	0	0	1	1
PSMD2	0	0	0	0	0	0	0	0	0	0	0	1	1
PSMD3	0	0	0	0	0	0	0	0	0	0	0	1	1
PSMD4	0	0	0	0	0	0	0	0	0	0	0	1	1
PSMD6	0	0	0	0	0	0	0	0	0	0	0	1	1
PSMD7	0	0	0	0	0	0	0	0	0	0	0	1	1
PSME1	0	0	0	0	0	0	0	0	0	0	0	1	1
PSME2	0	0	0	0	0	0	0	0	0	0	0	1	1
PSMF1	0	0	0	0	0	0	0	0	0	0	0	1	1
PTPN1	0	0	0	0	0	0	0	0	0	0	0	1	0
PYGB	0	0	0	0	0	0	0	0	0	0	0	0	0
RAB5C	0	0	0	0	1	1	1	0	0	0	0	0	0
RAC1	0	0	0	0	0	0	0	0	0	0	0	1	1
RAP1A	0	0	0	0	0	0	0	0	0	0	0	1	1
RASA1	0	0	0	0	0	0	0	0	0	0	0	0	0
RDX	0	0	0	0	0	0	0	0	0	0	0	0	0
RELA	0	0	0	0	0	0	0	0	0	0	1	1	1
ROCK1	0	0	0	0	0	0	0	0	0	0	0	0	0
ROCK2	0	0	0	0	0	0	0	0	0	0	0	0	0
RPL10A	1	1	1	0	0	0	0	0	0	0	0	0	0
RPL11	1	1	1	0	0	0	0	0	0	0	0	0	0
RPL12	1	1	1	0	0	0	0	0	0	0	0	0	0
RPL13	1	1	1	0	0	0	0	0	0	0	0	0	0
RPL14	1	1	1	0	0	0	0	0	0	0	0	0	0
RPL17	1	1	1	0	0	0	0	0	0	0	0	0	0

NAME	REACTOME_CLASS_I_MHC_MEDIATED_ANTIGEN_PROCESSING_PRESENTATION	REACTOME_APOPTOSIS	REACTOME_ANTIGEN_PROCESSING_UBIQUITINATION_PROTEASOME_DEGRADATION	REACTOME_ING_BY_WNT	REACTOME_REGULATION_OF_MRNA_STABILITY_BY_PROTEINS_THAT_BIND_AU_RICH_ELEMENTS	REACTOME_DESTABILIZATION_OF_MRNA_BY_AUF1_HNRNP_D0	REACTOME_REGULATION_OF_APOPTOSIS	REACTOME_REGULATION_OF_ORNITHINE_DECARBOXYLASE_ODC	REACTOME_CROSS_PRESENTATION_OF_SOLUBLE_EXOGENOUS_ANTIGENS_ENDOSOMES	REACTOME_VIF_MEDIATED_DEGRADATION_OF_APOBEC3G	REACTOME_REGULATION_OF_MITOTIC_CELL_CYCLE	REACTOME_APC_C_CDC20_MEDIATED_DEGRADATION_OF_MITOTIC_PROTEINS	REACTOME_CDT1_ASSOCIATION_WITH_THE_CDC6_ORC_ORIGIN_COMPLEX
PSMD14	1	1	1	1	1	1	1	1	1	1	1	1	1
PSMD2	1	1	1	1	1	1	1	1	1	1	1	1	1
PSMD3	1	1	1	1	1	1	1	1	1	1	1	1	1
PSMD4	1	1	1	1	1	1	1	1	1	1	1	1	1
PSMD6	1	1	1	1	1	1	1	1	1	1	1	1	1
PSMD7	1	1	1	1	1	1	1	1	1	1	1	1	1
PSME1	1	1	1	1	1	1	1	1	1	1	1	1	1
PSME2	1	1	1	1	1	1	1	1	1	1	1	1	1
PSMF1	1	1	1	1	1	1	1	1	1	1	1	1	1
PTPN1	0	0	0	0	0	0	0	0	0	0	0	0	0
PYGB	0	0	0	0	0	0	0	0	0	0	0	0	0
RAB5C	0	0	0	0	0	0	0	0	0	0	0	0	0
RAC1	0	0	0	0	0	0	0	0	0	0	0	0	0
RAP1A	0	0	0	0	0	0	0	0	0	0	0	0	0
RASA1	0	0	0	0	0	0	0	0	0	0	0	0	0
RDX	0	0	0	0	0	0	0	0	0	0	0	0	0
RELA	0	0	0	0	0	0	0	0	0	0	0	0	0
ROCK1	0	1	0	0	0	0	0	0	0	0	0	0	0
ROCK2	0	0	0	0	0	0	0	0	0	0	0	0	0
RPL10A	0	0	0	0	0	0	0	0	0	0	0	0	0
RPL11	0	0	0	0	0	0	0	0	0	0	0	0	0
RPL12	0	0	0	0	0	0	0	0	0	0	0	0	0
RPL13	0	0	0	0	0	0	0	0	0	0	0	0	0
RPL14	0	0	0	0	0	0	0	0	0	0	0	0	0
RPL17	0	0	0	0	0	0	0	0	0	0	0	0	0

NAME	REACTOME_APC_C_CDH1_MEDIATED_DEGRADATION_OF_CDC20_AND_OTHER_APC_C_CDH1_TARGETED_PROTEINS_IN_LATE_MITOSIS_EARLY_G1	REACTOME_CYCLIN_E_ASSOCIATED_EVENTS_DURING_G1_S_TRANSITION_	REACTOME_SCFSKP2_MEDIATED_DEGRADATION_OF_P27_P21	REACTOME_AUTODEGRADATION_OF_CDH1_BY_CDH1_APC_C	REACTOME_AUTODEGRADATION_OF_THE_E3_UBIQUITIN_LIGASE_COP1	REACTOME_SCF_BETA_TRCP_MEDIATED_DEGRADATION_OF_EMI1	REACTOME_P53_INDEPENDENT_G1_S_DNA_DAMAGE_CHECKPOINT	REACTOME_CDK_MEDIATED_PHOSPHORYLATION_AND_REMOVAL_OF_CDC6	REACTOME_P53_DEPENDENT_G1_DNA_DAMAGE_RESPONSE	REACTOME_ACTIVATION_OF_NF_KAPPAB_IN_B_CELLS	REACTOME_DOWNSTREAM_ING_EVENTS_OF_B_CELL_RECEPTOR_BCR	REACTOME_ING_BY_THE_B_CELL_RECEPTOR_BCR	REACTOME_ANTIGEN_PROCESSING_CROSS_PRESENTATION
PSMD14	1	1	1	1	1	1	1	1	1	1	1	1	1
PSMD2	1	1	1	1	1	1	1	1	1	1	1	1	1
PSMD3	1	1	1	1	1	1	1	1	1	1	1	1	1
PSMD4	1	1	1	1	1	1	1	1	1	1	1	1	1
PSMD6	1	1	1	1	1	1	1	1	1	1	1	1	1
PSMD7	1	1	1	1	1	1	1	1	1	1	1	1	1
PSME1	1	1	1	1	1	1	1	1	1	1	1	1	1
PSME2	1	1	1	1	1	1	1	1	1	1	1	1	1
PSMF1	1	1	1	1	1	1	1	1	1	1	1	1	1
PTPN1	0	0	0	0	0	0	0	0	0	0	0	0	0
PYGB	0	0	0	0	0	0	0	0	0	0	0	0	0
RAB5C	0	0	0	0	0	0	0	0	0	0	0	0	0
RAC1	0	0	0	0	0	0	0	0	0	0	0	0	0
RAP1A	0	0	0	0	0	0	0	0	0	0	0	0	0
RASA1	0	0	0	0	0	0	0	0	0	0	0	0	0
RDX	0	0	0	0	0	0	0	0	0	0	0	0	0
RELA	0	0	0	0	0	0	0	0	0	1	1	1	0
ROCK1	0	0	0	0	0	0	0	0	0	0	0	0	0
ROCK2	0	0	0	0	0	0	0	0	0	0	0	0	0
RPL10A	0	0	0	0	0	0	0	0	0	0	0	0	0
RPL11	0	0	0	0	0	0	0	0	0	0	0	0	0
RPL12	0	0	0	0	0	0	0	0	0	0	0	0	0
RPL13	0	0	0	0	0	0	0	0	0	0	0	0	0
RPL14	0	0	0	0	0	0	0	0	0	0	0	0	0
RPL17	0	0	0	0	0	0	0	0	0	0	0	0	0

NAME	REACTOME_ER_PHAGOSOME_PATHWAY	REACTOME_ING_BY_INSULIN_RECEPTOR	REACTOME_CELL_SURFACE_INTERACTIONS_AT_THE_VASCULAR_WALL	REACTOME_INTEGRIN_CELL_SURFACE_INTERACTIONS	REACTOME_RESPONSE_TO_ELEVATED_PLATELET_CYTOSOLIC_CA2_	REACTOME_HEMOSTASIS	REACTOME_PLATELET_ACTIVATION_ING_AND_AGGREGATION	REACTOME_SEMAPHORIN_INTERACTIONS	REACTOME_L1CAM_INTERACTIONS	REACTOME_DEVELOPMENTAL_BIOLOGY	REACTOME_AXON_GUIDANCE	REACTOME_INTEGRATION_OF_ENERGY_METABOLISM	REACTOME_REGULATION_OF_INSULIN_SECRETION
PSMD14	1	0	0	0	0	0	0	0	0	0	0	0	0
PSMD2	1	0	0	0	0	0	0	0	0	0	0	0	0
PSMD3	1	0	0	0	0	0	0	0	0	0	0	0	0
PSMD4	1	0	0	0	0	0	0	0	0	0	0	0	0
PSMD6	1	0	0	0	0	0	0	0	0	0	0	0	0
PSMD7	1	0	0	0	0	0	0	0	0	0	0	0	0
PSME1	1	0	0	0	0	0	0	0	0	0	0	0	0
PSME2	1	0	0	0	0	0	0	0	0	0	0	0	0
PSMF1	1	0	0	0	0	0	0	0	0	0	0	0	0
PTPN1	0	0	0	0	0	0	1	0	0	0	0	0	0
PYGB	0	0	0	0	0	0	0	0	0	0	0	0	0
RAB5C	0	0	0	0	0	0	0	0	0	0	0	0	0
RAC1	0	0	0	0	0	0	1	1	1	1	0	0	0
RAP1A	0	0	0	0	0	1	1	0	0	0	0	1	1
RASA1	0	0	0	0	0	0	0	0	0	0	0	0	0
RDX	0	0	0	0	0	0	0	0	1	1	1	0	0
RELA	0	0	0	0	0	0	0	0	0	1	0	0	0
ROCK1	0	0	0	0	0	0	0	1	0	0	0	0	0
ROCK2	0	0	0	0	0	0	0	1	0	1	1	0	0
RPL10A	0	0	0	0	0	0	0	0	0	0	0	0	0
RPL11	0	0	0	0	0	0	0	0	0	0	0	0	0
RPL12	0	0	0	0	0	0	0	0	0	0	0	0	0
RPL13	0	0	0	0	0	0	0	0	0	0	0	0	0
RPL14	0	0	0	0	0	0	0	0	0	0	0	0	0
RPL17	0	0	0	0	0	0	0	0	0	0	0	0	0

NAME	REACTOME_ING_BY_ILS	REACTOME_GPCR_DOWNSTREAM_ING	REACTOME_ING_BY_GPCR	REACTOME_LING_BY_NGF	REACTOME_NGF_LING_VIA_TRKA_FROM_THE_PLASMA_MEMBRANE	REACTOME_ING_BY_EGFR_IN_CANCER	REACTOME_ING_BY_FGFR_IN_DISEASE	REACTOME_ING_BY_FGFR	REACTOME_ING_BY_ERBB2	REACTOME_DOWNSTREAM__TRANSDUCTION	REACTOME_ING_BY_PDGF	KEGG_RIBOSOME	KEGG_PROTEASOME
PSMD14	0	0	0	0	0	0	0	0	0	0	0	0	1
PSMD2	0	0	0	0	0	0	0	0	0	0	0	0	1
PSMD3	0	0	0	0	0	0	0	0	0	0	0	0	1
PSMD4	0	0	0	0	0	0	0	0	0	0	0	0	1
PSMD6	0	0	0	0	0	0	0	0	0	0	0	0	1
PSMD7	0	0	0	0	0	0	0	0	0	0	0	0	1
PSME1	0	0	0	0	0	0	0	0	0	0	0	0	1
PSME2	0	0	0	0	0	0	0	0	0	0	0	0	1
PSMF1	0	0	0	0	0	0	0	0	0	0	0	0	1
PTPN1	0	0	0	0	0	0	0	0	0	0	0	0	0
PYGB	0	0	0	0	0	0	0	0	0	0	0	0	0
RAB5C	0	0	0	0	0	0	0	0	0	0	0	0	0
RAC1	0	1	1	1	0	0	0	0	0	0	0	0	0
RAP1A	0	0	0	1	1	0	0	0	0	0	0	0	0
RASA1	0	0	0	0	0	0	0	0	0	1	1	0	0
RDX	0	0	0	0	0	0	0	0	0	0	0	0	0
RELA	1	0	0	1	0	0	0	0	0	0	0	0	0
ROCK1	0	0	0	0	0	0	0	0	0	0	0	0	0
ROCK2	0	1	1	0	0	0	0	0	0	0	0	0	0
RPL10A	0	0	0	0	0	0	0	0	0	0	0	1	0
RPL11	0	0	0	0	0	0	0	0	0	0	0	1	0
RPL12	0	0	0	0	0	0	0	0	0	0	0	1	0
RPL13	0	0	0	0	0	0	0	0	0	0	0	1	0
RPL14	0	0	0	0	0	0	0	0	0	0	0	1	0
RPL17	0	0	0	0	0	0	0	0	0	0	0	1	0

NAME	KEGG_PYRUVATE_METABOLISM	KEGG_LYSOSOME	KEGG_ENDOCYTOSIS	KEGG_EPITHELIAL_CELL_ING_IN_HELICOBACTER_PYLORI_INFECTION	KEGG_AXON_GUIDANCE	KEGG_NEUROTROPHIN_ING_PATHWAY	KEGG_CHEMOKINE_ING_PATHWAY	KEGG_INSULIN_ING_PATHWAY	KEGG_PROSTATE_CANCER	KEGG_PATHWAYS_IN_CANCER	KEGG_ERBB_ING_PATHWAY	KEGG_MAPK_ING_PATHWAY	KEGG_PATHOGENIC_ESCHERICHIA_COLI_INFECTION
PSMD14	0	0	0	0	0	0	0	0	0	0	0	0	0
PSMD2	0	0	0	0	0	0	0	0	0	0	0	0	0
PSMD3	0	0	0	0	0	0	0	0	0	0	0	0	0
PSMD4	0	0	0	0	0	0	0	0	0	0	0	0	0
PSMD6	0	0	0	0	0	0	0	0	0	0	0	0	0
PSMD7	0	0	0	0	0	0	0	0	0	0	0	0	0
PSME1	0	0	0	0	0	0	0	0	0	0	0	0	0
PSME2	0	0	0	0	0	0	0	0	0	0	0	0	0
PSMF1	0	0	0	0	0	0	0	0	0	0	0	0	0
PTPN1	0	0	0	0	0	0	0	1	0	0	0	0	0
PYGB	0	0	0	0	0	0	0	1	0	0	0	0	0
RAB5C	0	0	1	0	0	0	0	0	0	0	0	0	0
RAC1	0	0	0	1	1	1	1	0	0	1	0	1	0
RAP1A	0	0	0	0	0	1	1	0	0	0	0	1	0
RASA1	0	0	0	0	1	0	0	0	0	0	0	1	0
RDX	0	0	0	0	0	0	0	0	0	0	0	0	0
RELA	0	0	0	1	0	1	1	0	1	1	0	1	0
ROCK1	0	0	0	0	0	0	1	0	0	0	0	0	1
ROCK2	0	0	0	0	1	0	1	0	0	0	0	0	1
RPL10A	0	0	0	0	0	0	0	0	0	0	0	0	0
RPL11	0	0	0	0	0	0	0	0	0	0	0	0	0
RPL12	0	0	0	0	0	0	0	0	0	0	0	0	0
RPL13	0	0	0	0	0	0	0	0	0	0	0	0	0
RPL14	0	0	0	0	0	0	0	0	0	0	0	0	0
RPL17	0	0	0	0	0	0	0	0	0	0	0	0	0

NAME	KEGG_TIGHT_JUNCTION	KEGG_ADHERENS_JUNCTION	KEGG_LEUKOCYTE_TRANSENDOTHELIAL_MIGRATION	KEGG_ARRHYTHMOGENIC_RIGHT_VENTRICULAR_CARDIOMYOPATHY_ARVC	KEGG_ECM_RECEPTOR_INTERACTION	KEGG_FOCAL_ADHESION	KEGG_FC_GAMMA_R_MEDIATED_PHAGOCYTOSIS	KEGG_REGULATION_OF_ACTIN_CYTOSKELETON
PSMD14	0	0	0	0	0	0	0	0
PSMD2	0	0	0	0	0	0	0	0
PSMD3	0	0	0	0	0	0	0	0
PSMD4	0	0	0	0	0	0	0	0
PSMD6	0	0	0	0	0	0	0	0
PSMD7	0	0	0	0	0	0	0	0
PSME1	0	0	0	0	0	0	0	0
PSME2	0	0	0	0	0	0	0	0
PSMF1	0	0	0	0	0	0	0	0
PTPN1	0	1	0	0	0	0	0	0
PYGB	0	0	0	0	0	0	0	0
RAB5C	0	0	0	0	0	0	0	0
RAC1	0	1	1	0	0	0	1	1
RAP1A	0	0	1	0	0	1	0	0
RASA1	0	0	0	0	0	0	0	0
RDX	0	0	0	0	0	0	0	1
RELA	0	0	0	0	0	0	0	0
ROCK1	0	0	0	0	0	0	0	1
ROCK2	0	0	1	0	0	1	0	1
RPL10A	0	0	0	0	0	0	0	0
RPL11	0	0	0	0	0	0	0	0
RPL12	0	0	0	0	0	0	0	0
RPL13	0	0	0	0	0	0	0	0
RPL14	0	0	0	0	0	0	0	0
RPL17	0	0	0	0	0	0	0	0

Table S6 The differential proteins contribute to the enrichment of the significant pathways for the public dataset

Core enrichment proteins in down-regulated pathways of hiPSCs/HFs (hiPSCs vs HFs)

NAME	REACTOME_GLUCOSE_METABOLISM	REACTOME_UNFOLDED_PROTEIN_RESPONSE	REACTOME_DIABETES_PATHWAYS	REACTOME_PROTEIN_FOLDING	REACTOME_PREFOLDIN_MEDIATED_TRANSFER_OF_SUBSTRATE_TO_CCT_TRIC	REACTOME_ASPARAGINE_N_LINKED_GLYCOSYLATION	REACTOME_POST_TRANSLATIONAL_PROTEIN_MODIFICATION	REACTOME_ACTIVATION_OF_THE_MRNA_UPON_BINDING_OF_THE_CAP_BINDING_COMPLEX_AND_EIFS_AND_SUBSEQUENT_BINDING_TO_43S	REACTOME_FORMATION_OF_THE_TERNARY_COMPLEX_AND_SUBSEQUENTLY_THE_43S_COMPLEX	REACTOME_METABOLISM_OF_PROTEINS	REACTOME_SRP_DEPENDENT_COTRANSLATIONAL_PROTEIN_TARGETING_TO_MEMBRANE	REACTOME_INFLUENZA_VIRAL_RNA_TRANSCRIPTION_AND_REPLICATION	REACTOME_NONSENSE_MEDIATED_DECAY_ENHANCED_BY_THE_EXON_JUNCTION_COMPLEX
RPL18	0	0	0	0	0	0	0	0	0	1	1	1	1
RPL18A	0	0	0	0	0	0	0	0	0	1	1	1	1
RPL19	0	0	0	0	0	0	0	0	0	1	1	1	1
RPL23A	0	0	0	0	0	0	0	0	0	1	1	1	1
RPL24	0	0	0	0	0	0	0	0	0	1	1	1	1
RPL26	0	0	0	0	0	0	0	0	0	1	1	1	1
RPL27	0	0	0	0	0	0	0	0	0	1	1	1	1
RPL27A	0	0	0	0	0	0	0	0	0	1	1	1	1
RPL28	0	0	0	0	0	0	0	0	0	1	1	1	1
RPL29	0	0	0	0	0	0	0	0	0	1	1	1	1
RPL30	0	0	0	0	0	0	0	0	0	1	1	1	1
RPL32	0	0	0	0	0	0	0	0	0	1	1	1	1
RPL34	0	0	0	0	0	0	0	0	0	1	1	1	1
RPL35A	0	0	0	0	0	0	0	0	0	1	1	1	1
RPL36	0	0	0	0	0	0	0	0	0	1	1	1	1
RPL4	0	0	0	0	0	0	0	0	0	1	1	1	1
RPL7A	0	0	0	0	0	0	0	0	0	1	1	1	1
RPL8	0	0	0	0	0	0	0	0	0	1	1	1	1
RPLP0	0	0	0	0	0	0	0	0	0	1	1	1	1
RPLP1	0	0	0	0	0	0	0	0	0	1	1	1	1
RPLP2	0	0	0	0	0	0	0	0	0	1	1	1	1
RPS11	0	0	0	0	0	0	0	1	1	1	1	1	1
RPS12	0	0	0	0	0	0	0	1	1	1	1	1	1
RPS13	0	0	0	0	0	0	0	1	1	1	1	1	1
RPS15A	0	0	0	0	0	0	0	1	1	1	1	1	1

NAME	REACTOME_PEPTIDE_CHAIN_ELONGATION	REACTOME_3_UTR_MEDIATED_TRANSLATIONAL_REGULATION	REACTOME_TRANSLATION	REACTOME_CELL_CELL_COMMUNICATION	REACTOME_GOLGI_ASSOCIATED_VESICLE_BIOGENESIS	REACTOME_TRANS_GOLGI_NETWORK_VESICLE_BUDDING	REACTOME_MEMBRANE_TRAFFICKING	REACTOME_TRANSMISSION_ACROSS_CHEMICAL_SYNAPSES	REACTOME_NEURONAL_SYSTEM	REACTOME_MHC_CLASS_II_ANTIGEN_PRESENTATION	REACTOME_INNATE_IMMUNE_SYSTEM	REACTOME_IMMUNE_SYSTEM	REACTOME_ADAPTIVE_IMMUNE_SYSTEM
RPL18	1	1	1	0	0	0	0	0	0	0	0	0	0
RPL18A	1	1	1	0	0	0	0	0	0	0	0	0	0
RPL19	1	1	1	0	0	0	0	0	0	0	0	0	0
RPL23A	1	1	1	0	0	0	0	0	0	0	0	0	0
RPL24	1	1	1	0	0	0	0	0	0	0	0	0	0
RPL26	1	1	1	0	0	0	0	0	0	0	0	0	0
RPL27	1	1	1	0	0	0	0	0	0	0	0	0	0
RPL27A	1	1	1	0	0	0	0	0	0	0	0	0	0
RPL28	1	1	1	0	0	0	0	0	0	0	0	0	0
RPL29	1	1	1	0	0	0	0	0	0	0	0	0	0
RPL30	1	1	1	0	0	0	0	0	0	0	0	0	0
RPL32	1	1	1	0	0	0	0	0	0	0	0	0	0
RPL34	1	1	1	0	0	0	0	0	0	0	0	0	0
RPL35A	1	1	1	0	0	0	0	0	0	0	0	0	0
RPL36	1	1	1	0	0	0	0	0	0	0	0	0	0
RPL4	1	1	1	0	0	0	0	0	0	0	0	0	0
RPL7A	1	1	1	0	0	0	0	0	0	0	0	0	0
RPL8	1	1	1	0	0	0	0	0	0	0	0	0	0
RPLP0	1	1	1	0	0	0	0	0	0	0	0	0	0
RPLP1	1	1	1	0	0	0	0	0	0	0	0	0	0
RPLP2	1	1	1	0	0	0	0	0	0	0	0	0	0
RPS11	1	1	1	0	0	0	0	0	0	0	0	0	0
RPS12	1	1	1	0	0	0	0	0	0	0	0	0	0
RPS13	1	1	1	0	0	0	0	0	0	0	0	0	0
RPS15A	1	1	1	0	0	0	0	0	0	0	0	0	0

NAME	REACTOME_CLASS_I_MHC_MEDIATED_ANTIGEN_PROCESSING_PRESENTATION	REACTOME_APOPTOSIS	REACTOME_ANTIGEN_PROCESSING_UBIQUITINATION_PROTEASOME_DEGRADATION	REACTOME_ING_BY_WNT	REACTOME_REGULATION_OF_MRNA_STABILITY_BY_PROTEINS_THAT_BIND_AU_RICH_ELEMENTS	REACTOME_DESTABILIZATION_OF_MRNA_BY_AUF1_HNRNP_D0	REACTOME_REGULATION_OF_APOPTOSIS	REACTOME_REGULATION_OF_ORNITHINE_DECARBOXYLASE_ODC	REACTOME_CROSS_PRESENTATION_OF_SOLUBLE_EXOGENOUS_ANTIGENS_ENDOSOMES	REACTOME_VIF_MEDIATED_DEGRADATION_OF_APOBEC3G	REACTOME_REGULATION_OF_MITOTIC_CELL_CYCLE	REACTOME_APC_C_CDC20_MEDIATED_DEGRADATION_OF_MITOTIC_PROTEINS	REACTOME_CDT1_ASSOCIATION_WITH_THE_CDC6_ORC_ORIGIN_COMPLEX
RPL18	0	0	0	0	0	0	0	0	0	0	0	0	0
RPL18A	0	0	0	0	0	0	0	0	0	0	0	0	0
RPL19	0	0	0	0	0	0	0	0	0	0	0	0	0
RPL23A	0	0	0	0	0	0	0	0	0	0	0	0	0
RPL24	0	0	0	0	0	0	0	0	0	0	0	0	0
RPL26	0	0	0	0	0	0	0	0	0	0	0	0	0
RPL27	0	0	0	0	0	0	0	0	0	0	0	0	0
RPL27A	0	0	0	0	0	0	0	0	0	0	0	0	0
RPL28	0	0	0	0	0	0	0	0	0	0	0	0	0
RPL29	0	0	0	0	0	0	0	0	0	0	0	0	0
RPL30	0	0	0	0	0	0	0	0	0	0	0	0	0
RPL32	0	0	0	0	0	0	0	0	0	0	0	0	0
RPL34	0	0	0	0	0	0	0	0	0	0	0	0	0
RPL35A	0	0	0	0	0	0	0	0	0	0	0	0	0
RPL36	0	0	0	0	0	0	0	0	0	0	0	0	0
RPL4	0	0	0	0	0	0	0	0	0	0	0	0	0
RPL7A	0	0	0	0	0	0	0	0	0	0	0	0	0
RPL8	0	0	0	0	0	0	0	0	0	0	0	0	0
RPLP0	0	0	0	0	0	0	0	0	0	0	0	0	0
RPLP1	0	0	0	0	0	0	0	0	0	0	0	0	0
RPLP2	0	0	0	0	0	0	0	0	0	0	0	0	0
RPS11	0	0	0	0	0	0	0	0	0	0	0	0	0
RPS12	0	0	0	0	0	0	0	0	0	0	0	0	0
RPS13	0	0	0	0	0	0	0	0	0	0	0	0	0
RPS15A	0	0	0	0	0	0	0	0	0	0	0	0	0

NAME	REACTOME_APC_C_CDH1_MEDIATED_DEGRADATION_OF_CDC20_AND_OTHER_APC_C_CDH1_TARGETED_PROTEINS_IN_LATE_MITOSIS_EARLY_G1	REACTOME_CYCLIN_E_ASSOCIATED_EVENTS_DURING_G1_S_TRANSITION_	REACTOME_SCFSKP2_MEDIATED_DEGRADATION_OF_P27_P21	REACTOME_AUTODEGRADATION_OF_CDH1_BY_CDH1_APC_C	REACTOME_AUTODEGRADATION_OF_THE_E3_UBIQUITIN_LIGASE_COP1	REACTOME_SCF_BETA_TRCP_MEDIATED_DEGRADATION_OF_EMI1	REACTOME_P53_INDEPENDENT_G1_S_DNA_DAMAGE_CHECKPOINT	REACTOME_CDK_MEDIATED_PHOSPHORYLATION_AND_REMOVAL_OF_CDC6	REACTOME_P53_DEPENDENT_G1_DNA_DAMAGE_RESPONSE	REACTOME_ACTIVATION_OF_NF_KAPPAB_IN_B_CELLS	REACTOME_DOWNSTREAM_ING_EVENTS_OF_B_CELL_RECEPTOR_BCR	REACTOME_ING_BY_THE_B_CELL_RECEPTOR_BCR	REACTOME_ANTIGEN_PROCESSING_CROSS_PRESENTATION
RPL18	0	0	0	0	0	0	0	0	0	0	0	0	0
RPL18A	0	0	0	0	0	0	0	0	0	0	0	0	0
RPL19	0	0	0	0	0	0	0	0	0	0	0	0	0
RPL23A	0	0	0	0	0	0	0	0	0	0	0	0	0
RPL24	0	0	0	0	0	0	0	0	0	0	0	0	0
RPL26	0	0	0	0	0	0	0	0	0	0	0	0	0
RPL27	0	0	0	0	0	0	0	0	0	0	0	0	0
RPL27A	0	0	0	0	0	0	0	0	0	0	0	0	0
RPL28	0	0	0	0	0	0	0	0	0	0	0	0	0
RPL29	0	0	0	0	0	0	0	0	0	0	0	0	0
RPL30	0	0	0	0	0	0	0	0	0	0	0	0	0
RPL32	0	0	0	0	0	0	0	0	0	0	0	0	0
RPL34	0	0	0	0	0	0	0	0	0	0	0	0	0
RPL35A	0	0	0	0	0	0	0	0	0	0	0	0	0
RPL36	0	0	0	0	0	0	0	0	0	0	0	0	0
RPL4	0	0	0	0	0	0	0	0	0	0	0	0	0
RPL7A	0	0	0	0	0	0	0	0	0	0	0	0	0
RPL8	0	0	0	0	0	0	0	0	0	0	0	0	0
RPLP0	0	0	0	0	0	0	0	0	0	0	0	0	0
RPLP1	0	0	0	0	0	0	0	0	0	0	0	0	0
RPLP2	0	0	0	0	0	0	0	0	0	0	0	0	0
RPS11	0	0	0	0	0	0	0	0	0	0	0	0	0
RPS12	0	0	0	0	0	0	0	0	0	0	0	0	0
RPS13	0	0	0	0	0	0	0	0	0	0	0	0	0
RPS15A	0	0	0	0	0	0	0	0	0	0	0	0	0

NAME	REACTOME_ER_PHAGOSOME_PATHWAY	REACTOME_ING_BY_INSULIN_RECEPTOR	REACTOME_CELL_SURFACE_INTERACTIONS_AT_THE_VASCULAR_WALL	REACTOME_INTEGRIN_CELL_SURFACE_INTERACTIONS	REACTOME_RESPONSE_TO_ELEVATED_PLATELET_CYTOSOLIC_CA2_	REACTOME_HEMOSTASIS	REACTOME_PLATELET_ACTIVATION_ING_AND_AGGREGATION	REACTOME_SEMAPHORIN_INTERACTIONS	REACTOME_L1CAM_INTERACTIONS	REACTOME_DEVELOPMENTAL_BIOLOGY	REACTOME_AXON_GUIDANCE	REACTOME_INTEGRATION_OF_ENERGY_METABOLISM	REACTOME_REGULATION_OF_INSULIN_SECRETION
RPL18	0	0	0	0	0	0	0	0	0	0	0	0	0
RPL18A	0	0	0	0	0	0	0	0	0	0	0	0	0
RPL19	0	0	0	0	0	0	0	0	0	0	0	0	0
RPL23A	0	0	0	0	0	0	0	0	0	0	0	0	0
RPL24	0	0	0	0	0	0	0	0	0	0	0	0	0
RPL26	0	0	0	0	0	0	0	0	0	0	0	0	0
RPL27	0	0	0	0	0	0	0	0	0	0	0	0	0
RPL27A	0	0	0	0	0	0	0	0	0	0	0	0	0
RPL28	0	0	0	0	0	0	0	0	0	0	0	0	0
RPL29	0	0	0	0	0	0	0	0	0	0	0	0	0
RPL30	0	0	0	0	0	0	0	0	0	0	0	0	0
RPL32	0	0	0	0	0	0	0	0	0	0	0	0	0
RPL34	0	0	0	0	0	0	0	0	0	0	0	0	0
RPL35A	0	0	0	0	0	0	0	0	0	0	0	0	0
RPL36	0	0	0	0	0	0	0	0	0	0	0	0	0
RPL4	0	0	0	0	0	0	0	0	0	0	0	0	0
RPL7A	0	0	0	0	0	0	0	0	0	0	0	0	0
RPL8	0	0	0	0	0	0	0	0	0	0	0	0	0
RPLP0	0	0	0	0	0	0	0	0	0	0	0	0	0
RPLP1	0	0	0	0	0	0	0	0	0	0	0	0	0
RPLP2	0	0	0	0	0	0	0	0	0	0	0	0	0
RPS11	0	0	0	0	0	0	0	0	0	0	0	0	0
RPS12	0	0	0	0	0	0	0	0	0	0	0	0	0
RPS13	0	0	0	0	0	0	0	0	0	0	0	0	0
RPS15A	0	0	0	0	0	0	0	0	0	0	0	0	0

NAME	REACTOME_ING_BY_ILS	REACTOME_GPCR_DOWNSTREAM_ING	REACTOME_ING_BY_GPCR	REACTOME_LING_BY_NGF	REACTOME_NGF_LING_VIA_TRKA_FROM_THE_PLASMA_MEMBRANE	REACTOME_ING_BY_EGFR_IN_CANCER	REACTOME_ING_BY_FGFR_IN_DISEASE	REACTOME_ING_BY_FGFR	REACTOME_ING_BY_ERBB2	REACTOME_DOWNSTREAM__TRANSDUCTION	REACTOME_ING_BY_PDGF	KEGG_RIBOSOME	KEGG_PROTEASOME
RPL18	0	0	0	0	0	0	0	0	0	0	0	1	0
RPL18A	0	0	0	0	0	0	0	0	0	0	0	1	0
RPL19	0	0	0	0	0	0	0	0	0	0	0	1	0
RPL23A	0	0	0	0	0	0	0	0	0	0	0	1	0
RPL24	0	0	0	0	0	0	0	0	0	0	0	1	0
RPL26	0	0	0	0	0	0	0	0	0	0	0	1	0
RPL27	0	0	0	0	0	0	0	0	0	0	0	1	0
RPL27A	0	0	0	0	0	0	0	0	0	0	0	1	0
RPL28	0	0	0	0	0	0	0	0	0	0	0	1	0
RPL29	0	0	0	0	0	0	0	0	0	0	0	1	0
RPL30	0	0	0	0	0	0	0	0	0	0	0	1	0
RPL32	0	0	0	0	0	0	0	0	0	0	0	1	0
RPL34	0	0	0	0	0	0	0	0	0	0	0	1	0
RPL35A	0	0	0	0	0	0	0	0	0	0	0	1	0
RPL36	0	0	0	0	0	0	0	0	0	0	0	1	0
RPL4	0	0	0	0	0	0	0	0	0	0	0	1	0
RPL7A	0	0	0	0	0	0	0	0	0	0	0	1	0
RPL8	0	0	0	0	0	0	0	0	0	0	0	1	0
RPLP0	0	0	0	0	0	0	0	0	0	0	0	1	0
RPLP1	0	0	0	0	0	0	0	0	0	0	0	1	0
RPLP2	0	0	0	0	0	0	0	0	0	0	0	1	0
RPS11	0	0	0	0	0	0	0	0	0	0	0	1	0
RPS12	0	0	0	0	0	0	0	0	0	0	0	1	0
RPS13	0	0	0	0	0	0	0	0	0	0	0	1	0
RPS15A	0	0	0	0	0	0	0	0	0	0	0	1	0

NAME	KEGG_PYRUVATE_METABOLISM	KEGG_LYSOSOME	KEGG_ENDOCYTOSIS	KEGG_EPITHELIAL_CELL_ING_IN_HELICOBACTER_PYLORI_INFECTION	KEGG_AXON_GUIDANCE	KEGG_NEUROTROPHIN_ING_PATHWAY	KEGG_CHEMOKINE_ING_PATHWAY	KEGG_INSULIN_ING_PATHWAY	KEGG_PROSTATE_CANCER	KEGG_PATHWAYS_IN_CANCER	KEGG_ERBB_ING_PATHWAY	KEGG_MAPK_ING_PATHWAY	KEGG_PATHOGENIC_ESCHERICHIA_COLI_INFECTION
RPL18	0	0	0	0	0	0	0	0	0	0	0	0	0
RPL18A	0	0	0	0	0	0	0	0	0	0	0	0	0
RPL19	0	0	0	0	0	0	0	0	0	0	0	0	0
RPL23A	0	0	0	0	0	0	0	0	0	0	0	0	0
RPL24	0	0	0	0	0	0	0	0	0	0	0	0	0
RPL26	0	0	0	0	0	0	0	0	0	0	0	0	0
RPL27	0	0	0	0	0	0	0	0	0	0	0	0	0
RPL27A	0	0	0	0	0	0	0	0	0	0	0	0	0
RPL28	0	0	0	0	0	0	0	0	0	0	0	0	0
RPL29	0	0	0	0	0	0	0	0	0	0	0	0	0
RPL30	0	0	0	0	0	0	0	0	0	0	0	0	0
RPL32	0	0	0	0	0	0	0	0	0	0	0	0	0
RPL34	0	0	0	0	0	0	0	0	0	0	0	0	0
RPL35A	0	0	0	0	0	0	0	0	0	0	0	0	0
RPL36	0	0	0	0	0	0	0	0	0	0	0	0	0
RPL4	0	0	0	0	0	0	0	0	0	0	0	0	0
RPL7A	0	0	0	0	0	0	0	0	0	0	0	0	0
RPL8	0	0	0	0	0	0	0	0	0	0	0	0	0
RPLP0	0	0	0	0	0	0	0	0	0	0	0	0	0
RPLP1	0	0	0	0	0	0	0	0	0	0	0	0	0
RPLP2	0	0	0	0	0	0	0	0	0	0	0	0	0
RPS11	0	0	0	0	0	0	0	0	0	0	0	0	0
RPS12	0	0	0	0	0	0	0	0	0	0	0	0	0
RPS13	0	0	0	0	0	0	0	0	0	0	0	0	0
RPS15A	0	0	0	0	0	0	0	0	0	0	0	0	0

NAME	KEGG_TIGHT_JUNCTION	KEGG_ADHERENS_JUNCTION	KEGG_LEUKOCYTE_TRANSENDOTHELIAL_MIGRATION	KEGG_ARRHYTHMOGENIC_RIGHT_VENTRICULAR_CARDIOMYOPATHY_ARVC	KEGG_ECM_RECEPTOR_INTERACTION	KEGG_FOCAL_ADHESION	KEGG_FC_GAMMA_R_MEDIATED_PHAGOCYTOSIS	KEGG_REGULATION_OF_ACTIN_CYTOSKELETON
RPL18	0	0	0	0	0	0	0	0
RPL18A	0	0	0	0	0	0	0	0
RPL19	0	0	0	0	0	0	0	0
RPL23A	0	0	0	0	0	0	0	0
RPL24	0	0	0	0	0	0	0	0
RPL26	0	0	0	0	0	0	0	0
RPL27	0	0	0	0	0	0	0	0
RPL27A	0	0	0	0	0	0	0	0
RPL28	0	0	0	0	0	0	0	0
RPL29	0	0	0	0	0	0	0	0
RPL30	0	0	0	0	0	0	0	0
RPL32	0	0	0	0	0	0	0	0
RPL34	0	0	0	0	0	0	0	0
RPL35A	0	0	0	0	0	0	0	0
RPL36	0	0	0	0	0	0	0	0
RPL4	0	0	0	0	0	0	0	0
RPL7A	0	0	0	0	0	0	0	0
RPL8	0	0	0	0	0	0	0	0
RPLP0	0	0	0	0	0	0	0	0
RPLP1	0	0	0	0	0	0	0	0
RPLP2	0	0	0	0	0	0	0	0
RPS11	0	0	0	0	0	0	0	0
RPS12	0	0	0	0	0	0	0	0
RPS13	0	0	0	0	0	0	0	0
RPS15A	0	0	0	0	0	0	0	0

Table S6 The differential proteins contribute to the enrichment of the significant pathways for the public dataset

Core enrichment proteins in down-regulated pathways of hiPSCs/HFs (hiPSCs vs HFs)

NAME	REACTOME_GLUCOSE_METABOLISM	REACTOME_UNFOLDED_PROTEIN_RESPONSE	REACTOME_DIABETES_PATHWAYS	REACTOME_PROTEIN_FOLDING	REACTOME_PREFOLDIN_MEDIATED_TRANSFER_OF_SUBSTRATE_TO_CCT_TRIC	REACTOME_ASPARAGINE_N_LINKED_GLYCOSYLATION	REACTOME_POST_TRANSLATIONAL_PROTEIN_MODIFICATION	REACTOME_ACTIVATION_OF_THE_MRNA_UPON_BINDING_OF_THE_CAP_BINDING_COMPLEX_AND_EIFS_AND_SUBSEQUENT_BINDING_TO_43S	REACTOME_FORMATION_OF_THE_TERNARY_COMPLEX_AND_SUBSEQUENTLY_THE_43S_COMPLEX	REACTOME_METABOLISM_OF_PROTEINS	REACTOME_SRP_DEPENDENT_COTRANSLATIONAL_PROTEIN_TARGETING_TO_MEMBRANE	REACTOME_INFLUENZA_VIRAL_RNA_TRANSCRIPTION_AND_REPLICATION	REACTOME_NONSENSE_MEDIATED_DECAY_ENHANCED_BY_THE_EXON_JUNCTION_COMPLEX
RPS16	0	0	0	0	0	0	0	1	1	1	1	1	1
RPS17	0	0	0	0	0	0	0	1	1	1	1	1	1
RPS18	0	0	0	0	0	0	0	1	1	1	1	1	1
RPS19	0	0	0	0	0	0	0	1	1	1	1	1	1
RPS2	0	0	0	0	0	0	0	1	1	1	1	1	1
RPS21	0	0	0	0	0	0	0	1	1	1	1	1	1
RPS23	0	0	0	0	0	0	0	1	1	1	1	1	1
RPS24	0	0	0	0	0	0	0	1	1	1	1	1	1
RPS27A	0	0	0	0	0	0	0	1	1	1	1	1	1
RPS28	0	0	0	0	0	0	0	1	1	1	1	1	1
RPS29	0	0	0	0	0	0	0	1	1	1	1	1	1
RPS3	0	0	0	0	0	0	0	1	1	1	1	1	1
RPS4X	0	0	0	0	0	0	0	1	1	1	1	1	1
RPS5	0	0	0	0	0	0	0	1	1	1	1	1	1
RPS6	0	0	0	0	0	0	0	1	1	1	1	1	1
RPS7	0	0	0	0	0	0	0	1	1	1	1	1	1
RPS8	0	0	0	0	0	0	0	1	1	1	1	1	1
RPS9	0	0	0	0	0	0	0	1	1	1	1	1	1
RRAS	0	0	0	0	0	0	0	0	0	0	0	0	0
SHC1	0	1	1	0	0	0	0	0	0	0	0	0	0
SRGAP1	0	0	0	0	0	0	0	0	0	0	0	0	0
STAM	0	0	0	0	0	0	0	0	0	0	0	0	0
STAT1	0	0	0	0	0	0	0	0	0	0	0	0	0
STAT3	0	0	0	0	0	0	0	0	0	0	0	0	0
TCIRG1	0	0	0	0	0	0	0	0	0	0	0	0	0

NAME	REACTOME_PEPTIDE_CHAIN_ELONGATION	REACTOME_3_UTR_MEDIATED_TRANSLATIONAL_REGULATION	REACTOME_TRANSLATION	REACTOME_CELL_CELL_COMMUNICATION	REACTOME_GOLGI_ASSOCIATED_VESICLE_BIOGENESIS	REACTOME_TRANS_GOLGI_NETWORK_VESICLE_BUDDING	REACTOME_MEMBRANE_TRAFFICKING	REACTOME_TRANSMISSION_ACROSS_CHEMICAL_SYNAPSES	REACTOME_NEURONAL_SYSTEM	REACTOME_MHC_CLASS_II_ANTIGEN_PRESENTATION	REACTOME_INNATE_IMMUNE_SYSTEM	REACTOME_IMMUNE_SYSTEM	REACTOME_ADAPTIVE_IMMUNE_SYSTEM
RPS16	1	1	1	0	0	0	0	0	0	0	0	0	0
RPS17	1	1	1	0	0	0	0	0	0	0	0	0	0
RPS18	1	1	1	0	0	0	0	0	0	0	0	0	0
RPS19	1	1	1	0	0	0	0	0	0	0	0	0	0
RPS2	1	1	1	0	0	0	0	0	0	0	0	0	0
RPS21	1	1	1	0	0	0	0	0	0	0	0	0	0
RPS23	1	1	1	0	0	0	0	0	0	0	0	0	0
RPS24	1	1	1	0	0	0	0	0	0	0	0	0	0
RPS27A	1	1	1	0	0	0	1	0	0	0	1	1	1
RPS28	1	1	1	0	0	0	0	0	0	0	0	0	0
RPS29	1	1	1	0	0	0	0	0	0	0	0	0	0
RPS3	1	1	1	0	0	0	0	0	0	0	0	0	0
RPS4X	1	1	1	0	0	0	0	0	0	0	0	0	0
RPS5	1	1	1	0	0	0	0	0	0	0	0	0	0
RPS6	1	1	1	0	0	0	0	0	0	0	0	0	0
RPS7	1	1	1	0	0	0	0	0	0	0	0	0	0
RPS8	1	1	1	0	0	0	0	0	0	0	0	0	0
RPS9	1	1	1	0	0	0	0	0	0	0	0	0	0
RRAS	0	0	0	0	0	0	0	1	1	0	0	0	0
SHC1	0	0	0	0	0	0	0	0	0	0	0	1	1
SRGAP1	0	0	0	0	0	0	0	0	0	0	0	0	0
STAM	0	0	0	0	0	0	1	0	0	0	0	0	0
STAT1	0	0	0	0	0	0	0	0	0	0	0	1	0
STAT3	0	0	0	0	0	0	0	0	0	0	0	1	0
TCIRG1	0	0	0	0	0	0	0	0	0	0	0	0	0

NAME	REACTOME_CLASS_I_MHC_MEDIATED_ANTIGEN_PROCESSING_PRESENTATION	REACTOME_APOPTOSIS	REACTOME_ANTIGEN_PROCESSING_UBIQUITINATION_PROTEASOME_DEGRADATION	REACTOME_ING_BY_WNT	REACTOME_REGULATION_OF_MRNA_STABILITY_BY_PROTEINS_THAT_BIND_AU_RICH_ELEMENTS	REACTOME_DESTABILIZATION_OF_MRNA_BY_AUF1_HNRNP_D0	REACTOME_REGULATION_OF_APOPTOSIS	REACTOME_REGULATION_OF_ORNITHINE_DECARBOXYLASE_ODC	REACTOME_CROSS_PRESENTATION_OF_SOLUBLE_EXOGENOUS_ANTIGENS_ENDOSOMES	REACTOME_VIF_MEDIATED_DEGRADATION_OF_APOBEC3G	REACTOME_REGULATION_OF_MITOTIC_CELL_CYCLE	REACTOME_APC_C_CDC20_MEDIATED_DEGRADATION_OF_MITOTIC_PROTEINS	REACTOME_CDT1_ASSOCIATION_WITH_THE_CDC6_ORC_ORIGIN_COMPLEX
RPS16	0	0	0	0	0	0	0	0	0	0	0	0	0
RPS17	0	0	0	0	0	0	0	0	0	0	0	0	0
RPS18	0	0	0	0	0	0	0	0	0	0	0	0	0
RPS19	0	0	0	0	0	0	0	0	0	0	0	0	0
RPS2	0	0	0	0	0	0	0	0	0	0	0	0	0
RPS21	0	0	0	0	0	0	0	0	0	0	0	0	0
RPS23	0	0	0	0	0	0	0	0	0	0	0	0	0
RPS24	0	0	0	0	0	0	0	0	0	0	0	0	0
RPS27A	1	1	1	1	1	1	1	0	0	1	1	1	1
RPS28	0	0	0	0	0	0	0	0	0	0	0	0	0
RPS29	0	0	0	0	0	0	0	0	0	0	0	0	0
RPS3	0	0	0	0	0	0	0	0	0	0	0	0	0
RPS4X	0	0	0	0	0	0	0	0	0	0	0	0	0
RPS5	0	0	0	0	0	0	0	0	0	0	0	0	0
RPS6	0	0	0	0	0	0	0	0	0	0	0	0	0
RPS7	0	0	0	0	0	0	0	0	0	0	0	0	0
RPS8	0	0	0	0	0	0	0	0	0	0	0	0	0
RPS9	0	0	0	0	0	0	0	0	0	0	0	0	0
RRAS	0	0	0	0	0	0	0	0	0	0	0	0	0
SHC1	0	0	0	0	0	0	0	0	0	0	0	0	0
SRGAP1	0	0	0	0	0	0	0	0	0	0	0	0	0
STAM	0	0	0	0	0	0	0	0	0	0	0	0	0
STAT1	0	0	0	0	0	0	0	0	0	0	0	0	0
STAT3	0	0	0	0	0	0	0	0	0	0	0	0	0
TCIRG1	0	0	0	0	0	0	0	0	0	0	0	0	0

NAME	REACTOME_APC_C_CDH1_MEDIATED_DEGRADATION_OF_CDC20_AND_OTHER_APC_C_CDH1_TARGETED_PROTEINS_IN_LATE_MITOSIS_EARLY_G1	REACTOME_CYCLIN_E_ASSOCIATED_EVENTS_DURING_G1_S_TRANSITION_	REACTOME_SCFSKP2_MEDIATED_DEGRADATION_OF_P27_P21	REACTOME_AUTODEGRADATION_OF_CDH1_BY_CDH1_APC_C	REACTOME_AUTODEGRADATION_OF_THE_E3_UBIQUITIN_LIGASE_COP1	REACTOME_SCF_BETA_TRCP_MEDIATED_DEGRADATION_OF_EMI1	REACTOME_P53_INDEPENDENT_G1_S_DNA_DAMAGE_CHECKPOINT	REACTOME_CDK_MEDIATED_PHOSPHORYLATION_AND_REMOVAL_OF_CDC6	REACTOME_P53_DEPENDENT_G1_DNA_DAMAGE_RESPONSE	REACTOME_ACTIVATION_OF_NF_KAPPAB_IN_B_CELLS	REACTOME_DOWNSTREAM_ING_EVENTS_OF_B_CELL_RECEPTOR_BCR	REACTOME_ING_BY_THE_B_CELL_RECEPTOR_BCR	REACTOME_ANTIGEN_PROCESSING_CROSS_PRESENTATION
RPS16	0	0	0	0	0	0	0	0	0	0	0	0	0
RPS17	0	0	0	0	0	0	0	0	0	0	0	0	0
RPS18	0	0	0	0	0	0	0	0	0	0	0	0	0
RPS19	0	0	0	0	0	0	0	0	0	0	0	0	0
RPS2	0	0	0	0	0	0	0	0	0	0	0	0	0
RPS21	0	0	0	0	0	0	0	0	0	0	0	0	0
RPS23	0	0	0	0	0	0	0	0	0	0	0	0	0
RPS24	0	0	0	0	0	0	0	0	0	0	0	0	0
RPS27A	1	1	1	1	1	1	1	1	1	1	1	1	1
RPS28	0	0	0	0	0	0	0	0	0	0	0	0	0
RPS29	0	0	0	0	0	0	0	0	0	0	0	0	0
RPS3	0	0	0	0	0	0	0	0	0	0	0	0	0
RPS4X	0	0	0	0	0	0	0	0	0	0	0	0	0
RPS5	0	0	0	0	0	0	0	0	0	0	0	0	0
RPS6	0	0	0	0	0	0	0	0	0	0	0	0	0
RPS7	0	0	0	0	0	0	0	0	0	0	0	0	0
RPS8	0	0	0	0	0	0	0	0	0	0	0	0	0
RPS9	0	0	0	0	0	0	0	0	0	0	0	0	0
RRAS	0	0	0	0	0	0	0	0	0	0	0	0	0
SHC1	0	0	0	0	0	0	0	0	0	0	0	1	0
SRGAP1	0	0	0	0	0	0	0	0	0	0	0	0	0
STAM	0	0	0	0	0	0	0	0	0	0	0	0	0
STAT1	0	0	0	0	0	0	0	0	0	0	0	0	0
STAT3	0	0	0	0	0	0	0	0	0	0	0	0	0
TCIRG1	0	0	0	0	0	0	0	0	0	0	0	0	0

NAME	REACTOME_ER_PHAGOSOME_PATHWAY	REACTOME_ING_BY_INSULIN_RECEPTOR	REACTOME_CELL_SURFACE_INTERACTIONS_AT_THE_VASCULAR_WALL	REACTOME_INTEGRIN_CELL_SURFACE_INTERACTIONS	REACTOME_RESPONSE_TO_ELEVATED_PLATELET_CYTOSOLIC_CA2_	REACTOME_HEMOSTASIS	REACTOME_PLATELET_ACTIVATION_ING_AND_AGGREGATION	REACTOME_SEMAPHORIN_INTERACTIONS	REACTOME_L1CAM_INTERACTIONS	REACTOME_DEVELOPMENTAL_BIOLOGY	REACTOME_AXON_GUIDANCE	REACTOME_INTEGRATION_OF_ENERGY_METABOLISM	REACTOME_REGULATION_OF_INSULIN_SECRETION
RPS16	0	0	0	0	0	0	0	0	0	0	0	0	0
RPS17	0	0	0	0	0	0	0	0	0	0	0	0	0
RPS18	0	0	0	0	0	0	0	0	0	0	0	0	0
RPS19	0	0	0	0	0	0	0	0	0	0	0	0	0
RPS2	0	0	0	0	0	0	0	0	0	0	0	0	0
RPS21	0	0	0	0	0	0	0	0	0	0	0	0	0
RPS23	0	0	0	0	0	0	0	0	0	0	0	0	0
RPS24	0	0	0	0	0	0	0	0	0	0	0	0	0
RPS27A	1	0	0	0	0	0	0	0	0	0	0	0	0
RPS28	0	0	0	0	0	0	0	0	0	0	0	0	0
RPS29	0	0	0	0	0	0	0	0	0	0	0	0	0
RPS3	0	0	0	0	0	0	0	0	0	0	0	0	0
RPS4X	0	0	0	0	0	0	0	0	0	0	0	0	0
RPS5	0	0	0	0	0	0	0	0	0	0	0	0	0
RPS6	0	0	0	0	0	0	0	0	0	0	0	0	0
RPS7	0	0	0	0	0	0	0	0	0	0	0	0	0
RPS8	0	0	0	0	0	0	0	0	0	0	0	0	0
RPS9	0	0	0	0	0	0	0	0	0	0	0	0	0
RRAS	0	0	0	0	0	0	0	1	0	1	1	0	0
SHC1	0	1	0	0	0	1	1	0	0	0	0	0	0
SRGAP1	0	0	0	0	0	0	0	0	0	1	1	0	0
STAM	0	0	0	0	0	0	0	0	0	0	0	0	0
STAT1	0	0	0	0	0	0	0	0	0	0	0	0	0
STAT3	0	0	0	0	0	0	0	0	0	0	0	0	0
TCIRG1	0	1	0	0	0	0	0	0	0	0	0	0	0

NAME	REACTOME_ING_BY_ILS	REACTOME_GPCR_DOWNSTREAM_ING	REACTOME_ING_BY_GPCR	REACTOME_LING_BY_NGF	REACTOME_NGF_LING_VIA_TRKA_FROM_THE_PLASMA_MEMBRANE	REACTOME_ING_BY_EGFR_IN_CANCER	REACTOME_ING_BY_FGFR_IN_DISEASE	REACTOME_ING_BY_FGFR	REACTOME_ING_BY_ERBB2	REACTOME_DOWNSTREAM__TRANSDUCTION	REACTOME_ING_BY_PDGF	KEGG_RIBOSOME	KEGG_PROTEASOME
RPS16	0	0	0	0	0	0	0	0	0	0	0	1	0
RPS17	0	0	0	0	0	0	0	0	0	0	0	1	0
RPS18	0	0	0	0	0	0	0	0	0	0	0	1	0
RPS19	0	0	0	0	0	0	0	0	0	0	0	1	0
RPS2	0	0	0	0	0	0	0	0	0	0	0	1	0
RPS21	0	0	0	0	0	0	0	0	0	0	0	1	0
RPS23	0	0	0	0	0	0	0	0	0	0	0	1	0
RPS24	0	0	0	0	0	0	0	0	0	0	0	1	0
RPS27A	0	0	0	0	0	0	0	0	0	0	0	1	0
RPS28	0	0	0	0	0	0	0	0	0	0	0	1	0
RPS29	0	0	0	0	0	0	0	0	0	0	0	1	0
RPS3	0	0	0	0	0	0	0	0	0	0	0	1	0
RPS4X	0	0	0	0	0	0	0	0	0	0	0	1	0
RPS5	0	0	0	0	0	0	0	0	0	0	0	1	0
RPS6	0	0	0	0	0	0	0	0	0	0	0	1	0
RPS7	0	0	0	0	0	0	0	0	0	0	0	1	0
RPS8	0	0	0	0	0	0	0	0	0	0	0	1	0
RPS9	0	0	0	0	0	0	0	0	0	0	0	1	0
RRAS	0	0	0	0	0	0	0	0	0	0	0	0	0
SHC1	1	0	0	1	1	1	1	1	1	0	0	0	0
SRGAP1	0	0	0	0	0	0	0	0	0	0	0	0	0
STAM	0	0	0	0	0	1	0	0	0	0	0	0	0
STAT1	1	0	0	0	0	0	1	0	0	1	1	0	0
STAT3	1	0	0	0	1	0	0	0	0	0	0	0	0
TCIRG1	0	0	0	0	0	0	0	0	0	0	0	0	0

NAME	KEGG_PYRUVATE_METABOLISM	KEGG_LYSOSOME	KEGG_ENDOCYTOSIS	KEGG_EPITHELIAL_CELL_ING_IN_HELICOBACTER_PYLORI_INFECTION	KEGG_AXON_GUIDANCE	KEGG_NEUROTROPHIN_ING_PATHWAY	KEGG_CHEMOKINE_ING_PATHWAY	KEGG_INSULIN_ING_PATHWAY	KEGG_PROSTATE_CANCER	KEGG_PATHWAYS_IN_CANCER	KEGG_ERBB_ING_PATHWAY	KEGG_MAPK_ING_PATHWAY	KEGG_PATHOGENIC_ESCHERICHIA_COLI_INFECTION
RPS16	0	0	0	0	0	0	0	0	0	0	0	0	0
RPS17	0	0	0	0	0	0	0	0	0	0	0	0	0
RPS18	0	0	0	0	0	0	0	0	0	0	0	0	0
RPS19	0	0	0	0	0	0	0	0	0	0	0	0	0
RPS2	0	0	0	0	0	0	0	0	0	0	0	0	0
RPS21	0	0	0	0	0	0	0	0	0	0	0	0	0
RPS23	0	0	0	0	0	0	0	0	0	0	0	0	0
RPS24	0	0	0	0	0	0	0	0	0	0	0	0	0
RPS27A	0	0	0	0	0	0	0	0	0	0	0	0	0
RPS28	0	0	0	0	0	0	0	0	0	0	0	0	0
RPS29	0	0	0	0	0	0	0	0	0	0	0	0	0
RPS3	0	0	0	0	0	0	0	0	0	0	0	0	0
RPS4X	0	0	0	0	0	0	0	0	0	0	0	0	0
RPS5	0	0	0	0	0	0	0	0	0	0	0	0	0
RPS6	0	0	0	0	0	0	0	0	0	0	0	0	0
RPS7	0	0	0	0	0	0	0	0	0	0	0	0	0
RPS8	0	0	0	0	0	0	0	0	0	0	0	0	0
RPS9	0	0	0	0	0	0	0	0	0	0	0	0	0
RRAS	0	0	0	0	0	0	0	0	0	0	0	1	0
SHC1	0	0	0	0	0	1	1	1	0	0	1	0	0
SRGAP1	0	0	0	0	1	0	0	0	0	0	0	0	0
STAM	0	0	1	0	0	0	0	0	0	0	0	0	0
STAT1	0	0	0	0	0	0	1	0	0	1	0	0	0
STAT3	0	0	0	0	0	0	1	0	0	0	0	0	0
TCIRG1	0	1	0	1	0	0	0	0	0	0	0	0	0

NAME	KEGG_TIGHT_JUNCTION	KEGG_ADHERENS_JUNCTION	KEGG_LEUKOCYTE_TRANSENDOTHELIAL_MIGRATION	KEGG_ARRHYTHMOGENIC_RIGHT_VENTRICULAR_CARDIOMYOPATHY_ARVC	KEGG_ECM_RECEPTOR_INTERACTION	KEGG_FOCAL_ADHESION	KEGG_FC_GAMMA_R_MEDIATED_PHAGOCYTOSIS	KEGG_REGULATION_OF_ACTIN_CYTOSKELETON
RPS16	0	0	0	0	0	0	0	0
RPS17	0	0	0	0	0	0	0	0
RPS18	0	0	0	0	0	0	0	0
RPS19	0	0	0	0	0	0	0	0
RPS2	0	0	0	0	0	0	0	0
RPS21	0	0	0	0	0	0	0	0
RPS23	0	0	0	0	0	0	0	0
RPS24	0	0	0	0	0	0	0	0
RPS27A	0	0	0	0	0	0	0	0
RPS28	0	0	0	0	0	0	0	0
RPS29	0	0	0	0	0	0	0	0
RPS3	0	0	0	0	0	0	0	0
RPS4X	0	0	0	0	0	0	0	0
RPS5	0	0	0	0	0	0	0	0
RPS6	0	0	0	0	0	0	0	0
RPS7	0	0	0	0	0	0	0	0
RPS8	0	0	0	0	0	0	0	0
RPS9	0	0	0	0	0	0	0	0
RRAS	1	0	0	0	0	0	0	1
SHC1	0	0	0	0	0	0	0	0
SRGAP1	0	0	0	0	0	0	0	0
STAM	0	0	0	0	0	0	0	0
STAT1	0	0	0	0	0	0	0	0
STAT3	0	0	0	0	0	0	0	0
TCIRG1	0	0	0	0	0	0	0	0

Table S6 The differential proteins contribute to the enrichment of the significant pathways for the public dataset

Core enrichment proteins in down-regulated pathways of hiPSCs/HFs (hiPSCs vs HFs)

NAME	REACTOME_GLUCOSE_METABOLISM	REACTOME_UNFOLDED_PROTEIN_RESPONSE	REACTOME_DIABETES_PATHWAYS	REACTOME_PROTEIN_FOLDING	REACTOME_PREFOLDIN_MEDIATED_TRANSFER_OF_SUBSTRATE_TO_CCT_TRIC	REACTOME_ASPARAGINE_N_LINKED_GLYCOSYLATION	REACTOME_POST_TRANSLATIONAL_PROTEIN_MODIFICATION	REACTOME_ACTIVATION_OF_THE_MRNA_UPON_BINDING_OF_THE_CAP_BINDING_COMPLEX_AND_EIFS_AND_SUBSEQUENT_BINDING_TO_43S	REACTOME_FORMATION_OF_THE_TERNARY_COMPLEX_AND_SUBSEQUENTLY_THE_43S_COMPLEX	REACTOME_METABOLISM_OF_PROTEINS	REACTOME_SRP_DEPENDENT_COTRANSLATIONAL_PROTEIN_TARGETING_TO_MEMBRANE	REACTOME_INFLUENZA_VIRAL_RNA_TRANSCRIPTION_AND_REPLICATION	REACTOME_NONSENSE_MEDIATED_DECAY_ENHANCED_BY_THE_EXON_JUNCTION_COMPLEX
TFRC	0	0	0	0	0	0	0	0	0	0	0	0	0
THBS1	0	0	0	0	0	0	0	0	0	0	0	0	0
TLN1	0	1	1	0	0	0	0	0	0	0	0	0	0
TUBA1C	0	0	0	1	1	0	0	0	0	1	0	0	0
TUBB3	0	0	0	1	1	0	0	0	0	1	0	0	0
TUBB6	0	0	0	1	1	0	0	0	0	1	0	0	0
VASP	0	0	0	0	0	0	0	0	0	0	0	0	0
VCL	0	0	0	0	0	0	0	0	0	0	0	0	0
VPS4A	0	0	0	0	0	0	0	0	0	0	0	0	0
VPS4B	0	0	0	0	0	0	0	0	0	0	0	0	0
YWHAB	0	0	0	0	0	0	0	0	0	0	0	0	0
YWHAZ	0	0	0	0	0	0	0	0	0	0	0	0	0
ABCC4	0	0	0	0	0	0	0	0	0	0	0	0	0
ACTR1A	0	0	0	0	0	0	0	0	0	0	0	0	0
AKAP9	0	0	0	0	0	0	0	0	0	0	0	0	0
ALDOA	1	0	0	0	0	0	0	0	0	0	0	0	0
ALG5	0	0	0	0	0	1	1	0	0	1	0	0	0
ANXA1	0	0	0	0	0	0	0	0	0	0	0	0	0
ARCN1	0	0	0	0	0	0	0	0	0	0	0	0	0
ARHGEF17	0	0	0	0	0	0	0	0	0	0	0	0	0
B2M	0	0	0	0	0	0	0	0	0	0	0	0	0
BCAP31	0	0	0	0	0	0	0	0	0	0	0	0	0
C19orf10	0	1	1	0	0	0	0	0	0	0	0	0	0
CAB39	0	0	0	0	0	0	0	0	0	0	0	0	0
CALR	0	1	1	0	0	1	1	0	0	1	0	0	0

NAME	REACTOME_PEPTIDE_CHAIN_ELONGATION	REACTOME_3_UTR_MEDIATED_TRANSLATIONAL_REGULATION	REACTOME_TRANSLATION	REACTOME_CELL_CELL_COMMUNICATION	REACTOME_GOLGI_ASSOCIATED_VESICLE_BIOGENESIS	REACTOME_TRANS_GOLGI_NETWORK_VESICLE_BUDDING	REACTOME_MEMBRANE_TRAFFICKING	REACTOME_TRANSMISSION_ACROSS_CHEMICAL_SYNAPSES	REACTOME_NEURONAL_SYSTEM	REACTOME_MHC_CLASS_II_ANTIGEN_PRESENTATION	REACTOME_INNATE_IMMUNE_SYSTEM	REACTOME_IMMUNE_SYSTEM	REACTOME_ADAPTIVE_IMMUNE_SYSTEM
TFRC	0	0	0	0	1	1	1	0	0	0	0	0	0
THBS1	0	0	0	0	0	0	0	0	0	0	0	0	0
TLN1	0	0	0	0	0	0	0	0	0	0	0	0	0
TUBA1C	0	0	0	0	0	0	0	0	0	0	0	0	0
TUBB3	0	0	0	0	0	0	0	0	0	0	0	0	0
TUBB6	0	0	0	0	0	0	0	0	0	0	0	0	0
VASP	0	0	0	0	0	0	0	0	0	0	0	1	1
VCL	0	0	0	0	0	0	0	0	0	0	0	0	0
VPS4A	0	0	0	0	0	0	1	0	0	0	0	0	0
VPS4B	0	0	0	0	0	0	1	0	0	0	0	0	0
YWHAB	0	0	0	0	0	0	0	0	0	0	0	1	1
YWHAZ	0	0	0	0	0	0	0	0	0	0	0	1	1
ABCC4	0	0	0	0	0	0	0	0	0	0	0	0	0
ACTR1A	0	0	0	0	0	0	0	0	0	1	0	1	1
AKAP9	0	0	0	0	0	0	0	0	1	0	0	0	0
ALDOA	0	0	0	0	0	0	0	0	0	0	0	0	0
ALG5	0	0	0	0	0	0	0	0	0	0	0	0	0
ANXA1	0	0	0	0	0	0	0	0	0	0	0	0	0
ARCN1	0	0	0	0	0	0	1	0	0	0	0	0	0
ARHGEF17	0	0	0	0	0	0	0	0	0	0	0	0	0
B2M	0	0	0	0	0	0	0	0	0	0	0	1	1
BCAP31	0	0	0	0	0	0	0	0	0	0	0	0	0
C19orf10	0	0	0	0	0	0	0	0	0	0	0	0	0
CAB39	0	0	0	0	0	0	0	0	0	0	0	0	0
CALR	0	0	0	0	0	0	0	0	0	0	0	1	1

NAME	REACTOME_CLASS_I_MHC_MEDIATED_ANTIGEN_PROCESSING_PRESENTATION	REACTOME_APOPTOSIS	REACTOME_ANTIGEN_PROCESSING_UBIQUITINATION_PROTEASOME_DEGRADATION	REACTOME_ING_BY_WNT	REACTOME_REGULATION_OF_MRNA_STABILITY_BY_PROTEINS_THAT_BIND_AU_RICH_ELEMENTS	REACTOME_DESTABILIZATION_OF_MRNA_BY_AUF1_HNRNP_D0	REACTOME_REGULATION_OF_APOPTOSIS	REACTOME_REGULATION_OF_ORNITHINE_DECARBOXYLASE_ODC	REACTOME_CROSS_PRESENTATION_OF_SOLUBLE_EXOGENOUS_ANTIGENS_ENDOSOMES	REACTOME_VIF_MEDIATED_DEGRADATION_OF_APOBEC3G	REACTOME_REGULATION_OF_MITOTIC_CELL_CYCLE	REACTOME_APC_C_CDC20_MEDIATED_DEGRADATION_OF_MITOTIC_PROTEINS	REACTOME_CDT1_ASSOCIATION_WITH_THE_CDC6_ORC_ORIGIN_COMPLEX
TFRC	0	0	0	0	0	0	0	0	0	0	0	0	0
THBS1	0	0	0	0	0	0	0	0	0	0	0	0	0
TLN1	0	0	0	0	0	0	0	0	0	0	0	0	0
TUBA1C	0	0	0	0	0	0	0	0	0	0	0	0	0
TUBB3	0	0	0	0	0	0	0	0	0	0	0	0	0
TUBB6	0	0	0	0	0	0	0	0	0	0	0	0	0
VASP	0	0	0	0	0	0	0	0	0	0	0	0	0
VCL	0	0	0	0	0	0	0	0	0	0	0	0	0
VPS4A	0	0	0	0	0	0	0	0	0	0	0	0	0
VPS4B	0	0	0	0	0	0	0	0	0	0	0	0	0
YWHAB	0	1	0	0	1	0	0	0	0	0	0	0	0
YWHAZ	0	0	0	0	1	0	0	0	0	0	0	0	0
ABCC4	0	0	0	0	0	0	0	0	0	0	0	0	0
ACTR1A	0	0	0	0	0	0	0	0	0	0	0	0	0
AKAP9	0	0	0	0	0	0	0	0	0	0	0	0	0
ALDOA	0	0	0	0	0	0	0	0	0	0	0	0	0
ALG5	0	0	0	0	0	0	0	0	0	0	0	0	0
ANXA1	0	0	0	0	0	0	0	0	0	0	0	0	0
ARCN1	0	0	0	0	0	0	0	0	0	0	0	0	0
ARHGEF17	0	0	0	0	0	0	0	0	0	0	0	0	0
B2M	1	0	0	0	0	0	0	0	0	0	0	0	0
BCAP31	0	1	0	0	0	0	0	0	0	0	0	0	0
C19orf10	0	0	0	0	0	0	0	0	0	0	0	0	0
CAB39	0	0	0	0	0	0	0	0	0	0	0	0	0
CALR	1	0	0	0	0	0	0	0	0	0	0	0	0

NAME	REACTOME_APC_C_CDH1_MEDIATED_DEGRADATION_OF_CDC20_AND_OTHER_APC_C_CDH1_TARGETED_PROTEINS_IN_LATE_MITOSIS_EARLY_G1	REACTOME_CYCLIN_E_ASSOCIATED_EVENTS_DURING_G1_S_TRANSITION_	REACTOME_SCFSKP2_MEDIATED_DEGRADATION_OF_P27_P21	REACTOME_AUTODEGRADATION_OF_CDH1_BY_CDH1_APC_C	REACTOME_AUTODEGRADATION_OF_THE_E3_UBIQUITIN_LIGASE_COP1	REACTOME_SCF_BETA_TRCP_MEDIATED_DEGRADATION_OF_EMI1	REACTOME_P53_INDEPENDENT_G1_S_DNA_DAMAGE_CHECKPOINT	REACTOME_CDK_MEDIATED_PHOSPHORYLATION_AND_REMOVAL_OF_CDC6	REACTOME_P53_DEPENDENT_G1_DNA_DAMAGE_RESPONSE	REACTOME_ACTIVATION_OF_NF_KAPPAB_IN_B_CELLS	REACTOME_DOWNSTREAM_ING_EVENTS_OF_B_CELL_RECEPTOR_BCR	REACTOME_ING_BY_THE_B_CELL_RECEPTOR_BCR	REACTOME_ANTIGEN_PROCESSING_CROSS_PRESENTATION
TFRC	0	0	0	0	0	0	0	0	0	0	0	0	0
THBS1	0	0	0	0	0	0	0	0	0	0	0	0	0
TLN1	0	0	0	0	0	0	0	0	0	0	0	0	0
TUBA1C	0	0	0	0	0	0	0	0	0	0	0	0	0
TUBB3	0	0	0	0	0	0	0	0	0	0	0	0	0
TUBB6	0	0	0	0	0	0	0	0	0	0	0	0	0
VASP	0	0	0	0	0	0	0	0	0	0	0	0	0
VCL	0	0	0	0	0	0	0	0	0	0	0	0	0
VPS4A	0	0	0	0	0	0	0	0	0	0	0	0	0
VPS4B	0	0	0	0	0	0	0	0	0	0	0	0	0
YWHAB	0	0	0	0	0	0	0	0	0	0	0	0	0
YWHAZ	0	0	0	0	0	0	0	0	0	0	0	0	0
ABCC4	0	0	0	0	0	0	0	0	0	0	0	0	0
ACTR1A	0	0	0	0	0	0	0	0	0	0	0	0	0
AKAP9	0	0	0	0	0	0	0	0	0	0	0	0	0
ALDOA	0	0	0	0	0	0	0	0	0	0	0	0	0
ALG5	0	0	0	0	0	0	0	0	0	0	0	0	0
ANXA1	0	0	0	0	0	0	0	0	0	0	0	0	0
ARCN1	0	0	0	0	0	0	0	0	0	0	0	0	0
ARHGEF17	0	0	0	0	0	0	0	0	0	0	0	0	0
B2M	0	0	0	0	0	0	0	0	0	0	0	0	1
BCAP31	0	0	0	0	0	0	0	0	0	0	0	0	0
C19orf10	0	0	0	0	0	0	0	0	0	0	0	0	0
CAB39	0	0	0	0	0	0	0	0	0	0	0	0	0
CALR	0	0	0	0	0	0	0	0	0	0	0	0	1

NAME	REACTOME_ER_PHAGOSOME_PATHWAY	REACTOME_ING_BY_INSULIN_RECEPTOR	REACTOME_CELL_SURFACE_INTERACTIONS_AT_THE_VASCULAR_WALL	REACTOME_INTEGRIN_CELL_SURFACE_INTERACTIONS	REACTOME_RESPONSE_TO_ELEVATED_PLATELET_CYTOSOLIC_CA2_	REACTOME_HEMOSTASIS	REACTOME_PLATELET_ACTIVATION_ING_AND_AGGREGATION	REACTOME_SEMAPHORIN_INTERACTIONS	REACTOME_L1CAM_INTERACTIONS	REACTOME_DEVELOPMENTAL_BIOLOGY	REACTOME_AXON_GUIDANCE	REACTOME_INTEGRATION_OF_ENERGY_METABOLISM	REACTOME_REGULATION_OF_INSULIN_SECRETION
TFRC	0	0	0	0	0	0	0	0	0	0	0	0	0
THBS1	0	0	0	1	1	1	1	0	0	0	0	0	0
TLN1	0	0	0	1	1	1	1	1	0	1	1	0	0
TUBA1C	0	0	0	0	0	0	0	0	0	0	0	0	0
TUBB3	0	0	0	0	0	0	0	0	0	0	0	0	0
TUBB6	0	0	0	0	0	0	0	0	0	0	0	0	0
VASP	0	0	0	0	0	0	0	0	0	1	1	0	0
VCL	0	0	0	0	1	1	1	0	0	0	0	0	0
VPS4A	0	0	0	0	0	0	0	0	0	0	0	0	0
VPS4B	0	0	0	0	0	0	0	0	0	0	0	0	0
YWHAB	0	1	0	0	0	0	0	0	0	1	1	0	0
YWHAZ	0	0	0	0	0	0	1	0	0	0	0	0	0
ABCC4	0	0	0	0	1	1	1	0	0	0	0	0	0
ACTR1A	0	0	0	0	0	0	0	0	0	0	0	0	0
AKAP9	0	0	0	0	0	0	0	0	0	0	0	0	0
ALDOA	0	0	0	0	0	0	1	0	0	0	0	0	0
ALG5	0	0	0	0	0	0	0	0	0	0	0	0	0
ANXA1	0	0	0	0	0	0	0	0	0	0	0	0	0
ARCN1	0	0	0	0	0	0	0	0	0	0	0	0	0
ARHGEF17	0	0	0	0	0	0	0	0	0	0	0	0	0
B2M	1	0	0	0	0	0	0	0	0	0	0	0	0
BCAP31	0	0	0	0	0	0	0	0	0	0	0	0	0
C19orf10	0	0	0	0	0	0	0	0	0	0	0	0	0
CAB39	0	1	0	0	0	0	0	0	0	0	0	0	0
CALR	1	0	0	0	0	0	0	0	0	0	0	0	0

NAME	REACTOME_ING_BY_ILS	REACTOME_GPCR_DOWNSTREAM_ING	REACTOME_ING_BY_GPCR	REACTOME_LING_BY_NGF	REACTOME_NGF_LING_VIA_TRKA_FROM_THE_PLASMA_MEMBRANE	REACTOME_ING_BY_EGFR_IN_CANCER	REACTOME_ING_BY_FGFR_IN_DISEASE	REACTOME_ING_BY_FGFR	REACTOME_ING_BY_ERBB2	REACTOME_DOWNSTREAM__TRANSDUCTION	REACTOME_ING_BY_PDGF	KEGG_RIBOSOME	KEGG_PROTEASOME
TFRC	0	0	0	0	0	0	0	0	0	0	0	0	0
THBS1	0	0	0	0	0	0	0	0	0	0	1	0	0
TLN1	0	0	0	0	0	0	0	0	0	0	0	0	0
TUBA1C	0	0	0	0	0	0	0	0	0	0	0	0	0
TUBB3	0	0	0	0	0	0	0	0	0	0	0	0	0
TUBB6	0	0	0	0	0	0	0	0	0	0	0	0	0
VASP	0	0	0	0	0	0	0	0	0	0	0	0	0
VCL	0	0	0	0	0	0	0	0	0	0	0	0	0
VPS4A	0	0	0	0	0	0	0	0	0	0	0	0	0
VPS4B	0	0	0	0	0	0	0	0	0	0	0	0	0
YWHAB	1	0	1	1	1	1	1	1	1	1	1	0	0
YWHAZ	1	0	0	0	0	0	0	0	0	0	0	0	0
ABCC4	0	0	0	0	0	0	0	0	0	0	0	0	0
ACTR1A	0	0	0	0	0	0	0	0	0	0	0	0	0
AKAP9	0	0	0	0	0	0	0	0	0	0	0	0	0
ALDOA	0	0	0	0	0	0	0	0	0	0	0	0	0
ALG5	0	0	0	0	0	0	0	0	0	0	0	0	0
ANXA1	0	1	1	0	0	0	0	0	0	0	0	0	0
ARCN1	0	0	0	0	0	0	0	0	0	0	0	0	0
ARHGEF17	0	1	1	1	0	0	0	0	0	0	0	0	0
B2M	0	0	0	0	0	0	0	0	0	0	0	0	0
BCAP31	0	0	0	0	0	0	0	0	0	0	0	0	0
C19orf10	0	0	0	0	0	0	0	0	0	0	0	0	0
CAB39	0	0	0	0	0	0	0	0	0	0	0	0	0
CALR	0	0	0	0	0	0	0	0	0	0	0	0	0

NAME	KEGG_PYRUVATE_METABOLISM	KEGG_LYSOSOME	KEGG_ENDOCYTOSIS	KEGG_EPITHELIAL_CELL_ING_IN_HELICOBACTER_PYLORI_INFECTION	KEGG_AXON_GUIDANCE	KEGG_NEUROTROPHIN_ING_PATHWAY	KEGG_CHEMOKINE_ING_PATHWAY	KEGG_INSULIN_ING_PATHWAY	KEGG_PROSTATE_CANCER	KEGG_PATHWAYS_IN_CANCER	KEGG_ERBB_ING_PATHWAY	KEGG_MAPK_ING_PATHWAY	KEGG_PATHOGENIC_ESCHERICHIA_COLI_INFECTION
TFRC	0	0	1	0	0	0	0	0	0	0	0	0	0
THBS1	0	0	0	0	0	0	0	0	0	0	0	0	0
TLN1	0	0	0	0	0	0	0	0	0	0	0	0	0
TUBA1C	0	0	0	0	0	0	0	0	0	0	0	0	1
TUBB3	0	0	0	0	0	0	0	0	0	0	0	0	1
TUBB6	0	0	0	0	0	0	0	0	0	0	0	0	1
VASP	0	0	0	0	0	0	0	0	0	0	0	0	0
VCL	0	0	0	0	0	0	0	0	0	0	0	0	0
VPS4A	0	0	1	0	0	0	0	0	0	0	0	0	0
VPS4B	0	0	1	0	0	0	0	0	0	0	0	0	0
YWHAB	0	0	0	0	0	1	0	0	0	0	0	0	0
YWHAZ	0	0	0	0	0	1	0	0	0	0	0	0	1
ABCC4	0	0	0	0	0	1	0	0	0	0	0	0	0
ACTR1A	1	0	0	0	0	0	0	0	0	0	0	0	0
AKAP9	0	1	0	0	0	0	0	0	0	0	0	0	0
ALDOA	0	0	1	0	0	0	0	0	0	0	0	0	0
ALG5	0	0	1	0	0	0	0	0	0	0	0	0	0
ANXA1	0	0	0	0	0	0	0	0	0	0	0	0	1
ARCN1	0	0	0	0	0	0	0	0	0	0	0	0	1
ARHGEF17	0	0	0	0	0	0	0	0	0	0	0	0	1
B2M	0	0	0	0	0	0	0	0	0	0	0	0	1
BCAP31	0	0	0	0	0	0	0	0	0	0	0	0	1
C19orf10	0	0	0	0	0	0	0	0	0	0	0	0	1
CAB39	0	0	0	0	0	0	0	0	0	0	0	0	0
CALR	0	0	0	0	0	0	0	0	0	0	0	0	0

NAME	KEGG_TIGHT_JUNCTION	KEGG_ADHERENS_JUNCTION	KEGG_LEUKOCYTE_TRANSENDOTHELIAL_MIGRATION	KEGG_ARRHYTHMOGENIC_RIGHT_VENTRICULAR_CARDIOMYOPATHY_ARVC	KEGG_ECM_RECEPTOR_INTERACTION	KEGG_FOCAL_ADHESION	KEGG_FC_GAMMA_R_MEDIATED_PHAGOCYTOSIS	KEGG_REGULATION_OF_ACTIN_CYTOSKELETON
TFRC	0	0	0	0	0	0	0	0
THBS1	0	0	0	0	1	1	0	0
TLN1	0	0	0	0	0	1	0	0
TUBA1C	0	0	0	0	0	0	0	0
TUBB3	0	0	0	0	0	0	0	0
TUBB6	0	0	0	0	0	0	0	0
VASP	0	0	1	0	0	1	1	0
VCL	0	1	1	0	0	1	0	1
VPS4A	0	0	0	0	0	0	0	0
VPS4B	0	0	0	0	0	0	0	0
YWHAB	0	0	0	0	0	0	0	0
YWHAZ	0	0	0	0	0	0	0	0
ABCC4	0	0	0	0	0	0	0	0
ACTR1A	0	0	0	0	0	0	0	0
AKAP9	0	0	0	0	0	0	0	0
ALDOA	0	0	0	0	0	0	1	0
ALG5	0	0	0	0	0	0	0	0
ANXA1	0	0	0	0	0	0	1	1
ARCN1	0	0	0	0	0	0	1	1
ARHGEF17	0	0	0	0	0	0	1	1
B2M	0	0	0	0	0	0	1	1
BCAP31	0	0	0	0	0	0	1	1
C19orf10	0	0	0	0	0	0	0	1
CAB39	0	0	0	0	0	1	0	0
CALR	0	0	1	0	0	0	0	0

Table S6 The differential proteins contribute to the enrichment of the significant pathways for the public dataset

Core enrichment proteins in down-regulated pathways of hiPSCs/HFs (hiPSCs vs HFs)

NAME	REACTOME_GLUCOSE_METABOLISM	REACTOME_UNFOLDED_PROTEIN_RESPONSE	REACTOME_DIABETES_PATHWAYS	REACTOME_PROTEIN_FOLDING	REACTOME_PREFOLDIN_MEDIATED_TRANSFER_OF_SUBSTRATE_TO_CCT_TRIC	REACTOME_ASPARAGINE_N_LINKED_GLYCOSYLATION	REACTOME_POST_TRANSLATIONAL_PROTEIN_MODIFICATION	REACTOME_ACTIVATION_OF_THE_MRNA_UPON_BINDING_OF_THE_CAP_BINDING_COMPLEX_AND_EIFS_AND_SUBSEQUENT_BINDING_TO_43S	REACTOME_FORMATION_OF_THE_TERNARY_COMPLEX_AND_SUBSEQUENTLY_THE_43S_COMPLEX	REACTOME_METABOLISM_OF_PROTEINS	REACTOME_SRP_DEPENDENT_COTRANSLATIONAL_PROTEIN_TARGETING_TO_MEMBRANE	REACTOME_INFLUENZA_VIRAL_RNA_TRANSCRIPTION_AND_REPLICATION	REACTOME_NONSENSE_MEDIATED_DECAY_ENHANCED_BY_THE_EXON_JUNCTION_COMPLEX
CALU	0	0	0	0	0	0	0	0	0	0	0	0	0
CANX	0	0	0	0	0	1	0	0	0	1	0	0	0
CAP1	0	0	0	0	0	0	0	0	0	0	0	0	0
CAPZA1	0	0	0	0	0	0	0	0	0	0	0	0	0
CAPZA2	0	0	0	0	0	0	0	0	0	0	0	0	0
CASP6	0	0	0	0	0	0	0	0	0	0	0	0	0
CCT2	0	0	0	1	1	0	0	0	0	1	0	0	0
CCT3	0	0	0	1	1	0	0	0	0	1	0	0	0
CCT4	0	0	0	1	1	0	0	0	0	1	0	0	0
CCT5	0	0	0	1	1	0	0	0	0	1	0	0	0
CCT6A	0	0	0	1	1	0	0	0	0	1	0	0	0
CCT7	0	0	0	1	1	0	0	0	0	1	0	0	0
CCT8	0	0	0	1	1	0	0	0	0	1	0	0	0
CD151	0	0	0	0	0	0	0	0	0	0	0	0	0
CD81	0	0	0	0	0	0	0	0	0	0	0	0	0
CDC37	0	0	0	0	0	0	0	0	0	0	0	0	0
CDH2	0	0	0	0	0	0	0	0	0	0	0	0	0
CNTNAP1	0	0	0	0	0	0	0	0	0	0	0	0	0
COPA	0	0	0	0	0	0	0	0	0	0	0	0	0
COPB1	0	0	0	0	0	0	0	0	0	0	0	0	0
COPB2	0	0	0	0	0	0	0	0	0	0	0	0	0
COPE	0	0	0	0	0	0	0	0	0	0	0	0	0
COPG	0	0	0	0	0	0	0	0	0	0	0	0	0
COPZ1	0	0	0	0	0	0	0	0	0	0	0	0	0
DAD1	0	0	0	0	0	1	1	0	0	1	0	0	0

NAME	REACTOME_PEPTIDE_CHAIN_ELONGATION	REACTOME_3_UTR_MEDIATED_TRANSLATIONAL_REGULATION	REACTOME_TRANSLATION	REACTOME_CELL_CELL_COMMUNICATION	REACTOME_GOLGI_ASSOCIATED_VESICLE_BIOGENESIS	REACTOME_TRANS_GOLGI_NETWORK_VESICLE_BUDDING	REACTOME_MEMBRANE_TRAFFICKING	REACTOME_TRANSMISSION_ACROSS_CHEMICAL_SYNAPSES	REACTOME_NEURONAL_SYSTEM	REACTOME_MHC_CLASS_II_ANTIGEN_PRESENTATION	REACTOME_INNATE_IMMUNE_SYSTEM	REACTOME_IMMUNE_SYSTEM	REACTOME_ADAPTIVE_IMMUNE_SYSTEM
CALU	0	0	0	0	0	0	0	0	0	0	0	0	0
CANX	0	0	0	0	0	0	0	0	0	0	0	1	1
CAP1	0	0	0	0	0	0	0	0	0	0	0	0	0
CAPZA1	0	0	0	0	0	0	0	0	0	0	1	1	0
CAPZA2	0	0	0	0	0	0	0	0	0	0	1	1	0
CASP6	0	0	0	0	0	0	0	0	0	0	0	0	0
CCT2	0	0	0	0	0	0	0	0	0	0	0	0	0
CCT3	0	0	0	0	0	0	0	0	0	0	0	0	0
CCT4	0	0	0	0	0	0	0	0	0	0	0	0	0
CCT5	0	0	0	0	0	0	0	0	0	0	0	0	0
CCT6A	0	0	0	0	0	0	0	0	0	0	0	0	0
CCT7	0	0	0	0	0	0	0	0	0	0	0	0	0
CCT8	0	0	0	0	0	0	0	0	0	0	0	0	0
CD151	0	0	0	1	0	0	0	0	0	0	0	0	0
CD81	0	0	0	0	0	0	0	0	0	0	0	1	1
CDC37	0	0	0	0	0	0	0	0	0	0	0	0	0
CDH2	0	0	0	0	0	0	0	0	0	0	0	0	0
CNTNAP1	0	0	0	0	0	0	0	0	0	0	0	0	0
COPA	0	0	0	0	0	0	1	0	0	0	0	0	0
COPB1	0	0	0	0	0	0	1	0	0	0	0	0	0
COPB2	0	0	0	0	0	0	1	0	0	0	0	0	0
COPE	0	0	0	0	0	0	1	0	0	0	0	0	0
COPG	0	0	0	0	0	0	1	0	0	0	0	0	0
COPZ1	0	0	0	0	0	0	1	0	0	0	0	0	0
DAD1	0	0	0	0	0	0	0	0	0	0	0	0	0

NAME	REACTOME_CLASS_I_MHC_MEDIATED_ANTIGEN_PROCESSING_PRESENTATION	REACTOME_APOPTOSIS	REACTOME_ANTIGEN_PROCESSING_UBIQUITINATION_PROTEASOME_DEGRADATION	REACTOME_ING_BY_WNT	REACTOME_REGULATION_OF_MRNA_STABILITY_BY_PROTEINS_THAT_BIND_AU_RICH_ELEMENTS	REACTOME_DESTABILIZATION_OF_MRNA_BY_AUF1_HNRNP_D0	REACTOME_REGULATION_OF_APOPTOSIS	REACTOME_REGULATION_OF_ORNITHINE_DECARBOXYLASE_ODC	REACTOME_CROSS_PRESENTATION_OF_SOLUBLE_EXOGENOUS_ANTIGENS_ENDOSOMES	REACTOME_VIF_MEDIATED_DEGRADATION_OF_APOBEC3G	REACTOME_REGULATION_OF_MITOTIC_CELL_CYCLE	REACTOME_APC_C_CDC20_MEDIATED_DEGRADATION_OF_MITOTIC_PROTEINS	REACTOME_CDT1_ASSOCIATION_WITH_THE_CDC6_ORC_ORIGIN_COMPLEX
CALU	0	0	0	0	0	0	0	0	0	0	0	0	0
CANX	1	0	0	0	0	0	0	0	0	0	0	0	0
CAP1	0	0	0	0	0	0	0	0	0	0	0	0	0
CAPZA1	0	0	0	0	0	0	0	0	0	0	0	0	0
CAPZA2	0	0	0	0	0	0	0	0	0	0	0	0	0
CASP6	0	1	0	0	0	0	0	0	0	0	0	0	0
CCT2	0	0	0	0	0	0	0	0	0	0	0	0	0
CCT3	0	0	0	0	0	0	0	0	0	0	0	0	0
CCT4	0	0	0	0	0	0	0	0	0	0	0	0	0
CCT5	0	0	0	0	0	0	0	0	0	0	0	0	0
CCT6A	0	0	0	0	0	0	0	0	0	0	0	0	0
CCT7	0	0	0	0	0	0	0	0	0	0	0	0	0
CCT8	0	0	0	0	0	0	0	0	0	0	0	0	0
CD151	0	0	0	0	0	0	0	0	0	0	0	0	0
CD81	0	0	0	0	0	0	0	0	0	0	0	0	0
CDC37	0	0	0	0	0	0	0	0	0	0	0	0	0
CDH2	0	0	0	0	0	0	0	0	0	0	0	0	0
CNTNAP1	0	0	0	0	0	0	0	0	0	0	0	0	0
COPA	0	0	0	0	0	0	0	0	0	0	0	0	0
COPB1	0	0	0	0	0	0	0	0	0	0	0	0	0
COPB2	0	0	0	0	0	0	0	0	0	0	0	0	0
COPE	0	0	0	0	0	0	0	0	0	0	0	0	0
COPG	0	0	0	0	0	0	0	0	0	0	0	0	0
COPZ1	0	0	0	0	0	0	0	0	0	0	0	0	0
DAD1	0	0	0	0	0	0	0	0	0	0	0	0	0

NAME	REACTOME_APC_C_CDH1_MEDIATED_DEGRADATION_OF_CDC20_AND_OTHER_APC_C_CDH1_TARGETED_PROTEINS_IN_LATE_MITOSIS_EARLY_G1	REACTOME_CYCLIN_E_ASSOCIATED_EVENTS_DURING_G1_S_TRANSITION_	REACTOME_SCFSKP2_MEDIATED_DEGRADATION_OF_P27_P21	REACTOME_AUTODEGRADATION_OF_CDH1_BY_CDH1_APC_C	REACTOME_AUTODEGRADATION_OF_THE_E3_UBIQUITIN_LIGASE_COP1	REACTOME_SCF_BETA_TRCP_MEDIATED_DEGRADATION_OF_EMI1	REACTOME_P53_INDEPENDENT_G1_S_DNA_DAMAGE_CHECKPOINT	REACTOME_CDK_MEDIATED_PHOSPHORYLATION_AND_REMOVAL_OF_CDC6	REACTOME_P53_DEPENDENT_G1_DNA_DAMAGE_RESPONSE	REACTOME_ACTIVATION_OF_NF_KAPPAB_IN_B_CELLS	REACTOME_DOWNSTREAM_ING_EVENTS_OF_B_CELL_RECEPTOR_BCR	REACTOME_ING_BY_THE_B_CELL_RECEPTOR_BCR	REACTOME_ANTIGEN_PROCESSING_CROSS_PRESENTATION
CALU	0	0	0	0	0	0	0	0	0	0	0	0	0
CANX	0	0	0	0	0	0	0	0	0	0	0	0	0
CAP1	0	0	0	0	0	0	0	0	0	0	0	0	0
CAPZA1	0	0	0	0	0	0	0	0	0	0	0	0	0
CAPZA2	0	0	0	0	0	0	0	0	0	0	0	0	0
CASP6	0	0	0	0	0	0	0	0	0	0	0	0	0
CCT2	0	0	0	0	0	0	0	0	0	0	0	0	0
CCT3	0	0	0	0	0	0	0	0	0	0	0	0	0
CCT4	0	0	0	0	0	0	0	0	0	0	0	0	0
CCT5	0	0	0	0	0	0	0	0	0	0	0	0	0
CCT6A	0	0	0	0	0	0	0	0	0	0	0	0	0
CCT7	0	0	0	0	0	0	0	0	0	0	0	0	0
CCT8	0	0	0	0	0	0	0	0	0	0	0	0	0
CD151	0	0	0	0	0	0	0	0	0	0	0	0	0
CD81	0	0	0	0	0	0	0	0	0	0	0	0	0
CDC37	0	0	0	0	0	0	0	0	0	0	0	0	0
CDH2	0	0	0	0	0	0	0	0	0	0	0	0	0
CNTNAP1	0	0	0	0	0	0	0	0	0	0	0	0	0
COPA	0	0	0	0	0	0	0	0	0	0	0	0	0
COPB1	0	0	0	0	0	0	0	0	0	0	0	0	0
COPB2	0	0	0	0	0	0	0	0	0	0	0	0	0
COPE	0	0	0	0	0	0	0	0	0	0	0	0	0
COPG	0	0	0	0	0	0	0	0	0	0	0	0	0
COPZ1	0	0	0	0	0	0	0	0	0	0	0	0	0
DAD1	0	0	0	0	0	0	0	0	0	0	0	0	0

NAME	REACTOME_ER_PHAGOSOME_PATHWAY	REACTOME_ING_BY_INSULIN_RECEPTOR	REACTOME_CELL_SURFACE_INTERACTIONS_AT_THE_VASCULAR_WALL	REACTOME_INTEGRIN_CELL_SURFACE_INTERACTIONS	REACTOME_RESPONSE_TO_ELEVATED_PLATELET_CYTOSOLIC_CA2_	REACTOME_HEMOSTASIS	REACTOME_PLATELET_ACTIVATION_ING_AND_AGGREGATION	REACTOME_SEMAPHORIN_INTERACTIONS	REACTOME_L1CAM_INTERACTIONS	REACTOME_DEVELOPMENTAL_BIOLOGY	REACTOME_AXON_GUIDANCE	REACTOME_INTEGRATION_OF_ENERGY_METABOLISM	REACTOME_REGULATION_OF_INSULIN_SECRETION
CALU	0	0	0	0	1	1	1	0	0	0	0	0	0
CANX	0	0	0	0	0	0	0	0	0	0	0	0	0
CAP1	0	0	0	0	1	1	1	0	0	1	1	0	0
CAPZA1	0	0	0	0	0	1	0	0	0	0	0	0	0
CAPZA2	0	0	0	0	0	0	0	0	0	0	0	0	0
CASP6	0	0	0	0	0	0	0	0	0	0	0	0	0
CCT2	0	0	0	0	0	0	0	0	0	0	0	0	0
CCT3	0	0	0	0	0	0	0	0	0	0	0	0	0
CCT4	0	0	0	0	0	0	0	0	0	0	0	0	0
CCT5	0	0	0	0	0	0	0	0	0	0	0	0	0
CCT6A	0	0	0	0	0	0	0	0	0	0	0	0	0
CCT7	0	0	0	0	0	0	0	0	0	0	0	0	0
CCT8	0	0	0	0	0	0	0	0	0	0	0	0	0
CD151	0	0	0	0	0	0	0	0	0	0	0	0	0
CD81	0	0	0	0	0	0	0	0	0	0	0	0	0
CDC37	0	0	0	0	0	0	0	0	0	0	0	0	0
CDH2	0	0	0	0	0	0	0	0	0	1	0	0	0
CNTNAP1	0	0	0	0	0	0	0	0	1	1	1	0	0
COPA	0	0	0	0	0	0	0	0	0	0	0	0	0
COPB1	0	0	0	0	0	0	0	0	0	0	0	0	0
COPB2	0	0	0	0	0	0	0	0	0	0	0	0	0
COPE	0	0	0	0	0	0	0	0	0	0	0	0	0
COPG	0	0	0	0	0	0	0	0	0	0	0	0	0
COPZ1	0	0	0	0	0	0	0	0	0	0	0	0	0
DAD1	0	0	0	0	0	0	0	0	0	0	0	0	0

NAME	REACTOME_ING_BY_ILS	REACTOME_GPCR_DOWNSTREAM_ING	REACTOME_ING_BY_GPCR	REACTOME_LING_BY_NGF	REACTOME_NGF_LING_VIA_TRKA_FROM_THE_PLASMA_MEMBRANE	REACTOME_ING_BY_EGFR_IN_CANCER	REACTOME_ING_BY_FGFR_IN_DISEASE	REACTOME_ING_BY_FGFR	REACTOME_ING_BY_ERBB2	REACTOME_DOWNSTREAM__TRANSDUCTION	REACTOME_ING_BY_PDGF	KEGG_RIBOSOME	KEGG_PROTEASOME
CALU	0	0	0	0	0	0	0	0	0	0	0	0	0
CANX	0	0	0	0	0	0	0	0	0	0	0	0	0
CAP1	0	0	0	0	0	0	0	0	0	0	0	0	0
CAPZA1	0	0	0	0	0	0	0	0	0	0	0	0	0
CAPZA2	0	0	0	0	0	0	0	0	0	0	0	0	0
CASP6	0	0	0	0	0	0	0	0	0	0	0	0	0
CCT2	0	0	0	0	0	0	0	0	0	0	0	0	0
CCT3	0	0	0	0	0	0	0	0	0	0	0	0	0
CCT4	0	0	0	0	0	0	0	0	0	0	0	0	0
CCT5	0	0	0	0	0	0	0	0	0	0	0	0	0
CCT6A	0	0	0	0	0	0	0	0	0	0	0	0	0
CCT7	0	0	0	0	0	0	0	0	0	0	0	0	0
CCT8	0	0	0	0	0	0	0	0	0	0	0	0	0
CD151	0	0	0	0	0	0	0	0	0	0	0	0	0
CD81	0	0	0	0	0	0	0	0	0	0	0	0	0
CDC37	0	0	0	0	0	1	0	0	1	0	0	0	0
CDH2	0	0	0	0	0	0	0	0	0	0	0	0	0
CNTNAP1	0	0	0	0	0	0	0	0	0	0	0	0	0
COPA	0	0	0	0	0	0	0	0	0	0	0	0	0
COPB1	0	0	0	0	0	0	0	0	0	0	0	0	0
COPB2	0	0	0	0	0	0	0	0	0	0	0	0	0
COPE	0	0	0	0	0	0	0	0	0	0	0	0	0
COPG	0	0	0	0	0	0	0	0	0	0	0	0	0
COPZ1	0	0	0	0	0	0	0	0	0	0	0	0	0
DAD1	0	0	0	0	0	0	0	0	0	0	0	0	0

NAME	KEGG_PYRUVATE_METABOLISM	KEGG_LYSOSOME	KEGG_ENDOCYTOSIS	KEGG_EPITHELIAL_CELL_ING_IN_HELICOBACTER_PYLORI_INFECTION	KEGG_AXON_GUIDANCE	KEGG_NEUROTROPHIN_ING_PATHWAY	KEGG_CHEMOKINE_ING_PATHWAY	KEGG_INSULIN_ING_PATHWAY	KEGG_PROSTATE_CANCER	KEGG_PATHWAYS_IN_CANCER	KEGG_ERBB_ING_PATHWAY	KEGG_MAPK_ING_PATHWAY	KEGG_PATHOGENIC_ESCHERICHIA_COLI_INFECTION
CALU	0	0	0	0	1	0	0	0	0	0	0	0	0
CANX	0	0	0	0	0	0	0	0	0	0	0	0	1
CAP1	0	0	0	0	0	0	0	0	0	0	0	0	0
CAPZA1	0	0	1	0	0	0	0	0	0	0	0	0	0
CAPZA2	0	0	0	0	0	0	0	0	0	1	0	0	0
CASP6	0	0	1	0	0	0	0	0	0	0	0	0	0
CCT2	0	0	0	0	1	0	0	0	0	0	0	0	0
CCT3	0	0	1	0	0	0	0	0	0	0	0	0	0
CCT4	0	0	0	0	0	0	0	1	0	0	0	0	0
CCT5	0	0	0	0	0	0	0	0	0	0	0	1	0
CCT6A	1	0	0	0	0	0	0	0	0	0	0	0	0
CCT7	0	0	0	0	0	1	0	0	0	0	0	0	0
CCT8	1	0	0	0	0	0	0	0	0	0	0	0	0
CD151	0	1	0	0	0	0	0	0	0	0	0	0	0
CD81	0	1	0	0	0	0	0	0	0	0	0	0	0
CDC37	0	0	1	0	0	0	0	0	0	0	0	1	0
CDH2	0	0	0	0	0	0	0	0	0	0	0	0	0
CNTNAP1	0	0	1	0	0	0	0	0	0	0	0	0	0
COPA	0	0	0	0	0	0	0	0	0	1	0	0	0
COPB1	1	0	0	0	0	0	0	0	0	0	0	0	0
COPB2	0	0	0	0	0	0	0	0	0	0	0	0	0
COPE	1	0	0	0	0	0	0	0	0	0	0	0	0
COPG	0	0	0	0	0	0	0	0	0	0	0	0	0
COPZ1	0	0	1	0	0	0	0	0	0	0	0	0	0
DAD1	0	0	0	0	0	0	0	0	0	0	0	0	0

NAME	KEGG_TIGHT_JUNCTION	KEGG_ADHERENS_JUNCTION	KEGG_LEUKOCYTE_TRANSENDOTHELIAL_MIGRATION	KEGG_ARRHYTHMOGENIC_RIGHT_VENTRICULAR_CARDIOMYOPATHY_ARVC	KEGG_ECM_RECEPTOR_INTERACTION	KEGG_FOCAL_ADHESION	KEGG_FC_GAMMA_R_MEDIATED_PHAGOCYTOSIS	KEGG_REGULATION_OF_ACTIN_CYTOSKELETON
CALU	0	0	0	0	0	0	1	1
CANX	1	0	0	0	0	0	0	0
CAP1	0	0	0	0	0	0	0	1
CAPZA1	0	0	0	0	0	0	1	0
CAPZA2	0	0	0	0	0	0	0	0
CASP6	0	0	0	0	0	0	0	0
CCT2	0	0	0	0	0	0	0	0
CCT3	0	0	0	0	0	0	0	0
CCT4	0	0	0	0	0	0	0	0
CCT5	0	0	0	0	0	1	0	0
CCT6A	0	0	0	0	0	0	0	0
CCT7	0	0	0	0	0	0	0	0
CCT8	0	0	0	0	0	0	0	0
CD151	0	0	0	0	0	0	0	0
CD81	0	0	0	0	0	0	0	0
CDC37	0	0	0	0	0	0	0	0
CDH2	0	0	0	0	1	0	0	0
CNTNAP1	0	0	0	0	0	0	0	0
COPA	0	0	0	0	0	1	0	0
COPB1	0	0	0	0	0	0	0	0
COPB2	0	1	0	0	0	0	0	0
COPE	0	0	0	0	0	0	0	0
COPG	0	0	0	0	0	0	0	1
COPZ1	0	0	0	0	0	0	0	0
DAD1	0	0	0	0	0	0	0	1

Table S6 The differential proteins contribute to the enrichment of the significant pathways for the public dataset

Core enrichment proteins in down-regulated pathways of hiPSCs/HFs (hiPSCs vs HFs)

NAME	REACTOME_GLUCOSE_METABOLISM	REACTOME_UNFOLDED_PROTEIN_RESPONSE	REACTOME_DIABETES_PATHWAYS	REACTOME_PROTEIN_FOLDING	REACTOME_PREFOLDIN_MEDIATED_TRANSFER_OF_SUBSTRATE_TO_CCT_TRIC	REACTOME_ASPARAGINE_N_LINKED_GLYCOSYLATION	REACTOME_POST_TRANSLATIONAL_PROTEIN_MODIFICATION	REACTOME_ACTIVATION_OF_THE_MRNA_UPON_BINDING_OF_THE_CAP_BINDING_COMPLEX_AND_EIFS_AND_SUBSEQUENT_BINDING_TO_43S	REACTOME_FORMATION_OF_THE_TERNARY_COMPLEX_AND_SUBSEQUENTLY_THE_43S_COMPLEX	REACTOME_METABOLISM_OF_PROTEINS	REACTOME_SRP_DEPENDENT_COTRANSLATIONAL_PROTEIN_TARGETING_TO_MEMBRANE	REACTOME_INFLUENZA_VIRAL_RNA_TRANSCRIPTION_AND_REPLICATION	REACTOME_NONSENSE_MEDIATED_DECAY_ENHANCED_BY_THE_EXON_JUNCTION_COMPLEX
DBNL	0	0	0	0	0	0	0	0	0	0	0	0	0
DCTN1	0	1	1	0	0	0	0	0	0	0	0	0	0
DCTN2	0	0	0	0	0	0	0	0	0	0	0	0	0
DDOST	0	0	0	0	0	1	0	0	0	1	1	0	0
DDX58	0	0	0	0	0	0	0	0	0	0	0	0	0
DEFA1	0	0	0	0	0	0	0	0	0	0	0	0	0
DNAJB11	0	1	1	0	0	0	0	0	0	0	0	0	0
DNAJC3	0	1	1	0	0	0	0	0	0	0	0	1	0
DPM1	0	0	0	0	0	1	0	0	0	1	0	0	0
DPP4	0	0	0	0	0	0	0	0	0	0	0	0	0
DPYSL2	0	0	0	0	0	0	0	0	0	0	0	0	0
DYNC1H1	0	0	0	0	0	0	0	0	0	0	0	0	0
EEF1A1	0	0	0	0	0	0	0	0	0	1	0	0	0
EEF1D	0	0	0	0	0	0	0	0	0	1	0	0	0
EEF1G	0	0	0	0	0	0	0	0	0	1	0	0	0
EEF2	0	0	0	0	0	0	0	0	0	1	0	0	0
EIF2B1	0	0	0	0	0	0	0	0	0	1	0	0	0
EIF2B2	0	0	0	0	0	0	0	0	0	1	0	0	0
EIF2B5	0	0	0	0	0	0	0	0	0	1	0	0	0
EIF2S1	0	0	0	0	0	0	0	1	1	1	0	0	0
EIF2S2	0	0	0	0	0	0	0	1	1	1	0	0	0
EIF2S3	0	0	0	0	0	0	0	1	1	1	0	0	0
EIF3A	0	0	0	0	0	0	0	1	1	1	0	0	0
EIF3B	0	0	0	0	0	0	0	1	1	1	0	0	0
EIF3D	0	0	0	0	0	0	0	1	1	1	0	0	0

NAME	REACTOME_PEPTIDE_CHAIN_ELONGATION	REACTOME_3_UTR_MEDIATED_TRANSLATIONAL_REGULATION	REACTOME_TRANSLATION	REACTOME_CELL_CELL_COMMUNICATION	REACTOME_GOLGI_ASSOCIATED_VESICLE_BIOGENESIS	REACTOME_TRANS_GOLGI_NETWORK_VESICLE_BUDDING	REACTOME_MEMBRANE_TRAFFICKING	REACTOME_TRANSMISSION_ACROSS_CHEMICAL_SYNAPSES	REACTOME_NEURONAL_SYSTEM	REACTOME_MHC_CLASS_II_ANTIGEN_PRESENTATION	REACTOME_INNATE_IMMUNE_SYSTEM	REACTOME_IMMUNE_SYSTEM	REACTOME_ADAPTIVE_IMMUNE_SYSTEM
DBNL	0	0	0	0	0	0	0	0	0	0	0	0	0
DCTN1	0	0	0	0	0	0	0	0	0	1	0	1	1
DCTN2	0	0	0	0	0	0	0	0	0	1	0	1	1
DDOST	0	0	1	0	0	0	0	0	0	0	0	0	0
DDX58	0	0	0	0	0	0	0	0	0	0	1	1	0
DEFA1	0	0	0	0	0	0	0	0	0	0	1	1	0
DNAJB11	0	0	0	0	0	0	0	0	0	0	0	0	0
DNAJC3	0	0	0	0	0	0	0	0	0	0	0	0	0
DPM1	0	0	0	0	0	0	0	0	0	0	0	0	0
DPP4	0	0	0	0	0	0	0	0	0	0	0	0	0
DPYSL2	0	0	0	0	0	0	0	0	0	0	0	0	0
DYNC1H1	0	0	0	0	0	0	0	0	0	1	0	1	1
EEF1A1	1	0	1	0	0	0	0	0	0	0	0	0	0
EEF1D	0	0	1	0	0	0	0	0	0	0	0	0	0
EEF1G	0	0	1	0	0	0	0	0	0	0	0	0	0
EEF2	1	0	1	0	0	0	0	0	0	0	0	0	0
EIF2B1	0	0	1	0	0	0	0	0	0	0	0	0	0
EIF2B2	0	0	1	0	0	0	0	0	0	0	0	0	0
EIF2B5	0	0	1	0	0	0	0	0	0	0	0	0	0
EIF2S1	0	1	1	0	0	0	0	0	0	0	0	0	0
EIF2S2	0	1	1	0	0	0	0	0	0	0	0	0	0
EIF2S3	0	1	1	0	0	0	0	0	0	0	0	0	0
EIF3A	0	1	1	0	0	0	0	0	0	0	0	0	0
EIF3B	0	1	1	0	0	0	0	0	0	0	0	0	0
EIF3D	0	1	1	0	0	0	0	0	0	0	0	0	0

NAME	REACTOME_CLASS_I_MHC_MEDIATED_ANTIGEN_PROCESSING_PRESENTATION	REACTOME_APOPTOSIS	REACTOME_ANTIGEN_PROCESSING_UBIQUITINATION_PROTEASOME_DEGRADATION	REACTOME_ING_BY_WNT	REACTOME_REGULATION_OF_MRNA_STABILITY_BY_PROTEINS_THAT_BIND_AU_RICH_ELEMENTS	REACTOME_DESTABILIZATION_OF_MRNA_BY_AUF1_HNRNP_D0	REACTOME_REGULATION_OF_APOPTOSIS	REACTOME_REGULATION_OF_ORNITHINE_DECARBOXYLASE_ODC	REACTOME_CROSS_PRESENTATION_OF_SOLUBLE_EXOGENOUS_ANTIGENS_ENDOSOMES	REACTOME_VIF_MEDIATED_DEGRADATION_OF_APOBEC3G	REACTOME_REGULATION_OF_MITOTIC_CELL_CYCLE	REACTOME_APC_C_CDC20_MEDIATED_DEGRADATION_OF_MITOTIC_PROTEINS	REACTOME_CDT1_ASSOCIATION_WITH_THE_CDC6_ORC_ORIGIN_COMPLEX
DBNL	0	1	0	0	0	0	0	0	0	0	0	0	0
DCTN1	0	0	0	0	0	0	0	0	0	0	0	0	0
DCTN2	0	0	0	0	0	0	0	0	0	0	0	0	0
DDOST	0	0	0	0	0	0	0	0	0	0	0	0	0
DDX58	0	0	0	0	0	0	0	0	0	0	0	0	0
DEFA1	0	0	0	0	0	0	0	0	0	0	0	0	0
DNAJB11	0	0	0	0	0	0	0	0	0	0	0	0	0
DNAJC3	0	0	0	0	0	0	0	0	0	0	0	0	0
DPM1	0	0	0	0	0	0	0	0	0	0	0	0	0
DPP4	0	0	0	0	0	0	0	0	0	0	0	0	0
DPYSL2	0	0	0	0	0	0	0	0	0	0	0	0	0
DYNC1H1	0	0	0	0	0	0	0	0	0	0	0	0	0
EEF1A1	0	0	0	0	0	0	0	0	0	0	0	0	0
EEF1D	0	0	0	0	0	0	0	0	0	0	0	0	0
EEF1G	0	0	0	0	0	0	0	0	0	0	0	0	0
EEF2	0	0	0	0	0	0	0	0	0	0	0	0	0
EIF2B1	0	0	0	0	0	0	0	0	0	0	0	0	0
EIF2B2	0	0	0	0	0	0	0	0	0	0	0	0	0
EIF2B5	0	0	0	0	0	0	0	0	0	0	0	0	0
EIF2S1	0	0	0	0	0	0	0	0	0	0	0	0	0
EIF2S2	0	0	0	0	0	0	0	0	0	0	0	0	0
EIF2S3	0	0	0	0	0	0	0	0	0	0	0	0	0
EIF3A	0	0	0	0	0	0	0	0	0	0	0	0	0
EIF3B	0	0	0	0	0	0	0	0	0	0	0	0	0
EIF3D	0	0	0	0	0	0	0	0	0	0	0	0	0

NAME	REACTOME_APC_C_CDH1_MEDIATED_DEGRADATION_OF_CDC20_AND_OTHER_APC_C_CDH1_TARGETED_PROTEINS_IN_LATE_MITOSIS_EARLY_G1	REACTOME_CYCLIN_E_ASSOCIATED_EVENTS_DURING_G1_S_TRANSITION_	REACTOME_SCFSKP2_MEDIATED_DEGRADATION_OF_P27_P21	REACTOME_AUTODEGRADATION_OF_CDH1_BY_CDH1_APC_C	REACTOME_AUTODEGRADATION_OF_THE_E3_UBIQUITIN_LIGASE_COP1	REACTOME_SCF_BETA_TRCP_MEDIATED_DEGRADATION_OF_EMI1	REACTOME_P53_INDEPENDENT_G1_S_DNA_DAMAGE_CHECKPOINT	REACTOME_CDK_MEDIATED_PHOSPHORYLATION_AND_REMOVAL_OF_CDC6	REACTOME_P53_DEPENDENT_G1_DNA_DAMAGE_RESPONSE	REACTOME_ACTIVATION_OF_NF_KAPPAB_IN_B_CELLS	REACTOME_DOWNSTREAM_ING_EVENTS_OF_B_CELL_RECEPTOR_BCR	REACTOME_ING_BY_THE_B_CELL_RECEPTOR_BCR	REACTOME_ANTIGEN_PROCESSING_CROSS_PRESENTATION
DBNL	0	0	0	0	0	0	0	0	0	0	0	0	0
DCTN1	0	0	0	0	0	0	0	0	0	0	0	0	0
DCTN2	0	0	0	0	0	0	0	0	0	0	0	0	0
DDOST	0	0	0	0	0	0	0	0	0	0	0	0	0
DDX58	0	0	0	0	0	0	0	0	0	0	0	0	0
DEFA1	0	0	0	0	0	0	0	0	0	0	0	0	0
DNAJB11	0	0	0	0	0	0	0	0	0	0	0	0	0
DNAJC3	0	0	0	0	0	0	0	0	0	0	0	0	0
DPM1	0	0	0	0	0	0	0	0	0	0	0	0	0
DPP4	0	0	0	0	0	0	0	0	0	0	0	0	0
DPYSL2	0	0	0	0	0	0	0	0	0	0	0	0	0
DYNC1H1	0	0	0	0	0	0	0	0	0	0	0	0	0
EEF1A1	0	0	0	0	0	0	0	0	0	0	0	0	0
EEF1D	0	0	0	0	0	0	0	0	0	0	0	0	0
EEF1G	0	0	0	0	0	0	0	0	0	0	0	0	0
EEF2	0	0	0	0	0	0	0	0	0	0	0	0	0
EIF2B1	0	0	0	0	0	0	0	0	0	0	0	0	0
EIF2B2	0	0	0	0	0	0	0	0	0	0	0	0	0
EIF2B5	0	0	0	0	0	0	0	0	0	0	0	0	0
EIF2S1	0	0	0	0	0	0	0	0	0	0	0	0	0
EIF2S2	0	0	0	0	0	0	0	0	0	0	0	0	0
EIF2S3	0	0	0	0	0	0	0	0	0	0	0	0	0
EIF3A	0	0	0	0	0	0	0	0	0	0	0	0	0
EIF3B	0	0	0	0	0	0	0	0	0	0	0	0	0
EIF3D	0	0	0	0	0	0	0	0	0	0	0	0	0

NAME	REACTOME_ER_PHAGOSOME_PATHWAY	REACTOME_ING_BY_INSULIN_RECEPTOR	REACTOME_CELL_SURFACE_INTERACTIONS_AT_THE_VASCULAR_WALL	REACTOME_INTEGRIN_CELL_SURFACE_INTERACTIONS	REACTOME_RESPONSE_TO_ELEVATED_PLATELET_CYTOSOLIC_CA2_	REACTOME_HEMOSTASIS	REACTOME_PLATELET_ACTIVATION_ING_AND_AGGREGATION	REACTOME_SEMAPHORIN_INTERACTIONS	REACTOME_L1CAM_INTERACTIONS	REACTOME_DEVELOPMENTAL_BIOLOGY	REACTOME_AXON_GUIDANCE	REACTOME_INTEGRATION_OF_ENERGY_METABOLISM	REACTOME_REGULATION_OF_INSULIN_SECRETION
DBNL	0	0	0	0	0	0	0	0	0	0	0	0	0
DCTN1	0	0	0	0	0	0	0	0	0	0	0	0	0
DCTN2	0	0	0	0	0	0	0	0	0	0	0	0	0
DDOST	0	0	0	0	0	0	0	0	0	0	0	0	0
DDX58	0	0	0	0	0	0	0	0	0	0	0	0	0
DEFA1	0	0	0	0	0	0	0	0	0	0	0	0	0
DNAJB11	0	0	0	0	0	0	0	0	0	0	0	0	0
DNAJC3	0	0	0	0	0	0	0	0	0	0	0	0	0
DPM1	0	0	0	0	0	0	0	0	0	0	0	0	0
DPP4	0	0	0	0	0	0	0	0	0	0	0	1	1
DPYSL2	0	0	0	0	0	0	0	1	1	0	0	0	0
DYNC1H1	0	0	0	0	0	0	0	0	0	0	0	0	0
EEF1A1	0	0	0	0	0	0	0	0	0	0	0	0	0
EEF1D	0	0	0	0	0	0	0	0	0	0	0	0	0
EEF1G	0	0	0	0	0	0	0	0	0	0	0	0	0
EEF2	0	0	0	0	0	0	0	0	0	0	0	0	0
EIF2B1	0	0	0	0	0	0	0	0	0	0	0	0	0
EIF2B2	0	0	0	0	0	0	0	0	0	0	0	0	0
EIF2B5	0	0	0	0	0	0	0	0	0	0	0	0	0
EIF2S1	0	0	0	0	0	0	0	0	0	0	0	0	0
EIF2S2	0	0	0	0	0	0	0	0	0	0	0	0	0
EIF2S3	0	0	0	0	0	0	0	0	0	0	0	0	0
EIF3A	0	0	0	0	0	0	0	0	0	0	0	0	0
EIF3B	0	0	0	0	0	0	0	0	0	0	0	0	0
EIF3D	0	0	0	0	0	0	0	0	0	0	0	0	0

NAME	REACTOME_ING_BY_ILS	REACTOME_GPCR_DOWNSTREAM_ING	REACTOME_ING_BY_GPCR	REACTOME_LING_BY_NGF	REACTOME_NGF_LING_VIA_TRKA_FROM_THE_PLASMA_MEMBRANE	REACTOME_ING_BY_EGFR_IN_CANCER	REACTOME_ING_BY_FGFR_IN_DISEASE	REACTOME_ING_BY_FGFR	REACTOME_ING_BY_ERBB2	REACTOME_DOWNSTREAM__TRANSDUCTION	REACTOME_ING_BY_PDGF	KEGG_RIBOSOME	KEGG_PROTEASOME
DBNL	0	0	0	0	0	0	0	0	0	0	0	0	0
DCTN1	0	0	0	0	0	0	0	0	0	0	0	0	0
DCTN2	0	0	0	0	0	0	0	0	0	0	0	0	0
DDOST	0	0	0	0	0	0	0	0	0	0	0	0	0
DDX58	0	0	0	0	0	0	0	0	0	0	0	0	0
DEFA1	0	0	0	0	0	0	0	0	0	0	0	0	0
DNAJB11	0	0	0	0	0	0	0	0	0	0	0	0	0
DNAJC3	0	0	0	0	0	0	0	0	0	0	0	1	0
DPM1	0	0	0	0	0	0	0	0	0	0	0	1	0
DPP4	0	0	0	0	0	0	0	0	0	0	0	0	0
DPYSL2	0	0	0	0	0	0	0	0	0	0	0	0	0
DYNC1H1	0	0	0	0	0	0	0	0	0	0	0	0	0
EEF1A1	0	0	0	0	0	0	0	0	0	0	0	0	0
EEF1D	0	0	0	0	0	0	0	0	0	0	0	0	0
EEF1G	0	0	0	0	0	0	0	0	0	0	0	0	0
EEF2	0	0	0	0	0	0	0	0	0	0	0	0	0
EIF2B1	0	0	0	0	0	0	0	0	0	0	0	0	0
EIF2B2	0	0	0	0	0	0	0	0	0	0	0	0	0
EIF2B5	0	0	0	0	0	0	0	0	0	0	0	0	0
EIF2S1	0	0	0	0	0	0	0	0	0	0	0	0	0
EIF2S2	0	0	0	0	0	0	0	0	0	0	0	0	0
EIF2S3	0	0	0	0	0	0	0	0	0	0	0	0	0
EIF3A	0	0	0	0	0	0	0	0	0	0	0	0	0
EIF3B	0	0	0	0	0	0	0	0	0	0	0	0	0
EIF3D	0	0	0	0	0	0	0	0	0	0	0	0	0

NAME	KEGG_PYRUVATE_METABOLISM	KEGG_LYSOSOME	KEGG_ENDOCYTOSIS	KEGG_EPITHELIAL_CELL_ING_IN_HELICOBACTER_PYLORI_INFECTION	KEGG_AXON_GUIDANCE	KEGG_NEUROTROPHIN_ING_PATHWAY	KEGG_CHEMOKINE_ING_PATHWAY	KEGG_INSULIN_ING_PATHWAY	KEGG_PROSTATE_CANCER	KEGG_PATHWAYS_IN_CANCER	KEGG_ERBB_ING_PATHWAY	KEGG_MAPK_ING_PATHWAY	KEGG_PATHOGENIC_ESCHERICHIA_COLI_INFECTION
DBNL	0	0	1	0	0	0	0	0	0	0	0	0	0
DCTN1	0	0	0	0	1	0	0	0	0	0	0	0	0
DCTN2	0	0	0	0	0	0	0	0	0	0	0	0	0
DDOST	0	0	1	0	0	0	0	0	0	0	0	0	0
DDX58	0	0	0	0	0	0	0	0	0	0	0	0	0
DEFA1	0	0	1	0	0	0	0	0	0	0	0	0	0
DNAJB11	0	0	1	0	0	0	0	0	0	0	0	0	0
DNAJC3	0	0	0	0	0	0	0	0	0	0	0	0	0
DPM1	0	0	0	0	0	0	0	0	0	0	0	0	0
DPP4	0	1	0	0	0	0	0	0	0	0	0	0	0
DPYSL2	0	0	1	0	0	0	0	0	0	0	0	0	0
DYNC1H1	0	0	1	0	0	0	0	0	0	0	0	0	0
EEF1A1	0	0	0	0	0	0	0	0	0	1	0	0	0
EEF1D	0	0	0	0	0	0	0	0	0	0	0	0	0
EEF1G	0	0	0	0	0	1	0	0	0	0	0	0	0
EEF2	0	0	0	0	0	1	0	0	0	0	0	0	1
EIF2B1	0	0	0	0	0	0	0	0	0	0	0	0	0
EIF2B2	0	0	0	0	0	0	0	0	0	0	0	0	0
EIF2B5	0	0	0	0	0	0	0	0	0	0	0	0	0
EIF2S1	0	0	0	0	0	0	0	0	0	0	0	0	0
EIF2S2	0	0	0	0	0	0	0	0	0	0	0	0	0
EIF2S3	0	0	0	0	0	0	0	0	0	0	0	0	0
EIF3A	0	0	0	0	0	0	0	0	0	0	0	0	0
EIF3B	0	0	0	0	0	0	0	0	0	0	0	0	0
EIF3D	0	0	0	0	0	0	0	0	0	0	0	0	0

NAME	KEGG_TIGHT_JUNCTION	KEGG_ADHERENS_JUNCTION	KEGG_LEUKOCYTE_TRANSENDOTHELIAL_MIGRATION	KEGG_ARRHYTHMOGENIC_RIGHT_VENTRICULAR_CARDIOMYOPATHY_ARVC	KEGG_ECM_RECEPTOR_INTERACTION	KEGG_FOCAL_ADHESION	KEGG_FC_GAMMA_R_MEDIATED_PHAGOCYTOSIS	KEGG_REGULATION_OF_ACTIN_CYTOSKELETON
DBNL	0	0	0	0	0	0	1	1
DCTN1	0	0	0	0	0	0	0	0
DCTN2	0	0	0	0	0	0	0	1
DDOST	0	0	0	0	0	0	0	0
DDX58	1	0	0	0	0	0	0	0
DEFA1	0	0	0	0	0	0	0	0
DNAJB11	0	0	0	0	0	0	0	0
DNAJC3	0	0	0	0	0	0	0	0
DPM1	0	0	0	0	0	0	0	0
DPP4	0	0	0	0	0	0	0	0
DPYSL2	0	0	0	0	0	0	0	0
DYNC1H1	0	0	0	0	0	0	0	0
EEF1A1	0	0	0	0	0	0	0	0
EEF1D	0	0	1	0	0	0	0	0
EEF1G	0	0	0	0	0	0	0	0
EEF2	0	0	0	0	0	0	0	0
EIF2B1	0	0	0	0	0	1	0	0
EIF2B2	0	0	0	0	0	0	0	0
EIF2B5	0	0	0	0	0	0	0	0
EIF2S1	0	0	0	0	0	0	0	0
EIF2S2	0	0	0	0	0	0	0	0
EIF2S3	0	0	0	0	0	0	0	0
EIF3A	0	0	0	0	0	0	0	0
EIF3B	0	0	0	0	0	0	0	0
EIF3D	0	0	0	0	0	0	0	0

Table S6 The differential proteins contribute to the enrichment of the significant pathways for the public dataset

Core enrichment proteins in down-regulated pathways of hiPSCs/HFs (hiPSCs vs HFs)

NAME	REACTOME_GLUCOSE_METABOLISM	REACTOME_UNFOLDED_PROTEIN_RESPONSE	REACTOME_DIABETES_PATHWAYS	REACTOME_PROTEIN_FOLDING	REACTOME_PREFOLDIN_MEDIATED_TRANSFER_OF_SUBSTRATE_TO_CCT_TRIC	REACTOME_ASPARAGINE_N_LINKED_GLYCOSYLATION	REACTOME_POST_TRANSLATIONAL_PROTEIN_MODIFICATION	REACTOME_ACTIVATION_OF_THE_MRNA_UPON_BINDING_OF_THE_CAP_BINDING_COMPLEX_AND_EIFS_AND_SUBSEQUENT_BINDING_TO_43S	REACTOME_FORMATION_OF_THE_TERNARY_COMPLEX_AND_SUBSEQUENTLY_THE_43S_COMPLEX	REACTOME_METABOLISM_OF_PROTEINS	REACTOME_SRP_DEPENDENT_COTRANSLATIONAL_PROTEIN_TARGETING_TO_MEMBRANE	REACTOME_INFLUENZA_VIRAL_RNA_TRANSCRIPTION_AND_REPLICATION	REACTOME_NONSENSE_MEDIATED_DECAY_ENHANCED_BY_THE_EXON_JUNCTION_COMPLEX
EIF3E	0	0	0	0	0	0	0	1	1	1	0	0	0
EIF3F	0	0	0	0	0	0	0	1	1	1	0	0	0
EIF3G	0	0	0	0	0	0	0	1	1	1	0	0	0
EIF3H	0	0	0	0	0	0	0	1	1	1	0	0	0
EIF3I	0	0	0	0	0	0	0	1	1	1	0	0	0
EIF3J	0	0	0	0	0	0	0	1	1	1	0	0	0
EIF3K	0	0	0	0	0	0	0	1	1	1	0	0	0
EIF4A1	0	0	0	0	0	0	0	1	0	1	0	0	0
EIF4B	0	0	0	0	0	0	0	1	0	1	0	0	0
EIF4G3	0	0	0	0	0	0	0	0	0	0	0	0	0
EIF4H	0	0	0	0	0	0	0	1	0	1	0	0	0
EIF5	0	0	0	0	0	0	0	0	0	1	0	0	0
EIF5B	0	0	0	0	0	0	0	0	0	1	0	0	0
EPS15L1	0	0	0	0	0	0	0	0	0	0	0	0	0
ERAP1	0	0	0	0	0	0	0	0	0	0	0	0	0
ERO1L	0	0	1	0	0	0	0	0	0	0	0	0	0
ETF1	0	0	0	0	0	0	0	0	0	1	0	0	1
EXOC1	0	0	1	0	0	0	0	0	0	0	0	0	0
EXOC2	0	0	1	0	0	0	0	0	0	0	0	0	0
EXOC4	0	0	1	0	0	0	0	0	0	0	0	0	0
EXOC5	0	0	1	0	0	0	0	0	0	0	0	0	0
EXOC8	0	0	1	0	0	0	0	0	0	0	0	0	0
FLNB	0	0	0	0	0	0	0	0	0	0	0	0	0
FTL	0	0	0	0	0	0	0	0	0	0	0	0	0
GAK	0	0	0	0	0	0	0	0	0	0	0	0	0

NAME	REACTOME_PEPTIDE_CHAIN_ELONGATION	REACTOME_3_UTR_MEDIATED_TRANSLATIONAL_REGULATION	REACTOME_TRANSLATION	REACTOME_CELL_CELL_COMMUNICATION	REACTOME_GOLGI_ASSOCIATED_VESICLE_BIOGENESIS	REACTOME_TRANS_GOLGI_NETWORK_VESICLE_BUDDING	REACTOME_MEMBRANE_TRAFFICKING	REACTOME_TRANSMISSION_ACROSS_CHEMICAL_SYNAPSES	REACTOME_NEURONAL_SYSTEM	REACTOME_MHC_CLASS_II_ANTIGEN_PRESENTATION	REACTOME_INNATE_IMMUNE_SYSTEM	REACTOME_IMMUNE_SYSTEM	REACTOME_ADAPTIVE_IMMUNE_SYSTEM
EIF3E	0	1	1	0	0	0	0	0	0	0	0	0	0
EIF3F	0	1	1	0	0	0	0	0	0	0	0	0	0
EIF3G	0	1	1	0	0	0	0	0	0	0	0	0	0
EIF3H	0	1	1	0	0	0	0	0	0	0	0	0	0
EIF3I	0	1	1	0	0	0	0	0	0	0	0	0	0
EIF3J	0	1	1	0	0	0	0	0	0	0	0	0	0
EIF3K	0	1	1	0	0	0	0	0	0	0	0	0	0
EIF4A1	0	1	1	0	0	0	0	0	0	0	0	1	0
EIF4B	0	1	1	0	0	0	0	0	0	0	0	0	0
EIF4G3	0	0	0	0	0	0	0	0	0	0	0	1	0
EIF4H	0	1	1	0	0	0	0	0	0	0	0	0	0
EIF5	0	0	1	0	0	0	0	0	0	0	0	0	0
EIF5B	0	0	1	0	0	0	0	0	0	0	0	0	0
EPS15L1	0	0	0	0	0	0	0	0	0	0	0	0	0
ERAP1	0	0	0	0	0	0	0	0	0	0	0	1	1
ERO1L	0	0	0	0	0	0	0	0	0	0	0	0	0
ETF1	0	0	1	0	0	0	0	0	0	0	0	0	0
EXOC1	0	0	0	0	0	0	0	0	0	0	0	0	0
EXOC2	0	0	0	0	0	0	0	0	0	0	0	0	0
EXOC4	0	0	0	0	0	0	0	0	0	0	0	0	0
EXOC5	0	0	0	0	0	0	0	0	0	0	0	0	0
EXOC8	0	0	0	0	0	0	0	0	0	0	0	0	0
FLNB	0	0	0	0	0	0	0	0	0	0	0	1	0
FTL	0	0	0	0	1	1	0	0	0	0	0	0	0
GAK	0	0	0	0	1	1	1	0	0	0	0	0	0

NAME	REACTOME_CLASS_I_MHC_MEDIATED_ANTIGEN_PROCESSING_PRESENTATION	REACTOME_APOPTOSIS	REACTOME_ANTIGEN_PROCESSING_UBIQUITINATION_PROTEASOME_DEGRADATION	REACTOME_ING_BY_WNT	REACTOME_REGULATION_OF_MRNA_STABILITY_BY_PROTEINS_THAT_BIND_AU_RICH_ELEMENTS	REACTOME_DESTABILIZATION_OF_MRNA_BY_AUF1_HNRNP_D0	REACTOME_REGULATION_OF_APOPTOSIS	REACTOME_REGULATION_OF_ORNITHINE_DECARBOXYLASE_ODC	REACTOME_CROSS_PRESENTATION_OF_SOLUBLE_EXOGENOUS_ANTIGENS_ENDOSOMES	REACTOME_VIF_MEDIATED_DEGRADATION_OF_APOBEC3G	REACTOME_REGULATION_OF_MITOTIC_CELL_CYCLE	REACTOME_APC_C_CDC20_MEDIATED_DEGRADATION_OF_MITOTIC_PROTEINS	REACTOME_CDT1_ASSOCIATION_WITH_THE_CDC6_ORC_ORIGIN_COMPLEX
EIF3E	0	0	0	0	0	0	0	0	0	0	0	0	0
EIF3F	0	0	0	0	0	0	0	0	0	0	0	0	0
EIF3G	0	0	0	0	0	0	0	0	0	0	0	0	0
EIF3H	0	0	0	0	0	0	0	0	0	0	0	0	0
EIF3I	0	0	0	0	0	0	0	0	0	0	0	0	0
EIF3J	0	0	0	0	0	0	0	0	0	0	0	0	0
EIF3K	0	0	0	0	0	0	0	0	0	0	0	0	0
EIF4A1	0	0	0	0	0	0	0	0	0	0	0	0	0
EIF4B	0	0	0	0	0	0	0	0	0	0	0	0	0
EIF4G3	0	0	0	0	0	0	0	0	0	0	0	0	0
EIF4H	0	0	0	0	0	0	0	0	0	0	0	0	0
EIF5	0	0	0	0	0	0	0	0	0	0	0	0	0
EIF5B	0	0	0	0	0	0	0	0	0	0	0	0	0
EPS15L1	0	0	0	0	0	0	0	0	0	0	0	0	0
ERAP1	1	0	0	0	0	0	0	0	0	0	0	0	0
ERO1L	0	0	0	0	0	0	0	0	0	0	0	0	0
ETF1	0	0	0	0	0	0	0	0	0	0	0	0	0
EXOC1	0	0	0	0	0	0	0	0	0	0	0	0	0
EXOC2	0	0	0	0	0	0	0	0	0	0	0	0	0
EXOC4	0	0	0	0	0	0	0	0	0	0	0	0	0
EXOC5	0	0	0	0	0	0	0	0	0	0	0	0	0
EXOC8	0	0	0	0	0	0	0	0	0	0	0	0	0
FLNB	0	0	0	0	0	0	0	0	0	0	0	0	0
FTL	0	0	0	0	0	0	0	0	0	0	0	0	0
GAK	0	0	0	0	0	0	0	0	0	0	0	0	0

NAME	REACTOME_APC_C_CDH1_MEDIATED_DEGRADATION_OF_CDC20_AND_OTHER_APC_C_CDH1_TARGETED_PROTEINS_IN_LATE_MITOSIS_EARLY_G1	REACTOME_CYCLIN_E_ASSOCIATED_EVENTS_DURING_G1_S_TRANSITION_	REACTOME_SCFSKP2_MEDIATED_DEGRADATION_OF_P27_P21	REACTOME_AUTODEGRADATION_OF_CDH1_BY_CDH1_APC_C	REACTOME_AUTODEGRADATION_OF_THE_E3_UBIQUITIN_LIGASE_COP1	REACTOME_SCF_BETA_TRCP_MEDIATED_DEGRADATION_OF_EMI1	REACTOME_P53_INDEPENDENT_G1_S_DNA_DAMAGE_CHECKPOINT	REACTOME_CDK_MEDIATED_PHOSPHORYLATION_AND_REMOVAL_OF_CDC6	REACTOME_P53_DEPENDENT_G1_DNA_DAMAGE_RESPONSE	REACTOME_ACTIVATION_OF_NF_KAPPAB_IN_B_CELLS	REACTOME_DOWNSTREAM_ING_EVENTS_OF_B_CELL_RECEPTOR_BCR	REACTOME_ING_BY_THE_B_CELL_RECEPTOR_BCR	REACTOME_ANTIGEN_PROCESSING_CROSS_PRESENTATION
EIF3E	0	0	0	0	0	0	0	0	0	0	0	0	0
EIF3F	0	0	0	0	0	0	0	0	0	0	0	0	0
EIF3G	0	0	0	0	0	0	0	0	0	0	0	0	0
EIF3H	0	0	0	0	0	0	0	0	0	0	0	0	0
EIF3I	0	0	0	0	0	0	0	0	0	0	0	0	0
EIF3J	0	0	0	0	0	0	0	0	0	0	0	0	0
EIF3K	0	0	0	0	0	0	0	0	0	0	0	0	0
EIF4A1	0	0	0	0	0	0	0	0	0	0	0	0	0
EIF4B	0	0	0	0	0	0	0	0	0	0	0	0	0
EIF4G3	0	0	0	0	0	0	0	0	0	0	0	0	0
EIF4H	0	0	0	0	0	0	0	0	0	0	0	0	0
EIF5	0	0	0	0	0	0	0	0	0	0	0	0	0
EIF5B	0	0	0	0	0	0	0	0	0	0	0	0	0
EPS15L1	0	0	0	0	0	0	0	0	0	0	0	0	0
ERAP1	0	0	0	0	0	0	0	0	0	0	0	0	0
ERO1L	0	0	0	0	0	0	0	0	0	0	0	0	0
ETF1	0	0	0	0	0	0	0	0	0	0	0	0	0
EXOC1	0	0	0	0	0	0	0	0	0	0	0	0	0
EXOC2	0	0	0	0	0	0	0	0	0	0	0	0	0
EXOC4	0	0	0	0	0	0	0	0	0	0	0	0	0
EXOC5	0	0	0	0	0	0	0	0	0	0	0	0	0
EXOC8	0	0	0	0	0	0	0	0	0	0	0	0	0
FLNB	0	0	0	0	0	0	0	0	0	0	0	0	0
FTL	0	0	0	0	0	0	0	0	0	0	0	0	0
GAK	0	0	0	0	0	0	0	0	0	0	0	0	0

NAME	REACTOME_ER_PHAGOSOME_PATHWAY	REACTOME_ING_BY_INSULIN_RECEPTOR	REACTOME_CELL_SURFACE_INTERACTIONS_AT_THE_VASCULAR_WALL	REACTOME_INTEGRIN_CELL_SURFACE_INTERACTIONS	REACTOME_RESPONSE_TO_ELEVATED_PLATELET_CYTOSOLIC_CA2_	REACTOME_HEMOSTASIS	REACTOME_PLATELET_ACTIVATION_ING_AND_AGGREGATION	REACTOME_SEMAPHORIN_INTERACTIONS	REACTOME_L1CAM_INTERACTIONS	REACTOME_DEVELOPMENTAL_BIOLOGY	REACTOME_AXON_GUIDANCE	REACTOME_INTEGRATION_OF_ENERGY_METABOLISM	REACTOME_REGULATION_OF_INSULIN_SECRETION
EIF3E	0	0	0	0	0	0	0	0	0	0	0	0	0
EIF3F	0	0	0	0	0	0	0	0	0	0	0	0	0
EIF3G	0	0	0	0	0	0	0	0	0	0	0	0	0
EIF3H	0	0	0	0	0	0	0	0	0	0	0	0	0
EIF3I	0	0	0	0	0	0	0	0	0	0	0	0	0
EIF3J	0	0	0	0	0	0	0	0	0	0	0	0	0
EIF3K	0	0	0	0	0	0	0	0	0	0	0	0	0
EIF4A1	0	0	0	0	0	0	0	0	0	0	0	0	0
EIF4B	0	0	0	0	0	0	0	0	0	0	0	0	0
EIF4G3	0	0	0	0	0	0	0	0	0	0	0	0	0
EIF4H	0	0	0	0	0	0	0	0	0	0	0	0	0
EIF5	0	0	0	0	0	0	0	0	0	0	0	0	0
EIF5B	0	0	0	0	0	0	0	0	0	0	0	0	0
EPS15L1	0	0	0	0	0	0	0	0	0	0	0	0	0
ERAP1	0	0	0	0	0	0	0	0	0	0	0	0	0
ERO1L	0	0	0	0	0	0	0	0	0	0	0	0	0
ETF1	0	0	0	0	0	0	0	0	0	0	0	0	0
EXOC1	0	0	0	0	0	0	0	0	0	0	0	0	0
EXOC2	0	0	0	0	0	0	0	0	0	0	0	0	0
EXOC4	0	0	0	0	0	0	0	0	0	0	0	0	0
EXOC5	0	0	0	0	0	0	0	0	0	0	0	0	0
EXOC8	0	0	0	0	0	0	0	0	0	0	0	0	0
FLNB	0	0	0	0	0	0	0	0	0	0	0	0	0
FTL	0	0	0	0	0	0	0	0	0	0	0	0	0
GAK	0	0	0	0	0	0	0	0	0	0	0	0	0

NAME	REACTOME_ING_BY_ILS	REACTOME_GPCR_DOWNSTREAM_ING	REACTOME_ING_BY_GPCR	REACTOME_LING_BY_NGF	REACTOME_NGF_LING_VIA_TRKA_FROM_THE_PLASMA_MEMBRANE	REACTOME_ING_BY_EGFR_IN_CANCER	REACTOME_ING_BY_FGFR_IN_DISEASE	REACTOME_ING_BY_FGFR	REACTOME_ING_BY_ERBB2	REACTOME_DOWNSTREAM__TRANSDUCTION	REACTOME_ING_BY_PDGF	KEGG_RIBOSOME	KEGG_PROTEASOME
EIF3E	0	0	0	0	0	0	0	0	0	0	0	0	0
EIF3F	0	0	0	0	0	0	0	0	0	0	0	0	0
EIF3G	0	0	0	0	0	0	0	0	0	0	0	0	0
EIF3H	0	0	0	0	0	0	0	0	0	0	0	0	0
EIF3I	0	0	0	0	0	0	0	0	0	0	0	0	0
EIF3J	0	0	0	0	0	0	0	0	0	0	0	0	0
EIF3K	0	0	0	0	0	0	0	0	0	0	0	0	0
EIF4A1	0	0	0	0	0	0	0	0	0	0	0	0	0
EIF4B	0	0	0	0	0	0	0	0	0	0	0	0	0
EIF4G3	0	0	0	0	0	0	0	0	0	0	0	0	0
EIF4H	0	0	0	0	0	0	0	0	0	0	0	0	0
EIF5	0	0	0	0	0	0	0	0	0	0	0	0	0
EIF5B	0	0	0	0	0	0	0	0	0	0	0	0	0
EPS15L1	0	0	0	0	0	1	0	0	0	0	0	0	0
ERAP1	0	0	0	0	0	0	0	0	0	0	0	0	0
ERO1L	0	0	0	0	0	0	0	0	0	0	0	0	0
ETF1	0	0	0	0	0	0	0	0	0	0	0	0	0
EXOC1	0	0	0	0	0	0	0	0	0	0	0	0	0
EXOC2	0	0	0	0	0	0	0	0	0	0	0	0	0
EXOC4	0	0	0	0	0	0	0	0	0	0	0	0	0
EXOC5	0	0	0	0	0	0	0	0	0	0	0	0	0
EXOC8	0	0	0	0	0	0	0	0	0	0	0	0	0
FLNB	0	0	0	0	0	0	0	0	0	0	0	0	0
FTL	0	0	0	0	0	0	0	0	0	0	0	0	0
GAK	0	0	0	0	0	0	0	0	0	0	0	0	0

NAME	KEGG_PYRUVATE_METABOLISM	KEGG_LYSOSOME	KEGG_ENDOCYTOSIS	KEGG_EPITHELIAL_CELL_ING_IN_HELICOBACTER_PYLORI_INFECTION	KEGG_AXON_GUIDANCE	KEGG_NEUROTROPHIN_ING_PATHWAY	KEGG_CHEMOKINE_ING_PATHWAY	KEGG_INSULIN_ING_PATHWAY	KEGG_PROSTATE_CANCER	KEGG_PATHWAYS_IN_CANCER	KEGG_ERBB_ING_PATHWAY	KEGG_MAPK_ING_PATHWAY	KEGG_PATHOGENIC_ESCHERICHIA_COLI_INFECTION
EIF3E	0	0	0	0	0	0	0	0	0	0	0	0	0
EIF3F	0	0	0	0	0	0	0	0	0	0	0	0	0
EIF3G	0	0	0	0	0	0	0	0	0	0	0	0	0
EIF3H	0	0	0	0	0	0	0	0	0	0	0	0	0
EIF3I	0	0	0	0	0	0	0	0	0	0	0	0	0
EIF3J	0	0	0	0	0	0	0	0	0	0	0	0	0
EIF3K	0	0	0	0	0	0	0	0	0	0	0	0	0
EIF4A1	0	0	0	0	0	0	0	0	0	0	0	0	0
EIF4B	0	0	0	0	0	0	0	0	0	0	0	0	0
EIF4G3	0	0	0	0	0	0	0	0	0	0	0	0	0
EIF4H	0	0	0	0	0	0	0	0	0	0	0	0	0
EIF5	0	0	0	0	0	0	0	0	0	0	0	0	0
EIF5B	0	0	0	0	0	0	0	0	0	0	0	0	0
EPS15L1	0	0	0	0	0	0	0	0	0	0	0	0	0
ERAP1	0	0	0	0	0	0	0	0	0	0	0	0	0
ERO1L	0	0	0	0	0	0	0	0	0	0	0	0	0
ETF1	0	0	0	0	0	0	0	0	0	0	0	0	0
EXOC1	0	0	0	0	0	0	0	0	0	0	0	0	0
EXOC2	0	0	0	0	0	0	0	0	0	0	0	0	0
EXOC4	0	0	0	0	0	0	0	0	0	0	0	0	0
EXOC5	0	0	0	0	0	0	0	0	0	0	0	0	0
EXOC8	0	0	0	0	0	0	0	0	0	0	0	0	0
FLNB	0	0	0	0	0	0	0	0	0	0	0	0	0
FTL	0	0	0	0	0	0	0	0	0	0	0	0	0
GAK	0	0	0	0	0	0	0	0	0	0	0	0	0

NAME	KEGG_TIGHT_JUNCTION	KEGG_ADHERENS_JUNCTION	KEGG_LEUKOCYTE_TRANSENDOTHELIAL_MIGRATION	KEGG_ARRHYTHMOGENIC_RIGHT_VENTRICULAR_CARDIOMYOPATHY_ARVC	KEGG_ECM_RECEPTOR_INTERACTION	KEGG_FOCAL_ADHESION	KEGG_FC_GAMMA_R_MEDIATED_PHAGOCYTOSIS	KEGG_REGULATION_OF_ACTIN_CYTOSKELETON
EIF3E	0	0	0	0	0	0	0	0
EIF3F	0	0	0	0	0	0	0	0
EIF3G	0	0	0	0	0	0	0	0
EIF3H	0	0	0	0	0	0	0	0
EIF3I	0	0	0	0	0	0	0	0
EIF3J	0	0	0	0	0	0	0	0
EIF3K	0	0	0	0	0	0	0	0
EIF4A1	0	0	0	0	0	0	0	0
EIF4B	0	0	0	0	0	0	0	0
EIF4G3	0	0	0	0	0	0	0	0
EIF4H	0	0	0	0	0	0	0	0
EIF5	0	0	0	0	0	0	0	0
EIF5B	0	0	0	0	0	0	0	0
EPS15L1	0	0	0	0	0	0	0	0
ERAP1	0	0	0	0	0	0	0	0
ERO1L	0	0	0	0	0	0	0	0
ETF1	0	0	0	0	0	0	0	0
EXOC1	0	0	0	0	0	0	0	0
EXOC2	0	0	0	0	0	0	0	0
EXOC4	0	0	0	0	0	0	0	0
EXOC5	0	0	0	0	0	0	0	0
EXOC8	0	0	0	0	0	0	0	0
FLNB	0	0	0	0	0	0	0	0
FTL	0	0	0	0	0	0	0	0
GAK	0	0	0	0	0	0	0	0

Table S6 The differential proteins contribute to the enrichment of the significant pathways for the public dataset

Core enrichment proteins in down-regulated pathways of hiPSCs/HFs (hiPSCs vs HFs)

NAME	REACTOME_GLUCOSE_METABOLISM	REACTOME_UNFOLDED_PROTEIN_RESPONSE	REACTOME_DIABETES_PATHWAYS	REACTOME_PROTEIN_FOLDING	REACTOME_PREFOLDIN_MEDIATED_TRANSFER_OF_SUBSTRATE_TO_CCT_TRIC	REACTOME_ASPARAGINE_N_LINKED_GLYCOSYLATION	REACTOME_POST_TRANSLATIONAL_PROTEIN_MODIFICATION	REACTOME_ACTIVATION_OF_THE_MRNA_UPON_BINDING_OF_THE_CAP_BINDING_COMPLEX_AND_EIFS_AND_SUBSEQUENT_BINDING_TO_43S	REACTOME_FORMATION_OF_THE_TERNARY_COMPLEX_AND_SUBSEQUENTLY_THE_43S_COMPLEX	REACTOME_METABOLISM_OF_PROTEINS	REACTOME_SRP_DEPENDENT_COTRANSLATIONAL_PROTEIN_TARGETING_TO_MEMBRANE	REACTOME_INFLUENZA_VIRAL_RNA_TRANSCRIPTION_AND_REPLICATION	REACTOME_NONSENSE_MEDIATED_DECAY_ENHANCED_BY_THE_EXON_JUNCTION_COMPLEX
GALNT2	0	0	0	0	0	0	1	0	0	1	0	0	0
GAPDH	1	0	0	0	0	0	0	0	0	0	0	0	0
GBF1	0	0	0	0	0	0	0	0	0	0	0	0	0
GLS	0	0	0	0	0	0	0	0	0	0	0	0	0
GMPPA	0	0	0	0	0	1	1	0	0	1	0	0	0
GMPPB	0	0	0	0	0	1	1	0	0	1	0	0	0
GNB2	0	0	0	0	0	0	0	0	0	0	0	0	0
GPC1	0	0	0	0	0	0	0	0	0	0	0	0	0
GSK3A	0	0	0	0	0	0	0	0	0	0	0	0	0
GYG1	1	0	0	0	0	0	0	0	0	0	0	0	0
HEBP1	0	0	0	0	0	0	0	0	0	0	0	0	0
HSP90AA1	0	0	0	0	0	0	0	0	0	0	0	1	0
HSP90B1	0	0	0	0	0	0	0	0	0	0	0	0	0
HSPA5	0	1	1	0	0	0	0	0	0	0	0	0	0
HSPA8	0	0	0	0	0	0	0	0	0	0	0	0	0
IGF2R	0	0	0	0	0	0	0	0	0	0	0	0	0
IPO5	0	0	0	0	0	0	0	0	0	0	0	1	0
ISG15	0	0	0	0	0	0	0	0	0	0	0	0	0
KIF3B	0	0	0	0	0	0	0	0	0	0	0	0	0
KIF5B	0	0	1	0	0	0	0	0	0	0	0	0	0
KPNA4	0	0	0	0	0	0	0	0	0	0	0	0	0
KPNB1	0	0	0	0	0	0	0	0	0	0	0	0	0
LAMB1	0	0	0	0	0	0	0	0	0	0	0	0	0
LAMC1	0	0	0	0	0	0	0	0	0	0	0	0	0
LGALS3	0	0	0	0	0	0	0	0	0	0	0	0	0

NAME	REACTOME_PEPTIDE_CHAIN_ELONGATION	REACTOME_3_UTR_MEDIATED_TRANSLATIONAL_REGULATION	REACTOME_TRANSLATION	REACTOME_CELL_CELL_COMMUNICATION	REACTOME_GOLGI_ASSOCIATED_VESICLE_BIOGENESIS	REACTOME_TRANS_GOLGI_NETWORK_VESICLE_BUDDING	REACTOME_MEMBRANE_TRAFFICKING	REACTOME_TRANSMISSION_ACROSS_CHEMICAL_SYNAPSES	REACTOME_NEURONAL_SYSTEM	REACTOME_MHC_CLASS_II_ANTIGEN_PRESENTATION	REACTOME_INNATE_IMMUNE_SYSTEM	REACTOME_IMMUNE_SYSTEM	REACTOME_ADAPTIVE_IMMUNE_SYSTEM
GALNT2	0	0	0	0	0	0	0	0	0	0	0	0	0
GAPDH	0	0	0	0	0	0	0	0	0	0	0	0	0
GBF1	0	0	0	0	0	1	1	0	0	0	0	0	0
GLS	0	0	0	0	0	0	0	1	1	0	0	0	0
GMPPA	0	0	0	0	0	0	0	0	0	0	0	0	0
GMPPB	0	0	0	0	0	0	0	0	0	0	0	0	0
GNB2	0	0	0	0	0	0	0	0	1	0	0	0	0
GPC1	0	0	0	0	0	0	0	0	0	0	0	0	0
GSK3A	0	0	0	0	0	0	0	0	0	0	0	0	0
GYG1	0	0	0	0	0	0	0	0	0	0	0	0	0
HEBP1	0	0	0	0	0	0	0	0	0	0	0	0	0
HSP90AA1	0	0	0	0	0	0	0	0	0	0	0	0	0
HSP90B1	0	0	0	0	0	0	0	0	0	0	1	1	0
HSPA5	0	0	0	0	0	0	0	0	0	0	0	1	1
HSPA8	0	0	0	0	1	1	0	0	0	0	0	0	0
IGF2R	0	0	0	0	1	1	0	0	0	0	0	0	0
IPO5	0	0	0	0	0	0	0	0	0	0	0	0	0
ISG15	0	0	0	0	0	0	0	0	0	0	1	1	0
KIF3B	0	0	0	0	0	0	0	0	0	0	0	1	1
KIF5B	0	0	0	0	0	0	0	0	0	1	0	1	1
KPNA4	0	0	0	0	0	0	0	0	0	0	0	1	0
KPNB1	0	0	0	0	0	0	0	0	0	0	0	0	0
LAMB1	0	0	0	0	0	0	0	0	0	0	0	0	0
LAMC1	0	0	0	0	0	0	0	0	0	0	0	0	0
LGALS3	0	0	0	0	0	0	0	0	0	0	1	1	0

NAME	REACTOME_CLASS_I_MHC_MEDIATED_ANTIGEN_PROCESSING_PRESENTATION	REACTOME_APOPTOSIS	REACTOME_ANTIGEN_PROCESSING_UBIQUITINATION_PROTEASOME_DEGRADATION	REACTOME_ING_BY_WNT	REACTOME_REGULATION_OF_MRNA_STABILITY_BY_PROTEINS_THAT_BIND_AU_RICH_ELEMENTS	REACTOME_DESTABILIZATION_OF_MRNA_BY_AUF1_HNRNP_D0	REACTOME_REGULATION_OF_APOPTOSIS	REACTOME_REGULATION_OF_ORNITHINE_DECARBOXYLASE_ODC	REACTOME_CROSS_PRESENTATION_OF_SOLUBLE_EXOGENOUS_ANTIGENS_ENDOSOMES	REACTOME_VIF_MEDIATED_DEGRADATION_OF_APOBEC3G	REACTOME_REGULATION_OF_MITOTIC_CELL_CYCLE	REACTOME_APC_C_CDC20_MEDIATED_DEGRADATION_OF_MITOTIC_PROTEINS	REACTOME_CDT1_ASSOCIATION_WITH_THE_CDC6_ORC_ORIGIN_COMPLEX
GALNT2	0	0	0	0	0	0	0	0	0	0	0	0	0
GAPDH	0	0	0	0	0	0	0	0	0	0	0	0	0
GBF1	0	0	0	0	0	0	0	0	0	0	0	0	0
GLS	0	0	0	0	0	0	0	0	0	0	0	0	0
GMPPA	0	0	0	0	0	0	0	0	0	0	0	0	0
GMPPB	0	0	0	0	0	0	0	0	0	0	0	0	0
GNB2	0	0	0	0	0	0	0	0	0	0	0	0	0
GPC1	0	0	0	0	0	0	0	0	0	0	0	0	0
GSK3A	0	0	0	0	0	0	0	0	0	0	0	0	0
GYG1	0	0	0	0	0	0	0	0	0	0	0	0	0
HEBP1	0	0	0	0	0	0	0	0	0	0	0	0	0
HSP90AA1	0	0	0	0	0	0	0	0	0	0	0	0	0
HSP90B1	0	0	0	0	0	0	0	0	0	0	0	0	0
HSPA5	1	0	0	0	0	0	0	0	0	0	0	0	0
HSPA8	0	0	0	0	1	1	0	0	0	0	0	0	0
IGF2R	0	0	0	0	0	0	0	0	0	0	0	0	0
IPO5	0	0	0	0	0	0	0	0	0	0	0	0	0
ISG15	0	0	0	0	0	0	0	0	0	0	0	0	0
KIF3B	0	0	0	0	0	0	0	0	0	0	0	0	0
KIF5B	0	0	0	0	0	0	0	0	0	0	0	0	0
KPNA4	0	0	0	0	0	0	0	0	0	0	0	0	0
KPNB1	0	1	0	0	0	0	0	0	0	0	0	0	0
LAMB1	0	0	0	0	0	0	0	0	0	0	0	0	0
LAMC1	0	0	0	0	0	0	0	0	0	0	0	0	0
LGALS3	0	0	0	0	0	0	0	0	0	0	0	0	0

NAME	REACTOME_APC_C_CDH1_MEDIATED_DEGRADATION_OF_CDC20_AND_OTHER_APC_C_CDH1_TARGETED_PROTEINS_IN_LATE_MITOSIS_EARLY_G1	REACTOME_CYCLIN_E_ASSOCIATED_EVENTS_DURING_G1_S_TRANSITION_	REACTOME_SCFSKP2_MEDIATED_DEGRADATION_OF_P27_P21	REACTOME_AUTODEGRADATION_OF_CDH1_BY_CDH1_APC_C	REACTOME_AUTODEGRADATION_OF_THE_E3_UBIQUITIN_LIGASE_COP1	REACTOME_SCF_BETA_TRCP_MEDIATED_DEGRADATION_OF_EMI1	REACTOME_P53_INDEPENDENT_G1_S_DNA_DAMAGE_CHECKPOINT	REACTOME_CDK_MEDIATED_PHOSPHORYLATION_AND_REMOVAL_OF_CDC6	REACTOME_P53_DEPENDENT_G1_DNA_DAMAGE_RESPONSE	REACTOME_ACTIVATION_OF_NF_KAPPAB_IN_B_CELLS	REACTOME_DOWNSTREAM_ING_EVENTS_OF_B_CELL_RECEPTOR_BCR	REACTOME_ING_BY_THE_B_CELL_RECEPTOR_BCR	REACTOME_ANTIGEN_PROCESSING_CROSS_PRESENTATION
GALNT2	0	0	0	0	0	0	0	0	0	0	0	0	0
GAPDH	0	0	0	0	0	0	0	0	0	0	0	0	0
GBF1	0	0	0	0	0	0	0	0	0	0	0	0	0
GLS	0	0	0	0	0	0	0	0	0	0	0	0	0
GMPPA	0	0	0	0	0	0	0	0	0	0	0	0	0
GMPPB	0	0	0	0	0	0	0	0	0	0	0	0	0
GNB2	0	0	0	0	0	0	0	0	0	0	0	0	0
GPC1	0	0	0	0	0	0	0	0	0	0	0	0	0
GSK3A	0	0	0	0	0	0	0	0	0	0	1	1	0
GYG1	0	0	0	0	0	0	0	0	0	0	0	0	0
HEBP1	0	0	0	0	0	0	0	0	0	0	0	0	0
HSP90AA1	0	0	0	0	0	0	0	0	0	0	0	0	0
HSP90B1	0	0	0	0	0	0	0	0	0	0	0	0	0
HSPA5	0	0	0	0	0	0	0	0	0	0	0	0	0
HSPA8	0	0	0	0	0	0	0	0	0	0	0	0	0
IGF2R	0	0	0	0	0	0	0	0	0	0	0	0	0
IPO5	0	0	0	0	0	0	0	0	0	0	0	0	0
ISG15	0	0	0	0	0	0	0	0	0	0	0	0	0
KIF3B	0	0	0	0	0	0	0	0	0	0	0	0	0
KIF5B	0	0	0	0	0	0	0	0	0	0	0	0	0
KPNA4	0	0	0	0	0	0	0	0	0	0	0	0	0
KPNB1	0	0	0	0	0	0	0	0	0	0	0	0	0
LAMB1	0	0	0	0	0	0	0	0	0	0	0	0	0
LAMC1	0	0	0	0	0	0	0	0	0	0	0	0	0
LGALS3	0	0	0	0	0	0	0	0	0	0	0	0	0

NAME	REACTOME_ER_PHAGOSOME_PATHWAY	REACTOME_ING_BY_INSULIN_RECEPTOR	REACTOME_CELL_SURFACE_INTERACTIONS_AT_THE_VASCULAR_WALL	REACTOME_INTEGRIN_CELL_SURFACE_INTERACTIONS	REACTOME_RESPONSE_TO_ELEVATED_PLATELET_CYTOSOLIC_CA2_	REACTOME_HEMOSTASIS	REACTOME_PLATELET_ACTIVATION_ING_AND_AGGREGATION	REACTOME_SEMAPHORIN_INTERACTIONS	REACTOME_L1CAM_INTERACTIONS	REACTOME_DEVELOPMENTAL_BIOLOGY	REACTOME_AXON_GUIDANCE	REACTOME_INTEGRATION_OF_ENERGY_METABOLISM	REACTOME_REGULATION_OF_INSULIN_SECRETION
GALNT2	0	0	0	0	0	0	0	0	0	0	0	0	0
GAPDH	0	0	0	0	0	0	0	0	0	0	0	0	0
GBF1	0	0	0	0	0	0	0	0	0	0	0	0	0
GLS	0	0	0	0	0	0	0	0	0	0	0	0	0
GMPPA	0	0	0	0	0	0	0	0	0	0	0	0	0
GMPPB	0	0	0	0	0	0	0	0	0	0	0	0	0
GNB2	0	0	0	0	0	0	0	0	0	0	0	0	0
GPC1	0	0	0	0	0	0	0	0	0	1	1	0	0
GSK3A	0	0	0	0	0	0	0	0	0	0	0	0	0
GYG1	0	0	0	0	0	0	0	0	0	0	0	0	0
HEBP1	0	0	0	0	0	0	0	0	0	0	0	0	0
HSP90AA1	0	0	0	0	0	0	0	0	0	0	0	0	0
HSP90B1	0	0	0	0	0	0	0	0	0	0	0	0	0
HSPA5	0	0	0	0	0	0	1	0	0	0	0	0	0
HSPA8	0	0	0	0	0	0	0	0	0	0	0	0	0
IGF2R	0	0	0	0	0	0	0	0	0	0	0	0	0
IPO5	0	0	0	0	0	0	0	0	0	0	0	0	0
ISG15	0	0	0	0	0	0	0	0	0	0	0	0	0
KIF3B	0	0	0	0	0	0	0	0	0	0	0	0	0
KIF5B	0	0	0	0	0	1	0	0	0	0	0	0	0
KPNA4	0	0	0	0	0	0	0	0	0	0	0	0	0
KPNB1	0	0	0	0	0	0	0	0	0	0	0	0	0
LAMB1	0	0	0	0	0	0	0	0	1	0	0	0	0
LAMC1	0	0	0	0	0	0	0	0	1	0	0	0	0
LGALS3	0	0	0	0	0	0	0	0	0	0	0	0	0

NAME	REACTOME_ING_BY_ILS	REACTOME_GPCR_DOWNSTREAM_ING	REACTOME_ING_BY_GPCR	REACTOME_LING_BY_NGF	REACTOME_NGF_LING_VIA_TRKA_FROM_THE_PLASMA_MEMBRANE	REACTOME_ING_BY_EGFR_IN_CANCER	REACTOME_ING_BY_FGFR_IN_DISEASE	REACTOME_ING_BY_FGFR	REACTOME_ING_BY_ERBB2	REACTOME_DOWNSTREAM__TRANSDUCTION	REACTOME_ING_BY_PDGF	KEGG_RIBOSOME	KEGG_PROTEASOME
GALNT2	0	0	0	0	0	0	0	0	0	0	0	0	0
GAPDH	0	0	0	0	0	0	0	0	0	0	0	0	0
GBF1	0	0	0	0	0	0	0	0	0	0	0	0	0
GLS	0	0	0	0	0	0	0	0	0	0	0	0	0
GMPPA	0	0	0	0	0	0	0	0	0	0	0	0	0
GMPPB	0	0	0	0	0	0	0	0	0	0	0	0	0
GNB2	0	0	0	0	0	0	0	0	0	0	0	0	0
GPC1	0	0	0	0	0	0	0	0	0	0	0	0	0
GSK3A	0	0	0	0	0	0	0	0	0	0	0	0	0
GYG1	0	0	0	0	0	0	0	0	0	0	0	0	0
HEBP1	0	1	1	0	0	0	0	0	0	0	0	0	0
HSP90AA1	0	0	0	0	0	0	0	0	0	0	0	0	0
HSP90B1	0	0	0	0	0	0	0	0	0	0	0	0	0
HSPA5	0	0	0	0	0	0	0	0	0	0	0	0	0
HSPA8	0	0	0	0	0	0	0	0	0	0	0	0	0
IGF2R	0	0	0	0	0	0	0	0	0	0	0	0	0
IPO5	0	0	0	0	0	0	0	0	0	0	0	0	0
ISG15	0	0	0	0	0	0	0	0	0	0	0	0	0
KIF3B	0	0	0	0	0	0	0	0	0	0	0	0	0
KIF5B	0	0	0	0	0	0	0	0	0	0	0	0	0
KPNA4	0	0	0	0	0	0	0	0	0	0	0	0	0
KPNB1	0	0	0	0	0	0	0	0	0	0	0	0	0
LAMB1	0	0	0	0	0	0	0	0	0	0	0	0	0
LAMC1	0	0	0	0	0	0	0	0	0	0	0	0	0
LGALS3	0	0	0	0	0	0	0	0	0	0	0	0	0

NAME	KEGG_PYRUVATE_METABOLISM	KEGG_LYSOSOME	KEGG_ENDOCYTOSIS	KEGG_EPITHELIAL_CELL_ING_IN_HELICOBACTER_PYLORI_INFECTION	KEGG_AXON_GUIDANCE	KEGG_NEUROTROPHIN_ING_PATHWAY	KEGG_CHEMOKINE_ING_PATHWAY	KEGG_INSULIN_ING_PATHWAY	KEGG_PROSTATE_CANCER	KEGG_PATHWAYS_IN_CANCER	KEGG_ERBB_ING_PATHWAY	KEGG_MAPK_ING_PATHWAY	KEGG_PATHOGENIC_ESCHERICHIA_COLI_INFECTION
GALNT2	0	0	0	0	0	0	0	0	0	0	0	0	0
GAPDH	0	0	0	0	0	0	0	0	0	0	0	0	0
GBF1	0	0	0	0	0	0	0	0	0	0	0	0	0
GLS	0	0	0	0	0	0	0	0	0	0	0	0	0
GMPPA	0	0	0	0	0	0	0	0	0	0	0	0	0
GMPPB	0	0	0	0	0	0	0	0	0	0	0	0	0
GNB2	0	0	0	0	0	0	0	0	0	0	0	0	0
GPC1	0	0	0	0	0	0	0	0	0	0	0	0	0
GSK3A	0	0	0	0	0	0	0	0	0	0	0	0	0
GYG1	0	0	0	0	0	0	0	0	0	0	0	0	0
HEBP1	0	0	0	0	0	0	0	0	0	0	0	0	0
HSP90AA1	0	0	0	0	0	0	0	0	0	0	0	0	0
HSP90B1	0	0	0	0	0	0	0	0	0	0	0	0	0
HSPA5	0	0	0	0	0	0	0	0	0	0	0	0	0
HSPA8	0	0	0	0	0	0	0	0	0	0	0	0	0
IGF2R	0	0	0	0	0	0	0	0	0	0	0	0	0
IPO5	0	0	0	0	0	0	0	0	0	0	0	0	0
ISG15	0	0	0	0	0	0	0	0	0	0	0	0	0
KIF3B	0	0	0	0	0	0	0	0	0	0	0	0	0
KIF5B	0	0	0	0	0	0	0	0	0	0	0	0	0
KPNA4	0	0	0	0	0	0	0	0	0	0	0	0	0
KPNB1	0	0	0	0	0	0	0	0	0	0	0	0	0
LAMB1	0	0	0	0	0	0	0	0	0	0	0	0	0
LAMC1	0	0	0	0	0	0	0	0	0	0	0	0	0
LGALS3	0	0	0	0	0	0	0	0	0	0	0	0	0

NAME	KEGG_TIGHT_JUNCTION	KEGG_ADHERENS_JUNCTION	KEGG_LEUKOCYTE_TRANSENDOTHELIAL_MIGRATION	KEGG_ARRHYTHMOGENIC_RIGHT_VENTRICULAR_CARDIOMYOPATHY_ARVC	KEGG_ECM_RECEPTOR_INTERACTION	KEGG_FOCAL_ADHESION	KEGG_FC_GAMMA_R_MEDIATED_PHAGOCYTOSIS	KEGG_REGULATION_OF_ACTIN_CYTOSKELETON
GALNT2	0	0	0	0	0	0	0	0
GAPDH	0	0	0	0	0	0	0	0
GBF1	0	0	0	0	0	0	0	0
GLS	0	0	0	0	0	0	0	0
GMPPA	0	0	0	0	0	0	0	0
GMPPB	0	0	0	0	0	0	0	0
GNB2	0	0	0	0	0	0	0	0
GPC1	0	0	0	0	0	0	0	0
GSK3A	0	0	0	0	0	0	0	0
GYG1	0	0	0	0	0	0	0	0
HEBP1	0	0	0	0	0	0	0	0
HSP90AA1	0	0	0	0	0	0	0	0
HSP90B1	0	0	0	0	0	0	0	0
HSPA5	0	0	0	0	0	0	0	0
HSPA8	0	0	0	0	0	0	0	0
IGF2R	0	0	0	0	0	0	0	0
IPO5	0	0	0	0	0	0	0	0
ISG15	0	0	0	0	0	0	0	0
KIF3B	0	0	0	0	0	0	0	0
KIF5B	0	0	0	0	0	0	0	0
KPNA4	0	0	0	0	0	0	0	0
KPNB1	0	0	0	0	0	0	0	0
LAMB1	0	0	0	0	0	0	0	0
LAMC1	0	0	0	0	0	0	0	0
LGALS3	0	0	0	0	0	0	0	0

Table S6 The differential proteins contribute to the enrichment of the significant pathways for the public dataset

Core enrichment proteins in down-regulated pathways of hiPSCs/HFs (hiPSCs vs HFs)

NAME	REACTOME_GLUCOSE_METABOLISM	REACTOME_UNFOLDED_PROTEIN_RESPONSE	REACTOME_DIABETES_PATHWAYS	REACTOME_PROTEIN_FOLDING	REACTOME_PREFOLDIN_MEDIATED_TRANSFER_OF_SUBSTRATE_TO_CCT_TRIC	REACTOME_ASPARAGINE_N_LINKED_GLYCOSYLATION	REACTOME_POST_TRANSLATIONAL_PROTEIN_MODIFICATION	REACTOME_ACTIVATION_OF_THE_MRNA_UPON_BINDING_OF_THE_CAP_BINDING_COMPLEX_AND_EIFS_AND_SUBSEQUENT_BINDING_TO_43S	REACTOME_FORMATION_OF_THE_TERNARY_COMPLEX_AND_SUBSEQUENTLY_THE_43S_COMPLEX	REACTOME_METABOLISM_OF_PROTEINS	REACTOME_SRP_DEPENDENT_COTRANSLATIONAL_PROTEIN_TARGETING_TO_MEMBRANE	REACTOME_INFLUENZA_VIRAL_RNA_TRANSCRIPTION_AND_REPLICATION	REACTOME_NONSENSE_MEDIATED_DECAY_ENHANCED_BY_THE_EXON_JUNCTION_COMPLEX
LMAN1	0	0	0	0	0	1	1	0	0	1	0	0	0
LNPEP	0	0	0	0	0	0	0	0	0	0	0	0	0
MAVS	0	0	0	0	0	0	0	0	0	0	0	0	0
MGAT1	0	0	0	0	0	1	1	0	0	1	0	0	0
MRC2	0	0	0	0	0	0	0	0	0	0	0	0	0
MTOR	0	0	0	0	0	0	0	0	0	0	0	0	0
MYO18A	0	0	0	0	0	0	0	0	0	0	0	0	0
MYO5A	0	0	1	0	0	0	0	0	0	0	0	0	0
NAPA	0	0	0	0	0	0	0	0	0	0	0	0	0
NMT1	0	0	0	0	0	0	0	0	0	0	0	0	0
NPEPPS	0	0	0	0	0	0	0	0	0	0	0	0	0
NSF	0	0	0	0	0	0	0	0	0	0	0	0	0
PDIA3	0	0	0	0	0	0	0	0	0	1	0	0	0
PDIA5	0	1	1	0	0	0	0	0	0	0	0	0	0
PFDN1	0	0	0	1	1	0	0	0	0	1	0	0	0
PFDN2	0	0	0	1	1	0	0	0	0	1	0	0	0
PFDN4	0	0	0	1	1	0	0	0	0	1	0	0	0
PFDN5	0	0	0	1	1	0	0	0	0	1	0	0	0
PFDN6	0	0	0	1	1	0	0	0	0	1	0	0	0
PFKL	1	0	0	0	0	0	0	0	0	0	0	0	0
PFKP	1	0	0	0	0	0	0	0	0	0	0	0	0
PGM2	1	0	0	0	0	0	0	0	0	0	0	0	0
PICALM	0	0	0	0	0	0	0	0	0	0	0	0	0
PIGS	0	0	0	0	0	0	1	0	0	1	0	0	0
PIK3C3	0	0	0	0	0	0	0	0	0	0	0	0	0

NAME	REACTOME_PEPTIDE_CHAIN_ELONGATION	REACTOME_3_UTR_MEDIATED_TRANSLATIONAL_REGULATION	REACTOME_TRANSLATION	REACTOME_CELL_CELL_COMMUNICATION	REACTOME_GOLGI_ASSOCIATED_VESICLE_BIOGENESIS	REACTOME_TRANS_GOLGI_NETWORK_VESICLE_BUDDING	REACTOME_MEMBRANE_TRAFFICKING	REACTOME_TRANSMISSION_ACROSS_CHEMICAL_SYNAPSES	REACTOME_NEURONAL_SYSTEM	REACTOME_MHC_CLASS_II_ANTIGEN_PRESENTATION	REACTOME_INNATE_IMMUNE_SYSTEM	REACTOME_IMMUNE_SYSTEM	REACTOME_ADAPTIVE_IMMUNE_SYSTEM
LMAN1	0	0	0	0	0	0	0	0	0	0	0	0	0
LNPEP	0	0	0	0	0	0	0	0	0	0	0	1	1
MAVS	0	0	0	0	0	0	0	0	0	0	1	1	0
MGAT1	0	0	0	0	0	0	0	0	0	0	0	0	0
MRC2	0	0	0	0	0	0	0	0	0	0	0	1	1
MTOR	0	0	0	0	0	0	0	0	0	0	0	1	1
MYO18A	0	0	0	0	0	0	0	0	0	0	0	0	0
MYO5A	0	0	0	0	0	0	0	0	0	0	0	0	0
NAPA	0	0	0	0	1	1	1	0	0	0	0	0	0
NMT1	0	0	0	0	0	0	0	0	0	0	0	0	0
NPEPPS	0	0	0	0	0	0	0	0	0	0	0	1	1
NSF	0	0	0	0	0	0	0	1	1	0	0	0	0
PDIA3	0	0	0	0	0	0	0	0	0	0	0	0	0
PDIA5	0	0	0	0	0	0	0	0	0	0	0	0	0
PFDN1	0	0	0	0	0	0	0	0	0	0	0	0	0
PFDN2	0	0	0	0	0	0	0	0	0	0	0	0	0
PFDN4	0	0	0	0	0	0	0	0	0	0	0	0	0
PFDN5	0	0	0	0	0	0	0	0	0	0	0	0	0
PFDN6	0	0	0	0	0	0	0	0	0	0	0	0	0
PFKL	0	0	0	0	0	0	0	0	0	0	0	0	0
PFKP	0	0	0	0	0	0	0	0	0	0	0	0	0
PGM2	0	0	0	0	0	0	0	0	0	0	0	0	0
PICALM	0	0	0	0	1	1	1	0	0	0	0	0	0
PIGS	0	0	0	0	0	0	0	0	0	0	0	0	0
PIK3C3	0	0	0	0	0	0	0	0	0	0	1	1	0

NAME	REACTOME_CLASS_I_MHC_MEDIATED_ANTIGEN_PROCESSING_PRESENTATION	REACTOME_APOPTOSIS	REACTOME_ANTIGEN_PROCESSING_UBIQUITINATION_PROTEASOME_DEGRADATION	REACTOME_ING_BY_WNT	REACTOME_REGULATION_OF_MRNA_STABILITY_BY_PROTEINS_THAT_BIND_AU_RICH_ELEMENTS	REACTOME_DESTABILIZATION_OF_MRNA_BY_AUF1_HNRNP_D0	REACTOME_REGULATION_OF_APOPTOSIS	REACTOME_REGULATION_OF_ORNITHINE_DECARBOXYLASE_ODC	REACTOME_CROSS_PRESENTATION_OF_SOLUBLE_EXOGENOUS_ANTIGENS_ENDOSOMES	REACTOME_VIF_MEDIATED_DEGRADATION_OF_APOBEC3G	REACTOME_REGULATION_OF_MITOTIC_CELL_CYCLE	REACTOME_APC_C_CDC20_MEDIATED_DEGRADATION_OF_MITOTIC_PROTEINS	REACTOME_CDT1_ASSOCIATION_WITH_THE_CDC6_ORC_ORIGIN_COMPLEX
LMAN1	0	0	0	0	0	0	0	0	0	0	0	0	0
LNPEP	1	0	1	0	0	0	0	0	0	0	0	0	0
MAVS	0	0	0	0	0	0	0	0	0	0	0	0	0
MGAT1	0	0	0	0	0	0	0	0	0	0	0	0	0
MRC2	1	0	0	0	0	0	0	0	1	0	0	0	0
MTOR	0	0	0	0	0	0	0	0	0	0	0	0	0
MYO18A	0	0	0	0	0	0	0	0	0	0	0	0	0
MYO5A	0	0	0	0	0	0	0	0	0	0	0	0	0
NAPA	0	0	0	0	0	0	0	0	0	0	0	0	0
NMT1	0	1	0	0	0	0	0	0	0	0	0	0	0
NPEPPS	1	0	1	0	0	0	0	0	0	0	0	0	0
NSF	0	0	0	0	0	0	0	0	0	0	0	0	0
PDIA3	0	0	0	0	0	0	0	0	0	0	0	0	0
PDIA5	0	0	0	0	0	0	0	0	0	0	0	0	0
PFDN1	0	0	0	0	0	0	0	0	0	0	0	0	0
PFDN2	0	0	0	0	0	0	0	0	0	0	0	0	0
PFDN4	0	0	0	0	0	0	0	0	0	0	0	0	0
PFDN5	0	0	0	0	0	0	0	0	0	0	0	0	0
PFDN6	0	0	0	0	0	0	0	0	0	0	0	0	0
PFKL	0	0	0	0	0	0	0	0	0	0	0	0	0
PFKP	0	0	0	0	0	0	0	0	0	0	0	0	0
PGM2	0	0	0	0	0	0	0	0	0	0	0	0	0
PICALM	0	0	0	0	0	0	0	0	0	0	0	0	0
PIGS	0	0	0	0	0	0	0	0	0	0	0	0	0
PIK3C3	0	0	0	0	0	0	0	0	0	0	0	0	0

NAME	REACTOME_APC_C_CDH1_MEDIATED_DEGRADATION_OF_CDC20_AND_OTHER_APC_C_CDH1_TARGETED_PROTEINS_IN_LATE_MITOSIS_EARLY_G1	REACTOME_CYCLIN_E_ASSOCIATED_EVENTS_DURING_G1_S_TRANSITION_	REACTOME_SCFSKP2_MEDIATED_DEGRADATION_OF_P27_P21	REACTOME_AUTODEGRADATION_OF_CDH1_BY_CDH1_APC_C	REACTOME_AUTODEGRADATION_OF_THE_E3_UBIQUITIN_LIGASE_COP1	REACTOME_SCF_BETA_TRCP_MEDIATED_DEGRADATION_OF_EMI1	REACTOME_P53_INDEPENDENT_G1_S_DNA_DAMAGE_CHECKPOINT	REACTOME_CDK_MEDIATED_PHOSPHORYLATION_AND_REMOVAL_OF_CDC6	REACTOME_P53_DEPENDENT_G1_DNA_DAMAGE_RESPONSE	REACTOME_ACTIVATION_OF_NF_KAPPAB_IN_B_CELLS	REACTOME_DOWNSTREAM_ING_EVENTS_OF_B_CELL_RECEPTOR_BCR	REACTOME_ING_BY_THE_B_CELL_RECEPTOR_BCR	REACTOME_ANTIGEN_PROCESSING_CROSS_PRESENTATION
LMAN1	0	0	0	0	0	0	0	0	0	0	0	0	0
LNPEP	0	0	0	0	0	0	0	0	0	0	0	0	1
MAVS	0	0	0	0	0	0	0	0	0	0	0	0	0
MGAT1	0	0	0	0	0	0	0	0	0	0	0	0	0
MRC2	0	0	0	0	0	0	0	0	0	0	0	0	1
MTOR	0	0	0	0	0	0	0	0	0	0	1	1	0
MYO18A	0	0	0	0	0	0	0	0	0	0	0	0	0
MYO5A	0	0	0	0	0	0	0	0	0	0	0	0	0
NAPA	0	0	0	0	0	0	0	0	0	0	0	0	0
NMT1	0	0	0	0	0	0	0	0	0	0	0	0	0
NPEPPS	0	0	0	0	0	0	0	0	0	0	0	0	0
NSF	0	0	0	0	0	0	0	0	0	0	0	0	0
PDIA3	0	0	0	0	0	0	0	0	0	0	0	0	1
PDIA5	0	0	0	0	0	0	0	0	0	0	0	0	0
PFDN1	0	0	0	0	0	0	0	0	0	0	0	0	0
PFDN2	0	0	0	0	0	0	0	0	0	0	0	0	0
PFDN4	0	0	0	0	0	0	0	0	0	0	0	0	0
PFDN5	0	0	0	0	0	0	0	0	0	0	0	0	0
PFDN6	0	0	0	0	0	0	0	0	0	0	0	0	0
PFKL	0	0	0	0	0	0	0	0	0	0	0	0	0
PFKP	0	0	0	0	0	0	0	0	0	0	0	0	0
PGM2	0	0	0	0	0	0	0	0	0	0	0	0	0
PICALM	0	0	0	0	0	0	0	0	0	0	0	0	0
PIGS	0	0	0	0	0	0	0	0	0	0	0	0	0
PIK3C3	0	0	0	0	0	0	0	0	0	0	0	0	0

NAME	REACTOME_ER_PHAGOSOME_PATHWAY	REACTOME_ING_BY_INSULIN_RECEPTOR	REACTOME_CELL_SURFACE_INTERACTIONS_AT_THE_VASCULAR_WALL	REACTOME_INTEGRIN_CELL_SURFACE_INTERACTIONS	REACTOME_RESPONSE_TO_ELEVATED_PLATELET_CYTOSOLIC_CA2_	REACTOME_HEMOSTASIS	REACTOME_PLATELET_ACTIVATION_ING_AND_AGGREGATION	REACTOME_SEMAPHORIN_INTERACTIONS	REACTOME_L1CAM_INTERACTIONS	REACTOME_DEVELOPMENTAL_BIOLOGY	REACTOME_AXON_GUIDANCE	REACTOME_INTEGRATION_OF_ENERGY_METABOLISM	REACTOME_REGULATION_OF_INSULIN_SECRETION
LMAN1	0	0	0	0	0	0	0	0	0	0	0	0	0
LNPEP	0	0	0	0	0	0	0	0	0	0	0	0	0
MAVS	0	0	0	0	0	0	0	0	0	0	0	0	0
MGAT1	0	0	0	0	0	0	0	0	0	0	0	0	0
MRC2	0	0	0	0	0	0	0	0	0	0	0	0	0
MTOR	0	1	0	0	0	0	0	0	0	0	0	0	0
MYO18A	0	0	0	0	0	0	0	0	0	0	0	0	0
MYO5A	0	0	0	0	0	0	0	0	0	0	0	0	0
NAPA	0	0	0	0	0	0	0	0	0	0	0	0	0
NMT1	0	0	0	0	0	0	0	0	0	0	0	0	0
NPEPPS	0	0	0	0	0	0	0	0	0	0	0	0	0
NSF	0	0	0	0	0	0	0	0	0	0	0	0	0
PDIA3	1	0	0	0	0	0	0	0	0	0	0	0	0
PDIA5	0	0	0	0	0	0	0	0	0	0	0	0	0
PFDN1	0	0	0	0	0	0	0	0	0	0	0	0	0
PFDN2	0	0	0	0	0	0	0	0	0	0	0	0	0
PFDN4	0	0	0	0	0	0	0	0	0	0	0	0	0
PFDN5	0	0	0	0	0	0	0	0	0	0	0	0	0
PFDN6	0	0	0	0	0	0	0	0	0	0	0	0	0
PFKL	0	0	0	0	0	0	0	0	0	0	0	0	0
PFKP	0	0	0	0	0	0	0	0	0	0	0	0	0
PGM2	0	0	0	0	0	0	0	0	0	0	0	0	0
PICALM	0	0	0	0	0	0	0	0	0	0	0	0	0
PIGS	0	0	0	0	0	0	0	0	0	0	0	0	0
PIK3C3	0	1	0	0	0	0	0	0	0	0	0	0	0

NAME	REACTOME_ING_BY_ILS	REACTOME_GPCR_DOWNSTREAM_ING	REACTOME_ING_BY_GPCR	REACTOME_LING_BY_NGF	REACTOME_NGF_LING_VIA_TRKA_FROM_THE_PLASMA_MEMBRANE	REACTOME_ING_BY_EGFR_IN_CANCER	REACTOME_ING_BY_FGFR_IN_DISEASE	REACTOME_ING_BY_FGFR	REACTOME_ING_BY_ERBB2	REACTOME_DOWNSTREAM__TRANSDUCTION	REACTOME_ING_BY_PDGF	KEGG_RIBOSOME	KEGG_PROTEASOME
LMAN1	0	0	0	0	0	0	0	0	0	0	0	0	0
LNPEP	0	0	0	0	0	0	0	0	0	0	0	0	0
MAVS	0	0	0	0	0	0	0	0	0	0	0	0	0
MGAT1	0	0	0	0	0	0	0	0	0	0	0	0	0
MRC2	0	0	0	0	0	0	0	0	0	0	0	0	0
MTOR	0	0	0	0	0	0	0	0	0	0	0	0	0
MYO18A	0	0	0	0	0	0	1	0	0	0	0	0	0
MYO5A	0	0	0	0	0	0	0	0	0	0	0	0	0
NAPA	0	0	0	0	0	0	0	0	0	0	0	0	0
NMT1	0	0	0	0	0	0	0	0	0	0	0	0	0
NPEPPS	0	0	0	0	0	0	0	0	0	0	0	0	0
NSF	0	0	0	0	0	0	0	0	0	0	0	0	0
PDIA3	0	0	0	0	0	0	0	0	0	0	0	0	0
PDIA5	0	0	0	0	0	0	0	0	0	0	0	0	0
PFDN1	0	0	0	0	0	0	0	0	0	0	0	0	0
PFDN2	0	0	0	0	0	0	0	0	0	0	0	0	0
PFDN4	0	0	0	0	0	0	0	0	0	0	0	0	0
PFDN5	0	0	0	0	0	0	0	0	0	0	0	0	0
PFDN6	0	0	0	0	0	0	0	0	0	0	0	0	0
PFKL	0	0	0	0	0	0	0	0	0	0	0	0	0
PFKP	0	0	0	0	0	0	0	0	0	0	0	0	0
PGM2	0	0	0	0	0	0	0	0	0	0	0	0	0
PICALM	0	0	0	0	0	0	0	0	0	0	0	0	0
PIGS	0	0	0	0	0	0	0	0	0	0	0	0	0
PIK3C3	0	0	0	0	0	0	0	0	0	0	0	0	0

NAME	KEGG_PYRUVATE_METABOLISM	KEGG_LYSOSOME	KEGG_ENDOCYTOSIS	KEGG_EPITHELIAL_CELL_ING_IN_HELICOBACTER_PYLORI_INFECTION	KEGG_AXON_GUIDANCE	KEGG_NEUROTROPHIN_ING_PATHWAY	KEGG_CHEMOKINE_ING_PATHWAY	KEGG_INSULIN_ING_PATHWAY	KEGG_PROSTATE_CANCER	KEGG_PATHWAYS_IN_CANCER	KEGG_ERBB_ING_PATHWAY	KEGG_MAPK_ING_PATHWAY	KEGG_PATHOGENIC_ESCHERICHIA_COLI_INFECTION
LMAN1	0	0	0	0	0	0	0	0	0	0	0	0	0
LNPEP	0	0	0	0	0	0	0	0	0	0	0	0	0
MAVS	0	0	0	0	0	0	0	0	0	0	0	0	0
MGAT1	0	0	0	0	0	0	0	0	0	0	0	0	0
MRC2	0	0	0	0	0	0	0	0	0	0	0	0	0
MTOR	0	0	0	0	0	0	0	0	0	0	0	0	0
MYO18A	0	0	0	0	0	0	0	0	0	0	0	0	0
MYO5A	0	0	0	0	0	0	0	0	0	0	0	0	0
NAPA	0	0	0	0	0	0	0	0	0	0	0	0	0
NMT1	0	0	0	0	0	0	0	0	0	0	0	0	0
NPEPPS	0	0	0	0	0	0	0	0	0	0	0	0	0
NSF	0	0	0	0	0	0	0	0	0	0	0	0	0
PDIA3	0	0	0	0	0	0	0	0	0	0	0	0	0
PDIA5	0	0	0	0	0	0	0	0	0	0	0	0	0
PFDN1	0	0	0	0	0	0	0	0	0	0	0	0	0
PFDN2	0	0	0	0	0	0	0	0	0	0	0	0	0
PFDN4	0	0	0	0	0	0	0	0	0	0	0	0	0
PFDN5	0	0	0	0	0	0	0	0	0	0	0	0	0
PFDN6	0	0	0	0	0	0	0	0	0	0	0	0	0
PFKL	0	0	0	0	0	0	0	0	0	0	0	0	0
PFKP	0	0	0	0	0	0	0	0	0	0	0	0	0
PGM2	0	0	0	0	0	0	0	0	0	0	0	0	0
PICALM	0	0	0	0	0	0	0	0	0	0	0	0	0
PIGS	0	0	0	0	0	0	0	0	0	0	0	0	0
PIK3C3	0	0	0	0	0	0	0	0	0	0	0	0	0

NAME	KEGG_TIGHT_JUNCTION	KEGG_ADHERENS_JUNCTION	KEGG_LEUKOCYTE_TRANSENDOTHELIAL_MIGRATION	KEGG_ARRHYTHMOGENIC_RIGHT_VENTRICULAR_CARDIOMYOPATHY_ARVC	KEGG_ECM_RECEPTOR_INTERACTION	KEGG_FOCAL_ADHESION	KEGG_FC_GAMMA_R_MEDIATED_PHAGOCYTOSIS	KEGG_REGULATION_OF_ACTIN_CYTOSKELETON
LMAN1	0	0	0	0	0	0	0	0
LNPEP	0	0	0	0	0	0	0	0
MAVS	0	0	0	0	0	0	0	0
MGAT1	0	0	0	0	0	0	0	0
MRC2	0	0	0	0	0	0	0	0
MTOR	0	0	0	0	0	0	0	0
MYO18A	0	0	0	0	0	0	0	0
MYO5A	0	0	0	0	0	0	0	0
NAPA	0	0	0	0	0	0	0	0
NMT1	0	0	0	0	0	0	0	0
NPEPPS	0	0	0	0	0	0	0	0
NSF	0	0	0	0	0	0	0	0
PDIA3	0	0	0	0	0	0	0	0
PDIA5	0	0	0	0	0	0	0	0
PFDN1	0	0	0	0	0	0	0	0
PFDN2	0	0	0	0	0	0	0	0
PFDN4	0	0	0	0	0	0	0	0
PFDN5	0	0	0	0	0	0	0	0
PFDN6	0	0	0	0	0	0	0	0
PFKL	0	0	0	0	0	0	0	0
PFKP	0	0	0	0	0	0	0	0
PGM2	0	0	0	0	0	0	0	0
PICALM	0	0	0	0	0	0	0	0
PIGS	0	0	0	0	0	0	0	0
PIK3C3	0	0	0	0	0	0	0	0

Table S6 The differential proteins contribute to the enrichment of the significant pathways for the public dataset

Core enrichment proteins in down-regulated pathways of hiPSCs/HFs (hiPSCs vs HFs)

NAME	REACTOME_GLUCOSE_METABOLISM	REACTOME_UNFOLDED_PROTEIN_RESPONSE	REACTOME_DIABETES_PATHWAYS	REACTOME_PROTEIN_FOLDING	REACTOME_PREFOLDIN_MEDIATED_TRANSFER_OF_SUBSTRATE_TO_CCT_TRIC	REACTOME_ASPARAGINE_N_LINKED_GLYCOSYLATION	REACTOME_POST_TRANSLATIONAL_PROTEIN_MODIFICATION	REACTOME_ACTIVATION_OF_THE_MRNA_UPON_BINDING_OF_THE_CAP_BINDING_COMPLEX_AND_EIFS_AND_SUBSEQUENT_BINDING_TO_43S	REACTOME_FORMATION_OF_THE_TERNARY_COMPLEX_AND_SUBSEQUENTLY_THE_43S_COMPLEX	REACTOME_METABOLISM_OF_PROTEINS	REACTOME_SRP_DEPENDENT_COTRANSLATIONAL_PROTEIN_TARGETING_TO_MEMBRANE	REACTOME_INFLUENZA_VIRAL_RNA_TRANSCRIPTION_AND_REPLICATION	REACTOME_NONSENSE_MEDIATED_DECAY_ENHANCED_BY_THE_EXON_JUNCTION_COMPLEX
PIN1	0	0	0	0	0	0	0	0	0	0	0	0	0
PITPNA	0	0	0	0	0	0	0	0	0	0	0	0	0
PMM2	0	0	0	0	0	1	1	0	0	1	0	0	0
PPP2R2A	0	0	0	0	0	0	0	0	0	0	0	0	1
PPP2R5E	0	0	0	0	0	0	0	0	0	0	0	0	0
PRKCSH	0	0	0	0	0	1	1	0	0	1	0	0	0
PSMB5	0	0	0	0	0	0	0	0	0	0	0	0	0
PSMD5	0	0	0	0	0	0	0	0	0	0	0	0	0
PVR	0	0	0	0	0	0	0	0	0	0	0	0	0
PYCARD	0	0	0	0	0	0	0	0	0	0	0	0	0
RAB3A	0	0	0	0	0	0	0	0	0	0	0	0	0
RAB7A	0	0	0	0	0	0	0	0	0	0	0	0	0
RHOC	0	0	0	0	0	0	0	0	0	0	0	0	0
RHOG	0	0	0	0	0	0	0	0	0	0	0	0	0
RPN1	0	0	0	0	0	1	1	0	0	1	1	0	0
RPS14	0	0	0	0	0	0	0	1	1	1	1	1	1
RTN4	0	0	0	0	0	0	0	0	0	0	0	0	0
SEC11A	0	0	0	0	0	0	0	0	0	1	1	0	0
SEC23A	0	0	0	0	0	1	1	0	0	1	0	0	0
SEC24B	0	0	0	0	0	1	1	0	0	1	0	0	0
SEC24C	0	0	0	0	0	1	1	0	0	1	0	0	0
SEC24D	0	0	0	0	0	1	1	0	0	1	0	0	0
SEC61A1	0	0	0	0	0	0	0	0	0	1	1	0	0
SEC61B	0	0	0	0	0	0	0	0	0	1	1	0	0
SLC25A12	1	0	0	0	0	0	0	0	0	1	0	0	0

NAME	REACTOME_PEPTIDE_CHAIN_ELONGATION	REACTOME_3_UTR_MEDIATED_TRANSLATIONAL_REGULATION	REACTOME_TRANSLATION	REACTOME_CELL_CELL_COMMUNICATION	REACTOME_GOLGI_ASSOCIATED_VESICLE_BIOGENESIS	REACTOME_TRANS_GOLGI_NETWORK_VESICLE_BUDDING	REACTOME_MEMBRANE_TRAFFICKING	REACTOME_TRANSMISSION_ACROSS_CHEMICAL_SYNAPSES	REACTOME_NEURONAL_SYSTEM	REACTOME_MHC_CLASS_II_ANTIGEN_PRESENTATION	REACTOME_INNATE_IMMUNE_SYSTEM	REACTOME_IMMUNE_SYSTEM	REACTOME_ADAPTIVE_IMMUNE_SYSTEM
PIN1	0	0	0	0	0	0	0	0	0	0	1	1	0
PITPNA	0	0	0	0	0	0	0	0	0	0	0	0	0
PMM2	0	0	0	0	0	0	0	0	0	0	0	0	0
PPP2R2A	0	0	0	0	0	0	0	0	0	0	0	0	0
PPP2R5E	0	0	0	0	0	0	0	0	0	0	0	0	0
PRKCSH	0	0	0	0	0	0	0	0	0	0	1	1	0
PSMB5	0	0	0	0	0	0	0	0	0	0	0	0	0
PSMD5	0	0	0	0	0	0	0	0	0	0	0	1	1
PVR	0	0	0	1	0	0	0	0	0	0	0	1	1
PYCARD	0	0	0	0	0	0	0	0	0	0	1	1	0
RAB3A	0	0	0	0	0	0	0	1	1	0	0	0	0
RAB7A	0	0	0	0	0	0	0	0	0	0	0	1	1
RHOC	0	0	0	0	0	0	0	0	0	0	0	0	0
RHOG	0	0	0	0	0	0	0	0	0	0	0	0	0
RPN1	0	0	1	0	0	0	0	0	0	0	0	0	0
RPS14	1	1	1	0	0	0	0	0	0	0	0	0	0
RTN4	0	0	0	0	0	0	0	0	0	0	0	0	0
SEC11A	0	0	1	0	0	0	0	0	0	0	0	0	0
SEC23A	0	0	0	0	0	0	1	0	0	1	0	1	1
SEC24B	0	0	0	0	0	0	1	0	0	1	0	1	1
SEC24C	0	0	0	0	0	0	1	0	0	1	0	1	1
SEC24D	0	0	0	0	0	0	1	0	0	1	0	1	1
SEC61A1	0	0	1	0	0	0	0	0	0	0	0	1	1
SEC61B	0	0	1	0	0	0	0	0	0	0	0	1	1
SLC25A12	0	0	0	0	0	0	0	0	0	0	0	0	0

NAME	REACTOME_CLASS_I_MHC_MEDIATED_ANTIGEN_PROCESSING_PRESENTATION	REACTOME_APOPTOSIS	REACTOME_ANTIGEN_PROCESSING_UBIQUITINATION_PROTEASOME_DEGRADATION	REACTOME_ING_BY_WNT	REACTOME_REGULATION_OF_MRNA_STABILITY_BY_PROTEINS_THAT_BIND_AU_RICH_ELEMENTS	REACTOME_DESTABILIZATION_OF_MRNA_BY_AUF1_HNRNP_D0	REACTOME_REGULATION_OF_APOPTOSIS	REACTOME_REGULATION_OF_ORNITHINE_DECARBOXYLASE_ODC	REACTOME_CROSS_PRESENTATION_OF_SOLUBLE_EXOGENOUS_ANTIGENS_ENDOSOMES	REACTOME_VIF_MEDIATED_DEGRADATION_OF_APOBEC3G	REACTOME_REGULATION_OF_MITOTIC_CELL_CYCLE	REACTOME_APC_C_CDC20_MEDIATED_DEGRADATION_OF_MITOTIC_PROTEINS	REACTOME_CDT1_ASSOCIATION_WITH_THE_CDC6_ORC_ORIGIN_COMPLEX
PIN1	0	0	0	0	0	0	0	0	0	0	0	0	0
PITPNA	0	0	0	0	0	0	0	0	0	0	0	0	0
PMM2	0	0	0	0	0	0	0	0	0	0	0	0	0
PPP2R2A	0	0	0	0	0	0	0	0	0	0	0	0	0
PPP2R5E	0	0	0	1	0	0	0	0	0	0	0	0	0
PRKCSH	0	0	0	0	0	0	0	0	0	0	0	0	0
PSMB5	0	0	0	1	0	0	0	0	0	0	0	0	0
PSMD5	1	1	1	1	1	1	1	1	1	1	1	1	1
PVR	0	0	0	0	0	0	0	0	0	0	0	0	0
PYCARD	0	0	0	0	0	0	0	0	0	0	0	0	0
RAB3A	0	0	0	0	0	0	0	0	0	0	0	0	0
RAB7A	0	0	0	0	0	0	0	0	0	0	0	0	0
RHOC	0	0	0	0	0	0	0	0	0	0	0	0	0
RHOG	0	0	0	0	0	0	0	0	0	0	0	0	0
RPN1	0	0	0	0	0	0	0	0	0	0	0	0	0
RPS14	0	0	0	0	0	0	0	0	0	0	0	0	0
RTN4	0	0	0	0	0	0	0	0	0	0	0	0	0
SEC11A	0	0	0	0	0	0	0	0	0	0	0	0	0
SEC23A	1	0	0	0	0	0	0	0	0	0	0	0	0
SEC24B	1	0	0	0	0	0	0	0	0	0	0	0	0
SEC24C	1	0	0	0	0	0	0	0	0	0	0	0	0
SEC24D	1	0	0	0	0	0	0	0	0	0	0	0	0
SEC61A1	1	0	0	0	0	0	0	0	0	0	0	0	0
SEC61B	1	0	0	0	0	0	0	0	0	0	0	0	0
SLC25A12	0	0	0	0	0	0	0	0	0	0	0	0	0

NAME	REACTOME_APC_C_CDH1_MEDIATED_DEGRADATION_OF_CDC20_AND_OTHER_APC_C_CDH1_TARGETED_PROTEINS_IN_LATE_MITOSIS_EARLY_G1	REACTOME_CYCLIN_E_ASSOCIATED_EVENTS_DURING_G1_S_TRANSITION_	REACTOME_SCFSKP2_MEDIATED_DEGRADATION_OF_P27_P21	REACTOME_AUTODEGRADATION_OF_CDH1_BY_CDH1_APC_C	REACTOME_AUTODEGRADATION_OF_THE_E3_UBIQUITIN_LIGASE_COP1	REACTOME_SCF_BETA_TRCP_MEDIATED_DEGRADATION_OF_EMI1	REACTOME_P53_INDEPENDENT_G1_S_DNA_DAMAGE_CHECKPOINT	REACTOME_CDK_MEDIATED_PHOSPHORYLATION_AND_REMOVAL_OF_CDC6	REACTOME_P53_DEPENDENT_G1_DNA_DAMAGE_RESPONSE	REACTOME_ACTIVATION_OF_NF_KAPPAB_IN_B_CELLS	REACTOME_DOWNSTREAM_ING_EVENTS_OF_B_CELL_RECEPTOR_BCR	REACTOME_ING_BY_THE_B_CELL_RECEPTOR_BCR	REACTOME_ANTIGEN_PROCESSING_CROSS_PRESENTATION
PIN1	0	0	0	0	0	0	0	0	0	0	0	0	0
PITPNA	0	0	0	0	0	0	0	0	0	0	0	0	0
PMM2	0	0	0	0	0	0	0	0	0	0	0	0	0
PPP2R2A	0	0	0	0	0	0	0	0	0	0	0	0	0
PPP2R5E	0	0	0	0	0	0	0	0	0	0	0	0	0
PRKCSH	0	0	0	0	0	0	0	0	0	0	0	0	0
PSMB5	0	0	0	0	0	0	0	0	0	0	1	1	0
PSMD5	1	1	1	1	1	1	1	1	1	1	1	1	1
PVR	0	0	0	0	0	0	0	0	0	0	0	0	0
PYCARD	0	0	0	0	0	0	0	0	0	0	0	0	0
RAB3A	0	0	0	0	0	0	0	0	0	0	0	0	0
RAB7A	0	0	0	0	0	0	0	0	0	0	0	0	0
RHOC	0	0	0	0	0	0	0	0	0	0	0	0	0
RHOG	0	0	0	0	0	0	0	0	0	0	0	0	0
RPN1	0	0	0	0	0	0	0	0	0	0	0	0	0
RPS14	0	0	0	0	0	0	0	0	0	0	0	0	0
RTN4	0	0	0	0	0	0	0	0	0	0	0	0	0
SEC11A	0	0	0	0	0	0	0	0	0	0	0	0	0
SEC23A	0	0	0	0	0	0	0	0	0	0	0	0	0
SEC24B	0	0	0	0	0	0	0	0	0	0	0	0	0
SEC24C	0	0	0	0	0	0	0	0	0	0	0	0	0
SEC24D	0	0	0	0	0	0	0	0	0	0	0	0	0
SEC61A1	0	0	0	0	0	0	0	0	0	0	0	0	1
SEC61B	0	0	0	0	0	0	0	0	0	0	0	0	1
SLC25A12	0	0	0	0	0	0	0	0	0	0	0	0	0

NAME	REACTOME_ER_PHAGOSOME_PATHWAY	REACTOME_ING_BY_INSULIN_RECEPTOR	REACTOME_CELL_SURFACE_INTERACTIONS_AT_THE_VASCULAR_WALL	REACTOME_INTEGRIN_CELL_SURFACE_INTERACTIONS	REACTOME_RESPONSE_TO_ELEVATED_PLATELET_CYTOSOLIC_CA2_	REACTOME_HEMOSTASIS	REACTOME_PLATELET_ACTIVATION_ING_AND_AGGREGATION	REACTOME_SEMAPHORIN_INTERACTIONS	REACTOME_L1CAM_INTERACTIONS	REACTOME_DEVELOPMENTAL_BIOLOGY	REACTOME_AXON_GUIDANCE	REACTOME_INTEGRATION_OF_ENERGY_METABOLISM	REACTOME_REGULATION_OF_INSULIN_SECRETION
PIN1	0	0	0	0	0	0	0	0	0	0	0	0	0
PITPNA	0	0	0	0	0	0	0	0	0	1	0	0	0
PMM2	0	0	0	0	0	0	0	0	0	0	0	0	0
PPP2R2A	0	0	0	0	0	0	0	0	0	0	0	0	0
PPP2R5E	0	0	0	0	0	0	0	0	0	0	0	0	0
PRKCSH	0	0	0	0	0	0	0	0	0	0	0	0	0
PSMB5	0	0	0	0	0	0	0	0	0	0	0	0	0
PSMD5	1	0	0	0	0	0	0	0	0	0	0	0	0
PVR	0	0	0	0	0	0	0	0	0	0	0	0	0
PYCARD	0	0	0	0	0	0	0	0	0	0	0	0	0
RAB3A	0	0	0	0	0	0	0	0	0	0	0	0	0
RAB7A	0	0	0	0	0	0	0	0	0	0	0	0	0
RHOC	0	0	0	0	0	0	0	1	0	1	1	0	0
RHOG	0	0	0	0	0	1	1	1	0	1	1	0	0
RPN1	0	0	0	0	0	0	0	0	0	0	0	0	0
RPS14	0	0	0	0	0	0	0	0	0	0	0	0	0
RTN4	0	0	0	0	0	0	0	0	0	0	0	0	0
SEC11A	0	0	0	0	0	0	0	0	0	0	0	0	0
SEC23A	0	0	0	0	0	0	0	0	0	0	0	0	0
SEC24B	0	0	0	0	0	0	0	0	0	0	0	0	0
SEC24C	0	0	0	0	0	0	0	0	0	0	0	0	0
SEC24D	0	0	0	0	0	0	0	0	0	0	0	0	0
SEC61A1	1	0	0	0	0	0	0	0	0	0	0	0	0
SEC61B	1	0	0	0	0	0	0	0	0	0	0	0	0
SLC25A12	0	0	0	0	0	0	0	0	0	0	0	0	0

NAME	REACTOME_ING_BY_ILS	REACTOME_GPCR_DOWNSTREAM_ING	REACTOME_ING_BY_GPCR	REACTOME_LING_BY_NGF	REACTOME_NGF_LING_VIA_TRKA_FROM_THE_PLASMA_MEMBRANE	REACTOME_ING_BY_EGFR_IN_CANCER	REACTOME_ING_BY_FGFR_IN_DISEASE	REACTOME_ING_BY_FGFR	REACTOME_ING_BY_ERBB2	REACTOME_DOWNSTREAM__TRANSDUCTION	REACTOME_ING_BY_PDGF	KEGG_RIBOSOME	KEGG_PROTEASOME
PIN1	0	0	0	0	0	0	0	0	0	0	0	0	0
PITPNA	0	0	0	0	0	0	0	0	0	0	0	0	0
PMM2	0	0	0	0	0	0	0	0	0	0	0	0	0
PPP2R2A	0	0	0	0	0	0	0	0	0	0	0	0	0
PPP2R5E	0	0	0	0	0	0	0	0	0	0	0	0	0
PRKCSH	0	0	0	0	0	0	0	0	0	0	0	0	0
PSMB5	0	0	0	0	0	0	0	0	0	0	0	0	0
PSMD5	0	0	0	0	0	0	0	0	0	0	0	0	0
PVR	0	0	0	0	0	0	0	0	0	0	0	0	0
PYCARD	0	0	0	0	0	0	0	0	0	0	0	0	0
RAB3A	0	0	0	0	0	0	0	0	0	0	0	0	0
RAB7A	0	0	0	0	0	0	0	0	0	0	0	0	0
RHOC	0	1	1	0	0	0	0	0	0	0	0	0	0
RHOG	0	1	1	0	0	0	0	0	0	0	0	0	0
RPN1	0	0	0	0	0	0	0	0	0	0	0	0	0
RPS14	0	0	0	0	0	0	0	0	0	0	0	0	0
RTN4	0	0	0	1	0	0	0	0	0	0	0	0	0
SEC11A	0	0	0	0	0	0	0	0	0	0	0	0	0
SEC23A	0	0	0	0	0	0	0	0	0	0	0	0	0
SEC24B	0	0	0	0	0	0	0	0	0	0	0	0	0
SEC24C	0	0	0	0	0	0	0	0	0	0	0	0	0
SEC24D	0	0	0	0	0	0	0	0	0	0	0	0	0
SEC61A1	0	0	0	0	0	0	0	0	0	0	0	0	0
SEC61B	0	0	0	0	0	0	0	0	0	0	0	0	0
SLC25A12	0	0	0	0	0	0	0	0	0	0	0	0	0

NAME	KEGG_PYRUVATE_METABOLISM	KEGG_LYSOSOME	KEGG_ENDOCYTOSIS	KEGG_EPITHELIAL_CELL_ING_IN_HELICOBACTER_PYLORI_INFECTION	KEGG_AXON_GUIDANCE	KEGG_NEUROTROPHIN_ING_PATHWAY	KEGG_CHEMOKINE_ING_PATHWAY	KEGG_INSULIN_ING_PATHWAY	KEGG_PROSTATE_CANCER	KEGG_PATHWAYS_IN_CANCER	KEGG_ERBB_ING_PATHWAY	KEGG_MAPK_ING_PATHWAY	KEGG_PATHOGENIC_ESCHERICHIA_COLI_INFECTION
PIN1	0	0	0	0	0	0	0	0	0	0	0	0	0
PITPNA	0	0	0	0	0	0	0	0	0	0	0	0	0
PMM2	0	0	0	0	0	0	0	0	0	0	0	0	0
PPP2R2A	0	0	0	0	0	0	0	0	0	0	0	0	0
PPP2R5E	0	0	0	0	0	0	0	0	0	0	0	0	0
PRKCSH	0	0	0	0	0	0	0	0	0	0	0	0	0
PSMB5	0	0	0	0	0	0	0	0	0	0	0	0	0
PSMD5	0	0	0	0	0	0	0	0	0	0	0	0	0
PVR	0	0	0	0	0	0	0	0	0	0	0	0	0
PYCARD	0	0	0	0	0	0	0	0	0	0	0	0	0
RAB3A	0	0	0	0	0	0	0	0	0	0	0	0	0
RAB7A	0	0	0	0	0	0	0	0	0	0	0	0	0
RHOC	0	0	0	0	0	0	0	0	0	0	0	0	0
RHOG	0	0	0	0	0	0	0	0	0	0	0	0	0
RPN1	0	0	0	0	0	0	0	0	0	0	0	0	0
RPS14	0	0	0	0	0	0	0	0	0	0	0	0	0
RTN4	0	0	0	0	0	0	0	0	0	0	0	0	0
SEC11A	0	0	0	0	0	0	0	0	0	0	0	0	0
SEC23A	0	0	0	0	0	0	0	0	0	0	0	0	0
SEC24B	0	0	0	0	0	0	0	0	0	0	0	0	0
SEC24C	0	0	0	0	0	0	0	0	0	0	0	0	0
SEC24D	0	0	0	0	0	0	0	0	0	0	0	0	0
SEC61A1	0	0	0	0	0	0	0	0	0	0	0	0	0
SEC61B	0	0	0	0	0	0	0	0	0	0	0	0	0
SLC25A12	0	0	0	0	0	0	0	0	0	0	0	0	0

NAME	KEGG_TIGHT_JUNCTION	KEGG_ADHERENS_JUNCTION	KEGG_LEUKOCYTE_TRANSENDOTHELIAL_MIGRATION	KEGG_ARRHYTHMOGENIC_RIGHT_VENTRICULAR_CARDIOMYOPATHY_ARVC	KEGG_ECM_RECEPTOR_INTERACTION	KEGG_FOCAL_ADHESION	KEGG_FC_GAMMA_R_MEDIATED_PHAGOCYTOSIS	KEGG_REGULATION_OF_ACTIN_CYTOSKELETON
PIN1	0	0	0	0	0	0	0	0
PITPNA	0	0	0	0	0	0	0	0
PMM2	0	0	0	0	0	0	0	0
PPP2R2A	0	0	0	0	0	0	0	0
PPP2R5E	0	0	0	0	0	0	0	0
PRKCSH	0	0	0	0	0	0	0	0
PSMB5	0	0	0	0	0	0	0	0
PSMD5	0	0	0	0	0	0	0	0
PVR	0	0	0	0	0	0	0	0
PYCARD	0	0	0	0	0	0	0	0
RAB3A	0	0	0	0	0	0	0	0
RAB7A	0	0	0	0	0	0	0	0
RHOC	0	0	0	0	0	0	0	0
RHOG	0	0	0	0	0	0	0	0
RPN1	0	0	0	0	0	0	0	0
RPS14	0	0	0	0	0	0	0	0
RTN4	0	0	0	0	0	0	0	0
SEC11A	0	0	0	0	0	0	0	0
SEC23A	0	0	0	0	0	0	0	0
SEC24B	0	0	0	0	0	0	0	0
SEC24C	0	0	0	0	0	0	0	0
SEC24D	0	0	0	0	0	0	0	0
SEC61A1	0	0	0	0	0	0	0	0
SEC61B	0	0	0	0	0	0	0	0
SLC25A12	0	0	0	0	0	0	0	0

Table S6 The differential proteins contribute to the enrichment of the significant pathways for the public dataset

Core enrichment proteins in down-regulated pathways of hiPSCs/HFs (hiPSCs vs HFs)

NAME	REACTOME_GLUCOSE_METABOLISM	REACTOME_UNFOLDED_PROTEIN_RESPONSE	REACTOME_DIABETES_PATHWAYS	REACTOME_PROTEIN_FOLDING	REACTOME_PREFOLDIN_MEDIATED_TRANSFER_OF_SUBSTRATE_TO_CCT_TRIC	REACTOME_ASPARAGINE_N_LINKED_GLYCOSYLATION	REACTOME_POST_TRANSLATIONAL_PROTEIN_MODIFICATION	REACTOME_ACTIVATION_OF_THE_MRNA_UPON_BINDING_OF_THE_CAP_BINDING_COMPLEX_AND_EIFS_AND_SUBSEQUENT_BINDING_TO_43S	REACTOME_FORMATION_OF_THE_TERNARY_COMPLEX_AND_SUBSEQUENTLY_THE_43S_COMPLEX	REACTOME_METABOLISM_OF_PROTEINS	REACTOME_SRP_DEPENDENT_COTRANSLATIONAL_PROTEIN_TARGETING_TO_MEMBRANE	REACTOME_INFLUENZA_VIRAL_RNA_TRANSCRIPTION_AND_REPLICATION	REACTOME_NONSENSE_MEDIATED_DECAY_ENHANCED_BY_THE_EXON_JUNCTION_COMPLEX
SLC25A6	0	0	0	0	0	0	0	0	0	1	0	0	0
SNX9	0	0	0	0	0	0	0	0	0	0	0	0	0
SPARC	0	0	0	0	0	0	0	0	0	0	0	0	0
SPCS3	0	0	0	0	0	0	0	0	0	1	1	0	0
SQSTM1	0	0	0	0	0	0	0	0	0	0	0	0	0
SRP19	0	0	0	0	0	0	0	0	0	1	1	0	0
SRP68	0	0	0	0	0	0	0	0	0	1	1	0	0
SRP72	0	0	0	0	0	0	0	0	0	1	1	0	0
SRPR	0	1	1	0	0	0	0	0	0	1	1	0	0
SRPRB	0	1	1	0	0	0	0	0	0	1	1	0	0
SSR3	0	0	0	0	0	0	0	0	0	1	1	0	0
SSR4	0	0	0	0	0	0	0	0	0	1	1	0	0
STIM1	0	0	0	0	0	0	0	0	0	0	0	0	0
STT3A	0	0	0	0	0	1	1	0	0	1	0	0	0
STUB1	0	0	0	0	0	0	0	0	0	0	0	0	0
STX12	0	0	0	0	0	0	0	0	0	0	0	0	0
STX4	0	0	0	0	0	0	0	0	0	0	0	0	0
STX7	0	0	0	0	0	0	0	0	0	0	0	0	0
TAP1	0	0	0	0	0	0	0	0	0	0	0	0	0
TBC1D8B	0	0	0	0	0	0	0	0	0	0	0	0	0
TBCC	0	0	0	1	0	0	0	0	0	1	0	0	0
TCEB2	0	0	0	0	0	0	0	0	0	0	0	0	0
TCP1	0	0	0	1	1	0	0	0	0	1	0	0	0
TGOLN2	0	0	0	0	0	0	0	0	0	0	0	0	0
TRIM25	0	0	0	0	0	0	0	0	0	0	0	0	0

NAME	REACTOME_PEPTIDE_CHAIN_ELONGATION	REACTOME_3_UTR_MEDIATED_TRANSLATIONAL_REGULATION	REACTOME_TRANSLATION	REACTOME_CELL_CELL_COMMUNICATION	REACTOME_GOLGI_ASSOCIATED_VESICLE_BIOGENESIS	REACTOME_TRANS_GOLGI_NETWORK_VESICLE_BUDDING	REACTOME_MEMBRANE_TRAFFICKING	REACTOME_TRANSMISSION_ACROSS_CHEMICAL_SYNAPSES	REACTOME_NEURONAL_SYSTEM	REACTOME_MHC_CLASS_II_ANTIGEN_PRESENTATION	REACTOME_INNATE_IMMUNE_SYSTEM	REACTOME_IMMUNE_SYSTEM	REACTOME_ADAPTIVE_IMMUNE_SYSTEM
SLC25A6	0	0	0	0	0	0	0	0	0	0	0	0	0
SNX9	0	0	0	0	1	1	1	0	0	0	0	0	0
SPARC	0	0	0	0	0	0	0	0	0	0	0	0	0
SPCS3	0	0	1	0	0	0	0	0	0	0	0	0	0
SQSTM1	0	0	0	0	0	0	0	0	0	0	0	1	0
SRP19	0	0	1	0	0	0	0	0	0	0	0	0	0
SRP68	0	0	1	0	0	0	0	0	0	0	0	0	0
SRP72	0	0	1	0	0	0	0	0	0	0	0	0	0
SRPR	0	0	1	0	0	0	0	0	0	0	0	0	0
SRPRB	0	0	1	0	0	0	0	0	0	0	0	0	0
SSR3	0	0	1	0	0	0	0	0	0	0	0	0	0
SSR4	0	0	1	0	0	0	0	0	0	0	0	0	0
STIM1	0	0	0	0	0	0	0	0	0	0	0	1	1
STT3A	0	0	0	0	0	0	0	0	0	0	0	0	0
STUB1	0	0	0	0	0	0	0	0	0	0	0	1	1
STX12	0	0	0	0	0	0	0	0	1	0	0	0	0
STX4	0	0	0	0	0	1	1	0	1	0	0	0	0
STX7	0	0	0	0	0	0	0	0	1	0	0	0	0
TAP1	0	0	0	0	0	0	0	0	0	0	0	1	1
TBC1D8B	0	0	0	0	1	1	1	0	0	0	0	0	0
TBCC	0	0	0	0	0	0	0	0	0	0	0	0	0
TCEB2	0	0	0	0	0	0	0	0	0	0	0	1	1
TCP1	0	0	0	0	0	0	0	0	0	0	0	0	0
TGOLN2	0	0	0	0	1	1	1	0	0	0	0	0	0
TRIM25	0	0	0	0	0	0	0	0	0	0	1	1	0

NAME	REACTOME_CLASS_I_MHC_MEDIATED_ANTIGEN_PROCESSING_PRESENTATION	REACTOME_APOPTOSIS	REACTOME_ANTIGEN_PROCESSING_UBIQUITINATION_PROTEASOME_DEGRADATION	REACTOME_ING_BY_WNT	REACTOME_REGULATION_OF_MRNA_STABILITY_BY_PROTEINS_THAT_BIND_AU_RICH_ELEMENTS	REACTOME_DESTABILIZATION_OF_MRNA_BY_AUF1_HNRNP_D0	REACTOME_REGULATION_OF_APOPTOSIS	REACTOME_REGULATION_OF_ORNITHINE_DECARBOXYLASE_ODC	REACTOME_CROSS_PRESENTATION_OF_SOLUBLE_EXOGENOUS_ANTIGENS_ENDOSOMES	REACTOME_VIF_MEDIATED_DEGRADATION_OF_APOBEC3G	REACTOME_REGULATION_OF_MITOTIC_CELL_CYCLE	REACTOME_APC_C_CDC20_MEDIATED_DEGRADATION_OF_MITOTIC_PROTEINS	REACTOME_CDT1_ASSOCIATION_WITH_THE_CDC6_ORC_ORIGIN_COMPLEX
SLC25A6	0	0	0	0	0	0	0	0	0	0	0	0	0
SNX9	0	0	0	0	0	0	0	0	0	0	0	0	0
SPARC	0	0	0	0	0	0	0	0	0	0	0	0	0
SPCS3	0	0	0	0	0	0	0	0	0	0	0	0	0
SQSTM1	0	0	0	0	0	0	0	0	0	0	0	0	0
SRP19	0	0	0	0	0	0	0	0	0	0	0	0	0
SRP68	0	0	0	0	0	0	0	0	0	0	0	0	0
SRP72	0	0	0	0	0	0	0	0	0	0	0	0	0
SRPR	0	0	0	0	0	0	0	0	0	0	0	0	0
SRPRB	0	0	0	0	0	0	0	0	0	0	0	0	0
SSR3	0	0	0	0	0	0	0	0	0	0	0	0	0
SSR4	0	0	0	0	0	0	0	0	0	0	0	0	0
STIM1	0	0	0	0	0	0	0	0	0	0	0	0	0
STT3A	0	0	0	0	0	0	0	0	0	0	0	0	0
STUB1	1	0	1	0	0	0	0	0	0	0	0	0	0
STX12	0	0	0	0	0	0	0	0	0	0	0	0	0
STX4	0	0	0	0	0	0	0	0	0	0	0	0	0
STX7	0	0	0	0	0	0	0	0	0	0	0	0	0
TAP1	1	0	0	0	0	0	0	0	0	0	0	0	0
TBC1D8B	0	0	0	0	0	0	0	0	0	0	0	0	0
TBCC	0	0	0	0	0	0	0	0	0	0	0	0	0
TCEB2	1	0	1	0	0	0	0	0	0	1	0	0	0
TCP1	0	0	0	0	0	0	0	0	0	0	0	0	0
TGOLN2	0	0	0	0	0	0	0	0	0	0	0	0	0
TRIM25	0	0	0	0	0	0	0	0	0	0	0	0	0

NAME	REACTOME_APC_C_CDH1_MEDIATED_DEGRADATION_OF_CDC20_AND_OTHER_APC_C_CDH1_TARGETED_PROTEINS_IN_LATE_MITOSIS_EARLY_G1	REACTOME_CYCLIN_E_ASSOCIATED_EVENTS_DURING_G1_S_TRANSITION_	REACTOME_SCFSKP2_MEDIATED_DEGRADATION_OF_P27_P21	REACTOME_AUTODEGRADATION_OF_CDH1_BY_CDH1_APC_C	REACTOME_AUTODEGRADATION_OF_THE_E3_UBIQUITIN_LIGASE_COP1	REACTOME_SCF_BETA_TRCP_MEDIATED_DEGRADATION_OF_EMI1	REACTOME_P53_INDEPENDENT_G1_S_DNA_DAMAGE_CHECKPOINT	REACTOME_CDK_MEDIATED_PHOSPHORYLATION_AND_REMOVAL_OF_CDC6	REACTOME_P53_DEPENDENT_G1_DNA_DAMAGE_RESPONSE	REACTOME_ACTIVATION_OF_NF_KAPPAB_IN_B_CELLS	REACTOME_DOWNSTREAM_ING_EVENTS_OF_B_CELL_RECEPTOR_BCR	REACTOME_ING_BY_THE_B_CELL_RECEPTOR_BCR	REACTOME_ANTIGEN_PROCESSING_CROSS_PRESENTATION
SLC25A6	0	0	0	0	0	0	0	0	0	0	0	0	0
SNX9	0	0	0	0	0	0	0	0	0	0	0	0	0
SPARC	0	0	0	0	0	0	0	0	0	0	0	0	0
SPCS3	0	0	0	0	0	0	0	0	0	0	0	0	0
SQSTM1	0	0	0	0	0	0	0	0	0	0	0	0	0
SRP19	0	0	0	0	0	0	0	0	0	0	0	0	0
SRP68	0	0	0	0	0	0	0	0	0	0	0	0	0
SRP72	0	0	0	0	0	0	0	0	0	0	0	0	0
SRPR	0	0	0	0	0	0	0	0	0	0	0	0	0
SRPRB	0	0	0	0	0	0	0	0	0	0	0	0	0
SSR3	0	0	0	0	0	0	0	0	0	0	0	0	0
SSR4	0	0	0	0	0	0	0	0	0	0	0	0	0
STIM1	0	0	0	0	0	0	0	0	0	0	0	1	0
STT3A	0	0	0	0	0	0	0	0	0	0	0	0	0
STUB1	0	0	0	0	0	0	0	0	0	0	0	0	0
STX12	0	0	0	0	0	0	0	0	0	0	0	0	0
STX4	0	0	0	0	0	0	0	0	0	0	0	0	0
STX7	0	0	0	0	0	0	0	0	0	0	0	0	0
TAP1	0	0	0	0	0	0	0	0	0	0	0	0	1
TBC1D8B	0	0	0	0	0	0	0	0	0	0	0	0	0
TBCC	0	0	0	0	0	0	0	0	0	0	0	0	0
TCEB2	0	0	0	0	0	0	0	0	0	0	0	0	0
TCP1	0	0	0	0	0	0	0	0	0	0	0	0	0
TGOLN2	0	0	0	0	0	0	0	0	0	0	0	0	0
TRIM25	0	0	0	0	0	0	0	0	0	0	0	0	0

NAME	REACTOME_ER_PHAGOSOME_PATHWAY	REACTOME_ING_BY_INSULIN_RECEPTOR	REACTOME_CELL_SURFACE_INTERACTIONS_AT_THE_VASCULAR_WALL	REACTOME_INTEGRIN_CELL_SURFACE_INTERACTIONS	REACTOME_RESPONSE_TO_ELEVATED_PLATELET_CYTOSOLIC_CA2_	REACTOME_HEMOSTASIS	REACTOME_PLATELET_ACTIVATION_ING_AND_AGGREGATION	REACTOME_SEMAPHORIN_INTERACTIONS	REACTOME_L1CAM_INTERACTIONS	REACTOME_DEVELOPMENTAL_BIOLOGY	REACTOME_AXON_GUIDANCE	REACTOME_INTEGRATION_OF_ENERGY_METABOLISM	REACTOME_REGULATION_OF_INSULIN_SECRETION
SLC25A6	0	0	0	0	0	0	0	0	0	0	0	0	0
SNX9	0	0	0	0	0	0	0	0	0	0	0	0	0
SPARC	0	0	0	0	1	1	1	0	0	0	0	0	0
SPCS3	0	0	0	0	0	0	0	0	0	0	0	0	0
SQSTM1	0	0	0	0	0	0	0	0	0	0	0	0	0
SRP19	0	0	0	0	0	0	0	0	0	0	0	0	0
SRP68	0	0	0	0	0	0	0	0	0	0	0	0	0
SRP72	0	0	0	0	0	0	0	0	0	0	0	0	0
SRPR	0	0	0	0	0	0	0	0	0	0	0	0	0
SRPRB	0	0	0	0	0	0	0	0	0	0	0	0	0
SSR3	0	0	0	0	0	0	0	0	0	0	0	0	0
SSR4	0	0	0	0	0	0	0	0	0	0	0	0	0
STIM1	0	0	0	0	0	1	0	0	0	0	0	0	0
STT3A	0	0	0	0	0	0	0	0	0	0	0	0	0
STUB1	0	0	0	0	0	0	0	0	0	0	0	0	0
STX12	0	0	0	0	0	0	0	0	0	0	0	0	0
STX4	0	0	0	0	0	0	0	0	0	0	0	0	0
STX7	0	0	0	0	0	0	0	0	0	0	0	0	0
TAP1	1	0	0	0	0	0	0	0	0	0	0	0	0
TBC1D8B	0	0	0	0	0	0	0	0	0	0	0	0	0
TBCC	0	0	0	0	0	0	0	0	0	0	0	0	0
TCEB2	0	0	0	0	0	0	0	0	0	0	0	0	0
TCP1	0	0	0	0	0	0	0	0	0	0	0	0	0
TGOLN2	0	0	0	0	0	0	0	0	0	0	0	0	0
TRIM25	0	0	0	0	0	0	0	0	0	0	0	0	0

NAME	REACTOME_ING_BY_ILS	REACTOME_GPCR_DOWNSTREAM_ING	REACTOME_ING_BY_GPCR	REACTOME_LING_BY_NGF	REACTOME_NGF_LING_VIA_TRKA_FROM_THE_PLASMA_MEMBRANE	REACTOME_ING_BY_EGFR_IN_CANCER	REACTOME_ING_BY_FGFR_IN_DISEASE	REACTOME_ING_BY_FGFR	REACTOME_ING_BY_ERBB2	REACTOME_DOWNSTREAM__TRANSDUCTION	REACTOME_ING_BY_PDGF	KEGG_RIBOSOME	KEGG_PROTEASOME
SLC25A6	0	0	0	0	0	0	0	0	0	0	0	0	0
SNX9	0	0	0	0	0	0	0	0	0	0	0	0	0
SPARC	0	0	0	0	0	0	0	0	0	0	0	0	0
SPCS3	0	0	0	0	0	0	0	0	0	0	0	0	0
SQSTM1	1	0	0	1	0	0	0	0	0	0	0	0	0
SRP19	0	0	0	0	0	0	0	0	0	0	0	0	0
SRP68	0	0	0	0	0	0	0	0	0	0	0	0	0
SRP72	0	0	0	0	0	0	0	0	0	0	0	0	0
SRPR	0	0	0	0	0	0	0	0	0	0	0	0	0
SRPRB	0	0	0	0	0	0	0	0	0	0	0	0	0
SSR3	0	0	0	0	0	0	0	0	0	0	0	0	0
SSR4	0	0	0	0	0	0	0	0	0	0	0	0	0
STIM1	0	0	0	0	0	0	0	0	0	0	0	0	0
STT3A	0	0	0	0	0	0	0	0	0	0	0	0	0
STUB1	0	0	0	0	0	0	0	0	1	0	0	0	0
STX12	0	0	0	0	0	0	0	0	0	0	0	0	0
STX4	0	0	0	0	0	0	0	0	0	0	0	0	0
STX7	0	0	0	0	0	0	0	0	0	0	0	0	0
TAP1	0	0	0	0	0	0	0	0	0	0	0	0	0
TBC1D8B	0	0	0	0	0	0	0	0	0	0	0	0	0
TBCC	0	0	0	0	0	0	0	0	0	0	0	0	0
TCEB2	0	0	0	0	0	0	0	0	0	0	0	0	0
TCP1	0	0	0	0	0	0	0	0	0	0	0	0	0
TGOLN2	0	0	0	0	0	0	0	0	0	0	0	0	0
TRIM25	0	0	0	0	0	0	0	0	0	0	0	0	0

NAME	KEGG_PYRUVATE_METABOLISM	KEGG_LYSOSOME	KEGG_ENDOCYTOSIS	KEGG_EPITHELIAL_CELL_ING_IN_HELICOBACTER_PYLORI_INFECTION	KEGG_AXON_GUIDANCE	KEGG_NEUROTROPHIN_ING_PATHWAY	KEGG_CHEMOKINE_ING_PATHWAY	KEGG_INSULIN_ING_PATHWAY	KEGG_PROSTATE_CANCER	KEGG_PATHWAYS_IN_CANCER	KEGG_ERBB_ING_PATHWAY	KEGG_MAPK_ING_PATHWAY	KEGG_PATHOGENIC_ESCHERICHIA_COLI_INFECTION
SLC25A6	0	0	0	0	0	0	0	0	0	0	0	0	0
SNX9	0	0	0	0	0	0	0	0	0	0	0	0	0
SPARC	0	0	0	0	0	0	0	0	0	0	0	0	0
SPCS3	0	0	0	0	0	0	0	0	0	0	0	0	0
SQSTM1	0	0	0	0	0	0	0	0	0	0	0	0	0
SRP19	0	0	0	0	0	0	0	0	0	0	0	0	0
SRP68	0	0	0	0	0	0	0	0	0	0	0	0	0
SRP72	0	0	0	0	0	0	0	0	0	0	0	0	0
SRPR	0	0	0	0	0	0	0	0	0	0	0	0	0
SRPRB	0	0	0	0	0	0	0	0	0	0	0	0	0
SSR3	0	0	0	0	0	0	0	0	0	0	0	0	0
SSR4	0	0	0	0	0	0	0	0	0	0	0	0	0
STIM1	0	0	0	0	0	0	0	0	0	0	0	0	0
STT3A	0	0	0	0	0	0	0	0	0	0	0	0	0
STUB1	0	0	0	0	0	0	0	0	0	0	0	0	0
STX12	0	0	0	0	0	0	0	0	0	0	0	0	0
STX4	0	0	0	0	0	0	0	0	0	0	0	0	0
STX7	0	0	0	0	0	0	0	0	0	0	0	0	0
TAP1	0	0	0	0	0	0	0	0	0	0	0	0	0
TBC1D8B	0	0	0	0	0	0	0	0	0	0	0	0	0
TBCC	0	0	0	0	0	0	0	0	0	0	0	0	0
TCEB2	0	0	0	0	0	0	0	0	0	0	0	0	0
TCP1	0	0	0	0	0	0	0	0	0	0	0	0	0
TGOLN2	0	0	0	0	0	0	0	0	0	0	0	0	0
TRIM25	0	0	0	0	0	0	0	0	0	0	0	0	0

NAME	KEGG_TIGHT_JUNCTION	KEGG_ADHERENS_JUNCTION	KEGG_LEUKOCYTE_TRANSENDOTHELIAL_MIGRATION	KEGG_ARRHYTHMOGENIC_RIGHT_VENTRICULAR_CARDIOMYOPATHY_ARVC	KEGG_ECM_RECEPTOR_INTERACTION	KEGG_FOCAL_ADHESION	KEGG_FC_GAMMA_R_MEDIATED_PHAGOCYTOSIS	KEGG_REGULATION_OF_ACTIN_CYTOSKELETON
SLC25A6	0	0	0	0	0	0	0	0
SNX9	0	0	0	0	0	0	0	0
SPARC	0	0	0	0	0	0	0	0
SPCS3	0	0	0	0	0	0	0	0
SQSTM1	0	0	0	0	0	0	0	0
SRP19	0	0	0	0	0	0	0	0
SRP68	0	0	0	0	0	0	0	0
SRP72	0	0	0	0	0	0	0	0
SRPR	0	0	0	0	0	0	0	0
SRPRB	0	0	0	0	0	0	0	0
SSR3	0	0	0	0	0	0	0	0
SSR4	0	0	0	0	0	0	0	0
STIM1	0	0	0	0	0	0	0	0
STT3A	0	0	0	0	0	0	0	0
STUB1	0	0	0	0	0	0	0	0
STX12	0	0	0	0	0	0	0	0
STX4	0	0	0	0	0	0	0	0
STX7	0	0	0	0	0	0	0	0
TAP1	0	0	0	0	0	0	0	0
TBC1D8B	0	0	0	0	0	0	0	0
TBCC	0	0	0	0	0	0	0	0
TCEB2	0	0	0	0	0	0	0	0
TCP1	0	0	0	0	0	0	0	0
TGOLN2	0	0	0	0	0	0	0	0
TRIM25	0	0	0	0	0	0	0	0

Table S6 The differential proteins contribute to the enrichment of the significant pathways for the public dataset

Core enrichment proteins in down-regulated pathways of hiPSCs/HFs (hiPSCs vs HFs)

NAME	REACTOME_GLUCOSE_METABOLISM	REACTOME_UNFOLDED_PROTEIN_RESPONSE	REACTOME_DIABETES_PATHWAYS	REACTOME_PROTEIN_FOLDING	REACTOME_PREFOLDIN_MEDIATED_TRANSFER_OF_SUBSTRATE_TO_CCT_TRIC	REACTOME_ASPARAGINE_N_LINKED_GLYCOSYLATION	REACTOME_POST_TRANSLATIONAL_PROTEIN_MODIFICATION	REACTOME_ACTIVATION_OF_THE_MRNA_UPON_BINDING_OF_THE_CAP_BINDING_COMPLEX_AND_EIFS_AND_SUBSEQUENT_BINDING_TO_43S	REACTOME_FORMATION_OF_THE_TERNARY_COMPLEX_AND_SUBSEQUENTLY_THE_43S_COMPLEX	REACTOME_METABOLISM_OF_PROTEINS	REACTOME_SRP_DEPENDENT_COTRANSLATIONAL_PROTEIN_TARGETING_TO_MEMBRANE	REACTOME_INFLUENZA_VIRAL_RNA_TRANSCRIPTION_AND_REPLICATION	REACTOME_NONSENSE_MEDIATED_DECAY_ENHANCED_BY_THE_EXON_JUNCTION_COMPLEX
TXNDC5	0	0	0	0	0	0	0	0	0	0	0	0	0
UBA1	0	0	0	0	0	0	0	0	0	0	0	0	0
UBA6	0	0	0	0	0	0	0	0	0	0	0	0	0
UBE2M	0	0	0	0	0	0	0	0	0	0	0	0	0
UBR4	0	0	0	0	0	0	0	0	0	0	0	0	0
UGGT1	0	0	0	0	0	1	1	0	0	1	0	0	0
VAMP7	0	0	0	0	0	0	0	0	0	0	0	0	0
VBP1	0	0	0	1	1	0	0	0	0	1	0	0	0
VIM	0	0	0	0	0	0	0	0	0	0	0	0	0
VKORC1	0	0	0	0	0	0	1	0	0	1	0	0	0
WDR1	0	0	0	0	0	0	0	0	0	0	0	0	0
WFS1	0	1	1	0	0	0	0	0	0	0	0	0	0
ABL1	0	0	0	0	0	0	0	0	0	0	0	0	0
ACAT1	0	0	0	0	0	0	0	0	0	0	0	0	0
AGA	0	0	0	0	0	0	0	0	0	0	0	0	0
ARF6	0	0	0	0	0	0	0	0	0	0	0	0	0
ARFGAP3	0	0	0	0	0	0	0	0	0	0	0	0	0
ARPC1A	0	0	0	0	0	0	0	0	0	0	0	0	0
ARPC1B	0	0	0	0	0	0	0	0	0	0	0	0	0
ARPC2	0	0	0	0	0	0	0	0	0	0	0	0	0
ARPC4	0	0	0	0	0	0	0	0	0	0	0	0	0
ARPC5	0	0	0	0	0	0	0	0	0	0	0	0	0
ARPC5L	0	0	0	0	0	0	0	0	0	0	0	0	0
CAPN2	0	0	0	0	0	0	0	0	0	0	0	0	0
CD99	0	0	0	0	0	0	0	0	0	0	0	0	0

NAME	REACTOME_PEPTIDE_CHAIN_ELONGATION	REACTOME_3_UTR_MEDIATED_TRANSLATIONAL_REGULATION	REACTOME_TRANSLATION	REACTOME_CELL_CELL_COMMUNICATION	REACTOME_GOLGI_ASSOCIATED_VESICLE_BIOGENESIS	REACTOME_TRANS_GOLGI_NETWORK_VESICLE_BUDDING	REACTOME_MEMBRANE_TRAFFICKING	REACTOME_TRANSMISSION_ACROSS_CHEMICAL_SYNAPSES	REACTOME_NEURONAL_SYSTEM	REACTOME_MHC_CLASS_II_ANTIGEN_PRESENTATION	REACTOME_INNATE_IMMUNE_SYSTEM	REACTOME_IMMUNE_SYSTEM	REACTOME_ADAPTIVE_IMMUNE_SYSTEM
TXNDC5	0	0	0	0	1	1	1	0	0	0	0	0	0
UBA1	0	0	0	0	0	0	0	0	0	0	0	1	1
UBA6	0	0	0	0	0	0	0	0	0	0	0	1	1
UBE2M	0	0	0	0	0	0	0	0	0	0	0	1	1
UBR4	0	0	0	0	0	0	0	0	0	0	0	1	1
UGGT1	0	0	0	0	0	0	0	0	0	0	0	0	0
VAMP7	0	0	0	0	1	1	0	0	0	0	0	0	0
VBP1	0	0	0	0	0	0	0	0	0	0	0	0	0
VIM	0	0	0	0	0	0	0	0	0	0	0	0	0
VKORC1	0	0	0	0	0	0	0	0	0	0	0	0	0
WDR1	0	0	0	0	0	0	0	0	0	0	0	0	0
WFS1	0	0	0	0	0	0	0	0	0	0	0	0	0
ABL1	0	0	0	0	0	0	0	0	0	0	0	0	0
ACAT1	0	0	0	0	0	0	0	0	0	0	0	0	0
AGA	0	0	0	0	0	0	0	0	0	0	0	0	0
ARF6	0	0	0	0	0	0	0	0	0	0	0	0	0
ARFGAP3	0	0	0	0	0	0	0	0	0	0	0	0	0
ARPC1A	0	0	0	0	0	0	0	0	0	0	0	0	0
ARPC1B	0	0	0	0	0	0	0	0	0	0	0	0	0
ARPC2	0	0	0	0	0	0	0	0	0	0	0	0	0
ARPC4	0	0	0	0	0	0	0	0	0	0	0	0	0
ARPC5	0	0	0	0	0	0	0	0	0	0	0	0	0
ARPC5L	0	0	0	0	0	0	0	0	0	0	0	0	0
CAPN2	0	0	0	0	0	0	0	0	0	0	0	0	0
CD99	0	0	0	0	0	0	0	0	0	0	0	0	0

NAME	REACTOME_CLASS_I_MHC_MEDIATED_ANTIGEN_PROCESSING_PRESENTATION	REACTOME_APOPTOSIS	REACTOME_ANTIGEN_PROCESSING_UBIQUITINATION_PROTEASOME_DEGRADATION	REACTOME_ING_BY_WNT	REACTOME_REGULATION_OF_MRNA_STABILITY_BY_PROTEINS_THAT_BIND_AU_RICH_ELEMENTS	REACTOME_DESTABILIZATION_OF_MRNA_BY_AUF1_HNRNP_D0	REACTOME_REGULATION_OF_APOPTOSIS	REACTOME_REGULATION_OF_ORNITHINE_DECARBOXYLASE_ODC	REACTOME_CROSS_PRESENTATION_OF_SOLUBLE_EXOGENOUS_ANTIGENS_ENDOSOMES	REACTOME_VIF_MEDIATED_DEGRADATION_OF_APOBEC3G	REACTOME_REGULATION_OF_MITOTIC_CELL_CYCLE	REACTOME_APC_C_CDC20_MEDIATED_DEGRADATION_OF_MITOTIC_PROTEINS	REACTOME_CDT1_ASSOCIATION_WITH_THE_CDC6_ORC_ORIGIN_COMPLEX
TXNDC5	0	0	0	0	0	0	0	0	0	0	0	0	0
UBA1	1	0	1	0	0	0	0	0	0	0	0	0	0
UBA6	1	0	1	0	0	0	0	0	0	0	0	0	0
UBE2M	1	0	1	0	0	0	0	0	0	0	0	0	0
UBR4	1	0	1	0	0	0	0	0	0	0	0	0	0
UGGT1	0	0	0	0	0	0	0	0	0	0	0	0	0
VAMP7	0	0	0	0	0	0	0	0	0	0	0	0	0
VBP1	0	0	0	0	0	0	0	0	0	0	0	0	0
VIM	0	1	0	0	0	0	0	0	0	0	0	0	0
VKORC1	0	0	0	0	0	0	0	0	0	0	0	0	0
WDR1	0	0	0	0	0	0	0	0	0	0	0	0	0
WFS1	0	0	0	0	0	0	0	0	0	0	0	0	0
ABL1	0	0	0	0	0	0	0	0	0	0	0	0	0
ACAT1	0	0	0	0	0	0	0	0	0	0	0	0	0
AGA	0	0	0	0	0	0	0	0	0	0	0	0	0
ARF6	0	0	0	0	0	0	0	0	0	0	0	0	0
ARFGAP3	0	0	0	0	0	0	0	0	0	0	0	0	0
ARPC1A	0	0	0	0	0	0	0	0	0	0	0	0	0
ARPC1B	0	0	0	0	0	0	0	0	0	0	0	0	0
ARPC2	0	0	0	0	0	0	0	0	0	0	0	0	0
ARPC4	0	0	0	0	0	0	0	0	0	0	0	0	0
ARPC5	0	0	0	0	0	0	0	0	0	0	0	0	0
ARPC5L	0	0	0	0	0	0	0	0	0	0	0	0	0
CAPN2	0	0	0	0	0	0	0	0	0	0	0	0	0
CD99	0	0	0	0	0	0	0	0	0	0	0	0	0

NAME	REACTOME_APC_C_CDH1_MEDIATED_DEGRADATION_OF_CDC20_AND_OTHER_APC_C_CDH1_TARGETED_PROTEINS_IN_LATE_MITOSIS_EARLY_G1	REACTOME_CYCLIN_E_ASSOCIATED_EVENTS_DURING_G1_S_TRANSITION_	REACTOME_SCFSKP2_MEDIATED_DEGRADATION_OF_P27_P21	REACTOME_AUTODEGRADATION_OF_CDH1_BY_CDH1_APC_C	REACTOME_AUTODEGRADATION_OF_THE_E3_UBIQUITIN_LIGASE_COP1	REACTOME_SCF_BETA_TRCP_MEDIATED_DEGRADATION_OF_EMI1	REACTOME_P53_INDEPENDENT_G1_S_DNA_DAMAGE_CHECKPOINT	REACTOME_CDK_MEDIATED_PHOSPHORYLATION_AND_REMOVAL_OF_CDC6	REACTOME_P53_DEPENDENT_G1_DNA_DAMAGE_RESPONSE	REACTOME_ACTIVATION_OF_NF_KAPPAB_IN_B_CELLS	REACTOME_DOWNSTREAM_ING_EVENTS_OF_B_CELL_RECEPTOR_BCR	REACTOME_ING_BY_THE_B_CELL_RECEPTOR_BCR	REACTOME_ANTIGEN_PROCESSING_CROSS_PRESENTATION
TXNDC5	0	0	0	0	0	0	0	0	0	0	0	0	0
UBA1	0	0	0	0	0	0	0	0	0	0	0	0	0
UBA6	0	0	0	0	0	0	0	0	0	0	0	0	0
UBE2M	0	0	0	0	0	0	0	0	0	0	0	0	0
UBR4	0	0	0	0	0	0	0	0	0	0	0	0	0
UGGT1	0	0	0	0	0	0	0	0	0	0	0	0	0
VAMP7	0	0	0	0	0	0	0	0	0	0	0	0	0
VBP1	0	0	0	0	0	0	0	0	0	0	0	0	0
VIM	0	0	0	0	0	0	0	0	0	0	0	0	0
VKORC1	0	0	0	0	0	0	0	0	0	0	0	0	0
WDR1	0	0	0	0	0	0	0	0	0	0	0	0	0
WFS1	0	0	0	0	0	0	0	0	0	0	0	0	0
ABL1	0	0	0	0	0	0	0	0	0	0	0	0	0
ACAT1	0	0	0	0	0	0	0	0	0	0	0	0	0
AGA	0	0	0	0	0	0	0	0	0	0	0	0	0
ARF6	0	0	0	0	0	0	0	0	0	0	0	0	0
ARFGAP3	0	0	0	0	0	0	0	0	0	0	0	0	0
ARPC1A	0	0	0	0	0	0	0	0	0	0	0	0	0
ARPC1B	0	0	0	0	0	0	0	0	0	0	0	0	0
ARPC2	0	0	0	0	0	0	0	0	0	0	0	0	0
ARPC4	0	0	0	0	0	0	0	0	0	0	0	0	0
ARPC5	0	0	0	0	0	0	0	0	0	0	0	0	0
ARPC5L	0	0	0	0	0	0	0	0	0	0	0	0	0
CAPN2	0	0	0	0	0	0	0	0	0	0	0	0	0
CD99	0	0	0	0	0	0	0	0	0	0	0	0	0

NAME	REACTOME_ER_PHAGOSOME_PATHWAY	REACTOME_ING_BY_INSULIN_RECEPTOR	REACTOME_CELL_SURFACE_INTERACTIONS_AT_THE_VASCULAR_WALL	REACTOME_INTEGRIN_CELL_SURFACE_INTERACTIONS	REACTOME_RESPONSE_TO_ELEVATED_PLATELET_CYTOSOLIC_CA2_	REACTOME_HEMOSTASIS	REACTOME_PLATELET_ACTIVATION_ING_AND_AGGREGATION	REACTOME_SEMAPHORIN_INTERACTIONS	REACTOME_L1CAM_INTERACTIONS	REACTOME_DEVELOPMENTAL_BIOLOGY	REACTOME_AXON_GUIDANCE	REACTOME_INTEGRATION_OF_ENERGY_METABOLISM	REACTOME_REGULATION_OF_INSULIN_SECRETION
TXNDC5	0	0	0	0	0	0	0	0	0	0	0	0	0
UBA1	0	0	0	0	0	0	0	0	0	0	0	0	0
UBA6	0	0	0	0	0	0	0	0	0	0	0	0	0
UBE2M	0	0	0	0	0	0	0	0	0	0	0	0	0
UBR4	0	0	0	0	0	0	0	0	0	0	0	0	0
UGGT1	0	0	0	0	0	0	0	0	0	0	0	0	0
VAMP7	0	0	0	0	0	0	0	0	0	0	0	0	0
VBP1	0	0	0	0	0	0	0	0	0	0	0	0	0
VIM	0	0	0	0	0	0	0	0	0	0	0	0	0
VKORC1	0	0	0	0	0	0	0	0	0	0	0	0	0
WDR1	0	0	0	0	1	1	1	0	0	0	0	0	0
WFS1	0	0	0	0	0	0	0	0	0	0	0	0	0
ABL1	0	0	0	0	0	0	0	0	0	0	0	0	0
ACAT1	0	0	0	0	0	0	0	0	0	0	0	0	0
AGA	0	0	0	0	0	0	0	0	0	0	0	0	0
ARF6	0	0	0	0	0	0	0	0	0	0	0	0	0
ARFGAP3	0	0	0	0	0	0	0	0	0	0	0	0	0
ARPC1A	0	0	0	0	0	0	0	0	0	0	0	0	0
ARPC1B	0	0	0	0	0	0	0	0	0	0	0	0	0
ARPC2	0	0	0	0	0	0	0	0	0	0	0	0	0
ARPC4	0	0	0	0	0	0	0	0	0	0	0	0	0
ARPC5	0	0	0	0	0	0	0	0	0	0	0	0	0
ARPC5L	0	0	0	0	0	0	0	0	0	0	0	0	0
CAPN2	0	0	0	0	0	0	0	0	0	0	0	0	0
CD99	0	0	0	0	0	0	0	0	0	0	0	0	0

NAME	REACTOME_ING_BY_ILS	REACTOME_GPCR_DOWNSTREAM_ING	REACTOME_ING_BY_GPCR	REACTOME_LING_BY_NGF	REACTOME_NGF_LING_VIA_TRKA_FROM_THE_PLASMA_MEMBRANE	REACTOME_ING_BY_EGFR_IN_CANCER	REACTOME_ING_BY_FGFR_IN_DISEASE	REACTOME_ING_BY_FGFR	REACTOME_ING_BY_ERBB2	REACTOME_DOWNSTREAM__TRANSDUCTION	REACTOME_ING_BY_PDGF	KEGG_RIBOSOME	KEGG_PROTEASOME
TXNDC5	0	0	0	0	0	0	0	0	0	0	0	0	0
UBA1	0	0	0	0	0	0	0	0	0	0	0	0	0
UBA6	0	0	0	0	0	0	0	0	0	0	0	0	0
UBE2M	0	0	0	0	0	0	0	0	0	0	0	0	0
UBR4	0	0	0	0	0	0	0	0	0	0	0	0	0
UGGT1	0	0	0	0	0	0	0	0	0	0	0	0	0
VAMP7	0	0	0	0	0	0	0	0	0	0	0	0	0
VBP1	0	0	0	0	0	0	0	0	0	0	0	0	0
VIM	0	0	0	0	0	0	0	0	0	0	0	0	0
VKORC1	0	0	0	0	0	0	0	0	0	0	0	0	0
WDR1	0	0	0	0	0	0	0	0	0	0	0	0	0
WFS1	0	0	0	0	0	0	0	0	0	0	0	0	0
ABL1	0	0	0	0	0	0	0	0	0	0	0	0	0
ACAT1	0	0	0	0	0	0	0	0	0	0	0	0	0
AGA	0	0	0	0	0	0	0	0	0	0	0	0	0
ARF6	0	0	0	0	0	0	0	0	0	0	0	0	0
ARFGAP3	0	0	0	0	0	0	0	0	0	0	0	0	0
ARPC1A	0	0	0	0	0	0	0	0	0	0	0	0	0
ARPC1B	0	0	0	0	0	0	0	0	0	0	0	0	0
ARPC2	0	0	0	0	0	0	0	0	0	0	0	0	0
ARPC4	0	0	0	0	0	0	0	0	0	0	0	0	0
ARPC5	0	0	0	0	0	0	0	0	0	0	0	0	0
ARPC5L	0	0	0	0	0	0	0	0	0	0	0	0	0
CAPN2	0	0	0	0	0	0	0	0	0	0	0	0	0
CD99	0	0	0	0	0	0	0	0	0	0	0	0	0

NAME	KEGG_PYRUVATE_METABOLISM	KEGG_LYSOSOME	KEGG_ENDOCYTOSIS	KEGG_EPITHELIAL_CELL_ING_IN_HELICOBACTER_PYLORI_INFECTION	KEGG_AXON_GUIDANCE	KEGG_NEUROTROPHIN_ING_PATHWAY	KEGG_CHEMOKINE_ING_PATHWAY	KEGG_INSULIN_ING_PATHWAY	KEGG_PROSTATE_CANCER	KEGG_PATHWAYS_IN_CANCER	KEGG_ERBB_ING_PATHWAY	KEGG_MAPK_ING_PATHWAY	KEGG_PATHOGENIC_ESCHERICHIA_COLI_INFECTION
TXNDC5	0	0	0	0	0	0	0	0	0	0	0	0	0
UBA1	0	0	0	0	0	0	0	0	0	0	0	0	0
UBA6	0	0	0	0	0	0	0	0	0	0	0	0	0
UBE2M	0	0	0	0	0	0	0	0	0	0	0	0	0
UBR4	0	0	0	0	0	0	0	0	0	0	0	0	0
UGGT1	0	0	0	0	0	0	0	0	0	0	0	0	0
VAMP7	0	0	0	0	0	0	0	0	0	0	0	0	0
VBP1	0	0	0	0	0	0	0	0	0	0	0	0	0
VIM	0	0	0	0	0	0	0	0	0	0	0	0	0
VKORC1	0	0	0	0	0	0	0	0	0	0	0	0	0
WDR1	0	0	0	0	0	0	0	0	0	0	0	0	0
WFS1	0	0	0	0	0	0	0	0	0	0	0	0	0
ABL1	0	0	0	0	0	0	0	0	0	0	0	0	0
ACAT1	0	0	0	0	0	0	0	0	0	0	0	0	0
AGA	0	0	0	0	0	0	0	0	0	0	0	0	0
ARF6	0	0	0	0	0	0	0	0	0	0	0	0	0
ARFGAP3	0	0	0	0	0	0	0	0	0	0	0	0	0
ARPC1A	0	0	0	0	0	0	0	0	0	0	0	0	0
ARPC1B	0	0	0	0	0	0	0	0	0	0	0	0	0
ARPC2	0	0	0	0	0	0	0	0	0	0	0	0	0
ARPC4	0	0	0	0	0	0	0	0	0	0	0	0	0
ARPC5	0	0	0	0	0	0	0	0	0	0	0	0	0
ARPC5L	0	0	0	0	0	0	0	0	0	0	0	0	0
CAPN2	0	0	0	0	0	0	0	0	0	0	0	0	0
CD99	0	0	0	0	0	0	0	0	0	0	0	0	0

NAME	KEGG_TIGHT_JUNCTION	KEGG_ADHERENS_JUNCTION	KEGG_LEUKOCYTE_TRANSENDOTHELIAL_MIGRATION	KEGG_ARRHYTHMOGENIC_RIGHT_VENTRICULAR_CARDIOMYOPATHY_ARVC	KEGG_ECM_RECEPTOR_INTERACTION	KEGG_FOCAL_ADHESION	KEGG_FC_GAMMA_R_MEDIATED_PHAGOCYTOSIS	KEGG_REGULATION_OF_ACTIN_CYTOSKELETON
TXNDC5	0	0	0	0	0	0	0	0
UBA1	0	0	0	0	0	0	0	0
UBA6	0	0	0	0	0	0	0	0
UBE2M	0	0	0	0	0	0	0	0
UBR4	0	0	0	0	0	0	0	0
UGGT1	0	0	0	0	0	0	0	0
VAMP7	0	0	0	0	0	0	0	0
VBP1	0	0	0	0	0	0	0	0
VIM	0	0	0	0	0	0	0	0
VKORC1	0	0	0	0	0	0	0	0
WDR1	0	0	0	0	0	0	0	0
WFS1	0	0	0	0	0	0	0	0
ABL1	0	0	0	0	0	0	0	0
ACAT1	0	0	0	0	0	0	0	0
AGA	0	0	0	0	0	0	0	0
ARF6	0	0	0	0	0	0	0	0
ARFGAP3	0	0	0	0	0	0	0	0
ARPC1A	0	0	0	0	0	0	0	0
ARPC1B	0	0	0	0	0	0	0	0
ARPC2	0	0	0	0	0	0	0	0
ARPC4	0	0	0	0	0	0	0	0
ARPC5	0	0	0	0	0	0	0	0
ARPC5L	0	0	0	0	0	0	0	0
CAPN2	0	0	0	0	0	0	0	0
CD99	0	0	0	0	0	0	0	0

Table S6 The differential proteins contribute to the enrichment of the significant pathways for the public dataset

Core enrichment proteins in down-regulated pathways of hiPSCs/HFs (hiPSCs vs HFs)

NAME	REACTOME_GLUCOSE_METABOLISM	REACTOME_UNFOLDED_PROTEIN_RESPONSE	REACTOME_DIABETES_PATHWAYS	REACTOME_PROTEIN_FOLDING	REACTOME_PREFOLDIN_MEDIATED_TRANSFER_OF_SUBSTRATE_TO_CCT_TRIC	REACTOME_ASPARAGINE_N_LINKED_GLYCOSYLATION	REACTOME_POST_TRANSLATIONAL_PROTEIN_MODIFICATION	REACTOME_ACTIVATION_OF_THE_MRNA_UPON_BINDING_OF_THE_CAP_BINDING_COMPLEX_AND_EIFS_AND_SUBSEQUENT_BINDING_TO_43S	REACTOME_FORMATION_OF_THE_TERNARY_COMPLEX_AND_SUBSEQUENTLY_THE_43S_COMPLEX	REACTOME_METABOLISM_OF_PROTEINS	REACTOME_SRP_DEPENDENT_COTRANSLATIONAL_PROTEIN_TARGETING_TO_MEMBRANE	REACTOME_INFLUENZA_VIRAL_RNA_TRANSCRIPTION_AND_REPLICATION	REACTOME_NONSENSE_MEDIATED_DECAY_ENHANCED_BY_THE_EXON_JUNCTION_COMPLEX
CFL2	0	0	0	0	0	0	0	0	0	0	0	0	0
CTTN	0	0	0	0	0	0	0	0	0	0	0	0	0
CYFIP1	0	0	0	0	0	0	0	0	0	0	0	0	0
DNM3	0	0	0	0	0	0	0	0	0	0	0	0	0
EGLN1	0	0	0	0	0	0	0	0	0	0	0	0	0
EHD4	0	0	0	0	0	0	0	0	0	0	0	0	0
EPHA2	0	0	0	0	0	0	0	0	0	0	0	0	0
EPN1	0	0	0	0	0	0	0	0	0	0	0	0	0
EXOC7	0	0	0	0	0	0	0	0	0	0	0	0	0
FLNA	0	0	0	0	0	0	0	0	0	0	0	0	0
GRHPR	0	0	0	0	0	0	0	0	0	0	0	0	0
GSK3B	0	0	0	0	0	0	0	0	0	0	0	0	0
HAGH	0	0	0	0	0	0	0	0	0	0	0	0	0
HEXA	0	0	0	0	0	0	0	0	0	0	0	0	0
HEXB	0	0	0	0	0	0	0	0	0	0	0	0	0
HSPA2	0	0	0	0	0	0	0	0	0	0	0	0	0
HSPG2	0	0	0	0	0	0	0	0	0	0	0	0	0
ITCH	0	0	0	0	0	0	0	0	0	0	0	0	0
LAMB2	0	0	0	0	0	0	0	0	0	0	0	0	0
LDHA	0	0	0	0	0	0	0	0	0	0	0	0	0
LMO7	0	0	0	0	0	0	0	0	0	0	0	0	0
ME1	0	0	0	0	0	0	0	0	0	0	0	0	0
NCKAP1	0	0	0	0	0	0	0	0	0	0	0	0	0
PDCD6IP	0	0	0	0	0	0	0	0	0	0	0	0	0
PIP4K2A	0	0	0	0	0	0	0	0	0	0	0	0	0

NAME	REACTOME_PEPTIDE_CHAIN_ELONGATION	REACTOME_3_UTR_MEDIATED_TRANSLATIONAL_REGULATION	REACTOME_TRANSLATION	REACTOME_CELL_CELL_COMMUNICATION	REACTOME_GOLGI_ASSOCIATED_VESICLE_BIOGENESIS	REACTOME_TRANS_GOLGI_NETWORK_VESICLE_BUDDING	REACTOME_MEMBRANE_TRAFFICKING	REACTOME_TRANSMISSION_ACROSS_CHEMICAL_SYNAPSES	REACTOME_NEURONAL_SYSTEM	REACTOME_MHC_CLASS_II_ANTIGEN_PRESENTATION	REACTOME_INNATE_IMMUNE_SYSTEM	REACTOME_IMMUNE_SYSTEM	REACTOME_ADAPTIVE_IMMUNE_SYSTEM
CFL2	0	0	0	0	0	0	0	0	0	0	0	0	0
CTTN	0	0	0	0	0	0	0	0	0	0	0	0	0
CYFIP1	0	0	0	0	0	0	0	0	0	0	0	0	0
DNM3	0	0	0	0	0	0	0	0	0	0	0	0	0
EGLN1	0	0	0	0	0	0	0	0	0	0	0	0	0
EHD4	0	0	0	0	0	0	0	0	0	0	0	0	0
EPHA2	0	0	0	0	0	0	0	0	0	0	0	0	0
EPN1	0	0	0	0	0	0	0	0	0	0	0	0	0
EXOC7	0	0	0	0	0	0	0	0	0	0	0	0	0
FLNA	0	0	0	0	0	0	0	0	0	0	0	0	0
GRHPR	0	0	0	0	0	0	0	0	0	0	0	0	0
GSK3B	0	0	0	0	0	0	0	0	0	0	0	0	0
HAGH	0	0	0	0	0	0	0	0	0	0	0	0	0
HEXA	0	0	0	0	0	0	0	0	0	0	0	0	0
HEXB	0	0	0	0	0	0	0	0	0	0	0	0	0
HSPA2	0	0	0	0	0	0	0	0	0	0	0	0	0
HSPG2	0	0	0	0	0	0	0	0	0	0	0	0	0
ITCH	0	0	0	0	0	0	0	0	0	0	0	0	0
LAMB2	0	0	0	0	0	0	0	0	0	0	0	0	0
LDHA	0	0	0	0	0	0	0	0	0	0	0	0	0
LMO7	0	0	0	0	0	0	0	0	0	0	0	0	0
ME1	0	0	0	0	0	0	0	0	0	0	0	0	0
NCKAP1	0	0	0	0	0	0	0	0	0	0	0	0	0
PDCD6IP	0	0	0	0	0	0	0	0	0	0	0	0	0
PIP4K2A	0	0	0	0	0	0	0	0	0	0	0	0	0

NAME	REACTOME_CLASS_I_MHC_MEDIATED_ANTIGEN_PROCESSING_PRESENTATION	REACTOME_APOPTOSIS	REACTOME_ANTIGEN_PROCESSING_UBIQUITINATION_PROTEASOME_DEGRADATION	REACTOME_ING_BY_WNT	REACTOME_REGULATION_OF_MRNA_STABILITY_BY_PROTEINS_THAT_BIND_AU_RICH_ELEMENTS	REACTOME_DESTABILIZATION_OF_MRNA_BY_AUF1_HNRNP_D0	REACTOME_REGULATION_OF_APOPTOSIS	REACTOME_REGULATION_OF_ORNITHINE_DECARBOXYLASE_ODC	REACTOME_CROSS_PRESENTATION_OF_SOLUBLE_EXOGENOUS_ANTIGENS_ENDOSOMES	REACTOME_VIF_MEDIATED_DEGRADATION_OF_APOBEC3G	REACTOME_REGULATION_OF_MITOTIC_CELL_CYCLE	REACTOME_APC_C_CDC20_MEDIATED_DEGRADATION_OF_MITOTIC_PROTEINS	REACTOME_CDT1_ASSOCIATION_WITH_THE_CDC6_ORC_ORIGIN_COMPLEX
CFL2	0	0	0	0	0	0	0	0	0	0	0	0	0
CTTN	0	0	0	0	0	0	0	0	0	0	0	0	0
CYFIP1	0	0	0	0	0	0	0	0	0	0	0	0	0
DNM3	0	0	0	0	0	0	0	0	0	0	0	0	0
EGLN1	0	0	0	0	0	0	0	0	0	0	0	0	0
EHD4	0	0	0	0	0	0	0	0	0	0	0	0	0
EPHA2	0	0	0	0	0	0	0	0	0	0	0	0	0
EPN1	0	0	0	0	0	0	0	0	0	0	0	0	0
EXOC7	0	0	0	0	0	0	0	0	0	0	0	0	0
FLNA	0	0	0	0	0	0	0	0	0	0	0	0	0
GRHPR	0	0	0	0	0	0	0	0	0	0	0	0	0
GSK3B	0	0	0	0	0	0	0	0	0	0	0	0	0
HAGH	0	0	0	0	0	0	0	0	0	0	0	0	0
HEXA	0	0	0	0	0	0	0	0	0	0	0	0	0
HEXB	0	0	0	0	0	0	0	0	0	0	0	0	0
HSPA2	0	0	0	0	0	0	0	0	0	0	0	0	0
HSPG2	0	0	0	0	0	0	0	0	0	0	0	0	0
ITCH	0	0	0	0	0	0	0	0	0	0	0	0	0
LAMB2	0	0	0	0	0	0	0	0	0	0	0	0	0
LDHA	0	0	0	0	0	0	0	0	0	0	0	0	0
LMO7	0	0	0	0	0	0	0	0	0	0	0	0	0
ME1	0	0	0	0	0	0	0	0	0	0	0	0	0
NCKAP1	0	0	0	0	0	0	0	0	0	0	0	0	0
PDCD6IP	0	0	0	0	0	0	0	0	0	0	0	0	0
PIP4K2A	0	0	0	0	0	0	0	0	0	0	0	0	0

NAME	REACTOME_APC_C_CDH1_MEDIATED_DEGRADATION_OF_CDC20_AND_OTHER_APC_C_CDH1_TARGETED_PROTEINS_IN_LATE_MITOSIS_EARLY_G1	REACTOME_CYCLIN_E_ASSOCIATED_EVENTS_DURING_G1_S_TRANSITION_	REACTOME_SCFSKP2_MEDIATED_DEGRADATION_OF_P27_P21	REACTOME_AUTODEGRADATION_OF_CDH1_BY_CDH1_APC_C	REACTOME_AUTODEGRADATION_OF_THE_E3_UBIQUITIN_LIGASE_COP1	REACTOME_SCF_BETA_TRCP_MEDIATED_DEGRADATION_OF_EMI1	REACTOME_P53_INDEPENDENT_G1_S_DNA_DAMAGE_CHECKPOINT	REACTOME_CDK_MEDIATED_PHOSPHORYLATION_AND_REMOVAL_OF_CDC6	REACTOME_P53_DEPENDENT_G1_DNA_DAMAGE_RESPONSE	REACTOME_ACTIVATION_OF_NF_KAPPAB_IN_B_CELLS	REACTOME_DOWNSTREAM_ING_EVENTS_OF_B_CELL_RECEPTOR_BCR	REACTOME_ING_BY_THE_B_CELL_RECEPTOR_BCR	REACTOME_ANTIGEN_PROCESSING_CROSS_PRESENTATION
CFL2	0	0	0	0	0	0	0	0	0	0	0	0	0
CTTN	0	0	0	0	0	0	0	0	0	0	0	0	0
CYFIP1	0	0	0	0	0	0	0	0	0	0	0	0	0
DNM3	0	0	0	0	0	0	0	0	0	0	0	0	0
EGLN1	0	0	0	0	0	0	0	0	0	0	0	0	0
EHD4	0	0	0	0	0	0	0	0	0	0	0	0	0
EPHA2	0	0	0	0	0	0	0	0	0	0	0	0	0
EPN1	0	0	0	0	0	0	0	0	0	0	0	0	0
EXOC7	0	0	0	0	0	0	0	0	0	0	0	0	0
FLNA	0	0	0	0	0	0	0	0	0	0	0	0	0
GRHPR	0	0	0	0	0	0	0	0	0	0	0	0	0
GSK3B	0	0	0	0	0	0	0	0	0	0	0	0	0
HAGH	0	0	0	0	0	0	0	0	0	0	0	0	0
HEXA	0	0	0	0	0	0	0	0	0	0	0	0	0
HEXB	0	0	0	0	0	0	0	0	0	0	0	0	0
HSPA2	0	0	0	0	0	0	0	0	0	0	0	0	0
HSPG2	0	0	0	0	0	0	0	0	0	0	0	0	0
ITCH	0	0	0	0	0	0	0	0	0	0	0	0	0
LAMB2	0	0	0	0	0	0	0	0	0	0	0	0	0
LDHA	0	0	0	0	0	0	0	0	0	0	0	0	0
LMO7	0	0	0	0	0	0	0	0	0	0	0	0	0
ME1	0	0	0	0	0	0	0	0	0	0	0	0	0
NCKAP1	0	0	0	0	0	0	0	0	0	0	0	0	0
PDCD6IP	0	0	0	0	0	0	0	0	0	0	0	0	0
PIP4K2A	0	0	0	0	0	0	0	0	0	0	0	0	0

NAME	REACTOME_ER_PHAGOSOME_PATHWAY	REACTOME_ING_BY_INSULIN_RECEPTOR	REACTOME_CELL_SURFACE_INTERACTIONS_AT_THE_VASCULAR_WALL	REACTOME_INTEGRIN_CELL_SURFACE_INTERACTIONS	REACTOME_RESPONSE_TO_ELEVATED_PLATELET_CYTOSOLIC_CA2_	REACTOME_HEMOSTASIS	REACTOME_PLATELET_ACTIVATION_ING_AND_AGGREGATION	REACTOME_SEMAPHORIN_INTERACTIONS	REACTOME_L1CAM_INTERACTIONS	REACTOME_DEVELOPMENTAL_BIOLOGY	REACTOME_AXON_GUIDANCE	REACTOME_INTEGRATION_OF_ENERGY_METABOLISM	REACTOME_REGULATION_OF_INSULIN_SECRETION
CFL2	0	0	0	0	0	0	0	0	0	0	0	0	0
CTTN	0	0	0	0	0	0	0	0	0	0	0	0	0
CYFIP1	0	0	0	0	0	0	0	0	0	0	0	0	0
DNM3	0	0	0	0	0	0	0	0	0	0	0	0	0
EGLN1	0	0	0	0	0	0	0	0	0	0	0	0	0
EHD4	0	0	0	0	0	0	0	0	0	0	0	0	0
EPHA2	0	0	0	0	0	0	0	0	0	0	0	0	0
EPN1	0	0	0	0	0	0	0	0	0	0	0	0	0
EXOC7	0	0	0	0	0	0	0	0	0	0	0	0	0
FLNA	0	0	0	0	0	0	0	0	0	0	0	0	0
GRHPR	0	0	0	0	0	0	0	0	0	0	0	0	0
GSK3B	0	0	0	0	0	0	0	0	0	0	0	0	0
HAGH	0	0	0	0	0	0	0	0	0	0	0	0	0
HEXA	0	0	0	0	0	0	0	0	0	0	0	0	0
HEXB	0	0	0	0	0	0	0	0	0	0	0	0	0
HSPA2	0	0	0	0	0	0	0	0	0	0	0	0	0
HSPG2	0	0	0	0	0	0	0	0	0	0	0	0	0
ITCH	0	0	0	0	0	0	0	0	0	0	0	0	0
LAMB2	0	0	0	0	0	0	0	0	0	0	0	0	0
LDHA	0	0	0	0	0	0	0	0	0	0	0	0	0
LMO7	0	0	0	0	0	0	0	0	0	0	0	0	0
ME1	0	0	0	0	0	0	0	0	0	0	0	0	0
NCKAP1	0	0	0	0	0	0	0	0	0	0	0	0	0
PDCD6IP	0	0	0	0	0	0	0	0	0	0	0	0	0
PIP4K2A	0	0	0	0	0	0	0	0	0	0	0	0	0

NAME	REACTOME_ING_BY_ILS	REACTOME_GPCR_DOWNSTREAM_ING	REACTOME_ING_BY_GPCR	REACTOME_LING_BY_NGF	REACTOME_NGF_LING_VIA_TRKA_FROM_THE_PLASMA_MEMBRANE	REACTOME_ING_BY_EGFR_IN_CANCER	REACTOME_ING_BY_FGFR_IN_DISEASE	REACTOME_ING_BY_FGFR	REACTOME_ING_BY_ERBB2	REACTOME_DOWNSTREAM__TRANSDUCTION	REACTOME_ING_BY_PDGF	KEGG_RIBOSOME	KEGG_PROTEASOME
CFL2	0	0	0	0	0	0	0	0	0	0	0	0	0
CTTN	0	0	0	0	0	0	0	0	0	0	0	0	0
CYFIP1	0	0	0	0	0	0	0	0	0	0	0	0	0
DNM3	0	0	0	0	0	0	0	0	0	0	0	0	0
EGLN1	0	0	0	0	0	0	0	0	0	0	0	0	0
EHD4	0	0	0	0	0	0	0	0	0	0	0	0	0
EPHA2	0	0	0	0	0	0	0	0	0	0	0	0	0
EPN1	0	0	0	0	0	0	0	0	0	0	0	0	0
EXOC7	0	0	0	0	0	0	0	0	0	0	0	0	0
FLNA	0	0	0	0	0	0	0	0	0	0	0	0	0
GRHPR	0	0	0	0	0	0	0	0	0	0	0	0	0
GSK3B	0	0	0	0	0	0	0	0	0	0	0	0	0
HAGH	0	0	0	0	0	0	0	0	0	0	0	0	0
HEXA	0	0	0	0	0	0	0	0	0	0	0	0	0
HEXB	0	0	0	0	0	0	0	0	0	0	0	0	0
HSPA2	0	0	0	0	0	0	0	0	0	0	0	0	0
HSPG2	0	0	0	0	0	0	0	0	0	0	0	0	0
ITCH	0	0	0	0	0	0	0	0	0	0	0	0	0
LAMB2	0	0	0	0	0	0	0	0	0	0	0	0	0
LDHA	0	0	0	0	0	0	0	0	0	0	0	0	0
LMO7	0	0	0	0	0	0	0	0	0	0	0	0	0
ME1	0	0	0	0	0	0	0	0	0	0	0	0	0
NCKAP1	0	0	0	0	0	0	0	0	0	0	0	0	0
PDCD6IP	0	0	0	0	0	0	0	0	0	0	0	0	0
PIP4K2A	0	0	0	0	0	0	0	0	0	0	0	0	0

NAME	KEGG_PYRUVATE_METABOLISM	KEGG_LYSOSOME	KEGG_ENDOCYTOSIS	KEGG_EPITHELIAL_CELL_ING_IN_HELICOBACTER_PYLORI_INFECTION	KEGG_AXON_GUIDANCE	KEGG_NEUROTROPHIN_ING_PATHWAY	KEGG_CHEMOKINE_ING_PATHWAY	KEGG_INSULIN_ING_PATHWAY	KEGG_PROSTATE_CANCER	KEGG_PATHWAYS_IN_CANCER	KEGG_ERBB_ING_PATHWAY	KEGG_MAPK_ING_PATHWAY	KEGG_PATHOGENIC_ESCHERICHIA_COLI_INFECTION
CFL2	0	0	0	0	0	0	0	0	0	0	0	0	0
CTTN	0	0	0	0	0	0	0	0	0	0	0	0	0
CYFIP1	0	0	0	0	0	0	0	0	0	0	0	0	0
DNM3	0	0	0	0	0	0	0	0	0	0	0	0	0
EGLN1	0	0	0	0	0	0	0	0	0	0	0	0	0
EHD4	0	0	0	0	0	0	0	0	0	0	0	0	0
EPHA2	0	0	0	0	0	0	0	0	0	0	0	0	0
EPN1	0	0	0	0	0	0	0	0	0	0	0	0	0
EXOC7	0	0	0	0	0	0	0	0	0	0	0	0	0
FLNA	0	0	0	0	0	0	0	0	0	0	0	0	0
GRHPR	0	0	0	0	0	0	0	0	0	0	0	0	0
GSK3B	0	0	0	0	0	0	0	0	0	0	0	0	0
HAGH	0	0	0	0	0	0	0	0	0	0	0	0	0
HEXA	0	0	0	0	0	0	0	0	0	0	0	0	0
HEXB	0	0	0	0	0	0	0	0	0	0	0	0	0
HSPA2	0	0	0	0	0	0	0	0	0	0	0	0	0
HSPG2	0	0	0	0	0	0	0	0	0	0	0	0	0
ITCH	0	0	0	0	0	0	0	0	0	0	0	0	0
LAMB2	0	0	0	0	0	0	0	0	0	0	0	0	0
LDHA	0	0	0	0	0	0	0	0	0	0	0	0	0
LMO7	0	0	0	0	0	0	0	0	0	0	0	0	0
ME1	0	0	0	0	0	0	0	0	0	0	0	0	0
NCKAP1	0	0	0	0	0	0	0	0	0	0	0	0	0
PDCD6IP	0	0	0	0	0	0	0	0	0	0	0	0	0
PIP4K2A	0	0	0	0	0	0	0	0	0	0	0	0	0

NAME	KEGG_TIGHT_JUNCTION	KEGG_ADHERENS_JUNCTION	KEGG_LEUKOCYTE_TRANSENDOTHELIAL_MIGRATION	KEGG_ARRHYTHMOGENIC_RIGHT_VENTRICULAR_CARDIOMYOPATHY_ARVC	KEGG_ECM_RECEPTOR_INTERACTION	KEGG_FOCAL_ADHESION	KEGG_FC_GAMMA_R_MEDIATED_PHAGOCYTOSIS	KEGG_REGULATION_OF_ACTIN_CYTOSKELETON
CFL2	0	0	0	0	0	0	0	0
CTTN	0	0	0	0	0	0	0	0
CYFIP1	0	0	0	0	0	0	0	0
DNM3	0	0	0	0	0	0	0	0
EGLN1	0	0	0	0	0	0	0	0
EHD4	0	0	0	0	0	0	0	0
EPHA2	0	0	0	0	0	0	0	0
EPN1	0	0	0	0	0	0	0	0
EXOC7	0	0	0	0	0	0	0	0
FLNA	0	0	0	0	0	0	0	0
GRHPR	0	0	0	0	0	0	0	0
GSK3B	0	0	0	0	0	0	0	0
HAGH	0	0	0	0	0	0	0	0
HEXA	0	0	0	0	0	0	0	0
HEXB	0	0	0	0	0	0	0	0
HSPA2	0	0	0	0	0	0	0	0
HSPG2	0	0	0	0	0	0	0	0
ITCH	0	0	0	0	0	0	0	0
LAMB2	0	0	0	0	0	0	0	0
LDHA	0	0	0	0	0	0	0	0
LMO7	0	0	0	0	0	0	0	0
ME1	0	0	0	0	0	0	0	0
NCKAP1	0	0	0	0	0	0	0	0
PDCD6IP	0	0	0	0	0	0	0	0
PIP4K2A	0	0	0	0	0	0	0	0

Table S6 The differential proteins contribute to the enrichment of the significant pathways for the public dataset

Core enrichment proteins in down-regulated pathways of hiPSCs/HFs (hiPSCs vs HFs)

NAME	REACTOME_GLUCOSE_METABOLISM	REACTOME_UNFOLDED_PROTEIN_RESPONSE	REACTOME_DIABETES_PATHWAYS	REACTOME_PROTEIN_FOLDING	REACTOME_PREFOLDIN_MEDIATED_TRANSFER_OF_SUBSTRATE_TO_CCT_TRIC	REACTOME_ASPARAGINE_N_LINKED_GLYCOSYLATION	REACTOME_POST_TRANSLATIONAL_PROTEIN_MODIFICATION	REACTOME_ACTIVATION_OF_THE_MRNA_UPON_BINDING_OF_THE_CAP_BINDING_COMPLEX_AND_EIFS_AND_SUBSEQUENT_BINDING_TO_43S	REACTOME_FORMATION_OF_THE_TERNARY_COMPLEX_AND_SUBSEQUENTLY_THE_43S_COMPLEX	REACTOME_METABOLISM_OF_PROTEINS	REACTOME_SRP_DEPENDENT_COTRANSLATIONAL_PROTEIN_TARGETING_TO_MEMBRANE	REACTOME_INFLUENZA_VIRAL_RNA_TRANSCRIPTION_AND_REPLICATION	REACTOME_NONSENSE_MEDIATED_DECAY_ENHANCED_BY_THE_EXON_JUNCTION_COMPLEX
PIP4K2B	0	0	0	0	0	0	0	0	0	0	0	0	0
PLXNB2	0	0	0	0	0	0	0	0	0	0	0	0	0
PPP1CB	0	0	0	0	0	0	0	0	0	0	0	0	0
RAB11FIP5	0	0	0	0	0	0	0	0	0	0	0	0	0
RAB3B	0	0	0	0	0	0	0	0	0	0	0	0	0
RAB4A	0	0	0	0	0	0	0	0	0	0	0	0	0
RAB5B	0	0	0	0	0	0	0	0	0	0	0	0	0
RPL22L1	0	0	0	0	0	0	0	0	0	0	0	0	0
RPS27L	0	0	0	0	0	0	0	0	0	0	0	0	0
SCARB2	0	0	0	0	0	0	0	0	0	0	0	0	0
SH3GL1	0	0	0	0	0	0	0	0	0	0	0	0	0
SH3GLB1	0	0	0	0	0	0	0	0	0	0	0	0	0
TFG	0	0	0	0	0	0	0	0	0	0	0	0	0
THY1	0	0	0	0	0	0	0	0	0	0	0	0	0
YWHAG	0	0	0	0	0	0	0	0	0	0	0	0	0
YWHAQ	0	0	0	0	0	0	0	0	0	0	0	0	0
ZYX	0	0	0	0	0	0	0	0	0	0	0	0	0

NAME	REACTOME_PEPTIDE_CHAIN_ELONGATION	REACTOME_3_UTR_MEDIATED_TRANSLATIONAL_REGULATION	REACTOME_TRANSLATION	REACTOME_CELL_CELL_COMMUNICATION	REACTOME_GOLGI_ASSOCIATED_VESICLE_BIOGENESIS	REACTOME_TRANS_GOLGI_NETWORK_VESICLE_BUDDING	REACTOME_MEMBRANE_TRAFFICKING	REACTOME_TRANSMISSION_ACROSS_CHEMICAL_SYNAPSES	REACTOME_NEURONAL_SYSTEM	REACTOME_MHC_CLASS_II_ANTIGEN_PRESENTATION	REACTOME_INNATE_IMMUNE_SYSTEM	REACTOME_IMMUNE_SYSTEM	REACTOME_ADAPTIVE_IMMUNE_SYSTEM
PIP4K2B	0	0	0	0	0	0	0	0	0	0	0	0	0
PLXNB2	0	0	0	0	0	0	0	0	0	0	0	0	0
PPP1CB	0	0	0	0	0	0	0	0	0	0	0	0	0
RAB11FIP5	0	0	0	0	0	0	0	0	0	0	0	0	0
RAB3B	0	0	0	0	0	0	0	0	0	0	0	0	0
RAB4A	0	0	0	0	0	0	0	0	0	0	0	0	0
RAB5B	0	0	0	0	0	0	0	0	0	0	0	0	0
RPL22L1	0	0	0	0	0	0	0	0	0	0	0	0	0
RPS27L	0	0	0	0	0	0	0	0	0	0	0	0	0
SCARB2	0	0	0	0	0	0	0	0	0	0	0	0	0
SH3GL1	0	0	0	0	0	0	0	0	0	0	0	0	0
SH3GLB1	0	0	0	0	0	0	0	0	0	0	0	0	0
TFG	0	0	0	0	0	0	0	0	0	0	0	0	0
THY1	0	0	0	0	0	0	0	0	0	0	0	0	0
YWHAG	0	0	0	0	0	0	0	0	0	0	0	0	0
YWHAQ	0	0	0	0	0	0	0	0	0	0	0	0	0
ZYX	0	0	0	0	0	0	0	0	0	0	0	0	0

NAME	REACTOME_CLASS_I_MHC_MEDIATED_ANTIGEN_PROCESSING_PRESENTATION	REACTOME_APOPTOSIS	REACTOME_ANTIGEN_PROCESSING_UBIQUITINATION_PROTEASOME_DEGRADATION	REACTOME_ING_BY_WNT	REACTOME_REGULATION_OF_MRNA_STABILITY_BY_PROTEINS_THAT_BIND_AU_RICH_ELEMENTS	REACTOME_DESTABILIZATION_OF_MRNA_BY_AUF1_HNRNP_D0	REACTOME_REGULATION_OF_APOPTOSIS	REACTOME_REGULATION_OF_ORNITHINE_DECARBOXYLASE_ODC	REACTOME_CROSS_PRESENTATION_OF_SOLUBLE_EXOGENOUS_ANTIGENS_ENDOSOMES	REACTOME_VIF_MEDIATED_DEGRADATION_OF_APOBEC3G	REACTOME_REGULATION_OF_MITOTIC_CELL_CYCLE	REACTOME_APC_C_CDC20_MEDIATED_DEGRADATION_OF_MITOTIC_PROTEINS	REACTOME_CDT1_ASSOCIATION_WITH_THE_CDC6_ORC_ORIGIN_COMPLEX
PIP4K2B	0	0	0	0	0	0	0	0	0	0	0	0	0
PLXNB2	0	0	0	0	0	0	0	0	0	0	0	0	0
PPP1CB	0	0	0	0	0	0	0	0	0	0	0	0	0
RAB11FIP5	0	0	0	0	0	0	0	0	0	0	0	0	0
RAB3B	0	0	0	0	0	0	0	0	0	0	0	0	0
RAB4A	0	0	0	0	0	0	0	0	0	0	0	0	0
RAB5B	0	0	0	0	0	0	0	0	0	0	0	0	0
RPL22L1	0	0	0	0	0	0	0	0	0	0	0	0	0
RPS27L	0	0	0	0	0	0	0	0	0	0	0	0	0
SCARB2	0	0	0	0	0	0	0	0	0	0	0	0	0
SH3GL1	0	0	0	0	0	0	0	0	0	0	0	0	0
SH3GLB1	0	0	0	0	0	0	0	0	0	0	0	0	0
TFG	0	0	0	0	0	0	0	0	0	0	0	0	0
THY1	0	0	0	0	0	0	0	0	0	0	0	0	0
YWHAG	0	0	0	0	0	0	0	0	0	0	0	0	0
YWHAQ	0	0	0	0	0	0	0	0	0	0	0	0	0
ZYX	0	0	0	0	0	0	0	0	0	0	0	0	0

NAME	REACTOME_APC_C_CDH1_MEDIATED_DEGRADATION_OF_CDC20_AND_OTHER_APC_C_CDH1_TARGETED_PROTEINS_IN_LATE_MITOSIS_EARLY_G1	REACTOME_CYCLIN_E_ASSOCIATED_EVENTS_DURING_G1_S_TRANSITION_	REACTOME_SCFSKP2_MEDIATED_DEGRADATION_OF_P27_P21	REACTOME_AUTODEGRADATION_OF_CDH1_BY_CDH1_APC_C	REACTOME_AUTODEGRADATION_OF_THE_E3_UBIQUITIN_LIGASE_COP1	REACTOME_SCF_BETA_TRCP_MEDIATED_DEGRADATION_OF_EMI1	REACTOME_P53_INDEPENDENT_G1_S_DNA_DAMAGE_CHECKPOINT	REACTOME_CDK_MEDIATED_PHOSPHORYLATION_AND_REMOVAL_OF_CDC6	REACTOME_P53_DEPENDENT_G1_DNA_DAMAGE_RESPONSE	REACTOME_ACTIVATION_OF_NF_KAPPAB_IN_B_CELLS	REACTOME_DOWNSTREAM_ING_EVENTS_OF_B_CELL_RECEPTOR_BCR	REACTOME_ING_BY_THE_B_CELL_RECEPTOR_BCR	REACTOME_ANTIGEN_PROCESSING_CROSS_PRESENTATION
PIP4K2B	0	0	0	0	0	0	0	0	0	0	0	0	0
PLXNB2	0	0	0	0	0	0	0	0	0	0	0	0	0
PPP1CB	0	0	0	0	0	0	0	0	0	0	0	0	0
RAB11FIP5	0	0	0	0	0	0	0	0	0	0	0	0	0
RAB3B	0	0	0	0	0	0	0	0	0	0	0	0	0
RAB4A	0	0	0	0	0	0	0	0	0	0	0	0	0
RAB5B	0	0	0	0	0	0	0	0	0	0	0	0	0
RPL22L1	0	0	0	0	0	0	0	0	0	0	0	0	0
RPS27L	0	0	0	0	0	0	0	0	0	0	0	0	0
SCARB2	0	0	0	0	0	0	0	0	0	0	0	0	0
SH3GL1	0	0	0	0	0	0	0	0	0	0	0	0	0
SH3GLB1	0	0	0	0	0	0	0	0	0	0	0	0	0
TFG	0	0	0	0	0	0	0	0	0	0	0	0	0
THY1	0	0	0	0	0	0	0	0	0	0	0	0	0
YWHAG	0	0	0	0	0	0	0	0	0	0	0	0	0
YWHAQ	0	0	0	0	0	0	0	0	0	0	0	0	0
ZYX	0	0	0	0	0	0	0	0	0	0	0	0	0

NAME	REACTOME_ER_PHAGOSOME_PATHWAY	REACTOME_ING_BY_INSULIN_RECEPTOR	REACTOME_CELL_SURFACE_INTERACTIONS_AT_THE_VASCULAR_WALL	REACTOME_INTEGRIN_CELL_SURFACE_INTERACTIONS	REACTOME_RESPONSE_TO_ELEVATED_PLATELET_CYTOSOLIC_CA2_	REACTOME_HEMOSTASIS	REACTOME_PLATELET_ACTIVATION_ING_AND_AGGREGATION	REACTOME_SEMAPHORIN_INTERACTIONS	REACTOME_L1CAM_INTERACTIONS	REACTOME_DEVELOPMENTAL_BIOLOGY	REACTOME_AXON_GUIDANCE	REACTOME_INTEGRATION_OF_ENERGY_METABOLISM	REACTOME_REGULATION_OF_INSULIN_SECRETION
PIP4K2B	0	0	0	0	0	0	0	0	0	0	0	0	0
PLXNB2	0	0	0	0	0	0	0	0	0	0	0	0	0
PPP1CB	0	0	0	0	0	0	0	0	0	0	0	0	0
RAB11FIP5	0	0	0	0	0	0	0	0	0	0	0	0	0
RAB3B	0	0	0	0	0	0	0	0	0	0	0	0	0
RAB4A	0	0	0	0	0	0	0	0	0	0	0	0	0
RAB5B	0	0	0	0	0	0	0	0	0	0	0	0	0
RPL22L1	0	0	0	0	0	0	0	0	0	0	0	0	0
RPS27L	0	0	0	0	0	0	0	0	0	0	0	0	0
SCARB2	0	0	0	0	0	0	0	0	0	0	0	0	0
SH3GL1	0	0	0	0	0	0	0	0	0	0	0	0	0
SH3GLB1	0	0	0	0	0	0	0	0	0	0	0	0	0
TFG	0	0	0	0	0	0	0	0	0	0	0	0	0
THY1	0	0	0	0	0	0	0	0	0	0	0	0	0
YWHAG	0	0	0	0	0	0	0	0	0	0	0	0	0
YWHAQ	0	0	0	0	0	0	0	0	0	0	0	0	0
ZYX	0	0	0	0	0	0	0	0	0	0	0	0	0

NAME	REACTOME_ING_BY_ILS	REACTOME_GPCR_DOWNSTREAM_ING	REACTOME_ING_BY_GPCR	REACTOME_LING_BY_NGF	REACTOME_NGF_LING_VIA_TRKA_FROM_THE_PLASMA_MEMBRANE	REACTOME_ING_BY_EGFR_IN_CANCER	REACTOME_ING_BY_FGFR_IN_DISEASE	REACTOME_ING_BY_FGFR	REACTOME_ING_BY_ERBB2	REACTOME_DOWNSTREAM__TRANSDUCTION	REACTOME_ING_BY_PDGF	KEGG_RIBOSOME	KEGG_PROTEASOME
PIP4K2B	0	0	0	0	0	0	0	0	0	0	0	0	0
PLXNB2	0	0	0	0	0	0	0	0	0	0	0	0	0
PPP1CB	0	0	0	0	0	0	0	0	0	0	0	0	0
RAB11FIP5	0	0	0	0	0	0	0	0	0	0	0	0	0
RAB3B	0	0	0	0	0	0	0	0	0	0	0	0	0
RAB4A	0	0	0	0	0	0	0	0	0	0	0	0	0
RAB5B	0	0	0	0	0	0	0	0	0	0	0	0	0
RPL22L1	0	0	0	0	0	0	0	0	0	0	0	0	0
RPS27L	0	0	0	0	0	0	0	0	0	0	0	0	0
SCARB2	0	0	0	0	0	0	0	0	0	0	0	0	0
SH3GL1	0	0	0	0	0	0	0	0	0	0	0	0	0
SH3GLB1	0	0	0	0	0	0	0	0	0	0	0	0	0
TFG	0	0	0	0	0	0	0	0	0	0	0	0	0
THY1	0	0	0	0	0	0	0	0	0	0	0	0	0
YWHAG	0	0	0	0	0	0	0	0	0	0	0	0	0
YWHAQ	0	0	0	0	0	0	0	0	0	0	0	0	0
ZYX	0	0	0	0	0	0	0	0	0	0	0	0	0

NAME	KEGG_PYRUVATE_METABOLISM	KEGG_LYSOSOME	KEGG_ENDOCYTOSIS	KEGG_EPITHELIAL_CELL_ING_IN_HELICOBACTER_PYLORI_INFECTION	KEGG_AXON_GUIDANCE	KEGG_NEUROTROPHIN_ING_PATHWAY	KEGG_CHEMOKINE_ING_PATHWAY	KEGG_INSULIN_ING_PATHWAY	KEGG_PROSTATE_CANCER	KEGG_PATHWAYS_IN_CANCER	KEGG_ERBB_ING_PATHWAY	KEGG_MAPK_ING_PATHWAY	KEGG_PATHOGENIC_ESCHERICHIA_COLI_INFECTION
PIP4K2B	0	0	0	0	0	0	0	0	0	0	0	0	0
PLXNB2	0	0	0	0	0	0	0	0	0	0	0	0	0
PPP1CB	0	0	0	0	0	0	0	0	0	0	0	0	0
RAB11FIP5	0	0	0	0	0	0	0	0	0	0	0	0	0
RAB3B	0	0	0	0	0	0	0	0	0	0	0	0	0
RAB4A	0	0	0	0	0	0	0	0	0	0	0	0	0
RAB5B	0	0	0	0	0	0	0	0	0	0	0	0	0
RPL22L1	0	0	0	0	0	0	0	0	0	0	0	0	0
RPS27L	0	0	0	0	0	0	0	0	0	0	0	0	0
SCARB2	0	0	0	0	0	0	0	0	0	0	0	0	0
SH3GL1	0	0	0	0	0	0	0	0	0	0	0	0	0
SH3GLB1	0	0	0	0	0	0	0	0	0	0	0	0	0
TFG	0	0	0	0	0	0	0	0	0	0	0	0	0
THY1	0	0	0	0	0	0	0	0	0	0	0	0	0
YWHAG	0	0	0	0	0	0	0	0	0	0	0	0	0
YWHAQ	0	0	0	0	0	0	0	0	0	0	0	0	0
ZYX	0	0	0	0	0	0	0	0	0	0	0	0	0

NAME	KEGG_TIGHT_JUNCTION	KEGG_ADHERENS_JUNCTION	KEGG_LEUKOCYTE_TRANSENDOTHELIAL_MIGRATION	KEGG_ARRHYTHMOGENIC_RIGHT_VENTRICULAR_CARDIOMYOPATHY_ARVC	KEGG_ECM_RECEPTOR_INTERACTION	KEGG_FOCAL_ADHESION	KEGG_FC_GAMMA_R_MEDIATED_PHAGOCYTOSIS	KEGG_REGULATION_OF_ACTIN_CYTOSKELETON
PIP4K2B	0	0	0	0	0	0	0	0
PLXNB2	0	0	0	0	0	0	0	0
PPP1CB	0	0	0	0	0	0	0	0
RAB11FIP5	0	0	0	0	0	0	0	0
RAB3B	0	0	0	0	0	0	0	0
RAB4A	0	0	0	0	0	0	0	0
RAB5B	0	0	0	0	0	0	0	0
RPL22L1	0	0	0	0	0	0	0	0
RPS27L	0	0	0	0	0	0	0	0
SCARB2	0	0	0	0	0	0	0	0
SH3GL1	0	0	0	0	0	0	0	0
SH3GLB1	0	0	0	0	0	0	0	0
TFG	0	0	0	0	0	0	0	0
THY1	0	0	0	0	0	0	0	0
YWHAG	0	0	0	0	0	0	0	0
YWHAQ	0	0	0	0	0	0	0	0
ZYX	0	0	0	0	0	0	0	0

Table S6 The differential proteins contribute to the enrichment of the significant pathways for the public dataset

Core enrichment proteins in up-regulated pathways of hiPSCs/HFs (hiPSCs vs HFs)

NAME	REACTOME_MITOCHONDRIAL_PROTEIN_IMPORT_	REACTOME_DEADENYLATION_DEPENDENT_MRNA_DECAY_	REACTOME_RNA_POL_I_RNA_POL_III_AND_MITOCHONDRIAL_TRANSCRIPTION_	REACTOME_PROCESSING_OF_CAPPED_INTRON_CONTAINING_PRE_MRNA_	REACTOME_MRNA_PROCESSING_	REACTOME_MRNA_SPLICING_	REACTOME_MRNA_SPLICING_MINOR_PATHWAY_	REACTOME_TRANSCRIPTION_	REACTOME_RNA_POL_II_TRANSCRIPTION_	REACTOME_CLEAVAGE_OF_GROWING_TRANSCRIPT_IN_THE_TERMINATION_REGION__	REACTOME_MRNA_3_END_PROCESSING_	REACTOME_HIV_LIFE_CYCLE_	REACTOME_LATE_PHASE_OF_HIV_LIFE_CYCLE_
CCNB1	0	0	0	0	0	0	0	0	0	0	0	0	0
CCNB2	0	0	0	0	0	0	0	0	0	0	0	0	0
DDX23	0	0	0	1	1	1	1	0	0	0	0	0	0
EFTUD2	0	0	0	1	1	1	1	0	0	0	0	0	0
EIF4A3	0	1	0	0	0	0	0	0	0	0	0	0	0
EXOSC4	0	1	0	0	0	0	0	0	0	0	0	0	0
EXOSC5	0	1	0	0	0	0	0	0	0	0	0	0	0
EXOSC6	0	1	0	0	0	0	0	0	0	0	0	0	0
EXOSC7	0	1	0	0	0	0	0	0	0	0	0	0	0
EXOSC9	0	1	0	0	0	0	0	0	0	0	0	0	0
FEN1	0	0	0	0	0	0	0	0	0	0	0	1	0
HDAC2	0	0	0	0	0	0	0	0	0	0	0	0	0
HNRNPA1	0	0	0	1	1	1	0	0	0	0	0	0	0
HNRNPA3	0	0	0	1	1	1	0	0	0	0	0	0	0
HNRNPC	0	0	0	1	1	1	0	0	0	0	0	0	0
HNRNPK	0	0	0	1	1	1	0	0	0	0	0	0	0
HNRNPM	0	0	0	1	1	1	0	0	0	0	0	0	0
HNRNPU	0	0	0	1	1	1	0	0	0	0	0	0	0
HSPD1	1	0	0	0	0	0	0	0	0	0	0	0	0
LIG1	0	0	0	0	0	0	0	0	0	0	0	1	0
LSM2	0	1	0	1	1	1	1	0	0	0	0	0	0
LSM3	0	1	0	0	0	0	0	0	0	0	0	0	0
LSM4	0	1	0	0	0	0	0	0	0	0	0	0	0
LSM6	0	1	0	0	0	0	0	0	0	0	0	0	0
MAGOH	0	0	0	1	1	1	0	1	1	1	1	0	0
MCM2	0	0	0	0	0	0	0	0	0	0	0	0	0

NAME	REACTOME_METABOLISM_OF_NON_CODING_RNA_	REACTOME_TRANSPORT_OF_MATURE_TRANSCRIPT_TO_CYTOPLASM_	REACTOME_TRANSPORT_OF_MATURE_MRNA_DERIVED_FROM_AN_INTRONLESS_TRANSCRIPT_	REACTOME_INTERACTIONS_OF_VPR_WITH_HOST_CELLULAR_PROTEINS_	REACTOME_SLC_MEDIATED_TRANSMEMBRANE_TRANSPORT_	REACTOME_GLUCOSE_TRANSPORT_	REACTOME_NEP_NS2_INTERACTS_WITH_THE_CELLULAR_EXPORT_MACHINERY_	REACTOME_REGULATION_OF_GLUCOKINASE_BY_GLUCOKINASE_REGULATORY_PROTEIN_	REACTOME_TRANSPORT_OF_RIBONUCLEOPROTEINS_INTO_THE_HOST_NUCLEUS_	REACTOME_RNA_POL_II_PRE_TRANSCRIPTION_EVENTS_	REACTOME_DNA_REPAIR_	REACTOME_NUCLEOTIDE_EXCISION_REPAIR_	REACTOME_MITOTIC_PROMETAPHASE_
CCNB1	0	0	0	0	0	0	0	0	0	0	0	0	0
CCNB2	0	0	0	0	0	0	0	0	0	0	0	0	0
DDX23	0	0	0	0	0	0	0	0	0	0	0	0	0
EFTUD2	0	0	0	0	0	0	0	0	0	0	0	0	0
EIF4A3	0	0	0	0	0	0	0	0	0	0	0	0	0
EXOSC4	0	0	0	0	0	0	0	0	0	0	0	0	0
EXOSC5	0	0	0	0	0	0	0	0	0	0	0	0	0
EXOSC6	0	0	0	0	0	0	0	0	0	0	0	0	0
EXOSC7	0	0	0	0	0	0	0	0	0	0	0	0	0
EXOSC9	0	0	0	0	0	0	0	0	0	0	0	0	0
FEN1	0	0	0	0	0	0	0	0	0	0	1	0	0
HDAC2	0	0	0	0	0	0	0	0	0	0	0	0	0
HNRNPA1	0	0	0	0	0	0	0	0	0	0	0	0	0
HNRNPA3	0	0	0	0	0	0	0	0	0	0	0	0	0
HNRNPC	0	0	0	0	0	0	0	0	0	0	0	0	0
HNRNPK	0	0	0	0	0	0	0	0	0	0	0	0	0
HNRNPM	0	0	0	0	0	0	0	0	0	0	0	0	0
HNRNPU	0	0	0	0	0	0	0	0	0	0	0	0	0
HSPD1	0	0	0	0	0	0	0	0	0	0	0	0	0
LIG1	0	0	0	0	0	0	0	0	0	0	1	1	0
LSM2	0	0	0	0	0	0	0	0	0	0	0	0	0
LSM3	0	0	0	0	0	0	0	0	0	0	0	0	0
LSM4	0	0	0	0	0	0	0	0	0	0	0	0	0
LSM6	0	0	0	0	0	0	0	0	0	0	0	0	0
MAGOH	0	1	0	0	0	0	0	0	0	0	0	0	0
MCM2	0	0	0	0	0	0	0	0	0	0	0	0	0

NAME	REACTOME_MEIOSIS_	REACTOME_CHROMOSOME_MAINTENANCE_	REACTOME_CELL_CYCLE_MITOTIC_	REACTOME_CELL_CYCLE_	REACTOME_DNA_REPLICATION_	REACTOME_MITOTIC_M_M_G1_PHASES_	REACTOME_SYNTHESIS_OF_DNA_	REACTOME_S_PHASE_	REACTOME_CELL_CYCLE_CHECKPOINTS_	REACTOME_G1_S_TRANSITION_	REACTOME_MITOTIC_G1_G1_S_PHASES_	REACTOME_FACTORS_INVOLVED_IN_MEGAKARYOCYTE_DEVELOPMENT_AND_PLATELET_PRODUCTION	KEGG_SPLICEOSOME_
CCNB1	0	0	1	1	0	0	0	0	1	1	1	0	0
CCNB2	0	0	1	1	0	0	0	0	1	0	0	0	0
DDX23	0	0	0	0	0	0	0	0	0	0	0	0	1
EFTUD2	0	0	0	0	0	0	0	0	0	0	0	0	1
EIF4A3	0	0	0	0	0	0	0	0	0	0	0	0	1
EXOSC4	0	0	0	0	0	0	0	0	0	0	0	0	0
EXOSC5	0	0	0	0	0	0	0	0	0	0	0	0	0
EXOSC6	0	0	0	0	0	0	0	0	0	0	0	0	0
EXOSC7	0	0	0	0	0	0	0	0	0	0	0	0	0
EXOSC9	0	0	0	0	0	0	0	0	0	0	0	0	0
FEN1	0	1	1	1	1	0	1	1	0	0	0	0	0
HDAC2	0	0	0	0	0	0	0	0	0	0	0	1	0
HNRNPA1	0	0	0	0	0	0	0	0	0	0	0	0	1
HNRNPA3	0	0	0	0	0	0	0	0	0	0	0	0	1
HNRNPC	0	0	0	0	0	0	0	0	0	0	0	0	1
HNRNPK	0	0	0	0	0	0	0	0	0	0	0	0	1
HNRNPM	0	0	0	0	0	0	0	0	0	0	0	0	1
HNRNPU	0	0	0	0	0	0	0	0	0	0	0	0	1
HSPD1	0	0	0	0	0	0	0	0	0	0	0	0	0
LIG1	0	1	1	1	1	0	1	1	0	0	0	0	0
LSM2	0	0	0	0	0	0	0	0	0	0	0	0	1
LSM3	0	0	0	0	0	0	0	0	0	0	0	0	1
LSM4	0	0	0	0	0	0	0	0	0	0	0	0	1
LSM6	0	0	0	0	0	0	0	0	0	0	0	0	1
MAGOH	0	0	0	0	0	0	0	0	0	0	0	0	1
MCM2	0	0	1	1	1	1	1	1	1	1	1	0	0

NAME	KEGG_RNA_DEGRADATION_	KEGG_DNA_REPLICATION_	KEGG_CELL_CYCLE_	KEGG_OOCYTE_MEIOSIS_	KEGG_PYRIMIDINE_METABOLISM_	KEGG_VALINE_LEUCINE_AND_ISOLEUCINE_DEGRADATION_
CCNB1	0	0	1	1	0	0
CCNB2	0	0	1	1	0	0
DDX23	0	0	0	0	0	0
EFTUD2	0	0	0	0	0	0
EIF4A3	0	0	0	0	0	0
EXOSC4	1	0	0	0	0	0
EXOSC5	1	0	0	0	0	0
EXOSC6	1	0	0	0	0	0
EXOSC7	1	0	0	0	0	0
EXOSC9	1	0	0	0	0	0
FEN1	0	1	0	0	0	0
HDAC2	0	0	1	0	0	0
HNRNPA1	0	0	0	0	0	0
HNRNPA3	0	0	0	0	0	0
HNRNPC	0	0	0	0	0	0
HNRNPK	0	0	0	0	0	0
HNRNPM	0	0	0	0	0	0
HNRNPU	0	0	0	0	0	0
HSPD1	1	0	0	0	0	0
LIG1	0	1	0	0	0	0
LSM2	1	0	0	0	0	0
LSM3	1	0	0	0	0	0
LSM4	1	0	0	0	0	0
LSM6	1	0	0	0	0	0
MAGOH	0	0	0	0	0	0
MCM2	0	1	1	0	0	0

Table S6 The differential proteins contribute to the enrichment of the significant pathways for the public dataset

Core enrichment proteins in up-regulated pathways of hiPSCs/HFs (hiPSCs vs HFs)

NAME	REACTOME_MITOCHONDRIAL_PROTEIN_IMPORT_	REACTOME_DEADENYLATION_DEPENDENT_MRNA_DECAY_	REACTOME_RNA_POL_I_RNA_POL_III_AND_MITOCHONDRIAL_TRANSCRIPTION_	REACTOME_PROCESSING_OF_CAPPED_INTRON_CONTAINING_PRE_MRNA_	REACTOME_MRNA_PROCESSING_	REACTOME_MRNA_SPLICING_	REACTOME_MRNA_SPLICING_MINOR_PATHWAY_	REACTOME_TRANSCRIPTION_	REACTOME_RNA_POL_II_TRANSCRIPTION_	REACTOME_CLEAVAGE_OF_GROWING_TRANSCRIPT_IN_THE_TERMINATION_REGION__	REACTOME_MRNA_3_END_PROCESSING_	REACTOME_HIV_LIFE_CYCLE_	REACTOME_LATE_PHASE_OF_HIV_LIFE_CYCLE_
MCM3	0	0	0	0	0	0	0	0	0	0	0	0	0
MCM4	0	0	0	0	0	0	0	0	0	0	0	0	0
MCM6	0	0	0	0	0	0	0	0	0	0	0	0	0
MCM7	0	0	0	0	0	0	0	0	0	0	0	0	0
NCBP1	0	0	0	1	1	1	1	1	1	1	1	1	1
NHP2L1	0	0	0	1	1	1	1	0	0	0	0	0	0
ORC2	0	0	0	0	0	0	0	0	0	0	0	0	0
ORC3	0	0	0	0	0	0	0	0	0	0	0	0	0
PARN	0	1	0	0	0	0	0	0	0	0	0	0	0
PHF5A	0	0	0	1	1	1	0	0	0	0	0	0	0
PLK1	0	0	0	0	0	0	0	0	0	0	0	0	0
POLA2	0	0	0	0	0	0	0	0	0	0	0	0	0
POLD1	0	0	0	0	0	0	0	0	0	0	0	0	0
POLD3	0	0	0	0	0	0	0	0	0	0	0	0	0
POLR1B	0	0	1	0	0	0	0	1	0	0	0	0	0
POLR1C	0	0	0	0	0	0	0	1	0	0	0	0	0
POLR1D	0	0	0	0	0	0	0	1	0	0	0	0	0
POLR2A	0	0	0	1	1	1	1	1	1	0	0	1	1
POLR2B	0	0	0	1	1	1	1	1	1	0	0	1	1
POLR2C	0	0	0	1	1	1	1	1	1	0	0	1	1
POLR2E	0	0	1	1	1	1	1	1	1	0	0	1	1
POLR2H	0	0	1	1	1	1	1	1	1	0	0	1	1
POLR2L	0	0	0	1	1	1	1	1	1	0	0	1	1
POLR3A	0	0	1	0	0	0	0	1	0	0	0	0	0
POLR3D	0	0	1	0	0	0	0	1	0	0	0	0	0
PRIM2	0	0	0	0	0	0	0	0	0	0	0	0	0

NAME	REACTOME_METABOLISM_OF_NON_CODING_RNA_	REACTOME_TRANSPORT_OF_MATURE_TRANSCRIPT_TO_CYTOPLASM_	REACTOME_TRANSPORT_OF_MATURE_MRNA_DERIVED_FROM_AN_INTRONLESS_TRANSCRIPT_	REACTOME_INTERACTIONS_OF_VPR_WITH_HOST_CELLULAR_PROTEINS_	REACTOME_SLC_MEDIATED_TRANSMEMBRANE_TRANSPORT_	REACTOME_GLUCOSE_TRANSPORT_	REACTOME_NEP_NS2_INTERACTS_WITH_THE_CELLULAR_EXPORT_MACHINERY_	REACTOME_REGULATION_OF_GLUCOKINASE_BY_GLUCOKINASE_REGULATORY_PROTEIN_	REACTOME_TRANSPORT_OF_RIBONUCLEOPROTEINS_INTO_THE_HOST_NUCLEUS_	REACTOME_RNA_POL_II_PRE_TRANSCRIPTION_EVENTS_	REACTOME_DNA_REPAIR_	REACTOME_NUCLEOTIDE_EXCISION_REPAIR_	REACTOME_MITOTIC_PROMETAPHASE_
MCM3	0	0	0	0	0	0	0	0	0	0	0	0	0
MCM4	0	0	0	0	0	0	0	0	0	0	0	0	0
MCM6	0	0	0	0	0	0	0	0	0	0	0	0	0
MCM7	0	0	0	0	0	0	0	0	0	0	0	0	0
NCBP1	1	1	1	0	0	0	0	0	0	1	0	0	0
NHP2L1	0	0	0	0	0	0	0	0	0	0	0	0	0
ORC2	0	0	0	0	0	0	0	0	0	0	0	0	0
ORC3	0	0	0	0	0	0	0	0	0	0	0	0	0
PARN	0	0	0	0	0	0	0	0	0	0	0	0	0
PHF5A	0	0	0	0	0	0	0	0	0	0	0	0	0
PLK1	0	0	0	0	0	0	0	0	0	0	0	0	1
POLA2	0	0	0	0	0	0	0	0	0	0	0	0	0
POLD1	0	0	0	0	0	0	0	0	0	0	1	1	0
POLD3	0	0	0	0	0	0	0	0	0	0	1	1	0
POLR1B	0	0	0	0	0	0	0	0	0	0	0	0	0
POLR1C	0	0	0	0	0	0	0	0	0	0	0	0	0
POLR1D	0	0	0	0	0	0	0	0	0	0	0	0	0
POLR2A	0	0	0	0	0	0	0	0	0	1	1	1	0
POLR2B	0	0	0	0	0	0	0	0	0	1	1	1	0
POLR2C	0	0	0	0	0	0	0	0	0	1	1	1	0
POLR2E	0	0	0	0	0	0	0	0	0	1	1	1	0
POLR2H	0	0	0	0	0	0	0	0	0	1	1	1	0
POLR2L	0	0	0	0	0	0	0	0	0	1	1	1	0
POLR3A	0	0	0	0	0	0	0	0	0	0	0	0	0
POLR3D	0	0	0	0	0	0	0	0	0	0	0	0	0
PRIM2	0	0	0	0	0	0	0	0	0	0	0	0	0

NAME	REACTOME_MEIOSIS_	REACTOME_CHROMOSOME_MAINTENANCE_	REACTOME_CELL_CYCLE_MITOTIC_	REACTOME_CELL_CYCLE_	REACTOME_DNA_REPLICATION_	REACTOME_MITOTIC_M_M_G1_PHASES_	REACTOME_SYNTHESIS_OF_DNA_	REACTOME_S_PHASE_	REACTOME_CELL_CYCLE_CHECKPOINTS_	REACTOME_G1_S_TRANSITION_	REACTOME_MITOTIC_G1_G1_S_PHASES_	REACTOME_FACTORS_INVOLVED_IN_MEGAKARYOCYTE_DEVELOPMENT_AND_PLATELET_PRODUCTION	KEGG_SPLICEOSOME_
MCM3	0	0	1	1	1	1	1	1	1	1	1	0	0
MCM4	0	0	1	1	1	1	1	1	1	1	1	0	0
MCM6	0	0	1	1	1	1	1	1	1	1	1	0	0
MCM7	0	0	1	1	1	1	1	1	1	1	1	0	0
NCBP1	0	0	0	0	0	0	0	0	0	0	0	0	1
NHP2L1	0	0	0	0	0	0	0	0	0	0	0	0	1
ORC2	0	0	1	1	1	1	1	1	1	1	1	0	0
ORC3	0	0	1	1	1	1	1	1	1	1	1	0	0
PARN	0	0	0	0	0	0	0	0	0	0	0	0	0
PHF5A	0	0	0	0	0	0	0	0	0	0	0	0	1
PLK1	0	0	1	1	1	1	0	0	0	0	0	0	0
POLA2	0	0	0	0	1	1	0	0	0	0	0	0	0
POLD1	0	1	1	1	1	0	1	1	0	0	0	0	0
POLD3	0	1	1	1	1	0	1	1	0	0	0	0	0
POLR1B	0	0	0	0	0	0	0	0	0	0	0	0	0
POLR1C	0	0	0	0	0	0	0	0	0	0	0	0	0
POLR1D	0	0	0	0	0	0	0	0	0	0	0	0	0
POLR2A	0	0	0	0	0	0	0	0	0	0	0	0	0
POLR2B	0	0	0	0	0	0	0	0	0	0	0	0	0
POLR2C	0	0	0	0	0	0	0	0	0	0	0	0	0
POLR2E	0	0	0	0	0	0	0	0	0	0	0	0	0
POLR2H	0	0	0	0	0	0	0	0	0	0	0	0	0
POLR2L	0	0	0	0	0	0	0	0	0	0	0	0	0
POLR3A	0	0	0	0	0	0	0	0	0	0	0	0	0
POLR3D	0	0	0	0	0	0	0	0	0	0	0	0	0
PRIM2	0	1	1	1	1	1	1	1	0	1	1	0	0

NAME	KEGG_RNA_DEGRADATION_	KEGG_DNA_REPLICATION_	KEGG_CELL_CYCLE_	KEGG_OOCYTE_MEIOSIS_	KEGG_PYRIMIDINE_METABOLISM_	KEGG_VALINE_LEUCINE_AND_ISOLEUCINE_DEGRADATION_
MCM3	0	1	1	0	0	0
MCM4	0	1	1	0	0	0
MCM6	0	1	1	0	0	0
MCM7	0	1	1	0	0	0
NCBP1	0	0	0	0	0	0
NHP2L1	0	0	0	0	0	0
ORC2	0	0	1	0	0	0
ORC3	0	0	1	0	0	0
PARN	1	0	0	0	0	0
PHF5A	0	0	0	0	0	0
PLK1	0	0	1	1	0	0
POLA2	0	0	0	0	1	0
POLD1	0	1	0	0	1	0
POLD3	0	1	0	0	1	0
POLR1B	0	0	0	0	1	0
POLR1C	0	0	0	0	1	0
POLR1D	0	0	0	0	1	0
POLR2A	0	0	0	0	1	0
POLR2B	0	0	0	0	1	0
POLR2C	0	0	0	0	1	0
POLR2E	0	0	0	0	1	0
POLR2H	0	0	0	0	1	0
POLR2L	0	0	0	0	1	0
POLR3A	0	0	0	0	1	0
POLR3D	0	0	0	0	1	0
PRIM2	0	1	0	0	1	0

Table S6 The differential proteins contribute to the enrichment of the significant pathways for the public dataset

Core enrichment proteins in up-regulated pathways of hiPSCs/HFs (hiPSCs vs HFs)

NAME	REACTOME_MITOCHONDRIAL_PROTEIN_IMPORT_	REACTOME_DEADENYLATION_DEPENDENT_MRNA_DECAY_	REACTOME_RNA_POL_I_RNA_POL_III_AND_MITOCHONDRIAL_TRANSCRIPTION_	REACTOME_PROCESSING_OF_CAPPED_INTRON_CONTAINING_PRE_MRNA_	REACTOME_MRNA_PROCESSING_	REACTOME_MRNA_SPLICING_	REACTOME_MRNA_SPLICING_MINOR_PATHWAY_	REACTOME_TRANSCRIPTION_	REACTOME_RNA_POL_II_TRANSCRIPTION_	REACTOME_CLEAVAGE_OF_GROWING_TRANSCRIPT_IN_THE_TERMINATION_REGION__	REACTOME_MRNA_3_END_PROCESSING_	REACTOME_HIV_LIFE_CYCLE_	REACTOME_LATE_PHASE_OF_HIV_LIFE_CYCLE_
PRPF4	0	0	0	1	1	1	0	0	0	0	0	0	0
PRPF6	0	0	0	1	1	1	1	0	0	0	0	0	0
PRPF8	0	0	0	1	1	1	1	0	0	0	0	0	0
RAD21	0	0	0	0	0	0	0	0	0	0	0	0	0
RBM8A	0	0	0	1	1	1	0	1	1	1	1	0	0
RBMX	0	0	0	1	1	1	0	0	0	0	0	0	0
RFC2	0	0	0	0	0	0	0	0	0	0	0	0	0
RFC4	0	0	0	0	0	0	0	0	0	0	0	0	0
RPA1	0	0	0	0	0	0	0	0	0	0	0	0	0
RPA2	0	0	0	0	0	0	0	0	0	0	0	0	0
RPA3	0	0	0	0	0	0	0	0	0	0	0	0	0
RRM2	0	0	0	0	0	0	0	0	0	0	0	0	0
SF3A1	0	0	0	1	1	1	0	0	0	0	0	0	0
SF3A2	0	0	0	1	1	1	0	0	0	0	0	0	0
SF3A3	0	0	0	1	1	1	0	0	0	0	0	0	0
SF3B1	0	0	0	1	1	1	1	0	0	0	0	0	0
SF3B14	0	0	0	1	1	1	1	0	0	0	0	0	0
SF3B2	0	0	0	1	1	1	1	0	0	0	0	0	0
SF3B3	0	0	0	1	1	1	1	0	0	0	0	0	0
SF3B5	0	0	0	1	1	1	1	0	0	0	0	0	0
SMC1A	0	0	0	1	1	1	0	0	0	0	0	0	0
SMC3	0	0	0	0	0	0	0	0	0	0	0	0	0
SNRNP200	0	0	0	1	1	1	1	0	0	0	0	0	0
SNRNP40	0	0	0	1	1	1	1	0	0	0	0	0	0
SNRNP70	0	0	0	1	1	1	0	0	0	0	0	0	0
SNRPA1	0	0	0	1	1	1	0	0	0	0	0	0	0

NAME	REACTOME_METABOLISM_OF_NON_CODING_RNA_	REACTOME_TRANSPORT_OF_MATURE_TRANSCRIPT_TO_CYTOPLASM_	REACTOME_TRANSPORT_OF_MATURE_MRNA_DERIVED_FROM_AN_INTRONLESS_TRANSCRIPT_	REACTOME_INTERACTIONS_OF_VPR_WITH_HOST_CELLULAR_PROTEINS_	REACTOME_SLC_MEDIATED_TRANSMEMBRANE_TRANSPORT_	REACTOME_GLUCOSE_TRANSPORT_	REACTOME_NEP_NS2_INTERACTS_WITH_THE_CELLULAR_EXPORT_MACHINERY_	REACTOME_REGULATION_OF_GLUCOKINASE_BY_GLUCOKINASE_REGULATORY_PROTEIN_	REACTOME_TRANSPORT_OF_RIBONUCLEOPROTEINS_INTO_THE_HOST_NUCLEUS_	REACTOME_RNA_POL_II_PRE_TRANSCRIPTION_EVENTS_	REACTOME_DNA_REPAIR_	REACTOME_NUCLEOTIDE_EXCISION_REPAIR_	REACTOME_MITOTIC_PROMETAPHASE_
PRPF4	0	0	0	0	0	0	0	0	0	0	0	0	0
PRPF6	0	0	0	0	0	0	0	0	0	0	0	0	0
PRPF8	0	0	0	0	0	0	0	0	0	0	0	0	0
RAD21	0	0	0	0	0	0	0	0	0	0	0	0	1
RBM8A	0	1	0	0	0	0	0	0	0	0	0	0	0
RBMX	0	0	0	0	0	0	0	0	0	0	0	0	0
RFC2	0	0	0	0	0	0	0	0	0	0	1	1	0
RFC4	0	0	0	0	0	0	0	0	0	0	1	1	0
RPA1	0	0	0	0	0	0	0	0	0	0	1	1	0
RPA2	0	0	0	0	0	0	0	0	0	0	1	1	0
RPA3	0	0	0	0	0	0	0	0	0	0	1	1	0
RRM2	0	0	0	0	0	0	0	0	0	0	0	0	0
SF3A1	0	0	0	0	0	0	0	0	0	0	0	0	0
SF3A2	0	0	0	0	0	0	0	0	0	0	0	0	0
SF3A3	0	0	0	0	0	0	0	0	0	0	0	0	0
SF3B1	0	0	0	0	0	0	0	0	0	0	0	0	0
SF3B14	0	0	0	0	0	0	0	0	0	0	0	0	0
SF3B2	0	0	0	0	0	0	0	0	0	0	0	0	0
SF3B3	0	0	0	0	0	0	0	0	0	0	0	0	0
SF3B5	0	0	0	0	0	0	0	0	0	0	0	0	0
SMC1A	0	0	0	0	0	0	0	0	0	0	0	0	1
SMC3	0	0	0	0	0	0	0	0	0	0	0	0	1
SNRNP200	0	0	0	0	0	0	0	0	0	0	0	0	0
SNRNP40	0	0	0	0	0	0	0	0	0	0	0	0	0
SNRNP70	0	0	0	0	0	0	0	0	0	0	0	0	0
SNRPA1	0	0	0	0	0	0	0	0	0	0	0	0	0

NAME	REACTOME_MEIOSIS_	REACTOME_CHROMOSOME_MAINTENANCE_	REACTOME_CELL_CYCLE_MITOTIC_	REACTOME_CELL_CYCLE_	REACTOME_DNA_REPLICATION_	REACTOME_MITOTIC_M_M_G1_PHASES_	REACTOME_SYNTHESIS_OF_DNA_	REACTOME_S_PHASE_	REACTOME_CELL_CYCLE_CHECKPOINTS_	REACTOME_G1_S_TRANSITION_	REACTOME_MITOTIC_G1_G1_S_PHASES_	REACTOME_FACTORS_INVOLVED_IN_MEGAKARYOCYTE_DEVELOPMENT_AND_PLATELET_PRODUCTION	KEGG_SPLICEOSOME_
PRPF4	0	0	0	0	0	0	0	0	0	0	0	0	1
PRPF6	0	0	0	0	0	0	0	0	0	0	0	0	1
PRPF8	0	0	0	0	0	0	0	0	0	0	0	0	1
RAD21	1	1	1	1	1	1	0	0	0	0	0	0	0
RBM8A	0	0	0	0	0	0	0	0	0	0	0	0	1
RBMX	0	0	0	0	0	0	0	0	0	0	0	0	1
RFC2	0	1	1	1	1	0	1	1	1	0	0	0	0
RFC4	0	1	1	1	1	0	1	1	1	0	0	0	0
RPA1	1	1	1	1	1	1	1	1	1	1	1	0	0
RPA2	1	1	1	1	1	1	1	1	1	1	1	0	0
RPA3	1	1	1	1	1	1	1	1	1	1	1	0	0
RRM2	0	0	1	1	0	0	0	0	0	1	1	0	0
SF3A1	0	0	0	0	0	0	0	0	0	0	0	0	1
SF3A2	0	0	0	0	0	0	0	0	0	0	0	0	1
SF3A3	0	0	0	0	0	0	0	0	0	0	0	0	1
SF3B1	0	0	0	0	0	0	0	0	0	0	0	0	1
SF3B14	0	0	0	0	0	0	0	0	0	0	0	0	1
SF3B2	0	0	0	0	0	0	0	0	0	0	0	0	1
SF3B3	0	0	0	0	0	0	0	0	0	0	0	0	1
SF3B5	0	0	0	0	0	0	0	0	0	0	0	0	1
SMC1A	1	1	1	1	1	1	0	0	0	0	0	0	0
SMC3	1	1	1	1	1	1	0	0	0	0	0	0	0
SNRNP200	0	0	0	0	0	0	0	0	0	0	0	0	1
SNRNP40	0	0	0	0	0	0	0	0	0	0	0	0	1
SNRNP70	0	0	0	0	0	0	0	0	0	0	0	0	1
SNRPA1	0	0	0	0	0	0	0	0	0	0	0	0	1

NAME	KEGG_RNA_DEGRADATION_	KEGG_DNA_REPLICATION_	KEGG_CELL_CYCLE_	KEGG_OOCYTE_MEIOSIS_	KEGG_PYRIMIDINE_METABOLISM_	KEGG_VALINE_LEUCINE_AND_ISOLEUCINE_DEGRADATION_
PRPF4	0	0	0	0	0	0
PRPF6	0	0	0	0	0	0
PRPF8	0	0	0	0	0	0
RAD21	0	0	1	0	0	0
RBM8A	0	0	0	0	0	0
RBMX	0	0	0	0	0	0
RFC2	0	1	0	0	0	0
RFC4	0	1	0	0	0	0
RPA1	0	1	0	0	0	0
RPA2	0	1	0	0	0	0
RPA3	0	1	0	0	0	0
RRM2	0	0	0	0	1	0
SF3A1	0	0	0	0	0	0
SF3A2	0	0	0	0	0	0
SF3A3	0	0	0	0	0	0
SF3B1	0	0	0	0	0	0
SF3B14	0	0	0	0	0	0
SF3B2	0	0	0	0	0	0
SF3B3	0	0	0	0	0	0
SF3B5	0	0	0	0	0	0
SMC1A	0	0	1	1	0	0
SMC3	0	0	1	1	0	0
SNRNP200	0	0	0	0	0	0
SNRNP40	0	0	0	0	0	0
SNRNP70	0	0	0	0	0	0
SNRPA1	0	0	0	0	0	0

Table S6 The differential proteins contribute to the enrichment of the significant pathways for the public dataset

Core enrichment proteins in up-regulated pathways of hiPSCs/HFs (hiPSCs vs HFs)

NAME	REACTOME_MITOCHONDRIAL_PROTEIN_IMPORT_	REACTOME_DEADENYLATION_DEPENDENT_MRNA_DECAY_	REACTOME_RNA_POL_I_RNA_POL_III_AND_MITOCHONDRIAL_TRANSCRIPTION_	REACTOME_PROCESSING_OF_CAPPED_INTRON_CONTAINING_PRE_MRNA_	REACTOME_MRNA_PROCESSING_	REACTOME_MRNA_SPLICING_	REACTOME_MRNA_SPLICING_MINOR_PATHWAY_	REACTOME_TRANSCRIPTION_	REACTOME_RNA_POL_II_TRANSCRIPTION_	REACTOME_CLEAVAGE_OF_GROWING_TRANSCRIPT_IN_THE_TERMINATION_REGION__	REACTOME_MRNA_3_END_PROCESSING_	REACTOME_HIV_LIFE_CYCLE_	REACTOME_LATE_PHASE_OF_HIV_LIFE_CYCLE_
SNRPB	0	0	0	1	1	1	1	1	1	1	0	0	0
SNRPB2	0	0	0	1	1	1	0	0	0	0	0	0	0
SNRPD1	0	0	0	1	1	1	1	0	0	0	0	0	0
SNRPD2	0	0	0	1	1	1	1	0	0	0	0	0	0
SNRPD3	0	0	0	1	1	1	1	1	1	1	0	0	0
SNRPE	0	0	0	1	1	1	1	1	1	1	0	0	0
SNRPF	0	0	0	1	1	1	1	1	1	1	0	0	0
SRSF1	0	0	0	1	1	1	1	1	1	1	1	0	0
SRSF3	0	0	0	1	1	1	0	1	1	1	1	0	0
SRSF9	0	0	0	1	1	1	0	1	1	1	1	0	0
STAG2	0	0	0	0	0	0	0	0	0	0	0	0	0
THOC4	0	0	0	1	1	1	0	1	1	1	1	0	0
XAB2	0	0	0	0	0	0	0	0	0	0	0	0	0
AAAS	0	0	0	1	1	0	0	0	0	0	0	1	1
ACO2	1	0	0	0	0	0	0	0	0	0	0	0	0
AKAP1	0	0	0	0	0	0	0	0	0	0	0	0	0
BANF1	0	0	0	0	0	0	0	0	0	0	0	1	0
BLM	0	0	0	0	0	0	0	0	0	0	0	0	0
C2orf29	0	1	0	0	0	0	0	0	0	0	0	0	0
CBX3	0	0	1	0	0	0	0	1	0	0	0	0	0
CBX5	0	0	0	0	0	0	0	0	0	0	0	0	0
CCAR1	0	0	0	1	1	1	0	0	0	0	0	0	0
CDCA8	0	0	0	0	0	0	0	0	0	0	0	0	0
CKS1B	0	0	0	0	0	0	0	0	0	0	0	0	0
CLNS1A	0	0	0	0	0	0	0	0	0	0	0	0	0
CPSF1	0	0	0	1	1	1	0	1	1	1	1	0	0

NAME	REACTOME_METABOLISM_OF_NON_CODING_RNA_	REACTOME_TRANSPORT_OF_MATURE_TRANSCRIPT_TO_CYTOPLASM_	REACTOME_TRANSPORT_OF_MATURE_MRNA_DERIVED_FROM_AN_INTRONLESS_TRANSCRIPT_	REACTOME_INTERACTIONS_OF_VPR_WITH_HOST_CELLULAR_PROTEINS_	REACTOME_SLC_MEDIATED_TRANSMEMBRANE_TRANSPORT_	REACTOME_GLUCOSE_TRANSPORT_	REACTOME_NEP_NS2_INTERACTS_WITH_THE_CELLULAR_EXPORT_MACHINERY_	REACTOME_REGULATION_OF_GLUCOKINASE_BY_GLUCOKINASE_REGULATORY_PROTEIN_	REACTOME_TRANSPORT_OF_RIBONUCLEOPROTEINS_INTO_THE_HOST_NUCLEUS_	REACTOME_RNA_POL_II_PRE_TRANSCRIPTION_EVENTS_	REACTOME_DNA_REPAIR_	REACTOME_NUCLEOTIDE_EXCISION_REPAIR_	REACTOME_MITOTIC_PROMETAPHASE_
SNRPB	1	0	0	0	0	0	0	0	0	0	0	0	0
SNRPB2	0	0	0	0	0	0	0	0	0	0	0	0	0
SNRPD1	1	0	0	0	0	0	0	0	0	0	0	0	0
SNRPD2	1	0	0	0	0	0	0	0	0	0	0	0	0
SNRPD3	1	0	0	0	0	0	0	0	0	0	0	0	0
SNRPE	1	0	0	0	0	0	0	0	0	0	0	0	0
SNRPF	1	0	0	0	0	0	0	0	0	0	0	0	0
SRSF1	0	1	0	0	0	0	0	0	0	0	0	0	0
SRSF3	0	1	0	0	0	0	0	0	0	0	0	0	0
SRSF9	0	1	0	0	0	0	0	0	0	0	0	0	0
STAG2	0	0	0	0	0	0	0	0	0	0	0	0	1
THOC4	0	1	1	0	0	0	0	0	0	0	0	0	0
XAB2	0	0	0	0	0	0	0	0	0	0	1	1	0
AAAS	1	1	1	1	1	1	1	1	1	0	0	0	0
ACO2	0	0	0	0	0	0	0	0	0	0	0	0	0
AKAP1	0	0	0	0	0	0	0	0	0	0	0	0	0
BANF1	0	0	0	1	0	0	0	0	0	0	0	0	0
BLM	0	0	0	0	0	0	0	0	0	0	0	0	0
C2orf29	0	0	0	0	0	0	0	0	0	0	0	0	0
CBX3	0	0	0	0	0	0	0	0	0	0	0	0	0
CBX5	0	0	0	0	0	0	0	0	0	0	0	0	0
CCAR1	0	0	0	0	0	0	0	0	0	0	0	0	0
CDCA8	0	0	0	0	0	0	0	0	0	0	0	0	1
CKS1B	0	0	0	0	0	0	0	0	0	0	0	0	0
CLNS1A	1	0	0	0	0	0	0	0	0	0	0	0	0
CPSF1	0	1	1	0	0	0	0	0	0	0	0	0	0

NAME	REACTOME_MEIOSIS_	REACTOME_CHROMOSOME_MAINTENANCE_	REACTOME_CELL_CYCLE_MITOTIC_	REACTOME_CELL_CYCLE_	REACTOME_DNA_REPLICATION_	REACTOME_MITOTIC_M_M_G1_PHASES_	REACTOME_SYNTHESIS_OF_DNA_	REACTOME_S_PHASE_	REACTOME_CELL_CYCLE_CHECKPOINTS_	REACTOME_G1_S_TRANSITION_	REACTOME_MITOTIC_G1_G1_S_PHASES_	REACTOME_FACTORS_INVOLVED_IN_MEGAKARYOCYTE_DEVELOPMENT_AND_PLATELET_PRODUCTION_	KEGG_SPLICEOSOME_
SNRPB	0	0	0	0	0	0	0	0	0	0	0	0	1
SNRPB2	0	0	0	0	0	0	0	0	0	0	0	0	1
SNRPD1	0	0	0	0	0	0	0	0	0	0	0	0	1
SNRPD2	0	0	0	0	0	0	0	0	0	0	0	0	1
SNRPD3	0	0	0	0	0	0	0	0	0	0	0	0	1
SNRPE	0	0	0	0	0	0	0	0	0	0	0	0	1
SNRPF	0	0	0	0	0	0	0	0	0	0	0	0	1
SRSF1	0	0	0	0	0	0	0	0	0	0	0	0	1
SRSF3	0	0	0	0	0	0	0	0	0	0	0	0	1
SRSF9	0	0	0	0	0	0	0	0	0	0	0	0	1
STAG2	1	1	1	1	1	1	0	0	0	0	0	0	0
THOC4	0	0	0	0	0	0	0	0	0	0	0	0	1
XAB2	0	0	0	0	0	0	0	0	0	0	0	0	1
AAAS	0	0	0	0	0	0	0	0	0	0	0	0	0
ACO2	0	0	0	0	0	0	0	0	0	0	0	0	0
AKAP1	0	0	0	0	0	0	0	0	0	0	0	1	1
BANF1	0	0	0	0	0	0	0	0	0	0	0	0	0
BLM	1	0	0	0	0	0	0	0	0	0	0	0	0
C2orf29	0	0	0	0	0	0	0	0	0	0	0	0	0
CBX3	0	0	0	0	0	0	0	0	0	0	0	0	1
CBX5	0	0	0	0	0	0	0	0	0	0	0	1	0
CCAR1	0	0	0	0	0	0	0	0	0	0	0	0	1
CDCA8	0	0	1	1	1	1	0	0	0	0	0	0	1
CKS1B	0	0	1	1	0	0	0	1	0	1	1	0	1
CLNS1A	0	0	0	0	0	0	0	0	0	0	0	0	0
CPSF1	0	0	0	0	0	0	0	0	0	0	0	0	1

NAME	KEGG_RNA_DEGRADATION_	KEGG_DNA_REPLICATION_	KEGG_CELL_CYCLE_	KEGG_OOCYTE_MEIOSIS_	KEGG_PYRIMIDINE_METABOLISM_	KEGG_VALINE_LEUCINE_AND_ISOLEUCINE_DEGRADATION_
SNRPB	0	0	0	0	0	0
SNRPB2	0	0	0	0	0	0
SNRPD1	0	0	0	0	0	0
SNRPD2	0	0	0	0	0	0
SNRPD3	0	0	0	0	0	0
SNRPE	0	0	0	0	0	0
SNRPF	0	0	0	0	0	0
SRSF1	0	0	0	0	0	0
SRSF3	0	0	0	0	0	0
SRSF9	0	0	0	0	0	0
STAG2	0	0	1	0	0	0
THOC4	0	0	0	0	0	0
XAB2	0	0	0	0	0	0
AAAS	0	0	0	0	0	1
ACO2	0	0	0	0	0	1
AKAP1	0	0	0	0	0	0
BANF1	0	0	0	0	0	1
BLM	0	0	0	0	0	1
C2orf29	0	0	0	0	0	1
CBX3	0	0	0	0	0	0
CBX5	0	0	0	0	0	1
CCAR1	0	0	0	0	0	0
CDCA8	0	0	0	0	0	0
CKS1B	0	0	0	0	0	0
CLNS1A	0	0	0	0	1	0
CPSF1	0	0	0	0	0	0

Table S6 The differential proteins contribute to the enrichment of the significant pathways for the public dataset

Core enrichment proteins in up-regulated pathways of hiPSCs/HFs (hiPSCs vs HFs)

NAME	REACTOME_MITOCHONDRIAL_PROTEIN_IMPORT_	REACTOME_DEADENYLATION_DEPENDENT_MRNA_DECAY_	REACTOME_RNA_POL_I_RNA_POL_III_AND_MITOCHONDRIAL_TRANSCRIPTION_	REACTOME_PROCESSING_OF_CAPPED_INTRON_CONTAINING_PRE_MRNA_	REACTOME_MRNA_PROCESSING_	REACTOME_MRNA_SPLICING_	REACTOME_MRNA_SPLICING_MINOR_PATHWAY_	REACTOME_TRANSCRIPTION_	REACTOME_RNA_POL_II_TRANSCRIPTION_	REACTOME_CLEAVAGE_OF_GROWING_TRANSCRIPT_IN_THE_TERMINATION_REGION__	REACTOME_MRNA_3_END_PROCESSING_	REACTOME_HIV_LIFE_CYCLE_	REACTOME_LATE_PHASE_OF_HIV_LIFE_CYCLE_
CPSF2	0	0	0	1	1	1	0	1	1	1	1	0	0
CPSF3	0	0	0	1	1	1	0	1	1	1	1	0	0
CPSF7	0	0	0	1	1	1	0	1	1	1	1	0	0
CSTF1	0	0	0	1	1	1	0	1	1	1	1	0	0
CSTF2	0	0	0	1	1	1	0	1	1	1	1	0	0
CSTF3	0	0	0	1	1	1	0	1	1	1	1	0	0
CYC1	1	0	0	0	0	0	0	0	0	0	0	0	0
DDX20	0	0	0	0	0	0	0	0	0	0	0	0	0
DHX9	0	0	0	1	1	1	0	0	0	0	0	0	0
EHMT2	0	0	1	0	0	0	0	1	0	0	0	0	0
FUS	0	0	0	1	1	1	0	0	0	0	0	0	0
FXC1	1	0	0	0	0	0	0	0	0	0	0	0	0
FXN	1	0	0	0	0	0	0	0	0	0	0	0	0
GTF2E2	0	0	0	0	0	0	0	1	1	0	0	1	1
GTF3C2	0	0	1	0	0	0	0	1	0	0	0	0	0
GTF3C3	0	0	1	0	0	0	0	1	0	0	0	0	0
GTF3C4	0	0	1	0	0	0	0	1	0	0	0	0	0
H2AFX	0	0	0	0	0	0	0	1	0	0	0	0	0
HIST2H4B	0	0	1	0	0	0	0	1	0	0	0	0	0
HIST3H2BB	0	0	1	0	0	0	0	1	0	0	0	0	0
HMGA1	0	0	0	0	0	0	0	0	0	0	0	1	0
HNRNPA0	0	0	0	1	1	1	0	0	0	0	0	0	0
HNRNPA2B1	0	0	0	1	1	1	0	0	0	0	0	0	0
HNRNPD	0	0	0	1	1	1	0	0	0	0	0	0	0
HNRNPF	0	0	0	1	1	1	0	0	0	0	0	0	0
HNRNPH1	0	0	0	1	1	1	0	0	0	0	0	0	0

NAME	REACTOME_METABOLISM_OF_NON_CODING_RNA_	REACTOME_TRANSPORT_OF_MATURE_TRANSCRIPT_TO_CYTOPLASM_	REACTOME_TRANSPORT_OF_MATURE_MRNA_DERIVED_FROM_AN_INTRONLESS_TRANSCRIPT_	REACTOME_INTERACTIONS_OF_VPR_WITH_HOST_CELLULAR_PROTEINS_	REACTOME_SLC_MEDIATED_TRANSMEMBRANE_TRANSPORT_	REACTOME_GLUCOSE_TRANSPORT_	REACTOME_NEP_NS2_INTERACTS_WITH_THE_CELLULAR_EXPORT_MACHINERY_	REACTOME_REGULATION_OF_GLUCOKINASE_BY_GLUCOKINASE_REGULATORY_PROTEIN_	REACTOME_TRANSPORT_OF_RIBONUCLEOPROTEINS_INTO_THE_HOST_NUCLEUS_	REACTOME_RNA_POL_II_PRE_TRANSCRIPTION_EVENTS_	REACTOME_DNA_REPAIR_	REACTOME_NUCLEOTIDE_EXCISION_REPAIR_	REACTOME_MITOTIC_PROMETAPHASE_
CPSF2	0	1	1	0	0	0	0	0	0	0	0	0	0
CPSF3	0	1	1	0	0	0	0	0	0	0	0	0	0
CPSF7	0	0	0	0	0	0	0	0	0	0	0	0	0
CSTF1	0	0	0	0	0	0	0	0	0	0	0	0	0
CSTF2	0	0	0	0	0	0	0	0	0	0	0	0	0
CSTF3	0	0	0	0	0	0	0	0	0	0	0	0	0
CYC1	0	0	0	0	0	0	0	0	0	0	0	0	0
DDX20	1	0	0	0	0	0	0	0	0	0	0	0	0
DHX9	0	0	0	0	0	0	0	0	0	0	0	0	0
EHMT2	0	0	0	0	0	0	0	0	0	0	0	0	0
FUS	0	0	0	0	0	0	0	0	0	0	0	0	0
FXC1	0	0	0	0	0	0	0	0	0	0	0	0	0
FXN	0	0	0	0	0	0	0	0	0	0	0	0	0
GTF2E2	0	0	0	0	0	0	0	0	0	1	0	0	0
GTF3C2	0	0	0	0	0	0	0	0	0	0	0	0	0
GTF3C3	0	0	0	0	0	0	0	0	0	0	0	0	0
GTF3C4	0	0	0	0	0	0	0	0	0	0	0	0	0
H2AFX	0	0	0	0	0	0	0	0	0	0	1	0	0
HIST2H4B	0	0	0	0	0	0	0	0	0	0	0	0	0
HIST3H2BB	0	0	0	0	0	0	0	0	0	0	0	0	0
HMGA1	0	0	0	1	0	0	0	0	0	0	0	0	0
HNRNPA0	0	0	0	0	0	0	0	0	0	0	0	0	0
HNRNPA2B1	0	0	0	0	0	0	0	0	0	0	0	0	0
HNRNPD	0	0	0	0	0	0	0	0	0	0	0	0	0
HNRNPF	0	0	0	0	0	0	0	0	0	0	0	0	0
HNRNPH1	0	0	0	0	0	0	0	0	0	0	0	0	0

NAME	REACTOME_MEIOSIS_	REACTOME_CHROMOSOME_MAINTENANCE_	REACTOME_CELL_CYCLE_MITOTIC_	REACTOME_CELL_CYCLE_	REACTOME_DNA_REPLICATION_	REACTOME_MITOTIC_M_M_G1_PHASES_	REACTOME_SYNTHESIS_OF_DNA_	REACTOME_S_PHASE_	REACTOME_CELL_CYCLE_CHECKPOINTS_	REACTOME_G1_S_TRANSITION_	REACTOME_MITOTIC_G1_G1_S_PHASES_	REACTOME_FACTORS_INVOLVED_IN_MEGAKARYOCYTE_DEVELOPMENT_AND_PLATELET_PRODUCTION	KEGG_SPLICEOSOME_
CPSF2	0	0	0	0	0	0	0	0	0	0	0	0	0
CPSF3	0	0	0	0	0	0	0	0	0	0	0	0	1
CPSF7	0	0	0	0	0	0	0	0	0	0	0	0	1
CSTF1	0	0	0	0	0	0	0	0	0	0	0	0	1
CSTF2	0	0	0	0	0	0	0	0	0	0	0	0	1
CSTF3	0	0	0	0	0	0	0	0	0	0	0	0	0
CYC1	0	0	0	0	0	0	0	0	0	0	0	0	0
DDX20	0	0	0	0	0	0	0	0	0	0	0	0	0
DHX9	0	0	0	0	0	0	0	0	0	0	0	0	0
EHMT2	0	0	0	0	0	0	0	0	0	0	0	0	0
FUS	0	0	0	0	0	0	0	0	0	0	0	0	0
FXC1	0	0	0	0	0	0	0	0	0	0	0	0	1
FXN	0	0	0	0	0	0	0	0	0	0	0	0	1
GTF2E2	0	0	0	0	0	0	0	0	0	0	0	0	0
GTF3C2	0	0	0	0	0	0	0	0	0	0	0	0	0
GTF3C3	0	0	0	0	0	0	0	0	0	0	0	0	0
GTF3C4	0	0	0	0	0	0	0	0	0	0	0	0	0
H2AFX	0	0	0	0	0	0	0	0	0	0	0	0	0
HIST2H4B	1	1	0	1	0	0	0	0	0	0	0	0	1
HIST3H2BB	1	1	0	1	0	0	0	0	0	0	0	0	1
HMGA1	0	0	0	0	0	0	0	0	0	0	0	0	1
HNRNPA0	0	0	0	0	0	0	0	0	0	0	0	0	1
HNRNPA2B1	0	0	0	0	0	0	0	0	0	0	0	0	1
HNRNPD	0	0	0	0	0	0	0	0	0	0	0	0	1
HNRNPF	0	0	0	0	0	0	0	0	0	0	0	0	1
HNRNPH1	0	0	0	0	0	0	0	0	0	0	0	0	1

NAME	KEGG_RNA_DEGRADATION_	KEGG_DNA_REPLICATION_	KEGG_CELL_CYCLE_	KEGG_OOCYTE_MEIOSIS_	KEGG_PYRIMIDINE_METABOLISM_	KEGG_VALINE_LEUCINE_AND_ISOLEUCINE_DEGRADATION_
CPSF2	0	0	0	0	0	1
CPSF3	0	0	0	0	0	0
CPSF7	0	0	0	0	0	0
CSTF1	0	0	0	0	0	0
CSTF2	0	0	0	0	0	0
CSTF3	0	0	0	0	1	0
CYC1	1	0	0	0	0	0
DDX20	0	0	0	0	0	1
DHX9	0	0	0	0	0	1
EHMT2	0	0	0	0	0	1
FUS	0	0	0	1	0	0
FXC1	0	0	0	0	0	0
FXN	1	0	0	0	0	0
GTF2E2	0	0	0	0	0	1
GTF3C2	0	0	0	0	1	0
GTF3C3	0	0	0	0	1	0
GTF3C4	0	0	0	0	1	0
H2AFX	0	0	0	0	1	0
HIST2H4B	0	0	0	0	0	0
HIST3H2BB	0	0	0	0	0	0
HMGA1	0	0	0	0	0	0
HNRNPA0	0	0	0	0	0	0
HNRNPA2B1	0	0	0	0	0	0
HNRNPD	0	0	0	0	0	0
HNRNPF	0	0	0	0	0	0
HNRNPH1	0	0	0	0	0	0

Table S6 The differential proteins contribute to the enrichment of the significant pathways for the public dataset

Core enrichment proteins in up-regulated pathways of hiPSCs/HFs (hiPSCs vs HFs)

NAME	REACTOME_MITOCHONDRIAL_PROTEIN_IMPORT_	REACTOME_DEADENYLATION_DEPENDENT_MRNA_DECAY_	REACTOME_RNA_POL_I_RNA_POL_III_AND_MITOCHONDRIAL_TRANSCRIPTION_	REACTOME_PROCESSING_OF_CAPPED_INTRON_CONTAINING_PRE_MRNA_	REACTOME_MRNA_PROCESSING_	REACTOME_MRNA_SPLICING_	REACTOME_MRNA_SPLICING_MINOR_PATHWAY_	REACTOME_TRANSCRIPTION_	REACTOME_RNA_POL_II_TRANSCRIPTION_	REACTOME_CLEAVAGE_OF_GROWING_TRANSCRIPT_IN_THE_TERMINATION_REGION__	REACTOME_MRNA_3_END_PROCESSING_	REACTOME_HIV_LIFE_CYCLE_	REACTOME_LATE_PHASE_OF_HIV_LIFE_CYCLE_
HNRNPL	0	0	0	1	1	1	0	0	0	0	0	0	0
HNRNPR	0	0	0	1	1	1	0	0	0	0	0	0	0
HSPA9	1	0	0	0	0	0	0	0	0	0	0	0	0
INCENP	0	0	0	0	0	0	0	0	0	0	0	0	0
JMJD1C	0	0	0	0	0	0	0	0	0	0	0	0	0
KDM1A	0	0	0	0	0	0	0	0	0	0	0	0	0
KIF11	0	0	0	0	0	0	0	0	0	0	0	0	0
KIF20A	0	0	0	0	0	0	0	0	0	0	0	0	0
KIF22	0	0	0	0	0	0	0	0	0	0	0	0	0
KIF23	0	0	0	0	0	0	0	0	0	0	0	0	0
KIF2C	0	0	0	0	0	0	0	0	0	0	0	0	0
KIFC1	0	0	0	0	0	0	0	0	0	0	0	0	0
LMNB1	0	0	0	0	0	0	0	0	0	0	0	0	0
METTL3	0	0	0	1	1	0	0	0	0	0	0	0	0
MLF1IP	0	0	0	0	0	0	0	0	0	0	0	0	0
MPG	0	0	0	0	0	0	0	0	0	0	0	0	0
NPM1	0	0	0	0	0	0	0	0	0	0	0	0	0
NUDT21	0	0	0	1	1	1	0	1	1	1	1	0	0
NUP107	0	0	0	1	1	0	0	0	0	0	0	1	1
NUP133	0	0	0	1	1	0	0	0	0	0	0	1	1
NUP153	0	0	0	1	1	0	0	0	0	0	0	1	1
NUP155	0	0	0	1	1	0	0	0	0	0	0	1	1
NUP205	0	0	0	1	1	0	0	0	0	0	0	1	1
NUP210	0	0	0	1	1	0	0	0	0	0	0	1	1
NUP214	0	0	0	1	1	0	0	0	0	0	0	1	1
NUP35	0	0	0	1	1	0	0	0	0	0	0	1	1

NAME	REACTOME_METABOLISM_OF_NON_CODING_RNA_	REACTOME_TRANSPORT_OF_MATURE_TRANSCRIPT_TO_CYTOPLASM_	REACTOME_TRANSPORT_OF_MATURE_MRNA_DERIVED_FROM_AN_INTRONLESS_TRANSCRIPT_	REACTOME_INTERACTIONS_OF_VPR_WITH_HOST_CELLULAR_PROTEINS_	REACTOME_SLC_MEDIATED_TRANSMEMBRANE_TRANSPORT_	REACTOME_GLUCOSE_TRANSPORT_	REACTOME_NEP_NS2_INTERACTS_WITH_THE_CELLULAR_EXPORT_MACHINERY_	REACTOME_REGULATION_OF_GLUCOKINASE_BY_GLUCOKINASE_REGULATORY_PROTEIN_	REACTOME_TRANSPORT_OF_RIBONUCLEOPROTEINS_INTO_THE_HOST_NUCLEUS_	REACTOME_RNA_POL_II_PRE_TRANSCRIPTION_EVENTS_	REACTOME_DNA_REPAIR_	REACTOME_NUCLEOTIDE_EXCISION_REPAIR_	REACTOME_MITOTIC_PROMETAPHASE_
HNRNPL	0	0	0	0	0	0	0	0	0	0	0	0	0
HNRNPR	0	0	0	0	0	0	0	0	0	0	0	0	0
HSPA9	0	0	0	0	0	0	0	0	0	0	0	0	0
INCENP	0	0	0	0	0	0	0	0	0	0	0	0	1
JMJD1C	0	0	0	0	0	0	0	0	0	0	0	0	0
KDM1A	0	0	0	0	0	0	0	0	0	0	0	0	0
KIF11	0	0	0	0	0	0	0	0	0	0	0	0	0
KIF20A	0	0	0	0	0	0	0	0	0	0	0	0	0
KIF22	0	0	0	0	0	0	0	0	0	0	0	0	0
KIF23	0	0	0	0	0	0	0	0	0	0	0	0	0
KIF2C	0	0	0	0	0	0	0	0	0	0	0	0	1
KIFC1	0	0	0	0	0	0	0	0	0	0	0	0	0
LMNB1	0	0	0	0	0	0	0	0	0	0	0	0	0
METTL3	0	0	0	0	0	0	0	0	0	0	0	0	0
MLF1IP	0	0	0	0	0	0	0	0	0	0	0	0	1
MPG	0	0	0	0	0	0	0	0	0	0	1	0	0
NPM1	0	0	0	0	0	0	0	0	0	0	0	0	0
NUDT21	0	0	0	0	0	0	0	0	0	0	0	0	0
NUP107	1	1	1	1	1	1	1	1	1	0	0	0	0
NUP133	1	1	1	1	1	1	1	1	1	0	0	0	0
NUP153	1	1	1	1	1	1	1	1	1	0	0	0	0
NUP155	1	1	1	1	1	1	1	1	1	0	0	0	0
NUP205	1	1	1	1	1	1	1	1	1	0	0	0	0
NUP210	1	1	1	1	1	1	1	1	1	0	0	0	0
NUP214	1	1	1	1	1	1	1	1	1	0	0	0	0
NUP35	1	1	1	1	1	1	1	1	1	0	0	0	0

NAME	REACTOME_MEIOSIS_	REACTOME_CHROMOSOME_MAINTENANCE_	REACTOME_CELL_CYCLE_MITOTIC_	REACTOME_CELL_CYCLE_	REACTOME_DNA_REPLICATION_	REACTOME_MITOTIC_M_M_G1_PHASES_	REACTOME_SYNTHESIS_OF_DNA_	REACTOME_S_PHASE_	REACTOME_CELL_CYCLE_CHECKPOINTS_	REACTOME_G1_S_TRANSITION_	REACTOME_MITOTIC_G1_G1_S_PHASES_	REACTOME_FACTORS_INVOLVED_IN_MEGAKARYOCYTE_DEVELOPMENT_AND_PLATELET_PRODUCTION	KEGG_SPLICEOSOME_
HNRNPL	0	0	0	0	0	0	0	0	0	0	0	0	0
HNRNPR	0	0	0	0	0	0	0	0	0	0	0	0	0
HSPA9	0	0	0	0	0	0	0	0	0	0	0	0	1
INCENP	0	0	1	1	1	1	0	0	0	0	0	0	0
JMJD1C	0	0	0	0	0	0	0	0	0	0	0	1	1
KDM1A	0	0	0	0	0	0	0	0	0	0	0	1	1
KIF11	0	0	0	0	0	0	0	0	0	0	0	1	1
KIF20A	0	0	1	1	1	1	0	0	0	0	0	1	1
KIF22	0	0	0	0	0	0	0	0	0	0	0	1	1
KIF23	0	0	0	1	1	1	0	0	0	0	0	0	1
KIF2C	0	0	1	1	1	1	0	0	0	0	0	1	1
KIFC1	0	0	0	0	0	0	0	0	0	0	0	1	1
LMNB1	1	1	0	1	0	0	0	0	0	0	0	0	1
METTL3	0	0	0	0	0	0	0	0	0	0	0	0	1
MLF1IP	0	1	1	1	1	1	0	0	0	0	0	0	1
MPG	0	0	0	0	0	0	0	0	0	0	0	0	0
NPM1	0	1	0	1	0	0	0	0	0	0	0	0	0
NUDT21	0	0	0	0	0	0	0	0	0	0	0	0	0
NUP107	0	0	0	1	1	1	0	0	0	0	0	0	0
NUP133	0	0	0	0	0	1	0	0	0	0	0	0	0
NUP153	0	0	0	0	0	0	0	0	0	0	0	0	0
NUP155	0	0	0	0	0	0	0	0	0	0	0	0	0
NUP205	0	0	0	0	0	0	0	0	0	0	0	0	0
NUP210	0	0	0	0	0	0	0	0	0	0	0	0	0
NUP214	0	0	0	0	0	0	0	0	0	0	0	0	0
NUP35	0	0	0	0	0	0	0	0	0	0	0	0	0

NAME	KEGG_RNA_DEGRADATION_	KEGG_DNA_REPLICATION_	KEGG_CELL_CYCLE_	KEGG_OOCYTE_MEIOSIS_	KEGG_PYRIMIDINE_METABOLISM_	KEGG_VALINE_LEUCINE_AND_ISOLEUCINE_DEGRADATION_
HNRNPL	0	1	0	0	0	0
HNRNPR	0	1	0	0	0	0
HSPA9	0	0	0	0	0	0
INCENP	1	0	0	0	0	0
JMJD1C	0	0	0	0	0	0
KDM1A	0	0	0	0	0	0
KIF11	0	0	0	0	0	0
KIF20A	0	0	0	0	0	0
KIF22	0	0	0	0	0	0
KIF23	0	0	0	0	0	0
KIF2C	0	0	0	0	0	0
KIFC1	0	0	0	0	0	0
LMNB1	0	0	0	0	0	0
METTL3	0	0	0	0	0	0
MLF1IP	0	0	0	0	0	0
MPG	0	0	0	0	0	0
NPM1	0	0	0	0	0	0
NUDT21	0	0	0	0	0	0
NUP107	0	0	0	0	0	0
NUP133	0	0	0	0	0	0
NUP153	0	0	0	0	0	0
NUP155	0	0	0	0	0	0
NUP205	0	0	0	0	0	0
NUP210	0	0	0	0	0	0
NUP214	0	0	0	0	0	0
NUP35	0	0	0	0	0	0

Table S6 The differential proteins contribute to the enrichment of the significant pathways for the public dataset

Core enrichment proteins in up-regulated pathways of hiPSCs/HFs (hiPSCs vs HFs)

NAME	REACTOME_MITOCHONDRIAL_PROTEIN_IMPORT_	REACTOME_DEADENYLATION_DEPENDENT_MRNA_DECAY_	REACTOME_RNA_POL_I_RNA_POL_III_AND_MITOCHONDRIAL_TRANSCRIPTION_	REACTOME_PROCESSING_OF_CAPPED_INTRON_CONTAINING_PRE_MRNA_	REACTOME_MRNA_PROCESSING_	REACTOME_MRNA_SPLICING_	REACTOME_MRNA_SPLICING_MINOR_PATHWAY_	REACTOME_TRANSCRIPTION_	REACTOME_RNA_POL_II_TRANSCRIPTION_	REACTOME_CLEAVAGE_OF_GROWING_TRANSCRIPT_IN_THE_TERMINATION_REGION__	REACTOME_MRNA_3_END_PROCESSING_	REACTOME_HIV_LIFE_CYCLE_	REACTOME_LATE_PHASE_OF_HIV_LIFE_CYCLE_
NUP37	0	0	0	1	1	0	0	0	0	0	0	1	1
NUP43	0	0	0	1	1	0	0	0	0	0	0	1	1
NUP50	0	0	0	1	1	0	0	0	0	0	0	1	1
NUP54	0	0	0	1	1	0	0	0	0	0	0	1	1
NUP62	0	0	0	1	1	0	0	0	0	0	0	1	1
NUP88	0	0	0	1	1	0	0	0	0	0	0	1	1
NUP93	0	0	0	1	1	0	0	0	0	0	0	1	1
NUPL1	0	0	0	1	1	0	0	0	0	0	0	1	1
NXF1	0	0	0	1	1	0	0	0	0	0	0	0	0
PABPN1	0	0	0	1	1	1	0	1	1	1	1	0	0
PAPOLA	0	0	0	1	1	0	0	1	1	1	1	0	0
PCM1	0	0	0	0	0	0	0	0	0	0	0	0	0
PCNA	0	0	0	0	0	0	0	0	0	0	0	0	0
PMPCA	1	0	0	0	0	0	0	0	0	0	0	0	0
PMPCB	1	0	0	0	0	0	0	0	0	0	0	0	0
POLB	0	0	0	0	0	0	0	0	0	0	0	0	0
POLRMT	0	0	1	0	0	0	0	1	0	0	0	0	0
PRKDC	0	0	0	0	0	0	0	0	0	0	0	0	0
PSIP1	0	0	0	0	0	0	0	0	0	0	0	1	0
PTBP1	0	0	0	1	1	1	0	0	0	0	0	0	0
RAE1	0	0	0	1	1	0	0	0	0	0	0	0	1
RANBP2	0	0	0	1	1	0	0	0	0	0	0	1	1
RBBP4	0	0	0	0	0	0	0	0	0	0	0	0	0
RBM5	0	0	0	1	1	0	0	0	0	0	0	0	0
RCC1	0	0	0	0	0	0	0	0	0	0	0	1	1
RCC2	0	0	0	0	0	0	0	0	0	0	0	0	0

NAME	REACTOME_METABOLISM_OF_NON_CODING_RNA_	REACTOME_TRANSPORT_OF_MATURE_TRANSCRIPT_TO_CYTOPLASM_	REACTOME_TRANSPORT_OF_MATURE_MRNA_DERIVED_FROM_AN_INTRONLESS_TRANSCRIPT_	REACTOME_INTERACTIONS_OF_VPR_WITH_HOST_CELLULAR_PROTEINS_	REACTOME_SLC_MEDIATED_TRANSMEMBRANE_TRANSPORT_	REACTOME_GLUCOSE_TRANSPORT_	REACTOME_NEP_NS2_INTERACTS_WITH_THE_CELLULAR_EXPORT_MACHINERY_	REACTOME_REGULATION_OF_GLUCOKINASE_BY_GLUCOKINASE_REGULATORY_PROTEIN_	REACTOME_TRANSPORT_OF_RIBONUCLEOPROTEINS_INTO_THE_HOST_NUCLEUS_	REACTOME_RNA_POL_II_PRE_TRANSCRIPTION_EVENTS_	REACTOME_DNA_REPAIR_	REACTOME_NUCLEOTIDE_EXCISION_REPAIR_	REACTOME_MITOTIC_PROMETAPHASE_
NUP37	1	1	1	1	1	1	1	1	1	0	0	0	0
NUP43	1	1	1	1	1	1	1	1	1	0	0	0	0
NUP50	1	1	1	1	1	1	1	1	1	0	0	0	0
NUP54	1	1	1	1	1	1	1	1	1	0	0	0	0
NUP62	1	1	1	1	1	1	1	1	1	0	0	0	0
NUP88	1	1	1	1	1	1	1	1	1	0	0	0	0
NUP93	1	1	1	1	1	1	1	1	1	0	0	0	0
NUPL1	1	1	1	1	1	1	1	1	1	0	0	0	0
NXF1	0	1	1	0	0	0	0	0	0	0	0	0	0
PABPN1	0	0	0	0	0	0	0	0	0	0	0	0	0
PAPOLA	0	0	0	0	0	0	0	0	0	0	0	0	0
PCM1	0	0	0	0	0	0	0	0	0	0	0	0	0
PCNA	0	0	0	0	0	0	0	0	0	0	1	1	0
PMPCA	0	0	0	0	0	0	0	0	0	0	0	0	0
PMPCB	0	0	0	0	0	0	0	0	0	0	0	0	0
POLB	0	0	0	0	0	0	0	0	0	0	1	0	0
POLRMT	0	0	0	0	0	0	0	0	0	0	0	0	0
PRKDC	0	0	0	0	0	0	0	0	0	0	1	0	0
PSIP1	0	0	0	1	0	0	0	0	0	0	0	0	0
PTBP1	0	0	0	0	0	0	0	0	0	0	0	0	0
RAE1	1	1	1	1	1	1	1	1	1	0	0	0	0
RANBP2	1	1	1	1	1	1	1	1	1	0	0	0	0
RBBP4	0	0	0	0	0	0	0	0	0	0	0	0	0
RBM5	0	0	0	0	0	0	0	0	0	0	0	0	0
RCC1	0	0	0	0	0	0	0	0	0	0	0	0	0
RCC2	0	0	0	0	0	0	0	0	0	0	0	0	1

NAME	REACTOME_MEIOSIS_	REACTOME_CHROMOSOME_MAINTENANCE_	REACTOME_CELL_CYCLE_MITOTIC_	REACTOME_CELL_CYCLE_	REACTOME_DNA_REPLICATION_	REACTOME_MITOTIC_M_M_G1_PHASES_	REACTOME_SYNTHESIS_OF_DNA_	REACTOME_S_PHASE_	REACTOME_CELL_CYCLE_CHECKPOINTS_	REACTOME_G1_S_TRANSITION_	REACTOME_MITOTIC_G1_G1_S_PHASES_	REACTOME_FACTORS_INVOLVED_IN_MEGAKARYOCYTE_DEVELOPMENT_AND_PLATELET_PRODUCTION	KEGG_SPLICEOSOME_
NUP37	0	0	0	0	0	0	0	0	0	0	0	0	0
NUP43	0	0	0	0	0	0	0	0	0	0	0	0	0
NUP50	0	0	0	0	0	0	0	0	0	0	0	0	0
NUP54	0	0	0	0	0	0	0	0	0	0	0	0	0
NUP62	0	0	0	0	0	0	0	0	0	0	0	0	0
NUP88	0	0	0	0	0	0	0	0	0	0	0	0	0
NUP93	0	0	0	0	0	0	0	0	0	0	0	0	0
NUPL1	0	0	0	0	0	0	0	0	0	0	0	0	0
NXF1	0	0	0	0	0	0	0	0	0	0	0	0	0
PABPN1	0	0	0	0	0	0	0	0	0	0	0	0	0
PAPOLA	0	0	0	0	0	0	0	0	0	0	0	0	0
PCM1	0	0	1	1	0	0	0	0	0	0	0	0	0
PCNA	0	0	0	0	0	0	0	0	0	0	0	0	0
PMPCA	0	0	0	0	0	0	0	0	0	0	0	0	0
PMPCB	0	0	0	0	0	0	0	0	0	0	0	0	0
POLB	0	0	0	0	0	0	0	0	0	0	0	0	0
POLRMT	0	0	0	0	0	0	0	0	0	0	0	0	0
PRKDC	0	0	0	0	0	0	0	0	0	0	0	0	0
PSIP1	0	0	0	0	0	0	0	0	0	0	0	0	0
PTBP1	0	0	0	0	0	0	0	0	0	0	0	0	0
RAE1	0	0	0	0	0	0	0	0	0	0	0	0	0
RANBP2	0	0	1	1	1	1	0	0	0	0	0	0	0
RBBP4	0	1	1	1	0	0	0	0	0	0	1	0	0
RBM5	0	0	0	0	0	0	0	0	0	0	0	0	0
RCC1	0	0	0	0	0	0	0	0	0	0	0	0	0
RCC2	0	0	1	1	1	1	0	0	0	0	0	0	0

NAME	KEGG_RNA_DEGRADATION_	KEGG_DNA_REPLICATION_	KEGG_CELL_CYCLE_	KEGG_OOCYTE_MEIOSIS_	KEGG_PYRIMIDINE_METABOLISM_	KEGG_VALINE_LEUCINE_AND_ISOLEUCINE_DEGRADATION_
NUP37	0	0	0	0	0	0
NUP43	0	0	0	0	0	0
NUP50	0	0	0	0	0	0
NUP54	0	0	0	0	0	0
NUP62	0	0	0	0	0	0
NUP88	0	0	0	0	0	0
NUP93	0	0	0	0	0	0
NUPL1	0	0	0	0	0	0
NXF1	0	0	0	0	0	0
PABPN1	0	0	0	0	0	0
PAPOLA	0	0	0	0	0	0
PCM1	0	0	0	0	0	0
PCNA	0	0	0	0	0	0
PMPCA	0	0	0	0	0	0
PMPCB	0	0	0	0	0	0
POLB	0	0	0	0	0	0
POLRMT	0	0	0	0	0	0
PRKDC	0	0	0	0	0	0
PSIP1	0	0	0	0	0	0
PTBP1	0	0	0	0	0	0
RAE1	0	0	0	0	0	0
RANBP2	0	0	0	0	0	0
RBBP4	0	0	0	0	0	0
RBM5	0	0	0	0	0	0
RCC1	0	0	0	0	0	0
RCC2	0	0	0	0	0	0

Table S6 The differential proteins contribute to the enrichment of the significant pathways for the public dataset

Core enrichment proteins in up-regulated pathways of hiPSCs/HFs (hiPSCs vs HFs)

NAME	REACTOME_MITOCHONDRIAL_PROTEIN_IMPORT_	REACTOME_DEADENYLATION_DEPENDENT_MRNA_DECAY_	REACTOME_RNA_POL_I_RNA_POL_III_AND_MITOCHONDRIAL_TRANSCRIPTION_	REACTOME_PROCESSING_OF_CAPPED_INTRON_CONTAINING_PRE_MRNA_	REACTOME_MRNA_PROCESSING_	REACTOME_MRNA_SPLICING_	REACTOME_MRNA_SPLICING_MINOR_PATHWAY_	REACTOME_TRANSCRIPTION_	REACTOME_RNA_POL_II_TRANSCRIPTION_	REACTOME_CLEAVAGE_OF_GROWING_TRANSCRIPT_IN_THE_TERMINATION_REGION__	REACTOME_MRNA_3_END_PROCESSING_	REACTOME_HIV_LIFE_CYCLE_	REACTOME_LATE_PHASE_OF_HIV_LIFE_CYCLE_
RNMT	0	0	0	0	1	0	0	1	1	0	0	1	1
RNPS1	0	0	0	1	1	1	0	1	1	1	1	0	0
SAMM50	1	0	0	0	0	0	0	0	0	0	0	0	0
SEH1L	0	0	0	1	1	0	0	0	0	0	0	1	1
SIN3A	0	0	0	0	0	0	0	0	0	0	0	0	0
SLC12A7	0	0	0	0	0	0	0	0	0	0	0	0	0
SLC25A13	1	0	0	0	0	0	0	0	0	0	0	0	0
SLC25A4	1	0	0	0	0	0	0	0	0	0	0	0	0
SLC25A5	0	0	0	0	0	0	0	0	0	0	0	0	0
SLC2A1	0	0	0	0	0	0	0	0	0	0	0	0	0
SLC2A3	0	0	0	0	0	0	0	0	0	0	0	0	0
SLC3A2	0	0	0	0	0	0	0	0	0	0	0	0	0
SLC4A7	0	0	0	0	0	0	0	0	0	0	0	0	0
SLC7A3	0	0	0	0	0	0	0	0	0	0	0	0	0
SLC7A5	0	0	0	0	0	0	0	0	0	0	0	0	0
SMARCA5	0	0	0	0	0	0	0	0	0	0	0	0	0
SMN2	0	0	0	0	0	0	0	0	0	0	0	0	0
SNRPA	0	0	0	1	1	1	0	0	0	0	0	0	0
SRRM1	0	0	0	1	1	1	0	1	1	1	1	0	0
SRSF11	0	0	0	1	1	1	0	1	1	1	1	0	0
SRSF4	0	0	0	1	1	1	0	1	1	1	1	0	0
SSRP1	0	0	0	0	0	0	0	1	1	0	0	1	1
SUGP1	0	0	0	1	1	1	0	0	0	0	0	0	0
SUPT16H	0	0	0	0	0	0	0	1	1	0	0	1	1
SUPT5H	0	0	0	0	1	0	0	1	1	0	0	1	1
SYNE2	0	0	0	0	0	0	0	0	0	0	0	0	0

NAME	REACTOME_METABOLISM_OF_NON_CODING_RNA_	REACTOME_TRANSPORT_OF_MATURE_TRANSCRIPT_TO_CYTOPLASM_	REACTOME_TRANSPORT_OF_MATURE_MRNA_DERIVED_FROM_AN_INTRONLESS_TRANSCRIPT_	REACTOME_INTERACTIONS_OF_VPR_WITH_HOST_CELLULAR_PROTEINS_	REACTOME_SLC_MEDIATED_TRANSMEMBRANE_TRANSPORT_	REACTOME_GLUCOSE_TRANSPORT_	REACTOME_NEP_NS2_INTERACTS_WITH_THE_CELLULAR_EXPORT_MACHINERY_	REACTOME_REGULATION_OF_GLUCOKINASE_BY_GLUCOKINASE_REGULATORY_PROTEIN_	REACTOME_TRANSPORT_OF_RIBONUCLEOPROTEINS_INTO_THE_HOST_NUCLEUS_	REACTOME_RNA_POL_II_PRE_TRANSCRIPTION_EVENTS_	REACTOME_DNA_REPAIR_	REACTOME_NUCLEOTIDE_EXCISION_REPAIR_	REACTOME_MITOTIC_PROMETAPHASE_
RNMT	0	0	0	0	0	0	0	0	0	0	0	0	0
RNPS1	0	1	0	0	0	0	0	0	0	0	0	0	0
SAMM50	0	0	0	0	0	0	0	0	0	0	0	0	0
SEH1L	1	1	1	1	1	1	1	1	1	0	0	0	0
SIN3A	0	0	0	0	0	0	0	0	0	0	0	0	0
SLC12A7	0	0	0	0	1	0	0	0	0	0	0	0	0
SLC25A13	0	0	0	0	0	0	0	0	0	0	0	0	0
SLC25A4	0	0	0	1	0	0	0	0	0	0	0	0	0
SLC25A5	0	0	0	1	0	0	0	0	0	0	0	0	0
SLC2A1	0	0	0	0	1	1	0	0	0	0	0	0	0
SLC2A3	0	0	0	0	1	1	0	0	0	0	0	0	0
SLC3A2	0	0	0	0	1	0	0	0	0	0	0	0	0
SLC4A7	0	0	0	0	1	0	0	0	0	0	0	0	0
SLC7A3	0	0	0	0	1	0	0	0	0	0	0	0	0
SLC7A5	0	0	0	0	1	0	0	0	0	0	0	0	0
SMARCA5	0	0	0	0	0	0	0	0	0	0	0	0	0
SMN2	1	0	0	0	0	0	0	0	0	0	0	0	0
SNRPA	0	0	0	0	0	0	0	0	0	0	0	0	0
SRRM1	0	1	0	0	0	0	0	0	0	0	0	0	0
SRSF11	0	1	0	0	0	0	0	0	0	0	0	0	0
SRSF4	0	1	0	0	0	0	0	0	0	0	0	0	0
SSRP1	0	0	0	0	0	0	0	0	0	1	0	0	0
SUGP1	0	0	0	0	0	0	0	0	0	0	0	0	0
SUPT16H	0	0	0	0	0	0	0	0	0	1	0	0	0
SUPT5H	0	0	0	0	0	0	0	0	0	1	0	0	0
SYNE2	0	0	0	0	0	0	0	0	0	0	0	0	0

NAME	REACTOME_MEIOSIS_	REACTOME_CHROMOSOME_MAINTENANCE_	REACTOME_CELL_CYCLE_MITOTIC_	REACTOME_CELL_CYCLE_	REACTOME_DNA_REPLICATION_	REACTOME_MITOTIC_M_M_G1_PHASES_	REACTOME_SYNTHESIS_OF_DNA_	REACTOME_S_PHASE_	REACTOME_CELL_CYCLE_CHECKPOINTS_	REACTOME_G1_S_TRANSITION_	REACTOME_MITOTIC_G1_G1_S_PHASES_	REACTOME_FACTORS_INVOLVED_IN_MEGAKARYOCYTE_DEVELOPMENT_AND_PLATELET_PRODUCTION	KEGG_SPLICEOSOME_
RNMT	0	0	0	0	0	0	0	0	0	0	0	0	0
RNPS1	0	0	0	0	0	0	0	0	0	0	0	0	0
SAMM50	0	0	0	0	0	0	0	0	0	0	0	0	0
SEH1L	0	0	0	1	1	1	0	0	0	0	0	0	0
SIN3A	0	0	0	0	0	0	0	0	0	0	0	1	0
SLC12A7	0	0	0	0	0	0	0	0	0	0	0	0	0
SLC25A13	0	0	0	0	0	0	0	0	0	0	0	0	0
SLC25A4	0	0	0	0	0	0	0	0	0	0	0	0	0
SLC25A5	0	0	0	0	0	0	0	0	0	0	0	0	0
SLC2A1	0	0	0	0	0	0	0	0	0	0	0	0	0
SLC2A3	0	0	0	0	0	0	0	0	0	0	0	0	0
SLC3A2	0	0	0	0	0	0	0	0	0	0	0	0	0
SLC4A7	0	0	0	0	0	0	0	0	0	0	0	0	0
SLC7A3	0	0	0	0	0	0	0	0	0	0	0	0	0
SLC7A5	0	0	0	0	0	0	0	0	0	0	0	0	0
SMARCA5	0	1	0	1	0	0	0	0	0	0	0	0	0
SMN2	0	0	0	0	0	0	0	0	0	0	0	0	0
SNRPA	0	0	0	0	0	0	0	0	0	0	0	0	0
SRRM1	0	0	0	0	0	0	0	0	0	0	0	0	0
SRSF11	0	0	0	0	0	0	0	0	0	0	0	0	0
SRSF4	0	0	0	0	0	0	0	0	0	0	0	0	0
SSRP1	0	0	0	0	0	0	0	0	0	0	0	0	0
SUGP1	0	0	0	0	0	0	0	0	0	0	0	0	0
SUPT16H	0	0	0	0	0	0	0	0	0	0	0	0	0
SUPT5H	0	0	0	0	0	0	0	0	0	0	0	0	0
SYNE2	1	1	0	1	0	0	0	0	0	0	0	0	0

NAME	KEGG_RNA_DEGRADATION_	KEGG_DNA_REPLICATION_	KEGG_CELL_CYCLE_	KEGG_OOCYTE_MEIOSIS_	KEGG_PYRIMIDINE_METABOLISM_	KEGG_VALINE_LEUCINE_AND_ISOLEUCINE_DEGRADATION_
RNMT	0	0	0	0	0	0
RNPS1	0	0	0	0	0	0
SAMM50	0	0	0	0	0	0
SEH1L	0	0	0	0	0	0
SIN3A	0	0	0	0	0	0
SLC12A7	0	0	0	0	0	0
SLC25A13	0	0	0	0	0	0
SLC25A4	0	0	0	0	0	0
SLC25A5	0	0	0	0	0	0
SLC2A1	0	0	0	0	0	0
SLC2A3	0	0	0	0	0	0
SLC3A2	0	0	0	0	0	0
SLC4A7	0	0	0	0	0	0
SLC7A3	0	0	0	0	0	0
SLC7A5	0	0	0	0	0	0
SMARCA5	0	0	0	0	0	0
SMN2	0	0	0	0	0	0
SNRPA	0	0	0	0	0	0
SRRM1	0	0	0	0	0	0
SRSF11	0	0	0	0	0	0
SRSF4	0	0	0	0	0	0
SSRP1	0	0	0	0	0	0
SUGP1	0	0	0	0	0	0
SUPT16H	0	0	0	0	0	0
SUPT5H	0	0	0	0	0	0
SYNE2	0	0	0	0	0	0

Table S6 The differential proteins contribute to the enrichment of the significant pathways for the public dataset

Core enrichment proteins in up-regulated pathways of hiPSCs/HFs (hiPSCs vs HFs)

NAME	REACTOME_MITOCHONDRIAL_PROTEIN_IMPORT_	REACTOME_DEADENYLATION_DEPENDENT_MRNA_DECAY_	REACTOME_RNA_POL_I_RNA_POL_III_AND_MITOCHONDRIAL_TRANSCRIPTION_	REACTOME_PROCESSING_OF_CAPPED_INTRON_CONTAINING_PRE_MRNA_	REACTOME_MRNA_PROCESSING_	REACTOME_MRNA_SPLICING_	REACTOME_MRNA_SPLICING_MINOR_PATHWAY_	REACTOME_TRANSCRIPTION_	REACTOME_RNA_POL_II_TRANSCRIPTION_	REACTOME_CLEAVAGE_OF_GROWING_TRANSCRIPT_IN_THE_TERMINATION_REGION__	REACTOME_MRNA_3_END_PROCESSING_	REACTOME_HIV_LIFE_CYCLE_	REACTOME_LATE_PHASE_OF_HIV_LIFE_CYCLE_
TAF10	0	0	0	0	0	0	0	1	1	0	0	1	1
TAF4	0	0	0	0	0	0	0	1	1	0	0	1	1
TAF5	0	0	0	0	0	0	0	1	1	0	0	1	1
TBP	0	0	1	0	0	0	0	1	1	0	0	1	1
TCEA1	0	0	0	0	0	0	0	1	1	0	0	1	1
TFAM	0	0	1	0	0	0	0	1	0	0	0	0	0
TIMM10	1	0	0	0	0	0	0	0	0	0	0	0	0
TIMM13	1	0	0	0	0	0	0	0	0	0	0	0	0
TIMM44	1	0	0	0	0	0	0	0	0	0	0	0	0
TIMM50	1	0	0	0	0	0	0	0	0	0	0	0	0
TIMM8A	1	0	0	0	0	0	0	0	0	0	0	0	0
TIMM9	1	0	0	0	0	0	0	0	0	0	0	0	0
TOMM22	1	0	0	0	0	0	0	0	0	0	0	0	0
TOMM40	1	0	0	0	0	0	0	0	0	0	0	0	0
TPR	0	0	0	1	1	0	0	0	0	0	0	1	1
U2AF1	0	0	0	0	0	0	0	1	1	1	1	0	0
U2AF2	0	0	0	1	1	1	0	1	1	1	1	0	0
UBTF	0	0	1	0	0	0	0	1	0	0	0	0	0
XPC	0	0	0	0	0	0	0	0	0	0	0	0	0
XPO1	0	0	0	0	0	0	0	0	0	0	0	0	1
XRCC1	0	0	0	0	0	0	0	0	0	0	0	0	0
XRCC5	0	0	0	0	0	0	0	0	0	0	0	1	0
XRCC6	0	0	0	0	0	0	0	0	0	0	0	1	0
ACAA2	0	0	0	0	0	0	0	0	0	0	0	0	0
ACAT2	0	0	0	0	0	0	0	0	0	0	0	0	0
ACIN1	0	0	0	0	0	0	0	0	0	0	0	0	0

NAME	REACTOME_METABOLISM_OF_NON_CODING_RNA_	REACTOME_TRANSPORT_OF_MATURE_TRANSCRIPT_TO_CYTOPLASM_	REACTOME_TRANSPORT_OF_MATURE_MRNA_DERIVED_FROM_AN_INTRONLESS_TRANSCRIPT_	REACTOME_INTERACTIONS_OF_VPR_WITH_HOST_CELLULAR_PROTEINS_	REACTOME_SLC_MEDIATED_TRANSMEMBRANE_TRANSPORT_	REACTOME_GLUCOSE_TRANSPORT_	REACTOME_NEP_NS2_INTERACTS_WITH_THE_CELLULAR_EXPORT_MACHINERY_	REACTOME_REGULATION_OF_GLUCOKINASE_BY_GLUCOKINASE_REGULATORY_PROTEIN_	REACTOME_TRANSPORT_OF_RIBONUCLEOPROTEINS_INTO_THE_HOST_NUCLEUS_	REACTOME_RNA_POL_II_PRE_TRANSCRIPTION_EVENTS_	REACTOME_DNA_REPAIR_	REACTOME_NUCLEOTIDE_EXCISION_REPAIR_	REACTOME_MITOTIC_PROMETAPHASE_
TAF10	0	0	0	0	0	0	0	0	0	1	0	0	0
TAF4	0	0	0	0	0	0	0	0	0	1	0	0	0
TAF5	0	0	0	0	0	0	0	0	0	1	0	0	0
TBP	0	0	0	0	0	0	0	0	0	1	0	0	0
TCEA1	0	0	0	0	0	0	0	0	0	1	1	1	0
TFAM	0	0	0	0	0	0	0	0	0	0	0	0	0
TIMM10	0	0	0	0	0	0	0	0	0	0	0	0	0
TIMM13	0	0	0	0	0	0	0	0	0	0	0	0	0
TIMM44	0	0	0	0	0	0	0	0	0	0	0	0	0
TIMM50	0	0	0	0	0	0	0	0	0	0	0	0	0
TIMM8A	0	0	0	0	0	0	0	0	0	0	0	0	0
TIMM9	0	0	0	0	0	0	0	0	0	0	0	0	0
TOMM22	0	0	0	0	0	0	0	0	0	0	0	0	0
TOMM40	0	0	0	0	0	0	0	0	0	0	0	0	0
TPR	1	1	1	1	1	1	1	1	1	0	0	0	0
U2AF1	0	1	0	0	0	0	0	0	0	0	0	0	0
U2AF2	0	1	0	0	0	0	0	0	0	0	0	0	0
UBTF	0	0	0	0	0	0	0	0	0	0	0	0	0
XPC	0	0	0	0	0	0	0	0	0	0	1	1	0
XPO1	0	0	0	0	0	0	1	0	0	0	0	0	0
XRCC1	0	0	0	0	0	0	0	0	0	0	1	0	0
XRCC5	0	0	0	0	0	0	0	0	0	0	1	0	0
XRCC6	0	0	0	0	0	0	0	0	0	0	1	0	0
ACAA2	0	0	0	0	0	0	0	0	0	0	0	0	0
ACAT2	0	0	0	0	0	0	0	0	0	0	0	0	0
ACIN1	0	0	0	0	0	0	0	0	0	0	0	0	0

NAME	REACTOME_MEIOSIS_	REACTOME_CHROMOSOME_MAINTENANCE_	REACTOME_CELL_CYCLE_MITOTIC_	REACTOME_CELL_CYCLE_	REACTOME_DNA_REPLICATION_	REACTOME_MITOTIC_M_M_G1_PHASES_	REACTOME_SYNTHESIS_OF_DNA_	REACTOME_S_PHASE_	REACTOME_CELL_CYCLE_CHECKPOINTS_	REACTOME_G1_S_TRANSITION_	REACTOME_MITOTIC_G1_G1_S_PHASES_	REACTOME_FACTORS_INVOLVED_IN_MEGAKARYOCYTE_DEVELOPMENT_AND_PLATELET_PRODUCTION	KEGG_SPLICEOSOME_
TAF10	0	0	0	0	0	0	0	0	0	0	0	0	0
TAF4	0	0	0	0	0	0	0	0	0	0	0	0	0
TAF5	0	0	0	0	0	0	0	0	0	0	0	0	0
TBP	0	0	0	0	0	0	0	0	0	0	0	0	0
TCEA1	0	0	0	0	0	0	0	0	0	0	0	0	0
TFAM	0	0	0	0	0	0	0	0	0	0	0	0	0
TIMM10	0	0	0	0	0	0	0	0	0	0	0	0	0
TIMM13	0	0	0	0	0	0	0	0	0	0	0	0	0
TIMM44	0	0	0	0	0	0	0	0	0	0	0	0	0
TIMM50	0	0	0	0	0	0	0	0	0	0	0	0	0
TIMM8A	0	0	0	0	0	0	0	0	0	0	0	0	0
TIMM9	0	0	0	0	0	0	0	0	0	0	0	0	0
TOMM22	0	0	0	0	0	0	0	0	0	0	0	0	0
TOMM40	0	0	0	0	0	0	0	0	0	0	0	0	0
TPR	0	0	0	0	0	0	0	0	0	0	0	0	0
U2AF1	0	0	0	0	0	0	0	0	0	0	0	0	0
U2AF2	0	0	0	0	0	0	0	0	0	0	0	0	0
UBTF	0	0	0	0	0	0	0	0	0	0	0	0	0
XPC	0	0	0	0	0	0	0	0	0	0	0	0	0
XPO1	0	0	0	0	0	0	0	0	0	0	0	0	0
XRCC1	0	0	0	0	0	0	0	0	0	0	0	0	0
XRCC5	0	0	0	0	0	0	0	0	0	0	0	0	0
XRCC6	0	0	0	0	0	0	0	0	0	0	0	0	0
ACAA2	0	0	0	0	0	0	0	0	0	0	0	0	0
ACAT2	0	0	0	0	0	0	0	0	0	0	0	0	0
ACIN1	0	0	0	0	0	0	0	0	0	0	0	0	0

NAME	KEGG_RNA_DEGRADATION_	KEGG_DNA_REPLICATION_	KEGG_CELL_CYCLE_	KEGG_OOCYTE_MEIOSIS_	KEGG_PYRIMIDINE_METABOLISM_	KEGG_VALINE_LEUCINE_AND_ISOLEUCINE_DEGRADATION_
TAF10	0	0	0	0	0	0
TAF4	0	0	0	0	0	0
TAF5	0	0	0	0	0	0
TBP	0	0	0	0	0	0
TCEA1	0	0	0	0	0	0
TFAM	0	0	0	0	0	0
TIMM10	0	0	0	0	0	0
TIMM13	0	0	0	0	0	0
TIMM44	0	0	0	0	0	0
TIMM50	0	0	0	0	0	0
TIMM8A	0	0	0	0	0	0
TIMM9	0	0	0	0	0	0
TOMM22	0	0	0	0	0	0
TOMM40	0	0	0	0	0	0
TPR	0	0	0	0	0	0
U2AF1	0	0	0	0	0	0
U2AF2	0	0	0	0	0	0
UBTF	0	0	0	0	0	0
XPC	0	0	0	0	0	0
XPO1	0	0	0	0	0	0
XRCC1	0	0	0	0	0	0
XRCC5	0	0	0	0	0	0
XRCC6	0	0	0	0	0	0
ACAA2	0	0	0	0	0	0
ACAT2	0	0	0	0	0	0
ACIN1	0	0	0	0	0	0

Table S6 The differential proteins contribute to the enrichment of the significant pathways for the public dataset

Core enrichment proteins in up-regulated pathways of hiPSCs/HFs (hiPSCs vs HFs)

NAME	REACTOME_MITOCHONDRIAL_PROTEIN_IMPORT_	REACTOME_DEADENYLATION_DEPENDENT_MRNA_DECAY_	REACTOME_RNA_POL_I_RNA_POL_III_AND_MITOCHONDRIAL_TRANSCRIPTION_	REACTOME_PROCESSING_OF_CAPPED_INTRON_CONTAINING_PRE_MRNA_	REACTOME_MRNA_PROCESSING_	REACTOME_MRNA_SPLICING_	REACTOME_MRNA_SPLICING_MINOR_PATHWAY_	REACTOME_TRANSCRIPTION_	REACTOME_RNA_POL_II_TRANSCRIPTION_	REACTOME_CLEAVAGE_OF_GROWING_TRANSCRIPT_IN_THE_TERMINATION_REGION__	REACTOME_MRNA_3_END_PROCESSING_	REACTOME_HIV_LIFE_CYCLE_	REACTOME_LATE_PHASE_OF_HIV_LIFE_CYCLE_
ALDH1B1	0	0	0	0	0	0	0	0	0	0	0	0	0
ALDH6A1	0	0	0	0	0	0	0	0	0	0	0	0	0
ALDH7A1	0	0	0	0	0	0	0	0	0	0	0	0	0
AQR	0	0	0	0	0	0	0	0	0	0	0	0	0
AUH	0	0	0	0	0	0	0	0	0	0	0	0	0
BCAS2	0	0	0	0	0	0	0	0	0	0	0	0	0
CRNKL1	0	0	0	0	0	0	0	0	0	0	0	0	0
CTNNBL1	0	0	0	0	0	0	0	0	0	0	0	0	0
CTPS2	0	0	0	0	0	0	0	0	0	0	0	0	0
CWC15	0	0	0	0	0	0	0	0	0	0	0	0	0
DBT	0	0	0	0	0	0	0	0	0	0	0	0	0
DDX42	0	0	0	0	0	0	0	0	0	0	0	0	0
DDX5	0	0	0	0	0	0	0	0	0	0	0	0	0
DHX15	0	0	0	0	0	0	0	0	0	0	0	0	0
DHX8	0	0	0	0	0	0	0	0	0	0	0	0	0
DUT	0	0	0	0	0	0	0	0	0	0	0	0	0
EXOSC10	0	0	0	0	0	0	0	0	0	0	0	0	0
HADH	0	0	0	0	0	0	0	0	0	0	0	0	0
HIBCH	0	0	0	0	0	0	0	0	0	0	0	0	0
HMGCS1	0	0	0	0	0	0	0	0	0	0	0	0	0
IGF1R	0	0	0	0	0	0	0	0	0	0	0	0	0
ISY1	0	0	0	0	0	0	0	0	0	0	0	0	0
LSM7	0	0	0	0	0	0	0	0	0	0	0	0	0
MCCC2	0	0	0	0	0	0	0	0	0	0	0	0	0
NME3	0	0	0	0	0	0	0	0	0	0	0	0	0
PNP	0	0	0	0	0	0	0	0	0	0	0	0	0

NAME	REACTOME_METABOLISM_OF_NON_CODING_RNA_	REACTOME_TRANSPORT_OF_MATURE_TRANSCRIPT_TO_CYTOPLASM_	REACTOME_TRANSPORT_OF_MATURE_MRNA_DERIVED_FROM_AN_INTRONLESS_TRANSCRIPT_	REACTOME_INTERACTIONS_OF_VPR_WITH_HOST_CELLULAR_PROTEINS_	REACTOME_SLC_MEDIATED_TRANSMEMBRANE_TRANSPORT_	REACTOME_GLUCOSE_TRANSPORT_	REACTOME_NEP_NS2_INTERACTS_WITH_THE_CELLULAR_EXPORT_MACHINERY_	REACTOME_REGULATION_OF_GLUCOKINASE_BY_GLUCOKINASE_REGULATORY_PROTEIN_	REACTOME_TRANSPORT_OF_RIBONUCLEOPROTEINS_INTO_THE_HOST_NUCLEUS_	REACTOME_RNA_POL_II_PRE_TRANSCRIPTION_EVENTS_	REACTOME_DNA_REPAIR_	REACTOME_NUCLEOTIDE_EXCISION_REPAIR_	REACTOME_MITOTIC_PROMETAPHASE_
ALDH1B1	0	0	0	0	0	0	0	0	0	0	0	0	0
ALDH6A1	0	0	0	0	0	0	0	0	0	0	0	0	0
ALDH7A1	0	0	0	0	0	0	0	0	0	0	0	0	0
AQR	0	0	0	0	0	0	0	0	0	0	0	0	0
AUH	0	0	0	0	0	0	0	0	0	0	0	0	0
BCAS2	0	0	0	0	0	0	0	0	0	0	0	0	0
CRNKL1	0	0	0	0	0	0	0	0	0	0	0	0	0
CTNNBL1	0	0	0	0	0	0	0	0	0	0	0	0	0
CTPS2	0	0	0	0	0	0	0	0	0	0	0	0	0
CWC15	0	0	0	0	0	0	0	0	0	0	0	0	0
DBT	0	0	0	0	0	0	0	0	0	0	0	0	0
DDX42	0	0	0	0	0	0	0	0	0	0	0	0	0
DDX5	0	0	0	0	0	0	0	0	0	0	0	0	0
DHX15	0	0	0	0	0	0	0	0	0	0	0	0	0
DHX8	0	0	0	0	0	0	0	0	0	0	0	0	0
DUT	0	0	0	0	0	0	0	0	0	0	0	0	0
EXOSC10	0	0	0	0	0	0	0	0	0	0	0	0	0
HADH	0	0	0	0	0	0	0	0	0	0	0	0	0
HIBCH	0	0	0	0	0	0	0	0	0	0	0	0	0
HMGCS1	0	0	0	0	0	0	0	0	0	0	0	0	0
IGF1R	0	0	0	0	0	0	0	0	0	0	0	0	0
ISY1	0	0	0	0	0	0	0	0	0	0	0	0	0
LSM7	0	0	0	0	0	0	0	0	0	0	0	0	0
MCCC2	0	0	0	0	0	0	0	0	0	0	0	0	0
NME3	0	0	0	0	0	0	0	0	0	0	0	0	0
PNP	0	0	0	0	0	0	0	0	0	0	0	0	0

NAME	REACTOME_MEIOSIS_	REACTOME_CHROMOSOME_MAINTENANCE_	REACTOME_CELL_CYCLE_MITOTIC_	REACTOME_CELL_CYCLE_	REACTOME_DNA_REPLICATION_	REACTOME_MITOTIC_M_M_G1_PHASES_	REACTOME_SYNTHESIS_OF_DNA_	REACTOME_S_PHASE_	REACTOME_CELL_CYCLE_CHECKPOINTS_	REACTOME_G1_S_TRANSITION_	REACTOME_MITOTIC_G1_G1_S_PHASES_	REACTOME_FACTORS_INVOLVED_IN_MEGAKARYOCYTE_DEVELOPMENT_AND_PLATELET_PRODUCTION	KEGG_SPLICEOSOME_
ALDH1B1	0	0	0	0	0	0	0	0	0	0	0	0	0
ALDH6A1	0	0	0	0	0	0	0	0	0	0	0	0	0
ALDH7A1	0	0	0	0	0	0	0	0	0	0	0	0	0
AQR	0	0	0	0	0	0	0	0	0	0	0	0	0
AUH	0	0	0	0	0	0	0	0	0	0	0	0	0
BCAS2	0	0	0	0	0	0	0	0	0	0	0	0	0
CRNKL1	0	0	0	0	0	0	0	0	0	0	0	0	0
CTNNBL1	0	0	0	0	0	0	0	0	0	0	0	0	0
CTPS2	0	0	0	0	0	0	0	0	0	0	0	0	0
CWC15	0	0	0	0	0	0	0	0	0	0	0	0	0
DBT	0	0	0	0	0	0	0	0	0	0	0	0	0
DDX42	0	0	0	0	0	0	0	0	0	0	0	0	0
DDX5	0	0	0	0	0	0	0	0	0	0	0	0	0
DHX15	0	0	0	0	0	0	0	0	0	0	0	0	0
DHX8	0	0	0	0	0	0	0	0	0	0	0	0	0
DUT	0	0	0	0	0	0	0	0	0	0	0	0	0
EXOSC10	0	0	0	0	0	0	0	0	0	0	0	0	0
HADH	0	0	0	0	0	0	0	0	0	0	0	0	0
HIBCH	0	0	0	0	0	0	0	0	0	0	0	0	0
HMGCS1	0	0	0	0	0	0	0	0	0	0	0	0	0
IGF1R	0	0	0	0	0	0	0	0	0	0	0	0	0
ISY1	0	0	0	0	0	0	0	0	0	0	0	0	0
LSM7	0	0	0	0	0	0	0	0	0	0	0	0	0
MCCC2	0	0	0	0	0	0	0	0	0	0	0	0	0
NME3	0	0	0	0	0	0	0	0	0	0	0	0	0
PNP	0	0	0	0	0	0	0	0	0	0	0	0	0

NAME	KEGG_RNA_DEGRADATION_	KEGG_DNA_REPLICATION_	KEGG_CELL_CYCLE_	KEGG_OOCYTE_MEIOSIS_	KEGG_PYRIMIDINE_METABOLISM_	KEGG_VALINE_LEUCINE_AND_ISOLEUCINE_DEGRADATION_
ALDH1B1	0	0	0	0	0	0
ALDH6A1	0	0	0	0	0	0
ALDH7A1	0	0	0	0	0	0
AQR	0	0	0	0	0	0
AUH	0	0	0	0	0	0
BCAS2	0	0	0	0	0	0
CRNKL1	0	0	0	0	0	0
CTNNBL1	0	0	0	0	0	0
CTPS2	0	0	0	0	0	0
CWC15	0	0	0	0	0	0
DBT	0	0	0	0	0	0
DDX42	0	0	0	0	0	0
DDX5	0	0	0	0	0	0
DHX15	0	0	0	0	0	0
DHX8	0	0	0	0	0	0
DUT	0	0	0	0	0	0
EXOSC10	0	0	0	0	0	0
HADH	0	0	0	0	0	0
HIBCH	0	0	0	0	0	0
HMGCS1	0	0	0	0	0	0
IGF1R	0	0	0	0	0	0
ISY1	0	0	0	0	0	0
LSM7	0	0	0	0	0	0
MCCC2	0	0	0	0	0	0
NME3	0	0	0	0	0	0
PNP	0	0	0	0	0	0

Table S6 The differential proteins contribute to the enrichment of the significant pathways for the public dataset

Core enrichment proteins in up-regulated pathways of hiPSCs/HFs (hiPSCs vs HFs)

NAME	REACTOME_MITOCHONDRIAL_PROTEIN_IMPORT_	REACTOME_DEADENYLATION_DEPENDENT_MRNA_DECAY_	REACTOME_RNA_POL_I_RNA_POL_III_AND_MITOCHONDRIAL_TRANSCRIPTION_	REACTOME_PROCESSING_OF_CAPPED_INTRON_CONTAINING_PRE_MRNA_	REACTOME_MRNA_PROCESSING_	REACTOME_MRNA_SPLICING_	REACTOME_MRNA_SPLICING_MINOR_PATHWAY_	REACTOME_TRANSCRIPTION_	REACTOME_RNA_POL_II_TRANSCRIPTION_	REACTOME_CLEAVAGE_OF_GROWING_TRANSCRIPT_IN_THE_TERMINATION_REGION__	REACTOME_MRNA_3_END_PROCESSING_	REACTOME_HIV_LIFE_CYCLE_	REACTOME_LATE_PHASE_OF_HIV_LIFE_CYCLE_
PNPT1	0	0	0	0	0	0	0	0	0	0	0	0	0
POLR1E	0	0	0	0	0	0	0	0	0	0	0	0	0
PPIH	0	0	0	0	0	0	0	0	0	0	0	0	0
PPIL1	0	0	0	0	0	0	0	0	0	0	0	0	0
PQBP1	0	0	0	0	0	0	0	0	0	0	0	0	0
PRPF19	0	0	0	0	0	0	0	0	0	0	0	0	0
PRPF3	0	0	0	0	0	0	0	0	0	0	0	0	0
PRPF38A	0	0	0	0	0	0	0	0	0	0	0	0	0
RBM17	0	0	0	0	0	0	0	0	0	0	0	0	0
RBM25	0	0	0	0	0	0	0	0	0	0	0	0	0
RFC1	0	0	0	0	0	0	0	0	0	0	0	0	0
RNASEH2A	0	0	0	0	0	0	0	0	0	0	0	0	0
SART1	0	0	0	0	0	0	0	0	0	0	0	0	0
SKIV2L2	0	0	0	0	0	0	0	0	0	0	0	0	0
SMNDC1	0	0	0	0	0	0	0	0	0	0	0	0	0
SNW1	0	0	0	0	0	0	0	0	0	0	0	0	0
SRSF10	0	0	0	0	0	0	0	0	0	0	0	0	0
SRSF8	0	0	0	0	0	0	0	0	0	0	0	0	0
TCERG1	0	0	0	0	0	0	0	0	0	0	0	0	0
THOC1	0	0	0	0	0	0	0	0	0	0	0	0	0
THOC3	0	0	0	0	0	0	0	0	0	0	0	0	0
TRA2A	0	0	0	0	0	0	0	0	0	0	0	0	0
TRA2B	0	0	0	0	0	0	0	0	0	0	0	0	0
U2SURP	0	0	0	0	0	0	0	0	0	0	0	0	0
WBP11	0	0	0	0	0	0	0	0	0	0	0	0	0

NAME	REACTOME_METABOLISM_OF_NON_CODING_RNA_	REACTOME_TRANSPORT_OF_MATURE_TRANSCRIPT_TO_CYTOPLASM_	REACTOME_TRANSPORT_OF_MATURE_MRNA_DERIVED_FROM_AN_INTRONLESS_TRANSCRIPT_	REACTOME_INTERACTIONS_OF_VPR_WITH_HOST_CELLULAR_PROTEINS_	REACTOME_SLC_MEDIATED_TRANSMEMBRANE_TRANSPORT_	REACTOME_GLUCOSE_TRANSPORT_	REACTOME_NEP_NS2_INTERACTS_WITH_THE_CELLULAR_EXPORT_MACHINERY_	REACTOME_REGULATION_OF_GLUCOKINASE_BY_GLUCOKINASE_REGULATORY_PROTEIN_	REACTOME_TRANSPORT_OF_RIBONUCLEOPROTEINS_INTO_THE_HOST_NUCLEUS_	REACTOME_RNA_POL_II_PRE_TRANSCRIPTION_EVENTS_	REACTOME_DNA_REPAIR_	REACTOME_NUCLEOTIDE_EXCISION_REPAIR_	REACTOME_MITOTIC_PROMETAPHASE_
PNPT1	0	0	0	0	0	0	0	0	0	0	0	0	0
POLR1E	0	0	0	0	0	0	0	0	0	0	0	0	0
PPIH	0	0	0	0	0	0	0	0	0	0	0	0	0
PPIL1	0	0	0	0	0	0	0	0	0	0	0	0	0
PQBP1	0	0	0	0	0	0	0	0	0	0	0	0	0
PRPF19	0	0	0	0	0	0	0	0	0	0	0	0	0
PRPF3	0	0	0	0	0	0	0	0	0	0	0	0	0
PRPF38A	0	0	0	0	0	0	0	0	0	0	0	0	0
RBM17	0	0	0	0	0	0	0	0	0	0	0	0	0
RBM25	0	0	0	0	0	0	0	0	0	0	0	0	0
RFC1	0	0	0	0	0	0	0	0	0	0	0	0	0
RNASEH2A	0	0	0	0	0	0	0	0	0	0	0	0	0
SART1	0	0	0	0	0	0	0	0	0	0	0	0	0
SKIV2L2	0	0	0	0	0	0	0	0	0	0	0	0	0
SMNDC1	0	0	0	0	0	0	0	0	0	0	0	0	0
SNW1	0	0	0	0	0	0	0	0	0	0	0	0	0
SRSF10	0	0	0	0	0	0	0	0	0	0	0	0	0
SRSF8	0	0	0	0	0	0	0	0	0	0	0	0	0
TCERG1	0	0	0	0	0	0	0	0	0	0	0	0	0
THOC1	0	0	0	0	0	0	0	0	0	0	0	0	0
THOC3	0	0	0	0	0	0	0	0	0	0	0	0	0
TRA2A	0	0	0	0	0	0	0	0	0	0	0	0	0
TRA2B	0	0	0	0	0	0	0	0	0	0	0	0	0
U2SURP	0	0	0	0	0	0	0	0	0	0	0	0	0
WBP11	0	0	0	0	0	0	0	0	0	0	0	0	0

NAME	REACTOME_MEIOSIS_	REACTOME_CHROMOSOME_MAINTENANCE_	REACTOME_CELL_CYCLE_MITOTIC_	REACTOME_CELL_CYCLE_	REACTOME_DNA_REPLICATION_	REACTOME_MITOTIC_M_M_G1_PHASES_	REACTOME_SYNTHESIS_OF_DNA_	REACTOME_S_PHASE_	REACTOME_CELL_CYCLE_CHECKPOINTS_	REACTOME_G1_S_TRANSITION_	REACTOME_MITOTIC_G1_G1_S_PHASES_	REACTOME_FACTORS_INVOLVED_IN_MEGAKARYOCYTE_DEVELOPMENT_AND_PLATELET_PRODUCTION	KEGG_SPLICEOSOME_
PNPT1	0	0	0	0	0	0	0	0	0	0	0	0	0
POLR1E	0	0	0	0	0	0	0	0	0	0	0	0	0
PPIH	0	0	0	0	0	0	0	0	0	0	0	0	0
PPIL1	0	0	0	0	0	0	0	0	0	0	0	0	0
PQBP1	0	0	0	0	0	0	0	0	0	0	0	0	0
PRPF19	0	0	0	0	0	0	0	0	0	0	0	0	0
PRPF3	0	0	0	0	0	0	0	0	0	0	0	0	0
PRPF38A	0	0	0	0	0	0	0	0	0	0	0	0	0
RBM17	0	0	0	0	0	0	0	0	0	0	0	0	0
RBM25	0	0	0	0	0	0	0	0	0	0	0	0	0
RFC1	0	0	0	0	0	0	0	0	0	0	0	0	0
RNASEH2A	0	0	0	0	0	0	0	0	0	0	0	0	0
SART1	0	0	0	0	0	0	0	0	0	0	0	0	0
SKIV2L2	0	0	0	0	0	0	0	0	0	0	0	0	0
SMNDC1	0	0	0	0	0	0	0	0	0	0	0	0	0
SNW1	0	0	0	0	0	0	0	0	0	0	0	0	0
SRSF10	0	0	0	0	0	0	0	0	0	0	0	0	0
SRSF8	0	0	0	0	0	0	0	0	0	0	0	0	0
TCERG1	0	0	0	0	0	0	0	0	0	0	0	0	0
THOC1	0	0	0	0	0	0	0	0	0	0	0	0	0
THOC3	0	0	0	0	0	0	0	0	0	0	0	0	0
TRA2A	0	0	0	0	0	0	0	0	0	0	0	0	0
TRA2B	0	0	0	0	0	0	0	0	0	0	0	0	0
U2SURP	0	0	0	0	0	0	0	0	0	0	0	0	0
WBP11	0	0	0	0	0	0	0	0	0	0	0	0	0

NAME	KEGG_RNA_DEGRADATION_	KEGG_DNA_REPLICATION_	KEGG_CELL_CYCLE_	KEGG_OOCYTE_MEIOSIS_	KEGG_PYRIMIDINE_METABOLISM_	KEGG_VALINE_LEUCINE_AND_ISOLEUCINE_DEGRADATION_
PNPT1	0	0	0	0	0	0
POLR1E	0	0	0	0	0	0
PPIH	0	0	0	0	0	0
PPIL1	0	0	0	0	0	0
PQBP1	0	0	0	0	0	0
PRPF19	0	0	0	0	0	0
PRPF3	0	0	0	0	0	0
PRPF38A	0	0	0	0	0	0
RBM17	0	0	0	0	0	0
RBM25	0	0	0	0	0	0
RFC1	0	0	0	0	0	0
RNASEH2A	0	0	0	0	0	0
SART1	0	0	0	0	0	0
SKIV2L2	0	0	0	0	0	0
SMNDC1	0	0	0	0	0	0
SNW1	0	0	0	0	0	0
SRSF10	0	0	0	0	0	0
SRSF8	0	0	0	0	0	0
TCERG1	0	0	0	0	0	0
THOC1	0	0	0	0	0	0
THOC3	0	0	0	0	0	0
TRA2A	0	0	0	0	0	0
TRA2B	0	0	0	0	0	0
U2SURP	0	0	0	0	0	0
WBP11	0	0	0	0	0	0

Table S6 The differential proteins contribute to the enrichment of the significant pathways for the public dataset

Core enrichment proteins in down-regulated pathways of hESCs/HFs (hESCs vs HFs)

NAME	REACTOME_UNFOLDED_PROTEIN_RESPONSE	REACTOME_DIABETES_PATHWAYS	REACTOME_CELL_CELL_COMMUNICATION	REACTOME_GOLGI_ASSOCIATED_VESICLE_BIOGENESIS	REACTOME_TRANS_GOLGI_NETWORK_VESICLE_BUDDING	REACTOME_MEMBRANE_TRAFFICKING	REACTOME_NEURONAL_SYSTEM	REACTOME_TRANSMISSION_ACROSS_CHEMICAL_SYNAPSES	REACTOME_POST_TRANSLATIONAL_PROTEIN_MODIFICATION	REACTOME_ASPARAGINE_N_LINKED_GLYCOSYLATION	REACTOME_ING_BY_INSULIN_RECEPTOR	REACTOME_PREFOLDIN_MEDIATED_TRANSFER_OF_SUBSTRATE_TO_CCT_TRIC	REACTOME_PROTEIN_FOLDING
ACTB	0	0	0	0	0	0	0	0	0	0	0	1	1
ACTN2	0	0	1	0	0	0	1	1	0	0	0	0	0
ACTN4	0	0	1	0	0	0	0	0	0	0	0	0	0
AP1M1	0	0	0	1	1	1	0	0	0	0	0	0	0
AP2A1	0	0	0	0	0	0	1	1	0	0	0	0	0
AP2A2	0	0	0	0	0	0	0	0	0	0	0	0	0
AP2M1	0	0	0	0	0	1	1	1	0	0	0	0	0
AP3B1	0	0	0	1	1	1	0	0	0	0	0	0	0
AP3S1	0	0	0	1	1	1	0	0	0	0	0	0	0
ARFGAP1	1	1	0	0	0	1	0	0	0	0	0	0	0
ARHGAP35	0	0	0	0	0	0	0	0	0	0	0	0	0
ARHGDIA	0	0	0	0	0	0	0	0	0	0	0	0	0
ARSB	0	0	0	0	0	0	0	0	1	0	0	0	0
ATP6V0D1	1	1	0	0	0	0	0	0	0	0	1	0	0
ATP6V1B2	0	0	0	0	0	0	0	0	0	0	1	0	0
ATP6V1D	0	0	0	0	0	0	0	0	0	0	1	0	0
ATP6V1E1	0	0	0	0	0	0	0	0	0	0	1	0	0
ATP6V1F	0	0	0	0	0	0	0	0	0	0	1	0	0
ATP6V1G1	0	0	0	0	0	0	0	0	0	0	1	0	0
BAD	0	0	0	0	0	0	0	0	0	0	0	0	0
BID	0	0	0	0	0	0	0	0	0	0	0	0	0
CDC42	0	0	0	0	0	0	0	0	0	0	0	0	0
CDH2	0	0	1	0	0	0	0	0	0	0	0	0	0
CDK4	0	0	0	0	0	0	0	0	0	0	0	0	0
CFL1	0	0	0	0	0	0	0	0	0	0	0	0	0
CHMP5	0	0	0	0	0	1	0	0	0	0	0	0	0
CHUK	0	0	0	0	0	0	0	0	0	0	0	0	0
CLTA	0	0	0	1	1	1	0	0	0	0	0	0	0
CLTB	0	0	0	0	0	1	0	0	0	0	0	0	0
CLTC	0	0	0	1	1	1	0	0	0	0	0	0	0
COL1A1	0	0	0	0	0	0	0	0	0	0	0	0	0
COL1A2	0	0	0	0	0	0	0	0	0	0	0	0	0
COL5A1	0	0	0	0	0	0	0	0	0	0	0	0	0
COL6A1	0	0	0	0	0	0	0	0	0	0	0	0	0
COL6A2	0	0	0	0	0	0	0	0	0	0	0	0	0
CRK	0	0	0	0	0	0	0	0	0	0	1	0	0
CTSA	0	0	0	0	0	0	0	0	0	0	0	0	0
CTSB	0	0	0	0	0	0	0	0	0	0	0	0	0
CTSL1	0	0	0	0	0	0	0	0	0	0	0	0	0
CTSZ	0	0	0	0	1	1	0	0	0	0	0	0	0
EEA1	0	0	0	0	0	0	0	0	0	0	0	0	0
EGFR	0	0	0	0	0	0	0	0	0	0	0	0	0
EHD1	0	0	0	0	0	0	0	0	0	0	0	0	0
EHD2	0	0	0	0	0	0	0	0	0	0	0	0	0
EPN1	0	0	0	0	0	0	0	0	0	0	0	0	0
FZD7	0	0	0	0	0	0	0	0	0	0	0	0	0
GNG12	0	0	0	0	0	0	1	1	0	0	0	0	0
GNS	0	0	0	0	1	1	0	0	0	0	0	0	0
HGS	0	0	0	0	0	1	0	0	0	0	0	0	0
HSPB1	0	0	0	0	0	0	0	0	0	0	0	0	0
IGF2R	0	0	0	1	1	1	0	0	0	0	0	0	0
ILK	0	0	1	0	0	0	0	0	0	0	0	0	0

NAME	REACTOME_INNATE_IMMUNE_SYSTEM	REACTOME_MHC_CLASS_II_ANTIGEN_PRESENTATION	REACTOME_IMMUNE_SYSTEM	REACTOME_ADAPTIVE_IMMUNE_SYSTEM	REACTOME_APOPTOSIS	REACTOME_CLASS_I_MHC_MEDIATED_ANTIGEN_PROCESSING_PRESENTATION	REACTOME_ANTIGEN_PROCESSING_UBIQUITINATION_PROTEASOME_DEGRADATION	REACTOME_ER_PHAGOSOME_PATHWAY	REACTOME_ANTIGEN_PROCESSING_CROSS_PRESENTATION	REACTOME_REGULATION_OF_MRNA_STABILITY_BY_PROTEINS_THAT_BIND_AU_RICH_ELEMENTS	REACTOME_DESTABILIZATION_OF_MRNA_BY_AUF1_HNRNP_D0	REACTOME_REGULATION_OF_ORNITHINE_DECARBOXYLASE_ODC	REACTOME_REGULATION_OF_MITOTIC_CELL_CYCLE
ACTB	0	0	0	0	0	0	0	0	0	0	0	0	0
ACTN2	0	0	0	0	0	0	0	0	0	0	0	0	0
ACTN4	0	0	0	0	0	0	0	0	0	0	0	0	0
AP1M1	0	1	1	1	0	0	0	0	0	0	0	0	0
AP2A1	0	1	1	1	0	0	0	0	0	0	0	0	0
AP2A2	0	0	1	1	0	0	0	0	0	0	0	0	0
AP2M1	0	1	1	1	0	0	0	0	0	0	0	0	0
AP3B1	0	0	0	0	0	0	0	0	0	0	0	0	0
AP3S1	0	0	0	0	0	0	0	0	0	0	0	0	0
ARFGAP1	0	0	0	0	0	0	0	0	0	0	0	0	0
ARHGAP35	0	0	0	0	0	0	0	0	0	0	0	0	0
ARHGDIA	0	0	0	0	0	0	0	0	0	0	0	0	0
ARSB	0	0	0	0	0	0	0	0	0	0	0	0	0
ATP6V0D1	0	0	0	0	0	0	0	0	0	0	0	0	0
ATP6V1B2	0	0	0	0	0	0	0	0	0	0	0	0	0
ATP6V1D	0	0	0	0	0	0	0	0	0	0	0	0	0
ATP6V1E1	0	0	0	0	0	0	0	0	0	0	0	0	0
ATP6V1F	0	0	0	0	0	0	0	0	0	0	0	0	0
ATP6V1G1	0	0	0	0	0	0	0	0	0	0	0	0	0
BAD	0	0	1	1	1	0	0	0	0	0	0	0	0
BID	0	0	0	0	1	0	0	0	0	0	0	0	0
CDC42	0	0	1	1	0	0	0	0	0	0	0	0	0
CDH2	0	0	0	0	0	0	0	0	0	0	0	0	0
CDK4	0	0	0	0	0	0	0	0	0	0	0	0	0
CFL1	0	0	0	0	0	0	0	0	0	0	0	0	0
CHMP5	0	0	0	0	0	0	0	0	0	0	0	0	0
CHUK	1	0	1	1	0	0	0	0	0	0	0	0	0
CLTA	0	0	1	1	0	0	0	0	0	0	0	0	0
CLTB	0	0	0	0	0	0	0	0	0	0	0	0	0
CLTC	0	0	1	1	0	0	0	0	0	0	0	0	0
COL1A1	0	0	0	0	0	0	0	0	0	0	0	0	0
COL1A2	0	0	0	0	0	0	0	0	0	0	0	0	0
COL5A1	0	0	0	0	0	0	0	0	0	0	0	0	0
COL6A1	0	0	0	0	0	0	0	0	0	0	0	0	0
COL6A2	0	0	0	0	0	0	0	0	0	0	0	0	0
CRK	0	0	1	0	0	0	0	0	0	0	0	0	0
CTSA	0	1	1	1	0	0	0	0	0	0	0	0	0
CTSB	1	1	1	1	0	0	0	0	0	0	0	0	0
CTSL1	1	1	1	1	0	0	0	0	0	0	0	0	0
CTSZ	0	0	0	0	0	0	0	0	0	0	0	0	0
EEA1	1	0	1	0	0	0	0	0	0	0	0	0	0
EGFR	0	0	0	0	0	0	0	0	0	0	0	0	0
EHD1	0	0	0	0	0	0	0	0	0	0	0	0	0
EHD2	0	0	0	0	0	0	0	0	0	0	0	0	0
EPN1	0	0	0	0	0	0	0	0	0	0	0	0	0
FZD7	0	0	0	0	0	0	0	0	0	0	0	0	0
GNG12	0	0	0	0	0	0	0	0	0	0	0	0	0
GNS	0	0	0	0	0	0	0	0	0	0	0	0	0
HGS	0	0	0	0	0	0	0	0	0	0	0	0	0
HSPB1	0	0	0	0	0	0	0	0	0	1	1	0	0
IGF2R	0	0	0	0	0	0	0	0	0	0	0	0	0
ILK	0	0	0	0	0	0	0	0	0	0	0	0	0

NAME	REACTOME_CDT1_ASSOCIATION_WITH_THE_CDC6_ORC_ORIGIN_COMPLEX	REACTOME_APC_C_CDC20_MEDIATED_DEGRADATION_OF_MITOTIC_PROTEINS	REACTOME_APC_C_CDH1_MEDIATED_DEGRADATION_OF_CDC20_AND_OTHER_APC_C_CDH1_TARGETED_PROTEINS_IN_LATE_MITOSIS_EARLY_G1	REACTOME_CYCLIN_E_ASSOCIATED_EVENTS_DURING_G1_S_TRANSITION_	REACTOME_SCFSKP2_MEDIATED_DEGRADATION_OF_P27_P21	REACTOME_AUTODEGRADATION_OF_CDH1_BY_CDH1_APC_C	REACTOME_SCF_BETA_TRCP_MEDIATED_DEGRADATION_OF_EMI1	REACTOME_P53_DEPENDENT_G1_DNA_DAMAGE_RESPONSE	REACTOME_CDK_MEDIATED_PHOSPHORYLATION_AND_REMOVAL_OF_CDC6	REACTOME_AUTODEGRADATION_OF_THE_E3_UBIQUITIN_LIGASE_COP1	REACTOME_P53_INDEPENDENT_G1_S_DNA_DAMAGE_CHECKPOINT	REACTOME_VIF_MEDIATED_DEGRADATION_OF_APOBEC3G	REACTOME_CROSS_PRESENTATION_OF_SOLUBLE_EXOGENOUS_ANTIGENS_ENDOSOMES
ACTB	0	0	0	0	0	0	0	0	0	0	0	0	0
ACTN2	0	0	0	0	0	0	0	0	0	0	0	0	0
ACTN4	0	0	0	0	0	0	0	0	0	0	0	0	0
AP1M1	0	0	0	0	0	0	0	0	0	0	0	0	0
AP2A1	0	0	0	0	0	0	0	0	0	0	0	0	0
AP2A2	0	0	0	0	0	0	0	0	0	0	0	0	0
AP2M1	0	0	0	0	0	0	0	0	0	0	0	0	0
AP3B1	0	0	0	0	0	0	0	0	0	0	0	0	0
AP3S1	0	0	0	0	0	0	0	0	0	0	0	0	0
ARFGAP1	0	0	0	0	0	0	0	0	0	0	0	0	0
ARHGAP35	0	0	0	0	0	0	0	0	0	0	0	0	0
ARHGDIA	0	0	0	0	0	0	0	0	0	0	0	0	0
ARSB	0	0	0	0	0	0	0	0	0	0	0	0	0
ATP6V0D1	0	0	0	0	0	0	0	0	0	0	0	0	0
ATP6V1B2	0	0	0	0	0	0	0	0	0	0	0	0	0
ATP6V1D	0	0	0	0	0	0	0	0	0	0	0	0	0
ATP6V1E1	0	0	0	0	0	0	0	0	0	0	0	0	0
ATP6V1F	0	0	0	0	0	0	0	0	0	0	0	0	0
ATP6V1G1	0	0	0	0	0	0	0	0	0	0	0	0	0
BAD	0	0	0	0	0	0	0	0	0	0	0	0	0
BID	0	0	0	0	0	0	0	0	0	0	0	0	0
CDC42	0	0	0	0	0	0	0	0	0	0	0	0	0
CDH2	0	0	0	0	0	0	0	0	0	0	0	0	0
CDK4	0	0	0	0	0	0	0	0	0	0	0	0	0
CFL1	0	0	0	0	0	0	0	0	0	0	0	0	0
CHMP5	0	0	0	0	0	0	0	0	0	0	0	0	0
CHUK	0	0	0	0	0	0	0	0	0	0	0	0	0
CLTA	0	0	0	0	0	0	0	0	0	0	0	0	0
CLTB	0	0	0	0	0	0	0	0	0	0	0	0	0
CLTC	0	0	0	0	0	0	0	0	0	0	0	0	0
COL1A1	0	0	0	0	0	0	0	0	0	0	0	0	0
COL1A2	0	0	0	0	0	0	0	0	0	0	0	0	0
COL5A1	0	0	0	0	0	0	0	0	0	0	0	0	0
COL6A1	0	0	0	0	0	0	0	0	0	0	0	0	0
COL6A2	0	0	0	0	0	0	0	0	0	0	0	0	0
CRK	0	0	0	0	0	0	0	0	0	0	0	0	0
CTSA	0	0	0	0	0	0	0	0	0	0	0	0	0
CTSB	0	0	0	0	0	0	0	0	0	0	0	0	0
CTSL1	0	0	0	0	0	0	0	0	0	0	0	0	0
CTSZ	0	0	0	0	0	0	0	0	0	0	0	0	0
EEA1	0	0	0	0	0	0	0	0	0	0	0	0	0
EGFR	0	0	0	0	0	0	0	0	0	0	0	0	0
EHD1	0	0	0	0	0	0	0	0	0	0	0	0	0
EHD2	0	0	0	0	0	0	0	0	0	0	0	0	0
EPN1	0	0	0	0	0	0	0	0	0	0	0	0	0
FZD7	0	0	0	0	0	0	0	0	0	0	0	0	0
GNG12	0	0	0	0	0	0	0	0	0	0	0	0	0
GNS	0	0	0	0	0	0	0	0	0	0	0	0	0
HGS	0	0	0	0	0	0	0	0	0	0	0	0	0
HSPB1	0	0	0	0	0	0	0	0	0	0	0	0	0
IGF2R	0	0	0	0	0	0	0	0	0	0	0	0	0
ILK	0	0	0	0	0	0	0	0	0	0	0	0	0

NAME	REACTOME_ING_BY_WNT	REACTOME_REGULATION_OF_APOPTOSIS	REACTOME_ACTIVATION_OF_NF_KAPPAB_IN_B_CELLS	REACTOME_DOWNSTREAM_ING_EVENTS_OF_B_CELL_RECEPTOR_BCR	REACTOME_ING_BY_THE_B_CELL_RECEPTOR_BCR	REACTOME_METABOLISM_OF_MRNA	REACTOME_METABOLISM_OF_PROTEINS	REACTOME_FORMATION_OF_THE_TERNARY_COMPLEX_AND_SUBSEQUENTLY_THE_43S_COMPLEX	REACTOME_ACTIVATION_OF_THE_MRNA_UPON_BINDING_OF_THE_CAP_BINDING_COMPLEX_AND_EIFS_AND_SUBSEQUENT_BINDING_TO_43S	REACTOME_SRP_DEPENDENT_COTRANSLATIONAL_PROTEIN_TARGETING_TO_MEMBRANE	REACTOME_INFLUENZA_VIRAL_RNA_TRANSCRIPTION_AND_REPLICATION	REACTOME_NONSENSE_MEDIATED_DECAY_ENHANCED_BY_THE_EXON_JUNCTION_COMPLEX	REACTOME_PEPTIDE_CHAIN_ELONGATION
ACTB	0	0	0	0	0	0	1	0	0	0	0	0	0
ACTN2	0	0	0	0	0	0	0	0	0	0	0	0	0
ACTN4	0	0	0	0	0	0	0	0	0	0	0	0	0
AP1M1	0	0	0	0	0	0	0	0	0	0	0	0	0
AP2A1	0	0	0	0	0	0	0	0	0	0	0	0	0
AP2A2	0	0	0	0	0	0	0	0	0	0	0	0	0
AP2M1	0	0	0	0	0	0	0	0	0	0	0	0	0
AP3B1	0	0	0	0	0	0	0	0	0	0	0	0	0
AP3S1	0	0	0	0	0	0	0	0	0	0	0	0	0
ARFGAP1	0	0	0	0	0	0	0	0	0	0	0	0	0
ARHGAP35	0	0	0	0	0	0	0	0	0	0	0	0	0
ARHGDIA	0	0	0	0	0	0	0	0	0	0	0	0	0
ARSB	0	0	0	0	0	0	1	0	0	0	0	0	0
ATP6V0D1	0	0	0	0	0	0	0	0	0	0	0	0	0
ATP6V1B2	0	0	0	0	0	0	0	0	0	0	0	0	0
ATP6V1D	0	0	0	0	0	0	0	0	0	0	0	0	0
ATP6V1E1	0	0	0	0	0	0	0	0	0	0	0	0	0
ATP6V1F	0	0	0	0	0	0	0	0	0	0	0	0	0
ATP6V1G1	0	0	0	0	0	0	0	0	0	0	0	0	0
BAD	0	0	0	1	1	0	0	0	0	0	0	0	0
BID	0	0	0	0	0	0	0	0	0	0	0	0	0
CDC42	0	0	0	0	0	0	0	0	0	0	0	0	0
CDH2	0	0	0	0	0	0	0	0	0	0	0	0	0
CDK4	0	0	0	0	0	0	0	0	0	0	0	0	0
CFL1	0	0	0	0	0	0	0	0	0	0	0	0	0
CHMP5	0	0	0	0	0	0	0	0	0	0	0	0	0
CHUK	0	0	1	1	1	0	0	0	0	0	0	0	0
CLTA	0	0	0	0	0	0	0	0	0	0	0	0	0
CLTB	0	0	0	0	0	0	0	0	0	0	0	0	0
CLTC	0	0	0	0	0	0	0	0	0	0	0	0	0
COL1A1	0	0	0	0	0	0	0	0	0	0	0	0	0
COL1A2	0	0	0	0	0	0	0	0	0	0	0	0	0
COL5A1	0	0	0	0	0	0	0	0	0	0	0	0	0
COL6A1	0	0	0	0	0	0	0	0	0	0	0	0	0
COL6A2	0	0	0	0	0	0	0	0	0	0	0	0	0
CRK	0	0	0	0	0	0	0	0	0	0	0	0	0
CTSA	0	0	0	0	0	0	0	0	0	0	0	0	0
CTSB	0	0	0	0	0	0	0	0	0	0	0	0	0
CTSL1	0	0	0	0	0	0	0	0	0	0	0	0	0
CTSZ	0	0	0	0	0	0	0	0	0	0	0	0	0
EEA1	0	0	0	0	0	0	0	0	0	0	0	0	0
EGFR	0	0	0	0	0	0	0	0	0	0	0	0	0
EHD1	0	0	0	0	0	0	0	0	0	0	0	0	0
EHD2	0	0	0	0	0	0	0	0	0	0	0	0	0
EPN1	0	0	0	0	0	0	0	0	0	0	0	0	0
FZD7	0	0	0	0	0	0	0	0	0	0	0	0	0
GNG12	0	0	0	0	0	0	0	0	0	0	0	0	0
GNS	0	0	0	0	0	0	0	0	0	0	0	0	0
HGS	0	0	0	0	0	0	0	0	0	0	0	0	0
HSPB1	0	0	0	0	0	1	0	0	0	0	0	0	0
IGF2R	0	0	0	0	0	0	0	0	0	0	0	0	0
ILK	0	0	0	0	0	0	0	0	0	0	0	0	0

NAME	REACTOME_3_UTR_MEDIATED_TRANSLATIONAL_REGULATION	REACTOME_TRANSLATION	REACTOME_CELL_SURFACE_INTERACTIONS_AT_THE_VASCULAR_WALL	REACTOME_INTEGRIN_CELL_SURFACE_INTERACTIONS	REACTOME_RESPONSE_TO_ELEVATED_PLATELET_CYTOSOLIC_CA2_	REACTOME_HEMOSTASIS	REACTOME_REGULATION_OF_INSULIN_SECRETION	REACTOME_INTEGRATION_OF_ENERGY_METABOLISM	REACTOME_GPCR_DOWNSTREAM_ING	REACTOME_ING_BY_GPCR	REACTOME_ING_BY_ILS	REACTOME_ING_BY_FGFR_IN_DISEASE	REACTOME_ING_BY_ERBB2
ACTB	0	0	0	0	0	0	0	0	0	0	0	0	0
ACTN2	0	0	0	0	1	1	0	0	0	0	0	0	0
ACTN4	0	0	0	0	1	1	0	0	0	0	0	0	0
AP1M1	0	0	0	0	0	0	0	0	0	0	0	0	0
AP2A1	0	0	0	0	0	0	0	0	0	0	0	0	0
AP2A2	0	0	0	0	0	0	0	0	0	0	0	0	0
AP2M1	0	0	0	0	0	0	0	0	0	0	0	0	0
AP3B1	0	0	0	0	0	0	0	0	0	0	0	0	0
AP3S1	0	0	0	0	0	0	0	0	0	0	0	0	0
ARFGAP1	0	0	0	0	0	0	0	0	0	0	0	0	0
ARHGAP35	0	0	0	0	0	0	0	0	0	0	0	0	0
ARHGDIA	0	0	0	0	0	0	0	0	0	0	0	0	0
ARSB	0	0	0	0	0	0	0	0	0	0	0	0	0
ATP6V0D1	0	0	0	0	0	0	0	0	0	0	0	0	0
ATP6V1B2	0	0	0	0	0	0	0	0	0	0	0	0	0
ATP6V1D	0	0	0	0	0	0	0	0	0	0	0	0	0
ATP6V1E1	0	0	0	0	0	0	0	0	0	0	0	0	0
ATP6V1F	0	0	0	0	0	0	0	0	0	0	0	0	0
ATP6V1G1	0	0	0	0	0	0	0	0	0	0	0	0	0
BAD	0	0	0	0	0	0	0	0	0	0	0	1	1
BID	0	0	0	0	0	0	0	0	0	0	0	0	0
CDC42	0	0	0	0	0	1	0	0	1	1	0	0	0
CDH2	0	0	0	0	0	0	0	0	0	0	0	0	0
CDK4	0	0	0	0	0	0	0	0	0	0	0	0	0
CFL1	0	0	0	0	1	1	0	0	0	0	0	0	0
CHMP5	0	0	0	0	0	0	0	0	0	0	0	0	0
CHUK	0	0	0	0	0	0	0	0	0	0	1	1	1
CLTA	0	0	0	0	0	0	0	0	0	0	0	0	0
CLTB	0	0	0	0	0	0	0	0	0	0	0	0	0
CLTC	0	0	0	0	0	0	0	0	0	0	0	0	0
COL1A1	0	0	1	1	0	1	0	0	0	0	0	0	0
COL1A2	0	0	1	1	0	1	0	0	0	0	0	0	0
COL5A1	0	0	0	0	0	0	0	0	0	0	0	0	0
COL6A1	0	0	0	0	0	0	0	0	0	0	0	0	0
COL6A2	0	0	0	0	0	0	0	0	0	0	0	0	0
CRK	0	0	0	1	0	1	0	0	0	0	1	0	0
CTSA	0	0	0	0	0	0	0	0	0	0	0	0	0
CTSB	0	0	0	0	0	0	0	0	0	0	0	0	0
CTSL1	0	0	0	0	0	0	0	0	0	0	0	0	0
CTSZ	0	0	0	0	0	0	0	0	0	0	0	0	0
EEA1	0	0	0	0	0	0	0	0	0	0	0	0	0
EGFR	0	0	0	0	0	0	0	0	0	1	0	0	1
EHD1	0	0	0	0	0	1	0	0	0	0	0	0	0
EHD2	0	0	0	0	0	1	0	0	0	0	0	0	0
EPN1	0	0	0	0	0	0	0	0	0	0	0	0	0
FZD7	0	0	0	0	0	0	0	0	0	1	0	0	0
GNG12	0	0	0	0	0	1	1	1	1	1	0	0	0
GNS	0	0	0	0	0	0	0	0	0	0	0	0	0
HGS	0	0	0	0	0	0	0	0	0	0	0	0	0
HSPB1	0	0	0	0	0	0	0	0	0	0	0	0	0
IGF2R	0	0	0	0	0	0	0	0	0	0	0	0	0
ILK	0	0	0	0	0	0	0	0	0	0	0	0	0

NAME	REACTOME_ING_BY_FGFR	REACTOME_DOWNSTREAM__TRANSDUCTION	REACTOME_LING_BY_NGF	REACTOME_NGF_LING_VIA_TRKA_FROM_THE_PLASMA_MEMBRANE	REACTOME_ING_BY_EGFR_IN_CANCER	REACTOME_ING_BY_PDGF	REACTOME_SEMAPHORIN_INTERACTIONS	REACTOME_L1CAM_INTERACTIONS	REACTOME_DEVELOPMENTAL_BIOLOGY	REACTOME_AXON_GUIDANCE	KEGG_ERBB_ING_PATHWAY	KEGG_INSULIN_ING_PATHWAY
ACTB	0	0	0	0	0	0	0	0	0	0	0	0
ACTN2	0	0	0	0	0	0	0	0	0	0	0	0
ACTN4	0	0	0	0	0	0	0	0	0	0	0	0
AP1M1	0	0	0	0	0	0	0	0	0	0	0	0
AP2A1	0	0	1	1	1	0	0	1	1	1	0	0
AP2A2	0	0	1	1	1	0	0	0	0	0	0	0
AP2M1	0	0	1	1	1	0	0	1	1	1	0	0
AP3B1	0	0	0	0	0	0	0	0	0	0	0	0
AP3S1	0	0	0	0	0	0	0	0	0	0	0	0
ARFGAP1	0	0	0	0	0	0	0	0	0	0	0	0
ARHGAP35	0	0	0	0	0	0	1	0	1	1	0	0
ARHGDIA	0	0	1	0	0	0	0	0	0	0	0	0
ARSB	0	0	0	0	0	0	0	0	0	0	0	0
ATP6V0D1	0	0	0	0	0	0	0	0	0	0	0	0
ATP6V1B2	0	0	0	0	0	0	0	0	0	0	0	0
ATP6V1D	0	0	0	0	0	0	0	0	0	0	0	0
ATP6V1E1	0	0	0	0	0	0	0	0	0	0	0	0
ATP6V1F	0	0	0	0	0	0	0	0	0	0	0	0
ATP6V1G1	0	0	0	0	0	0	0	0	0	0	0	0
BAD	1	1	1	1	1	1	0	0	0	0	1	1
BID	0	0	0	0	0	0	0	0	0	0	0	0
CDC42	0	0	0	0	1	0	1	0	1	1	0	0
CDH2	0	0	0	0	0	0	0	0	1	0	0	0
CDK4	0	0	0	0	0	0	0	0	1	0	0	0
CFL1	0	0	0	0	0	0	1	0	1	1	0	0
CHMP5	0	0	0	0	0	0	0	0	0	0	0	0
CHUK	1	1	1	1	1	1	0	0	0	0	0	0
CLTA	0	0	1	1	1	0	0	0	0	0	0	0
CLTB	0	0	0	0	0	0	0	0	0	0	0	0
CLTC	0	0	1	1	1	0	0	0	0	0	0	0
COL1A1	0	0	0	0	0	1	0	0	1	1	0	0
COL1A2	0	0	0	0	0	1	0	0	1	1	0	0
COL5A1	0	0	0	0	0	1	0	0	1	1	0	0
COL6A1	0	0	0	0	0	1	0	0	1	1	0	0
COL6A2	0	0	0	0	0	1	0	0	1	1	0	0
CRK	0	1	1	1	0	0	0	0	0	0	1	1
CTSA	0	0	0	0	0	0	0	0	0	0	0	0
CTSB	0	0	0	0	0	0	0	0	0	0	0	0
CTSL1	0	0	0	0	0	0	0	0	0	0	0	0
CTSZ	0	0	0	0	0	0	0	0	0	0	0	0
EEA1	0	0	0	0	0	0	0	0	0	0	0	0
EGFR	0	0	0	0	1	0	0	1	1	1	1	0
EHD1	0	0	0	0	0	0	0	0	0	0	0	0
EHD2	0	0	0	0	0	0	0	0	0	0	0	0
EPN1	0	0	0	0	1	0	0	0	0	0	0	0
FZD7	0	0	0	0	0	0	0	0	0	0	0	0
GNG12	0	0	0	0	0	0	0	0	0	0	0	0
GNS	0	0	0	0	0	0	0	0	0	0	0	0
HGS	0	0	0	0	1	0	0	0	0	0	0	0
HSPB1	0	0	0	0	0	0	0	0	0	0	0	0
IGF2R	0	0	0	0	0	0	0	0	0	0	0	0
ILK	0	0	0	0	0	0	0	0	0	0	0	0

NAME	KEGG_PATHWAYS_IN_CANCER	KEGG_PROSTATE_CANCER	KEGG_CHEMOKINE_ING_PATHWAY	KEGG_NEUROTROPHIN_ING_PATHWAY	KEGG_MAPK_ING_PATHWAY	KEGG_ARRHYTHMOGENIC_RIGHT_VENTRICULAR_CARDIOMYOPATHY_ARVC	KEGG_ECM_RECEPTOR_INTERACTION	KEGG_FOCAL_ADHESION
ACTB	0	0	0	0	0	1	0	1
ACTN2	0	0	0	0	0	1	0	1
ACTN4	0	0	0	0	0	1	0	1
AP1M1	0	0	0	0	0	0	0	0
AP2A1	0	0	0	0	0	0	0	0
AP2A2	0	0	0	0	0	0	0	0
AP2M1	0	0	0	0	0	0	0	0
AP3B1	0	0	0	0	0	0	0	0
AP3S1	0	0	0	0	0	0	0	0
ARFGAP1	0	0	0	0	0	0	0	0
ARHGAP35	0	0	0	0	0	0	0	0
ARHGDIA	0	0	0	1	0	0	0	0
ARSB	0	0	0	0	0	0	0	0
ATP6V0D1	0	0	0	0	0	0	0	0
ATP6V1B2	0	0	0	0	0	0	0	0
ATP6V1D	0	0	0	0	0	0	0	0
ATP6V1E1	0	0	0	0	0	0	0	0
ATP6V1F	0	0	0	0	0	0	0	0
ATP6V1G1	0	0	0	0	0	0	0	0
BAD	1	1	0	1	0	0	0	1
BID	1	0	0	0	0	0	0	0
CDC42	1	0	1	1	1	0	0	0
CDH2	0	0	0	0	0	1	0	0
CDK4	1	0	0	0	0	0	0	0
CFL1	0	0	0	0	0	0	0	0
CHMP5	0	0	0	0	0	0	0	0
CHUK	1	1	1	0	1	0	0	0
CLTA	0	0	0	0	0	0	0	0
CLTB	0	0	0	0	0	0	0	0
CLTC	0	0	0	0	0	0	0	0
COL1A1	0	0	0	0	0	0	1	1
COL1A2	0	0	0	0	0	0	1	1
COL5A1	0	0	0	0	0	0	1	1
COL6A1	0	0	0	0	0	0	1	1
COL6A2	0	0	0	0	0	0	1	1
CRK	1	0	1	1	1	0	0	1
CTSA	0	0	0	0	0	0	0	0
CTSB	0	0	0	0	0	0	0	0
CTSL1	0	0	0	0	0	0	0	0
CTSZ	0	0	0	0	0	0	0	0
EEA1	0	0	0	0	0	0	0	0
EGFR	1	1	0	0	1	0	0	1
EHD1	0	0	0	0	0	0	0	0
EHD2	0	0	0	0	0	0	0	0
EPN1	0	0	0	0	0	0	0	0
FZD7	1	0	0	0	0	0	0	0
GNG12	0	0	1	0	1	0	0	0
GNS	0	0	0	0	0	0	0	0
HGS	0	0	0	0	0	0	0	0
HSPB1	0	0	0	0	1	0	0	0
IGF2R	0	0	0	0	0	0	0	0
ILK	0	0	0	0	0	0	0	1

Table S6 The differential proteins contribute to the enrichment of the significant pathways for the public dataset

Core enrichment proteins in down-regulated pathways of hESCs/HFs (hESCs vs HFs)

NAME	REACTOME_UNFOLDED_PROTEIN_RESPONSE	REACTOME_DIABETES_PATHWAYS	REACTOME_CELL_CELL_COMMUNICATION	REACTOME_GOLGI_ASSOCIATED_VESICLE_BIOGENESIS	REACTOME_TRANS_GOLGI_NETWORK_VESICLE_BUDDING	REACTOME_MEMBRANE_TRAFFICKING	REACTOME_NEURONAL_SYSTEM	REACTOME_TRANSMISSION_ACROSS_CHEMICAL_SYNAPSES	REACTOME_POST_TRANSLATIONAL_PROTEIN_MODIFICATION	REACTOME_ASPARAGINE_N_LINKED_GLYCOSYLATION	REACTOME_ING_BY_INSULIN_RECEPTOR	REACTOME_PREFOLDIN_MEDIATED_TRANSFER_OF_SUBSTRATE_TO_CCT_TRIC	REACTOME_PROTEIN_FOLDING
IQGAP1	0	0	1	0	0	0	0	0	0	0	0	0	0
ITGA2	0	0	0	0	0	0	0	0	0	0	0	0	0
ITGA3	0	0	0	0	0	0	0	0	0	0	0	0	0
ITGA5	0	0	0	0	0	0	0	0	0	0	0	0	0
ITGAV	0	0	0	0	0	0	0	0	0	0	0	0	0
ITGB1	0	0	1	0	0	0	0	0	0	0	0	0	0
JAK1	0	0	0	0	0	0	0	0	0	0	0	0	0
LAMB1	0	0	0	0	0	0	0	0	0	0	0	0	0
LMNA	1	1	0	0	0	0	0	0	0	0	0	0	0
MAP2K2	0	0	0	0	0	0	0	0	0	0	1	0	0
MAP2K3	0	0	0	0	0	0	0	0	0	0	0	0	0
MAPK1	0	0	0	0	0	0	1	1	0	0	1	0	0
MARCKS	0	0	0	0	0	0	0	0	0	0	0	0	0
MSN	0	0	0	0	0	0	0	0	0	0	0	0	0
MYH9	0	0	0	0	0	0	0	0	0	0	0	0	0
MYL9	0	0	0	0	0	0	0	0	0	0	0	0	0
PAK2	0	0	0	0	0	0	0	0	0	0	0	0	0
PARVA	0	0	1	0	0	0	0	0	0	0	0	0	0
PDGFRB	0	0	0	0	0	0	0	0	0	0	0	0	0
PFN1	0	0	0	0	0	0	0	0	0	0	0	0	0
PPP2CB	0	0	0	0	0	0	0	0	0	0	0	0	0
PRKAA1	0	0	0	0	0	0	0	0	0	0	1	0	0
PRKAR1A	0	0	0	0	0	0	0	0	0	0	0	0	0
PRKAR2A	0	0	0	0	0	0	0	0	0	0	0	0	0
PRKCA	0	0	0	0	0	0	1	1	0	0	0	0	0
PSMA1	0	0	0	0	0	0	0	0	0	0	0	0	0
PSMA2	0	0	0	0	0	0	0	0	0	0	0	0	0
PSMA3	0	0	0	0	0	0	0	0	0	0	0	0	0
PSMA5	0	0	0	0	0	0	0	0	0	0	0	0	0
PSMA6	0	0	0	0	0	0	0	0	0	0	0	0	0
PSMA7	0	0	0	0	0	0	0	0	0	0	0	0	0
PSMB1	0	0	0	0	0	0	0	0	0	0	0	0	0
PSMB2	0	0	0	0	0	0	0	0	0	0	0	0	0
PSMB3	0	0	0	0	0	0	0	0	0	0	0	0	0
PSMB4	0	0	0	0	0	0	0	0	0	0	0	0	0
PSMB5	0	0	0	0	0	0	0	0	0	0	0	0	0
PSMB7	0	0	0	0	0	0	0	0	0	0	0	0	0
PSMC1	0	0	0	0	0	0	0	0	0	0	0	0	0
PSMC2	0	0	0	0	0	0	0	0	0	0	0	0	0
PSMC3	0	0	0	0	0	0	0	0	0	0	0	0	0
PSMC5	0	0	0	0	0	0	0	0	0	0	0	0	0
PSMD11	0	0	0	0	0	0	0	0	0	0	0	0	0
PSMD12	0	0	0	0	0	0	0	0	0	0	0	0	0
PSMD13	0	0	0	0	0	0	0	0	0	0	0	0	0
PSMD14	0	0	0	0	0	0	0	0	0	0	0	0	0
PSMD2	0	0	0	0	0	0	0	0	0	0	0	0	0
PSMD3	0	0	0	0	0	0	0	0	0	0	0	0	0
PSMD4	0	0	0	0	0	0	0	0	0	0	0	0	0
PSMD6	0	0	0	0	0	0	0	0	0	0	0	0	0
PSMD7	0	0	0	0	0	0	0	0	0	0	0	0	0
PSME1	0	0	0	0	0	0	0	0	0	0	0	0	0
PSME2	0	0	0	0	0	0	0	0	0	0	0	0	0

NAME	REACTOME_INNATE_IMMUNE_SYSTEM	REACTOME_MHC_CLASS_II_ANTIGEN_PRESENTATION	REACTOME_IMMUNE_SYSTEM	REACTOME_ADAPTIVE_IMMUNE_SYSTEM	REACTOME_APOPTOSIS	REACTOME_CLASS_I_MHC_MEDIATED_ANTIGEN_PROCESSING_PRESENTATION	REACTOME_ANTIGEN_PROCESSING_UBIQUITINATION_PROTEASOME_DEGRADATION	REACTOME_ER_PHAGOSOME_PATHWAY	REACTOME_ANTIGEN_PROCESSING_CROSS_PRESENTATION	REACTOME_REGULATION_OF_MRNA_STABILITY_BY_PROTEINS_THAT_BIND_AU_RICH_ELEMENTS	REACTOME_DESTABILIZATION_OF_MRNA_BY_AUF1_HNRNP_D0	REACTOME_REGULATION_OF_ORNITHINE_DECARBOXYLASE_ODC	REACTOME_REGULATION_OF_MITOTIC_CELL_CYCLE
IQGAP1	0	0	0	0	0	0	0	0	0	0	0	0	0
ITGA2	0	0	0	0	0	0	0	0	0	0	0	0	0
ITGA3	0	0	0	0	0	0	0	0	0	0	0	0	0
ITGA5	0	0	0	0	0	0	0	0	0	0	0	0	0
ITGAV	0	0	1	1	0	1	0	0	1	0	0	0	0
ITGB1	0	0	1	1	0	0	0	0	0	0	0	0	0
JAK1	0	0	1	0	0	0	0	0	0	0	0	0	0
LAMB1	0	0	0	0	0	0	0	0	0	0	0	0	0
LMNA	0	0	0	0	1	0	0	0	0	0	0	0	0
MAP2K2	1	0	1	0	0	0	0	0	0	0	0	0	0
MAP2K3	1	0	1	0	0	0	0	0	0	0	0	0	0
MAPK1	1	0	1	0	0	0	0	0	0	0	0	0	0
MARCKS	0	0	0	0	0	0	0	0	0	0	0	0	0
MSN	0	0	0	0	0	0	0	0	0	0	0	0	0
MYH9	0	0	0	0	0	0	0	0	0	0	0	0	0
MYL9	0	0	0	0	0	0	0	0	0	0	0	0	0
PAK2	0	0	1	1	1	0	0	0	0	0	0	0	0
PARVA	0	0	0	0	0	0	0	0	0	0	0	0	0
PDGFRB	0	0	0	0	0	0	0	0	0	0	0	0	0
PFN1	0	0	0	0	0	0	0	0	0	0	0	0	0
PPP2CB	1	0	1	1	0	0	0	0	0	0	0	0	0
PRKAA1	0	0	0	0	0	0	0	0	0	0	0	0	0
PRKAR1A	0	0	0	0	0	0	0	0	0	0	0	0	0
PRKAR2A	0	0	0	0	0	0	0	0	0	0	0	0	0
PRKCA	0	0	0	0	0	0	0	0	0	1	0	0	0
PSMA1	0	0	1	1	1	1	1	1	1	1	1	1	1
PSMA2	0	0	1	1	1	1	1	1	1	1	1	1	1
PSMA3	0	0	0	1	1	1	1	1	1	1	1	1	1
PSMA5	0	0	1	1	1	1	1	1	1	1	1	1	1
PSMA6	0	0	0	1	1	1	1	1	1	1	1	1	1
PSMA7	0	0	1	1	1	1	1	1	1	1	1	1	1
PSMB1	0	0	0	1	1	1	1	1	1	1	1	1	1
PSMB2	0	0	1	1	1	1	1	1	1	1	1	1	1
PSMB3	0	0	1	1	1	1	1	1	1	1	1	1	1
PSMB4	0	0	1	1	1	1	1	1	1	1	1	1	1
PSMB5	0	0	1	1	1	1	1	1	1	1	1	1	1
PSMB7	0	0	0	1	1	1	1	1	1	1	1	1	1
PSMC1	0	0	1	1	1	1	1	1	1	1	1	1	1
PSMC2	0	0	0	1	1	1	1	1	1	1	1	1	1
PSMC3	0	0	0	0	1	1	1	1	1	1	1	1	1
PSMC5	0	0	0	1	1	1	1	1	1	1	1	1	1
PSMD11	0	0	0	1	1	1	1	1	1	1	1	1	1
PSMD12	0	0	0	0	1	1	1	1	1	1	1	1	1
PSMD13	0	0	0	0	1	1	1	1	1	1	1	1	1
PSMD14	0	0	0	0	1	1	1	1	1	1	1	1	1
PSMD2	0	0	1	1	1	1	1	1	1	1	1	1	1
PSMD3	0	0	0	0	1	1	1	1	1	1	1	1	1
PSMD4	0	0	1	1	1	1	1	1	1	1	1	1	1
PSMD6	0	0	0	1	1	1	1	1	1	1	1	1	1
PSMD7	0	0	0	0	1	1	1	1	1	1	1	1	1
PSME1	0	0	1	1	1	1	1	1	1	1	1	1	1
PSME2	0	0	1	1	1	1	1	1	1	1	1	1	1

NAME	REACTOME_CDT1_ASSOCIATION_WITH_THE_CDC6_ORC_ORIGIN_COMPLEX	REACTOME_APC_C_CDC20_MEDIATED_DEGRADATION_OF_MITOTIC_PROTEINS	REACTOME_APC_C_CDH1_MEDIATED_DEGRADATION_OF_CDC20_AND_OTHER_APC_C_CDH1_TARGETED_PROTEINS_IN_LATE_MITOSIS_EARLY_G1	REACTOME_CYCLIN_E_ASSOCIATED_EVENTS_DURING_G1_S_TRANSITION_	REACTOME_SCFSKP2_MEDIATED_DEGRADATION_OF_P27_P21	REACTOME_AUTODEGRADATION_OF_CDH1_BY_CDH1_APC_C	REACTOME_SCF_BETA_TRCP_MEDIATED_DEGRADATION_OF_EMI1	REACTOME_P53_DEPENDENT_G1_DNA_DAMAGE_RESPONSE	REACTOME_CDK_MEDIATED_PHOSPHORYLATION_AND_REMOVAL_OF_CDC6	REACTOME_AUTODEGRADATION_OF_THE_E3_UBIQUITIN_LIGASE_COP1	REACTOME_P53_INDEPENDENT_G1_S_DNA_DAMAGE_CHECKPOINT	REACTOME_VIF_MEDIATED_DEGRADATION_OF_APOBEC3G	REACTOME_CROSS_PRESENTATION_OF_SOLUBLE_EXOGENOUS_ANTIGENS_ENDOSOMES
IQGAP1	0	0	0	0	0	0	0	0	0	0	0	0	0
ITGA2	0	0	0	0	0	0	0	0	0	0	0	0	0
ITGA3	0	0	0	0	0	0	0	0	0	0	0	0	0
ITGA5	0	0	0	0	0	0	0	0	0	0	0	0	0
ITGAV	0	0	0	0	0	0	0	0	0	0	0	0	0
ITGB1	0	0	0	0	0	0	0	0	0	0	0	0	0
JAK1	0	0	0	0	0	0	0	0	0	0	0	0	0
LAMB1	0	0	0	0	0	0	0	0	0	0	0	0	0
LMNA	0	0	0	0	0	0	0	0	0	0	0	0	0
MAP2K2	0	0	0	0	0	0	0	0	0	0	0	0	0
MAP2K3	0	0	0	0	0	0	0	0	0	0	0	0	0
MAPK1	0	0	0	0	0	0	0	0	0	0	0	0	0
MARCKS	0	0	0	0	0	0	0	0	0	0	0	0	0
MSN	0	0	0	0	0	0	0	0	0	0	0	0	0
MYH9	0	0	0	0	0	0	0	0	0	0	0	0	0
MYL9	0	0	0	0	0	0	0	0	0	0	0	0	0
PAK2	0	0	0	0	0	0	0	0	0	0	0	0	0
PARVA	0	0	0	0	0	0	0	0	0	0	0	0	0
PDGFRB	0	0	0	0	0	0	0	0	0	0	0	0	0
PFN1	0	0	0	0	0	0	0	0	0	0	0	0	0
PPP2CB	0	0	0	0	0	0	0	0	0	0	0	0	0
PRKAA1	0	0	0	0	0	0	0	0	0	0	0	0	0
PRKAR1A	0	0	0	0	0	0	0	0	0	0	0	0	0
PRKAR2A	0	0	0	0	0	0	0	0	0	0	0	0	0
PRKCA	0	0	0	0	0	0	0	0	0	0	0	0	0
PSMA1	1	1	1	1	1	1	1	1	1	1	1	1	1
PSMA2	1	1	1	1	1	1	1	1	1	1	1	1	1
PSMA3	1	1	1	1	1	1	1	1	1	1	1	1	1
PSMA5	1	1	1	1	1	1	1	1	1	1	1	1	1
PSMA6	1	1	1	1	1	1	1	1	1	1	1	1	1
PSMA7	1	1	1	1	1	1	1	1	1	1	1	1	1
PSMB1	1	1	1	1	1	1	1	1	1	1	1	1	1
PSMB2	1	1	1	1	1	1	1	1	1	1	1	1	1
PSMB3	1	1	1	1	1	1	1	1	1	1	1	1	1
PSMB4	1	1	1	1	1	1	1	1	1	1	1	1	1
PSMB5	1	1	1	1	1	1	1	1	1	1	1	1	1
PSMB7	1	1	1	1	1	1	1	1	1	1	1	1	1
PSMC1	1	1	1	1	1	1	1	1	1	1	1	1	1
PSMC2	1	1	1	1	1	1	1	1	1	1	1	1	1
PSMC3	1	1	1	1	1	1	1	1	1	1	1	1	1
PSMC5	1	1	1	1	1	1	1	1	1	1	1	1	1
PSMD11	1	1	1	1	1	1	1	1	1	1	1	1	1
PSMD12	1	1	1	1	1	1	1	1	1	1	1	1	1
PSMD13	1	1	1	1	1	1	1	1	1	1	1	1	1
PSMD14	1	1	1	1	1	1	1	1	1	1	1	1	1
PSMD2	1	1	1	1	1	1	1	1	1	1	1	1	1
PSMD3	1	1	1	1	1	1	1	1	1	1	1	1	1
PSMD4	1	1	1	1	1	1	1	1	1	1	1	1	1
PSMD6	1	1	1	1	1	1	1	1	1	1	1	1	1
PSMD7	1	1	1	1	1	1	1	1	1	1	1	1	1
PSME1	1	1	1	1	1	1	1	1	1	1	1	1	1
PSME2	1	1	1	1	1	1	1	1	1	1	1	1	1

NAME	REACTOME_ING_BY_WNT	REACTOME_REGULATION_OF_APOPTOSIS	REACTOME_ACTIVATION_OF_NF_KAPPAB_IN_B_CELLS	REACTOME_DOWNSTREAM_ING_EVENTS_OF_B_CELL_RECEPTOR_BCR	REACTOME_ING_BY_THE_B_CELL_RECEPTOR_BCR	REACTOME_METABOLISM_OF_MRNA	REACTOME_METABOLISM_OF_PROTEINS	REACTOME_FORMATION_OF_THE_TERNARY_COMPLEX_AND_SUBSEQUENTLY_THE_43S_COMPLEX	REACTOME_ACTIVATION_OF_THE_MRNA_UPON_BINDING_OF_THE_CAP_BINDING_COMPLEX_AND_EIFS_AND_SUBSEQUENT_BINDING_TO_43S	REACTOME_SRP_DEPENDENT_COTRANSLATIONAL_PROTEIN_TARGETING_TO_MEMBRANE	REACTOME_INFLUENZA_VIRAL_RNA_TRANSCRIPTION_AND_REPLICATION	REACTOME_NONSENSE_MEDIATED_DECAY_ENHANCED_BY_THE_EXON_JUNCTION_COMPLEX	REACTOME_PEPTIDE_CHAIN_ELONGATION
IQGAP1	0	0	0	0	0	0	0	0	0	0	0	0	0
ITGA2	0	0	0	0	0	0	0	0	0	0	0	0	0
ITGA3	0	0	0	0	0	0	0	0	0	0	0	0	0
ITGA5	0	0	0	0	0	0	0	0	0	0	0	0	0
ITGAV	0	0	0	0	0	0	0	0	0	0	0	0	0
ITGB1	0	0	0	0	0	0	0	0	0	0	0	0	0
JAK1	0	0	0	0	0	0	0	0	0	0	0	0	0
LAMB1	0	0	0	0	0	0	0	0	0	0	0	0	0
LMNA	0	0	0	0	0	0	0	0	0	0	0	0	0
MAP2K2	0	0	0	0	0	0	0	0	0	0	0	0	0
MAP2K3	0	0	0	0	0	0	0	0	0	0	0	0	0
MAPK1	0	0	0	0	0	0	0	0	0	0	0	0	0
MARCKS	0	0	0	0	0	0	0	0	0	0	0	0	0
MSN	0	0	0	0	0	0	0	0	0	0	0	0	0
MYH9	0	0	0	0	0	0	0	0	0	0	0	0	0
MYL9	0	0	0	0	0	0	0	0	0	0	0	0	0
PAK2	0	1	0	0	0	0	0	0	0	0	0	0	0
PARVA	0	0	0	0	0	0	0	0	0	0	0	0	0
PDGFRB	0	0	0	0	0	0	0	0	0	0	0	0	0
PFN1	0	0	0	0	0	0	0	0	0	0	0	0	0
PPP2CB	1	0	0	0	0	0	0	0	0	0	0	0	0
PRKAA1	0	0	0	0	0	0	0	0	0	0	0	0	0
PRKAR1A	0	0	0	0	0	0	0	0	0	0	0	0	0
PRKAR2A	0	0	0	0	0	0	0	0	0	0	0	0	0
PRKCA	0	0	0	0	0	1	0	0	0	0	0	0	0
PSMA1	1	1	1	1	1	1	0	0	0	0	0	0	0
PSMA2	1	1	1	1	1	1	0	0	0	0	0	0	0
PSMA3	1	1	1	1	1	1	0	0	0	0	0	0	0
PSMA5	1	1	1	1	1	1	0	0	0	0	0	0	0
PSMA6	1	1	1	1	1	1	0	0	0	0	0	0	0
PSMA7	1	1	1	1	1	1	0	0	0	0	0	0	0
PSMB1	1	1	1	1	1	1	0	0	0	0	0	0	0
PSMB2	1	1	1	1	1	1	0	0	0	0	0	0	0
PSMB3	1	1	1	1	1	1	0	0	0	0	0	0	0
PSMB4	1	1	1	1	1	1	0	0	0	0	0	0	0
PSMB5	1	1	1	1	1	1	0	0	0	0	0	0	0
PSMB7	1	1	1	1	1	1	0	0	0	0	0	0	0
PSMC1	1	1	1	1	1	1	0	0	0	0	0	0	0
PSMC2	1	1	1	1	1	1	0	0	0	0	0	0	0
PSMC3	1	1	1	1	1	1	0	0	0	0	0	0	0
PSMC5	1	1	1	1	1	1	0	0	0	0	0	0	0
PSMD11	1	1	1	1	1	1	0	0	0	0	0	0	0
PSMD12	1	1	1	1	1	1	0	0	0	0	0	0	0
PSMD13	1	1	1	1	1	1	0	0	0	0	0	0	0
PSMD14	1	1	1	1	1	1	0	0	0	0	0	0	0
PSMD2	1	1	1	1	1	1	0	0	0	0	0	0	0
PSMD3	1	1	1	1	1	1	0	0	0	0	0	0	0
PSMD4	1	1	1	1	1	1	0	0	0	0	0	0	0
PSMD6	1	1	1	1	1	1	0	0	0	0	0	0	0
PSMD7	1	1	1	1	1	1	0	0	0	0	0	0	0
PSME1	1	1	1	1	1	1	0	0	0	0	0	0	0
PSME2	1	1	1	1	1	1	0	0	0	0	0	0	0

NAME	REACTOME_3_UTR_MEDIATED_TRANSLATIONAL_REGULATION	REACTOME_TRANSLATION	REACTOME_CELL_SURFACE_INTERACTIONS_AT_THE_VASCULAR_WALL	REACTOME_INTEGRIN_CELL_SURFACE_INTERACTIONS	REACTOME_RESPONSE_TO_ELEVATED_PLATELET_CYTOSOLIC_CA2_	REACTOME_HEMOSTASIS	REACTOME_REGULATION_OF_INSULIN_SECRETION	REACTOME_INTEGRATION_OF_ENERGY_METABOLISM	REACTOME_GPCR_DOWNSTREAM_ING	REACTOME_ING_BY_GPCR	REACTOME_ING_BY_ILS	REACTOME_ING_BY_FGFR_IN_DISEASE	REACTOME_ING_BY_ERBB2
IQGAP1	0	0	0	0	0	0	1	1	0	0	0	0	0
ITGA2	0	0	0	1	0	1	0	0	0	0	0	0	0
ITGA3	0	0	1	1	0	1	0	0	0	0	0	0	0
ITGA5	0	0	1	1	0	1	0	0	0	0	0	0	0
ITGAV	0	0	1	1	0	1	0	0	0	0	0	0	0
ITGB1	0	0	1	1	0	1	0	0	0	0	0	0	0
JAK1	0	0	0	0	0	0	0	0	0	0	1	0	0
LAMB1	0	0	0	1	0	0	0	0	0	0	0	0	0
LMNA	0	0	0	0	0	0	0	0	0	0	0	0	0
MAP2K2	0	0	0	0	0	0	0	0	0	1	1	1	1
MAP2K3	0	0	0	0	0	0	0	0	0	0	0	0	0
MAPK1	0	0	0	0	0	1	0	0	0	1	1	1	1
MARCKS	0	0	0	0	0	0	0	1	0	0	0	0	0
MSN	0	0	0	0	0	0	0	0	0	0	0	0	0
MYH9	0	0	0	0	0	0	0	0	0	0	0	0	0
MYL9	0	0	0	0	0	0	0	0	0	0	0	0	0
PAK2	0	0	0	0	0	0	0	0	0	0	0	0	0
PARVA	0	0	0	0	0	0	0	0	0	0	0	0	0
PDGFRB	0	0	0	0	0	0	0	0	0	0	0	0	0
PFN1	0	0	0	0	1	1	0	0	0	0	0	0	0
PPP2CB	0	0	0	0	0	1	0	1	0	1	0	1	0
PRKAA1	0	0	0	0	0	0	0	0	0	0	0	0	0
PRKAR1A	0	0	0	0	0	1	1	1	0	1	0	1	1
PRKAR2A	0	0	0	0	0	0	0	1	0	1	0	1	1
PRKCA	0	0	0	0	1	1	1	1	1	1	0	1	1
PSMA1	0	0	0	0	0	0	0	0	0	0	0	0	0
PSMA2	0	0	0	0	0	0	0	0	0	0	0	0	0
PSMA3	0	0	0	0	0	0	0	0	0	0	0	0	0
PSMA5	0	0	0	0	0	0	0	0	0	0	0	0	0
PSMA6	0	0	0	0	0	0	0	0	0	0	0	0	0
PSMA7	0	0	0	0	0	0	0	0	0	0	0	0	0
PSMB1	0	0	0	0	0	0	0	0	0	0	0	0	0
PSMB2	0	0	0	0	0	0	0	0	0	0	0	0	0
PSMB3	0	0	0	0	0	0	0	0	0	0	0	0	0
PSMB4	0	0	0	0	0	0	0	0	0	0	0	0	0
PSMB5	0	0	0	0	0	0	0	0	0	0	0	0	0
PSMB7	0	0	0	0	0	0	0	0	0	0	0	0	0
PSMC1	0	0	0	0	0	0	0	0	0	0	0	0	0
PSMC2	0	0	0	0	0	0	0	0	0	0	0	0	0
PSMC3	0	0	0	0	0	0	0	0	0	0	0	0	0
PSMC5	0	0	0	0	0	0	0	0	0	0	0	0	0
PSMD11	0	0	0	0	0	0	0	0	0	0	0	0	0
PSMD12	0	0	0	0	0	0	0	0	0	0	0	0	0
PSMD13	0	0	0	0	0	0	0	0	0	0	0	0	0
PSMD14	0	0	0	0	0	0	0	0	0	0	0	0	0
PSMD2	0	0	0	0	0	0	0	0	0	0	0	0	0
PSMD3	0	0	0	0	0	0	0	0	0	0	0	0	0
PSMD4	0	0	0	0	0	0	0	0	0	0	0	0	0
PSMD6	0	0	0	0	0	0	0	0	0	0	0	0	0
PSMD7	0	0	0	0	0	0	0	0	0	0	0	0	0
PSME1	0	0	0	0	0	0	0	0	0	0	0	0	0
PSME2	0	0	0	0	0	0	0	0	0	0	0	0	0

NAME	REACTOME_ING_BY_FGFR	REACTOME_DOWNSTREAM__TRANSDUCTION	REACTOME_LING_BY_NGF	REACTOME_NGF_LING_VIA_TRKA_FROM_THE_PLASMA_MEMBRANE	REACTOME_ING_BY_EGFR_IN_CANCER	REACTOME_ING_BY_PDGF	REACTOME_SEMAPHORIN_INTERACTIONS	REACTOME_L1CAM_INTERACTIONS	REACTOME_DEVELOPMENTAL_BIOLOGY	REACTOME_AXON_GUIDANCE	KEGG_ERBB_ING_PATHWAY	KEGG_INSULIN_ING_PATHWAY
IQGAP1	0	0	0	0	0	0	0	0	0	0	0	0
ITGA2	0	0	0	0	0	0	0	1	1	1	0	0
ITGA3	0	0	0	0	0	0	0	0	0	0	0	0
ITGA5	0	0	0	0	0	0	0	1	1	1	0	0
ITGAV	0	0	0	0	0	0	0	1	1	1	0	0
ITGB1	0	0	0	0	0	0	1	1	1	1	0	0
JAK1	0	0	0	0	0	0	0	0	0	0	0	0
LAMB1	0	0	0	0	0	0	0	1	1	1	0	0
LMNA	0	0	0	0	0	0	0	0	0	0	0	0
MAP2K2	1	1	1	1	1	0	0	1	1	1	1	1
MAP2K3	0	0	0	0	0	0	0	0	0	0	0	0
MAPK1	1	1	1	1	1	1	0	1	1	1	1	1
MARCKS	0	0	0	0	0	0	0	0	0	0	0	0
MSN	0	0	0	0	0	0	0	1	1	1	0	0
MYH9	0	0	0	0	0	0	1	0	1	1	0	0
MYL9	0	0	0	0	0	0	1	0	1	1	0	0
PAK2	0	0	0	0	0	0	1	0	1	1	1	0
PARVA	0	0	0	0	0	0	0	0	0	0	0	0
PDGFRB	0	1	0	0	0	1	0	0	0	0	0	0
PFN1	0	0	0	0	0	0	0	0	1	1	0	0
PPP2CB	1	0	1	1	0	0	0	0	0	0	0	0
PRKAA1	0	0	0	0	0	0	0	0	0	0	0	1
PRKAR1A	1	1	1	1	1	1	0	0	0	0	0	1
PRKAR2A	1	1	1	1	1	0	0	0	0	0	0	1
PRKCA	1	1	1	1	1	1	0	0	0	0	1	0
PSMA1	0	0	0	0	0	0	0	0	0	0	0	0
PSMA2	0	0	0	0	0	0	0	0	0	0	0	0
PSMA3	0	0	0	0	0	0	0	0	0	0	0	0
PSMA5	0	0	0	0	0	0	0	0	0	0	0	0
PSMA6	0	0	0	0	0	0	0	0	0	0	0	0
PSMA7	0	0	0	0	0	0	0	0	0	0	0	0
PSMB1	0	0	0	0	0	0	0	0	0	0	0	0
PSMB2	0	0	0	0	0	0	0	0	0	0	0	0
PSMB3	0	0	0	0	0	0	0	0	0	0	0	0
PSMB4	0	0	0	0	0	0	0	0	0	0	0	0
PSMB5	0	0	0	0	0	0	0	0	0	0	0	0
PSMB7	0	0	0	0	0	0	0	0	0	0	0	0
PSMC1	0	0	0	0	0	0	0	0	0	0	0	0
PSMC2	0	0	0	0	0	0	0	0	0	0	0	0
PSMC3	0	0	0	0	0	0	0	0	0	0	0	0
PSMC5	0	0	0	0	0	0	0	0	0	0	0	0
PSMD11	0	0	0	0	0	0	0	0	0	0	0	0
PSMD12	0	0	0	0	0	0	0	0	0	0	0	0
PSMD13	0	0	0	0	0	0	0	0	0	0	0	0
PSMD14	0	0	0	0	0	0	0	0	0	0	0	0
PSMD2	0	0	0	0	0	0	0	0	0	0	0	0
PSMD3	0	0	0	0	0	0	0	0	0	0	0	0
PSMD4	0	0	0	0	0	0	0	0	0	0	0	0
PSMD6	0	0	0	0	0	0	0	0	0	0	0	0
PSMD7	0	0	0	0	0	0	0	0	0	0	0	0
PSME1	0	0	0	0	0	0	0	0	0	0	0	0
PSME2	0	0	0	0	0	0	0	0	0	0	0	0

NAME	KEGG_PATHWAYS_IN_CANCER	KEGG_PROSTATE_CANCER	KEGG_CHEMOKINE_ING_PATHWAY	KEGG_NEUROTROPHIN_ING_PATHWAY	KEGG_MAPK_ING_PATHWAY	KEGG_ARRHYTHMOGENIC_RIGHT_VENTRICULAR_CARDIOMYOPATHY_ARVC	KEGG_ECM_RECEPTOR_INTERACTION	KEGG_FOCAL_ADHESION
IQGAP1	0	0	0	0	0	0	0	0
ITGA2	1	0	0	0	0	1	1	1
ITGA3	1	0	0	0	0	1	1	1
ITGA5	0	0	0	0	0	1	1	1
ITGAV	1	0	0	0	0	1	0	1
ITGB1	1	0	0	0	0	1	1	1
JAK1	1	0	0	0	0	0	0	0
LAMB1	1	0	0	0	0	0	0	1
LMNA	0	0	0	0	0	1	0	0
MAP2K2	1	0	0	1	1	0	0	0
MAP2K3	0	0	0	0	1	0	0	0
MAPK1	1	1	1	1	1	0	0	1
MARCKS	0	0	0	0	0	0	0	0
MSN	0	0	0	0	0	0	0	0
MYH9	0	0	0	0	0	0	0	0
MYL9	0	0	0	0	0	0	0	1
PAK2	0	0	0	0	1	0	0	1
PARVA	0	0	0	0	0	0	0	1
PDGFRB	1	1	0	0	1	0	0	1
PFN1	0	0	0	0	0	0	0	0
PPP2CB	0	0	0	0	0	0	0	0
PRKAA1	0	0	0	0	0	0	0	0
PRKAR1A	0	0	0	0	0	0	0	0
PRKAR2A	0	0	0	0	0	0	0	0
PRKCA	1	0	0	0	1	0	0	1
PSMA1	0	0	0	0	0	0	0	0
PSMA2	0	0	0	0	0	0	0	0
PSMA3	0	0	0	0	0	0	0	0
PSMA5	0	0	0	0	0	0	0	0
PSMA6	0	0	0	0	0	0	0	0
PSMA7	0	0	0	0	0	0	0	0
PSMB1	0	0	0	0	0	0	0	0
PSMB2	0	0	0	0	0	0	0	0
PSMB3	0	0	0	0	0	0	0	0
PSMB4	0	0	0	0	0	0	0	0
PSMB5	0	0	0	0	0	0	0	0
PSMB7	0	0	0	0	0	0	0	0
PSMC1	0	0	0	0	0	0	0	0
PSMC2	0	0	0	0	0	0	0	0
PSMC3	0	0	0	0	0	0	0	0
PSMC5	0	0	0	0	0	0	0	0
PSMD11	0	0	0	0	0	0	0	0
PSMD12	0	0	0	0	0	0	0	0
PSMD13	0	0	0	0	0	0	0	0
PSMD14	0	0	0	0	0	0	0	0
PSMD2	0	0	0	0	0	0	0	0
PSMD3	0	0	0	0	0	0	0	0
PSMD4	0	0	0	0	0	0	0	0
PSMD6	0	0	0	0	0	0	0	0
PSMD7	0	0	0	0	0	0	0	0
PSME1	0	0	0	0	0	0	0	0
PSME2	0	0	0	0	0	0	0	0

Table S6 The differential proteins contribute to the enrichment of the significant pathways for the public dataset

Core enrichment proteins in down-regulated pathways of hESCs/HFs (hESCs vs HFs)

NAME	REACTOME_UNFOLDED_PROTEIN_RESPONSE	REACTOME_DIABETES_PATHWAYS	REACTOME_CELL_CELL_COMMUNICATION	REACTOME_GOLGI_ASSOCIATED_VESICLE_BIOGENESIS	REACTOME_TRANS_GOLGI_NETWORK_VESICLE_BUDDING	REACTOME_MEMBRANE_TRAFFICKING	REACTOME_NEURONAL_SYSTEM	REACTOME_TRANSMISSION_ACROSS_CHEMICAL_SYNAPSES	REACTOME_POST_TRANSLATIONAL_PROTEIN_MODIFICATION	REACTOME_ASPARAGINE_N_LINKED_GLYCOSYLATION	REACTOME_ING_BY_INSULIN_RECEPTOR	REACTOME_PREFOLDIN_MEDIATED_TRANSFER_OF_SUBSTRATE_TO_CCT_TRIC	REACTOME_PROTEIN_FOLDING
PSMF1	0	0	0	0	0	0	0	0	0	0	0	0	0
PTPN1	0	0	0	0	0	0	0	0	0	0	0	0	0
RAB5C	0	0	0	1	1	1	0	0	0	0	0	0	0
RAC1	0	0	0	0	0	0	0	0	0	0	0	0	0
RAP1A	0	0	0	0	0	0	0	0	0	0	0	0	0
RASA1	0	0	0	0	0	0	0	0	0	0	0	0	0
RDX	0	0	0	0	0	0	0	0	0	0	0	0	0
RELA	0	0	0	0	0	0	0	0	0	0	0	0	0
ROCK1	0	0	0	0	0	0	0	0	0	0	0	0	0
ROCK2	0	0	0	0	0	0	0	0	0	0	0	0	0
RPL10A	0	0	0	0	0	0	0	0	0	0	0	0	0
RPL11	0	0	0	0	0	0	0	0	0	0	0	0	0
RPL12	0	0	0	0	0	0	0	0	0	0	0	0	0
RPL13	0	0	0	0	0	0	0	0	0	0	0	0	0
RPL14	0	0	0	0	0	0	0	0	0	0	0	0	0
RPL17	0	0	0	0	0	0	0	0	0	0	0	0	0
RPL18	0	0	0	0	0	0	0	0	0	0	0	0	0
RPL18A	0	0	0	0	0	0	0	0	0	0	0	0	0
RPL19	0	0	0	0	0	0	0	0	0	0	0	0	0
RPL23	0	0	0	0	0	0	0	0	0	0	0	0	0
RPL23A	0	0	0	0	0	0	0	0	0	0	0	0	0
RPL24	0	0	0	0	0	0	0	0	0	0	0	0	0
RPL26	0	0	0	0	0	0	0	0	0	0	0	0	0
RPL27	0	0	0	0	0	0	0	0	0	0	0	0	0
RPL27A	0	0	0	0	0	0	0	0	0	0	0	0	0
RPL28	0	0	0	0	0	0	0	0	0	0	0	0	0
RPL29	0	0	0	0	0	0	0	0	0	0	0	0	0
RPL30	0	0	0	0	0	0	0	0	0	0	0	0	0
RPL32	0	0	0	0	0	0	0	0	0	0	0	0	0
RPL34	0	0	0	0	0	0	0	0	0	0	0	0	0
RPL35A	0	0	0	0	0	0	0	0	0	0	0	0	0
RPL36	0	0	0	0	0	0	0	0	0	0	0	0	0
RPL36A	0	0	0	0	0	0	0	0	0	0	0	0	0
RPL38	0	0	0	0	0	0	0	0	0	0	0	0	0
RPL4	0	0	0	0	0	0	0	0	0	0	0	0	0
RPL7A	0	0	0	0	0	0	0	0	0	0	0	0	0
RPL8	0	0	0	0	0	0	0	0	0	0	0	0	0
RPLP0	0	0	0	0	0	0	0	0	0	0	0	0	0
RPLP1	0	0	0	0	0	0	0	0	0	0	0	0	0
RPLP2	0	0	0	0	0	0	0	0	0	0	0	0	0
RPS11	0	0	0	0	0	0	0	0	0	0	0	0	0
RPS12	0	0	0	0	0	0	0	0	0	0	0	0	0
RPS13	0	0	0	0	0	0	0	0	0	0	0	0	0
RPS15A	0	0	0	0	0	0	0	0	0	0	0	0	0
RPS16	0	0	0	0	0	0	0	0	0	0	0	0	0
RPS17	0	0	0	0	0	0	0	0	0	0	0	0	0
RPS18	0	0	0	0	0	0	0	0	0	0	0	0	0
RPS19	0	0	0	0	0	0	0	0	0	0	0	0	0
RPS2	0	0	0	0	0	0	0	0	0	0	0	0	0
RPS21	0	0	0	0	0	0	0	0	0	0	0	0	0
RPS23	0	0	0	0	0	0	0	0	0	0	0	0	0
RPS24	0	0	0	0	0	0	0	0	0	0	0	0	0

NAME	REACTOME_INNATE_IMMUNE_SYSTEM	REACTOME_MHC_CLASS_II_ANTIGEN_PRESENTATION	REACTOME_IMMUNE_SYSTEM	REACTOME_ADAPTIVE_IMMUNE_SYSTEM	REACTOME_APOPTOSIS	REACTOME_CLASS_I_MHC_MEDIATED_ANTIGEN_PROCESSING_PRESENTATION	REACTOME_ANTIGEN_PROCESSING_UBIQUITINATION_PROTEASOME_DEGRADATION	REACTOME_ER_PHAGOSOME_PATHWAY	REACTOME_ANTIGEN_PROCESSING_CROSS_PRESENTATION	REACTOME_REGULATION_OF_MRNA_STABILITY_BY_PROTEINS_THAT_BIND_AU_RICH_ELEMENTS	REACTOME_DESTABILIZATION_OF_MRNA_BY_AUF1_HNRNP_D0	REACTOME_REGULATION_OF_ORNITHINE_DECARBOXYLASE_ODC	REACTOME_REGULATION_OF_MITOTIC_CELL_CYCLE
PSMF1	0	0	1	1	1	1	1	1	1	1	1	1	1
PTPN1	0	0	1	0	0	0	0	0	0	0	0	0	0
RAB5C	0	0	0	0	0	0	0	0	0	0	0	0	0
RAC1	0	0	1	1	0	0	0	0	0	0	0	0	0
RAP1A	0	0	1	1	0	0	0	0	0	0	0	0	0
RASA1	0	0	0	0	0	0	0	0	0	0	0	0	0
RDX	0	0	0	0	0	0	0	0	0	0	0	0	0
RELA	1	0	1	1	0	0	0	0	0	0	0	0	0
ROCK1	0	0	0	0	1	0	0	0	0	0	0	0	0
ROCK2	0	0	0	0	0	0	0	0	0	0	0	0	0
RPL10A	0	0	0	0	0	0	0	0	0	0	0	0	0
RPL11	0	0	0	0	0	0	0	0	0	0	0	0	0
RPL12	0	0	0	0	0	0	0	0	0	0	0	0	0
RPL13	0	0	0	0	0	0	0	0	0	0	0	0	0
RPL14	0	0	0	0	0	0	0	0	0	0	0	0	0
RPL17	0	0	0	0	0	0	0	0	0	0	0	0	0
RPL18	0	0	0	0	0	0	0	0	0	0	0	0	0
RPL18A	0	0	0	0	0	0	0	0	0	0	0	0	0
RPL19	0	0	0	0	0	0	0	0	0	0	0	0	0
RPL23	0	0	0	0	0	0	0	0	0	0	0	0	0
RPL23A	0	0	0	0	0	0	0	0	0	0	0	0	0
RPL24	0	0	0	0	0	0	0	0	0	0	0	0	0
RPL26	0	0	0	0	0	0	0	0	0	0	0	0	0
RPL27	0	0	0	0	0	0	0	0	0	0	0	0	0
RPL27A	0	0	0	0	0	0	0	0	0	0	0	0	0
RPL28	0	0	0	0	0	0	0	0	0	0	0	0	0
RPL29	0	0	0	0	0	0	0	0	0	0	0	0	0
RPL30	0	0	0	0	0	0	0	0	0	0	0	0	0
RPL32	0	0	0	0	0	0	0	0	0	0	0	0	0
RPL34	0	0	0	0	0	0	0	0	0	0	0	0	0
RPL35A	0	0	0	0	0	0	0	0	0	0	0	0	0
RPL36	0	0	0	0	0	0	0	0	0	0	0	0	0
RPL36A	0	0	0	0	0	0	0	0	0	0	0	0	0
RPL38	0	0	0	0	0	0	0	0	0	0	0	0	0
RPL4	0	0	0	0	0	0	0	0	0	0	0	0	0
RPL7A	0	0	0	0	0	0	0	0	0	0	0	0	0
RPL8	0	0	0	0	0	0	0	0	0	0	0	0	0
RPLP0	0	0	0	0	0	0	0	0	0	0	0	0	0
RPLP1	0	0	0	0	0	0	0	0	0	0	0	0	0
RPLP2	0	0	0	0	0	0	0	0	0	0	0	0	0
RPS11	0	0	0	0	0	0	0	0	0	0	0	0	0
RPS12	0	0	0	0	0	0	0	0	0	0	0	0	0
RPS13	0	0	0	0	0	0	0	0	0	0	0	0	0
RPS15A	0	0	0	0	0	0	0	0	0	0	0	0	0
RPS16	0	0	0	0	0	0	0	0	0	0	0	0	0
RPS17	0	0	0	0	0	0	0	0	0	0	0	0	0
RPS18	0	0	0	0	0	0	0	0	0	0	0	0	0
RPS19	0	0	0	0	0	0	0	0	0	0	0	0	0
RPS2	0	0	0	0	0	0	0	0	0	0	0	0	0
RPS21	0	0	0	0	0	0	0	0	0	0	0	0	0
RPS23	0	0	0	0	0	0	0	0	0	0	0	0	0
RPS24	0	0	0	0	0	0	0	0	0	0	0	0	0

NAME	REACTOME_CDT1_ASSOCIATION_WITH_THE_CDC6_ORC_ORIGIN_COMPLEX	REACTOME_APC_C_CDC20_MEDIATED_DEGRADATION_OF_MITOTIC_PROTEINS	REACTOME_APC_C_CDH1_MEDIATED_DEGRADATION_OF_CDC20_AND_OTHER_APC_C_CDH1_TARGETED_PROTEINS_IN_LATE_MITOSIS_EARLY_G1	REACTOME_CYCLIN_E_ASSOCIATED_EVENTS_DURING_G1_S_TRANSITION_	REACTOME_SCFSKP2_MEDIATED_DEGRADATION_OF_P27_P21	REACTOME_AUTODEGRADATION_OF_CDH1_BY_CDH1_APC_C	REACTOME_SCF_BETA_TRCP_MEDIATED_DEGRADATION_OF_EMI1	REACTOME_P53_DEPENDENT_G1_DNA_DAMAGE_RESPONSE	REACTOME_CDK_MEDIATED_PHOSPHORYLATION_AND_REMOVAL_OF_CDC6	REACTOME_AUTODEGRADATION_OF_THE_E3_UBIQUITIN_LIGASE_COP1	REACTOME_P53_INDEPENDENT_G1_S_DNA_DAMAGE_CHECKPOINT	REACTOME_VIF_MEDIATED_DEGRADATION_OF_APOBEC3G	REACTOME_CROSS_PRESENTATION_OF_SOLUBLE_EXOGENOUS_ANTIGENS_ENDOSOMES
PSMF1	1	1	1	1	1	1	1	1	1	1	1	1	1
PTPN1	0	0	0	0	0	0	0	0	0	0	0	0	0
RAB5C	0	0	0	0	0	0	0	0	0	0	0	0	0
RAC1	0	0	0	0	0	0	0	0	0	0	0	0	0
RAP1A	0	0	0	0	0	0	0	0	0	0	0	0	0
RASA1	0	0	0	0	0	0	0	0	0	0	0	0	0
RDX	0	0	0	0	0	0	0	0	0	0	0	0	0
RELA	0	0	0	0	0	0	0	0	0	0	0	0	0
ROCK1	0	0	0	0	0	0	0	0	0	0	0	0	0
ROCK2	0	0	0	0	0	0	0	0	0	0	0	0	0
RPL10A	0	0	0	0	0	0	0	0	0	0	0	0	0
RPL11	0	0	0	0	0	0	0	0	0	0	0	0	0
RPL12	0	0	0	0	0	0	0	0	0	0	0	0	0
RPL13	0	0	0	0	0	0	0	0	0	0	0	0	0
RPL14	0	0	0	0	0	0	0	0	0	0	0	0	0
RPL17	0	0	0	0	0	0	0	0	0	0	0	0	0
RPL18	0	0	0	0	0	0	0	0	0	0	0	0	0
RPL18A	0	0	0	0	0	0	0	0	0	0	0	0	0
RPL19	0	0	0	0	0	0	0	0	0	0	0	0	0
RPL23	0	0	0	0	0	0	0	0	0	0	0	0	0
RPL23A	0	0	0	0	0	0	0	0	0	0	0	0	0
RPL24	0	0	0	0	0	0	0	0	0	0	0	0	0
RPL26	0	0	0	0	0	0	0	0	0	0	0	0	0
RPL27	0	0	0	0	0	0	0	0	0	0	0	0	0
RPL27A	0	0	0	0	0	0	0	0	0	0	0	0	0
RPL28	0	0	0	0	0	0	0	0	0	0	0	0	0
RPL29	0	0	0	0	0	0	0	0	0	0	0	0	0
RPL30	0	0	0	0	0	0	0	0	0	0	0	0	0
RPL32	0	0	0	0	0	0	0	0	0	0	0	0	0
RPL34	0	0	0	0	0	0	0	0	0	0	0	0	0
RPL35A	0	0	0	0	0	0	0	0	0	0	0	0	0
RPL36	0	0	0	0	0	0	0	0	0	0	0	0	0
RPL36A	0	0	0	0	0	0	0	0	0	0	0	0	0
RPL38	0	0	0	0	0	0	0	0	0	0	0	0	0
RPL4	0	0	0	0	0	0	0	0	0	0	0	0	0
RPL7A	0	0	0	0	0	0	0	0	0	0	0	0	0
RPL8	0	0	0	0	0	0	0	0	0	0	0	0	0
RPLP0	0	0	0	0	0	0	0	0	0	0	0	0	0
RPLP1	0	0	0	0	0	0	0	0	0	0	0	0	0
RPLP2	0	0	0	0	0	0	0	0	0	0	0	0	0
RPS11	0	0	0	0	0	0	0	0	0	0	0	0	0
RPS12	0	0	0	0	0	0	0	0	0	0	0	0	0
RPS13	0	0	0	0	0	0	0	0	0	0	0	0	0
RPS15A	0	0	0	0	0	0	0	0	0	0	0	0	0
RPS16	0	0	0	0	0	0	0	0	0	0	0	0	0
RPS17	0	0	0	0	0	0	0	0	0	0	0	0	0
RPS18	0	0	0	0	0	0	0	0	0	0	0	0	0
RPS19	0	0	0	0	0	0	0	0	0	0	0	0	0
RPS2	0	0	0	0	0	0	0	0	0	0	0	0	0
RPS21	0	0	0	0	0	0	0	0	0	0	0	0	0
RPS23	0	0	0	0	0	0	0	0	0	0	0	0	0
RPS24	0	0	0	0	0	0	0	0	0	0	0	0	0

NAME	REACTOME_ING_BY_WNT	REACTOME_REGULATION_OF_APOPTOSIS	REACTOME_ACTIVATION_OF_NF_KAPPAB_IN_B_CELLS	REACTOME_DOWNSTREAM_ING_EVENTS_OF_B_CELL_RECEPTOR_BCR	REACTOME_ING_BY_THE_B_CELL_RECEPTOR_BCR	REACTOME_METABOLISM_OF_MRNA	REACTOME_METABOLISM_OF_PROTEINS	REACTOME_FORMATION_OF_THE_TERNARY_COMPLEX_AND_SUBSEQUENTLY_THE_43S_COMPLEX	REACTOME_ACTIVATION_OF_THE_MRNA_UPON_BINDING_OF_THE_CAP_BINDING_COMPLEX_AND_EIFS_AND_SUBSEQUENT_BINDING_TO_43S	REACTOME_SRP_DEPENDENT_COTRANSLATIONAL_PROTEIN_TARGETING_TO_MEMBRANE	REACTOME_INFLUENZA_VIRAL_RNA_TRANSCRIPTION_AND_REPLICATION	REACTOME_NONSENSE_MEDIATED_DECAY_ENHANCED_BY_THE_EXON_JUNCTION_COMPLEX	REACTOME_PEPTIDE_CHAIN_ELONGATION
PSMF1	1	1	1	1	1	1	0	0	0	0	0	0	0
PTPN1	0	0	0	0	0	0	0	0	0	0	0	0	0
RAB5C	0	0	0	0	0	0	0	0	0	0	0	0	0
RAC1	0	0	0	0	0	0	0	0	0	0	0	0	0
RAP1A	0	0	0	0	0	0	0	0	0	0	0	0	0
RASA1	0	0	0	0	0	0	0	0	0	0	0	0	0
RDX	0	0	0	0	0	0	0	0	0	0	0	0	0
RELA	0	0	1	1	1	0	0	0	0	0	0	0	0
ROCK1	0	0	0	0	0	0	0	0	0	0	0	0	0
ROCK2	0	0	0	0	0	0	0	0	0	0	0	0	0
RPL10A	0	0	0	0	0	1	1	0	0	1	1	1	1
RPL11	0	0	0	0	0	0	0	0	0	1	1	1	1
RPL12	0	0	0	0	0	1	1	0	0	1	1	1	1
RPL13	0	0	0	0	0	1	1	0	0	1	1	1	1
RPL14	0	0	0	0	0	1	1	0	0	1	1	1	1
RPL17	0	0	0	0	0	1	1	0	0	1	1	1	1
RPL18	0	0	0	0	0	1	1	0	0	1	1	1	1
RPL18A	0	0	0	0	0	1	1	0	0	1	1	1	1
RPL19	0	0	0	0	0	1	1	0	0	1	1	1	1
RPL23	0	0	0	0	0	1	1	0	0	1	1	1	1
RPL23A	0	0	0	0	0	1	1	0	0	1	1	1	1
RPL24	0	0	0	0	0	1	1	0	0	1	1	1	1
RPL26	0	0	0	0	0	1	1	0	0	1	1	1	1
RPL27	0	0	0	0	0	1	1	0	0	1	1	1	1
RPL27A	0	0	0	0	0	1	1	0	0	1	1	1	1
RPL28	0	0	0	0	0	1	1	0	0	1	1	1	1
RPL29	0	0	0	0	0	1	1	0	0	1	1	1	1
RPL30	0	0	0	0	0	1	1	0	0	1	1	1	1
RPL32	0	0	0	0	0	1	1	0	0	1	1	1	1
RPL34	0	0	0	0	0	1	1	0	0	1	1	1	1
RPL35A	0	0	0	0	0	1	1	0	0	1	1	1	1
RPL36	0	0	0	0	0	1	1	0	0	1	1	1	1
RPL36A	0	0	0	0	0	0	0	0	0	1	1	1	1
RPL38	0	0	0	0	0	0	0	0	0	1	1	1	1
RPL4	0	0	0	0	0	0	0	0	0	1	1	1	1
RPL7A	0	0	0	0	0	1	1	0	0	1	1	1	1
RPL8	0	0	0	0	0	1	1	0	0	1	1	1	1
RPLP0	0	0	0	0	0	1	1	0	0	1	1	1	1
RPLP1	0	0	0	0	0	1	1	0	0	1	1	1	1
RPLP2	0	0	0	0	0	1	1	0	0	1	1	1	1
RPS11	0	0	0	0	0	1	1	1	1	1	1	1	1
RPS12	0	0	0	0	0	0	0	1	1	1	1	1	1
RPS13	0	0	0	0	0	1	1	1	1	1	1	1	1
RPS15A	0	0	0	0	0	0	0	1	1	1	1	1	1
RPS16	0	0	0	0	0	1	1	1	1	1	1	1	1
RPS17	0	0	0	0	0	1	1	1	1	1	1	1	1
RPS18	0	0	0	0	0	1	1	1	1	1	1	1	1
RPS19	0	0	0	0	0	1	1	1	1	1	1	1	1
RPS2	0	0	0	0	0	1	1	1	1	1	1	1	1
RPS21	0	0	0	0	0	1	1	1	1	1	1	1	1
RPS23	0	0	0	0	0	1	1	1	1	1	1	1	1
RPS24	0	0	0	0	0	1	1	1	1	1	1	1	1

NAME	REACTOME_3_UTR_MEDIATED_TRANSLATIONAL_REGULATION	REACTOME_TRANSLATION	REACTOME_CELL_SURFACE_INTERACTIONS_AT_THE_VASCULAR_WALL	REACTOME_INTEGRIN_CELL_SURFACE_INTERACTIONS	REACTOME_RESPONSE_TO_ELEVATED_PLATELET_CYTOSOLIC_CA2_	REACTOME_HEMOSTASIS	REACTOME_REGULATION_OF_INSULIN_SECRETION	REACTOME_INTEGRATION_OF_ENERGY_METABOLISM	REACTOME_GPCR_DOWNSTREAM_ING	REACTOME_ING_BY_GPCR	REACTOME_ING_BY_ILS	REACTOME_ING_BY_FGFR_IN_DISEASE	REACTOME_ING_BY_ERBB2
PSMF1	0	0	0	0	0	0	0	0	0	0	0	0	0
PTPN1	0	0	0	0	0	0	0	0	0	0	0	0	0
RAB5C	0	0	0	0	0	0	0	0	0	0	0	0	0
RAC1	0	0	0	0	0	0	0	0	1	1	0	0	0
RAP1A	0	0	0	0	0	1	0	1	0	0	0	0	0
RASA1	0	0	0	0	0	0	0	0	0	0	0	0	0
RDX	0	0	0	0	0	0	0	0	0	0	0	0	0
RELA	0	0	0	0	0	0	0	0	0	0	1	0	0
ROCK1	0	0	0	0	0	0	0	0	0	1	0	0	0
ROCK2	0	0	0	0	0	0	0	0	1	1	0	0	0
RPL10A	1	1	0	0	0	0	0	0	0	0	0	0	0
RPL11	1	0	0	0	0	0	0	0	0	0	0	0	0
RPL12	1	1	0	0	0	0	0	0	0	0	0	0	0
RPL13	1	1	0	0	0	0	0	0	0	0	0	0	0
RPL14	1	1	0	0	0	0	0	0	0	0	0	0	0
RPL17	1	1	0	0	0	0	0	0	0	0	0	0	0
RPL18	1	1	0	0	0	0	0	0	0	0	0	0	0
RPL18A	1	1	0	0	0	0	0	0	0	0	0	0	0
RPL19	1	1	0	0	0	0	0	0	0	0	0	0	0
RPL23	1	1	0	0	0	0	0	0	0	0	0	0	0
RPL23A	1	1	0	0	0	0	0	0	0	0	0	0	0
RPL24	1	1	0	0	0	0	0	0	0	0	0	0	0
RPL26	1	1	0	0	0	0	0	0	0	0	0	0	0
RPL27	1	1	0	0	0	0	0	0	0	0	0	0	0
RPL27A	1	1	0	0	0	0	0	0	0	0	0	0	0
RPL28	1	1	0	0	0	0	0	0	0	0	0	0	0
RPL29	1	1	0	0	0	0	0	0	0	0	0	0	0
RPL30	1	1	0	0	0	0	0	0	0	0	0	0	0
RPL32	1	1	0	0	0	0	0	0	0	0	0	0	0
RPL34	1	1	0	0	0	0	0	0	0	0	0	0	0
RPL35A	1	1	0	0	0	0	0	0	0	0	0	0	0
RPL36	1	1	0	0	0	0	0	0	0	0	0	0	0
RPL36A	1	0	0	0	0	0	0	0	0	0	0	0	0
RPL38	1	0	0	0	0	0	0	0	0	0	0	0	0
RPL4	1	1	0	0	0	0	0	0	0	0	0	0	0
RPL7A	1	1	0	0	0	0	0	0	0	0	0	0	0
RPL8	1	1	0	0	0	0	0	0	0	0	0	0	0
RPLP0	1	1	0	0	0	0	0	0	0	0	0	0	0
RPLP1	1	1	0	0	0	0	0	0	0	0	0	0	0
RPLP2	1	1	0	0	0	0	0	0	0	0	0	0	0
RPS11	1	1	0	0	0	0	0	0	0	0	0	0	0
RPS12	1	1	0	0	0	0	0	0	0	0	0	0	0
RPS13	1	1	0	0	0	0	0	0	0	0	0	0	0
RPS15A	1	1	0	0	0	0	0	0	0	0	0	0	0
RPS16	1	1	0	0	0	0	0	0	0	0	0	0	0
RPS17	1	1	0	0	0	0	0	0	0	0	0	0	0
RPS18	1	1	0	0	0	0	0	0	0	0	0	0	0
RPS19	1	1	0	0	0	0	0	0	0	0	0	0	0
RPS2	1	1	0	0	0	0	0	0	0	0	0	0	0
RPS21	1	1	0	0	0	0	0	0	0	0	0	0	0
RPS23	1	1	0	0	0	0	0	0	0	0	0	0	0
RPS24	1	1	0	0	0	0	0	0	0	0	0	0	0

NAME	REACTOME_ING_BY_FGFR	REACTOME_DOWNSTREAM__TRANSDUCTION	REACTOME_LING_BY_NGF	REACTOME_NGF_LING_VIA_TRKA_FROM_THE_PLASMA_MEMBRANE	REACTOME_ING_BY_EGFR_IN_CANCER	REACTOME_ING_BY_PDGF	REACTOME_SEMAPHORIN_INTERACTIONS	REACTOME_L1CAM_INTERACTIONS	REACTOME_DEVELOPMENTAL_BIOLOGY	REACTOME_AXON_GUIDANCE	KEGG_ERBB_ING_PATHWAY	KEGG_INSULIN_ING_PATHWAY
PSMF1	0	0	0	0	0	0	0	0	0	0	0	0
PTPN1	0	0	0	0	0	0	0	0	0	0	0	1
RAB5C	0	0	0	0	0	0	0	0	0	0	0	0
RAC1	0	0	1	0	0	0	1	1	1	1	0	0
RAP1A	0	0	1	1	0	0	0	0	0	0	0	0
RASA1	0	1	0	0	0	1	0	0	0	0	0	0
RDX	0	0	0	0	0	0	0	1	1	1	0	0
RELA	0	0	1	0	0	0	0	0	1	0	0	0
ROCK1	0	0	0	0	0	0	0	0	0	0	0	0
ROCK2	0	0	0	0	0	0	1	0	1	1	0	0
RPL10A	0	0	0	0	0	0	0	0	0	0	0	0
RPL11	0	0	0	0	0	0	0	0	0	0	0	0
RPL12	0	0	0	0	0	0	0	0	0	0	0	0
RPL13	0	0	0	0	0	0	0	0	0	0	0	0
RPL14	0	0	0	0	0	0	0	0	0	0	0	0
RPL17	0	0	0	0	0	0	0	0	0	0	0	0
RPL18	0	0	0	0	0	0	0	0	0	0	0	0
RPL18A	0	0	0	0	0	0	0	0	0	0	0	0
RPL19	0	0	0	0	0	0	0	0	0	0	0	0
RPL23	0	0	0	0	0	0	0	0	0	0	0	0
RPL23A	0	0	0	0	0	0	0	0	0	0	0	0
RPL24	0	0	0	0	0	0	0	0	0	0	0	0
RPL26	0	0	0	0	0	0	0	0	0	0	0	0
RPL27	0	0	0	0	0	0	0	0	0	0	0	0
RPL27A	0	0	0	0	0	0	0	0	0	0	0	0
RPL28	0	0	0	0	0	0	0	0	0	0	0	0
RPL29	0	0	0	0	0	0	0	0	0	0	0	0
RPL30	0	0	0	0	0	0	0	0	0	0	0	0
RPL32	0	0	0	0	0	0	0	0	0	0	0	0
RPL34	0	0	0	0	0	0	0	0	0	0	0	0
RPL35A	0	0	0	0	0	0	0	0	0	0	0	0
RPL36	0	0	0	0	0	0	0	0	0	0	0	0
RPL36A	0	0	0	0	0	0	0	0	0	0	0	0
RPL38	0	0	0	0	0	0	0	0	0	0	0	0
RPL4	0	0	0	0	0	0	0	0	0	0	0	0
RPL7A	0	0	0	0	0	0	0	0	0	0	0	0
RPL8	0	0	0	0	0	0	0	0	0	0	0	0
RPLP0	0	0	0	0	0	0	0	0	0	0	0	0
RPLP1	0	0	0	0	0	0	0	0	0	0	0	0
RPLP2	0	0	0	0	0	0	0	0	0	0	0	0
RPS11	0	0	0	0	0	0	0	0	0	0	0	0
RPS12	0	0	0	0	0	0	0	0	0	0	0	0
RPS13	0	0	0	0	0	0	0	0	0	0	0	0
RPS15A	0	0	0	0	0	0	0	0	0	0	0	0
RPS16	0	0	0	0	0	0	0	0	0	0	0	0
RPS17	0	0	0	0	0	0	0	0	0	0	0	0
RPS18	0	0	0	0	0	0	0	0	0	0	0	0
RPS19	0	0	0	0	0	0	0	0	0	0	0	0
RPS2	0	0	0	0	0	0	0	0	0	0	0	0
RPS21	0	0	0	0	0	0	0	0	0	0	0	0
RPS23	0	0	0	0	0	0	0	0	0	0	0	0
RPS24	0	0	0	0	0	0	0	0	0	0	0	0

NAME	KEGG_PATHWAYS_IN_CANCER	KEGG_PROSTATE_CANCER	KEGG_CHEMOKINE_ING_PATHWAY	KEGG_NEUROTROPHIN_ING_PATHWAY	KEGG_MAPK_ING_PATHWAY	KEGG_ARRHYTHMOGENIC_RIGHT_VENTRICULAR_CARDIOMYOPATHY_ARVC	KEGG_ECM_RECEPTOR_INTERACTION	KEGG_FOCAL_ADHESION
PSMF1	0	0	0	0	0	0	0	0
PTPN1	0	0	0	0	0	0	0	0
RAB5C	0	0	0	0	0	0	0	0
RAC1	1	0	1	1	1	0	0	0
RAP1A	0	0	1	1	1	0	0	0
RASA1	0	0	0	0	1	0	0	0
RDX	0	0	0	0	0	0	0	0
RELA	1	1	1	1	1	0	0	0
ROCK1	0	0	0	0	0	0	0	0
ROCK2	0	0	1	0	0	0	0	1
RPL10A	0	0	0	0	0	0	0	0
RPL11	0	0	0	0	0	0	0	0
RPL12	0	0	0	0	0	0	0	0
RPL13	0	0	0	0	0	0	0	0
RPL14	0	0	0	0	0	0	0	0
RPL17	0	0	0	0	0	0	0	0
RPL18	0	0	0	0	0	0	0	0
RPL18A	0	0	0	0	0	0	0	0
RPL19	0	0	0	0	0	0	0	0
RPL23	0	0	0	0	0	0	0	0
RPL23A	0	0	0	0	0	0	0	0
RPL24	0	0	0	0	0	0	0	0
RPL26	0	0	0	0	0	0	0	0
RPL27	0	0	0	0	0	0	0	0
RPL27A	0	0	0	0	0	0	0	0
RPL28	0	0	0	0	0	0	0	0
RPL29	0	0	0	0	0	0	0	0
RPL30	0	0	0	0	0	0	0	0
RPL32	0	0	0	0	0	0	0	0
RPL34	0	0	0	0	0	0	0	0
RPL35A	0	0	0	0	0	0	0	0
RPL36	0	0	0	0	0	0	0	0
RPL36A	0	0	0	0	0	0	0	0
RPL38	0	0	0	0	0	0	0	0
RPL4	0	0	0	0	0	0	0	0
RPL7A	0	0	0	0	0	0	0	0
RPL8	0	0	0	0	0	0	0	0
RPLP0	0	0	0	0	0	0	0	0
RPLP1	0	0	0	0	0	0	0	0
RPLP2	0	0	0	0	0	0	0	0
RPS11	0	0	0	0	0	0	0	0
RPS12	0	0	0	0	0	0	0	0
RPS13	0	0	0	0	0	0	0	0
RPS15A	0	0	0	0	0	0	0	0
RPS16	0	0	0	0	0	0	0	0
RPS17	0	0	0	0	0	0	0	0
RPS18	0	0	0	0	0	0	0	0
RPS19	0	0	0	0	0	0	0	0
RPS2	0	0	0	0	0	0	0	0
RPS21	0	0	0	0	0	0	0	0
RPS23	0	0	0	0	0	0	0	0
RPS24	0	0	0	0	0	0	0	0

Table S6 The differential proteins contribute to the enrichment of the significant pathways for the public dataset

Core enrichment proteins in down-regulated pathways of hESCs/HFs (hESCs vs HFs)

NAME	REACTOME_UNFOLDED_PROTEIN_RESPONSE	REACTOME_DIABETES_PATHWAYS	REACTOME_CELL_CELL_COMMUNICATION	REACTOME_GOLGI_ASSOCIATED_VESICLE_BIOGENESIS	REACTOME_TRANS_GOLGI_NETWORK_VESICLE_BUDDING	REACTOME_MEMBRANE_TRAFFICKING	REACTOME_NEURONAL_SYSTEM	REACTOME_TRANSMISSION_ACROSS_CHEMICAL_SYNAPSES	REACTOME_POST_TRANSLATIONAL_PROTEIN_MODIFICATION	REACTOME_ASPARAGINE_N_LINKED_GLYCOSYLATION	REACTOME_ING_BY_INSULIN_RECEPTOR	REACTOME_PREFOLDIN_MEDIATED_TRANSFER_OF_SUBSTRATE_TO_CCT_TRIC	REACTOME_PROTEIN_FOLDING
RPS27A	0	0	0	0	0	0	0	0	0	0	0	0	0
RPS28	0	0	0	0	0	0	0	0	0	0	0	0	0
RPS29	0	0	0	0	0	0	0	0	0	0	0	0	0
RPS3	0	0	0	0	0	0	0	0	0	0	0	0	0
RPS4X	0	0	0	0	0	0	0	0	0	0	0	0	0
RPS5	0	0	0	0	0	0	0	0	0	0	0	0	0
RPS6	0	0	0	0	0	0	0	0	0	0	1	0	0
RPS7	0	0	0	0	0	0	0	0	0	0	0	0	0
RPS8	0	0	0	0	0	0	0	0	0	0	0	0	0
RPS9	0	0	0	0	0	0	0	0	0	0	0	0	0
RRAS	0	0	0	0	0	0	1	1	0	0	0	0	0
SHC1	1	1	0	0	0	0	0	0	0	0	1	0	0
SRGAP1	0	0	0	0	0	0	0	0	0	0	0	0	0
STAM	0	0	0	0	0	1	0	0	0	0	0	0	0
STAT1	0	0	0	0	0	0	0	0	0	0	0	0	0
TCIRG1	0	0	0	0	0	0	0	0	0	0	1	0	0
TFRC	0	0	0	1	1	1	0	0	0	0	0	0	0
THBS1	0	0	0	0	0	0	0	0	0	0	0	0	0
TLN1	1	1	0	0	0	0	0	0	0	0	0	0	0
TUBA1C	0	0	0	0	0	0	0	0	0	0	0	1	1
TUBB3	0	0	0	0	0	0	0	0	0	0	0	1	1
TUBB6	0	0	0	0	0	0	0	0	0	0	0	1	1
VASP	0	0	0	0	0	0	0	0	0	0	0	0	0
VCL	0	0	0	0	0	0	0	0	0	0	0	0	0
VPS4A	0	0	0	0	0	1	0	0	0	0	0	0	0
VPS4B	0	0	0	0	0	1	0	0	0	0	0	0	0
YWHAB	0	0	0	0	0	0	0	0	0	0	1	0	0
YWHAZ	0	0	0	0	0	0	0	0	0	0	0	0	0
ABCC4	0	0	0	0	0	0	0	0	0	0	0	0	0
ACTR1A	0	0	0	0	0	0	0	0	0	0	0	0	0
ALG5	0	0	0	0	0	0	0	0	1	1	0	0	0
ANXA1	0	0	0	0	0	0	0	0	0	0	0	0	0
APPL1	0	0	0	0	0	0	0	0	0	0	0	0	0
ARCN1	0	0	0	0	0	1	0	0	0	0	0	0	0
ARHGEF17	0	0	0	0	0	0	0	0	0	0	0	0	0
ATP6V0C	0	0	0	0	0	0	0	0	0	0	1	0	0
ATP6V1A	0	0	0	0	0	0	0	0	0	0	1	0	0
B2M	0	0	0	0	0	0	0	0	0	0	0	0	0
BAX	0	0	0	0	0	0	0	0	0	0	0	0	0
BCAP31	0	0	0	0	0	0	0	0	0	0	0	0	0
C19orf10	1	1	0	0	0	0	0	0	0	0	0	0	0
CAB39	0	0	0	0	0	0	0	0	0	0	1	0	0
CALR	1	1	0	0	0	0	0	0	1	1	0	0	0
CALU	0	0	0	0	0	0	0	0	0	0	0	0	0
CAP1	0	0	0	0	0	0	0	0	0	0	0	0	0
CAPZA1	0	0	0	0	0	0	0	0	0	0	0	0	0
CAPZA2	0	0	0	0	0	0	0	0	0	0	0	0	0
CCT2	0	0	0	0	0	0	0	0	0	0	0	1	1
CCT3	0	0	0	0	0	0	0	0	0	0	0	1	1
CCT4	0	0	0	0	0	0	0	0	0	0	0	1	1
CCT5	0	0	0	0	0	0	0	0	0	0	0	1	1
CCT6A	0	0	0	0	0	0	0	0	0	0	0	1	1

NAME	REACTOME_INNATE_IMMUNE_SYSTEM	REACTOME_MHC_CLASS_II_ANTIGEN_PRESENTATION	REACTOME_IMMUNE_SYSTEM	REACTOME_ADAPTIVE_IMMUNE_SYSTEM	REACTOME_APOPTOSIS	REACTOME_CLASS_I_MHC_MEDIATED_ANTIGEN_PROCESSING_PRESENTATION	REACTOME_ANTIGEN_PROCESSING_UBIQUITINATION_PROTEASOME_DEGRADATION	REACTOME_ER_PHAGOSOME_PATHWAY	REACTOME_ANTIGEN_PROCESSING_CROSS_PRESENTATION	REACTOME_REGULATION_OF_MRNA_STABILITY_BY_PROTEINS_THAT_BIND_AU_RICH_ELEMENTS	REACTOME_DESTABILIZATION_OF_MRNA_BY_AUF1_HNRNP_D0	REACTOME_REGULATION_OF_ORNITHINE_DECARBOXYLASE_ODC	REACTOME_REGULATION_OF_MITOTIC_CELL_CYCLE
RPS27A	0	0	1	1	1	1	1	1	1	1	1	0	1
RPS28	0	0	0	0	0	0	0	0	0	0	0	0	0
RPS29	0	0	0	0	0	0	0	0	0	0	0	0	0
RPS3	0	0	0	0	0	0	0	0	0	0	0	0	0
RPS4X	0	0	0	0	0	0	0	0	0	0	0	0	0
RPS5	0	0	0	0	0	0	0	0	0	0	0	0	0
RPS6	0	0	0	0	0	0	0	0	0	0	0	0	0
RPS7	0	0	0	0	0	0	0	0	0	0	0	0	0
RPS8	0	0	0	0	0	0	0	0	0	0	0	0	0
RPS9	0	0	0	0	0	0	0	0	0	0	0	0	0
RRAS	0	0	0	0	0	0	0	0	0	0	0	0	0
SHC1	0	0	1	1	0	0	0	0	0	0	0	0	0
SRGAP1	0	0	0	0	0	0	0	0	0	0	0	0	0
STAM	0	0	0	0	0	0	0	0	0	0	0	0	0
STAT1	0	0	1	0	0	0	0	0	0	0	0	0	0
TCIRG1	0	0	0	0	0	0	0	0	0	0	0	0	0
TFRC	0	0	0	0	0	0	0	0	0	0	0	0	0
THBS1	0	0	0	0	0	0	0	0	0	0	0	0	0
TLN1	0	0	0	0	0	0	0	0	0	0	0	0	0
TUBA1C	0	0	0	0	0	0	0	0	0	0	0	0	0
TUBB3	0	0	0	0	0	0	0	0	0	0	0	0	0
TUBB6	0	0	0	0	0	0	0	0	0	0	0	0	0
VASP	0	0	1	1	0	0	0	0	0	0	0	0	0
VCL	0	0	0	0	0	0	0	0	0	0	0	0	0
VPS4A	0	0	0	0	0	0	0	0	0	0	0	0	0
VPS4B	0	0	0	0	0	0	0	0	0	0	0	0	0
YWHAB	0	0	1	1	1	0	0	0	0	1	0	0	0
YWHAZ	0	0	1	1	0	0	0	0	0	1	0	0	0
ABCC4	0	0	0	0	0	0	0	0	0	0	0	0	0
ACTR1A	0	1	1	1	0	0	0	0	0	0	0	0	0
ALG5	0	0	0	0	0	0	0	0	0	0	0	0	0
ANXA1	0	0	0	0	0	0	0	0	0	0	0	0	0
APPL1	0	0	0	0	1	0	0	0	0	0	0	0	0
ARCN1	0	0	0	0	0	0	0	0	0	0	0	0	0
ARHGEF17	0	0	0	0	0	0	0	0	0	0	0	0	0
ATP6V0C	0	0	0	0	0	0	0	0	0	0	0	0	0
ATP6V1A	0	0	0	0	0	0	0	0	0	0	0	0	0
B2M	0	0	1	1	0	1	0	1	1	0	0	0	0
BAX	0	0	0	0	1	0	0	0	0	0	0	0	0
BCAP31	0	0	0	0	1	0	0	0	0	0	0	0	0
C19orf10	0	0	0	0	0	0	0	0	0	0	0	0	0
CAB39	0	0	0	0	0	0	0	0	0	0	0	0	0
CALR	0	0	1	1	0	1	0	1	1	0	0	0	0
CALU	0	0	0	0	0	0	0	0	0	0	0	0	0
CAP1	0	0	0	0	0	0	0	0	0	0	0	0	0
CAPZA1	1	0	1	0	0	0	0	0	0	0	0	0	0
CAPZA2	0	0	1	0	0	0	0	0	0	0	0	0	0
CCT2	0	0	0	0	0	0	0	0	0	0	0	0	0
CCT3	0	0	0	0	0	0	0	0	0	0	0	0	0
CCT4	0	0	0	0	0	0	0	0	0	0	0	0	0
CCT5	0	0	0	0	0	0	0	0	0	0	0	0	0
CCT6A	0	0	0	0	0	0	0	0	0	0	0	0	0

NAME	REACTOME_CDT1_ASSOCIATION_WITH_THE_CDC6_ORC_ORIGIN_COMPLEX	REACTOME_APC_C_CDC20_MEDIATED_DEGRADATION_OF_MITOTIC_PROTEINS	REACTOME_APC_C_CDH1_MEDIATED_DEGRADATION_OF_CDC20_AND_OTHER_APC_C_CDH1_TARGETED_PROTEINS_IN_LATE_MITOSIS_EARLY_G1	REACTOME_CYCLIN_E_ASSOCIATED_EVENTS_DURING_G1_S_TRANSITION_	REACTOME_SCFSKP2_MEDIATED_DEGRADATION_OF_P27_P21	REACTOME_AUTODEGRADATION_OF_CDH1_BY_CDH1_APC_C	REACTOME_SCF_BETA_TRCP_MEDIATED_DEGRADATION_OF_EMI1	REACTOME_P53_DEPENDENT_G1_DNA_DAMAGE_RESPONSE	REACTOME_CDK_MEDIATED_PHOSPHORYLATION_AND_REMOVAL_OF_CDC6	REACTOME_AUTODEGRADATION_OF_THE_E3_UBIQUITIN_LIGASE_COP1	REACTOME_P53_INDEPENDENT_G1_S_DNA_DAMAGE_CHECKPOINT	REACTOME_VIF_MEDIATED_DEGRADATION_OF_APOBEC3G	REACTOME_CROSS_PRESENTATION_OF_SOLUBLE_EXOGENOUS_ANTIGENS_ENDOSOMES
RPS27A	1	1	1	1	1	1	1	1	1	1	1	1	0
RPS28	0	0	0	0	0	0	0	0	0	0	0	0	0
RPS29	0	0	0	0	0	0	0	0	0	0	0	0	0
RPS3	0	0	0	0	0	0	0	0	0	0	0	0	0
RPS4X	0	0	0	0	0	0	0	0	0	0	0	0	0
RPS5	0	0	0	0	0	0	0	0	0	0	0	0	0
RPS6	0	0	0	0	0	0	0	0	0	0	0	0	0
RPS7	0	0	0	0	0	0	0	0	0	0	0	0	0
RPS8	0	0	0	0	0	0	0	0	0	0	0	0	0
RPS9	0	0	0	0	0	0	0	0	0	0	0	0	0
RRAS	0	0	0	0	0	0	0	0	0	0	0	0	0
SHC1	0	0	0	0	0	0	0	0	0	0	0	0	0
SRGAP1	0	0	0	0	0	0	0	0	0	0	0	0	0
STAM	0	0	0	0	0	0	0	0	0	0	0	0	0
STAT1	0	0	0	0	0	0	0	0	0	0	0	0	0
TCIRG1	0	0	0	0	0	0	0	0	0	0	0	0	0
TFRC	0	0	0	0	0	0	0	0	0	0	0	0	0
THBS1	0	0	0	0	0	0	0	0	0	0	0	0	0
TLN1	0	0	0	0	0	0	0	0	0	0	0	0	0
TUBA1C	0	0	0	0	0	0	0	0	0	0	0	0	0
TUBB3	0	0	0	0	0	0	0	0	0	0	0	0	0
TUBB6	0	0	0	0	0	0	0	0	0	0	0	0	0
VASP	0	0	0	0	0	0	0	0	0	0	0	0	0
VCL	0	0	0	0	0	0	0	0	0	0	0	0	0
VPS4A	0	0	0	0	0	0	0	0	0	0	0	0	0
VPS4B	0	0	0	0	0	0	0	0	0	0	0	0	0
YWHAB	0	0	0	0	0	0	0	0	0	0	0	0	0
YWHAZ	0	0	0	0	0	0	0	0	0	0	0	0	0
ABCC4	0	0	0	0	0	0	0	0	0	0	0	0	0
ACTR1A	0	0	0	0	0	0	0	0	0	0	0	0	0
ALG5	0	0	0	0	0	0	0	0	0	0	0	0	0
ANXA1	0	0	0	0	0	0	0	0	0	0	0	0	0
APPL1	0	0	0	0	0	0	0	0	0	0	0	0	0
ARCN1	0	0	0	0	0	0	0	0	0	0	0	0	0
ARHGEF17	0	0	0	0	0	0	0	0	0	0	0	0	0
ATP6V0C	0	0	0	0	0	0	0	0	0	0	0	0	0
ATP6V1A	0	0	0	0	0	0	0	0	0	0	0	0	0
B2M	0	0	0	0	0	0	0	0	0	0	0	0	0
BAX	0	0	0	0	0	0	0	0	0	0	0	0	0
BCAP31	0	0	0	0	0	0	0	0	0	0	0	0	0
C19orf10	0	0	0	0	0	0	0	0	0	0	0	0	0
CAB39	0	0	0	0	0	0	0	0	0	0	0	0	0
CALR	0	0	0	0	0	0	0	0	0	0	0	0	0
CALU	0	0	0	0	0	0	0	0	0	0	0	0	0
CAP1	0	0	0	0	0	0	0	0	0	0	0	0	0
CAPZA1	0	0	0	0	0	0	0	0	0	0	0	0	0
CAPZA2	0	0	0	0	0	0	0	0	0	0	0	0	0
CCT2	0	0	0	0	0	0	0	0	0	0	0	0	0
CCT3	0	0	0	0	0	0	0	0	0	0	0	0	0
CCT4	0	0	0	0	0	0	0	0	0	0	0	0	0
CCT5	0	0	0	0	0	0	0	0	0	0	0	0	0
CCT6A	0	0	0	0	0	0	0	0	0	0	0	0	0

NAME	REACTOME_ING_BY_WNT	REACTOME_REGULATION_OF_APOPTOSIS	REACTOME_ACTIVATION_OF_NF_KAPPAB_IN_B_CELLS	REACTOME_DOWNSTREAM_ING_EVENTS_OF_B_CELL_RECEPTOR_BCR	REACTOME_ING_BY_THE_B_CELL_RECEPTOR_BCR	REACTOME_METABOLISM_OF_MRNA	REACTOME_METABOLISM_OF_PROTEINS	REACTOME_FORMATION_OF_THE_TERNARY_COMPLEX_AND_SUBSEQUENTLY_THE_43S_COMPLEX	REACTOME_ACTIVATION_OF_THE_MRNA_UPON_BINDING_OF_THE_CAP_BINDING_COMPLEX_AND_EIFS_AND_SUBSEQUENT_BINDING_TO_43S	REACTOME_SRP_DEPENDENT_COTRANSLATIONAL_PROTEIN_TARGETING_TO_MEMBRANE	REACTOME_INFLUENZA_VIRAL_RNA_TRANSCRIPTION_AND_REPLICATION	REACTOME_NONSENSE_MEDIATED_DECAY_ENHANCED_BY_THE_EXON_JUNCTION_COMPLEX	REACTOME_PEPTIDE_CHAIN_ELONGATION
RPS27A	1	1	1	1	1	1	1	1	1	1	1	1	1
RPS28	0	0	0	0	0	1	1	1	1	1	1	1	1
RPS29	0	0	0	0	0	1	1	1	1	1	1	1	1
RPS3	0	0	0	0	0	1	1	1	1	1	1	1	1
RPS4X	0	0	0	0	0	1	1	1	1	1	1	1	1
RPS5	0	0	0	0	0	0	0	1	1	1	1	1	1
RPS6	0	0	0	0	0	1	1	1	1	1	1	1	1
RPS7	0	0	0	0	0	1	1	1	1	1	1	1	1
RPS8	0	0	0	0	0	1	1	1	1	1	1	1	1
RPS9	0	0	0	0	0	1	1	1	1	1	1	1	1
RRAS	0	0	0	0	0	0	0	0	0	0	0	0	0
SHC1	0	0	0	0	1	0	0	0	0	0	0	0	0
SRGAP1	0	0	0	0	0	0	0	0	0	0	0	0	0
STAM	0	0	0	0	0	0	0	0	0	0	0	0	0
STAT1	0	0	0	0	0	0	0	0	0	0	0	0	0
TCIRG1	0	0	0	0	0	0	0	0	0	0	0	0	0
TFRC	0	0	0	0	0	0	0	0	0	0	0	0	0
THBS1	0	0	0	0	0	0	0	0	0	0	0	0	0
TLN1	0	0	0	0	0	0	0	0	0	0	0	0	0
TUBA1C	0	0	0	0	0	0	1	0	0	0	0	0	0
TUBB3	0	0	0	0	0	0	1	0	0	0	0	0	0
TUBB6	0	0	0	0	0	0	1	0	0	0	0	0	0
VASP	0	0	0	0	0	0	0	0	0	0	0	0	0
VCL	0	0	0	0	0	0	0	0	0	0	0	0	0
VPS4A	0	0	0	0	0	0	0	0	0	0	0	0	0
VPS4B	0	0	0	0	0	0	0	0	0	0	0	0	0
YWHAB	0	0	0	0	0	1	0	0	0	0	0	0	0
YWHAZ	0	0	0	0	0	1	0	0	0	0	0	0	0
ABCC4	0	0	0	0	0	0	0	0	0	0	0	0	0
ACTR1A	0	0	0	0	0	0	0	0	0	0	0	0	0
ALG5	0	0	0	0	0	0	1	0	0	0	0	0	0
ANXA1	0	0	0	0	0	0	0	0	0	0	0	0	0
APPL1	0	1	0	0	0	0	0	0	0	0	0	0	0
ARCN1	0	0	0	0	0	0	0	0	0	0	0	0	0
ARHGEF17	0	0	0	0	0	0	0	0	0	0	0	0	0
ATP6V0C	0	0	0	0	0	0	0	0	0	0	0	0	0
ATP6V1A	0	0	0	0	0	0	0	0	0	0	0	0	0
B2M	0	0	0	0	0	0	0	0	0	0	0	0	0
BAX	0	0	0	0	0	0	0	0	0	0	0	0	0
BCAP31	0	0	0	0	0	0	0	0	0	0	0	0	0
C19orf10	0	0	0	0	0	0	0	0	0	0	0	0	0
CAB39	0	0	0	0	0	0	0	0	0	0	0	0	0
CALR	0	0	0	0	0	0	1	0	0	0	0	0	0
CALU	0	0	0	0	0	0	0	0	0	0	0	0	0
CAP1	0	0	0	0	0	0	0	0	0	0	0	0	0
CAPZA1	0	0	0	0	0	0	0	0	0	0	0	0	0
CAPZA2	0	0	0	0	0	0	0	0	0	0	0	0	0
CCT2	0	0	0	0	0	0	1	0	0	0	0	0	0
CCT3	0	0	0	0	0	0	1	0	0	0	0	0	0
CCT4	0	0	0	0	0	0	1	0	0	0	0	0	0
CCT5	0	0	0	0	0	0	1	0	0	0	0	0	0
CCT6A	0	0	0	0	0	0	1	0	0	0	0	0	0

NAME	REACTOME_3_UTR_MEDIATED_TRANSLATIONAL_REGULATION	REACTOME_TRANSLATION	REACTOME_CELL_SURFACE_INTERACTIONS_AT_THE_VASCULAR_WALL	REACTOME_INTEGRIN_CELL_SURFACE_INTERACTIONS	REACTOME_RESPONSE_TO_ELEVATED_PLATELET_CYTOSOLIC_CA2_	REACTOME_HEMOSTASIS	REACTOME_REGULATION_OF_INSULIN_SECRETION	REACTOME_INTEGRATION_OF_ENERGY_METABOLISM	REACTOME_GPCR_DOWNSTREAM_ING	REACTOME_ING_BY_GPCR	REACTOME_ING_BY_ILS	REACTOME_ING_BY_FGFR_IN_DISEASE	REACTOME_ING_BY_ERBB2
RPS27A	1	1	0	0	0	0	0	0	0	0	0	0	0
RPS28	1	1	0	0	0	0	0	0	0	0	0	0	0
RPS29	1	1	0	0	0	0	0	0	0	0	0	0	0
RPS3	1	1	0	0	0	0	0	0	0	0	0	0	0
RPS4X	1	1	0	0	0	0	0	0	0	0	0	0	0
RPS5	1	1	0	0	0	0	0	0	0	0	0	0	0
RPS6	1	1	0	0	0	0	0	0	0	0	0	0	0
RPS7	1	1	0	0	0	0	0	0	0	0	0	0	0
RPS8	1	1	0	0	0	0	0	0	0	0	0	0	0
RPS9	1	1	0	0	0	0	0	0	0	0	0	0	0
RRAS	0	0	0	0	0	0	0	0	0	0	0	0	0
SHC1	0	0	1	1	0	1	0	0	0	0	1	1	1
SRGAP1	0	0	0	0	0	0	0	0	0	0	0	0	0
STAM	0	0	0	0	0	0	0	0	0	0	0	0	0
STAT1	0	0	0	0	0	0	0	0	0	0	1	1	0
TCIRG1	0	0	0	0	0	0	0	0	0	0	0	0	0
TFRC	0	0	0	0	0	0	0	0	0	0	0	0	0
THBS1	0	0	0	1	1	1	0	0	0	0	0	0	0
TLN1	0	0	0	1	1	1	0	0	0	0	0	0	0
TUBA1C	0	0	0	0	0	0	0	0	0	0	0	0	0
TUBB3	0	0	0	0	0	0	0	0	0	0	0	0	0
TUBB6	0	0	0	0	0	0	0	0	0	0	0	0	0
VASP	0	0	0	0	0	0	0	0	0	0	0	0	0
VCL	0	0	0	0	1	1	0	0	0	0	0	0	0
VPS4A	0	0	0	0	0	0	0	0	0	0	0	0	0
VPS4B	0	0	0	0	0	0	0	0	0	0	0	0	0
YWHAB	0	0	0	0	0	0	0	0	0	1	1	1	1
YWHAZ	0	0	0	0	0	0	0	0	0	0	1	0	0
ABCC4	0	0	0	0	1	1	0	0	0	0	0	0	0
ACTR1A	0	0	0	0	0	0	0	0	0	0	0	0	0
ALG5	0	0	0	0	0	0	0	0	0	0	0	0	0
ANXA1	0	0	0	0	0	0	0	0	1	1	0	0	0
APPL1	0	0	0	0	0	0	0	0	0	0	0	0	0
ARCN1	0	0	0	0	0	0	0	0	0	0	0	0	0
ARHGEF17	0	0	0	0	0	0	0	0	1	1	0	0	0
ATP6V0C	0	0	0	0	0	0	0	0	0	0	0	0	0
ATP6V1A	0	0	0	0	0	0	0	0	0	0	0	0	0
B2M	0	0	0	0	0	0	0	0	0	0	0	0	0
BAX	0	0	0	0	0	0	0	0	0	0	0	0	0
BCAP31	0	0	0	0	0	0	0	0	0	0	0	0	0
C19orf10	0	0	0	0	0	0	0	0	0	0	0	0	0
CAB39	0	0	0	0	0	0	0	0	0	0	0	0	0
CALR	0	0	0	0	0	0	0	0	0	0	0	0	0
CALU	0	0	0	0	1	1	0	0	0	0	0	0	0
CAP1	0	0	0	0	1	1	0	0	0	0	0	0	0
CAPZA1	0	0	0	0	0	1	0	0	0	0	0	0	0
CAPZA2	0	0	0	0	0	0	0	0	0	0	0	0	0
CCT2	0	0	0	0	0	0	0	0	0	0	0	0	0
CCT3	0	0	0	0	0	0	0	0	0	0	0	0	0
CCT4	0	0	0	0	0	0	0	0	0	0	0	0	0
CCT5	0	0	0	0	0	0	0	0	0	0	0	0	0
CCT6A	0	0	0	0	0	0	0	0	0	0	0	0	0

NAME	REACTOME_ING_BY_FGFR	REACTOME_DOWNSTREAM__TRANSDUCTION	REACTOME_LING_BY_NGF	REACTOME_NGF_LING_VIA_TRKA_FROM_THE_PLASMA_MEMBRANE	REACTOME_ING_BY_EGFR_IN_CANCER	REACTOME_ING_BY_PDGF	REACTOME_SEMAPHORIN_INTERACTIONS	REACTOME_L1CAM_INTERACTIONS	REACTOME_DEVELOPMENTAL_BIOLOGY	REACTOME_AXON_GUIDANCE	KEGG_ERBB_ING_PATHWAY	KEGG_INSULIN_ING_PATHWAY
RPS27A	0	0	1	0	1	0	0	0	0	0	0	0
RPS28	0	0	0	0	0	0	0	0	0	0	0	0
RPS29	0	0	0	0	0	0	0	0	0	0	0	0
RPS3	0	0	0	0	0	0	0	0	0	0	0	0
RPS4X	0	0	0	0	0	0	0	0	0	0	0	0
RPS5	0	0	0	0	0	0	0	0	0	0	0	0
RPS6	0	0	0	0	0	0	0	0	0	0	0	0
RPS7	0	0	0	0	0	0	0	0	0	0	0	0
RPS8	0	0	0	0	0	0	0	0	0	0	0	0
RPS9	0	0	0	0	0	0	0	0	0	0	0	0
RRAS	0	0	0	0	0	0	1	0	1	1	0	0
SHC1	1	0	1	1	1	0	0	0	0	0	1	1
SRGAP1	0	0	0	0	0	0	0	0	1	1	0	0
STAM	0	0	0	0	1	0	0	0	0	0	0	0
STAT1	0	1	0	0	0	1	0	0	0	0	0	0
TCIRG1	0	0	0	0	0	0	0	0	0	0	0	0
TFRC	0	0	0	0	0	0	0	0	0	0	0	0
THBS1	0	0	0	0	0	1	0	0	0	0	0	0
TLN1	0	0	0	0	0	0	1	0	1	1	0	0
TUBA1C	0	0	0	0	0	0	0	0	0	0	0	0
TUBB3	0	0	0	0	0	0	0	0	0	0	0	0
TUBB6	0	0	0	0	0	0	0	0	0	0	0	0
VASP	0	0	0	0	0	0	0	0	1	1	0	0
VCL	0	0	0	0	0	0	0	0	0	0	0	0
VPS4A	0	0	0	0	0	0	0	0	0	0	0	0
VPS4B	0	0	0	0	0	0	0	0	0	0	0	0
YWHAB	1	1	1	1	1	0	0	0	1	1	0	0
YWHAZ	0	0	0	0	0	0	0	0	0	0	0	0
ABCC4	0	0	0	0	0	0	0	0	0	0	0	0
ACTR1A	0	0	0	0	0	0	0	0	0	0	1	1
ALG5	0	0	0	0	0	0	0	0	0	0	0	0
ANXA1	0	0	0	0	0	0	0	0	0	0	0	0
APPL1	0	0	0	0	0	0	0	0	0	0	0	0
ARCN1	0	0	0	0	0	0	0	0	0	0	0	0
ARHGEF17	0	0	1	0	0	0	0	0	0	0	0	0
ATP6V0C	0	0	0	0	0	0	0	0	0	0	0	0
ATP6V1A	0	0	0	0	0	0	0	0	0	0	0	0
B2M	0	0	0	0	0	0	0	0	0	0	0	0
BAX	0	0	0	0	0	0	0	0	0	0	0	0
BCAP31	0	0	0	0	0	0	0	0	0	0	0	0
C19orf10	0	0	0	0	0	0	0	0	0	0	0	0
CAB39	0	0	0	0	0	0	0	0	0	0	0	0
CALR	0	0	0	0	0	0	0	0	0	0	0	0
CALU	0	0	0	0	0	0	0	0	0	0	0	0
CAP1	0	0	0	0	0	0	0	0	1	1	0	0
CAPZA1	0	0	0	0	0	0	0	0	0	0	0	0
CAPZA2	0	0	0	0	0	0	0	0	0	0	0	0
CCT2	0	0	0	0	0	0	0	0	0	0	0	0
CCT3	0	0	0	0	0	0	0	0	0	0	0	0
CCT4	0	0	0	0	0	0	0	0	0	0	0	0
CCT5	0	0	0	0	0	0	0	0	0	0	0	1
CCT6A	0	0	0	0	0	0	0	0	0	0	0	0

NAME	KEGG_PATHWAYS_IN_CANCER	KEGG_PROSTATE_CANCER	KEGG_CHEMOKINE_ING_PATHWAY	KEGG_NEUROTROPHIN_ING_PATHWAY	KEGG_MAPK_ING_PATHWAY	KEGG_ARRHYTHMOGENIC_RIGHT_VENTRICULAR_CARDIOMYOPATHY_ARVC	KEGG_ECM_RECEPTOR_INTERACTION	KEGG_FOCAL_ADHESION
RPS27A	0	0	0	0	0	0	0	0
RPS28	0	0	0	0	0	0	0	0
RPS29	0	0	0	0	0	0	0	0
RPS3	0	0	0	0	0	0	0	0
RPS4X	0	0	0	0	0	0	0	0
RPS5	0	0	0	0	0	0	0	0
RPS6	0	0	0	0	0	0	0	0
RPS7	0	0	0	0	0	0	0	0
RPS8	0	0	0	0	0	0	0	0
RPS9	0	0	0	0	0	0	0	0
RRAS	0	0	0	0	1	0	0	0
SHC1	0	0	1	1	0	0	0	1
SRGAP1	0	0	0	0	0	0	0	0
STAM	0	0	0	0	0	0	0	0
STAT1	1	0	1	0	0	0	0	0
TCIRG1	0	0	0	0	0	0	0	0
TFRC	0	0	0	0	0	0	0	0
THBS1	0	0	0	0	0	0	1	1
TLN1	0	0	0	0	0	0	0	1
TUBA1C	0	0	0	0	0	0	0	0
TUBB3	0	0	0	0	0	0	0	0
TUBB6	0	0	0	0	0	0	0	0
VASP	0	0	0	0	0	0	0	1
VCL	0	0	0	0	0	0	0	1
VPS4A	0	0	0	0	0	0	0	0
VPS4B	0	0	0	0	0	0	0	0
YWHAB	0	0	0	1	0	0	0	0
YWHAZ	0	0	0	1	0	0	0	0
ABCC4	0	0	0	0	0	0	0	0
ACTR1A	0	0	0	0	0	0	0	0
ALG5	0	0	0	0	0	0	0	0
ANXA1	0	0	0	0	0	0	0	0
APPL1	0	0	0	0	0	0	0	0
ARCN1	0	0	0	0	0	0	0	0
ARHGEF17	0	0	0	0	0	0	0	0
ATP6V0C	0	0	0	0	0	0	0	0
ATP6V1A	0	0	0	0	0	0	0	0
B2M	0	0	0	0	0	0	0	0
BAX	0	0	0	0	0	0	0	0
BCAP31	0	0	0	0	1	0	0	0
C19orf10	0	0	0	0	0	0	0	1
CAB39	0	0	0	0	0	0	0	0
CALR	0	0	0	0	0	0	0	0
CALU	0	0	0	0	0	0	0	0
CAP1	0	0	0	0	0	0	0	0
CAPZA1	0	0	0	0	0	0	0	0
CAPZA2	0	0	0	0	0	0	0	0
CCT2	0	0	0	0	0	0	0	0
CCT3	0	0	0	0	0	0	0	0
CCT4	0	0	0	0	0	0	0	0
CCT5	0	0	0	0	0	0	0	0
CCT6A	0	0	0	0	1	0	0	1

Table S6 The differential proteins contribute to the enrichment of the significant pathways for the public dataset

Core enrichment proteins in down-regulated pathways of hESCs/HFs (hESCs vs HFs)

NAME	REACTOME_UNFOLDED_PROTEIN_RESPONSE	REACTOME_DIABETES_PATHWAYS	REACTOME_CELL_CELL_COMMUNICATION	REACTOME_GOLGI_ASSOCIATED_VESICLE_BIOGENESIS	REACTOME_TRANS_GOLGI_NETWORK_VESICLE_BUDDING	REACTOME_MEMBRANE_TRAFFICKING	REACTOME_NEURONAL_SYSTEM	REACTOME_TRANSMISSION_ACROSS_CHEMICAL_SYNAPSES	REACTOME_POST_TRANSLATIONAL_PROTEIN_MODIFICATION	REACTOME_ASPARAGINE_N_LINKED_GLYCOSYLATION	REACTOME_ING_BY_INSULIN_RECEPTOR	REACTOME_PREFOLDIN_MEDIATED_TRANSFER_OF_SUBSTRATE_TO_CCT_TRIC	REACTOME_PROTEIN_FOLDING
CCT7	0	0	0	0	0	0	0	0	0	0	0	1	1
CCT8	0	0	0	0	0	0	0	0	0	0	0	1	1
CD151	0	0	1	0	0	0	0	0	0	0	0	0	0
CD81	0	0	0	0	0	0	0	0	0	0	0	0	0
CDC37	0	0	0	0	0	0	0	0	0	0	0	0	0
CNTNAP1	0	0	0	0	0	0	0	0	0	0	0	0	0
COPA	0	0	0	0	0	1	0	0	0	0	0	0	0
COPB1	0	0	0	0	0	1	0	0	0	0	0	0	0
COPB2	0	0	0	0	0	1	0	0	0	0	0	0	0
COPE	0	0	0	0	0	1	0	0	0	0	0	0	0
COPG	0	0	0	0	0	1	0	0	0	0	0	0	0
COPZ1	0	0	0	0	0	1	0	0	0	0	0	0	0
CSNK1A1	0	0	0	0	0	0	0	0	0	0	0	0	0
DAD1	0	0	0	0	0	0	0	0	0	0	0	0	0
DBNL	0	0	0	0	0	0	0	0	0	0	0	0	0
DCTN1	1	1	0	0	0	0	0	0	0	0	0	0	0
DCTN2	0	0	0	0	0	0	0	0	0	0	0	0	0
DDOST	0	0	0	0	0	0	0	0	0	0	0	0	0
DNAJB11	1	1	0	0	0	0	0	0	0	0	0	0	0
DNAJC3	1	1	0	0	0	0	0	0	0	0	0	0	0
DPM1	0	0	0	0	0	0	0	0	0	0	0	0	0
DPP4	0	0	0	0	0	0	0	0	0	0	0	0	0
DYNC1H1	0	0	0	0	0	0	0	0	0	0	0	0	0
EEF1A1	0	0	0	0	0	0	0	0	0	0	0	0	0
EEF1D	0	0	0	0	0	0	0	0	0	0	0	0	0
EEF1G	0	0	0	0	0	0	0	0	0	0	0	0	0
EEF2	0	0	0	0	0	0	0	0	0	0	0	0	0
EIF2B1	0	0	0	0	0	0	0	0	0	0	0	0	0
EIF2B2	0	0	0	0	0	0	0	0	0	0	0	0	0
EIF2B5	0	0	0	0	0	0	0	0	0	0	0	0	0
EIF2S1	0	0	0	0	0	0	0	0	0	0	0	0	0
EIF2S2	0	0	0	0	0	0	0	0	0	0	0	0	0
EIF2S3	0	0	0	0	0	0	0	0	0	0	0	0	0
EIF3A	0	0	0	0	0	0	0	0	0	0	0	0	0
EIF3B	0	0	0	0	0	0	0	0	0	0	0	0	0
EIF3D	0	0	0	0	0	0	0	0	0	0	0	0	0
EIF3E	0	0	0	0	0	0	0	0	0	0	0	0	0
EIF3F	0	0	0	0	0	0	0	0	0	0	0	0	0
EIF3G	0	0	0	0	0	0	0	0	0	0	0	0	0
EIF3H	0	0	0	0	0	0	0	0	0	0	0	0	0
EIF3I	0	0	0	0	0	0	0	0	0	0	0	0	0
EIF3J	0	0	0	0	0	0	0	0	0	0	0	0	0
EIF3K	0	0	0	0	0	0	0	0	0	0	0	0	0
EIF4A1	0	0	0	0	0	0	0	0	0	0	0	0	0
EIF4B	0	0	0	0	0	0	0	0	0	0	1	0	0
EIF4G2	0	0	0	0	0	0	0	0	0	0	0	0	0
EIF4G3	0	0	0	0	0	0	0	0	0	0	0	0	0
EIF4H	0	0	0	0	0	0	0	0	0	0	0	0	0
EIF5	0	0	0	0	0	0	0	0	0	0	0	0	0
EIF5B	0	0	0	0	0	0	0	0	0	0	0	0	0
EPS15L1	0	0	0	0	0	0	0	0	0	0	0	0	0
ERAP1	0	0	0	0	0	0	0	0	0	0	0	0	0

NAME	REACTOME_INNATE_IMMUNE_SYSTEM	REACTOME_MHC_CLASS_II_ANTIGEN_PRESENTATION	REACTOME_IMMUNE_SYSTEM	REACTOME_ADAPTIVE_IMMUNE_SYSTEM	REACTOME_APOPTOSIS	REACTOME_CLASS_I_MHC_MEDIATED_ANTIGEN_PROCESSING_PRESENTATION	REACTOME_ANTIGEN_PROCESSING_UBIQUITINATION_PROTEASOME_DEGRADATION	REACTOME_ER_PHAGOSOME_PATHWAY	REACTOME_ANTIGEN_PROCESSING_CROSS_PRESENTATION	REACTOME_REGULATION_OF_MRNA_STABILITY_BY_PROTEINS_THAT_BIND_AU_RICH_ELEMENTS	REACTOME_DESTABILIZATION_OF_MRNA_BY_AUF1_HNRNP_D0	REACTOME_REGULATION_OF_ORNITHINE_DECARBOXYLASE_ODC	REACTOME_REGULATION_OF_MITOTIC_CELL_CYCLE
CCT7	0	0	0	0	0	0	0	0	0	0	0	0	0
CCT8	0	0	0	0	0	0	0	0	0	0	0	0	0
CD151	0	0	0	0	0	0	0	0	0	0	0	0	0
CD81	0	0	1	1	0	0	0	0	0	0	0	0	0
CDC37	0	0	0	0	0	0	0	0	0	0	0	0	0
CNTNAP1	0	0	0	0	0	0	0	0	0	0	0	0	0
COPA	0	0	0	0	0	0	0	0	0	0	0	0	0
COPB1	0	0	0	0	0	0	0	0	0	0	0	0	0
COPB2	0	0	0	0	0	0	0	0	0	0	0	0	0
COPE	0	0	0	0	0	0	0	0	0	0	0	0	0
COPG	0	0	0	0	0	0	0	0	0	0	0	0	0
COPZ1	0	0	0	0	0	0	0	0	0	0	0	0	0
CSNK1A1	0	0	0	0	0	0	0	0	0	0	0	0	0
DAD1	0	0	0	0	0	0	0	0	0	0	0	0	0
DBNL	0	0	0	0	1	0	0	0	0	0	0	0	0
DCTN1	0	1	1	1	0	0	0	0	0	0	0	0	0
DCTN2	0	1	1	1	0	0	0	0	0	0	0	0	0
DDOST	0	0	0	0	0	0	0	0	0	0	0	0	0
DNAJB11	0	0	0	0	0	0	0	0	0	0	0	0	0
DNAJC3	0	0	0	0	0	0	0	0	0	0	0	0	0
DPM1	0	0	0	0	0	0	0	0	0	0	0	0	0
DPP4	0	0	0	0	0	0	0	0	0	0	0	0	0
DYNC1H1	0	1	1	1	0	0	0	0	0	0	0	0	0
EEF1A1	0	0	0	0	0	0	0	0	0	0	0	0	0
EEF1D	0	0	0	0	0	0	0	0	0	0	0	0	0
EEF1G	0	0	0	0	0	0	0	0	0	0	0	0	0
EEF2	0	0	0	0	0	0	0	0	0	0	0	0	0
EIF2B1	0	0	0	0	0	0	0	0	0	0	0	0	0
EIF2B2	0	0	0	0	0	0	0	0	0	0	0	0	0
EIF2B5	0	0	0	0	0	0	0	0	0	0	0	0	0
EIF2S1	0	0	0	0	0	0	0	0	0	0	0	0	0
EIF2S2	0	0	0	0	0	0	0	0	0	0	0	0	0
EIF2S3	0	0	0	0	0	0	0	0	0	0	0	0	0
EIF3A	0	0	0	0	0	0	0	0	0	0	0	0	0
EIF3B	0	0	0	0	0	0	0	0	0	0	0	0	0
EIF3D	0	0	0	0	0	0	0	0	0	0	0	0	0
EIF3E	0	0	0	0	0	0	0	0	0	0	0	0	0
EIF3F	0	0	0	0	0	0	0	0	0	0	0	0	0
EIF3G	0	0	0	0	0	0	0	0	0	0	0	0	0
EIF3H	0	0	0	0	0	0	0	0	0	0	0	0	0
EIF3I	0	0	0	0	0	0	0	0	0	0	0	0	0
EIF3J	0	0	0	0	0	0	0	0	0	0	0	0	0
EIF3K	0	0	0	0	0	0	0	0	0	0	0	0	0
EIF4A1	0	0	1	0	0	0	0	0	0	0	0	0	0
EIF4B	0	0	0	0	0	0	0	0	0	0	0	0	0
EIF4G2	0	0	1	0	0	0	0	0	0	0	0	0	0
EIF4G3	0	0	1	0	0	0	0	0	0	0	0	0	0
EIF4H	0	0	0	0	0	0	0	0	0	0	0	0	0
EIF5	0	0	0	0	0	0	0	0	0	0	0	0	0
EIF5B	0	0	0	0	0	0	0	0	0	0	0	0	0
EPS15L1	0	0	0	0	0	0	0	0	0	0	0	0	0
ERAP1	0	0	1	1	0	1	0	0	0	0	0	0	0

NAME	REACTOME_CDT1_ASSOCIATION_WITH_THE_CDC6_ORC_ORIGIN_COMPLEX	REACTOME_APC_C_CDC20_MEDIATED_DEGRADATION_OF_MITOTIC_PROTEINS	REACTOME_APC_C_CDH1_MEDIATED_DEGRADATION_OF_CDC20_AND_OTHER_APC_C_CDH1_TARGETED_PROTEINS_IN_LATE_MITOSIS_EARLY_G1	REACTOME_CYCLIN_E_ASSOCIATED_EVENTS_DURING_G1_S_TRANSITION_	REACTOME_SCFSKP2_MEDIATED_DEGRADATION_OF_P27_P21	REACTOME_AUTODEGRADATION_OF_CDH1_BY_CDH1_APC_C	REACTOME_SCF_BETA_TRCP_MEDIATED_DEGRADATION_OF_EMI1	REACTOME_P53_DEPENDENT_G1_DNA_DAMAGE_RESPONSE	REACTOME_CDK_MEDIATED_PHOSPHORYLATION_AND_REMOVAL_OF_CDC6	REACTOME_AUTODEGRADATION_OF_THE_E3_UBIQUITIN_LIGASE_COP1	REACTOME_P53_INDEPENDENT_G1_S_DNA_DAMAGE_CHECKPOINT	REACTOME_VIF_MEDIATED_DEGRADATION_OF_APOBEC3G	REACTOME_CROSS_PRESENTATION_OF_SOLUBLE_EXOGENOUS_ANTIGENS_ENDOSOMES
CCT7	0	0	0	0	0	0	0	0	0	0	0	0	0
CCT8	0	0	0	0	0	0	0	0	0	0	0	0	0
CD151	0	0	0	0	0	0	0	0	0	0	0	0	0
CD81	0	0	0	0	0	0	0	0	0	0	0	0	0
CDC37	0	0	0	0	0	0	0	0	0	0	0	0	0
CNTNAP1	0	0	0	0	0	0	0	0	0	0	0	0	0
COPA	0	0	0	0	0	0	0	0	0	0	0	0	0
COPB1	0	0	0	0	0	0	0	0	0	0	0	0	0
COPB2	0	0	0	0	0	0	0	0	0	0	0	0	0
COPE	0	0	0	0	0	0	0	0	0	0	0	0	0
COPG	0	0	0	0	0	0	0	0	0	0	0	0	0
COPZ1	0	0	0	0	0	0	0	0	0	0	0	0	0
CSNK1A1	0	0	0	0	0	0	0	0	0	0	0	0	0
DAD1	0	0	0	0	0	0	0	0	0	0	0	0	0
DBNL	0	0	0	0	0	0	0	0	0	0	0	0	0
DCTN1	0	0	0	0	0	0	0	0	0	0	0	0	0
DCTN2	0	0	0	0	0	0	0	0	0	0	0	0	0
DDOST	0	0	0	0	0	0	0	0	0	0	0	0	0
DNAJB11	0	0	0	0	0	0	0	0	0	0	0	0	0
DNAJC3	0	0	0	0	0	0	0	0	0	0	0	0	0
DPM1	0	0	0	0	0	0	0	0	0	0	0	0	0
DPP4	0	0	0	0	0	0	0	0	0	0	0	0	0
DYNC1H1	0	0	0	0	0	0	0	0	0	0	0	0	0
EEF1A1	0	0	0	0	0	0	0	0	0	0	0	0	0
EEF1D	0	0	0	0	0	0	0	0	0	0	0	0	0
EEF1G	0	0	0	0	0	0	0	0	0	0	0	0	0
EEF2	0	0	0	0	0	0	0	0	0	0	0	0	0
EIF2B1	0	0	0	0	0	0	0	0	0	0	0	0	0
EIF2B2	0	0	0	0	0	0	0	0	0	0	0	0	0
EIF2B5	0	0	0	0	0	0	0	0	0	0	0	0	0
EIF2S1	0	0	0	0	0	0	0	0	0	0	0	0	0
EIF2S2	0	0	0	0	0	0	0	0	0	0	0	0	0
EIF2S3	0	0	0	0	0	0	0	0	0	0	0	0	0
EIF3A	0	0	0	0	0	0	0	0	0	0	0	0	0
EIF3B	0	0	0	0	0	0	0	0	0	0	0	0	0
EIF3D	0	0	0	0	0	0	0	0	0	0	0	0	0
EIF3E	0	0	0	0	0	0	0	0	0	0	0	0	0
EIF3F	0	0	0	0	0	0	0	0	0	0	0	0	0
EIF3G	0	0	0	0	0	0	0	0	0	0	0	0	0
EIF3H	0	0	0	0	0	0	0	0	0	0	0	0	0
EIF3I	0	0	0	0	0	0	0	0	0	0	0	0	0
EIF3J	0	0	0	0	0	0	0	0	0	0	0	0	0
EIF3K	0	0	0	0	0	0	0	0	0	0	0	0	0
EIF4A1	0	0	0	0	0	0	0	0	0	0	0	0	0
EIF4B	0	0	0	0	0	0	0	0	0	0	0	0	0
EIF4G2	0	0	0	0	0	0	0	0	0	0	0	0	0
EIF4G3	0	0	0	0	0	0	0	0	0	0	0	0	0
EIF4H	0	0	0	0	0	0	0	0	0	0	0	0	0
EIF5	0	0	0	0	0	0	0	0	0	0	0	0	0
EIF5B	0	0	0	0	0	0	0	0	0	0	0	0	0
EPS15L1	0	0	0	0	0	0	0	0	0	0	0	0	0
ERAP1	0	0	0	0	0	0	0	0	0	0	0	0	0

NAME	REACTOME_ING_BY_WNT	REACTOME_REGULATION_OF_APOPTOSIS	REACTOME_ACTIVATION_OF_NF_KAPPAB_IN_B_CELLS	REACTOME_DOWNSTREAM_ING_EVENTS_OF_B_CELL_RECEPTOR_BCR	REACTOME_ING_BY_THE_B_CELL_RECEPTOR_BCR	REACTOME_METABOLISM_OF_MRNA	REACTOME_METABOLISM_OF_PROTEINS	REACTOME_FORMATION_OF_THE_TERNARY_COMPLEX_AND_SUBSEQUENTLY_THE_43S_COMPLEX	REACTOME_ACTIVATION_OF_THE_MRNA_UPON_BINDING_OF_THE_CAP_BINDING_COMPLEX_AND_EIFS_AND_SUBSEQUENT_BINDING_TO_43S	REACTOME_SRP_DEPENDENT_COTRANSLATIONAL_PROTEIN_TARGETING_TO_MEMBRANE	REACTOME_INFLUENZA_VIRAL_RNA_TRANSCRIPTION_AND_REPLICATION	REACTOME_NONSENSE_MEDIATED_DECAY_ENHANCED_BY_THE_EXON_JUNCTION_COMPLEX	REACTOME_PEPTIDE_CHAIN_ELONGATION
CCT7	0	0	0	0	0	0	1	0	0	0	0	0	0
CCT8	0	0	0	0	0	0	1	0	0	0	0	0	0
CD151	0	0	0	0	0	0	0	0	0	0	0	0	0
CD81	0	0	0	0	0	0	0	0	0	0	0	0	0
CDC37	0	0	0	0	0	0	0	0	0	0	0	0	0
CNTNAP1	0	0	0	0	0	0	0	0	0	0	0	0	0
COPA	0	0	0	0	0	0	0	0	0	0	0	0	0
COPB1	0	0	0	0	0	0	0	0	0	0	0	0	0
COPB2	0	0	0	0	0	0	0	0	0	0	0	0	0
COPE	0	0	0	0	0	0	0	0	0	0	0	0	0
COPG	0	0	0	0	0	0	0	0	0	0	0	0	0
COPZ1	0	0	0	0	0	0	0	0	0	0	0	0	0
CSNK1A1	1	0	0	0	0	0	0	0	0	0	0	0	0
DAD1	0	0	0	0	0	0	1	0	0	0	0	0	0
DBNL	0	0	0	0	0	0	0	0	0	0	0	0	0
DCTN1	0	0	0	0	0	0	0	0	0	0	0	0	0
DCTN2	0	0	0	0	0	0	0	0	0	0	0	0	0
DDOST	0	0	0	0	0	0	1	0	0	1	0	0	0
DNAJB11	0	0	0	0	0	0	0	0	0	0	0	0	0
DNAJC3	0	0	0	0	0	0	0	0	0	0	1	0	0
DPM1	0	0	0	0	0	0	1	0	0	0	0	0	0
DPP4	0	0	0	0	0	0	0	0	0	0	0	0	0
DYNC1H1	0	0	0	0	0	0	0	0	0	0	0	0	0
EEF1A1	0	0	0	0	0	0	1	0	0	0	0	0	1
EEF1D	0	0	0	0	0	0	1	0	0	0	0	0	0
EEF1G	0	0	0	0	0	0	1	0	0	0	0	0	0
EEF2	0	0	0	0	0	0	1	0	0	0	0	0	1
EIF2B1	0	0	0	0	0	0	1	0	0	0	0	0	0
EIF2B2	0	0	0	0	0	0	1	0	0	0	0	0	0
EIF2B5	0	0	0	0	0	0	1	0	0	0	0	0	0
EIF2S1	0	0	0	0	0	0	1	1	1	0	0	0	0
EIF2S2	0	0	0	0	0	0	1	1	1	0	0	0	0
EIF2S3	0	0	0	0	0	0	1	1	1	0	0	0	0
EIF3A	0	0	0	0	0	0	1	1	1	0	0	0	0
EIF3B	0	0	0	0	0	0	1	1	1	0	0	0	0
EIF3D	0	0	0	0	0	0	1	1	1	0	0	0	0
EIF3E	0	0	0	0	0	0	1	1	1	0	0	0	0
EIF3F	0	0	0	0	0	0	1	1	1	0	0	0	0
EIF3G	0	0	0	0	0	0	1	1	1	0	0	0	0
EIF3H	0	0	0	0	0	0	1	1	1	0	0	0	0
EIF3I	0	0	0	0	0	0	1	1	1	0	0	0	0
EIF3J	0	0	0	0	0	0	1	1	1	0	0	0	0
EIF3K	0	0	0	0	0	0	1	1	1	0	0	0	0
EIF4A1	0	0	0	0	0	1	1	0	1	0	0	0	0
EIF4B	0	0	0	0	0	1	1	0	1	0	0	0	0
EIF4G2	0	0	0	0	0	0	0	0	0	0	0	0	0
EIF4G3	0	0	0	0	0	0	0	0	0	0	0	0	0
EIF4H	0	0	0	0	0	0	1	0	1	0	0	0	0
EIF5	0	0	0	0	0	0	1	0	0	0	0	0	0
EIF5B	0	0	0	0	0	0	0	0	0	0	0	0	0
EPS15L1	0	0	0	0	0	0	0	0	0	0	0	0	0
ERAP1	0	0	0	0	0	0	0	0	0	0	0	0	0

NAME	REACTOME_3_UTR_MEDIATED_TRANSLATIONAL_REGULATION	REACTOME_TRANSLATION	REACTOME_CELL_SURFACE_INTERACTIONS_AT_THE_VASCULAR_WALL	REACTOME_INTEGRIN_CELL_SURFACE_INTERACTIONS	REACTOME_RESPONSE_TO_ELEVATED_PLATELET_CYTOSOLIC_CA2_	REACTOME_HEMOSTASIS	REACTOME_REGULATION_OF_INSULIN_SECRETION	REACTOME_INTEGRATION_OF_ENERGY_METABOLISM	REACTOME_GPCR_DOWNSTREAM_ING	REACTOME_ING_BY_GPCR	REACTOME_ING_BY_ILS	REACTOME_ING_BY_FGFR_IN_DISEASE	REACTOME_ING_BY_ERBB2
CCT7	0	0	0	0	0	0	0	0	0	0	0	0	0
CCT8	0	0	0	0	0	0	0	0	0	0	0	0	0
CD151	0	0	0	0	0	0	0	0	0	0	0	0	0
CD81	0	0	0	0	0	0	0	0	0	0	0	0	0
CDC37	0	0	0	0	0	0	0	0	0	0	0	0	1
CNTNAP1	0	0	0	0	0	0	0	0	0	0	0	0	0
COPA	0	0	0	0	0	0	0	0	0	0	0	0	0
COPB1	0	0	0	0	0	0	0	0	0	0	0	0	0
COPB2	0	0	0	0	0	0	0	0	0	0	0	0	0
COPE	0	0	0	0	0	0	0	0	0	0	0	0	0
COPG	0	0	0	0	0	0	0	0	0	0	0	0	0
COPZ1	0	0	0	0	0	0	0	0	0	0	0	0	0
CSNK1A1	0	0	0	0	0	0	0	0	0	0	0	0	0
DAD1	0	0	0	0	0	0	0	0	0	0	0	0	0
DBNL	0	0	0	0	0	0	0	0	0	0	0	0	0
DCTN1	0	0	0	0	0	0	0	0	0	0	0	0	0
DCTN2	0	0	0	0	0	0	0	0	0	0	0	0	0
DDOST	0	1	0	0	0	0	0	0	0	0	0	0	0
DNAJB11	0	0	0	0	0	0	0	0	0	0	0	0	0
DNAJC3	0	0	0	0	0	0	0	0	0	0	0	0	0
DPM1	0	0	0	0	0	0	0	0	0	0	0	0	0
DPP4	0	0	0	0	0	0	1	1	0	0	0	0	0
DYNC1H1	0	0	0	0	0	0	0	0	0	0	0	0	0
EEF1A1	0	1	0	0	0	0	0	0	0	0	0	0	0
EEF1D	0	1	0	0	0	0	0	0	0	0	0	0	0
EEF1G	0	1	0	0	0	0	0	0	0	0	0	0	0
EEF2	0	1	0	0	0	0	0	0	0	0	0	0	0
EIF2B1	0	1	0	0	0	0	0	0	0	0	0	0	0
EIF2B2	0	1	0	0	0	0	0	0	0	0	0	0	0
EIF2B5	0	1	0	0	0	0	0	0	0	0	0	0	0
EIF2S1	1	1	0	0	0	0	0	0	0	0	0	0	0
EIF2S2	1	1	0	0	0	0	0	0	0	0	0	0	0
EIF2S3	1	1	0	0	0	0	0	0	0	0	0	0	0
EIF3A	1	1	0	0	0	0	0	0	0	0	0	0	0
EIF3B	1	1	0	0	0	0	0	0	0	0	0	0	0
EIF3D	1	1	0	0	0	0	0	0	0	0	0	0	0
EIF3E	1	1	0	0	0	0	0	0	0	0	0	0	0
EIF3F	1	1	0	0	0	0	0	0	0	0	0	0	0
EIF3G	1	1	0	0	0	0	0	0	0	0	0	0	0
EIF3H	1	1	0	0	0	0	0	0	0	0	0	0	0
EIF3I	1	1	0	0	0	0	0	0	0	0	0	0	0
EIF3J	1	1	0	0	0	0	0	0	0	0	0	0	0
EIF3K	1	1	0	0	0	0	0	0	0	0	0	0	0
EIF4A1	1	1	0	0	0	0	0	0	0	0	0	0	0
EIF4B	1	1	0	0	0	0	0	0	0	0	0	0	0
EIF4G2	0	0	0	0	0	0	0	0	0	0	0	0	0
EIF4G3	0	0	0	0	0	0	0	0	0	0	0	0	0
EIF4H	1	1	0	0	0	0	0	0	0	0	0	0	0
EIF5	0	1	0	0	0	0	0	0	0	0	0	0	0
EIF5B	0	1	0	0	0	0	0	0	0	0	0	0	0
EPS15L1	0	0	0	0	0	0	0	0	0	0	0	0	0
ERAP1	0	0	0	0	0	0	0	0	0	0	0	0	0

NAME	REACTOME_ING_BY_FGFR	REACTOME_DOWNSTREAM__TRANSDUCTION	REACTOME_LING_BY_NGF	REACTOME_NGF_LING_VIA_TRKA_FROM_THE_PLASMA_MEMBRANE	REACTOME_ING_BY_EGFR_IN_CANCER	REACTOME_ING_BY_PDGF	REACTOME_SEMAPHORIN_INTERACTIONS	REACTOME_L1CAM_INTERACTIONS	REACTOME_DEVELOPMENTAL_BIOLOGY	REACTOME_AXON_GUIDANCE	KEGG_ERBB_ING_PATHWAY	KEGG_INSULIN_ING_PATHWAY
CCT7	0	0	0	0	0	0	0	0	0	0	0	0
CCT8	0	0	0	0	0	0	0	0	0	0	0	1
CD151	0	0	0	0	0	0	0	0	0	0	0	0
CD81	0	0	0	0	0	0	0	0	0	0	0	0
CDC37	0	0	0	0	1	0	0	0	0	0	0	0
CNTNAP1	0	0	0	0	0	0	0	1	1	1	0	0
COPA	0	0	0	0	0	0	0	0	0	0	0	0
COPB1	0	0	0	0	0	0	0	0	0	0	0	0
COPB2	0	0	0	0	0	0	0	0	0	0	0	0
COPE	0	0	0	0	0	0	0	0	0	0	0	0
COPG	0	0	0	0	0	0	0	0	0	0	0	0
COPZ1	0	0	0	0	0	0	0	0	0	0	0	0
CSNK1A1	0	0	0	0	0	0	0	0	0	0	0	0
DAD1	0	0	0	0	0	0	0	0	0	0	0	1
DBNL	0	0	0	0	0	0	0	0	0	0	0	0
DCTN1	0	0	0	0	0	0	0	0	0	0	0	0
DCTN2	0	0	0	0	0	0	0	0	0	0	0	0
DDOST	0	0	0	0	0	0	0	0	0	0	0	0
DNAJB11	0	0	0	0	0	0	0	0	0	0	0	0
DNAJC3	0	0	0	0	0	0	0	0	0	0	0	0
DPM1	0	0	0	0	0	0	0	0	0	0	0	0
DPP4	0	0	0	0	0	0	0	0	0	0	0	0
DYNC1H1	0	0	0	0	0	0	0	0	0	0	0	0
EEF1A1	0	0	0	0	0	0	0	0	0	0	0	0
EEF1D	0	0	0	0	0	0	0	0	0	0	0	0
EEF1G	0	0	0	0	0	0	0	0	0	0	0	0
EEF2	0	0	0	0	0	0	0	0	0	0	0	0
EIF2B1	0	0	0	0	0	0	0	0	0	0	0	0
EIF2B2	0	0	0	0	0	0	0	0	0	0	0	0
EIF2B5	0	0	0	0	0	0	0	0	0	0	0	0
EIF2S1	0	0	0	0	0	0	0	0	0	0	0	0
EIF2S2	0	0	0	0	0	0	0	0	0	0	0	0
EIF2S3	0	0	0	0	0	0	0	0	0	0	0	0
EIF3A	0	0	0	0	0	0	0	0	0	0	0	0
EIF3B	0	0	0	0	0	0	0	0	0	0	0	0
EIF3D	0	0	0	0	0	0	0	0	0	0	0	0
EIF3E	0	0	0	0	0	0	0	0	0	0	0	0
EIF3F	0	0	0	0	0	0	0	0	0	0	0	0
EIF3G	0	0	0	0	0	0	0	0	0	0	0	0
EIF3H	0	0	0	0	0	0	0	0	0	0	0	0
EIF3I	0	0	0	0	0	0	0	0	0	0	0	0
EIF3J	0	0	0	0	0	0	0	0	0	0	0	0
EIF3K	0	0	0	0	0	0	0	0	0	0	0	0
EIF4A1	0	0	0	0	0	0	0	0	0	0	0	0
EIF4B	0	0	0	0	0	0	0	0	0	0	0	0
EIF4G2	0	0	0	0	0	0	0	0	0	0	0	0
EIF4G3	0	0	0	0	0	0	0	0	0	0	0	0
EIF4H	0	0	0	0	0	0	0	0	0	0	0	0
EIF5	0	0	0	0	0	0	0	0	0	0	0	0
EIF5B	0	0	0	0	0	0	0	0	0	0	0	0
EPS15L1	0	0	0	0	1	0	0	0	0	0	0	0
ERAP1	0	0	0	0	0	0	0	0	0	0	0	0

NAME	KEGG_PATHWAYS_IN_CANCER	KEGG_PROSTATE_CANCER	KEGG_CHEMOKINE_ING_PATHWAY	KEGG_NEUROTROPHIN_ING_PATHWAY	KEGG_MAPK_ING_PATHWAY	KEGG_ARRHYTHMOGENIC_RIGHT_VENTRICULAR_CARDIOMYOPATHY_ARVC	KEGG_ECM_RECEPTOR_INTERACTION	KEGG_FOCAL_ADHESION
CCT7	0	0	0	0	0	0	0	0
CCT8	0	0	0	0	0	0	0	0
CD151	0	0	0	0	0	0	0	0
CD81	0	0	0	0	0	0	1	0
CDC37	0	0	0	0	0	0	0	0
CNTNAP1	0	0	0	0	0	0	0	0
COPA	0	0	0	0	0	0	0	0
COPB1	0	0	0	0	0	0	0	0
COPB2	0	0	0	0	0	0	0	0
COPE	0	0	0	0	0	0	0	0
COPG	0	0	0	0	0	0	0	0
COPZ1	0	0	0	0	0	0	0	0
CSNK1A1	0	0	0	0	0	0	0	0
DAD1	0	0	0	0	0	0	0	0
DBNL	0	0	0	0	0	0	0	0
DCTN1	0	0	0	0	0	0	0	0
DCTN2	0	0	0	0	0	0	0	0
DDOST	0	0	0	0	0	0	0	0
DNAJB11	0	0	0	0	0	0	0	0
DNAJC3	0	0	0	0	0	0	0	0
DPM1	0	0	0	0	0	0	0	0
DPP4	0	0	0	0	0	0	0	0
DYNC1H1	0	0	0	0	0	0	0	0
EEF1A1	1	0	0	0	0	0	0	0
EEF1D	0	0	0	0	0	0	0	0
EEF1G	0	0	0	1	0	0	0	0
EEF2	0	0	0	0	0	0	0	1
EIF2B1	0	0	0	0	0	0	0	0
EIF2B2	0	0	0	0	0	0	0	0
EIF2B5	0	0	0	0	0	0	0	0
EIF2S1	0	0	0	0	0	0	0	0
EIF2S2	0	0	0	0	0	0	0	0
EIF2S3	0	0	0	0	0	0	0	0
EIF3A	0	0	0	0	0	0	0	0
EIF3B	0	0	0	0	0	0	0	0
EIF3D	0	0	0	0	0	0	0	0
EIF3E	0	0	0	0	0	0	0	0
EIF3F	0	0	0	0	0	0	0	0
EIF3G	0	0	0	0	0	0	0	0
EIF3H	0	0	0	0	0	0	0	0
EIF3I	0	0	0	0	0	0	0	0
EIF3J	0	0	0	0	0	0	0	0
EIF3K	0	0	0	0	0	0	0	0
EIF4A1	0	0	0	0	0	0	0	0
EIF4B	0	0	0	0	0	0	0	0
EIF4G2	0	0	0	0	0	0	0	0
EIF4G3	0	0	0	0	0	0	0	0
EIF4H	0	0	0	0	0	0	0	0
EIF5	0	0	0	0	0	0	0	0
EIF5B	0	0	0	0	0	0	0	0
EPS15L1	0	0	0	0	0	0	0	0
ERAP1	0	0	0	0	0	0	0	0

Table S6 The differential proteins contribute to the enrichment of the significant pathways for the public dataset

Core enrichment proteins in down-regulated pathways of hESCs/HFs (hESCs vs HFs)

NAME	REACTOME_UNFOLDED_PROTEIN_RESPONSE	REACTOME_DIABETES_PATHWAYS	REACTOME_CELL_CELL_COMMUNICATION	REACTOME_GOLGI_ASSOCIATED_VESICLE_BIOGENESIS	REACTOME_TRANS_GOLGI_NETWORK_VESICLE_BUDDING	REACTOME_MEMBRANE_TRAFFICKING	REACTOME_NEURONAL_SYSTEM	REACTOME_TRANSMISSION_ACROSS_CHEMICAL_SYNAPSES	REACTOME_POST_TRANSLATIONAL_PROTEIN_MODIFICATION	REACTOME_ASPARAGINE_N_LINKED_GLYCOSYLATION	REACTOME_ING_BY_INSULIN_RECEPTOR	REACTOME_PREFOLDIN_MEDIATED_TRANSFER_OF_SUBSTRATE_TO_CCT_TRIC	REACTOME_PROTEIN_FOLDING
ETF1	0	0	0	0	0	0	0	0	0	0	0	0	0
EXOC1	0	1	0	0	0	0	0	0	0	0	0	0	0
EXOC2	0	1	0	0	0	0	0	0	0	0	0	0	0
EXOC3	0	1	0	0	0	0	0	0	0	0	0	0	0
EXOC4	0	1	0	0	0	0	0	0	0	0	0	0	0
EXOC5	0	1	0	0	0	0	0	0	0	0	0	0	0
EXOC8	0	1	0	0	0	0	0	0	0	0	0	0	0
GAK	0	0	0	1	0	0	0	0	0	0	0	0	0
GALNT2	0	0	0	0	0	0	0	0	1	0	0	0	0
GBF1	0	0	0	0	1	1	0	0	0	0	0	0	0
GLS	0	0	0	0	0	0	1	1	0	0	0	0	0
GMPPA	0	0	0	0	0	0	0	0	1	1	0	0	0
GMPPB	0	0	0	0	0	0	0	0	1	1	0	0	0
GPC1	0	0	0	0	0	0	0	0	0	0	0	0	0
GSK3A	0	0	0	0	0	0	0	0	0	0	0	0	0
GTF2F2	0	0	0	0	0	0	0	0	0	0	0	0	0
HEBP1	0	0	0	0	0	0	0	0	0	0	0	0	0
HSP90AA1	0	0	0	0	0	0	0	0	0	0	0	0	0
HSPA1B	0	0	0	0	0	0	0	0	0	0	0	0	0
HSPA5	1	1	0	0	0	0	0	0	0	0	0	0	0
HSPA8	0	0	0	0	0	0	0	0	0	0	0	0	0
IPO5	0	0	0	0	0	0	0	0	0	0	0	0	0
ISG15	0	0	0	0	0	0	0	0	0	0	0	0	0
KIF3B	0	0	0	0	0	0	0	0	0	0	0	0	0
KIF5B	0	1	0	0	0	0	0	0	0	0	0	0	0
KPNA4	0	0	0	0	0	0	0	0	0	0	0	0	0
KPNB1	0	0	0	0	0	0	0	0	0	0	0	0	0
KRAS	0	0	0	0	0	0	0	0	0	0	0	0	0
LGALS3	0	0	0	0	0	0	0	0	0	0	0	0	0
LMAN1	0	0	0	0	0	0	0	0	1	1	0	0	0
LNPEP	0	0	0	0	0	0	0	0	0	0	0	0	0
LSM1	0	0	0	0	0	0	0	0	0	0	0	0	0
MAVS	0	0	0	0	0	0	0	0	0	0	0	0	0
MGAT1	0	0	0	0	0	0	0	0	1	1	0	0	0
MRC2	0	0	0	0	0	0	0	0	0	0	0	0	0
MYO5A	0	1	0	0	0	0	0	0	0	0	0	0	0
NAPA	0	0	0	1	1	1	0	0	0	0	0	0	0
NMT1	0	0	0	0	0	0	0	0	0	0	0	0	0
NPEPPS	0	0	0	0	0	0	0	0	0	0	0	0	0
NSF	0	0	0	0	0	0	1	1	0	0	0	0	0
PDIA3	0	0	0	0	0	0	0	0	0	0	0	0	0
PFDN1	0	0	0	0	0	0	0	0	0	0	0	1	1
PFDN2	0	0	0	0	0	0	0	0	0	0	0	1	1
PFDN4	0	0	0	0	0	0	0	0	0	0	0	1	1
PFDN5	0	0	0	0	0	0	0	0	0	0	0	1	1
PFDN6	0	0	0	0	0	0	0	0	0	0	0	1	1
PICALM	0	0	0	1	1	1	0	0	0	0	0	0	0
PIGS	0	0	0	0	0	0	0	0	0	0	0	0	0
PIK3C3	0	0	0	0	0	0	0	0	0	0	1	0	0
PIN1	0	0	0	0	0	0	0	0	0	0	0	0	0
PITPNA	0	0	0	0	0	0	0	0	0	0	0	0	0
PMM2	0	0	0	0	0	0	0	0	1	1	0	0	0

NAME	REACTOME_INNATE_IMMUNE_SYSTEM	REACTOME_MHC_CLASS_II_ANTIGEN_PRESENTATION	REACTOME_IMMUNE_SYSTEM	REACTOME_ADAPTIVE_IMMUNE_SYSTEM	REACTOME_APOPTOSIS	REACTOME_CLASS_I_MHC_MEDIATED_ANTIGEN_PROCESSING_PRESENTATION	REACTOME_ANTIGEN_PROCESSING_UBIQUITINATION_PROTEASOME_DEGRADATION	REACTOME_ER_PHAGOSOME_PATHWAY	REACTOME_ANTIGEN_PROCESSING_CROSS_PRESENTATION	REACTOME_REGULATION_OF_MRNA_STABILITY_BY_PROTEINS_THAT_BIND_AU_RICH_ELEMENTS	REACTOME_DESTABILIZATION_OF_MRNA_BY_AUF1_HNRNP_D0	REACTOME_REGULATION_OF_ORNITHINE_DECARBOXYLASE_ODC	REACTOME_REGULATION_OF_MITOTIC_CELL_CYCLE
ETF1	0	0	0	0	0	0	0	0	0	0	0	0	0
EXOC1	0	0	0	0	0	0	0	0	0	0	0	0	0
EXOC2	0	0	0	0	0	0	0	0	0	0	0	0	0
EXOC3	0	0	0	0	0	0	0	0	0	0	0	0	0
EXOC4	0	0	0	0	0	0	0	0	0	0	0	0	0
EXOC5	0	0	0	0	0	0	0	0	0	0	0	0	0
EXOC8	0	0	0	0	0	0	0	0	0	0	0	0	0
GAK	0	0	0	0	0	0	0	0	0	0	0	0	0
GALNT2	0	0	0	0	0	0	0	0	0	0	0	0	0
GBF1	0	0	0	0	0	0	0	0	0	0	0	0	0
GLS	0	0	0	0	0	0	0	0	0	0	0	0	0
GMPPA	0	0	0	0	0	0	0	0	0	0	0	0	0
GMPPB	0	0	0	0	0	0	0	0	0	0	0	0	0
GPC1	0	0	0	0	0	0	0	0	0	0	0	0	0
GSK3A	0	0	0	0	0	0	0	0	0	0	0	0	0
GTF2F2	0	0	0	0	0	0	0	0	0	0	0	0	0
HEBP1	0	0	0	0	0	0	0	0	0	0	0	0	0
HSP90AA1	0	0	0	0	0	0	0	0	0	0	0	0	0
HSPA1B	0	0	0	0	0	0	0	0	0	0	1	0	0
HSPA5	0	0	1	1	0	1	0	0	0	0	0	0	0
HSPA8	0	0	0	0	0	0	0	0	0	1	1	0	0
IPO5	0	0	0	0	0	0	0	0	0	0	0	0	0
ISG15	1	0	1	0	0	0	0	0	0	0	0	0	0
KIF3B	0	1	1	1	0	0	0	0	0	0	0	0	0
KIF5B	0	1	1	1	0	0	0	0	0	0	0	0	0
KPNA4	0	0	1	0	0	0	0	0	0	0	0	0	0
KPNB1	0	0	0	0	1	0	0	0	0	0	0	0	0
KRAS	0	0	0	0	0	0	0	0	0	0	0	0	0
LGALS3	1	0	1	0	0	0	0	0	0	0	0	0	0
LMAN1	0	0	0	0	0	0	0	0	0	0	0	0	0
LNPEP	0	0	1	1	0	1	1	0	1	0	0	0	0
LSM1	0	0	0	0	0	0	0	0	0	0	0	0	0
MAVS	0	0	1	0	0	0	0	0	0	0	0	0	0
MGAT1	0	0	0	0	0	0	0	0	0	0	0	0	0
MRC2	0	0	1	1	0	1	0	0	1	0	0	0	0
MYO5A	0	0	0	0	0	0	0	0	0	0	0	0	0
NAPA	0	0	0	0	0	0	0	0	0	0	0	0	0
NMT1	0	0	0	0	1	0	0	0	0	0	0	0	0
NPEPPS	0	0	1	1	0	1	1	0	0	0	0	0	0
NSF	0	0	0	0	0	0	0	0	0	0	0	0	0
PDIA3	0	0	0	0	0	0	0	0	0	0	0	0	0
PFDN1	0	0	0	0	0	0	0	0	0	0	0	0	0
PFDN2	0	0	0	0	0	0	0	0	0	0	0	0	0
PFDN4	0	0	0	0	0	0	0	0	0	0	0	0	0
PFDN5	0	0	0	0	0	0	0	0	0	0	0	0	0
PFDN6	0	0	0	0	0	0	0	0	0	0	0	0	0
PICALM	0	0	0	0	0	0	0	0	0	0	0	0	0
PIGS	0	0	0	0	0	0	0	0	0	0	0	0	0
PIK3C3	0	0	0	0	0	0	0	0	0	0	0	0	0
PIN1	1	0	1	0	0	0	0	0	0	0	0	0	0
PITPNA	0	0	0	0	0	0	0	0	0	0	0	0	0
PMM2	0	0	0	0	0	0	0	0	0	0	0	0	0

NAME	REACTOME_CDT1_ASSOCIATION_WITH_THE_CDC6_ORC_ORIGIN_COMPLEX	REACTOME_APC_C_CDC20_MEDIATED_DEGRADATION_OF_MITOTIC_PROTEINS	REACTOME_APC_C_CDH1_MEDIATED_DEGRADATION_OF_CDC20_AND_OTHER_APC_C_CDH1_TARGETED_PROTEINS_IN_LATE_MITOSIS_EARLY_G1	REACTOME_CYCLIN_E_ASSOCIATED_EVENTS_DURING_G1_S_TRANSITION_	REACTOME_SCFSKP2_MEDIATED_DEGRADATION_OF_P27_P21	REACTOME_AUTODEGRADATION_OF_CDH1_BY_CDH1_APC_C	REACTOME_SCF_BETA_TRCP_MEDIATED_DEGRADATION_OF_EMI1	REACTOME_P53_DEPENDENT_G1_DNA_DAMAGE_RESPONSE	REACTOME_CDK_MEDIATED_PHOSPHORYLATION_AND_REMOVAL_OF_CDC6	REACTOME_AUTODEGRADATION_OF_THE_E3_UBIQUITIN_LIGASE_COP1	REACTOME_P53_INDEPENDENT_G1_S_DNA_DAMAGE_CHECKPOINT	REACTOME_VIF_MEDIATED_DEGRADATION_OF_APOBEC3G	REACTOME_CROSS_PRESENTATION_OF_SOLUBLE_EXOGENOUS_ANTIGENS_ENDOSOMES
ETF1	0	0	0	0	0	0	0	0	0	0	0	0	0
EXOC1	0	0	0	0	0	0	0	0	0	0	0	0	0
EXOC2	0	0	0	0	0	0	0	0	0	0	0	0	0
EXOC3	0	0	0	0	0	0	0	0	0	0	0	0	0
EXOC4	0	0	0	0	0	0	0	0	0	0	0	0	0
EXOC5	0	0	0	0	0	0	0	0	0	0	0	0	0
EXOC8	0	0	0	0	0	0	0	0	0	0	0	0	0
GAK	0	0	0	0	0	0	0	0	0	0	0	0	0
GALNT2	0	0	0	0	0	0	0	0	0	0	0	0	0
GBF1	0	0	0	0	0	0	0	0	0	0	0	0	0
GLS	0	0	0	0	0	0	0	0	0	0	0	0	0
GMPPA	0	0	0	0	0	0	0	0	0	0	0	0	0
GMPPB	0	0	0	0	0	0	0	0	0	0	0	0	0
GPC1	0	0	0	0	0	0	0	0	0	0	0	0	0
GSK3A	0	0	0	0	0	0	0	0	0	0	0	0	0
GTF2F2	0	0	0	0	0	0	0	0	0	0	0	0	0
HEBP1	0	0	0	0	0	0	0	0	0	0	0	0	0
HSP90AA1	0	0	0	0	0	0	0	0	0	0	0	0	0
HSPA1B	0	0	0	0	0	0	0	0	0	0	0	0	0
HSPA5	0	0	0	0	0	0	0	0	0	0	0	0	0
HSPA8	0	0	0	0	0	0	0	0	0	0	0	0	0
IPO5	0	0	0	0	0	0	0	0	0	0	0	0	0
ISG15	0	0	0	0	0	0	0	0	0	0	0	0	0
KIF3B	0	0	0	0	0	0	0	0	0	0	0	0	0
KIF5B	0	0	0	0	0	0	0	0	0	0	0	0	0
KPNA4	0	0	0	0	0	0	0	0	0	0	0	0	0
KPNB1	0	0	0	0	0	0	0	0	0	0	0	0	0
KRAS	0	0	0	0	0	0	0	0	0	0	0	0	0
LGALS3	0	0	0	0	0	0	0	0	0	0	0	0	0
LMAN1	0	0	0	0	0	0	0	0	0	0	0	0	0
LNPEP	0	0	0	0	0	0	0	0	0	0	0	0	0
LSM1	0	0	0	0	0	0	0	0	0	0	0	0	0
MAVS	0	0	0	0	0	0	0	0	0	0	0	0	0
MGAT1	0	0	0	0	0	0	0	0	0	0	0	0	0
MRC2	0	0	0	0	0	0	0	0	0	0	0	0	1
MYO5A	0	0	0	0	0	0	0	0	0	0	0	0	0
NAPA	0	0	0	0	0	0	0	0	0	0	0	0	0
NMT1	0	0	0	0	0	0	0	0	0	0	0	0	0
NPEPPS	0	0	0	0	0	0	0	0	0	0	0	0	0
NSF	0	0	0	0	0	0	0	0	0	0	0	0	0
PDIA3	0	0	0	0	0	0	0	0	0	0	0	0	0
PFDN1	0	0	0	0	0	0	0	0	0	0	0	0	0
PFDN2	0	0	0	0	0	0	0	0	0	0	0	0	0
PFDN4	0	0	0	0	0	0	0	0	0	0	0	0	0
PFDN5	0	0	0	0	0	0	0	0	0	0	0	0	0
PFDN6	0	0	0	0	0	0	0	0	0	0	0	0	0
PICALM	0	0	0	0	0	0	0	0	0	0	0	0	0
PIGS	0	0	0	0	0	0	0	0	0	0	0	0	0
PIK3C3	0	0	0	0	0	0	0	0	0	0	0	0	0
PIN1	0	0	0	0	0	0	0	0	0	0	0	0	0
PITPNA	0	0	0	0	0	0	0	0	0	0	0	0	0
PMM2	0	0	0	0	0	0	0	0	0	0	0	0	0

NAME	REACTOME_ING_BY_WNT	REACTOME_REGULATION_OF_APOPTOSIS	REACTOME_ACTIVATION_OF_NF_KAPPAB_IN_B_CELLS	REACTOME_DOWNSTREAM_ING_EVENTS_OF_B_CELL_RECEPTOR_BCR	REACTOME_ING_BY_THE_B_CELL_RECEPTOR_BCR	REACTOME_METABOLISM_OF_MRNA	REACTOME_METABOLISM_OF_PROTEINS	REACTOME_FORMATION_OF_THE_TERNARY_COMPLEX_AND_SUBSEQUENTLY_THE_43S_COMPLEX	REACTOME_ACTIVATION_OF_THE_MRNA_UPON_BINDING_OF_THE_CAP_BINDING_COMPLEX_AND_EIFS_AND_SUBSEQUENT_BINDING_TO_43S	REACTOME_SRP_DEPENDENT_COTRANSLATIONAL_PROTEIN_TARGETING_TO_MEMBRANE	REACTOME_INFLUENZA_VIRAL_RNA_TRANSCRIPTION_AND_REPLICATION	REACTOME_NONSENSE_MEDIATED_DECAY_ENHANCED_BY_THE_EXON_JUNCTION_COMPLEX	REACTOME_PEPTIDE_CHAIN_ELONGATION
ETF1	0	0	0	0	0	1	1	0	0	0	0	1	0
EXOC1	0	0	0	0	0	0	0	0	0	0	0	0	0
EXOC2	0	0	0	0	0	0	0	0	0	0	0	0	0
EXOC3	0	0	0	0	0	0	0	0	0	0	0	0	0
EXOC4	0	0	0	0	0	0	0	0	0	0	0	0	0
EXOC5	0	0	0	0	0	0	0	0	0	0	0	0	0
EXOC8	0	0	0	0	0	0	0	0	0	0	0	0	0
GAK	0	0	0	0	0	0	0	0	0	0	0	0	0
GALNT2	0	0	0	0	0	0	1	0	0	0	0	0	0
GBF1	0	0	0	0	0	0	0	0	0	0	0	0	0
GLS	0	0	0	0	0	0	0	0	0	0	0	0	0
GMPPA	0	0	0	0	0	0	1	0	0	0	0	0	0
GMPPB	0	0	0	0	0	0	1	0	0	0	0	0	0
GPC1	0	0	0	0	0	0	0	0	0	0	0	0	0
GSK3A	0	0	0	1	1	0	0	0	0	0	0	0	0
GTF2F2	0	0	0	0	0	0	0	0	0	0	1	0	0
HEBP1	0	0	0	0	0	0	0	0	0	0	0	0	0
HSP90AA1	0	0	0	0	0	0	0	0	0	0	1	0	0
HSPA1B	0	0	0	0	0	1	0	0	0	0	0	0	0
HSPA5	0	0	0	0	0	0	0	0	0	0	0	0	0
HSPA8	0	0	0	0	0	1	0	0	0	0	0	0	0
IPO5	0	0	0	0	0	0	0	0	0	0	1	0	0
ISG15	0	0	0	0	0	0	0	0	0	0	0	0	0
KIF3B	0	0	0	0	0	0	0	0	0	0	0	0	0
KIF5B	0	0	0	0	0	0	0	0	0	0	0	0	0
KPNA4	0	0	0	0	0	0	0	0	0	0	0	0	0
KPNB1	0	0	0	0	0	0	0	0	0	0	0	0	0
KRAS	0	0	0	1	1	0	0	0	0	0	0	0	0
LGALS3	0	0	0	0	0	0	0	0	0	0	0	0	0
LMAN1	0	0	0	0	0	0	1	0	0	0	0	0	0
LNPEP	0	0	0	0	0	0	0	0	0	0	0	0	0
LSM1	0	0	0	0	0	1	0	0	0	0	0	0	0
MAVS	0	0	0	0	0	0	0	0	0	0	0	0	0
MGAT1	0	0	0	0	0	0	1	0	0	0	0	0	0
MRC2	0	0	0	0	0	0	0	0	0	0	0	0	0
MYO5A	0	0	0	0	0	0	0	0	0	0	0	0	0
NAPA	0	0	0	0	0	0	0	0	0	0	0	0	0
NMT1	0	0	0	0	0	0	0	0	0	0	0	0	0
NPEPPS	0	0	0	0	0	0	0	0	0	0	0	0	0
NSF	0	0	0	0	0	0	0	0	0	0	0	0	0
PDIA3	0	0	0	0	0	0	1	0	0	0	0	0	0
PFDN1	0	0	0	0	0	0	1	0	0	0	0	0	0
PFDN2	0	0	0	0	0	0	1	0	0	0	0	0	0
PFDN4	0	0	0	0	0	0	1	0	0	0	0	0	0
PFDN5	0	0	0	0	0	0	1	0	0	0	0	0	0
PFDN6	0	0	0	0	0	0	1	0	0	0	0	0	0
PICALM	0	0	0	0	0	0	0	0	0	0	0	0	0
PIGS	0	0	0	0	0	0	1	0	0	0	0	0	0
PIK3C3	0	0	0	0	0	0	0	0	0	0	0	0	0
PIN1	0	0	0	0	0	0	0	0	0	0	0	0	0
PITPNA	0	0	0	0	0	0	0	0	0	0	0	0	0
PMM2	0	0	0	0	0	0	1	0	0	0	0	0	0

NAME	REACTOME_3_UTR_MEDIATED_TRANSLATIONAL_REGULATION	REACTOME_TRANSLATION	REACTOME_CELL_SURFACE_INTERACTIONS_AT_THE_VASCULAR_WALL	REACTOME_INTEGRIN_CELL_SURFACE_INTERACTIONS	REACTOME_RESPONSE_TO_ELEVATED_PLATELET_CYTOSOLIC_CA2_	REACTOME_HEMOSTASIS	REACTOME_REGULATION_OF_INSULIN_SECRETION	REACTOME_INTEGRATION_OF_ENERGY_METABOLISM	REACTOME_GPCR_DOWNSTREAM_ING	REACTOME_ING_BY_GPCR	REACTOME_ING_BY_ILS	REACTOME_ING_BY_FGFR_IN_DISEASE	REACTOME_ING_BY_ERBB2
ETF1	0	1	0	0	0	0	0	0	0	0	0	0	0
EXOC1	0	0	0	0	0	0	0	0	0	0	0	0	0
EXOC2	0	0	0	0	0	0	0	0	0	0	0	0	0
EXOC3	0	0	0	0	0	0	0	0	0	0	0	0	0
EXOC4	0	0	0	0	0	0	0	0	0	0	0	0	0
EXOC5	0	0	0	0	0	0	0	0	0	0	0	0	0
EXOC8	0	0	0	0	0	0	0	0	0	0	0	0	0
GAK	0	0	0	0	0	0	0	0	0	0	0	0	0
GALNT2	0	0	0	0	0	0	0	0	0	0	0	0	0
GBF1	0	0	0	0	0	0	0	0	0	0	0	0	0
GLS	0	0	0	0	0	0	0	0	0	0	0	0	0
GMPPA	0	0	0	0	0	0	0	0	0	0	0	0	0
GMPPB	0	0	0	0	0	0	0	0	0	0	0	0	0
GPC1	0	0	0	0	0	0	0	0	0	0	0	0	0
GSK3A	0	0	0	0	0	0	0	0	0	0	0	0	0
GTF2F2	0	0	0	0	0	0	0	0	0	0	0	0	0
HEBP1	0	0	0	0	0	0	0	0	1	1	0	0	0
HSP90AA1	0	0	0	0	0	0	0	0	0	0	0	0	0
HSPA1B	0	0	0	0	0	0	0	0	0	0	0	0	0
HSPA5	0	0	0	0	1	1	0	0	0	0	0	0	0
HSPA8	0	0	0	0	0	0	0	0	0	0	0	0	0
IPO5	0	0	0	0	0	0	0	0	0	0	0	0	0
ISG15	0	0	0	0	0	0	0	0	0	0	0	0	0
KIF3B	0	0	0	0	0	1	0	0	0	0	0	0	0
KIF5B	0	0	0	0	0	1	0	0	0	0	0	0	0
KPNA4	0	0	0	0	0	0	0	0	0	0	0	0	0
KPNB1	0	0	0	0	0	0	0	0	0	0	0	0	0
KRAS	0	0	0	0	0	0	0	0	0	0	0	0	0
LGALS3	0	0	0	0	0	0	0	0	0	0	0	0	0
LMAN1	0	0	0	0	0	0	0	0	0	0	0	0	0
LNPEP	0	0	0	0	0	0	0	0	0	0	0	0	0
LSM1	0	0	0	0	0	0	0	0	0	0	0	0	0
MAVS	0	0	0	0	0	0	0	0	0	0	0	0	0
MGAT1	0	0	0	0	0	0	0	0	0	0	0	0	0
MRC2	0	0	0	0	0	0	0	0	0	0	0	0	0
MYO5A	0	0	0	0	0	0	0	0	0	0	0	0	0
NAPA	0	0	0	0	0	0	0	0	0	0	0	0	0
NMT1	0	0	0	0	0	0	0	0	0	0	0	0	0
NPEPPS	0	0	0	0	0	0	0	0	0	0	0	0	0
NSF	0	0	0	0	0	0	0	0	0	0	0	0	0
PDIA3	0	0	0	0	0	0	0	0	0	0	0	0	0
PFDN1	0	0	0	0	0	0	0	0	0	0	0	0	0
PFDN2	0	0	0	0	0	0	0	0	0	0	0	0	0
PFDN4	0	0	0	0	0	0	0	0	0	0	0	0	0
PFDN5	0	0	0	0	0	0	0	0	0	0	0	0	0
PFDN6	0	0	0	0	0	0	0	0	0	0	0	0	0
PICALM	0	0	0	0	0	0	0	0	0	0	0	0	0
PIGS	0	0	0	0	0	0	0	0	0	0	0	0	0
PIK3C3	0	0	0	0	0	0	0	0	0	0	0	0	0
PIN1	0	0	0	0	0	0	0	0	0	0	0	0	0
PITPNA	0	0	0	0	0	0	0	0	0	0	0	0	0
PMM2	0	0	0	0	0	0	0	0	0	0	0	0	0

NAME	REACTOME_ING_BY_FGFR	REACTOME_DOWNSTREAM__TRANSDUCTION	REACTOME_LING_BY_NGF	REACTOME_NGF_LING_VIA_TRKA_FROM_THE_PLASMA_MEMBRANE	REACTOME_ING_BY_EGFR_IN_CANCER	REACTOME_ING_BY_PDGF	REACTOME_SEMAPHORIN_INTERACTIONS	REACTOME_L1CAM_INTERACTIONS	REACTOME_DEVELOPMENTAL_BIOLOGY	REACTOME_AXON_GUIDANCE	KEGG_ERBB_ING_PATHWAY	KEGG_INSULIN_ING_PATHWAY
ETF1	0	0	0	0	0	0	0	0	0	0	0	0
EXOC1	0	0	0	0	0	0	0	0	0	0	0	0
EXOC2	0	0	0	0	0	0	0	0	0	0	0	0
EXOC3	0	0	0	0	0	0	0	0	0	0	0	0
EXOC4	0	0	0	0	0	0	0	0	0	0	0	0
EXOC5	0	0	0	0	0	0	0	0	0	0	0	0
EXOC8	0	0	0	0	0	0	0	0	0	0	0	0
GAK	0	0	0	0	0	0	0	0	0	0	0	0
GALNT2	0	0	0	0	0	0	0	0	0	0	0	0
GBF1	0	0	0	0	0	0	0	0	0	0	0	0
GLS	0	0	0	0	0	0	0	0	0	0	0	0
GMPPA	0	0	0	0	0	0	0	0	0	0	0	0
GMPPB	0	0	0	0	0	0	0	0	0	0	0	0
GPC1	0	0	0	0	0	0	0	0	1	1	0	0
GSK3A	0	0	0	0	0	0	0	0	0	0	0	0
GTF2F2	0	0	0	0	0	0	0	0	0	0	0	0
HEBP1	0	0	0	0	0	0	0	0	0	0	0	0
HSP90AA1	0	0	0	0	0	0	0	0	0	0	0	0
HSPA1B	0	0	0	0	0	0	0	0	0	0	0	0
HSPA5	0	0	0	0	0	0	0	0	0	0	0	0
HSPA8	0	0	0	0	0	0	0	0	0	0	0	0
IPO5	0	0	0	0	0	0	0	0	0	0	0	0
ISG15	0	0	0	0	0	0	0	0	0	0	0	0
KIF3B	0	0	0	0	0	0	0	0	0	0	0	0
KIF5B	0	0	0	0	0	0	0	0	0	0	0	0
KPNA4	0	0	0	0	0	0	0	0	0	0	0	0
KPNB1	0	0	0	0	0	0	0	0	0	0	0	0
KRAS	0	0	0	0	0	0	0	0	0	0	0	0
LGALS3	0	0	0	0	0	0	0	0	0	0	0	0
LMAN1	0	0	0	0	0	0	0	0	0	0	0	0
LNPEP	0	0	0	0	0	0	0	0	0	0	0	0
LSM1	0	0	0	0	0	0	0	0	0	0	0	0
MAVS	0	0	0	0	0	0	0	0	0	0	0	0
MGAT1	0	0	0	0	0	0	0	0	0	0	0	0
MRC2	0	0	0	0	0	0	0	0	0	0	0	0
MYO5A	0	0	0	0	0	0	0	0	0	0	0	0
NAPA	0	0	0	0	0	0	0	0	0	0	0	0
NMT1	0	0	0	0	0	0	0	0	0	0	0	0
NPEPPS	0	0	0	0	0	0	0	0	0	0	0	0
NSF	0	0	0	0	0	0	0	0	0	0	0	0
PDIA3	0	0	0	0	0	0	0	0	0	0	0	0
PFDN1	0	0	0	0	0	0	0	0	0	0	0	0
PFDN2	0	0	0	0	0	0	0	0	0	0	0	0
PFDN4	0	0	0	0	0	0	0	0	0	0	0	0
PFDN5	0	0	0	0	0	0	0	0	0	0	0	0
PFDN6	0	0	0	0	0	0	0	0	0	0	0	0
PICALM	0	0	0	0	0	0	0	0	0	0	0	0
PIGS	0	0	0	0	0	0	0	0	0	0	0	0
PIK3C3	0	0	0	0	0	0	0	0	0	0	0	0
PIN1	0	0	0	0	0	0	0	0	0	0	0	0
PITPNA	0	0	0	0	0	0	0	0	1	1	0	0
PMM2	0	0	0	0	0	0	0	0	0	0	0	0

NAME	KEGG_PATHWAYS_IN_CANCER	KEGG_PROSTATE_CANCER	KEGG_CHEMOKINE_ING_PATHWAY	KEGG_NEUROTROPHIN_ING_PATHWAY	KEGG_MAPK_ING_PATHWAY	KEGG_ARRHYTHMOGENIC_RIGHT_VENTRICULAR_CARDIOMYOPATHY_ARVC	KEGG_ECM_RECEPTOR_INTERACTION	KEGG_FOCAL_ADHESION
ETF1	0	0	0	0	0	0	0	0
EXOC1	0	0	0	0	0	0	0	0
EXOC2	0	0	0	0	0	0	0	0
EXOC3	0	0	0	0	0	0	0	0
EXOC4	0	0	0	0	0	0	0	0
EXOC5	0	0	0	0	0	0	0	0
EXOC8	0	0	0	0	0	0	0	0
GAK	0	0	0	0	0	0	0	0
GALNT2	0	0	0	0	0	0	0	0
GBF1	0	0	0	0	0	0	0	0
GLS	0	0	0	0	0	0	0	0
GMPPA	0	0	0	0	0	0	0	0
GMPPB	0	0	0	0	0	0	0	0
GPC1	0	0	0	0	0	0	0	0
GSK3A	0	0	0	0	0	0	0	0
GTF2F2	0	0	0	0	0	0	0	0
HEBP1	0	0	0	0	0	0	0	0
HSP90AA1	0	0	0	0	0	0	0	0
HSPA1B	0	0	0	0	0	0	0	0
HSPA5	0	0	0	0	0	0	0	0
HSPA8	0	0	0	0	0	0	0	0
IPO5	0	0	0	0	0	0	0	0
ISG15	0	0	0	0	0	0	0	0
KIF3B	0	0	0	0	0	0	0	0
KIF5B	0	0	0	0	0	0	0	0
KPNA4	0	0	0	0	0	0	0	0
KPNB1	0	0	0	0	0	0	0	0
KRAS	0	0	0	0	0	0	0	0
LGALS3	0	0	0	0	0	0	0	0
LMAN1	0	0	0	0	0	0	0	0
LNPEP	0	0	0	0	0	0	0	0
LSM1	0	0	0	0	0	0	0	0
MAVS	0	0	0	0	0	0	0	0
MGAT1	0	0	0	0	0	0	0	0
MRC2	0	0	0	0	0	0	0	0
MYO5A	0	0	0	0	0	0	0	0
NAPA	0	0	0	0	0	0	0	0
NMT1	0	0	0	0	0	0	0	0
NPEPPS	0	0	0	0	0	0	0	0
NSF	0	0	0	0	0	0	0	0
PDIA3	0	0	0	0	0	0	0	0
PFDN1	0	0	0	0	0	0	0	0
PFDN2	0	0	0	0	0	0	0	0
PFDN4	0	0	0	0	0	0	0	0
PFDN5	0	0	0	0	0	0	0	0
PFDN6	0	0	0	0	0	0	0	0
PICALM	0	0	0	0	0	0	0	0
PIGS	0	0	0	0	0	0	0	0
PIK3C3	0	0	0	0	0	0	0	0
PIN1	0	0	0	0	0	0	0	0
PITPNA	0	0	0	0	0	0	0	0
PMM2	0	0	0	0	0	0	0	0

Table S6 The differential proteins contribute to the enrichment of the significant pathways for the public dataset

Core enrichment proteins in down-regulated pathways of hESCs/HFs (hESCs vs HFs)

NAME	REACTOME_UNFOLDED_PROTEIN_RESPONSE	REACTOME_DIABETES_PATHWAYS	REACTOME_CELL_CELL_COMMUNICATION	REACTOME_GOLGI_ASSOCIATED_VESICLE_BIOGENESIS	REACTOME_TRANS_GOLGI_NETWORK_VESICLE_BUDDING	REACTOME_MEMBRANE_TRAFFICKING	REACTOME_NEURONAL_SYSTEM	REACTOME_TRANSMISSION_ACROSS_CHEMICAL_SYNAPSES	REACTOME_POST_TRANSLATIONAL_PROTEIN_MODIFICATION	REACTOME_ASPARAGINE_N_LINKED_GLYCOSYLATION	REACTOME_ING_BY_INSULIN_RECEPTOR	REACTOME_PREFOLDIN_MEDIATED_TRANSFER_OF_SUBSTRATE_TO_CCT_TRIC	REACTOME_PROTEIN_FOLDING
PPP2R2A	0	0	0	0	0	0	0	0	0	0	0	0	0
PRKCSH	0	0	0	0	0	0	0	0	0	0	0	0	0
PSMC6	0	0	0	0	0	0	0	0	0	0	0	0	0
PSMD5	0	0	0	0	0	0	0	0	0	0	0	0	0
PVR	0	0	1	0	0	0	0	0	0	0	0	0	0
RAB3A	0	0	0	0	0	0	1	1	0	0	0	0	0
RHOC	0	0	0	0	0	0	0	0	0	0	0	0	0
RHOG	0	0	0	0	0	0	0	0	0	0	0	0	0
RPN1	0	0	0	0	0	0	0	0	0	0	0	0	0
RPS14	0	0	0	0	0	0	0	0	0	0	0	0	0
RTN4	0	0	0	0	0	0	0	0	0	0	0	0	0
SEC11A	0	0	0	0	0	0	0	0	0	0	0	0	0
SEC23A	0	0	0	0	0	1	0	0	1	1	0	0	0
SEC24B	0	0	0	0	0	1	0	0	1	1	0	0	0
SEC24C	0	0	0	0	0	1	0	0	1	1	0	0	0
SEC24D	0	0	0	0	0	1	0	0	1	1	0	0	0
SEC61A1	0	0	0	0	0	0	0	0	0	0	0	0	0
SEC61B	0	0	0	0	0	0	0	0	0	0	0	0	0
SLC25A12	0	0	0	0	0	0	0	0	0	0	0	0	0
SLC25A6	0	0	0	0	0	0	0	0	0	0	0	0	0
SLC38A2	0	0	0	0	0	0	1	1	0	0	0	0	0
SNX9	0	0	0	1	1	1	0	0	0	0	0	0	0
SPARC	0	0	0	0	0	0	0	0	0	0	0	0	0
SPCS3	0	0	0	0	0	0	0	0	0	0	0	0	0
SQSTM1	0	0	0	0	0	0	0	0	0	0	0	0	0
SRP19	0	0	0	0	0	0	0	0	0	0	0	0	0
SRP68	0	0	0	0	0	0	0	0	0	0	0	0	0
SRP72	0	0	0	0	0	0	0	0	0	0	0	0	0
SRPR	1	1	0	0	0	0	0	0	0	0	0	0	0
SRPRB	1	1	0	0	0	0	0	0	0	0	0	0	0
SSR3	0	0	0	0	0	0	0	0	0	0	0	0	0
SSR4	0	0	0	0	0	0	0	0	0	0	0	0	0
STAT3	0	0	0	0	0	0	0	0	0	0	0	0	0
STIM1	0	0	0	0	0	0	0	0	0	0	0	0	0
STT3A	0	0	0	0	0	0	0	0	0	0	0	0	0
STUB1	0	0	0	0	0	0	0	0	0	0	0	0	0
STX12	0	0	0	0	0	0	1	0	0	0	0	0	0
TAP1	0	0	0	0	0	0	0	0	0	0	0	0	0
TBCC	0	0	0	0	0	0	0	0	0	0	0	0	1
TCEB2	0	0	0	0	0	0	0	0	0	0	0	0	0
TCP1	0	0	0	0	0	0	0	0	0	0	0	1	1
TGOLN2	0	0	0	1	1	1	0	0	0	0	0	0	0
TNKS1BP1	0	0	0	0	0	0	0	0	0	0	0	0	0
TRIM25	0	0	0	0	0	0	0	0	0	0	0	0	0
TUBB2A	0	0	0	0	0	0	0	0	0	0	0	1	1
TXNDC5	0	0	0	1	1	1	0	0	0	0	0	0	0
UBA1	0	0	0	0	0	0	0	0	0	0	0	0	0
UBA6	0	0	0	0	0	0	0	0	0	0	0	0	0
UBE2M	0	0	0	0	0	0	0	0	0	0	0	0	0
UBE2N	0	0	0	0	0	0	0	0	0	0	0	0	0
UBE2V2	0	0	0	0	0	0	0	0	0	0	0	0	0
UBR4	0	0	0	0	0	0	0	0	0	0	0	0	0

NAME	REACTOME_INNATE_IMMUNE_SYSTEM	REACTOME_MHC_CLASS_II_ANTIGEN_PRESENTATION	REACTOME_IMMUNE_SYSTEM	REACTOME_ADAPTIVE_IMMUNE_SYSTEM	REACTOME_APOPTOSIS	REACTOME_CLASS_I_MHC_MEDIATED_ANTIGEN_PROCESSING_PRESENTATION	REACTOME_ANTIGEN_PROCESSING_UBIQUITINATION_PROTEASOME_DEGRADATION	REACTOME_ER_PHAGOSOME_PATHWAY	REACTOME_ANTIGEN_PROCESSING_CROSS_PRESENTATION	REACTOME_REGULATION_OF_MRNA_STABILITY_BY_PROTEINS_THAT_BIND_AU_RICH_ELEMENTS	REACTOME_DESTABILIZATION_OF_MRNA_BY_AUF1_HNRNP_D0	REACTOME_REGULATION_OF_ORNITHINE_DECARBOXYLASE_ODC	REACTOME_REGULATION_OF_MITOTIC_CELL_CYCLE
PPP2R2A	0	0	0	0	0	0	0	0	0	0	0	0	0
PRKCSH	0	0	0	0	0	0	0	0	0	0	0	0	0
PSMC6	0	0	0	0	0	0	0	0	0	0	1	0	0
PSMD5	0	0	1	1	1	1	1	1	1	1	1	1	1
PVR	0	0	1	1	0	0	0	0	0	0	0	0	0
RAB3A	0	0	0	0	0	0	0	0	0	0	0	0	0
RHOC	0	0	0	0	0	0	0	0	0	0	0	0	0
RHOG	0	0	0	0	0	0	0	0	0	0	0	0	0
RPN1	0	0	0	0	0	0	0	0	0	0	0	0	0
RPS14	0	0	0	0	0	0	0	0	0	0	0	0	0
RTN4	0	0	0	0	0	0	0	0	0	0	0	0	0
SEC11A	0	0	0	0	0	0	0	0	0	0	0	0	0
SEC23A	0	1	1	1	0	1	0	0	0	0	0	0	0
SEC24B	0	1	1	1	0	1	0	0	0	0	0	0	0
SEC24C	0	1	1	1	0	1	0	0	0	0	0	0	0
SEC24D	0	1	1	1	0	1	0	0	0	0	0	0	0
SEC61A1	0	0	1	1	0	1	0	1	1	0	0	0	0
SEC61B	0	0	1	1	0	1	0	1	1	0	0	0	0
SLC25A12	0	0	0	0	0	0	0	0	0	0	0	0	0
SLC25A6	0	0	0	0	0	0	0	0	0	0	0	0	0
SLC38A2	0	0	0	0	0	0	0	0	0	0	0	0	0
SNX9	0	0	0	0	0	0	0	0	0	0	0	0	0
SPARC	0	0	0	0	0	0	0	0	0	0	0	0	0
SPCS3	0	0	0	0	0	0	0	0	0	0	0	0	0
SQSTM1	0	0	1	0	0	0	0	0	0	0	0	0	0
SRP19	0	0	0	0	0	0	0	0	0	0	0	0	0
SRP68	0	0	0	0	0	0	0	0	0	0	0	0	0
SRP72	0	0	0	0	0	0	0	0	0	0	0	0	0
SRPR	0	0	0	0	0	0	0	0	0	0	0	0	0
SRPRB	0	0	0	0	0	0	0	0	0	0	0	0	0
SSR3	0	0	0	0	0	0	0	0	0	0	0	0	0
SSR4	0	0	0	0	0	0	0	0	0	0	0	0	0
STAT3	0	0	1	0	0	0	0	0	0	0	0	0	0
STIM1	0	0	1	1	0	0	0	0	0	0	0	0	0
STT3A	0	0	0	0	0	0	0	0	0	0	0	0	0
STUB1	0	0	1	1	0	1	1	0	0	0	0	0	0
STX12	0	0	0	0	0	0	0	0	0	0	0	0	0
TAP1	0	0	1	1	0	1	0	1	1	0	0	0	0
TBCC	0	0	0	0	0	0	0	0	0	0	0	0	0
TCEB2	0	0	0	1	0	1	1	0	0	0	0	0	0
TCP1	0	0	0	0	0	0	0	0	0	0	0	0	0
TGOLN2	0	0	0	0	0	0	0	0	0	0	0	0	0
TNKS1BP1	0	0	0	0	0	0	0	0	0	0	0	0	0
TRIM25	1	0	1	0	0	0	0	0	0	0	0	0	0
TUBB2A	0	0	0	0	0	0	0	0	0	0	0	0	0
TXNDC5	0	0	0	0	0	0	0	0	0	0	0	0	0
UBA1	0	0	0	0	0	1	1	0	0	0	0	0	0
UBA6	0	0	1	1	0	1	1	0	0	0	0	0	0
UBE2M	0	0	1	1	0	1	1	0	0	0	0	0	0
UBE2N	0	0	0	0	0	1	1	0	0	0	0	0	0
UBE2V2	0	0	0	0	0	1	1	0	0	0	0	0	0
UBR4	0	0	0	1	0	1	1	0	0	0	0	0	0

NAME	REACTOME_CDT1_ASSOCIATION_WITH_THE_CDC6_ORC_ORIGIN_COMPLEX	REACTOME_APC_C_CDC20_MEDIATED_DEGRADATION_OF_MITOTIC_PROTEINS	REACTOME_APC_C_CDH1_MEDIATED_DEGRADATION_OF_CDC20_AND_OTHER_APC_C_CDH1_TARGETED_PROTEINS_IN_LATE_MITOSIS_EARLY_G1	REACTOME_CYCLIN_E_ASSOCIATED_EVENTS_DURING_G1_S_TRANSITION_	REACTOME_SCFSKP2_MEDIATED_DEGRADATION_OF_P27_P21	REACTOME_AUTODEGRADATION_OF_CDH1_BY_CDH1_APC_C	REACTOME_SCF_BETA_TRCP_MEDIATED_DEGRADATION_OF_EMI1	REACTOME_P53_DEPENDENT_G1_DNA_DAMAGE_RESPONSE	REACTOME_CDK_MEDIATED_PHOSPHORYLATION_AND_REMOVAL_OF_CDC6	REACTOME_AUTODEGRADATION_OF_THE_E3_UBIQUITIN_LIGASE_COP1	REACTOME_P53_INDEPENDENT_G1_S_DNA_DAMAGE_CHECKPOINT	REACTOME_VIF_MEDIATED_DEGRADATION_OF_APOBEC3G	REACTOME_CROSS_PRESENTATION_OF_SOLUBLE_EXOGENOUS_ANTIGENS_ENDOSOMES
PPP2R2A	0	0	0	0	0	0	0	0	0	0	0	0	0
PRKCSH	0	0	0	0	0	0	0	0	0	0	0	0	0
PSMC6	0	0	0	0	0	0	0	0	0	0	0	0	0
PSMD5	1	1	1	1	1	1	1	1	1	1	1	1	1
PVR	0	0	0	0	0	0	0	0	0	0	0	0	0
RAB3A	0	0	0	0	0	0	0	0	0	0	0	0	0
RHOC	0	0	0	0	0	0	0	0	0	0	0	0	0
RHOG	0	0	0	0	0	0	0	0	0	0	0	0	0
RPN1	0	0	0	0	0	0	0	0	0	0	0	0	0
RPS14	0	0	0	0	0	0	0	0	0	0	0	0	0
RTN4	0	0	0	0	0	0	0	0	0	0	0	0	0
SEC11A	0	0	0	0	0	0	0	0	0	0	0	0	0
SEC23A	0	0	0	0	0	0	0	0	0	0	0	0	0
SEC24B	0	0	0	0	0	0	0	0	0	0	0	0	0
SEC24C	0	0	0	0	0	0	0	0	0	0	0	0	0
SEC24D	0	0	0	0	0	0	0	0	0	0	0	0	0
SEC61A1	0	0	0	0	0	0	0	0	0	0	0	0	0
SEC61B	0	0	0	0	0	0	0	0	0	0	0	0	0
SLC25A12	0	0	0	0	0	0	0	0	0	0	0	0	0
SLC25A6	0	0	0	0	0	0	0	0	0	0	0	0	0
SLC38A2	0	0	0	0	0	0	0	0	0	0	0	0	0
SNX9	0	0	0	0	0	0	0	0	0	0	0	0	0
SPARC	0	0	0	0	0	0	0	0	0	0	0	0	0
SPCS3	0	0	0	0	0	0	0	0	0	0	0	0	0
SQSTM1	0	0	0	0	0	0	0	0	0	0	0	0	0
SRP19	0	0	0	0	0	0	0	0	0	0	0	0	0
SRP68	0	0	0	0	0	0	0	0	0	0	0	0	0
SRP72	0	0	0	0	0	0	0	0	0	0	0	0	0
SRPR	0	0	0	0	0	0	0	0	0	0	0	0	0
SRPRB	0	0	0	0	0	0	0	0	0	0	0	0	0
SSR3	0	0	0	0	0	0	0	0	0	0	0	0	0
SSR4	0	0	0	0	0	0	0	0	0	0	0	0	0
STAT3	0	0	0	0	0	0	0	0	0	0	0	0	0
STIM1	0	0	0	0	0	0	0	0	0	0	0	0	0
STT3A	0	0	0	0	0	0	0	0	0	0	0	0	0
STUB1	0	0	0	0	0	0	0	0	0	0	0	0	0
STX12	0	0	0	0	0	0	0	0	0	0	0	0	0
TAP1	0	0	0	0	0	0	0	0	0	0	0	0	0
TBCC	0	0	0	0	0	0	0	0	0	0	0	0	0
TCEB2	0	0	0	0	0	0	0	0	0	0	0	1	0
TCP1	0	0	0	0	0	0	0	0	0	0	0	0	0
TGOLN2	0	0	0	0	0	0	0	0	0	0	0	0	0
TNKS1BP1	0	0	0	0	0	0	0	0	0	0	0	0	0
TRIM25	0	0	0	0	0	0	0	0	0	0	0	0	0
TUBB2A	0	0	0	0	0	0	0	0	0	0	0	0	0
TXNDC5	0	0	0	0	0	0	0	0	0	0	0	0	0
UBA1	0	0	0	0	0	0	0	0	0	0	0	0	0
UBA6	0	0	0	0	0	0	0	0	0	0	0	0	0
UBE2M	0	0	0	0	0	0	0	0	0	0	0	0	0
UBE2N	0	0	0	0	0	0	0	0	0	0	0	0	0
UBE2V2	0	0	0	0	0	0	0	0	0	0	0	0	0
UBR4	0	0	0	0	0	0	0	0	0	0	0	0	0

NAME	REACTOME_ING_BY_WNT	REACTOME_REGULATION_OF_APOPTOSIS	REACTOME_ACTIVATION_OF_NF_KAPPAB_IN_B_CELLS	REACTOME_DOWNSTREAM_ING_EVENTS_OF_B_CELL_RECEPTOR_BCR	REACTOME_ING_BY_THE_B_CELL_RECEPTOR_BCR	REACTOME_METABOLISM_OF_MRNA	REACTOME_METABOLISM_OF_PROTEINS	REACTOME_FORMATION_OF_THE_TERNARY_COMPLEX_AND_SUBSEQUENTLY_THE_43S_COMPLEX	REACTOME_ACTIVATION_OF_THE_MRNA_UPON_BINDING_OF_THE_CAP_BINDING_COMPLEX_AND_EIFS_AND_SUBSEQUENT_BINDING_TO_43S	REACTOME_SRP_DEPENDENT_COTRANSLATIONAL_PROTEIN_TARGETING_TO_MEMBRANE	REACTOME_INFLUENZA_VIRAL_RNA_TRANSCRIPTION_AND_REPLICATION	REACTOME_NONSENSE_MEDIATED_DECAY_ENHANCED_BY_THE_EXON_JUNCTION_COMPLEX	REACTOME_PEPTIDE_CHAIN_ELONGATION
PPP2R2A	0	0	0	0	0	1	0	0	0	0	0	1	0
PRKCSH	0	0	0	0	0	0	1	0	0	0	0	0	0
PSMC6	0	0	0	1	1	1	0	0	0	0	0	0	0
PSMD5	1	1	1	1	1	1	0	0	0	0	0	0	0
PVR	0	0	0	0	0	0	0	0	0	0	0	0	0
RAB3A	0	0	0	0	0	0	0	0	0	0	0	0	0
RHOC	0	0	0	0	0	0	0	0	0	0	0	0	0
RHOG	0	0	0	0	0	0	0	0	0	0	0	0	0
RPN1	0	0	0	0	0	0	1	0	0	1	0	0	0
RPS14	0	0	0	0	0	1	1	1	1	1	1	1	1
RTN4	0	0	0	0	0	0	0	0	0	0	0	0	0
SEC11A	0	0	0	0	0	0	0	0	0	1	0	0	0
SEC23A	0	0	0	0	0	0	1	0	0	0	0	0	0
SEC24B	0	0	0	0	0	0	1	0	0	0	0	0	0
SEC24C	0	0	0	0	0	0	1	0	0	0	0	0	0
SEC24D	0	0	0	0	0	0	1	0	0	0	0	0	0
SEC61A1	0	0	0	0	0	0	1	0	0	1	0	0	0
SEC61B	0	0	0	0	0	0	1	0	0	1	0	0	0
SLC25A12	0	0	0	0	0	0	1	0	0	0	0	0	0
SLC25A6	0	0	0	0	0	0	1	0	0	0	0	0	0
SLC38A2	0	0	0	0	0	0	0	0	0	0	0	0	0
SNX9	0	0	0	0	0	0	0	0	0	0	0	0	0
SPARC	0	0	0	0	0	0	0	0	0	0	0	0	0
SPCS3	0	0	0	0	0	0	0	0	0	1	0	0	0
SQSTM1	0	0	0	0	0	0	0	0	0	0	0	0	0
SRP19	0	0	0	0	0	0	1	0	0	1	0	0	0
SRP68	0	0	0	0	0	0	1	0	0	1	0	0	0
SRP72	0	0	0	0	0	0	1	0	0	1	0	0	0
SRPR	0	0	0	0	0	0	1	0	0	1	0	0	0
SRPRB	0	0	0	0	0	0	1	0	0	1	0	0	0
SSR3	0	0	0	0	0	0	1	0	0	1	0	0	0
SSR4	0	0	0	0	0	0	1	0	0	1	0	0	0
STAT3	0	0	0	0	0	0	0	0	0	0	0	0	0
STIM1	0	0	0	0	1	0	0	0	0	0	0	0	0
STT3A	0	0	0	0	0	0	1	0	0	0	0	0	0
STUB1	0	0	0	0	0	0	0	0	0	0	0	0	0
STX12	0	0	0	0	0	0	0	0	0	0	0	0	0
TAP1	0	0	0	0	0	0	0	0	0	0	0	0	0
TBCC	0	0	0	0	0	0	1	0	0	0	0	0	0
TCEB2	0	0	0	0	0	0	0	0	0	0	0	0	0
TCP1	0	0	0	0	0	0	1	0	0	0	0	0	0
TGOLN2	0	0	0	0	0	0	0	0	0	0	0	0	0
TNKS1BP1	0	0	0	0	0	1	0	0	0	0	0	0	0
TRIM25	0	0	0	0	0	0	0	0	0	0	0	0	0
TUBB2A	0	0	0	0	0	0	1	0	0	0	0	0	0
TXNDC5	0	0	0	0	0	0	0	0	0	0	0	0	0
UBA1	0	0	0	0	0	0	0	0	0	0	0	0	0
UBA6	0	0	0	0	0	0	0	0	0	0	0	0	0
UBE2M	0	0	0	0	0	0	0	0	0	0	0	0	0
UBE2N	0	0	0	0	0	0	0	0	0	0	0	0	0
UBE2V2	0	0	0	0	0	0	0	0	0	0	0	0	0
UBR4	0	0	0	0	0	0	0	0	0	0	0	0	0

NAME	REACTOME_3_UTR_MEDIATED_TRANSLATIONAL_REGULATION	REACTOME_TRANSLATION	REACTOME_CELL_SURFACE_INTERACTIONS_AT_THE_VASCULAR_WALL	REACTOME_INTEGRIN_CELL_SURFACE_INTERACTIONS	REACTOME_RESPONSE_TO_ELEVATED_PLATELET_CYTOSOLIC_CA2_	REACTOME_HEMOSTASIS	REACTOME_REGULATION_OF_INSULIN_SECRETION	REACTOME_INTEGRATION_OF_ENERGY_METABOLISM	REACTOME_GPCR_DOWNSTREAM_ING	REACTOME_ING_BY_GPCR	REACTOME_ING_BY_ILS	REACTOME_ING_BY_FGFR_IN_DISEASE	REACTOME_ING_BY_ERBB2
PPP2R2A	0	0	0	0	0	0	0	0	0	0	0	0	0
PRKCSH	0	0	0	0	0	0	0	0	0	0	0	0	0
PSMC6	0	0	0	0	0	0	0	0	0	0	0	0	0
PSMD5	0	0	0	0	0	0	0	0	0	0	0	0	0
PVR	0	0	0	0	0	0	0	0	0	0	0	0	0
RAB3A	0	0	0	0	0	0	0	0	0	0	0	0	0
RHOC	0	0	0	0	0	0	0	0	1	1	0	0	0
RHOG	0	0	0	0	0	1	0	0	1	1	0	0	0
RPN1	0	1	0	0	0	0	0	0	0	0	0	0	0
RPS14	1	1	0	0	0	0	0	0	0	0	0	0	0
RTN4	0	0	0	0	0	0	0	0	0	0	0	0	0
SEC11A	0	1	0	0	0	0	0	0	0	0	0	0	0
SEC23A	0	0	0	0	0	0	0	0	0	0	0	0	0
SEC24B	0	0	0	0	0	0	0	0	0	0	0	0	0
SEC24C	0	0	0	0	0	0	0	0	0	0	0	0	0
SEC24D	0	0	0	0	0	0	0	0	0	0	0	0	0
SEC61A1	0	1	0	0	0	0	0	0	0	0	0	0	0
SEC61B	0	1	0	0	0	0	0	0	0	0	0	0	0
SLC25A12	0	0	0	0	0	0	0	0	0	0	0	0	0
SLC25A6	0	0	0	0	0	0	0	0	0	0	0	0	0
SLC38A2	0	0	0	0	0	0	0	0	0	0	0	0	0
SNX9	0	0	0	0	0	0	0	0	0	0	0	0	0
SPARC	0	0	0	0	1	1	0	0	0	0	0	0	0
SPCS3	0	1	0	0	0	0	0	0	0	0	0	0	0
SQSTM1	0	0	0	0	0	0	0	0	0	0	1	0	0
SRP19	0	1	0	0	0	0	0	0	0	0	0	0	0
SRP68	0	1	0	0	0	0	0	0	0	0	0	0	0
SRP72	0	1	0	0	0	0	0	0	0	0	0	0	0
SRPR	0	1	0	0	0	0	0	0	0	0	0	0	0
SRPRB	0	1	0	0	0	0	0	0	0	0	0	0	0
SSR3	0	1	0	0	0	0	0	0	0	0	0	0	0
SSR4	0	1	0	0	0	0	0	0	0	0	0	0	0
STAT3	0	0	0	0	0	0	0	0	0	0	0	0	0
STIM1	0	0	0	0	0	1	0	0	0	0	0	0	0
STT3A	0	0	0	0	0	0	0	0	0	0	0	0	0
STUB1	0	0	0	0	0	0	0	0	0	0	0	0	1
STX12	0	0	0	0	0	0	0	0	0	0	0	0	0
TAP1	0	0	0	0	0	0	0	0	0	0	0	0	0
TBCC	0	0	0	0	0	0	0	0	0	0	0	0	0
TCEB2	0	0	0	0	0	0	0	0	0	0	0	0	0
TCP1	0	0	0	0	0	0	0	0	0	0	0	0	0
TGOLN2	0	0	0	0	0	0	0	0	0	0	0	0	0
TNKS1BP1	0	0	0	0	0	0	0	0	0	0	0	0	0
TRIM25	0	0	0	0	0	0	0	0	0	0	0	0	0
TUBB2A	0	0	0	0	0	0	0	0	0	0	0	0	0
TXNDC5	0	0	0	0	0	0	0	0	0	0	0	0	0
UBA1	0	0	0	0	0	0	0	0	0	0	0	0	0
UBA6	0	0	0	0	0	0	0	0	0	0	0	0	0
UBE2M	0	0	0	0	0	0	0	0	0	0	0	0	0
UBE2N	0	0	0	0	0	0	0	0	0	0	0	0	0
UBE2V2	0	0	0	0	0	0	0	0	0	0	0	0	0
UBR4	0	0	0	0	0	0	0	0	0	0	0	0	0

NAME	REACTOME_ING_BY_FGFR	REACTOME_DOWNSTREAM__TRANSDUCTION	REACTOME_LING_BY_NGF	REACTOME_NGF_LING_VIA_TRKA_FROM_THE_PLASMA_MEMBRANE	REACTOME_ING_BY_EGFR_IN_CANCER	REACTOME_ING_BY_PDGF	REACTOME_SEMAPHORIN_INTERACTIONS	REACTOME_L1CAM_INTERACTIONS	REACTOME_DEVELOPMENTAL_BIOLOGY	REACTOME_AXON_GUIDANCE	KEGG_ERBB_ING_PATHWAY	KEGG_INSULIN_ING_PATHWAY
PPP2R2A	0	0	0	0	0	0	0	0	0	0	0	0
PRKCSH	0	0	0	0	0	0	0	0	0	0	0	0
PSMC6	0	0	0	0	0	0	0	0	0	0	0	0
PSMD5	0	0	0	0	0	0	0	0	0	0	0	0
PVR	0	0	0	0	0	0	0	0	0	0	0	0
RAB3A	0	0	0	0	0	0	0	0	0	0	0	0
RHOC	0	0	0	0	0	0	1	0	1	1	0	0
RHOG	0	0	0	0	0	0	1	0	1	1	0	0
RPN1	0	0	0	0	0	0	0	0	0	0	0	0
RPS14	0	0	0	0	0	0	0	0	0	0	0	0
RTN4	0	0	1	0	0	0	0	0	0	0	0	0
SEC11A	0	0	0	0	0	0	0	0	0	0	0	0
SEC23A	0	0	0	0	0	0	0	0	0	0	0	0
SEC24B	0	0	0	0	0	0	0	0	0	0	0	0
SEC24C	0	0	0	0	0	0	0	0	0	0	0	0
SEC24D	0	0	0	0	0	0	0	0	0	0	0	0
SEC61A1	0	0	0	0	0	0	0	0	0	0	0	0
SEC61B	0	0	0	0	0	0	0	0	0	0	0	0
SLC25A12	0	0	0	0	0	0	0	0	0	0	0	0
SLC25A6	0	0	0	0	0	0	0	0	0	0	0	0
SLC38A2	0	0	0	0	0	0	0	0	0	0	0	0
SNX9	0	0	0	0	0	0	0	0	0	0	0	0
SPARC	0	0	0	0	0	0	0	0	0	0	0	0
SPCS3	0	0	0	0	0	0	0	0	0	0	0	0
SQSTM1	0	0	1	0	0	0	0	0	0	0	0	0
SRP19	0	0	0	0	0	0	0	0	0	0	0	0
SRP68	0	0	0	0	0	0	0	0	0	0	0	0
SRP72	0	0	0	0	0	0	0	0	0	0	0	0
SRPR	0	0	0	0	0	0	0	0	0	0	0	0
SRPRB	0	0	0	0	0	0	0	0	0	0	0	0
SSR3	0	0	0	0	0	0	0	0	0	0	0	0
SSR4	0	0	0	0	0	0	0	0	0	0	0	0
STAT3	0	0	1	1	0	0	0	0	0	0	0	0
STIM1	0	0	0	0	0	0	0	0	0	0	0	0
STT3A	0	0	0	0	0	0	0	0	0	0	0	0
STUB1	0	0	0	0	0	0	0	0	0	0	0	0
STX12	0	0	0	0	0	0	0	0	0	0	0	0
TAP1	0	0	0	0	0	0	0	0	0	0	0	0
TBCC	0	0	0	0	0	0	0	0	0	0	0	0
TCEB2	0	0	0	0	0	0	0	0	0	0	0	0
TCP1	0	0	0	0	0	0	0	0	0	0	0	0
TGOLN2	0	0	0	0	0	0	0	0	0	0	0	0
TNKS1BP1	0	0	0	0	0	0	0	0	0	0	0	0
TRIM25	0	0	0	0	0	0	0	0	0	0	0	0
TUBB2A	0	0	0	0	0	0	0	0	0	0	0	0
TXNDC5	0	0	0	0	0	0	0	0	0	0	0	0
UBA1	0	0	0	0	0	0	0	0	0	0	0	0
UBA6	0	0	0	0	0	0	0	0	0	0	0	0
UBE2M	0	0	0	0	0	0	0	0	0	0	0	0
UBE2N	0	0	0	0	0	0	0	0	0	0	0	0
UBE2V2	0	0	0	0	0	0	0	0	0	0	0	0
UBR4	0	0	0	0	0	0	0	0	0	0	0	0

NAME	KEGG_PATHWAYS_IN_CANCER	KEGG_PROSTATE_CANCER	KEGG_CHEMOKINE_ING_PATHWAY	KEGG_NEUROTROPHIN_ING_PATHWAY	KEGG_MAPK_ING_PATHWAY	KEGG_ARRHYTHMOGENIC_RIGHT_VENTRICULAR_CARDIOMYOPATHY_ARVC	KEGG_ECM_RECEPTOR_INTERACTION	KEGG_FOCAL_ADHESION
PPP2R2A	0	0	0	0	0	0	0	0
PRKCSH	0	0	0	0	0	0	0	0
PSMC6	0	0	0	0	0	0	0	0
PSMD5	0	0	0	0	0	0	0	0
PVR	0	0	0	0	0	0	0	0
RAB3A	0	0	0	0	0	0	0	0
RHOC	0	0	0	0	0	0	0	0
RHOG	0	0	0	0	0	0	0	0
RPN1	0	0	0	0	0	0	0	0
RPS14	0	0	0	0	0	0	0	0
RTN4	0	0	0	0	0	0	0	0
SEC11A	0	0	0	0	0	0	0	0
SEC23A	0	0	0	0	0	0	0	0
SEC24B	0	0	0	0	0	0	0	0
SEC24C	0	0	0	0	0	0	0	0
SEC24D	0	0	0	0	0	0	0	0
SEC61A1	0	0	0	0	0	0	0	0
SEC61B	0	0	0	0	0	0	0	0
SLC25A12	0	0	0	0	0	0	0	0
SLC25A6	0	0	0	0	0	0	0	0
SLC38A2	0	0	0	0	0	0	0	0
SNX9	0	0	0	0	0	0	0	0
SPARC	0	0	0	0	0	0	0	0
SPCS3	0	0	0	0	0	0	0	0
SQSTM1	0	0	0	0	0	0	0	0
SRP19	0	0	0	0	0	0	0	0
SRP68	0	0	0	0	0	0	0	0
SRP72	0	0	0	0	0	0	0	0
SRPR	0	0	0	0	0	0	0	0
SRPRB	0	0	0	0	0	0	0	0
SSR3	0	0	0	0	0	0	0	0
SSR4	0	0	0	0	0	0	0	0
STAT3	0	0	0	0	0	0	0	0
STIM1	0	0	0	0	0	0	0	0
STT3A	0	0	0	0	0	0	0	0
STUB1	0	0	0	0	0	0	0	0
STX12	0	0	0	0	0	0	0	0
TAP1	0	0	0	0	0	0	0	0
TBCC	0	0	0	0	0	0	0	0
TCEB2	0	0	0	0	0	0	0	0
TCP1	0	0	0	0	0	0	0	0
TGOLN2	0	0	0	0	0	0	0	0
TNKS1BP1	0	0	0	0	0	0	0	0
TRIM25	0	0	0	0	0	0	0	0
TUBB2A	0	0	0	0	0	0	0	0
TXNDC5	0	0	0	0	0	0	0	0
UBA1	0	0	0	0	0	0	0	0
UBA6	0	0	0	0	0	0	0	0
UBE2M	0	0	0	0	0	0	0	0
UBE2N	0	0	0	0	0	0	0	0
UBE2V2	0	0	0	0	0	0	0	0
UBR4	0	0	0	0	0	0	0	0

Table S6 The differential proteins contribute to the enrichment of the significant pathways for the public dataset

Core enrichment proteins in down-regulated pathways of hESCs/HFs (hESCs vs HFs)

NAME	REACTOME_UNFOLDED_PROTEIN_RESPONSE	REACTOME_DIABETES_PATHWAYS	REACTOME_CELL_CELL_COMMUNICATION	REACTOME_GOLGI_ASSOCIATED_VESICLE_BIOGENESIS	REACTOME_TRANS_GOLGI_NETWORK_VESICLE_BUDDING	REACTOME_MEMBRANE_TRAFFICKING	REACTOME_NEURONAL_SYSTEM	REACTOME_TRANSMISSION_ACROSS_CHEMICAL_SYNAPSES	REACTOME_POST_TRANSLATIONAL_PROTEIN_MODIFICATION	REACTOME_ASPARAGINE_N_LINKED_GLYCOSYLATION	REACTOME_INSULIN_RECEPTOR_ING_BY_INSULIN_RECEPTOR	REACTOME_PREFOLDIN_MEDIATED_TRANSFER_OF_SUBSTRATE_TO_CCT_TRIC	REACTOME_PROTEIN_FOLDING
UGGT1	0	0	0	0	0	0	0	0	1	1	0	0	0
VAMP7	0	0	0	1	1	1	0	0	0	0	0	0	0
VBP1	0	0	0	0	0	0	0	0	0	0	0	1	1
VIM	0	0	0	0	0	0	0	0	0	0	0	0	0
VKORC1	0	0	0	0	0	0	0	0	0	0	0	0	0
WDR1	0	0	0	0	0	0	0	0	0	0	0	0	0
WFS1	1	1	0	0	0	0	0	0	0	0	0	0	0
AGA	0	0	0	0	0	0	0	0	0	0	0	0	0
ARAF	0	0	0	0	0	0	0	0	0	0	0	0	0
ARF6	0	0	0	0	0	0	0	0	0	0	0	0	0
ARFGAP3	0	0	0	0	0	0	0	0	0	0	0	0	0
ARPC1A	0	0	0	0	0	0	0	0	0	0	0	0	0
ARPC1B	0	0	0	0	0	0	0	0	0	0	0	0	0
ARPC2	0	0	0	0	0	0	0	0	0	0	0	0	0
ARPC4	0	0	0	0	0	0	0	0	0	0	0	0	0
ARPC5	0	0	0	0	0	0	0	0	0	0	0	0	0
ARPC5L	0	0	0	0	0	0	0	0	0	0	0	0	0
ATP6V0A1	0	0	0	0	0	0	0	0	0	0	0	0	0
CACNA2D1	0	0	0	0	0	0	0	0	0	0	0	0	0
CAPN2	0	0	0	0	0	0	0	0	0	0	0	0	0
CD99	0	0	0	0	0	0	0	0	0	0	0	0	0
CFL2	0	0	0	0	0	0	0	0	0	0	0	0	0
CHP	0	0	0	0	0	0	0	0	0	0	0	0	0
CTTN	0	0	0	0	0	0	0	0	0	0	0	0	0
CYFIP1	0	0	0	0	0	0	0	0	0	0	0	0	0
DNM3	0	0	0	0	0	0	0	0	0	0	0	0	0
DVL2	0	0	0	0	0	0	0	0	0	0	0	0	0
EHD4	0	0	0	0	0	0	0	0	0	0	0	0	0
EPHA2	0	0	0	0	0	0	0	0	0	0	0	0	0
EXOC7	0	0	0	0	0	0	0	0	0	0	0	0	0
FLNA	0	0	0	0	0	0	0	0	0	0	0	0	0
GSK3B	0	0	0	0	0	0	0	0	0	0	0	0	0
GYS1	0	0	0	0	0	0	0	0	0	0	0	0	0
HEXB	0	0	0	0	0	0	0	0	0	0	0	0	0
HSPG2	0	0	0	0	0	0	0	0	0	0	0	0	0
ITCH	0	0	0	0	0	0	0	0	0	0	0	0	0
LAMP1	0	0	0	0	0	0	0	0	0	0	0	0	0
LMO7	0	0	0	0	0	0	0	0	0	0	0	0	0
NCKAP1	0	0	0	0	0	0	0	0	0	0	0	0	0
PDCD6IP	0	0	0	0	0	0	0	0	0	0	0	0	0
PFN2	0	0	0	0	0	0	0	0	0	0	0	0	0
PIP4K2B	0	0	0	0	0	0	0	0	0	0	0	0	0
PLXNB2	0	0	0	0	0	0	0	0	0	0	0	0	0
PSME3	0	0	0	0	0	0	0	0	0	0	0	0	0
PYGB	0	0	0	0	0	0	0	0	0	0	0	0	0
RAB11FIP5	0	0	0	0	0	0	0	0	0	0	0	0	0
RAB3B	0	0	0	0	0	0	0	0	0	0	0	0	0
RAB4A	0	0	0	0	0	0	0	0	0	0	0	0	0
RAB5B	0	0	0	0	0	0	0	0	0	0	0	0	0
RPL22L1	0	0	0	0	0	0	0	0	0	0	0	0	0
RPS27L	0	0	0	0	0	0	0	0	0	0	0	0	0
SCARB2	0	0	0	0	0	0	0	0	0	0	0	0	0

NAME	REACTOME_INNATE_IMMUNE_SYSTEM	REACTOME_MHC_CLASS_II_ANTIGEN_PRESENTATION	REACTOME_IMMUNE_SYSTEM	REACTOME_ADAPTIVE_IMMUNE_SYSTEM	REACTOME_APOPTOSIS	REACTOME_CLASS_I_MHC_MEDIATED_ANTIGEN_PROCESSING_PRESENTATION	REACTOME_ANTIGEN_PROCESSING_UBIQUITINATION_PROTEASOME_DEGRADATION	REACTOME_ER_PHAGOSOME_PATHWAY	REACTOME_ANTIGEN_PROCESSING_CROSS_PRESENTATION	REACTOME_REGULATION_OF_MRNA_STABILITY_BY_PROTEINS_THAT_BIND_AU_RICH_ELEMENTS	REACTOME_DESTABILIZATION_OF_MRNA_BY_AUF1_HNRNP_D0	REACTOME_REGULATION_OF_ORNITHINE_DECARBOXYLASE_ODC	REACTOME_REGULATION_OF_MITOTIC_CELL_CYCLE
UGGT1	0	0	0	0	0	0	0	0	0	0	0	0	0
VAMP7	0	0	0	0	0	0	0	0	0	0	0	0	0
VBP1	0	0	0	0	0	0	0	0	0	0	0	0	0
VIM	0	0	0	0	1	0	0	0	0	0	0	0	0
VKORC1	0	0	0	0	0	0	0	0	0	0	0	0	0
WDR1	0	0	0	0	0	0	0	0	0	0	0	0	0
WFS1	0	0	0	0	0	0	0	0	0	0	0	0	0
AGA	0	0	0	0	0	0	0	0	0	0	0	0	0
ARAF	0	0	0	0	0	0	0	0	0	0	0	0	0
ARF6	0	0	0	0	0	0	0	0	0	0	0	0	0
ARFGAP3	0	0	0	0	0	0	0	0	0	0	0	0	0
ARPC1A	0	0	0	0	0	0	0	0	0	0	0	0	0
ARPC1B	0	0	0	0	0	0	0	0	0	0	0	0	0
ARPC2	0	0	0	0	0	0	0	0	0	0	0	0	0
ARPC4	0	0	0	0	0	0	0	0	0	0	0	0	0
ARPC5	0	0	0	0	0	0	0	0	0	0	0	0	0
ARPC5L	0	0	0	0	0	0	0	0	0	0	0	0	0
ATP6V0A1	0	0	0	0	0	0	0	0	0	0	0	0	0
CACNA2D1	0	0	0	0	0	0	0	0	0	0	0	0	0
CAPN2	0	0	0	0	0	0	0	0	0	0	0	0	0
CD99	0	0	0	0	0	0	0	0	0	0	0	0	0
CFL2	0	0	0	0	0	0	0	0	0	0	0	0	0
CHP	0	0	0	0	0	0	0	0	0	0	0	0	0
CTTN	0	0	0	0	0	0	0	0	0	0	0	0	0
CYFIP1	0	0	0	0	0	0	0	0	0	0	0	0	0
DNM3	0	0	0	0	0	0	0	0	0	0	0	0	0
DVL2	0	0	0	0	0	0	0	0	0	0	0	0	0
EHD4	0	0	0	0	0	0	0	0	0	0	0	0	0
EPHA2	0	0	0	0	0	0	0	0	0	0	0	0	0
EXOC7	0	0	0	0	0	0	0	0	0	0	0	0	0
FLNA	0	0	0	0	0	0	0	0	0	0	0	0	0
GSK3B	0	0	0	0	0	0	0	0	0	0	0	0	0
GYS1	0	0	0	0	0	0	0	0	0	0	0	0	0
HEXB	0	0	0	0	0	0	0	0	0	0	0	0	0
HSPG2	0	0	0	0	0	0	0	0	0	0	0	0	0
ITCH	0	0	0	0	0	0	0	0	0	0	0	0	0
LAMP1	0	0	0	0	0	0	0	0	0	0	0	0	0
LMO7	0	0	0	0	0	0	0	0	0	0	0	0	0
NCKAP1	0	0	0	0	0	0	0	0	0	0	0	0	0
PDCD6IP	0	0	0	0	0	0	0	0	0	0	0	0	0
PFN2	0	0	0	0	0	0	0	0	0	0	0	0	0
PIP4K2B	0	0	0	0	0	0	0	0	0	0	0	0	0
PLXNB2	0	0	0	0	0	0	0	0	0	0	0	0	0
PSME3	0	0	0	0	0	0	0	0	0	0	0	0	0
PYGB	0	0	0	0	0	0	0	0	0	0	0	0	0
RAB11FIP5	0	0	0	0	0	0	0	0	0	0	0	0	0
RAB3B	0	0	0	0	0	0	0	0	0	0	0	0	0
RAB4A	0	0	0	0	0	0	0	0	0	0	0	0	0
RAB5B	0	0	0	0	0	0	0	0	0	0	0	0	0
RPL22L1	0	0	0	0	0	0	0	0	0	0	0	0	0
RPS27L	0	0	0	0	0	0	0	0	0	0	0	0	0
SCARB2	0	0	0	0	0	0	0	0	0	0	0	0	0

NAME	REACTOME_CDT1_ASSOCIATION_WITH_THE_CDC6_ORC_ORIGIN_COMPLEX	REACTOME_APC_C_CDC20_MEDIATED_DEGRADATION_OF_MITOTIC_PROTEINS	REACTOME_APC_C_CDH1_MEDIATED_DEGRADATION_OF_CDC20_AND_OTHER_APC_C_CDH1_TARGETED_PROTEINS_IN_LATE_MITOSIS_EARLY_G1	REACTOME_CYCLIN_E_ASSOCIATED_EVENTS_DURING_G1_S_TRANSITION_	REACTOME_SCFSKP2_MEDIATED_DEGRADATION_OF_P27_P21	REACTOME_AUTODEGRADATION_OF_CDH1_BY_CDH1_APC_C	REACTOME_SCF_BETA_TRCP_MEDIATED_DEGRADATION_OF_EMI1	REACTOME_P53_DEPENDENT_G1_DNA_DAMAGE_RESPONSE	REACTOME_CDK_MEDIATED_PHOSPHORYLATION_AND_REMOVAL_OF_CDC6	REACTOME_AUTODEGRADATION_OF_THE_E3_UBIQUITIN_LIGASE_COP1	REACTOME_P53_INDEPENDENT_G1_S_DNA_DAMAGE_CHECKPOINT	REACTOME_VIF_MEDIATED_DEGRADATION_OF_APOBEC3G	REACTOME_CROSS_PRESENTATION_OF_SOLUBLE_EXOGENOUS_ANTIGENS_ENDOSOMES
UGGT1	0	0	0	0	0	0	0	0	0	0	0	0	0
VAMP7	0	0	0	0	0	0	0	0	0	0	0	0	0
VBP1	0	0	0	0	0	0	0	0	0	0	0	0	0
VIM	0	0	0	0	0	0	0	0	0	0	0	0	0
VKORC1	0	0	0	0	0	0	0	0	0	0	0	0	0
WDR1	0	0	0	0	0	0	0	0	0	0	0	0	0
WFS1	0	0	0	0	0	0	0	0	0	0	0	0	0
AGA	0	0	0	0	0	0	0	0	0	0	0	0	0
ARAF	0	0	0	0	0	0	0	0	0	0	0	0	0
ARF6	0	0	0	0	0	0	0	0	0	0	0	0	0
ARFGAP3	0	0	0	0	0	0	0	0	0	0	0	0	0
ARPC1A	0	0	0	0	0	0	0	0	0	0	0	0	0
ARPC1B	0	0	0	0	0	0	0	0	0	0	0	0	0
ARPC2	0	0	0	0	0	0	0	0	0	0	0	0	0
ARPC4	0	0	0	0	0	0	0	0	0	0	0	0	0
ARPC5	0	0	0	0	0	0	0	0	0	0	0	0	0
ARPC5L	0	0	0	0	0	0	0	0	0	0	0	0	0
ATP6V0A1	0	0	0	0	0	0	0	0	0	0	0	0	0
CACNA2D1	0	0	0	0	0	0	0	0	0	0	0	0	0
CAPN2	0	0	0	0	0	0	0	0	0	0	0	0	0
CD99	0	0	0	0	0	0	0	0	0	0	0	0	0
CFL2	0	0	0	0	0	0	0	0	0	0	0	0	0
CHP	0	0	0	0	0	0	0	0	0	0	0	0	0
CTTN	0	0	0	0	0	0	0	0	0	0	0	0	0
CYFIP1	0	0	0	0	0	0	0	0	0	0	0	0	0
DNM3	0	0	0	0	0	0	0	0	0	0	0	0	0
DVL2	0	0	0	0	0	0	0	0	0	0	0	0	0
EHD4	0	0	0	0	0	0	0	0	0	0	0	0	0
EPHA2	0	0	0	0	0	0	0	0	0	0	0	0	0
EXOC7	0	0	0	0	0	0	0	0	0	0	0	0	0
FLNA	0	0	0	0	0	0	0	0	0	0	0	0	0
GSK3B	0	0	0	0	0	0	0	0	0	0	0	0	0
GYS1	0	0	0	0	0	0	0	0	0	0	0	0	0
HEXB	0	0	0	0	0	0	0	0	0	0	0	0	0
HSPG2	0	0	0	0	0	0	0	0	0	0	0	0	0
ITCH	0	0	0	0	0	0	0	0	0	0	0	0	0
LAMP1	0	0	0	0	0	0	0	0	0	0	0	0	0
LMO7	0	0	0	0	0	0	0	0	0	0	0	0	0
NCKAP1	0	0	0	0	0	0	0	0	0	0	0	0	0
PDCD6IP	0	0	0	0	0	0	0	0	0	0	0	0	0
PFN2	0	0	0	0	0	0	0	0	0	0	0	0	0
PIP4K2B	0	0	0	0	0	0	0	0	0	0	0	0	0
PLXNB2	0	0	0	0	0	0	0	0	0	0	0	0	0
PSME3	0	0	0	0	0	0	0	0	0	0	0	0	0
PYGB	0	0	0	0	0	0	0	0	0	0	0	0	0
RAB11FIP5	0	0	0	0	0	0	0	0	0	0	0	0	0
RAB3B	0	0	0	0	0	0	0	0	0	0	0	0	0
RAB4A	0	0	0	0	0	0	0	0	0	0	0	0	0
RAB5B	0	0	0	0	0	0	0	0	0	0	0	0	0
RPL22L1	0	0	0	0	0	0	0	0	0	0	0	0	0
RPS27L	0	0	0	0	0	0	0	0	0	0	0	0	0
SCARB2	0	0	0	0	0	0	0	0	0	0	0	0	0

NAME	REACTOME_ING_BY_WNT	REACTOME_REGULATION_OF_APOPTOSIS	REACTOME_ACTIVATION_OF_NF_KAPPAB_IN_B_CELLS	REACTOME_DOWNSTREAM_ING_EVENTS_OF_B_CELL_RECEPTOR_BCR	REACTOME_ING_BY_THE_B_CELL_RECEPTOR_BCR	REACTOME_METABOLISM_OF_MRNA	REACTOME_METABOLISM_OF_PROTEINS	REACTOME_FORMATION_OF_THE_TERNARY_COMPLEX_AND_SUBSEQUENTLY_THE_43S_COMPLEX	REACTOME_ACTIVATION_OF_THE_MRNA_UPON_BINDING_OF_THE_CAP_BINDING_COMPLEX_AND_EIFS_AND_SUBSEQUENT_BINDING_TO_43S	REACTOME_SRP_DEPENDENT_COTRANSLATIONAL_PROTEIN_TARGETING_TO_MEMBRANE	REACTOME_INFLUENZA_VIRAL_RNA_TRANSCRIPTION_AND_REPLICATION	REACTOME_NONSENSE_MEDIATED_DECAY_ENHANCED_BY_THE_EXON_JUNCTION_COMPLEX	REACTOME_PEPTIDE_CHAIN_ELONGATION
UGGT1	0	0	0	0	0	0	1	0	0	0	0	0	0
VAMP7	0	0	0	0	0	0	0	0	0	0	0	0	0
VBP1	0	0	0	0	0	0	1	0	0	0	0	0	0
VIM	0	0	0	0	0	0	0	0	0	0	0	0	0
VKORC1	0	0	0	0	0	0	1	0	0	0	0	0	0
WDR1	0	0	0	0	0	0	0	0	0	0	0	0	0
WFS1	0	0	0	0	0	0	0	0	0	0	0	0	0
AGA	0	0	0	0	0	0	0	0	0	0	0	0	0
ARAF	0	0	0	0	0	0	0	0	0	0	0	0	0
ARF6	0	0	0	0	0	0	0	0	0	0	0	0	0
ARFGAP3	0	0	0	0	0	0	0	0	0	0	0	0	0
ARPC1A	0	0	0	0	0	0	0	0	0	0	0	0	0
ARPC1B	0	0	0	0	0	0	0	0	0	0	0	0	0
ARPC2	0	0	0	0	0	0	0	0	0	0	0	0	0
ARPC4	0	0	0	0	0	0	0	0	0	0	0	0	0
ARPC5	0	0	0	0	0	0	0	0	0	0	0	0	0
ARPC5L	0	0	0	0	0	0	0	0	0	0	0	0	0
ATP6V0A1	0	0	0	0	0	0	0	0	0	0	0	0	0
CACNA2D1	0	0	0	0	0	0	0	0	0	0	0	0	0
CAPN2	0	0	0	0	0	0	0	0	0	0	0	0	0
CD99	0	0	0	0	0	0	0	0	0	0	0	0	0
CFL2	0	0	0	0	0	0	0	0	0	0	0	0	0
CHP	0	0	0	0	0	0	0	0	0	0	0	0	0
CTTN	0	0	0	0	0	0	0	0	0	0	0	0	0
CYFIP1	0	0	0	0	0	0	0	0	0	0	0	0	0
DNM3	0	0	0	0	0	0	0	0	0	0	0	0	0
DVL2	0	0	0	0	0	0	0	0	0	0	0	0	0
EHD4	0	0	0	0	0	0	0	0	0	0	0	0	0
EPHA2	0	0	0	0	0	0	0	0	0	0	0	0	0
EXOC7	0	0	0	0	0	0	0	0	0	0	0	0	0
FLNA	0	0	0	0	0	0	0	0	0	0	0	0	0
GSK3B	0	0	0	0	0	0	0	0	0	0	0	0	0
GYS1	0	0	0	0	0	0	0	0	0	0	0	0	0
HEXB	0	0	0	0	0	0	0	0	0	0	0	0	0
HSPG2	0	0	0	0	0	0	0	0	0	0	0	0	0
ITCH	0	0	0	0	0	0	0	0	0	0	0	0	0
LAMP1	0	0	0	0	0	0	0	0	0	0	0	0	0
LMO7	0	0	0	0	0	0	0	0	0	0	0	0	0
NCKAP1	0	0	0	0	0	0	0	0	0	0	0	0	0
PDCD6IP	0	0	0	0	0	0	0	0	0	0	0	0	0
PFN2	0	0	0	0	0	0	0	0	0	0	0	0	0
PIP4K2B	0	0	0	0	0	0	0	0	0	0	0	0	0
PLXNB2	0	0	0	0	0	0	0	0	0	0	0	0	0
PSME3	0	0	0	0	0	0	0	0	0	0	0	0	0
PYGB	0	0	0	0	0	0	0	0	0	0	0	0	0
RAB11FIP5	0	0	0	0	0	0	0	0	0	0	0	0	0
RAB3B	0	0	0	0	0	0	0	0	0	0	0	0	0
RAB4A	0	0	0	0	0	0	0	0	0	0	0	0	0
RAB5B	0	0	0	0	0	0	0	0	0	0	0	0	0
RPL22L1	0	0	0	0	0	0	0	0	0	0	0	0	0
RPS27L	0	0	0	0	0	0	0	0	0	0	0	0	0
SCARB2	0	0	0	0	0	0	0	0	0	0	0	0	0

NAME	REACTOME_3_UTR_MEDIATED_TRANSLATIONAL_REGULATION	REACTOME_TRANSLATION	REACTOME_CELL_SURFACE_INTERACTIONS_AT_THE_VASCULAR_WALL	REACTOME_INTEGRIN_CELL_SURFACE_INTERACTIONS	REACTOME_RESPONSE_TO_ELEVATED_PLATELET_CYTOSOLIC_CA2_	REACTOME_HEMOSTASIS	REACTOME_REGULATION_OF_INSULIN_SECRETION	REACTOME_INTEGRATION_OF_ENERGY_METABOLISM	REACTOME_GPCR_DOWNSTREAM_ING	REACTOME_ING_BY_GPCR	REACTOME_ING_BY_ILS	REACTOME_ING_BY_FGFR_IN_DISEASE	REACTOME_ING_BY_ERBB2
UGGT1	0	0	0	0	0	0	0	0	0	0	0	0	0
VAMP7	0	0	0	0	0	0	0	0	0	0	0	0	0
VBP1	0	0	0	0	0	0	0	0	0	0	0	0	0
VIM	0	0	0	0	0	0	0	0	0	0	0	0	0
VKORC1	0	0	0	0	0	0	0	0	0	0	0	0	0
WDR1	0	0	0	0	1	1	0	0	0	0	0	0	0
WFS1	0	0	0	0	0	0	0	0	0	0	0	0	0
AGA	0	0	0	0	0	0	0	0	0	0	0	0	0
ARAF	0	0	0	0	0	0	0	0	0	0	0	0	0
ARF6	0	0	0	0	0	0	0	0	0	0	0	0	0
ARFGAP3	0	0	0	0	0	0	0	0	0	0	0	0	0
ARPC1A	0	0	0	0	0	0	0	0	0	0	0	0	0
ARPC1B	0	0	0	0	0	0	0	0	0	0	0	0	0
ARPC2	0	0	0	0	0	0	0	0	0	0	0	0	0
ARPC4	0	0	0	0	0	0	0	0	0	0	0	0	0
ARPC5	0	0	0	0	0	0	0	0	0	0	0	0	0
ARPC5L	0	0	0	0	0	0	0	0	0	0	0	0	0
ATP6V0A1	0	0	0	0	0	0	0	0	0	0	0	0	0
CACNA2D1	0	0	0	0	0	0	0	0	0	0	0	0	0
CAPN2	0	0	0	0	0	0	0	0	0	0	0	0	0
CD99	0	0	0	0	0	0	0	0	0	0	0	0	0
CFL2	0	0	0	0	0	0	0	0	0	0	0	0	0
CHP	0	0	0	0	0	0	0	0	0	0	0	0	0
CTTN	0	0	0	0	0	0	0	0	0	0	0	0	0
CYFIP1	0	0	0	0	0	0	0	0	0	0	0	0	0
DNM3	0	0	0	0	0	0	0	0	0	0	0	0	0
DVL2	0	0	0	0	0	0	0	0	0	0	0	0	0
EHD4	0	0	0	0	0	0	0	0	0	0	0	0	0
EPHA2	0	0	0	0	0	0	0	0	0	0	0	0	0
EXOC7	0	0	0	0	0	0	0	0	0	0	0	0	0
FLNA	0	0	0	0	0	0	0	0	0	0	0	0	0
GSK3B	0	0	0	0	0	0	0	0	0	0	0	0	0
GYS1	0	0	0	0	0	0	0	0	0	0	0	0	0
HEXB	0	0	0	0	0	0	0	0	0	0	0	0	0
HSPG2	0	0	0	0	0	0	0	0	0	0	0	0	0
ITCH	0	0	0	0	0	0	0	0	0	0	0	0	0
LAMP1	0	0	0	0	0	0	0	0	0	0	0	0	0
LMO7	0	0	0	0	0	0	0	0	0	0	0	0	0
NCKAP1	0	0	0	0	0	0	0	0	0	0	0	0	0
PDCD6IP	0	0	0	0	0	0	0	0	0	0	0	0	0
PFN2	0	0	0	0	0	0	0	0	0	0	0	0	0
PIP4K2B	0	0	0	0	0	0	0	0	0	0	0	0	0
PLXNB2	0	0	0	0	0	0	0	0	0	0	0	0	0
PSME3	0	0	0	0	0	0	0	0	0	0	0	0	0
PYGB	0	0	0	0	0	0	0	0	0	0	0	0	0
RAB11FIP5	0	0	0	0	0	0	0	0	0	0	0	0	0
RAB3B	0	0	0	0	0	0	0	0	0	0	0	0	0
RAB4A	0	0	0	0	0	0	0	0	0	0	0	0	0
RAB5B	0	0	0	0	0	0	0	0	0	0	0	0	0
RPL22L1	0	0	0	0	0	0	0	0	0	0	0	0	0
RPS27L	0	0	0	0	0	0	0	0	0	0	0	0	0
SCARB2	0	0	0	0	0	0	0	0	0	0	0	0	0

NAME	REACTOME_ING_BY_FGFR	REACTOME_DOWNSTREAM__TRANSDUCTION	REACTOME_LING_BY_NGF	REACTOME_NGF_LING_VIA_TRKA_FROM_THE_PLASMA_MEMBRANE	REACTOME_ING_BY_EGFR_IN_CANCER	REACTOME_ING_BY_PDGF	REACTOME_SEMAPHORIN_INTERACTIONS	REACTOME_L1CAM_INTERACTIONS	REACTOME_DEVELOPMENTAL_BIOLOGY	REACTOME_AXON_GUIDANCE	KEGG_ERBB_ING_PATHWAY	KEGG_INSULIN_ING_PATHWAY
UGGT1	0	0	0	0	0	0	0	0	0	0	0	0
VAMP7	0	0	0	0	0	0	0	0	0	0	0	0
VBP1	0	0	0	0	0	0	0	0	0	0	0	0
VIM	0	0	0	0	0	0	0	0	0	0	0	0
VKORC1	0	0	0	0	0	0	0	0	0	0	0	0
WDR1	0	0	0	0	0	0	0	0	0	0	0	0
WFS1	0	0	0	0	0	0	0	0	0	0	0	0
AGA	0	0	0	0	0	0	0	0	0	0	0	0
ARAF	0	0	0	0	0	0	0	0	0	0	0	0
ARF6	0	0	0	0	0	0	0	0	0	0	0	0
ARFGAP3	0	0	0	0	0	0	0	0	0	0	0	0
ARPC1A	0	0	0	0	0	0	0	0	0	0	0	0
ARPC1B	0	0	0	0	0	0	0	0	0	0	0	0
ARPC2	0	0	0	0	0	0	0	0	0	0	0	0
ARPC4	0	0	0	0	0	0	0	0	0	0	0	0
ARPC5	0	0	0	0	0	0	0	0	0	0	0	0
ARPC5L	0	0	0	0	0	0	0	0	0	0	0	0
ATP6V0A1	0	0	0	0	0	0	0	0	0	0	0	0
CACNA2D1	0	0	0	0	0	0	0	0	0	0	0	0
CAPN2	0	0	0	0	0	0	0	0	0	0	0	0
CD99	0	0	0	0	0	0	0	0	0	0	0	0
CFL2	0	0	0	0	0	0	0	0	0	0	0	0
CHP	0	0	0	0	0	0	0	0	0	0	0	0
CTTN	0	0	0	0	0	0	0	0	0	0	0	0
CYFIP1	0	0	0	0	0	0	0	0	0	0	0	0
DNM3	0	0	0	0	0	0	0	0	0	0	0	0
DVL2	0	0	0	0	0	0	0	0	0	0	0	0
EHD4	0	0	0	0	0	0	0	0	0	0	0	0
EPHA2	0	0	0	0	0	0	0	0	0	0	0	0
EXOC7	0	0	0	0	0	0	0	0	0	0	0	0
FLNA	0	0	0	0	0	0	0	0	0	0	0	0
GSK3B	0	0	0	0	0	0	0	0	0	0	0	0
GYS1	0	0	0	0	0	0	0	0	0	0	0	0
HEXB	0	0	0	0	0	0	0	0	0	0	0	0
HSPG2	0	0	0	0	0	0	0	0	0	0	0	0
ITCH	0	0	0	0	0	0	0	0	0	0	0	0
LAMP1	0	0	0	0	0	0	0	0	0	0	0	0
LMO7	0	0	0	0	0	0	0	0	0	0	0	0
NCKAP1	0	0	0	0	0	0	0	0	0	0	0	0
PDCD6IP	0	0	0	0	0	0	0	0	0	0	0	0
PFN2	0	0	0	0	0	0	0	0	0	0	0	0
PIP4K2B	0	0	0	0	0	0	0	0	0	0	0	0
PLXNB2	0	0	0	0	0	0	0	0	0	0	0	0
PSME3	0	0	0	0	0	0	0	0	0	0	0	0
PYGB	0	0	0	0	0	0	0	0	0	0	0	0
RAB11FIP5	0	0	0	0	0	0	0	0	0	0	0	0
RAB3B	0	0	0	0	0	0	0	0	0	0	0	0
RAB4A	0	0	0	0	0	0	0	0	0	0	0	0
RAB5B	0	0	0	0	0	0	0	0	0	0	0	0
RPL22L1	0	0	0	0	0	0	0	0	0	0	0	0
RPS27L	0	0	0	0	0	0	0	0	0	0	0	0
SCARB2	0	0	0	0	0	0	0	0	0	0	0	0

NAME	KEGG_PATHWAYS_IN_CANCER	KEGG_PROSTATE_CANCER	KEGG_CHEMOKINE_ING_PATHWAY	KEGG_NEUROTROPHIN_ING_PATHWAY	KEGG_MAPK_ING_PATHWAY	KEGG_ARRHYTHMOGENIC_RIGHT_VENTRICULAR_CARDIOMYOPATHY_ARVC	KEGG_ECM_RECEPTOR_INTERACTION	KEGG_FOCAL_ADHESION
UGGT1	0	0	0	0	0	0	0	0
VAMP7	0	0	0	0	0	0	0	0
VBP1	0	0	0	0	0	0	0	0
VIM	0	0	0	0	0	0	0	0
VKORC1	0	0	0	0	0	0	0	0
WDR1	0	0	0	0	0	0	0	0
WFS1	0	0	0	0	0	0	0	0
AGA	0	0	0	0	0	0	0	0
ARAF	0	0	0	0	0	0	0	0
ARF6	0	0	0	0	0	0	0	0
ARFGAP3	0	0	0	0	0	0	0	0
ARPC1A	0	0	0	0	0	0	0	0
ARPC1B	0	0	0	0	0	0	0	0
ARPC2	0	0	0	0	0	0	0	0
ARPC4	0	0	0	0	0	0	0	0
ARPC5	0	0	0	0	0	0	0	0
ARPC5L	0	0	0	0	0	0	0	0
ATP6V0A1	0	0	0	0	0	0	0	0
CACNA2D1	0	0	0	0	0	0	0	0
CAPN2	0	0	0	0	0	0	0	0
CD99	0	0	0	0	0	0	0	0
CFL2	0	0	0	0	0	0	0	0
CHP	0	0	0	0	0	0	0	0
CTTN	0	0	0	0	0	0	0	0
CYFIP1	0	0	0	0	0	0	0	0
DNM3	0	0	0	0	0	0	0	0
DVL2	0	0	0	0	0	0	0	0
EHD4	0	0	0	0	0	0	0	0
EPHA2	0	0	0	0	0	0	0	0
EXOC7	0	0	0	0	0	0	0	0
FLNA	0	0	0	0	0	0	0	0
GSK3B	0	0	0	0	0	0	0	0
GYS1	0	0	0	0	0	0	0	0
HEXB	0	0	0	0	0	0	0	0
HSPG2	0	0	0	0	0	0	0	0
ITCH	0	0	0	0	0	0	0	0
LAMP1	0	0	0	0	0	0	0	0
LMO7	0	0	0	0	0	0	0	0
NCKAP1	0	0	0	0	0	0	0	0
PDCD6IP	0	0	0	0	0	0	0	0
PFN2	0	0	0	0	0	0	0	0
PIP4K2B	0	0	0	0	0	0	0	0
PLXNB2	0	0	0	0	0	0	0	0
PSME3	0	0	0	0	0	0	0	0
PYGB	0	0	0	0	0	0	0	0
RAB11FIP5	0	0	0	0	0	0	0	0
RAB3B	0	0	0	0	0	0	0	0
RAB4A	0	0	0	0	0	0	0	0
RAB5B	0	0	0	0	0	0	0	0
RPL22L1	0	0	0	0	0	0	0	0
RPS27L	0	0	0	0	0	0	0	0
SCARB2	0	0	0	0	0	0	0	0

Table S6 The differential proteins contribute to the enrichment of the significant pathways for the public dataset

Core enrichment proteins in down-regulated pathways of hESCs/HFs (hESCs vs HFs)

NAME	REACTOME_UNFOLDED_PROTEIN_RESPONSE	REACTOME_DIABETES_PATHWAYS	REACTOME_CELL_CELL_COMMUNICATION	REACTOME_GOLGI_ASSOCIATED_VESICLE_BIOGENESIS	REACTOME_TRANS_GOLGI_NETWORK_VESICLE_BUDDING	REACTOME_MEMBRANE_TRAFFICKING	REACTOME_NEURONAL_SYSTEM	REACTOME_TRANSMISSION_ACROSS_CHEMICAL_SYNAPSES	REACTOME_POST_TRANSLATIONAL_PROTEIN_MODIFICATION	REACTOME_ASPARAGINE_N_LINKED_GLYCOSYLATION	REACTOME_ING_BY_INSULIN_RECEPTOR	REACTOME_PREFOLDIN_MEDIATED_TRANSFER_OF_SUBSTRATE_TO_CCT_TRIC	REACTOME_PROTEIN_FOLDING
SH3GL1	0	0	0	0	0	0	0	0	0	0	0	0	0
SH3GLB1	0	0	0	0	0	0	0	0	0	0	0	0	0
TFG	0	0	0	0	0	0	0	0	0	0	0	0	0
THY1	0	0	0	0	0	0	0	0	0	0	0	0	0
YWHAQ	0	0	0	0	0	0	0	0	0	0	0	0	0
ZYX	0	0	0	0	0	0	0	0	0	0	0	0	0

NAME	REACTOME_INNATE_IMMUNE_SYSTEM	REACTOME_MHC_CLASS_II_ANTIGEN_PRESENTATION	REACTOME_IMMUNE_SYSTEM	REACTOME_ADAPTIVE_IMMUNE_SYSTEM	REACTOME_APOPTOSIS	REACTOME_CLASS_I_MHC_MEDIATED_ANTIGEN_PROCESSING_PRESENTATION	REACTOME_ANTIGEN_PROCESSING_UBIQUITINATION_PROTEASOME_DEGRADATION	REACTOME_ER_PHAGOSOME_PATHWAY	REACTOME_ANTIGEN_PROCESSING_CROSS_PRESENTATION	REACTOME_REGULATION_OF_MRNA_STABILITY_BY_PROTEINS_THAT_BIND_AU_RICH_ELEMENTS	REACTOME_DESTABILIZATION_OF_MRNA_BY_AUF1_HNRNP_D0	REACTOME_REGULATION_OF_ORNITHINE_DECARBOXYLASE_ODC	REACTOME_REGULATION_OF_MITOTIC_CELL_CYCLE
SH3GL1	0	0	0	0	0	0	0	0	0	0	0	0	0
SH3GLB1	0	0	0	0	0	0	0	0	0	0	0	0	0
TFG	0	0	0	0	0	0	0	0	0	0	0	0	0
THY1	0	0	0	0	0	0	0	0	0	0	0	0	0
YWHAQ	0	0	0	0	0	0	0	0	0	0	0	0	0
ZYX	0	0	0	0	0	0	0	0	0	0	0	0	0

NAME	REACTOME_CDT1_ASSOCIATION_WITH_THE_CDC6_ORC_ORIGIN_COMPLEX	REACTOME_APC_C_CDC20_MEDIATED_DEGRADATION_OF_MITOTIC_PROTEINS	REACTOME_APC_C_CDH1_MEDIATED_DEGRADATION_OF_CDC20_AND_OTHER_APC_C_CDH1_TARGETED_PROTEINS_IN_LATE_MITOSIS_EARLY_G1	REACTOME_CYCLIN_E_ASSOCIATED_EVENTS_DURING_G1_S_TRANSITION_	REACTOME_SCFSKP2_MEDIATED_DEGRADATION_OF_P27_P21	REACTOME_AUTODEGRADATION_OF_CDH1_BY_CDH1_APC_C	REACTOME_SCF_BETA_TRCP_MEDIATED_DEGRADATION_OF_EMI1	REACTOME_P53_DEPENDENT_G1_DNA_DAMAGE_RESPONSE	REACTOME_CDK_MEDIATED_PHOSPHORYLATION_AND_REMOVAL_OF_CDC6	REACTOME_AUTODEGRADATION_OF_THE_E3_UBIQUITIN_LIGASE_COP1	REACTOME_P53_INDEPENDENT_G1_S_DNA_DAMAGE_CHECKPOINT	REACTOME_VIF_MEDIATED_DEGRADATION_OF_APOBEC3G	REACTOME_CROSS_PRESENTATION_OF_SOLUBLE_EXOGENOUS_ANTIGENS_ENDOSOMES
SH3GL1	0	0	0	0	0	0	0	0	0	0	0	0	0
SH3GLB1	0	0	0	0	0	0	0	0	0	0	0	0	0
TFG	0	0	0	0	0	0	0	0	0	0	0	0	0
THY1	0	0	0	0	0	0	0	0	0	0	0	0	0
YWHAQ	0	0	0	0	0	0	0	0	0	0	0	0	0
ZYX	0	0	0	0	0	0	0	0	0	0	0	0	0

NAME	REACTOME_ING_BY_WNT	REACTOME_REGULATION_OF_APOPTOSIS	REACTOME_ACTIVATION_OF_NF_KAPPAB_IN_B_CELLS	REACTOME_DOWNSTREAM_ING_EVENTS_OF_B_CELL_RECEPTOR_BCR	REACTOME_ING_BY_THE_B_CELL_RECEPTOR_BCR	REACTOME_METABOLISM_OF_MRNA	REACTOME_METABOLISM_OF_PROTEINS	REACTOME_FORMATION_OF_THE_TERNARY_COMPLEX_AND_SUBSEQUENTLY_THE_43S_COMPLEX	REACTOME_ACTIVATION_OF_THE_MRNA_UPON_BINDING_OF_THE_CAP_BINDING_COMPLEX_AND_EIFS_AND_SUBSEQUENT_BINDING_TO_43S	REACTOME_SRP_DEPENDENT_COTRANSLATIONAL_PROTEIN_TARGETING_TO_MEMBRANE	REACTOME_INFLUENZA_VIRAL_RNA_TRANSCRIPTION_AND_REPLICATION	REACTOME_NONSENSE_MEDIATED_DECAY_ENHANCED_BY_THE_EXON_JUNCTION_COMPLEX	REACTOME_PEPTIDE_CHAIN_ELONGATION
SH3GL1	0	0	0	0	0	0	0	0	0	0	0	0	0
SH3GLB1	0	0	0	0	0	0	0	0	0	0	0	0	0
TFG	0	0	0	0	0	0	0	0	0	0	0	0	0
THY1	0	0	0	0	0	0	0	0	0	0	0	0	0
YWHAQ	0	0	0	0	0	0	0	0	0	0	0	0	0
ZYX	0	0	0	0	0	0	0	0	0	0	0	0	0

NAME	REACTOME_3_UTR_MEDIATED_TRANSLATIONAL_REGULATION	REACTOME_TRANSLATION	REACTOME_CELL_SURFACE_INTERACTIONS_AT_THE_VASCULAR_WALL	REACTOME_INTEGRIN_CELL_SURFACE_INTERACTIONS	REACTOME_RESPONSE_TO_ELEVATED_PLATELET_CYTOSOLIC_CA2_	REACTOME_HEMOSTASIS	REACTOME_REGULATION_OF_INSULIN_SECRETION	REACTOME_INTEGRATION_OF_ENERGY_METABOLISM	REACTOME_GPCR_DOWNSTREAM_ING	REACTOME_ING_BY_GPCR	REACTOME_ING_BY_ILS	REACTOME_ING_BY_FGFR_IN_DISEASE	REACTOME_ING_BY_ERBB2
SH3GL1	0	0	0	0	0	0	0	0	0	0	0	0	0
SH3GLB1	0	0	0	0	0	0	0	0	0	0	0	0	0
TFG	0	0	0	0	0	0	0	0	0	0	0	0	0
THY1	0	0	0	0	0	0	0	0	0	0	0	0	0
YWHAQ	0	0	0	0	0	0	0	0	0	0	0	0	0
ZYX	0	0	0	0	0	0	0	0	0	0	0	0	0

NAME	REACTOME_ING_BY_FGFR	REACTOME_DOWNSTREAM__TRANSDUCTION	REACTOME_LING_BY_NGF	REACTOME_NGF_LING_VIA_TRKA_FROM_THE_PLASMA_MEMBRANE	REACTOME_ING_BY_EGFR_IN_CANCER	REACTOME_ING_BY_PDGF	REACTOME_SEMAPHORIN_INTERACTIONS	REACTOME_L1CAM_INTERACTIONS	REACTOME_DEVELOPMENTAL_BIOLOGY	REACTOME_AXON_GUIDANCE	KEGG_ERBB_ING_PATHWAY	KEGG_INSULIN_ING_PATHWAY
SH3GL1	0	0	0	0	0	0	0	0	0	0	0	0
SH3GLB1	0	0	0	0	0	0	0	0	0	0	0	0
TFG	0	0	0	0	0	0	0	0	0	0	0	0
THY1	0	0	0	0	0	0	0	0	0	0	0	0
YWHAQ	0	0	0	0	0	0	0	0	0	0	0	0
ZYX	0	0	0	0	0	0	0	0	0	0	0	0

NAME	KEGG_PATHWAYS_IN_CANCER	KEGG_PROSTATE_CANCER	KEGG_CHEMOKINE_ING_PATHWAY	KEGG_NEUROTROPHIN_ING_PATHWAY	KEGG_MAPK_ING_PATHWAY	KEGG_ARRHYTHMOGENIC_RIGHT_VENTRICULAR_CARDIOMYOPATHY_ARVC	KEGG_ECM_RECEPTOR_INTERACTION	KEGG_FOCAL_ADHESION
SH3GL1	0	0	0	0	0	0	0	0
SH3GLB1	0	0	0	0	0	0	0	0
TFG	0	0	0	0	0	0	0	0
THY1	0	0	0	0	0	0	0	0
YWHAQ	0	0	0	0	0	0	0	0
ZYX	0	0	0	0	0	0	0	0

Table S6 The differential proteins contribute to the enrichment of the significant pathways for the public dataset

Core enrichment proteins in up-regulated pathways of hESCs/HFs (hESCs vs HFs)

NAME	REACTOME_MITOCHONDRIAL_PROTEIN_IMPORT	REACTOME_DEADENYLATION_DEPENDENT_MRNA_DECAY	REACTOME_FACTORS_INVOLVED_IN_MEGAKARYOCYTE_DEVELOPMENT_AND_PLATELET_PRODUCTION	REACTOME_DNA_REPAIR	REACTOME_NUCLEOTIDE_EXCISION_REPAIR	REACTOME_MEIOSIS	REACTOME_MITOTIC_PROMETAPHASE	REACTOME_CHROMOSOME_MAINTENANCE	REACTOME_TELOMERE_MAINTENANCE	REACTOME_CELL_CYCLE	REACTOME_CELL_CYCLE_MITOTIC	REACTOME_DNA_REPLICATION	REACTOME_MITOTIC_M_M_G1_PHASES
CCNB1	0	0	0	0	0	0	0	0	0	1	1	0	0
CCNB2	0	0	0	0	0	0	0	0	0	1	1	0	0
DDX23	0	0	0	0	0	0	0	0	0	0	0	0	0
EFTUD2	0	0	0	0	0	0	0	0	0	0	0	0	0
EHMT2	0	0	0	0	0	0	0	0	0	0	0	0	0
EIF4A3	0	1	0	0	0	0	0	0	0	0	0	0	0
EP300	0	0	1	0	0	0	0	0	0	0	0	0	0
EXOSC4	0	1	0	0	0	0	0	0	0	0	0	0	0
EXOSC5	0	1	0	0	0	0	0	0	0	0	0	0	0
EXOSC6	0	1	0	0	0	0	0	0	0	0	0	0	0
EXOSC7	0	1	0	0	0	0	0	0	0	0	0	0	0
EXOSC9	0	1	0	0	0	0	0	0	0	0	0	0	0
HDAC2	0	0	1	0	0	0	0	0	0	0	0	0	0
HNRNPA1	0	0	0	0	0	0	0	0	0	0	0	0	0
HNRNPA3	0	0	0	0	0	0	0	0	0	0	0	0	0
HNRNPC	0	0	0	0	0	0	0	0	0	0	0	0	0
HNRNPK	0	0	0	0	0	0	0	0	0	0	0	0	0
HNRNPM	0	0	0	0	0	0	0	0	0	0	0	0	0
HNRNPU	0	0	0	0	0	0	0	0	0	0	0	0	0
HSPA9	1	0	0	0	0	0	0	0	0	0	0	0	0
HSPD1	1	0	0	0	0	0	0	0	0	0	0	0	0
LIG1	0	0	0	1	1	0	0	1	1	1	1	1	0
LSM2	0	1	0	0	0	0	0	0	0	0	0	0	0
LSM3	0	1	0	0	0	0	0	0	0	0	0	0	0
LSM4	0	1	0	0	0	0	0	0	0	0	0	0	0
LSM6	0	1	0	0	0	0	0	0	0	0	0	0	0
MAGOH	0	0	0	0	0	0	0	0	0	0	0	0	0
MCM2	0	0	0	0	0	0	0	0	0	1	1	1	1
MCM3	0	0	0	0	0	0	0	0	0	1	1	1	1
MCM4	0	0	0	0	0	0	0	0	0	1	1	1	1
MCM6	0	0	0	0	0	0	0	0	0	1	1	1	1
MCM7	0	0	0	0	0	0	0	0	0	1	1	1	1
NCBP1	0	0	0	0	0	0	0	0	0	0	0	0	0
NHP2L1	0	0	0	0	0	0	0	0	0	0	0	0	0
ORC2	0	0	0	0	0	0	0	0	0	1	1	1	1
ORC3	0	0	0	0	0	0	0	0	0	1	1	1	1
PAPOLA	0	0	0	0	0	0	0	0	0	0	0	0	0
PARN	0	1	0	0	0	0	0	0	0	0	0	0	0
PCBP1	0	0	0	0	0	0	0	0	0	0	0	0	0
PHF5A	0	0	0	0	0	0	0	0	0	0	0	0	0
PLK1	0	0	0	0	0	0	1	0	0	1	1	1	1
POLA1	0	0	0	0	0	0	0	1	1	1	1	1	1
POLD1	0	0	0	1	1	0	0	1	1	1	1	1	0
POLD3	0	0	0	1	1	0	0	1	1	1	1	1	0
POLR1B	0	0	0	0	0	0	0	0	0	0	0	0	0
POLR2A	0	0	0	1	1	0	0	0	0	0	0	0	0
POLR2B	0	0	0	1	0	0	0	0	0	0	0	0	0
POLR2C	0	0	0	1	0	0	0	0	0	0	0	0	0
POLR2E	0	0	0	1	1	0	0	0	0	0	0	0	0
POLR2H	0	0	0	1	1	0	0	0	0	0	0	0	0
POLR2L	0	0	0	1	0	0	0	0	0	0	0	0	0
POLR3A	0	0	0	0	0	0	0	0	0	0	0	0	0
POLR3D	0	0	0	0	0	0	0	0	0	0	0	0	0
PRIM2	0	0	0	0	0	0	0	1	1	1	1	1	1
PRPF4	0	0	0	0	0	0	0	0	0	0	0	0	0
PRPF6	0	0	0	0	0	0	0	0	0	0	0	0	0

NAME	REACTOME_CELL_CYCLE_CHECKPOINTS	REACTOME_S_PHASE	REACTOME_SYNTHESIS_OF_DNA	REACTOME_G1_S_TRANSITION	REACTOME_MITOTIC_G1_G1_S_PHASES	REACTOME_RNA_POL_I_RNA_POL_III_AND_MITOCHONDRIAL_TRANSCRIPTION	REACTOME_MRNA_PROCESSING	REACTOME_PROCESSING_OF_CAPPED_INTRON_CONTAINING_PRE_MRNA	REACTOME_MRNA_SPLICING	REACTOME_MRNA_SPLICING_MINOR_PATHWAY	REACTOME_TRANSCRIPTION	REACTOME_RNA_POL_II_TRANSCRIPTION	REACTOME_CLEAVAGE_OF_GROWING_TRANSCRIPT_IN_THE_TERMINATION_REGION_
CCNB1	1	0	0	1	1	0	0	0	0	0	0	0	0
CCNB2	1	0	0	0	0	0	0	0	0	0	0	0	0
DDX23	0	0	0	0	0	0	1	1	1	1	0	0	0
EFTUD2	0	0	0	0	0	0	1	1	1	1	0	0	0
EHMT2	0	0	0	0	0	1	0	0	0	0	1	0	0
EIF4A3	0	0	0	0	0	0	0	0	0	0	0	0	0
EP300	0	0	0	0	0	0	0	0	0	0	0	0	0
EXOSC4	0	0	0	0	0	0	0	0	0	0	0	0	0
EXOSC5	0	0	0	0	0	0	0	0	0	0	0	0	0
EXOSC6	0	0	0	0	0	0	0	0	0	0	0	0	0
EXOSC7	0	0	0	0	0	0	0	0	0	0	0	0	0
EXOSC9	0	0	0	0	0	0	0	0	0	0	0	0	0
HDAC2	0	0	0	0	0	0	0	0	0	0	0	0	0
HNRNPA1	0	0	0	0	0	0	1	1	1	0	0	0	0
HNRNPA3	0	0	0	0	0	0	1	1	1	0	0	0	0
HNRNPC	0	0	0	0	0	0	1	1	1	0	0	0	0
HNRNPK	0	0	0	0	0	0	1	1	1	0	0	0	0
HNRNPM	0	0	0	0	0	0	1	1	1	0	0	0	0
HNRNPU	0	0	0	0	0	0	1	1	1	0	0	0	0
HSPA9	0	0	0	0	0	0	0	0	0	0	0	0	0
HSPD1	0	0	0	0	0	0	0	0	0	0	0	0	0
LIG1	0	1	1	0	0	0	0	0	0	0	0	0	0
LSM2	0	0	0	0	0	0	1	1	1	1	0	0	0
LSM3	0	0	0	0	0	0	0	0	0	0	0	0	0
LSM4	0	0	0	0	0	0	0	0	0	0	0	0	0
LSM6	0	0	0	0	0	0	0	0	0	0	0	0	0
MAGOH	0	0	0	0	0	0	1	1	1	0	1	1	1
MCM2	1	1	1	1	1	0	0	0	0	0	0	0	0
MCM3	1	1	1	1	1	0	0	0	0	0	0	0	0
MCM4	1	1	1	1	1	0	0	0	0	0	0	0	0
MCM6	1	1	1	1	1	0	0	0	0	0	0	0	0
MCM7	1	1	1	1	1	0	0	0	0	0	0	0	0
NCBP1	0	0	0	0	0	0	1	1	1	1	1	1	1
NHP2L1	0	0	0	0	0	0	1	1	1	1	0	0	0
ORC2	1	1	1	1	1	0	0	0	0	0	0	0	0
ORC3	1	1	1	1	1	0	0	0	0	0	0	0	0
PAPOLA	0	0	0	0	0	0	1	1	1	0	1	1	1
PARN	0	0	0	0	0	0	0	0	0	0	0	0	0
PCBP1	0	0	0	0	0	0	1	1	1	0	0	0	0
PHF5A	0	0	0	0	0	0	1	1	1	0	0	0	0
PLK1	0	0	0	0	0	0	0	0	0	0	0	0	0
POLA1	0	1	1	1	1	0	0	0	0	0	0	0	0
POLD1	0	1	1	0	0	0	0	0	0	0	0	0	0
POLD3	0	1	1	0	0	0	0	0	0	0	0	0	0
POLR1B	0	0	0	0	0	1	0	0	0	0	1	0	0
POLR2A	0	0	0	0	0	0	1	1	1	1	1	1	0
POLR2B	0	0	0	0	0	0	1	1	1	1	1	1	0
POLR2C	0	0	0	0	0	0	1	1	1	1	1	1	0
POLR2E	0	0	0	0	0	1	1	1	1	1	1	1	0
POLR2H	0	0	0	0	0	1	1	1	1	1	1	1	0
POLR2L	0	0	0	0	0	0	1	1	1	1	1	1	0
POLR3A	0	0	0	0	0	1	0	0	0	0	1	0	0
POLR3D	0	0	0	0	0	1	0	0	0	0	1	0	0
PRIM2	0	1	1	1	1	0	0	0	0	0	0	0	0
PRPF4	0	0	0	0	0	0	1	1	1	0	0	0	0
PRPF6	0	0	0	0	0	0	1	1	1	1	0	0	0

NAME	REACTOME_MRNA_3_END_PROCESSING	REACTOME_RNA_POL_II_PRE_TRANSCRIPTION_EVENTS	REACTOME_METABOLISM_OF_NON_CODING_RNA	REACTOME_TRANSPORT_OF_MATURE_TRANSCRIPT_TO_CYTOPLASM	REACTOME_TRANSPORT_OF_MATURE_MRNA_DERIVED_FROM_AN_INTRONLESS_TRANSCRIPT	REACTOME_INTERACTIONS_OF_VPR_WITH_HOST_CELLULAR_PROTEINS	REACTOME_GLUCOSE_TRANSPORT	REACTOME_REGULATION_OF_GLUCOKINASE_BY_GLUCOKINASE_REGULATORY_PROTEIN	REACTOME_TRANSPORT_OF_RIBONUCLEOPROTEINS_INTO_THE_HOST_NUCLEUS	REACTOME_NEP_NS2_INTERACTS_WITH_THE_CELLULAR_EXPORT_MACHINERY	REACTOME_SLC_MEDIATED_TRANSMEMBRANE_TRANSPORT	REACTOME_ANTIVIRAL_MECHANISM_BY_IFN_STIMULATED_GENES	REACTOME_HIV_LIFE_CYCLE
CCNB1	0	0	0	0	0	0	0	0	0	0	0	0	0
CCNB2	0	0	0	0	0	0	0	0	0	0	0	0	0
DDX23	0	0	0	0	0	0	0	0	0	0	0	0	0
EFTUD2	0	0	0	0	0	0	0	0	0	0	0	0	0
EHMT2	0	0	0	0	0	0	0	0	0	0	0	0	0
EIF4A3	0	0	0	0	0	0	0	0	0	0	0	1	0
EP300	0	0	0	0	0	0	0	0	0	0	0	0	0
EXOSC4	0	0	0	0	0	0	0	0	0	0	0	0	0
EXOSC5	0	0	0	0	0	0	0	0	0	0	0	0	0
EXOSC6	0	0	0	0	0	0	0	0	0	0	0	0	0
EXOSC7	0	0	0	0	0	0	0	0	0	0	0	0	0
EXOSC9	0	0	0	0	0	0	0	0	0	0	0	0	0
HDAC2	0	0	0	0	0	0	0	0	0	0	0	0	0
HNRNPA1	0	0	0	0	0	0	0	0	0	0	0	0	0
HNRNPA3	0	0	0	0	0	0	0	0	0	0	0	0	0
HNRNPC	0	0	0	0	0	0	0	0	0	0	0	0	0
HNRNPK	0	0	0	0	0	0	0	0	0	0	0	0	0
HNRNPM	0	0	0	0	0	0	0	0	0	0	0	0	0
HNRNPU	0	0	0	0	0	0	0	0	0	0	0	0	0
HSPA9	0	0	0	0	0	0	0	0	0	0	0	0	0
HSPD1	0	0	0	0	0	0	0	0	0	0	0	0	0
LIG1	0	0	0	0	0	0	0	0	0	0	0	0	1
LSM2	0	0	0	0	0	0	0	0	0	0	0	0	0
LSM3	0	0	0	0	0	0	0	0	0	0	0	0	0
LSM4	0	0	0	0	0	0	0	0	0	0	0	0	0
LSM6	0	0	0	0	0	0	0	0	0	0	0	0	0
MAGOH	1	0	0	1	0	0	0	0	0	0	0	0	0
MCM2	0	0	0	0	0	0	0	0	0	0	0	0	0
MCM3	0	0	0	0	0	0	0	0	0	0	0	0	0
MCM4	0	0	0	0	0	0	0	0	0	0	0	0	0
MCM6	0	0	0	0	0	0	0	0	0	0	0	0	0
MCM7	0	0	0	0	0	0	0	0	0	0	0	0	0
NCBP1	1	1	1	1	1	0	0	0	0	0	0	0	1
NHP2L1	0	0	0	0	0	0	0	0	0	0	0	0	0
ORC2	0	0	0	0	0	0	0	0	0	0	0	0	0
ORC3	0	0	0	0	0	0	0	0	0	0	0	0	0
PAPOLA	1	0	0	0	0	0	0	0	0	0	0	0	0
PARN	0	0	0	0	0	0	0	0	0	0	0	0	0
PCBP1	0	0	0	0	0	0	0	0	0	0	0	0	0
PHF5A	0	0	0	0	0	0	0	0	0	0	0	0	0
PLK1	0	0	0	0	0	0	0	0	0	0	0	0	0
POLA1	0	0	0	0	0	0	0	0	0	0	0	0	0
POLD1	0	0	0	0	0	0	0	0	0	0	0	0	0
POLD3	0	0	0	0	0	0	0	0	0	0	0	0	0
POLR1B	0	0	0	0	0	0	0	0	0	0	0	0	0
POLR2A	0	1	0	0	0	0	0	0	0	0	0	0	1
POLR2B	0	1	0	0	0	0	0	0	0	0	0	0	1
POLR2C	0	1	0	0	0	0	0	0	0	0	0	0	1
POLR2E	0	1	0	0	0	0	0	0	0	0	0	0	1
POLR2H	0	1	0	0	0	0	0	0	0	0	0	0	1
POLR2L	0	1	0	0	0	0	0	0	0	0	0	0	1
POLR3A	0	0	0	0	0	0	0	0	0	0	0	0	0
POLR3D	0	0	0	0	0	0	0	0	0	0	0	0	0
PRIM2	0	0	0	0	0	0	0	0	0	0	0	0	0
PRPF4	0	0	0	0	0	0	0	0	0	0	0	0	0
PRPF6	0	0	0	0	0	0	0	0	0	0	0	0	0

NAME	REACTOME_HIV_INFECTION	KEGG_SPLICEOSOME_	KEGG_RNA_DEGRADATION_	KEGG_ARGININE_AND_PROLINE_METABOLISM_	KEGG_FATTY_ACID_METABOLISM_	KEGG_VALINE_LEUCINE_AND_ISOLEUCINE_DEGRADATION_	KEGG_LYSINE_DEGRADATION_	KEGG_PYRIMIDINE_METABOLISM_	KEGG_DNA_REPLICATION_	KEGG_CELL_CYCLE_	KEGG_OOCYTE_MEIOSIS_
CCNB1	0	0	0	0	0	0	0	0	0	1	1
CCNB2	0	0	0	0	0	0	0	0	0	1	1
DDX23	0	1	0	0	0	0	0	0	0	0	0
EFTUD2	0	1	0	0	0	0	0	0	0	0	0
EHMT2	0	0	0	0	0	0	1	0	0	0	0
EIF4A3	0	1	0	0	0	0	0	0	0	0	0
EP300	0	0	0	0	0	0	0	0	0	1	0
EXOSC4	0	0	1	0	0	0	0	0	0	0	0
EXOSC5	0	0	1	0	0	0	0	0	0	0	0
EXOSC6	0	0	1	0	0	0	0	0	0	0	0
EXOSC7	0	0	1	0	0	0	0	0	0	0	0
EXOSC9	0	0	1	0	0	0	0	0	0	0	0
HDAC2	0	0	0	0	0	0	0	0	0	1	0
HNRNPA1	0	1	0	0	0	0	0	0	0	0	0
HNRNPA3	0	1	0	0	0	0	0	0	0	0	0
HNRNPC	0	1	0	0	0	0	0	0	0	0	0
HNRNPK	0	1	0	0	0	0	0	0	0	0	0
HNRNPM	0	1	0	0	0	0	0	0	0	0	0
HNRNPU	0	1	0	0	0	0	0	0	0	0	0
HSPA9	0	0	1	0	0	0	0	0	0	0	0
HSPD1	0	0	1	0	0	0	0	0	0	0	0
LIG1	1	0	0	0	0	0	0	0	1	0	0
LSM2	0	1	1	0	0	0	0	0	0	0	0
LSM3	0	1	1	0	0	0	0	0	0	0	0
LSM4	0	1	1	0	0	0	0	0	0	0	0
LSM6	0	1	1	0	0	0	0	0	0	0	0
MAGOH	0	1	0	0	0	0	0	0	0	0	0
MCM2	0	0	0	0	0	0	0	0	1	1	0
MCM3	0	0	0	0	0	0	0	0	1	1	0
MCM4	0	0	0	0	0	0	0	0	1	0	0
MCM6	0	0	0	0	0	0	0	0	1	1	0
MCM7	0	0	0	0	0	0	0	0	1	1	0
NCBP1	1	1	0	0	0	0	0	0	0	0	0
NHP2L1	0	1	0	0	0	0	0	0	0	0	0
ORC2	0	0	0	0	0	0	0	0	0	1	0
ORC3	0	0	0	0	0	0	0	0	0	1	0
PAPOLA	0	0	1	0	0	0	0	0	0	0	0
PARN	0	0	1	0	0	0	0	0	0	0	0
PCBP1	0	1	0	0	0	0	0	0	0	0	0
PHF5A	0	1	0	0	0	0	0	0	0	0	0
PLK1	0	0	0	0	0	0	0	0	0	1	1
POLA1	0	0	0	0	0	0	0	1	1	0	0
POLD1	0	0	0	0	0	0	0	1	1	0	0
POLD3	0	0	0	0	0	0	0	1	1	0	0
POLR1B	0	0	0	0	0	0	0	1	0	0	0
POLR2A	1	0	0	0	0	0	0	1	0	0	0
POLR2B	0	0	0	0	0	0	0	1	0	0	0
POLR2C	0	0	0	0	0	0	0	1	0	0	0
POLR2E	1	0	0	0	0	0	0	1	0	0	0
POLR2H	1	0	0	0	0	0	0	1	0	0	0
POLR2L	0	0	0	0	0	0	0	1	0	0	0
POLR3A	0	0	0	0	0	0	0	1	0	0	0
POLR3D	0	0	0	0	0	0	0	1	0	0	0
PRIM2	0	0	0	0	0	0	0	1	1	0	0
PRPF4	0	1	0	0	0	0	0	0	0	0	0
PRPF6	0	1	0	0	0	0	0	0	0	0	0

NAME
CCNB1
CCNB2
DDX23
EFTUD2
EHMT2
EIF4A3
EP300
EXOSC4
EXOSC5
EXOSC6
EXOSC7
EXOSC9
HDAC2
HNRNPA1
HNRNPA3
HNRNPC
HNRNPK
HNRNPM
HNRNPU
HSPA9
HSPD1
LIG1
LSM2
LSM3
LSM4
LSM6
MAGOH
MCM2
MCM3
MCM4
MCM6
MCM7
NCBP1
NHP2L1
ORC2
ORC3
PAPOLA
PARN
PCBP1
PHF5A
PLK1
POLA1
POLD1
POLD3
POLR1B
POLR2A
POLR2B
POLR2C
POLR2E
POLR2H
POLR2L
POLR3A
POLR3D
PRIM2
PRPF4
PRPF6

Table S6 The differential proteins contribute to the enrichment of the significant pathways for the public dataset

Core enrichment proteins in up-regulated pathways of hESCs/HFs (hESCs vs HFs)

NAME	REACTOME_MITOCHONDRIAL_PROTEIN_IMPORT	REACTOME_DEADENYLATION_DEPENDENT_MRNA_DECAY	REACTOME_FACTORS_INVOLVED_IN_MEGAKARYOCYTE_DEVELOPMENT_AND_PLATELET_PRODUCTION	REACTOME_DNA_REPAIR	REACTOME_NUCLEOTIDE_EXCISION_REPAIR	REACTOME_MEIOSIS	REACTOME_MITOTIC_PROMETAPHASE	REACTOME_CHROMOSOME_MAINTENANCE	REACTOME_TELOMERE_MAINTENANCE	REACTOME_CELL_CYCLE	REACTOME_CELL_CYCLE_MITOTIC	REACTOME_DNA_REPLICATION	REACTOME_MITOTIC_M_M_G1_PHASES
PRPF8	0	0	0	0	0	0	0	0	0	0	0	0	0
RAD21	0	0	0	0	0	1	1	1	0	1	1	1	1
RBM8A	0	0	0	0	0	0	0	0	0	0	0	0	0
RBMX	0	0	0	0	0	0	0	0	0	0	0	0	0
RFC2	0	0	0	1	1	0	0	1	1	1	1	1	0
RFC4	0	0	0	1	1	0	0	1	1	1	1	1	0
RPA1	0	0	0	1	1	1	0	1	1	1	1	1	1
RPA2	0	0	0	1	1	1	0	1	1	1	1	1	1
RPA3	0	0	0	1	1	1	0	1	1	1	1	1	1
SF3A1	0	0	0	0	0	0	0	0	0	0	0	0	0
SF3A2	0	0	0	0	0	0	0	0	0	0	0	0	0
SF3A3	0	0	0	0	0	0	0	0	0	0	0	0	0
SF3B1	0	0	0	0	0	0	0	0	0	0	0	0	0
SF3B14	0	0	0	0	0	0	0	0	0	0	0	0	0
SF3B2	0	0	0	0	0	0	0	0	0	0	0	0	0
SF3B3	0	0	0	0	0	0	0	0	0	0	0	0	0
SF3B5	0	0	0	0	0	0	0	0	0	0	0	0	0
SMC1A	0	0	0	0	0	1	1	1	0	1	1	1	1
SMC3	0	0	0	0	0	1	1	1	0	1	1	1	1
SNRNP200	0	0	0	0	0	0	0	0	0	0	0	0	0
SNRNP40	0	0	0	0	0	0	0	0	0	0	0	0	0
SNRNP70	0	0	0	0	0	0	0	0	0	0	0	0	0
SNRPA	0	0	0	0	0	0	0	0	0	0	0	0	0
SNRPA1	0	0	0	0	0	0	0	0	0	0	0	0	0
SNRPB	0	0	0	0	0	0	0	0	0	0	0	0	0
SNRPB2	0	0	0	0	0	0	0	0	0	0	0	0	0
SNRPD1	0	0	0	0	0	0	0	0	0	0	0	0	0
SNRPD2	0	0	0	0	0	0	0	0	0	0	0	0	0
SNRPD3	0	0	0	0	0	0	0	0	0	0	0	0	0
SNRPE	0	0	0	0	0	0	0	0	0	0	0	0	0
SNRPF	0	0	0	0	0	0	0	0	0	0	0	0	0
SRSF1	0	0	0	0	0	0	0	0	0	0	0	0	0
SRSF3	0	0	0	0	0	0	0	0	0	0	0	0	0
SRSF4	0	0	0	0	0	0	0	0	0	0	0	0	0
SRSF9	0	0	0	0	0	0	0	0	0	0	0	0	0
STAG2	0	0	0	0	0	1	1	1	0	1	1	1	1
U2AF1	0	0	0	0	0	0	0	0	0	0	0	0	0
U2AF2	0	0	0	0	0	0	0	0	0	0	0	0	0
XAB2	0	0	0	1	1	0	0	0	0	0	0	0	0
AAAS	0	0	0	0	0	0	0	0	0	0	0	0	0
ACO2	1	0	0	0	0	0	0	0	0	0	0	0	0
AKAP1	0	0	1	0	0	0	0	0	0	0	0	0	0
APEX1	0	0	0	1	0	0	0	0	0	0	0	0	0
ATP5A1	1	0	0	0	0	0	0	0	0	0	0	0	0
ATP5B	1	0	0	0	0	0	0	0	0	0	0	0	0
BANF1	0	0	0	0	0	0	0	0	0	0	0	0	0
BLM	0	0	0	0	0	1	0	0	0	0	0	0	0
C2orf29	0	1	0	0	0	0	0	0	0	0	0	0	0
CBX3	0	0	0	0	0	0	0	0	0	0	0	0	0
CBX5	0	0	1	0	0	0	0	0	0	0	0	0	0
CCAR1	0	0	0	0	0	0	0	0	0	0	0	0	0
CDCA8	0	0	0	0	0	0	1	0	0	1	0	0	0
CKS1B	0	0	0	0	0	0	0	0	0	1	1	0	0
CLNS1A	0	0	0	0	0	0	0	0	0	0	0	0	0
CPSF1	0	0	0	0	0	0	0	0	0	0	0	0	0
CPSF2	0	0	0	0	0	0	0	0	0	0	0	0	0

NAME	REACTOME_CELL_CYCLE_CHECKPOINTS	REACTOME_S_PHASE	REACTOME_SYNTHESIS_OF_DNA	REACTOME_G1_S_TRANSITION	REACTOME_MITOTIC_G1_G1_S_PHASES	REACTOME_RNA_POL_I_RNA_POL_III_AND_MITOCHONDRIAL_TRANSCRIPTION	REACTOME_MRNA_PROCESSING	REACTOME_PROCESSING_OF_CAPPED_INTRON_CONTAINING_PRE_MRNA	REACTOME_MRNA_SPLICING	REACTOME_MRNA_SPLICING_MINOR_PATHWAY	REACTOME_TRANSCRIPTION	REACTOME_RNA_POL_II_TRANSCRIPTION	REACTOME_CLEAVAGE_OF_GROWING_TRANSCRIPT_IN_THE_TERMINATION_REGION_
PRPF8	0	0	0	0	0	0	1	1	1	1	0	0	0
RAD21	0	0	0	0	0	0	0	0	0	0	0	0	0
RBM8A	0	0	0	0	0	0	1	1	1	0	1	1	1
RBMX	0	0	0	0	0	0	1	1	1	0	0	0	0
RFC2	1	1	1	0	0	0	0	0	0	0	0	0	0
RFC4	1	1	1	0	0	0	0	0	0	0	0	0	0
RPA1	1	1	1	1	1	0	0	0	0	0	0	0	0
RPA2	1	1	1	1	1	0	0	0	0	0	0	0	0
RPA3	1	1	1	1	1	0	0	0	0	0	0	0	0
SF3A1	0	0	0	0	0	0	1	1	1	0	0	0	0
SF3A2	0	0	0	0	0	0	1	1	1	0	0	0	0
SF3A3	0	0	0	0	0	0	1	1	1	0	0	0	0
SF3B1	0	0	0	0	0	0	1	1	1	1	0	0	0
SF3B14	0	0	0	0	0	0	1	1	1	1	0	0	0
SF3B2	0	0	0	0	0	0	1	1	1	1	0	0	0
SF3B3	0	0	0	0	0	0	1	1	1	1	0	0	0
SF3B5	0	0	0	0	0	0	1	1	1	1	0	0	0
SMC1A	0	0	0	0	0	0	1	1	1	0	0	0	0
SMC3	0	0	0	0	0	0	0	0	0	0	0	0	0
SNRNP200	0	0	0	0	0	0	1	1	1	1	0	0	0
SNRNP40	0	0	0	0	0	0	1	1	1	1	0	0	0
SNRNP70	0	0	0	0	0	0	1	1	1	0	0	0	0
SNRPA	0	0	0	0	0	0	1	1	1	0	0	0	0
SNRPA1	0	0	0	0	0	0	1	1	1	0	0	0	0
SNRPB	0	0	0	0	0	0	1	1	1	1	1	1	1
SNRPB2	0	0	0	0	0	0	1	1	1	0	0	0	0
SNRPD1	0	0	0	0	0	0	1	1	1	1	0	0	0
SNRPD2	0	0	0	0	0	0	1	1	1	1	0	0	0
SNRPD3	0	0	0	0	0	0	1	1	1	1	1	1	1
SNRPE	0	0	0	0	0	0	1	1	1	1	1	1	1
SNRPF	0	0	0	0	0	0	1	1	1	1	1	1	1
SRSF1	0	0	0	0	0	0	1	1	1	1	1	1	1
SRSF3	0	0	0	0	0	0	1	1	1	0	1	1	1
SRSF4	0	0	0	0	0	0	1	1	1	0	1	1	1
SRSF9	0	0	0	0	0	0	1	1	1	0	1	1	1
STAG2	0	0	0	0	0	0	0	0	0	0	0	0	0
U2AF1	0	0	0	0	0	0	1	1	1	0	1	1	1
U2AF2	0	0	0	0	0	0	1	1	1	0	1	1	1
XAB2	0	0	0	0	0	0	0	0	0	0	0	0	0
AAAS	0	0	0	0	0	0	1	1	0	0	0	0	0
ACO2	0	0	0	0	0	0	0	0	0	0	0	0	0
AKAP1	0	0	0	0	0	0	0	0	0	0	0	0	0
APEX1	0	0	0	0	0	0	0	0	0	0	0	0	0
ATP5A1	0	0	0	0	0	0	0	0	0	0	0	0	0
ATP5B	0	0	0	0	0	0	0	0	0	0	0	0	0
BANF1	0	0	0	0	0	0	0	0	0	0	0	0	0
BLM	0	0	0	0	0	0	0	0	0	0	0	0	0
C2orf29	0	0	0	0	0	0	0	0	0	0	0	0	0
CBX3	0	0	0	0	0	1	0	0	0	0	1	0	0
CBX5	0	0	0	0	0	0	0	0	0	0	0	0	0
CCAR1	0	0	0	0	0	0	1	1	1	0	0	0	0
CDCA8	0	0	0	0	0	0	0	0	0	0	0	0	0
CKS1B	0	1	0	1	1	0	0	0	0	0	0	0	0
CLNS1A	0	0	0	0	0	0	0	0	0	0	0	0	0
CPSF1	0	0	0	0	0	0	1	1	1	0	1	1	1
CPSF2	0	0	0	0	0	0	1	1	1	0	1	1	1

NAME	REACTOME_MRNA_3_END_PROCESSING	REACTOME_RNA_POL_II_PRE_TRANSCRIPTION_EVENTS	REACTOME_METABOLISM_OF_NON_CODING_RNA	REACTOME_TRANSPORT_OF_MATURE_TRANSCRIPT_TO_CYTOPLASM	REACTOME_TRANSPORT_OF_MATURE_MRNA_DERIVED_FROM_AN_INTRONLESS_TRANSCRIPT	REACTOME_INTERACTIONS_OF_VPR_WITH_HOST_CELLULAR_PROTEINS	REACTOME_GLUCOSE_TRANSPORT	REACTOME_REGULATION_OF_GLUCOKINASE_BY_GLUCOKINASE_REGULATORY_PROTEIN	REACTOME_TRANSPORT_OF_RIBONUCLEOPROTEINS_INTO_THE_HOST_NUCLEUS	REACTOME_NEP_NS2_INTERACTS_WITH_THE_CELLULAR_EXPORT_MACHINERY	REACTOME_SLC_MEDIATED_TRANSMEMBRANE_TRANSPORT	REACTOME_ANTIVIRAL_MECHANISM_BY_IFN_STIMULATED_GENES	REACTOME_HIV_LIFE_CYCLE
PRPF8	0	0	0	0	0	0	0	0	0	0	0	0	0
RAD21	0	0	0	0	0	0	0	0	0	0	0	0	0
RBM8A	1	0	0	1	0	0	0	0	0	0	0	0	0
RBMX	0	0	0	0	0	0	0	0	0	0	0	0	0
RFC2	0	0	0	0	0	0	0	0	0	0	0	0	0
RFC4	0	0	0	0	0	0	0	0	0	0	0	0	0
RPA1	0	0	0	0	0	0	0	0	0	0	0	0	0
RPA2	0	0	0	0	0	0	0	0	0	0	0	0	0
RPA3	0	0	0	0	0	0	0	0	0	0	0	0	0
SF3A1	0	0	0	0	0	0	0	0	0	0	0	0	0
SF3A2	0	0	0	0	0	0	0	0	0	0	0	0	0
SF3A3	0	0	0	0	0	0	0	0	0	0	0	0	0
SF3B1	0	0	0	0	0	0	0	0	0	0	0	0	0
SF3B14	0	0	0	0	0	0	0	0	0	0	0	0	0
SF3B2	0	0	0	0	0	0	0	0	0	0	0	0	0
SF3B3	0	0	0	0	0	0	0	0	0	0	0	0	0
SF3B5	0	0	0	0	0	0	0	0	0	0	0	0	0
SMC1A	0	0	0	0	0	0	0	0	0	0	0	0	0
SMC3	0	0	0	0	0	0	0	0	0	0	0	0	0
SNRNP200	0	0	0	0	0	0	0	0	0	0	0	0	0
SNRNP40	0	0	0	0	0	0	0	0	0	0	0	0	0
SNRNP70	0	0	0	0	0	0	0	0	0	0	0	0	0
SNRPA	0	0	0	0	0	0	0	0	0	0	0	0	0
SNRPA1	0	0	0	0	0	0	0	0	0	0	0	0	0
SNRPB	0	0	1	0	0	0	0	0	0	0	0	0	0
SNRPB2	0	0	0	0	0	0	0	0	0	0	0	0	0
SNRPD1	0	0	1	0	0	0	0	0	0	0	0	0	0
SNRPD2	0	0	1	0	0	0	0	0	0	0	0	0	0
SNRPD3	0	0	1	0	0	0	0	0	0	0	0	0	0
SNRPE	0	0	1	0	0	0	0	0	0	0	0	0	0
SNRPF	0	0	1	0	0	0	0	0	0	0	0	0	0
SRSF1	1	0	0	1	0	0	0	0	0	0	0	0	0
SRSF3	1	0	0	1	0	0	0	0	0	0	0	0	0
SRSF4	1	0	0	1	0	0	0	0	0	0	0	0	0
SRSF9	1	0	0	1	0	0	0	0	0	0	0	0	0
STAG2	0	0	0	0	0	0	0	0	0	0	0	0	0
U2AF1	1	0	0	1	0	0	0	0	0	0	0	0	0
U2AF2	1	0	0	1	0	0	0	0	0	0	0	0	0
XAB2	0	0	0	0	0	0	0	0	0	0	0	0	0
AAAS	0	0	1	1	1	1	1	1	1	1	1	1	1
ACO2	0	0	0	0	0	0	0	0	0	0	0	0	0
AKAP1	0	0	0	0	0	0	0	0	0	0	0	0	0
APEX1	0	0	0	0	0	0	0	0	0	0	0	0	0
ATP5A1	0	0	0	0	0	0	0	0	0	0	0	0	0
ATP5B	0	0	0	0	0	0	0	0	0	0	0	0	0
BANF1	0	0	0	0	0	1	0	0	0	0	0	0	1
BLM	0	0	0	0	0	0	0	0	0	0	0	0	0
C2orf29	0	0	0	0	0	0	0	0	0	0	0	0	0
CBX3	0	0	0	0	0	0	0	0	0	0	0	0	0
CBX5	0	0	0	0	0	0	0	0	0	0	0	0	0
CCAR1	0	0	0	0	0	0	0	0	0	0	0	0	0
CDCA8	0	0	0	0	0	0	0	0	0	0	0	0	0
CKS1B	0	0	0	0	0	0	0	0	0	0	0	0	0
CLNS1A	0	0	1	0	0	0	0	0	0	0	0	0	0
CPSF1	1	0	0	1	1	0	0	0	0	0	0	0	0
CPSF2	1	0	0	1	1	0	0	0	0	0	0	0	0

NAME	REACTOME_HIV_INFECTION	KEGG_SPLICEOSOME_	KEGG_RNA_DEGRADATION_	KEGG_ARGININE_AND_PROLINE_METABOLISM_	KEGG_FATTY_ACID_METABOLISM_	KEGG_VALINE_LEUCINE_AND_ISOLEUCINE_DEGRADATION_	KEGG_LYSINE_DEGRADATION_	KEGG_PYRIMIDINE_METABOLISM_	KEGG_DNA_REPLICATION_	KEGG_CELL_CYCLE_	KEGG_OOCYTE_MEIOSIS_
PRPF8	0	1	0	0	0	0	0	0	0	0	0
RAD21	0	0	0	0	0	0	0	0	0	1	0
RBM8A	0	1	0	0	0	0	0	0	0	0	0
RBMX	0	1	0	0	0	0	0	0	0	0	0
RFC2	0	0	0	0	0	0	0	0	1	0	0
RFC4	0	0	0	0	0	0	0	0	1	0	0
RPA1	0	0	0	0	0	0	0	0	1	0	0
RPA2	0	0	0	0	0	0	0	0	1	0	0
RPA3	0	0	0	0	0	0	0	0	1	0	0
SF3A1	0	1	0	0	0	0	0	0	0	0	0
SF3A2	0	1	0	0	0	0	0	0	0	0	0
SF3A3	0	1	0	0	0	0	0	0	0	0	0
SF3B1	0	1	0	0	0	0	0	0	0	0	0
SF3B14	0	1	0	0	0	0	0	0	0	0	0
SF3B2	0	1	0	0	0	0	0	0	0	0	0
SF3B3	0	1	0	0	0	0	0	0	0	0	0
SF3B5	0	1	0	0	0	0	0	0	0	0	0
SMC1A	0	0	0	0	0	0	0	0	0	1	1
SMC3	0	0	0	0	0	0	0	0	0	1	1
SNRNP200	0	1	0	0	0	0	0	0	0	0	0
SNRNP40	0	1	0	0	0	0	0	0	0	0	0
SNRNP70	0	1	0	0	0	0	0	0	0	0	0
SNRPA	0	1	0	0	0	0	0	0	0	0	0
SNRPA1	0	1	0	0	0	0	0	0	0	0	0
SNRPB	0	1	0	0	0	0	0	0	0	0	0
SNRPB2	0	1	0	0	0	0	0	0	0	0	0
SNRPD1	0	1	0	0	0	0	0	0	0	0	0
SNRPD2	0	1	0	0	0	0	0	0	0	0	0
SNRPD3	0	1	0	0	0	0	0	0	0	0	0
SNRPE	0	1	0	0	0	0	0	0	0	0	0
SNRPF	0	1	0	0	0	0	0	0	0	0	0
SRSF1	0	1	0	0	0	0	0	0	0	0	0
SRSF3	0	1	0	0	0	0	0	0	0	0	0
SRSF4	0	1	0	0	0	0	0	0	0	0	0
SRSF9	0	1	0	0	0	0	0	0	0	0	0
STAG2	0	0	0	0	0	0	0	0	0	1	0
U2AF1	0	1	0	0	0	0	0	0	0	0	0
U2AF2	0	1	0	0	0	0	0	0	0	0	0
XAB2	0	1	0	0	0	0	0	0	0	0	0
AAAS	1	0	0	0	0	0	1	0	0	0	0
ACO2	0	0	0	0	1	1	0	0	0	0	0
AKAP1	0	1	0	0	0	0	0	0	0	0	0
APEX1	0	0	0	1	1	1	1	0	0	0	0
ATP5A1	0	0	0	1	1	1	1	0	0	0	0
ATP5B	0	0	0	0	0	1	0	0	0	0	0
BANF1	1	0	0	0	1	1	0	0	0	0	0
BLM	0	1	0	0	0	0	0	0	0	0	0
C2orf29	0	0	0	1	0	0	0	0	0	0	0
CBX3	0	0	0	0	0	1	0	0	0	0	0
CBX5	0	1	0	0	0	0	0	0	0	0	0
CCAR1	0	0	0	0	0	0	0	1	0	0	0
CDCA8	0	0	0	1	0	0	0	0	0	0	0
CKS1B	0	0	0	1	0	0	0	0	0	0	0
CLNS1A	0	0	0	0	1	0	0	0	0	0	0
CPSF1	0	1	0	0	0	0	0	0	0	0	0
CPSF2	0	0	0	0	0	0	0	1	0	0	0

NAME
PRPF8
RAD21
RBM8A
RBMX
RFC2
RFC4
RPA1
RPA2
RPA3
SF3A1
SF3A2
SF3A3
SF3B1
SF3B14
SF3B2
SF3B3
SF3B5
SMC1A
SMC3
SNRNP200
SNRNP40
SNRNP70
SNRPA
SNRPA1
SNRPB
SNRPB2
SNRPD1
SNRPD2
SNRPD3
SNRPE
SNRPF
SRSF1
SRSF3
SRSF4
SRSF9
STAG2
U2AF1
U2AF2
XAB2
AAAS
ACO2
AKAP1
APEX1
ATP5A1
ATP5B
BANF1
BLM
C2orf29
CBX3
CBX5
CCAR1
CDCA8
CKS1B
CLNS1A
CPSF1
CPSF2

Table S6 The differential proteins contribute to the enrichment of the significant pathways for the public dataset

Core enrichment proteins in up-regulated pathways of hESCs/HFs (hESCs vs HFs)

NAME	REACTOME_MITOCHONDRIAL_PROTEIN_IMPORT	REACTOME_DEADENYLATION_DEPENDENT_MRNA_DECAY	REACTOME_FACTORS_INVOLVED_IN_MEGAKARYOCYTE_DEVELOPMENT_AND_PLATELET_PRODUCTION	REACTOME_DNA_REPAIR	REACTOME_NUCLEOTIDE_EXCISION_REPAIR	REACTOME_MEIOSIS	REACTOME_MITOTIC_PROMETAPHASE	REACTOME_CHROMOSOME_MAINTENANCE	REACTOME_TELOMERE_MAINTENANCE	REACTOME_CELL_CYCLE	REACTOME_CELL_CYCLE_MITOTIC	REACTOME_DNA_REPLICATION	REACTOME_MITOTIC_M_M_G1_PHASES
CPSF3	0	0	0	0	0	0	0	0	0	0	0	0	0
CPSF7	0	0	0	0	0	0	0	0	0	0	0	0	0
CSTF1	0	0	0	0	0	0	0	0	0	0	0	0	0
CSTF2	0	0	0	0	0	0	0	0	0	0	0	0	0
CSTF3	0	0	0	0	0	0	0	0	0	0	0	0	0
CYC1	1	0	0	0	0	0	0	0	0	0	0	0	0
DDB1	0	0	0	1	0	0	0	0	0	0	0	0	0
DDX20	0	0	0	0	0	0	0	0	0	0	0	0	0
DHX9	0	0	0	0	0	0	0	0	0	0	0	0	0
DIDO1	0	0	0	0	0	0	0	1	0	1	0	0	0
FEN1	0	0	0	1	0	0	0	1	1	1	0	0	0
FLNB	0	0	0	0	0	0	0	0	0	0	0	0	0
FUS	0	0	0	0	0	0	0	0	0	0	0	0	0
FXC1	1	0	0	0	0	0	0	0	0	0	0	0	0
FXN	1	0	0	0	0	0	0	0	0	0	0	0	0
GTF2E2	0	0	0	0	0	0	0	0	0	0	0	0	0
GTF3C2	0	0	0	0	0	0	0	0	0	0	0	0	0
GTF3C3	0	0	0	0	0	0	0	0	0	0	0	0	0
GTF3C4	0	0	0	0	0	0	0	0	0	0	0	0	0
H2AFX	0	0	0	1	0	0	0	0	0	0	0	0	0
HIST2H4B	0	0	0	0	0	1	0	1	1	1	0	0	0
HMGA1	0	0	0	0	0	0	0	0	0	0	0	0	0
HNRNPA0	0	0	0	0	0	0	0	0	0	0	0	0	0
HNRNPA2B1	0	0	0	0	0	0	0	0	0	0	0	0	0
HNRNPD	0	0	0	0	0	0	0	0	0	0	0	0	0
HNRNPF	0	0	0	0	0	0	0	0	0	0	0	0	0
HNRNPH1	0	0	0	0	0	0	0	0	0	0	0	0	0
HNRNPL	0	0	0	0	0	0	0	0	0	0	0	0	0
HNRNPR	0	0	0	0	0	0	0	0	0	0	0	0	0
HNRNPUL1	0	0	0	0	0	0	0	0	0	0	0	0	0
INCENP	0	0	0	0	0	0	1	0	0	1	1	1	1
JMJD1C	0	0	1	0	0	0	0	0	0	0	0	0	0
KDM1A	0	0	1	0	0	0	0	0	0	0	0	0	0
KIF11	0	0	1	0	0	0	0	0	0	0	0	0	0
KIF20A	0	0	1	0	0	0	0	0	0	1	1	1	1
KIF22	0	0	1	0	0	0	0	0	0	0	0	0	0
KIF2C	0	0	1	0	0	0	1	0	0	1	1	1	1
KIFC1	0	0	1	0	0	0	0	0	0	0	0	0	0
KPNA2	0	0	0	0	0	0	0	0	0	0	0	0	0
LMNB1	0	0	0	0	0	1	0	1	0	1	0	0	0
METTL3	0	0	0	0	0	0	0	0	0	0	0	0	0
MLF1IP	0	0	0	0	0	0	1	1	0	1	1	1	1
MPG	0	0	0	1	0	0	0	0	0	0	0	0	0
MRE11A	0	0	0	1	0	0	0	0	0	0	0	0	0
NPM1	0	0	0	0	0	0	0	1	0	1	0	0	0
NUDT21	0	0	0	0	0	0	0	0	0	0	0	0	0
NUMA1	0	0	0	0	0	0	0	0	0	1	1	0	0
NUP107	0	0	0	0	0	0	1	0	0	1	1	1	1
NUP133	0	0	0	0	0	0	1	0	0	1	1	1	1
NUP153	0	0	0	0	0	0	0	0	0	0	0	0	0
NUP155	0	0	0	0	0	0	0	0	0	0	0	0	0
NUP205	0	0	0	0	0	0	0	0	0	0	0	0	0
NUP210	0	0	0	0	0	0	0	0	0	0	0	0	0
NUP214	0	0	0	0	0	0	0	0	0	0	0	0	0
NUP35	0	0	0	0	0	0	0	0	0	0	0	0	0
NUP37	0	0	0	0	0	0	1	0	0	1	1	1	1

NAME	REACTOME_CELL_CYCLE_CHECKPOINTS	REACTOME_S_PHASE	REACTOME_SYNTHESIS_OF_DNA	REACTOME_G1_S_TRANSITION	REACTOME_MITOTIC_G1_G1_S_PHASES	REACTOME_RNA_POL_I_RNA_POL_III_AND_MITOCHONDRIAL_TRANSCRIPTION	REACTOME_MRNA_PROCESSING	REACTOME_PROCESSING_OF_CAPPED_INTRON_CONTAINING_PRE_MRNA	REACTOME_MRNA_SPLICING	REACTOME_MRNA_SPLICING_MINOR_PATHWAY	REACTOME_TRANSCRIPTION	REACTOME_RNA_POL_II_TRANSCRIPTION	REACTOME_CLEAVAGE_OF_GROWING_TRANSCRIPT_IN_THE_TERMINATION_REGION_
CPSF3	0	0	0	0	0	0	1	1	1	0	1	1	1
CPSF7	0	0	0	0	0	0	1	1	1	0	1	1	1
CSTF1	0	0	0	0	0	0	1	1	1	0	1	1	1
CSTF2	0	0	0	0	0	0	1	1	1	0	1	1	1
CSTF3	0	0	0	0	0	0	1	1	1	0	1	1	1
CYC1	0	0	0	0	0	0	0	0	0	0	0	0	0
DDB1	0	0	0	0	0	0	0	0	0	0	0	0	0
DDX20	0	0	0	0	0	0	0	0	0	0	0	0	0
DHX9	0	0	0	0	0	0	1	1	1	0	0	0	0
DIDO1	0	0	0	0	0	0	0	0	0	0	0	0	0
FEN1	0	0	0	0	0	0	0	0	0	0	0	0	0
FLNB	0	0	0	0	0	0	0	0	0	0	0	0	0
FUS	0	0	0	0	0	0	1	1	1	0	0	0	0
FXC1	0	0	0	0	0	0	0	0	0	0	0	0	0
FXN	0	0	0	0	0	0	0	0	0	0	0	0	0
GTF2E2	0	0	0	0	0	0	0	0	0	0	1	1	0
GTF3C2	0	0	0	0	0	1	0	0	0	0	1	0	0
GTF3C3	0	0	0	0	0	1	0	0	0	0	1	0	0
GTF3C4	0	0	0	0	0	1	0	0	0	0	1	0	0
H2AFX	0	0	0	0	0	1	0	0	0	0	1	0	0
HIST2H4B	0	0	0	0	0	1	0	0	0	0	1	0	0
HMGA1	0	0	0	0	0	0	0	0	0	0	0	0	0
HNRNPA0	0	0	0	0	0	0	1	1	1	0	0	0	0
HNRNPA2B1	0	0	0	0	0	0	1	1	1	0	0	0	0
HNRNPD	0	0	0	0	0	0	1	1	1	0	0	0	0
HNRNPF	0	0	0	0	0	0	1	1	1	0	0	0	0
HNRNPH1	0	0	0	0	0	0	1	1	1	0	0	0	0
HNRNPL	0	0	0	0	0	0	1	1	1	0	0	0	0
HNRNPR	0	0	0	0	0	0	1	1	1	0	0	0	0
HNRNPUL1	0	0	0	0	0	0	1	1	1	0	0	0	0
INCENP	0	0	0	0	0	0	0	0	0	0	0	0	0
JMJD1C	0	0	0	0	0	0	0	0	0	0	0	0	0
KDM1A	0	0	0	0	0	0	0	0	0	0	0	0	0
KIF11	0	0	0	0	0	0	0	0	0	0	0	0	0
KIF20A	0	0	0	0	0	0	0	0	0	0	0	0	0
KIF22	0	0	0	0	0	0	0	0	0	0	0	0	0
KIF2C	0	0	0	0	0	0	0	0	0	0	0	0	0
KIFC1	0	0	0	0	0	0	0	0	0	0	0	0	0
KPNA2	0	0	0	0	0	0	0	0	0	0	0	0	0
LMNB1	0	0	0	0	0	0	0	0	0	0	0	0	0
METTL3	0	0	0	0	0	0	1	1	0	0	0	0	0
MLF1IP	0	0	0	0	0	0	0	0	0	0	0	0	0
MPG	0	0	0	0	0	0	0	0	0	0	0	0	0
MRE11A	0	0	0	0	0	0	0	0	0	0	0	0	0
NPM1	0	0	0	0	0	0	0	0	0	0	0	0	0
NUDT21	0	0	0	0	0	0	1	1	1	0	1	1	1
NUMA1	0	0	0	0	0	0	0	0	0	0	0	0	0
NUP107	0	0	0	0	0	0	1	1	0	0	0	0	0
NUP133	0	0	0	0	0	0	1	1	0	0	0	0	0
NUP153	0	0	0	0	0	0	1	1	0	0	0	0	0
NUP155	0	0	0	0	0	0	1	1	0	0	0	0	0
NUP205	0	0	0	0	0	0	1	1	0	0	0	0	0
NUP210	0	0	0	0	0	0	1	1	0	0	0	0	0
NUP214	0	0	0	0	0	0	1	1	0	0	0	0	0
NUP35	0	0	0	0	0	0	1	1	0	0	0	0	0
NUP37	0	0	0	0	0	0	1	1	0	0	0	0	0

NAME	REACTOME_MRNA_3_END_PROCESSING	REACTOME_RNA_POL_II_PRE_TRANSCRIPTION_EVENTS	REACTOME_METABOLISM_OF_NON_CODING_RNA	REACTOME_TRANSPORT_OF_MATURE_TRANSCRIPT_TO_CYTOPLASM	REACTOME_TRANSPORT_OF_MATURE_MRNA_DERIVED_FROM_AN_INTRONLESS_TRANSCRIPT	REACTOME_INTERACTIONS_OF_VPR_WITH_HOST_CELLULAR_PROTEINS	REACTOME_GLUCOSE_TRANSPORT	REACTOME_REGULATION_OF_GLUCOKINASE_BY_GLUCOKINASE_REGULATORY_PROTEIN	REACTOME_TRANSPORT_OF_RIBONUCLEOPROTEINS_INTO_THE_HOST_NUCLEUS	REACTOME_NEP_NS2_INTERACTS_WITH_THE_CELLULAR_EXPORT_MACHINERY	REACTOME_SLC_MEDIATED_TRANSMEMBRANE_TRANSPORT	REACTOME_ANTIVIRAL_MECHANISM_BY_IFN_STIMULATED_GENES	REACTOME_HIV_LIFE_CYCLE
CPSF3	1	0	0	1	1	0	0	0	0	0	0	0	0
CPSF7	1	0	0	0	0	0	0	0	0	0	0	0	0
CSTF1	1	0	0	0	0	0	0	0	0	0	0	0	0
CSTF2	1	0	0	0	0	0	0	0	0	0	0	0	0
CSTF3	1	0	0	0	0	0	0	0	0	0	0	0	0
CYC1	0	0	0	0	0	0	0	0	0	0	0	0	0
DDB1	0	0	0	0	0	0	0	0	0	0	0	0	0
DDX20	0	0	1	0	0	0	0	0	0	0	0	0	0
DHX9	0	0	0	0	0	0	0	0	0	0	0	0	0
DIDO1	0	0	0	0	0	0	0	0	0	0	0	0	0
FEN1	0	0	0	0	0	0	0	0	0	0	0	0	1
FLNB	0	0	0	0	0	0	0	0	0	0	0	1	0
FUS	0	0	0	0	0	0	0	0	0	0	0	0	0
FXC1	0	0	0	0	0	0	0	0	0	0	0	0	0
FXN	0	0	0	0	0	0	0	0	0	0	0	0	0
GTF2E2	0	1	0	0	0	0	0	0	0	0	0	0	1
GTF3C2	0	0	0	0	0	0	0	0	0	0	0	0	0
GTF3C3	0	0	0	0	0	0	0	0	0	0	0	0	0
GTF3C4	0	0	0	0	0	0	0	0	0	0	0	0	0
H2AFX	0	0	0	0	0	0	0	0	0	0	0	0	0
HIST2H4B	0	0	0	0	0	0	0	0	0	0	0	0	0
HMGA1	0	0	0	0	0	1	0	0	0	0	0	0	1
HNRNPA0	0	0	0	0	0	0	0	0	0	0	0	0	0
HNRNPA2B1	0	0	0	0	0	0	0	0	0	0	0	0	0
HNRNPD	0	0	0	0	0	0	0	0	0	0	0	0	0
HNRNPF	0	0	0	0	0	0	0	0	0	0	0	0	0
HNRNPH1	0	0	0	0	0	0	0	0	0	0	0	0	0
HNRNPL	0	0	0	0	0	0	0	0	0	0	0	0	0
HNRNPR	0	0	0	0	0	0	0	0	0	0	0	0	0
HNRNPUL1	0	0	0	0	0	0	0	0	0	0	0	0	0
INCENP	0	0	0	0	0	0	0	0	0	0	0	0	0
JMJD1C	0	0	0	0	0	0	0	0	0	0	0	0	0
KDM1A	0	0	0	0	0	0	0	0	0	0	0	0	0
KIF11	0	0	0	0	0	0	0	0	0	0	0	0	0
KIF20A	0	0	0	0	0	0	0	0	0	0	0	0	0
KIF22	0	0	0	0	0	0	0	0	0	0	0	0	0
KIF2C	0	0	0	0	0	0	0	0	0	0	0	0	0
KIFC1	0	0	0	0	0	0	0	0	0	0	0	0	0
KPNA2	0	0	0	0	0	0	0	0	0	0	0	1	0
LMNB1	0	0	0	0	0	0	0	0	0	0	0	0	0
METTL3	0	0	0	0	0	0	0	0	0	0	0	0	0
MLF1IP	0	0	0	0	0	0	0	0	0	0	0	0	0
MPG	0	0	0	0	0	0	0	0	0	0	0	0	0
MRE11A	0	0	0	0	0	0	0	0	0	0	0	0	0
NPM1	0	0	0	0	0	0	0	0	0	0	0	0	0
NUDT21	1	0	0	0	0	0	0	0	0	0	0	0	0
NUMA1	0	0	0	0	0	0	0	0	0	0	0	0	0
NUP107	0	0	1	1	1	1	1	1	1	1	1	1	1
NUP133	0	0	1	1	1	1	1	1	1	1	1	1	1
NUP153	0	0	1	1	1	1	1	1	1	1	1	1	1
NUP155	0	0	1	1	1	1	1	1	1	1	1	1	1
NUP205	0	0	1	1	1	1	1	1	1	1	1	1	1
NUP210	0	0	1	1	1	1	1	1	1	1	1	1	1
NUP214	0	0	1	1	1	1	1	1	1	1	1	1	1
NUP35	0	0	1	1	1	1	1	1	1	1	1	1	1
NUP37	0	0	1	1	1	1	1	1	1	1	1	1	1

NAME	REACTOME_HIV_INFECTION	KEGG_SPLICEOSOME_	KEGG_RNA_DEGRADATION_	KEGG_ARGININE_AND_PROLINE_METABOLISM_	KEGG_FATTY_ACID_METABOLISM_	KEGG_VALINE_LEUCINE_AND_ISOLEUCINE_DEGRADATION_	KEGG_LYSINE_DEGRADATION_	KEGG_PYRIMIDINE_METABOLISM_	KEGG_DNA_REPLICATION_	KEGG_CELL_CYCLE_	KEGG_OOCYTE_MEIOSIS_
CPSF3	0	1	0	0	0	0	0	0	0	0	0
CPSF7	0	0	0	0	0	1	0	0	0	0	0
CSTF1	0	1	0	0	0	0	0	0	0	0	0
CSTF2	0	1	0	0	0	0	0	0	0	0	0
CSTF3	0	1	0	0	0	0	0	0	0	0	0
CYC1	0	1	0	0	0	0	0	0	0	0	0
DDB1	0	0	0	0	0	0	0	1	0	0	0
DDX20	0	0	1	0	0	0	0	0	0	0	0
DHX9	0	0	0	0	1	0	1	0	0	0	0
DIDO1	0	0	0	0	1	1	0	0	0	0	0
FEN1	1	0	0	0	0	1	0	0	0	0	0
FLNB	0	1	0	0	0	0	0	0	0	0	0
FUS	0	1	1	0	0	0	0	0	0	0	0
FXC1	0	0	0	0	0	1	0	0	0	0	0
FXN	0	0	0	0	0	0	0	1	0	0	0
GTF2E2	1	0	0	0	0	0	1	0	0	0	0
GTF3C2	0	0	0	0	0	0	0	1	0	0	0
GTF3C3	0	0	1	0	0	0	0	1	0	0	0
GTF3C4	0	0	0	0	0	0	0	1	0	0	0
H2AFX	0	0	0	0	0	0	0	1	0	0	0
HIST2H4B	0	0	0	0	0	0	0	1	0	0	0
HMGA1	1	1	0	0	0	0	0	0	0	0	0
HNRNPA0	0	1	0	0	0	0	0	0	0	0	0
HNRNPA2B1	0	1	0	0	0	0	0	0	0	0	0
HNRNPD	0	0	0	1	0	0	0	0	0	0	0
HNRNPF	0	1	0	0	0	0	0	0	0	0	0
HNRNPH1	0	1	0	0	0	0	0	0	0	0	0
HNRNPL	0	1	0	0	0	0	0	0	0	0	0
HNRNPR	0	1	0	0	0	0	0	0	0	0	0
HNRNPUL1	0	1	0	0	0	0	0	0	0	0	0
INCENP	0	0	0	0	0	0	0	0	1	0	0
JMJD1C	0	0	0	0	0	0	0	0	1	0	0
KDM1A	0	1	0	0	0	0	0	0	0	0	0
KIF11	0	0	1	0	0	0	0	0	0	0	0
KIF20A	0	1	0	0	0	0	0	0	0	0	0
KIF22	0	1	0	0	0	0	0	0	0	0	0
KIF2C	0	1	0	0	0	0	0	0	0	0	0
KIFC1	0	1	0	0	0	0	0	0	0	0	0
KPNA2	0	1	0	0	0	0	0	0	0	0	0
LMNB1	0	1	0	0	0	0	0	0	0	0	0
METTL3	0	1	0	0	0	0	0	0	0	0	0
MLF1IP	0	1	0	0	0	0	0	0	0	0	0
MPG	0	1	0	0	0	0	0	0	0	0	0
MRE11A	0	1	0	0	0	0	0	0	0	0	0
NPM1	1	1	0	0	0	0	0	0	0	0	0
NUDT21	0	1	0	0	0	0	0	0	0	0	0
NUMA1	0	0	0	0	0	0	0	0	0	0	0
NUP107	1	0	0	0	0	0	0	0	0	0	0
NUP133	1	0	0	0	0	0	0	0	0	0	0
NUP153	1	0	0	0	0	0	0	0	0	0	0
NUP155	1	0	0	0	0	0	0	0	0	0	0
NUP205	1	0	0	0	0	0	0	0	0	0	0
NUP210	1	0	0	0	0	0	0	0	0	0	0
NUP214	1	0	0	0	0	0	0	0	0	0	0
NUP35	1	0	0	0	0	0	0	0	0	0	0
NUP37	1	0	0	0	0	0	0	0	0	0	0

NAME
CPSF3
CPSF7
CSTF1
CSTF2
CSTF3
CYC1
DDB1
DDX20
DHX9
DIDO1
FEN1
FLNB
FUS
FXC1
FXN
GTF2E2
GTF3C2
GTF3C3
GTF3C4
H2AFX
HIST2H4B
HMGA1
HNRNPA0
HNRNPA2B1
HNRNPD
HNRNPF
HNRNPH1
HNRNPL
HNRNPR
HNRNPUL1
INCENP
JMJD1C
KDM1A
KIF11
KIF20A
KIF22
KIF2C
KIFC1
KPNA2
LMNB1
METTL3
MLF1IP
MPG
MRE11A
NPM1
NUDT21
NUMA1
NUP107
NUP133
NUP153
NUP155
NUP205
NUP210
NUP214
NUP35
NUP37

Table S6 The differential proteins contribute to the enrichment of the significant pathways for the public dataset

Core enrichment proteins in up-regulated pathways of hESCs/HFs (hESCs vs HFs)

NAME	REACTOME_MITOCHONDRIAL_PROTEIN_IMPORT	REACTOME_DEADENYLATION_DEPENDENT_MRNA_DECAY	REACTOME_FACTORS_INVOLVED_IN_MEGAKARYOCYTE_DEVELOPMENT_AND_PLATELET_PRODUCTION	REACTOME_DNA_REPAIR	REACTOME_NUCLEOTIDE_EXCISION_REPAIR	REACTOME_MEIOSIS	REACTOME_MITOTIC_PROMETAPHASE	REACTOME_CHROMOSOME_MAINTENANCE	REACTOME_TELOMERE_MAINTENANCE	REACTOME_CELL_CYCLE	REACTOME_CELL_CYCLE_MITOTIC	REACTOME_DNA_REPLICATION	REACTOME_MITOTIC_M_M_G1_PHASES
NUP43	0	0	0	0	0	0	1	0	0	1	1	0	0
NUP50	0	0	0	0	0	0	0	0	0	0	0	0	0
NUP54	0	0	0	0	0	0	0	0	0	0	0	0	0
NUP62	0	0	0	0	0	0	0	0	0	0	0	0	0
NUP88	0	0	0	0	0	0	0	0	0	0	0	0	0
NUP93	0	0	0	0	0	0	0	0	0	0	0	0	0
NUPL1	0	0	0	0	0	0	0	0	0	0	0	0	0
PABPN1	0	0	0	0	0	0	0	0	0	0	0	0	0
PCM1	0	0	0	0	0	0	0	0	0	1	1	0	0
POLB	0	0	0	1	0	0	0	0	0	0	0	0	0
POLRMT	0	0	0	0	0	0	0	0	0	0	0	0	0
PRKAR2B	0	0	0	0	0	0	0	0	0	1	1	0	0
PRKDC	0	0	0	1	0	0	0	0	0	0	0	0	0
PSIP1	0	0	0	0	0	0	0	0	0	0	0	0	0
PTBP1	0	0	0	0	0	0	0	0	0	0	0	0	0
RAD50	0	0	0	1	0	0	0	0	0	0	0	0	0
RAE1	0	0	0	0	0	0	0	0	0	0	0	0	0
RANBP2	0	0	0	0	0	0	1	0	0	1	1	1	1
RBBP4	0	0	0	0	0	0	0	1	0	1	1	0	0
RBBP7	0	0	0	0	0	0	0	1	0	1	0	0	0
RBM5	0	0	0	0	0	0	0	0	0	0	0	0	0
RCC1	0	0	0	0	0	0	0	0	0	0	0	0	0
RCC2	0	0	0	0	0	0	1	0	0	1	1	1	1
RNMT	0	0	0	0	0	0	0	0	0	0	0	0	0
RNPS1	0	0	0	0	0	0	0	0	0	0	0	0	0
RSF1	0	0	0	0	0	0	0	1	0	1	0	0	0
SAMM50	1	0	0	0	0	0	0	0	0	0	0	0	0
SEH1L	0	0	0	0	0	0	1	0	0	1	1	1	1
SIN3A	0	0	1	0	0	0	0	0	0	0	0	0	0
SLC12A7	0	0	0	0	0	0	0	0	0	0	0	0	0
SLC25A13	1	0	0	0	0	0	0	0	0	0	0	0	0
SLC25A4	1	0	0	0	0	0	0	0	0	0	0	0	0
SLC2A1	0	0	0	0	0	0	0	0	0	0	0	0	0
SLC2A3	0	0	0	0	0	0	0	0	0	0	0	0	0
SLC4A7	0	0	0	0	0	0	0	0	0	0	0	0	0
SLC7A3	0	0	0	0	0	0	0	0	0	0	0	0	0
SMARCA5	0	0	0	0	0	0	0	1	0	1	0	0	0
SMN2	0	0	0	0	0	0	0	0	0	0	0	0	0
SRRM1	0	0	0	0	0	0	0	0	0	0	0	0	0
SRSF11	0	0	0	0	0	0	0	0	0	0	0	0	0
SSRP1	0	0	0	0	0	0	0	0	0	0	0	0	0
SUGP1	0	0	0	0	0	0	0	0	0	0	0	0	0
SUPT16H	0	0	0	0	0	0	0	0	0	0	0	0	0
SUPT5H	0	0	0	0	0	0	0	0	0	0	0	0	0
SYNE2	0	0	0	0	0	1	0	1	0	1	0	0	0
TAF10	0	0	0	0	0	0	0	0	0	0	0	0	0
TAF4	0	0	0	0	0	0	0	0	0	0	0	0	0
TAF5	0	0	0	0	0	0	0	0	0	0	0	0	0
TBP	0	0	0	0	0	0	0	0	0	0	0	0	0
TCEA1	0	0	0	1	1	0	0	0	0	0	0	0	0
TERF2	0	0	0	0	0	1	0	1	1	1	0	0	0
TFAM	0	0	0	0	0	0	0	0	0	0	0	0	0
TIMM10	1	0	0	0	0	0	0	0	0	0	0	0	0
TIMM13	1	0	0	0	0	0	0	0	0	0	0	0	0
TIMM44	1	0	0	0	0	0	0	0	0	0	0	0	0
TIMM50	1	0	0	0	0	0	0	0	0	0	0	0	0

NAME	REACTOME_CELL_CYCLE_CHECKPOINTS	REACTOME_S_PHASE	REACTOME_SYNTHESIS_OF_DNA	REACTOME_G1_S_TRANSITION	REACTOME_MITOTIC_G1_G1_S_PHASES	REACTOME_RNA_POL_I_RNA_POL_III_AND_MITOCHONDRIAL_TRANSCRIPTION	REACTOME_MRNA_PROCESSING	REACTOME_PROCESSING_OF_CAPPED_INTRON_CONTAINING_PRE_MRNA	REACTOME_MRNA_SPLICING	REACTOME_MRNA_SPLICING_MINOR_PATHWAY	REACTOME_TRANSCRIPTION	REACTOME_RNA_POL_II_TRANSCRIPTION	REACTOME_CLEAVAGE_OF_GROWING_TRANSCRIPT_IN_THE_TERMINATION_REGION_
NUP43	0	0	0	0	0	0	1	1	0	0	0	0	0
NUP50	0	0	0	0	0	0	1	1	0	0	0	0	0
NUP54	0	0	0	0	0	0	1	1	0	0	0	0	0
NUP62	0	0	0	0	0	0	1	1	0	0	0	0	0
NUP88	0	0	0	0	0	0	1	1	0	0	0	0	0
NUP93	0	0	0	0	0	0	1	1	0	0	0	0	0
NUPL1	0	0	0	0	0	0	1	1	0	0	0	0	0
PABPN1	0	0	0	0	0	0	1	1	1	0	1	1	1
PCM1	0	0	0	0	0	0	0	0	0	0	0	0	0
POLB	0	0	0	0	0	0	0	0	0	0	0	0	0
POLRMT	0	0	0	0	0	1	0	0	0	0	1	0	0
PRKAR2B	0	0	0	0	0	0	0	0	0	0	0	0	0
PRKDC	0	0	0	0	0	0	0	0	0	0	0	0	0
PSIP1	0	0	0	0	0	0	0	0	0	0	0	0	0
PTBP1	0	0	0	0	0	0	1	1	1	0	0	0	0
RAD50	0	0	0	0	0	0	0	0	0	0	0	0	0
RAE1	0	0	0	0	0	0	1	1	0	0	0	0	0
RANBP2	0	0	0	0	0	0	1	1	0	0	0	0	0
RBBP4	0	0	0	0	1	0	0	0	0	0	0	0	0
RBBP7	0	0	0	0	0	0	0	0	0	0	0	0	0
RBM5	0	0	0	0	0	0	1	1	1	0	0	0	0
RCC1	0	0	0	0	0	0	0	0	0	0	0	0	0
RCC2	0	0	0	0	0	0	0	0	0	0	0	0	0
RNMT	0	0	0	0	0	0	1	0	0	0	1	1	0
RNPS1	0	0	0	0	0	0	1	1	1	0	1	1	1
RSF1	0	0	0	0	0	0	0	0	0	0	0	0	0
SAMM50	0	0	0	0	0	0	0	0	0	0	0	0	0
SEH1L	0	0	0	0	0	0	1	1	0	0	0	0	0
SIN3A	0	0	0	0	0	0	0	0	0	0	0	0	0
SLC12A7	0	0	0	0	0	0	0	0	0	0	0	0	0
SLC25A13	0	0	0	0	0	0	0	0	0	0	0	0	0
SLC25A4	0	0	0	0	0	0	0	0	0	0	0	0	0
SLC2A1	0	0	0	0	0	0	0	0	0	0	0	0	0
SLC2A3	0	0	0	0	0	0	0	0	0	0	0	0	0
SLC4A7	0	0	0	0	0	0	0	0	0	0	0	0	0
SLC7A3	0	0	0	0	0	0	0	0	0	0	0	0	0
SMARCA5	0	0	0	0	0	0	0	0	0	0	0	0	0
SMN2	0	0	0	0	0	0	0	0	0	0	0	0	0
SRRM1	0	0	0	0	0	0	1	1	1	0	1	1	1
SRSF11	0	0	0	0	0	0	1	1	1	0	1	1	1
SSRP1	0	0	0	0	0	0	0	0	0	0	1	1	0
SUGP1	0	0	0	0	0	0	1	1	1	0	0	0	0
SUPT16H	0	0	0	0	0	0	0	0	0	0	1	1	0
SUPT5H	0	0	0	0	0	0	1	0	0	0	1	1	0
SYNE2	0	0	0	0	0	0	0	0	0	0	0	0	0
TAF10	0	0	0	0	0	0	0	0	0	0	1	1	0
TAF4	0	0	0	0	0	0	0	0	0	0	1	1	0
TAF5	0	0	0	0	0	0	0	0	0	0	1	1	0
TBP	0	0	0	0	0	1	0	0	0	0	1	1	0
TCEA1	0	0	0	0	0	0	0	0	0	0	1	1	0
TERF2	0	0	0	0	0	0	0	0	0	0	0	0	0
TFAM	0	0	0	0	0	1	0	0	0	0	1	0	0
TIMM10	0	0	0	0	0	0	0	0	0	0	0	0	0
TIMM13	0	0	0	0	0	0	0	0	0	0	0	0	0
TIMM44	0	0	0	0	0	0	0	0	0	0	0	0	0
TIMM50	0	0	0	0	0	0	0	0	0	0	0	0	0

NAME	REACTOME_MRNA_3_END_PROCESSING	REACTOME_RNA_POL_II_PRE_TRANSCRIPTION_EVENTS	REACTOME_METABOLISM_OF_NON_CODING_RNA	REACTOME_TRANSPORT_OF_MATURE_TRANSCRIPT_TO_CYTOPLASM	REACTOME_TRANSPORT_OF_MATURE_MRNA_DERIVED_FROM_AN_INTRONLESS_TRANSCRIPT	REACTOME_INTERACTIONS_OF_VPR_WITH_HOST_CELLULAR_PROTEINS	REACTOME_GLUCOSE_TRANSPORT	REACTOME_REGULATION_OF_GLUCOKINASE_BY_GLUCOKINASE_REGULATORY_PROTEIN	REACTOME_TRANSPORT_OF_RIBONUCLEOPROTEINS_INTO_THE_HOST_NUCLEUS	REACTOME_NEP_NS2_INTERACTS_WITH_THE_CELLULAR_EXPORT_MACHINERY	REACTOME_SLC_MEDIATED_TRANSMEMBRANE_TRANSPORT	REACTOME_ANTIVIRAL_MECHANISM_BY_IFN_STIMULATED_GENES	REACTOME_HIV_LIFE_CYCLE
NUP43	0	0	1	1	1	1	1	1	1	1	1	1	1
NUP50	0	0	1	1	1	1	1	1	1	1	1	1	1
NUP54	0	0	1	1	1	1	1	1	1	1	1	1	1
NUP62	0	0	1	1	1	1	1	1	1	1	1	1	1
NUP88	0	0	1	1	1	1	1	1	1	1	0	0	1
NUP93	0	0	1	1	1	1	1	1	1	1	1	1	1
NUPL1	0	0	1	1	1	1	1	1	1	1	1	1	1
PABPN1	1	0	0	0	0	0	0	0	0	0	0	0	0
PCM1	0	0	0	0	0	0	0	0	0	0	0	0	0
POLB	0	0	0	0	0	0	0	0	0	0	0	0	0
POLRMT	0	0	0	0	0	0	0	0	0	0	0	0	0
PRKAR2B	0	0	0	0	0	0	0	0	0	0	0	0	0
PRKDC	0	0	0	0	0	0	0	0	0	0	0	0	0
PSIP1	0	0	0	0	0	1	0	0	0	0	0	0	1
PTBP1	0	0	0	0	0	0	0	0	0	0	0	0	0
RAD50	0	0	0	0	0	0	0	0	0	0	0	0	0
RAE1	0	0	1	1	1	1	1	1	1	1	0	0	1
RANBP2	0	0	1	1	1	1	1	1	1	1	1	1	1
RBBP4	0	0	0	0	0	0	0	0	0	0	0	0	0
RBBP7	0	0	0	0	0	0	0	0	0	0	0	0	0
RBM5	0	0	0	0	0	0	0	0	0	0	0	0	0
RCC1	0	0	0	0	0	0	0	0	0	0	0	0	1
RCC2	0	0	0	0	0	0	0	0	0	0	0	0	0
RNMT	0	0	0	0	0	0	0	0	0	0	0	0	1
RNPS1	1	0	0	1	0	0	0	0	0	0	0	0	0
RSF1	0	0	0	0	0	0	0	0	0	0	0	0	0
SAMM50	0	0	0	0	0	0	0	0	0	0	0	0	0
SEH1L	0	0	1	1	1	1	1	1	1	1	1	1	1
SIN3A	0	0	0	0	0	0	0	0	0	0	0	0	0
SLC12A7	0	0	0	0	0	0	0	0	0	0	1	0	0
SLC25A13	0	0	0	0	0	0	0	0	0	0	0	0	0
SLC25A4	0	0	0	0	0	1	0	0	0	0	0	0	0
SLC2A1	0	0	0	0	0	0	1	0	0	0	1	0	0
SLC2A3	0	0	0	0	0	0	1	0	0	0	1	0	0
SLC4A7	0	0	0	0	0	0	0	0	0	0	1	0	0
SLC7A3	0	0	0	0	0	0	0	0	0	0	1	0	0
SMARCA5	0	0	0	0	0	0	0	0	0	0	0	0	0
SMN2	0	0	1	0	0	0	0	0	0	0	0	0	0
SRRM1	1	0	0	1	0	0	0	0	0	0	0	0	0
SRSF11	1	0	0	1	0	0	0	0	0	0	0	0	0
SSRP1	0	1	0	0	0	0	0	0	0	0	0	0	1
SUGP1	0	0	0	0	0	0	0	0	0	0	0	0	0
SUPT16H	0	1	0	0	0	0	0	0	0	0	0	0	1
SUPT5H	0	1	0	0	0	0	0	0	0	0	0	0	1
SYNE2	0	0	0	0	0	0	0	0	0	0	0	0	0
TAF10	0	1	0	0	0	0	0	0	0	0	0	0	1
TAF4	0	1	0	0	0	0	0	0	0	0	0	0	1
TAF5	0	1	0	0	0	0	0	0	0	0	0	0	1
TBP	0	1	0	0	0	0	0	0	0	0	0	0	1
TCEA1	0	1	0	0	0	0	0	0	0	0	0	0	1
TERF2	0	0	0	0	0	0	0	0	0	0	0	0	0
TFAM	0	0	0	0	0	0	0	0	0	0	0	0	0
TIMM10	0	0	0	0	0	0	0	0	0	0	0	0	0
TIMM13	0	0	0	0	0	0	0	0	0	0	0	0	0
TIMM44	0	0	0	0	0	0	0	0	0	0	0	0	0
TIMM50	0	0	0	0	0	0	0	0	0	0	0	0	0

NAME	REACTOME_HIV_INFECTION	KEGG_SPLICEOSOME_	KEGG_RNA_DEGRADATION_	KEGG_ARGININE_AND_PROLINE_METABOLISM_	KEGG_FATTY_ACID_METABOLISM_	KEGG_VALINE_LEUCINE_AND_ISOLEUCINE_DEGRADATION_	KEGG_LYSINE_DEGRADATION_	KEGG_PYRIMIDINE_METABOLISM_	KEGG_DNA_REPLICATION_	KEGG_CELL_CYCLE_	KEGG_OOCYTE_MEIOSIS_
NUP43	1	0	0	0	0	0	0	0	0	0	0
NUP50	1	0	0	0	0	0	0	0	0	0	0
NUP54	1	0	0	0	0	0	0	0	0	0	0
NUP62	1	0	0	0	0	0	0	0	0	0	0
NUP88	0	0	0	0	0	0	0	0	0	0	0
NUP93	1	0	0	0	0	0	0	0	0	0	0
NUPL1	1	0	0	0	0	0	0	0	0	0	0
PABPN1	0	0	0	0	0	0	0	0	0	0	0
PCM1	0	0	0	0	0	0	0	0	0	0	0
POLB	0	0	0	0	0	0	0	0	0	0	0
POLRMT	0	0	0	0	0	0	0	0	0	0	0
PRKAR2B	0	0	0	0	0	0	0	0	0	0	0
PRKDC	0	0	0	0	0	0	0	0	0	0	0
PSIP1	1	0	0	0	0	0	0	0	0	0	0
PTBP1	0	0	0	0	0	0	0	0	0	0	0
RAD50	0	0	0	0	0	0	0	0	0	0	0
RAE1	0	0	0	0	0	0	0	0	0	0	0
RANBP2	1	0	0	0	0	0	0	0	0	0	0
RBBP4	0	0	0	0	0	0	0	0	0	0	0
RBBP7	0	0	0	0	0	0	0	0	0	0	0
RBM5	0	0	0	0	0	0	0	0	0	0	0
RCC1	1	0	0	0	0	0	0	0	0	0	0
RCC2	0	0	0	0	0	0	0	0	0	0	0
RNMT	1	0	0	0	0	0	0	0	0	0	0
RNPS1	0	0	0	0	0	0	0	0	0	0	0
RSF1	0	0	0	0	0	0	0	0	0	0	0
SAMM50	0	0	0	0	0	0	0	0	0	0	0
SEH1L	1	0	0	0	0	0	0	0	0	0	0
SIN3A	0	0	0	0	0	0	0	0	0	0	0
SLC12A7	0	0	0	0	0	0	0	0	0	0	0
SLC25A13	0	0	0	0	0	0	0	0	0	0	0
SLC25A4	1	0	0	0	0	0	0	0	0	0	0
SLC2A1	0	0	0	0	0	0	0	0	0	0	0
SLC2A3	0	0	0	0	0	0	0	0	0	0	0
SLC4A7	0	0	0	0	0	0	0	0	0	0	0
SLC7A3	0	0	0	0	0	0	0	0	0	0	0
SMARCA5	0	0	0	0	0	0	0	0	0	0	0
SMN2	0	0	0	0	0	0	0	0	0	0	0
SRRM1	0	0	0	0	0	0	0	0	0	0	0
SRSF11	0	0	0	0	0	0	0	0	0	0	0
SSRP1	1	0	0	0	0	0	0	0	0	0	0
SUGP1	0	0	0	0	0	0	0	0	0	0	0
SUPT16H	1	0	0	0	0	0	0	0	0	0	0
SUPT5H	0	0	0	0	0	0	0	0	0	0	0
SYNE2	0	0	0	0	0	0	0	0	0	0	0
TAF10	0	0	0	0	0	0	0	0	0	0	0
TAF4	1	0	0	0	0	0	0	0	0	0	0
TAF5	1	0	0	0	0	0	0	0	0	0	0
TBP	1	0	0	0	0	0	0	0	0	0	0
TCEA1	1	0	0	0	0	0	0	0	0	0	0
TERF2	0	0	0	0	0	0	0	0	0	0	0
TFAM	0	0	0	0	0	0	0	0	0	0	0
TIMM10	0	0	0	0	0	0	0	0	0	0	0
TIMM13	0	0	0	0	0	0	0	0	0	0	0
TIMM44	0	0	0	0	0	0	0	0	0	0	0
TIMM50	0	0	0	0	0	0	0	0	0	0	0

NAME
NUP43
NUP50
NUP54
NUP62
NUP88
NUP93
NUPL1
PABPN1
PCM1
POLB
POLRMT
PRKAR2B
PRKDC
PSIP1
PTBP1
RAD50
RAE1
RANBP2
RBBP4
RBBP7
RBM5
RCC1
RCC2
RNMT
RNPS1
RSF1
SAMM50
SEH1L
SIN3A
SLC12A7
SLC25A13
SLC25A4
SLC2A1
SLC2A3
SLC4A7
SLC7A3
SMARCA5
SMN2
SRRM1
SRSF11
SSRP1
SUGP1
SUPT16H
SUPT5H
SYNE2
TAF10
TAF4
TAF5
TBP
TCEA1
TERF2
TFAM
TIMM10
TIMM13
TIMM44
TIMM50

Table S6 The differential proteins contribute to the enrichment of the significant pathways for the public dataset

Core enrichment proteins in up-regulated pathways of hESCs/HFs (hESCs vs HFs)

NAME	REACTOME_MITOCHONDRIAL_PROTEIN_IMPORT	REACTOME_DEADENYLATION_DEPENDENT_MRNA_DECAY	REACTOME_FACTORS_INVOLVED_IN_MEGAKARYOCYTE_DEVELOPMENT_AND_PLATELET_PRODUCTION	REACTOME_DNA_REPAIR	REACTOME_NUCLEOTIDE_EXCISION_REPAIR	REACTOME_MEIOSIS	REACTOME_MITOTIC_PROMETAPHASE	REACTOME_CHROMOSOME_MAINTENANCE	REACTOME_TELOMERE_MAINTENANCE	REACTOME_CELL_CYCLE	REACTOME_CELL_CYCLE_MITOTIC	REACTOME_DNA_REPLICATION	REACTOME_MITOTIC_M_M_G1_PHASES
TIMM8A	1	0	0	0	0	0	0	0	0	0	0	0	0
TIMM9	1	0	0	0	0	0	0	0	0	0	0	0	0
TOMM22	1	0	0	0	0	0	0	0	0	0	0	0	0
TOMM40	1	0	0	0	0	0	0	0	0	0	0	0	0
TPR	0	0	0	0	0	0	0	0	0	0	0	0	0
TUBA4A	0	0	0	0	0	0	0	0	0	1	1	0	0
UBTF	0	0	0	0	0	0	0	0	0	0	0	0	0
XPO1	0	0	0	0	0	0	0	0	0	0	0	0	0
XRCC1	0	0	0	1	0	0	0	0	0	0	0	0	0
XRCC5	0	0	0	1	0	0	0	0	0	0	0	0	0
XRCC6	0	0	0	1	0	0	0	0	0	0	0	0	0
AASS	0	0	0	0	0	0	0	0	0	0	0	0	0
ACAA2	0	0	0	0	0	0	0	0	0	0	0	0	0
ACIN1	0	0	0	0	0	0	0	0	0	0	0	0	0
ALDH1B1	0	0	0	0	0	0	0	0	0	0	0	0	0
ALDH3A2	0	0	0	0	0	0	0	0	0	0	0	0	0
ALDH6A1	0	0	0	0	0	0	0	0	0	0	0	0	0
ALDH7A1	0	0	0	0	0	0	0	0	0	0	0	0	0
AQR	0	0	0	0	0	0	0	0	0	0	0	0	0
ARG2	0	0	0	0	0	0	0	0	0	0	0	0	0
AUH	0	0	0	0	0	0	0	0	0	0	0	0	0
BCAS2	0	0	0	0	0	0	0	0	0	0	0	0	0
CAD	0	0	0	0	0	0	0	0	0	0	0	0	0
CKB	0	0	0	0	0	0	0	0	0	0	0	0	0
CKMT1B	0	0	0	0	0	0	0	0	0	0	0	0	0
CPT2	0	0	0	0	0	0	0	0	0	0	0	0	0
CTNNBL1	0	0	0	0	0	0	0	0	0	0	0	0	0
CTPS2	0	0	0	0	0	0	0	0	0	0	0	0	0
CWC15	0	0	0	0	0	0	0	0	0	0	0	0	0
DBT	0	0	0	0	0	0	0	0	0	0	0	0	0
DDX42	0	0	0	0	0	0	0	0	0	0	0	0	0
DDX5	0	0	0	0	0	0	0	0	0	0	0	0	0
DHX15	0	0	0	0	0	0	0	0	0	0	0	0	0
DHX8	0	0	0	0	0	0	0	0	0	0	0	0	0
DUT	0	0	0	0	0	0	0	0	0	0	0	0	0
EXOSC10	0	0	0	0	0	0	0	0	0	0	0	0	0
GCDH	0	0	0	0	0	0	0	0	0	0	0	0	0
HADH	0	0	0	0	0	0	0	0	0	0	0	0	0
HMGCS1	0	0	0	0	0	0	0	0	0	0	0	0	0
ISY1	0	0	0	0	0	0	0	0	0	0	0	0	0
LSM7	0	0	0	0	0	0	0	0	0	0	0	0	0
MCCC2	0	0	0	0	0	0	0	0	0	0	0	0	0
NME3	0	0	0	0	0	0	0	0	0	0	0	0	0
PIPOX	0	0	0	0	0	0	0	0	0	0	0	0	0
PNP	0	0	0	0	0	0	0	0	0	0	0	0	0
PNPT1	0	0	0	0	0	0	0	0	0	0	0	0	0
POLA2	0	0	0	0	0	0	0	0	0	0	0	0	0
POLR1C	0	0	0	0	0	0	0	0	0	0	0	0	0
POLR1E	0	0	0	0	0	0	0	0	0	0	0	0	0
PPIH	0	0	0	0	0	0	0	0	0	0	0	0	0
PPIL1	0	0	0	0	0	0	0	0	0	0	0	0	0
PQBP1	0	0	0	0	0	0	0	0	0	0	0	0	0
PRODH	0	0	0	0	0	0	0	0	0	0	0	0	0
PRPF19	0	0	0	0	0	0	0	0	0	0	0	0	0
PRPF3	0	0	0	0	0	0	0	0	0	0	0	0	0
PRPF38A	0	0	0	0	0	0	0	0	0	0	0	0	0

NAME	REACTOME_CELL_CYCLE_CHECKPOINTS	REACTOME_S_PHASE	REACTOME_SYNTHESIS_OF_DNA	REACTOME_G1_S_TRANSITION	REACTOME_MITOTIC_G1_G1_S_PHASES	REACTOME_RNA_POL_I_RNA_POL_III_AND_MITOCHONDRIAL_TRANSCRIPTION	REACTOME_MRNA_PROCESSING	REACTOME_PROCESSING_OF_CAPPED_INTRON_CONTAINING_PRE_MRNA	REACTOME_MRNA_SPLICING	REACTOME_MRNA_SPLICING_MINOR_PATHWAY	REACTOME_TRANSCRIPTION	REACTOME_RNA_POL_II_TRANSCRIPTION	REACTOME_CLEAVAGE_OF_GROWING_TRANSCRIPT_IN_THE_TERMINATION_REGION_
TIMM8A	0	0	0	0	0	0	0	0	0	0	0	0	0
TIMM9	0	0	0	0	0	0	0	0	0	0	0	0	0
TOMM22	0	0	0	0	0	0	0	0	0	0	0	0	0
TOMM40	0	0	0	0	0	0	0	0	0	0	0	0	0
TPR	0	0	0	0	0	0	1	1	0	0	0	0	0
TUBA4A	0	0	0	0	0	0	0	0	0	0	0	0	0
UBTF	0	0	0	0	0	1	0	0	0	0	1	0	0
XPO1	0	0	0	0	0	0	0	0	0	0	0	0	0
XRCC1	0	0	0	0	0	0	0	0	0	0	0	0	0
XRCC5	0	0	0	0	0	0	0	0	0	0	0	0	0
XRCC6	0	0	0	0	0	0	0	0	0	0	0	0	0
AASS	0	0	0	0	0	0	0	0	0	0	0	0	0
ACAA2	0	0	0	0	0	0	0	0	0	0	0	0	0
ACIN1	0	0	0	0	0	0	0	0	0	0	0	0	0
ALDH1B1	0	0	0	0	0	0	0	0	0	0	0	0	0
ALDH3A2	0	0	0	0	0	0	0	0	0	0	0	0	0
ALDH6A1	0	0	0	0	0	0	0	0	0	0	0	0	0
ALDH7A1	0	0	0	0	0	0	0	0	0	0	0	0	0
AQR	0	0	0	0	0	0	0	0	0	0	0	0	0
ARG2	0	0	0	0	0	0	0	0	0	0	0	0	0
AUH	0	0	0	0	0	0	0	0	0	0	0	0	0
BCAS2	0	0	0	0	0	0	0	0	0	0	0	0	0
CAD	0	0	0	0	0	0	0	0	0	0	0	0	0
CKB	0	0	0	0	0	0	0	0	0	0	0	0	0
CKMT1B	0	0	0	0	0	0	0	0	0	0	0	0	0
CPT2	0	0	0	0	0	0	0	0	0	0	0	0	0
CTNNBL1	0	0	0	0	0	0	0	0	0	0	0	0	0
CTPS2	0	0	0	0	0	0	0	0	0	0	0	0	0
CWC15	0	0	0	0	0	0	0	0	0	0	0	0	0
DBT	0	0	0	0	0	0	0	0	0	0	0	0	0
DDX42	0	0	0	0	0	0	0	0	0	0	0	0	0
DDX5	0	0	0	0	0	0	0	0	0	0	0	0	0
DHX15	0	0	0	0	0	0	0	0	0	0	0	0	0
DHX8	0	0	0	0	0	0	0	0	0	0	0	0	0
DUT	0	0	0	0	0	0	0	0	0	0	0	0	0
EXOSC10	0	0	0	0	0	0	0	0	0	0	0	0	0
GCDH	0	0	0	0	0	0	0	0	0	0	0	0	0
HADH	0	0	0	0	0	0	0	0	0	0	0	0	0
HMGCS1	0	0	0	0	0	0	0	0	0	0	0	0	0
ISY1	0	0	0	0	0	0	0	0	0	0	0	0	0
LSM7	0	0	0	0	0	0	0	0	0	0	0	0	0
MCCC2	0	0	0	0	0	0	0	0	0	0	0	0	0
NME3	0	0	0	0	0	0	0	0	0	0	0	0	0
PIPOX	0	0	0	0	0	0	0	0	0	0	0	0	0
PNP	0	0	0	0	0	0	0	0	0	0	0	0	0
PNPT1	0	0	0	0	0	0	0	0	0	0	0	0	0
POLA2	0	0	0	0	0	0	0	0	0	0	0	0	0
POLR1C	0	0	0	0	0	0	0	0	0	0	0	0	0
POLR1E	0	0	0	0	0	0	0	0	0	0	0	0	0
PPIH	0	0	0	0	0	0	0	0	0	0	0	0	0
PPIL1	0	0	0	0	0	0	0	0	0	0	0	0	0
PQBP1	0	0	0	0	0	0	0	0	0	0	0	0	0
PRODH	0	0	0	0	0	0	0	0	0	0	0	0	0
PRPF19	0	0	0	0	0	0	0	0	0	0	0	0	0
PRPF3	0	0	0	0	0	0	0	0	0	0	0	0	0
PRPF38A	0	0	0	0	0	0	0	0	0	0	0	0	0

NAME	REACTOME_MRNA_3_END_PROCESSING	REACTOME_RNA_POL_II_PRE_TRANSCRIPTION_EVENTS	REACTOME_METABOLISM_OF_NON_CODING_RNA	REACTOME_TRANSPORT_OF_MATURE_TRANSCRIPT_TO_CYTOPLASM	REACTOME_TRANSPORT_OF_MATURE_MRNA_DERIVED_FROM_AN_INTRONLESS_TRANSCRIPT	REACTOME_INTERACTIONS_OF_VPR_WITH_HOST_CELLULAR_PROTEINS	REACTOME_GLUCOSE_TRANSPORT	REACTOME_REGULATION_OF_GLUCOKINASE_BY_GLUCOKINASE_REGULATORY_PROTEIN	REACTOME_TRANSPORT_OF_RIBONUCLEOPROTEINS_INTO_THE_HOST_NUCLEUS	REACTOME_NEP_NS2_INTERACTS_WITH_THE_CELLULAR_EXPORT_MACHINERY	REACTOME_SLC_MEDIATED_TRANSMEMBRANE_TRANSPORT	REACTOME_ANTIVIRAL_MECHANISM_BY_IFN_STIMULATED_GENES	REACTOME_HIV_LIFE_CYCLE
TIMM8A	0	0	0	0	0	0	0	0	0	0	0	0	0
TIMM9	0	0	0	0	0	0	0	0	0	0	0	0	0
TOMM22	0	0	0	0	0	0	0	0	0	0	0	0	0
TOMM40	0	0	0	0	0	0	0	0	0	0	0	0	0
TPR	0	0	1	1	1	1	1	1	1	1	1	1	1
TUBA4A	0	0	0	0	0	0	0	0	0	0	0	0	0
UBTF	0	0	0	0	0	0	0	0	0	0	0	0	0
XPO1	0	0	0	0	0	0	0	0	0	0	0	0	1
XRCC1	0	0	0	0	0	0	0	0	0	0	0	0	0
XRCC5	0	0	0	0	0	0	0	0	0	0	0	0	1
XRCC6	0	0	0	0	0	0	0	0	0	0	0	0	1
AASS	0	0	0	0	0	0	0	0	0	0	0	0	0
ACAA2	0	0	0	0	0	0	0	0	0	0	0	0	0
ACIN1	0	0	0	0	0	0	0	0	0	0	0	0	0
ALDH1B1	0	0	0	0	0	0	0	0	0	0	0	0	0
ALDH3A2	0	0	0	0	0	0	0	0	0	0	0	0	0
ALDH6A1	0	0	0	0	0	0	0	0	0	0	0	0	0
ALDH7A1	0	0	0	0	0	0	0	0	0	0	0	0	0
AQR	0	0	0	0	0	0	0	0	0	0	0	0	0
ARG2	0	0	0	0	0	0	0	0	0	0	0	0	0
AUH	0	0	0	0	0	0	0	0	0	0	0	0	0
BCAS2	0	0	0	0	0	0	0	0	0	0	0	0	0
CAD	0	0	0	0	0	0	0	0	0	0	0	0	0
CKB	0	0	0	0	0	0	0	0	0	0	0	0	0
CKMT1B	0	0	0	0	0	0	0	0	0	0	0	0	0
CPT2	0	0	0	0	0	0	0	0	0	0	0	0	0
CTNNBL1	0	0	0	0	0	0	0	0	0	0	0	0	0
CTPS2	0	0	0	0	0	0	0	0	0	0	0	0	0
CWC15	0	0	0	0	0	0	0	0	0	0	0	0	0
DBT	0	0	0	0	0	0	0	0	0	0	0	0	0
DDX42	0	0	0	0	0	0	0	0	0	0	0	0	0
DDX5	0	0	0	0	0	0	0	0	0	0	0	0	0
DHX15	0	0	0	0	0	0	0	0	0	0	0	0	0
DHX8	0	0	0	0	0	0	0	0	0	0	0	0	0
DUT	0	0	0	0	0	0	0	0	0	0	0	0	0
EXOSC10	0	0	0	0	0	0	0	0	0	0	0	0	0
GCDH	0	0	0	0	0	0	0	0	0	0	0	0	0
HADH	0	0	0	0	0	0	0	0	0	0	0	0	0
HMGCS1	0	0	0	0	0	0	0	0	0	0	0	0	0
ISY1	0	0	0	0	0	0	0	0	0	0	0	0	0
LSM7	0	0	0	0	0	0	0	0	0	0	0	0	0
MCCC2	0	0	0	0	0	0	0	0	0	0	0	0	0
NME3	0	0	0	0	0	0	0	0	0	0	0	0	0
PIPOX	0	0	0	0	0	0	0	0	0	0	0	0	0
PNP	0	0	0	0	0	0	0	0	0	0	0	0	0
PNPT1	0	0	0	0	0	0	0	0	0	0	0	0	0
POLA2	0	0	0	0	0	0	0	0	0	0	0	0	0
POLR1C	0	0	0	0	0	0	0	0	0	0	0	0	0
POLR1E	0	0	0	0	0	0	0	0	0	0	0	0	0
PPIH	0	0	0	0	0	0	0	0	0	0	0	0	0
PPIL1	0	0	0	0	0	0	0	0	0	0	0	0	0
PQBP1	0	0	0	0	0	0	0	0	0	0	0	0	0
PRODH	0	0	0	0	0	0	0	0	0	0	0	0	0
PRPF19	0	0	0	0	0	0	0	0	0	0	0	0	0
PRPF3	0	0	0	0	0	0	0	0	0	0	0	0	0
PRPF38A	0	0	0	0	0	0	0	0	0	0	0	0	0

NAME	REACTOME_HIV_INFECTION	KEGG_SPLICEOSOME_	KEGG_RNA_DEGRADATION_	KEGG_ARGININE_AND_PROLINE_METABOLISM_	KEGG_FATTY_ACID_METABOLISM_	KEGG_VALINE_LEUCINE_AND_ISOLEUCINE_DEGRADATION_	KEGG_LYSINE_DEGRADATION_	KEGG_PYRIMIDINE_METABOLISM_	KEGG_DNA_REPLICATION_	KEGG_CELL_CYCLE_	KEGG_OOCYTE_MEIOSIS_
TIMM8A	0	0	0	0	0	0	0	0	0	0	0
TIMM9	0	0	0	0	0	0	0	0	0	0	0
TOMM22	0	0	0	0	0	0	0	0	0	0	0
TOMM40	0	0	0	0	0	0	0	0	0	0	0
TPR	1	0	0	0	0	0	0	0	0	0	0
TUBA4A	0	0	0	0	0	0	0	0	0	0	0
UBTF	0	0	0	0	0	0	0	0	0	0	0
XPO1	0	0	0	0	0	0	0	0	0	0	0
XRCC1	0	0	0	0	0	0	0	0	0	0	0
XRCC5	1	0	0	0	0	0	0	0	0	0	0
XRCC6	1	0	0	0	0	0	0	0	0	0	0
AASS	0	0	0	0	0	0	0	0	0	0	0
ACAA2	0	0	0	0	0	0	0	0	0	0	0
ACIN1	0	0	0	0	0	0	0	0	0	0	0
ALDH1B1	0	0	0	0	0	0	0	0	0	0	0
ALDH3A2	0	0	0	0	0	0	0	0	0	0	0
ALDH6A1	0	0	0	0	0	0	0	0	0	0	0
ALDH7A1	0	0	0	0	0	0	0	0	0	0	0
AQR	0	0	0	0	0	0	0	0	0	0	0
ARG2	0	0	0	0	0	0	0	0	0	0	0
AUH	0	0	0	0	0	0	0	0	0	0	0
BCAS2	0	0	0	0	0	0	0	0	0	0	0
CAD	0	0	0	0	0	0	0	0	0	0	0
CKB	0	0	0	0	0	0	0	0	0	0	0
CKMT1B	0	0	0	0	0	0	0	0	0	0	0
CPT2	0	0	0	0	0	0	0	0	0	0	0
CTNNBL1	0	0	0	0	0	0	0	0	0	0	0
CTPS2	0	0	0	0	0	0	0	0	0	0	0
CWC15	0	0	0	0	0	0	0	0	0	0	0
DBT	0	0	0	0	0	0	0	0	0	0	0
DDX42	0	0	0	0	0	0	0	0	0	0	0
DDX5	0	0	0	0	0	0	0	0	0	0	0
DHX15	0	0	0	0	0	0	0	0	0	0	0
DHX8	0	0	0	0	0	0	0	0	0	0	0
DUT	0	0	0	0	0	0	0	0	0	0	0
EXOSC10	0	0	0	0	0	0	0	0	0	0	0
GCDH	0	0	0	0	0	0	0	0	0	0	0
HADH	0	0	0	0	0	0	0	0	0	0	0
HMGCS1	0	0	0	0	0	0	0	0	0	0	0
ISY1	0	0	0	0	0	0	0	0	0	0	0
LSM7	0	0	0	0	0	0	0	0	0	0	0
MCCC2	0	0	0	0	0	0	0	0	0	0	0
NME3	0	0	0	0	0	0	0	0	0	0	0
PIPOX	0	0	0	0	0	0	0	0	0	0	0
PNP	0	0	0	0	0	0	0	0	0	0	0
PNPT1	0	0	0	0	0	0	0	0	0	0	0
POLA2	0	0	0	0	0	0	0	0	0	0	0
POLR1C	0	0	0	0	0	0	0	0	0	0	0
POLR1E	0	0	0	0	0	0	0	0	0	0	0
PPIH	0	0	0	0	0	0	0	0	0	0	0
PPIL1	0	0	0	0	0	0	0	0	0	0	0
PQBP1	0	0	0	0	0	0	0	0	0	0	0
PRODH	0	0	0	0	0	0	0	0	0	0	0
PRPF19	0	0	0	0	0	0	0	0	0	0	0
PRPF3	0	0	0	0	0	0	0	0	0	0	0
PRPF38A	0	0	0	0	0	0	0	0	0	0	0

NAME
TIMM8A
TIMM9
TOMM22
TOMM40
TPR
TUBA4A
UBTF
XPO1
XRCC1
XRCC5
XRCC6
AASS
ACAA2
ACIN1
ALDH1B1
ALDH3A2
ALDH6A1
ALDH7A1
AQR
ARG2
AUH
BCAS2
CAD
CKB
CKMT1B
CPT2
CTNNBL1
CTPS2
CWC15
DBT
DDX42
DDX5
DHX15
DHX8
DUT
EXOSC10
GCDH
HADH
HMGCS1
ISY1
LSM7
MCCC2
NME3
PIPOX
PNP
PNPT1
POLA2
POLR1C
POLR1E
PPIH
PPIL1
PQBP1
PRODH
PRPF19
PRPF3
PRPF38A

Table S6 The differential proteins contribute to the enrichment of the significant pathways for the public dataset

Core enrichment proteins in up-regulated pathways of hESCs/HFs (hESCs vs HFs)

NAME	REACTOME_MITOCHONDRIAL_PROTEIN_IMPORT	REACTOME_DEADENYLATION_DEPENDENT_MRNA_DECAY	REACTOME_FACTORS_INVOLVED_IN_MEGAKARYOCYTE_DEVELOPMENT_AND_PLATELET_PRODUCTION	REACTOME_DNA_REPAIR	REACTOME_NUCLEOTIDE_EXCISION_REPAIR	REACTOME_MEIOSIS	REACTOME_MITOTIC_PROMETAPHASE	REACTOME_CHROMOSOME_MAINTENANCE	REACTOME_TELOMERE_MAINTENANCE	REACTOME_CELL_CYCLE	REACTOME_CELL_CYCLE_MITOTIC	REACTOME_DNA_REPLICATION	REACTOME_MITOTIC_M_M_G1_PHASES
RBM17	0	0	0	0	0	0	0	0	0	0	0	0	0
RBM25	0	0	0	0	0	0	0	0	0	0	0	0	0
RFC1	0	0	0	0	0	0	0	0	0	0	0	0	0
RNASEH2A	0	0	0	0	0	0	0	0	0	0	0	0	0
SART1	0	0	0	0	0	0	0	0	0	0	0	0	0
SKIV2L2	0	0	0	0	0	0	0	0	0	0	0	0	0
SMNDC1	0	0	0	0	0	0	0	0	0	0	0	0	0
SNRPC	0	0	0	0	0	0	0	0	0	0	0	0	0
SNW1	0	0	0	0	0	0	0	0	0	0	0	0	0
SRSF10	0	0	0	0	0	0	0	0	0	0	0	0	0
SRSF8	0	0	0	0	0	0	0	0	0	0	0	0	0
TCERG1	0	0	0	0	0	0	0	0	0	0	0	0	0
THOC1	0	0	0	0	0	0	0	0	0	0	0	0	0
THOC3	0	0	0	0	0	0	0	0	0	0	0	0	0
TRA2A	0	0	0	0	0	0	0	0	0	0	0	0	0
TRA2B	0	0	0	0	0	0	0	0	0	0	0	0	0
U2SURP	0	0	0	0	0	0	0	0	0	0	0	0	0
WBP11	0	0	0	0	0	0	0	0	0	0	0	0	0

NAME	REACTOME_CELL_CYCLE_CHECKPOINTS	REACTOME_S_PHASE	REACTOME_SYNTHESIS_OF_DNA	REACTOME_G1_S_TRANSITION	REACTOME_MITOTIC_G1_G1_S_PHASES	REACTOME_RNA_POL_I_RNA_POL_III_AND_MITOCHONDRIAL_TRANSCRIPTION	REACTOME_MRNA_PROCESSING	REACTOME_PROCESSING_OF_CAPPED_INTRON_CONTAINING_PRE_MRNA	REACTOME_MRNA_SPLICING	REACTOME_MRNA_SPLICING_MINOR_PATHWAY	REACTOME_TRANSCRIPTION	REACTOME_RNA_POL_II_TRANSCRIPTION	REACTOME_CLEAVAGE_OF_GROWING_TRANSCRIPT_IN_THE_TERMINATION_REGION_
RBM17	0	0	0	0	0	0	0	0	0	0	0	0	0
RBM25	0	0	0	0	0	0	0	0	0	0	0	0	0
RFC1	0	0	0	0	0	0	0	0	0	0	0	0	0
RNASEH2A	0	0	0	0	0	0	0	0	0	0	0	0	0
SART1	0	0	0	0	0	0	0	0	0	0	0	0	0
SKIV2L2	0	0	0	0	0	0	0	0	0	0	0	0	0
SMNDC1	0	0	0	0	0	0	0	0	0	0	0	0	0
SNRPC	0	0	0	0	0	0	0	0	0	0	0	0	0
SNW1	0	0	0	0	0	0	0	0	0	0	0	0	0
SRSF10	0	0	0	0	0	0	0	0	0	0	0	0	0
SRSF8	0	0	0	0	0	0	0	0	0	0	0	0	0
TCERG1	0	0	0	0	0	0	0	0	0	0	0	0	0
THOC1	0	0	0	0	0	0	0	0	0	0	0	0	0
THOC3	0	0	0	0	0	0	0	0	0	0	0	0	0
TRA2A	0	0	0	0	0	0	0	0	0	0	0	0	0
TRA2B	0	0	0	0	0	0	0	0	0	0	0	0	0
U2SURP	0	0	0	0	0	0	0	0	0	0	0	0	0
WBP11	0	0	0	0	0	0	0	0	0	0	0	0	0

NAME	REACTOME_MRNA_3_END_PROCESSING	REACTOME_RNA_POL_II_PRE_TRANSCRIPTION_EVENTS	REACTOME_METABOLISM_OF_NON_CODING_RNA	REACTOME_TRANSPORT_OF_MATURE_TRANSCRIPT_TO_CYTOPLASM	REACTOME_TRANSPORT_OF_MATURE_MRNA_DERIVED_FROM_AN_INTRONLESS_TRANSCRIPT	REACTOME_INTERACTIONS_OF_VPR_WITH_HOST_CELLULAR_PROTEINS	REACTOME_GLUCOSE_TRANSPORT	REACTOME_REGULATION_OF_GLUCOKINASE_BY_GLUCOKINASE_REGULATORY_PROTEIN	REACTOME_TRANSPORT_OF_RIBONUCLEOPROTEINS_INTO_THE_HOST_NUCLEUS	REACTOME_NEP_NS2_INTERACTS_WITH_THE_CELLULAR_EXPORT_MACHINERY	REACTOME_SLC_MEDIATED_TRANSMEMBRANE_TRANSPORT	REACTOME_ANTIVIRAL_MECHANISM_BY_IFN_STIMULATED_GENES	REACTOME_HIV_LIFE_CYCLE
RBM17	0	0	0	0	0	0	0	0	0	0	0	0	0
RBM25	0	0	0	0	0	0	0	0	0	0	0	0	0
RFC1	0	0	0	0	0	0	0	0	0	0	0	0	0
RNASEH2A	0	0	0	0	0	0	0	0	0	0	0	0	0
SART1	0	0	0	0	0	0	0	0	0	0	0	0	0
SKIV2L2	0	0	0	0	0	0	0	0	0	0	0	0	0
SMNDC1	0	0	0	0	0	0	0	0	0	0	0	0	0
SNRPC	0	0	0	0	0	0	0	0	0	0	0	0	0
SNW1	0	0	0	0	0	0	0	0	0	0	0	0	0
SRSF10	0	0	0	0	0	0	0	0	0	0	0	0	0
SRSF8	0	0	0	0	0	0	0	0	0	0	0	0	0
TCERG1	0	0	0	0	0	0	0	0	0	0	0	0	0
THOC1	0	0	0	0	0	0	0	0	0	0	0	0	0
THOC3	0	0	0	0	0	0	0	0	0	0	0	0	0
TRA2A	0	0	0	0	0	0	0	0	0	0	0	0	0
TRA2B	0	0	0	0	0	0	0	0	0	0	0	0	0
U2SURP	0	0	0	0	0	0	0	0	0	0	0	0	0
WBP11	0	0	0	0	0	0	0	0	0	0	0	0	0

NAME	REACTOME_HIV_INFECTION	KEGG_SPLICEOSOME_	KEGG_RNA_DEGRADATION_	KEGG_ARGININE_AND_PROLINE_METABOLISM_	KEGG_FATTY_ACID_METABOLISM_	KEGG_VALINE_LEUCINE_AND_ISOLEUCINE_DEGRADATION_	KEGG_LYSINE_DEGRADATION_	KEGG_PYRIMIDINE_METABOLISM_	KEGG_DNA_REPLICATION_	KEGG_CELL_CYCLE_	KEGG_OOCYTE_MEIOSIS_
RBM17	0	0	0	0	0	0	0	0	0	0	0
RBM25	0	0	0	0	0	0	0	0	0	0	0
RFC1	0	0	0	0	0	0	0	0	0	0	0
RNASEH2A	0	0	0	0	0	0	0	0	0	0	0
SART1	0	0	0	0	0	0	0	0	0	0	0
SKIV2L2	0	0	0	0	0	0	0	0	0	0	0
SMNDC1	0	0	0	0	0	0	0	0	0	0	0
SNRPC	0	0	0	0	0	0	0	0	0	0	0
SNW1	0	0	0	0	0	0	0	0	0	0	0
SRSF10	0	0	0	0	0	0	0	0	0	0	0
SRSF8	0	0	0	0	0	0	0	0	0	0	0
TCERG1	0	0	0	0	0	0	0	0	0	0	0
THOC1	0	0	0	0	0	0	0	0	0	0	0
THOC3	0	0	0	0	0	0	0	0	0	0	0
TRA2A	0	0	0	0	0	0	0	0	0	0	0
TRA2B	0	0	0	0	0	0	0	0	0	0	0
U2SURP	0	0	0	0	0	0	0	0	0	0	0
WBP11	0	0	0	0	0	0	0	0	0	0	0

NAME
RBM17
RBM25
RFC1
RNASEH2A
SART1
SKIV2L2
SMNDC1
SNRPC
SNW1
SRSF10
SRSF8
TCERG1
THOC1
THOC3
TRA2A
TRA2B
U2SURP
WBP11

Table S6 The differential proteins contribute to the enrichment of the significant pathways for the public dataset

Core enrichment proteins in down-regulated pathways of hiPSCs/hESCs(hiPSCs vs hESCs)

NAME	REACTOME_RESPIRATORY_ELECTRON_TRANSPORT_ATP_SYNTHESIS_BY_CHEMIOSMOTIC_COUPLING_AND_HEAT_PRODUCTION_BY_UNCOUPLING_PROTEI	REACTOME_TCA_CYCLE_AND_RESPIRATORY_ELECTRON_TRANSPORT	REACTOME_PYRUVATE_METABOLISM_AND_CITRIC_ACID_TCA_CYCLE	REACTOME_PROCESSING_OF_CAPPED_INTRON_CONTAINING_PRE_MRNA	REACTOME_NEP_NS2_INTERACTS_WITH_THE_CELLULAR_EXPORT_MACHINERY	REACTOME_TRANSPORT_OF_RIBONUCLEOPROTEINS_INTO_THE_HOST_NUCLEUS	REACTOME_REGULATION_OF_GLUCOKINASE_BY_GLUCOKINASE_REGULATORY_PROTEIN	REACTOME_GLUCOSE_TRANSPORT	KEGG_FATTY_ACID_METABOLISM	KEGG_ARGININE_AND_PROLINE_METABOLISM	KEGG_PEROXISOME
SDHA	1	1	1	0	0	0	0	0	0	0	0
SDHB	1	1	1	0	0	0	0	0	0	0	0
ATP5I	1	1	0	0	0	0	0	0	0	0	0
ATP5H	1	1	0	0	0	0	0	0	0	0	0
ATP5O	1	1	0	0	0	0	0	0	0	0	0
UQCRH	1	1	0	0	0	0	0	0	0	0	0
ATP5B	1	1	0	0	0	0	0	0	0	0	0
ATP5D	1	1	0	0	0	0	0	0	0	0	0
CYC1	1	1	0	0	0	0	0	0	0	0	0
CYCS	1	1	0	0	0	0	0	0	0	0	0
ETFB	1	1	0	0	0	0	0	0	0	0	0
ATP5F1	1	1	0	0	0	0	0	0	0	0	0
NDUFB6	1	1	0	0	0	0	0	0	0	0	0
ATP5A1	1	1	0	0	0	0	0	0	0	0	0
NDUFA8	1	1	0	0	0	0	0	0	0	0	0
PDK3	0	1	1	0	0	0	0	0	0	0	0
PDHX	0	1	1	0	0	0	0	0	0	0	0
IDH2	0	1	1	0	0	0	0	0	1	1	1
DLD	0	1	1	0	0	0	0	0	0	0	0
FH	0	1	1	0	0	0	0	0	0	0	0
DLST	0	1	1	0	0	0	0	0	0	0	0
PDHB	0	1	1	0	0	0	0	0	0	0	0
PDPR	0	1	1	0	0	0	0	0	0	0	0
PDP1	0	1	1	0	0	0	0	0	0	0	0
SLC2A1	0	0	0	0	0	0	0	1	0	0	0
SLC2A3	0	0	0	0	0	0	0	1	0	0	0
XPO1	0	0	0	0	1	0	0	0	0	0	0
HNRNPU	0	0	0	1	0	0	0	0	0	0	0
CCAR1	0	0	0	1	0	0	0	0	0	0	0
EFTUD2	0	0	0	1	0	0	0	0	0	0	0
SNRPD2	0	0	0	1	0	0	0	0	0	0	0
SNRPD1	0	0	0	1	0	0	0	0	0	0	0
MAGOH	0	0	0	1	0	0	0	0	0	0	0
PAPOLA	0	0	0	1	0	0	0	0	0	0	0
SNRPD3	0	0	0	1	0	0	0	0	0	0	0
METTL3	0	0	0	1	0	0	0	0	0	0	0
GTF2F2	0	0	0	1	0	0	0	0	0	0	0
SF3B14	0	0	0	1	0	0	0	0	0	0	0
PRPF4	0	0	0	1	0	0	0	0	0	0	0
PRPF6	0	0	0	1	0	0	0	0	0	0	0
HNRNPUL1	0	0	0	1	0	0	0	0	0	0	0
CD2BP2	0	0	0	1	0	0	0	0	0	0	0
SRSF3	0	0	0	1	0	0	0	0	0	0	0
SNRPE	0	0	0	1	0	0	0	0	0	0	0
SRSF4	0	0	0	1	0	0	0	0	0	0	0
SNRPF	0	0	0	1	0	0	0	0	0	0	0
SNRNP200	0	0	0	1	0	0	0	0	0	0	0
SNRPA	0	0	0	1	0	0	0	0	0	0	0
SNRPB	0	0	0	1	0	0	0	0	0	0	0
SRSF9	0	0	0	1	0	0	0	0	0	0	0
RBM8A	0	0	0	1	0	0	0	0	0	0	0
DHX9	0	0	0	1	0	0	0	0	0	0	0

Table S6 The differential proteins contribute to the enrichment of the significant pathways for the public dataset

Core enrichment proteins in down-regulated pathways of hiPSCs/hESCs(hiPSCs vs hESCs)

NAME	REACTOME_RESPIRATORY_ELECTRON_TRANSPORT_ATP_SYNTHESIS_BY_CHEMIOSMOTIC_COUPLING_AND_HEAT_PRODUCTION_BY_UNCOUPLING_PROTEI	REACTOME_TCA_CYCLE_AND_RESPIRATORY_ELECTRON_TRANSPORT	REACTOME_PYRUVATE_METABOLISM_AND_CITRIC_ACID_TCA_CYCLE	REACTOME_PROCESSING_OF_CAPPED_INTRON_CONTAINING_PRE_MRNA	REACTOME_NEP_NS2_INTERACTS_WITH_THE_CELLULAR_EXPORT_MACHINERY	REACTOME_TRANSPORT_OF_RIBONUCLEOPROTEINS_INTO_THE_HOST_NUCLEUS	REACTOME_REGULATION_OF_GLUCOKINASE_BY_GLUCOKINASE_REGULATORY_PROTEIN	REACTOME_GLUCOSE_TRANSPORT	KEGG_FATTY_ACID_METABOLISM	KEGG_ARGININE_AND_PROLINE_METABOLISM	KEGG_PEROXISOME
CSTF3	0	0	0	1	0	0	0	0	0	0	0
CSTF2	0	0	0	1	0	0	0	0	0	0	0
PRPF8	0	0	0	1	0	0	0	0	0	0	0
PCBP1	0	0	0	1	0	0	0	0	0	0	0
CSTF1	0	0	0	1	0	0	0	0	0	0	0
DHX38	0	0	0	1	0	0	0	0	0	0	0
CPSF7	0	0	0	1	0	0	0	0	0	0	0
CPSF1	0	0	0	1	0	0	0	0	0	0	0
FUS	0	0	0	1	0	0	0	0	0	0	0
CPSF3	0	0	0	1	0	0	0	0	0	0	0
CPSF2	0	0	0	1	0	0	0	0	0	0	0
LSM2	0	0	0	1	0	0	0	0	0	0	0
HNRNPL	0	0	0	1	0	0	0	0	0	0	0
HNRNPM	0	0	0	1	0	0	0	0	0	0	0
HNRNPK	0	0	0	1	0	0	0	0	0	0	0
HNRNPF	0	0	0	1	0	0	0	0	0	0	0
HNRNPD	0	0	0	1	0	0	0	0	0	0	0
RNPS1	0	0	0	1	0	0	0	0	0	0	0
HNRNPC	0	0	0	1	0	0	0	0	0	0	0
SUGP1	0	0	0	1	0	0	0	0	0	0	0
PTBP1	0	0	0	1	0	0	0	0	0	0	0
SNRNP70	0	0	0	1	0	0	0	0	0	0	0
RBM5	0	0	0	1	0	0	0	0	0	0	0
NCBP1	0	0	0	1	0	0	0	0	0	0	0
SMC1A	0	0	0	1	0	0	0	0	0	0	0
NHP2L1	0	0	0	1	0	0	0	0	0	0	0
HNRNPH1	0	0	0	1	0	0	0	0	0	0	0
SNRPA1	0	0	0	1	0	0	0	0	0	0	0
HNRNPH2	0	0	0	1	0	0	0	0	0	0	0
DNAJC8	0	0	0	1	0	0	0	0	0	0	0
SF3B5	0	0	0	1	0	0	0	0	0	0	0
SF3B2	0	0	0	1	0	0	0	0	0	0	0
SF3B3	0	0	0	1	0	0	0	0	0	0	0
SRSF1	0	0	0	1	0	0	0	0	0	0	0
DDX23	0	0	0	1	0	0	0	0	0	0	0
U2AF1	0	0	0	1	0	0	0	0	0	0	0
NXF1	0	0	0	1	0	0	0	0	0	0	0
POLR2A	0	0	0	1	0	0	0	0	0	0	0
PABPN1	0	0	0	1	0	0	0	0	0	0	0
POLR2B	0	0	0	1	0	0	0	0	0	0	0
U2AF2	0	0	0	1	0	0	0	0	0	0	0
POLR2C	0	0	0	1	0	0	0	0	0	0	0
POLR2E	0	0	0	1	0	0	0	0	0	0	0
SNRPB2	0	0	0	1	0	0	0	0	0	0	0
POLR2H	0	0	0	1	0	0	0	0	0	0	0
HNRNPA1	0	0	0	1	0	0	0	0	0	0	0
EIF4E	0	0	0	1	0	0	0	0	0	0	0
SF3B1	0	0	0	1	0	0	0	0	0	0	0
SRSF11	0	0	0	1	0	0	0	0	0	0	0
POLR2L	0	0	0	1	0	0	0	0	0	0	0
HNRNPA0	0	0	0	1	0	0	0	0	0	0	0
SF3A3	0	0	0	1	0	0	0	0	0	0	0

Table S6 The differential proteins contribute to the enrichment of the significant pathways for the public dataset

Core enrichment proteins in down-regulated pathways of hiPSCs/hESCs(hiPSCs vs hESCs)

NAME	REACTOME_RESPIRATORY_ELECTRON_TRANSPORT_ATP_SYNTHESIS_BY_CHEMIOSMOTIC_COUPLING_AND_HEAT_PRODUCTION_BY_UNCOUPLING_PROTEI	REACTOME_TCA_CYCLE_AND_RESPIRATORY_ELECTRON_TRANSPORT	REACTOME_PYRUVATE_METABOLISM_AND_CITRIC_ACID_TCA_CYCLE	REACTOME_PROCESSING_OF_CAPPED_INTRON_CONTAINING_PRE_MRNA	REACTOME_NEP_NS2_INTERACTS_WITH_THE_CELLULAR_EXPORT_MACHINERY	REACTOME_TRANSPORT_OF_RIBONUCLEOPROTEINS_INTO_THE_HOST_NUCLEUS	REACTOME_REGULATION_OF_GLUCOKINASE_BY_GLUCOKINASE_REGULATORY_PROTEIN	REACTOME_GLUCOSE_TRANSPORT	KEGG_FATTY_ACID_METABOLISM	KEGG_ARGININE_AND_PROLINE_METABOLISM	KEGG_PEROXISOME
SF3A1	0	0	0	1	0	0	0	0	0	0	0
HNRNPA3	0	0	0	1	0	0	0	0	0	0	0
SF3A2	0	0	0	1	0	0	0	0	0	0	0
NUDT21	0	0	0	1	0	0	0	0	0	0	0
SNRNP40	0	0	0	1	0	0	0	0	0	0	0
PHF5A	0	0	0	1	0	0	0	0	0	0	0
HNRNPA2B1	0	0	0	1	0	0	0	0	0	0	0
RBMX	0	0	0	1	0	0	0	0	0	0	0
KPNB1	0	0	0	0	0	1	0	0	0	0	0
NUP210	0	0	0	1	1	1	1	1	0	0	0
NUP43	0	0	0	1	1	1	1	1	0	0	0
NUP50	0	0	0	1	0	1	1	0	0	0	0
NUP107	0	0	0	1	1	1	1	0	0	0	0
NUP214	0	0	0	1	1	1	1	0	0	0	0
NUP93	0	0	0	1	1	1	1	0	0	0	0
NUP205	0	0	0	1	1	1	1	0	0	0	0
SEH1L	0	0	0	1	1	1	1	0	0	0	0
AAAS	0	0	0	1	1	1	1	0	0	0	0
TPR	0	0	0	1	1	1	1	0	0	0	0
NUP88	0	0	0	1	1	1	1	0	0	0	0
NUP155	0	0	0	1	1	1	1	0	0	0	0
NUP153	0	0	0	1	1	1	1	0	0	0	0
NUP62	0	0	0	1	1	1	1	0	0	0	0
NUP133	0	0	0	1	1	1	1	0	0	0	0
NUP54	0	0	0	1	1	1	1	0	0	0	0
RAE1	0	0	0	1	1	1	1	0	0	0	0
RANBP2	0	0	0	1	1	1	1	0	0	0	0
NUP35	0	0	0	1	1	1	1	0	0	0	0
NUPL1	0	0	0	1	1	1	1	0	0	0	0
NUP37	0	0	0	1	1	1	1	0	0	0	0
ALDH1B1	0	0	0	0	0	0	0	0	1	1	0
ACAA2	0	0	0	0	0	0	0	0	1	0	0
ACAT1	0	0	0	0	0	0	0	0	1	0	0
HADHB	0	0	0	0	0	0	0	0	1	0	0
HADHA	0	0	0	0	0	0	0	0	1	0	0
CPT2	0	0	0	0	0	0	0	0	1	0	0
HADH	0	0	0	0	0	0	0	0	1	0	0
P4HA1	0	0	0	0	0	0	0	0	0	1	0
CKMT1B	0	0	0	0	0	0	0	0	0	1	0
PRODH	0	0	0	0	0	0	0	0	0	1	0
ECI2	0	0	0	0	0	0	0	0	1	0	1
ACOX1	0	0	0	0	0	0	0	0	1	0	1
PEX11B	0	0	0	0	0	0	0	0	0	0	1
ECH1	0	0	0	0	0	0	0	0	0	0	1
PIPOX	0	0	0	0	0	0	0	0	0	0	1
PEX14	0	0	0	0	0	0	0	0	0	0	1
PMVK	0	0	0	0	0	0	0	0	0	0	1
CAT	0	0	0	0	0	0	0	0	0	0	1
HACL1	0	0	0	0	0	0	0	0	0	0	1
CRAT	0	0	0	0	0	0	0	0	0	0	1

Table S6 The differential proteins contribute to the enrichment of the significant pathways for the public dataset

Core enrichment proteins in up-regulated pathways of hiPSCs/hESCs(hiPSCs vs hESCs)

NAME	REACTOME_CELL_SURFACE_INTERACTIONS_AT_THE_VASCULAR_WALL	REACTOME_INTEGRIN_CELL_SURFACE_INTERACTIONS_	KEGG_EPITHELIAL_CELL_ING_IN_HELICOBACTER_PYLORI_INFECTION	KEGG_ECM_RECEPTOR_INTERACTION_	KEGG_ARRHYTHMOGENIC_RIGHT_VENTRICULAR_CARDIOMYOPATHY_ARVC	KEGG_FOCAL_ADHESION_	KEGG_REGULATION_OF_ACTIN_CYTOSKELETON_	KEGG_DNA_REPLICATION_
COL4A1	0	1	0	1	0	1	0	0
ITGA5	1	1	0	1	1	1	1	0
ITGAV	1	1	0	1	1	1	1	0
ITGB1	1	1	0	1	1	1	1	0
ITGB5	0	1	0	1	1	1	1	0
KRAS	1	0	0	0	0	0	1	0
LAMB1	0	1	0	1	0	1	0	0
PLCG1	1	0	1	0	0	0	0	0
SHC1	1	1	0	0	0	1	0	0
THBS1	0	1	0	1	0	1	0	0
TLN1	0	1	0	0	0	1	0	0
ABI2	0	0	0	0	0	0	1	0
ACTB	0	0	0	0	1	1	1	0
ACTN2	0	0	0	0	0	1	1	0
ADAM10	0	0	1	0	0	0	0	0
ARAF	0	0	0	0	0	0	1	0
ARHGAP35	0	0	0	0	0	1	1	0
ARPC1A	0	0	0	0	0	0	1	0
ARPC2	0	0	0	0	0	0	1	0
ARPC4	0	0	0	0	0	0	1	0
ARPC5	0	0	0	0	0	0	1	0
ATP6AP1	0	0	1	0	0	0	0	0
ATP6V0A1	0	0	1	0	0	0	0	0
ATP6V0C	0	0	1	0	0	0	0	0
ATP6V0D1	0	0	1	0	0	0	0	0
ATP6V1D	0	0	1	0	0	0	0	0
ATP6V1E1	0	0	1	0	0	0	0	0
ATP6V1F	0	0	1	0	0	0	0	0
ATP6V1G1	0	0	1	0	0	0	0	0
CAPN2	0	0	0	0	0	1	0	0
CASP3	0	0	1	0	0	0	0	0
CDH2	0	0	0	0	1	0	0	0
CFL1	0	0	0	0	0	0	1	0
CHUK	0	0	1	0	0	0	0	0
COL5A1	0	0	0	1	0	1	0	0
COL6A1	0	0	0	0	0	1	0	0
COL6A2	0	0	0	0	0	1	0	0
CRK	0	0	0	0	0	1	1	0
CYFIP1	0	0	0	0	0	0	1	0
DAG1	0	0	0	1	1	0	0	0
DIAPH1	0	0	0	0	0	1	1	0
DOCK1	0	0	0	0	0	1	1	0
ERBB2	0	0	0	0	0	1	0	0
EZR	0	0	0	0	0	0	1	0
FGF2	0	0	0	0	0	0	1	0
GNG12	0	0	0	0	0	0	1	0
GSK3B	0	0	0	0	0	1	0	0
HSPG2	0	0	0	1	0	0	0	0
IGF1R	0	0	0	0	0	1	0	0
ILK	0	0	0	0	0	1	0	0
IQGAP1	0	0	0	0	0	0	1	0
MAPK1	0	0	0	0	0	1	1	0
MSN	0	0	0	0	0	0	1	0
MYH9	0	0	0	0	0	0	1	0
MYL9	0	0	0	0	0	1	1	0
NCKAP1	0	0	0	0	0	0	1	0
PAK1	0	0	1	0	0	1	1	0
PAK2	0	0	0	0	0	1	1	0

Table S6 The differential proteins contribute to the enrichment of the significant pathways for the public dataset

Core enrichment proteins in up-regulated pathways of hiPSCs/hESCs(hiPSCs vs hESCs)

NAME	REACTOME_CELL_SURFACE_INTERACTIONS_AT_THE_VASCULAR_WALL	REACTOME_INTEGRIN_CELL_SURFACE_INTERACTIONS_	KEGG_EPITHELIAL_CELL_ING_IN_HELICOBACTER_PYLORI_INFECTION	KEGG_ECM_RECEPTOR_INTERACTION_	KEGG_ARRHYTHMOGENIC_RIGHT_VENTRICULAR_CARDIOMYOPATHY_ARVC	KEGG_FOCAL_ADHESION_	KEGG_REGULATION_OF_ACTIN_CYTOSKELETON_	KEGG_DNA_REPLICATION_
PARVA	0	0	0	0	0	1	0	0
PCNA	0	0	0	0	0	0	0	1
PFN1	0	0	0	0	0	0	1	0
PIP4K2B	0	0	0	0	0	0	1	0
POLA1	0	0	0	0	0	0	0	1
POLA2	0	0	0	0	0	0	0	1
POLD3	0	0	0	0	0	0	0	1
POLE3	0	0	0	0	0	0	0	1
PPP1CA	0	0	0	0	0	1	1	0
PRIM2	0	0	0	0	0	0	0	1
RAC1	0	0	1	0	0	1	1	0
RDX	0	0	0	0	0	0	1	0
RELA	0	0	1	0	0	0	0	0
RFC1	0	0	0	0	0	0	0	1
ROCK1	0	0	0	0	0	1	1	0
ROCK2	0	0	0	0	0	1	1	0
TLN2	0	0	0	0	0	1	0	0
VASP	0	0	0	0	0	1	0	0
VCL	0	0	0	0	0	1	1	0
ZYX	0	0	0	0	0	1	0	0
ATP1B1	1	0	0	0	0	0	0	0
ATP1B3	1	0	0	0	0	0	0	0
SLC3A2	1	0	0	0	0	0	0	0
SLC7A5	1	0	0	0	0	0	0	0
